# Supplementary material for: Genetic associations between genes in the renin-angiotensin-aldosterone system and renal disease: a systematic review and meta-analysis
Source: BMJ Open. 2019 May 1;9(4):e026777. doi: 10.1136/bmjopen-2018-026777 (PMC6501980; doi:10.1136/bmjopen-2018-026777)
Supplement: Supplementary data [file bmjopen-2018-026777supp001.pdf]

**Supplementary Table S1:** Search Terms

| *Gene* | **Search Terms** |
|---|---|
| *ACE* | Angiotensin I Converting Enzyme<br>Angiotensin I Converting Enzyme (Peptidyl-Dipeptidase A) 1<br>Dipeptidyl Carboxypeptidase I<br>CD143 Antigen<br>Kininase II<br>DCP1<br>DCP<br>Angiotensin I Converting Enzyme Peptidyl-Dipeptidase A 1 Transcript<br>Angiotensin Converting Enzyme, Somatic Isoform<br>Peptidyl-Dipeptidase A<br>Carboxycathepsin<br>Testicular ECA<br>Peptidase P<br>EC 3.4.15.1<br>EC 3.2.1.-<br>CD143<br>MVCD3<br>ACE1<br>ACE<br>ICH<br><br>AND<br><br>Kidney<br>Nephrology<br>Nephropathy<br>Renal<br><br>AND<br><br>SNP<br>Polymorphism<br>Variant<br>Allele<br>Genotype |
| *ACE2* | Angiotensin I Converting Enzyme 2<br>Angiotensin I Converting Enzyme (Peptidyl-Dipeptidase A) 2<br>Angiotensin-Converting Enzyme Homolog<br>ACE-Related Carboxypeptidase<br>Metalloprotease MPROT15<br>Peptidyl-Dipeptidase A<br>ACEH<br>EC 3.4.17.23<br>EC 3.4.17<br>ACE2<br><br>AND |

| | |
|---|---|
| | Kidney<br>Nephrology<br>Nephropathy<br>Renal<br><br><u>AND</u><br><br>SNP<br>Polymorphism<br>Variant<br>Allele<br>Genotype |
| *AGT* | Angiotensinogen<br>Angiotensinogen (Serpin Peptidase Inhibitor, Clade A, Member 8)<br>Serpin Peptidase Inhibitor, Clade A, Member 8<br>Serpin A8<br>SERPINA8<br>Serine (Or Cysteine) Proteinase Inhibitor<br>Alpha-1 Antiproteinase, Antitrypsin<br>Alpha-1 Antiproteinase<br>Pre-Angiotensinogen<br>Angiotensin II<br>Angiotensin I<br>Antitrypsin<br>ANHU<br>AGT<br><br><u>AND</u><br><br>Kidney<br>Nephrology<br>Nephropathy<br>Renal<br><br><u>AND</u><br><br>SNP<br>Polymorphism<br>Variant<br>Allele<br>Genotype |
| *AGTR1* | Angiotensin II Receptor Type 1<br>Angiotensin II Receptor, Type 1<br>AGTR1B<br>AT1AR<br>AT1BR<br>AT2R1<br>AT1<br>Type-1B Angiotensin II Receptor<br>Angiotensin II Type-1 Receptor<br>Angiotensin Receptor 1B |

| | |
|---|---|
| | AGTR1A<br>AT2R1B<br>HAT1R<br>AG2S<br>AT1B<br>AT1R<br>AGTR1<br>AT2R1A<br><br>AND<br><br>Kidney<br>Nephrology<br>Nephropathy<br>Renal<br><br>AND<br><br>SNP<br>Polymorphism<br>Variant<br>Allele<br>Genotype |
| *AGTR2* | Angiotensin II Receptor Type 2<br>Angiotensin II Receptor, Type 2<br>Angiotensin II Type-2 Receptor<br>AT2<br>Angiotensin Receptor 2<br>ATGR2<br>MRX88<br><br>AND<br><br>Kidney<br>Nephrology<br>Nephropathy<br>Renal<br><br>AND<br><br>SNP<br>Polymorphism<br>Variant<br>Allele<br>Genotype |
| *REN* | Renin<br>Angiotensinogenase<br>EC 3.4.23.15<br>Angiotensin-Forming Enzyme<br>Renin Precursor, Renal<br>EC 3.4.23 |

| | |
|---|---|
| | HNFJ2<br>REN<br><br><u>AND</u><br><br>Kidney<br>Nephrology<br>Nephropathy<br>Renal<br><br><u>AND</u><br><br>SNP<br>Polymorphism<br>Variant<br>Allele<br>Genotype |
| *Additional filters added to database searches* | English / Spanish language<br>Human studies<br>Case / Control studies<br>Not clinical trials<br>Not review articles<br>Not a case report<br>Not a meta-analysis |

**Supplementary Table S2:** Ethnicity Codes

| Population | Population Code |
|---|---|
| Ad Mixed American (Southern USA and Central America) | AMR |
| African American | AFR |
| East Asian | EAS |
| European | EUR |
| Middle Eastern | ME |
| Mix of ethnicities | MIX |
| North African | NA |
| South Asian | SAS |

**Supplementary Table S3a:** Excluded studies from the *ACE* search

*Exclusion stage relates to the stage at which the article was removed as shown in the flow diagram (Supplementary Figure S1a)

| Exclusion Stage | Title | Authors | Pubmed ID or WoS ID if Pubmed ID not available | Reason |
|---|---|---|---|---|
| 1 | Angiotensin I - Converting enzyme gene polymorphism modulates the consequences of in utero growth retardation on plasma insulin in young adults | Cambien F et al. | 9519756 | No data for ACE |
| 1 | Clinical utility of chitotriosidase enzyme activity in nephropathic cystinosis. | Elmonem MA et al. | 25407738 | No data for ACE |
| 1 | Polymorphisms in the gene encoding angiotensin I converting enzyme 2 and diabetic nephropathy | Frojdo S et al. | 16211375 | No data for ACE |
| 1 | Effects of erythropoietin, angiotensin II, and angiotensin-converting enzyme inhibitor on erythroid precursors in patients with posttransplantation erythrocytosis. | Glicklich D et al. | 10428268 | No data for ACE |
| 1 | DDOST, PRKCSH and LGALS3, which encode AGE-receptors 1, 2 and 3, respectively, are not associated with diabetic nephropathy in type 1 diabetes. | Hoverfelt A et al. | 20490454 | No data for ACE |
| 1 | Neuropeptide YY1 receptor polymorphism as a prognostic predictor in Japanese patients with IgA nephropathy | Ito H et al. | 10363627 | No data for ACE |
| 1 | Manganese superoxide dismutase gene polymorphism (V16A) is associated with stages of albuminuria in Korean type 2 diabetic patients. | Lee SJ et al. | 16324912 | No data for ACE |
| 1 | Impact of the preintervention rate of renal function decline on outcome of renoprotective intervention. | Lely AT et al. | 18077786 | No data for ACE |

| | | | | |
|---|---|---|---|---|
| 1 | Kinin-dependent hypersensitivity reactions in hemodialysis: metabolic and genetic factors. | Molinaro G et al. | 17003818 | No data for ACE |
| 1 | Effect of a polymorphism of endothelial nitric oxide synthase gene in Japanese patients with IgA nephropathy. | Morita T et al. | 10543322 | No data for ACE |
| 1 | Nephropathy in type 1 diabetes: a manifestation of insulin resistance and multiple genetic susceptibilities? Further evidence from the Pittsburgh Epidemiology of Diabetes Complication Study. | Orchard TJ et al. | 12164879 | No data for ACE |
| 1 | Role of glycaemic control in development of microalbuminuria in patients with insulin dependent diabetes. | Powrie JK et al. | 7819935 | No data for ACE |
| 1 | Association of TNF-Î± -308 G > A and ACE I/D gene polymorphisms in hemodialysis patients with arteriovenous fistula thrombosis. | Sener EF et al. | 24126814 | No data for ACE |
| 1 | N-domain angiotensin I-converting enzyme expression in renal artery of Wistar, Wistar Kyoto, and spontaneously hypertensive rats | Bueno V et al. | 15194348 | Non-human study |
| 1 | Kallikrein and amylase contents in tissues from a mutant mouse model for human cystic fibrosis. | Catanzaro OL et al. | 6186886 | Non-human study |
| 1 | Connexin 43 is not essential for the control of renin synthesis and secretion | Gerl M et al. | 24062052 | Non-human study |
| 1 | Multi-species comparative analysis of the equine ACE gene identifies a highly conserved potential transcription factor binding site in intron 16. | Hamilton NA et al. | 23408978 | Non-human study |
| 1 | Renal angiotensin converting enzyme promotes renal damage during ureteral obstruction | Stoneking BJ et al. | 9719278 | Non-human study |
| 1 | Angiotensin-converting enzyme genotype is a predictive factor in the peak panel-reactive antibody response. | Akcay A et al. | 15013293 | Not a case-control study |
| 1 | Association of the genetic polymorphisms of the renin-angiotensin system and endothelial nitric oxide synthase with chronic renal transplant dysfunction. | Akcay A et al. | 15385810 | Not a case-control study |

| | | | | |
|---|---|---|---|---|
| 1 | PAI-1 4G/5G and ACE I/D gene polymorphisms and the occurrence of myocardial infarction in patients on intermittent dialysis. | Aucella F et al. | 12748347 | Not a case-control study |
| 1 | Angiotensin-converting-enzyme insertion/deletion genotype and long-term renal allograft survival. | Beige J et al. | 9550656 | Not a case-control study |
| 1 | Deletion insertion polymorphism of the angiotensin converting enzyme gene and progression of diabetic nephropathy. | Bjorck S et al. | 9269704 | Not a case-control study |
| 1 | Genetics of angiotensin I-converting enzyme | Costerousse O et al. | 9247746 | Not a case-control study |
| 1 | Captopril enhances transforming growth factor (TGF)-beta1 expression in peripheral blood mononuclear cells: a mechanism independent from angiotensin converting enzyme inhibition? A study in cyclosporine-treated kidney-transplanted patients. | Di Paolo S et al. | 12499886 | Not a case-control study |
| 1 | Insertion/deletion polymorphism of the angiotensin-converting enzyme predicts left ventricular hypertrophy after renal transplantation. | Fedor R et al. | 21620105 | Not a case-control study |
| 1 | Antiproteinuric effect of candesartan cilexetil in Japanese subjects with type 2 diabetes and nephropathy. | Haneda M et al. | 15364166 | Not a case-control study |
| 1 | Association between Angiotensin I-Converting Enzyme Insertion/Deletion Polymorphism and Prognosis of Kidney Transplantation: A Meta-Analysis | Huang ZK et al. | 26000752 | Not a case-control study |
| 1 | Is there a role of angiotensin-converting enzyme gene polymorphism in the failure of arteriovenous femoral shunts for hemodialysis? | Isbir CS et al. | 11525534 | Not a case-control study |
| 1 | Smoking has no impact on survival and it is not associated with ACE gene I/D polymorphism in hemodialysis patients. | Kiss I et al. | 28058974 | Not a case-control study |
| 1 | Antihypertensive treatment modulates the association between the D/I ACE gene polymorphism and left ventricular hypertrophy: a meta-analysis | Kuznetsova T et al. | 10918550 | Not a case-control study |

| | | | | |
|---|---|---|---|---|
| 1 | Genetics and the prediction of complications in type 1 diabetes | Marre M | 10097900 | Not a case-control study |
| 1 | Hereditary factors in the development of diabetic renal disease | Marre M et al. | 10922971 | Not a case-control study |
| 1 | The effect of angiotensin receptor blockade ARB on the regression of left ventricular hypertrophy in hemodialysis patients: comparison between patients with D allele and non-D allele ACE gene polymorphism. | Nakayama M et al. | 16312263 | Not a case-control study |
| 1 | Role of ACE and IL-1Î² Gene Polymorphisms in Erythropoeitin Hyporesponsive Patients with Chronic Kidney Disease with Anemia. | Nand N et al. | 28457029 | Not a case-control study |
| 1 | Angiotensin-I converting enzyme insertion/deletion polymorphism and its association with diabetic nephropathy: a meta-analysis of studies reported between 1994 and 2004 and comprising 14,727 subjects | Ng D et al. | 15830182 | Not a case-control study |
| 1 | Is the presence of retinopathy of practical value in defining cases of diabetic nephropathy in genetic association studies? The experience with the ACE insertion/deletion polymorphism in 53 studies comprising 17,791 subjects | Ng DPK et al. | 18523141 | Not a case-control study |
| 1 | Correlates of ACE activity in macroalbuminuric type 2 diabetic patients treated with chronic ACE inhibition. | Nikzamir A et al. | 17986476 | Not a case-control study |
| 1 | Insertion/deletion polymorphism in intron 16 of the ACE gene and left ventricular hypertrophy in patients with end-stage renal disease. | Osono E et al. | 9820440 | Not a case-control study |
| 1 | Risk factors for the progression of microalbuminuria in Japanese type 2 diabetic patients - a 10-year follow-up study | Oue T et al. | 10580616 | Not a case-control study |
| 1 | Survival in type 2 diabetic patients in dialysis and the number of risk alleles in polymorphisms of the renin-angiotensin system genes. | Padro-Miquel A et al. | 19014923 | Not a case-control study |

| | | | | |
|---|---|---|---|---|
| 1 | Effect of deletion polymorphism of angiotensin converting enzyme gene on progression of diabetic nephropathy during inhibition of angiotensin converting enzyme: observational follow up study. | Parving HH et al. | 8806248 | Not a case-control study |
| 1 | Altered activities of kininase II, an angiotensin converting enzyme, prekallikrein, and nitric oxide in Kuwaiti patients with type 2 diabetes | Sharma JN et al. | 25964383 | Not a case-control study |
| 1 | Impact of genetic polymorphisms of the renin-angiotensin system and of non-genetic factors on kidney transplant function--a single-center experience. | Siekierka-Harreis M et al. | 19681973 | Not a case-control study |
| 1 | Angiotensin-converting enzyme (ACE) inhibition in type 2, diabetic patients-- interaction with ACE insertion/deletion polymorphism. | So WY et al. | 16395257 | Not a case-control study |
| 1 | Microfluidic chip-based method for genotyping microsatellites, VNTRs and insertion/deletion polymorphisms. | Sohni YR et al. | 12554058 | Not a case-control study |
| 1 | M235T angiotensinogen gene polymorphism and cardiovascular renal risk | Staessen JA et al. | 10100088 | Not a case-control study |
| 1 | Mistyping of the human angiotensin-converting enzyme gene polymorphism: Frequency, causes and possible methods to avoid errors in typing | Ueda S et al. | 8863184 | Not a case-control study |
| 1 | Association between angiotensin-converting-enzyme gene polymorphism and failure of renoprotective therapy. | van Essen GG et al. | 8538349 | Not a case-control study |
| 1 | Contribution of gene polymorphisms in the renin-angiotensin system to macroangiopathy in patients with diabetic nephropathy. | Wong TY et al. | 11431175 | Not a case-control study |
| 1 | Disease progression, response to ACEI/ATRA therapy and influence of ACE gene in IgA nephritis. | Woo KT et al. | 17601378 | Not a case-control study |
| 1 | Angiotensin-converting enzyme inhibitor versus angiotensin 2 receptor antagonist therapy and the influence of angiotensin-converting enzyme gene polymorphism in IgA nephritis. | Woo KT et al. | 18536822 | Not a case-control study |

| | | | | |
|---|---|---|---|---|
| 1 | Angiotensin converting enzyme gene polymorphism and development of post-transplant erythrocytosis. | Yildiz A et al. | 12832741 | Not a case-control study |
| 1 | Gene polymorphisms of the renin-angiotensin-aldosterone system and angiotensin II type 1-receptor activating antibodies in renal rejection. | Zhang G et al. | 17984617 | Not a case-control study |
| 1 | Angiotensin-converting enzyme gene polymorphism in Kuwaiti patients with systemic lupus erythematosus. | Al-Awadhi AM et al. | 17631741 | Not a renal disease focus |
| 1 | Association of Angiotensin Converting Enzyme Insertion-Deletion Polymorphism with Hypertension in Emiratis with Type 2 Diabetes Mellitus and Its Interaction with Obesity Status. | Alsafar H et al. | 26491214 | Not a renal disease focus |
| 1 | Association of ACE gene D polymorphism with left ventricular hypertrophy in patients with diastolic heart failure: a case-control study. | Bahramali E et al. | 26861937 | Not a renal disease focus |
| 1 | A novel human heparanase splice variant, T5, endowed with protumorigenic characteristics. | Barash U et al. | 20007507 | Not a renal disease focus |
| 1 | Synergistic effect of alpha-adducin and ACE genes causes blood pressure changes with body sodium and volume expansion. | Barlassina C et al. | 10720960 | Not a renal disease focus |
| 1 | Isolated polycystic liver disease genes define effectors of polycystin-1 function. | Besse W et al. | 28375157 | Not a renal disease focus |
| 1 | Association between plasma activities of semicarbazide-sensitive amine oxidase and angiotensin-converting enzyme in patients with type 1 diabetes mellitus. | Boomsma F et al. | 15830186 | Not a renal disease focus |
| 1 | CCL18: a urinary marker of Gaucher cell burden in Gaucher patients. | Boot RG et al. | 16736095 | Not a renal disease focus |
| 1 | Renin-angiotensin system gene polymorphisms: assessment of the risk of coronary heart disease. | Buraczynska M et al. | 14502296 | Not a renal disease focus |
| 1 | Angiotensin-converting enzyme (ACE) haplotypes and cyclosporine A (CsA) response: a model of the complex relationship between ACE quantitative trait locus and pathological phenotypes. | Catarsi P et al. | 16002416 | Not a renal disease focus |

| | | | | |
|---|---|---|---|---|
| 1 | A study on the association between angiotensin-I converting enzyme I/D dimorphism and type-2 diabetes mellitus. | Chmaisse HN et al. | 19861867 | Not a renal disease focus |
| 1 | alpha-adducin and angiotensin I-converting enzyme polymorphisms in essential hypertension | Clark CJ et al. | 11116113 | Not a renal disease focus |
| 1 | Elevated urinary albumin excretion is not linked to the angiotensin I-converting enzyme gene polymorphism in clinically healthy subjects | Clausen P et al. | 10872702 | Not a renal disease focus |
| 1 | Association of renin-angiotensin and endothelial nitric oxide synthase gene polymorphisms with blood pressure progression and incident hypertension: prospective cohort study. | Conen D et al. | 18698212 | Not a renal disease focus |
| 1 | Angiotensin I-converting enzyme (kininase II) in cardiovascular and renal regulations and diseases | Costerousse O et al. | 9830503 | Not a renal disease focus |
| 1 | ACE and PC-1 gene polymorphisms in normoalbuminuric Type 1 diabetic patients: a 10-year prospective study. | de Azevedo MJ et al. | 12126783 | Not a renal disease focus |
| 1 | Insertion/Deletion Polymorphism of Angiotensin I-converting Enzyme Gene Is Linked With Chromophobe Renal Cell Carcinoma | de Martino M et al. | 21477733 | Not a renal disease focus |
| 1 | Polymorphisms in genes of the renin-angiotensin-aldosterone system and renal cell cancer risk: interplay with hypertension and intakes of sodium, potassium and fluid. | Deckers IA et al. | 24978482 | Not a renal disease focus |
| 1 | ACE gene insertion/deletion polymorphism modulates capillary permeability in hypertension | Dell'omo G et al. | 16889537 | Not a renal disease focus |
| 1 | Genetic polymorphisms associated with exertional rhabdomyolysis | Deuster PA et al. | 23543093 | Not a renal disease focus |
| 1 | Distribution of different HLA antigens in Greek hypertensives according to the angiotensin-converting enzyme genotype. | Diamantopoulos EJ et al. | 10821349 | Not a renal disease focus |
| 1 | Are the angiotensin-converting enzyme gene and activity risk factors for stroke? | Dikmen M et al. | 16791358 | Not a renal disease focus |
| 1 | Angiotensin-converting enzyme (insertion/deletion) and endothelial nitric oxide synthase polymorphisms in patients with systemic lupus erythematosus. | Douglas G et al. | 15338496 | Not a renal disease focus |

| | | | | |
|---|---|---|---|---|
| 1 | Abnormal hepatocystin caused by truncating PRKCSH mutations leads to autosomal dominant polycystic liver disease. | Drenth JP et al. | 15057895 | Not a renal disease focus |
| 1 | The angiotensin I-converting enzyme gene insertion/deletion polymorphism is linked to early gastric cancer | Ebert MPA et al. | 16365022 | Not a renal disease focus |
| 1 | Association of chitotriosidase enzyme activity and genotype with the risk of nephropathy in type 2 diabetes. | Elmonem MA et al. | 26589000 | Not a renal disease focus |
| 1 | Relationship of bradykinin B2 receptor gene polymorphism with essential hypertension and left ventricular hypertrophy. | Fu Y et al. | 15894833 | Not a renal disease focus |
| 1 | Deletion polymorphism of the angiotensin-converting enzyme gene is independently associated with left ventricular mass and geometric remodeling in systemic hypertension. | Gharavi AG et al. | 8677872 | Not a renal disease focus |
| 1 | The presence of PAI-1 4G/5G and ACE DD genotypes increases the risk of early-stage AVF thrombosis in hemodialysis patients. | Gungor Y et al. | 21332339 | Not a renal disease focus |
| 1 | The Captopril Prevention Project (CAPPP) in hypertension--baseline data and current status. | Hansson L et al. | 9495662 | Not a renal disease focus |
| 1 | Relationship of eNOS gene variants to diseases that have in common an endothelial cell dysfunction. | Heltianu C et al. | 15784171 | Not a renal disease focus |
| 1 | Relationship of the angiotensin-converting enzyme gene polymorphism to glucose intolerance, insulin resistance, and hypertension in NIDDM. | Huang XH et al. | 9544854 | Not a renal disease focus |
| 1 | Distribution of human leukocyte antigen alleles in systemic lupus erythematosus patients with angiotensin converting enzyme insertion/deletion polymorphism. | Hussain N et al. | 23448612 | Not a renal disease focus |
| 1 | Comprehensive analysis of the renin-angiotensin gene polymorphisms with relation to hypertension in the Japanese | Kato N et al. | 10953993 | Not a renal disease focus |
| 1 | Association of clinical manifestations with HLA-B alleles in Takayasu arteritis | Kitamura H et al. | 9951811 | Not a renal disease focus |
| 1 | Lys(173)Arg and -344T/C variants of CYP11B2 in Japanese patients with low-renin hypertension. | Komiya I et al. | 10720581 | Not a renal disease focus |

| | | | | |
|---|---|---|---|---|
| 1 | Effects of angiotensin-converting enzyme gene polymorphism and serum vitamin D levels on ambulatory blood pressure measurement and left ventricular mass in Turkish hypertensive population. | Kulah E et al. | 17625392 | Not a renal disease focus |
| 1 | Efficacy of Korean Red Ginseng by Single Nucleotide Polymorphism in Obese Women: Randomized, Double-blind, Placebo-controlled Trial | Kwon DH et al. | 23717118 | Not a renal disease focus |
| 1 | Angiotensin-Converting Enzyme Gene Polymorphism Has No Influence On The Circulating Renin-Angiotensin-Aldosterone System Or Blood-Pressure In Normotensive Subjects | Lachurie Ml et al. | 7796503 | Not a renal disease focus |
| 1 | Mutations in PRKCSH cause isolated autosomal dominant polycystic liver disease. | Li A et al. | 12529853 | Not a renal disease focus |
| 1 | Angiotensin-converting enzyme (ACE) gene II genotype protects against the development of diabetic peripheral neuropathy in type 2 diabetes mellitus. | Mansoor Q et al. | 22607040 | Not a renal disease focus |
| 1 | Renal changes on hyperglycemia and angiotensin-converting enzyme in type 1 diabetes | Marre M et al. | 10082486 | Not a renal disease focus |
| 1 | Detection of the association between a deletion polymorphism in the gene encoding angiotensin I-converting enzyme and advanced diabetic retinopathy | Matsumoto A et al. | 11106834 | Not a renal disease focus |
| 1 | Association of the D allele of the angiotensin I converting enzyme polymorphism with malignant vascular injury | Mayer NJ et al. | 11836444 | Not a renal disease focus |
| 1 | Late-onset acid maltase deficiency. Detection of patients and heterozygotes by urinary enzyme assay. | Mehler M et al. | 9923 | Not a renal disease focus |
| 1 | Association of polymorphisms of angiotensin I converting enzyme 2 with retinopathy in type 2 diabetes mellitus among Chinese individuals | Meng N et al. | 25359286 | Not a renal disease focus |
| 1 | Angiotensin converting enzyme gene polymorphism and renal hemodynamic function in early diabetes. | Miller JA et al. | 8995725 | Not a renal disease focus |

| | | | | |
|---|---|---|---|---|
| 1 | Genetic risk for renal artery stenosis: association with deletion polymorphism in angiotensin 1-converting enzyme gene. | Missouris CG et al. | 8821841 | Not a renal disease focus |
| 1 | Renal ACE immunohistochemical localization in NIDDM patients with nephropathy. | Mizuiri S et al. | 9469501 | Not a renal disease focus |
| 1 | Association of B2 receptor polymorphisms and ACE activity with ACE inhibitor-induced angioedema in black and mixed-race South Africans. | Moholisa RR et al. | 23730990 | Not a renal disease focus |
| 1 | Renal outcome and vascular morbidity in systemic lupus erythematosus (SLE): lack of association with the angiotensin-converting enzyme gene polymorphism. | Molad Y et al. | 11071585 | Not a renal disease focus |
| 1 | Contribution of angiotensin I converting enzyme gene polymorphism and angiotensinogen gene polymorphism to blood pressure regulation in essential hypertension. | Mondorf UF et al. | 9524045 | Not a renal disease focus |
| 1 | Inhibition of tissue angiotensin converting enzyme activity prevents malignant hypertension in TGR(mREN2)27. | Montgomery HE et al. | 9797175 | Not a renal disease focus |
| 1 | Independent, Marked Associations Of Alleles Of The Insulin-Receptor And Dipeptidyl Carboxypeptidase-I Genes With Essential-Hypertension | Morris Bj et al. | 8104754 | Not a renal disease focus |
| 1 | Increased D allele frequency of the angiotensin-converting enzyme gene in pulmonary fibrosis. | Morrison CD et al. | 11381371 | Not a renal disease focus |
| 1 | Angiotensin converting enzyme (ACE) insertion/deletion (I/D) polymorphism, and diabetic retinopathy in subjects with IDDM and NIDDM. | Nagi DK et al. | 8582133 | Not a renal disease focus |
| 1 | Angiotensin-converting enzyme (ACE) gene insertion/deletion polymorphism is not a risk factor for hypertension in SLE nephritis. | Negi VS et al. | 25957879 | Not a renal disease focus |
| 1 | Carotid intima-media thickness and ACE-gene polymorphism in hemodialysis patients. | Nergizoglu G et al. | 10493570 | Not a renal disease focus |
| 1 | Association of angiotensin-converting enzyme gene insertion/deletion polymorphism with metabolic syndrome in Iranians with type 2 diabetes mellitus. | Nikzamir A et al. | 18154415 | Not a renal disease focus |

| | | | | |
|---|---|---|---|---|
| 1 | Angiotensin Converting Enzyme Gene Insertion/Deletion Variant and Familial Mediterranean Fever-related Amyloidosis. | Nursal AF et al. | 29891744 | Not a renal disease focus |
| 1 | Pharmacogenetic analysis of the effect of angiotensin-converting enzyme inhibitor on restenosis after percutaneous transluminal coronary angioplasty. | Okamura A et al. | 10535720 | Not a renal disease focus |
| 1 | Genetic polymorphisms of the renin-angiotensin system and atheromatous renal artery stenosis. | Olivieri O et al. | 10567188 | Not a renal disease focus |
| 1 | Different impact of deletion polymorphism of gene on the risk of renal and coronary artery disease. | Olivieri O et al. | 11791024 | Not a renal disease focus |
| 1 | Increased frequency of the angiotensin-converting enzyme gene D-allele is associated with noninfectious pulmonary dysfunction following allogeneic stem cell transplant. | Onizuka M et al. | 16044138 | Not a renal disease focus |
| 1 | Hepatocystin is Essential for TRPM7 Function During Early Embryogenesis. | Overton JD et al. | 26671672 | Not a renal disease focus |
| 1 | Angiotensin-converting enzyme and angiotensin II receptor subtype 2 genotypes in type 1 diabetes and severe hypoglycaemia requiring emergency treatment: a case cohort study. | Pedersen-Bjergaard U et al. | 19820429 | Not a renal disease focus |
| 1 | Impact of maternal angiotensinogen M235T polymorphism and angiotensin-converting enzyme insertion/deletion polymorphism on blood pressure, protein excretion and fetal outcome in pregnancy. | Pfab T et al. | 17563539 | Not a renal disease focus |
| 1 | Renovascular disease: effect of ACE gene deletion polymorphism and endovascular revascularization. | Pizzolo F et al. | 14718831 | Not a renal disease focus |
| 1 | Angiotensin-converting enzyme gene polymorphism in patients with systemic lupus. | Prkacin I et al. | 11505631 | Not a renal disease focus |
| 1 | The relationship between ACE/AGT gene polymorphisms and the risk of diabetic retinopathy in Chinese patients with type 2 diabetes. | Qiao YC et al. | 29378484 | Not a renal disease focus |
| 1 | Association of angiotensin-converting enzyme gene dimorphisms with severity of lupus disease. | Rabbani MA et al. | 18711292 | Not a renal disease focus |

| | | | | |
|---|---|---|---|---|
| 1 | Leukocyte beta-glucosidase in homozygotes and heterozygotes for Gaucher disease. | Raghavan SS et al. | 6770675 | Not a renal disease focus |
| 1 | Association of angiotensinogen M235T and A(-6)G gene polymorphisms with coronary heart disease with independence of essential hypertension: the PROCAGENE study. Prospective Cardiac Gene. | Rodriquez-Perez JC et al. | 11345362 | Not a renal disease focus |
| 1 | Testing of potential glycan-based heparanase inhibitors in a fluorescence activity assay using either bacterial heparinase II or human heparanase. | Schoenfeld AK et al. | 24667567 | Not a renal disease focus |
| 1 | Angiotensin-converting enzyme gene I/D polymorphism increases the susceptibility to hypertension and additive diseases: A study on North Indian patients. | Singh M et al. | 27030424 | Not a renal disease focus |
| 1 | Association of APOE (Hha1) and ACE (I/D) gene polymorphisms with type 2 diabetes mellitus in North West India | Singh PP et al. | 16621107 | Not a renal disease focus |
| 1 | Angiotensin-converting enzyme gene I/D polymorphism in malignant hypertension. | Stefansson B et al. | 10855732 | Not a renal disease focus |
| 1 | Increased amount of the angiotensin-converting enzyme (ACE) mRNA originating from the ACE allele with deletion. | Suehiro T et al. | 15164285 | Not a renal disease focus |
| 1 | Genetic variants in hypertensive patients with coronary artery disease and coexisting atheromatous renal artery stenosis. | Szperl M et al. | 19043368 | Not a renal disease focus |
| 1 | Angiotensin I converting enzyme gene polymorphisms in systemic lupus erythematosus: decreased prevalence of DD genotype in African American patients | Tassiulas IO et al. | 9710341 | Not a renal disease focus |
| 1 | Albuminuria and the renin-angiotensin system gene polymorphisms in type-2-diabetic and in normoglycemic hypertensive Chinese. | Thomas GN et al. | 11200871 | Not a renal disease focus |
| 1 | Peripheral vascular disease in Type 2 diabetic Chinese patients: associations with metabolic indices, concomitant vascular disease and genetic factors. | Thomas GN et al. | 14632699 | Not a renal disease focus |
| 1 | Angiotensin-converting enzyme gene polymorphism and vascular manifestations in Korean patients with SLE. | Uhm WS et al. | 12043886 | Not a renal disease focus |

| | | | | |
|---|---|---|---|---|
| 1 | Genetic risk of atherosclerotic renal artery disease: the candidate gene approach in a renal angiography cohort. | van Onna M et al. | 15326089 | Not a renal disease focus |
| 1 | Angiotensin I-converting enzyme and angiotensinogen gene interaction and prediction of essential hypertension | Vasku A et al. | 9607178 | Not a renal disease focus |
| 1 | Association between ACE gene polymorphisms and Alzheimer's disease in Han population in Hebei Peninsula | Wang XL et al. | WOS:000412148800122 | Not a renal disease focus |
| 1 | COL4A1 mutations in patients with sporadic late-onset intracerebral hemorrhage | Weng YC et al. | 22522439 | Not a renal disease focus |
| 1 | Increased expression of angiotensin II type 1 receptor (AGTR1) in heart transplant recipients with recurrent rejection. | Yamani MH et al. | 17097490 | Not a renal disease focus |
| 1 | No association between deletion-type angiotensin-converting enzyme gene polymorphism and left-ventricular hypertrophy in hemodialysis patients. | Yildiz A et al. | 10657713 | Not a renal disease focus |
| 1 | Frequencies Of Variants Of Candidate Genes In Different Age-Groups Of Hypertensives | Zee Ryl et al. | 7882587 | Not a renal disease focus |
| 1 | [Renin-angiotensin system genes in chronic glomerulonephritis]. | Buraczynska M et al. | 11865575 | Not English or Spanish |
| 1 | [Association of the renin-angiotensin system gene polymorphism with nephropathy in type II diabetes]. | Buraczynska M et al. | 12476891 | Not English or Spanish |
| 1 | [Genetic predisposition to systemic complications of arterial hypertension in maintenance haemodialysis patients]. | Bzoma B et al. | 19112833 | Not English or Spanish |
| 1 | [Is PstI polymorphism of the angiotensin I converting enzyme gene associated with nephropathy development in non-insulin-dependent diabetes mellitus (preliminary study)]. | Grzeszczak W et al. | 9499204 | Not English or Spanish |
| 1 | [Angiotensin-converting enzyme gene polymorphism and the clinical pathological features and progression in lupus nephritis]. | Guan T et al. | 10436947 | Not English or Spanish |
| 1 | [I/D relationship between polymorphism of ACE gene and progression of chronic glomerulonephritis]. | Kaliev RR et al. | 16078593 | Not English or Spanish |
| 1 | [Association of the complex of polymorphic markers of ACE genes, aldosteron synthetase and endothelial synthetase of nitric oxide with progression of chronic glomerulonephritis]. | Kamysheva ES et al. | 15532370 | Not English or Spanish |

| | | | | |
|---|---|---|---|---|
| 1 | [Angiotensin-converting enyme insertion/deletion polymorphism and blood pressure regulation in type 2 diabetic patients]. | Krajina-Andricevic M et al. | 23120809 | Not English or Spanish |
| 1 | [Polymorphism studies of angiotensin converting enzyme gene in chronic glomerulonephritis]. | Kutyrina IM et al. | 10420452 | Not English or Spanish |
| 1 | [Relationship between serum angiotensin I-converting enzyme activity and diabetic nephropathy in patients with type II diabetes]. | Liao L et al. | 12016801 | Not English or Spanish |
| 1 | [Study on candidate genes of benazepril related cough in Chinese hypertensives]. | Lu J et al. | 12848919 | Not English or Spanish |
| 1 | [Association of alpha-adducin and angiotensin converting enzyme gene polymorphisms with salt-sensitive hypertension and early renal injury]. | Lu LH et al. | 18393230 | Not English or Spanish |
| 1 | [Association between insertion-deletion polymorphism of the angiotensin-converting enzyme gene and development of angiopathies in patients with non-insulin dependent diabetes mellitus from the Chuvash Republic]. | Miloserdova OV et al. | 11234416 | Not English or Spanish |
| 1 | [Relationship between I/D polymorphism of angiotensin I converting enzyme gene and microvascular complications in type 2 diabetic patients]. | Moleda P et al. | 17941464 | Not English or Spanish |
| 1 | [A study on angiotensin-I converting enzyme polymorphism in CAPD patients]. | Nishina M | 9014479 | Not English or Spanish |
| 1 | [Arterial hypertension in glomerulonephritis]. | Oko A et al. | 14974362 | Not English or Spanish |
| 1 | [Association between angiotensin-converting enzyme 2 gene polymorphisms and childhood primary nephrotic syndrome]. | Qiu MY et al. | 25815490 | Not English or Spanish |
| 1 | [Association between ACE gene polymorphism and therapeutic responsiveness of ACEI in diabetic nephropathy]. | Wang L et al. | 10923445 | Not English or Spanish |
| 1 | [Relationship between angiotensin 1 converting enzyme gene polymorphism and diabetic nephropathy]. | Wu S et al. | 9596955 | Not English or Spanish |

| | | | | |
|---|---|---|---|---|
| 1 | [Correlative study between angiotensin-converting enzyme gene polymorphism and hepatorenal syndrome]. | Wu XX et al. | 15698501 | Not English or Spanish |
| 1 | DD genotype of ACE gene in boys: may it be a risk factor for minimal change nephrotic syndrome? | Alasehirli B et al. | 22017506 | Paediatric individuals |
| 1 | Angiotensin-converting enzyme gene insertion/deletion polymorphism and renal damage in childhood uropathies. | al-Eisa A et al. | 10986863 | Paediatric individuals |
| 1 | Angiotensin converting enzyme gene insertion/deletion polymorphism in idiopathic nephrotic syndrome in Kuwaiti Arab children. | Al-Eisa A et al. | 11487079 | Paediatric individuals |
| 1 | Polymorphisms in angiotensin-converting enzyme gene and severity of renal disease in Henoch-Schoenlein patients. Italian Group of Renal Immunopathology. | Amoroso A et al. | 9870486 | Paediatric individuals |
| 1 | Angiotensin converting enzyme gene polymorphism in Asian Indian children with congenital uropathies. | Bajpai M et al. | 14713838 | Paediatric individuals |
| 1 | Late effects on renal glomerular and tubular function in childhood cancer survivors. | Bardi E et al. | 15390293 | Paediatric individuals |
| 1 | Angiotensin-converting enzyme genotype is not a significant genetic risk factor for idiopathic nephrotic syndrome in Croatian children. | Batinc D et al. | 25997642 | Paediatric individuals |
| 1 | HPSE2 mutations in urofacial syndrome, non-neurogenic neurogenic bladder and lower urinary tract dysfunction. | Bulum B et al. | 25924634 | Paediatric individuals |
| 1 | ACE gene polymorphism in Turkish children with nephrotic syndrome. | Celik US et al. | 16825089 | Paediatric individuals |
| 1 | Glycosphingolipid levels in an unusual neurovisceral storage disease characterized by lactosylceramide galactosyl hydrolase deficiency: lactosylceramidosis. | Dawson G | 5016302 | Paediatric individuals |
| 1 | Association of ACE and MDR1 Gene Polymorphisms with Steroid Resistance in Children with Idiopathic Nephrotic Syndrome. | Dhandapani MC et al. | 26154535 | Paediatric individuals |
| 1 | Polymorphisms of the angiotensin converting enzyme and angiotensin II type 1 receptor genes and renal scarring in non-uropathic children with recurrent urinary tract infection. | Ece A et al. | 16109085 | Paediatric individuals |

| | | | | |
|---|---|---|---|---|
| 1 | Is ACE gene polymorphism a risk factor for renal scarring with low-grade reflux? | Erdogan H et al. | 15138870 | Paediatric individuals |
| 1 | ACE gene polymorphism in Egyptian children with idiopathic nephrotic syndrome. | Fahmy ME et al. | 18792483 | Paediatric individuals |
| 1 | Genetic polymorphisms of the renin-angiotensin system and the outcome of focal segmental glomerulosclerosis in children. | Frishberg Y et al. | 9853248 | Paediatric individuals |
| 1 | Angiotensinogen gene T235 variant: a marker for the development of persistent microalbuminuria in children and adolescents with type 1 diabetes mellitus | Gallego PH et al. | 18413222 | Paediatric individuals |
| 1 | Implication of genetic variations in congenital obstructive nephropathy. | Hahn H et al. | 16133060 | Paediatric individuals |
| 1 | Angiotensin-converting enzyme insertion/deletion gene polymorphism in Egyptian children with systemic lupus erythematosus: a possible relation to proliferative nephritis. | Hammad A et al. | 27956582 | Paediatric individuals |
| 1 | ACE gene polymorphism and renal scarring in primary vesicoureteric reflux. | Haszon I et al. | 12478352 | Paediatric individuals |
| 1 | ACE I/D gene polymorphism predicts renal damage in congenital uropathies. | Hohenfellner K et al. | 10452281 | Paediatric individuals |
| 1 | Impact of ACE I/D gene polymorphism on congenital renal malformations. | Hohenfellner K et al. | 11354781 | Paediatric individuals |
| 1 | Significance of ACE genotypes and medical treatments in childhood focal glomerulosclerosis. | Hori C et al. | 11474225 | Paediatric individuals |
| 1 | Impact of common functional polymorphisms in renin angiotensin system genes on the risk of renal parenchymal scarring following childhood urinary tract infection. | Hussein A et al. | 25939993 | Paediatric individuals |
| 1 | Gene polymorphisms of adducin GLY460TRP, ACE I/D, AND AGT M235T in pediatric hypertension patients. | Kaplan I et al. | 25262176 | Paediatric individuals |
| 1 | ACE serum level and I/D gene polymorphism in children with obstructive uropathies and other congenital anomalies of the kidney and urinary tract. | Kostadinova ES et al. | 27206329 | Paediatric individuals |

| | | | | |
|---|---|---|---|---|
| 1 | ACE and AT1 receptor gene polymorphisms and renal scarring in urinary bladder dysfunction | Kostic M et al. | 15179569 | Paediatric individuals |
| 1 | Association of angiotensin converting enzyme and angiotensin type 2 receptor gene polymorphisms with renal damage in posterior urethral valves. | Laksmi NK et al. | 20149750 | Paediatric individuals |
| 1 | Renin-angiotensin system polymorphisms in Taiwanese primary vesicoureteral reflux. | Liu KP et al. | 15045574 | Paediatric individuals |
| 1 | Polymorphisms of renin-angiotensin system genes in childhood IgA nephropathy. | Maruyama K et al. | 11354780 | Paediatric individuals |
| 1 | Angiotensin-Converting Enzyme Gene Polymorphism in Children with Idiopathic Nephrotic Syndrome, Effect on Biopsy Findings. | Monajemzadeh M et al. | 28481137 | Paediatric individuals |
| 1 | ACE I/D gene polymorphism in primary FSGS and steroid-sensitive nephrotic syndrome. | Oktem F et al. | 14986085 | Paediatric individuals |
| 1 | Implications of certain genetic polymorphisms in scarring in vesicoureteric reflux: importance of ACE polymorphism. | Ozen S et al. | 10401028 | Paediatric individuals |
| 1 | Renin-angiotensin system gene polymorphisms: association with susceptibility to Henoch-Schonlein purpura and renal involvement | Ozkaya O et al. | 16521052 | Paediatric individuals |
| 1 | Renin-angiotensin gene polymorphism in children with uremia and essential hypertension. | Papp F et al. | 12579405 | Paediatric individuals |
| 1 | Renin-angiotensin system polymorphisms and renal scarring. | Pardo R et al. | 12579398 | Paediatric individuals |
| 1 | Angiotensin converting enzyme gene polymorphism in Indian children with steroid sensitive nephrotic syndrome. | Patil SJ et al. | 16272677 | Paediatric individuals |
| 1 | Angiotensin I-converting enzyme-gene-polymorphism: Relationship to albumin excretion and blood pressure in pediatric patients with type-I-diabetes mellitus | Pavlovic M et al. | 9354852 | Paediatric individuals |
| 1 | Angiotensin I converting enzyme and angiotensinogen gene polymorphisms related to 24-h blood pressure in paediatric type I diabetes mellitus | Pavlovic M et al. | 9950302 | Paediatric individuals |

| | | | | |
|---|---|---|---|---|
| 1 | Low renin-angiotensin system activity gene polymorphism and dysplasia associated with posterior urethral valves. | Peruzzi L et al. | 16006956 | Paediatric individuals |
| 1 | Modification of epigenetic patterns in low birth weight children: importance of hypomethylation of the ACE gene promoter. | Rangel M et al. | 25170764 | Paediatric individuals |
| 1 | Angiotensin-converting enzyme and angiotensin type 2 receptor gene genotype distributions in Italian children with congenital uropathies | Rigoli L et al. | 15470205 | Paediatric individuals |
| 1 | Effect of angiotensin-converting enzyme gene insertion/deletion polymorphism on steroid resistance in Egyptian children with idiopathic nephrotic syndrome. | Saber-Ayad M et al. | 20418353 | Paediatric individuals |
| 1 | ACE gene polymorphism in children with nephrotic syndrome in the Indonesian population. | Sasongko TH et al. | 16421456 | Paediatric individuals |
| 1 | Polymorphisms of the TNF-alpha and ACE genes, and renal scarring in infants with urinary tract infection. | Savvidou A et al. | 20022049 | Paediatric individuals |
| 1 | ACE gene insertion/deletion polymorphism and renal scarring in children with urinary tract infections. | Sekerli E et al. | 19603195 | Paediatric individuals |
| 1 | ACE gene insertion/deletion polymorphism in childhood idiopathic nephrotic syndrome. | Serdaroglu E et al. | 16208534 | Paediatric individuals |
| 1 | Association of the ACE-II genotype with the risk of nephrotic syndrome in Pakistani children. | Shahid S et al. | 22033511 | Paediatric individuals |
| 1 | ACE gene polymorphism in childhood IgA nephropathy: association with clinicopathologic findings. | Tanaka R et al. | 9590186 | Paediatric individuals |
| 1 | Role of platelet-activating factor acetylhydrolase gene mutation in Japanese childhood IgA nephropathy. | Tanaka R et al. | 10430976 | Paediatric individuals |
| 1 | Angiotensin-converting enzyme gene polymorphism in children with idiopathic nephrotic syndrome. | Tsai IJ et al. | 16645262 | Paediatric individuals |
| 1 | Angiotensin-converting enzyme gene insertion/deletion polymorphism in children with Henoch-Schonlein purpua nephritis. | Zhou J et al. | 15315169 | Paediatric individuals |
| 1 | Estimation of the relationship between the polymorphisms of selected genes: ACE, AGTR1, TGFÎ²1 and GNB3 with the occurrence of primary vesicoureteral reflux. | Zyczkowski M et al. | 27988909 | Paediatric individuals |

| | | | | |
|---|---|---|---|---|
| 1 | Long-term renoprotective effects of losartan in diabetic nephropathy: interaction with ACE insertion/deletion genotype? | Andersen S et al. | 12716812 | Pharmaceutical drug focus |
| 1 | High serum enalaprilat in chronic renal failure | Elung-Jensen T et al. | 11881130 | Pharmaceutical drug focus |
| 1 | Randomized placebo-controlled trial of perindopril in normotensive, normoalbuminuric patients with type 1 diabetes mellitus. | Kvetny J et al. | 11181984 | Pharmaceutical drug focus |
| 1 | The influence of the ACE ( I/D) polymorphism on systemic and renal vascular responses to angiotensins in normotensive, normoalbuminuric Type 1 diabetes mellitus. | Luik PT et al. | 12856080 | Pharmaceutical drug focus |
| 1 | Renin-angiotensin system polymorphisms and hemoglobin level in renal allografts: a comparative study between losartan and enalapril. | Noroozianavval M et al. | 17524880 | Pharmaceutical drug focus |
| 1 | ACE gene polymorphism and losartan treatment in type 2 diabetic patients with nephropathy | Parving HH et al. | 18199798 | Pharmaceutical drug focus |
| 1 | Effect of angiotensin-converting enzyme (ACE) gene polymorphism on progression of renal disease and the influence of ACE inhibition in IDDM patients: findings from the EUCLID Randomized Controlled Trial. EURODIAB Controlled Trial of Lisinopril in IDDM. | Penno G et al. | 9726242 | Pharmaceutical drug focus |
| 1 | Enalapril and losartan affect lipid peroxidation in renal transplant recipients with renin-angiotensin system polymorphisms. | Rashtchizadeh N et al. | 17222813 | Pharmaceutical drug focus |
| 1 | Chronic proteinuric nephropathies. II. Outcomes and response to treatment in a prospective cohort of 352 patients: differences between women and men in relation to the ACE gene polymorphism. Gruppo Italiano di Studi Epidemologici in Nefrologia (Gisen) | Ruggenenti P et al. | 10616844 | Pharmaceutical drug focus |
| 2 | The DD genotype of the ACE gene polymorphism is associated with diabetic nephropathy in the type-1 diabetics. | Azar ST et al. | 11428725 | Incorrect patient group |

| | | | | |
|---|---|---|---|---|
| 2 | Genetic variation at the ACE gene is associated with persistent microalbuminuria and severe nephropathy in type 1 diabetes: the DCCT/EDIC Genetics Study. | Boright AP et al. | 15793268 | Incorrect patient group |
| 2 | ACE, PAI-1, decorin and Werner helicase genes are not associated with the development of renal disease in European patients with type 1 diabetes. | De Cosmo S et al. | 10495473 | Incorrect patient group |
| 2 | Genetic Predisposition To Diabetic Nephropathy - Evidence For A Role Of The Angiotensin I-Converting Enzyme Gene | Doria A et al. | 7909524 | Incorrect patient group |
| 2 | Polymorphism of angiotensin converting enzyme, angiotensinogen, and angiotensin II type 1 receptor genes and end-stage renal failure in IgA nephropathy: IGARAS - A study of 274 men | Frimat L et al. | 11053482 | Incorrect patient group |
| 2 | Polymorphism of the angiotensin I-converting enzyme gene in diabetic nephropathy in type II diabetic patients with proliferative retinopathy | Hanyu O et al. | 9509566 | Incorrect patient group |
| 2 | The angiotensin I-converting enzyme (ACE) locus is strongly associated with age and duration of diabetes in patients with type I diabetes | Hibberd ML et al. | 9025006 | Incorrect patient group |
| 2 | Hypertensive nephropathy and the gene for angiotensin-converting enzyme. | Kario K et al. | 9081678 | Incorrect patient group |
| 2 | Effect of ACE gene on diabetic nephropathy in NIDDM patients with insulin resistance. | Kuramoto N et al. | 10023638 | Incorrect patient group |
| 2 | Relationships between angiotensin I converting enzyme gene polymorphism and renal complications in Korean IDDM patients. | Oh TG et al. | 8854649 | Incorrect patient group |
| 2 | The frequency of factor V Leiden mutation, ACE gene polymorphism, serum ACE activity and response to ACE inhibitor and angiotensin II receptor antagonist drugs in Iranians type II diabetic patients with microalbuminuria. | Rahimi Z et al. | 20853144 | Incorrect patient group |
| 2 | Interaction of MTHFR 1298C with ACE D allele augments the risk of diabetic nephropathy in Western Iran. | Rahimi Z et al. | 21942443 | Incorrect patient group |

| | | | | |
|---|---|---|---|---|
| 2 | The ACE insertion/deletion polymorphism has no influence on progression of renal function loss in autosomal dominant polycystic kidney disease. | van Dijk MA et al. | 10831637 | Incorrect patient group |
| 2 | Genetic polymorphisms of renin-angiotensin system and progression of interstitial nephritis. | Buraczynska M et al. | 12898858 | No data |
| 2 | Frequency of angiotensin-converting enzyme gene polymorphism in Turkish type 2 diabetic patients. | Degirmenci I et al. | 16178979 | No data |
| 2 | Association of the DD genotype and development of Japanese type 2 diabetic nephropathy. | Gohda T et al. | 11770799 | No data |
| 2 | Angiotensin I converting enzyme and angiotensinogen gene polymorphisms in non-insulin-dependent diabetes mellitus. Lack of relationship with diabetic nephropathy and retinopathy in a Caucasian Mediterranean population | Gutierrez C et al. | 9258285 | No data |
| 2 | Association between angiotensin-converting enzyme gene polymorphisms and diabetic nephropathy: case-control, haplotype, and family-based study in three European populations. | Hadjadj S et al. | 17376814 | No data |
| 2 | Prognostic value of the insertion/deletion polymorphism of the ACE gene in type 2 diabetic subjects: results from the Non-insulin-dependent Diabetes, Hypertension, Microalbuminuria or Proteinuria, Cardiovascular Events, and Ramipril (DIABHYCAR), Diabete de type 2, Nephropathie et Genetique (DIAB2NEP... | Hadjadj S et al. | 18523145 | No data |
| 2 | Angiotensin-converting enzyme gene polymorphism in non-insulin dependent diabetes mellitus and its relationship with diabetic nephropathy. | Jeffers BW et al. | 9264004 | No data |
| 2 | Genetic polymorphisms of the renin-angiotensin-aldosterone system in end-stage renal disease. | Lovati E et al. | 11422735 | No data |
| 2 | Effects of the genetic polymorphisms of the renin-angiotensin system on focal segmental glomerulosclerosis. | Luther Y et al. | 14610337 | No data |
| 2 | The renin--angiotensin system gene polymorphisms and clinicopathological correlations in IgA nephropathy. | Ong-Ajyooth S et al. | 10511770 | No data |

| | | | | |
|---|---|---|---|---|
| 2 | Combinational effect of genes for the renin-angiotensin system in conferring susceptibility to diabetic nephropathy. | Osawa N et al. | 17143591 | No data |
| 2 | Association of a uteroglobin polymorphism with rate of progression in patients with IgA nephropathy. | Szelestei T et al. | 10977777 | No data |
| 2 | Lack of synergism between long-term poor glycaemic control and three gene polymorphisms of the renin angiotensin system on risk of developing diabetic nephropathy in type I diabetic patients. | Tarnow L et al. | 10907125 | No data |
| 2 | ACE gene polymorphism and disease progression of IgA nephropathy in Asians in Singapore. | Lau YK et al. | 12119485 | Overlap in patient group |
| 2 | Polymorphism of renin-angiotensin system genes in IgA nephropathy | Woo KT et al. | 15504143 | Overlap in patient group |
| 3 | Angiotensin-converting enzyme (ACE) serum levels and gene polymorphism in Egyptian patients with systemic lupus erythematosus. | Abbas D et al. | 21976404 | < 3 populations reported per SNP |
| 3 | Association of an insertion polymorphism of angiotensin-converting enzyme gene with the activity of lupus nephritis. | Akai Y et al. | 10099886 | < 3 populations reported per SNP |
| 3 | Clinical impact of an angiotensin I-converting enzyme insertion/deletion and kinin B2 receptor +9/-9 polymorphisms in the prognosis of renal transplantation | Amorim et al. | 23362199 | < 3 populations reported per SNP |
| 3 | Lack of association between the angiotensin-converting enzyme gene (I/D) polymorphism and diabetic nephropathy in Tunisian type 2 diabetic patients. | Arfa I et al. | 18404607 | < 3 populations reported per SNP |
| 3 | Polymorphism of the angiotensin-converting enzyme gene in end-stage renal failure patients. | Aucella F et al. | 10773756 | < 3 populations reported per SNP |
| 3 | Polymorphism of the renin–angiotensin–aldosterone system in patients with chronic allograft dysfunction | Ayed K et al. | 16635753 | < 3 populations reported per SNP |
| 3 | The presence of allele D of angiotensin-converting enzyme polymorphism is associated with diabetic nephropathy in patients with less than 10 years duration of Type 2 diabetes. | Canani LH et al. | 16108844 | < 3 populations reported per SNP |

| | | | | |
|---|---|---|---|---|
| 3 | Identification of specific angiotensin-converting enzyme variants and haplotypes that confer risk and protection against type 2 diabetic nephropathy. | Ezzidi I et al. | 19787680 | < 3 populations reported per SNP |
| 3 | Genetic polymorphisms of the renin-angiotensin-aldosterone system and renal insufficiency in essential hypertension | Fabris B et al. | 15662219 | < 3 populations reported per SNP |
| 3 | Angiotensin converting enzyme gene I/D polymorphism in essential hypertension and nephroangiosclerosis. | Fernández-Llama P et al. | 9607207 | < 3 populations reported per SNP |
| 3 | DNA polymorphisms in the ACE gene, serum ACE activity and the risk of nephropathy in insulin-dependent diabetes mellitus | Freire MBS et al. | 9794558 | < 3 populations reported per SNP |
| 3 | Association between genetic polymorphisms of ACE & eNOS and diabetic nephropathy. | Huo P et al. | 25227524 | < 3 populations reported per SNP |
| 3 | Genetic Clues To The Etiology Of Balkan Endemic Nephropathy: Investigating The Role Of Ace And At1R Polymorphisms | Krcunovic Z et al. | WOS:000287217500011 | < 3 populations reported per SNP |
| 3 | Angiotensin-I Converting Enzyme Polymorphism and Diabetic Nephropathy in North India | Kumar A et al. | NA | < 3 populations reported per SNP |
| 3 | Angiotensin-converting enzyme gene polymorphism in patients with minimal-change nephrotic syndrome and focal segmental glomerulosclerosis. | Lee DY et al. | 9434071 | < 3 populations reported per SNP |
| 3 | Association of the genetic polymorphisms of the ACE gene and the eNOS gene with lupus nephropathy in northern Chinese population. | Li X et al. | 20540812 | < 3 populations reported per SNP |
| 3 | Genes involved in the regulation of vascular homeostasis determine renal survival rate in patients with chronic glomerulonephritis | Litovkina O et al. | 24727057 | < 3 populations reported per SNP |
| 3 | Contribution of genetic polymorphism in the renin-angiotensin system to the development of renal complications in insulin-dependent diabetes | Marre M et al. | 9120002 | < 3 populations reported per SNP |
| 3 | Influence of the alpha-adducin and ACE gene polymorphism on the progression of autosomal-dominant polycystic kidney disease. | Merta M et al. | 12697976 | < 3 populations reported per SNP |

| | | | | |
|---|---|---|---|---|
| 3 | Genetic polymorphism of renin-angiotensin system is not associated with diabetic vascular complications in Japanese subjects with long-term insulin dependent diabetes mellitus. | Miura J et al. | 10499884 | < 3 populations reported per SNP |
| 3 | The effect of polymorphisms in the renin-angiotensin-aldosterone system on diabetic nephropathy risk. | Möllsten A et al. | 18413189 | < 3 populations reported per SNP |
| 3 | [Angiotensin-1 converting enzyme insertion/deletion gene polymorphism in a Mexican population with diabetic nephropathy]. | Ortega-Pierres LE et al. | 17570179 | < 3 populations reported per SNP |
| 3 | Influence of genetic variability at the ACE locus in intron 16 on Diabetic Nephropathy in T1DM patients. | Parchwani DN et al. | 26214998 | < 3 populations reported per SNP |
| 3 | Renin-angiotensin system gene polymorphisms predict the progression to renal insufficiency among Asians with lupus nephritis. | Parsa A et al. | 15789057 | < 3 populations reported per SNP |
| 3 | Chronic renal insufficiency among Asian Indians with type 2 diabetes: I. Role of RAAS gene polymorphisms | Prasad P et al. | WOS:000238369400001 | < 3 populations reported per SNP |
| 3 | Influence of angiotensin converting enzyme (ACE) gene rs4362 polymorphism on the progression of kidney failure in patients with autosomal dominant polycystic kidney disease (ADPKD) | Ramanathan G et al. | 27748299 | < 3 populations reported per SNP |
| 3 | Genetic variants of ACE (Insertion/Deletion) and AGT (M268T) genes in patients with diabetes and nephropathy. | Shaikh R et al. | 24737640 | < 3 populations reported per SNP |
| 3 | Polymorphisms of the renin-angiotensin system genes in Brazilian patients with lupus nephropathy. | Sprovieri SR et al. | 15934435 | < 3 populations reported per SNP |
| 3 | Gene polymorphisms of angiotensin-converting enzyme and angiotensin II Type 1 receptor among chronic kidney disease patients in a Chinese population | Su SL et al. | 22147663 | < 3 populations reported per SNP |
| 3 | Genetic risk factors for renal failure among north Indian ESRD patients. | Tripathi G et al. | 18242170 | < 3 populations reported per SNP |
| 3 | Association between angiotensin converting enzyme gene polymorphism and clinical features in autosomal dominant polycystic kidney disease. | Uemasu J et al. | 9180368 | < 3 populations reported per SNP |
| 3 | The DD genotype of the ACE gene polymorphism is associated with progression of diabetic nephropathy to end stage renal failure in IDDM. | Vleming LJ et al. | 10099885 | < 3 populations reported per SNP |

**Supplementary Table S3b:** Excluded studies from the *ACE2* search

*Exclusion stage relates to the stage at which the article was removed as shown in the flow diagram (Supplementary Figure S1b)

| Exclusion Stage | Title | Authors | Pubmed ID or WoS ID if Pubmed ID not available | Reason |
|---|---|---|---|---|
| 1 | Angiotensin converting enzyme gene I/D polymorphism in essential hypertension and nephroangiosclerosis | Fernandez-Llama P et al. | 9607207 | No data for ACE2 |
| 1 | Genetic predisposition to Balkan endemic nephropathy: ability to hydroxylate debrisoquine as a host risk factor. | Nikolov IG et al. | 1820343 | No data for ACE2 |
| 1 | Immunoglobulin C mu gene restriction fragment length polymorphisms associated with chronic renal failure. | Demaine AG et al. | 2985495 | No data for ACE2 |
| 1 | Genetic polymorphism of C3 and Bf in IgA nephropathy. | Rambausek M et al. | 3118258 | No data for ACE2 |
| 1 | Factor B subtypes in Japanese patients with IgA nephropathy and with idiopathic membranous nephropathy. | Nishimukai H et al. | 3272818 | No data for ACE2 |
| 1 | Major-histocompatibility-complex extended haplotypes in membranoproliferative glomerulonephritis. | Welch TR et al. | 3458025 | No data for ACE2 |
| 1 | A simultaneous study of the polymorphism of five proteins in the serum and the urine of nephrotic patients. | Papacostas S et al. | 6688659 | No data for ACE2 |
| 1 | Identification of mutants of pyruvate kinase from red blood cells by means of trypsinization, electrophoresis, kinetic properties and immunological methods. | Jacobasch G et al. | 7315101 | No data for ACE2 |
| 1 | Angiotensin-converting enzyme polymorphism and development of diabetic nephropathy in non-insulin-dependent diabetes mellitus. | Mizuiri S et al. | 7477652 | No data for ACE2 |
| 1 | No association of converting enzyme insertion/deletion polymorphism with immunoglobulin A glomerulonephritis. | Schmidt S et al. | 7485124 | No data for ACE2 |
| 1 | Protein loss and genetic polymorphism of apolipoprotein(a) modulate serum lipoprotein(a) in CAPD patients. | Wanner C et al. | 7724034 | No data for ACE2 |

| | | | | |
|---|---|---|---|---|
| 1 | Lack of relationship between an insertion/deletion polymorphism in the angiotensin I-converting enzyme gene and diabetic nephropathy and proliferative retinopathy in IDDM patients. | Tarnow L et al. | 7729604 | No data for ACE2 |
| 1 | Role of glycaemic control in development of microalbuminuria in patients with insulin dependent diabetes. | Powrie JK et al. | 7819935 | No data for ACE2 |
| 1 | The N-acetyltransferase (NAT) gene: an early risk marker for diabetic nephropathy in Japanese type 2 diabetic patients? | Neugebauer S et al. | 7851073 | No data for ACE2 |
| 1 | Genetic predisposition to diabetic nephropathy. Evidence for a role of the angiotensin I--converting enzyme gene. | Doria A et al. | 7909524 | No data for ACE2 |
| 1 | Relationships between angiotensin I converting enzyme gene polymorphism, plasma levels, and diabetic retinal and renal complications. | Marre M et al. | 8314010 | No data for ACE2 |
| 1 | Elevated plasma concentrations of lipoprotein(a) in patients with end-stage renal disease are not related to the size polymorphism of apolipoprotein(a). | Dieplinger H et al. | 8432847 | No data for ACE2 |
| 1 | Polymorphism of the angiotensin converting enzyme gene and clinical aspects of IgA nephropathy. | Yorioka T et al. | 8529313 | No data for ACE2 |
| 1 | Association between angiotensin-converting-enzyme gene polymorphism and failure of renoprotective therapy. | van Essen GG et al. | 8538349 | No data for ACE2 |
| 1 | Association analyses of the polymorphisms of angiotensin-converting enzyme and angiotensinogen genes with diabetic nephropathy in Japanese non-insulin-dependent diabetics. | Ohno T et al. | 8596493 | No data for ACE2 |
| 1 | Association between a polymorphism in the angiotensin-converting enzyme gene and microvascular complications in Japanese patients with NIDDM. | Doi Y et al. | 8720609 | No data for ACE2 |
| 1 | Angiotensin-converting enzyme polymorphism in patients with terminal renal failure. | Schmidt A et al. | 8785402 | No data for ACE2 |
| 1 | Relationships between angiotensin I converting enzyme gene polymorphism and renal complications in Korean IDDM patients. | Oh TG et al. | 8854649 | No data for ACE2 |

| | | | | |
|---|---|---|---|---|
| 1 | The angiotensin I-converting enzyme (ACE) locus is strongly associated with age and duration of diabetes in patients with type I diabetes. | Hibberd ML et al. | 9025006 | No data for ACE2 |
| 1 | Hypertensive nephropathy and the gene for angiotensin-converting enzyme. | Kario K et al. | 9081678 | No data for ACE2 |
| 1 | Evaluation of risk factors for the development of nephropathy in patients with IDDM: insertion/deletion angiotensin converting enzyme gene polymorphism, hypertension and metabolic control. | Barnas U et al. | 9084972 | No data for ACE2 |
| 1 | Association between angiotensin converting enzyme gene polymorphism and clinical features in autosomal dominant polycystic kidney disease. | Uemasu J et al. | 9180368 | No data for ACE2 |
| 1 | Angiotensin-converting enzyme genotype and renal allograft survival. | Beige J et al. | 9259361 | No data for ACE2 |
| 1 | Association of angiotensinogen gene T235 variant with progression of immunoglobin A nephropathy in Caucasian patients. | Pei Y et al. | 9259580 | No data for ACE2 |
| 1 | Angiotensin-converting enzyme gene polymorphism in non-insulin dependent diabetes mellitus and its relationship with diabetic nephropathy. | Jeffers BW et al. | 9264004 | No data for ACE2 |
| 1 | Angiotensin I converting enzyme gene polymorphism and diabetic nephropathy in type II diabetes. | Schmidt S et al. | 9269698 | No data for ACE2 |
| 1 | Deletion insertion polymorphism of the angiotensin converting enzyme gene and progression of diabetic nephropathy. | Bjorck S et al. | 9269704 | No data for ACE2 |
| 1 | Genetic regulation of the impaired immune response to hepatitis-B vaccine associated with low TCR density in end stage renal disease patients: contribution of complement C4 and factor B alleles. | Kramer J et al. | 9334852 | No data for ACE2 |
| 1 | Gene-polymorphisms of angiotensin converting enzyme and endothelial nitric oxide synthase in patients with primary glomerulonephritis. | Burg M et al. | 9352153 | No data for ACE2 |
| 1 | Angiotensin-converting enzyme gene polymorphism in patients with minimal-change nephrotic syndrome and focal segmental glomerulosclerosis. | Lee DY et al. | 9434071 | No data for ACE2 |
| 1 | Genetic variants of microsomal metabolism and susceptibility to hydrocarbon-associated glomerulonephritis. | Pai P et al. | 9474350 | No data for ACE2 |

| | | | | |
|---|---|---|---|---|
| 1 | Polymorphism of the angiotensin I-converting enzyme gene in diabetic nephropathy in type II diabetic patients with proliferative retinopathy. | Hanyu O et al. | 9509566 | No data for ACE2 |
| 1 | Identification of human plasma kallikrein gene polymorphisms and evaluation of their role in end-stage renal disease. | Yu H et al. | 9535413 | No data for ACE2 |
| 1 | Relationship of the angiotensin-converting enzyme gene polymorphism to glucose intolerance, insulin resistance, and hypertension in NIDDM. | Huang XH et al. | 9544854 | No data for ACE2 |
| 1 | Angiotensin-converting-enzyme insertion/deletion genotype and long-term renal allograft survival. | Beige J et al. | 9550656 | No data for ACE2 |
| 1 | A new polymorphic restriction site in the human 11 beta-hydroxysteroid dehydrogenase type 2 gene. | Smolenicka Z et al. | 9589699 | No data for ACE2 |
| 1 | Angiotensin I converting enzyme gene polymorphisms in systemic lupus erythematosus: decreased prevalence of DD genotype in African American patients. | Tassiulas IO et al. | 9710341 | No data for ACE2 |
| 1 | Angiotensin I-converting enzyme gene polymorphisms: relationship to nephropathy in patients with non-insulin dependent diabetes mellitus. | Grzeszczak W et al. | 9727375 | No data for ACE2 |
| 1 | The serum paraoxonase activity in patients with chronic renal failure and hyperlipidemia. | Paragh G et al. | 9736814 | No data for ACE2 |
| 1 | Homocysteine, B vitamins, and vascular-access thrombosis in patients treated with hemodialysis. | Tamura T et al. | 9740165 | No data for ACE2 |
| 1 | DNA polymorphisms in the ACE gene, serum ACE activity and the risk of nephropathy in insulin-dependent diabetes mellitus. | Freire MB et al. | 9794558 | No data for ACE2 |
| 1 | Insertion/deletion polymorphism in intron 16 of the ACE gene and left ventricular hypertrophy in patients with end-stage renal disease. | Osono E et al. | 9820440 | No data for ACE2 |
| 1 | The C825T polymorphism in the human G-protein beta3 subunit gene is not associated with diabetic nephropathy in Type I diabetes mellitus. | Fogarty DG et al. | 9833937 | No data for ACE2 |
| 1 | Effect of ACE gene on diabetic nephropathy in NIDDM patients with insulin resistance. | Kuramoto N et al. | 10023638 | No data for ACE2 |
| 1 | The DD genotype of the ACE gene polymorphism is associated with progression of diabetic nephropathy to end stage renal failure in IDDM. | Vleming LJ et al. | 10099885 | No data for ACE2 |

| | | | | |
|---|---|---|---|---|
| 1 | Increased frequency of G-protein beta 3-subunit 825 T allele in dialyzed patients with type 2 diabetes. | Bluthner M et al. | 10200987 | No data for ACE2 |
| 1 | Lack of association of angiotensin-converting enzyme (DD/II) and angiotensinogen M235T gene polymorphism with renal function among Chinese patients with type II diabetes. | Wong TY et al. | 10352194 | No data for ACE2 |
| 1 | Angiotensin I-converting enzyme genotype significantly affects progression of IgA glomerulonephritis in an italian population. | Stratta P et al. | 10352195 | No data for ACE2 |
| 1 | Lipoprotein(a) and apolipoprotein(a) isoforms and proteinuria in patients with moderate renal failure. | Sechi LA et al. | 10469373 | No data for ACE2 |
| 1 | ACE, PAI-1, decorin and Werner helicase genes are not associated with the development of renal disease in European patients with type 1 diabetes. | De Cosmo S et al. | 10495473 | No data for ACE2 |
| 1 | The renin--angiotensin system gene polymorphisms and clinicopathological correlations in IgA nephropathy. | Ong-Ajyooth S et al. | 10511770 | No data for ACE2 |
| 1 | Effect of a polymorphism of endothelial nitric oxide synthase gene in Japanese patients with IgA nephropathy. | Morita T et al. | 10543322 | No data for ACE2 |
| 1 | Endothelial nitric oxide synthase gene polymorphism in intron 4 affects the progression of renal failure in non-diabetic renal diseases. | Wang Y et al. | 10570094 | No data for ACE2 |
| 1 | Risk factors for the progression of microalbuminuria in Japanese type 2 diabetic patients--a 10 year follow-up study. | Oue T et al. | 10580616 | No data for ACE2 |
| 1 | Chronic proteinuric nephropathies. II. Outcomes and response to treatment in a prospective cohort of 352 patients: differences between women and men in relation to the ACE gene polymorphism. Gruppo Italiano di Studi Epidemologici in Nefrologia (Gisen) | Ruggenenti P et al. | 10616844 | No data for ACE2 |
| 1 | Risk of advanced diabetic nephropathy in type 1 diabetes is associated with endothelial nitric oxide synthase gene polymorphism. | Zanchi A et al. | 10652017 | No data for ACE2 |
| 1 | HDL cholesterol and TaqIB cholesteryl ester transfer protein gene polymorphism in renal transplant recipients. | Radeau T et al. | 10754410 | No data for ACE2 |

| | | | | |
|---|---|---|---|---|
| 1 | Polymorphism of the angiotensin-converting enzyme gene in end-stage renal failure patients. | Aucella F et al. | 10773756 | No data for ACE2 |
| 1 | The ACE insertion/deletion polymorphism has no influence on progression of renal function loss in autosomal dominant polycystic kidney disease. | van Dijk MA et al. | 10831637 | No data for ACE2 |
| 1 | Increased frequency of angiotensin-converting enzyme DD genotype in patients with type 2 diabetes in Taiwan. | Hsieh MC et al. | 10862639 | No data for ACE2 |
| 1 | Association of the nitric oxide synthase gene polymorphism with an increased risk for progression to diabetic nephropathy in type 2 diabetes. | Neugebauer S et al. | 10868974 | No data for ACE2 |
| 1 | Lack of synergism between long-term poor glycaemic control and three gene polymorphisms of the renin angiotensin system on risk of developing diabetic nephropathy in type I diabetic patients. | Tarnow L et al. | 10907125 | No data for ACE2 |
| 1 | Differential expression of cyclin-dependent kinase inhibitors in human glomerular disease: role in podocyte proliferation and maturation. | Shankland SJ et al. | 10916090 | No data for ACE2 |
| 1 | G-Protein beta(3) subunit C825T variant, nephropathy and hypertension in patients with type 2 (Non-insulin-dependent) diabetes mellitus. | Zychma MJ et al. | 10970984 | No data for ACE2 |
| 1 | Association of a uteroglobin polymorphism with rate of progression in patients with IgA nephropathy. | Szelestei T et al. | 10977777 | No data for ACE2 |
| 1 | Deregulated platelet-activating factor levels and acetylhydrolase activity in patients with idiopathic IgA nephropathy. | Denizot Y et al. | 10978389 | No data for ACE2 |
| 1 | Structural analysis of the 11beta-hydroxysteroid dehydrogenase type 2 gene in end-stage renal disease. | Zaehner T et al. | 11012876 | No data for ACE2 |
| 1 | Polymorphism of angiotensin converting enzyme, angiotensinogen, and angiotensin II type 1 receptor genes and end-stage renal failure in IgA nephropathy: IGARAS--a study of 274 Men. | Frimat L et al. | 11053482 | No data for ACE2 |
| 1 | Genetic analysis of nitric oxide and endothelin in end-stage renal disease. | Freedman BI et al. | 11071967 | No data for ACE2 |
| 1 | Angiotensinogen M235T and chymase gene CMA/B polymorphisms are not associated with nephropathy in type II diabetes. | Zychma MJ et al. | 11096141 | No data for ACE2 |

| | | | | |
|---|---|---|---|---|
| 1 | Association between a variant in the 11 beta-hydroxysteroid dehydrogenase type 2 gene and primary hypertension. | Melander O et al. | 11114699 | No data for ACE2 |
| 1 | Polymorphisms of human paraoxonase 1 gene (PON1) and susceptibility to diabetic nephropathy in type I diabetes mellitus. | Araki S et al. | 11151764 | No data for ACE2 |
| 1 | Posttransplantation relapse of FSGS is characterized by glomerular epithelial cell transdifferentiation. | Bariety J et al. | 11158216 | No data for ACE2 |
| 1 | Membranous nephropathy, hydrocarbon exposure and genetic variants of hydrocarbon detoxification. | Gradden CW et al. | 11181983 | No data for ACE2 |
| 1 | Albuminuria and the renin-angiotensin system gene polymorphisms in type-2-diabetic and in normoglycemic hypertensive Chinese. | Thomas GN et al. | 11200871 | No data for ACE2 |
| 1 | Paraoxonase2 polymorphisms are associated with nephropathy in Type II diabetes. | Pinizzotto M et al. | 11206400 | No data for ACE2 |
| 1 | ACE gene polymorphism and long-term renal graft function. | Viklicky O et al. | 11239522 | No data for ACE2 |
| 1 | The C825T polymorphism in the G-protein beta3 subunit gene and diabetic complications in IDDM patients. | Shcherbak NS et al. | 11322658 | No data for ACE2 |
| 1 | Polymorphisms in the hANP (human atrial natriuretic peptide) gene, albuminuria, and hypertension. | Nannipieri M et al. | 11408388 | No data for ACE2 |
| 1 | Genetic polymorphisms of the renin-angiotensin-aldosterone system in end-stage renal disease. | Lovati E et al. | 11422735 | No data for ACE2 |
| 1 | The DD genotype of the ACE gene polymorphism is associated with diabetic nephropathy in the type-1 diabetics. | Azar ST et al. | 11428725 | No data for ACE2 |
| 1 | Contribution of gene polymorphisms in the renin-angiotensin system to macroangiopathy in patients with diabetic nephropathy. | Wong TY et al. | 11431175 | No data for ACE2 |
| 1 | Catalase/superoxide dismutase (SOD) and catalase/paraoxonase (PON) ratios may implicate poor glycemic control. | Sozmen EY et al. | 11440784 | No data for ACE2 |
| 1 | Serum paraoxonase and arylesterase activities in hemodialysis patients. | Itahara T et al. | 11480456 | No data for ACE2 |
| 1 | Influence of Bsml vitamin D receptor gene polymorphism on the response to a single bolus of calcitrol in hemodialysis patients. | Marco MP et al. | 11522087 | No data for ACE2 |

| | | | | |
|---|---|---|---|---|
| 1 | Is there a role of angiotensin-converting enzyme gene polymorphism in the failure of arteriovenous femoral shunts for hemodialysis? | Isbir CS et al. | 11525534 | No data for ACE2 |
| 1 | Endothelial nitric oxide synthase intron 4 polymorphism influences the progression of renal disease. | Asakimori Y et al. | 11549906 | No data for ACE2 |
| 1 | Dinucleotide repeat polymorphism of matrix metalloproteinase-9 gene is associated with diabetic nephropathy. | Maeda S et al. | 11576356 | No data for ACE2 |
| 1 | Angiotensin-converting enzyme gene polymorphism and microvascular complications in Turkish type 2 diabetic patients. | Araz M et al. | 11640993 | No data for ACE2 |
| 1 | Effect of human OGG1 1245C-->G gene polymorphism on 8-hydroxy-2'-deoxyguanosine levels of leukocyte DNA among patients undergoing chronic hemodialysis. | Tarng DC et al. | 11675410 | No data for ACE2 |
| 1 | Association of the DD genotype and development of Japanese type 2 diabetic nephropathy. | Gohda T et al. | 11770799 | No data for ACE2 |
| 1 | Different impact of deletion polymorphism of gene on the risk of renal and coronary artery disease. | Olivieri O et al. | 11791024 | No data for ACE2 |
| 1 | Gene mutations in lymphoproliferative disorders of T and NK/T cell phenotypes developing in renal transplant patients. | Hoshida Y et al. | 11896204 | No data for ACE2 |
| 1 | Association of a functional inducible nitric oxide synthase promoter variant with complications in type 2 diabetes. | Morris BJ et al. | 11907646 | No data for ACE2 |
| 1 | Association studies between the HSD11B2 gene (encoding human 11beta-hydroxysteroid dehydrogenase type 2), type 1 diabetes mellitus and diabetic nephropathy. | Lavery GG et al. | 11916625 | No data for ACE2 |
| 1 | Polymorphism in ecto-nucleotide pyrophosphatase/phosphodiesterase 1 gene (ENPP1/PC-1) and early development of advanced diabetic nephropathy in type 1 diabetes. | Canani LH et al. | 11916943 | No data for ACE2 |
| 1 | Role of the alpha-adducin genotype on renal disease progression. | Nicod J et al. | 11918733 | No data for ACE2 |
| 1 | Hypertension after renal transplantation and polymorphism of genes involved in essential hypertension: ACE, AGT, AT1 R and ecNOS. | Basset el-EA et al. | 11926202 | No data for ACE2 |

| | | | | |
|---|---|---|---|---|
| 1 | ACE gene insertion/deletion polymorphism associated with 1998 World Health Organization definition of metabolic syndrome in Chinese type 2 diabetic patients. | Lee YJ et al. | 12032106 | No data for ACE2 |
| 1 | Angiotensin-converting enzyme gene polymorphism and vascular manifestations in Korean patients with SLE. | Uhm WS et al. | 12043886 | No data for ACE2 |
| 1 | Parathyroid hormone gene polymorphism and secondary hyperparathyroidism in hemodialysis patients. | Gohda T et al. | 12046039 | No data for ACE2 |
| 1 | Endothelial nitric oxide synthase affects the progression of autosomal dominant polycystic kidney disease. | Reiterova J et al. | 12077489 | No data for ACE2 |
| 1 | ACE gene polymorphism and disease progression of IgA nephropathy in Asians in Singapore. | Lau YK et al. | 12119485 | No data for ACE2 |
| 1 | T(-786)-->C polymorphism of the endothelial nitric oxide synthase gene influences the progression of renal disease. | Asakimori Y et al. | 12138283 | No data for ACE2 |
| 1 | The role of PC-1 and ACE genes in diabetic nephropathy in type 1 diabetic patients: evidence for a polygenic control of kidney disease progression. | De Cosmo S et al. | 12147786 | No data for ACE2 |
| 1 | Nephropathy in type 1 diabetes: a manifestation of insulin resistance and multiple genetic susceptibilities? Further evidence from the Pittsburgh Epidemiology of Diabetes Complication Study. | Orchard TJ et al. | 12164879 | No data for ACE2 |
| 1 | Influence of the endothelial nitric oxide synthase polymorphism on the progression of autosomal dominant polycystic kidney disease and IgA nephropathy. | Merta M et al. | 12212826 | No data for ACE2 |
| 1 | Serum paraoxonase (PON1) concentration in patients undergoing hemodialysis. | Suehiro T et al. | 12226554 | No data for ACE2 |
| 1 | Genetic determinants of delayed graft function after kidney transplantation. | St Peter SD et al. | 12364860 | No data for ACE2 |
| 1 | Endothelial nitric oxide synthase gene polymorphism in dialysis patients. | de Prado A et al. | 12402580 | No data for ACE2 |
| 1 | Endothelial nitric oxide synthase gene and the development of diabetic nephropathy. | Shimizu T et al. | 12413777 | No data for ACE2 |

| | | | | |
|---|---|---|---|---|
| 1 | G-protein beta-3-subunit and eNOS gene polymorphism in transplant recipients with long-term renal graft function. | Viklicky O et al. | 12424427 | No data for ACE2 |
| 1 | Microfluidic chip-based method for genotyping microsatellites, VNTRs and insertion/deletion polymorphisms. | Sohni YR et al. | 12554058 | No data for ACE2 |
| 1 | The role of ACE gene polymorphism in rapidity of progression of focal segmental glomerulosclerosis. | Dixit M et al. | 12571380 | No data for ACE2 |
| 1 | The response of antioxidant genes to hyperglycemia is abnormal in patients with type 1 diabetes and diabetic nephropathy. | Hodgkinson AD et al. | 12606529 | No data for ACE2 |
| 1 | Epidermal growth factor receptor polymorphism and autosomal dominant polycystic kidney disease. | Magistroni R et al. | 12653106 | No data for ACE2 |
| 1 | Cardiac hypertrophy and remodeling in relation to ACE and angiotensinogen genes genotypes in Chinese dialysis patients. | Wang AY et al. | 12675870 | No data for ACE2 |
| 1 | Nitric oxide synthase gene polymorphisms and diabetic nephropathy. | Rippin JD et al. | 12687343 | No data for ACE2 |
| 1 | Influence of the alpha-adducin and ACE gene polymorphism on the progression of autosomal-dominant polycystic kidney disease. | Merta M et al. | 12697976 | No data for ACE2 |
| 1 | Association of ecNOS gene polymorphisms with end stage renal diseases. | Nagase S et al. | 12701818 | No data for ACE2 |
| 1 | PAI-1 4G/5G and ACE I/D gene polymorphisms and the occurrence of myocardial infarction in patients on intermittent dialysis. | Aucella F et al. | 12748347 | No data for ACE2 |
| 1 | Paraoxonase activity and paraoxonase 1 gene polymorphism in patients with uremia. | Biasioli S et al. | 12790379 | No data for ACE2 |
| 1 | Evaluation of genetic variation and association in the matrix metalloproteinase 9 (MMP9) gene in ESRD patients. | Hirakawa S et al. | 12830465 | No data for ACE2 |
| 1 | Angiotensin converting enzyme gene polymorphism and development of post-transplant erythrocytosis. | Yildiz A et al. | 12832741 | No data for ACE2 |
| 1 | Identification of a common risk haplotype for diabetic nephropathy at the protein kinase C-beta1 (PRKCB1) gene locus. | Araki S et al. | 12874455 | No data for ACE2 |
| 1 | Genotypic and phenotypic properties of coagulase-negative staphylococci causing dialysis catheter-related sepsis. | Spare MK et al. | 12919757 | No data for ACE2 |

| | | | | |
|---|---|---|---|---|
| 1 | Elevation of IgG antibodies against tissue transglutaminase as a diagnostic tool for coeliac disease in selective IgA deficiency. | Korponay-Szabo IR et al. | 14570724 | No data for ACE2 |
| 1 | Association of the p22phox component of NAD(P)H oxidase with susceptibility to diabetic nephropathy in patients with type 1 diabetes. | Hodgkinson AD et al. | 14578247 | No data for ACE2 |
| 1 | Effects of the genetic polymorphisms of the renin-angiotensin system on focal segmental glomerulosclerosis. | Luther Y et al. | 14610337 | No data for ACE2 |
| 1 | Interaction between gene polymorphisms of nitric oxide synthase and renin-angiotensin system in the progression of membranous glomerulonephritis. | Stratta P et al. | 14767013 | No data for ACE2 |
| 1 | Angiotensin-converting enzyme polymorphism gene and evolution of nephropathy to end-stage renal disease. | Ortiz MA et al. | 15012717 | No data for ACE2 |
| 1 | Angiotensin-converting enzyme genotype is a predictive factor in the peak panel-reactive antibody response. | Akcay A et al. | 15013293 | No data for ACE2 |
| 1 | Quantitative polymorphism of complement receptor type 1 (CR1) in patients undergoing haemodialysis. | Tamano M et al. | 15069174 | No data for ACE2 |
| 1 | Renin-angiotensin system gene polymorphisms: its impact on IgAN and its progression to end-stage renal failure among Chinese in Singapore. | Lau YK et al. | 15153745 | No data for ACE2 |
| 1 | Role of alpha-adducin DNA polymorphisms in the genetic predisposition to diabetic nephropathy. | Conway BR et al. | 15187197 | No data for ACE2 |
| 1 | Estrogen receptor is significantly associated with the epithelioid variants of renal angiomyolipoma: a clinicopathological and immunohistochemical study of 67 cases. | Cho NH et al. | 15189505 | No data for ACE2 |
| 1 | Paraoxonase 192 polymorphism and its relationship to serum lipids in Turkish renal transplant recipients. | Agachan B et al. | 15251338 | No data for ACE2 |
| 1 | Angiotensin converting enzyme genotype and chronic allograft nephropathy in protocol biopsies. | Hueso M et al. | 15284309 | No data for ACE2 |
| 1 | The influence of G-protein beta3-subunit gene and endothelial nitric oxide synthase gene in exon 7 polymorphisms on progression of autosomal dominant polycystic kidney disease. | Reiterova J et al. | 15287194 | No data for ACE2 |

| | | | | |
|---|---|---|---|---|
| 1 | Endothelial nitric oxide synthase gene intron 4 polymorphism in patients with end-stage renal disease. | Buraczynska M et al. | 15299097 | No data for ACE2 |
| 1 | Low prevalence of nonconservative mutations of serum and glucocorticoid-regulated kinase (SGK1) gene in hypertensive and renal patients. | Trochen N et al. | 15304560 | No data for ACE2 |
| 1 | Relations between eNOS Glu298Asp polymorphism and progression of diabetic nephropathy. | Shin Shin Y et al. | 15331206 | No data for ACE2 |
| 1 | Angiotensin-converting enzyme (insertion/deletion) and endothelial nitric oxide synthase polymorphisms in patients with systemic lupus erythematosus. | Douglas G et al. | 15338496 | No data for ACE2 |
| 1 | Angiotensin-I converting enzyme gene polymorphism in Turkish type 2 diabetic patients. | Arzu Ergen H et al. | 15365253 | No data for ACE2 |
| 1 | Identification of NQO1 and GSTs genotype frequencies in Bulgarian patients with Balkan endemic nephropathy. | Toncheva DI et al. | 15365958 | No data for ACE2 |
| 1 | Balkan endemic nephropathy and genetic variants of glutathione S-transferases. | Andonova IE et al. | 15365959 | No data for ACE2 |
| 1 | Association of the genetic polymorphisms of the renin-angiotensin system and endothelial nitric oxide synthase with chronic renal transplant dysfunction. | Akcay A et al. | 15385810 | No data for ACE2 |
| 1 | Genetic polymorphisms of cytochrome P450 among patients with Balkan endemic nephropathy (BEN). | Atanasova SY et al. | 15708542 | No data for ACE2 |
| 1 | Evidence for association of endothelial cell nitric oxide synthase gene polymorphism with earlier progression to end-stage renal disease in a cohort of Hellens from Greece and Cyprus. | Lamnissou K et al. | 15727257 | No data for ACE2 |
| 1 | Mutation analysis of autosomal dominant polycystic kidney disease genes in Han Chinese. | Zhang S et al. | 15775720 | No data for ACE2 |
| 1 | Genetic variation at the ACE gene is associated with persistent microalbuminuria and severe nephropathy in type 1 diabetes: the DCCT/EDIC Genetics Study. | Boright AP et al. | 15793268 | No data for ACE2 |

| | | | | |
|---|---|---|---|---|
| 1 | Nitric oxide- and EDHF-mediated arteriolar tone in uremia is unaffected by selective inhibition of vascular cytochrome P450 2C9. | Passauer J et al. | 15840038 | No data for ACE2 |
| 1 | Association between vitamin D receptor FokI. Polymorphism and serum parathyroid hormone level in patients with chronic renal failure. | Vigo Gago E et al. | 15887856 | No data for ACE2 |
| 1 | Polymorphisms of the renin-angiotensin system genes in Brazilian patients with lupus nephropathy. | Sprovieri SR et al. | 15934435 | No data for ACE2 |
| 1 | Effect of hepatic lipase -514C->T polymorphism and its interactions with apolipoprotein C3 -482C->T and apolipoprotein E exon 4 polymorphisms on the risk of nephropathy in chinese type 2 diabetic patients. | Baum L et al. | 15983323 | No data for ACE2 |
| 1 | The impact of thiopurine s-methyltransferase polymorphism on azathioprine-induced myelotoxicity in renal transplant recipients. | Kurzawski M et al. | 16044099 | No data for ACE2 |
| 1 | Carnosine as a protective factor in diabetic nephropathy: association with a leucine repeat of the carnosinase gene CNDP1. | Janssen B et al. | 16046297 | No data for ACE2 |
| 1 | Impact of polymorphisms in the genes encoding xylosyltransferase I and a homologue in type 1 diabetic patients with and without nephropathy. | Schon S et al. | 16164625 | No data for ACE2 |
| 1 | Frequency of angiotensin-converting enzyme gene polymorphism in Turkish type 2 diabetic patients. | Degirmenci I et al. | 16178979 | No data for ACE2 |
| 1 | Haplotype analysis of NAD(P)H oxidase p22 phox polymorphisms in end-stage renal disease. | Doi K et al. | 16215641 | No data for ACE2 |
| 1 | The effect of angiotensin receptor blockade ARB on the regression of left ventricular hypertrophy in hemodialysis patients: comparison between patients with D allele and non-D allele ACE gene polymorphism. | Nakayama M et al. | 16312263 | No data for ACE2 |
| 1 | Manganese superoxide dismutase gene polymorphism (V16A) is associated with stages of albuminuria in Korean type 2 diabetic patients. | Lee SJ et al. | 16324912 | No data for ACE2 |
| 1 | Hidden population substructures in an apparently homogeneous population bias association studies. | Berger M et al. | 16333311 | No data for ACE2 |
| 1 | Relationship of p22phox C242T polymorphism with nephropathy in type 2 diabetic patients. | Santos KG et al. | 16358232 | No data for ACE2 |

| | | | | |
|---|---|---|---|---|
| 1 | Genetic polymorphisms of the renin-angiotensin system in end-stage renal disease. | Buraczynska M et al. | 16384824 | No data for ACE2 |
| 1 | Angiotensin-converting enzyme (ACE) inhibition in type 2, diabetic patients-- interaction with ACE insertion/deletion polymorphism. | So WY et al. | 16395257 | No data for ACE2 |
| 1 | The influence of the endothelin-converting enzyme-1 gene polymorphism on the progression of autosomal dominant polycystic kidney disease. | Reiterova J et al. | 16526315 | No data for ACE2 |
| 1 | Aldosterone synthase (CYP11B2) -344T/C polymorphism is not associated with the initiation and progression of diabetic nephropathy in Caucasian Type 1 diabetic patients. | Lajer M et al. | 16759311 | No data for ACE2 |
| 1 | Novel sequence variants in the human xylosyltransferase I gene and their role in diabetic nephropathy. | Bahr C et al. | 16759312 | No data for ACE2 |
| 1 | Relationship between common functional polymorphisms of the p22phox gene (-930A > G and +242C > T) and nephropathy as a result of Type 2 diabetes in a Chinese population. | Lim SC et al. | 16922713 | No data for ACE2 |
| 1 | A disease haplotype for advanced nephropathy in type 2 diabetes at the ACE locus. | Ng DP et al. | 16936219 | No data for ACE2 |
| 1 | Efficient screening method of the thiopurine methyltransferase polymorphisms for patients considering taking thiopurine drugs in a Chinese Han population in Henan Province (central China). | Zhang LR et al. | 16952345 | No data for ACE2 |
| 1 | Kinin-dependent hypersensitivity reactions in hemodialysis: metabolic and genetic factors. | Molinaro G et al. | 17003818 | No data for ACE2 |
| 1 | High prevalence of ACE DD genotype among north Indian end stage renal disease patients. | Tripathi G et al. | 17042963 | No data for ACE2 |
| 1 | Sirolimus population pharmacokinetic/pharmacogenetic analysis and bayesian modelling in kidney transplant recipients. | Djebli N et al. | 17048977 | No data for ACE2 |
| 1 | Glu298Asp and NOS4ab polymorphisms in diabetic nephropathy. | Mollsten A et al. | 17101543 | No data for ACE2 |

| | | | | |
|---|---|---|---|---|
| 1 | Combinational effect of genes for the renin-angiotensin system in conferring susceptibility to diabetic nephropathy. | Osawa N et al. | 17143591 | No data for ACE2 |
| 1 | The emergence of cytomegalovirus resistance to ganciclovir therapy in kidney transplant recipients. | Nogueira E et al. | 17161359 | No data for ACE2 |
| 1 | Paraoxonase gene polymorphism and serum activity in progressive IgA nephropathy. | Kovacs TJ et al. | 17173245 | No data for ACE2 |
| 1 | A functional polymorphism in the manganese superoxide dismutase gene and diabetic nephropathy. | Mollsten A et al. | 17192491 | No data for ACE2 |
| 1 | A leucine repeat in the carnosinase gene CNDP1 is associated with diabetic end-stage renal disease in European Americans. | Freedman BI et al. | 17205963 | No data for ACE2 |
| 1 | Variants of C1GALT1 gene are associated with the genetic susceptibility to IgA nephropathy. | Li GS et al. | 17228361 | No data for ACE2 |
| 1 | Resequencing of genes for transforming growth factor beta1 (TGFB1) type 1 and 2 receptors (TGFBR1, TGFBR2), and association analysis of variants with diabetic nephropathy. | McKnight AJ et al. | 17319955 | No data for ACE2 |
| 1 | Association between angiotensin-converting enzyme gene polymorphisms and diabetic nephropathy: case-control, haplotype, and family-based study in three European populations. | Hadjadj S et al. | 17376814 | No data for ACE2 |
| 1 | Association of endothelial nitric oxide synthase gene intron 4 polymorphism with end-stage renal disease. | Bellini MH et al. | 17498125 | No data for ACE2 |
| 1 | [Angiotensin-1 converting enzyme insertion/deletion gene polymorphism in a Mexican population with diabetic nephropathy]. | Ortega-Pierres LE et al. | 17570179 | No data for ACE2 |
| 1 | Disease progression, response to ACEI/ATRA therapy and influence of ACE gene in IgA nephritis. | Woo KT et al. | 17601378 | No data for ACE2 |
| 1 | Relationship of angiotensin-converting enzyme gene polymorphism with nephropathy associated with Type 2 diabetes mellitus in Asian Indians. | Movva S et al. | 17616353 | No data for ACE2 |
| 1 | Impact of ENPP1 genotype on arterial calcification in patients with end-stage renal failure. | Eller P et al. | 17848394 | No data for ACE2 |

| | | | | |
|---|---|---|---|---|
| 1 | Multiple superoxide dismutase 1/splicing factor serine alanine 15 variants are associated with the development and progression of diabetic nephropathy: the Diabetes Control and Complications Trial/Epidemiology of Diabetes Interventions and Complications Genetics study. | Al-Kateb H et al. | 17914031 | No data for ACE2 |
| 1 | Association between vitamin D receptor gene polymorphisms and susceptibility to chronic kidney disease and periodontitis. | de Souza CM et al. | 17914260 | No data for ACE2 |
| 1 | Gene polymorphisms of the renin-angiotensin-aldosterone system and angiotensin II type 1-receptor activating antibodies in renal rejection. | Zhang G et al. | 17984617 | No data for ACE2 |
| 1 | Correlates of ACE activity in macroalbuminuric type 2 diabetic patients treated with chronic ACE inhibition. | Nikzamir A et al. | 17986476 | No data for ACE2 |
| 1 | Genotyping with a dried blood spot method: a useful technique for application in pharmacogenetics. | Wijnen PA et al. | 18028890 | No data for ACE2 |
| 1 | Relationship between GSTs gene polymorphism and susceptibility to end stage renal disease among North Indians. | Agrawal S et al. | 18067039 | No data for ACE2 |
| 1 | Polymorphism of the endothelial nitric oxide synthase gene is associated with diabetic retinopathy in a cohort of West Africans. | Chen Y et al. | 18079690 | No data for ACE2 |
| 1 | Association of the distal region of the ectonucleotide pyrophosphatase/phosphodiesterase 1 gene with type 2 diabetes in an African-American population enriched for nephropathy. | Keene KL et al. | 18184924 | No data for ACE2 |
| 1 | Genetic risk factors for renal failure among north Indian ESRD patients. | Tripathi G et al. | 18242170 | No data for ACE2 |
| 1 | Donor DNA is detected in recipient blood for years after kidney transplantation using sensitive forensic medicine methods. | Rutkowska J et al. | 18290564 | No data for ACE2 |
| 1 | Anti-glutathione S-transferase T1 antibody-mediated rejection in C4d-positive renal allograft recipients. | Aguilera I et al. | 18308775 | No data for ACE2 |
| 1 | Endothelial nitric oxide synthase polymorphisms are associated with hypertension and cardiovascular disease in renal transplantation. | Bhandary UV et al. | 18331440 | No data for ACE2 |
| 1 | Endothelial nitric oxide synthase gene haplotypes and diabetic nephropathy among Asian Indians. | Ahluwalia TS et al. | 18401556 | No data for ACE2 |

| | | | | |
|---|---|---|---|---|
| 1 | Lack of association between the angiotensin-converting enzyme gene (I/D) polymorphism and diabetic nephropathy in Tunisian type 2 diabetic patients. | Arfa I et al. | 18404607 | No data for ACE2 |
| 1 | Association of the angiotensinogen M235T and angiotensin-converting enzyme insertion/deletion gene polymorphisms in Turkish type 2 diabetic patients with and without nephropathy. | Eroglu Z et al. | 18413162 | No data for ACE2 |
| 1 | Oxidative stress pathway genes and chronic renal insufficiency in Asian Indians with Type 2 diabetes. | Tiwari AK et al. | 18413200 | No data for ACE2 |
| 1 | Association of endothelial nitric oxide synthase Glu298Asp, 4b/a, and -786T>C gene variants with diabetic nephropathy. | Ezzidi I et al. | 18413207 | No data for ACE2 |
| 1 | The number of activating KIR genes inversely correlates with the rate of CMV infection/reactivation in kidney transplant recipients. | Stern M et al. | 18444913 | No data for ACE2 |
| 1 | Prognostic value of the insertion/deletion polymorphism of the ACE gene in type 2 diabetic subjects: results from the Non-insulin-dependent Diabetes, Hypertension, Microalbuminuria or Proteinuria, Cardiovascular Events, and Ramipril (DIABHYCAR), Diabete de type 2, Nephropathie et Genetique (DIAB2NEP... | Hadjadj S et al. | 18523145 | No data for ACE2 |
| 1 | Angiotensin-converting enzyme inhibitor versus angiotensin 2 receptor antagonist therapy and the influence of angiotensin-converting enzyme gene polymorphism in IgA nephritis. | Woo KT et al. | 18536822 | No data for ACE2 |
| 1 | Calpain 10 SNP-44 gene polymorphism affects susceptibility to type 2 diabetes mellitus and diabetic-related conditions. | Demirci H et al. | 18554168 | No data for ACE2 |
| 1 | Association of angiotensin-converting enzyme and endothelial Nitric Oxide synthase gene polymorphisms with vascular disease in ESRD patients in a Chinese population. | Tang FY et al. | 18629615 | No data for ACE2 |
| 1 | Exclusion of polymorphisms in carnosinase genes (CNDP1 and CNDP2) as a cause of diabetic nephropathy in type 1 diabetes: results of large case-control and follow-up studies. | Wanic K et al. | 18753673 | No data for ACE2 |

| | | | | |
|---|---|---|---|---|
| 1 | Polymorphism of the aldosterone synthase gene is not associated with progression of diabetic nephropathy, but associated with hypertension in type 2 diabetic patients. | Ko GJ et al. | 18771471 | No data for ACE2 |
| 1 | Association of endothelial nitric oxide synthase Glu298Asp polymorphism with end-stage renal disease. | Thaha M et al. | 18793530 | No data for ACE2 |
| 1 | MYH9 is associated with nondiabetic end-stage renal disease in African Americans. | Kao WH et al. | 18794854 | No data for ACE2 |
| 1 | MYH9 is a major-effect risk gene for focal segmental glomerulosclerosis. | Kopp JB et al. | 18794856 | No data for ACE2 |
| 1 | Characterization of the transcriptional regulation of the human MT1-MMP gene and association of risk reduction for focal-segmental glomerulosclerosis with two functional promoter SNPs. | Munkert A et al. | 18927121 | No data for ACE2 |
| 1 | The manganese superoxide dismutase Val16Ala polymorphism is associated with decreased risk of diabetic nephropathy in Chinese patients with type 2 diabetes. | Liu L et al. | 18989629 | No data for ACE2 |
| 1 | Survival in type 2 diabetic patients in dialysis and the number of risk alleles in polymorphisms of the renin-angiotensin system genes. | Padro-Miquel A et al. | 19014923 | No data for ACE2 |
| 1 | ACE genotype, body weight changes and target organ damage in renal transplant recipients. | Stratta P et al. | 19034872 | No data for ACE2 |
| 1 | Relationships between thiopurine S-methyltransferase polymorphism and azathioprine-related adverse drug reactions in Chinese renal transplant recipients. | Xin HW et al. | 19048245 | No data for ACE2 |
| 1 | Influence of CYP3A5 genetic polymorphism on tacrolimus daily dose requirements and acute rejection in renal graft recipients. | Quteineh L et al. | 19067682 | No data for ACE2 |
| 1 | Tripterygium wilfordii hook f increase the blood concentration of tacrolimus. | Wen J et al. | 19100464 | No data for ACE2 |
| 1 | ACE variants interact with the RAS pathway to confer risk and protection against type 2 diabetic nephropathy. | Ahluwalia TS et al. | 19108684 | No data for ACE2 |
| 1 | A novel method for monitoring glucocorticoid-induced changes of the glucocorticoid receptor in kidney transplant recipients. | Chen Y et al. | 19162184 | No data for ACE2 |

| | | | | |
|---|---|---|---|---|
| 1 | Polymorphisms in the non-muscle myosin heavy chain 9 gene (MYH9) are strongly associated with end-stage renal disease historically attributed to hypertension in African Americans. | Freedman BI et al. | 19177153 | No data for ACE2 |
| 1 | Matrix metalloproteinase-1 and matrix metalloproteinase-3 gene promoter polymorphisms are associated with mortality in haemodialysis patients. | Cozzolino M et al. | 19221176 | No data for ACE2 |
| 1 | TPMT but not ITPA gene polymorphism influences the risk of azathioprine intolerance in renal transplant recipients. | Kurzawski M et al. | 19229528 | No data for ACE2 |
| 1 | Genetic variant of C1GalT1 contributes to the susceptibility to IgA nephropathy. | Pirulli D et al. | 19229831 | No data for ACE2 |
| 1 | The endothelial nitric oxide synthase gene and risk of diabetic nephropathy and development of cardiovascular disease in type 1 diabetes. | Mollsten A et al. | 19246226 | No data for ACE2 |
| 1 | Association of genetic variants with chronic kidney disease in Japanese individuals with type 2 diabetes mellitus. | Yoshida T et al. | 19288030 | No data for ACE2 |
| 1 | The PTPN22 C1858T (R620W) functional polymorphism in kidney transplantation. | Sfar I et al. | 19328948 | No data for ACE2 |
| 1 | Elevated MBL concentrations are not an indication of association between the MBL2 gene and type 1 diabetes or diabetic nephropathy. | Kaunisto MA et al. | 19366862 | No data for ACE2 |
| 1 | The influence of carnosinase gene polymorphisms on diabetic nephropathy risk in African-Americans. | McDonough CW et al. | 19373489 | No data for ACE2 |
| 1 | Endothelial nitric oxide synthetase, methylenetetrahydrofolate reductase polymorphisms, and cardiovascular complications in Tunisian patients with nondiabetic renal disease. | Kerkeni M et al. | 19376104 | No data for ACE2 |
| 1 | Association of genetic variants with chronic kidney disease in Japanese individuals. | Yoshida T et al. | 19406964 | No data for ACE2 |
| 1 | Microarray analysis of multiple candidate genes and associated plasma proteins for nephropathy secondary to type 2 diabetes among Chinese individuals. | Lim SC et al. | 19415232 | No data for ACE2 |

| | | | | |
|---|---|---|---|---|
| 1 | Association of gene polymorphisms with chronic kidney disease in high- or low-risk subjects defined by conventional risk factors. | Yoshida T et al. | 19424605 | No data for ACE2 |
| 1 | Influence of genetic polymorphisms in GSTM1, GSTM3, GSTT1 and GSTP1 on allograft outcome in renal transplant recipients. | Singh R et al. | 19486347 | No data for ACE2 |
| 1 | Association and interaction analyses of genetic variants in ADIPOQ, ENPP1, GHSR, PPARgamma and TCF7L2 genes for diabetic nephropathy in a Taiwanese population with type 2 diabetes. | Wu LS et al. | 19506043 | No data for ACE2 |
| 1 | Non-muscle myosin heavy chain 9 gene MYH9 associations in African Americans with clinically diagnosed type 2 diabetes mellitus-associated ESRD. | Freedman BI et al. | 19567477 | No data for ACE2 |
| 1 | Association of genetic variants with chronic kidney disease in individuals with different lipid profiles. | Yoshida T et al. | 19578796 | No data for ACE2 |
| 1 | A HindIII polymorphism of fibronectin gene is associated with nephrolithiasis. | Onaran M et al. | 19616291 | No data for ACE2 |
| 1 | Impact of genetic polymorphisms of the renin-angiotensin system and of non-genetic factors on kidney transplant function--a single-center experience. | Siekierka-Harreis M et al. | 19681973 | No data for ACE2 |
| 1 | Association between inosine triphosphate pyrophosphohydrolase deficiency and azathioprine-related adverse drug reactions in the Chinese kidney transplant recipients. | Xiong H et al. | 19682085 | No data for ACE2 |
| 1 | The prevalence of uridine diphosphate-glucuronosyltransferase 1A9 (UGT1A9) gene promoter region single-nucleotide polymorphisms T-275A and C-2152T and its influence on mycophenolic acid pharmacokinetics in stable renal transplant patients. | Sanchez-Fructuoso AI et al. | 19715905 | No data for ACE2 |
| 1 | Association of gene polymorphisms with chronic kidney disease in Japanese individuals. | Yoshida T et al. | 19724895 | No data for ACE2 |
| 1 | A rare haplotype of the vitamin D receptor gene is protective against diabetic nephropathy. | Martin RJ et al. | 19783860 | No data for ACE2 |

| | | | | |
|---|---|---|---|---|
| 1 | Identification of specific angiotensin-converting enzyme variants and haplotypes that confer risk and protection against type 2 diabetic nephropathy. | Ezzidi I et al. | 19787680 | No data for ACE2 |
| 1 | XbaI GLUT1 gene polymorphism and the risk of type 2 diabetes with nephropathy. | Stefanidis I et al. | 19822956 | No data for ACE2 |
| 1 | The V16A polymorphism in SOD2 is associated with increased risk of diabetic nephropathy and cardiovascular disease in type 1 diabetes. | Mollsten A et al. | 19834686 | No data for ACE2 |
| 1 | Time of drug administration, CYP3A5 and ABCB1 genotypes, and analytical method influence tacrolimus pharmacokinetics: a population pharmacokinetic study. | Musuamba FT et al. | 19855314 | No data for ACE2 |
| 1 | Impact of donor-dependent genetic factors on long-term renal graft function. | Krajewska M et al. | 19857655 | No data for ACE2 |
| 1 | Uromodulin levels associate with a common UMOD variant and risk for incident CKD. | Kottgen A et al. | 19959715 | No data for ACE2 |
| 1 | Endothelial nitric oxide synthase (eNOS) gene polymorphism in early term chronic allograft nephropathy. | Yilmaz E et al. | 20005399 | No data for ACE2 |
| 1 | Circulating methylarginine levels and the decline in renal function in patients with chronic kidney disease are modulated by DDAH1 polymorphisms. | Caplin B et al. | 20010544 | No data for ACE2 |
| 1 | Dense mapping of MYH9 localizes the strongest kidney disease associations to the region of introns 13 to 15. | Nelson GW et al. | 20124285 | No data for ACE2 |
| 1 | African ancestry allelic variation at the MYH9 gene contributes to increased susceptibility to non-diabetic end-stage kidney disease in Hispanic Americans. | Behar DM et al. | 20144966 | No data for ACE2 |
| 1 | Pharmacogenetics of immunosuppressant polymorphism of CYP3A5 in renal transplant recipients. | Larriba J et al. | 20172323 | No data for ACE2 |
| 1 | Patients with Epstein-Fechtner syndromes owing to MYH9 R702 mutations develop progressive proteinuric renal disease. | Sekine T et al. | 20200500 | No data for ACE2 |

| | | | | |
|---|---|---|---|---|
| 1 | Association analysis of ADPRT1, AKR1B1, RAGE, GFPT2 and PAI-1 gene polymorphisms with chronic renal insufficiency among Asian Indians with type-2 diabetes. | Prasad P et al. | 20353610 | No data for ACE2 |
| 1 | Optimization of initial tacrolimus dose using pharmacogenetic testing. | Thervet E et al. | 20393454 | No data for ACE2 |
| 1 | Identification of GDNF gene sequence variations in patients with medullary sponge kidney disease. | Torregrossa R et al. | 20448065 | No data for ACE2 |
| 1 | Association of polymorphisms in the klotho gene with severity of non-diabetic ESRD in African Americans. | Bostrom MA et al. | 20466664 | No data for ACE2 |
| 1 | DDOST, PRKCSH and LGALS3, which encode AGE-receptors 1, 2 and 3, respectively, are not associated with diabetic nephropathy in type 1 diabetes. | Hoverfelt A et al. | 20490454 | No data for ACE2 |
| 1 | The acetyl-coenzyme A carboxylase beta (ACACB) gene is associated with nephropathy in Chinese patients with type 2 diabetes. | Tang SC et al. | 20519229 | No data for ACE2 |
| 1 | Association of trypanolytic ApoL1 variants with kidney disease in African Americans. | Genovese G et al. | 20647424 | No data for ACE2 |
| 1 | A risk allele for focal segmental glomerulosclerosis in African Americans is located within a region containing APOL1 and MYH9. | Genovese G et al. | 20668430 | No data for ACE2 |
| 1 | ACE gene polymorphism and serum ACE activity in Iranians type II diabetic patients with macroalbuminuria. | Felehgari V et al. | 20830509 | No data for ACE2 |
| 1 | Endothelial nitric oxide genotypes and haplotypes are not associated with end-stage renal disease. | Marson BP et al. | 20849252 | No data for ACE2 |
| 1 | The frequency of factor V Leiden mutation, ACE gene polymorphism, serum ACE activity and response to ACE inhibitor and angiotensin II receptor antagonist drugs in Iranians type II diabetic patients with microalbuminuria. | Rahimi Z et al. | 20853144 | No data for ACE2 |
| 1 | Association of glutathione S-transferase M1 and T1 gene polymorphism with oxidative stress in diabetic and nondiabetic chronic kidney disease. | Datta SK et al. | 20954980 | No data for ACE2 |
| 1 | Toward personalized medicine in renal transplantation. | Lampreabe I et al. | 20970553 | No data for ACE2 |

| | | | | |
|---|---|---|---|---|
| 1 | Relation between development of cardiovascular disease and the C242T CYBA polymorphism of the NADPH oxidase in ESRD patients. | Tang FY et al. | 21045268 | No data for ACE2 |
| 1 | Association analysis of Notch pathway signalling genes in diabetic nephropathy. | Kavanagh D et al. | 21103979 | No data for ACE2 |
| 1 | [Clinical and genetic basis of hypertensive nephrosclerosis. NEFROSEN Study]. | Diez Ojea B et al. | 21113220 | No data for ACE2 |
| 1 | Impact of polymorphisms of the genes encoding angiotensin II-forming enzymes on the progression of IgA nephropathy. | Jung ES et al. | 21150220 | No data for ACE2 |
| 1 | Genetic polymorphisms of the renin-angiotensin-aldosterone system in Chinese patients with end-stage renal disease secondary to IgA nephropathy. | Huang HD et al. | 21163122 | No data for ACE2 |
| 1 | Novel polymorphisms associated with tacrolimus trough concentrations: results from a multicenter kidney transplant consortium. | Jacobson PA et al. | 21206424 | No data for ACE2 |
| 1 | Angiotensin-converting enzyme gene polymorphisms and T2DM in a case-control association study of the Bahraini population. | Al-Harbi EM et al. | 21207118 | No data for ACE2 |
| 1 | Polymorphisms in the nonmuscle myosin heavy chain 9 gene (MYH9) are associated with the progression of IgA nephropathy in Chinese. | Cheng W et al. | 21245129 | No data for ACE2 |
| 1 | Association of eNOS gene polymorphisms with renal disease in Caucasians with type 2 diabetes. | Santos KG et al. | 21255858 | No data for ACE2 |
| 1 | Genetic variation in the matrix metalloproteinase genes and diabetic nephropathy in type 1 diabetes. | Kure M et al. | 21277817 | No data for ACE2 |
| 1 | Tacrolimus dosing in Chinese renal transplant recipients: a population-based pharmacogenetics study. | Li L et al. | 21331500 | No data for ACE2 |
| 1 | Analysis of insertion/deletion polymorphisms of the angiotensin converting enzyme gene in Malaysian end-stage renal disease patients. | Ali A et al. | 21421653 | No data for ACE2 |
| 1 | In vivo activity of epoxide hydrolase according to sequence variation affects the progression of human IgA nephropathy. | Lee JP et al. | 21429967 | No data for ACE2 |
| 1 | Impact of aldosterone synthase gene C-344T polymorphism on IgA nephropathy. | Bantis C et al. | 21476902 | No data for ACE2 |

| | | | | |
|---|---|---|---|---|
| 1 | Expression of CYP3A5 and P-glycoprotein in renal allografts with histological signs of calcineurin inhibitor nephrotoxicity. | Metalidis C et al. | 21544031 | No data for ACE2 |
| 1 | Common variants in CNDP1 and CNDP2, and risk of nephropathy in type 2 diabetes. | Ahluwalia TS et al. | 21573905 | No data for ACE2 |
| 1 | Insertion/deletion polymorphism of the angiotensin-converting enzyme predicts left ventricular hypertrophy after renal transplantation. | Fedor R et al. | 21620105 | No data for ACE2 |
| 1 | Polymorphisms of pon1 and pon2 genes in hemodialyzed patients. | Rajkovic MG et al. | 21620813 | No data for ACE2 |
| 1 | A polymorphism of NADPH oxidase p22 phox is associated with reduced susceptibility to acute rejection in renal allograft recipients. | Bhandary UV et al. | 21624462 | No data for ACE2 |
| 1 | Exome sequencing identified MYO1E and NEIL1 as candidate genes for human autosomal recessive steroid-resistant nephrotic syndrome. | Sanna-Cherchi S et al. | 21697813 | No data for ACE2 |
| 1 | Differential effects of MYH9 and APOL1 risk variants on FRMD3 Association with Diabetic ESRD in African Americans. | Freedman BI et al. | 21698141 | No data for ACE2 |
| 1 | Influence of aldosterone synthase gene C-344T polymorphism on focal segmental glomerulosclerosis. | Bantis C et al. | 21777344 | No data for ACE2 |
| 1 | Thiopurine S-methyltransferase polymorphism in Iranian kidney transplant recipients. | Aghdaie MH et al. | 21819368 | No data for ACE2 |
| 1 | Sickle cell trait is not independently associated with susceptibility to end-stage renal disease in African Americans. | Hicks PJ et al. | 21849968 | No data for ACE2 |
| 1 | Association between CYP3A5 polymorphisms and blood pressure in kidney transplant recipients receiving calcineurin inhibitors. | Ferraresso M et al. | 21851254 | No data for ACE2 |
| 1 | Impact of nitric oxide synthase Glu298Asp polymorphism on the development of end-stage renal disease in type 2 diabetic Egyptian patients. | El-Din Bessa SS et al. | 21854353 | No data for ACE2 |
| 1 | Assessment of matrix Gla protein, Klotho gene polymorphisms, and oxidative stress in chronic kidney disease. | Karsli Ceppioglu S et al. | 21859400 | No data for ACE2 |
| 1 | Influence of CYP3A5 and ABCB1 gene polymorphisms and other factors on tacrolimus dosing in Caucasian liver and kidney transplant patients. | Provenzani A et al. | 21922127 | No data for ACE2 |

| | | | | |
|---|---|---|---|---|
| 1 | Interaction of MTHFR 1298C with ACE D allele augments the risk of diabetic nephropathy in Western Iran. | Rahimi Z et al. | 21942443 | No data for ACE2 |
| 1 | Association of genetic variants in the promoter region of genes encoding p22phox (CYBA) and glutamate cysteine ligase catalytic subunit (GCLC) and renal disease in patients with type 1 diabetes mellitus. | Vieira SM et al. | 21962117 | No data for ACE2 |
| 1 | Polymorphisms in MYH9 are associated with diabetic nephropathy in European Americans. | Cooke JN et al. | 21968013 | No data for ACE2 |
| 1 | Angiotensin-converting enzyme (ACE) serum levels and gene polymorphism in Egyptian patients with systemic lupus erythematosus. | Abbas D et al. | 21976404 | No data for ACE2 |
| 1 | Association of eGFR-Related Loci Identified by GWAS with Incident CKD and ESRD. | Boger CA et al. | 21980298 | No data for ACE2 |
| 1 | APOL1 variants increase risk for FSGS and HIVAN but not IgA nephropathy. | Papeta N et al. | 21997397 | No data for ACE2 |
| 1 | Genetic and functional effects of membrane metalloendopeptidase on diabetic nephropathy development. | Zhang D et al. | 22024547 | No data for ACE2 |
| 1 | The interactions of age, sex, body mass index, genetics, and steroid weight-based doses on tacrolimus dosing requirement after adult kidney transplantation. | Stratta P et al. | 22101623 | No data for ACE2 |
| 1 | Genetic polymorphisms and the risk of progressive renal failure in elderly Hungarian patients. | Zsom M et al. | 22111818 | No data for ACE2 |
| 1 | Glutathione S-transferases T1 null genotype is associated with susceptibility to aristolochic acid nephropathy. | Chen B et al. | 22116675 | No data for ACE2 |
| 1 | Nosocomial Pneumocystis jirovecii pneumonia: lessons from a cluster in kidney transplant recipients. | Phipps LM et al. | 22129760 | No data for ACE2 |
| 1 | Gene polymorphisms of angiotensin-converting enzyme and angiotensin II type 1 receptor among chronic kidney disease patients in a Chinese population. | Su SL et al. | 22147663 | No data for ACE2 |
| 1 | Clinical and genetic factors affecting tacrolimus trough levels and drug-related outcomes in Korean kidney transplant recipients. | Kim IW et al. | 22183771 | No data for ACE2 |

| | | | | |
|---|---|---|---|---|
| 1 | SIRTUIN 1 gene polymorphisms are associated with cholesterol metabolism and coronary artery calcification in Japanese hemodialysis patients. | Shimoyama Y et al. | 22200427 | No data for ACE2 |
| 1 | Association of UDP-glucuronosyltransferase 1A9 (UGT1A9) gene polymorphism with kidney allograft function. | Pazik J et al. | 22210424 | No data for ACE2 |
| 1 | The association of the UGT1A8, SLCO1B3 and ABCC2/ABCG2 genetic polymorphisms with the pharmacokinetics of mycophenolic acid and its phenolic glucuronide metabolite in Chinese individuals. | Geng F et al. | 22227166 | No data for ACE2 |
| 1 | BsmI polymorphisms in vitamin D receptor gene are associated with diabetic nephropathy in type 2 diabetes in the Han Chinese population. | Zhang H et al. | 22245613 | No data for ACE2 |
| 1 | Matrix metalloproteinase (MMP)-2 genetic variants modify the circulating MMP-2 levels in end-stage kidney disease. | Marson BP et al. | 22302011 | No data for ACE2 |
| 1 | Impact of cytochrome P450 3A and ATP-binding cassette subfamily B member 1 polymorphisms on tacrolimus dose-adjusted trough concentrations among Korean renal transplant recipients. | Cho JH et al. | 22310591 | No data for ACE2 |
| 1 | Endothelial nitric oxide synthase gene polymorphisms and the risk of diabetic nephropathy in type 2 diabetes mellitus. | Shoukry A et al. | 22313046 | No data for ACE2 |
| 1 | MTHFR C677T, A1298C and ACE I/D polymorphisms as risk factors for diabetic nephropathy among type 2 diabetic patients. | El-Baz R et al. | 22554825 | No data for ACE2 |
| 1 | Paraoxonase 1 polymorphisms in patients with primary glomerulonephritis: a single-center study in Turkey. | Eren Z et al. | 22555481 | No data for ACE2 |
| 1 | Association of base excision repair gene polymorphisms with ESRD risk in a Chinese population. | Cai Z et al. | 22720119 | No data for ACE2 |
| 1 | Base excision repair gene polymorphisms are associated with inflammation in patients undergoing chronic hemodialysis. | Cai Z et al. | 22780951 | No data for ACE2 |
| 1 | Relationship between antioxidant enzyme genotype and activity and kidney function: a case-control study. | Crawford A et al. | 22790458 | No data for ACE2 |

| | | | | |
|---|---|---|---|---|
| 1 | Genetic polymorphisms located in genes related to immune and inflammatory processes are associated with end-stage renal disease: a preliminary study. | Jimenez-Sousa MA et al. | 22817530 | No data for ACE2 |
| 1 | Apolipoprotein L1 gene variants associate with hypertension-attributed nephropathy and the rate of kidney function decline in African Americans. | Lipkowitz MS et al. | 22832513 | No data for ACE2 |
| 1 | Donor age and ABCB1 1199G>A genetic polymorphism are independent factors affecting long-term renal function after kidney transplantation. | De Meyer M et al. | 22835948 | No data for ACE2 |
| 1 | The effect of glutathion S-transferase polymoprhisms and anti-GSTT1 antibodies on allograft functions in recipients of renal transplant. | Akgul SU et al. | 22841242 | No data for ACE2 |
| 1 | Primary hyperoxaluria type 1, a too often missed diagnosis and potentially treatable cause of end-stage renal disease in adults: results of the Dutch cohort. | van der Hoeven SM et al. | 22844106 | No data for ACE2 |
| 1 | Genetic and functional analyses of MRAS and HNF1A genes in diabetes and diabetic nephropathy. | Horova E et al. | 22849862 | No data for ACE2 |
| 1 | Genetic variation in thrombin-activatable fibrinolysis inhibitor is associated with the risk of diabetic nephropathy. | Xu CW et al. | 22932273 | No data for ACE2 |
| 1 | Genetic variation in APOL1 and MYH9 genes is associated with chronic kidney disease among Nigerians. | Tayo BO et al. | 22956460 | No data for ACE2 |
| 1 | Glutathione S-transferase A1, M1, P1 and T1 null or low-activity genotypes are associated with enhanced oxidative damage among haemodialysis patients. | Suvakov S et al. | 23034843 | No data for ACE2 |
| 1 | Association of vitamin D receptor FokI and ApaI polymorphisms with human cytomegalovirus disease in the first three months following kidney transplantation. | Zhao YG et al. | 23044313 | No data for ACE2 |
| 1 | Vitamin D receptor gene BsmI, FokI, ApaI and TaqI polymorphisms and the risk of systemic lupus erythematosus. | Mostowska A et al. | 23065277 | No data for ACE2 |
| 1 | ACACÎ² gene (rs2268388) and AGTR1 gene (rs5186) polymorphism and the risk of nephropathy in Asian Indian patients with type 2 diabetes. | Shah VN et al. | 23081748 | No data for ACE2 |

| | | | | |
|---|---|---|---|---|
| 1 | Chronic renal impairment and DDAH2-1151 A/C polymorphism determine ADMA levels in type 2 diabetic subjects. | Marra M et al. | 23129820 | No data for ACE2 |
| 1 | Relationship of BsmI polymorphism of vitamin D receptor gene with left ventricular hypertrophy and atherosclerosis in hemodialysis patients. | El-Shehaby AM et al. | 23198772 | No data for ACE2 |
| 1 | CYP3A4 genetic polymorphisms predict cyclosporine-related clinical events in Chinese renal transplant recipients. | Wang YY et al. | 23217392 | No data for ACE2 |
| 1 | Clinical impact of an angiotensin I-converting enzyme insertion/deletion and kinin B2 receptor +9/-9 polymorphisms in the prognosis of renal transplantation. | Amorim CE et al. | 23362199 | No data for ACE2 |
| 1 | The A736V TMPRSS6 polymorphism influences hepcidin and iron metabolism in chronic hemodialysis patients: TMPRSS6 and hepcidin in hemodialysis. | Pelusi S et al. | 23433094 | No data for ACE2 |
| 1 | Identification of chromosome 3q28 and ALPK1 as susceptibility loci for chronic kidney disease in Japanese individuals by a genome-wide association study. | Yamada Y et al. | 23539754 | No data for ACE2 |
| 1 | Replication study for the association of 3 SNP loci identified in a genome-wide association study for diabetic nephropathy in European type 1 diabetes with diabetic nephropathy in Japanese patients with type 2 diabetes. | Maeda S et al. | 23543049 | No data for ACE2 |
| 1 | eNOS 4a/b polymorphism and its interaction with eNOS G894T variants in type 2 diabetes mellitus: modifying the risk of diabetic nephropathy. | Rahimi Z et al. | 23594559 | No data for ACE2 |
| 1 | Association of CYP1A1 gene polymorphism with chronic kidney disease: a case control study. | Siddarth M et al. | 23619522 | No data for ACE2 |
| 1 | Influence of GSTO2 (N142D) genetic polymorphism on acute renal rejection. | Nekooie-Marnany N et al. | 23649768 | No data for ACE2 |
| 1 | Association of aldosterone synthase (CYP11B2) gene -344T/C polymorphism with the risk of primary chronic glomerulonephritis in the Polish population. | Pawlik M et al. | 23681285 | No data for ACE2 |
| 1 | Association of POL1, MALT1, MC4R, PHLPP and DSEL single nucleotide polymorphisms in chromosome 18q region with type 2 diabetes in Tunisians. | Turki A et al. | 23727064 | No data for ACE2 |

| | | | | |
|---|---|---|---|---|
| 1 | ADAMTS13 predicts renal and cardiovascular events in type 2 diabetic patients and response to therapy. | Rurali E et al. | 23733198 | No data for ACE2 |
| 1 | Characterization of three vasopressin receptor 2 variants: an apparent polymorphism (V266A) and two loss-of-function mutations (R181C and M311V). | Armstrong SP et al. | 23762448 | No data for ACE2 |
| 1 | Distribution of dimethylarginine-dimethylaminohydrolase-II (DDAH2) gene polymorphism in hemodialysis patients. | Thaha M et al. | 23770786 | No data for ACE2 |
| 1 | Genetic predisposition for development of nephropathy in type 2 diabetes mellitus. | Kumar R et al. | 23846111 | No data for ACE2 |
| 1 | CYP2C9 and ABCG2 polymorphisms as risk factors for developing adverse drug reactions in renal transplant patients taking fluvastatin: a case-control study. | Mirosevic Skvrce N et al. | 24024895 | No data for ACE2 |
| 1 | Impact of CYP3A5 genotype on tacrolimus versus midazolam clearance in renal transplant recipients: new insights in CYP3A5-mediated drug metabolism. | de Jonge H et al. | 24024898 | No data for ACE2 |
| 1 | Suppressed soluble Fms-like tyrosine kinase-1 production aggravates atherosclerosis in chronic kidney disease. | Matsui M et al. | 24048373 | No data for ACE2 |
| 1 | Impact of CYP3A4*22 allele on tacrolimus pharmacokinetics in early period after renal transplantation: toward updated genotype-based dosage guidelines. | Elens L et al. | 24052064 | No data for ACE2 |
| 1 | Association of TNF-Î± -308 G > A and ACE I/D gene polymorphisms in hemodialysis patients with arteriovenous fistula thrombosis. | Sener EF et al. | 24126814 | No data for ACE2 |
| 1 | Association of E-selectin gene polymorphism and serum PAPP-A with carotid atherosclerosis in end-stage renal disease. | Issac MS et al. | 24151105 | No data for ACE2 |
| 1 | MYH9-related disease: a novel prognostic model to predict the clinical evolution of the disease based on genotype-phenotype correlations. | Pecci A et al. | 24186861 | No data for ACE2 |
| 1 | Increased level of organochlorine pesticides in chronic kidney disease patients of unknown etiology: role of GSTM1/GSTT1 polymorphism. | Siddarth M et al. | 24216264 | No data for ACE2 |

| | | | | |
|---|---|---|---|---|
| 1 | An integrative study of the genetic, social and environmental determinants of chronic kidney disease characterized by tubulointerstitial damages in the North Central Region of Sri Lanka. | Nanayakkara S et al. | 24351856 | No data for ACE2 |
| 1 | Immune response following liver transplantation compared to kidney transplantation: usefulness of monitoring peripheral blood CD4+ adenosine triphosphate activity and cytochrome P450 3A5 genotype assay. | Nobuoka Y et al. | 24454479 | No data for ACE2 |
| 1 | Relationship of CYP3A5 genotype and ABCB1 diplotype to tacrolimus disposition in Brazilian kidney transplant patients. | Cusinato DA et al. | 24528196 | No data for ACE2 |
| 1 | Evaluation of candidate nephropathy susceptibility genes in a genome-wide association study of African American diabetic kidney disease. | Palmer ND et al. | 24551085 | No data for ACE2 |
| 1 | Identification of novel mutations of PKD1 gene in Chinese patients with autosomal dominant polycystic kidney disease by targeted next-generation sequencing. | Yang T et al. | 24582653 | No data for ACE2 |
| 1 | Association of BH3 interacting domain death agonist (BID) gene polymorphisms with proteinuria of immunoglobulin A nephropathy. | Park HJ et al. | 24621205 | No data for ACE2 |
| 1 | Association studies of cytochrome P450, family 2, subfamily E, polypeptide 1 (CYP2E1) gene polymorphisms with acute rejection in kidney transplantation recipients. | Kim SK et al. | 24654912 | No data for ACE2 |
| 1 | MYH9 and APOL1 gene polymorphisms and the risk of CKD in patients with lupus nephritis from an admixture population. | Colares VS et al. | 24658608 | No data for ACE2 |
| 1 | Cys327Cys polymorphism of the PAPP-A gene (pregnancy associated plasma protein A) is related to mortality of long term hemodialysis patients. | Kalousova M et al. | 24667032 | No data for ACE2 |
| 1 | Genes involved in the regulation of vascular homeostasis determine renal survival rate in patients with chronic glomerulonephritis. | Litovkina O et al. | 24727057 | No data for ACE2 |
| 1 | Lack of serologic evidence to link IgA nephropathy with celiac disease or immune reactivity to gluten. | Moeller S et al. | 24732864 | No data for ACE2 |
| 1 | Genetic variants of ACE (Insertion/Deletion) and AGT (M268T) genes in patients with diabetes and nephropathy. | Shaikh R et al. | 24737640 | No data for ACE2 |

| | | | | |
|---|---|---|---|---|
| 1 | Single nucleotide polymorphisms at erythropoietin, superoxide dismutase 1, splicing factor, arginine/serin-rich 15 and plasmacytoma variant translocation genes association with diabetic nephropathy. | Alwohhaib M et al. | 24821155 | No data for ACE2 |
| 1 | NOS3 tagSNPs does not modify the chronic kidney disease progression in autosomal dominant polycystic kidney disease. | Ramanathan G et al. | 24824375 | No data for ACE2 |
| 1 | Renin-angiotensin-aldosterone system related gene polymorphisms and urinary total arsenic is related to chronic kidney disease. | Chen WJ et al. | 24907556 | No data for ACE2 |
| 1 | Gene-gene interactions in renin-angiotensin-aldosterone system contributes to end-stage renal disease susceptibility in a Han Chinese population. | Su SL et al. | 24977181 | No data for ACE2 |
| 1 | Polymorphisms in oxidative stress pathway genes and risk of diabetic nephropathy in South Indian type 2 diabetic patients. | Narne P et al. | 25041504 | No data for ACE2 |
| 1 | Rare mutations associating with serum creatinine and chronic kidney disease. | Sveinbjornsson G et al. | 25082825 | No data for ACE2 |
| 1 | Is increased susceptibility to Balkan endemic nephropathy in carriers of common GSTA1 (*A/*B) polymorphism linked with the catalytic role of GSTA1 in ochratoxin a biotransformation? Serbian case control study and in silico analysis. | Reljic Z et al. | 25111321 | No data for ACE2 |
| 1 | Association between genetic polymorphisms of ACE & eNOS and diabetic nephropathy. | Huo P et al. | 25227524 | No data for ACE2 |
| 1 | DNA repair genes XPD and XRCC1 polymorphisms and risk of end-stage renal disease in Egyptian population. | Radwan WM et al. | 25310768 | No data for ACE2 |
| 1 | Clinical utility of chitotriosidase enzyme activity in nephropathic cystinosis. | Elmonem MA et al. | 25407738 | No data for ACE2 |
| 1 | Synergism between asymmetric dimethylarginine (ADMA) and a genetic marker of uric acid in CKD progression. | Testa A et al. | 25435339 | No data for ACE2 |
| 1 | The functional Q84R polymorphism of TRIB3 gene is associated with diabetic nephropathy in Chinese type 2 diabetic patients. | Zhang W et al. | 25447894 | No data for ACE2 |

| | | | | |
|---|---|---|---|---|
| 1 | Circulating angiotensin-converting enzyme 2 activity in patients with chronic kidney disease without previous history of cardiovascular disease | Anguiano L et al. | 25813276 | No data for ACE2 |
| 1 | Allelic variations in the CYBA gene of NADPH oxidase and risk of kidney complications in patients with type 1 diabetes. | Patente TA et al. | 25862415 | No data for ACE2 |
| 1 | Associations between INSR and MTOR polymorphisms in type 2 diabetes mellitus and diabetic nephropathy in a Northeast Chinese Han population. | Zhu AN et al. | 25867326 | No data for ACE2 |
| 1 | Polymorphism of the CYP3A5 gene and its effect on tacrolimus blood level. | Nair SS et al. | 25894154 | No data for ACE2 |
| 1 | Copy Number Variation at the APOL1 Locus. | Ruchi R et al. | 25933006 | No data for ACE2 |
| 1 | Aldosterone Synthase CYP11B2 Gene Promoter Polymorphism in a Turkish Population With Chronic Kidney Disease. | Yilmaz M et al. | 25957425 | No data for ACE2 |
| 1 | Is Klotho F352V Polymorphism the Missing Piece of the Bone Loss Puzzle in Renal Transplant Recipients? | Ozdem S et al. | 26022923 | No data for ACE2 |
| 1 | Identification of new susceptibility loci for IgA nephropathy in Han Chinese. | Li M et al. | 26028593 | No data for ACE2 |
| 1 | Association between C1GALT1 variants and genetic susceptibility to IgA nephropathy in Uygur. | Li WL et al. | 26125729 | No data for ACE2 |
| 1 | Estimated glomerular filtration rate (eGFR), 25(OH) D3, chronic kidney disease (CKD), the MYH9 (myosin heavy chain 9) gene in old and very elderly people. | Otero Gonzalez A et al. | 26152646 | No data for ACE2 |
| 1 | Influence of genetic variability at the ACE locus in intron 16 on Diabetic Nephropathy in T1DM patients. | Parchwani DN et al. | 26214998 | No data for ACE2 |
| 1 | How to handle missed or delayed doses of tacrolimus in renal transplant recipients? A pharmacokinetic investigation. | Saint-Marcoux F et al. | 26316426 | No data for ACE2 |
| 1 | Re-Sequencing of the APOL1-APOL4 and MYH9 Gene Regions in African Americans Does Not Identify Additional Risks for CKD Progression. | Hawkins GA et al. | 26343748 | No data for ACE2 |

| | | | | |
|---|---|---|---|---|
| 1 | Prevalence of angiotensin converting enzyme (ACE) gene insertion/deletion polymorphism in South Indian population with hypertension and chronic kidney disease. | Shanmuganathan R et al. | 26440392 | No data for ACE2 |
| 1 | RAGE and CYBA polymorphisms are associated with microalbuminuria and end-stage renal disease onset in a cohort of type 1 diabetes mellitus patients over a 20-year follow-up. | Franko B et al. | 26607824 | No data for ACE2 |
| 1 | Polymorphisms in NADPH oxidase CYBA gene modify the risk of ESRD in patients with chronic glomerulonephritis. | Zhou H et al. | 26627442 | No data for ACE2 |
| 1 | Impact of CYP3A5 polymorphism on trough concentrations and outcomes of tacrolimus minimization during the early period after kidney transplantation. | Yaowakulpatana K et al. | 26635230 | No data for ACE2 |
| 1 | Relationship between rs1047763 polymorphism of the C1GALT1 gene and susceptibility to immunoglobulin A nephropathy in Xinjiang Uyghur people. | Xue JN et al. | 26782518 | No data for ACE2 |
| 1 | Manganese superoxide dismutase, glutathione peroxidase and catalase gene polymorphisms and clinical outcomes in acute kidney injury. | Kidir V et al. | 26787049 | No data for ACE2 |
| 1 | Genetic analysis and functional characterization of novel mutations in a series of patients with atypical hemolytic uremic syndrome. | Szarvas N et al. | 26826462 | No data for ACE2 |
| 1 | A Lack of Significant Effect of POR*28 Allelic Variant on Tacrolimus Exposure in Kidney Transplant Recipients. | Jannot AS et al. | 26829596 | No data for ACE2 |
| 1 | Association of RAC1 Gene Polymorphisms with Primary End-Stage Renal Disease in Chinese Renal Recipients. | Liu Y et al. | 26841219 | No data for ACE2 |
| 1 | Nodular glomerulosclerosis and renin angiotensin system in Chinese patients with type 2 diabetes | Wang M et al. | 26973293 | No data for ACE2 |
| 1 | Genome-Wide Association Study of Acute Renal Graft Rejection. | Ghisdal L et al. | 27272414 | No data for ACE2 |
| 1 | The angiotensin-I converting enzyme gene I/D variation contributes to end-stage renal disease risk in Chinese patients with type 2 diabetes receiving hemodialysis. | Lu M et al. | 27633502 | No data for ACE2 |

| | | | | |
|---|---|---|---|---|
| 1 | Pin1 and secondary hyperparathyroidism of chronic kidney disease: gene polymorphisms and protein levels. | Zhao Y et al. | 27876426 | No data for ACE2 |
| 1 | Lysine 63 ubiquitination is involved in the progression of tubular damage in diabetic nephropathy. | Pontrelli P et al. | 27881486 | No data for ACE2 |
| 1 | Mutational Analysis of Agxt in Tunisian Population with Primary Hyperoxaluria Type 1. | M'dimegh S et al. | 27935012 | No data for ACE2 |
| 1 | Mutation screening of ACKR3 and COPS8 in kidney cancer cases from the CONFIRM study. | Mahmoodi M et al. | 28063109 | No data for ACE2 |
| 1 | Glutathione Peroxidase 1 Gene Polymorphism in Nephrolithiasis Patients From South of Iran. | Aghakhani R et al. | 28174350 | No data for ACE2 |
| 1 | Association of MMP-9 gene polymorphisms with nephrolithiasis patients. | Mehde AA et al. | 28205286 | No data for ACE2 |
| 1 | Galactosylation of IgA1 Is Associated with Common Variation in C1GALT1. | Gale DP et al. | 28209808 | No data for ACE2 |
| 1 | Relationship of Serum Klotho Level With ACE Gene Polymorphism in Stable Kidney Allograft Recipients. | Zaare Nahandi M et al. | 28270648 | No data for ACE2 |
| 1 | GSTM1 genotype is an independent prognostic factor in clear cell renal cell carcinoma. | Coric VM et al. | 28284893 | No data for ACE2 |
| 1 | Effect of AMP-activated protein kinase subunit alpha 2 (PRKAA2) genetic polymorphisms on susceptibility to type 2 diabetes mellitus and diabetic nephropathy in a Chinese population. | Li Q et al. | 28322508 | No data for ACE2 |
| 1 | Transcriptional Coactivator p300 and Silent Information Regulator 1 (SIRT1) Gene Polymorphism Associated with Diabetic Kidney Disease in a Chinese Cohort. | Tang K et al. | 28444663 | No data for ACE2 |
| 1 | Association of polymorphic variants of PTPN22, TNF and VDR genes in children with lupus nephritis: A study in Colombian family triads. | Garavito G et al. | 28527290 | No data for ACE2 |
| 1 | The Contribution of MMP-7 Promoter Polymorphisms in Renal Cell Carcinoma. | Liao CH et al. | 28652430 | No data for ACE2 |
| 1 | MDR-1 and CYP3A5 Polymorphisms in Pediatric Idiopathic Nephrotic Syndrome: Impact on Susceptibility and Response to Steroids (Preliminary Results). | Moussa A et al. | 28792718 | No data for ACE2 |

| | | | | |
|---|---|---|---|---|
| 1 | CYP3A genotypes of donors but not those of the patients increase the risk of acute rejection in renal transplant recipients on calcineurin inhibitors: a pilot study. | Gervasini G et al. | 29043387 | No data for ACE2 |
| 1 | Association of EPHX2 R287Q Polymorphism with Diabetic Nephropathy in Chinese Type 2 Diabetic Patients. | Ma L et al. | 29629376 | No data for ACE2 |
| 1 | Systemic redox biomarkers and their relationship to prognostic risk markers in autosomal dominant polycystic kidney disease and IgA nephropathy. | Tariq A et al. | 29655960 | No data for ACE2 |
| 1 | Collagenase-1 (-1607 1G/2G), Gelatinase-A (-1306 C/T), Stromelysin-1 (-1171 5A/6A) functional promoter polymorphisms in risk prediction of type 2 diabetic nephropathy. | Gantala SR et al. | 29883760 | No data for ACE2 |
| 1 | Influence of uridine diphosphate-glucuronosyltransferases (1A9) polymorphisms on mycophenolic acid pharmacokinetics in patients with renal transplant. | Ciftci HS et al. | 30012031 | No data for ACE2 |
| 1 | CpG island in the region of an autosomal dominant polycystic kidney disease locus defines the 5' end of a gene encoding a putative proton channel. | Gillespie GA et al. | 1709739 | Non-human study |
| 1 | Recombinant human acetylcholinesterase is secreted from transiently transfected 293 cells as a soluble globular enzyme. | Velan B et al. | 1849451 | Non-human study |
| 1 | Enhanced acetaminophen toxicity in rats with bilirubin glucuronyl transferase deficiency. | de Morais SM et al. | 2501210 | Non-human study |
| 1 | Control of lysosomal acid phosphatase expression in man-mouse cell hybrids. | Shaws TB et al. | 4856818 | Non-human study |
| 1 | Kallikrein and amylase contents in tissues from a mutant mouse model for human cystic fibrosis. | Catanzaro OL et al. | 6186886 | Non-human study |
| 1 | Effect of phosphate supplementation on the expression of the mutant phenotype in murine X-linked hypophosphatemic rickets. | Tenenhouse HS et al. | 7873297 | Non-human study |
| 1 | Purification and characterization of UDP-GlcNAc:IV3 beta Gal-Gb4Cer beta-1,6-GlcNAc transferase from mouse kidney. | Sekine M et al. | 7983056 | Non-human study |
| 1 | Production and characterization of recombinant Goodpasture antigen in insect cells. | Turner N et al. | 8006020 | Non-human study |

| | | | | |
|---|---|---|---|---|
| 1 | Expression of a dominant allele of human ARF1 inhibits membrane traffic in vivo. | Zhang CJ et al. | 8294513 | Non-human study |
| 1 | Testosterone modulation of N-acetylation in mouse kidney. | Smolen TN et al. | 8437129 | Non-human study |
| 1 | Evidence that syntaxin 1A is involved in storage in the secretory pathway. | Bittner MA et al. | 8626670 | Non-human study |
| 1 | Role of the ceramide-signaling pathway in cytokine responses to P-fimbriated Escherichia coli. | Hedlund M et al. | 8642245 | Non-human study |
| 1 | Tissue-specific imprinting of the mouse insulin-like growth factor II receptor gene correlates with differential allele-specific DNA methylation. | Hu JF et al. | 9482664 | Non-human study |
| 1 | Chronic renal failure in a mouse model of human adenine phosphoribosyltransferase deficiency. | Stockelman MG et al. | 9689017 | Non-human study |
| 1 | Renal angiotensin converting enzyme promotes renal damage during ureteral obstruction | Stoneking BJ et al. | 9719278 | Non-human study |
| 1 | Detection of mutation(s) or polymorphic loci in the genome of experimental animal and human cancer tissues by RAPD/AP-PCR depend on DNA polymerase. | Singh KP et al. | 10087325 | Non-human study |
| 1 | Truncated human leptin (delta133) associated with extreme obesity undergoes proteasomal degradation after defective intracellular transport. | Rau H et al. | 10098508 | Non-human study |
| 1 | The mll-AF9 gene fusion in mice controls myeloproliferation and specifies acute myeloid leukaemogenesis. | Dobson CL et al. | 10393173 | Non-human study |
| 1 | Nitric oxide-dependent renal vasodilatation is not altered in rat with rHuEpo-induced hypertension. | Migliori M et al. | 10394113 | Non-human study |
| 1 | Decreased matrix metalloproteinase activity in the kidneys of hereditary nephrotic mice (ICGN strain). | Uchio K et al. | 11014984 | Non-human study |
| 1 | p300 interacts with the nuclear proto-oncoprotein SYT as part of the active control of cell adhesion. | Eid JE et al. | 11030627 | Non-human study |

| | | | | |
|---|---|---|---|---|
| 1 | Telomerase inhibition in RenCa, a murine tumor cell line with short telomeres, by overexpression of a dominant negative mTERT mutant, reveals fundamental differences in telomerase regulation between human and murine cells. | Sachsinger J et al. | 11454711 | Non-human study |
| 1 | An alternative promoter of the human neuronal nitric oxide synthase gene is expressed specifically in Leydig cells. | Wang Y et al. | 11786430 | Non-human study |
| 1 | Three novel sarco/endoplasmic reticulum Ca2+-ATPase (SERCA) 3 isoforms. Expression, regulation, and function of the membranes of the SERCA3 family. | Martin V et al. | 11956212 | Non-human study |
| 1 | The gene INPPL1, encoding the lipid phosphatase SHIP2, is a candidate for type 2 diabetes in rat and man. | Marion E et al. | 12086927 | Non-human study |
| 1 | Organizational diversity among distinct glycoprotein endoplasmic reticulum-associated degradation programs. | Cabral CM et al. | 12181335 | Non-human study |
| 1 | Metabolic fate of pitavastatin (NK-104), a new inhibitor of 3-hydroxy-3-methyl-glutaryl coenzyme A reductase. Effects on drug-metabolizing systems in rats and humans. | Fujino H et al. | 12442637 | Non-human study |
| 1 | Modulation of splicing events in histone deacetylase 3 by various extracellular and signal transduction pathways. | Gray SG et al. | 12691522 | Non-human study |
| 1 | Use of the tetracycline system for inducible protein synthesis in the kidney. | Gallagher AR et al. | 12874458 | Non-human study |
| 1 | Implications of glucose transporter protein type 1 (GLUT1)-haplodeficiency in embryonic stem cells for their survival in response to hypoxic stress. | Heilig C et al. | 14578187 | Non-human study |
| 1 | Susceptibility to vascular neoplasms but no increased susceptibility to renal carcinogenesis in Vhl knockout mice. | Kleymenova E et al. | 14604887 | Non-human study |
| 1 | Gain-of-function polymorphism in mouse and human Ltk: implications for the pathogenesis of systemic lupus erythematosus. | Li N et al. | 14695357 | Non-human study |
| 1 | Cytotoxicity of RNases is increased by cationization and counteracted by K(Ca) channels. | Ilinskaya ON et al. | 14733942 | Non-human study |
| 1 | N-Domain angiotensin I-converting enzyme expression in renal artery of Wistar, Wistar Kyoto, and spontaneously hypertensive rats. | Bueno V et al. | 15194348 | Non-human study |

| | | | | |
|---|---|---|---|---|
| 1 | Fluorescence resonance energy transfer reports properties of syntaxin1a interaction with Munc18-1 in vivo. | Liu J et al. | 15489225 | Non-human study |
| 1 | Sprouty1 is a critical regulator of GDNF/RET-mediated kidney induction. | Basson MA et al. | 15691764 | Non-human study |
| 1 | Alternative splicing of vitamin D-24-hydroxylase: a novel mechanism for the regulation of extrarenal 1,25-dihydroxyvitamin D synthesis. | Ren S et al. | 15788398 | Non-human study |
| 1 | Hypoxia induces a functionally significant and translationally efficient neuronal NO synthase mRNA variant. | Ward ME et al. | 16276418 | Non-human study |
| 1 | Luteinizing hormone receptor ectodomain splice variant misroutes the full-length receptor into a subcompartment of the endoplasmic reticulum. | Apaja PM et al. | 16495341 | Non-human study |
| 1 | N-terminal residues control proteasomal degradation of RGS2, RGS4, and RGS5 in human embryonic kidney 293 cells. | Bodenstein J et al. | 17220356 | Non-human study |
| 1 | Characterization of transcriptional regulation of neurogranin by nitric oxide and the role of neurogranin in SNP-induced cell death: implication of neurogranin in an increased neuronal susceptibility to oxidative stress. | Gui J et al. | 17389928 | Non-human study |
| 1 | Fecal carriage of Escherichia coli O157:H7 and carcass contamination in cattle at slaughter in northern Italy. | Alonso S et al. | 17661289 | Non-human study |
| 1 | Generation and functional characterization of mice with a disrupted glutathione S-transferase, theta 1 gene. | Fujimoto K et al. | 17827337 | Non-human study |
| 1 | Functional genetic variation in aminopeptidase A (ENPEP): lack of clear association with focal and segmental glomerulosclerosis (FSGS). | Tonna S et al. | 18206321 | Non-human study |
| 1 | Hepatitis C virus replication is inhibited by 22beta-methoxyolean-12-ene-3beta, 24(4beta)-diol (ME3738) through enhancing interferon-beta. | Hiasa Y et al. | 18459156 | Non-human study |
| 1 | Characterization of a novel cardiac isoform of the cell cycle-related kinase that is regulated during heart failure. | Qiu H et al. | 18508765 | Non-human study |
| 1 | Increased insulin action in SKIP heterozygous knockout mice. | Ijuin T et al. | 18573875 | Non-human study |
| 1 | Mitochondrial dysfunction in mut methylmalonic acidemia. | Chandler RJ et al. | 19088183 | Non-human study |
| 1 | Overexpression of cytochrome P450 4F2 in mice increases 20-hydroxyeicosatetraenoic acid production and arterial blood pressure. | Liu X et al. | 19279555 | Non-human study |

| | | | | |
|---|---|---|---|---|
| 1 | Kallikrein genes are associated with lupus and glomerular basement membrane-specific antibody-induced nephritis in mice and humans. | Liu K et al. | 19307730 | Non-human study |
| 1 | p38MAPK controls expression of multiple cell cycle inhibitors and islet proliferation with advancing age. | Wong ES et al. | 19619499 | Non-human study |
| 1 | Blocking the salt-inducible kinase 1 network prevents the increases in cell sodium transport caused by a hypertension-linked mutation in human alpha-adducin. | Stenstrom K et al. | 19657284 | Non-human study |
| 1 | Maitotoxin converts the plasmalemmal Ca(2+) pump into a Ca(2+)-permeable nonselective cation channel. | Sinkins WG et al. | 19794142 | Non-human study |
| 1 | MyoD control of SKIP expression during pig skeletal muscle development. | Xiong Q et al. | 20336382 | Non-human study |
| 1 | Targeted deletion of the Nesp55 DMR defines another Gnas imprinting control region and provides a mouse model of autosomal dominant PHP-Ib. | Frohlich LF et al. | 20427744 | Non-human study |
| 1 | Autosomal recessive polycystic kidney disease epithelial cell model reveals multiple basolateral epidermal growth factor receptor sorting pathways. | Ryan S et al. | 20519437 | Non-human study |
| 1 | The activating mutation R201C in GNAS promotes intestinal tumourigenesis in Apc(Min/+) mice through activation of Wnt and ERK1/2 MAPK pathways. | Wilson CH et al. | 20531296 | Non-human study |
| 1 | Pitchfork regulates primary cilia disassembly and left-right asymmetry. | Kinzel D et al. | 20643351 | Non-human study |
| 1 | Mediation of CTCF transcriptional insulation by DEAD-box RNA-binding protein p68 and steroid receptor RNA activator SRA. | Yao H et al. | 20966046 | Non-human study |
| 1 | A common polymorphism in the human aromatase gene alters the risk for polycystic ovary syndrome and modifies aromatase activity in vitro. | Wang H et al. | 21282199 | Non-human study |
| 1 | Transgenic overexpression of the extra-large GsÎ± variant XLÎ±s enhances GsÎ±-mediated responses in the mouse renal proximal tubule in vivo. | Liu Z et al. | 21303955 | Non-human study |
| 1 | Rat Ace allele variation determines susceptibility to AngII-induced renal damage | Kamilic J et al. | 21788250 | Non-human study |
| 1 | Carnosine treatment largely prevents alterations of renal carnosine metabolism in diabetic mice. | Peters V et al. | 21833769 | Non-human study |

| | | | | |
|---|---|---|---|---|
| 1 | YME1L controls the accumulation of respiratory chain subunits and is required for apoptotic resistance, cristae morphogenesis, and cell proliferation. | Stiburek L et al. | 22262461 | Non-human study |
| 1 | Primary hyperoxaluria in Coton de Tulear. | Vidgren G et al. | 22486513 | Non-human study |
| 1 | Role of angiotensin-converting enzyme (ACE and ACE2) imbalance on tourniquet-induced remote kidney injury in a mouse hindlimb ischemia-reperfusion model | Yang XH et al. | 22580272 | Non-human study |
| 1 | Tubule-specific ablation of endogenous Î²-catenin aggravates acute kidney injury in mice. | Zhou D et al. | 22622501 | Non-human study |
| 1 | Î±1,3-galactosyltransferase deficiency in germ-free miniature pigs increases N-glycolylneuraminic acids as the xenoantigenic determinant in pig-human xenotransplantation. | Park JY et al. | 22775484 | Non-human study |
| 1 | Cystic echinococcosis in Romania: an epidemiological survey of livestock demonstrates the persistence of hyperendemicity. | Mitrea IL et al. | 23075460 | Non-human study |
| 1 | First WNK4-hypokalemia animal model identified by genome-wide association in Burmese cats. | Gandolfi B et al. | 23285264 | Non-human study |
| 1 | Identification of aldo-keto reductases as NRF2-target marker genes in human cells. | Jung KA et al. | 23305850 | Non-human study |
| 1 | An inducible transgenic mouse model for familial hypertension with hyperkalaemia (Gordon's syndrome or pseudohypoaldosteronism type II). | Chowdhury JA et al. | 23336180 | Non-human study |
| 1 | Presence of N-glycosylated transthyretin in plasma of V30M carriers in familial amyloidotic polyneuropathy: an escape from ERAD. | Teixeira AC et al. | 23387326 | Non-human study |
| 1 | Multi-species comparative analysis of the equine ACE gene identifies a highly conserved potential transcription factor binding site in intron 16. | Hamilton NA et al. | 23408978 | Non-human study |
| 1 | Renal collecting duct NOS1 maintains fluid-electrolyte homeostasis and blood pressure. | Hyndman KA et al. | 23608660 | Non-human study |
| 1 | ARHGDIA mutations cause nephrotic syndrome via defective RHO GTPase signaling. | Gee HY et al. | 23867502 | Non-human study |

| | | | | |
|---|---|---|---|---|
| 1 | A knock-in mouse model of human PHD2 gene-associated erythrocytosis establishes a haploinsufficiency mechanism. | Arsenault PR et al. | 24121508 | Non-human study |
| 1 | The Pex1-G844D mouse: a model for mild human Zellweger spectrum disorder. | Hiebler S et al. | 24503136 | Non-human study |
| 1 | Holocarboxylase synthetase interacts physically with nuclear receptor co-repressor, histone deacetylase 1 and a novel splicing variant of histone deacetylase 1 to repress repeats. | Liu D et al. | 24840043 | Non-human study |
| 1 | Factor VIIa binding to endothelial cell protein C receptor protects vascular barrier integrity in vivo. | Sundaram J et al. | 24977291 | Non-human study |
| 1 | Atrial natriuretic peptide locally counteracts the deleterious effects of cardiomyocyte mineralocorticoid receptor activation. | Nakagawa H et al. | 25027872 | Non-human study |
| 1 | The T309G murine double minute 2 gene polymorphism is an independent prognostic factor for patients with renal cell carcinoma. | de Martino M et al. | 25415135 | Non-human study |
| 1 | Generation of WNK1 knockout cell lines by CRISPR/Cas-mediated genome editing. | Roy A et al. | 25477473 | Non-human study |
| 1 | PTPN2 controls differentiation of CD4â�º T cells and limits intestinal inflammation and intestinal dysbiosis. | Spalinger MR et al. | 25492475 | Non-human study |
| 1 | A functional 12T-insertion polymorphism in the ATP1A1 promoter confers decreased susceptibility to hypertension in a male Sardinian population. | Herrera VL et al. | 25615575 | Non-human study |
| 1 | BAP1 missense mutation c.2054 A>T (p.E685V) completely disrupts normal splicing through creation of a novel 5' splice site in a human mesothelioma cell line. | Morrison A et al. | 25830670 | Non-human study |
| 1 | Identification of a receptor for extracellular renalase. | Wang L et al. | 25906147 | Non-human study |
| 1 | Mammalian target of rapamycin signaling inhibition ameliorates vascular calcification via Klotho upregulation. | Zhao Y et al. | 26061549 | Non-human study |
| 1 | TNFÎ± Amplifies DNaseI Expression in Renal Tubular Cells while IL-1Î² Promotes Nuclear DNaseI Translocation in an Endonuclease-Inactive Form. | Thiyagarajan D et al. | 26065428 | Non-human study |
| 1 | DAPIT Over-Expression Modulates Glucose Metabolism and Cell Behaviour in HEK293T Cells. | Kontro H et al. | 26161955 | Non-human study |

| | | | | |
|---|---|---|---|---|
| 1 | Allelic Variants in Arhgef11 via the Rho-Rock Pathway Are Linked to Epithelial-Mesenchymal Transition and Contributes to Kidney Injury in the Dahl Salt-Sensitive Rat. | Jia Z et al. | 26172442 | Non-human study |
| 1 | Human GRK4Î³142V Variant Promotes Angiotensin II Type I Receptor-Mediated Hypertension via Renal Histone Deacetylase Type 1 Inhibition. | Wang Z et al. | 26667412 | Non-human study |
| 1 | Dynamin-2 is a novel NOS1Î² interacting protein and negative regulator in the collecting duct. | Hyndman KA et al. | 26791826 | Non-human study |
| 1 | Re-engineering the zinc fingers of PRDM9 reverses hybrid sterility in mice. | Davies B et al. | 26840484 | Non-human study |
| 1 | Cardiac angiotensin-(1-12) expression and systemic hypertension in rats expressing the human angiotensinogen gene. | Ferrario CM et al. | 26873967 | Non-human study |
| 1 | From man to fish: What can Zebrafish tell us about ApoL1 nephropathy? | Olabisi O et al. | 27509583 | Non-human study |
| 1 | Cabozantinib inhibits tumor growth and metastasis of a patient-derived xenograft model of papillary renal cell carcinoma with MET mutation. | Zhao H et al. | 27715452 | Non-human study |
| 1 | Renin-angiotensin system transgenic mouse model recapitulates pathophysiology similar to human preeclampsia with renal injury that may be mediated through VEGF. | Denney JM et al. | 27927648 | Non-human study |
| 1 | Inhibition of TRPC6 channels ameliorates renalÂ fibrosis and contributes to renal protectionÂ byÂ soluble klotho. | Wu YL et al. | 27979597 | Non-human study |
| 1 | Activated ERK1/2 increases CD44 in glomerular parietal epithelial cells leading to matrix expansion. | Roeder SS et al. | 27998643 | Non-human study |
| 1 | Parallel microarray profiling identifies ErbB4 as a determinant of cyst growth in ADPKD and a prognostic biomarker for disease progression. | Streets AJ et al. | 28077374 | Non-human study |
| 1 | Targeting mTOR Signaling Can Prevent the Progression of FSGS. | Zschiedrich S et al. | 28270414 | Non-human study |
| 1 | The leukotriene B(4)-leukotriene B(4) receptor axisÂ promotes cisplatin-induced acute kidney injuryÂ byÂ modulating neutrophil recruitment. | Deng B et al. | 28318626 | Non-human study |
| 1 | DNase-active TREX1 frame-shift mutants induce serologic autoimmunity in mice. | Sakai T et al. | 28325644 | Non-human study |
| 1 | Human SR-BII mediates SAA uptake and contributes to SAA pro-inflammatory signaling in vitro and in vivo. | Baranova IN et al. | 28423002 | Non-human study |

| | | | | |
|---|---|---|---|---|
| 1 | Bim gene dosage is critical in modulating nephron progenitor survival in the absence of microRNAs during kidney development. | Cerqueira DM et al. | 28446592 | Non-human study |
| 1 | DBA/2J Haplotype on Distal Chromosome 2 Reduces Mertk Expression, Restricts Efferocytosis, and Increases Susceptibility to Atherosclerosis. | Kayashima Y et al. | 28473436 | Non-human study |
| 1 | Inhibition of HDAC enhances STAT acetylation, blocks NF-Î°B, and suppresses the renal inflammation and fibrosis in Npr1 haplotype male mice. | Kumar P et al. | 28566502 | Non-human study |
| 1 | The regulatory 1Î± subunit of protein kinase A modulates renal cystogenesis. | Ye H et al. | 28615245 | Non-human study |
| 1 | Functional analysis of human aromatic amino acid transporter MCT10/TAT1 using the yeast Saccharomyces cerevisiae. | Uemura S et al. | 28754537 | Non-human study |
| 1 | Kindlin-2 Association with Rho GDP-Dissociation Inhibitor Î± Suppresses Rac1 Activation and Podocyte Injury. | Sun Y et al. | 28775002 | Non-human study |
| 1 | Interference with GsÎ±-Coupled Receptor Signaling in Renin-Producing Cells Leads to Renal Endothelial Damage. | Lachmann P et al. | 28775003 | Non-human study |
| 1 | Blood Pressure Control by a Secreted FGFBP1 (Fibroblast Growth Factor-Binding Protein). | Tassi E et al. | 29158353 | Non-human study |
| 1 | Caspase-independent programmed cell death triggers Ca(2)PO(4) deposition in an in vitro model of nephrocalcinosis. | Priante G et al. | 29208768 | Non-human study |
| 1 | Knockout of the interleukin-36 receptor protects against renal ischemia-reperfusion injury by reduction of proinflammatory cytokines. | Nishikawa H et al. | 29241623 | Non-human study |
| 1 | Xyloketal B exerts antihypertensive effect in renovascular hypertensive rats via the NO-sGC-cGMP pathway and calcium signaling. | Zhao LY et al. | 29595193 | Non-human study |
| 1 | Mice Carrying a Dominant-Negative Human PI3K Mutation Are Protected From Obesity and Hepatic Steatosis but Not Diabetes. | Solheim MH et al. | 29724723 | Non-human study |
| 1 | A homozygous KAT2B variant modulates the clinical phenotype of ADD3 deficiency in humans and flies. | Goncalves S et al. | 29768408 | Non-human study |
| 1 | Haploinsufficiency of the Mouse Atp6v1b1 Gene Leads to a Mild Acid-Base Disturbance with Implications for Kidney Stone Disease. | Bourgeois S et al. | 29843146 | Non-human study |
| 1 | RFLP-DR beta and serological HLA-DR typing of 200 kidney recipients and 1000 controls. | Mytilineos J et al. | 1975140 | Not a case-control study |

| | | | | |
|---|---|---|---|---|
| 1 | Superoxide dismutase isozymes in different human tissues, their genetic control and intracellular localization. | Beckman G et al. | 4775457 | Not a case-control study |
| 1 | Genetic counselling in lethal X-linked disorders. | Emery AE et al. | 4976958 | Not a case-control study |
| 1 | DNA-based presymptomatic diagnosis for the von Hippel-Lindau disease by linkage analysis. | Olschwang S et al. | 7552140 | Not a case-control study |
| 1 | Role of the deletion of polymorphism of the angiotensin converting enzyme gene in the progression and therapeutic responsiveness of IgA nephropathy. | Yoshida H et al. | 7593601 | Not a case-control study |
| 1 | Mistyping of the human angiotensin-converting enzyme gene polymorphism: frequency, causes and possible methods to avoid errors in typing. | Ueda S et al. | 8863184 | Not a case-control study |
| 1 | Analysis of linkage disequilibrium between polymorphisms in the KCNJ9 gene with type 2 diabetes mellitus in Pima Indians. | Wolford JK et al. | 11350189 | Not a case-control study |
| 1 | A family of autosomal dominant hypocalcemia with a positive correlation between serum calcium and magnesium: identification of a novel gain of function mutation (Ser(820)Phe) in the calcium-sensing receptor. | Nagase T et al. | 12050233 | Not a case-control study |
| 1 | Captopril enhances transforming growth factor (TGF)-beta1 expression in peripheral blood mononuclear cells: a mechanism independent from angiotensin converting enzyme inhibition? A study in cyclosporine-treated kidney-transplanted patients. | Di Paolo S et al. | 12499886 | Not a case-control study |
| 1 | Clinical assessment and mutation analysis of Kallmann syndrome 1 (KAL1) and fibroblast growth factor receptor 1 (FGFR1, or KAL2) in five families and 18 sporadic patients. | Sato N et al. | 15001591 | Not a case-control study |
| 1 | The effect of CYP3A5 and MDR1 (ABCB1) polymorphisms on cyclosporine and tacrolimus dose requirements and trough blood levels in stable renal transplant patients. | Haufroid V et al. | 15167702 | Not a case-control study |
| 1 | Antiproteinuric effect of candesartan cilexetil in Japanese subjects with type 2 diabetes and nephropathy. | Haneda M et al. | 15364166 | Not a case-control study |
| 1 | An unusual association of contralateral congenital small kidney, reduced renal function and hyperparathyroidism in sponge kidney patients: on the track of the molecular basis. | Gambaro G et al. | 15814540 | Not a case-control study |

| | | | | |
|---|---|---|---|---|
| 1 | Presentation and role of transplantation in adult patients with type 1 primary hyperoxaluria and the I244T AGXT mutation: Single-center experience. | Lorenzo V et al. | 16912707 | Not a case-control study |
| 1 | Familial nonsyndromic pheochromocytoma. | Opocher G et al. | 17102081 | Not a case-control study |
| 1 | Synergistic expression of angiotensin-converting enzyme (ACE) and ACE2 in human renal tissue and confounding effects of hypertension on the ACE to ACE2 ratio | Wakahara et al. | 17303661 | Not a case-control study |
| 1 | Verification of consumers' experiences and perceptions of genetic discrimination and its impact on utilization of genetic testing. | Barlow-Stewart K et al. | 19287242 | Not a case-control study |
| 1 | Porphyria in Switzerland, 15 years experience. | Schneider-Yin X et al. | 19350426 | Not a case-control study |
| 1 | Whole-genome linkage and association scan in primary, nonsyndromic vesicoureteric reflux. | Cordell HJ et al. | 19959718 | Not a case-control study |
| 1 | Identification of a gene for renal-hepatic-pancreatic dysplasia by microarray-based homozygosity mapping. | Fiskerstrand T et al. | 20007846 | Not a case-control study |
| 1 | Bardet-Biedl syndrome in Denmark--report of 13 novel sequence variations in six genes. | Hjortshoj TD et al. | 20120035 | Not a case-control study |
| 1 | Chromosome 7p linkage and association study for diabetes related traits and type 2 diabetes in an African-American population enriched for nephropathy. | Leak TS et al. | 20144192 | Not a case-control study |
| 1 | MYH9 genetic variants associated with glomerular disease: what is the role for genetic testing? | Kopp JB et al. | 20807613 | Not a case-control study |
| 1 | A prospective, open-label, observational clinical cohort study of the association between delayed renal allograft function, tacrolimus exposure, and CYP3A5 genotype in adult recipients. | Kuypers DR et al. | 21118736 | Not a case-control study |
| 1 | Acute kidney injury reduces the hepatic metabolism of midazolam in critically ill patients. | Kirwan CJ et al. | 22005822 | Not a case-control study |
| 1 | ABCB1 polymorphisms are associated with cyclosporine-induced nephrotoxicity and gingival hyperplasia in renal transplant recipients. | Garcia M et al. | 22886152 | Not a case-control study |

| | | | | |
|---|---|---|---|---|
| 1 | Novel SMARCAL1 bi-allelic mutations associated with a chromosomal breakage phenotype in a severe SIOD patient. | Simon AJ et al. | 24197801 | Not a case-control study |
| 1 | Brief Report: identification of MTMR3 as a novel susceptibility gene for lupus nephritis in northern Han Chinese by shared-gene analysis with IgA nephropathy. | Zhou XJ et al. | 24943867 | Not a case-control study |
| 1 | An efficient and comprehensive strategy for genetic diagnostics of polycystic kidney disease. | Eisenberger T et al. | 25646624 | Not a case-control study |
| 1 | ABCB1 (MDR-1) pharmacogenetics of tacrolimus in renal transplanted patients: a Next Generation Sequencing approach. | Tavira B et al. | 25781547 | Not a case-control study |
| 1 | Novel mutations in the inverted formin 2 gene of Chinese families contribute to focal segmental glomerulosclerosis. | Xie J et al. | 26039629 | Not a case-control study |
| 1 | A Novel von Hippel Lindau Gene Intronic Variant and Its Reclassification from VUS to Pathogenic: the Impact on a Large Family. | Sexton A et al. | 26323595 | Not a case-control study |
| 1 | Genome-wide Association Studies Identify Genetic Loci Associated With Albuminuria in Diabetes. | Teumer A et al. | 26631737 | Not a case-control study |
| 1 | Updated genetic testing of Italian patients referred with a clinical diagnosis of primary hyperoxaluria. | Pelle A et al. | 26946417 | Not a case-control study |
| 1 | Polypoid urothelial tumor with inverted growth pattern in the renal pelvis: morphologic and molecular characteristics of a unique diagnostic entity. | Wobker SE et al. | 27574810 | Not a case-control study |
| 1 | Haploinsufficiency in tumor predisposition syndromes: altered genomic transcription in morphologically normal cells heterozygous for VHL or TSC mutation. | Peri S et al. | 27682873 | Not a case-control study |
| 1 | CYP3A4*1G and CYP3A5*3 genetic polymorphisms alter the antihypertensive efficacy of amlodipine in patients with hypertension following renal transplantationâ€©. | Huang Y et al. | 27841150 | Not a case-control study |
| 1 | Clinical, Genetic and Innate Immunity Characteristics of Melanoma in Organ Transplant Recipients. | Brocard A et al. | 27868139 | Not a case-control study |
| 1 | Clinical impact of the CYP3A5 6986A>G allelic variant on kidney transplantation outcomes. | Flahault A et al. | 27977332 | Not a case-control study |
| 1 | Smoking has no impact on survival and it is not associated with ACE gene I/D polymorphism in hemodialysis patients. | Kiss I et al. | 28058974 | Not a case-control study |

| | | | | |
|---|---|---|---|---|
| 1 | Evaluation of Glutathione Peroxidase and KCNJ11 Gene Polymorphisms in Patients with New Onset Diabetes Mellitus After Renal Transplantation. | Yalin GY et al. | 28073131 | Not a case-control study |
| 1 | The CYP3A biomarker 4Î²-hydroxycholesterol does not improve tacrolimus dose predictions early after kidney transplantation. | Storset E et al. | 28146606 | Not a case-control study |
| 1 | Prograf produces more benefits for CYP3A5 low expression patients in early stage after kidney transplantation. | Fan B et al. | 28157649 | Not a case-control study |
| 1 | The genetic and clinical spectrum of a large cohort of patients with distal renal tubular acidosis. | Palazzo V et al. | 28233610 | Not a case-control study |
| 1 | Impact of the CYP3A5 genotype on the distributions of dose-adjusted trough concentrations and incidence of rejection in Japanese renal transplant recipients receiving different tacrolimus formulations. | Niioka T et al. | 28271256 | Not a case-control study |
| 1 | Influence of ABCC2, CYP2C8, and CYP2J2 Polymorphisms on Tacrolimus and Mycophenolate Sodium-Based Treatment in Brazilian Kidney Transplant Recipients. | Genvigir FDV et al. | 28316087 | Not a case-control study |
| 1 | Role of ACE and IL-1Î² Gene Polymorphisms in Erythropoeitin Hyporesponsive Patients with Chronic Kidney Disease with Anemia. | Nand N et al. | 28457029 | Not a case-control study |
| 1 | Pretransplant 4Î²-hydroxycholesterol does not predict tacrolimus exposure or dose requirements during the first days after kidney transplantation. | Vanhove T et al. | 28603840 | Not a case-control study |
| 1 | Sequencing of FIC1, BSEP and MDR3 in a large cohort of patients with cholestasis revealed a high number of different genetic variants. | Droge C et al. | 28733223 | Not a case-control study |
| 1 | Low renal but high extrarenal phenotype variability in Schimke immuno-osseous dysplasia. | Lipska-Zietkiewicz BS et al. | 28796785 | Not a case-control study |
| 1 | Wuzhi Tablet (Schisandra sphenanthera Extract) is a Promising Tacrolimus-Sparing Agent for Renal Transplant Recipients Who are CYP3A5 Expressers: a Two-Phase Prospective Study. | Li J et al. | 28864749 | Not a case-control study |
| 1 | Crizotinib achieves long-lasting disease control in advanced papillary renal-cell carcinoma type 1 patients with MET mutations or amplification. EORTC 90101 CREATE trial. | Schoffski P et al. | 29149761 | Not a case-control study |

| | | | | |
|---|---|---|---|---|
| 1 | Cost-effectiveness analysis of elbasvir-grazoprevir regimen for treating hepatitis C virus genotype 1 infection in stage 4-5 chronic kidney disease patients in France. | Maunoury F et al. | 29543897 | Not a case-control study |
| 1 | Late-onset acid maltase deficiency. Detection of patients and heterozygotes by urinary enzyme assay. | Mehler M et al. | 9923 | Not a renal disease focus |
| 1 | Differential inhibition of the products of the human alkaline phosphatase loci. | Mulivor RA et al. | 686677 | Not a renal disease focus |
| 1 | A classification of tumor development based on an analysis of enzymes in tissue sections of hypernephroid carcinoma in man. | Wachsmuth ED et al. | 1016198 | Not a renal disease focus |
| 1 | Identification of mutations associated with peroxisome-to-mitochondrion mistargeting of alanine/glyoxylate aminotransferase in primary hyperoxaluria type 1. | Purdue PE et al. | 1703535 | Not a renal disease focus |
| 1 | Concentrations of ochratoxin A in the urine of endemic nephropathy patients and controls in Bulgaria: lack of detection of 4-hydroxyochratoxin A. | Castegnaro M et al. | 1820331 | Not a renal disease focus |
| 1 | T-cell receptor beta-subunit gene polymorphism and autoimmune disease. | Niven MJ et al. | 1969400 | Not a renal disease focus |
| 1 | Role of P450IID6, the target of the sparteine-debrisoquin oxidation polymorphism, in the metabolism of imipramine. | Brosen K et al. | 2060250 | Not a renal disease focus |
| 1 | Immunological heterogeneity of hepatic alanine:glyoxylate aminotransferase in primary hyperoxaluria type 1. | Wise PJ et al. | 2443389 | Not a renal disease focus |
| 1 | Renin in blood vessels in human pulmonary tumors. An immunohistochemical and biochemical study. | Taylor GM et al. | 2450464 | Not a renal disease focus |
| 1 | Elevated concentrations of brain-type glycogen phosphorylase in renal cell carcinoma. | Takashi M et al. | 2515181 | Not a renal disease focus |
| 1 | Origin of rare Ha-ras alleles: relationship of VTR length to a 5' polymorphic Xho I site. | Baxter GD et al. | 2558964 | Not a renal disease focus |
| 1 | Loss of heterozygosity at the c-raf locus in small cell lung carcinoma. | Sithanandam G et al. | 2566144 | Not a renal disease focus |
| 1 | Mechanisms of suppression of renal kallikrein activity in low renin essential hypertension and renoparenchymal hypertension. | Shimamoto K et al. | 2676859 | Not a renal disease focus |

| | | | | |
|---|---|---|---|---|
| 1 | Reduced expression of multiple forms of the alpha subunit of the stimulatory GTP-binding protein in pseudohypoparathyroidism type Ia. | Carter A et al. | 2890163 | Not a renal disease focus |
| 1 | The beta-subunit of follicle-stimulating hormone is deleted in patients with aniridia and Wilms' tumour, allowing a further definition of the WAGR locus. | Glaser T et al. | 3014343 | Not a renal disease focus |
| 1 | Further studies on the activity and subcellular distribution of alanine:glyoxylate aminotransferase in the livers of patients with primary hyperoxaluria type 1. | Danpure CJ et al. | 3416563 | Not a renal disease focus |
| 1 | Acetylator genotype-dependent metabolic activation of carcinogenic N-hydroxyarylamines by S-acetyl coenzyme A-dependent enzymes of inbred hamster tissue cytosols: relationship to arylamine N-acetyltransferase. | Hein DW et al. | 3677303 | Not a renal disease focus |
| 1 | Persistence of streptococcal group A antibody in patients with rheumatic valvular disease. | Dudding BA et al. | 5682941 | Not a renal disease focus |
| 1 | Studies of an abnormal serum albumin unstable upon storage. | Rousseaux J et al. | 6215190 | Not a renal disease focus |
| 1 | Allotypes of properdin factor B(Bf) and lymphocytotoxic antibody production. | Davidson JA et al. | 6399879 | Not a renal disease focus |
| 1 | Control of serum C3 levels by beta 1H and C3b inactivator. | Wyatt RJ et al. | 6445926 | Not a renal disease focus |
| 1 | Leukocyte beta-glucosidase in homozygotes and heterozygotes for Gaucher disease. | Raghavan SS et al. | 6770675 | Not a renal disease focus |
| 1 | Structural organization of the human neuronal nitric oxide synthase gene (NOS1). | Hall AV et al. | 7528745 | Not a renal disease focus |
| 1 | Analysis of meningiomas by methylation- and transcription-based clonality assays. | Zhu J et al. | 7641206 | Not a renal disease focus |
| 1 | Cytochrome P4501A1 gene polymorphism and homozygous deletion of the glutathione S-transferase M1 gene in urothelial cancer patients. | Katoh T et al. | 7697828 | Not a renal disease focus |
| 1 | Distribution of MHC class II alleles in primary systemic vasculitis. | Zhang L et al. | 7731160 | Not a renal disease focus |

| | | | | |
|---|---|---|---|---|
| 1 | Angiotensin-converting enzyme gene polymorphism has no influence on the circulating renin-angiotensin-aldosterone system or blood pressure in normotensive subjects | Lachurie ML et al. | 7796503 | Not a renal disease focus |
| 1 | p53 mutations and MDM-2 amplification in renal cell cancers. | Imai Y et al. | 7824511 | Not a renal disease focus |
| 1 | Frequencies of variants of candidate genes in different age groups of hypertensives. | Zee RY et al. | 7882587 | Not a renal disease focus |
| 1 | Independent, marked associations of alleles of the insulin receptor and dipeptidyl carboxypeptidase-I genes with essential hypertension. | Morris BJ et al. | 8104754 | Not a renal disease focus |
| 1 | Increased expression of vascular permeability factor (vascular endothelial growth factor) and its receptors in kidney and bladder carcinomas. | Brown LF et al. | 8238242 | Not a renal disease focus |
| 1 | Alpha-1-proteinase inhibitor and pulmonary haemorrhage in systemic vasculitis. | O'Donoghue DJ et al. | 8296629 | Not a renal disease focus |
| 1 | Expression of NAD(P)H:quinone oxidoreductase and glutathione S-transferases alpha and pi in human renal cell carcinoma and in kidney cancer-derived cell lines. | Eickelmann P et al. | 8313512 | Not a renal disease focus |
| 1 | A homoallelic Gly317-->Asp mutation in ALPL causes the perinatal (lethal) form of hypophosphatasia in Canadian mennonites. | Greenberg CR et al. | 8406453 | Not a renal disease focus |
| 1 | Angiotensin converting enzyme (ACE) insertion/deletion (I/D) polymorphism, and diabetic retinopathy in subjects with IDDM and NIDDM. | Nagi DK et al. | 8582133 | Not a renal disease focus |
| 1 | Characterization of Gas6, a member of the superfamily of G domain-containing proteins, as a ligand for Rse and Axl. | Mark MR et al. | 8621659 | Not a renal disease focus |
| 1 | C-antineutrophil cytoplasmic antibody positivity in vasculitis patients is associated with the Z allele of alpha-1-antitrypsin, and P-antineutrophil cytoplasmic antibody positivity with the S allele. | Griffith ME et al. | 8671812 | Not a renal disease focus |
| 1 | Absence of mutations in parathyroid hormone (PTH)/PTH-related protein receptor complementary deoxyribonucleic acid in patients with pseudohypoparathyroidism type Ib. | Fukumoto S et al. | 8675577 | Not a renal disease focus |
| 1 | Deletion polymorphism of the angiotensin-converting enzyme gene is independently associated with left ventricular mass and geometric remodeling in systemic hypertension. | Gharavi AG et al. | 8677872 | Not a renal disease focus |

| | | | | |
|---|---|---|---|---|
| 1 | Azathioprine pharmacogenetics: the relationship between 6-thioguanine nucleotides and thiopurine methyltransferase in patients after heart and kidney transplantation. | Schutz E et al. | 8721407 | Not a renal disease focus |
| 1 | Examination of the role of nitric oxide synthase and renal kallikrein as candidate genes for essential hypertension. | Friend LR et al. | 8800585 | Not a renal disease focus |
| 1 | Genetic risk for renal artery stenosis: association with deletion polymorphism in angiotensin 1-converting enzyme gene. | Missouris CG et al. | 8821841 | Not a renal disease focus |
| 1 | Chromosome 11p15.5 regional imprinting: comparative analysis of KIP2 and H19 in human tissues and Wilms' tumors. | Chung WY et al. | 8842727 | Not a renal disease focus |
| 1 | Germline mutations in glial cell line-derived neurotrophic factor (GDNF) and RET in a Hirschsprung disease patient. | Angrist M et al. | 8896568 | Not a renal disease focus |
| 1 | Germline mutations of the RET ligand GDNF are not sufficient to cause Hirschsprung disease. | Salomon R et al. | 8896569 | Not a renal disease focus |
| 1 | Increased expression of the insulin-like growth factor-II gene in Wilms' tumor is not dependent on loss of genomic imprinting or loss of heterozygosity. | Wang WH et al. | 8910385 | Not a renal disease focus |
| 1 | Genetic analysis of the NAT2 and CYP2D6 polymorphisms in white patients with non-insulin-dependent diabetes mellitus. | Agundez JA et al. | 8946479 | Not a renal disease focus |
| 1 | Renal cell carcinoma of end-stage renal disease: a histopathologic and molecular genetic study. | Hughson MD et al. | 8959640 | Not a renal disease focus |
| 1 | Reduced expression of the cyclin-dependent kinase inhibitor gene p57KIP2 in Wilms' tumor. | Thompson JS et al. | 8971182 | Not a renal disease focus |
| 1 | Angiotensin converting enzyme gene polymorphism and renal hemodynamic function in early diabetes. | Miller JA et al. | 8995725 | Not a renal disease focus |
| 1 | Somatic inactivation of the VHL gene in Von Hippel-Lindau disease tumors. | Prowse AH et al. | 9106522 | Not a renal disease focus |
| 1 | Association of diabetic neuropathy with Na/K ATPase gene polymorphism. | Vague P et al. | 9165217 | Not a renal disease focus |
| 1 | Angiotensin I-converting enzyme and angiotensinogen gene polymorphisms in non-insulin-dependent diabetes mellitus. Lack of relationship with diabetic nephropathy and retinopathy in a Caucasian Mediterranean population. | Gutierrez C et al. | 9258285 | Not a renal disease focus |

| | | | | |
|---|---|---|---|---|
| 1 | Influence of polymorphisms of GSTM1 and GSTT1 for risk of renal cell cancer in workers with long-term high occupational exposure to trichloroethene. | Bruning T et al. | 9285043 | Not a renal disease focus |
| 1 | FHIT gene and the FRA3B region are not involved in the genetics of renal cell carcinomas. | Bugert P et al. | 9290948 | Not a renal disease focus |
| 1 | Isolation and characterization of UGT2B15(Y85): a UDP-glucuronosyltransferase encoded by a polymorphic gene. | Levesque E et al. | 9295060 | Not a renal disease focus |
| 1 | Analyses of mutations in the human renal kallikrein (hKLK1) gene and their possible relevance to blood pressure regulation and risk of myocardial infarction. | Berge KE et al. | 9298743 | Not a renal disease focus |
| 1 | Coding mutations in p57KIP2 are present in some cases of Beckwith-Wiedemann syndrome but are rare or absent in Wilms tumors. | O'Keefe D et al. | 9311733 | Not a renal disease focus |
| 1 | Loss of heterozygosity of the nm23-H1 gene in human renal cell carcinomas. | Bosnar MH et al. | 9341897 | Not a renal disease focus |
| 1 | Biological monitoring of workers exposed to low levels of 2-butoxyethanol. | Haufroid V et al. | 9342622 | Not a renal disease focus |
| 1 | Renal ACE immunohistochemical localization in NIDDM patients with nephropathy. | Mizuiri S et al. | 9469501 | Not a renal disease focus |
| 1 | The Captopril Prevention Project (CAPPP) in hypertension--baseline data and current status. | Hansson L et al. | 9495662 | Not a renal disease focus |
| 1 | Angiotensin I-converting enzyme gene polymorphism modulates the consequences of in utero growth retardation on plasma insulin in young adults. | Cambien F et al. | 9519756 | Not a renal disease focus |
| 1 | Contribution of angiotensin I converting enzyme gene polymorphism and angiotensinogen gene polymorphism to blood pressure regulation in essential hypertension. | Mondorf UF et al. | 9524045 | Not a renal disease focus |
| 1 | Evidence of association of the ecNOS gene polymorphism with plasma NO metabolite levels in humans. | Tsukada T et al. | 9535806 | Not a renal disease focus |
| 1 | Human GFRA1: cloning, mapping, genomic structure, and evaluation as a candidate gene for Hirschsprung disease susceptibility. | Angrist M et al. | 9545641 | Not a renal disease focus |
| 1 | Serum paraoxonase activity and its relationship to diabetic complications in patients with non-insulin-dependent diabetes mellitus. | Ikeda Y et al. | 9591753 | Not a renal disease focus |

| | | | | |
|---|---|---|---|---|
| 1 | Inhibition by platelet-activating factor of Src- and hepatocyte growth factor-dependent invasiveness of intestinal and kidney epithelial cells. Phosphatidylinositol 3'-kinase is a critical mediator of tumor invasion. | Kotelevets L et al. | 9603913 | Not a renal disease focus |
| 1 | Angiotensin I-converting enzyme and angiotensinogen gene interaction and prediction of essential hypertension. | Vasku A et al. | 9607178 | Not a renal disease focus |
| 1 | Phosphorylation sites in the autoinhibitory domain participate in p70(s6k) activation loop phosphorylation. | Dennis PB et al. | 9614086 | Not a renal disease focus |
| 1 | EGF-r gene copy number changes in renal cell carcinoma detected by fluorescence in situ hybridization. | Moch H et al. | 9664910 | Not a renal disease focus |
| 1 | Point mutation and homozygous deletion of PTEN/MMAC1 in primary bladder cancers. | Cairns P et al. | 9671402 | Not a renal disease focus |
| 1 | Duplication and overexpression of the mutant allele of the MET proto-oncogene in multiple hereditary papillary renal cell tumours. | Fischer J et al. | 9715275 | Not a renal disease focus |
| 1 | Trisomy 7-harbouring non-random duplication of the mutant MET allele in hereditary papillary renal carcinomas. | Zhuang Z et al. | 9731534 | Not a renal disease focus |
| 1 | An analysis of phenotypic variation in the familial cancer syndrome von Hippel-Lindau disease: evidence for modifier effects. | Webster AR et al. | 9758595 | Not a renal disease focus |
| 1 | CYP2E1 genotyping in renal cell/urothelial cancer patients in comparison with control populations. | Farker K et al. | 9760005 | Not a renal disease focus |
| 1 | Identification of fifteen novel mutations in the tissue-nonspecific alkaline phosphatase (TNSALP) gene in European patients with severe hypophosphatasia. | Mornet E et al. | 9781036 | Not a renal disease focus |
| 1 | Impact of CYP2E1 genotype in renal cell and urothelial cancer patients. | Farker K et al. | 9784018 | Not a renal disease focus |
| 1 | Inhibition of tissue angiotensin converting enzyme activity prevents malignant hypertension in TGR(mREN2)27. | Montgomery HE et al. | 9797175 | Not a renal disease focus |
| 1 | Analysis of 3p allelic losses in renal cell carcinomas: comparison with cytogenetic results. | Bernues M et al. | 9844606 | Not a renal disease focus |
| 1 | Renal changes on hyperglycemia and angiotensin-converting enzyme in type 1 diabetes. | Marre M et al. | 10082486 | Not a renal disease focus |
| 1 | Association of an insertion polymorphism of angiotensin-converting enzyme gene with the activity of lupus nephritis. | Akai Y et al. | 10099886 | Not a renal disease focus |

| | | | | |
|---|---|---|---|---|
| 1 | G-Protein beta3 subunit C825T variant and ambulatory blood pressure in essential hypertension. | Beige J et al. | 10205246 | Not a renal disease focus |
| 1 | Novel mutations in the 1alpha-hydroxylase (P450c1) gene in three families with pseudovitamin D-deficiency rickets resulting in loss of functional enzyme activity in blood-derived macrophages. | Smith SJ et al. | 10320521 | Not a renal disease focus |
| 1 | Gene structure, chromosomal location, and expression pattern of maleylacetoacetate isomerase. | Fernandez-Canon JM et al. | 10373324 | Not a renal disease focus |
| 1 | Candidate genetic modifiers of individual susceptibility to renal cell carcinoma: a study of polymorphic human xenobiotic-metabolizing enzymes. | Longuemaux S et al. | 10383153 | Not a renal disease focus |
| 1 | Absence of PTEN germ-line mutations in men with a potential inherited predisposition to prostate cancer. | Cooney KA et al. | 10389923 | Not a renal disease focus |
| 1 | Increased fragmentation of von Willebrand factor, due to abnormal cleavage of the subunit, parallels disease activity in recurrent hemolytic uremic syndrome and thrombotic thrombocytopenic purpura and discloses predisposition in families. The Italian Registry of Familial and Recurrent HUS/TTP. | Galbusera M et al. | 10397728 | Not a renal disease focus |
| 1 | Mutations of the VHL gene in sporadic renal cell carcinoma: definition of a risk factor for VHL patients to develop an RCC. | Gallou C et al. | 10408776 | Not a renal disease focus |
| 1 | Novel somatic mutations in the VHL gene in Swedish archived sporadic renal cell carcinomas. | Yang K et al. | 10454237 | Not a renal disease focus |
| 1 | The gene encoding hydroxypyruvate reductase (GRHPR) is mutated in patients with primary hyperoxaluria type II. | Cramer SD et al. | 10484776 | Not a renal disease focus |
| 1 | Carotid intima-media thickness and ACE-gene polymorphism in hemodialysis patients. | Nergizoglu G et al. | 10493570 | Not a renal disease focus |
| 1 | Telomerase activity and telomere lengths: alterations in renal cell carcinomas. | Dahse R et al. | 10504477 | Not a renal disease focus |
| 1 | Genotyping and functional analysis of a polymorphic (CCTTT)(n) repeat of NOS2A in diabetic retinopathy. | Warpeha KM et al. | 10506586 | Not a renal disease focus |
| 1 | Molecular basis of human salt sensitivity: the role of the 11beta-hydroxysteroid dehydrogenase type 2. | Lovati E et al. | 10523024 | Not a renal disease focus |

| | | | | |
|---|---|---|---|---|
| 1 | Genetic polymorphisms of the renin-angiotensin system and atheromatous renal artery stenosis. | Olivieri O et al. | 10567188 | Not a renal disease focus |
| 1 | Molecular characterization of the human PEA15 gene on 1q21-q22 and association with type 2 diabetes mellitus in Pima Indians. | Wolford JK et al. | 10607908 | Not a renal disease focus |
| 1 | Somatic mutation and homozygous deletion of PTEN/MMAC1 gene of 10q23 in renal cell carcinoma. | Alimov A et al. | 10628321 | Not a renal disease focus |
| 1 | No association between deletion-type angiotensin-converting enzyme gene polymorphism and left-ventricular hypertrophy in hemodialysis patients. | Yildiz A et al. | 10657713 | Not a renal disease focus |
| 1 | Analysis of point mutation in exon 2 of CYP2E1 gene in renal cell/urothelial cancer patients in comparison with control population. | Farker K et al. | 10667834 | Not a renal disease focus |
| 1 | Association between diabetic retinopathy and genetic variations in alpha2beta1 integrin, a platelet receptor for collagen. | Matsubara Y et al. | 10688808 | Not a renal disease focus |
| 1 | Lys(173)Arg and -344T/C variants of CYP11B2 in Japanese patients with low-renin hypertension. | Komiya I et al. | 10720581 | Not a renal disease focus |
| 1 | Synergistic effect of alpha-adducin and ACE genes causes blood pressure changes with body sodium and volume expansion. | Barlassina C et al. | 10720960 | Not a renal disease focus |
| 1 | Somatic mutations of the MET oncogene are selected during metastatic spread of human HNSC carcinomas. | Di Renzo MF et al. | 10734314 | Not a renal disease focus |
| 1 | Identification of fifteen novel PHEX gene mutations in Finnish patients with hypophosphatemic rickets. | Tyynismaa H et al. | 10737991 | Not a renal disease focus |
| 1 | Abnormal RNA expression of 11p15 imprinted genes and kidney developmental genes in Wilms' tumor. | Schwienbacher C et al. | 10749116 | Not a renal disease focus |
| 1 | VHL alterations in human clear cell renal cell carcinoma: association with advanced tumor stage and a novel hot spot mutation. | Brauch H et al. | 10766184 | Not a renal disease focus |
| 1 | An insertion/deletion polymorphism in intron 18 of the type B human natriuretic peptide receptor gene is not associated with cerebral infarction. | Rahmutula D et al. | 10770265 | Not a renal disease focus |
| 1 | Glutathione S-transferase M1, T1, and P1 polymorphisms as risk factors for renal cell carcinoma: a case-control study. | Sweeney C et al. | 10794492 | Not a renal disease focus |
| 1 | Familial clear cell renal cell carcinoma (FCRC): clinical features and mutation analysis of the VHL, MET, and CUL2 candidate genes. | Woodward ER et al. | 10807693 | Not a renal disease focus |

| | | | | |
|---|---|---|---|---|
| 1 | Trisomy 4 leading to duplication of a mutated KIT allele in acute myeloid leukemia with mast cell involvement. | Beghini A et al. | 10812167 | Not a renal disease focus |
| 1 | Distribution of different HLA antigens in Greek hypertensives according to the angiotensin-converting enzyme genotype. | Diamantopoulos EJ et al. | 10821349 | Not a renal disease focus |
| 1 | Angiotensin-converting enzyme gene I/D polymorphism in malignant hypertension. | Stefansson B et al. | 10855732 | Not a renal disease focus |
| 1 | An intron 4 gene polymorphism in endothelial cell nitric oxide synthase might modulate volume-dependent hypertension in patients on hemodialysis. | Yokoyama K et al. | 10867538 | Not a renal disease focus |
| 1 | Elevated urinary albumin excretion is not linked to the angiotensin I-converting enzyme gene polymorphism in clinically healthy subjects. | Clausen P et al. | 10872702 | Not a renal disease focus |
| 1 | Functional analyses of amino acid substitutions Arg883Ser and Asp905Tyr of protein phosphatase-1 G-subunit. | Permana PA et al. | 10873397 | Not a renal disease focus |
| 1 | Functional synergism between the most common polymorphism in human alanine:glyoxylate aminotransferase and four of the most common disease-causing mutations. | Lumb MJ et al. | 10960483 | Not a renal disease focus |
| 1 | G-protein beta(3)-subunit C825T genotype and nephropathy in diabetes mellitus. | Beige J et al. | 10978395 | Not a renal disease focus |
| 1 | Alterations of the DNA repair gene OGG1 in human clear cell carcinomas of the kidney. | Audebert M et al. | 10987279 | Not a renal disease focus |
| 1 | Missense mutation of the MET gene detected in human glioma. | Moon YW et al. | 11007037 | Not a renal disease focus |
| 1 | Renal outcome and vascular morbidity in systemic lupus erythematosus (SLE): lack of association with the angiotensin-converting enzyme gene polymorphism. | Molad Y et al. | 11071585 | Not a renal disease focus |
| 1 | Serum arylesterase/diazoxonase activity and genetic polymorphisms in patients with type 2 diabetes. | Inoue M et al. | 11092501 | Not a renal disease focus |
| 1 | Detection of the association between a deletion polymorphism in the gene encoding angiotensin I-converting enzyme and advanced diabetic retinopathy. | Matsumoto A et al. | 11106834 | Not a renal disease focus |
| 1 | Proteinase 3 gene polymorphisms and Wegener's granulomatosis. | Gencik M et al. | 11115080 | Not a renal disease focus |

| | | | | |
|---|---|---|---|---|
| 1 | alpha-adducin and angiotensin I-converting enzyme polymorphisms in essential hypertension. | Clark CJ et al. | 11116113 | Not a renal disease focus |
| 1 | Alleviating transcript insufficiency caused by Friedreich's ataxia triplet repeats. | Grabczyk E et al. | 11121484 | Not a renal disease focus |
| 1 | Glutathione transferase activities in renal carcinomas and adjacent normal renal tissues: factors influencing renal carcinogenesis induced by xenobiotics. | Delbanco EH et al. | 11218045 | Not a renal disease focus |
| 1 | Random mutagenesis-PCR to introduce alterations into defined DNA sequences for validation of SNP and mutation detection methods. | Nickerson ML et al. | 11241843 | Not a renal disease focus |
| 1 | Gain-of-function mutation at the extracellular domain of KIT in gastrointestinal stromal tumours. | Hirota S et al. | 11276010 | Not a renal disease focus |
| 1 | Gene-environment interactions in renal cell carcinoma. | Semenza JC et al. | 11323315 | Not a renal disease focus |
| 1 | von Hippel-Lindau protein mutants linked to type 2C VHL disease preserve the ability to downregulate HIF. | Hoffman MA et al. | 11331612 | Not a renal disease focus |
| 1 | Association of angiotensinogen M235T and A(-6)G gene polymorphisms with coronary heart disease with independence of essential hypertension: the PROCAGENE study. Prospective Cardiac Gene. | Rodriquez-Perez JC et al. | 11345362 | Not a renal disease focus |
| 1 | Cholesteryl ester transfer protein polymorphism associated with macroangiopathy in Japanese patients with type 2 diabetes. | Meguro S et al. | 11369008 | Not a renal disease focus |
| 1 | Mixed epithelial and stromal tumor of the kidney lacks the genetic alterations of cellular congenital mesoblastic nephroma. | Pierson CR et al. | 11381370 | Not a renal disease focus |
| 1 | Increased D allele frequency of the angiotensin-converting enzyme gene in pulmonary fibrosis. | Morrison CD et al. | 11381371 | Not a renal disease focus |
| 1 | Class II HLA associations with autoantibodies in scleroderma: a highly significant role for HLA-DP. | Gilchrist FC et al. | 11393660 | Not a renal disease focus |
| 1 | IRAK1b, a novel alternative splice variant of interleukin-1 receptor-associated kinase (IRAK), mediates interleukin-1 signaling and has prolonged stability. | Jensen LE et al. | 11397809 | Not a renal disease focus |
| 1 | Cancer risk in heterozygotes for ataxia-telangiectasia. | Geoffroy-Perez B et al. | 11410879 | Not a renal disease focus |
| 1 | VHL c.505 T>C mutation confers a high age related penetrance but no increased overall mortality. | Bender BU et al. | 11483638 | Not a renal disease focus |

| | | | | |
|---|---|---|---|---|
| 1 | Angiotensin-converting enzyme gene polymorphism in patients with systemic lupus. | Prkacin I et al. | 11505631 | Not a renal disease focus |
| 1 | Polymorphism screening of the insulin receptor-related receptor gene (INSRR) on 1q in Pima Indians. | Wolford JK et al. | 11513557 | Not a renal disease focus |
| 1 | The pVHL-associated SCF ubiquitin ligase complex: molecular genetic analysis of elongin B and C, Rbx1 and HIF-1alpha in renal cell carcinoma. | Clifford SC et al. | 11526493 | Not a renal disease focus |
| 1 | Meningioma: a cytogenetic model of a complex benign human tumor, including data on 394 karyotyped cases. | Zang KD | 11528114 | Not a renal disease focus |
| 1 | Epigenetic inactivation of the RASSF1A 3p21.3 tumor suppressor gene in both clear cell and papillary renal cell carcinoma. | Morrissey C et al. | 11585766 | Not a renal disease focus |
| 1 | Crystal structures of NK1-heparin complexes reveal the basis for NK1 activity and enable engineering of potent agonists of the MET receptor. | Lietha D et al. | 11597998 | Not a renal disease focus |
| 1 | Detection of AGXT bgene mutations by denaturing high-performance liquid chromatography for diagnosis of hyperoxaluria type 1. | Pirulli D et al. | 11699734 | Not a renal disease focus |
| 1 | Identification of six novel MYH9 mutations and genotype-phenotype relationships in autosomal dominant macrothrombocytopenia with leukocyte inclusions. | Kunishima S et al. | 11776386 | Not a renal disease focus |
| 1 | Biallelic inactivation of the von Hippel-Lindau tumor suppressor gene in sporadic renal cell carcinoma. | Hamano K et al. | 11792959 | Not a renal disease focus |
| 1 | Association of the D allele of the angiotensin I converting enzyme polymorphism with malignant vascular injury. | Mayer NJ et al. | 11836444 | Not a renal disease focus |
| 1 | Comprehensive mutational analysis of the VHL gene in sporadic renal cell carcinoma: relationship to clinicopathological parameters. | Kondo K et al. | 11921283 | Not a renal disease focus |
| 1 | Mutations in the von Hippel-Lindau (VHL) gene refine differential diagnostic criteria in renal cell carcinoma. | Barnabas N et al. | 11967908 | Not a renal disease focus |
| 1 | Enhanced activity of variant phospholipase C-delta1 protein (R257H) detected in patients with coronary artery spasm. | Nakano T et al. | 11980680 | Not a renal disease focus |
| 1 | Inhibition of HIF is necessary for tumor suppression by the von Hippel-Lindau protein. | Kondo K et al. | 12086860 | Not a renal disease focus |

| | | | | |
|---|---|---|---|---|
| 1 | Identification of cyclin D1 and other novel targets for the von Hippel-Lindau tumor suppressor gene by expression array analysis and investigation of cyclin D1 genotype as a modifier in von Hippel-Lindau disease. | Zatyka M et al. | 12097293 | Not a renal disease focus |
| 1 | ACE and PC-1 gene polymorphisms in normoalbuminuric Type 1 diabetic patients: a 10-year prospective study. | de Azevedo MJ et al. | 12126783 | Not a renal disease focus |
| 1 | Microsatellite instability and immunostaining for MSH-2 and MLH-1 in cutaneous and internal tumors from patients with the Muir-Torre syndrome. | Machin P et al. | 12139636 | Not a renal disease focus |
| 1 | No association between a genetic variant of the p22(phox) component of NAD(P)H oxidase and the incidence and progression of IgA nephropathy. | Wolf G et al. | 12147803 | Not a renal disease focus |
| 1 | Analysis of MGEA5 on 10q24.1-q24.3 encoding the beta-O-linked N-acetylglucosaminidase as a candidate gene for type 2 diabetes mellitus in Pima Indians. | Farook VS et al. | 12359146 | Not a renal disease focus |
| 1 | Distinct patterns of chromosomal losses in clinically synchronous and asynchronous bilateral renal cell carcinoma. | Kito H et al. | 12442000 | Not a renal disease focus |
| 1 | The relationship among the polymorphisms of SULT1A1, 1A2 and different types of cancers in Taiwanese. | Peng CT et al. | 12469224 | Not a renal disease focus |
| 1 | Variant screening of PRKAB2, a type 2 diabetes mellitus susceptibility candidate gene on 1q in Pima Indians. | Prochazka M et al. | 12490143 | Not a renal disease focus |
| 1 | Genetics, clinical and pathological features of glomerulonephritis associated with mutations of nonmuscle myosin IIA (Fechtner syndrome). | Ghiggeri GM et al. | 12500226 | Not a renal disease focus |
| 1 | Mutations in PRKCSH cause isolated autosomal dominant polycystic liver disease. | Li A et al. | 12529853 | Not a renal disease focus |
| 1 | Impact of clarithromycin resistance and CYP2C19 genetic polymorphism on treatment efficacy of Helicobacter pylori infection with lansoprazole- or rabeprazole-based triple therapy in Japan. | Miki I et al. | 12544691 | Not a renal disease focus |
| 1 | The inhibitory gamma subunit of the type 6 retinal cGMP phosphodiesterase functions to link c-Src and G-protein-coupled receptor kinase 2 in a signaling unit that regulates p42/p44 mitogen-activated protein kinase by epidermal growth factor. | Wan KF et al. | 12624098 | Not a renal disease focus |

| | | | | |
|---|---|---|---|---|
| 1 | Serum extracellular superoxide dismutase in patients with type 2 diabetes: relationship to the development of micro- and macrovascular complications. | Kimura F et al. | 12663605 | Not a renal disease focus |
| 1 | Vascular endothelial growth factor gene polymorphism is associated with calcium oxalate stone disease. | Chen WC et al. | 12719950 | Not a renal disease focus |
| 1 | Proinflammatory genotype of interleukin-1 and interleukin-1 receptor antagonist is associated with ESRD in proteinase 3-ANCA vasculitis patients. | Borgmann S et al. | 12722027 | Not a renal disease focus |
| 1 | Mutation analysis and clinical implications of von Willebrand factor-cleaving protease deficiency. | Assink K et al. | 12753286 | Not a renal disease focus |
| 1 | Association of homozygous deletion of the Humhv3005 and the VH3-30.3 genes with renal involvement in systemic lupus erythematosus. | Cho ML et al. | 12765304 | Not a renal disease focus |
| 1 | A biallelic gene polymorphism of CYP11B2 predicts increased aldosterone to renin ratio in selected hypertensive patients. | Nicod J et al. | 12788845 | Not a renal disease focus |
| 1 | MYH9-related disease: May-Hegglin anomaly, Sebastian syndrome, Fechtner syndrome, and Epstein syndrome are not distinct entities but represent a variable expression of a single illness. | Seri M et al. | 12792306 | Not a renal disease focus |
| 1 | A single nucleotide polymorphism in the matrix metalloproteinase-1 promoter is associated with conventional renal cell carcinoma. | Hirata H et al. | 12845675 | Not a renal disease focus |
| 1 | Aberrant methylation and silencing of ARHI, an imprinted tumor suppressor gene in which the function is lost in breast cancers. | Yuan J et al. | 12874023 | Not a renal disease focus |
| 1 | Oxidative stress-related factors in Bartter's and Gitelman's syndromes: relevance for angiotensin II signalling. | Calo LA et al. | 12897089 | Not a renal disease focus |
| 1 | Frequent allelic changes at chromosome 7q34 but lack of mutation of the BRAF in papillary renal cell tumors. | Nagy A et al. | 12918080 | Not a renal disease focus |
| 1 | Relevance of nuclear and cytoplasmic von hippel lindau protein expression for renal carcinoma progression. | Schraml P et al. | 12937142 | Not a renal disease focus |
| 1 | GBPI, a novel gastrointestinal- and brain-specific PP1-inhibitory protein, is activated by PKC and inactivated by PKA. | Liu QR et al. | 12974676 | Not a renal disease focus |
| 1 | Renin-angiotensin system gene polymorphisms: assessment of the risk of coronary heart disease. | Buraczynska M et al. | 14502296 | Not a renal disease focus |
| 1 | Glutathione S-transferases M1-1 and T1-1 as risk modifiers for renal cell cancer associated with occupational exposure to chemicals. | Buzio L et al. | 14504370 | Not a renal disease focus |

| | | | | |
|---|---|---|---|---|
| 1 | Expression of the proto-oncogene Axl in renal cell carcinoma. | Chung BI et al. | 14565870 | Not a renal disease focus |
| 1 | Paraoxonase (Pon1) Q192R polymorphism and serum Pon1 activity in diabetic patients on maintenance hemodialysis. | Zhang B et al. | 14579940 | Not a renal disease focus |
| 1 | Endothelial nitric oxide synthase gene intron 4 polymorphism in type 2 diabetes mellitus. | Ksiazek P et al. | 14580231 | Not a renal disease focus |
| 1 | Cytochrome P450 and manganese superoxide dismutase genes polymorphisms in systemic lupus erythematosus. | Yen JH et al. | 14611903 | Not a renal disease focus |
| 1 | Peripheral vascular disease in Type 2 diabetic Chinese patients: associations with metabolic indices, concomitant vascular disease and genetic factors. | Thomas GN et al. | 14632699 | Not a renal disease focus |
| 1 | The rapid effects of 1,25-dihydroxyvitamin D3 require the vitamin D receptor and influence 24-hydroxylase activity: studies in human skin fibroblasts bearing vitamin D receptor mutations. | Nguyen TM et al. | 14665637 | Not a renal disease focus |
| 1 | Renovascular disease: effect of ACE gene deletion polymorphism and endovascular revascularization. | Pizzolo F et al. | 14718831 | Not a renal disease focus |
| 1 | Genetic characterization and structural analysis of VHL Spanish families to define genotype-phenotype correlations. | Ruiz-Llorente S et al. | 14722919 | Not a renal disease focus |
| 1 | Congenital disorder of oxygen sensing: association of the homozygous Chuvash polycythemia VHL mutation with thrombosis and vascular abnormalities but not tumors. | Gordeuk VR et al. | 14726398 | Not a renal disease focus |
| 1 | Defects in translational regulation mediated by the alpha subunit of eukaryotic initiation factor 2 inhibit antiviral activity and facilitate the malignant transformation of human fibroblasts. | Perkins DJ et al. | 14966282 | Not a renal disease focus |
| 1 | Polymorphisms of the CYP1B1 gene as risk factors for human renal cell cancer. | Sasaki M et al. | 15041720 | Not a renal disease focus |
| 1 | Abnormal hepatocystin caused by truncating PRKCSH mutations leads to autosomal dominant polycystic liver disease. | Drenth JP et al. | 15057895 | Not a renal disease focus |
| 1 | KIT expression in chromophobe renal cell carcinoma: comparative immunohistochemical analysis of KIT expression in different renal cell neoplasms. | Petit A et al. | 15105658 | Not a renal disease focus |

| | | | | |
|---|---|---|---|---|
| 1 | Association of the serum and glucocorticoid regulated kinase (sgk1) gene with QT interval. | Busjahn A et al. | 15107590 | Not a renal disease focus |
| 1 | Three novel missense mutations of WNK4, a kinase mutated in inherited hypertension, in Japanese hypertensives: implication of clinical phenotypes. | Kamide K et al. | 15110905 | Not a renal disease focus |
| 1 | Intron 4 polymorphism of the endothelial nitric oxide synthase gene is associated with the development of lupus nephritis. | Lee YH et al. | 15119548 | Not a renal disease focus |
| 1 | Identification of PIK3C3 promoter variant associated with bipolar disorder and schizophrenia. | Stopkova P et al. | 15121481 | Not a renal disease focus |
| 1 | The role of the LRPPRC (leucine-rich pentatricopeptide repeat cassette) gene in cytochrome oxidase assembly: mutation causes lowered levels of COX (cytochrome c oxidase) I and COX III mRNA. | Xu F et al. | 15139850 | Not a renal disease focus |
| 1 | A naturally occurring human Nedd4-2 variant displays impaired ENaC regulation in Xenopus laevis oocytes. | Fouladkou F et al. | 15140763 | Not a renal disease focus |
| 1 | Increased amount of the angiotensin-converting enzyme (ACE) mRNA originating from the ACE allele with deletion. | Suehiro T et al. | 15164285 | Not a renal disease focus |
| 1 | G protein beta3 subunit C825T polymorphism in primary IgA nephropathy. | Thibaudin L et al. | 15200440 | Not a renal disease focus |
| 1 | A novel missense substitution (Val1483Ile) in the fatty acid synthase gene (FAS) is associated with percentage of body fat and substrate oxidation rates in nondiabetic Pima Indians. | Kovacs P et al. | 15220220 | Not a renal disease focus |
| 1 | Functional analysis of polymorphisms in the promoter regions of genes on 22q11. | Hoogendoorn B et al. | 15221787 | Not a renal disease focus |
| 1 | Clinical implications of mutation analysis in primary hyperoxaluria type 1. | van Woerden CS et al. | 15253729 | Not a renal disease focus |
| 1 | Paraoxonase 1 Gln/Arg polymorphism is associated with the risk of microangiopathy in Type 2 diabetes mellitus. | Murata M et al. | 15270786 | Not a renal disease focus |
| 1 | Identification of 108 SNPs in TSC, WNK1, and WNK4 and their association with hypertension in a Japanese general population. | Kokubo Y et al. | 15309683 | Not a renal disease focus |
| 1 | Association of a haplotype of matrix metalloproteinase (MMP)-1 and MMP-3 polymorphisms with renal cell carcinoma. | Hirata H et al. | 15319295 | Not a renal disease focus |
| 1 | Genetic risk of atherosclerotic renal artery disease: the candidate gene approach in a renal angiography cohort. | van Onna M et al. | 15326089 | Not a renal disease focus |

| | | | | |
|---|---|---|---|---|
| 1 | Evaluation of mutation screening as a first line test for the diagnosis of the primary hyperoxalurias. | Rumsby G et al. | 15327387 | Not a renal disease focus |
| 1 | G-protein beta3 subunit gene C825T polymorphism in patients with vesico-ureteric reflux. | Zagradisnik B et al. | 15337465 | Not a renal disease focus |
| 1 | An intronic variant of the TGFBR1 gene is associated with carcinomas of the kidney and bladder. | Chen T et al. | 15382067 | Not a renal disease focus |
| 1 | Polymorphisms in the 5'-upstream region of the PKCbeta gene in Japanese patients with Type 2 diabetes. | Ikeda Y et al. | 15384959 | Not a renal disease focus |
| 1 | Glutathione S-transferase T1 deletion is a risk factor for developing end-stage renal disease in diabetic patients. | Yang Y et al. | 15492856 | Not a renal disease focus |
| 1 | CHEK2 is a multiorgan cancer susceptibility gene. | Cybulski C et al. | 15492928 | Not a renal disease focus |
| 1 | Molecular basis of hypoxanthine-guanine phosphoribosyltransferase deficiency in Italian Lesch-Nyhan patients: identification of nine novel mutations. | Bertelli M et al. | 15505382 | Not a renal disease focus |
| 1 | Distinct patterns of abnormal GNAS imprinting in familial and sporadic pseudohypoparathyroidism type IB. | Liu J et al. | 15537666 | Not a renal disease focus |
| 1 | Chromophobe renal cell carcinoma: a comparative study of histological, immunohistochemical and ultrastructural features using high throughput tissue microarray. | Abrahams NA et al. | 15569050 | Not a renal disease focus |
| 1 | Association of a functional single-nucleotide polymorphism of PTPN22, encoding lymphoid protein phosphatase, with rheumatoid arthritis and systemic lupus erythematosus. | Orozco G et al. | 15641066 | Not a renal disease focus |
| 1 | Altered gene expression in phenotypically normal renal cells from carriers of tumor suppressor gene mutations. | Stoyanova R et al. | 15662135 | Not a renal disease focus |
| 1 | Thrombin activatable fibrinolysis inhibitor in BehÃ§et's disease. | Donmez A et al. | 15668188 | Not a renal disease focus |
| 1 | Allelic variants of the human scavenger receptor class B type 1 and paraoxonase 1 on coronary heart disease: genotype-phenotype correlations. | Rodriquez-Esparragon F et al. | 15681296 | Not a renal disease focus |
| 1 | A novel recessive mutation in fibroblast growth factor-23 causes familial tumoral calcinosis. | Larsson T et al. | 15687325 | Not a renal disease focus |

| | | | | |
|---|---|---|---|---|
| 1 | DNase II polymorphisms associated with risk of renal disorder among systemic lupus erythematosus patients. | Shin HD et al. | 15723160 | Not a renal disease focus |
| 1 | Analysis of the apolipoprotein(a) size polymorphism in patients with systemic lupus erythematosus. | Peros E et al. | 15754029 | Not a renal disease focus |
| 1 | Relationship of eNOS gene variants to diseases that have in common an endothelial cell dysfunction. | Heltianu C et al. | 15784171 | Not a renal disease focus |
| 1 | Variable number of tandem repeat of the 5'-flanking region of type-C human natriuretic peptide receptor gene influences blood pressure levels in obesity-associated hypertension. | Aoi N et al. | 15785005 | Not a renal disease focus |
| 1 | Renin-angiotensin system gene polymorphisms predict the progression to renal insufficiency among Asians with lupus nephritis. | Parsa A et al. | 15789057 | Not a renal disease focus |
| 1 | A novel STX16 deletion in autosomal dominant pseudohypoparathyroidism type Ib redefines the boundaries of a cis-acting imprinting control element of GNAS. | Linglart A et al. | 15800843 | Not a renal disease focus |
| 1 | Overexpression of human alanine:glyoxylate aminotransferase in Escherichia coli: renaturation from guanidine-HCl and affinity for pyridoxal phosphate co-factor. | Coulter-Mackie MB et al. | 15802217 | Not a renal disease focus |
| 1 | Association between plasma activities of semicarbazide-sensitive amine oxidase and angiotensin-converting enzyme in patients with type 1 diabetes mellitus. | Boomsma F et al. | 15830186 | Not a renal disease focus |
| 1 | Relationship of bradykinin B2 receptor gene polymorphism with essential hypertension and left ventricular hypertrophy. | Fu Y et al. | 15894833 | Not a renal disease focus |
| 1 | Genetic variation in the bleomycin hydrolase gene and bleomycin-induced pulmonary toxicity in germ cell cancer patients. | Nuver J et al. | 15900213 | Not a renal disease focus |
| 1 | Prevalence of von Hippel-Lindau gene mutations in sporadic renal cell carcinoma: results from The Netherlands cohort study. | van Houwelingen KP et al. | 15932632 | Not a renal disease focus |
| 1 | Inducible nitric oxide synthase polymorphism is associated with susceptibility to Henoch-SchÃ¶nlein purpura in northwestern Spain. | Martin J et al. | 15940772 | Not a renal disease focus |
| 1 | Association between CYP2C9 slow metabolizer genotypes and severe hypoglycaemia on medication with sulphonylurea hypoglycaemic agents. | Holstein A et al. | 15963101 | Not a renal disease focus |

| | | | | |
|---|---|---|---|---|
| 1 | The major allele of the alanine:glyoxylate aminotransferase gene: nine novel mutations and polymorphisms associated with primary hyperoxaluria type 1. | Coulter-Mackie MB et al. | 15963748 | Not a renal disease focus |
| 1 | Angiotensin-converting enzyme (ACE) haplotypes and cyclosporine A (CsA) response: a model of the complex relationship between ACE quantitative trait locus and pathological phenotypes. | Catarsi P et al. | 16002416 | Not a renal disease focus |
| 1 | Increased frequency of the angiotensin-converting enzyme gene D-allele is associated with noninfectious pulmonary dysfunction following allogeneic stem cell transplant. | Onizuka M et al. | 16044138 | Not a renal disease focus |
| 1 | Association between polymorphisms of the renin-angiotensin system and more severe histological forms of lupus nephritis. | Sprovieri SR et al. | 16047641 | Not a renal disease focus |
| 1 | The R620W C/T polymorphism of the gene PTPN22 is associated with SLE independently of the association of PDCD1. | Reddy MV et al. | 16052172 | Not a renal disease focus |
| 1 | Does complement factor B have a role in the pathogenesis of atypical HUS? | Kavanagh D et al. | 16061287 | Not a renal disease focus |
| 1 | Association of NEDD4L ubiquitin ligase with essential hypertension. | Russo CJ et al. | 16103266 | Not a renal disease focus |
| 1 | CYP3A5 genotype is associated with elevated blood pressure. | Fromm MF et al. | 16141800 | Not a renal disease focus |
| 1 | Complement factor B allotypes in the susceptibility and severity of coeliac disease in patients and relatives. | da Rosa Utiyama SR et al. | 16164698 | Not a renal disease focus |
| 1 | The Prevalence of CYP2C8, 2C9, 2J2, and soluble epoxide hydrolase polymorphisms in African Americans with hypertension. | Dreisbach AW et al. | 16202848 | Not a renal disease focus |
| 1 | Natural history of Fabry disease in females in the Fabry Outcome Survey. | Deegan PB et al. | 16227523 | Not a renal disease focus |
| 1 | ADAM33: a newly identified gene in the pathogenesis of asthma. | Holgate ST et al. | 16257631 | Not a renal disease focus |
| 1 | Alternative splicing of fibroblast growth factor receptor 3 produces a secreted isoform that inhibits fibroblast growth factor-induced proliferation and is repressed in urothelial carcinoma cell lines. | Tomlinson DC et al. | 16288035 | Not a renal disease focus |
| 1 | Homozygous and compound heterozygous mutations in ZMPSTE24 cause the laminopathy restrictive dermopathy. | Moulson CL et al. | 16297189 | Not a renal disease focus |

| | | | | |
|---|---|---|---|---|
| 1 | The PTPN22 620W allele is a risk factor for Wegener's granulomatosis. | Jagiello P et al. | 16320352 | Not a renal disease focus |
| 1 | Hypoxia-regulated expression of attenuated diphtheria toxin A fused with hypoxia-inducible factor-1alpha oxygen-dependent degradation domain preferentially induces apoptosis of hypoxic cells in solid tumor. | Koshikawa N et al. | 16357173 | Not a renal disease focus |
| 1 | Is factor V Leiden a risk factor for thrombotic microangiopathies without severe ADAMTS 13 deficiency? | Krieg S et al. | 16411392 | Not a renal disease focus |
| 1 | Int7G24A variant of transforming growth factor-beta receptor type I is associated with invasive breast cancer. | Chen T et al. | 16428477 | Not a renal disease focus |
| 1 | Antiepidermal growth factor variant III scFv fragment: effect of radioiodination method on tumor targeting and normal tissue clearance. | Shankar S et al. | 16459265 | Not a renal disease focus |
| 1 | Matrix metalloproteinase-9 polymorphisms and renal cell carcinoma in a Japanese population. | Awakura Y et al. | 16466849 | Not a renal disease focus |
| 1 | Association of CYP3A5 genotypes with blood pressure and renal function in African families. | Bochud M et al. | 16612255 | Not a renal disease focus |
| 1 | Relationship of serum paraoxonase 1 activity and paraoxonase 1 genotype to risk of systemic lupus erythematosus. | Tripi LM et al. | 16729301 | Not a renal disease focus |
| 1 | CCL18: a urinary marker of Gaucher cell burden in Gaucher patients. | Boot RG et al. | 16736095 | Not a renal disease focus |
| 1 | Adult leydig cell tumors of the testis caused by germline fumarate hydratase mutations. | Carvajal-Carmona LG et al. | 16757530 | Not a renal disease focus |
| 1 | Are the angiotensin-converting enzyme gene and activity risk factors for stroke? | Dikmen M et al. | 16791358 | Not a renal disease focus |
| 1 | Increased neutrophil membrane expression and plasma level of proteinase 3 in systemic vasculitis are not a consequence of the - 564 A/G promotor polymorphism. | Abdgawad M et al. | 16792675 | Not a renal disease focus |
| 1 | Slight association between type 1 diabetes and "ff" VDR FokI genotype in patients from the Italian Lazio Region. Lack of association with diabetes complications. | Capoluongo E et al. | 16806146 | Not a renal disease focus |

| | | | | |
|---|---|---|---|---|
| 1 | No association of the CYP3A5*1 allele with blood pressure and left ventricular mass and geometry: the KORA/MONICA Augsburg echocardiographic substudy. | Lieb W et al. | 16822233 | Not a renal disease focus |
| 1 | Autosomal-dominant pseudohypoparathyroidism type Ib is caused by different microdeletions within or upstream of the GNAS locus. | Juppner H et al. | 16831926 | Not a renal disease focus |
| 1 | VHL P25L is not a pathogenic von Hippel-Lindau mutation: a family study. | Pettman RK et al. | 16884327 | Not a renal disease focus |
| 1 | ACE gene insertion/deletion polymorphism modulates capillary permeability in hypertension. | Dell'omo G et al. | 16889537 | Not a renal disease focus |
| 1 | Allelic variation in the CNDP1 gene and its lack of association with longevity and coronary heart disease. | Zschocke J et al. | 16965804 | Not a renal disease focus |
| 1 | Ubiquitin ligase gp78 increases solubility and facilitates degradation of the Z variant of alpha-1-antitrypsin. | Shen Y et al. | 16979136 | Not a renal disease focus |
| 1 | NFkappaB and its inhibitor IkappaB in relation to type 2 diabetes and its microvascular and atherosclerotic complications. | Romzova M et al. | 17002901 | Not a renal disease focus |
| 1 | Predicting the impact of population level risk reduction in cardio-vascular disease and stroke on acute hospital admission rates over a 5 year period--a pilot study. | Whitfield MD et al. | 17084425 | Not a renal disease focus |
| 1 | Increased expression of angiotensin II type 1 receptor (AGTR1) in heart transplant recipients with recurrent rejection. | Yamani MH et al. | 17097490 | Not a renal disease focus |
| 1 | Effect of synthetic corticosteroids on vascular reactivity in the human forearm. | Mangos GJ et al. | 17132537 | Not a renal disease focus |
| 1 | Association of genetic polymorphisms of ACADSB and COMT with human hypertension. | Kamide K et al. | 17143180 | Not a renal disease focus |
| 1 | Role of the STK15 Phe31Ile polymorphism in renal cell carcinoma. | Hammerschmied CG et al. | 17143471 | Not a renal disease focus |
| 1 | Genetic and epigenetic analysis of CHEK2 in sporadic breast, colon, and ovarian cancers. | Williams LH et al. | 17145815 | Not a renal disease focus |
| 1 | Blastemal expression of type I insulin-like growth factor receptor in Wilms' tumors is driven by increased copy number and correlates with relapse. | Natrajan R et al. | 17145858 | Not a renal disease focus |

| | | | | |
|---|---|---|---|---|
| 1 | Identification of a novel BBS gene (BBS12) highlights the major role of a vertebrate-specific branch of chaperonin-related proteins in Bardet-Biedl syndrome. | Stoetzel C et al. | 17160889 | Not a renal disease focus |
| 1 | Association of the C825T polymorphism of the G-protein beta3 subunit gene with hypertension, obesity, hyperlipidemia, insulin resistance, diabetes, diabetic complications, and diabetic therapies among Japanese. | Hayakawa T et al. | 17161225 | Not a renal disease focus |
| 1 | Adrenal hyperplasia and adenomas are associated with inhibition of phosphodiesterase 11A in carriers of PDE11A sequence variants that are frequent in the population. | Horvath A et al. | 17178847 | Not a renal disease focus |
| 1 | Mucinous tubular and spindle cell carcinoma of kidney is probably a variant of papillary renal cell carcinoma with spindle cell features. | Shen SS et al. | 17240302 | Not a renal disease focus |
| 1 | EGFR sequence variations and real-time quantitative polymerase chain reaction analysis of gene dosage in brain metastases of solid tumors. | Franco-Hernandez C et al. | 17284372 | Not a renal disease focus |
| 1 | Nosocomial outbreak of CTX-M-15-producing E. coli in Norway. | Naseer U et al. | 17295678 | Not a renal disease focus |
| 1 | Association of genotypes of thrombin-activatable fibrinolysis inhibitors with thrombotic microangiopathies--a pilot study. | Sucker C et al. | 17327284 | Not a renal disease focus |
| 1 | Functional polymorphism in human CYP4F2 decreases 20-HETE production. | Stec DE et al. | 17341693 | Not a renal disease focus |
| 1 | Immunoexpression of lactoferrin in human sporadic renal cell carcinomas. | Giuffre G et al. | 17390038 | Not a renal disease focus |
| 1 | Association of ATP1A1 and dear single-nucleotide polymorphism haplotypes with essential hypertension: sex-specific and haplotype-specific effects. | Glorioso N et al. | 17446437 | Not a renal disease focus |
| 1 | Comprehensive mutation screening in 55 probands with type 1 primary hyperoxaluria shows feasibility of a gene-based diagnosis. | Monico CG et al. | 17460142 | Not a renal disease focus |
| 1 | Mutations in the SBDS gene in acquired aplastic anemia. | Calado RT et al. | 17478638 | Not a renal disease focus |
| 1 | Re-assessment of the influence of polymorphisms of phase-II metabolic enzymes on renal cell cancer risk of trichloroethylene-exposed workers. | Wiesenhutter B et al. | 17479278 | Not a renal disease focus |

| | | | | |
|---|---|---|---|---|
| 1 | Uncommon CHEK2 mis-sense variant and reduced risk of tobacco-related cancers: case control study. | Brennan P et al. | 17517688 | Not a renal disease focus |
| 1 | Impact of maternal angiotensinogen M235T polymorphism and angiotensin-converting enzyme insertion/deletion polymorphism on blood pressure, protein excretion and fetal outcome in pregnancy. | Pfab T et al. | 17563539 | Not a renal disease focus |
| 1 | Lack of association between endothelial nitric oxide synthase gene polymorphisms, microalbuminuria and endothelial dysfunction in hypertensive men. | Dell'Omo G et al. | 17563560 | Not a renal disease focus |
| 1 | Circulating protein biomarkers of pharmacodynamic activity of sunitinib in patients with metastatic renal cell carcinoma: modulation of VEGF and VEGF-related proteins. | Deprimo SE et al. | 17605814 | Not a renal disease focus |
| 1 | Glutathione S-transferase polymorphisms, cruciferous vegetable intake and cancer risk in the Central and Eastern European Kidney Cancer Study. | Moore LE et al. | 17617661 | Not a renal disease focus |
| 1 | Effects of angiotensin-converting enzyme gene polymorphism and serum vitamin D levels on ambulatory blood pressure measurement and left ventricular mass in Turkish hypertensive population. | Kulah E et al. | 17625392 | Not a renal disease focus |
| 1 | Manganese superoxide dismutase (Mn-SOD) gene polymorphisms in urolithiasis. | Tugcu V et al. | 17628794 | Not a renal disease focus |
| 1 | Angiotensin-converting enzyme gene polymorphism in Kuwaiti patients with systemic lupus erythematosus. | Al-Awadhi AM et al. | 17631741 | Not a renal disease focus |
| 1 | MDM2 SNP309 polymorphism as risk factor for susceptibility and poor prognosis in renal cell carcinoma. | Hirata H et al. | 17634539 | Not a renal disease focus |
| 1 | Protein expression and mutational analysis of epidermal growth factor receptor in renal angiomyolipomas. | Lim SD et al. | 17685929 | Not a renal disease focus |
| 1 | Germline VHL gene mutations in three Serbian families with von Hippel-Lindau disease. | Stanojevic BR et al. | 17688370 | Not a renal disease focus |
| 1 | The E3 ligase HACE1 is a critical chromosome 6q21 tumor suppressor involved in multiple cancers. | Zhang L et al. | 17694067 | Not a renal disease focus |
| 1 | Genetic variation in the paraoxonase-3 (PON3) gene is associated with serum PON1 activity. | Sanghera DK et al. | 17900266 | Not a renal disease focus |
| 1 | Genetic polymorphisms in OGG1 and their association with angiomyolipoma, a benign kidney tumor in patients with tuberous sclerosis. | Habib SL et al. | 17932460 | Not a renal disease focus |

| | | | | |
|---|---|---|---|---|
| 1 | Human G(salpha) mutant causes pseudohypoparathyroidism type Ia/neonatal diarrhea, a potential cell-specific role of the palmitoylation cycle. | Makita N et al. | 17962410 | Not a renal disease focus |
| 1 | Association of the nitric oxide synthase (eNOS) gene polymorphism with increased risk for both lupus glomerulonephritis and rheumatoid arthritis in a single genetically homogeneous population. | Vazgiourakis V et al. | 17971359 | Not a renal disease focus |
| 1 | Lack of association between matrix metalloproteinase-1 (MMP-1) promoter polymorphism and risk of renal cell carcinoma. | Piccoli MF et al. | 17980059 | Not a renal disease focus |
| 1 | Differential regulation of serum- and glucocorticoid-inducible kinase 1 (SGK1) splice variants based on alternative initiation of transcription. | Simon P et al. | 17982254 | Not a renal disease focus |
| 1 | Polymorphisms in genes related to activation or detoxification of carcinogens might interact with smoking to increase renal cancer risk: results from The Netherlands Cohort Study on diet and cancer. | Smits KM et al. | 17982751 | Not a renal disease focus |
| 1 | Dominant-negative HIF-3 alpha 4 suppresses VHL-null renal cell carcinoma progression. | Maynard MA et al. | 17998805 | Not a renal disease focus |
| 1 | The extracolonic cancer spectrum in females with the common 'South African' hMLH1 c.C1528T mutation. | Blokhuis MM et al. | 18049911 | Not a renal disease focus |
| 1 | Position of nonmuscle myosin heavy chain IIA (NMMHC-IIA) mutations predicts the natural history of MYH9-related disease. | Pecci A et al. | 18059020 | Not a renal disease focus |
| 1 | Lack of association of a functional single nucleotide polymorphism of PTPN22, encoding lymphoid protein phosphatase, with susceptibility to Henoch-SchÃ¶nlein purpura. | Orozco G et al. | 18078626 | Not a renal disease focus |
| 1 | Progesterone receptor reactivity in renal oncocytoma and chromophobe renal cell carcinoma. | Mai KT et al. | 18081814 | Not a renal disease focus |
| 1 | Constant allelic alteration on chromosome 16p (TSC2 gene) in perivascular epithelioid cell tumour (PEComa): genetic evidence for the relationship of PEComa with angiomyolipoma. | Pan CC et al. | 18085521 | Not a renal disease focus |
| 1 | Association of angiotensin-converting enzyme gene insertion/deletion polymorphism with metabolic syndrome in Iranians with type 2 diabetes mellitus. | Nikzamir A et al. | 18154415 | Not a renal disease focus |
| 1 | Association of CDKAL1, IGF2BP2, CDKN2A/B, HHEX, SLC30A8, and KCNJ11 with susceptibility to type 2 diabetes in a Japanese population. | Omori S et al. | 18162508 | Not a renal disease focus |

| | | | | |
|---|---|---|---|---|
| 1 | Renal oncocytoma with and without intravascular extension into the branches of renal vein have the same morphological, immunohistochemical and genetic features. | Hes O et al. | 18196270 | Not a renal disease focus |
| 1 | Association of SGK1 gene polymorphisms with type 2 diabetes. | Schwab M et al. | 18209482 | Not a renal disease focus |
| 1 | Association of a functional cytochrome P450 4F2 haplotype with urinary 20-HETE and hypertension. | Liu H et al. | 18235092 | Not a renal disease focus |
| 1 | Haplotypes of the HRES-1 endogenous retrovirus are associated with development and disease manifestations of systemic lupus erythematosus. | Pullmann R Jr et al. | 18240231 | Not a renal disease focus |
| 1 | Genetic and epigenetic alterations in the von hippel-lindau gene: the influence on renal cancer prognosis. | Smits KM et al. | 18245539 | Not a renal disease focus |
| 1 | AMP-activated protein kinase inhibits transforming growth factor-beta-induced Smad3-dependent transcription and myofibroblast transdifferentiation. | Mishra R et al. | 18250161 | Not a renal disease focus |
| 1 | Oncocytic papillary renal cell carcinoma with solid architecture: mimic of renal oncocytoma. | Mai KT et al. | 18251779 | Not a renal disease focus |
| 1 | Structure of the N-terminal region of complement factor H and conformational implications of disease-linked sequence variations. | Hocking HG et al. | 18252712 | Not a renal disease focus |
| 1 | The role of type 1 and type 2 5'-deiodinase in the pathophysiology of the 3,5,3'-triiodothyronine toxicosis of McCune-Albright syndrome. | Celi FS et al. | 18349068 | Not a renal disease focus |
| 1 | N-acetyltransferase 8, a positional candidate for blood pressure and renal regulation: resequencing, association and in silico study. | Juhanson P et al. | 18402670 | Not a renal disease focus |
| 1 | Association analysis in african americans of European-derived type 2 diabetes single nucleotide polymorphisms from whole-genome association studies. | Lewis JP et al. | 18443202 | Not a renal disease focus |
| 1 | PPP2R2B CAG repeat length in the Han Chinese in Taiwan: Association analyses in neurological and psychiatric disorders and potential functional implications. | Chen CM et al. | 18484086 | Not a renal disease focus |
| 1 | Glutathione S-transferase variants and hypertension. | Delles C et al. | 18551009 | Not a renal disease focus |
| 1 | Allelic loss analysis of tumor suppressor genes regardless of heterozygosity: von Hippel-Lindau gene loss in renal cell carcinoma. | Mochida J et al. | 18554638 | Not a renal disease focus |

| | | | | |
|---|---|---|---|---|
| 1 | Renal cell carcinoma, occupational pesticide exposure and modification by glutathione S-transferase polymorphisms. | Karami S et al. | 18566013 | Not a renal disease focus |
| 1 | No association between single nucleotide polymorphisms and the development of nephrotoxicity after orthotopic heart transplantation. | Klauke B et al. | 18582803 | Not a renal disease focus |
| 1 | Association of GSTM3 intron 6 variant with cigarette smoking, tobacco chewing and alcohol as modifier factors for prostate cancer risk. | Kesarwani P et al. | 18668224 | Not a renal disease focus |
| 1 | Association of renin-angiotensin and endothelial nitric oxide synthase gene polymorphisms with blood pressure progression and incident hypertension: prospective cohort study. | Conen D et al. | 18698212 | Not a renal disease focus |
| 1 | Association of angiotensin-converting enzyme gene dimorphisms with severity of lupus disease. | Rabbani MA et al. | 18711292 | Not a renal disease focus |
| 1 | Development of human cell models for assessing the carcinogenic potential of chemicals. | Pang Y et al. | 18778725 | Not a renal disease focus |
| 1 | SIRT1 genetic variants associate with the metabolic response of Caucasians to a controlled lifestyle intervention--the TULIP Study. | Weyrich P et al. | 19014491 | Not a renal disease focus |
| 1 | Influence of XPD and APE1 DNA repair gene polymorphism on bladder cancer susceptibility in north India. | Gangwar R et al. | 19041121 | Not a renal disease focus |
| 1 | Genetic variants in hypertensive patients with coronary artery disease and coexisting atheromatous renal artery stenosis. | Szperl M et al. | 19043368 | Not a renal disease focus |
| 1 | When should genetic testing be obtained in a patient with phaeochromocytoma or paraganglioma? | Erlic Z et al. | 19067729 | Not a renal disease focus |
| 1 | Identification of novel mutations and sequence variation in the Zellweger syndrome spectrum of peroxisome biogenesis disorders. | Yik WY et al. | 19105186 | Not a renal disease focus |
| 1 | High aldosterone-to-renin variants of CYP11B2 and pregnancy outcome. | Escher G et al. | 19151144 | Not a renal disease focus |
| 1 | The Leu262Val polymorphism of presenilin associated rhomboid like protein (PARL) is associated with earlier onset of type 2 diabetes and increased urinary microalbumin creatinine ratio in an Irish case-control population. | Hatunic M et al. | 19185381 | Not a renal disease focus |
| 1 | A pilot study of genetic polymorphisms and hemodialysis vascular access thrombosis. | Brophy DF et al. | 19210273 | Not a renal disease focus |

| | | | | |
|---|---|---|---|---|
| 1 | CYP1B1 mutations in Spanish patients with primary congenital glaucoma: phenotypic and functional variability. | Campos-Mollo E et al. | 19234632 | Not a renal disease focus |
| 1 | Impact of nucleotide excision repair ERCC2 and base excision repair APEX1 genes polymorphism and its association with recurrence after adjuvant BCG immunotherapy in bladder cancer patients of North India. | Gangawar R et al. | 19242824 | Not a renal disease focus |
| 1 | Functional basis of protection against age-related macular degeneration conferred by a common polymorphism in complement factor B. | Montes T et al. | 19255449 | Not a renal disease focus |
| 1 | Insight into mechanism of oxidative DNA damage in angiomyolipomas from TSC patients. | Habib SL | 19265534 | Not a renal disease focus |
| 1 | Alu-Alu recombination underlies the vast majority of large VHL germline deletions: Molecular characterization and genotype-phenotype correlations in VHL patients. | Franke G et al. | 19280651 | Not a renal disease focus |
| 1 | Loss of function of Sco1 and its interaction with cytochrome c oxidase. | Stiburek L et al. | 19295170 | Not a renal disease focus |
| 1 | Expression of hepatocyte growth factor and its receptor met in Wilms' tumors and nephrogenic rests reflects their roles in kidney development. | Vuononvirta R et al. | 19318497 | Not a renal disease focus |
| 1 | A human polymorphism affects NEDD4L subcellular targeting by leading to two isoforms that contain or lack a C2 domain. | Garrone NF et al. | 19364400 | Not a renal disease focus |
| 1 | The action and mode of binding of thiazolidinedione ligands at free fatty acid receptor 1. | Smith NJ et al. | 19398560 | Not a renal disease focus |
| 1 | Tropical calcific pancreatitis and its association with CTRC and SPINK1 (p.N34S) variants. | Derikx MH et al. | 19404200 | Not a renal disease focus |
| 1 | GSTM1, GSTM3 and GSTT1 gene variants and risk of benign prostate hyperplasia in North India. | Mittal RD et al. | 19407363 | Not a renal disease focus |
| 1 | Structural bioinformatics mutation analysis reveals genotype-phenotype correlations in von Hippel-Lindau disease and suggests molecular mechanisms of tumorigenesis. | Forman JR et al. | 19408298 | Not a renal disease focus |
| 1 | Caspase 9 and caspase 8 gene polymorphisms and susceptibility to bladder cancer in north Indian population. | Gangwar R et al. | 19412632 | Not a renal disease focus |
| 1 | Is polymorphism within eNOS gene associated with the late onset of myocardial infarction? A pilot study. | Gluba A et al. | 19505886 | Not a renal disease focus |

| | | | | |
|---|---|---|---|---|
| 1 | Functional phosphodiesterase 11A mutations may modify the risk of familial and bilateral testicular germ cell tumors. | Horvath A et al. | 19549888 | Not a renal disease focus |
| 1 | Analysis of germline variants in CDH1, IGFBP3, MMP1, MMP3, STK15 and VEGF in familial and sporadic renal cell carcinoma. | Ricketts C et al. | 19551141 | Not a renal disease focus |
| 1 | The calcineurin homologous protein-1 increases Na(+)/H(+) -exchanger 3 trafficking via ezrin phosphorylation. | Di Sole F et al. | 19556366 | Not a renal disease focus |
| 1 | A functional variant of NEDD4L is associated with hypertension, antihypertensive response, and orthostatic hypotension. | Luo F et al. | 19635985 | Not a renal disease focus |
| 1 | A significantly joint effect between arsenic and occupational exposures and risk genotypes/diplotypes of CYP2E1, GSTO1 and GSTO2 on risk of urothelial carcinoma. | Wang YH et al. | 19686770 | Not a renal disease focus |
| 1 | Genetic polymorphisms in genes encoding antioxidant enzymes are associated with diabetic retinopathy in type 1 diabetes. | Hovnik T et al. | 19752172 | Not a renal disease focus |
| 1 | Loss of heterozygosity at 2q37 in sporadic Wilms' tumor: putative role for miR-562. | Drake KM et al. | 19789318 | Not a renal disease focus |
| 1 | Apolipoprotein E/C1 locus variants modify renal cell carcinoma risk. | Moore LE et al. | 19808960 | Not a renal disease focus |
| 1 | Do DNA repair genes OGG1, XRCC3 and XRCC7 have an impact on susceptibility to bladder cancer in the North Indian population? | Gangwar R et al. | 19815090 | Not a renal disease focus |
| 1 | Angiotensin-converting enzyme and angiotensin II receptor subtype 2 genotypes in type 1 diabetes and severe hypoglycaemia requiring emergency treatment: a case cohort study. | Pedersen-Bjergaard U et al. | 19820429 | Not a renal disease focus |
| 1 | Clinical predictors and algorithm for the genetic diagnosis of pheochromocytoma patients. | Erlic Z et al. | 19825962 | Not a renal disease focus |
| 1 | A polymorphism within the fructosamine-3-kinase gene is associated with HbA1c Levels and the onset of type 2 diabetes mellitus. | Mohas M et al. | 19834870 | Not a renal disease focus |
| 1 | A study on the association between angiotensin-I converting enzyme I/D dimorphism and type-2 diabetes mellitus. | Chmaisse HN et al. | 19861867 | Not a renal disease focus |
| 1 | The Int7G24A variant of transforming growth factor-beta receptor type I is a risk factor for colorectal cancer in the male Spanish population: a case-control study. | Castillejo A et al. | 19930569 | Not a renal disease focus |

| | | | | |
|---|---|---|---|---|
| 1 | The role of polymorphisms within paraoxonases (192 Gln/Arg in PON1 and 311Ser/Cys in PON2) in the modulation of cardiovascular risk: a pilot study. | Gluba A et al. | 19939821 | Not a renal disease focus |
| 1 | Endothelial nitric oxide synthase and nicotinamide adenosine dinucleotide phosphate oxidase p22phox gene (C242T) polymorphisms and systemic lupus erythematosus in a Chinese Population. | Tang FY et al. | 19965945 | Not a renal disease focus |
| 1 | A novel human heparanase splice variant, T5, endowed with protumorigenic characteristics. | Barash U et al. | 20007507 | Not a renal disease focus |
| 1 | Influence of CYP3A5 and ABCB1 gene polymorphisms on calcineurin inhibitor-related neurotoxicity after hematopoietic stem cell transplantation. | Yanagimachi M et al. | 20030680 | Not a renal disease focus |
| 1 | Development of a multiplex ligation-dependent probe amplification (MLPA) assay for quantification of the OCRL1 gene. | Coutton C et al. | 20043897 | Not a renal disease focus |
| 1 | DNA repair gene X-ray repair cross-complementing group 1 and xeroderma pigmentosum group D polymorphisms and risk of prostate cancer: a study from North India. | Mandal RK et al. | 20070155 | Not a renal disease focus |
| 1 | Renal cell carcinoma Fuhrman grade and histological subtype correlate with complete polymorphic deletion of glutathione S-transferase M1 gene. | De Martino M et al. | 20083259 | Not a renal disease focus |
| 1 | Genetic variations in the sodium balance-regulating genes ENaC, NEDD4L, NDFIP2 and USP2 influence blood pressure and hypertension. | Jin HS et al. | 20090362 | Not a renal disease focus |
| 1 | Multiorgan detection and characterization of protease-resistant prion protein in a case of variant CJD examined in the United States. | Notari S et al. | 20098730 | Not a renal disease focus |
| 1 | Genetic disorders in complement (regulating) genes in patients with atypical haemolytic uraemic syndrome (aHUS). | Westra D et al. | 20106822 | Not a renal disease focus |
| 1 | CYP4F2 gene V433M polymorphism is associated with ischemic stroke in the male Northern Chinese Han population. | Deng S et al. | 20227456 | Not a renal disease focus |
| 1 | Subtype-specific FBXW7 mutation and MYCN copy number gain in Wilms' tumor. | Williams RD et al. | 20332316 | Not a renal disease focus |
| 1 | Association of selected variants in genes involved in cell cycle and apoptosis with bladder cancer risk in North Indian population. | Gangwar R et al. | 20380574 | Not a renal disease focus |
| 1 | Association of CAPN10 gene with insulin sensitivity, glucose tolerance and renal function in essential hypertensive patients. | Zhou X et al. | 20406624 | Not a renal disease focus |

| | | | | |
|---|---|---|---|---|
| 1 | Clinical and molecular features of familial and sporadic cases of von Hippel-Lindau disease from Mexico. | Chacon-Camacho OF et al. | 20447124 | Not a renal disease focus |
| 1 | N-glycosylation of carnosinase influences protein secretion and enzyme activity: implications for hyperglycemia. | Riedl E et al. | 20460427 | Not a renal disease focus |
| 1 | Combined classical cytogenetics and microarray-based genomic copy number analysis reveal frequent 3;5 rearrangements in clear cell renal cell carcinoma. | Pei J et al. | 20461753 | Not a renal disease focus |
| 1 | The association of glutathione-S-transferase gene polymorphisms (GSTM1, GSTT1, GSTP1) with idiopathic male infertility. | Safarinejad MR et al. | 20505681 | Not a renal disease focus |
| 1 | The non-muscle Myosin heavy chain 9 gene (MYH9) is not associated with lupus nephritis in African Americans. | Freedman BI et al. | 20523037 | Not a renal disease focus |
| 1 | Association of the genetic polymorphisms of the ACE gene and the eNOS gene with lupus nephropathy in northern Chinese population. | Li X et al. | 20540812 | Not a renal disease focus |
| 1 | eNOS gene polymorphism association with retinopathy in type 1 diabetes. | Bazzaz JT et al. | 20565248 | Not a renal disease focus |
| 1 | Clinical and molecular characterization of Brazilian families with von Hippel-Lindau disease: a need for delineating genotype-phenotype correlation. | Gomy I et al. | 20567917 | Not a renal disease focus |
| 1 | Functional polymorphisms in cell death pathway genes and risk of renal cell carcinoma. | Zhu J et al. | 20572163 | Not a renal disease focus |
| 1 | Role of MMP-3 and MMP-9 and their haplotypes in risk of bladder cancer in North Indian cohort. | Srivastava P et al. | 20574775 | Not a renal disease focus |
| 1 | The role of endothelial nitric oxide synthase (eNOS) T-786C, G894T, and 4a/b gene polymorphisms in the risk of idiopathic male infertility. | Safarinejad MR et al. | 20586099 | Not a renal disease focus |
| 1 | Polymorphic variants of DNA repair gene XRCC3 and XRCC7 and risk of prostate cancer: a study from North Indian population. | Mandal RK et al. | 20590474 | Not a renal disease focus |
| 1 | Association of TNFAIP3 polymorphism with susceptibility to systemic lupus erythematosus in a Japanese population. | Kawasaki A et al. | 20617138 | Not a renal disease focus |
| 1 | Functional polymorphisms in the CYP3A4, CYP3A5, and CYP21A2 genes in the risk for hypertension in pregnancy. | Coto E et al. | 20617557 | Not a renal disease focus |
| 1 | Functional polymorphism of the CK2alpha intronless gene plays oncogenic roles in lung cancer. | Hung MS et al. | 20625391 | Not a renal disease focus |

| | | | | |
|---|---|---|---|---|
| 1 | Occupational trichloroethylene exposure and renal carcinoma risk: evidence of genetic susceptibility by reductive metabolism gene variants. | Moore LE et al. | 20663906 | Not a renal disease focus |
| 1 | Influence of caspases 8 and 9 gene promoter polymorphism on prostate cancer susceptibility and early development of hormone refractory prostate cancer. | Kesarwani P et al. | 20804486 | Not a renal disease focus |
| 1 | Are cell cycle and apoptosis genes associated with prostate cancer risk in North Indian population? | Mandal RK et al. | 20822933 | Not a renal disease focus |
| 1 | Bladder cancer risk associated with genotypic polymorphism of the matrix metalloproteinase-1 and 7 in North Indian population. | Srivastava P et al. | 20826916 | Not a renal disease focus |
| 1 | Association of TNFAIP3 interacting protein 1, TNIP1 with systemic lupus erythematosus in a Japanese population: a case-control association study. | Kawasaki A et al. | 20849588 | Not a renal disease focus |
| 1 | S-adenosyl methionine improves early viral responses and interferon-stimulated gene induction in hepatitis C nonresponders. | Feld JJ et al. | 20854821 | Not a renal disease focus |
| 1 | Phosphodiesterase 11A (PDE11A) genetic variants may increase susceptibility to prostatic cancer. | Faucz FR et al. | 20881257 | Not a renal disease focus |
| 1 | A functional variant of the NEDD4L gene is associated with beneficial treatment response with Î²-blockers and diuretics in hypertensive patients. | Svensson-Farbom P et al. | 21052022 | Not a renal disease focus |
| 1 | Characterization of UDP-glucuronosyltransferase 2A1 (UGT2A1) variants and their potential role in tobacco carcinogenesis. | Bushey RT et al. | 21164388 | Not a renal disease focus |
| 1 | hOGG1 Ser326Cys polymorphism and renal cell carcinoma risk in a Chinese population. | Zhao H et al. | 21166493 | Not a renal disease focus |
| 1 | Cancer risks for monoallelic MUTYH mutation carriers with a family history of colorectal cancer. | Win AK et al. | 21171015 | Not a renal disease focus |
| 1 | Isocitrate dehydrogenase 1/2 mutational analyses and 2-hydroxyglutarate measurements in Wilms tumors. | Rakheja D et al. | 21225914 | Not a renal disease focus |
| 1 | A WNK4 gene variant relates to osteoporosis and not to hypertension in the Portuguese population. | Mendes AI et al. | 21236712 | Not a renal disease focus |
| 1 | Three novel mutations in the PHEX gene in Chinese subjects with hypophosphatemic rickets extends genotypic variability. | Jap TS et al. | 21293852 | Not a renal disease focus |

| | | | | |
|---|---|---|---|---|
| 1 | Bimodal distribution of RNA expression levels in human skeletal muscle tissue. | Mason CC et al. | 21299892 | Not a renal disease focus |
| 1 | Investigating paraoxonase-1 gene Q192R and L55M polymorphism in patients with renal cell cancer. | Uyar OA et al. | 21308654 | Not a renal disease focus |
| 1 | The presence of PAI-1 4G/5G and ACE DD genotypes increases the risk of early-stage AVF thrombosis in hemodialysis patients. | Gungor Y et al. | 21332339 | Not a renal disease focus |
| 1 | Association of genetic polymorphisms of CYP 2C19 with hypertension in a Chinese Han population. | Ma Y et al. | 21332417 | Not a renal disease focus |
| 1 | Association between renin-angiotensin-aldosterone system-related genes and blood pressure in a Korean population. | Song SB et al. | 21342026 | Not a renal disease focus |
| 1 | Admixture aberration analysis: application to mapping in admixed population using pooled DNA. | Bercovici S et al. | 21385031 | Not a renal disease focus |
| 1 | Polymorphisms and haplotypes in caspases 8 and 9 genes and risk for prostate cancer: a case-control study in cohort of North India. | George GP et al. | 21396853 | Not a renal disease focus |
| 1 | A novel MECA3 region in human 3p21.3 harboring putative tumor suppressor genes and oncogenes. | Braga E et al. | 21423093 | Not a renal disease focus |
| 1 | GSTT1, GSTM1, and CYP1B1 gene polymorphisms and susceptibility to sporadic renal cell cancer. | Salinas-Sanchez AS et al. | 21458313 | Not a renal disease focus |
| 1 | Insertion/deletion polymorphism of angiotensin I-converting enzyme gene is linked with chromophobe renal cell carcinoma. | de Martino M et al. | 21477733 | Not a renal disease focus |
| 1 | Genetic polymorphisms in APE1 are associated with renal cell carcinoma risk in a Chinese population. | Cao Q et al. | 21538578 | Not a renal disease focus |
| 1 | In vitro transforming potential, intracellular signaling properties, and sensitivity to a kinase inhibitor (sorafenib) of RET proto-oncogene variants Glu511Lys, Ser649Leu, and Arg886Trp. | Prazeres H et al. | 21551259 | Not a renal disease focus |
| 1 | Hypercholesterolemia and a candidate gene within the 12q24 locus. | Gragnoli C. | 21554682 | Not a renal disease focus |
| 1 | Lack of association of Klotho gene variants with valvular and vascular calcification in Caucasians: a candidate gene study of the Framingham Offspring Cohort. | Tangri N et al. | 21565945 | Not a renal disease focus |

| | | | | |
|---|---|---|---|---|
| 1 | Clear-cell papillary renal cell carcinoma: molecular and immunohistochemical analysis with emphasis on the von Hippel-Lindau gene and hypoxia-inducible factor pathway-related proteins. | Rohan SM et al. | 21602815 | Not a renal disease focus |
| 1 | Selected AGXT gene mutations analysis provides a genetic diagnosis in 28% of Tunisian patients with primary hyperoxaluria. | Benhaj Mbarek I et al. | 21612638 | Not a renal disease focus |
| 1 | Familial renal cell carcinoma from the Swedish Family-Cancer Database. | Liu H et al. | 21621909 | Not a renal disease focus |
| 1 | Adult renal cell carcinoma with rhabdoid morphology represents a neoplastic dedifferentiation analogous to sarcomatoid carcinoma. | Chapman-Fredricks JR et al. | 21665507 | Not a renal disease focus |
| 1 | Association of caspases with an increased prostate cancer risk in north Indian population. | Mittal RD et al. | 21668377 | Not a renal disease focus |
| 1 | Association of death receptor 4, Caspase 3 and 5 gene polymorphism with increased risk to bladder cancer in North Indians. | Mittal RD et al. | 21700414 | Not a renal disease focus |
| 1 | The polymorphisms in the VHL and HIF1A genes are associated with the prognosis but not the development of renal cell carcinoma. | Qin C et al. | 21778301 | Not a renal disease focus |
| 1 | Association of single nucleotide polymorphisms in promoter of matrix metalloproteinase-2, 8 genes with bladder cancer risk in Northern India. | Srivastava P et al. | 21784671 | Not a renal disease focus |
| 1 | Association of PTPN22 gene polymorphism and systemic lupus erythematosus in a cohort of Egyptian patients: impact on clinical and laboratory results. | Moez P et al. | 21818561 | Not a renal disease focus |
| 1 | Thrombotic events in MYH9 gene-related autosomal macrothrombocytopenias (old May-Hegglin, Sebastian, Fechtner and Epstein syndromes). | Girolami A et al. | 21842307 | Not a renal disease focus |
| 1 | JAK3 in clear cell renal cell carcinoma: mutational screening and clinical implications. | de Martino M et al. | 21868263 | Not a renal disease focus |
| 1 | Genetic diagnosis of X-linked dominant Hypophosphatemic Rickets in a cohort study: tubular reabsorption of phosphate and 1,25(OH)2D serum levels are associated with PHEX mutation type. | Morey M et al. | 21902834 | Not a renal disease focus |
| 1 | Gene-gene interaction of BLK, TNFSF4, TRAF1, TNFAIP3, and REL in systemic lupus erythematosus. | Zhou XJ et al. | 21905002 | Not a renal disease focus |
| 1 | Vitamin D receptor FokI and BsmI gene polymorphism and its association with grade and stage of renal cell carcinoma in North Indian population. | Arjumand W et al. | 21931993 | Not a renal disease focus |

| | | | | |
|---|---|---|---|---|
| 1 | The polymorphisms of P53 codon 72 and MDM2 SNP309 and renal cell carcinoma risk in a low arsenic exposure area. | Huang CY et al. | 21982800 | Not a renal disease focus |
| 1 | Risk factor for clear cell renal cell carcinoma in Chinese population: a case-control study. | Wang G et al. | 22000673 | Not a renal disease focus |
| 1 | Base excision repair pathway genes polymorphism in prostate and bladder cancer risk in North Indian population. | Mittal RD et al. | 22019847 | Not a renal disease focus |
| 1 | Von Hippel-Lindau (VHL) inactivation in sporadic clear cell renal cancer: associations with germline VHL polymorphisms and etiologic risk factors. | Moore LE et al. | 22022277 | Not a renal disease focus |
| 1 | Impact of glutathione transferase M1, T1, and P1 gene polymorphisms in the genetic susceptibility of North Indian population to renal cell carcinoma. | Ahmad ST et al. | 22054067 | Not a renal disease focus |
| 1 | Whole-exome sequencing of human pancreatic cancers and characterization of genomic instability caused by MLH1 haploinsufficiency and complete deficiency. | Wang L et al. | 22156295 | Not a renal disease focus |
| 1 | Association between urothelial carcinoma after kidney transplantation and aristolochic acid exposure: the potential role of aristolochic acid in HRas and TP53 gene mutations. | Xiao J et al. | 22172840 | Not a renal disease focus |
| 1 | Mapping of the UGT1A locus identifies an uncommon coding variant that affects mRNA expression and protects from bladder cancer. | Tang W et al. | 22228101 | Not a renal disease focus |
| 1 | Paravertebral ligament ossification in vitamin D-resistant rickets: incidence, clinical significance, and genetic evaluation. | Lee SH et al. | 22261628 | Not a renal disease focus |
| 1 | Risk of renal cell carcinoma and polymorphism in phase I xenobiotic metabolizing CYP1A1 and CYP2D6 enzymes. | Ahmad ST et al. | 22281432 | Not a renal disease focus |
| 1 | Relationship between CYP1A1 genetic polymorphisms and renal cancer in China. | Chen J et al. | 22296350 | Not a renal disease focus |
| 1 | Genetic variations in the ADAMTS12 gene are associated with schizophrenia in Puerto Rican patients of Spanish descent. | Bespalova IN et al. | 22322903 | Not a renal disease focus |
| 1 | Association of promoter polymorphisms in MMP2 and TIMP2 with prostate cancer susceptibility in North India. | Srivastava P et al. | 22374248 | Not a renal disease focus |
| 1 | Polymorphisms in base-excision & nucleotide-excision repair genes & prostate cancer risk in north Indian population. | Mandal RK et al. | 22382185 | Not a renal disease focus |
| 1 | Common genetic variants at the 11q13.3 renal cancer susceptibility locus influence binding of HIF to an enhancer of cyclin D1 expression. | Schodel J et al. | 22406644 | Not a renal disease focus |

| | | | | |
|---|---|---|---|---|
| 1 | Mutation screening of the EYA1, SIX1, and SIX5 genes in an East Asian cohort with branchio-oto-renal syndrome. | Wang SH et al. | 22447252 | Not a renal disease focus |
| 1 | Association of Ku70 A-31G polymorphism and risk of renal cell carcinoma in a Chinese population. | Wang W et al. | 22455395 | Not a renal disease focus |
| 1 | INPPL1 is associated with the metabolic syndrome in men with Type 1 diabetes, but not with diabetic nephropathy. | Hyvonen ME et al. | 22486725 | Not a renal disease focus |
| 1 | MicroRNA target site polymorphisms in the VHL-HIF1Î± pathway predict renal cell carcinoma risk. | Wei H et al. | 22517515 | Not a renal disease focus |
| 1 | A two-stage matched case-control study on multiple hypertensive candidate genes in Han Chinese. | Kuo TY et al. | 22534794 | Not a renal disease focus |
| 1 | Somatic mutation analysis of the SDHB, SDHC, SDHD, and RET genes in the clinical assessment of sporadic and hereditary pheochromocytoma. | Weber A et al. | 22573489 | Not a renal disease focus |
| 1 | Mutational analysis of patients with FGF23-related hypophosphatemic rickets. | Kinoshita Y et al. | 22577109 | Not a renal disease focus |
| 1 | A functional polymorphism C-1310G in the promoter region of Ku70/XRCC6 is associated with risk of renal cell carcinoma. | Wang W et al. | 22593040 | Not a renal disease focus |
| 1 | Angiotensin-converting enzyme (ACE) gene II genotype protects against the development of diabetic peripheral neuropathy in type 2 diabetes mellitus. | Mansoor Q et al. | 22607040 | Not a renal disease focus |
| 1 | LMP2, a novel immunohistochemical marker to distinguish renal oncocytoma from the eosinophilic variant of chromophobe renal cell carcinoma. | Zheng G et al. | 22705098 | Not a renal disease focus |
| 1 | BRAF mutations in metanephric adenoma of the kidney. | Choueiri T et al. | 22727996 | Not a renal disease focus |
| 1 | Resequencing the whole MYH7 gene (including the intronic, promoter, and 3' UTR sequences) in hypertrophic cardiomyopathy. | Coto E et al. | 22765922 | Not a renal disease focus |
| 1 | Higher frequency of paraoxonase gene polymorphism and cardiovascular impairment among Brazilian Fabry Disease patients. | Barris-Oliveira AC et al. | 22796398 | Not a renal disease focus |
| 1 | Paraoxonase 1 (PON1) C/T-108 association with longitudinal mean arterial blood pressure. | Bhatnagar V et al. | 22854640 | Not a renal disease focus |

| | | | | |
|---|---|---|---|---|
| 1 | Patients with Lynch syndrome mismatch repair gene mutations are at higher risk for not only upper tract urothelial cancer but also bladder cancer. | Skeldon SC et al. | 22883484 | Not a renal disease focus |
| 1 | Novel missense mutations of WNK1 in patients with hypokalemic salt-losing tubulopathies. | Zhang C et al. | 22934535 | Not a renal disease focus |
| 1 | Proteasome modulator 9 and depression in type 2 diabetes. | Gragnoli C | 22934761 | Not a renal disease focus |
| 1 | Regulatory regions of the paraoxonase 1 (PON1) gene are associated with neovascular age-related macular degeneration (AMD). | Oczos J et al. | 22956172 | Not a renal disease focus |
| 1 | New insights into Dok-4 PTB domain structure and function. | Hooker E et al. | 22982678 | Not a renal disease focus |
| 1 | The role of XRCC6 T-991C functional polymorphism in renal cell carcinoma. | Chang WS et al. | 22993329 | Not a renal disease focus |
| 1 | Integrated multiplex ligation dependent probe amplification (MLPA) assays for the detection of alterations in the HEXB, GM2A and SMARCAL1 genes to support the diagnosis of Morbus Sandhoff, M. Tay-Sachs variant AB and Schimke immuno-osseous dysplasia in humans. | Sobek AK et al. | 23010210 | Not a renal disease focus |
| 1 | Endothelial nitric oxide synthase Glu298Asp polymorphism as a risk factor for prostate cancer. | Ziaei SA et al. | 23015399 | Not a renal disease focus |
| 1 | Calpain-10 gene polymorphisms in type 2 diabetes and its micro- and macrovascular complications. | Buraczynska M et al. | 23021796 | Not a renal disease focus |
| 1 | Genetic variants in metabolizing genes NQO1, NQO2, MTHFR and risk of prostate cancer: a study from North India. | Mandal RK et al. | 23054000 | Not a renal disease focus |
| 1 | Soluble FLT1 binds lipid microdomains in podocytes to control cell morphology and glomerular barrier function. | Jin J et al. | 23063127 | Not a renal disease focus |
| 1 | Polymorphism in protein tyrosine phosphatase receptor delta is associated with the risk of clear cell renal cell carcinoma. | Du Y et al. | 23069849 | Not a renal disease focus |
| 1 | Impact of glutathione S-transferase T1 gene polymorphisms on acute cellular rejection in living donor liver transplantation. | Kamei H et al. | 23153768 | Not a renal disease focus |
| 1 | Interaction of C1GALT1-IL5RA on the susceptibility to IgA nephropathy in Southern Han Chinese. | Wang W et al. | 23190752 | Not a renal disease focus |
| 1 | A functional variant in the MTOR promoter modulates its expression and is associated with renal cell cancer risk. | Cao Q et al. | 23209702 | Not a renal disease focus |

| | | | | |
|---|---|---|---|---|
| 1 | Association of the formiminotransferase N-terminal sub-domain containing gene and thrombospondin, type 1, domain-containing 7A gene with the prevalence of vertebral fracture in 2427 consecutive autopsy cases. | Zhou H et al. | 23303384 | Not a renal disease focus |
| 1 | Phenotypic spectrum and prevalence of INPP5E mutations in Joubert syndrome and related disorders. | Travaglini L et al. | 23386033 | Not a renal disease focus |
| 1 | CYP24A1 and CYP27B1 polymorphisms modulate vitamin D metabolism in colon cancer cells. | Jacobs ET et al. | 23423976 | Not a renal disease focus |
| 1 | Tumor-specific isoform switch of the fibroblast growth factor receptor 2 underlies the mesenchymal and malignant phenotypes of clear cell renal cell carcinomas. | Zhao Q et al. | 23444225 | Not a renal disease focus |
| 1 | Distribution of human leukocyte antigen alleles in systemic lupus erythematosus patients with angiotensin converting enzyme insertion/deletion polymorphism. | Hussain N et al. | 23448612 | Not a renal disease focus |
| 1 | Genetic predisposition to left ventricular hypertrophy and the potential involvement of cystatin-C in untreated hypertension. | Tousoulis D et al. | 23479071 | Not a renal disease focus |
| 1 | Epistatic role of the MYH9/APOL1 region on familial hematuria genes. | Voskarides K et al. | 23516419 | Not a renal disease focus |
| 1 | Pre-treatment role of inosine triphosphate pyrophosphatase polymorphism for predicting anemia in Egyptian hepatitis C virus patients. | Ahmed WH et al. | 23538996 | Not a renal disease focus |
| 1 | Metanephric adenoma and solid variant of papillary renal cell carcinoma: common and distinctive features. | Mantoan Padilha M et al. | 23551615 | Not a renal disease focus |
| 1 | Fluid intake, genetic variants of UDP-glucuronosyltransferases, and bladder cancer risk. | Wang J et al. | 23632476 | Not a renal disease focus |
| 1 | Influence of survivin (BIRC5) and caspase-9 (CASP9) functional polymorphisms in renal cell carcinoma development: a study in a southern European population. | Marques I et al. | 23645041 | Not a renal disease focus |
| 1 | Germline BAP1 mutations predispose to renal cell carcinomas. | Popova T et al. | 23684012 | Not a renal disease focus |
| 1 | Association of B2 receptor polymorphisms and ACE activity with ACE inhibitor-induced angioedema in black and mixed-race South Africans. | Moholisa RR et al. | 23730990 | Not a renal disease focus |

| | | | | |
|---|---|---|---|---|
| 1 | Klotho gene polymorphism of rs3752472 is associated with the risk of urinary calculi in the population of Han nationality in Eastern China. | Xu C et al. | 23756195 | Not a renal disease focus |
| 1 | Oncocytic papillary renal cell carcinoma: a clinicopathological study emphasizing distinct morphology, extended immunohistochemical profile and cytogenetic features. | Xia QY et al. | 23826421 | Not a renal disease focus |
| 1 | Impact of MMP-3 and TIMP-3 gene polymorphisms on prostate cancer susceptibility in North Indian cohort. | Srivastava P et al. | 23872201 | Not a renal disease focus |
| 1 | Clinical phenotypes of Chinese primary hyperparathyroidism patients are associated with the calcium-sensing receptor gene R990G polymorphism. | Han G et al. | 23946278 | Not a renal disease focus |
| 1 | Q222R polymorphism in DNAse I gene is a risk factor for nephritis in South Indian SLE patients. | Panneer D et al. | 23963431 | Not a renal disease focus |
| 1 | cGMP-dependent protein kinase 1 polymorphisms underlie renal sodium handling impairment. | Citterio L et al. | 24060892 | Not a renal disease focus |
| 1 | Cancer risk and overall survival in mismatch repair proficient hereditary non-polyposis colorectal cancer, Lynch syndrome and sporadic colorectal cancer. | Garre P et al. | 24061861 | Not a renal disease focus |
| 1 | Clinical and Genetic Factors Associated With Severe Hematological Toxicity in Glioblastoma Patients During Radiation Plus Temozolomide Treatment: A Prospective Study. | Lombardi G et al. | 24064758 | Not a renal disease focus |
| 1 | Mutations in the mevalonate kinase (MVK) gene cause nonsyndromic retinitis pigmentosa. | Siemiatkowska AM et al. | 24084495 | Not a renal disease focus |
| 1 | Three novel mutations in the carnitine-acylcarnitine translocase (CACT) gene in patients with CACT deficiency and in healthy individuals. | Fukushima T et al. | 24088670 | Not a renal disease focus |
| 1 | Delineation of PIGV mutation spectrum and associated phenotypes in hyperphosphatasia with mental retardation syndrome. | Horn D et al. | 24129430 | Not a renal disease focus |
| 1 | Genetic variation in the GSTM3 promoter confer risk and prognosis of renal cell carcinoma by reducing gene expression. | Tan X et al. | 24157827 | Not a renal disease focus |
| 1 | Early (2008-2010) hospital outbreak of Klebsiella pneumoniae producing OXA-48 carbapenemase in the UK. | Thomas CP et al. | 24207018 | Not a renal disease focus |
| 1 | RTK/ERK pathway under natural selection associated with prostate cancer. | Chen Y et al. | 24223781 | Not a renal disease focus |

| | | | | |
|---|---|---|---|---|
| 1 | Amylase Î±-1A (AMY1A): a novel immunohistochemical marker to differentiate chromophobe renal cell carcinoma from benign oncocytoma. | Jain S et al. | 24225843 | Not a renal disease focus |
| 1 | Clear cell papillary renal cell carcinoma: a clinicopathological study emphasizing ultrastructural features and cytogenetic heterogeneity. | Shi SS et al. | 24294381 | Not a renal disease focus |
| 1 | The epidemic of extended-spectrum-Î²-lactamase-producing Escherichia coli ST131 is driven by a single highly pathogenic subclone, H30-Rx. | Price LB et al. | 24345742 | Not a renal disease focus |
| 1 | Mutations in the UQCC1-interacting protein, UQCC2, cause human complex III deficiency associated with perturbed cytochrome b protein expression. | Tucker EJ et al. | 24385928 | Not a renal disease focus |
| 1 | The association of endothelial nitric oxide synthase gene G894T polymorphism and serum nitric oxide concentration with microalbuminuria in patients with gestational diabetes. | Atay AE et al. | 24403014 | Not a renal disease focus |
| 1 | Association of candidate genetic variants with restless legs syndrome in end stage renal disease: a multicenter case-control study in Taiwan. | Lin CH et al. | 24433515 | Not a renal disease focus |
| 1 | VHL and HIF-1Î±: gene variations and prognosis in early-stage clear cell renal cell carcinoma. | Lessi F et al. | 24446253 | Not a renal disease focus |
| 1 | Genomic architecture and evolution of clear cell renal cell carcinomas defined by multiregion sequencing. | Gerlinger M et al. | 24487277 | Not a renal disease focus |
| 1 | IFT27, encoding a small GTPase component of IFT particles, is mutated in a consanguineous family with Bardet-Biedl syndrome. | Aldahmesh MA et al. | 24488770 | Not a renal disease focus |
| 1 | TNFAIP3 gene polymorphisms associated with differential susceptibility to rheumatoid arthritis and systemic lupus erythematosus in the Korean population. | Kim SK et al. | 24489017 | Not a renal disease focus |
| 1 | HER2 protein overexpression and gene amplification in upper urinary tract urothelial carcinoma-an analysis of 171 patients. | Sasaki Y et al. | 24551292 | Not a renal disease focus |
| 1 | Genotype-phenotype correlations, and retinal function and structure in von Hippel-Lindau disease. | Wittstrom E et al. | 24555745 | Not a renal disease focus |
| 1 | Higher thrombin activatable fibrinolysis inhibitor levels are associated with inflammation in attack-free familial Mediterranean fever patients. | Bavbek N et al. | 24580410 | Not a renal disease focus |
| 1 | Contribution of CDKAL1 rs7756992 and IGF2BP2 rs4402960 polymorphisms in type 2 diabetes, diabetic complications, obesity risk and hypertension in the Tunisian population. | Lasram K et al. | 24636221 | Not a renal disease focus |

| | | | | |
|---|---|---|---|---|
| 1 | Testing of potential glycan-based heparanase inhibitors in a fluorescence activity assay using either bacterial heparinase II or human heparanase. | Schoenfeld AK et al. | 24667567 | Not a renal disease focus |
| 1 | Impact of the common genetic associations of age-related macular degeneration upon systemic complement component C3d levels. | Ristau T et al. | 24675670 | Not a renal disease focus |
| 1 | Lipoprotein (a) concentrations, apolipoprotein (a) phenotypes, and peripheral arterial disease in three independent cohorts. | Laschkolnig A et al. | 24760552 | Not a renal disease focus |
| 1 | TMPRSS6 rs855791 polymorphism influences the susceptibility to iron deficiency anemia in women at reproductive age. | Pei SN et al. | 24782651 | Not a renal disease focus |
| 1 | Manganese superoxide dismutase (SOD2) polymorphisms, plasma advanced oxidation protein products (AOPP) concentration and risk of kidney complications in subjects with type 1 diabetes. | Mohammedi K et al. | 24819633 | Not a renal disease focus |
| 1 | A labor- and cost-effective non-optical semiconductor (Ion Torrent) next-generation sequencing of the SLC12A3 and CLCNKA/B genes in Gitelman's syndrome patients. | Tavira B et al. | 24830959 | Not a renal disease focus |
| 1 | Genetic polymorphisms of paraoxonase1 192 and glutathione peroxidase1 197 enzymes in familial Mediterranean fever. | Oktem F et al. | 24841661 | Not a renal disease focus |
| 1 | Common variants of cGKII/PRKG2 are not associated with gout susceptibility. | Sakiyama M et al. | 24882840 | Not a renal disease focus |
| 1 | Bobby Sox homology regulates odontoblast differentiation of human dental pulp stem cells/progenitors. | Choi YA et al. | 24885382 | Not a renal disease focus |
| 1 | A complement factor B mutation in a large kindred with atypical hemolytic uremic syndrome. | Funato M et al. | 24906628 | Not a renal disease focus |
| 1 | Recurrent somatic mutation in DROSHA induces microRNA profile changes in Wilms tumour. | Torrezan GT et al. | 24909261 | Not a renal disease focus |
| 1 | Breast cancer risk, nightwork, and circadian clock gene polymorphisms. | Truong T et al. | 24919398 | Not a renal disease focus |
| 1 | The role of endothelial nitric oxide synthase gene G894T and intron 4 VNTR polymorphisms in hemodialysis patients with vascular access thrombosis. | Sener EF et al. | 24936541 | Not a renal disease focus |
| 1 | The consensus-based approach for gene/enzyme replacement therapies and crystallization strategies: the case of human alanine-glyoxylate aminotransferase. | Mesa-Torres N et al. | 24957194 | Not a renal disease focus |

| | | | | |
|---|---|---|---|---|
| 1 | Polymorphisms in genes of the renin-angiotensin-aldosterone system and renal cell cancer risk: interplay with hypertension and intakes of sodium, potassium and fluid. | Deckers IA et al. | 24978482 | Not a renal disease focus |
| 1 | The effect of a single nucleotide polymorphism of the CYP4F2 gene on blood pressure and 20-hydroxyeicosatetraenoic acid excretion after weight loss. | Ward NC et al. | 24984178 | Not a renal disease focus |
| 1 | Cytochrome P450 1B1 polymorphisms and risk of renal cell carcinoma in men. | Chang I et al. | 25027399 | Not a renal disease focus |
| 1 | Single nucleotide polymorphism-single nucleotide polymorphism interactions among inflammation genes in the genetic architecture of blood pressure in the Framingham Heart Study. | Basson JJ et al. | 25063733 | Not a renal disease focus |
| 1 | Germline BAP1 mutations predispose also to multiple basal cell carcinomas. | de la Fouchardiere A et al. | 25080371 | Not a renal disease focus |
| 1 | Fluorescence in situ hybridization of chromosomeÂ 17 polysomy in breast cancer using thin tissue sections causes the loss of CEP17 and HER2 signals. | Jiang H et al. | 25119636 | Not a renal disease focus |
| 1 | Single-nucleotide polymorphisms in the UDP-glucuronosyltransferase 1A-3' untranslated region are associated with atazanavir-induced nephrolithiasis in patients with HIV-1 infection: a pharmacogenetic study. | Nishijima T et al. | 25151207 | Not a renal disease focus |
| 1 | A low-frequency variant in MAPK14 provides mechanistic evidence of a link with myeloperoxidase: a prognostic cardiovascular risk marker. | Waterworth DM et al. | 25164947 | Not a renal disease focus |
| 1 | Identification of genetic markers for treatment success in heart failure patients: insight from cardiac resynchronization therapy. | Schmitz B et al. | 25210049 | Not a renal disease focus |
| 1 | Two single nucleotide polymorphisms in the von Hippel-Lindau tumor suppressor gene in Taiwanese with renal cell carcinoma. | Wang WC et al. | 25217002 | Not a renal disease focus |
| 1 | Replicative study of GWAS TP63C/T, TERTC/T, and SLC14A1C/T with susceptibility to bladder cancer in North Indians. | Singh V et al. | 25218484 | Not a renal disease focus |
| 1 | Molecular epidemiology of extended-spectrum beta-lactamase (ESBL)-positive Klebsiella pneumoniae from bloodstream infections and risk factors for mortality. | Gurntke S et al. | 25224765 | Not a renal disease focus |
| 1 | Pharmacologic rescue of an enzyme-trafficking defect in primary hyperoxaluria 1. | Miyata N et al. | 25237136 | Not a renal disease focus |

| | | | | |
|---|---|---|---|---|
| 1 | Variants in angiogenesis-related genes and the risk of clear cell renal cell carcinoma. | Qin C et al. | 25239121 | Not a renal disease focus |
| 1 | PYGM expression analysis in white blood cells: a complementary tool for diagnosing McArdle disease? | de Luna N et al. | 25240406 | Not a renal disease focus |
| 1 | LRRK2 G2385R and R1628P mutations are associated with an increased risk of Parkinson's disease in the Malaysian population. | Gopalai AA et al. | 25243190 | Not a renal disease focus |
| 1 | Gender differences in impact of CYP2C19 polymorphism on development of coronary artery disease. | Hokimoto S et al. | 25264752 | Not a renal disease focus |
| 1 | Genotype and phenotype frequencies of paraoxonase 1 in fertile and infertile men. | Tavilani H et al. | 25264968 | Not a renal disease focus |
| 1 | Phase 1 trial of tivantinib in combination with sorafenib in adult patients with advanced solid tumors. | Puzanov I et al. | 25294187 | Not a renal disease focus |
| 1 | G-protein receptor kinase 4 polymorphism and response to antihypertensive therapy. | Muskalla AM et al. | 25301854 | Not a renal disease focus |
| 1 | Downregulation of NDUFB6 due to 9p24.1-p13.3 loss is implicated in metastatic clear cell renal cell carcinoma. | Narimatsu T et al. | 25315157 | Not a renal disease focus |
| 1 | Association of polymorphisms of angiotensin I converting enzyme 2 with retinopathy in type 2 diabetes mellitus among Chinese individuals | Meng N et al. | 25359286 | Not a renal disease focus |
| 1 | Matrix metalloproteinase 9 polymorphisms and systemic lupus erythematosus: correlation with systemic inflammatory markers and oxidative stress. | Bahrehmand F et al. | 25416694 | Not a renal disease focus |
| 1 | Rhabdoid tumor predisposition syndrome. | Sredni ST et al. | 25494491 | Not a renal disease focus |
| 1 | Autosomal recessive lissencephaly with cerebellar hypoplasia is associated with a loss-of-function mutation in CDK5. | Magen D et al. | 25560765 | Not a renal disease focus |
| 1 | Exome-wide Sequencing Shows Low Mutation Rates and Identifies Novel Mutated Genes in Seminomas. | Cutcutache I et al. | 25597018 | Not a renal disease focus |
| 1 | Molecular and immunohistochemical characterization reveals novel BRAF mutations in metanephric adenoma. | Udager AM et al. | 25602792 | Not a renal disease focus |
| 1 | Genome-wide association study of clinically defined gout identifies multiple risk loci and its association with clinical subtypes. | Matsuo H et al. | 25646370 | Not a renal disease focus |

| | | | | |
|---|---|---|---|---|
| 1 | The role of hOGG1 C1245G polymorphism in the susceptibility to lupus nephritis and modulation of the plasma 8-OHdG in patients with systemic lupus erythematosus. | Lee HT et al. | 25671815 | Not a renal disease focus |
| 1 | Endothelial nitric oxide synthase gene intron 4 variable number tandem repeat polymorphism in Î²-thalassemia major: relation to cardiovascular complications. | Tantawy AA et al. | 25699607 | Not a renal disease focus |
| 1 | Genetic variants in five novel loci including CFB and CD40 predispose to chronic hepatitis B. | Jiang DK et al. | 25802187 | Not a renal disease focus |
| 1 | SNP Variants in RET and PAX2 and Their Possible Contribution to the Primary Hyperoxaluria Type 1 Phenotype. | Coulter-Mackie MB | 25854853 | Not a renal disease focus |
| 1 | Polymorphic differences in the SOD-2 gene may affect the pathogenesis of nephropathy in patients with diabetes and diabetic complications. | Houldsworth A et al. | 25858271 | Not a renal disease focus |
| 1 | Retrospective analysis of FFPE based Wilms' Tumor samples through copy number and somatic mutation related Molecular Inversion Probe Based Array. | Singh N et al. | 25913740 | Not a renal disease focus |
| 1 | Genotype-phenotype analysis of von Hippel-Lindau syndrome in fifteen Indian families. | Vikkath N et al. | 25952756 | Not a renal disease focus |
| 1 | Angiotensin-converting enzyme (ACE) gene insertion/deletion polymorphism is not a risk factor for hypertension in SLE nephritis. | Negi VS et al. | 25957879 | Not a renal disease focus |
| 1 | A novel phenotype in N-glycosylation disorders: Gillessen-Kaesbach-Nishimura skeletal dysplasia due to pathogenic variants in ALG9. | Tham E et al. | 25966638 | Not a renal disease focus |
| 1 | Loss of BAP1 Expression in Basal Cell Carcinomas in Patients With Germline BAP1 Mutations. | Mochel MC et al. | 25972334 | Not a renal disease focus |
| 1 | Spectrum of mutations in the ATP binding domain of ATP7B gene of Wilson Disease in a regional Indian cohort. | Guggilla SR et al. | 25982861 | Not a renal disease focus |
| 1 | Prevalence of Mycobacterium avium subsp. paratuberculosis and Escherichia coli in blood samples from patients with inflammatory bowel disease. | Nazareth N et al. | 25994082 | Not a renal disease focus |
| 1 | Matrix metalloproteinase-2 (MMP-2) gene polymorphism and cardiovascular comorbidity in type 2 diabetes patients. | Buraczynska M et al. | 26025700 | Not a renal disease focus |
| 1 | Genetic Variants in Caveolin-1 and RhoA/ROCK1 Are Associated with Clear Cell Renal Cell Carcinoma Risk in a Chinese Population. | Zhao R et al. | 26066055 | Not a renal disease focus |

| | | | | |
|---|---|---|---|---|
| 1 | Genetic and Functional Analysis of Polymorphisms in the Human Dopamine Receptor and Transporter Genes in Small Cell Lung Cancer. | Cherubini E et al. | 26081799 | Not a renal disease focus |
| 1 | Exploring genotype-phenotype relationships in Bardet-Biedl syndrome families. | Castro-Sanchez S et al. | 26082521 | Not a renal disease focus |
| 1 | Whole Exome Sequencing Reveals Novel PHEX Splice Site Mutations in Patients with Hypophosphatemic Rickets. | Ma SL et al. | 26107949 | Not a renal disease focus |
| 1 | Exome sequencing in seven families and gene-based association studies indicate genetic heterogeneity and suggest possible candidates for fibromuscular dysplasia. | Kiando SR et al. | 26147384 | Not a renal disease focus |
| 1 | TERT Promoter Mutations in Papillary Thyroid Microcarcinomas. | de Biase D et al. | 26148423 | Not a renal disease focus |
| 1 | A genome-wide screening and SNPs-to-genes approach to identify novel genetic risk factors associated with frontotemporal dementia. | Ferrari R et al. | 26154020 | Not a renal disease focus |
| 1 | GSTM1, GSTT1 and GSTP1 Genetic Variants in Multiple Urologic Cancers. | Chirila DN et al. | 26158735 | Not a renal disease focus |
| 1 | Renal cell carcinoma risk is associated with the interactions of APOE, VHL and MTHFR gene polymorphisms. | Lv C et al. | 26191297 | Not a renal disease focus |
| 1 | Financial incentives for kidney donation: A comparative case study using synthetic controls. | Bilgel F et al. | 26218985 | Not a renal disease focus |
| 1 | Novel mutations of the ATP7B gene in Han Chinese families with pre-symptomatic Wilson's disease. | Yuan ZF et al. | 26253413 | Not a renal disease focus |
| 1 | Lack of genetic association of the TGM2 gene with schizophrenia in a Chinese population. | Wang J et al. | 26307914 | Not a renal disease focus |
| 1 | Genetic Polymorphism of MMP2 Gene and Susceptibility to Prostate Cancer. | Adabi Z et al. | 26319608 | Not a renal disease focus |
| 1 | A method for predicting target drug efficiency in cancer based on the analysis of signaling pathway activation. | Artemov A et al. | 26320181 | Not a renal disease focus |
| 1 | Effect of a functional polymorphism in the pre-miR-146a gene on the risk and prognosis of renal cell carcinoma. | Huang Z et al. | 26323945 | Not a renal disease focus |
| 1 | The sirtuin inhibitor sirtinol inhibits hepatitis A virus (HAV) replication by inhibiting HAV internal ribosomal entry site activity. | Kanda T et al. | 26388050 | Not a renal disease focus |

| | | | | |
|---|---|---|---|---|
| 1 | Molecular characterization of multidrug-resistant Klebsiella pneumoniae isolates. | Hou XH et al. | 26413058 | Not a renal disease focus |
| 1 | Variants in Vitamin D Binding Protein Gene Are Associated With Gestational Diabetes Mellitus. | Wang Y et al. | 26448018 | Not a renal disease focus |
| 1 | Role of PTPN22 and CSK gene polymorphisms as predictors of susceptibility and clinical heterogeneity in patients with Henoch-SchÃ¶nlein purpura (IgA vasculitis). | Lopez-Mejias R et al. | 26458874 | Not a renal disease focus |
| 1 | Association of polymorphisms in PRKCI gene and risk of prostate cancer in a sample of Iranian Population. | Hashemi M et al. | 26475383 | Not a renal disease focus |
| 1 | Analysis of Multiple Families With Single Individuals Affected by Pseudohypoparathyroidism Type Ib (PHP1B) Reveals Only One Novel Maternally Inherited GNAS Deletion. | Takatani R et al. | 26479409 | Not a renal disease focus |
| 1 | Constitutional de novo deletion of the FBXW7 gene in a patient with focal segmental glomerulosclerosis and multiple primitive tumors. | Roversi G et al. | 26482194 | Not a renal disease focus |
| 1 | MKS1 regulates ciliary INPP5E levels in Joubert syndrome. | Slaats GG et al. | 26490104 | Not a renal disease focus |
| 1 | Association of Angiotensin Converting Enzyme Insertion-Deletion Polymorphism with Hypertension in Emiratis with Type 2 Diabetes Mellitus and Its Interaction with Obesity Status. | Alsafar H et al. | 26491214 | Not a renal disease focus |
| 1 | Association of chitotriosidase enzyme activity and genotype with the risk of nephropathy in type 2 diabetes. | Elmonem MA et al. | 26589000 | Not a renal disease focus |
| 1 | Use of a High-Density Protein Microarray to Identify Autoantibodies in Subjects with Type 2 Diabetes Mellitus and an HLA Background Associated with Reduced Insulin Secretion. | Chang DC et al. | 26606528 | Not a renal disease focus |
| 1 | Analysis of urinary cathepsin C for diagnosing Papillon-LefÃ¨vre syndrome. | Hamon Y et al. | 26607765 | Not a renal disease focus |
| 1 | Genetic alteration in notch pathway is associated with better prognosis in renal cell carcinoma. | Feng C et al. | 26662507 | Not a renal disease focus |
| 1 | Genotype-guided tacrolimus dosing in African-American kidney transplant recipients. | Sanghavi K et al. | 26667830 | Not a renal disease focus |
| 1 | Hepatocystin is Essential for TRPM7 Function During Early Embryogenesis. | Overton JD et al. | 26671672 | Not a renal disease focus |

| | | | | |
|---|---|---|---|---|
| 1 | XRCC3 Thr241Met and XPD Lys751Gln gene polymorphisms and risk of clear cell renal cell carcinoma. | Loghin A et al. | 26682510 | Not a renal disease focus |
| 1 | Germline BAP1 Mutational Landscape of Asbestos-Exposed Malignant Mesothelioma Patients with Family History of Cancer. | Ohar JA et al. | 26719535 | Not a renal disease focus |
| 1 | HABP2 G534E Variant in Papillary Thyroid Carcinoma. | Tomsic J et al. | 26745718 | Not a renal disease focus |
| 1 | Glutathione peroxidase-1 gene (GPX1) variants, oxidative stress and risk of kidney complications in people with type 1 diabetes. | Mohammedi K et al. | 26773925 | Not a renal disease focus |
| 1 | Serum paraoxonase-1 gene polymorphism and enzyme activity in patients with urolithiasis. | Atar A et al. | 26795139 | Not a renal disease focus |
| 1 | Association of ACE gene D polymorphism with left ventricular hypertrophy in patients with diastolic heart failure: a case-control study. | Bahramali E et al. | 26861937 | Not a renal disease focus |
| 1 | Association of the BsmI, ApaI, TaqI, Tru9I and FokI Polymorphisms of the Vitamin D Receptor Gene with Nephrolithiasis in the Turkish Population. | Cakir OO et al. | 26945655 | Not a renal disease focus |
| 1 | System-Wide Modulation of HECT E3 Ligases with Selective Ubiquitin Variant Probes. | Zhang W et al. | 26949039 | Not a renal disease focus |
| 1 | Spondyloenchondrodysplasia Due to Mutations in ACP5: A Comprehensive Survey. | Briggs TA et al. | 26951490 | Not a renal disease focus |
| 1 | Novel NEK8 Mutations Cause Severe Syndromic Renal Cystic Dysplasia through YAP Dysregulation. | Grampa V et al. | 26967905 | Not a renal disease focus |
| 1 | Angiotensin-converting enzyme gene I/D polymorphism increases the susceptibility to hypertension and additive diseases: A study on North Indian patients. | Singh M et al. | 27030424 | Not a renal disease focus |
| 1 | Cystinosin deficiency causes podocyte damage and loss associated with increased cell motility. | Ivanova EA et al. | 27083281 | Not a renal disease focus |
| 1 | The VHL short variant involves in protein quality control. | Liu Y et al. | 27196060 | Not a renal disease focus |
| 1 | Vascular endothelial growth factor polymorphism (-460 T/C) is related to hypertension-associated chronic kidney disease. | Malkiewicz A et al. | 27367286 | Not a renal disease focus |
| 1 | The association between fructosamine-3 kinase 900C/G polymorphism, transferrin polymorphism and human herpesvirus-8 infection in diabetics living in South Kivu. | Cikomola JC et al. | 27461879 | Not a renal disease focus |

| | | | | |
|---|---|---|---|---|
| 1 | Pro198Leu Polymorphism in the Glutathione Peroxidase 1 Gene Contributes to Diabetic Peripheral Neuropathy in Type 2 Diabetes Patients. | Buraczynska M et al. | 27592002 | Not a renal disease focus |
| 1 | ERBB4 gene polymorphisms and the risk of prostate cancer in a sample of Iranian Population. | Hashemi M et al. | 27609473 | Not a renal disease focus |
| 1 | VEGF-A and VEGFR1 SNPs associate with preeclampsia in a Philippine population. | Amosco MD et al. | 27668980 | Not a renal disease focus |
| 1 | Assessment of Human Tribbles Homolog 3 Genetic Variation (rs2295490) Effects on Type 2 Diabetes Patients with Glucose Control and Blood Pressure Lowering Treatment. | He F et al. | 27793583 | Not a renal disease focus |
| 1 | TET2 binds the androgen receptor and loss is associated with prostate cancer. | Nickerson ML et al. | 27819678 | Not a renal disease focus |
| 1 | PCSK9 genetic variants and risk of type 2 diabetes: a mendelian randomisation study. | Schmidt AF et al. | 27908689 | Not a renal disease focus |
| 1 | Hypertension is a characteristic complication of X-linked hypophosphatemia. | Nakamura Y et al. | 28025445 | Not a renal disease focus |
| 1 | URAT1 inhibition by ALPK1 is associated with uric acid homeostasis. | Kuo TM et al. | 28039413 | Not a renal disease focus |
| 1 | Autophagy-related gene LRRK2 is likely a susceptibility gene for systemic lupus erythematosus in northern Han Chinese. | Zhang YM et al. | 28099919 | Not a renal disease focus |
| 1 | Association Study of Klotho Gene Polymorphism With Calcium Oxalate Stones in The Uyghur Population of Xinjiang, China. | Ali A et al. | 28116736 | Not a renal disease focus |
| 1 | Rare and low-frequency coding variants alter human adult height. | Marouli E et al. | 28146470 | Not a renal disease focus |
| 1 | An association between overexpression of DNA methyltransferase 3B4 and clear cell renal cell carcinoma. | Liu Y et al. | 28160561 | Not a renal disease focus |
| 1 | BARD1 Gene Polymorphisms Confer Nephroblastoma Susceptibility. | Fu W et al. | 28161399 | Not a renal disease focus |
| 1 | Genetic susceptibility variants for lung cancer: replication study and assessment as expression quantitative trait loci. | Pintarelli G et al. | 28181565 | Not a renal disease focus |
| 1 | Relationship between salivary/pancreatic amylase and body mass index: a systems biology approach. | Bonnefond A et al. | 28228143 | Not a renal disease focus |

| | | | | |
|---|---|---|---|---|
| 1 | Development of hypomelanotic macules is associated with constitutive activated mTORC1 in tuberous sclerosis complex. | Moller LB et al. | 28336152 | Not a renal disease focus |
| 1 | A Low-Frequency Inactivating AKT2 Variant Enriched in the Finnish Population Is Associated With Fasting Insulin Levels and Type 2 Diabetes Risk. | Manning A et al. | 28341696 | Not a renal disease focus |
| 1 | Cost-effectiveness Analysis for Genotyping before Allopurinol Treatment to Prevent Severe Cutaneous Adverse Drug Reactions. | Ke CH et al. | 28365572 | Not a renal disease focus |
| 1 | Hepatitis C viral load, genotype, and increased risk of developing end-stage renal disease: REVEAL-HCV study. | Lai TS et al. | 28370058 | Not a renal disease focus |
| 1 | Isolated polycystic liver disease genes define effectors of polycystin-1 function. | Besse W et al. | 28375157 | Not a renal disease focus |
| 1 | Role of MMP-7 in the pathogenesis of systemic lupus erythematosus (SLE). | Vira H et al. | 28420044 | Not a renal disease focus |
| 1 | Frequent somatic mutations in epigenetic regulators in newly diagnosed chronic myeloid leukemia. | Togasaki E et al. | 28452984 | Not a renal disease focus |
| 1 | Ocular Pathology of Oculocerebrorenal Syndrome of Lowe: Novel Mutations and Genotype-Phenotype Analysis. | Song E et al. | 28473699 | Not a renal disease focus |
| 1 | A novel, de novo mutation in the PRKAG2 gene: infantile-onset phenotype and the signaling pathway involved. | Xu Y et al. | 28550180 | Not a renal disease focus |
| 1 | Genome-wide association study of erythrocyte density in sickle cell disease patients. | Ilboudo Y et al. | 28552477 | Not a renal disease focus |
| 1 | Functional polymorphism at the miR-502-binding site in the 3' untranslated region of the SETD8 gene increased the risk of prostate cancer in a sample of Iranian population. | Narouie B et al. | 28578017 | Not a renal disease focus |
| 1 | Acute lymphoblastic leukemia and genetic variations in BHMT gene: Case-control study and computational characterization. | Bellampalli R et al. | 28582843 | Not a renal disease focus |
| 1 | Unusual clinical outcome of primary Hyperoxaluria type 1 in Tunisian patients carrying 33_34InsC mutation. | Mbarek IB et al. | 28619084 | Not a renal disease focus |
| 1 | Disease-linked mutations in factor H reveal pivotal role of cofactor activity in self-surface-selective regulation of complement activation. | Kerr H et al. | 28637873 | Not a renal disease focus |
| 1 | Glomerular Hyperfiltration in Obese African American Hypertensive Patients Is Associated With Elevated Urinary Mitochondrial-DNA Copy Number. | Eirin A et al. | 28641368 | Not a renal disease focus |

| | | | | |
|---|---|---|---|---|
| 1 | Expression and secretion of the Giardia duodenalis variant surface protein 9B10A by transfected trophozoites causes damage to epithelial cell monolayers mediated by protease activity. | Cabrera-Licona A et al. | 28668253 | Not a renal disease focus |
| 1 | Identification of sequence polymorphisms in the mitochondrial cytochrome c oxidase genes as risk factors for hepatocellular carcinoma. | Wang H et al. | 28703354 | Not a renal disease focus |
| 1 | Genome-wide association study identifies inversion in the CTRB1-CTRB2 locus to modify risk for alcoholic and non-alcoholic chronic pancreatitis. | Rosendahl J et al. | 28754779 | Not a renal disease focus |
| 1 | A role of the endothelial nitric oxide system in acute renal colic caused by ureteral stone. | Bulbul E et al. | 28802544 | Not a renal disease focus |
| 1 | An apical MRCK-driven morphogenetic pathway controls epithelial polarity. | Zihni C et al. | 28825699 | Not a renal disease focus |
| 1 | Frequent Genetic Aberrations in the CDK4 Pathway in Acral Melanoma Indicate the Potential for CDK4/6 Inhibitors in Targeted Therapy. | Kong Y et al. | 28830923 | Not a renal disease focus |
| 1 | Association between interferon-induced helicase (IFIH1) rs1990760 polymorphism and seasonal variation in the onset of type 1 diabetes mellitus. | Jermendy A et al. | 28929635 | Not a renal disease focus |
| 1 | AGXT2 rs37369 polymorphism predicts the renal function in patients with chronic heart failure. | Hu XL et al. | 28942034 | Not a renal disease focus |
| 1 | The Endothelial Nitric Oxide Synthase Gene Polymorphism is Associated with the Susceptibility to Immunoglobulin a Nephropathy in Chinese Population. | Gao J et al. | 28946141 | Not a renal disease focus |
| 1 | APOL1, CDKN2A/CDKN2B, and HDAC9 polymorphisms and small vessel ischemic stroke. | Akinyemi R et al. | 28975602 | Not a renal disease focus |
| 1 | Polymorphisms of DNA repair genes XRCC1 and LIG4 and idiopathic male infertility. | Ghasemi H et al. | 28991497 | Not a renal disease focus |
| 1 | Alcohol consumption, variability in alcohol dehydrogenase genes and risk of renal cell carcinoma. | Antwi SO et al. | 29023769 | Not a renal disease focus |
| 1 | Rare ADAR and RNASEH2B variants and a type I interferon signature in glioma and prostate carcinoma risk and tumorigenesis. | Beyer U et al. | 29030706 | Not a renal disease focus |
| 1 | Familial Mediterranean fever mutations are hypermorphic mutations that specifically decrease the activation threshold of the Pyrin inflammasome. | Jamilloux Y et al. | 29040788 | Not a renal disease focus |

| | | | | |
|---|---|---|---|---|
| 1 | Circulating Endothelial Markers in Retinal Vasculopathy With Cerebral Leukoencephalopathy and Systemic Manifestations. | Pelzer N et al. | 29114091 | Not a renal disease focus |
| 1 | Relationship between rs854560 PON1 Gene Polymorphism and Tobacco Smoking with Coronary Artery Disease. | Iwanicka J et al. | 29118461 | Not a renal disease focus |
| 1 | Polymorphism of ERCC1 rs3212986 in Chinese Han women with preeclampsia. | Liu MC et al. | 29153678 | Not a renal disease focus |
| 1 | Analysis of the influence of the T393C polymorphism of the GNAS gene on the clinical expression of primary hyperparathyroidism. | Piedra M et al. | 29179855 | Not a renal disease focus |
| 1 | Association Between Klotho Gene Polymorphism and Markers of Bone Metabolism in Patients Receiving Maintenance Hemodialysis in Iran. | Nazarian A et al. | 29190606 | Not a renal disease focus |
| 1 | Association Between HACE1 Gene Polymorphisms and Wilms' Tumor Risk in a Chinese Population. | Jia W et al. | 29243987 | Not a renal disease focus |
| 1 | PDE1A polymorphism contributes to the susceptibility of nephrolithiasis. | Yang Z et al. | 29262781 | Not a renal disease focus |
| 1 | Genetic screening and functional analysis of CASP9 mutations in a Chinese cohort with neural tube defects. | Liu XZ et al. | 29365368 | Not a renal disease focus |
| 1 | The relationship between ACE/AGT gene polymorphisms and the risk of diabetic retinopathy in Chinese patients with type 2 diabetes. | Qiao YC et al. | 29378484 | Not a renal disease focus |
| 1 | HIPK2 polymorphisms rs2058265, rs6464214, and rs7456421 were associated with kidney stone disease in Chinese males not females. | Lin H et al. | 29428801 | Not a renal disease focus |
| 1 | The association of endothelial nitric oxide synthase gene single nucleotide polymorphisms with paediatric systemic lupus erythematosus. | Zhu J et al. | 29465350 | Not a renal disease focus |
| 1 | Common Variants in ALPL Gene Contribute to the Risk of Kidney Stones in the Han Chinese Population. | Li X et al. | 29489416 | Not a renal disease focus |
| 1 | Impact of eNOS 27-bp VNTR (4b/a) gene polymorphism with the risk of Systemic Lupus Erythematosus in south Indian subjects. | Katkam SK et al. | 29524578 | Not a renal disease focus |
| 1 | Rationale, design, and preliminary results of the Quebec Warfarin Cohort Study. | Perreault S et al. | 29542828 | Not a renal disease focus |
| 1 | The evaluation of two genetic polymorphisms of paraoxonase 1 in patients with pulmonary embolism. | Basol N et al. | 29682786 | Not a renal disease focus |

| | | | | |
|---|---|---|---|---|
| 1 | HCMV UL97 phosphotransferase gene mutations may be associated with antiviral resistance in immunocompromised patients in BelÃ©m, PA, Northern Brazil. | Silva DFLD et al. | 29768545 | Not a renal disease focus |
| 1 | Screening for mutation hotspots in Bardet-Biedl syndrome patients from India. | Chandrasekar SP et al. | 29806606 | Not a renal disease focus |
| 1 | Angiotensin Converting Enzyme Gene Insertion/Deletion Variant and Familial Mediterranean Fever-related Amyloidosis. | Nursal AF et al. | 29891744 | Not a renal disease focus |
| 1 | Base Excision Repair Gene Polymorphisms and Wilms Tumor Susceptibility. | Zhu J et al. | 29937070 | Not a renal disease focus |
| 1 | Concomitance of Polymorphisms in Glutathione Transferase Omega Genes Is Associated with Risk of Clear Cell Renal Cell Carcinoma. | Radic TM et al. | 30224590 | Not a renal disease focus |
| 1 | Dissociation of Fatty Liver and Insulin Resistance in I148M PNPLA3 Carriers: Differences in Diacylglycerol (DAG) FA18:1 Lipid Species as a Possible Explanation. | Franko A et al. | 30227635 | Not a renal disease focus |
| 1 | [Uric acid stones in children. Identification and therapy of a newly detected defect of adenine-phosphoribosyltransferase (author's transl)]. | Simmonds HA | 118367 | Not English or Spanish |
| 1 | [The functional properties of the erythrocyte membranes in patients with chronic glomerulonephritis]. | Rakhov DA et al. | 1475876 | Not English or Spanish |
| 1 | [Lipid peroxidation and the antioxidant system in bicarbonate hemodialysis and acetate-free biofiltration]. | Eiselt J et al. | 7762187 | Not English or Spanish |
| 1 | [A study on angiotensin-I converting enzyme polymorphism in CAPD patients]. | Nishina M | 9014479 | Not English or Spanish |
| 1 | [Is PstI polymorphism of the angiotensin I converting enzyme gene associated with nephropathy development in non-insulin-dependent diabetes mellitus (preliminary study)]. | Grzeszczak W et al. | 9499204 | Not English or Spanish |
| 1 | [Relationship between angiotensin 1 converting enzyme gene polymorphism and diabetic nephropathy]. | Wu S et al. | 9596955 | Not English or Spanish |
| 1 | [Polymorphism studies of angiotensin converting enzyme gene in chronic glomerulonephritis]. | Kutyrina IM et al. | 10420452 | Not English or Spanish |
| 1 | [Angiotensin-converting enzyme gene polymorphism and the clinical pathological features and progression in lupus nephritis]. | Guan T et al. | 10436947 | Not English or Spanish |

| | | | | |
|---|---|---|---|---|
| 1 | [Association between ACE gene polymorphism and therapeutic responsiveness of ACEI in diabetic nephropathy]. | Wang L et al. | 10923445 | Not English or Spanish |
| 1 | [Increased clinical and economic advantages using PROSIT (proteinuria screening and intervention) in type 2 diabetic patients]. | Gozzoli V et al. | 11075242 | Not English or Spanish |
| 1 | [Association between insertion-deletion polymorphism of the angiotensin-converting enzyme gene and development of angiopathies in patients with non-insulin dependent diabetes mellitus from the Chuvash Republic]. | Miloserdova OV et al. | 11234416 | Not English or Spanish |
| 1 | [Renin-angiotensin system genes in chronic glomerulonephritis]. | Buraczynska M et al. | 11865575 | Not English or Spanish |
| 1 | [The association between polymorphism of endothelial nitric oxide synthase gene and diabetic nephropathy]. | Li C et al. | 11930675 | Not English or Spanish |
| 1 | [Relationship between serum angiotensin I-converting enzyme activity and diabetic nephropathy in patients with type II diabetes]. | Liao L et al. | 12016801 | Not English or Spanish |
| 1 | [Acquired mutation of type II transforming growth factor-beta receptor gene in glomerulosclerosis]. | Liu Z et al. | 12126523 | Not English or Spanish |
| 1 | [Renal effects of low doses of mercury]. | Alinovi R et al. | 12197269 | Not English or Spanish |
| 1 | [Association of ecNOS 4 b/a polymorphism and end-stage chronic renal failure]. | Lin S et al. | 12421496 | Not English or Spanish |
| 1 | [Associative analysis of the connection between mutation C1167T in the catalase gene and polymorphic marker D6S392 nearby the Mn-dependent superoxide dismutase gene with diabetic microangiopathies]. | Chistiakov D et al. | 12469627 | Not English or Spanish |
| 1 | [Association of the renin-angiotensin system gene polymorphism with nephropathy in type II diabetes]. | Buraczynska M et al. | 12476891 | Not English or Spanish |
| 1 | [Study on candidate genes of benazepril related cough in Chinese hypertensives]. | Lu J et al. | 12848919 | Not English or Spanish |
| 1 | [Polymorphism at the glutathione S-transferase pi locus as a risk factor for ifosfamide nephrotoxicity in children]. | Zielinska E et al. | 12868187 | Not English or Spanish |
| 1 | [Correlation analysis between WNK4 gene and essential hypertension]. | Sun ZJ et al. | 12905707 | Not English or Spanish |

| | | | | |
|---|---|---|---|---|
| 1 | [Arterial hypertension in glomerulonephritis]. | Oko A et al. | 14974362 | Not English or Spanish |
| 1 | [Search for the association of polymorphic markers for genes coding for antioxidant defense enzymes, with development of diabetic polyneuropathies in patients with type 1 diabetes mellitus]. | Zotova EV et al. | 15125229 | Not English or Spanish |
| 1 | [Association of the complex of polymorphic markers of ACE genes, aldosteron synthetase and endothelial synthetase of nitric oxide with progression of chronic glomerulonephritis]. | Kamysheva ES et al. | 15532370 | Not English or Spanish |
| 1 | [Correlative study between angiotensin-converting enzyme gene polymorphism and hepatorenal syndrome]. | Wu XX et al. | 15698501 | Not English or Spanish |
| 1 | [Screening for new binding proteins which interact with BM2 of influenza B virus with yeast two-hybrid system]. | Yu H et al. | 16027793 | Not English or Spanish |
| 1 | [I/D relationship between polymorphism of ACE gene and progression of chronic glomerulonephritis]. | Kaliev RR et al. | 16078593 | Not English or Spanish |
| 1 | [A search for association between the polymorphic markers of PON1 and PON2 genes and diabetic nephropathy in patients with type I diabetes mellitus]. | Voron'ko OE et al. | 16080611 | Not English or Spanish |
| 1 | [Correlation of cyclin D1 overexpression to mutations of von hippel-lindau gene in renal clear cell carcinoma]. | Ren YY et al. | 16480581 | Not English or Spanish |
| 1 | [Activation of RHOA gene transcription in epithelial tumors may be caused by gene amplification and/or demethylation of its promotor region]. | Braga EA et al. | 17086988 | Not English or Spanish |
| 1 | [Association of aldosterone synthase gene -344 T/C polymorphism with early renal damage in Han nationality with essential hypertension]. | Sun XJ et al. | 17407071 | Not English or Spanish |
| 1 | [Relationship between I/D polymorphism of angiotensin I converting enzyme gene and microvascular complications in type 2 diabetic patients]. | Moleda P et al. | 17941464 | Not English or Spanish |
| 1 | [Inactivation of the VHL gene in sporadic clear cell renal cancer]. | Mikhailenko DS et al. | 18389622 | Not English or Spanish |
| 1 | [Association of alpha-adducin and angiotensin converting enzyme gene polymorphisms with salt-sensitive hypertension and early renal injury]. | Lu LH et al. | 18393230 | Not English or Spanish |
| 1 | [Distribution of polymorphism in endothelial nitric oxide synthase gene in Singapore Chinese and its association with diabetic nephropathy]. | Dong JJ et al. | 18476541 | Not English or Spanish |

| | | | | |
|---|---|---|---|---|
| 1 | [Effect of tissue factor pathway inhibitor on apoptosis of rat mesangial cells and Fas and bcl-2 expression]. | Lin YF et al. | 19094710 | Not English or Spanish |
| 1 | [Genetic predisposition to systemic complications of arterial hypertension in maintenance haemodialysis patients]. | Bzoma B et al. | 19112833 | Not English or Spanish |
| 1 | [Factors responsible for inter-individual variations in dosage/concentration of tacrolimus in renal transplant recipients]. | Fu SJ et al. | 19114346 | Not English or Spanish |
| 1 | [Association analysis of genetic polymorphisms of TCF7L2, CDKAL1, SLC30A8, HHEX genes and microvascular complications of type 2 diabetes mellitus]. | Fu LL et al. | 22487833 | Not English or Spanish |
| 1 | [Angiotensin-converting enyme insertion/deletion polymorphism and blood pressure regulation in type 2 diabetic patients]. | Krajina-Andricevic M et al. | 23120809 | Not English or Spanish |
| 1 | [Association between mannose-binding lectin 2 gene and protein kinase C-beta 1 gene polymorphisms and type 2 diabetic macrovascular complications in northern Chinese Han population]. | Zhang NN et al. | 23225056 | Not English or Spanish |
| 1 | [Gene polymorphisms of CYP3A5 and MDR-1 in Hans renal transplant recipients in Hunan Province]. | Shao M et al. | 23981990 | Not English or Spanish |
| 1 | [Association of CYP3A5 and MDR1 genetic polymorphisms with the blood concentration of tacrolimus in Chinese liver and renal transplant recipients]. | Sun JY et al. | 24059111 | Not English or Spanish |
| 1 | [Gene mutation analysis of X-linked hypophosphatemic rickets]. | Song Y et al. | 24229582 | Not English or Spanish |
| 1 | [Mutational analysis of MYO1E in children with sporadic steroid-resistant nephrotic syndrome in Chinese Han ethnic group]. | Zhao F et al. | 25224051 | Not English or Spanish |
| 1 | [Children with idiopathic hypogonadotropic hypogonadism: clinical data analysis and mutations analysis of KAL1 and FGFR1 gene]. | Qin M et al. | 25619354 | Not English or Spanish |
| 1 | [DNA repair XRCC1, XPD genes polymorphism as associated with the development of bladder cancer and renal cell carcinoma]. | Akhmadishina LZ et al. | 25715450 | Not English or Spanish |
| 1 | [Association between angiotensin-converting enzyme 2 gene polymorphisms and childhood primary nephrotic syndrome]. | Qiu MY et al. | 25815490 | Not English or Spanish |
| 1 | [GENETIC RISK FACTORS FOR MULTIPLE KIDNEY STONE FORMATION IN THE RUSSIAN POPULATION]. | Apolihin OI et al. | 26665756 | Not English or Spanish |

| | | | | |
|---|---|---|---|---|
| 1 | [Polymorphism at the miR-502 binding site in the 3' untranslated region of SET8 gene is associated with the risk of clear cell renal cell carcinoma]. | Xu JS et al. | 27346408 | Not English or Spanish |
| 1 | [Expression of cadherin17 in metanephric adenoma and its value in differential diagnosis]. | Wang X et al. | 27430690 | Not English or Spanish |
| 1 | [Clinical and immunological analysis of patients with activated phosphoinositide 3-kinase Î´ syndrome resulting from PIK3CD mutation]. | Tang WJ et al. | 28072954 | Not English or Spanish |
| 1 | [Molecular features of metanephric adenoma and their values in differential diagnosis]. | Wang X et al. | 28072975 | Not English or Spanish |
| 1 | [Early diagnosis of risk for developing calcium oxalate urolithiasis]. | Apolikhin OI et al. | 28845932 | Not English or Spanish |
| 1 | HLA extended haplotypes in steroid-sensitive nephrotic syndrome of childhood. | Lagueruela CC et al. | 1974661 | Paediatric individuals |
| 1 | Major histocompatibility complex antigens in steroid-responsive nephrotic syndrome. | McEnery PT et al. | 2702084 | Paediatric individuals |
| 1 | Glycosphingolipid levels in an unusual neurovisceral storage disease characterized by lactosylceramide galactosyl hydrolase deficiency: lactosylceramidosis. | Dawson G. | 5016302 | Paediatric individuals |
| 1 | Differentiation of human adult and fetal intestinal alkaline phosphatases with monoclonal antibodies. | Vockley J et al. | 6437214 | Paediatric individuals |
| 1 | Deficient fumarylacetoacetate fumarylhydrolase activity in lymphocytes and fibroblasts from patients with hereditary tyrosinemia. | Kvittingen EA et al. | 6622096 | Paediatric individuals |
| 1 | A polymorphism at codon 160 of human O6-methylguanine-DNA methyltransferase gene in young patients with adult type cancers and functional assay. | Imai Y et al. | 7586149 | Paediatric individuals |
| 1 | Streptokinase activity among group A streptococci in relation to streptokinase genotype, plasminogen binding, and disease manifestations. | Tewodros W et al. | 7783598 | Paediatric individuals |
| 1 | Heterogeneous AVPR2 gene mutations in congenital nephrogenic diabetes insipidus. | Wildin RS et al. | 7913579 | Paediatric individuals |
| 1 | Variability in the rate of 6-mercaptopurine methylation in the erythrocytes, liver and kidney in an Italian population. | Ferroni MA et al. | 8880047 | Paediatric individuals |

| | | | | |
|---|---|---|---|---|
| 1 | Prostanoid biosynthesis by blood monocytes of children with hyperprostaglandin E syndrome. | Nusing RM et al. | 9262230 | Paediatric individuals |
| 1 | Angiotensin I-converting enzyme-gene-polymorphism: relationship to albumin excretion and blood pressure in pediatric patients with type-I-diabetes mellitus. | Pavlovic M et al. | 9354852 | Paediatric individuals |
| 1 | Inherited mutations in PTEN that are associated with breast cancer, cowden disease, and juvenile polyposis. | Lynch ED et al. | 9399897 | Paediatric individuals |
| 1 | ACE gene polymorphism in childhood IgA nephropathy: association with clinicopathologic findings. | Tanaka R et al. | 9590186 | Paediatric individuals |
| 1 | ETV6-NTRK3 gene fusions and trisomy 11 establish a histogenetic link between mesoblastic nephroma and congenital fibrosarcoma. | Knezevich SR et al. | 9823307 | Paediatric individuals |
| 1 | Genetic polymorphisms of the renin-angiotensin system and the outcome of focal segmental glomerulosclerosis in children. | Frishberg Y et al. | 9853248 | Paediatric individuals |
| 1 | Platelet-activating factor acetylhydrolase gene mutation in Japanese nephrotic children. | Xu H et al. | 9853251 | Paediatric individuals |
| 1 | Polymorphisms in angiotensin-converting enzyme gene and severity of renal disease in Henoch-Schoenlein patients. Italian Group of Renal Immunopathology. | Amoroso A et al. | 9870486 | Paediatric individuals |
| 1 | Implications of certain genetic polymorphisms in scarring in vesicoureteric reflux: importance of ACE polymorphism. | Ozen S et al. | 10401028 | Paediatric individuals |
| 1 | Role of platelet-activating factor acetylhydrolase gene mutation in Japanese childhood IgA nephropathy. | Tanaka R et al. | 10430976 | Paediatric individuals |
| 1 | ACE I/D gene polymorphism predicts renal damage in congenital uropathies. | Hohenfellner K et al. | 10452281 | Paediatric individuals |
| 1 | Thiopurine methyltransferase activity and its relationship to the occurrence of rejection episodes in paediatric renal transplant recipients treated with azathioprine. | Dervieux T et al. | 10594482 | Paediatric individuals |
| 1 | Platelet-activating factor acetylhydrolase gene mutation in Japanese children with Escherichia coli O157-associated hemolytic uremic syndrome. | Xu H et al. | 10873870 | Paediatric individuals |
| 1 | Angiotensin-converting enzyme gene insertion/deletion polymorphism and renal damage in childhood uropathies. | al-Eisa A et al. | 10986863 | Paediatric individuals |

| | | | | |
|---|---|---|---|---|
| 1 | Genetic polymorphism in paraoxonase is a risk factor for childhood focal segmental glomerulosclerosis. | Frishberg Y et al. | 11096050 | Paediatric individuals |
| 1 | Polymorphisms of renin-angiotensin system genes in childhood IgA nephropathy. | Maruyama K et al. | 11354780 | Paediatric individuals |
| 1 | Impact of ACE I/D gene polymorphism on congenital renal malformations. | Hohenfellner K et al. | 11354781 | Paediatric individuals |
| 1 | Significance of ACE genotypes and medical treatments in childhood focal glomerulosclerosis. | Hori C et al. | 11474225 | Paediatric individuals |
| 1 | Angiotensin converting enzyme gene insertion/deletion polymorphism in idiopathic nephrotic syndrome in Kuwaiti Arab children. | Al-Eisa A et al. | 11487079 | Paediatric individuals |
| 1 | Distribution and development of CLN2 protein, the late-infantile neuronal ceroid lipofuscinosis gene product. | Kurachi Y et al. | 11547947 | Paediatric individuals |
| 1 | G protein beta3 subunit 825T genotype is not associated with differing outcome in pediatric renal transplant recipients. | Hocher B et al. | 12000471 | Paediatric individuals |
| 1 | Genomic rearrangements of EYA1 account for a large fraction of families with BOR syndrome. | Vervoort VS et al. | 12404110 | Paediatric individuals |
| 1 | Novel ATP6V1B1 and ATP6V0A4 mutations in autosomal recessive distal renal tubular acidosis with new evidence for hearing loss. | Stover EH et al. | 12414817 | Paediatric individuals |
| 1 | ACE gene polymorphism and renal scarring in primary vesicoureteric reflux. | Haszon I et al. | 12478352 | Paediatric individuals |
| 1 | Renin-angiotensin system polymorphisms and renal scarring. | Pardo R et al. | 12579398 | Paediatric individuals |
| 1 | Renin-angiotensin gene polymorphism in children with uremia and essential hypertension. | Papp F et al. | 12579405 | Paediatric individuals |
| 1 | HbA1c levels and erythrocyte transport functions in complication-free type 1 diabetic children and adolescents. | Deak B et al. | 12682823 | Paediatric individuals |
| 1 | Genetic heterogeneity of peroxisome biogenesis disorders among Japanese patients: evidence for a founder haplotype for the most common PEX10 gene mutation. | Shimozawa N et al. | 12794690 | Paediatric individuals |
| 1 | Angiotensin converting enzyme gene polymorphism in Asian Indian children with congenital uropathies. | Bajpai M et al. | 14713838 | Paediatric individuals |

| | | | | |
|---|---|---|---|---|
| 1 | ACE I/D gene polymorphism in primary FSGS and steroid-sensitive nephrotic syndrome. | Oktem F et al. | 14986085 | Paediatric individuals |
| 1 | Renin-angiotensin system polymorphisms in Taiwanese primary vesicoureteral reflux. | Liu KP et al. | 15045574 | Paediatric individuals |
| 1 | Is ACE gene polymorphism a risk factor for renal scarring with low-grade reflux? | Erdogan H et al. | 15138870 | Paediatric individuals |
| 1 | ACE and AT1 receptor gene polymorphisms and renal scarring in urinary bladder dysfunction. | Kostic M et al. | 15179569 | Paediatric individuals |
| 1 | Angiotensin-converting enzyme gene insertion/deletion polymorphism in children with Henoch-Schonlein purpua nephritis. | Zhou J et al. | 15315169 | Paediatric individuals |
| 1 | Late effects on renal glomerular and tubular function in childhood cancer survivors. | Bardi E et al. | 15390293 | Paediatric individuals |
| 1 | Role of truncating mutations in MME gene in fetomaternal alloimmunisation and antenatal glomerulopathies. | Debiec H et al. | 15464186 | Paediatric individuals |
| 1 | Angiotensin-converting enzyme and angiotensin type 2 receptor gene genotype distributions in Italian children with congenital uropathies. | Rigoli L et al. | 15470205 | Paediatric individuals |
| 1 | Low renin-angiotensin system activity gene polymorphism and dysplasia associated with posterior urethral valves. | Peruzzi L et al. | 16006956 | Paediatric individuals |
| 1 | Polymorphisms of the angiotensin converting enzyme and angiotensin II type 1 receptor genes and renal scarring in non-uropathic children with recurrent urinary tract infection. | Ece A et al. | 16109085 | Paediatric individuals |
| 1 | Implication of genetic variations in congenital obstructive nephropathy. | Hahn H et al. | 16133060 | Paediatric individuals |
| 1 | Is paraoxonase 192 gene polymorphism a risk factor for membranoproliferative glomerulonephritis in children? | Bilge I et al. | 16175651 | Paediatric individuals |
| 1 | ACE gene insertion/deletion polymorphism in childhood idiopathic nephrotic syndrome. | Serdaroglu E et al. | 16208534 | Paediatric individuals |
| 1 | Angiotensin converting enzyme gene polymorphism in Indian children with steroid sensitive nephrotic syndrome. | Patil SJ et al. | 16272677 | Paediatric individuals |
| 1 | Evaluation of the antitumor efficacy, pharmacokinetics, and pharmacodynamics of the histone deacetylase inhibitor depsipeptide in childhood cancer models in vivo. | Graham C et al. | 16397046 | Paediatric individuals |

| | | | | |
|---|---|---|---|---|
| 1 | ACE gene polymorphism in children with nephrotic syndrome in the Indonesian population. | Sasongko TH et al. | 16421456 | Paediatric individuals |
| 1 | Renin-angiotensin system gene polymorphisms: association with susceptibility to Henoch-Schonlein purpura and renal involvement. | Ozkaya O et al. | 16521052 | Paediatric individuals |
| 1 | Paraoxonase 1 192 and 55 polymorphisms in nephrotic children. | Biyikli NK et al. | 16565923 | Paediatric individuals |
| 1 | Angiotensin-converting enzyme gene polymorphism in children with idiopathic nephrotic syndrome. | Tsai IJ et al. | 16645262 | Paediatric individuals |
| 1 | ACE gene polymorphism in Turkish children with nephrotic syndrome. | Celik US et al. | 16825089 | Paediatric individuals |
| 1 | Roles of paraoxonase and oxidative stress in adolescents with uraemic, essential or obesity-induced hypertension. | Barath A et al. | 16912512 | Paediatric individuals |
| 1 | Endothelial nitric oxide synthase gene intron 4 a/b VNTR polymorphism in children with APSGN. | Dursun H et al. | 16941147 | Paediatric individuals |
| 1 | Bardet-Biedl syndrome gene variants are associated with both childhood and adult common obesity in French Caucasians. | Benzinou M et al. | 17003356 | Paediatric individuals |
| 1 | HLA class II influences humoral autoimmunity in patients with type 2 autoimmune hepatitis. | Djilali-Saiah et al. | 17050030 | Paediatric individuals |
| 1 | Nitric oxide synthase gene polymorphisms in children with primary nocturnal enuresis: a preliminary study. | Balat A et al. | 17365914 | Paediatric individuals |
| 1 | Molecular and functional characterization of novel glycerol-3-phosphate dehydrogenase 1 like gene (GPD1-L) mutations in sudden infant death syndrome. | Van Norstrand DW et al. | 17967976 | Paediatric individuals |
| 1 | Sudden infant death syndrome and activating GNAS1 gene mutations. | Roman R et al. | 18075835 | Paediatric individuals |
| 1 | Endothelial nitric oxide synthase gene T-786C and 27-bp repeat gene polymorphisms in retinopathy of prematurity. | Rusai K et al. | 18334945 | Paediatric individuals |
| 1 | Loss of nephrocystin-3 function can cause embryonic lethality, Meckel-Gruber-like syndrome, situs inversus, and renal-hepatic-pancreatic dysplasia. | Bergmann C et al. | 18371931 | Paediatric individuals |
| 1 | The polymorphism in insulin receptor substrate-1 gene and birth weight in neonates at term. | Simonska-Cichocka E et al. | 18615395 | Paediatric individuals |

| | | | | |
|---|---|---|---|---|
| 1 | Report of a family segregating mutations in both the APC and MSH2 genes: juvenile onset of colorectal cancer in a double heterozygote. | Uhrhammer N et al. | 18629513 | Paediatric individuals |
| 1 | ACE gene polymorphism in Egyptian children with idiopathic nephrotic syndrome. | Fahmy ME et al. | 18792483 | Paediatric individuals |
| 1 | A common RET variant is associated with reduced newborn kidney size and function. | Zhang Z et al. | 18820179 | Paediatric individuals |
| 1 | Activation of the AKT/mTOR pathway in autosomal recessive polycystic kidney disease (ARPKD). | Fischer DC et al. | 19176689 | Paediatric individuals |
| 1 | Mutations of NPHP2 and NPHP3 in infantile nephronophthisis. | Tory K et al. | 19177160 | Paediatric individuals |
| 1 | Lethal cystic kidney disease in Amish neonates associated with homozygous nonsense mutation of NPHP3. | Simpson MA et al. | 19303681 | Paediatric individuals |
| 1 | Nitric oxide synthase gene polymorphisms in children with minimal change nephrotic syndrome. | Alasehirli B et al. | 19371282 | Paediatric individuals |
| 1 | ACE gene insertion/deletion polymorphism and renal scarring in children with urinary tract infections. | Sekerli E et al. | 19603195 | Paediatric individuals |
| 1 | Effect of paraoxonase 1 gene polymorphisms on clinical course of Henoch-SchÃ¶nlein purpura. | Yilmaz A et al. | 19967651 | Paediatric individuals |
| 1 | Hypophosphatemia, hyperphosphaturia, and bisphosphonate treatment are associated with survival beyond infancy in generalized arterial calcification of infancy. | Rutsch F et al. | 20016754 | Paediatric individuals |
| 1 | Changes in glomerular mesangium in kidneys with congenital nephrotic syndrome of the Finnish type. | Kaukinen A et al. | 20020158 | Paediatric individuals |
| 1 | Polymorphisms of the TNF-alpha and ACE genes, and renal scarring in infants with urinary tract infection. | Savvidou A et al. | 20022049 | Paediatric individuals |
| 1 | Genetic polymorphisms of 17 Î²-hydroxysteroid dehydrogenase 3 and the risk of hypospadias. | Sata F et al. | 20059664 | Paediatric individuals |
| 1 | Association of angiotensin converting enzyme and angiotensin type 2 receptor gene polymorphisms with renal damage in posterior urethral valves. | Laksmi NK et al. | 20149750 | Paediatric individuals |
| 1 | Glutathione S-transferase T1-null seems to be associated with graft failure in hematopoietic SCT. | Elhasid R et al. | 20348973 | Paediatric individuals |

| | | | | |
|---|---|---|---|---|
| 1 | Linkage and association study of discoidin domain receptor 1 as a novel susceptibility gene for childhood IgA nephropathy. | Hahn WH et al. | 20372823 | Paediatric individuals |
| 1 | Effect of angiotensin-converting enzyme gene insertion/deletion polymorphism on steroid resistance in Egyptian children with idiopathic nephrotic syndrome. | Saber-Ayad M et al. | 20418353 | Paediatric individuals |
| 1 | Phosphodiesterase-5 gene (PDE5A) polymorphisms are associated with progression of childhood IgA nephropathy. | Hahn WH et al. | 20563733 | Paediatric individuals |
| 1 | Mutational analysis of the PLCE1 gene in steroid resistant nephrotic syndrome. | Boyer O et al. | 20591883 | Paediatric individuals |
| 1 | The association of genetic variability in patatin-like phospholipase domain-containing protein 3 (PNPLA3) with histological severity of nonalcoholic fatty liver disease. | Rotman Y et al. | 20684021 | Paediatric individuals |
| 1 | Founder mutations in the ATP6V1B1 gene explain most Cypriot cases of distal renal tubular acidosis: first prenatal diagnosis. | Elia A et al. | 20805693 | Paediatric individuals |
| 1 | Polymorphisms of insulin-like growth factor-1 (IGF-1) and IGF-1 receptor (IGF-1R) contribute to pathologic progression in childhood IgA nephropathy. | Hahn WH et al. | 21047277 | Paediatric individuals |
| 1 | Renal manifestations of patients with MYH9-related disorders. | Han KH et al. | 21210153 | Paediatric individuals |
| 1 | Clinical utility of genetic testing in children and adults with steroid-resistant nephrotic syndrome. | Santin S et al. | 21415313 | Paediatric individuals |
| 1 | Induction of podocyte-derived VEGF ameliorates podocyte injury and subsequent abnormal glomerular development caused by puromycin aminonucleoside. | Ma J et al. | 21451433 | Paediatric individuals |
| 1 | ADAMTS13 gene mutations in children with hemolytic uremic syndrome. | Choi HS et al. | 21488199 | Paediatric individuals |
| 1 | RET and GDNF mutations are rare in fetuses with renal agenesis or other severe kidney development defects. | Jeanpierre C et al. | 21490379 | Paediatric individuals |
| 1 | Endothelial nitric oxide synthase gene intron4 VNTR polymorphism in patients with chronic kidney disease. | Elshamaa MF et al. | 21519233 | Paediatric individuals |
| 1 | Disruption of PTPRO causes childhood-onset nephrotic syndrome. | Ozaltin F et al. | 21722858 | Paediatric individuals |

| | | | | |
|---|---|---|---|---|
| 1 | Age and CYP3A5 genotype affect tacrolimus dosing requirements after transplant in pediatric heart recipients. | Gijsen V et al. | 21930396 | Paediatric individuals |
| 1 | DD genotype of ACE gene in boys: may it be a risk factor for minimal change nephrotic syndrome? | Alasehirli B et al. | 22017506 | Paediatric individuals |
| 1 | Association of the ACE-II genotype with the risk of nephrotic syndrome in Pakistani children. | Shahid S et al. | 22033511 | Paediatric individuals |
| 1 | C1GALT1 polymorphisms are associated with Henoch-SchÃ¶nlein purpura nephritis. | He X et al. | 22544166 | Paediatric individuals |
| 1 | Genes in the ureteric budding pathway: association study on vesico-ureteral reflux patients. | van Eerde AM et al. | 22558067 | Paediatric individuals |
| 1 | Genetic polymorphisms influence the steroid treatment of children with idiopathic nephrotic syndrome. | Chiou YH et al. | 22610055 | Paediatric individuals |
| 1 | Mutations in NEK8 link multiple organ dysplasia with altered Hippo signalling and increased c-MYC expression. | Frank V et al. | 23418306 | Paediatric individuals |
| 1 | Association of eNOS gene intron 4 a/b VNTR polymorphisms in children with nephrotic syndrome. | Dursun H et al. | 23570878 | Paediatric individuals |
| 1 | A molecular genetic analysis of childhood nephrotic syndrome in a cohort of Saudi Arabian families. | Al-Hamed MH et al. | 23595123 | Paediatric individuals |
| 1 | Cost-effective PKHD1 genetic testing for autosomal recessive polycystic kidney disease. | Krall P et al. | 24162162 | Paediatric individuals |
| 1 | EIF2AK3 mutations in South Indian children with permanent neonatal diabetes mellitus associated with Wolcott-Rallison syndrome. | Jahnavi S et al. | 24168455 | Paediatric individuals |
| 1 | Molecular diagnosis of distal renal tubular acidosis in Tunisian patients: proposed algorithm for Northern Africa populations for the ATP6V1B1, ATP6V0A4 and SCL4A1 genes. | Elhayek D et al. | 24252324 | Paediatric individuals |
| 1 | MMP-1 and -3 haplotype is associated with congenital anomalies of the kidney and urinary tract. | Djuric T et al. | 24414606 | Paediatric individuals |
| 1 | Mutation screening and array comparative genomic hybridization using a 180K oligonucleotide array in VACTERL association. | Winberg J et al. | 24416387 | Paediatric individuals |
| 1 | Conversion from twice- to once-daily tacrolimus in pediatric kidney recipients: a pharmacokinetic and bioequivalence study. | Lapeyraque AL et al. | 24435759 | Paediatric individuals |

| | | | | |
|---|---|---|---|---|
| 1 | Muscle involvement in Dent disease 2. | Park E et al. | 24912603 | Paediatric individuals |
| 1 | PON1 Q192R polymorphism (rs662) is associated with childhood embryonal tumors. | Vasconcelos GM et al. | 24972570 | Paediatric individuals |
| 1 | Genetic analysis of strictly defined Leber congenital amaurosis with (and without) neurodevelopmental delay. | Khan AO et al. | 24997176 | Paediatric individuals |
| 1 | Modification of epigenetic patterns in low birth weight children: importance of hypomethylation of the ACE gene promoter. | Rangel M et al. | 25170764 | Paediatric individuals |
| 1 | Gene polymorphisms of adducin GLY460TRP, ACE I/D, AND AGT M235T in pediatric hypertension patients. | Kaplan I et al. | 25262176 | Paediatric individuals |
| 1 | Endothelial nitric oxide synthase gene intron 4 VNTR polymorphism in sickle cell disease: relation to vasculopathy and disease severity. | Tantawy AA et al. | 25263931 | Paediatric individuals |
| 1 | HLA-DQA1 and PLCG2 Are Candidate Risk Loci for Childhood-Onset Steroid-Sensitive Nephrotic Syndrome. | Gbadegesin RA et al. | 25349203 | Paediatric individuals |
| 1 | Activation of human telomerase reverse transcriptase through gene fusion in clear cell sarcoma of the kidney. | Karlsson J et al. | 25481751 | Paediatric individuals |
| 1 | Coinheritance of COL4A5 and MYO1E mutations accentuate the severity of kidney disease. | Lennon R et al. | 25739341 | Paediatric individuals |
| 1 | Novel carboxypeptidase A6 (CPA6) mutations identified in patients with juvenile myoclonic and generalized epilepsy. | Sapio MR et al. | 25875328 | Paediatric individuals |
| 1 | HPSE2 mutations in urofacial syndrome, non-neurogenic neurogenic bladder and lower urinary tract dysfunction. | Bulum B et al. | 25924634 | Paediatric individuals |
| 1 | Impact of common functional polymorphisms in renin angiotensin system genes on the risk of renal parenchymal scarring following childhood urinary tract infection. | Hussein A et al. | 25939993 | Paediatric individuals |
| 1 | Angiotensin-converting enzyme genotype is not a significant genetic risk factor for idiopathic nephrotic syndrome in Croatian children. | Batinic D et al. | 25997642 | Paediatric individuals |
| 1 | BRAF mutations in pediatric metanephric tumors. | Chami R et al. | 26014474 | Paediatric individuals |
| 1 | Expressions of mRNA for innate immunity-associated functional molecules in urinary sediment in immunoglobulin A nephropathy. | Tsuruga K et al. | 26058859 | Paediatric individuals |

| | | | | |
|---|---|---|---|---|
| 1 | Association of ACE and MDR1 Gene Polymorphisms with Steroid Resistance in Children with Idiopathic Nephrotic Syndrome. | Dhandapani MC et al. | 26154535 | Paediatric individuals |
| 1 | Inflammasome polymorphisms in juvenile systemic lupus erythematosus. | Pontillo A et al. | 26182076 | Paediatric individuals |
| 1 | Associations of the eNOS G894T gene polymorphism with target organ damage in children with newly diagnosed primary hypertension. | Sladowska-Kozlowska J et al. | 26227630 | Paediatric individuals |
| 1 | Paraoxnase1 Gene Polymorphism in Childhood Idiopathic Nephrotic Syndrome. | Al-Eisa AA et al. | 26780374 | Paediatric individuals |
| 1 | Coagulation, thrombophilia and patency of arteriovenous fistula in children undergoing haemodialysis compared with healthy volunteers: a prospective analysis. | Fadel FI et al. | 26829282 | Paediatric individuals |
| 1 | Components of the lectin pathway of complement activation in paediatric patients of intensive care units. | Swierzko AS et al. | 26850322 | Paediatric individuals |
| 1 | ACE serum level and I/D gene polymorphism in children with obstructive uropathies and other congenital anomalies of the kidney and urinary tract. | Kostadinova ES et al. | 27206329 | Paediatric individuals |
| 1 | Association study between matrix metalloproteinase-9 gene (MMP9) polymorphisms and the risk of Henoch-SchÃ¶nlein purpura in children. | Xu ED et al. | 27323137 | Paediatric individuals |
| 1 | Clinicopathological features and BRAF(V600E) mutations in patients with isolated hypothalamic-pituitary Langerhans cell histiocytosis. | Huo Z et al. | 27760550 | Paediatric individuals |
| 1 | BRAF exon 15 mutations in pediatric renal stromal tumors: prevalence in metanephric stromal tumors. | Marsden L et al. | 27769870 | Paediatric individuals |
| 1 | Angiotensin-converting enzyme insertion/deletion gene polymorphism in Egyptian children with systemic lupus erythematosus: a possible relation to proliferative nephritis. | Hammad A et al. | 27956582 | Paediatric individuals |
| 1 | Estimation of the relationship between the polymorphisms of selected genes: ACE, AGTR1, TGFÎ²1 and GNB3 with the occurrence of primary vesicoureteral reflux. | Zyczkowski M et al. | 27988909 | Paediatric individuals |
| 1 | Spectrum of mutations in Chinese children with steroid-resistant nephrotic syndrome. | Wang F et al. | 28204945 | Paediatric individuals |
| 1 | Lectin pathway factors in patients suffering from juvenile idiopathic arthritis. | Kasperkiewicz K et al. | 28405017 | Paediatric individuals |

| | | | | |
|---|---|---|---|---|
| 1 | Association of Endothelial Nitric Oxide Synthase Gene Polymorphism with Susceptibility and Nephritis Development of Henoch-SchÃ¶nlein Purpura in Chinese Han Children. | Wang A et al. | 28409662 | Paediatric individuals |
| 1 | Angiotensin-Converting Enzyme Gene Polymorphism in Children with Idiopathic Nephrotic Syndrome, Effect on Biopsy Findings. | Monajemzadeh M et al. | 28481137 | Paediatric individuals |
| 1 | Inducible nitric oxide synthase gene polymorphisms are associated with a risk of nephritis in Henoch-SchÃ¶nlein purpura children. | Jiang J et al. | 28593405 | Paediatric individuals |
| 1 | Inborn errors in RNA polymerase III underlie severe varicella zoster virus infections. | Ogunjimi B et al. | 28783042 | Paediatric individuals |
| 1 | Association of single-nucleotide polymorphism in the FKBP5 gene with response to steroids in pediatric patients with primary nephrotic syndromeâ€©. | Du N et al. | 28992850 | Paediatric individuals |
| 1 | Monogenic diabetes in overweight and obese youth diagnosed with type 2 diabetes: the TODAY clinical trial. | Kleinberger JW et al. | 29758564 | Paediatric individuals |
| 1 | Evaluation of Genetic Polymorphisms for Determining Steroid Response in Nephrotic Children. | Kara A et al. | 30143489 | Paediatric individuals |
| 1 | A randomized clinical trial of age and genotype-guided tacrolimus dosing after pediatric solid organ transplantation. | Min S et al. | 30178515 | Paediatric individuals |
| 1 | Pharmacogenetics of acute azathioprine toxicity: relationship to thiopurine methyltransferase genetic polymorphism. | Lennard L et al. | 2758725 | Pharmaceutical drug focus |
| 1 | Genetic association of 11 beta-hydroxysteroid dehydrogenase type 2 (HSD11B2) flanking microsatellites with essential hypertension in blacks. | Watson B Jr et al. | 8794836 | Pharmaceutical drug focus |
| 1 | Effect of deletion polymorphism of angiotensin converting enzyme gene on progression of diabetic nephropathy during inhibition of angiotensin converting enzyme: observational follow up study. | Parving HH et al. | 8806248 | Pharmaceutical drug focus |
| 1 | Effect of angiotensin-converting enzyme (ACE) gene polymorphism on progression of renal disease and the influence of ACE inhibition in IDDM patients: findings from the EUCLID Randomized Controlled Trial. EURODIAB Controlled Trial of Lisinopril in IDDM. | Penno G et al. | 9726242 | Pharmaceutical drug focus |
| 1 | Effects of erythropoietin, angiotensin II, and angiotensin-converting enzyme inhibitor on erythroid precursors in patients with posttransplantation erythrocytosis. | Glicklich D et al. | 10428268 | Pharmaceutical drug focus |

| | | | | |
|---|---|---|---|---|
| 1 | Nifedipine improves endothelial function in hypercholesterolemia, independently of an effect on blood pressure or plasma lipids. | Verhaar MC et al. | 10533616 | Pharmaceutical drug focus |
| 1 | Pharmacogenetic analysis of the effect of angiotensin-converting enzyme inhibitor on restenosis after percutaneous transluminal coronary angioplasty. | Okamura A et al. | 10535720 | Pharmaceutical drug focus |
| 1 | The norbornenyl moiety of cyclothiazide determines the preference for flip-flop variants of AMPA receptor subunits. | Kessler M et al. | 10854736 | Pharmaceutical drug focus |
| 1 | Randomized placebo-controlled trial of perindopril in normotensive, normoalbuminuric patients with type 1 diabetes mellitus. | Kvetny J et al. | 11181984 | Pharmaceutical drug focus |
| 1 | Human thiopurine S-methyltransferase activity in uremia and after renal transplantation. | Weyer N et al. | 11417444 | Pharmaceutical drug focus |
| 1 | Long-term renoprotective effects of losartan in diabetic nephropathy: interaction with ACE insertion/deletion genotype? | Andersen S et al. | 12716812 | Pharmaceutical drug focus |
| 1 | The influence of the ACE ( I/D) polymorphism on systemic and renal vascular responses to angiotensins in normotensive, normoalbuminuric Type 1 diabetes mellitus. | Luik PT et al. | 12856080 | Pharmaceutical drug focus |
| 1 | Venous response to nitroglycerin is enhanced in young, healthy carriers of the 825T allele of the G protein beta3 subunit gene (GNB3). | Mitchell A et al. | 14586390 | Pharmaceutical drug focus |
| 1 | Matrix metalloproteinase-1 gene polymorphism in renal transplant patients with and without gingival enlargement. | Kurzawski M et al. | 16945025 | Pharmaceutical drug focus |
| 1 | Enalapril and losartan affect lipid peroxidation in renal transplant recipients with renin-angiotensin system polymorphisms. | Rashtchizadeh N et al. | 17222813 | Pharmaceutical drug focus |
| 1 | Renin-angiotensin system polymorphisms and hemoglobin level in renal allografts: a comparative study between losartan and enalapril. | Noroozianavval M et al. | 17524880 | Pharmaceutical drug focus |
| 1 | Genetic analysis of fluvastatin response and dyslipidemia in renal transplant recipients. | Singer JB et al. | 17563401 | Pharmaceutical drug focus |
| 1 | Impact of the preintervention rate of renal function decline on outcome of renoprotective intervention. | Lely AT et al. | 18077786 | Pharmaceutical drug focus |
| 1 | CYP3A5 genotype is not associated with a higher risk of acute rejection in tacrolimus-treated renal transplant recipients. | Hesselink DA et al. | 18334918 | Pharmaceutical drug focus |
| 1 | Endothelial nitric oxide synthase gene polymorphisms and the renal hemodynamic response to L-arginine. | Cherney DZ et al. | 19037250 | Pharmaceutical drug focus |

| | | | | |
|---|---|---|---|---|
| 1 | Modulating effect of atorvastatin on paraoxonase 1 activity in type 2 diabetic Egyptian patients with or without nephropathy. | Abdin AA et al. | 19553142 | Pharmaceutical drug focus |
| 1 | CYP2C9 genotype and pharmacodynamic responses to losartan in patients with primary and secondary kidney diseases. | Joy MS et al. | 19669737 | Pharmaceutical drug focus |
| 1 | Matrix metalloproteinase-3 gene polymorphism in renal transplant patients with gingival overgrowth. | Drozdzik A et al. | 19778329 | Pharmaceutical drug focus |
| 1 | Presence of CYP2C9*3 allele increases risk for hypoglycemia in Type 2 diabetic patients treated with sulfonylureas. | Ragia G et al. | 19891554 | Pharmaceutical drug focus |
| 1 | Drug-resistant cytomegalovirus in transplant recipients: a French cohort study. | Hantz S et al. | 20961907 | Pharmaceutical drug focus |
| 1 | CYP3A5 genotype does not influence everolimus in vitro metabolism and clinical pharmacokinetics in renal transplant recipients. | Picard N et al. | 21389905 | Pharmaceutical drug focus |
| 1 | Cytochrome P450 polymorphisms and the response of lupus nephritis to cyclophosphamide therapy. | Winoto J et al. | 21543025 | Pharmaceutical drug focus |
| 1 | Exaggerated natriuresis during clamping of systemic NO supply in healthy young men. | Simonsen JA et al. | 21749320 | Pharmaceutical drug focus |
| 1 | In vivo CYP3A activity is significantly lower in cyclosporine-treated as compared with tacrolimus-treated renal allograft recipients. | de Jonge H et al. | 21753749 | Pharmaceutical drug focus |
| 1 | Lack of association between the Trp719Arg polymorphism in kinesin-like protein-6 and cardiovascular risk and efficacy of atorvastatin among subjects with diabetes on dialysis: the 4D study. | Hoffmann MM et al. | 21871624 | Pharmaceutical drug focus |
| 1 | Influence of NAT2 polymorphisms on sulfamethoxazole pharmacokinetics in renal transplant recipients. | Kagaya H et al. | 22106207 | Pharmaceutical drug focus |
| 1 | Comparing antihypertensive effect and plasma ciclosporin concentration between amlodipine and valsartan regimens in hypertensive renal transplant patients receiving ciclosporin therapy. | Cai J et al. | 22149319 | Pharmaceutical drug focus |
| 1 | Genetic and clinical determinants of early, acute calcineurin inhibitor-related nephrotoxicity: results from a kidney transplant consortium. | Jacobson PA et al. | 22334041 | Pharmaceutical drug focus |
| 1 | Risk of tacrolimus toxicity in CYP3A5 nonexpressors treated with intravenous nicardipine after kidney transplantation. | Hooper DK et al. | 22491658 | Pharmaceutical drug focus |
| 1 | CYP3A5 polymorphism effect on cyclosporine pharmacokinetics in living donor renal transplant recipients: analysis by population pharmacokinetics. | Song J et al. | 22947591 | Pharmaceutical drug focus |

| | | | | |
|---|---|---|---|---|
| 1 | Comparison of pharmacokinetics and pharmacogenetics of once- and twice-daily tacrolimus in the early stage after renal transplantation. | Niioka T et al. | 23073468 | Pharmaceutical drug focus |
| 1 | Impact of tacrolimus intraindividual variability and CYP3A5 genetic polymorphism on acute rejection in kidney transplantation. | Ro H et al. | 23149441 | Pharmaceutical drug focus |
| 1 | Endothelial nitric oxide synthase gene polymorphisms and renal responsiveness to RAS inhibition therapy in type 2 diabetic Asian Indians. | Cheema BS et al. | 23260854 | Pharmaceutical drug focus |
| 1 | Individualization of tacrolimus dosage basing on cytochrome P450 3A5 polymorphism--a prospective, randomized, controlled study. | Chen SY et al. | 23432535 | Pharmaceutical drug focus |
| 1 | Genetic polymorphisms of UGT1A8, UGT1A9 and HNF-1Î± and gastrointestinal symptoms in renal transplant recipients taking mycophenolic acid. | Vu D et al. | 23721685 | Pharmaceutical drug focus |
| 1 | CYP2C9*2 allele increases risk for hypoglycemia in POR*1/*1 type 2 diabetic patients treated with sulfonylureas. | Ragia G et al. | 24464600 | Pharmaceutical drug focus |
| 1 | Association of CYP3A4*18B and CYP3A5*3 polymorphism with cyclosporine-related liver injury in Chinese renal transplant recipients. | Xin HW et al. | 24691060 | Pharmaceutical drug focus |
| 1 | Genetic variance in ABCB1 and CYP3A5 does not contribute toward the development of chronic kidney disease after liver transplantation. | Tapirdamaz O et al. | 25014506 | Pharmaceutical drug focus |
| 1 | Associations of HSD11B1 polymorphisms with tacrolimus concentrations in Chinese renal transplant recipients with prednisone combined therapy. | Liu X et al. | 25587129 | Pharmaceutical drug focus |
| 3 | Impact of interaction of cigarette smoking with angiotensin-converting enzyme polymorphisms on end-stage renal disease risk in a Han Chinese population | Yang HY et al. | 23477970 | < 3 populations reported per SNP |
| 3 | Polymorphisms in the gene encoding angiotensin I converting enzyme 2 and diabetic nephropathy | Frojdo S et al. | 16211375 | < 3 populations reported per SNP |
| 3 | Investigation of ACE, ACE2 and AGTR1 genes for association with nephropathy in Type 1 diabetes mellitus. | Currie D et al. | 20854388 | < 3 populations reported per SNP |

**Supplementary Table S3c:** Excluded studies from the *AGT* search

*Exclusion stage relates to the stage at which the article was removed as shown in the flow diagram (Supplementary Figure S1c)

| Exclusion Stage | Title | Authors | Pubmed ID or WoS ID if Pubmed ID not available | Reason |
|---|---|---|---|---|
| 1 | Identification of potential candidate genes for hypertensive nephropathy based on gene expression profile | Chen Z et al. | 27756246 | Gene expression based study |
| 1 | Defining Uremic Arterial Functional Abnormalities in Patients Recently Started on Haemodialysis: Combined In Vivo and Ex Vivo Assessment | Abushufa AM et al. | 25546407 | No data for AGT |
| 1 | Angiotensin I Converting Enzyme Gene Polymorphism in Type 2 Diabetes Mellitus with Nephropathy in Saudi Population | Alharbi SA et al. | WOS:000415063100003 | No data for AGT |
| 1 | Clinical impact of an angiotensin I-converting enzyme insertion/deletion and kinin B2 receptor +9/-9 polymorphisms in the prognosis of renal transplantation | Amorim CEN et al. | 23362199 | No data for AGT |
| 1 | Transforming growth factor beta and progression of renal disease | August P et al. | WOS:000185812000016 | No data for AGT |
| 1 | Associations of fractalkine receptor (CX3CR1) and CCR5 gene variants with hypertension, diabetes and atherosclerosis in chronic renal failure patients undergoing hemodialysis | Bagci B et al. | 27118566 | No data for AGT |
| 1 | Effect of ACE and AT-2 inhibitors on mortality and progression to microalbuminuria in a nested case-control study of diabetic nephropathy in diabetes mellitus type 2: results from the GENDIAN study. | Boger CA et al. | 16961167 | No data for AGT |
| 1 | The ras responsive transcription factor RREB1 is a novel candidate gene for type 2 diabetes associated end-stage kidney disease | Bonomo JA et al. | 25027322 | No data for AGT |
| 1 | Angiotensin II type 1 receptor gene polymorphism in end-stage renal disease | Buraczyńska M et al. | 12187084 | No data for AGT |

| | | | | |
|---|---|---|---|---|
| 1 | Endothelial nitric oxide synthase gene polymorphisms and renal responsiveness to RAS inhibition therapy in type 2 diabetic Asian Indians | Cheema BS et al. | 23260854 | No data for AGT |
| 1 | Polymorphism of angiotensin II receptor gene and microangiopathies in patients with insulin-dependent diabetes mellitus | Chistyakov DA et al. | WOS:000084973600016 | No data for AGT |
| 1 | Association of renin-angiotensin and endothelial nitric oxide synthase gene polymorphisms with blood pressure progression and incident hypertension: prospective cohort study. | Conen D et al. | 18698212 | No data for AGT |
| 1 | Serological and genetic factors in early recurrence of IgA nephropathy after renal transplantation | Coppo R et al. | 17988266 | No data for AGT |
| 1 | Plasma renin and prorenin and renin gene variation in patients with insulin-dependent diabetes mellitus and nephropathy | Deinum J et al. | 10462269 | No data for AGT |
| 1 | Insertion/deletion polymorphism of the angiotensin-converting enzyme gene in normalcy and among diabetics with vascular complications | Demurov LM et al. | WOS:A1997XE98500008 | No data for AGT |
| 1 | Haplotype analysis of NAD(P)H oxidase p22 phox polymorphisms in end-stage renal disease | Doi K et al. | 16215641 | No data for AGT |
| 1 | Genetic predisposition to diabetic nephropathy. Evidence for a role of the angiotensin I--converting enzyme gene | Doria A et al. | 7909524 | No data for AGT |
| 1 | Synergistic effect of angiotensin II type 1 receptor genotype and poor glycaemic control on risk of nephropathy in IDDM | Doria A et al. | 9389421 | No data for AGT |
| 1 | Analysis of three genetic markers in IgA nephropathy patients from a single region | Drouet M et al. | 12005241 | No data for AGT |
| 1 | Angiotensin II type 1 receptor (A1166C) gene polymorphism in Egyptian adult hemodialysis patients | El-Banawy H et al. | WOS:000365886400009 | No data for AGT |
| 1 | Angiotensin-I converting enzyme gene polymorphism in Turkish type 2 diabetic patients | Ergen HA et al. | WOS:000223974500007 | No data for AGT |

| | | | | |
|---|---|---|---|---|
| 1 | ACE gene polymorphism and serum ACE activity in Iranians type II diabetic patients with macroalbuminuria | Felehgari V et al. | 20830509 | No data for AGT |
| 1 | DNA polymorphisms in the ACE gene, serum ACE activity and the risk of nephropathy in insulin-dependent diabetes mellitus | Freire MBS et al. | 9794558 | No data for AGT |
| 1 | Polymorphisms in the gene encoding angiotensin I converting enzyme 2 and diabetic nephropathy | Frojdo S et al. | 16211375 | No data for AGT |
| 1 | Association of fibronectin Msp iv polymorphism and diabetic nephropathy susceptibility in Chinese Han population | Gao JX et al. | 26045844 | No data for AGT |
| 1 | Effects of erythropoietin, angiotensin II, and angiotensin-converting enzyme inhibitor on erythroid precursors in patients with posttransplantation erythrocytosis | Glicklich D et al. | 10428268 | No data for AGT |
| 1 | Association of Angiotensin converting Enzyme (ACE) gene polymorphism and diabetic nephropathy | Golmohamadi T et al. | WOS:000242619000003 | No data for AGT |
| 1 | Angiotensin I-converting enzyme gene polymorphisms: Relationship to nephropathy in patients with non-insulin dependent diabetes mellitus | Grzeszczak W et al. | 9727375 | No data for AGT |
| 1 | Susceptibility and progression of end stage renal disease are not associated with angiotensin II type 1 receptor gene polymorphism | Hanna MOF et al. | 25316403 | No data for AGT |
| 1 | Polymorphism of the angiotensin I-converting enzyme gene in diabetic nephropathy in type II diabetic patients with proliferative retinopathy | Hanyu O et al. | 9509566 | No data for AGT |
| 1 | Angiotensin Converting Enzyme Insertion/Deletion gene polymorphism and genomic sequence in Diabetic Nephropathy | Haque SF et al. | WOS:000300381000004 | No data for AGT |
| 1 | The angiotensin I-converting enzyme (ACE) locus is strongly associated with age and duration of diabetes in patients with type I diabetes | Hibberd ML et al. | 9025006 | No data for AGT |
| 1 | Role of transforming growth factor beta-1 gene polymorphisms in the development of chronic allograft nephropathy in renal transplant recipients | Inigo P et al. | 14558330 | No data for AGT |

| | | | | |
|---|---|---|---|---|
| 1 | Neuropeptide YY1 receptor polymorphism as a prognostic predictor in Japanese patients with IgA nephropathy | Ito H et al. | 10363627 | No data for AGT |
| 1 | Carnosine as a protective factor in diabetic nephropathy - Association with a leucine repeat of the carnosinase gene CNDP1 | Janssen B et al. | 16046297 | No data for AGT |
| 1 | Impact of Polymorphisms of the Genes Encoding Angiotensin II-Forming Enzymes on the Progression of IgA Nephropathy | Jung ES et al. | 21150220 | No data for AGT |
| 1 | Proteomic analysis of alpha-1-antitrypsin in immunoglobulin A nephropathy | Kwak NJ et al. | 21136694 | No data for AGT |
| 1 | Renal perfusion and the renal hemodynamic response to blocking the renin system in diabetes - Are the forces leading to vasodilation and vasoconstriction linked? | Lansang MC et al. | 12086929 | No data for AGT |
| 1 | Polymorphism in IgA nephropathy | Liu ZH et al. | WOS:A1997WW80600013 | No data for AGT |
| 1 | The angiotensin-I converting enzyme gene I/D variation contributes to end-stage renal disease risk in Chinese patients with type 2 diabetes receiving hemodialysis | Lu M et al. | 27633502 | No data for AGT |
| 1 | The influence of three endothelin-1 polymorphisms on the progression of IgA nephropathy | Maixnerova D et al. | 17328840 | No data for AGT |
| 1 | The influence of two megsin polymorphisms on the progression of IgA nephropathy | Maixnerova D et al. | 18498720 | No data for AGT |
| 1 | Relationships Between Angiotensin-I Converting-Enzyme Gene Polymorphism, Plasma-Levels, And Diabetic Retinal And Renal Complications | Marre M et al. | 8314010 | No data for AGT |
| 1 | Hereditary factors in the development of diabetic renal disease | Marre M et al. | 10922971 | No data for AGT |
| 1 | A rare haplotype of the vitamin D receptor gene is protective against diabetic nephropathy | Martin RJL et al. | 19783860 | No data for AGT |
| 1 | Mutational Analysis of Agxt in Tunisian Population with Primary Hyperoxaluria Type 1. | M'dimegh S et al. | 27935012 | No data for AGT |
| 1 | Kinin-dependent hypersensitivity reactions in hemodialysis: metabolic and genetic factors. | Molinaro G et al. | 17003818 | No data for AGT |

| | | | | |
|---|---|---|---|---|
| 1 | A polymorphism in the angiotensin II type 1 receptor gene has different effects on the risk of diabetic nephropathy in men and women | Mollsten A et al. | 21316998 | No data for AGT |
| 1 | Relationship of angiotensin-converting enzyme gene polymorphism with nephropathy associated with Type 2 diabetes mellitus in Asian Indians | Movva S et al. | 17616353 | No data for AGT |
| 1 | Relationship of Serum Klotho Level With ACE Gene Polymorphism in Stable Kidney Allograft Recipients | Nahandi MZ et al. | 28270648 | No data for AGT |
| 1 | The effect of angiotensin receptor blockade (ARB) on the regression of left ventricular hypertrophy in hemodialysis patients: comparison between patients with D allele and non-D allele (ACE gene polymorphism) | Nakayama M et al. | 16312263 | No data for AGT |
| 1 | Different Mechanisms for the Progression of CKD with ACE Gene Polymorphisms | Nakayama Y et al. | 19293592 | No data for AGT |
| 1 | A disease haplotype for advanced nephropathy in type 2 diabetes at the ACE locus | Ng DPK et al. | 16936219 | No data for AGT |
| 1 | Relationships between angiotensin I converting enzyme gene polymorphism and renal complications in Korean IDDM patients. | Oh TG et al. | 8854649 | No data for AGT |
| 1 | alpha(1)-antitrypsin gene polymorphisms are not associated with renal arterial fibromuscular dysplasia | Perdu J et al. | 16531799 | No data for AGT |
| 1 | The frequency of factor V Leiden mutation, ACE gene polymorphism, serum ACE activity and response to ACE inhibitor and angiotensin II receptor antagonist drugs in Iranians type II diabetic patients with microalbuminuria | Rahimi Z et al. | 20853144 | No data for AGT |
| 1 | AGTR1 rs5186 variants in patients with type 2 diabetes mellitus and nephropathy | Razi F et al. | WOS:000419720600009 | No data for AGT |
| 1 | Angiotensin-converting enzyme polymorphism in patients with terminal renal failure | Schmidt A et al. | 8785402 | No data for AGT |

| | | | | |
|---|---|---|---|---|
| 1 | No association of converting enzyme insertion/deletion polymorphism with immunoglobulin A glomerulonephritis | Schmidt S et al. | 7485124 | No data for AGT |
| 1 | Angiotensin I converting enzyme gene polymorphism and diabetic nephropathy in type II diabetes | Schmidt S et al. | 9269698 | No data for AGT |
| 1 | ACACÎ² gene (rs2268388) and AGTR1 gene (rs5186) polymorphism and the risk of nephropathy in Asian Indian patients with type 2 diabetes. | Shah VN et al. | 23081748 | No data for AGT |
| 1 | Relations between eNOS Glu298Asp polymorphism and progression of diabetic nephropathy | Shin Shin Y et al. | 15331206 | No data for AGT |
| 1 | Angiotensin I-converting enzyme genotype significantly affects progression of IgA glomerulonephritis in an Italian population | Stratta P et al. | 10352195 | No data for AGT |
| 1 | The association of LOX-1 rs1050283 polymorphism with renal hypertension susceptibility in a Chinese population | Sun YC et al. | WOS:000395739000087 | No data for AGT |
| 1 | Lack Of Relationship Between An Insertion Deletion Polymorphism In The Angiotensin I-Converting Enzyme Gene And Diabetic Nephropathy And Proliferative Retinopathy In Iddm Patients | Tarnow L et al. | 7729604 | No data for AGT |
| 1 | Angiotensin-II type 1 receptor gene polymorphism and diabetic microangiopathy. | Tarnow L et al. | 8671962 | No data for AGT |
| 1 | High prevalence of ACE DD genotype among north Indian end stage renal disease patients. | Tripathi G et al. | 17042963 | No data for AGT |
| 1 | Angiotensin-converting enzyme gene polymorphism and vascular manifestations in Korean patients with SLE | Uhm WS et al. | 12043886 | No data for AGT |
| 1 | Increased expression of monocytic angiotensin-converting enzyme in dialysis patients with cardiovascular disease | Ulrich C et al. | 16476718 | No data for AGT |
| 1 | Synergistic expression of angiotensin-converting enzyme (ACE) and ACE2 in human renal tissue and confounding effects of hypertension on the ACE to ACE2 ratio | Wakahara S et al. | 17303661 | No data for AGT |
| 1 | Angiotensin-converting enzyme inhibitor versus angiotensin 2 receptor antagonist therapy and the influence of angiotensin-converting enzyme gene polymorphism in IgA nephritis. | Woo KT et al. | 18536822 | No data for AGT |

| | | | | |
|---|---|---|---|---|
| 1 | Involvement of platelet-derived growth factor and histocompatibility of DRB 1 in chronic renal allograft nephropathy | Yamada K et al. | 11349729 | No data for AGT |
| 1 | Angiotensin converting enzyme gene polymorphism and development of post-transplant erythrocytosis. | Yildiz A et al. | 12832741 | No data for AGT |
| 1 | Angiotensin II type 2 receptor gene is not responsible for familial vesicoureteral reflux | Yoneda A et al. | 12187255 | No data for AGT |
| 1 | Polymorphism Of The Angiotensin-Converting Enzyme Gene And Clinical Aspects Of Iga Nephropathy | Yorioka T et al. | 8529313 | No data for AGT |
| 1 | Interleukin-4 (IL4)-590C/T (rs2243250) gene polymorphism is not associated with diabetic nephropathy (DN) in Caucasians with type 2 diabetes mellitus (T2DM) | Zavrsnik M et al. | 29514038 | No data for AGT |
| 1 | Hemodynamic Parameters During Normal And Hypertensive Pregnancy In Rats: Evaluation Of Renal Salt And Water Transporters | Abreu N et al. | 18293204 | Non-human study |
| 1 | DPP-4 Inhibition on Top of Angiotensin Receptor Blockade Offers a New Therapeutic Approach for Diabetic Nephropathy | Alter ML et al. | 23171828 | Non-human study |
| 1 | Long-Term Angiotensin II Receptor Blockade Limits Hypertension, Aortic Dysfunction, and Structural Remodeling in a Rat Model of Chronic Kidney Disease | Ameer OZ et al. | 27880955 | Non-human study |
| 1 | Effects of Diets with Different Proportions of Protein/Carbohydrate on Retinal Manifestations in db Mice | Arimura E et al. | 29475908 | Non-human study |
| 1 | Identification of Cathepsin L as a Potential Sex-Specific Biomarker for Renal Damage | Bauer Y et al. | 21357272 | Non-human study |
| 1 | Blood pressure (BP) and renal vasoconstrictor responses to acute blockade of nitric oxide: persistence of renal vasoconstriction despite normalization of BP with either verapamil or sodium nitroprusside. | Baylis C et al. | 7562480 | Non-human study |
| 1 | Specific pregnancy-induced angiotensin II type-1 receptor expression in ovine uterine artery does not involve formation of alternate splice variants or alternate promoter usage | Bird IM et al. | 9687288 | Non-human study |

| | | | | |
|---|---|---|---|---|
| 1 | N-terminal residues control proteasomal degradation of RGS2, RGS4, and RGS5 in human embryonic kidney 293 cells | Bodenstein J et al. | 17220356 | Non-human study |
| 1 | Angiotensinogen concentrations and renin clearance : implications for blood pressure regulation. | Bohlender J et al. | 10720595 | Non-human study |
| 1 | Blood pressure and renin-angiotensin system resetting in transgenic rats with elevated plasma Val(5)-angiotensinogen | Bohlender J et al. | 22728903 | Non-human study |
| 1 | CCN1 expression in interleukin-6 deficient mouse kidney in experimental model of heart failure | Bonda TA et al. | 23690222 | Non-human study |
| 1 | Advanced Glycated End-Products Affect HIF-Transcriptional Activity in Renal Cells | Bondeva T et al. | 24030251 | Non-human study |
| 1 | Lack of in vivo function of osteopontin in experimental anti-GBM nephritis | Bonvini JM et al. | 10966489 | Non-human study |
| 1 | Hypoxia/Reoxygenation of Rat Renal Arteries Impairs Vasorelaxation via Modulation of Endothelium-Independent sGC/cGMP/PKG Signaling | Braun D et al. | 29773995 | Non-human study |
| 1 | Angiotensin II mesenteric and renal vasoregulation: Dissimilar modulatory effects with nitroprusside | Broome M et al. | 11065204 | Non-human study |
| 1 | N-domain angiotensin I-converting enzyme expression in renal artery of Wistar, Wistar Kyoto, and spontaneously hypertensive rats | Bueno V et al. | 15194348 | Non-human study |
| 1 | Organizational diversity among distinct glycoprotein endoplasmic reticulum-associated degradation programs | Cabral CM et al. | 12181335 | Non-human study |
| 1 | Role of NOX2 in the regulation of afferent arteriole responsiveness | Carlstrom M et al. | 18987286 | Non-human study |
| 1 | Adrenomedullin gene expression differences in mice do not affect blood pressure but modulate hypertension-induced pathology in males | Caron K et al. | 17360661 | Non-human study |
| 1 | Appropriate regulation of renin and blood pressure in 45-kb human renin/human angiotensinogen transgenic mice. | Catanzaro DF et al. | 9931123 | Non-human study |
| 1 | Mice lacking endothelial ACE - Normal blood pressure with elevated angiotensin | Cole JM et al. | 12574101 | Non-human study |

| | | | | |
|---|---|---|---|---|
| 1 | Natriuretic peptides buffer renin-dependent hypertension | Demerath T et al. | 24717731 | Non-human study |
| 1 | Renin-angiotensin system transgenic mouse model recapitulates pathophysiology similar to human preeclampsia with renal injury that may be mediated through VEGF. | Denney JM et al. | 27927648 | Non-human study |
| 1 | Mycophenolate mofetil prevents cerebrovascular injury in stroke-prone spontaneously hypertensive rats | Dhande IS et al. | 28011882 | Non-human study |
| 1 | Inhibition of proximal tubular fluid absorption by nitric oxide and atrial natriuretic peptide in rat kidney | Eitle E et al. | 9575805 | Non-human study |
| 1 | Complex interactions of NO/cGMP/PKG systems on Ca2+ signaling in afferent arteriolar vascular smooth muscle | Fellner SK et al. | 19880669 | Non-human study |
| 1 | Cardiac angiotensin-(1-12) expression and systemic hypertension in rats expressing the human angiotensinogen gene. | Ferrario CM et al. | 26873967 | Non-human study |
| 1 | Attenuation of accelerated renal cystogenesis in Pkd1 mice by renin-angiotensin system blockade | Fitzgibbon WR et al. | 29021226 | Non-human study |
| 1 | Renal cyst growth is the main determinant for hypertension and concentrating deficit in Pkd1-deficient mice | Fonseca JM et al. | 24429399 | Non-human study |
| 1 | Acute elevations in salt intake and reduced renal mass hypertension compromise arteriolar dilation in rat cremaster muscle | Frisbee JC et al. | 10329253 | Non-human study |
| 1 | Contribution of cytochrome P-450 omega-hydroxylase to altered arteriolar reactivity with high-salt diet and hypertension | Frisbee JC et al. | 10775129 | Non-human study |
| 1 | Adenosine A(1) receptor-dependent and independent pathways in modulating renal vascular responses to angiotensin II | Gao X et al. | 25251152 | Non-human study |
| 1 | Connexin 43 is not essential for the control of renin synthesis and secretion | Gerl M et al. | 24062052 | Non-human study |
| 1 | Metformin prevents the impairment of endothelium-dependent vascular relaxation induced by high glucose challenge in rabbit isolated perfused kidneys | Gomes MB et al. | 16133490 | Non-human study |
| 1 | Effect of the angiotensinogen genotype on experimental hypertension in mice | Handtrack C et al. | 17333097 | Non-human study |

| | | | | |
|---|---|---|---|---|
| 1 | Cytoprotective effects of nitrates in a cellular model of hydronephrosis | Hegarty NJ et al. | 12081565 | Non-human study |
| 1 | The genetic deletion of Mas abolishes salt induced hypertension in mice | Heringer-Walther S et al. | 22652430 | Non-human study |
| 1 | Pressor and sympathoexcitatory effects of nitric oxide in the rostral ventrolateral medulla | Hirooka Y et al. | 8934360 | Non-human study |
| 1 | An essential role of angiotensin II receptor type 1a in recipient kidney, not in transplanted peripheral blood leukocytes, in progressive immune-mediated renal injury | Hisada Y et al. | 11555672 | Non-human study |
| 1 | The angiotensin type II receptor tonically inhibits angiotensin-converting enzyme in AT2 null mutant mice | Hunley TE et al. | 10652034 | Non-human study |
| 1 | NOS1-dependent negative feedback regulation of the epithelial sodium channel in the collecting duct | Hyndman KA et al. | 25391901 | Non-human study |
| 1 | Collecting Duct Nitric Oxide Synthase 1 beta Activation Maintains Sodium Homeostasis During High Sodium Intake Through Suppression of Aldosterone and Renal Angiotensin II Pathways | Hyndman KA et al. | 29066445 | Non-human study |
| 1 | Pathophysiological roles of adrenomedullin-RAMP2 system in acute and chronic cerebral ischemia | Igarashi K et al. | 25252154 | Non-human study |
| 1 | Role of A(1) receptors in renal sympathetic neurotransmission in the mouse kidney | Jackson EK et al. | 22874760 | Non-human study |
| 1 | mPGES-1 deletion potentiates urine concentrating capability after water deprivation | Jia ZJ et al. | 22237797 | Non-human study |
| 1 | Transfer of a salt-resistant renin allele raises blood pressure in Dahl salt-sensitive rats | Jiang J et al. | 9040448 | Non-human study |
| 1 | Angiotensin-converting enzyme inhibition attenuates the progression of rat hepatic fibrosis | Jonsson JR et al. | 11438504 | Non-human study |
| 1 | Rat Ace allele variation determines susceptibility to AngII-induced renal damage | Kamilic J et al. | 21788250 | Non-human study |
| 1 | Genetic-Control Of Blood-Pressure And The Angiotensinogen Locus | Kim HS et al. | 7708716 | Non-human study |

| | | | | |
|---|---|---|---|---|
| 1 | Regulation of renin secretion and expression in mice deficient in ss 1-and ss 2-adrenergic receptors | Kim SM et al. | 17515456 | Non-human study |
| 1 | Low Blood Pressure in Endothelial Cell-Specific Endothelin 1 Knockout Mice | Kisanuki YY et al. | 20516397 | Non-human study |
| 1 | Lack of an effect of collecting duct-specific deletion of adenylyl cyclase 3 on renal Na+ and water excretion or arterial pressure | Kittikulsuth W et al. | 24431204 | Non-human study |
| 1 | Effect of the plasminogen-plasmin system on hypertensive renal and cardiac damage | Knier B et al. | 21610512 | Non-human study |
| 1 | Axl mediates vascular remodeling induced by deoxycorticosterone acetate-salt hypertension | Korshunov VA et al. | 17923589 | Non-human study |
| 1 | Reciprocal expression of connexin 40 and 45 during phenotypical changes in renin-secreting cells | Kurt B et al. | 21209011 | Non-human study |
| 1 | Stimulation of renin secretion by NO donors is related to the cAMP pathway | Kurtz A et al. | 9575895 | Non-human study |
| 1 | The angiotensin II receptor blocker candesartan improves survival and mesenteric perfusion in an acute porcine endotoxin model | Laesser M et al. | 14995942 | Non-human study |
| 1 | Physiological impact of increased expression of the AT(1) angiotensin receptor | Le TH et al. | 12963678 | Non-human study |
| 1 | Nitric oxide reduces the molecular activity of Na+,K+-ATPase in opossum kidney cells | Liang MY et al. | 10432402 | Non-human study |
| 1 | Regulation of sympathetic nerve activity in heart failure - A role for nitric oxide and angiotensin II | Liu JL et al. | 10066676 | Non-human study |
| 1 | Overexpression of cytochrome P450 4F2 in mice increases 20-hydroxyeicosatetraenoic acid production and arterial blood pressure | Liu XL et al. | 19279555 | Non-human study |
| 1 | Sodium-Nitroprusside Increases Glomerular Capillary Hydraulic Conductivity In Isolated Rat Glomeruli | Lovell HB et al. | 7706899 | Non-human study |
| 1 | Macula Densa Nitric Oxide Synthase 1 beta Protects against Salt-Sensitive Hypertension | Lu Y et al. | 26647426 | Non-human study |
| 1 | Correction of an enzyme trafficking defect in hereditary kidney stone disease in vitro | Lumb MJ et al. | 12737622 | Non-human study |

| | | | | |
|---|---|---|---|---|
| 1 | Development and Analysis of Alpha 1-Antitrypsin Neoglycoproteins: The Impact of Additional N-Glycosylation Sites on Serum Half-Life | Lusch A et al. | 23668542 | Non-human study |
| 1 | Connexin 40 is dispensable for vascular renin cell recruitment but is indispensable for vascular baroreceptor control of renin secretion | Machura K et al. | 25241776 | Non-human study |
| 1 | Angiotensin receptor-binding protein ATRAP/Agtrap inhibits metabolic dysfunction with visceral obesity. | Maeda A et al. | 23902639 | Non-human study |
| 1 | Effect of sodium nitroprusside on norepinephrine overflow and antidiuresis induced by stimulation of renal nerves in anesthetized dogs | Maekawa H et al. | 8720419 | Non-human study |
| 1 | Hypertension in unilaterally nephrectomized rats induced by single-kidney transfection with angiotensinogen cDNA | Marley WS et al. | 10567853 | Non-human study |
| 1 | A novel rodent model of pregnancy complications associated with genetically determined angiotensin-converting enzyme (ACE) activity | Mata-Greenwood E et al. | 29360395 | Non-human study |
| 1 | Chronic regulation of arterial blood pressure by ANP: role of endogenous vasoactive endothelial factors | Melo LG et al. | 9815091 | Non-human study |
| 1 | Chronic hypertension and altered baroreflex responses in transgenic mice containing the human renin and human angiotensinogen genes | Merrill DC et al. | 8613528 | Non-human study |
| 1 | Role of endothelium-derived relaxing factors in the renal response to vasoactive agents in hypothyroid rats | Moreno JM et al. | 12657567 | Non-human study |
| 1 | Hypervolemia of pregnancy is not maintained in mice chronically overexpressing angiotensinogen | Morgan TK et al. | 16796982 | Non-human study |
| 1 | Vascular angiotensin-converting enzyme expression regulates local angiotensin II | Muller DN et al. | 9039087 | Non-human study |
| 1 | AKAP150 is required for stuttering persistent Ca2+ sparklets and angiotensin II-induced hypertension | Navedo MF et al. | 18174462 | Non-human study |
| 1 | Angiotensin-Ii Enhances Norepinephrine Spillover During Sympathetic Activation In Conscious Rabbits | Noshiro T et al. | WOS:A1994NP99600022 | Non-human study |

| | | | | |
|---|---|---|---|---|
| 1 | Effects of potassium adaptation on blood pressure and pressor responses in normotensive and renal hypertensive Wistar rats | Omogbai EL et al. | 15834453 | Non-human study |
| 1 | Altered regulation of renal interstitial hydrostatic pressure and the renal renin-angiotensin system in the absence of atrial natriuretic peptide | O'Tierney PF et al. | 18192845 | Non-human study |
| 1 | Effects of Stimulation of Soluble Guanylate Cyclase on Diabetic Nephropathy in Diabetic eNOS Knockout Mice on Top of Angiotensin II Receptor Blockade | Ott IM et al. | 22900035 | Non-human study |
| 1 | Nitric oxide modulates angiotensin II- and norepinephrine-dependent vasoconstriction in rat kidney | Parekh N et al. | 8780230 | Non-human study |
| 1 | Gene Trapping Uncovers Sex-Specific Mechanisms for Upstream Stimulatory Factors 1 and 2 in Angiotensinogen Expression | Park S et al. | 22547438 | Non-human study |
| 1 | Hyperplastic Growth Of Aortic Smooth-Muscle Cells In Renovascular Hypertensive Rabbits Is Characterized By The Expansion Of An Immature Cell Phenotype | Pauletto P et al. | 8156626 | Non-human study |
| 1 | Mechanical Basis of Osmosensory Transduction in Magnocellular Neurosecretory Neurones of the Rat Supraoptic Nucleus | Prager-Khoutorsky M et al. | 25712904 | Non-human study |
| 1 | Calcium/Calmodulin-Dependent Kinase II Inhibition in Smooth Muscle Reduces Angiotensin II-Induced Hypertension by Controlling Aortic Remodeling and Baroreceptor Function | Prasad AM et al. | 26077587 | Non-human study |
| 1 | Endothelium-Dependent Control Of Vascular Tone In The Rabbit Kidney After Ischemia And Reperfusion | Pruneau D et al. | 8453977 | Non-human study |
| 1 | Angiotensin II stimulates the synthesis and secretion of vascular permeability factor vascular endothelial growth factor in human mesangial cells | Pupilli C et al. | 10215323 | Non-human study |
| 1 | Loss of Notch3 Signaling in Vascular Smooth Muscle Cells Promotes Severe Heart Failure Upon Hypertension | Ragot H et al. | 27296994 | Non-human study |
| 1 | Knockdown of parathyroid hormone related protein in smooth muscle cells alters renal hemodynamics but not blood pressure | Raison D et al. | 23720345 | Non-human study |

| | | | | |
|---|---|---|---|---|
| 1 | Nephron-specific deletion of the prorenin receptor causes a urine concentration defect | Ramkumar N et al. | 25995108 | Non-human study |
| 1 | Possible role for nephron-derived angiotensinogen in angiotensin-II dependent hypertension | Ramkumar N et al. | WOS:000380233800013 | Non-human study |
| 1 | 20-Hydroxyeicosatetraenoic Acid (HETE)-dependent Hypertension in Human Cytochrome P450 (CYP) 4A11 Transgenic Mice NORMALIZATION OF BLOOD PRESSURE BY SODIUM RESTRICTION, HYDROCHLOROTHIAZIDE, OR BLOCKADE OF THE TYPE 1 ANGIOTENSIN II RECEPTOR | Savas S et al. | WOS:000380826700046 | Non-human study |
| 1 | Angiotensin converting enzyme (ACE) gene expression in experimentally induced liver cirrhosis in rats | Shahid SM et al. | 24035938 | Non-human study |
| 1 | Strain differences in angiotensin-converting enzyme and angiotensin II type I receptor expression. Possible implications for experimental chronic renal transplant failure | Smit-van Oosten A et al. | 11984748 | Non-human study |
| 1 | Association of a novel polymorphism in the bovine PPARGC1A gene with growth, slaughter and meat quality traits in Brangus steers | Soria LA et al. | 19665052 | Non-human study |
| 1 | Angiotensin II Type 1A Receptors in Vascular Smooth Muscle Cells Do Not Influence Aortic Remodeling in Hypertension | Sparks MA et al. | 21242463 | Non-human study |
| 1 | Thromboxane Receptors in Smooth Muscle Promote Hypertension, Vascular Remodeling, and Sudden Death | Sparks MA et al. | 23150508 | Non-human study |
| 1 | Losartan and Sodium Nitroprusside Effectively Protect against Renal Impairments after Ischemia and Reperfusion in Rats | Srisawat U et al. | 25947921 | Non-human study |
| 1 | Natriuretic Peptide Receptor Guanylyl Cyclase-A in Podocytes is Renoprotective but Dispensable for Physiologic Renal Function | Staffel J et al. | 27153922 | Non-human study |
| 1 | Co-operation between particulate and soluble guanylyl cyclase systems in the rat renal glomeruli | Stepinski J et al. | 11016869 | Non-human study |
| 1 | Renal angiotensin converting enzyme promotes renal damage during ureteral obstruction | Stoneking BJ et al. | 9719278 | Non-human study |
| 1 | Mediation of tubuloglomerular feedback by adenosine: Evidence from mice lacking adenosine 1 receptors | Sun DQ et al. | 11504952 | Non-human study |

| | | | | |
|---|---|---|---|---|
| 1 | Cross-species transcriptomic analysis elucidates constitutive aryl hydrocarbon receptor activity | Sun RX et al. | 25467400 | Non-human study |
| 1 | Blood Pressure Control by a Secreted FGFBP1 (Fibroblast Growth Factor-Binding Protein). | Tassi E et al. | 29158353 | Non-human study |
| 1 | Regulation of Na+/K+-ATPase activity by nitric oxide in the kidney and gill of the brown trout (Salmo trutta) | Tipsmark CK et al. | 12654889 | Non-human study |
| 1 | Mechanisms of Renal. Control of Potassium Homeostasis in Complete Aldosterone Deficiency | Todkar A et al. | 25071088 | Non-human study |
| 1 | Functional genetic variation in aminopeptidase A (ENPEP): Lack of clear association with focal and segmental glomerulosclerosis (FSGS) | Tonna S et al. | 18206321 | Non-human study |
| 1 | Angiotensin II-dependent chronic hypertension and cardiac hypertrophy are unaffected by gp91phox-containing NADPH oxidase | Touyz RM et al. | 15753233 | Non-human study |
| 1 | Increased availability of nitric oxide leads to enhanced nitric oxide dependency of tubuloglomerular feedback in the contralateral kidney of rats with2-kidney, 1-clip Goldblatt hypertension | Turkstra E et al. | 10523346 | Non-human study |
| 1 | Cardiac phenotype and angiotensin II levels in AT(1a), AT(1b), and AT(2) receptor single, double, and triple knockouts | van Esch JHM et al. | 20071356 | Non-human study |
| 1 | Blood pressure and renal hemodynamic responses to acute angiotensin II infusion are enhanced in a female mouse model of systemic lupus erythematosus | Venegas-Pont M et al. | 21900645 | Non-human study |
| 1 | Compensatory up-regulation of angiotensin II subtype 1 receptors in alpha ENaC knockout heterozygous mice | Wang Q et al. | 11380824 | Non-human study |
| 1 | Inhibition of Nitric Oxide Synthase 1 Induces Salt-Sensitive Hypertension in Nitric Oxide Synthase 1 alpha Knockout and Wild-Type Mice | Wang XM et al. | 26883268 | Non-human study |
| 1 | Nebivolol treatment improves resistant arterial function and reduces ventricular hypertrophy and angiotensin II in spontaneously hypertension rats | Wang Y et al. | 23263161 | Non-human study |

| | | | | |
|---|---|---|---|---|
| 1 | Human GRK4 gamma(142V) Variant Promotes Angiotensin II Type I Receptor-Mediated Hypertension via Renal Histone Deacetylase Type 1 Inhibition | Wang Z et al. | 26667412 | Non-human study |
| 1 | Role of neutral endopeptidase 24.11 in AV fistular rat model of heart failure | Wegner M et al. | 8759244 | Non-human study |
| 1 | Lysine-Specific Demethylase 1: An Epigenetic Regulator of Salt-Sensitive Hypertension | Williams JS et al. | 22534796 | Non-human study |
| 1 | Role of angiotensin-converting enzyme (ACE and ACE2) imbalance on tourniquet-induced remote kidney injury in a mouse hindlimb ischemia-reperfusion model | Yang XH et al. | 22580272 | Non-human study |
| 1 | Renal redox-sensitive signaling, but not blood pressure, is attenuated by Nox1 knockout in angiotensin II-dependent chronic hypertension | Yogi A et al. | 18195161 | Non-human study |
| 1 | Signal transduction through Ca2+/calmodulin-dependent Ras-GTPase and protein kinase II contributes to development of diabetes-induced renal vascular dysfunction | Yousif MH | 16287213 | Non-human study |
| 1 | Cosegregation of spontaneously hypertensive rat renin gene with elevated blood pressure in an F-2 generation | Yu H et al. | 9794718 | Non-human study |
| 1 | Add-on angiotensin receptor blockade with maximized ACE inhibition | Agarwal R | 11380832 | Not a case-control study |
| 1 | Angiotensin-converting enzyme genotype is a predictive factor in the peak panel-reactive antibody response | Akcay A et al. | 15013293 | Not a case-control study |
| 1 | Association of the genetic polymorphisms of the renin-angiotensin system and endothelial nitric oxide synthase with chronic renal transplant dysfunction | Akcay A et al. | 15385810 | Not a case-control study |
| 1 | Major clinical trials of hypertension - What should be done next? | Alderman MH et al. | WOS:000230012700001 | Not a case-control study |
| 1 | Allergic reaction related to ramipril use: a case report | Alencar RC et al. | 20180980 | Not a case-control study |

| | | | | |
|---|---|---|---|---|
| 1 | CYP3A5 and ABCB1 genes and hypertension | Bochud M et al. | 19290795 | Not a case-control study |
| 1 | Disorders of mineralocorticoid synthesis | Connell JMC et al. | 11469810 | Not a case-control study |
| 1 | Whole-Genome Linkage and Association Scan in Primary, Nonsyndromic Vesicoureteric Reflux | Cordell HJ et al. | 19959718 | Not a case-control study |
| 1 | Genetics of angiotensin I-converting enzyme | Costerousse O et al. | 9247746 | Not a case-control study |
| 1 | Renin Angiotensin System and Cytokines in Chronic Kidney Disease: Clinical and Experimental Evidence | da Silva AAS et al. | 28820061 | Not a case-control study |
| 1 | Association between two genetic polymorphisms of the renin-angiotensin-aldosterone system and diabetic nephropathy: a meta-analysis | Ding W et al. | 21607620 | Not a case-control study |
| 1 | A systematic review and meta-analysis of the association between angiotensin II type 1 receptor A1166C gene polymorphism and myocardial infarction susceptibility | Feng X et al. | 23178513 | Not a case-control study |
| 1 | Polymorphism of angiotensin converting enzyme, angiotensinogen, and angiotensin II type 1 receptor genes and end-stage renal failure in IgA nephropathy: IGARAS - A study of 274 men | Frimat L et al. | 11053482 | Not a case-control study |
| 1 | HLA genes in ANCA-associated vasculitides | Griffith ME et al. | 9493788 | Not a case-control study |
| 1 | Antiproteinuric effect of candesartan cilexetil in Japanese subjects with type 2 diabetes and nephropathy | Haneda M et al. | 15364166 | Not a case-control study |
| 1 | Nonmodulation and essential hypertension | Hollenberg NK et al. | 16672145 | Not a case-control study |
| 1 | Angiotensinogen genotype affects renal and adrenal responses to angiotensin II in essential hypertension | Hopkins PN et al. | 11997278 | Not a case-control study |
| 1 | Association between Angiotensin I-Converting Enzyme Insertion/Deletion Polymorphism and Prognosis of Kidney Transplantation: A Meta-Analysis | Huang ZK et al. | 26000752 | Not a case-control study |

| | | | | |
|---|---|---|---|---|
| 1 | Association of angiotensinogen gene M235T and angiotensin-converting enzyme gene I/D polymorphisms with essential hypertension in Han Chinese population: a meta-analysis | Ji LD et al. | 20087216 | Not a case-control study |
| 1 | The role of renin-angiotensin-aldosterone system genes in the progression of chronic kidney disease: findings from the Chronic Renal Insufficiency Cohort (CRIC) study. | Kelly TN et al. | 25906781 | Not a case-control study |
| 1 | The Phenotypic Patterns of Essential Hypertension Are the Key to Identifying "High Blood Pressure" Genes | Korner PI | 21208016 | Not a case-control study |
| 1 | Glucocorticoids Reduce Aberrant O-Glycosylation of IgA1 in IgA Nephropathy Patients | Kosztyu P et al. | 29529610 | Not a case-control study |
| 1 | Antihypertensive treatment modulates the association between the D/I ACE gene polymorphism and left ventricular hypertrophy: a meta-analysis | Kuznetsova T et al. | 10918550 | Not a case-control study |
| 1 | The renoprotective effect of antihypertensive drugs | Locatelli F et al. | 10048500 | Not a case-control study |
| 1 | microRNAs in Essential Hypertension and Blood Pressure Regulation | Marques FZ et al. | 26663185 | Not a case-control study |
| 1 | Genetics and the prediction of complications in type 1 diabetes | Marre M | 10097900 | Not a case-control study |
| 1 | Improvement of nephrotic syndrome by intensive lipid-lowering therapy in a patient with lipoprotein glomerulopathy | Matsunaga A et al. | 19603250 | Not a case-control study |
| 1 | Mechanisms of plasminogen activator inhibitor 1 action in stromal remodeling and related diseases | Milenkovic J et al. | 29097819 | Not a case-control study |
| 1 | Angiotensinogen gene variation and renoprotective efficacy of renin-angiotensin system blockade in IgA nephropathy | Narita I et al. | 12911556 | Not a case-control study |
| 1 | Angiotensin-I converting enzyme insertion/deletion polymorphism and its association with diabetic nephropathy: a meta-analysis of studies reported between 1994 and 2004 and comprising 14,727 subjects | Ng D et al. | 15830182 | Not a case-control study |

| | | | | |
|---|---|---|---|---|
| 1 | Is the presence of retinopathy of practical value in defining cases of diabetic nephropathy in genetic association studies? The experience with the ACE insertion/deletion polymorphism in 53 studies comprising 17,791 subjects | Ng DPK et al. | 18523141 | Not a case-control study |
| 1 | Partial Deletion of the AGXT Gene (EX1_EX7del): A New Genotype in Hyperoxaluria Type 1 | Nogueira PK et al. | 10737993 | Not a case-control study |
| 1 | A case of rapid amelioration of hepatitis C virus-associated cryoglobulinemic membranoproliferative glomerulonephritis treated by interferon-free directly acting antivirals for HCV in the absence of immunosuppressant | Obata F et al. | 28509128 | Not a case-control study |
| 1 | Survival in type 2 diabetic patients in dialysis and the number of risk alleles in polymorphisms of the renin-angiotensin system genes | Padro-Miquel A et al. | 19014923 | Not a case-control study |
| 1 | Normative genetic profiles of RAAS pathway gene Polymorphisms in north Indian and south Indian Populations | Prasad P et al. | 18027817 | Not a case-control study |
| 1 | Stress, Genes, and Hypertension. Contribution of the ISIAH Rat Strain Study | Redina et al. | 29909475 | Not a case-control study |
| 1 | A Synergistic Association of ACE I/D and eNOS G894T Gene Variants with the Progression of Immunoglobulin A Nephropathy - A Pilot Study | Rodriguez-Perez JC et al. | 19546528 | Not a case-control study |
| 1 | Diabetic nephropathy is associated with AGT polymorphism T235 - Results of a family-based study | Rogus JJ et al. | 9461232 | Not a case-control study |
| 1 | Risk of developing diabetic nephropathy is not associated with synergism between the angiotensin II (type 1) receptor C-1166 allele and poor glycaemic control | Savage DA et al. | 10328465 | Not a case-control study |
| 1 | ACE gene polymorphism and IgA nephropathy: An ethnically homogeneous study and a meta-analysis | Schena FP et al. | 11473656 | Not a case-control study |
| 1 | Genetic determinants of diabetic renal disease and their impact on therapeutic interventions | Schmidt S et al. | WOS:A1997YJ60500008 | Not a case-control study |

| | | | | |
|---|---|---|---|---|
| 1 | Impact of genetic polymorphisms of the renin-angiotensin system and of non-genetic factors on kidney transplant function - a single-center experience | Siekierka-Harreis M et al. | 19681973 | Not a case-control study |
| 1 | The deletion/insertion polymorphism of the angiotensin converting enzyme gene and cardiovascular-renal risk | Staessen JA et al. | 9488209 | Not a case-control study |
| 1 | M235T angiotensinogen gene polymorphism and cardiovascular renal risk | Staessen JA et al. | 10100088 | Not a case-control study |
| 1 | Weber-Christian Disease Associated with the Tip Variant of Focal Segmental Glomerulosclerosis: A Case Report | Sterling KA et al. | 23216240 | Not a case-control study |
| 1 | Gastric-Carcinoma With Osteoclast-Like Giant-Cells - Report Of 4 Cases | Straccapansa V et al. | 7726143 | Not a case-control study |
| 1 | Analysis of baseline parameters in the HALT polycystic kidney disease trials. | Torres VE et al. | 22205355 | Not a case-control study |
| 1 | Mistyping of the human angiotensin-converting enzyme gene polymorphism: Frequency, causes and possible methods to avoid errors in typing | Ueda S et al. | 8863184 | Not a case-control study |
| 1 | Varicella-zoster virus (VZV) and alpha 1 antitrypsin: a fatal outcome in a patient affected by endemic pemphigus foliaceus | Velez AMA et al. | WOS:000305514500009 | Not a case-control study |
| 1 | NHLBI Family Blood Pressure Program: Methodology and recruitment in the HyperGEN network | Williams RR et al. | 10964005 | Not a case-control study |
| 1 | Contribution of gene polymorphisms in the renin-angiotensin system to macroangiopathy in patients with diabetic nephropathy | Wong TYH et al. | 11431175 | Not a case-control study |
| 1 | Gene Polymorphisms of the Renin-AngiotensinAldosterone system and angiotensin 11 type I-Receptor activating antibodies in renal rejection | Zhang G et al. | 17984617 | Not a case-control study |
| 1 | Associations between angiotensinogen M235T polymorphisms and the risk of diabetic nephropathy: A meta-analysis | Zhou B et al. | 29775675 | Not a case-control study |
| 1 | Association of angiotensin II type-1 receptor A1166C gene polymorphism with the susceptibility of end-stage renal disease | Zhou TB et al. | 23971628 | Not a case-control study |

| | | | | |
|---|---|---|---|---|
| 1 | Variants in blood pressure genes and the risk of renal cell carcinoma | Andreotti G et al. | 20047954 | Not a renal disease focus |
| 1 | Association of angiotensin II type 1 receptor gene A1166C polymorphism with the presence of diabetes mellitus and metabolic syndrome in patients with documented coronary artery disease | Assali A et al. | 21570644 | Not a renal disease focus |
| 1 | Germline genetic variations at 11q13 and 12p11 locus modulate age at onset for renal cell carcinoma. | Audenet F et al. | 23911636 | Not a renal disease focus |
| 1 | Angiotensinogen M235T and angiotensin-converting enzyme I/D gene polymorphism and their association with type 2 diabetes in Egypt | Badr S et al. | WOS:000309728600182 | Not a renal disease focus |
| 1 | Polymorphism in angiotensin II receptor genes and hypertension | Baudin B | 15640279 | Not a renal disease focus |
| 1 | Analyses of mutations in the human renal kallikrein (hKLK1) gene and their possible relevance to blood pressure regulation and risk of myocardial infarction | Berge KE et al. | 9298743 | Not a renal disease focus |
| 1 | Single Strand Conformation Polymorphism (SSCP) as a quick and reliable method to genotype M235T polymorphism of angiotensinogen gene | Bettinaglio P et al. | 12270765 | Not a renal disease focus |
| 1 | Polymorphisms of the renin-angiotensin system in patients with multifocal renal arterial fibromuscular dysplasia | Bofinger A et al. | 11317203 | Not a renal disease focus |
| 1 | Association between plasma activities of semicarbazide-sensitive amine oxidase and angiotensin-converting enzyme in patients with type 1 diabetes mellitus. | Boomsma F et al. | 15830186 | Not a renal disease focus |
| 1 | Can the choice of diet undermine the potential genetic risk of AT1R 1166A>C gene polymorphism? | Bozina T et al. | 30205174 | Not a renal disease focus |
| 1 | Structure-Based Analysis of Single Nucleotide Variants in the Renin-Angiotensinogen Complex | Brown DK et al. | 28302554 | Not a renal disease focus |
| 1 | Interactions between serotonin and endogenous and exogenous noradrenaline in the human forearm. | Bruning TA et al. | 7866595 | Not a renal disease focus |

| | | | | |
|---|---|---|---|---|
| 1 | Renin-angiotensin system gene polymorphisms: assessment of the risk of coronary heart disease. | Buraczyńska M et al. | 14502296 | Not a renal disease focus |
| 1 | Oxidative stress-related factors in Bartter's and Gitelman's syndromes: relevance for angiotensin II signalling. | Calo LA et al. | 12897089 | Not a renal disease focus |
| 1 | alpha(1)-antitrypsin (AAT) deficiency and ANCA-positive systemic vasculitis: genetic and clinical implications | Callea F et al. | 9279535 | Not a renal disease focus |
| 1 | Evaluation of Alpha-1 Antitrypsin Levels and SERPINA1 Gene Polymorphisms in Sickle Cell Disease | Carvalho MOS et al. | 29163550 | Not a renal disease focus |
| 1 | Role of GRK4 in the Regulation of Arterial AT(1) Receptor in Hypertension | Chen K et al. | 24218433 | Not a renal disease focus |
| 1 | A study on the association between angiotensin-I converting enzyme I/D dimorphism and type-2 diabetes mellitus. | Chmaisse HN et al. | 19861867 | Not a renal disease focus |
| 1 | alpha-adducin and angiotensin I-converting enzyme polymorphisms in essential hypertension | Clark CJ et al. | 11116113 | Not a renal disease focus |
| 1 | Elevated urinary albumin excretion is not linked to the angiotensin I-converting enzyme gene polymorphism in clinically healthy subjects | Clausen P et al. | 10872702 | Not a renal disease focus |
| 1 | Role of ACE inhibitors in patients with diabetes mellitus | Cordonnier DJ et al. | 11708761 | Not a renal disease focus |
| 1 | Molecular genetics of the renin-angiotensin-aldosterone system in human hypertension | Corvol P et al. | 9296068 | Not a renal disease focus |
| 1 | Angiotensin I-converting enzyme (kininase II) in cardiovascular and renal regulations and diseases | Costerousse O et al. | 9830503 | Not a renal disease focus |
| 1 | Functional polymorphisms in genes of the Angiotensin and Serotonin systems and risk of hypertrophic cardiomyopathy: AT1R as a potential modifier. | Coto E et al. | 20594303 | Not a renal disease focus |
| 1 | SNP Variants in RET and PAX2 and Their Possible Contribution to the Primary Hyperoxaluria Type 1 Phenotype | Coulter-Mackie MB | 25854853 | Not a renal disease focus |
| 1 | Overexpression of human alanine:glyoxylate aminotransferase in Escherichia coli: renaturation from guanidine-HCl and affinity for pyridoxal phosphate co-factor. | Coulter-Mackie MB et al. | 15802217 | Not a renal disease focus |

| | | | | |
|---|---|---|---|---|
| 1 | The major allele of the alanine:glyoxylate aminotransferase gene: nine novel mutations and polymorphisms associated with primary hyperoxaluria type 1. | Coulter-Mackie MB et al. | 15963748 | Not a renal disease focus |
| 1 | Mutation-based diagnostic testing for primary hyperoxaluria type 1: survey of results. | Coulter-Mackie MB et al. | 18282470 | Not a renal disease focus |
| 1 | Further studies on the activity and subcellular distribution of alanine:glyoxylate aminotransferase in the livers of patients with primary hyperoxaluria type 1. | Danpure CJ et al. | 3416563 | Not a renal disease focus |
| 1 | Alanine : glyoxylate aminotransferase peroxisome-to-mitochondrion mistargeting in human hereditary kidney stone disease | Danpure CJ et al. | 12686111 | Not a renal disease focus |
| 1 | Insertion/Deletion Polymorphism of Angiotensin I-converting Enzyme Gene Is Linked With Chromophobe Renal Cell Carcinoma | de Martino M et al. | 21477733 | Not a renal disease focus |
| 1 | Polymorphisms in genes of the renin-angiotensin-aldosterone system and renal cell cancer risk: Interplay with hypertension and intakes of sodium, potassium and fluid | Deckers IA et al. | 24978482 | Not a renal disease focus |
| 1 | ACE gene insertion/deletion polymorphism modulates capillary permeability in hypertension | Dell'omo G et al. | 16889537 | Not a renal disease focus |
| 1 | Single nucleotide polymorphism of adiponectin +276 G/T is associated with the susceptibility to essential hypertension in a Turkish population. | Demir AK et al. | 27936341 | Not a renal disease focus |
| 1 | Genetic polymorphisms associated with exertional rhabdomyolysis | Deuster PA et al. | 23543093 | Not a renal disease focus |
| 1 | Vasodilator-derived nitric oxide inhibits fetal calf serum- and angiotensin-II-induced growth of renal arteriolar smooth muscle cells | Dubey RK et al. | 8169847 | Not a renal disease focus |
| 1 | The angiotensin I-converting enzyme gene insertion/deletion polymorphism is linked to early gastric cancer | Ebert MPA et al. | 16365022 | Not a renal disease focus |
| 1 | A new theory of essential hypertension based on analysis of the association between a polymorphism of the alpha(2)-adrenoceptor at the 10q24-q26 locus and hypertension in African-Americans | Eggers AE | 26243176 | Not a renal disease focus |

| | | | | |
|---|---|---|---|---|
| 1 | CYP3A5 genotype is associated with elevated blood pressure. | Fromm MF et al. | 16141800 | Not a renal disease focus |
| 1 | Functional variant of CYP4A11 20-hydroxyeicosatetraenoic acid synthase is associated with essential hypertension | Gainer JV et al. | 15611369 | Not a renal disease focus |
| 1 | The interaction of AGT and NOS3 gene polymorphisms with conventional risk factors increases predisposition to hypertension | Gatti RR et al. | 22791701 | Not a renal disease focus |
| 1 | Somatic Von Hippel-Lindau Mutation In Clear-Cell Papillary Cystadenoma Of The Epididymis | Gilcrease MZ et al. | 8522307 | Not a renal disease focus |
| 1 | Phenotype-genotype analysis in two Chinese families with Liddle syndrome. | Gong L et al. | 24474657 | Not a renal disease focus |
| 1 | alpha(1)-Antitrypsin Deficiency in Fraternal Twins Born With Familial Spontaneous Pneumothorax | Greene DN et al. | 22215832 | Not a renal disease focus |
| 1 | C-antineutrophil cytoplasmic antibody positivity in vasculitis patients is associated with the Z allele of alpha-1-antitrypsin, and P-antineutrophil cytoplasmic antibody positivity with the S allele. | Griffith ME et al. | 8671812 | Not a renal disease focus |
| 1 | Potential Benefits of Rho-kinase Inhibition in Arterial Hypertension | Grisk O | 23852615 | Not a renal disease focus |
| 1 | A Computational Model of the Circulating Renin-Angiotensin System and Blood Pressure Regulation | Guillaud F et al. | 20683640 | Not a renal disease focus |
| 1 | The presence of PAI-1 4G/5G and ACE DD genotypes increases the risk of early-stage AVF thrombosis in hemodialysis patients. | Gungor Y et al. | 21332339 | Not a renal disease focus |
| 1 | Renin-aldosterone response, urinary Na/K ratio and growth in pseudohypoaldosteronism patients with mutations in epithelial sodium channel (ENaC) subunit genes | Hanukoglu A et al. | 18634878 | Not a renal disease focus |
| 1 | Blunted renal vascular response to angiotensin II is associated with a common Variant of the angiotensinogen gene and obesity | Hopkins PN et al. | 8728297 | Not a renal disease focus |
| 1 | Vitamin B6 in Primary Hyperoxaluria I: First Prospective Trial after 40 Years of Practice | Hoyer-Kuhn H et al. | 24385516 | Not a renal disease focus |

| | | | | |
|---|---|---|---|---|
| 1 | Genetic variation of the renin-angiotensin system and chronic kidney disease progression in black individuals in the atherosclerosis risk in communities study | Hsu CCC et al. | 16396964 | Not a renal disease focus |
| 1 | Clinical and Genetic Factors Associated With Thiazide-Induced Hyponatremia. | Huang CC et al. | 26313793 | Not a renal disease focus |
| 1 | A case control association study of ACE gene polymorphism (I/D) with hypertension in Punjabi population from Faisalabad, Pakistan | Hussain M et al. | 29058472 | Not a renal disease focus |
| 1 | Genetic variant of the renin-angiotensin system and prevalence of type 2 diabetes mellitus: a modest but significant effect of aldosterone synthase | Ichikawa M et al. | 24549414 | Not a renal disease focus |
| 1 | No association between alpha-adducin 460 polymorphism and essential hypertension in a Japanese population | Ishikawa K et al. | 9607391 | Not a renal disease focus |
| 1 | Renin-Angiotensin System Gene Variants and Type 2 Diabetes Mellitus: Influence of Angiotensinogen | Joyce-Tan SM et al. | WOS:000370250500001 | Not a renal disease focus |
| 1 | Lack of association between the alpha-adducin locus and essential hypertension in the Japanese population | Kato N et al. | 9495254 | Not a renal disease focus |
| 1 | Comprehensive analysis of the renin-angiotensin gene polymorphisms with relation to hypertension in the Japanese | Kato N et al. | 10953993 | Not a renal disease focus |
| 1 | Lys(173)Arg and -344T/C variants of CYP11B2 in Japanese patients with low-renin hypertension. | Komiya I et al. | 10720581 | Not a renal disease focus |
| 1 | Molecular-Biology Of Hypertension | Krieger Je et al. | WOS:A1991GF39900002 | Not a renal disease focus |
| 1 | Effects of angiotensin-converting enzyme gene polymorphism and serum vitamin D levels on ambulatory blood pressure measurement and left ventricular mass in Turkish hypertensive population | Kulah E et al. | 17625392 | Not a renal disease focus |
| 1 | Efficacy of Korean Red Ginseng by Single Nucleotide Polymorphism in Obese Women: Randomized, Double-blind, Placebo-controlled Trial | Kwon DH et al. | 23717118 | Not a renal disease focus |
| 1 | Serum liver enzymes in Turner syndrome | Larizza D et al. | 10664223 | Not a renal disease focus |

| | | | | |
|---|---|---|---|---|
| 1 | Risk given by AGT polymorphisms in inducing susceptibility to essential hypertension among isolated populations from a remote region of China: A case-control study among the isolated populations | Li Q et al. | 26391364 | Not a renal disease focus |
| 1 | Single-trait and multi-trait genome-wide association analyses identify novel loci for blood pressure in African-ancestry populations. | Liang J et al. | 28498854 | Not a renal disease focus |
| 1 | No Evidence for the Expression of Renin-Angiotensin-Aldosterone System in Otosclerotic Stapes Footplates | Liktor B et al. | 23370555 | Not a renal disease focus |
| 1 | Functional synergism between the most common polymorphism in human alanine:glyoxylate aminotransferase and four of the most common disease-causing mutations. | Lumb MJ et al. | 10960483 | Not a renal disease focus |
| 1 | Effects of organic anion transporting polypeptide IBI haplotype on pharmacokinetics of pravastatin, valsartan, and temocapril | Maeda K et al. | 16678545 | Not a renal disease focus |
| 1 | Low-salt diet and diuretic effect on blood pressure and organ damage | Manunta P et al. | 14684671 | Not a renal disease focus |
| 1 | Renal changes on hyperglycemia and angiotensin-converting enzyme in type 1 diabetes | Marre M et al. | 10082486 | Not a renal disease focus |
| 1 | Association of TNFRSF4 gene polymorphisms with essential hypertension | Mashimo Y et al. | 18398332 | Not a renal disease focus |
| 1 | Alternative transcripts of the SERPINA1 gene in alpha-1 antitrypsin deficiency | Matamala N et al. | 26141700 | Not a renal disease focus |
| 1 | Detection of the association between a deletion polymorphism in the gene encoding angiotensin I-converting enzyme and advanced diabetic retinopathy | Matsumoto A et al. | 11106834 | Not a renal disease focus |
| 1 | Association of the D allele of the angiotensin I converting enzyme polymorphism with malignant vascular injury | Mayer NJ et al. | WOS:000173608200004 | Not a renal disease focus |
| 1 | Genotypic interactions of renin-angiotensin system genes with diabetes type 2 in a Tunisian population | Mehri S et al. | 20580725 | Not a renal disease focus |

| | | | | |
|---|---|---|---|---|
| 1 | Association of polymorphisms of angiotensin I converting enzyme 2 with retinopathy in type 2 diabetes mellitus among Chinese individuals | Meng N et al. | 25359286 | Not a renal disease focus |
| 1 | The consensus-based approach for gene/enzyme replacement therapies and crystallization strategies: the case of human alanine-glyoxylate aminotransferase. | Mesa-Torres N et al. | 24957194 | Not a renal disease focus |
| 1 | Angiotensin II type 1 receptor gene polymorphism and the response to hyperglycemia in early type 1 diabetes | Miller JA et al. | 10969844 | Not a renal disease focus |
| 1 | Pharmacologic rescue of an enzyme-trafficking defect in primary hyperoxaluria 1. | Miyata N et al. | 25237136 | Not a renal disease focus |
| 1 | Association of Polymorphisms in Endothelial Nitric Oxide Synthesis and Renin-Angiotensin-Aldosterone System with Developing of Coronary Artery Disease in Bulgarian Patients | Mokretar K et al. | 26670794 | Not a renal disease focus |
| 1 | Contribution of angiotensin I converting enzyme gene polymorphism and angiotensinogen gene polymorphism to blood pressure regulation in essential hypertension. | Mondorf UF et al. | 9524045 | Not a renal disease focus |
| 1 | Comprehensive mutation screening in 55 probands with type 1 primary hyperoxaluria shows feasibility of a gene-based diagnosis. | Monico CG et al. | 17460142 | Not a renal disease focus |
| 1 | Inhibition of tissue angiotensin converting enzyme activity prevents malignant hypertension in TGR(mREN2)27. | Montgomery HE et al. | 9797175 | Not a renal disease focus |
| 1 | Two novel point mutations in the lecithin:cholesterol acyltransferase (LCAT) gene resulting in LCAT deficiency: LCAT (G873 deletion) and LCAT (Gly344-->Ser) | Moriyama K et al. | 8656071 | Not a renal disease focus |
| 1 | Independent, Marked Associations Of Alleles Of The Insulin-Receptor And Dipeptidyl Carboxypeptidase-I Genes With Essential-Hypertension | Morris BJ et al. | 8104754 | Not a renal disease focus |
| 1 | ANCA-associated vasculitis is linked to carriage of the Z allele of alpha(1) antitrypsin and its polymers | Morris H et al. | 21821620 | Not a renal disease focus |
| 1 | Is the influence of variation in the ACE gene on the prospective risk of Type 2 diabetes in middle-aged men modified by obesity? | Muthumala A et al. | 17624939 | Not a renal disease focus |

| | | | | |
|---|---|---|---|---|
| 1 | Angiotensin converting enzyme (ACE) insertion/deletion (I/D) polymorphism, and diabetic retinopathy in subjects with IDDM and NIDDM | Nagi Dk et al. | 8582133 | Not a renal disease focus |
| 1 | Association between RAS gene polymorphisms (ACE I/D, AGT M235T) and Henoch-Schonlein purpura in a Turkish population | Nalbantoglu S et al. | 23151617 | Not a renal disease focus |
| 1 | Enhanced renal production of cyclic GMP and reduced free water clearance during sodium nitroprusside infusion in healthy man | Nielsen CB et al. | 8393795 | Not a renal disease focus |
| 1 | Alpha-1-proteinase inhibitor and pulmonary haemorrhage in systemic vasculitis. | O'Donoghue DJ et al. | 8296629 | Not a renal disease focus |
| 1 | Genetic polymorphisms of the renin-angiotensin system and atheromatous renal artery stenosis | Olivieri O et al. | 10567188 | Not a renal disease focus |
| 1 | Endothelial nitric oxide synthase gene/gender interactions and the renal hemodynamic response to angiotensin II | Page A et al. | 16093452 | Not a renal disease focus |
| 1 | Angiotensin-converting enzyme and angiotensin II receptor subtype 2 genotypes in type 1 diabetes and severe hypoglycaemia requiring emergency treatment: a case cohort study | Pedersen-Bjergaard U et al. | 19820429 | Not a renal disease focus |
| 1 | Deep-targeted exon sequencing reveals renal polymorphisms associate with postexercise hypotension among African Americans | Pescatello LS et al. | WOS:000387445200013 | Not a renal disease focus |
| 1 | Impact of maternal angiotensinogen M235T polymorphism and angiotensin-converting enzyme insertion/deletion polymorphism on blood pressure, protein excretion and fetal outcome in pregnancy. | Pfab T et al. | 17563539 | Not a renal disease focus |
| 1 | Effect of ACE inhibitors and beta-blockers on homocysteine levels in essential hypertension | Poduri A et al. | 18200034 | Not a renal disease focus |
| 1 | The state and responsiveness of the renin-angiotensin-aldosterone system in patients with type II diabetes mellitus | Price DA et al. | 10232494 | Not a renal disease focus |
| 1 | Identification of mutations associated with peroxisome-to-mitochondrion mistargeting of alanine/glyoxylate aminotransferase in primary hyperoxaluria type 1. | Purdue PE et al. | 1703535 | Not a renal disease focus |
| 1 | The relationship between ACE/AGT gene polymorphisms and the risk of diabetic retinopathy in Chinese patients with type 2 diabetes. | Qiao YC et al. | 29378484 | Not a renal disease focus |

| | | | | |
|---|---|---|---|---|
| 1 | Association of angiotensin-converting enzyme gene dimorphisms with severity of lupus disease. | Rabbani MA et al. | 18711292 | Not a renal disease focus |
| 1 | Association of angiotensinogen M235T and A(-6)G gene polymorphisms with coronary heart disease with independence of essential hypertension: the PROCAGENE study. Prospective Cardiac Gene. | Rodriquez-Perez JC et al. | 11345362 | Not a renal disease focus |
| 1 | Evaluation of mutation screening as a first line test for the diagnosis of the primary hyperoxalurias. | Rumsby G et al. | 15327387 | Not a renal disease focus |
| 1 | Angiotensin II acutely attenuates range of arterial baroreflex control of renal sympathetic nerve activity | Sanderford MG et al. | 11009467 | Not a renal disease focus |
| 1 | An Erythropoietin Gene Polymorphism in the Hypoxia-Responsive Element at Position 3434 Is Possibly Associated with Hypertension | Schulz EG et al. | 21912181 | Not a renal disease focus |
| 1 | Ubiquitin ligase gp78 increases solubility and facilitates degradation of the Z variant of alpha-1-antitrypsin. | Shen Y et al. | 16979136 | Not a renal disease focus |
| 1 | Association of APOE (Hha1) and ACE (I/D) gene polymorphisms with type 2 diabetes mellitus in North West India | Singh PP et al. | 16621107 | Not a renal disease focus |
| 1 | Renal haemodynamics are not related to genotypes in offspring of parents with essential hypertension | Skov K et al. | 17083073 | Not a renal disease focus |
| 1 | Association between renin-angiotensin-aldosterone system-related genes and blood pressure in a Korean population. | Song SB et al. | 21342026 | Not a renal disease focus |
| 1 | Angiotensin II sensitivity in nonpregnant formerly preeclamptic women and healthy parous controls | Spaanderman MEA et al. | 15350256 | Not a renal disease focus |
| 1 | The genetic predisposition to produce high levels of TGF-beta 1 impacts on the severity of eclampsia/pre-eclampsia | Stanczuk GA et al. | 17653872 | Not a renal disease focus |
| 1 | Angiotensin-converting enzyme gene I/D polymorphism in malignant hypertension | Stefansson B et al. | 10855732 | Not a renal disease focus |
| 1 | A Polymorphism Regulates CYP4A11 Transcriptional Activity and Is Associated With Hypertension in a Japanese Population | Sugimoto K et al. | 18936345 | Not a renal disease focus |

| | | | | |
|---|---|---|---|---|
| 1 | Blunted cGMP response to agonists and enhanced glomerular cyclic 3',5'-nucleotide phosphodiesterase activities in experimental congestive heart failure | Supaporn T et al. | 8914042 | Not a renal disease focus |
| 1 | Alpha1-antitrypsin phenotypes and anti-neutrophil cytoplasmic auto-antibodies in inflammatory bowel disease. | Taddei C et al. | 10563543 | Not a renal disease focus |
| 1 | Angiotensin I converting enzyme gene polymorphisms in systemic lupus erythematosus: decreased prevalence of DD genotype in African American patients | Tassiulas IO et al. | 9710341 | Not a renal disease focus |
| 1 | Middle cerebral artery stenosis in type II diabetic Chinese patients is associated with conventional risk factors but not with polymorphisms of the renin-angiotensin system genes | Thomas GN et al. | 12865608 | Not a renal disease focus |
| 1 | Peripheral vascular disease in type 2 diabetic Chinese patients: associations with metabolic indices, concomitant vascular disease and genetic factors | Thomas GN et al. | 14632699 | Not a renal disease focus |
| 1 | Genetic predisposition to left ventricular hypertrophy and the potential involvement of cystatin-C in untreated hypertension. | Tousoulis D et al. | 23479071 | Not a renal disease focus |
| 1 | Genomic association analysis identifies multiple loci influencing antihypertensive response to an angiotensin II receptor blocker. | Turner ST et al. | 22566498 | Not a renal disease focus |
| 1 | The M235T polymorphism in theangiotensinogen gene is associated with the risk of malignant hypertension in white patients | van den Born BJH et al. | 17921816 | Not a renal disease focus |
| 1 | Genetic risk of atherosclerotic renal artery disease - The candidate gene approach in a renal angiography cohort | van Onna M et al. | 15326089 | Not a renal disease focus |
| 1 | Eprosartan modulates the reflex activation of the sympathetic nervous system in sodium restricted healthy humans | Vase H et al. | 18341678 | Not a renal disease focus |
| 1 | Angiotensin I-converting enzyme and angiotensinogen gene interaction and prediction of essential hypertension | Vasku A et al. | 9607178 | Not a renal disease focus |
| 1 | Association between ACE gene polymorphisms and Alzheimer's disease in Han population in Hebei Peninsula | Wang XL et al. | WOS:000412148800122 | Not a renal disease focus |
| 1 | Genotype-phenotype analysis of angiotensinogen polymorphisms and essential hypertension: the importance of haplotypes | Watkins WS et al. | 19770777 | Not a renal disease focus |

| | | | | |
|---|---|---|---|---|
| 1 | No association between a genetic variant of the p22(phox) component of NAD(P)H oxidase and the incidence and progression of IgA nephropathy | Wolf G et al. | 12147803 | Not a renal disease focus |
| 1 | Genetic polymorphisms of the angiotensin II type 1 receptor gene and diastolic heart failure | Wu CK et al. | 19330904 | Not a renal disease focus |
| 1 | Increased expression of angiotensin II type 1 receptor (AGTR1) in heart transplant recipients with recurrent rejection | Yamani MH et al. | 17097490 | Not a renal disease focus |
| 1 | G-protein beta 3 subunit gene C825T polymorphism in patients with vesico-ureteric reflux | Zagradisnik B et al. | 15337465 | Not a renal disease focus |
| 1 | Frequencies of variants of candidate genes in different age groups of hypertensives | Zee RY et al. | 7882587 | Not a renal disease focus |
| 1 | Crystal structure of alanine: Glyoxylate aminotransferase and the relationship between genotype and enzymatic phenotype in primary hyperoxaluria type 1 | Zhang XX et al. | 12899834 | Not a renal disease focus |
| 1 | Rab1 Small GTP-Binding Protein Regulates Cell Surface Trafficking of the Human Calcium-Sensing Receptor | Zhuang XL et al. | 20861236 | Not a renal disease focus |
| 1 | [Renin-angiotensin system genes in chronic glomerulonephritis]. | Buraczyńska M et al. | 11865575 | Not written in English or Spanish |
| 1 | [Association of the renin-angiotensin system gene polymorphism with nephropathy in type II diabetes]. | Buraczyńska M et al. | 12476891 | Not written in English or Spanish |
| 1 | [Genetic predisposition to systemic complications of arterial hypertension in maintenance haemodialysis patients]. | Bzoma B et al. | 19112833 | Not written in English or Spanish |
| 1 | [Polymorphism of gene encoding vascular angiotensin II receptor and microangiopathies in patients with insulin-dependent diabetes mellitus]. | Chistiakov DA et al. | 10576062 | Not written in English or Spanish |
| 1 | [Arterial hypertension and chronic hemodialysis]. | Ermolenko VM et al. | 7700 | Not written in English or Spanish |
| 1 | [Is PstI polymorphism of the angiotensin I converting enzyme gene associated with nephropathy development in non-insulin-dependent diabetes mellitus (preliminary study)]. | Grzeszczak W et al. | 9499204 | Not written in English or Spanish |

| | | | | |
|---|---|---|---|---|
| 1 | [Effect of eprosartan on the hemostatic system in patients with chronic kidney disease associated with hereditary thrombophilia]. | Kaliuzhin VV et al. | 24261234 | Not written in English or Spanish |
| 1 | [Polymorphism studies of angiotensin converting enzyme gene in chronic glomerulonephritis]. | Kutyrina IM et al. | 10420452 | Not written in English or Spanish |
| 1 | [Relationship between serum angiotensin I-converting enzyme activity and diabetic nephropathy in patients with type II diabetes]. | Liao L et al. | 12016801 | Not written in English or Spanish |
| 1 | [Relationship between I/D polymorphism of angiotensin I converting enzyme gene and microvascular complications in type 2 diabetic patients]. | Moleda P et al. | 17941464 | Not written in English or Spanish |
| 1 | [Pharmacogenetic aspects of candesartan application for the treatment of arterial hypertension in patients with chronic pyelonephritis]. | Mormol' IA | 25286611 | Not written in English or Spanish |
| 1 | [A study on angiotensin-I converting enzyme polymorphism in CAPD patients]. | Nishina M | 9014479 | Not written in English or Spanish |
| 1 | [Identification of the locus associated with diabetic nephropathy in type 1 diabetes mellitus]. | Savost'ianov KV et al. | 12500539 | Not written in English or Spanish |
| 1 | [Relationships of angiotensinogen gene M235T variant with diabetic nephropathy in Chinese type 2 diabetes mellitus]. | Wang J et al. | 10514536 | Not written in English or Spanish |
| 1 | [Association between angiotensin-II receptor gene type I polymorphism and diabetic nephropathy in type 2 diabetes mellitus]. | Xue Y et al. | 11798574 | Not written in English or Spanish |
| 1 | [AGTR1 A1166C polymorphism is associated with risk of diabetic nephropathy]. | Yin X et al. | 23505107 | Not written in English or Spanish |
| 1 | Posterior urethral valves: Preliminary observations on the significance of plasma renin activity as a prognostic marker | Bajpai M et al. | 15643266 | Paediatric Individuals |
| 1 | Donor and recipient ACE I/D genotype are associated with loss of renal function in children following renal transplantation | Buscher R et al. | 21309964 | Paediatric Individuals |
| 1 | Polymorphisms of the angiotensin converting enzyme and angiotensin II type 1 receptor genes and renal scarring in non-uropathic children with recurrent urinary tract infection | Ece A et al. | 16109085 | Paediatric Individuals |

| | | | | |
|---|---|---|---|---|
| 1 | Genetic polymorphism of ACE and the angiotensin II type1 receptor genes in children with chronic kidney disease | Elshamaa MF et al. | 21859496 | Paediatric Individuals |
| 1 | ACE gene polymorphism in Egyptian children with idiopathic nephrotic syndrome | Fahmy ME et al. | 18792483 | Paediatric Individuals |
| 1 | Identification of a novel splice site mutation of CLCN5 gene and characterization of a new alternative 5 ' UTR end of ClC-5 mRNA in human renal tissue and leukocytes | Forino M et al. | 14673707 | Paediatric Individuals |
| 1 | Genetic polymorphisms of the renin-angiotensin system and the outcome of focal segmental glomerulosclerosis in children | Frishberg Y et al. | 9853248 | Paediatric Individuals |
| 1 | Angiotensinogen gene T235 variant: a marker for the development of persistent microalbuminuria in children and adolescents with type 1 diabetes mellitus | Gallego PH et al. | 18413222 | Paediatric Individuals |
| 1 | Autosomal dominant pseudohypoaldosteronism type 1: Mechanisms, evidence for neonatal lethality, and phenotypic expression in adults | Geller DS et al. | 16611713 | Paediatric Individuals |
| 1 | Implication of genetic variations in congenital obstructive nephropathy | Hahn H et al. | 16133060 | Paediatric Individuals |
| 1 | Variants of alpha(1)-proteinase inhibitor in black and white South African patients with focal glomerulosclerosis and minimal change nephrotic syndrome | Halkas AC et al. | 9475086 | Paediatric Individuals |
| 1 | Angiotensin-converting enzyme insertion/deletion gene polymorphism in Egyptian children with systemic lupus erythematosus: a possible relation to proliferative nephritis. | Hammad A et al. | 27956582 | Paediatric Individuals |
| 1 | ACE gene polymorphism and renal scarring in primary vesicoureteric reflux | Haszon I et al. | 12478352 | Paediatric Individuals |
| 1 | Angiotensin type 2 receptor is important in the normal development of the ureter | Hohenfellner K et al. | 10353402 | Paediatric Individuals |
| 1 | Impact of common functional polymorphisms in renin angiotensin system genes on the risk of renal parenchymal scarring following childhood urinary tract infection | Hussein A et al. | 25939993 | Paediatric Individuals |

| | | | | |
|---|---|---|---|---|
| 1 | Gene Polymorphisms of Adducin GLY460TRP, ACE I/D, and AGT M235T in Pediatric Hypertension Patients | Kaplan I et al. | 25262176 | Paediatric Individuals |
| 1 | ACE and AT1 receptor gene polymorphisms and renal scarring in urinary bladder dysfunction | Kostic M et al. | 15179569 | Paediatric Individuals |
| 1 | Renal tubular dysgenesis, a not uncommon autosomal recessive disorder leading to oligohydramnios: Role of the Renin-Angiotensin system. | Lacoste M et al. | 16790508 | Paediatric Individuals |
| 1 | Hemorrhagic shock and encephalopathy: clinical, pathologic, and biochemical features. | Levin M et al. | 2783733 | Paediatric Individuals |
| 1 | Renin-angiotensin system gene polymorphisms in children with Henoch-Schonlein purpura in West China | Liu DS et al. | WOS:000284763300006 | Paediatric Individuals |
| 1 | Renin-angiotensin system polymorphisms in Taiwanese primary vesicoureteral reflux | Liu KP et al. | 15045574 | Paediatric Individuals |
| 1 | Polymorphisms of renin-angiotensin system genes in childhood IgA nephropathy | Maruyama K et al. | 11354780 | Paediatric Individuals |
| 1 | Angiotensin-Converting Enzyme Gene Polymorphism in Children with Idiopathic Nephrotic Syndrome, Effect on Biopsy Findings. | Monajemzadeh M et al. | 28481137 | Paediatric Individuals |
| 1 | A-20C angiotensinogen gene polymorphism and proteinuria in childhood IgA nephropathy | Nakanishi K et al. | 14648325 | Paediatric Individuals |
| 1 | Implications of certain genetic polymorphisms in scarring in vesicoureteric reflux: importance of ACE polymorphism. | Ozen S et al. | 10401028 | Paediatric Individuals |
| 1 | Renin-angiotensin system gene polymorphisms: association with susceptibility to Henoch-Schonlein purpura and renal involvement | Ozkaya O et al. | 16521052 | Paediatric Individuals |
| 1 | Renin-angiotensin gene polymorphism in children with uremia and essential hypertension | Papp F et al. | 12579405 | Paediatric Individuals |
| 1 | Renin-angiotensin system polymorphisms and renal scarring | Pardo R et al. | 12579398 | Paediatric Individuals |
| 1 | Angiotensin I-converting enzyme-gene-polymorphism: Relationship to albumin excretion and blood pressure in pediatric patients with type-I-diabetes mellitus | Pavlovic M et al. | 9354852 | Paediatric Individuals |

| | | | | |
|---|---|---|---|---|
| 1 | Angiotensin I converting enzyme and angiotensinogen gene polymorphisms related to 24-h blood pressure in paediatric type I diabetes mellitus | Pavlovic M et al. | 9950302 | Paediatric Individuals |
| 1 | Low renin-angiotensin system activity gene polymorphism and dysplasia associated with posterior urethral valves | Peruzzi L et al. | 16006956 | Paediatric Individuals |
| 1 | Angiotensin-converting enzyme and angiotensin type 2 receptor gene genotype distributions in Italian children with congenital uropathies | Rigoli L et al. | 15470205 | Paediatric Individuals |
| 1 | Angiotensin II type 2 receptor gene polymorphism in Caucasian children with a wide spectrum of congenital anomalies of the kidney and urinary tract | Siomou E et al. | 17515833 | Paediatric Individuals |
| 1 | Effects of genetic polymorphisms of the renin-angiotensin system in children with nephrotic syndrome | Tabel Y et al. | 16525944 | Paediatric Individuals |
| 1 | ACE gene polymorphism in childhood IgA nephropathy: Association with clinicopathologic findings | Tanaka R et al. | 9590186 | Paediatric Individuals |
| 1 | Genetic risk factors in typical haemolytic uraemic syndrome | Taranta A et al. | 19110485 | Paediatric Individuals |
| 1 | The juxtaglomerular apparatus in Bartter's syndrome and related tubulopathies. An immunocytochemical and electron microscopic study. | Taugner R et al. | 3128915 | Paediatric Individuals |
| 1 | Genetic polymorphism of the renin-angiotensin system on the development of primary vesicoureteral reflux | Yim HE et al. | 14764974 | Paediatric Individuals |
| 1 | Genetic control of VEGF and TGF-beta 1 gene polymorphisms in childhood urinary tract infection and vesicoureteral reflux | Yim HE et al. | 17597658 | Paediatric Individuals |
| 1 | Estimation of the relationship between the polymorphisms of selected genes: ACE, AGTR1, TGF beta 1 and GNB3 with the occurrence of primary vesicoureteral reflux | Zyczkowski et al. | 27988909 | Paediatric Individuals |
| 1 | Randomized Controlled Trial: Lisinopril Reduces Proteinuria, Ammonia, and Renal Polypeptide Tubular Catabolism in Patients With Chronic Allograft Nephropathy | Amara AB et al. | 20061926 | Pharmaceutical drug focus |

| | | | | |
|---|---|---|---|---|
| 1 | Long-term renoprotective effects of losartan in diabetic nephropathy: interaction with ACE insertion/deletion genotype? | Andersen S et al. | 12716812 | Pharmaceutical drug focus |
| 1 | Effects of losartan and enalapril on high-sensitivity C-reactive protein and total antioxidant in renal transplant recipients with renin-angiotensin system polymorphisms | Argani H et al. | 18261537 | Pharmaceutical drug focus |
| 1 | Efficacy and safety of the angiotensin II receptor blocker losartan for hypertrophic cardiomyopathy: the INHERIT randomised, double-blind, placebo-controlled trial | Axelsson A et al. | 25533774 | Pharmaceutical drug focus |
| 1 | Angiotensin I - Converting enzyme gene polymorphism modulates the consequences of in utero growth retardation on plasma insulin in young adults | Cambien F et al. | 9519756 | Pharmaceutical drug focus |
| 1 | Losartan decreases plasma levels of TGF-beta 1 in transplant patients with chronic allograft nephropathy | Campistol JM et al. | 10432413 | Pharmaceutical drug focus |
| 1 | High serum enalaprilat in chronic renal failure | Elung-Jensen et al. | 11881130 | Pharmaceutical drug focus |
| 1 | The influence of renin-angiotensin system genotypes on the antiproteinuric response to high doses of olmesartan in non-diabetic protein uric nephropathies | Goyache-Goni B et al. | 24241364 | Pharmaceutical drug focus |
| 1 | Renal implications of angiotensin receptor blockers | Hollenberg NK | 11459212 | Pharmaceutical drug focus |
| 1 | CYP2C9 genotype and pharmacodynamic responses to losartan in patients with primary and secondary kidney diseases. | Joy MS et al. | 19669737 | Pharmaceutical drug focus |
| 1 | Association of angiotensinogen gene polymorphism with erythropoietin-induced hypertension: a preliminary report | Kuriyama S et al. | 11675943 | Pharmaceutical drug focus |
| 1 | The influence of the ACE (I/D) polymorphism on systemic and renal vascular responses to angiotensins in normotensive, normoalbuminuric Type 1 diabetes mellitus | Luik PT et al. | 12856080 | Pharmaceutical drug focus |
| 1 | Renin-angiotensin system polymorphisms and hemoglobin level in renal allografts: A comparative study between losartan and enalapril | Noroozianavval M et al. | 17524880 | Pharmaceutical drug focus |
| 1 | ACE gene polymorphism and losartan treatment in type 2 diabetic patients with nephropathy | Parving HH et al. | 18199798 | Pharmaceutical drug focus |

| | | | | |
|---|---|---|---|---|
| 1 | Enalapril and losartan affect lipid peroxidation in renal transplant recipients with renin-angiotensin system polymorphisms | Rashtchizadeh N et al. | 17222813 | Pharmaceutical drug focus |
| 1 | Exaggerated natriuresis during clamping of systemic NO supply in healthy young men | Simonsen JA et al. | 21749320 | Pharmaceutical drug focus |
| 2 | Influence of genetic polymorphisms of the renin-angiotensin system on IgA nephropathy | Bantis C et al. | 15031629 | No data |
| 2 | Genetic polymorphisms of renin-angiotensin system and progression of interstitial nephritis. | Buraczyńska M et al. | 12898858 | No data |
| 2 | Polymorphism of the angiotensinogen gene and genetic predisposition to diabetic nephropathy in diabetes mellitus type 1 | Chistyakov DA et al. | WOS:000079798700011 | No data |
| 2 | U.K. Prospective Diabetes Study. XV: Relationship of renin-angiotensin system gene polymorphisms with microalbuminuria in NIDDM. | Dudley CR et al. | 8587251 | No data |
| 2 | Angiotensin I-converting enzyme and angiotensinogen gene polymorphisms in non-insulin-dependent diabetes mellitus. Lack of relationship with diabetic nephropathy and retinopathy in a Caucasian Mediterranean population. | Gutierrez C et al. | 9258285 | No data |
| 2 | Effects of the genetic polymorphisms of the renin-angiotensin system on focal segmental glomerulosclerosis. | Luther Y et al. | 14610337 | No data |
| 2 | The renin--angiotensin system gene polymorphisms and clinicopathological correlations in IgA nephropathy. | Ong-Ajyooth S et al. | 10511770 | No data |
| 2 | Combinational effect of genes for the renin-angiotensin system in conferring susceptibility to diabetic nephropathy. | Osawa N et al. | 17143591 | No data |
| 2 | The relationship between genetic and haemodynamic factors in diabetic nephropathy (DN): Case-control study in type 1 diabetes mellitus (T1DM) | Shestakova MV et al. | WOS:000242410600010 | No data |
| 2 | Relationship between angiotensinogen gene M235T variant with diabetic nephropathy in Chinese NIDDM | Wang JJ et al. | 11717948 | No data |
| 2 | Lack of association of angiotensin-converting enzyme (DID/II) and angiotensinogen M235T gene polymorphism with renal function among Chinese patients with type II diabetes | Wong TYH et al. | 10352194 | No data |

| | | | | |
|---|---|---|---|---|
| 2 | Polymorphism of renin-angiotensin system genes in IgA nephropathy | Woo KT et al. | 15504143 | No data |
| 3 | ACE variants interact with the RAS pathway to confer risk and protection against type 2 diabetic nephropathy. | Ahluwalia TS et al. | 19108684 | < 3 populations reported per SNP |
| 3 | Analysis of polymorphism in renin angiotensin system and other related genes in South Indian chronic kidney disease patients. | Anbazhagan K et al. | 19520069 | < 3 populations reported per SNP |
| 3 | Polymorphism of the renin–angiotensin–aldosterone system in patients with chronic allograft dysfunction | Ayed K et al. | 16635753 | < 3 populations reported per SNP |
| 3 | Renin-angiotensin-aldosterone system related gene polymorphisms and urinary total arsenic is related to chronic kidney disease | Chen WJ et al. | 24907556 | < 3 populations reported per SNP |
| 3 | Hypertension after renal transplantation and polymorphism of genes involved in essential hypertension: ACE, AGT, AT1R and ecNOS | El-Essawy AB et al. | 11926202 | < 3 populations reported per SNP |
| 3 | Genetic polymorphisms of the renin-angiotensin-aldosterone system and renal insufficiency in essential hypertension. | Fabris B et al. | 15662219 | < 3 populations reported per SNP |
| 3 | Angiotensinogen gene polymorphisms and progression of chronic kidney disease in ADPKD patients | Gnanasambandan R et al. | 26482465 | < 3 populations reported per SNP |
| 3 | Angiotensinogen and angiotensin II type 1 receptor gene polymorphism in patients with autosomal dominant polycystic kidney disease: effect on hypertension and ESRD. | Lee KB et al. | 12950120 | < 3 populations reported per SNP |
| 3 | Genes involved in the regulation of vascular homeostasis determine renal survival rate in patients with chronic glomerulonephritis | Litovkina O et al. | 24727057 | < 3 populations reported per SNP |
| 3 | Contribution of genetic polymorphism in the renin-angiotensin system to the development of renal complications in insulin-dependent diabetes | Marre M et al. | 9120002 | < 3 populations reported per SNP |
| 3 | Genetic polymorphism of renin-angiotensin system is not associated with diabetic vascular complications in Japanese subjects with long-term insulin dependent diabetes mellitus. | Miura J et al. | 10499884 | < 3 populations reported per SNP |
| 3 | Renin-angiotensin-aldosterone system genotypes and haplotypes affect the susceptibility to nephropathy in type 2 diabetes patients | Mtiraoui N et al. | 21421655 | < 3 populations reported per SNP |

| | | | | |
|---|---|---|---|---|
| 3 | Renin-angiotensin system gene polymorphisms predict the progression to renal insufficiency among Asians with lupus nephritis | Parsa A et al. | 15789057 | < 3 populations reported per SNP |
| 3 | Association of aldosterone synthase (CYP11B2) gene -344T/C polymorphism with the risk of primary chronic glomerulonephritis in the Polish population. | Pawlik M et al. | 23681285 | < 3 populations reported per SNP |
| 3 | Association of angiotensinogen gene T235 variant with progression of immunoglobin A nephropathy in Caucasian patients | Pei Y et al. | 9259580 | < 3 populations reported per SNP |
| 3 | Chronic renal insufficiency among Asian Indians with type 2 diabetes: I. Role of RAAS gene polymorphisms. | Prasad P et al. | 16672053 | < 3 populations reported per SNP |
| 3 | Angiotensinogen and plasminogen activator inhibitor-1 gene polymorphism in relation to renovascular disease. | Reis KA et al. | 16228848 | < 3 populations reported per SNP |
| 3 | M235T Polymorphism in the AGT Gene and A/GI8-83 Substitution in the REN Gene Correlate with End-Stage Renal Disease | Sarkar S et al. | 25660845 | < 3 populations reported per SNP |
| 3 | Genetic variants of ACE (Insertion/Deletion) and AGT (M268T) genes in patients with diabetes and nephropathy | Shaikh R et al. | 24737640 | < 3 populations reported per SNP |
| 3 | Polymorphisms of the renin-angiotensin system genes in Brazilian patients with lupus nephropathy. | Sprovieri SR et al. | 15934435 | < 3 populations reported per SNP |
| 3 | Interaction between gene polymorphisms of nitric oxide synthase and renin-angiotensin system in the progression of membranous glomerulonephritis | Stratta P et al. | 14767013 | < 3 populations reported per SNP |
| 3 | Gene polymorphisms of angiotensin-converting enzyme and angiotensin II type 1 receptor among chronic kidney disease patients in a Chinese population. | Su SL et al. | 22147663 | < 3 populations reported per SNP |
| 3 | Genetic polymorphisms and the risk of progressive renal failure in elderly Hungarian patients. | Zsom M et al. | 22111818 | < 3 populations reported per SNP |

**Supplementary Table S3d:** Excluded studies from the *AGTR1* search

*Exclusion stage relates to the stage at which the article was removed as shown in the flow diagram (Supplementary Figure S1d)

| Exclusion Stage | Title | Authors | Pubmed ID or WoS ID if Pubmed ID not available | Reason |
|---|---|---|---|---|
| 1 | Identification of potential candidate genes for hypertensive nephropathy based on gene expression profile | Chen Z et al. | 27756246 | Gene expression based study |
| 1 | Polymorphism in IgA nephropathy | Liu ZH et al. | WOS:A1997WW80600013 | No data for AGTR1 |
| 1 | The effect of angiotensin receptor blockade ARB on the regression of left ventricular hypertrophy in hemodialysis patients: comparison between patients with D allele and non-D allele ACE gene polymorphism. | Nakayama M et al. | 16312263 | No data for AGTR1 |
| 1 | Angiotensin II receptor type 1 A1166C modifies the association between angiotensinogen M235T and chronic kidney disease | Su SL et al. | 29296205 | No data for AGTR1 |
| 1 | Specific pregnancy-induced angiotensin II type-1 receptor expression in ovine uterine artery does not involve formation of alternate splice variants or alternate promoter usage | Bird IM et al. | 9687288 | Non-human study |
| 1 | CCN1 expression in interleukin-6 deficient mouse kidney in experimental model of heart failure | Bonda TA et al. | 23690222 | Non-human study |
| 1 | The genetic deletion of Mas abolishes salt induced hypertension in mice | Heringer-Walther S et al. | 22652430 | Non-human study |
| 1 | The angiotensin type II receptor tonically inhibits angiotensin-converting enzyme in AT2 null mutant mice | Hunley TE et al. | 10652034 | Non-human study |
| 1 | Collecting Duct Nitric Oxide Synthase 1 beta Activation Maintains Sodium Homeostasis During High Sodium Intake Through Suppression of Aldosterone and Renal Angiotensin II Pathways | Hyndman KA et al. | 29066445 | Non-human study |
| 1 | Physiological impact of increased expression of the AT(1) angiotensin receptor | Le TH et al. | 12963678 | Non-human study |

| | | | | |
|---|---|---|---|---|
| 1 | Angiotensin receptor-binding protein ATRAP/Agtrap inhibits metabolic dysfunction with visceral obesity. | Maeda A et al. | 23902639 | Non-human study |
| 1 | A novel rodent model of pregnancy complications associated with genetically determined angiotensin-converting enzyme (ACE) activity | Mata-Greenwood E et al. | 29360395 | Non-human study |
| 1 | Losartan and Sodium Nitroprusside Effectively Protect against Renal Impairments after Ischemia and Reperfusion in Rats | Srisawat U et al. | 25947921 | Non-human study |
| 1 | Blood Pressure Control by a Secreted FGFBP1 (Fibroblast Growth Factor-Binding Protein). | Tassi E et al. | 29158353 | Non-human study |
| 1 | Mechanisms of Renal. Control of Potassium Homeostasis in Complete Aldosterone Deficiency | Todkar A et al. | 25071088 | Non-human study |
| 1 | Cardiac phenotype and angiotensin II levels in AT(1a), AT(1b), and AT(2) receptor single, double, and triple knockouts | van Esch JHM et al. | 20071356 | Non-human study |
| 1 | Human GRK4 gamma(142V) Variant Promotes Angiotensin II Type I Receptor-Mediated Hypertension via Renal Histone Deacetylase Type 1 Inhibition | Wang Z et al. | 26667412 | Non-human study |
| 1 | Add-on angiotensin receptor blockade with maximized ACE inhibition | Agarwal R | 11380832 | Not a case-control study |
| 1 | Association of the genetic polymorphisms of the renin-angiotensin system and endothelial nitric oxide synthase with chronic renal transplant dysfunction. | Akcay A et al. | 15385810 | Not a case-control study |
| 1 | Influence of genetic polymorphisms of the renin-angiotensin system on IgA nephropathy | Bantis C et al. | 15031629 | Not a case-control study |
| 1 | Genetic determinants of acute renal damage risk and prognosis: a systematic review | Cardinal-Fernandez P et al. | 22436318 | Not a case-control study |
| 1 | Association between two genetic polymorphisms of the renin-angiotensin-aldosterone system and diabetic nephropathy: a meta-analysis | Ding W et al. | 21607620 | Not a case-control study |
| 1 | A systematic review and meta-analysis of the association between angiotensin II type 1 receptor A1166C gene polymorphism and myocardial infarction susceptibility | Feng X et al. | 23178513 | Not a case-control study |

| | | | | |
|---|---|---|---|---|
| 1 | Polymorphism of angiotensin converting enzyme, angiotensinogen, and angiotensin II type 1 receptor genes and end-stage renal failure in IgA nephropathy: IGARAS - A study of 274 men | Frimat L et al. | 11053482 | Not a case-control study |
| 1 | Survival in type 2 diabetic patients in dialysis and the number of risk alleles in polymorphisms of the renin-angiotensin system genes | Padro-Miquel A et al. | 19014923 | Not a case-control study |
| 1 | A Synergistic Association of ACE I/D and eNOS G894T Gene Variants with the Progression of Immunoglobulin A Nephropathy - A Pilot Study | Rodriguez-Perez JC et al. | 19546528 | Not a case-control study |
| 1 | Impact of genetic polymorphisms of the renin-angiotensin system and of non-genetic factors on kidney transplant function - a single-center experience | Siekierka-Harreis M et al. | 19681973 | Not a case-control study |
| 1 | Analysis of baseline parameters in the HALT polycystic kidney disease trials. | Torres VE et al. | 22205355 | Not a case-control study |
| 1 | Angiotensin-converting enzyme inhibitor versus angiotensin 2 receptor antagonist therapy and the influence of angiotensin-converting enzyme gene polymorphism in IgA nephritis. | Woo KT et al. | 18536822 | Not a case-control study |
| 1 | Gene Polymorphisms of the Renin-AngiotensinAldosterone system and angiotensin 11 type I-Receptor activating antibodies in renal rejection | Zhang G et al. | 17984617 | Not a case-control study |
| 1 | Association of angiotensin II type-1 receptor A1166C gene polymorphism with the susceptibility of end-stage renal disease | Zhou TB et al. | 23971628 | Not a case-control study |
| 1 | Association of angiotensin II type 1 receptor gene A1166C polymorphism with the presence of diabetes mellitus and metabolic syndrome in patients with documented coronary artery disease | Assali A et al. | 21570644 | Not a renal disease focus |
| 1 | Polymorphism in angiotensin II receptor genes and hypertension | Baudin B | 15640279 | Not a renal disease focus |
| 1 | Polymorphisms of the renin-angiotensin system in patients with multifocal renal arterial fibromuscular dysplasia | Bofinger A et al. | 11317203 | Not a renal disease focus |
| 1 | Can the choice of diet undermine the potential genetic risk of AT1R 1166A>C gene polymorphism? | Bozina T et al. | 30205174 | Not a renal disease focus |
| 1 | Renin-angiotensin system gene polymorphisms: assessment of the risk of coronary heart disease. | Buraczynska M et al. | 14502296 | Not a renal disease focus |

| | | | | |
|---|---|---|---|---|
| 1 | Association of renin-angiotensin and endothelial nitric oxide synthase gene polymorphisms with blood pressure progression and incident hypertension: prospective cohort study. | Conen D et al. | 18698212 | Not a renal disease focus |
| 1 | Functional polymorphisms in genes of the Angiotensin and Serotonin systems and risk of hypertrophic cardiomyopathy: AT1R as a potential modifier. | Coto E et al. | 20594303 | Not a renal disease focus |
| 1 | Polymorphisms in genes of the renin-angiotensin-aldosterone system and renal cell cancer risk: Interplay with hypertension and intakes of sodium, potassium and fluid | Deckers IA et al. | 24978482 | Not a renal disease focus |
| 1 | Single nucleotide polymorphism of adiponectin +276 G/T is associated with the susceptibility to essential hypertension in a Turkish population. | Demir AK et al. | 27936341 | Not a renal disease focus |
| 1 | Genetic variation of the renin-angiotensin system and chronic kidney disease progression in black individuals in the atherosclerosis risk in communities study | Hsu CCC et al. | 16396964 | Not a renal disease focus |
| 1 | Genetic variant of the renin-angiotensin system and prevalence of type 2 diabetes mellitus: a modest but significant effect of aldosterone synthase | Ichikawa M et al. | 24549414 | Not a renal disease focus |
| 1 | Renin-Angiotensin System Gene Variants and Type 2 Diabetes Mellitus: Influence of Angiotensinogen | Joyce-Tan SM et al. | 26682227 | Not a renal disease focus |
| 1 | Comprehensive analysis of the renin-angiotensin gene polymorphisms with relation to hypertension in the Japanese | Kato N et al. | 10953993 | Not a renal disease focus |
| 1 | Renin-angiotensin system gene polymorphisms: its impact on IgAN and its progression to end-stage renal failure among Chinese in Singapore. | Lau YK et al. | 15153745 | Not a renal disease focus |
| 1 | Angiotensin II type 1 receptor gene polymorphism and the response to hyperglycemia in early type 1 diabetes | Miller JA et al. | 10969844 | Not a renal disease focus |
| 1 | Deep-targeted exon sequencing reveals renal polymorphisms associate with postexercise hypotension among African Americans | Pescatello LS et al. | 27940662 | Not a renal disease focus |
| 1 | Association between renin-angiotensin-aldosterone system-related genes and blood pressure in a Korean population. | Song SB et al. | 21342026 | Not a renal disease focus |
| 1 | Angiotensin II Type 1A Receptors in Vascular Smooth Muscle Cells Do Not Influence Aortic Remodeling in Hypertension | Sparks MA et al. | 21242463 | Not a renal disease focus |

| | | | | |
|---|---|---|---|---|
| 1 | Peripheral vascular disease in type 2 diabetic Chinese patients: associations with metabolic indices, concomitant vascular disease and genetic factors | Thomas GN et al. | 14632699 | Not a renal disease focus |
| 1 | Genomic association analysis identifies multiple loci influencing antihypertensive response to an angiotensin II receptor blocker. | Turner ST et al. | 22566498 | Not a renal disease focus |
| 1 | The M235T polymorphism in theangiotensinogen gene is associated with the risk of malignant hypertension in white patients | van den Born BJH et al. | 17921816 | Not a renal disease focus |
| 1 | Genetic risk of atherosclerotic renal artery disease - The candidate gene approach in a renal angiography cohort | van Onna M et al. | 15326089 | Not a renal disease focus |
| 1 | Eprosartan modulates the reflex activation of the sympathetic nervous system in sodium restricted healthy humans | Vase H et al. | 18341678 | Not a renal disease focus |
| 1 | Genetic polymorphisms of the angiotensin II type 1 receptor gene and diastolic heart failure | Wu CK et al. | 19330904 | Not a renal disease focus |
| 1 | Increased expression of angiotensin II type 1 receptor (AGTR1) in heart transplant recipients with recurrent rejection | Yamani MH et al. | 17097490 | Not a renal disease focus |
| 1 | [Renin-angiotensin system genes in chronic glomerulonephritis]. | Buraczynska M et al. | 11865575 | Not written in English or Spanish |
| 1 | [Association of the renin-angiotensin system gene polymorphism with nephropathy in type II diabetes]. | Buraczynska M et al. | 12476891 | Not written in English or Spanish |
| 1 | [Genetic predisposition to systemic complications of arterial hypertension in maintenance haemodialysis patients]. | Bzoma B et al. | 19112833 | Not written in English or Spanish |
| 1 | [Polymorphism of gene encoding vascular angiotensin II receptor and microangiopathies in patients with insulin-dependent diabetes mellitus]. | Chistiakov DA et al. | 10576062 | Not written in English or Spanish |
| 1 | [Effect of eprosartan on the hemostatic system in patients with chronic kidney disease associated with hereditary thrombophilia]. | Kaliuzhin VV et al. | 24261234 | Not written in English or Spanish |

| | | | | |
|---|---|---|---|---|
| 1 | [Pharmacogenetic aspects of candesartan application for the treatment of arterial hypertension in patients with chronic pyelonephritis]. | Mormol' IA | 25286611 | Not written in English or Spanish |
| 1 | [Identification of the locus associated with diabetic nephropathy in type 1 diabetes mellitus]. | Savost'ianov KV et al. | 12500539 | Not written in English or Spanish |
| 1 | [AGTR1 A1166C polymorphism is associated with risk of diabetic nephropathy]. | Yin X et al. | 23505107 | Not written in English or Spanish |
| 1 | Donor and recipient ACE I/D genotype are associated with loss of renal function in children following renal transplantation | Buscher R et al. | 21309964 | Paediatric Individuals |
| 1 | Polymorphisms of the angiotensin converting enzyme and angiotensin II type 1 receptor genes and renal scarring in non-uropathic children with recurrent urinary tract infection | Ece A et al. | 16109085 | Paediatric Individuals |
| 1 | Cyclosporine A responsive congenital nephrotic syndrome with single heterozygous variants in NPHS1, NPHS2, and PLCE1 | Eichinger A et al. | 29663071 | Paediatric Individuals |
| 1 | Genetic polymorphism of ACE and the angiotensin II type1 receptor genes in children with chronic kidney disease | Elshamaa MF et al. | 21859496 | Paediatric Individuals |
| 1 | Genetic polymorphisms of the renin-angiotensin system and the outcome of focal segmental glomerulosclerosis in children | Frishberg Y et al. | 9853248 | Paediatric Individuals |
| 1 | Angiotensinogen gene T235 variant: a marker for the development of persistent microalbuminuria in children and adolescents with type 1 diabetes mellitus | Gallego PH et al. | 18413222 | Paediatric Individuals |
| 1 | ACE gene polymorphism and renal scarring in primary vesicoureteric reflux | Haszon I et al. | 12478352 | Paediatric Individuals |
| 1 | Angiotensin type 2 receptor is important in the normal development of the ureter | Hohenfellner K et al. | 10353402 | Paediatric Individuals |
| 1 | Impact of common functional polymorphisms in renin angiotensin system genes on the risk of renal parenchymal scarring following childhood urinary tract infection | Hussein A et al. | 25939993 | Paediatric Individuals |
| 1 | ACE and AT1 receptor gene polymorphisms and renal scarring in urinary bladder dysfunction | Kostic M et al. | 15179569 | Paediatric Individuals |

| | | | | |
|---|---|---|---|---|
| 1 | Renin-angiotensin system polymorphisms in Taiwanese primary vesicoureteral reflux | Liu KP et al. | 15045574 | Paediatric Individuals |
| 1 | Polymorphisms of renin-angiotensin system genes in childhood IgA nephropathy | Maruyama K et al. | 11354780 | Paediatric Individuals |
| 1 | Implications of certain genetic polymorphisms in scarring in vesicoureteric reflux: importance of ACE polymorphism. | Ozen S et al. | 10401028 | Paediatric Individuals |
| 1 | Renin-angiotensin system gene polymorphisms: association with susceptibility to Henoch-Schonlein purpura and renal involvement | Ozkaya O et al. | 16521052 | Paediatric Individuals |
| 1 | Renin-angiotensin gene polymorphism in children with uremia and essential hypertension | Papp F et al. | 12579405 | Paediatric Individuals |
| 1 | Renin-angiotensin system polymorphisms and renal scarring | Pardo R et al. | 12579398 | Paediatric Individuals |
| 1 | Effects of genetic polymorphisms of the renin-angiotensin system in children with nephrotic syndrome. | Tabel Y et al. | 16525944 | Paediatric Individuals |
| 1 | Genetic risk factors in typical haemolytic uraemic syndrome | Taranta A et al. | 19110485 | Paediatric Individuals |
| 1 | Mapping candidate regions and genes for congenital anomalies of the kidneys and urinary tract (CAKUT) by array-based comparative genomic hybridization | Weber S et al. | 20605837 | Paediatric Individuals |
| 1 | Genetic polymorphism of the renin-angiotensin system on the development of primary vesicoureteral reflux | Yim HE et al. | 14764974 | Paediatric Individuals |
| 1 | Estimation of the relationship between the polymorphisms of selected genes: ACE, AGTR1, TGFÎ²1 and GNB3 with the occurrence of primary vesicoureteral reflux. | Zyczkowski M et al. | 27988909 | Paediatric Individuals |
| 1 | Long-term renoprotective effects of losartan in diabetic nephropathy: interaction with ACE insertion/deletion genotype? | Andersen S et al. | 12716812 | Pharmaceutical drug focus |
| 1 | Effect of ACE and AT-2 inhibitors on mortality and progression to microalbuminuria in a nested case-control study of diabetic nephropathy in diabetes mellitus type 2: results from the GENDIAN study. | Boger CA et al. | 16961167 | Pharmaceutical drug focus |
| 1 | Genetic polymorphisms of renin-angiotensin system and progression of interstitial nephritis. | Buraczynska M et al. | 12898858 | Pharmaceutical drug focus |

| | | | | |
|---|---|---|---|---|
| 1 | The influence of renin-angiotensin system genotypes on the antiproteinuric response to high doses of olmesartan in non-diabetic protein uric nephropathies | Goyache-Goni B et al. | 24241364 | Pharmaceutical drug focus |
| 1 | CYP2C9 genotype and pharmacodynamic responses to losartan in patients with primary and secondary kidney diseases. | Joy MS et al. | 19669737 | Pharmaceutical drug focus |
| 1 | Genotypic interactions of renin-angiotensin system genes with diabetes type 2 in a Tunisian population | Mehri S et al. | 20580725 | Pharmaceutical drug focus |
| 1 | Evaluation of Candidate Nephropathy Susceptibility Genes in a Genome-Wide Association Study of African American Diabetic Kidney Disease | Palmer ND et al. | 24551085 | Pharmaceutical drug focus |
| 1 | Chronic renal insufficiency among Asian Indians with type 2 diabetes: I. Role of RAAS gene polymorphisms. | Prasad P et al. | 16672053 | Pharmaceutical drug focus |
| 1 | Enalapril and losartan affect lipid peroxidation in renal transplant recipients with renin-angiotensin system polymorphisms | Rashtchizadeh N et al. | 17222813 | Pharmaceutical drug focus |
| 2 | A polymorphism in the angiotensin II type 1 receptor gene has different effects on the risk of diabetic nephropathy in men and women | Möllsten A et al. | 21316998 | Incorrect patient group |
| 2 | Effects of the genetic polymorphisms of the renin-angiotensin system on focal segmental glomerulosclerosis | Luther Y et al. | 14610337 | No data |
| 2 | The renin--angiotensin system gene polymorphisms and clinicopathological correlations in IgA nephropathy. | Ong-Ajyooth S et al. | 10511770 | No data |
| 2 | Combinational effect of genes for the renin-angiotensin system in conferring susceptibility to diabetic nephropathy. | Osawa N et al. | 17143591 | No data |
| 2 | Lack of synergism between long-term poor glycaemic control and three gene polymorphisms of the renin angiotensin system on risk of developing diabetic nephropathy in type I diabetic patients. | Tarnow L et al. | 10907125 | No data |
| 2 | Gene polymorphisms of angiotensin-converting enzyme and angiotensin II Type 1 receptor among chronic kidney disease patients in a Chinese population | Su SL et al. | 22147663 | Overlap in patient group |
| 2 | Polymorphism of renin-angiotensin system genes in IgA nephropathy | Woo KT et al. | 15504143 | Overlap in patient group |
| 2 | Association of a polymorphism of the apolipoprotein E gene with chronic kidney disease in Japanese individuals with metabolic syndrome | Yoshida T et al. | 19056482 | Overlap in patient group |

| | | | | |
|---|---|---|---|---|
| 3 | Polymorphism of the renin–angiotensin–aldosterone system in patients with chronic allograft dysfunction | Ayed K et al. | 16635753 | < 3 populations reported per SNP |
| 3 | Angiotensin II type 1 receptor gene polymorphism in end-stage renal disease | Buraczynska M et al. | 12187084 | < 3 populations reported per SNP |
| 3 | Genetic polymorphisms of the renin-angiotensin system in end-stage renal disease. | Buraczynska M et al. | 16384824 | < 3 populations reported per SNP |
| 3 | Renin-angiotensin-aldosterone system related gene polymorphisms and urinary total arsenic is related to chronic kidney disease. | Chen WJ et al. | 24907556 | < 3 populations reported per SNP |
| 3 | Hypertension after renal transplantation and polymorphism of genes involved in essential hypertension: ACE, AGT, AT1R and ecNOS | El-Essawy AB et al. | WOS:000174306600003 (11926202) | < 3 populations reported per SNP |
| 3 | Genetic polymorphisms of the renin-angiotensin-aldosterone system and renal insufficiency in essential hypertension | Fabris B et al. | 15662219 | < 3 populations reported per SNP |
| 3 | Relationship between polymorphisms in the renin-angiotensin system and nephropathy in type 2 diabetic patients. | Fradin S et al. | 11938025 | < 3 populations reported per SNP |
| 3 | Susceptibility and progression of end stage renal disease are not associated with angiotensin II type 1 receptor gene polymorphism | Hanna MO et al. | 25316403 | < 3 populations reported per SNP |
| 3 | Renin-angiotensin system component gene polymorphisms in Japanese maintenance haemodialysis patients | Kawada N et al. | WOS:000071880400005 | < 3 populations reported per SNP |
| 3 | GENETIC CLUES TO THE ETIOLOGY OF BALKAN ENDEMIC NEPHROPATHY: INVESTIGATING THE ROLE OF ACE AND AT1R POLYMORPHISMS | Krcunovic Z et al. | WOS:000287217500011 | < 3 populations reported per SNP |
| 3 | Angiotensinogen and angiotensin II type 1 receptor gene polymorphism in patients with autosomal dominant polycystic kidney disease: Effect on hypertension and ESRD | Lee KB et al. | 12950120 | < 3 populations reported per SNP |

| | | | | |
|---|---|---|---|---|
| 3 | Genes involved in the regulation of vascular homeostasis determine renal survival rate in patients with chronic glomerulonephritis | Litovkina O et al. | 24727057 | < 3 populations reported per SNP |
| 3 | Polymorphism of renin-angiotensin system genes in dialysis patients - association with cerebrovascular disease | Losito A et al. | WOS:000179668600025 (12454231) | < 3 populations reported per SNP |
| 3 | Contribution of genetic polymorphism in the renin-angiotensin system to the development of renal complications in insulin-dependent diabetes | Marre M et al. | 9120002 | < 3 populations reported per SNP |
| 3 | Association of angiotensinogen gene T235 variant with progression of immunoglobin A nephropathy in Caucasian patients. | Pei Y et al. | 9259580 | < 3 populations reported per SNP |
| 3 | Polymorphisms of the renin-angiotensin system genes in Brazilian patients with lupus nephropathy. | Sprovieri SR et al. | 15934435 | < 3 populations reported per SNP |
| 3 | Interaction between gene polymorphisms of nitric oxide synthase and renin-angiotensin system in the progression of membranous glomerulonephritis. | Stratta P et al. | 14767013 | < 3 populations reported per SNP |
| 3 | Gene-Gene Interactions in Renin-Angiotensin-Aldosterone System Contributes to End-Stage Renal Disease Susceptibility in a Han Chinese Population | Su SL et al. | 24977181 | < 3 populations reported per SNP |
| 3 | Association of Genetic Variants with Chronic Kidney Disease in Japanese Individuals | Yoshida T et al. | 19406964 | < 3 populations reported per SNP |
| 3 | Genetic polymorphisms and the risk of progressive renal failure in elderly Hungarian patients. | Zsom M et al. | 22111818 | < 3 populations reported per SNP |

**Supplementary Table S3e:** Excluded studies from the *AGTR2* search

*Exclusion stage relates to the stage at which the article was removed as shown in the flow diagram (Supplementary Figure S1e)

| Exclusion Stage | Title | Authors | Pubmed ID | Reason |
|---|---|---|---|---|
| 1 | Association of angiotensinogen gene T235 variant with progression of immunoglobin A nephropathy in Caucasian patients. | Pei Y et al. | 9259580 | No data for AGTR2 |
| 1 | Synergistic effect of angiotensin II type 1 receptor genotype and poor glycaemic control on risk of nephropathy in IDDM. | Doria A et al. | 9389421 | No data for AGTR2 |
| 1 | Lack of synergism between long-term poor glycaemic control and three gene polymorphisms of the renin angiotensin system on risk of developing diabetic nephropathy in type I diabetic patients. | Tarnow L et al. | 10907125 | No data for AGTR2 |
| 1 | Angiotensin II type 1 receptor gene polymorphism and the response to hyperglycemia in early type 1 diabetes. | Miller JA et al. | 10969844 | No data for AGTR2 |
| 1 | Polymorphism of angiotensin converting enzyme, angiotensinogen, and angiotensin II type 1 receptor genes and end-stage renal failure in IgA nephropathy: IGARAS--a study of 274 Men. | Frimat L et al. | 11053482 | No data for AGTR2 |
| 1 | Altered regulation of renal interstitial hydrostatic pressure and the renal renin-angiotensin system in the absence of atrial natriuretic peptide | O'Tierney PF et al. | 18192845 | No data for AGTR2 |
| 1 | The angiotensin type II receptor tonically inhibits angiotensin-converting enzyme in AT2 null mutant mice | Hunley TE et al. | 10652034 | Non-human study |
| 1 | A novel rodent model of pregnancy complications associated with genetically determined angiotensin-converting enzyme (ACE) activity | Mata-Greenwood E et al. | 29360395 | Non-human study |
| 1 | Renin-angiotensin system polymorphisms and renal scarring | Pardo R et al. | 12579398 | Not a case-control study |

| | | | | |
|---|---|---|---|---|
| 1 | Association of the genetic polymorphisms of the renin-angiotensin system and endothelial nitric oxide synthase with chronic renal transplant dysfunction. | Akcay A et al. | 15385810 | Not a case-control study |
| 1 | Whole-genome linkage and association scan in primary, nonsyndromic vesicoureteric reflux. | Cordell HJ et al. | 19959718 | Not a case-control study |
| 1 | Association of angiotensin converting enzyme and angiotensin type 2 receptor gene polymorphisms with renal damage in posterior urethral valves. | Laksmi NK et al. | 20149750 | Not a case-control study |
| 1 | Angiotensin type 2 receptor is important in the normal development of the ureter | Hohenfellner K et al. | 10353402 | Not a renal disease focus |
| 1 | Angiotensin II type 2 receptor gene is not responsible for familial vesicoureteral reflux | Yoneda A et al. | 12187255 | Not a renal disease focus |
| 1 | Genetic polymorphism of the renin-angiotensin system on the development of primary vesicoureteral reflux | Yim HE et al. | 14764974 | Not a renal disease focus |
| 1 | Polymorphism in angiotensin II receptor genes and hypertension | Baudin B et al. | 15640279 | Not a renal disease focus |
| 1 | No evidence for angiotensin type 2 receptor gene polymorphism in intron 1 in patients with coarctation of the aorta and Ullrich-Turner syndrome. | Struwe E et al. | 16944335 | Not a renal disease focus |
| 1 | [Effect of eprosartan on the hemostatic system in patients with chronic kidney disease associated with hereditary thrombophilia]. | Kaliuzhin VV et al. | 24261234 | Not written in English or Spanish |
| 1 | No evidence for AT2R gene derangement in human urinary tract anomalies. | Hiraoka M et al. | 11260384 | Paediatric Individuals |
| 1 | Polymorphisms of renin-angiotensin system genes in childhood IgA nephropathy. | Maruyama K et al. | 11354780 | Paediatric Individuals |
| 1 | Angiotensin-converting enzyme and angiotensin type 2 receptor gene genotype distributions in Italian children with congenital uropathies. | Rigoli L et al. | 15470205 | Paediatric Individuals |
| 1 | Implication of genetic variations in congenital obstructive nephropathy. | Hahn H et al. | 16133060 | Paediatric Individuals |

| | | | | |
|---|---|---|---|---|
| 1 | Angiotensin II type 2 receptor gene polymorphism in Caucasian children with a wide spectrum of congenital anomalies of the kidney and urinary tract | Siomou E et al. | 17515833 | Paediatric Individuals |
| 1 | Association of angiotensin type 2 receptor gene polymorphisms with ureteropelvic junction obstruction in Brazilian patients. | Miranda DM et al. | 24995698 | Paediatric Individuals |
| 2 | Angiotensin-converting enzyme and angiotensin II receptor subtype 2 genotypes in type 1 diabetes and severe hypoglycaemia requiring emergency treatment: a case cohort study. | Pedersen-Bjergaard U et al. | 19820429 | Incorrect patient group |

**Supplementary Table S3f:** Excluded studies from the *REN* search

*Exclusion stage relates to the stage at which the article was removed as shown in the flow diagram (Supplementary Figure S1f)

| Exclusion Stage | Title | Authors | Pubmed ID or WoS ID if Pubmed ID not available | Reason |
|---|---|---|---|---|
| 1 | Increased amount of the angiotensin-converting enzyme (ACE) mRNA originating from the ACE allele with deletion. | Suehiro T et al. | 15164285 | Gene expression based study |
| 1 | Increased expression of monocytic angiotensin-converting enzyme in dialysis patients with cardiovascular disease | Ulrich C et al. | 16476718 | Gene expression based study |
| 1 | Synergistic expression of angiotensin-converting enzyme (ACE) and ACE2 in human renal tissue and confounding effects of hypertension on the ACE to ACE2 ratio | Wakahara S et al. | 17303661 | Gene expression based study |
| 1 | Insertion / Deletion Polymorphism of Angiotensin Converting Enzyme Gene Does Not Contribute to Chronic Kidney Disease in Palestine | Abuaisha AM et al. | WOS:000433049000003 | No data for REN |
| 1 | Relationship between GSTs gene polymorphism and susceptibility to end stage renal disease among North Indians. | Agrawal S et al. | 18067039 | No data for REN |
| 1 | ACE variants interact with the RAS pathway to confer risk and protection against type 2 diabetic nephropathy. | Ahluwalia TS et al. | 19108684 | No data for REN |
| 1 | Analysis of insertion/deletion polymorphisms of the angiotensin converting enzyme gene in Malaysian end-stage renal disease patients. | Ali A et al. | 21421653 | No data for REN |
| 1 | Apparent Mineralocorticoid Excess Caused By A Novel Mutation In 11-Beta Hydroxysteroid Dehydrogenase Type 2 Enzyme: Its Genetics And Response To Therapy | Alzahrani AS et al. | 24936560 | No data for REN |
| 1 | Clinical impact of an angiotensin I-converting enzyme insertion/deletion and kinin B2 receptor +9/-9 polymorphisms in the prognosis of renal transplantation | Amorim CEN et al. | 23362199 | No data for REN |

| | | | | |
|---|---|---|---|---|
| 1 | Analysis of polymorphism in Renin Angiotensin System and other related genes in South Indian chronic kidney disease patients | Anbazhagan K et al. | 19520069 | No data for REN |
| 1 | Circulating angiotensin-converting enzyme 2 activity in patients with chronic kidney disease without previous history of cardiovascular disease | Anguiano L et al. | 25813276 | No data for REN |
| 1 | Lack of association between the angiotensin-converting enzyme gene (I/D) polymorphism and diabetic nephropathy in Tunisian type 2 diabetic patients. | Arfa I et al. | 18404607 | No data for REN |
| 1 | Associations between apolipoprotein E gene polymorphism and plasminogen activator inhibitor-1 and atherogenic lipid profile in dialysis patients. | Arikan H et al. | 17763167 | No data for REN |
| 1 | The DD genotype of the ACE gene polymorphism is associated with diabetic nephropathy in type-1 diabetics | Azar ST et al. | 11428725 | No data for REN |
| 1 | Association of sequence polymorphism in the mitochondrial D-loop with chronic kidney disease. | Bai Y et al. | 24576051 | No data for REN |
| 1 | Impact of aldosterone synthase gene C-344T polymorphism on IgA nephropathy. | Bantis C et al. | 21476902 | No data for REN |
| 1 | Influence of cytokine gene polymorphisms on focal segmental glomerulosclerosis | Bantis C et al. | 15308875 | No data for REN |
| 1 | Influence of aldosterone synthase gene C-344T polymorphism on focal segmental glomerulosclerosis | Bantis C et al. | 21777344 | No data for REN |
| 1 | Identification of Cathepsin L as a Potential Sex-Specific Biomarker for Renal Damage | Bauer Y et al. | 21357272 | No data for REN |
| 1 | Determinants of Progression in Early Autosomal Dominant Polycystic Kidney Disease: Is it Blood Pressure or Renin-Angiotensin-Aldosterone-System Blockade? | Brosnahan GM et al. | 29564978 | No data for REN |
| 1 | Genetic determination of TNF and myeloperoxidase production in dialyzed patients with diabetic nephropathy. | Buraczynska M et al. | 15600254 | No data for REN |

| | | | | |
|---|---|---|---|---|
| 1 | Genetic polymorphisms of the renin-angiotensin system in end-stage renal disease | Buraczynska M et al. | 16384824 | No data for REN |
| 1 | Angiotensin II type 1 receptor gene polymorphism in end-stage renal disease | Buraczynska M et al. | 12187084 | No data for REN |
| 1 | Familial renal glucosuria: SLC5A2 mutation analysis and evidence of salt-wasting | Calado J et al. | 16518345 | No data for REN |
| 1 | Non-relation of parathyroid hormone gene polymorphisms to secondary hyperparathyroidism in Chinese hemodialysis patients. | Chen JB et al. | 15083922 | No data for REN |
| 1 | Effect of IL-6 C-572G polymorphism on idiopathic membranous nephropathy risk in a Han Chinese population. | Chen SY et al. | 20954977 | No data for REN |
| 1 | Lack of association between transient receptor potential cation channel 6 polymorphisms and primary membranous glomerulonephritis. | Chen WC et al. | 20540633 | No data for REN |
| 1 | Renin-angiotensin-aldosterone system related gene polymorphisms and urinary total arsenic is related to chronic kidney disease | Chen WJ et al. | 24907556 | No data for REN |
| 1 | Toll-like receptor 9 SNPs are susceptible to the development and progression of membranous glomerulonephritis: 27 years follow-up in Taiwan. | Chen YT et al. | 23964786 | No data for REN |
| 1 | Endothelial nitric oxide synthase gene polymorphisms and the renal hemodynamic response to L-arginine | Cherney DZI et al. | 19037250 | No data for REN |
| 1 | Influence of uridine diphosphate-glucuronosyltransferases (1A9) polymorphisms on mycophenolic acid pharmacokinetics in patients with renal transplant. | Ciftci HS et al. | 30012031 | No data for REN |
| 1 | Serological and genetic factors in early recurrence of IgA nephropathy after renal transplantation | Coppo R et al. | 17988266 | No data for REN |
| 1 | Association of glutathione S-transferase M1 and T1 gene polymorphism with oxidative stress in diabetic and nondiabetic chronic kidney disease. | Datta SK et al. | 20954980 | No data for REN |
| 1 | ACE, PAI-1, decorin and Warner helicase genes are not associated with the development of renal disease in European patients with Type 1 diabetes | De Cosmo S et al. | 10495473 | No data for REN |
| 1 | U.K. Prospective Diabetes Study. XV: Relationship of renin-angiotensin system gene polymorphisms with microalbuminuria in NIDDM. | Dudley CR et al. | 8587251 | No data for REN |

| | | | | |
|---|---|---|---|---|
| 1 | Angiotensin II type 1 receptor (A1166C) gene polymorphism in Egyptian adult hemodialysis patients | El-Banawy H et al. | WOS:000365886400009 | No data for REN |
| 1 | Mthfr C677T, A1298C And Ace I/D Polymorphisms As Risk Factors For Diabetic Nephropathy Among Type 2 Diabetic Patients. | El-Baz R et al. | 22554825 | No data for REN |
| 1 | Impact of nitric oxide synthase Glu298Asp polymorphism on the development of end-stage renal disease in type 2 diabetic Egyptian patients. | El-Din Bessa SS et al. | 21854353 | No data for REN |
| 1 | Angiotensin-I converting enzyme gene polymorphism in Turkish type 2 diabetic patients | Ergen HA et al. | 15365253 | No data for REN |
| 1 | GAS6 intron 8 c.834 + 7G > A gene polymorphism in diabetic nephropathy. | Erkoc R et al. | 25869052 | No data for REN |
| 1 | DNA polymorphisms in the ACE gene, serum ACE activity and the risk of nephropathy in insulin-dependent diabetes mellitus | Freire MBS et al. | 9794558 | No data for REN |
| 1 | Polymorphism of angiotensin converting enzyme, angiotensinogen, and angiotensin II type 1 receptor genes and end-stage renal failure in IgA nephropathy: IGARAS - A study of 274 men | Frimat L et al. | 11053482 | No data for REN |
| 1 | Polymorphisms in the gene encoding angiotensin I converting enzyme 2 and diabetic nephropathy | Frojdo S et al. | 16211375 | No data for REN |
| 1 | Lack of association between the heparan sulfate proteoglycan gene polymorphism and diabetic nephropathy in Japanese NIDDM with proliferative diabetic retinopathy. | Fujita H et al. | 10586428 | No data for REN |
| 1 | Effects of erythropoietin, angiotensin II, and angiotensin-converting enzyme inhibitor on erythroid precursors in patients with posttransplantation erythrocytosis | Glicklich D et al. | 10428268 | No data for REN |
| 1 | Angiotensinogen gene polymorphisms and progression of chronic kidney disease in ADPKD patients | Gnanasambandan R et al. | 26482465 | No data for REN |
| 1 | Association study of ACE polymorphisms and systemic lupus erythematosus in Northern Chinese Han population | Gong AM et al. | 22729880 | No data for REN |

| | | | | |
|---|---|---|---|---|
| 1 | Angiotensin I converting enzyme and angiotensinogen gene polymorphisms in non-insulin-dependent diabetes mellitus. Lack of relationship with diabetic nephropathy and retinopathy in a Caucasian Mediterranean population | Gutierrez C et al. | 9258285 | No data for REN |
| 1 | Renin-aldosterone response, urinary Na/K ratio and growth in pseudohypoaldosteronism patients with mutations in epithelial sodium channel (ENaC) subunit genes | Hanukoglu A et al. | 18634878 | No data for REN |
| 1 | Polymorphism of the angiotensin I-converting enzyme gene in diabetic nephropathy in type II diabetic patients with proliferative retinopathy. | Hanyu O et al. | 9509566 | No data for REN |
| 1 | Increased frequency of angiotensin-converting enzyme DD genotype in patients with type 2 diabetes in Taiwan | Hsieh MC et al. | 10862639 | No data for REN |
| 1 | Genetic polymorphisms of the renin-angiotensin-aldosterone system in Chinese patients with end-stage renal disease secondary to IgA nephropathy | Huang HD et al. | 21163122 | No data for REN |
| 1 | Impact of Polymorphisms of the Genes Encoding Angiotensin II-Forming Enzymes on the Progression of IgA Nephropathy | Jung ES et al. | 21150220 | No data for REN |
| 1 | Study of the association of -667 aquaporin-2 (AQP-2) A/G promoter polymorphism with the incidence and clinical course of chronic kidney disease in Korea. | Kang SW et al. | 17763164 | No data for REN |
| 1 | Assessment of matrix Gla protein, Klotho gene polymorphisms, and oxidative stress in chronic kidney disease. | Karsli Ceppioglu S et al. | 21859400 | No data for REN |
| 1 | Renin-angiotensin system component gene polymorphisms in Japanese maintenance haemodialysis patients | Kawada N et al. | WOS:000071880400005 | No data for REN |
| 1 | Manganese superoxide dismutase, glutathione peroxidase and catalase gene polymorphisms and clinical outcomes in acute kidney injury. | Kidir V et al. | 26787049 | No data for REN |
| 1 | Blood Pressure-Related Genes and the Progression of IgA Nephropathy | Kim SM et al. | 19729965 | No data for REN |
| 1 | Genetic Clues To The Etiology Of Balkan Endemic Nephropathy: Investigating The Role Of Ace And At1R Polymorphisms | Krcunovic Z et al. | WOS:000287217500011 | No data for REN |

| | | | | |
|---|---|---|---|---|
| 1 | Effect of ACE gene on diabetic nephropathy in NIDDM patients with insulin resistance | Kuramoto N et al. | 10023638 | No data for REN |
| 1 | Association of apolipoprotein E gene polymorphism with end-stage renal disease and hyperlipidemia in patients on long-term hemodialysis. | Lahrach H et al. | 25155022 | No data for REN |
| 1 | Aldosterone synthase (CYP11B2)-344T/C polymorph ism is not associated with the initiation and progression of diabetic nephropathy in Caucasian Type 1 diabetic patients | Lajer M et al. | 16759311 | No data for REN |
| 1 | Renal perfusion and the renal hemodynamic response to blocking the renin system in diabetes - Are the forces leading to vasodilation and vasoconstriction linked? | Lansang MC et al. | 12086929 | No data for REN |
| 1 | Renin-angiotensin system gene polymorphisms: its impact on IgAN and its progression to end-stage renal failure among Chinese in Singapore. | Lau YK et al. | 15153745 | No data for REN |
| 1 | Association studies between the HSD11B2 gene (encoding human 11 beta-hydroxysteroid dehydrogenase type 2), type 1 diabetes mellitus and diabetic nephropathy | Lavery GG et al. | 11916625 | No data for REN |
| 1 | Association of the genetic polymorphisms of the ACE gene and the eNOS gene with lupus nephropathy in northern Chinese population | Li X et al. | 20540812 | No data for REN |
| 1 | The relationship between the TGF-beta1 gene -509C/T polymorphism and tubulointerstitial damage resulting from primary nephrotic syndrome. | Li Y et al. | 20446778 | No data for REN |
| 1 | Genes involved in the regulation of vascular homeostasis determine renal survival rate in patients with chronic glomerulonephritis | Litovkina O et al. | 24727057 | No data for REN |
| 1 | Factors affecting progression of renal insufficiency | Locatelli F et al. | 9387138 | No data for REN |
| 1 | Polymorphism of renin-angiotensin system genes in dialysis patients - association with cerebrovascular disease | Losito A et al. | 12454231 | No data for REN |
| 1 | Genetic polymorphisms of the renin-angiotensin-aldosterone system in end-stage renal disease | Lovati E et al. | 11422735 | No data for REN |

| | | | | |
|---|---|---|---|---|
| 1 | Effects of the genetic polymorphisms of the renin-angiotensin system on focal segmental glomerulosclerosis | Luther Y et al. | 14610337 | No data for REN |
| 1 | Angiotensinogen (AGT) gene missense polymorphisms (rs699 and rs4762) and diabetic nephropathy in Caucasians with type 2 diabetes mellitus. | Makuc J et al. | 28488548 | No data for REN |
| 1 | Impact of interferon-gamma and interleukin-4 gene polymorphisms on development and progression of IgA nephropathy in Japanese patients | Masutani K et al. | 12552499 | No data for REN |
| 1 | Influence of the endothelial nitric oxide synthase polymorphism on the progression of autosomal dominant polycystic kidney disease and IgA nephropathy. | Merta M et al. | 12212826 | No data for REN |
| 1 | Manganese Superoxide Dismutase (SOD2) Polymorphisms, Plasma Advanced Oxidation Protein Products (AOPP) Concentration and Risk of Kidney Complications in Subjects with Type 1 Diabetes | Mohammedi K et al. | 24819633 | No data for REN |
| 1 | The effect of polymorphisms in the renin-angiotensin-aldosterone system on diabetic nephropathy risk | Mollsten A et al. | 18413189 | No data for REN |
| 1 | A polymorphism in the angiotensin II type 1 receptor gene has different effects on the risk of diabetic nephropathy in men and women | Mollsten A et al. | 21316998 | No data for REN |
| 1 | Different Mechanisms for the Progression of CKD with ACE Gene Polymorphisms | Nakayama Y et al. | 19293592 | No data for REN |
| 1 | Pronatriodilatin gene polymorphisms, microvascular permeability, and diabetic nephropathy in type 1 diabetes mellitus | Nannipieri M et al. | 10405209 | No data for REN |
| 1 | A biallelic gene polymorphism of CYP11B2 predicts increased aldosterone to renin ratio in selected hypertensive patients. | Nicod J et al. | 12788845 | No data for REN |
| 1 | Role of the alpha-adducin genotype on renal disease progression | Nicod J et al. | 11918733 | No data for REN |
| 1 | Association between CCDC132, FDX1 and TNFSF13 gene polymorphisms and the risk of IgA nephropathy. | Niu D et al. | 26370181 | No data for REN |
| 1 | Chemerin rs17173608 and vaspin rs2236242 gene variants on the risk of end stage renal disease (ESRD) and correlation with plasma malondialdehyde (MDA) level. | Nomani H et al. | 29644922 | No data for REN |

| | | | | |
|---|---|---|---|---|
| 1 | Renal clearance of endogenous erythropoietin in patients with proteinuria. | Nowicki M et al. | 7759206 | No data for REN |
| 1 | The renin--angiotensin system gene polymorphisms and clinicopathological correlations in IgA nephropathy. | Ong-Ajyooth S et al. | 10511770 | No data for REN |
| 1 | Angiotensin-converting enzyme polymorphism gene and evolution of nephropathy to end-stage renal disease | Ortiz MA et al. | 15012717 | No data for REN |
| 1 | Combinational effect of genes for the renin-angiotensin system in conferring susceptibility to diabetic nephropathy | Osawa N et al. | 17143591 | No data for REN |
| 1 | Bcii--RFLP profiles for serum amiloid A1 and mutated MEFV gene prevalence in chronic renal failure patients requiring long-term hemodialysis. | Ozdemir O et al. | 25394530 | No data for REN |
| 1 | Survival in type 2 diabetic patients in dialysis and the number of risk alleles in polymorphisms of the renin-angiotensin system genes | Padro-Miquel A et al. | 19014923 | No data for REN |
| 1 | Association of aldosterone synthase (CYP11B2) gene -344T/C polymorphism with the risk of primary chronic glomerulonephritis in the Polish population. | Pawlik M et al. | 23681285 | No data for REN |
| 1 | Does copy number variation of APOL1 gene affect the susceptibility to focal segmental glomerulosclerosis? | Peng T et al. | 28494221 | No data for REN |
| 1 | Association analysis of ADPRT1, AKR1B1, RAGE, GFPT2 and PAI-1 gene polymorphisms with chronic renal insufficiency among Asian Indians with type-2 diabetes | Prasad P et al. | 20353610 | No data for REN |
| 1 | DNA repair genes XPD and XRCC1 polymorphisms and risk of end-stage renal disease in Egyptian population. | Radwan WM et al. | 25310768 | No data for REN |
| 1 | ACE I/D and MMP-7 A-181G variants and the risk of end stage renal disease | Rahimi Z et al. | 28447048 | No data for REN |
| 1 | Influence of angiotensin converting enzyme (ACE) gene rs4362 polymorphism on the progression of kidney failure in patients with autosomal dominant polycystic kidney disease (ADPKD) | Ramanathan G et al. | 27748299 | No data for REN |

| | | | | |
|---|---|---|---|---|
| 1 | Aldosterone synthase gene is not a major susceptibility gene for progression of chronic kidney disease in patients with autosomal dominant polycystic kidney disease | Ramanathan G et al. | 28540892 | No data for REN |
| 1 | AGTR1 rs5186 variants in patients with type 2 diabetes mellitus and nephropathy | Razi F et al. | WOS:000419720600009 | No data for REN |
| 1 | Association of the angiotensinogen M235T and APO E gene polymorphisms in Turkish type 2 diabetic patients with and without nephropathy. | Reis KA et al. | 21500980 | No data for REN |
| 1 | The influence of the endothelin-converting enzyme-1 gene polymorphism on the progression of autosomal dominant polycystic kidney disease. | Reiterova J et al. | 16526315 | No data for REN |
| 1 | The influence of G-protein beta3-subunit gene and endothelial nitric oxide synthase gene in exon 7 polymorphisms on progression of autosomal dominant polycystic kidney disease. | Reiterova J et al. | 15287194 | No data for REN |
| 1 | Role of ADDUCIN Gly460Trp, ACE I/D and AGT M235T Gene Polymorphisms in Genetic Susceptibility to Diabetic Nephropathy | Sancakdar E et al. | WOS:000367541400005 | No data for REN |
| 1 | Angiotensin I converting enzyme gene polymorphism and diabetic nephropathy in type II diabetes | Schmidt S et al. | 9269698 | No data for REN |
| 1 | ACACÎ² gene (rs2268388) and AGTR1 gene (rs5186) polymorphism and the risk of nephropathy in Asian Indian patients with type 2 diabetes. | Shah VN et al. | 23081748 | No data for REN |
| 1 | Genetic variants of ACE (Insertion/Deletion) and AGT (M268T) genes in patients with diabetes and nephropathy | Shaikh R et al. | 24737640 | No data for REN |
| 1 | Distribution of ACE I/D Polymorphism in the Patients of Diabetes and Nephropathy in Pakistan | Shaikh R et al. | WOS:000312053000001 | No data for REN |
| 1 | The relationship between genetic and haemodynamic factors in diabetic nephropathy (DN): Case-control study in type 1 diabetes mellitus (T1DM) | Shestakova MV et al. | WOS:000242410600010 | No data for REN |

| | | | | |
|---|---|---|---|---|
| 1 | SIRTUIN 1 gene polymorphisms are associated with cholesterol metabolism and coronary artery calcification in Japanese hemodialysis patients. | Shimoyama Y et al. | 22200427 | No data for REN |
| 1 | Interleukin 1 receptor antagonist and tumor necrosis factor-alpha gene polymorphism in patients with end-stage renal failure. | Shu KH et al. | 15717635 | No data for REN |
| 1 | Angiotensin-converting enzyme (ACE) inhibition in type 2, diabetic patients - interaction with ACE insertion/deletion polymorphism | So WY et al. | 16395257 | No data for REN |
| 1 | Vitamin D binding protein and the need for vitamin D in hemodialysis patients. | Speeckaert MM et al. | 18721734 | No data for REN |
| 1 | Polymorphisms of the renin-angiotensin system genes in Brazilian patients with lupus nephropathy | Sprovieri SRS et al. | 15934435 | No data for REN |
| 1 | Prospective study on the potential of RAAS blockade to halt renal disease in Alport syndrome patients with heterozygous mutations. | Stock J et al. | 27402170 | No data for REN |
| 1 | Interaction between gene polymorphisms of nitric oxide synthase and renin-angiotensin system in the progression of membranous glomerulonephritis | Stratta P et al. | 14767013 | No data for REN |
| 1 | Angiotensin II receptor type 1 A1166C modifies the association between angiotensinogen M235T and chronic kidney disease | Su SL et al. | 29296205 | No data for REN |
| 1 | Gene polymorphisms of angiotensin-converting enzyme and angiotensin II Type 1 receptor among chronic kidney disease patients in a Chinese population | Su SL et al. | 22147663 | No data for REN |
| 1 | Gene-Gene Interactions in Renin-Angiotensin-Aldosterone System Contributes to End-Stage Renal Disease Susceptibility in a Han Chinese Population | Su SL et al. | 24977181 | No data for REN |
| 1 | Study on 3'-UTR length polymorphism in peripheral blood mononuclear cells of uremia patient. | Sui W et al. | 26554293 | No data for REN |
| 1 | Angiotensin-II type 1 receptor gene polymorphism and diabetic microangiopathy. | Tarnow L et al. | 8671962 | No data for REN |

| | | | | |
|---|---|---|---|---|
| 1 | Lack Of Relationship Between An Insertion Deletion Polymorphism In The Angiotensin I-Converting Enzyme Gene And Diabetic Nephropathy And Proliferative Retinopathy In Iddm Patients | Tarnow L et al. | 7729604 | No data for REN |
| 1 | Lack of synergism between long-term poor glycaemic control and three gene polymorphisms of the renin angiotensin system on risk of developing diabetic nephropathy in Type I diabetic patients | Tarnow L et al. | 10907125 | No data for REN |
| 1 | High prevalence of ACE DD genotype among north Indian end stage renal disease patients. | Tripathi G et al. | 17042963 | No data for REN |
| 1 | Vitamin D receptor genetic variants among patients with end-stage renal disease. | Tripathi G et al. | 20722565 | No data for REN |
| 1 | Association between angiotensin converting enzyme gene polymorphism and clinical features in autosomal dominant polycystic kidney disease | Uemasu J et al. | 9180368 | No data for REN |
| 1 | The DD genotype of the ACE gene polymorphism is associated with progression of diabetic nephropathy to end stage renal failure in IDDM | Vleming LJ et al. | 10099885 | No data for REN |
| 1 | Relationship between angiotensinogen gene M235T variant with diabetic nephropathy in Chinese NIDDM. | Wang J et al. | 11717948 | No data for REN |
| 1 | Nodular glomerulosclerosis and renin angiotensin system in Chinese patients with type 2 diabetes | Wang M et al. | 26973293 | No data for REN |
| 1 | Predicting the development of diabetic nephropathy and its progression | William J et al. | 15822056 | No data for REN |
| 1 | Polymorphism of renin-angiotensin system genes in IgA nephropathy | Woo KT et al. | 15504143 | No data for REN |
| 1 | Platelet glycoprotein IIb HPA-3 a/b polymorphism is associated with native arteriovenous fistula thrombosis in chronic hemodialysis patients. | Wu JH et al. | 22880801 | No data for REN |
| 1 | Association of plasminogen activator inhibitor-1 gene polymorphism and type 2 diabetic nephropathy. | Xu F et al. | 26616527 | No data for REN |
| 1 | A candidate gene approach to genetic contributors to the development of IgA nephropathy | Yamamoto R et al. | 21737517 | No data for REN |

| | | | | |
|---|---|---|---|---|
| 1 | Impact of interaction of cigarette smoking with angiotensin-converting enzyme polymorphisms on end-stage renal disease risk in a Han Chinese population. | Yang HY et al. | 23477970 | No data for REN |
| 1 | Angiotensin converting enzyme gene polymorphism and development of post-transplant erythrocytosis. | Yildiz A et al. | 12832741 | No data for REN |
| 1 | Aldosterone Synthase CYP11B2 Gene Promoter Polymorphism in a Turkish Population With Chronic Kidney Disease | Yilmaz M et al. | 25957425 | No data for REN |
| 1 | Structural analysis of the 11 beta-hydroxysteroid dehydrogenase type 2 gene in end-stage renal disease | Zaehner T et al. | 11012876 | No data for REN |
| 1 | Arg913Gln of SLC12A3 gene promotes development and progression of end-stage renal disease in Chinese type 2 diabetes mellitus | Zhang R et al. | 28744814 | No data for REN |
| 1 | Pin1 and secondary hyperparathyroidism of chronic kidney disease: gene polymorphisms and protein levels. | Zhao Y et al. | 27876426 | No data for REN |
| 1 | Polymorphisms in NADPH oxidase CYBA gene modify the risk of ESRD in patients with chronic glomerulonephritis. | Zhou H et al. | 26627442 | No data for REN |
| 1 | Angiotensinogen M235T and chymase gene CMA/B polymorphisms are not associated with nephropathy in type II diabetes | Zychma MJ et al. | 11096141 | No data for REN |
| 1 | Effects of Diets with Different Proportions of Protein/Carbohydrate on Retinal Manifestations in db Mice | Arimura E et al. | 29475908 | Non-human study |
| 1 | Effects of plasma kallikrein deficiency on haemostasis and thrombosis in mice: Murine Ortholog of the Fletcher Trait | Bird JE et al. | 22398951 | Non-human study |
| 1 | Blood pressure and renin-angiotensin system resetting in transgenic rats with elevated plasma Val(5)-angiotensinogen | Bohlender J et al. | 22728903 | Non-human study |
| 1 | Angiotensin II mesenteric and renal vasoregulation: Dissimilar modulatory effects with nitroprusside | Broome M et al. | 11065204 | Non-human study |
| 1 | N-domain angiotensin I-converting enzyme expression in renal artery of Wistar, Wistar Kyoto, and spontaneously hypertensive rats | Bueno V et al. | 15194348 | Non-human study |
| 1 | Adrenomedullin gene expression differences in mice do not affect blood pressure but modulate hypertension-induced pathology in males | Caron K et al. | 17360661 | Non-human study |

| | | | | |
|---|---|---|---|---|
| 1 | Appropriate regulation of renin and blood pressure in 45-kb human renin/human angiotensinogen transgenic mice. | Catanzaro DF et al. | 9931123 | Non-human study |
| 1 | Vascular and renal effects of vasopeptidase inhibition and angiotensin-converting enzyme blockade in spontaneously diabetic Goto-Kakizaki rats | Cheng ZJ et al. | 16093923 | Non-human study |
| 1 | Mice lacking endothelial ACE - Normal blood pressure with elevated angiotensin | Cole JM et al. | 12574101 | Non-human study |
| 1 | Thyroid hormones stimulate renal expression of CFTR | de Andrade Pinto AC et al. | 17595518 | Non-human study |
| 1 | Renin-angiotensin system transgenic mouse model recapitulates pathophysiology similar to human preeclampsia with renal injury that may be mediated through VEGF. | Denney JM et al. | 27927648 | Non-human study |
| 1 | Cardiac angiotensin-(1-12) expression and systemic hypertension in rats expressing the human angiotensinogen gene. | Ferrario CM et al. | 26873967 | Non-human study |
| 1 | Attenuation of accelerated renal cystogenesis in Pkd1 mice by renin-angiotensin system blockade | Fitzgibbon WR et al. | 29021226 | Non-human study |
| 1 | Renal cyst growth is the main determinant for hypertension and concentrating deficit in Pkd1-deficient mice | Fonseca JM et al. | 24429399 | Non-human study |
| 1 | Adenosine A(1) receptor-dependent and independent pathways in modulating renal vascular responses to angiotensin II | Gao X et al. | 25251152 | Non-human study |
| 1 | Collecting duct-specific knockout of nitric oxide synthase 3 impairs water excretion in a sex-dependent manner | Gao Y et al. | 27707708 | Non-human study |
| 1 | Metformin prevents the impairment of endothelium-dependent vascular relaxation induced by high glucose challenge in rabbit isolated perfused kidneys | Gomes MB et al. | 16133490 | Non-human study |
| 1 | Effect Of Nitric-Oxide On Renin Secretion .1. Studies In Isolated Juxtaglomerular Granular Cells | Greenberg SG et al. | 7771523 | Non-human study |

| | | | | |
|---|---|---|---|---|
| 1 | Effect of the angiotensinogen genotype on experimental hypertension in mice | Handtrack C et al. | 17333097 | Non-human study |
| 1 | Pituitary adenylate cyclase-activating polypeptide stimulates renin secretion via activation of PAC1 receptors | Hautmann M et al. | 17360952 | Non-human study |
| 1 | The genetic deletion of Mas abolishes salt induced hypertension in mice | Heringer-Walther S et al. | 22652430 | Non-human study |
| 1 | An essential role of angiotensin II receptor type 1a in recipient kidney, not in transplanted peripheral blood leukocytes, in progressive immune-mediated renal injury | Hisada Y et al. | 11555672 | Non-human study |
| 1 | The angiotensin type II receptor tonically inhibits angiotensin-converting enzyme in AT2 null mutant mice | Hunley TE et al. | 10652034 | Non-human study |
| 1 | Collecting Duct Nitric Oxide Synthase 1 beta Activation Maintains Sodium Homeostasis During High Sodium Intake Through Suppression of Aldosterone and Renal Angiotensin II Pathways | Hyndman KA et al. | 29066445 | Non-human study |
| 1 | Transfer of a salt-resistant renin allele raises blood pressure in Dahl salt-sensitive rats | Jiang J et al. | 9040448 | Non-human study |
| 1 | Angiotensin-converting enzyme inhibition attenuates the progression of rat hepatic fibrosis | Jonsson JR et al. | 11438504 | Non-human study |
| 1 | Rat Ace allele variation determines susceptibility to AngII-induced renal damage | Kamilic J et al. | 21788250 | Non-human study |
| 1 | Regulation of renin secretion and expression in mice deficient in ss 1- and ss 2-adrenergic receptors | Kim SM et al. | 17515456 | Non-human study |
| 1 | Low Blood Pressure in Endothelial Cell-Specific Endothelin 1 Knockout Mice | Kisanuki YY et al. | 20516397 | Non-human study |
| 1 | Lack of an effect of collecting duct-specific deletion of adenylyl cyclase 3 on renal Na+ and water excretion or arterial pressure | Kittikulsuth W et al. | 24431204 | Non-human study |

| | | | | |
|---|---|---|---|---|
| 1 | A High Fat Diet During Pregnancy and Lactation Induces Cardiac and Renal Abnormalities in GLUT4+/- Male Mice | Kruse M et al. | 28750406 | Non-human study |
| 1 | Blood pressure and renal hemodynamic responses to acute angiotensin II infusion are enhanced in a female mouse model of systemic lupus erythematosus | Kumari S et al. | 27350671 | Non-human study |
| 1 | Control of renin secretion from kidneys with renin cell hyperplasia | Kurt B et al. | 24285498 | Non-human study |
| 1 | Reciprocal expression of connexin 40 and 45 during phenotypical changes in renin-secreting cells | Kurt B et al. | 21209011 | Non-human study |
| 1 | Stimulation of renin secretion by NO donors is related to the cAMP pathway | Kurtz A et al. | 9575895 | Non-human study |
| 1 | Stimulation of renin secretion by nitric oxide is mediated by phosphodiesterase 3 | Kurtz A et al. | 9539809 | Non-human study |
| 1 | Replacement of connexin 40 by connexin 45 causes ectopic localization of renin-producing cells in the kidney but maintains in vivo control of renin gene expression | Kurtz L et al. | 19474190 | Non-human study |
| 1 | Interference with GsÎ±-Coupled Receptor Signaling in Renin-Producing Cells Leads to Renal Endothelial Damage. | Lachmann P et al. | 28775003 | Non-human study |
| 1 | The angiotensin II receptor blocker candesartan improves survival and mesenteric perfusion in an acute porcine endotoxin model | Laesser M et al. | 14995942 | Non-human study |
| 1 | Mice with targeted disruption of the acyl-CoA binding protein display attenuated urine concentrating ability and diminished renal aquaporin-3 abundance | Langaa S et al. | 22237802 | Non-human study |
| 1 | Physiological impact of increased expression of the AT(1) angiotensin receptor | Le TH et al. | 12963678 | Non-human study |
| 1 | Genetic analysis of the S-A and Na+/K+-ATPase alpha(1) genes in the Milan hypertensive rat | Lodwick D et al. | 9535139 | Non-human study |

| | | | | |
|---|---|---|---|---|
| 1 | Hypertension in unilaterally nephrectomized rats induced by single-kidney transfection with angiotensinogen cDNA | Marley WS et al. | 10567853 | Non-human study |
| 1 | Cardiovascular dysfunction in Zucker obese and Zucker diabetic fatty rats: role of hydronephrosis | Marsh SA et al. | 17351065 | Non-human study |
| 1 | A novel rodent model of pregnancy complications associated with genetically determined angiotensin-converting enzyme (ACE) activity | Mata-Greenwood E et al. | 29360395 | Non-human study |
| 1 | Chronic hypertension and altered baroreflex responses in transgenic mice containing the human renin and human angiotensinogen genes | Merrill DC et al. | 8613528 | Non-human study |
| 1 | Hypervolemia of pregnancy is not maintained in mice chronically overexpressing angiotensinogen | Morgan TK et al. | 16796982 | Non-human study |
| 1 | Vascular angiotensin-converting enzyme expression regulates local angiotensin II | Muller DN et al. | 9039087 | Non-human study |
| 1 | Angiotensin-Ii Enhances Norepinephrine Spillover During Sympathetic Activation In Conscious Rabbits | Noshiro T et al. | 8203585 | Non-human study |
| 1 | Effect Of Angiotensin-Converting Enzyme-Inhibition On Renal Norepinephrine Spillover Rate And Baroreflex Responses In Conscious Rabbits | Noshiro T et al. | 1648463 | Non-human study |
| 1 | Altered regulation of renal interstitial hydrostatic pressure and the renal renin-angiotensin system in the absence of atrial natriuretic peptide | O'Tierney PF et al. | 18192845 | Non-human study |
| 1 | Gene Trapping Uncovers Sex-Specific Mechanisms for Upstream Stimulatory Factors 1 and 2 in Angiotensinogen Expression | Park S et al. | 22547438 | Non-human study |
| 1 | Knockdown of parathyroid hormone related protein in smooth muscle cells alters renal hemodynamics but not blood pressure | Raison D et al. | 23720345 | Non-human study |
| 1 | Nephron-specific deletion of the prorenin receptor causes a urine concentration defect | Ramkumar N et al. | 25995108 | Non-human study |
| 1 | Alternative splicing of vitamin D-24-hydroxylase: a novel mechanism for the regulation of extrarenal 1,25-dihydroxyvitamin D synthesis. | Ren S et al. | 15788398 | Non-human study |

| | | | | |
|---|---|---|---|---|
| 1 | 20-Hydroxyeicosatetraenoic Acid (HETE)-dependent Hypertension in Human Cytochrome P450 (CYP) 4A11 Transgenic Mice NORMALIZATION OF BLOOD PRESSURE BY SODIUM RESTRICTION, HYDROCHLOROTHIAZIDE, OR BLOCKADE OF THE TYPE 1 ANGIOTENSIN II RECEPTOR | Savas S et al. | 27298316 | Non-human study |
| 1 | Parallel regulation of renin and lysosomal integral membrane protein 2 in renin-producing cells: further evidence for a lysosomal nature of renin secretory vesicles | Schmid J et al. | 23229015 | Non-human study |
| 1 | Stimulation of renin release by prostaglandin E(2) is mediated by EP(2) and EP(4) receptors in mouse kidneys | Schweda F et al. | 15113745 | Non-human study |
| 1 | Preserved macula densa-dependent renin secretion in A(1) adenosine receptor knockout mice | Schweda F et al. | 12475747 | Non-human study |
| 1 | Angiotensin converting enzyme (ACE) gene expression in experimentally induced liver cirrhosis in rats | Shahid SM et al. | 24035938 | Non-human study |
| 1 | Elevated blood pressures in mice lacking endothelial nitric oxide synthase | Shesely EG et al. | 8917564 | Non-human study |
| 1 | Endothelium-Dependent Relaxation In The Isolated Rat-Kidney - Impairment By Cyclosporine-A | Stephan D et al. | 8606521 | Non-human study |
| 1 | Local Renal Circadian Clocks Control Fluid-Electrolyte Homeostasis and BP | Tokonami N et al. | 24652800 | Non-human study |
| 1 | Functional genetic variation in aminopeptidase A (ENPEP): Lack of clear association with focal and segmental glomerulosclerosis (FSGS) | Tonna S et al. | 18206321 | Non-human study |
| 1 | Antihypertensive Role of Tissue Kallikrein in Hyperaldosteronism in the Mouse | Waeckel L et al. | 22669897 | Non-human study |
| 1 | Role of cGMP-kinase II in the control of renin secretion and renin expression | Wagner C et al. | 9788971 | Non-human study |
| 1 | GPR48 Increases Mineralocorticoid Receptor Gene Expression | Wang J et al. | 22135314 | Non-human study |
| 1 | Compensatory up-regulation of angiotensin II subtype 1 receptors in alpha ENaC knockout heterozygous mice | Wang Q et al. | 11380824 | Non-human study |

| | | | | |
|---|---|---|---|---|
| 1 | Nebivolol treatment improves resistant arterial function and reduces ventricular hypertrophy and angiotensin II in spontaneously hypertension rats | Wang Y et al. | 23263161 | Non-human study |
| 1 | The elevated blood pressure of human GRK4 gamma A142V transgenic mice is not associated with increased ROS production | Wang Z et al. | 17259440 | Non-human study |
| 1 | Role of neutral endopeptidase 24.11 in AV fistular rat model of heart failure | Wegner M et al. | 8759244 | Non-human study |
| 1 | Role of angiotensin-converting enzyme (ACE and ACE2) imbalance on tourniquet-induced remote kidney injury in a mouse hindlimb ischemia-reperfusion model | Yang XH et al. | 22580272 | Non-human study |
| 1 | Effect of SWL on renal hemodynamics: could a change in renal artery contraction-relaxation responses be the cause? | Yilmaz E et al. | 22945811 | Non-human study |
| 1 | Cosegregation of spontaneously hypertensive rat renin gene with elevated blood pressure in an F-2 generation | Yu H et al. | 9794718 | Non-human study |
| 1 | Role of blood pressure and the renin-angiotensin system in development of diabetic nephropathy (DN) in eNOS(-/-) db/db mice | Zhang MZ et al. | 22114203 | Non-human study |
| 1 | Dendritic cell nuclear protein-1, a novel depression-related protein, upregulates corticotropin-releasing hormone expression. | Zhou T et al. | 20693543 | Non-human study |
| 1 | Angiotensin-converting enzyme genotype is a predictive factor in the peak panel-reactive antibody response | Akcay A et al. | 15013293 | Not a case-control study |
| 1 | Association of the genetic polymorphisms of the renin-angiotensin system and endothelial nitric oxide synthase with chronic renal transplant dysfunction | Akcay A et al. | 15385810 | Not a case-control study |
| 1 | Randomized Controlled Trial: Lisinopril Reduces Proteinuria, Ammonia, and Renal Polypeptide Tubular Catabolism in Patients With Chronic Allograft Nephropathy | Amara AB et al. | 20061926 | Not a case-control study |
| 1 | Influence of cytokine gene polymorphisms on IgA nephropathy. | Bantis C et al. | 18300111 | Not a case-control study |
| 1 | Influence of genetic polymorphisms of the renin-angiotensin system on IgA nephropathy | Bantis C et al. | 15031629 | Not a case-control study |

| | | | | |
|---|---|---|---|---|
| 1 | Angiotensin-converting-enzyme insertion/deletion genotype and long-term renal allograft survival | Beige J et al. | 9550656 | Not a case-control study |
| 1 | Angiotensin-converting enzyme genotype and renal allograft survival | Beige J et al. | 9259361 | Not a case-control study |
| 1 | Deletion insertion polymorphism of the angiotensin converting enzyme gene and progression of diabetic nephropathy | Bjorck S et al. | 9269704 | Not a case-control study |
| 1 | Angiotensinogen concentrations and renin clearance : implications for blood pressure regulation. | Bohlender J et al. | 10720595 | Not a case-control study |
| 1 | Genetic polymorphisms of renin-angiotensin system and progression of interstitial nephritis. | Buraczynska M et al. | 12898858 | Not a case-control study |
| 1 | The Epithelial Sodium Channel gamma-Subunit Gene and Blood Pressure Family Based Association, Renal Gene Expression, and Physiological Analyses | Busst CJ et al. | 22006290 | Not a case-control study |
| 1 | Twenty-one additional cases of familial renal glucosuria: absence of genetic heterogeneity, high prevalence of private mutations and further evidence of volume depletion | Calado J et al. | 18622023 | Not a case-control study |
| 1 | Discovery of a novel dominant mutation in the REN gene after forty years of renal disease: a case report | Clissold RL et al. | 28701203 | Not a case-control study |
| 1 | Disorders of mineralocorticoid synthesis | Connell JMC et al. | 11469810 | Not a case-control study |
| 1 | Successful treatment of decompensated chronic viral hepatitis by bursal disease virus vaccine | Csatary LK et al. | 10216467 | Not a case-control study |
| 1 | Natriuretic peptides buffer renin-dependent hypertension | Demerath T et al. | 24717731 | Not a case-control study |
| 1 | Association between two genetic polymorphisms of the renin-angiotensin-aldosterone system and diabetic nephropathy: a meta-analysis | Ding W et al. | 21607620 | Not a case-control study |
| 1 | The effects of angiotensin-converting enzyme gene polymorphism on the progression of immunoglobulin A nephropathy in Malaysian patients | Draman CR et al. | 19037561 | Not a case-control study |

| | | | | |
|---|---|---|---|---|
| 1 | Juxtaglomerular cell tumor of the kidney: Report of a non-functioning variant | Endoh Y et al. | 9211527 | Not a case-control study |
| 1 | Prograf produces more benefits for CYP3A5 low expression patients in early stage after kidney transplantation. | Fan B et al. | 28157649 | Not a case-control study |
| 1 | Diabetes, nephropathy, and the renin system | Hollenberg NK | 16601578 | Not a case-control study |
| 1 | Association between Angiotensin I-Converting Enzyme Insertion/Deletion Polymorphism and Prognosis of Kidney Transplantation: A Meta-Analysis | Huang ZK et al. | 26000752 | Not a case-control study |
| 1 | Is there a role of angiotensin-converting enzyme gene polymorphism in the failure of arteriovenous femoral shunts for hemodialysis? | Isbir CS et al. | 11525534 | Not a case-control study |
| 1 | Hyperuricemia, Acute and Chronic Kidney Disease, Hypertension, and Cardiovascular Disease: Report of a Scientific Workshop Organized by the National Kidney Foundation | Johnson RJ et al. | 29496260 | Not a case-control study |
| 1 | Diagnosis of a case of Gitelman's syndrome based on renal clearance studies and gene analysis of a novel mutation of the thiazide-sensitive Na-Cl cotransporter | Kageyama K et al. | 16370563 | Not a case-control study |
| 1 | Paricalcitol as an Antiproteinuric Agent Can Result in the Deterioration of Renal and Heart Function in a Patient with Fabry Disease | Keber T et al. | 28596512 | Not a case-control study |
| 1 | The role of renin-angiotensin-aldosterone system genes in the progression of chronic kidney disease: findings from the Chronic Renal Insufficiency Cohort (CRIC) study. | Kelly TN et al. | 25906781 | Not a case-control study |
| 1 | Smoking has no impact on survival and it is not associated with ACE gene I/D polymorphism in hemodialysis patients. | Kiss I et al. | 28058974 | Not a case-control study |
| 1 | ACE gene polymorphism in focal segmental glomerulosclerosis and membranous glomerulonephritis - Is observed difference of clinical significance? | Kuzmanic D et al. | WOS:000087339600012 | Not a case-control study |
| 1 | Antihypertensive treatment modulates the association between the D/I ACE gene polymorphism and left ventricular hypertrophy: a meta-analysis | Kuznetsova T et al. | 10918550 | Not a case-control study |

| | | | | |
|---|---|---|---|---|
| 1 | Bilateral high origins of testicular arteries: a rare variant. | Li J et al. | 22732819 | Not a case-control study |
| 1 | A Genetic Variant in the Distal Enhancer Region of the Human Renin Gene Affects Renin Expression | Makino Y et al. | 26366736 | Not a case-control study |
| 1 | Genetics and the prediction of complications in type 1 diabetes | Marre M | 10097900 | Not a case-control study |
| 1 | Angiotensin converting enzyme gene polymorphism and renal hemodynamic function in early diabetes | Miller JA et al. | 8995725 | Not a case-control study |
| 1 | The kidney in diabetes: How to control renal and related cardiovascular complications | Mogensen CE | 11158852 | Not a case-control study |
| 1 | A female with X-linked Alport syndrome and compound heterozygous COL4A5 mutations | Mohammad M et al. | 24337245 | Not a case-control study |
| 1 | Catalase activity, allelic variations in the catalase gene and risk of kidney complications in patients with type 1 diabetes | Mohammedi K et al. | 24057136 | Not a case-control study |
| 1 | Renal Tubular Dysgenesis in Israel: Pathologist's Experience and Literature Review | Moldavsky M | 19344005 | Not a case-control study |
| 1 | Tip lesion variant of primary focal and segmental glomerulosclerosis: clinicopathological analysis of 20 cases. | Mungan S et al. | 25857429 | Not a case-control study |
| 1 | Genome-Wide Association Analysis of Plasma B-Type Natriuretic Peptide in Blacks The Jackson Heart Study | Musani SK et al. | 25561047 | Not a case-control study |
| 1 | Angiotensinogen gene variation and renoprotective efficacy of renin-angiotensin system blockade in IgA nephropathy | Narita I et al. | 12911556 | Not a case-control study |
| 1 | Renoprotective efficacy of renin-angiotensin inhibitors in IgA nephropathy is influenced by ACE A2350G polymorphism | Narita I et al. | 14684698 | Not a case-control study |
| 1 | Correlates of ACE activity in macroalbuminuric type 2 diabetic patients treated with chronic ACE inhibition. | Nikzamir et al. | 17986476 | Not a case-control study |
| 1 | Epidemiology and genetics of calcific aortic valve disease | O'Brien KD | 17963677 | Not a case-control study |

| | | | | |
|---|---|---|---|---|
| 1 | Impact of polymorphisms in the renin-angiotensin-aldosterone system on hypertrophic cardiomyopathy | Orenes-Pinero E et al. | 21507890 | Not a case-control study |
| 1 | Relationships between HLA-A, -B, -DQ and -DR antigens and interstitial fibrosis in renal allografts. | Ozdemir BH et al. | 15354972 | Not a case-control study |
| 1 | Renin-angiotensin system gene polymorphisms predict the progression to renal insufficiency among Asians with lupus nephritis | Parsa A et al. | 15789057 | Not a case-control study |
| 1 | Association of angiotensinogen gene T235 variant with progression of immunoglobin A nephropathy in Caucasian patients | Pei Y et al. | 9259580 | Not a case-control study |
| 1 | Normative genetic profiles of RAAS pathway gene Polymorphisms in north Indian and south Indian Populations | Prasad P et al. | 18027817 | Not a case-control study |
| 1 | Identification of a novel mutation in the human mineralocorticoid receptor gene in a German family with autosomal-dominant pseudohypoaldosteronism type 1: Further evidence for marked interindividual clinical heterogeneity | Riepe FG et al. | 12679457 | Not a case-control study |
| 1 | Genetics of diabetic nephropathy | Rippin JD et al. | 11554775 | Not a case-control study |
| 1 | A Synergistic Association of ACE I/D and eNOS G894T Gene Variants with the Progression of Immunoglobulin A Nephropathy - A Pilot Study | Rodriquez-Perez JC et al. | 19546528 | Not a case-control study |
| 1 | Increased left ventricular mass in normotensive type 1 diabetic patients with diabetic nephropathy | Sato A et al. | 9727905 | Not a case-control study |
| 1 | Risk of developing diabetic nephropathy is not associated with synergism between the angiotensin II (type 1) receptor C-1166 allele and poor glycaemic control | Savage DA et al. | 10328465 | Not a case-control study |
| 1 | Alport Syndrome in Women and Girls | Savige J et al. | 27287265 | Not a case-control study |
| 1 | Expert Guidelines for the Management of Alport Syndrome and Thin Basement Membrane Nephropathy | Savige J et al. | 23349312 | Not a case-control study |
| 1 | Genetic determinants of diabetic renal disease and their impact on therapeutic interventions | Schmidt S et al. | 9407416 | Not a case-control study |

| | | | | |
|---|---|---|---|---|
| 1 | Rho kinase polymorphism influences blood pressure and systemic vascular resistance in human twins - Role of heredity | Seasholtz TM et al. | 16585408 | Not a case-control study |
| 1 | Impact of genetic polymorphisms of the renin-angiotensin system and of non-genetic factors on kidney transplant function - a single-center experience | Siekierka-Harreis M et al. | 19681973 | Not a case-control study |
| 1 | The deletion/insertion polymorphism of the angiotensin converting enzyme gene and cardiovascular-renal risk | Staessen JA et al. | 9488209 | Not a case-control study |
| 1 | Renal angiomyolipoma - Further immunophenotypic characterization of an expanding morphologic spectrum | Stone CH et al. | 11371226 | Not a case-control study |
| 1 | Frequencies of apolipoprotein E alleles in depressed patients undergoing hemodialysis--a case-control study. | Su YY et al. | 25707516 | Not a case-control study |
| 1 | Assessing genetic susceptibility to diabetic nephropathy | Tanaka N et al. | 16174281 | Not a case-control study |
| 1 | Tip variant of focal segmental glomerulosclerosis: is it truly a benign variant? | Trivedi M et al. | 25721429 | Not a case-control study |
| 1 | Roles of Loss of Chromosome 14q Allele in the Prognosis of Renal Cell Carcinoma with C-reactive Protein Abnormity. | Wang G et al. | 28875953 | Not a case-control study |
| 1 | Contribution of gene polymorphisms in the renin-angiotensin system to macroangiopathy in patients with diabetic nephropathy | Wong et al. | 11431175 | Not a case-control study |
| 1 | Prognostic role of serum ACE activity on outcome of type 2 diabetic patients on chronic ambulatory peritoneal dialysis | Wong TYH et al. | 11979350 | Not a case-control study |
| 1 | Novel mutations in the inverted formin 2 gene of Chinese families contribute to focal segmental glomerulosclerosis. | Xie J et al. | 26039629 | Not a case-control study |
| 1 | Role Of The Deletion Polymorphism Of The Angiotensin-Converting Enzyme Gene In The Progression And Therapeutic Responsiveness Of Iga Nephropathy | Yoshida H et al. | 7593601 | Not a case-control study |
| 1 | Gene Polymorphisms of the Renin-AngiotensinAldosterone system and angiotensin 11 type I-Receptor activating antibodies in renal rejection | Zhang G et al. | 17984617 | Not a case-control study |

| | | | | |
|---|---|---|---|---|
| 1 | Rapid detection and quantification of apolipoprotein L1 genetic variants and total levels in plasma by ultra-performance liquid chromatography/tandem mass spectrometry. | Zhou H et al. | 24591025 | Not a case-control study |
| 1 | Pharmacogenetic association of the angiotensin-converting enzyme insertion/deletion polymorphism on blood pressure and cardiovascular risk in relation to antihypertensive treatment - The genetics of hypertension-associated treatment (GenHAT) study | Arnett DK et al. | 15967849 | Not a renal disease focus |
| 1 | A role for CETP TaqIB polymorphism in determining susceptibility to atrial fibrillation: a nested case control study | Asselbergs FW et al. | 16623947 | Not a renal disease focus |
| 1 | Serum paraoxonase-1 gene polymorphism and enzyme activity in patients with urolithiasis. | Atar A et al. | 26795139 | Not a renal disease focus |
| 1 | Association of ACE gene D polymorphism with left ventricular hypertrophy in patients with diastolic heart failure: a case-control study | Bahramali E et al. | 26861937 | Not a renal disease focus |
| 1 | Amiloride, a specific drug for hypertension in black people with T594M variant? | Baker EH et al. | 12105131 | Not a renal disease focus |
| 1 | Association of hypertension with T594M mutation in beta subunit of epithelial sodium channels in black people resident in London | Baker EH et al. | 9593408 | Not a renal disease focus |
| 1 | Alpha-adducin polymorphism in hypertensives of South African ancestry. | Barlassina C et al. | 10912759 | Not a renal disease focus |
| 1 | Common genetic variants and haplotypes in renal CLCNKA gene are associated to salt-sensitive hypertension | Barlassina C et al. | 17510212 | Not a renal disease focus |
| 1 | Synergistic effect of alpha-adducin and ACE genes causes blood pressure changes with body sodium and volume expansion | Barlassina C et al. | 10720960 | Not a renal disease focus |
| 1 | Evaluation of risk factors for the development of nephropathy in patients with IDDM: Insertion/deletion angiotensin converting enzyme gene polymorphism, hypertension and metabolic control | Barnas U et al. | 9084972 | Not a renal disease focus |
| 1 | Polymorphism in angiotensin II receptor genes and hypertension | Baudin B et al. | 15640279 | Not a renal disease focus |
| 1 | Higher thrombin activatable fibrinolysis inhibitor levels are associated with inflammation in attack-free familial Mediterranean fever patients. | Bavbek N et al. | 24580410 | Not a renal disease focus |

| | | | | |
|---|---|---|---|---|
| 1 | Insertion-deletion polymorphism in the angiotensin-converting enzyme (ACE) gene among Sudanese, Somalis, Emiratis, and Omanis | Bayoumi RA et al. | 16900885 | Not a renal disease focus |
| 1 | Single Strand Conformation Polymorphism (SSCP) as a quick and reliable method to genotype M235T polymorphism of angiotensinogen gene | Bettinaglio P et al. | 12270765 | Not a renal disease focus |
| 1 | CYP3A5 and ABCB1 genes and hypertension | Bochud M et al. | 19290795 | Not a renal disease focus |
| 1 | Polymorphisms of the renin-angiotensin system in patients with multifocal renal arterial fibromuscular dysplasia | Bofinger A et al. | 11317203 | Not a renal disease focus |
| 1 | Association between plasma activities of semicarbazide-sensitive amine oxidase and angiotensin-converting enzyme in patients with type 1 diabetes mellitus. | Boomsma F et al. | 15830186 | Not a renal disease focus |
| 1 | Structure-Based Analysis of Single Nucleotide Variants in the Renin-Angiotensinogen Complex | Brown DK et al. | 28302554 | Not a renal disease focus |
| 1 | Renin-angiotensin system gene polymorphisms: assessment of the risk of coronary heart disease. | Buraczynska M et al. | 14502296 | Not a renal disease focus |
| 1 | Oxidative stress-related factors in Bartter's and Gitelman's syndromes: relevance for angiotensin II signalling. | Calo LA et al. | 12897089 | Not a renal disease focus |
| 1 | Efficacy of large doses of IL-2-activated human leukocyte antigen haploidentical peripheral blood stem cells on refractory metastatic renal cell carcinoma. | Cao S et al. | 21812652 | Not a renal disease focus |
| 1 | Biochemical and genetic characterization of 11 beta-hydroxysteroid dehydrogenase type 2 in low-renin essential hypertensives. | Carvajal CA et al. | 15643127 | Not a renal disease focus |
| 1 | Role of GRK4 in the Regulation of Arterial AT(1) Receptor in Hypertension | Chen K et al. | 24218433 | Not a renal disease focus |
| 1 | CARD8 rs2043211 polymorphism is associated with gout in a Chinese male population. | Chen Y et al. | 25790751 | Not a renal disease focus |
| 1 | Association of renin-angiotensin and endothelial nitric oxide synthase gene polymorphisms with blood pressure progression and incident hypertension: prospective cohort study. | Conen D et al. | 18698212 | Not a renal disease focus |
| 1 | Role of ACE inhibitors in patients with diabetes mellitus | Cordonnier DJ et al. | 11708761 | Not a renal disease focus |

| | | | | |
|---|---|---|---|---|
| 1 | Molecular genetics of the renin-angiotensin-aldosterone system in human hypertension | Corvol P et al. | 9296068 | Not a renal disease focus |
| 1 | Vitamin D receptor gene polymorphisms and plasma renin activity in essential hypertensive individuals. | Cottone S et al. | 25500899 | Not a renal disease focus |
| 1 | Genetic polymorphism of Na-K cotransport in essential hypertension. | Cusi D et al. | 2258861 | Not a renal disease focus |
| 1 | Extensive personal experience - Examination of genotype and phenotype relationships in 14 patients with apparent mineralocorticoid excess | Dave-Sharma S et al. | 9661590 | Not a renal disease focus |
| 1 | ACE and PC-1 gene polymorphisms in normoalbuminuric Type 1 diabetic patients - A 10-year prospective study | de Azevedo MJ et al. | 12126783 | Not a renal disease focus |
| 1 | Polymorphisms in genes of the renin-angiotensin-aldosterone system and renal cell cancer risk: Interplay with hypertension and intakes of sodium, potassium and fluid | Deckers IA et al. | 24978482 | Not a renal disease focus |
| 1 | Adsorption of cytotoxic anti-HLA antibodies with HLA class I immunosorbant beads. | DeVito LD et al. | 2186524 | Not a renal disease focus |
| 1 | Clinical and laboratory characterization of 114 cases of Castleman disease patients from a single centre: paraneoplastic pemphigus is an unfavourable prognostic factor. | Dong Y et al. | 25824806 | Not a renal disease focus |
| 1 | CYP2C9 genotype modifies activity of the renin-angiotensin-aldosterone system in hypertensive men | Donner KM et al. | 19593208 | Not a renal disease focus |
| 1 | TGF-beta1 gene polymorphisms and peritoneal equilibration test results in CAPD patients. | Ebinc FA et al. | 18197538 | Not a renal disease focus |
| 1 | A new theory of essential hypertension based on analysis of the association between a polymorphism of the alpha(2)-adrenoceptor at the 10q24-q26 locus and hypertension in African-Americans | Eggers AE | 26243176 | Not a renal disease focus |
| 1 | High aldosterone-to-renin variants of CYP11B2 and pregnancy outcome. | Escher G et al. | 19151144 | Not a renal disease focus |
| 1 | Cardiovascular effects of aldosterone: insight from adult carriers of mineralocorticoid receptor mutations. | Escoubet B et al. | 23852419 | Not a renal disease focus |

| | | | | |
|---|---|---|---|---|
| 1 | Genetic polymorphisms of the renin-angiotensin-aldosterone system and renal insufficiency in essential hypertension | Fabris B et al. | 15662219 | Not a renal disease focus |
| 1 | Recurrence of the R947X mutation in unrelated families with autosomal dominant pseudohypoaldosteronism type 1: evidence for a mutational hot spot in the mineralocorticoid receptor gene. | Fernandes-Rosa FL et al. | 16757525 | Not a renal disease focus |
| 1 | Angiotensin converting enzyme gene I/D polymorphism in essential hypertension and nephroangiosclerosis | Fernandez-Llama P et al. | 9607207 | Not a renal disease focus |
| 1 | Renin-Angiotensin System Polymorphisms and Risk of Hypertension: Influence of Environmental Factors | Forman JP et al. | 18550936 | Not a renal disease focus |
| 1 | CYP3A5 genotype is associated with elevated blood pressure. | Fromm MF et al. | 16141800 | Not a renal disease focus |
| 1 | Associations between the human insulin gene 5' VNTR and clinical variables of the renin-angiotensin system. | Frossard PM et al. | 14647005 | Not a renal disease focus |
| 1 | The interaction of AGT and NOS3 gene polymorphisms with conventional risk factors increases predisposition to hypertension | Gatti RR et al. | 22791701 | Not a renal disease focus |
| 1 | Connexin 43 is not essential for the control of renin synthesis and secretion | Gerl M et al. | 24062052 | Not a renal disease focus |
| 1 | Deletion polymorphism of the angiotensin-converting enzyme gene is independently associated with left ventricular mass and geometric remodeling in systemic hypertension. | Gharavi AG et al. | 8677872 | Not a renal disease focus |
| 1 | A Computational Model of the Circulating Renin-Angiotensin System and Blood Pressure Regulation | Guillaud F et al. | 20683640 | Not a renal disease focus |
| 1 | The presence of PAI-1 4G/5G and ACE DD genotypes increases the risk of early-stage AVF thrombosis in hemodialysis patients. | Gungor Y et al. | 21332339 | Not a renal disease focus |
| 1 | ADAM33: a newly identified gene in the pathogenesis of asthma. | Holgate ST et al. | 16257631 | Not a renal disease focus |
| 1 | Nonmodulation and essential hypertension | Hollenberg NK et al. | 16672145 | Not a renal disease focus |

| | | | | |
|---|---|---|---|---|
| 1 | Angiotensinogen genotype affects renal and adrenal responses to angiotensin II in essential hypertension | Hopkins PN et al. | 11997278 | Not a renal disease focus |
| 1 | Blunted renal vascular response to angiotensin II is associated with a common Variant of the angiotensinogen gene and obesity | Hopkins PN et al. | 8728297 | Not a renal disease focus |
| 1 | Genetic variation of the renin-angiotensin system and chronic kidney disease progression in black individuals in the atherosclerosis risk in communities study | Hsu CCC et al. | 16396964 | Not a renal disease focus |
| 1 | Unclassified renal cell carcinoma: a clinicopathological, comparative genomic hybridization, and whole-genome exon sequencing study. | Hu ZY et al. | 25120763 | Not a renal disease focus |
| 1 | A case control association study of ACE gene polymorphism (I/D) with hypertension in Punjabi population from Faisalabad, Pakistan | Hussain M et al. | 29058472 | Not a renal disease focus |
| 1 | Genetic variant of the renin-angiotensin system and prevalence of type 2 diabetes mellitus: a modest but significant effect of aldosterone synthase | Ichikawa M et al. | 24549414 | Not a renal disease focus |
| 1 | The cardiovascular system in familial hypocalciuric hypercalcemia: a cross-sectional study on physiological effects of inactivating variants in the calcium-sensing receptor gene | Jakobsen NFB et al. | 27418061 | Not a renal disease focus |
| 1 | Genetic variants in five novel loci including CFB and CD40 predispose to chronic hepatitis B. | Jiang DK et al. | 25802187 | Not a renal disease focus |
| 1 | The association of the R563Q genotype of the ENaC with phenotypic variation in Southern Africa. | Jones ES et al. | 22895453 | Not a renal disease focus |
| 1 | Renin-Angiotensin System Gene Variants and Type 2 Diabetes Mellitus: Influence of Angiotensinogen | Joyce-Tan SM et al. | 26682227 | Not a renal disease focus |
| 1 | Comprehensive analysis of the renin-angiotensin gene polymorphisms with relation to hypertension in the Japanese | Kato N et al. | 10953993 | Not a renal disease focus |
| 1 | Genetic-Control Of Blood-Pressure And The Angiotensinogen Locus | Kim HS et al. | 7708716 | Not a renal disease focus |
| 1 | Effect of the plasminogen-plasmin system on hypertensive renal and cardiac damage | Knier B et al. | 21610512 | Not a renal disease focus |

| | | | | |
|---|---|---|---|---|
| 1 | Polymorphism of the aldosterone synthase gene is not associated with progression of diabetic nephropathy, but associated with hypertension in type 2 diabetic patients | Ko GJ et al. | 18771471 | Not a renal disease focus |
| 1 | Haplotype-based case-control study revealing an association between the adrenomedullin gene and proteinuria in subjects with essential hypertension | Kobayashi Y et al. | 16097366 | Not a renal disease focus |
| 1 | Lys(173)Arg and -344T/C variants of CYP11B2 in Japanese patients with low-renin hypertension. | Komiya I et al. | 10720581 | Not a renal disease focus |
| 1 | Molecular-Biology Of Hypertension | Krieger JE et al. | 1832415 | Not a renal disease focus |
| 1 | Beneficial role of D allele in controlling ACE levels: a study among Brahmins of north India | Kumari S et al. | 27350671 | Not a renal disease focus |
| 1 | Juxtaglomerular cell tumor: A morphological, immunohistochemical and genetic study of six cases | Kuroda N et al. | 22939575 | Not a renal disease focus |
| 1 | Angiotensin-converting enzyme gene polymorphism has no influence on the circulating renin-angiotensin-aldosterone system or blood pressure in normotensive subjects | Lachurie ML et al. | 7796503 | Not a renal disease focus |
| 1 | Angiotensinogen and angiotensin II type 1 receptor gene polymorphism in patients with autosomal dominant polycystic kidney disease: Effect on hypertension and ESRD | Lee KB et al. | 12950120 | Not a renal disease focus |
| 1 | ACE gene insertion/deletion polymorphism associated with 1998 World Health Organization definition of metabolic syndrome in Chinese type 2 diabetic patients | Lee YJ et al. | 12032106 | Not a renal disease focus |
| 1 | The brain and salt-sensitive hypertension | Leenen FHH et al. | 11884268 | Not a renal disease focus |
| 1 | Risk given by AGT polymorphisms in inducing susceptibility to essential hypertension among isolated populations from a remote region of China: A case-control study among the isolated populations | Li Q et al. | 26391364 | Not a renal disease focus |
| 1 | Association Between Polymorphisms of ADRBK1 Gene and Plasma Renin Activity in Hypertensive Patients: A Case-Control Study | Li Y et al. | 27555048 | Not a renal disease focus |
| 1 | No Evidence for the Expression of Renin-Angiotensin-Aldosterone System in Otosclerotic Stapes Footplates | Liktor B et al. | 23370555 | Not a renal disease focus |

| | | | | |
|---|---|---|---|---|
| 1 | Papillary renal cell carcinoma: a clinicopathological and whole-genome exon sequencing study. | Liu K et al. | 26339402 | Not a renal disease focus |
| 1 | Common variation in KLKB1 and essential hypertension risk: tagging-SNP haplotype analysis in a case-control study | Lu XF et al. | 17318641 | Not a renal disease focus |
| 1 | The Connexin40 A96S Mutation Causes Renin-Dependent Hypertension | Lubkemeier I et al. | 21597036 | Not a renal disease focus |
| 1 | Connexin 40 is dispensable for vascular renin cell recruitment but is indispensable for vascular baroreceptor control of renin secretion | Machura K et al. | 25241776 | Not a renal disease focus |
| 1 | The association between vitamin D receptor gene polymorphisms (TaqI and FokI), Type 2 diabetes, and micro-/macrovascular complications in postmenopausal women | Maia J et al. | 27536155 | Not a renal disease focus |
| 1 | Angiotensin-converting enzyme (ACE) gene II genotype protects against the development of diabetic peripheral neuropathy in type 2 diabetes mellitus. | Mansoor Q et al. | 22607040 | Not a renal disease focus |
| 1 | Apparent mineralocorticoid excess: Type I and type II | Mantero F et al. | 8732999 | Not a renal disease focus |
| 1 | Low-salt diet and diuretic effect on blood pressure and organ damage | Manunta P et al. | 14684671 | Not a renal disease focus |
| 1 | Renal genetic mechanisms of essential hypertension | Manunta P et al. | 9377722 | Not a renal disease focus |
| 1 | alpha-Adducin polymorphisms and renal sodium handling in essential hypertensive patients | Manunta P et al. | 9607177 | Not a renal disease focus |
| 1 | Is angiotensin-converting enzyme inhibitors/angiotensin receptor blockers therapy protective against prostate cancer? | Mao YQ et al. | 26760503 | Not a renal disease focus |
| 1 | Detection of the association between a deletion polymorphism in the gene encoding angiotensin I-converting enzyme and advanced diabetic retinopathy | Matsumoto A et al. | 11106834 | Not a renal disease focus |
| 1 | Genotypic interactions of renin-angiotensin system genes with diabetes type 2 in a Tunisian population | Mehri S et al. | 20580725 | Not a renal disease focus |

| | | | | |
|---|---|---|---|---|
| 1 | Angiotensin II type 1 receptor gene polymorphism and the response to hyperglycemia in early type 1 diabetes | Miller JA et al. | 10969844 | Not a renal disease focus |
| 1 | Renal ACE immunohistochemical localization in NIDDM patients with nephropathy | Mizuiri S et al. | 9469501 | Not a renal disease focus |
| 1 | Randomised controlled trial of dual blockade of renin-angiotensin system in patients with hypertension, microalbuminuria, and non-insulin dependent diabetes: the candesartan and Lisinopril microalbuminuria (CALM) study | Mogensen CE et al. | 11110735 | Not a renal disease focus |
| 1 | Association of Polymorphisms in Endothelial Nitric Oxide Synthesis and Renin-Angiotensin-Aldosterone System with Developing of Coronary Artery Disease in Bulgarian Patients | Mokretar K et al. | 26670794 | Not a renal disease focus |
| 1 | Contribution of angiotensin I converting enzyme gene polymorphism and angiotensinogen gene polymorphism to blood pressure regulation in essential hypertension. | Mondorf UF et al. | 9524045 | Not a renal disease focus |
| 1 | Inhibition of tissue angiotensin converting enzyme activity prevents malignant hypertension in TGR(mREN2)27. | Montgomery HE et al. | 9797175 | Not a renal disease focus |
| 1 | [Possible pathogenetic role of 11 beta-hydroxysteroid dehydrogenase type 1 (11betaHSD1) gene polymorphisms in arterial hypertension]. | Morales MA et al. | 18769825 | Not a renal disease focus |
| 1 | Inefficient arterial hypertension control in patients with metabolic syndrome and its link to renin-angiotensin-aldosterone system polymorphisms | Morales-Suarez-Varela MM et al. | 21471972 | Not a renal disease focus |
| 1 | Trientine and renin-angiotensin system blockade ameliorate progression of glomerular morphology in hypertensive experimental diabetic nephropathy. | Moya-Olano L et al. | 22029676 | Not a renal disease focus |
| 1 | Is the influence of variation in the ACE gene on the prospective risk of Type 2 diabetes in middle-aged men modified by obesity? | Muthumala A et al. | 17624939 | Not a renal disease focus |
| 1 | Evaluation Of The Sa Locus In Human Hypertension | Nabika T et al. | 7843754 | Not a renal disease focus |
| 1 | Atrial Natriuretic Peptide Locally Counteracts the Deleterious Effects of Cardiomyocyte Mineralocorticoid Receptor Activation | Nakagawa H et al. | 25027872 | Not a renal disease focus |

| | | | | |
|---|---|---|---|---|
| 1 | Association between RAS gene polymorphisms (ACE I/D, AGT M235T) and Henoch-Schonlein purpura in a Turkish population | Nalbantoglu S et al. | 23151617 | Not a renal disease focus |
| 1 | Urine exosomes from healthy and hypertensive pregnancies display elevated level of alpha-subunit and cleaved alpha- and gamma-subunits of the epithelial sodium channel-ENaC | Nielsen MR et al. | 28405801 | Not a renal disease focus |
| 1 | Angiotensin Converting Enzyme Gene Insertion/Deletion Variant and Familial Mediterranean Fever-related Amyloidosis. | Nursal AF et al. | 29891744 | Not a renal disease focus |
| 1 | Genetic polymorphisms of the renin-angiotensin system and atheromatous renal artery stenosis | Olivieri O et al. | 10567188 | Not a renal disease focus |
| 1 | Three Reportedly Unrelated Families With Liddle Syndrome Inherited From a Common Ancestor. | Pagani L et al. | 29229744 | Not a renal disease focus |
| 1 | Endothelial nitric oxide synthase gene/gender interactions and the renal hemodynamic response to angiotensin II | Page A et al. | 16093452 | Not a renal disease focus |
| 1 | The alpha-adducin Gly460Trp polymorphism and the antihypertensive effects of exercise among men with high blood pressure. | Pescatello LS et al. | 17472579 | Not a renal disease focus |
| 1 | Impact of maternal angiotensinogen M235T polymorphism and angiotensin-converting enzyme insertion/deletion polymorphism on blood pressure, protein excretion and fetal outcome in pregnancy. | Pfab T et al. | 17563539 | Not a renal disease focus |
| 1 | The state and responsiveness of the renin-angiotensin-aldosterone system in patients with type II diabetes mellitus | Price DA et al. | 10232494 | Not a renal disease focus |
| 1 | Angiotensin-converting enzyme gene polymorphism in patients with systemic lupus. | Prkacin I et al. | 11505631 | Not a renal disease focus |
| 1 | Long-term follow-up of patients with Bartter syndrome type I and II. | Puricelli E et al. | 20219833 | Not a renal disease focus |
| 1 | The relationship between ACE/AGT gene polymorphisms and the risk of diabetic retinopathy in Chinese patients with type 2 diabetes. | Qiao YC et al. | 29378484 | Not a renal disease focus |
| 1 | Possible role for nephron-derived angiotensinogen in angiotensin-II dependent hypertension | Ramkumar N et al. | 26755736 | Not a renal disease focus |

| | | | | |
|---|---|---|---|---|
| 1 | A new mutation, R563Q, of the beta subunit of the epithelial sodium channel associated with low-renin, low-aldosterone hypertension | Rayner BL et al. | 12714866 | Not a renal disease focus |
| 1 | Network-based regularization for high dimensional SNP data in the case-control study of Type 2 diabetes. | Ren J et al. | 28511641 | Not a renal disease focus |
| 1 | Pigsties near dwellings as a potential risk factor for the prevalence of Japanese encephalitis virus in adult in Shanxi, China. | Ren X et al. | 28592296 | Not a renal disease focus |
| 1 | Association of angiotensinogen M235T and A(-6)G gene polymorphisms with coronary heart disease with independence of essential hypertension: the PROCAGENE study. Prospective Cardiac Gene. | Rodriquez-Perez JC et al. | 11345362 | Not a renal disease focus |
| 1 | A clinical phenotype mimicking essential hypertension in a newly discovered family with Liddle's syndrome. | Rossi E et al. | 21525970 | Not a renal disease focus |
| 1 | Vitamin D Deficiency in the Pathogenesis of Hypertension: Still an Unsettled Question | Rostand SG et al. | 24929953 | Not a renal disease focus |
| 1 | Amelioration of genetic hypertension by suppression of renal G protein-coupled receptor kinase type 4 expression | Sanada H et al. | 16636192 | Not a renal disease focus |
| 1 | Prevalence of angiotensin converting enzyme (ACE) gene insertion/deletion polymorphism in South Indian population with hypertension and chronic kidney disease. | Shanmuganathan R et al. | 26440392 | Not a renal disease focus |
| 1 | Novel SLC12A3 mutations in Chinese patients with Gitelman's syndrome. | Shao L et al. | 18287808 | Not a renal disease focus |
| 1 | Mechanisms of suppression of renal kallikrein activity in low renin essential hypertension and renoparenchymal hypertension. | Shimamoto K et al. | 2676859 | Not a renal disease focus |
| 1 | Systemic nitric oxide clamping in normal humans guided by total peripheral resistance | Simonsen JA et al. | 19785629 | Not a renal disease focus |
| 1 | Exaggerated natriuresis during clamping of systemic NO supply in healthy young men | Simonsen JA et al. | 21749320 | Not a renal disease focus |

| | | | | |
|---|---|---|---|---|
| 1 | Angiotensin-converting enzyme gene I/D polymorphism increases the susceptibility to hypertension and additive diseases: A study on North Indian patients. | Singh M et al. | 27030424 | Not a renal disease focus |
| 1 | Renal haemodynamics are not related to genotypes in offspring of parents with essential hypertension | Skov K et al. | 17083073 | Not a renal disease focus |
| 1 | Association between renin-angiotensin-aldosterone system-related genes and blood pressure in a Korean population. | Song SB et al. | 21342026 | Not a renal disease focus |
| 1 | Phenotype-genotype interactions on renal function in type 2 diabetes: an analysis using structural equation modelling | Song XY et al. | 19479237 | Not a renal disease focus |
| 1 | Alpha Adducin G460T Variant is a Risk Factor for Hypertension in Tunisian Population | Soualmia H et al. | 27349000 | Not a renal disease focus |
| 1 | Angiotensin II sensitivity in nonpregnant formerly preeclamptic women and healthy parous controls | Spaanderman MEA et al. | 15350256 | Not a renal disease focus |
| 1 | Angiotensin II Type 1A Receptors in Vascular Smooth Muscle Cells Do Not Influence Aortic Remodeling in Hypertension | Sparks MA et al. | 21242463 | Not a renal disease focus |
| 1 | Association between polymorphisms of the renin-angiotensin system and more severe histological forms of lupus nephritis | Sprovieri SRS et al. | 16047641 | Not a renal disease focus |
| 1 | Wilms' tumor protein (-KTS) modulates renin gene transcription | Steege A et al. | 18496514 | Not a renal disease focus |
| 1 | Angiotensin-converting enzyme gene I/D polymorphism in malignant hypertension | Stefansson B et al. | 10855732 | Not a renal disease focus |
| 1 | Transforming growth factor-beta(1) hyperexpression in African-American hypertensives: A novel mediator of hypertension and/or target organ damage | Suthanthiran M et al. | 10725360 | Not a renal disease focus |
| 1 | A functional variant of the NEDD4L gene is associated with beneficial treatment response with Î²-blockers and diuretics in hypertensive patients. | Svensson-Farbom P et al. | 21052022 | Not a renal disease focus |
| 1 | Genetic variants in hypertensive patients with coronary artery disease and coexisting atheromatous renal artery stenosis | Szperl M et al. | 19043368 | Not a renal disease focus |

| | | | | |
|---|---|---|---|---|
| 1 | Melatonin prevents maternal fructose intake-induced programmed hypertension in the offspring: roles of nitric oxide and arachidonic acid metabolites | Tain YL et al. | 24867192 | Not a renal disease focus |
| 1 | Genetic variants in the inositol phosphate metabolism pathway and risk of different types of cancer. | Tan J et al. | 25683757 | Not a renal disease focus |
| 1 | Renin in blood vessels in human pulmonary tumors. An immunohistochemical and biochemical study. | Taylor GM et al. | 2450464 | Not a renal disease focus |
| 1 | Incidence of renal failure and nephroprotection by RAAS inhibition in heterozygous carriers of X-chromosomal and autosomal recessive Alport mutations. | Temme J et al. | 22237748 | Not a renal disease focus |
| 1 | Peripheral vascular disease in type 2 diabetic Chinese patients: associations with metabolic indices, concomitant vascular disease and genetic factors | Thomas GN et al. | 14632699 | Not a renal disease focus |
| 1 | Albuminuria and the renin-angiotensin system gene polymorphisms in type-2-diabetic and in normoglycemic hypertensive Chinese | Thomas GN et al. | 11200871 | Not a renal disease focus |
| 1 | Middle cerebral artery stenosis in type II diabetic Chinese patients is associated with conventional risk factors but not with polymorphisms of the renin-angiotensin system genes | Thomas GN et al. | 12865608 | Not a renal disease focus |
| 1 | Genetic variation in the KCNMA1 potassium channel alpha subunit as risk factor for severe essential hypertension and myocardial infarction | Tomas M et al. | 18854754 | Not a renal disease focus |
| 1 | Renal Mechanisms of Association between Fibroblast Growth Factor 1 and Blood Pressure | Tomaszewski M et al. | 25918036 | Not a renal disease focus |
| 1 | Angiotensin II-dependent chronic hypertension and cardiac hypertrophy are unaffected by gp91phox-containing NADPH oxidase | Touyz RM et al. | 15753233 | Not a renal disease focus |
| 1 | Renin Production In Congenital Mesoblastic Nephroma In Comparison With That In Wilms-Tumor | Tsuchida Y et al. | 8385325 | Not a renal disease focus |
| 1 | Genomic association analysis identifies multiple loci influencing antihypertensive response to an angiotensin II receptor blocker. | Turner ST et al. | 22566498 | Not a renal disease focus |

| | | | | |
|---|---|---|---|---|
| 1 | Angiotensin-converting enzyme gene polymorphism and vascular manifestations in Korean patients with SLE | Uhm WS et al. | 12043886 | Not a renal disease focus |
| 1 | The M235T polymorphism in theangiotensinogen gene is associated with the risk of malignant hypertension in white patients | van den Born BJH et al. | 17921816 | Not a renal disease focus |
| 1 | Genetic risk of atherosclerotic renal artery disease - The candidate gene approach in a renal angiography cohort | van Onna M et al. | 15326089 | Not a renal disease focus |
| 1 | Activation of the hypothalamic-pituitary-adrenal axis in adults with mineralocorticoid receptor haploinsufficiency. | Walker BR et al. | 24712576 | Not a renal disease focus |
| 1 | Liver pyruvate kinase polymorphisms are associated with type 2 diabetes in northern European Caucasians. | Wang H et al. | 12196482 | Not a renal disease focus |
| 1 | miR149 rs71428439 polymorphism and risk of clear cell renal cell carcinoma: a case-control study. | Wang Z et al. | 25213695 | Not a renal disease focus |
| 1 | Genotype-phenotype analysis of angiotensinogen polymorphisms and essential hypertension: the importance of haplotypes | Watkins WS et al. | 19770777 | Not a renal disease focus |
| 1 | Angiotensin-converting enzyme gene does not contribute to genetic susceptibility to systemic sclerosis in European Caucasians. | Wipff J et al. | 19132786 | Not a renal disease focus |
| 1 | Androgen-Sensitive Hypertension Associates with Upregulated Vascular CYP4A12-20-HETE Synthase | Wu CC et al. | 23641057 | Not a renal disease focus |
| 1 | Urinary UMOD excretion and chronic kidney disease in gout patients: cross-sectional case-control study. | Wu CH et al. | 21332338 | Not a renal disease focus |
| 1 | Unknown face of known drugs - what else can we expect from angiotensin converting enzyme inhibitors? | Wzgarda A et al. | 28087255 | Not a renal disease focus |
| 1 | Malignant Nephrosclerosis in a Patient with Familial Mediterranean Fever | Yamanouchi M et al. | 26466703 | Not a renal disease focus |
| 1 | Gene polymorphism of vascular endothelial growth factor-1154 G > A is associated with hypertensive nephropathy in a Hispanic population | Yang JW et al. | 21080079 | Not a renal disease focus |
| 1 | TBX6 compound inheritance leads to congenital vertebral malformations in humans and mice. | Yang N et al. | 30307510 | Not a renal disease focus |

| | | | | |
|---|---|---|---|---|
| 1 | Identification of diuretic non-responders with poor long-term clinical outcomes: a 1-year follow-up of 176 non-azotaemic cirrhotic patients with moderate ascites | Yang YY et al. | 21692745 | Not a renal disease focus |
| 1 | Renal redox-sensitive signaling, but not blood pressure, is attenuated by Nox1 knockout in angiotensin II-dependent chronic hypertension | Yogi A et al. | 18195161 | Not a renal disease focus |
| 1 | Angiotensin II type 2 receptor gene is not responsible for familial vesicoureteral reflux | Yoneda A et al. | 12187255 | Not a renal disease focus |
| 1 | Angiotensinogen T235 and ACE insertion/deletion polymorphisms associated with albuminuria in Chinese type 2 diabetic patients | Young RP et al. | 9540028 | Not a renal disease focus |
| 1 | Common genetic variants in the chromogranin a promoter are associated with renal injury in IgA nephropathy patients with malignant hypertension. | Yu L et al. | 20113265 | Not a renal disease focus |
| 1 | Frequencies Of Variants Of Candidate Genes In Different Age-Groups Of Hypertensives | Zee RY et al. | 7882587 | Not a renal disease focus |
| 1 | Association of angiotensin-converting enzyme gene polymorphisms with Crohn's disease in a Chinese Han population | Zhou J et al. | 26823847 | Not a renal disease focus |
| 1 | Molecular variants of the sodium/hydrogen exchanger type 3 gene and essential hypertension | Zhu HD et al. | 15201541 | Not a renal disease focus |
| 1 | [Renin-angiotensin system genes in chronic glomerulonephritis]. | Buraczynska M et al. | 11865575 | Not English or Spanish |
| 1 | [Association of the renin-angiotensin system gene polymorphism with nephropathy in type II diabetes]. | Buraczynska M et al. | 12476891 | Not English or Spanish |
| 1 | [Genetic predisposition to systemic complications of arterial hypertension in maintenance haemodialysis patients]. | Bzoma B et al. | 19112833 | Not English or Spanish |
| 1 | [Arterial hypertension and chronic hemodialysis]. | Ermolenko VM et al. | 7700 | Not English or Spanish |
| 1 | [Mutations in NPHS2 in familial steroid-resistant nephrotic syndrome in Southern Chinese Han ethnic group]. | Fu R et al. | 19099831 | Not English or Spanish |

| | | | | |
|---|---|---|---|---|
| 1 | [Is PstI polymorphism of the angiotensin I converting enzyme gene associated with nephropathy development in non-insulin-dependent diabetes mellitus (preliminary study)]. | Grzeszczak W et al. | 9499204 | Not English or Spanish |
| 1 | [Angiotensin-converting enzyme gene polymorphism and the clinical pathological features and progression in lupus nephritis]. | Guan T et al. | 10436947 | Not English or Spanish |
| 1 | [Angiotensin-converting enyme insertion/deletion polymorphism and blood pressure regulation in type 2 diabetic patients]. | Krajina-Andricevic M et al. | 23120809 | Not English or Spanish |
| 1 | [Relationship between I/D polymorphism of angiotensin I converting enzyme gene and microvascular complications in type 2 diabetic patients]. | Moleda P et al. | 17941464 | Not English or Spanish |
| 1 | [Genomics of type 1 diabetes mellitus and its late complications] | Nosikov VV | 15042845 | Not English or Spanish |
| 1 | [Correlation between HLA-DQA1 allele and anaphylactoid purpura in juvenile Hans residing in Inner Mongolia]. | Ren S et al. | 11836690 | Not English or Spanish |
| 1 | [Correlation of cyclin D1 overexpression to mutations of von hippel-lindau gene in renal clear cell carcinoma]. | Ren YY et al. | 16480581 | Not English or Spanish |
| 1 | [Morphofunctional characteristics of endocrine nephropathy in primary aldosteronism]. | Sokolova RI et al. | 2678677 | Not English or Spanish |
| 1 | [Gene mutation analysis of X-linked hypophosphatemic rickets]. | Song Y et al. | 24229582 | Not English or Spanish |
| 1 | [A novel COL4A5 splicing mutation causing Alport syndrome in a Chinese family]. | Tang Z et al. | 19065523 | Not English or Spanish |
| 1 | [Association of single nucleotide polymorphism of megsin gene with IgA nephropathy]. | Wang ZH et al. | 16796905 | Not English or Spanish |
| 1 | [Study on the association between the polymorphism of HLA-DQA1 alleles and type 2 diabetes in Yunnan Han nationality]. | Yang HY et al. | 15192842 | Not English or Spanish |
| 1 | DD Genotype of ACE Gene in Boys: May it be a Risk Factor for Minimal Change Nephrotic Syndrome? | Alasehirli B et al. | 22017506 | Paediatric individuals |
| 1 | Angiotensin-converting enzyme gene insertion/deletion polymorphism and renal damage in childhood uropathies | Al-Eisa A et al. | 10986863 | Paediatric individuals |

| | | | | |
|---|---|---|---|---|
| 1 | Angiotensin converting enzyme gene polymorphism in Asian Indian children with congenital uropathies | Bajpai M et al. | 14713838 | Paediatric individuals |
| 1 | Posterior urethral valves: Preliminary observations on the significance of plasma renin activity as a prognostic marker | Bajpai M et al. | 15643266 | Paediatric individuals |
| 1 | Nitric oxide synthase gene polymorphisms in children with primary nocturnal enuresis: a preliminary study. | Balat A et al. | 17365914 | Paediatric individuals |
| 1 | MCP1 2518 A/G polymorphism affects progression of childhood focal segmental glomerulosclerosis. | Besbas N et al. | 26335292 | Paediatric individuals |
| 1 | Heterozygous Loss-of-Function SEC61A1 Mutations Cause Autosomal-Dominant Tubulo-Interstitial and Glomerulocystic Kidney Disease with Anemia | Bolar NA et al. | 27392076 | Paediatric individuals |
| 1 | Donor and recipient ACE I/D genotype are associated with loss of renal function in children following renal transplantation | Buscher R et al. | 21309964 | Paediatric individuals |
| 1 | ACE gene polymorphism in Turkish children with nephrotic syndrome. | Celik US et al. | 16825089 | Paediatric individuals |
| 1 | Increased HLA- A*11 in Chinese children with steroid-responsive nephrotic syndrome. | Cheung W et al. | 11956863 | Paediatric individuals |
| 1 | Liddle syndrome: clinical and genetic profiles. | Cui Y et al. | 27896928 | Paediatric individuals |
| 1 | NPHS2 variation in Chinese southern infants with late steroid-resistant nephrotic syndrome. | Dai Y et al. | 25112471 | Paediatric individuals |
| 1 | Earlier Onset of Complications in Youth With Type 2 Diabetes | Dart AB et al. | 24130346 | Paediatric individuals |
| 1 | ACE gene polymorphism in Egyptian children with idiopathic nephrotic syndrome | Fahmy ME et al. | 18792483 | Paediatric individuals |
| 1 | Genetic polymorphisms of the renin-angiotensin system and the outcome of focal segmental glomerulosclerosis in children | Frishberg Y et al. | 9853248 | Paediatric individuals |
| 1 | Angiotensinogen gene T235 variant: a marker for the development of persistent microalbuminuria in children and adolescents with type 1 diabetes mellitus | Gallego PH et al. | 18413222 | Paediatric individuals |

| | | | | |
|---|---|---|---|---|
| 1 | Autosomal dominant pseudohypoaldosteronism type 1: Mechanisms, evidence for neonatal lethality, and phenotypic expression in adults | Geller DS et al. | 16611713 | Paediatric individuals |
| 1 | The role of vitamin D receptor gene polymorphisms in Turkish infants with urolithiasis. | Goknar N et al. | 26908058 | Paediatric individuals |
| 1 | Risk factors for loss of residual renal function in children treated with chronic peritoneal dialysis | Ha IS et al. | 25874598 | Paediatric individuals |
| 1 | Implication of genetic variations in congenital obstructive nephropathy | Hahn H et al. | 16133060 | Paediatric individuals |
| 1 | ACE gene polymorphism and renal scarring in primary vesicoureteric reflux | Haszon I et al. | 12478352 | Paediatric individuals |
| 1 | Impact of ACE I/D gene polymorphism on congenital renal malformations | Hohenfellner K et al. | 11354781 | Paediatric individuals |
| 1 | Impact of common functional polymorphisms in renin angiotensin system genes on the risk of renal parenchymal scarring following childhood urinary tract infection | Hussein A et al. | 25939993 | Paediatric individuals |
| 1 | An insertion/deletion ACE polymorphism and kidney size in Polish full-term newborns | Kaczmarczyk M et al. | 22674971 | Paediatric individuals |
| 1 | ACE and AT1 receptor gene polymorphisms and renal scarring in urinary bladder dysfunction | Kostic M et al. | 15179569 | Paediatric individuals |
| 1 | Renal tubular dysgenesis, a not uncommon autosomal recessive disorder leading to oligohydramnios: Role of the Renin-Angiotensin system. | Lacoste M et al. | 16790508 | Paediatric individuals |
| 1 | Renin-angiotensin system gene polymorphisms in children with Henoch-Schonlein purpura in West China | Liu DS et al. | 20702504 | Paediatric individuals |
| 1 | Renin-angiotensin system polymorphisms in Taiwanese primary vesicoureteral reflux | Liu KP et al. | 15045574 | Paediatric individuals |
| 1 | Polymorphisms of renin-angiotensin system genes in childhood IgA nephropathy | Maruyama K et al. | 11354780 | Paediatric individuals |
| 1 | Association of angiotensin type 2 receptor gene polymorphisms with ureteropelvic junction obstruction in Brazilian patients | Miranda DM et al. | 24995698 | Paediatric individuals |

| | | | | |
|---|---|---|---|---|
| 1 | Association of angiotensin converting enzyme and angiotensin type 2 receptor gene polymorphisms with renal damage in posterior urethral valves | Narasimhan KL et al. | WOS:000290840100006 | Paediatric individuals |
| 1 | Prioritization and burden analysis of rare variants in 208 candidate genes suggest they do not play a major role in CAKUT | Nicolaou N et al. | 26489027 | Paediatric individuals |
| 1 | ACE I/D gene polymorphism in primary FSGS and steroid-sensitive nephrotic syndrome | Oktem F et al. | 14986085 | Paediatric individuals |
| 1 | Implications of certain genetic polymorphisms in scarring in vesicoureteric reflux: importance of ACE polymorphism. | Ozen S et al. | 10401028 | Paediatric individuals |
| 1 | Renin-angiotensin system gene polymorphisms: association with susceptibility to Henoch-Schonlein purpura and renal involvement | Ozkaya O et al. | 16521052 | Paediatric individuals |
| 1 | Renin-angiotensin gene polymorphism in children with uremia and essential hypertension | Papp F et al. | 12579405 | Paediatric individuals |
| 1 | A rare case of juvenile hypertension: coexistence of type 2 multiple endocrine neoplasia -related bilateral pheochromocytoma and reninoma in a young patient with ACE gene polymorphism | Paragliola RM et al. | 26084817 | Paediatric individuals |
| 1 | Renin-angiotensin system polymorphisms and renal scarring | Pardo R et al. | 12579398 | Paediatric individuals |
| 1 | Low renin-angiotensin system activity gene polymorphism and dysplasia associated with posterior urethral valves | Peruzzi L et al. | 16006956 | Paediatric individuals |
| 1 | Effect of angiotensin-converting enzyme gene insertion/deletion polymorphism on steroid resistance in Egyptian children with idiopathic nephrotic syndrome. | Saber-Ayad M et al. | 20418353 | Paediatric individuals |
| 1 | ACE gene insertion/deletion polymorphism in childhood idiopathic nephrotic syndrome | Serdaroglu E et al. | 16208534 | Paediatric individuals |
| 1 | Effects of genetic polymorphisms of the renin-angiotensin system in children with nephrotic syndrome | Tabel Y et al. | 16525944 | Paediatric individuals |
| 1 | Genetic risk factors in typical haemolytic uraemic syndrome | Taranta A et al. | 19110485 | Paediatric individuals |

| | | | | |
|---|---|---|---|---|
| 1 | The juxtaglomerular apparatus in Bartter's syndrome and related tubulopathies. An immunocytochemical and electron microscopic study. | Taugner R et al. | 3128915 | Paediatric individuals |
| 1 | Mapping candidate regions and genes for congenital anomalies of the kidneys and urinary tract (CAKUT) by array-based comparative genomic hybridization | Weber S et al. | 20605837 | Paediatric individuals |
| 1 | Genetic polymorphism of the renin-angiotensin system on the development of primary vesicoureteral reflux | Yim HE et al. | 14764974 | Paediatric individuals |
| 1 | The association of endothelial nitric oxide synthase gene single nucleotide polymorphisms with paediatric systemic lupus erythematosus | Zhu J et al. | 29465350 | Paediatric individuals |
| 1 | Add-on angiotensin receptor blockade with maximized ACE inhibition | Agarwal R | 11380832 | Pharmaceutical drug focus |
| 1 | DPP-4 Inhibition on Top of Angiotensin Receptor Blockade Offers a New Therapeutic Approach for Diabetic Nephropathy | Alter ML et al. | 23171828 | Pharmaceutical drug focus |
| 1 | Effects of losartan and enalapril on high-sensitivity C-reactive protein and total antioxidant in renal transplant recipients with renin-angiotensin system polymorphisms | Argani H et al. | 18261537 | Pharmaceutical drug focus |
| 1 | Losartan decreases plasma levels of TGF-beta 1 in transplant patients with chronic allograft nephropathy | Campistol JM et al. | 10432413 | Pharmaceutical drug focus |
| 1 | Captopril enhances transforming growth factor (TGF)-beta1 expression in peripheral blood mononuclear cells: a mechanism independent from angiotensin converting enzyme inhibition? A study in cyclosporine-treated kidney-transplanted patients. | Di Paolo S et al. | 12499886 | Pharmaceutical drug focus |
| 1 | Thiazolidinediones and the renal and hormonal response to water immersion-induced volume expansion in type 2 diabetes mellitus | Goenka N et al. | 18230694 | Pharmaceutical drug focus |
| 1 | The influence of renin-angiotensin system genotypes on the antiproteinuric response to high doses of olmesartan in non-diabetic protein uric nephropathies | Goyache-Goni B et al. | 24241364 | Pharmaceutical drug focus |
| 1 | Antiproteinuric effect of candesartan cilexetil in Japanese subjects with type 2 diabetes and nephropathy | Haneda M et al. | 15364166 | Pharmaceutical drug focus |

| | | | | |
|---|---|---|---|---|
| 1 | Renal implications of angiotensin receptor blockers | Hollenberg NK | 11459212 | Pharmaceutical drug focus |
| 1 | The influence of the ACE (I/D) polymorphism on systemic and renal vascular responses to angiotensins in normotensive, normoalbuminuric Type 1 diabetes mellitus | Luik PT et al. | 12856080 | Pharmaceutical drug focus |
| 1 | Application of Direct Renin Inhibition to Chronic Kidney Disease | Mende CW | 20490905 | Pharmaceutical drug focus |
| 1 | Pentoxifylline for Renoprotection in Diabetic Nephropathy: the PREDIAN study. Rationale and basal results | Navarro-Gonzalez JF et al. | 21144773 | Pharmaceutical drug focus |
| 1 | Renin-angiotensin system polymorphisms and hemoglobin level in renal allografts: A comparative study between losartan and enalapril | Noroozianavval M et al. | 17524880 | Pharmaceutical drug focus |
| 1 | Enalapril and losartan affect lipid peroxidation in renal transplant recipients with renin-angiotensin system polymorphisms | Rashtchizadeh N et al. | 17222813 | Pharmaceutical drug focus |
| 2 | Relationship between the renin-angiotensin system genes and diabetic nephropathy in the Chinese. | Wu S et al. | 11776100 | No data |
| 3 | Plasma renin and prorenin and renin gene variation in patients with insulin-dependent diabetes mellitus and nephropathy. | Deinum J et al. | 10462269 | < 3 populations reported per SNP |
| 3 | Renin–angiotensin–aldosterone system genotypes and haplotypes affect the susceptibility to nephropathy in type 2 diabetes patients | Mtiraoui N et al. | 21421655 | < 3 populations reported per SNP |
| 3 | Chronic renal insufficiency among Asian Indians with type 2 diabetes: I. Role of RAAS gene polymorphisms | Prasad P et al. | 16672053 | < 3 populations reported per SNP |
| 3 | Renin gene rs1464816 polymorphism contributes to chronic kidney disease progression in ADPKD. | Ramanathan G et al. | 26753721 | < 3 populations reported per SNP |
| 3 | M235T polymorphism in the AGT gene and A/G(I8-83) substitution in the REN gene correlate with end-stage renal disease | Sarkar S et al. | 25660845 | < 3 populations reported per SNP |

**Supplementary Table S4:** Data included in quantitative analysis for *ACE*

| | | | | Cases | | | | Controls | | | |
|---|---|---|---|---|---|---|---|---|---|---|---|
| **Author** | **PMID/ WOS ID** | **Ethnicity** | **SNP** | **Definition** | **N** | **I Frequency** | **D Frequency** | **Definition** | **N** | **I Frequency** | **D Frequency** |
| Anbazhagan K et al. | 19520069 | SAS | Ins/Del | CKD | 118 | 124 | 112 | Healthy | 98 | 99 | 97 |
| Chen WJ et al. | 24907556 | SAS | Ins/Del | CKD | 233 | 314 | 152 | Healthy | 449 | 617 | 281 |
| Nagamani S et al. | None | SAS | Ins/Del | CKD | 147 | 152 | 142 | Healthy | 221 | 195 | 227 |
| Shanmuganathan R et al. | 26440392 | SAS | Ins/Del | CKD | 30 | 10 | 50 | Healthy | 30 | 55 | 5 |
| Ali A et al. | 21421653 | EAS | Ins/Del | ESRD | 190 | 219 | 161 | Healthy | 190 | 262 | 118 |
| Kawada N et al. | 000071880400005 | EAS | Ins/Del | ESRD | 216 | 280 | 152 | Healthy | 208 | 264 | 152 |
| Su SL et al. | 24977181 | EAS | Ins/Del | ESRD | 647 | 792 | 502 | Healthy | 644 | 859 | 429 |
| Tang FY et al. | 18629615 | EAS | Ins/Del | ESRD | 153 | 100 | 206 | Healthy | 148 | 138 | 158 |
| Wang AY et al. | 12675870 | EAS | Ins/Del | ESRD | 246 | 316 | 176 | Healthy | 183 | 230 | 136 |
| Yang HY et al. | 23477970 | EAS | Ins/Del | ESRD | 683 | 833 | 533 | Healthy | 653 | 867 | 439 |
| Buraczynska M et al. | 16384824 | EUR | Ins/Del | ESRD | 745 | 688 | 802 | Healthy | 520 | 492 | 548 |
| Dixit M et al. | 12571380 | EUR | Ins/Del | ESRD | 26 | 32 | 20 | Healthy | 22 | 17 | 27 |
| Losito A et al. | 12454231 | EUR | Ins/Del | ESRD | 160 | 135 | 185 | Healthy | 169 | 128 | 210 |
| McLaughlin KJ et al. | 8901844 | EUR | Ins/Del | ESRD | 460 | 654 | 990 | Healthy | 371 | 353 | 389 |
| Nicod J et al. | 11918733 | EUR | Ins/Del | ESRD | 260 | 282 | 238 | Healthy | 260 | 261 | 259 |
| Ortiz MA et al. | 15012717 | EUR | Ins/Del | ESRD | 117 | 68 | 166 | Healthy | 129 | 80 | 178 |
| Schmidt A et al. | 8785402 | EUR | Ins/Del | ESRD | 106 | 105 | 107 | Healthy | 95 | 80 | 110 |
| van der Sman-de Beer F et al. | 16221224 | EUR | Ins/Del | ESRD | 415 | 400 | 430 | Healthy | 472 | 459 | 485 |
| Aucella F et al. | 12748347 | EUR | Ins/Del | ESRD | 461 | 315 | 607 | Healthy | 1307 | 916 | 1698 |
| Abuaisha AM et al. | 000433049000003 | ME | Ins/Del | ESRD | 86 | 37 | 135 | Healthy | 100 | 50 | 150 |
| Rahimi Z et al. | 28447048 | ME | Ins/Del | ESRD | 99 | 64 | 126 | Healthy | 117 | 89 | 145 |
| Zaare Nahandi et al. | 28270648 | ME | Ins/Del | ESRD | 30 | 23 | 37 | Healthy | 27 | 21 | 33 |

| | | | | | | | | | | | |
|---|---|---|---|---|---|---|---|---|---|---|---|
| Guo Y et al. | 00038665 3400063 | EAS | Ins/Del | IgAN | 45 | 54 | 36 | Healthy | 45 | 42 | 48 |
| Huang HD et al. | 21163122 | EAS | Ins/Del | IgAN | 130 | 155 | 105 | Healthy | 120 | 168 | 72 |
| Jung ES et al. | 21150220 | EAS | Ins/Del | IgAN | 261 | 298 | 224 | Healthy | 300 | 360 | 240 |
| Lau YK et al. | 15153745 | EAS | Ins/Del | IgAN | 118 | 154 | 82 | Healthy | 94 | 137 | 51 |
| Suzuki H et al. | 15481848 | EAS | Ins/Del | IgAN | 319 | 378 | 260 | Healthy | 270 | 328 | 212 |
| Yoon HJ et al. | 12220450 | EAS | Ins/Del | IgAN | 191 | 204 | 178 | Healthy | 233 | 268 | 198 |
| Yorioka T et al. | 8529313 | EAS | Ins/Del | IgAN | 48 | 67 | 29 | Healthy | 104 | 139 | 67 |
| Yoshida H et al. | 7593601 | EAS | Ins/Del | IgAN | 53 | 57 | 49 | Healthy | 46 | 62 | 30 |
| Burg M et al.* | 9352153 | EUR | Ins/Del | IgAN | 70 | 26 | 28 | Healthy | 60 | 60 | 60 |
| Drouet M et al. | 12005241 | EUR | Ins/Del | IgAN | 125 | 78 | 172 | Healthy | 83 | 54 | 100 |
| Harden PN et al. | 7791440 | EUR | Ins/Del | IgAN | 100 | 79 | 121 | Healthy | 98 | 76 | 120 |
| Pawlik M et al.* | 23681285 | EUR | Ins/Del | IgAN | 31 | 25 | 37 | Healthy | 187 | 180 | 194 |
| Pei Y et al. | 9259580 | EUR | Ins/Del | IgAN | 168 | 145 | 191 | Healthy | 100 | 91 | 109 |
| Schmidt S et al. (B) | 7485124 | EUR | Ins/Del | IgAN | 204 | 168 | 240 | Healthy | 234 | 197 | 271 |
| Stratta P et al. | 10352195 | EUR | Ins/Del | IgAN | 81 | 62 | 100 | Healthy | 50 | 38 | 62 |
| Burg M et al.* | 9352153 | EUR | Ins/Del | Primary GN | 46 | 44 | 48 | Healthy | 60 | 60 | 60 |
| Pawlik M et al.* | 23681285 | EUR | Ins/Del | Primary GN | 109 | 101 | 117 | Healthy | 187 | 180 | 194 |
| Stratta P et al. | 14767013 | EUR | Ins/Del | Primary GN | 117 | 96 | 138 | Organ donors | 171 | 134 | 208 |
| Zsom M et al. | 22111818 | EUR | Ins/Del | Primary GN | 73 | 62 | 84 | Healthy | 200 | 198 | 202 |
| Beige J et al. | 9259361 | EUR | Ins/Del | RTx recipients | 269 | 251 | 287 | Kidney Donor | 269 | 244 | 294 |
| El-Essawy AB et al. | 11926202 | EUR | Ins/Del | RTx recipients | 294 | 246 | 342 | Healthy | 181 | 170 | 192 |
| Hueso M et al. | 15284309 | EUR | Ins/Del | RTx recipients | 180 | 122 | 238 | Healthy & cadaveric renal | 113 | 96 | 130 |

| | | | | | | | | | | | |
|---|---|---|---|---|---|---|---|---|---|---|---|
| | | | | | | | | allograft donors | | | |
| Stratta P et al. | 19034872 | EUR | Ins/Del | RTx recipients | 169 | 122 | 216 | Healthy | 169 | 112 | 226 |
| Viklický O et al. | 11239522 | EUR | Ins/Del | RTx recipients | 30 | 28 | 32 | Healthy | 653 | 599 | 707 |
| Barnas U et al. | 9084972 | EUR | Ins/Del | T1DN | 63 | 45 | 55 | T1DM | 59 | 51 | 29 |
| Chowdhury TA et al. | 8877296 | EUR | Ins/Del | T1DN | 242 | 204 | 280 | T1DM | 166 | 143 | 189 |
| Currie D et al. | 20854388 | EUR | Ins/Del | T1DN | 718 | 603 | 777 | T1DM | 749 | 696 | 764 |
| De Cosmo S et al. | 10495473 | EUR | Ins/Del | T1DN | 175 | 125 | 225 | T1DM | 136 | 89 | 183 |
| Demurov LM et al. | A1997XE9 8500008 | EUR | Ins/Del | T1DN | 56 | 35 | 77 | T1DM | 76 | 72 | 80 |
| Hadjadj S et al. | 11181802 | EUR | Ins/Del | T1DN | 6 | 4 | 8 | T1DM | 251 | 208 | 294 |
| Hibberd ML et al. | 9025006 | EUR | Ins/Del | T1DN | 72 | 60 | 84 | T1DM | 86 | 57 | 115 |
| Marre M et al. | 8314010 | EUR | Ins/Del | T1DN | 62 | 43 | 81 | T1DM | 62 | 58 | 66 |
| Ringel J et al.* | 9049480 | EUR | Ins/Del | T1DN | 134 | 130 | 138 | T1DM | 226 | 208 | 244 |
| Schmidt S et al. (A) | 7783416 | EUR | Ins/Del | T1DN | 114 | 86 | 142 | T1DM | 133 | 101 | 165 |
| Shestakova MV et al. | 00024241 0600010 | EUR | Ins/Del | T1DN | 63 | 65 | 61 | T1DM | 66 | 78 | 54 |
| Tarnow L et al. | 7729604 | EUR | Ins/Del | T1DN | 198 | 175 | 221 | T1DM | 190 | 169 | 211 |
| van Ittersum FJ et al. | 10862638 | EUR | Ins/Del | T1DN | 30 | 33 | 27 | T1DM | 188 | 192 | 184 |
| Walder B et al. | None | EUR | Ins/Del | T1DN | 55 | 49 | 61 | T1DM | 44 | 40 | 48 |
| Fujisawa T et al. | 7555560 | EAS | Ins/Del | T2DN | 54 | 71 | 37 | T2DM | 35 | 46 | 24 |
| Ha SK et al. | 12722028 | EAS | Ins/Del | T2DN | 140 | 132 | 148 | T2DM | 99 | 123 | 75 |
| Hsieh MC et al. | 10862639 | EAS | Ins/Del | T2DN | 179 | 219 | 139 | T2DM | 157 | 222 | 92 |
| Mizuiri S et al. | 7477652 | EAS | Ins/Del | T2DN | 80 | 72 | 88 | T2DM | 31 | 33 | 29 |
| Nakajima S et al. | 8941475 | EAS | Ins/Del | T2DN | 54 | 67 | 41 | T2DM | 41 | 55 | 27 |
| Ohno T et al. | 8596493 | EAS | Ins/Del | T2DN | 25 | 28 | 22 | T2DM | 53 | 81 | 25 |
| Shin Shin Y et al. | 15331206 | EAS | Ins/Del | T2DN | 82 | 112 | 52 | T2DM | 59 | 70 | 48 |
| Thomas GN et al. | 11200871 | EAS | Ins/Del | T2DN | 51 | 72 | 30 | T2DM | 255 | 346 | 164 |
| Wang M et al. | 26973293 | EAS | Ins/Del | T2DN | 106 | 78 | 56 | T2DM | 741 | 1020 | 438 |
| Wu SH et al. | 11776100 | EAS | Ins/Del | T2DN | 71 | 75 | 67 | T2DM | 41 | 58 | 24 |

| | | | | | | | | | | | |
|---|---|---|---|---|---|---|---|---|---|---|---|
| Young RP et al. | 9540028 | EAS | Ins/Del | T2DN | 20 | 27 | 13 | T2DM | 54 | 72 | 36 |
| Dudley CR et al. | 8587251 | EUR | Ins/Del | T2DN | 163 | 246 | 288 | T2DM | 267 | 246 | 288 |
| Fradin S et al. | 11938025 | EUR | Ins/Del | T2DN | 39 | 94 | 142 | T2DM | 118 | 94 | 142 |
| Ng DP et al. | 16936219 | EUR | Ins/Del | T2DN | 291 | 147 | 187 | T2DM | 167 | 147 | 187 |
| Nikzamir A et al. | 19502259 | EUR | Ins/Del | T2DN | 48 | 159 | 131 | T2DM | 145 | 159 | 131 |
| Schmidt S et al. (B) | 9269698 | EUR | Ins/Del | T2DN | 311 | 278 | 416 | T2DM | 347 | 278 | 416 |
| Al-Harbi EM et al. | 21207118 | ME | Ins/Del | T2DN | 110 | 63 | 157 | T2DM | 196 | 125 | 267 |
| Alharbi SA et al. | 000415063100003 | ME | Ins/Del | T2DN | 61 | 31 | 91 | T2DM | 61 | 47 | 75 |
| Araz M et al. | 11640993 | ME | Ins/Del | T2DN | 62 | 52 | 72 | T2DM | 123 | 103 | 143 |
| El-Baz R et al. | 22554825 | ME | Ins/Del | T2DN | 102 | 66 | 138 | T2DM | 100 | 82 | 118 |
| Ergen HA et al. | 15365253 | ME | Ins/Del | T2DN | 25 | 21 | 29 | T2DM | 50 | 31 | 69 |
| Eroglu Z et al. | 18413162 | ME | Ins/Del | T2DN | 46 | 43 | 49 | T2DM | 56 | 50 | 62 |
| Felehgari V et al. | 20830509 | ME | Ins/Del | T2DN | 68 | 42 | 94 | T2DM | 72 | 60 | 84 |
| Sancakdar E et al. | 000367541400005 | ME | Ins/Del | T2DN | 194 | 180 | 208 | T2DM | 100 | 62 | 138 |
| Ahluwalia TS et al. | 19108684 | SAS | Ins/Del | T2DN | 240 | 152 | 328 | T2DM | 200 | 215 | |
| Kumar R et al. | 23846111 | SAS | Ins/Del | T2DN | 407 | 448 | 366 | T2DM | 185 | 190 | |
| Movva S et al. | 17616353 | SAS | Ins/Del | T2DN | 174 | 182 | 166 | T2DM | 175 | 222 | |
| Naresh VV et al. | 20535249 | SAS | Ins/Del | T2DN | 30 | 19 | 41 | T2DM | 30 | 35 | |
| Shaikh R et al. | 000312053000001 | SAS | Ins/Del | T2DN | 168 | 134 | 202 | T2DM | 296 | 394 | |
| Vishwanathan V et al. | 11867868 | SAS | Ins/Del | T2DN | 86 | 79 | 93 | T2DM | 23 | 28 | |
| Doi Y et al. | 8720609 | EAS | Ins/Del | T2DN-ESRD | 100 | 115 | 85 | T2DM | 124 | 168 | |
| Jayapalan JJ et al. | 21031056 | EAS | Ins/Del | T2DN-ESRD | 127 | 163 | 91 | T2DM | 81 | 93 | |
| Lu M et al. | 27633502 | EAS | Ins/Del | T2DN-ESRD | 210 | 238 | 182 | T2DM | 222 | 307 | |
| Park HC et al. | 16385653 | EAS | Ins/Del | T2DN-ESRD | 103 | 103 | 103 | T2DM | 88 | 111 | |

| | | | | | | | | | | | |
|---|---|---|---|---|---|---|---|---|---|---|---|
| Taniwaki H et al. | 11522715 | EAS | Ins/Del | T2DN-ESRD | 42 | 53 | 31 | T2DM | 69 | 88 | |
| Grzeszczak W et al. | 9727375 | EUR | Ins/Del | T2DN-ESRD | 127 | 128 | 126 | T2DM | 254 | 244 | |
| Ringel J et al.* | 9049480 | EUR | Ins/Del | T2DN-ESRD | 161 | 150 | 172 | T2DM | 140 | 141 | |
| Schmidt S et al. (A) | 9075119 | EUR | Ins/Del | T2DN-ESRD | 61 | 29 | 93 | T2DM | 347 | 416 | |

*Burg M et al., Pawlik M et al., and Ringel J et al., are included twice as these articles contained two phenotypic comparisons

Abbreviations: EAS, East Asian; EUR, European; ME, Middle Eastern; SAS, South Asian; CKD, Chronic Kidney Disease; ESRD, End-Stage Renal Disease; IgAN, IgA Nephropathy; GN, Glomerulonephritis; RTx, Renal Transplant; T1DM, Type 1 Diabetes Mellitus; T1DN, Type 1 Diabetic Nephropathy; T2DM, Type 2 Diabetes Mellitus; T2DN, Type 2 Diabetic Nephropathy.

**Supplementary Table S5:** Data included in quantitative analysis for *AGT*

| | | | | Cases | | | | Controls | | | |
|---|---|---|---|---|---|---|---|---|---|---|---|
| **Author** | **PMID/ WOS ID** | **Ethnicity** | **SNP** | **Definition** | **N** | **T Allele Frequency** | **C Allele Frequency** | **Definition** | **N** | **T Allele Frequency** | **C Allele Frequency** |
| Beige J et al. | 8856207 | EUR | rs699 | ESRD | 269 | 285 | 253 | Healthy | 269 | 307 | 229 |
| Buraczynska M et al. | 16384824 | EUR | rs699 | ESRD | 745 | 734 | 756 | Healthy | 520 | 564 | 476 |
| Losito A et al. | 12454231 | EUR | rs699 | ESRD | 160 | 165 | 155 | Healthy | 169 | 190 | 148 |
| Lovati E et al. | 11422735 | EUR | rs699 | ESRD | 260 | 299 | 221 | Healthy | 327 | 393 | 261 |
| Doria A et al. | 8621207 | EUR | rs699 | T1DN | 139 | 158 | 120 | T1DM | 75 | 96 | 54 |
| Fogarty DG et al. | 8772723 | EUR | rs699 | T1DN | 95 | 114 | 76 | T1DM | 100 | 129 | 71 |
| Möllsten A et al. | 18413189 | EUR | rs699 | T1DN | 73 | 62 | 34 | T1DM | 197 | 220 | 174 |
| Ringel J et al. | 9049480 | EUR | rs699 | T1DN | 134 | 145 | 123 | T1DM | 226 | 257 | 195 |
| Schmidt S et al.* | 8918618 | EUR | rs699 | T1DN | 108 | 121 | 95 | T1DM | 120 | 132 | 108 |
| van Ittersum FJ et al. | 10862638 | EUR | rs699 | T1DN | 30 | 31 | 29 | T1DM | 188 | 238 | 138 |
| Walder B et al. | None | EUR | rs699 | T1DN | 55 | 60 | 50 | T1DM | 44 | 63 | 25 |
| Ohno T et al. | 8596493 | EAS | rs699 | T2DN | 25 | 16 | 34 | T2DM | 53 | 25 | 81 |
| Oue T et al. | 10580616 | EAS | rs699 | T2DN | 27 | 6 | 36 | T2DM | 40 | 13 | 47 |
| Thomas GN et al. | 11200871 | EAS | rs699 | T2DN | 51 | 18 | 84 | T2DM | 255 | 79 | 431 |
| Wang M et al. | 26973293 | EAS | rs699 | T2DN | 189 | 19 | 175 | T2DM | 473 | 154 | 792 |
| Wu SH et al. | 11776100 | EAS | rs699 | T2DN | 71 | 21 | 121 | T2DM | 41 | 26 | 56 |
| Young RP et al. | 9540028 | EAS | rs699 | T2DN | 20 | 4 | 36 | T2DM | 54 | 25 | 83 |
| Fradin S et al. | 11938025 | EUR | rs699 | T2DN | 39 | 45 | 33 | T2DM | 118 | 139 | 97 |
| Freire MB et al. | 9535411 | EUR | rs699 | T2DN | 117 | 134 | 96 | T2DM | 125 | 148 | 88 |
| Makuc J et al. | 28488548 | EUR | rs699 | T2DN | 276 | 294 | 258 | T2DM | 375 | 394 | 356 |
| Schmidt S et al.* | 8918618 | EUR | rs699 | T2DN | 127 | 149 | 105 | T2DM | 107 | 117 | 97 |
| Zychma MJ et al. | 11096141 | EUR | rs699 | T2DN | 127 | 133 | 121 | T2DM | 243 | 242 | 244 |
| Eroglu Z et al. | 18413162 | ME | rs699 | T2DN | 46 | 48 | 44 | T2DM | 56 | 62 | 50 |
| Reis KA et al. | 21500980 | ME | rs699 | T2DN | 108 | 114 | 102 | T2DM | 111 | 89 | 133 |
| Sancakdar E et al. | 000367541 400005 | ME | rs699 | T2DN | 194 | 189 | 199 | T2DM | 100 | 95 | 105 |

| | | | | | | | | | | | |
|---|---|---|---|---|---|---|---|---|---|---|---|
| Gao J et al. | 26588355 | EAS | rs699 | IgAN | 351 | 144 | 556 | Healthy | 310 | 125 | 495 |
| Guo Y et al. | 000386653400063 | EAS | rs699 | IgAN | 45 | 19 | 71 | Healthy | 45 | 16 | 74 |
| Huang HD et al. | 21163122 | EAS | rs699 | IgAN | 130 | 35 | 225 | Healthy | 120 | 40 | 200 |
| Kim SM et al. | 19729965 | EAS | rs699 | IgAN | 238 | 89 | 387 | Healthy | 300 | 115 | 485 |
| Lau YK et al. | 15153745 | EAS | rs699 | IgAN | 118 | 27 | 209 | Healthy | 94 | 31 | 157 |
| Kawada N et al. | 000071880400005 | EAS | rs699 | ESRD | 216 | 91 | 333 | Healthy | 208 | 64 | 340 |
| Su SL et al. | 29296205 | EAS | rs699 | ESRD | 634 | 194 | 1074 | Healthy | 739 | 279 | 1478 |
| Wang AY et al. | 12675870 | EAS | rs699 | ESRD | 246 | 76 | 416 | Healthy | 183 | 64 | 302 |

*Schmidt S et al., is included twice as the article contained two phenotypic comparisons.

Abbreviations: EAS, East Asian; EUR, European; ME, Middle Eastern; ESRD, End-Stage Renal Disease; IgAN, IgA Nephropathy; T1DM, Type 1 Diabetes Mellitus; T1DN, Type 1 Diabetic Nephropathy; T2DM, Type 2 Diabetes Mellitus; T2DN, Type 2 Diabetic Nephropathy.

**Supplementary Table S6:** Data included in quantitative analysis for *AGTR1*

| | | | | Cases | | | | Controls | | | |
|---|---|---|---|---|---|---|---|---|---|---|---|
| Author | PMID | Ethnicity | SNP | Definition | N | A Allele Frequency | C Allele Frequency | Definition | N | A Allele Frequency | C Allele Frequency |
| Gao J et al. | 26588355 | EAS | rs5186 | IgAN | 351 | 659 | 43 | Healthy | 310 | 577 | 43 |
| Huang HD et al. | 21163122 | EAS | rs5186 | IgAN | 130 | 243 | 17 | Healthy | 120 | 220 | 20 |
| Kim SM et al. | 19729965 | EAS | rs5186 | IgAN | 238 | 452 | 24 | Healthy | 300 | 576 | 24 |
| Lau YK et al. | 15153745 | EAS | rs5186 | IgAN | 118 | 227 | 9 | Healthy | 94 | 178 | 10 |
| Currie D et al. | 20854388 | EUR | rs5186 | T1DN | 718 | 1029 | 385 | T1DM | 749 | 1052 | 418 |
| Doria A et al. | 9389421 | EUR | rs5186 | T1DN | 73 | 99 | 47 | T1DM | 79 | 119 | 39 |
| Möllsten A et al. | 18413189 | EUR | rs5186 | T1DN | 72 | 78 | 18 | T1DM | 197 | 290 | 104 |
| Savage DA et al. | 10328465 | EUR | rs5186 | T1DN | 95 | 136 | 54 | T1DM | 97 | 137 | 57 |
| Tarnow L et al. | 8671962 | EUR | rs5186 | T1DN | 198 | 287 | 109 | T1DM | 190 | 274 | 106 |
| van Ittersum FJ et al. | 10862638 | EUR | rs5186 | T1DN | 30 | 47 | 13 | T1DM | 188 | 238 | 138 |
| Thomas GN et al. | 11200871 | EAS | rs5186 | T2DN | 51 | 99 | 3 | T2DM | 255 | 483 | 27 |
| Wu SH et al. | 11776100 | EAS | rs5186 | T2DN | 71 | 127 | 15 | T2DM | 41 | 74 | 8 |
| Young RP et al. | 9540028 | EAS | rs5186 | T2DN | 20 | 38 | 2 | T2DM | 54 | 103 | 5 |
| Ahluwalia TS et al. | 19108684 | SAS | rs5186 | T2DN | 240 | 320 | 160 | T2DM | 255 | 381 | 129 |
| Prasad P et al. | 16672053 | SAS | rs5186 | T2DN | 196 | 360 | 26 | T2DM | 225 | 417 | 33 |
| Shah VN et al. | 23081748 | SAS | rs5186 | T2DN | 596 | 789 | 403 | T2DM | 566 | 840 | 282 |

Abbreviations: EAS, East Asian; EUR, European; SAS, South Asian; IgAN, IgA Nephropathy; T1DM, Type 1 Diabetes Mellitus; T1DN, Type 1 Diabetic Nephropathy; T2DM, Type 2 Diabetes Mellitus; T2DN, Type 2 Diabetic Nephropathy.

**Supplementary Table S7.** Included studies not complying with Hardy Weinberg equilibrium in cases or controls

| | | | | | Cases | | Controls | |
|---|---|---|---|---|---|---|---|---|
| **Gene** | **Author** | **PMID/ WODS ID** | **Ethnicity** | **SNP** | **Definition** | **P value (where significant)** | **Definition** | **P value (where significant)** |
| *ACE* | Jung ES et al. | 21150220 | EAS | Ins/Del | IgAN | | Healthy | 0.016 |
| | Shanmuganathan R et al. | 26440392 | SAS | Ins/Del | CKD | $1.31x10^{-04}$ | Healthy | $1.9x10^{-05}$ |
| | Tang FY et al. | 18629615 | EAS | Ins/Del | ESRD | $1.8x10^{-05}$ | Healthy | $1x10^{-06}$ |
| | Nicod J et al. | 11918733 | EUR | Ins/Del | ESRD | 0.0008 | Healthy | 0.004 |
| | McLaughlin KJ et al. | 8901844 | EUR | Ins/Del | ESRD | 0.04 | Healthy | |
| | Yorioka T et al. | 8529313 | EAS | Ins/Del | IgAN | 0.013 | Healthy | |
| | Yoshida H et al. | 7593601 | EAS | Ins/Del | IgAN | 0.01 | Healthy | |
| | Yoon HJ et al. | 12220450 | EAS | Ins/Del | IgAN | 0.002 | Healthy | |
| | Schmidt S et al. | 7485124 | EUR | Ins/Del | IgAN | 0.007 | Healthy | |
| | Suzuki H et al. | 15481848 | EAS | Ins/Del | IgAN | 0.036 | Healthy | 0.004 |
| | Zsom M et al. | 22111818 | EUR | Ins/Del | Primary GN | 0.005 | Healthy | |
| | Currie D et al. | 20854388 | EUR | Ins/Del | T1DN | | T1DM | 0.005 |
| | Ringel J et al. | 9049480 | EUR | Ins/Del | T1DN | | T1DM | 0.018 |
| | Tarnow L et al. | 7729604 | EUR | Ins/Del | T1DN | | T1DM | 0.013 |
| | Schmidt S et al. | 7783416 | EUR | Ins/Del | T1DN | 0.002 | T1DM | |
| | Mizuiri S et al. | 7477652 | EAS | Ins/Del | T2DN | 0.019 | T2DM | |
| | Hsieh MC et al. | 10862639 | EAS | Ins/Del | T2DN | $4.2x10^{-05}$ | T2DM | 0.004 |
| | Ha SK et al. | 12722028 | EAS | Ins/Del | T2DN | | T2DM | 0.026 |
| | Schmidt S et al. | 9269698 | EUR | Ins/Del | T2DN | 0.0147 | T2DM | |
| | El-Baz R et al. | 22554825 | ME | Ins/Del | T2DN | 0.0025 | T2DM | $1x10^{-06}$ |
| | Sancakdar E et al. | 000367541400005 | ME | Ins/Del | T2DN | 0.0076 | T2DM | 0.003 |
| | Kumar R et al. | 23846111 | SAS | Ins/Del | T2DN | 0.0016 | T2DM | 0.0003 |
| | Park HC et al. | 16385653 | EAS | Ins/Del | T2DN-ESRD | | T2DM | 0.022 |
| *AGT* | Fogarty DG et al. | 8772723 | EUR | rs699 | T1DN | | T1DM | 0.044 |
| | Wu SH et al. | 11776100 | EAS | rs699 | T2DN | 0.0008 | T2DM | |
| | Freire MB et al. | 9535411 | EUR | rs699 | T2DN | 0.022 | T2DM | |
| | Reis KA et al. | 21500980 | ME | rs699 | T2DN | 0.006 | T2DM | |

| | | | | | | | | |
|---|---|---|---|---|---|---|---|---|
| | Fradin S et al. | 11938025 | EUR | rs699 | T2DN | 0.008 | T2DM | |
| | Su SL et al. | 29296205 | EAS | rs699 | ESRD | | Healthy | 0.010 |
| *AGTR1* | Ahluwalia TS et al. | 19108684 | SAS | rs5186 | T2DN | | T2DM | 0.0002 |
| | Shah VN et al. | 23081748 | SAS | rs5186 | T2DN | | T2DM | $4.2x10^{-08}$ |
| | Lau YK et al. | 15153745 | EAS | rs5186 | IgAN | 0.038 | Healthy | |
| | Möllsten A et al. | 8856207 | EUR | rs5186 | T1DN | 0.029 | T1DM | |

Abbreviations: EAS, East Asian; EUR, European; ME, Middle Eastern; SAS, South Asian; CKD, Chronic Kidney Disease; ESRD, End-Stage Renal Disease; GN, Glomerulonephritis; IgAN, IgA Nephropathy; T1DM, Type 1 Diabetes Mellitus; T1DN, Type 1 Diabetic Nephropathy; T2DM, Type 2 Diabetes Mellitus; T2DN, Type 2 Diabetic Nephropathy.
