# Supplementary material for: Genetic associations between genes in the renin-angiotensin-aldosterone system and renal disease: a systematic review and meta-analysis
Source: BMJ Open. 2019 May 1;9(4):e026777. doi: 10.1136/bmjopen-2018-026777 (PMC6501980; doi:10.1136/bmjopen-2018-026777)
Supplement: Supplementary data [file bmjopen-2018-026777supp002.pdf]

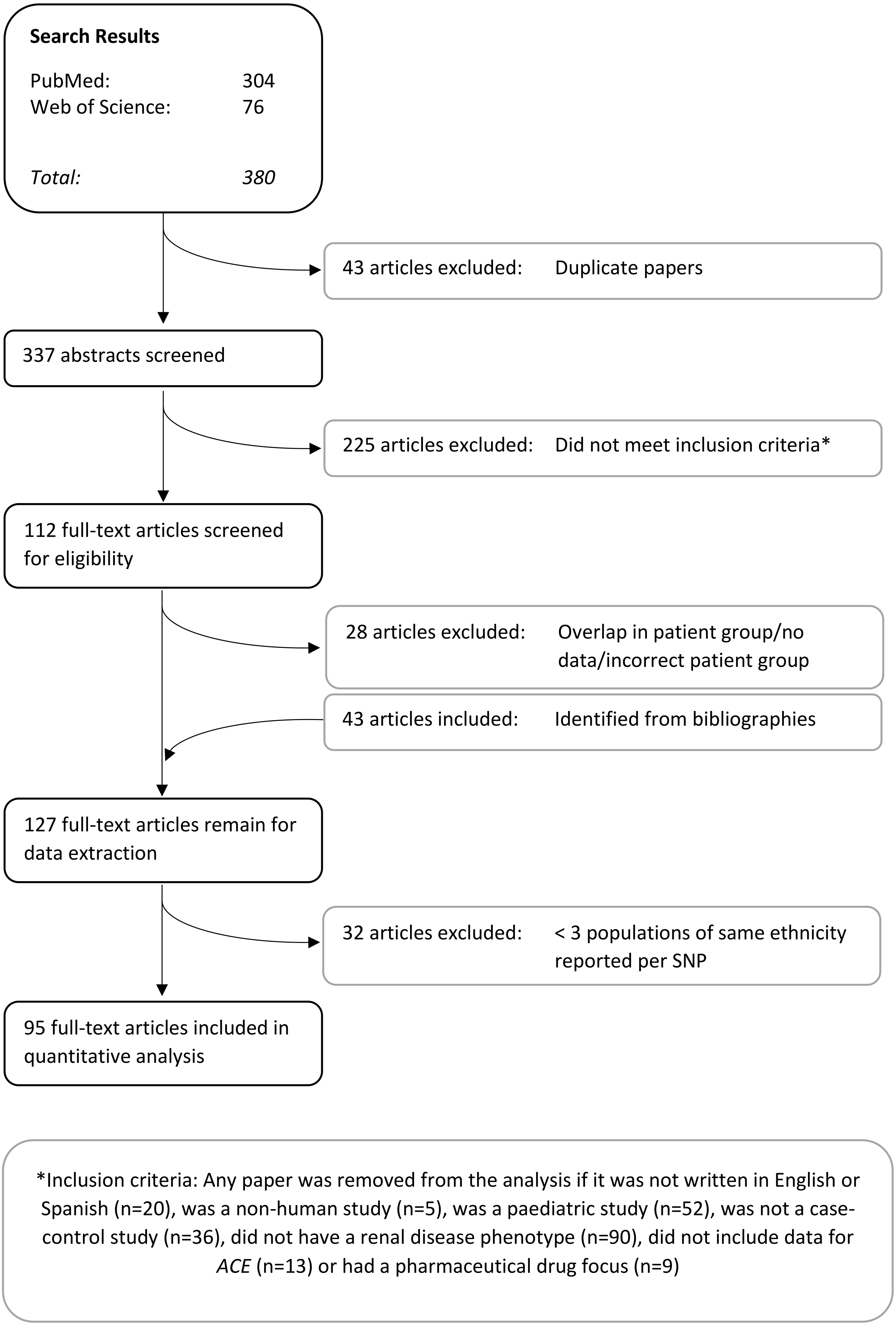

Supplementary Figure S1a: *ACE* Study Flow Diagram

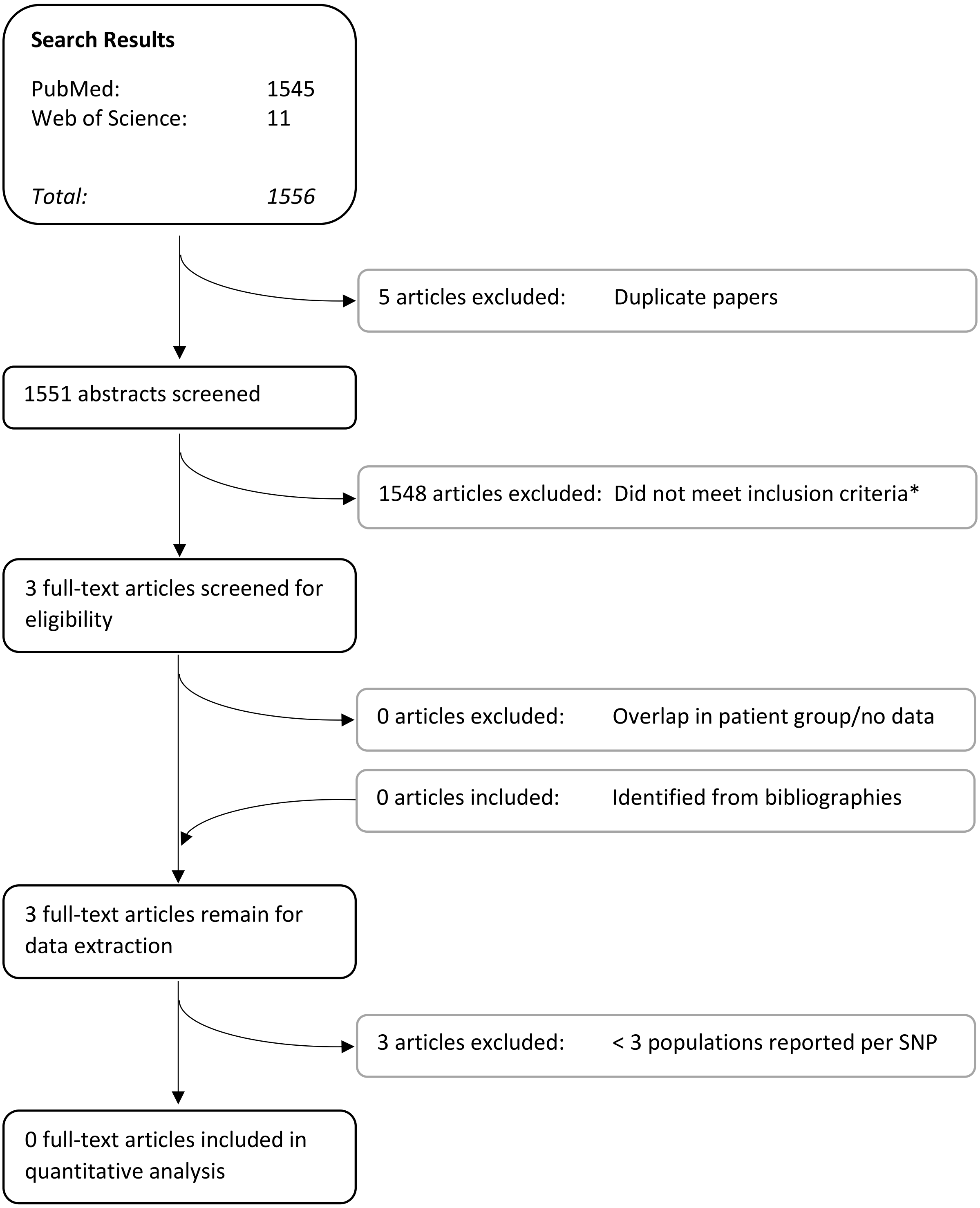

*Inclusion criteria: Any paper was removed from the analysis if it was not written in English or Spanish (n=55), was a non-human study (n=119), was a paediatric study (n=149), was not a case-control study (n=53), did not have a renal disease phenotype (n=693), did not include data for *ACE2* (n=435) or had a pharmaceutical drug focus (n=44)

Supplementary Figure S1b: *ACE2* Study Flow Diagram

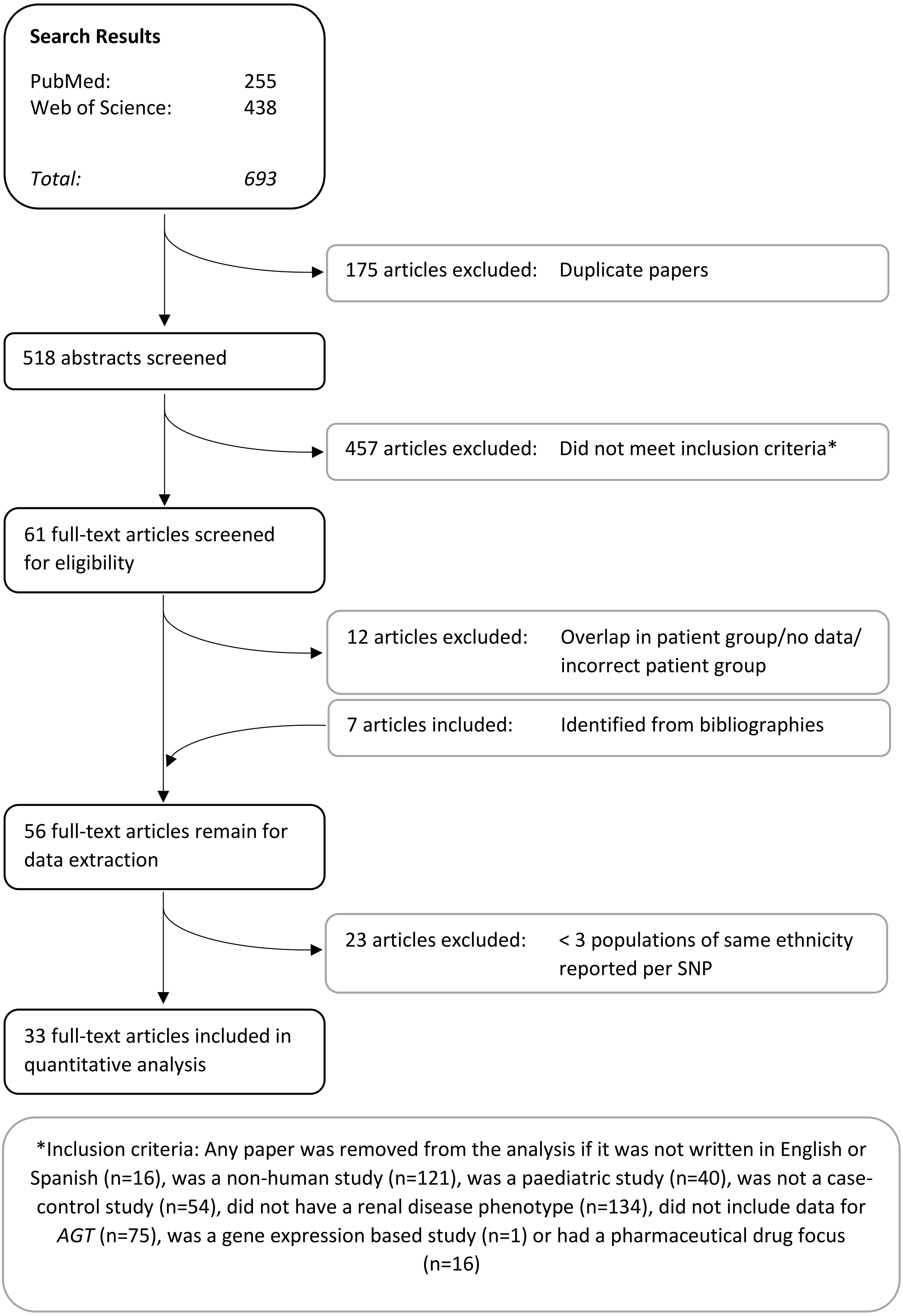

Supplementary Figure S1c: *AGT* Study Flow Diagram

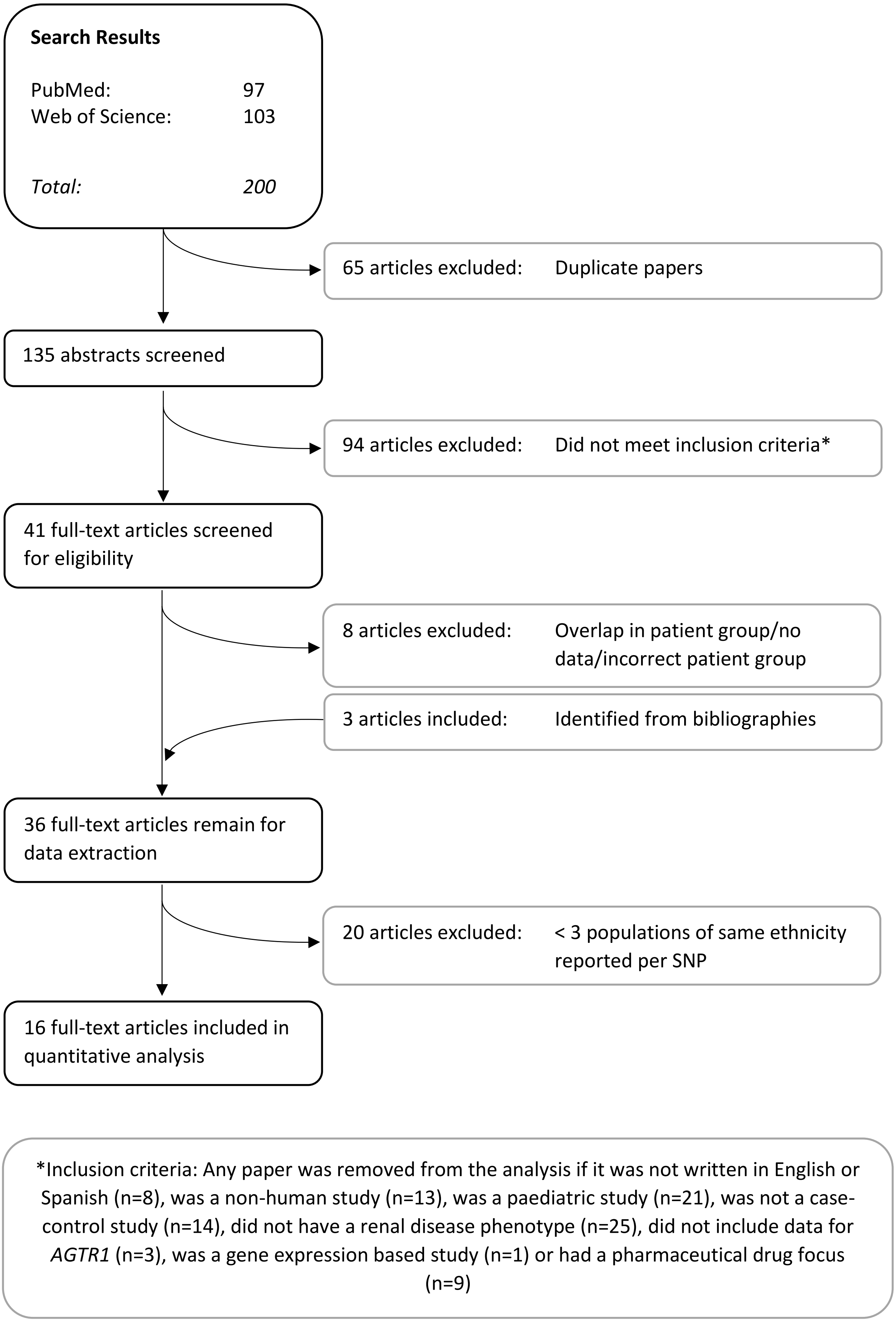

Supplementary Figure S1d: *AGTR1* Study Flow Diagram

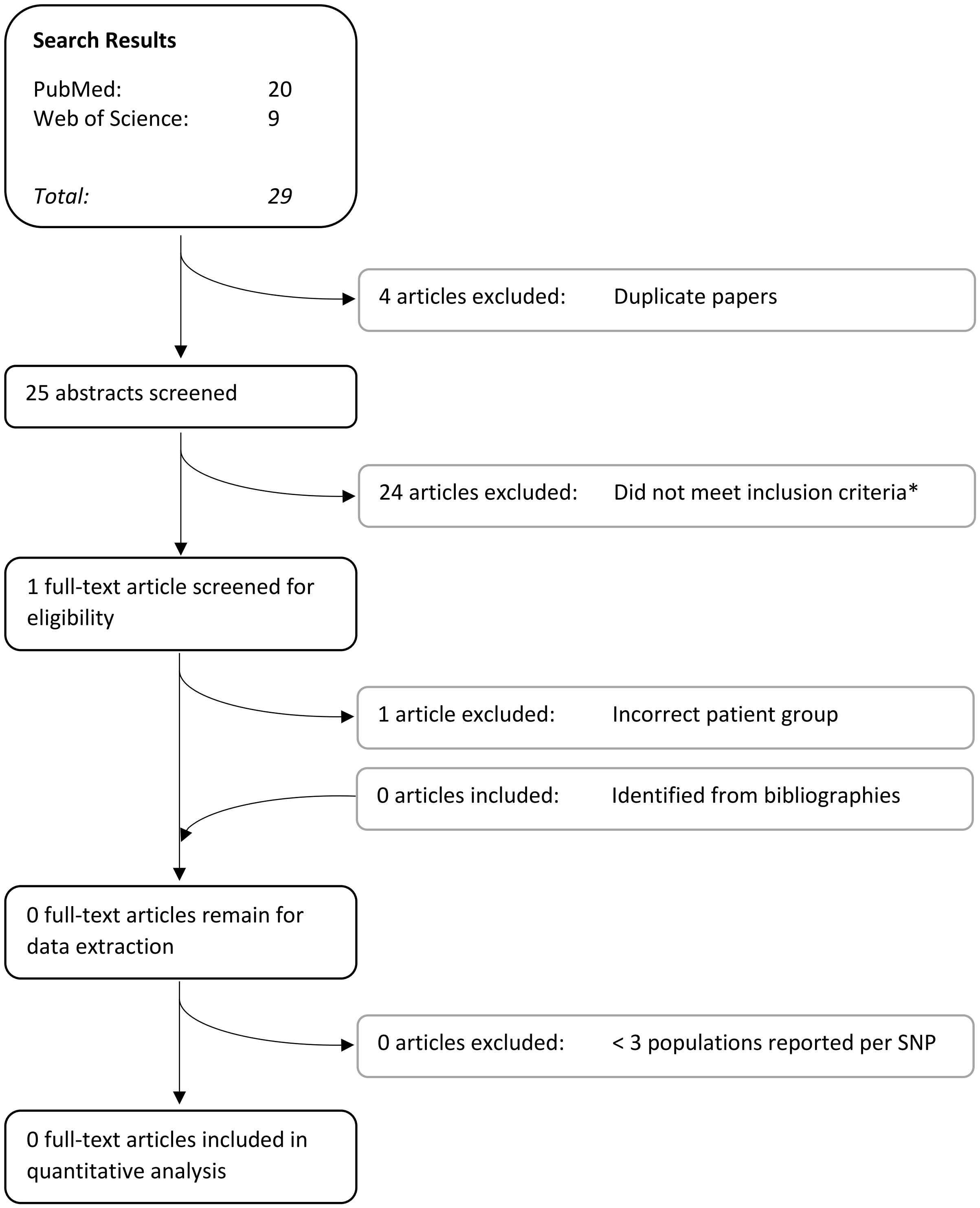

*Inclusion criteria: Any paper was removed from the analysis if it was not written in English or Spanish (n=1), was a non-human study (n=2), was a paediatric study (n=6), was not a case-control study (n=4), did not have a renal disease phenotype (n=5) or did not include data for *AGTR2* (n=6)

Supplementary Figure S1e: *AGTR2* Study Flow Diagram

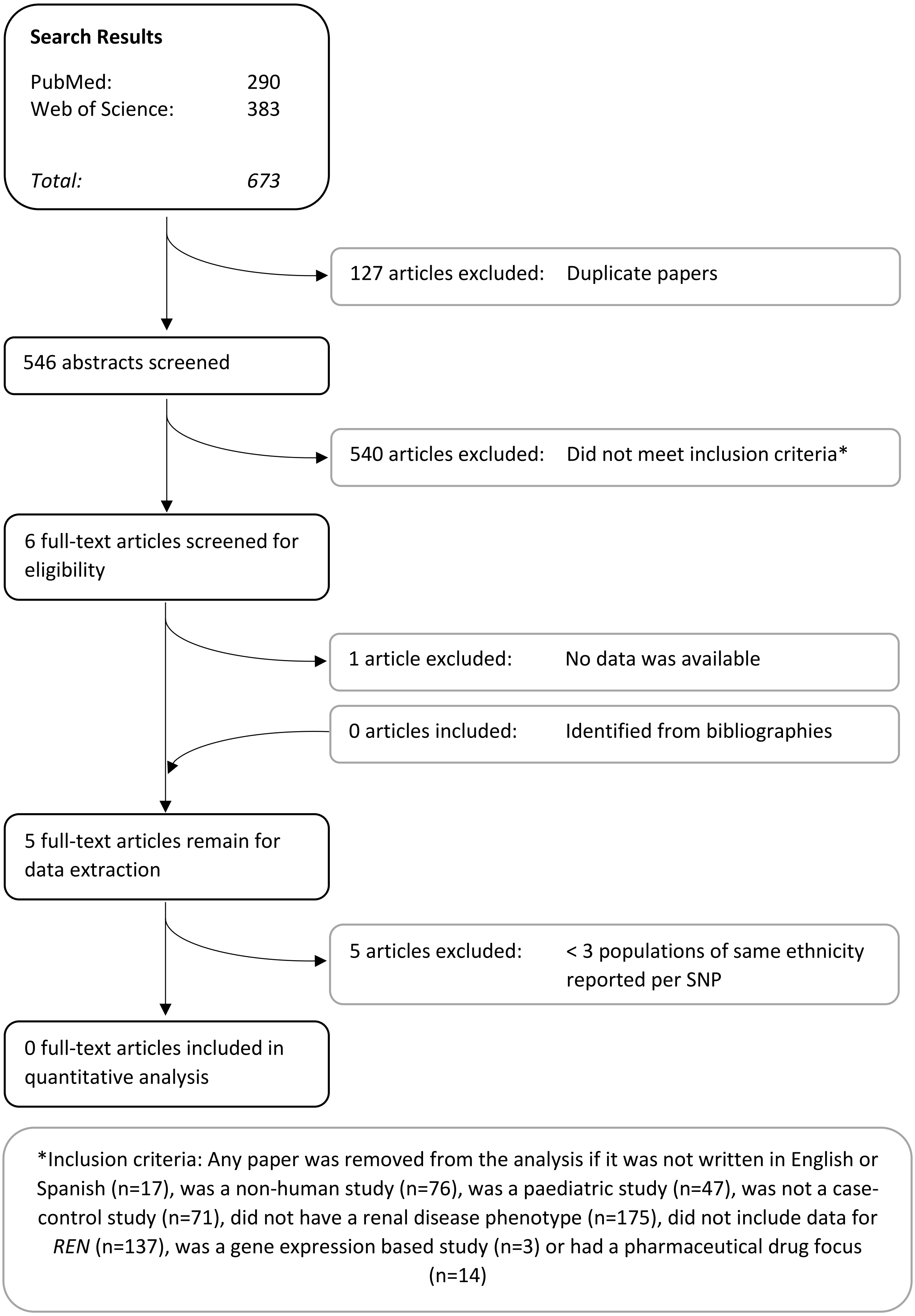

Supplementary Figure S1f: *REN* Study Flow Diagram

Study or Subgroup	Case Events	Case Total	Control Events	Control Total	Weight	Odds Ratio M-H, Random, 95% CI
Barnas 1997	45	100	51	80	4.0%	0.47 [0.25, 0.85]
Chowdhury 1996	204	484	143	332	10.6%	0.96 [0.73, 1.28]
Currie 2010	603	1380	696	1460	15.8%	0.85 [0.73, 0.99]
De Cosmo 1999	125	350	89	272	8.9%	1.14 [0.82, 1.60]
Demurov 1997	35	112	72	152	5.2%	0.51 [0.30, 0.84]
Hadjadj 2001	4	12	208	502	1.2%	0.71 [0.21, 2.38]
Hibberd 1997	60	144	57	172	6.0%	1.44 [0.91, 2.28]
Marre 1994	43	124	58	124	5.2%	0.60 [0.36, 1.01]
Ringel 1997	130	268	208	452	9.9%	1.11 [0.82, 1.50]
Schmidt 1995 A	86	228	101	266	8.1%	0.99 [0.69, 1.42]
Shestakova 2006	65	126	78	132	5.5%	0.74 [0.45, 1.21]
Tarnow 1995	175	396	169	380	10.5%	0.99 [0.74, 1.31]
van Ittersum 2000	33	60	192	376	4.7%	1.17 [0.68, 2.02]
Walder 1998	49	110	40	88	4.5%	0.96 [0.55, 1.69]
**Total (95% CI)**		**3894**		**4788**	**100.0%**	**0.91 [0.79, 1.04]**
Total events	1657		2162			

Heterogeneity: Tau² = 0.02; Chi² = 22.72, df = 13 (P = 0.05); I² = 43%
Test for overall effect: Z = 1.37 (P = 0.17)

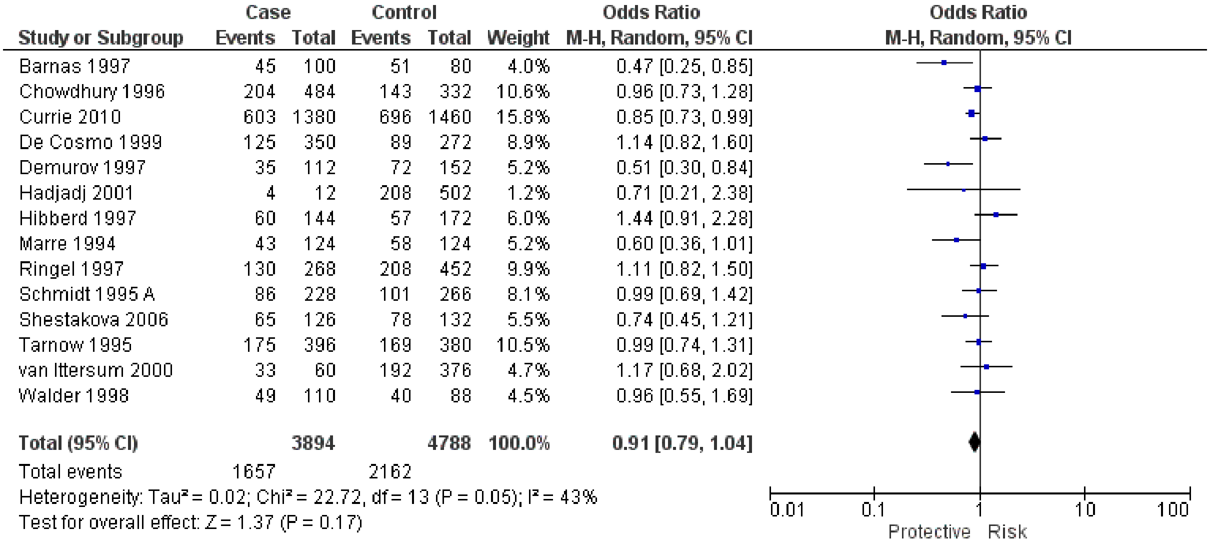

Supplementary Figure S2a: Forest plot - *ACE* I/D investigation, individuals with type 1 diabetic nephropathy compared to individuals with type 1 diabetes mellitus in a European population (*ACE* insertion compared to deletion).

Study or Subgroup	Case Events	Case Total	Control Events	Control Total	Weight	Odds Ratio M-H, Random, 95% CI
Dudley 1995	147	326	246	534	21.8%	0.96 [0.73, 1.27]
Fradin 2002	34	78	94	236	16.9%	1.17 [0.70, 1.96]
Ng 2006	242	582	147	334	21.9%	0.91 [0.69, 1.19]
Nikzamir 2009	21	96	159	290	16.5%	0.23 [0.13, 0.39]
Schmidt 1997 B	251	622	278	694	22.8%	1.01 [0.81, 1.26]
**Total (95% CI)**		**1704**		**2088**	**100.0%**	**0.78 [0.54, 1.15]**
Total events	695		924			

Heterogeneity: Tau² = 0.15; Chi² = 27.08, df = 4 (P < 0.0001); I² = 85%
Test for overall effect: Z = 1.26 (P = 0.21)

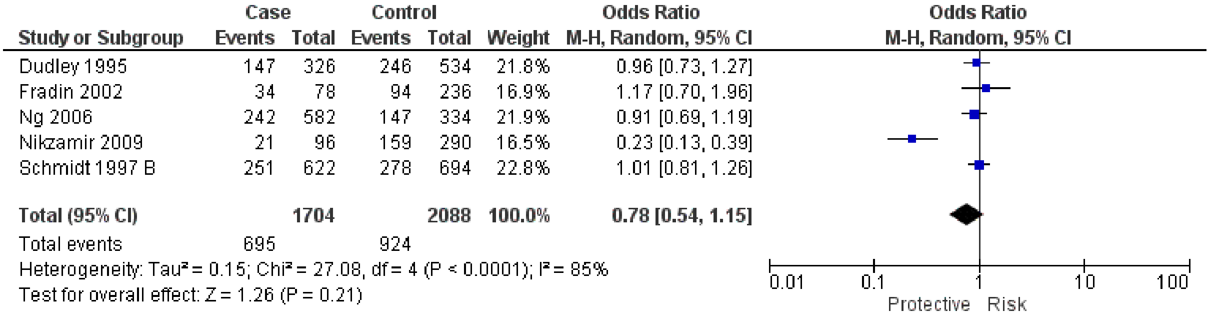

Supplementary Figure S2b: Forest plot - *ACE* I/D investigation, individuals with type 2 diabetic nephropathy compared to individuals with type 2 diabetes mellitus in a European population (*ACE* insertion compared to deletion).

Study or Subgroup	Case Events	Case Total	Control Events	Control Total	Weight	Odds Ratio M-H, Random, 95% CI	Odds Ratio M-H, Random, 95% CI
Al-Harbi 2011	63	220	125	392	12.7%	0.86 [0.60, 1.23]	
Alharbi 2017	31	122	47	122	10.2%	0.54 [0.31, 0.94]	
Araz 2001	52	124	103	246	11.7%	1.00 [0.65, 1.55]	
El-Baz 2012	66	204	82	200	12.1%	0.69 [0.46, 1.03]	
Ergen 2004	21	50	31	100	8.4%	1.61 [0.80, 3.26]	
Eroglu 2008	43	92	50	112	10.2%	1.09 [0.63, 1.89]	
Felehgari 2011	42	136	60	144	11.0%	0.63 [0.38, 1.02]	
Rahimi 2012	42	136	60	144	11.0%	0.63 [0.38, 1.02]	
Sancakdar 2015	180	388	62	200	12.7%	1.93 [1.34, 2.76]	
**Total (95% CI)**		**1472**		**1660**	**100.0%**	**0.91 [0.67, 1.22]**	
Total events	540		620				

Heterogeneity: Tau² = 0.15; Chi² = 29.48, df = 8 (P = 0.0003); I² = 73%
Test for overall effect: Z = 0.65 (P = 0.52)

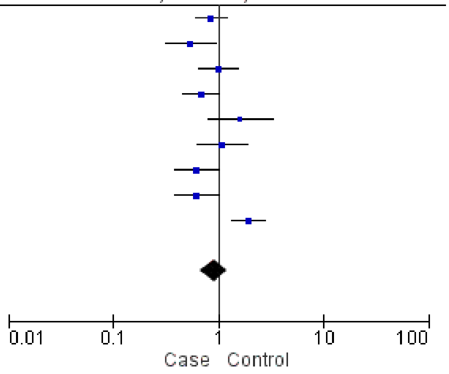

Supplementary Figure S2c: Forest plot - *ACE* I/D investigation, individuals with type 2 diabetic nephropathy compared to individuals with type 2 diabetes mellitus in a Middle Eastern population (*ACE* insertion compared to deletion).

Study or Subgroup	Case Events	Case Total	Control Events	Control Total	Weight	Odds Ratio M-H, Random, 95% CI	Odds Ratio M-H, Random, 95% CI
Ahluwalia 2009	124	236	99	196	26.9%	1.08 [0.74, 1.58]	
Chen 2014	314	466	617	898	28.0%	0.94 [0.74, 1.20]	
Nagamani 2015	152	294	195	422	27.6%	1.25 [0.92, 1.68]	
Shanmuganathan 2015	10	60	55	60	17.6%	0.02 [0.01, 0.06]	
**Total (95% CI)**		**1056**		**1576**	**100.0%**	**0.53 [0.24, 1.14]**	
Total events	600		966				

Heterogeneity: Tau² = 0.53; Chi² = 50.55, df = 3 (P < 0.00001); I² = 94%
Test for overall effect: Z = 1.63 (P = 0.10)

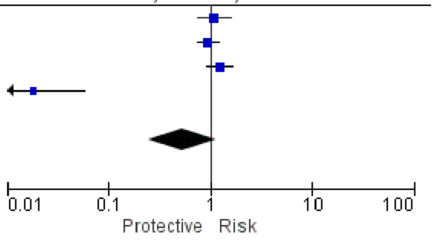

Supplementary Figure S2d: Forest plot - *ACE* I/D investigation, individuals with chronic kidney disease compared to healthy controls in a South Asian population (*ACE* insertion compared to deletion).

Study or Subgroup	Case Events	Case Total	Control Events	Control Total	Weight	Odds Ratio M-H, Random, 95% CI
Aucella 2003	315	922	916	2614	15.6%	0.96 [0.82, 1.13]
Buraczynska 2006	688	1490	492	1040	15.6%	0.96 [0.82, 1.12]
Dixit 2002	32	52	17	44	2.4%	2.54 [1.11, 5.80]
Losito 2002	135	320	128	338	9.7%	1.20 [0.88, 1.64]
McLaughlin 1996	654	1644	353	742	14.9%	0.73 [0.61, 0.87]
Nicod 2002	282	520	261	520	12.1%	1.18 [0.92, 1.50]
Ortiz 2003	68	234	80	258	7.7%	0.91 [0.62, 1.34]
Schmidt 1996	105	212	80	190	7.5%	1.35 [0.91, 2.00]
van der Sman-de Beer 2005	400	830	459	944	14.4%	0.98 [0.82, 1.18]
**Total (95% CI)**		**6224**		**6690**	**100.0%**	**1.01 [0.88, 1.16]**
Total events	2679		2786			

Heterogeneity: Tau² = 0.02; Chi² = 22.57, df = 8 (P = 0.004); I² = 65%
Test for overall effect: Z = 0.17 (P = 0.86)

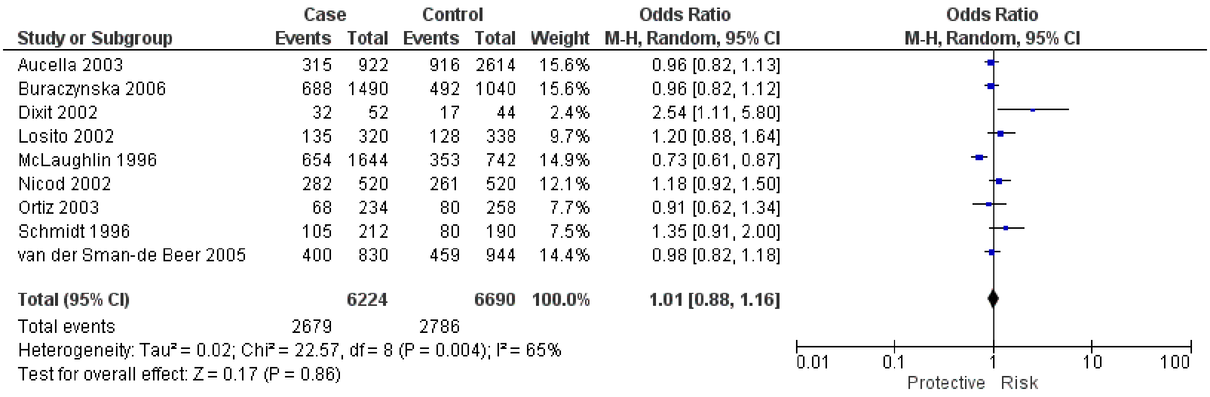

Supplementary Figure S2e: Forest plot - *ACE* I/D investigation, individuals with end-stage renal disease compared to healthy controls in a European population (*ACE* insertion compared to deletion).

Study or Subgroup	Protective Events	Protective Total	Risk Events	Risk Total	Weight	Odds Ratio M-H, Random, 95% CI
Abuaisha 2018	37	172	50	200	34.8%	0.82 [0.51, 1.33]
Rahimi 2017	64	190	89	234	50.9%	0.83 [0.55, 1.23]
Zaare Nahandi 2017	23	60	21	54	14.3%	0.98 [0.46, 2.08]
**Total (95% CI)**		**422**		**488**	**100.0%**	**0.85 [0.64, 1.13]**
Total events	124		160			

Heterogeneity: Tau² = 0.00; Chi² = 0.16, df = 2 (P = 0.92); I² = 0%
Test for overall effect: Z = 1.15 (P = 0.25)

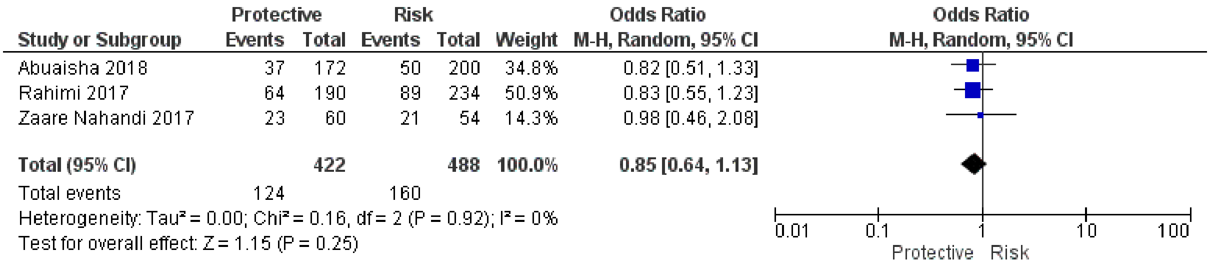

Supplementary Figure S2f: Forest plot - *ACE* I/D investigation, individuals with end-stage renal disease compared to healthy controls in a Middle Eastern population (*ACE* insertion compared to deletion).

Study or Subgroup	Case Events	Case Total	Control Events	Control Total	Weight	Odds Ratio M-H, Random, 95% CI
Doi 1996	115	200	168	248	20.6%	0.64 [0.44, 0.95]
Jayapalan 2010	163	254	93	162	20.1%	1.33 [0.89, 1.99]
Lu 2016	238	420	307	444	24.0%	0.58 [0.44, 0.77]
Park 2005	103	206	111	176	19.9%	0.59 [0.39, 0.88]
Taniwaki 2001	53	84	88	138	15.5%	0.97 [0.55, 1.71]
**Total (95% CI)**		**1164**		**1168**	**100.0%**	**0.76 [0.55, 1.05]**
Total events	672		767			

Heterogeneity: $Tau^2 = 0.09$; $Chi^2 = 13.42$, df = 4 (P = 0.009); $I^2 = 70\%$
Test for overall effect: Z = 1.66 (P = 0.10)

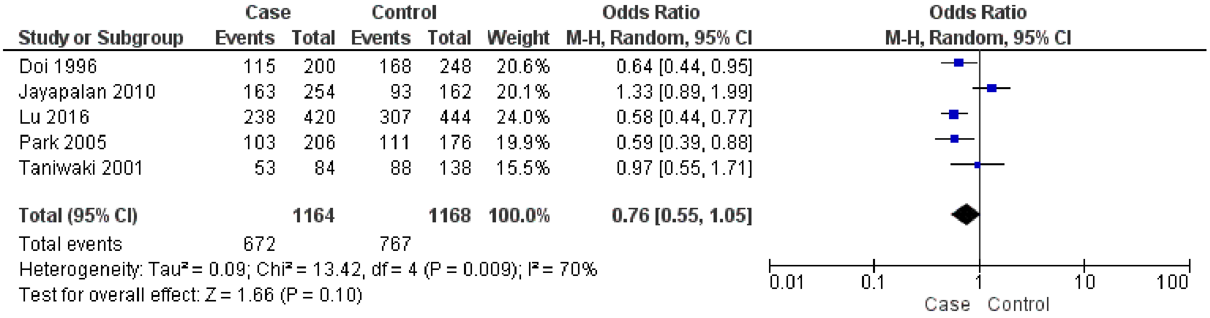

Supplementary Figure S2g: Forest plot - *ACE* I/D investigation, individuals with end-stage renal disease linked to type 2 diabetic nephropathy compared to individuals with type 2 diabetes mellitus in an East Asian population (*ACE* insertion compared to deletion).

Study or Subgroup	Case Events	Case Total	Control Events	Control Total	Weight	Odds Ratio M-H, Random, 95% CI
Grzeszczak 1998	128	254	244	508	33.9%	1.10 [0.81, 1.49]
Ringel 1997	150	322	141	280	33.7%	0.86 [0.62, 1.18]
Schmidt 1997 A	29	122	416	694	32.4%	0.21 [0.13, 0.32]
**Total (95% CI)**		**698**		**1482**	**100.0%**	**0.59 [0.24, 1.44]**
Total events	307		801			

Heterogeneity: $Tau^2 = 0.58$; $Chi^2 = 38.91$, df = 2 (P < 0.00001); $I^2 = 95\%$
Test for overall effect: Z = 1.16 (P = 0.25)

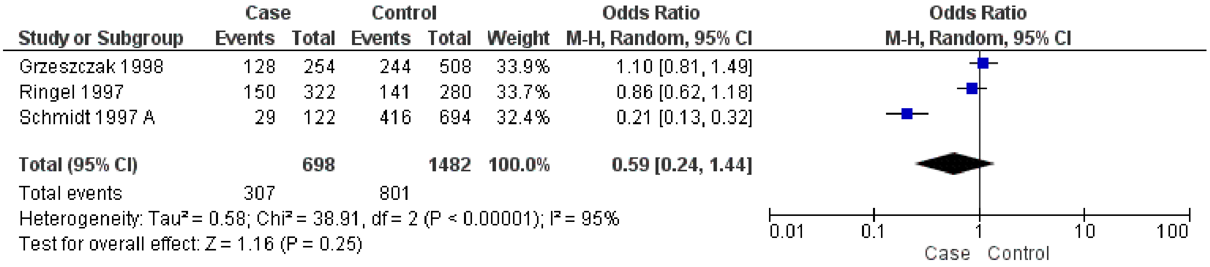

Supplementary Figure S2h: Forest plot - *ACE* I/D investigation, individuals with end-stage renal disease linked to type 2 diabetic nephropathy compared to individuals with type 2 diabetes mellitus in a European population (*ACE* insertion compared to deletion).

Study or Subgroup	Case Events	Case Total	Control Events	Control Total	Weight	Odds Ratio M-H, Random, 95% CI
Guo 2016	54	90	42	90	6.5%	1.71 [0.95, 3.10]
Huang 2010	155	260	168	240	12.5%	0.63 [0.44, 0.92]
Jung 2010	298	522	360	600	19.2%	0.89 [0.70, 1.13]
Lau 2004	154	236	137	188	10.7%	0.70 [0.46, 1.06]
Suzuki 2004	378	638	328	540	19.5%	0.94 [0.74, 1.19]
Yoon 2002	204	382	268	466	17.2%	0.85 [0.64, 1.11]
Yorioka 1995	67	96	139	206	7.8%	1.11 [0.66, 1.88]
Yoshida 1995	57	106	62	92	6.7%	0.56 [0.32, 1.00]
**Total (95% CI)**		**2330**		**2422**	**100.0%**	**0.86 [0.72, 1.02]**
Total events	1367		1504			

Heterogeneity: Tau² = 0.02; Chi² = 12.42, df = 7 (P = 0.09); I² = 44%

Test for overall effect: Z = 1.79 (P = 0.07)

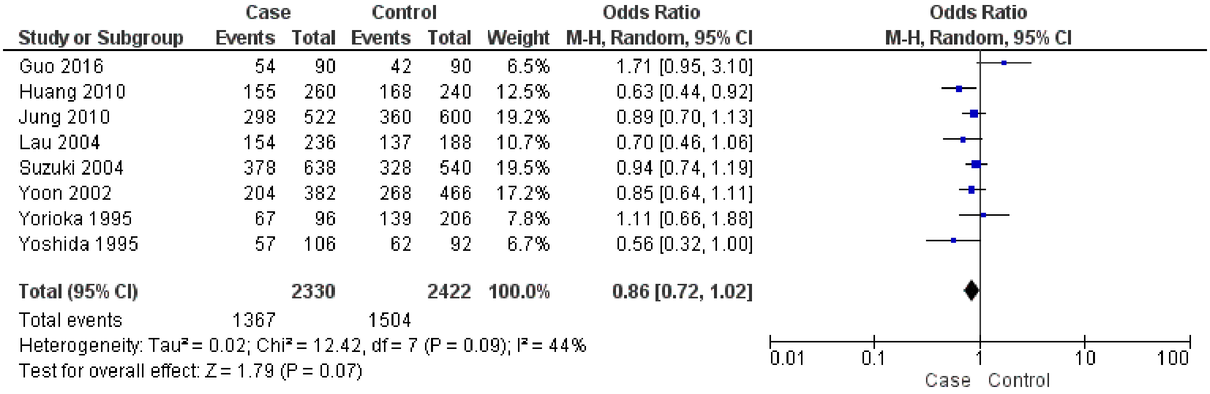

Supplementary Figure S2i: Forest plot - *ACE* I/D investigation, individuals with IgA nephropathy compared to healthy controls in an East Asian population (*ACE* insertion compared to deletion).

Study or Subgroup	Case Events	Case Total	Control Events	Control Total	Weight	Odds Ratio M-H, Random, 95% CI
Burg 1997	26	54	60	120	5.6%	0.93 [0.49, 1.77]
Drouet 2002	78	250	54	154	12.8%	0.84 [0.55, 1.29]
Harden 1995	79	200	76	196	14.3%	1.03 [0.69, 1.54]
Pawlik 2014	25	62	180	374	7.8%	0.73 [0.42, 1.26]
Pei 1997	145	336	91	200	18.7%	0.91 [0.64, 1.29]
Schmidt 1995 A	168	408	197	468	32.0%	0.96 [0.74, 1.26]
Stratta 1999	62	162	38	100	8.8%	1.01 [0.61, 1.69]
**Total (95% CI)**		**1472**		**1612**	**100.0%**	**0.93 [0.80, 1.08]**
Total events	583		696			

Heterogeneity: Tau² = 0.00; Chi² = 1.42, df = 6 (P = 0.96); I² = 0%

Test for overall effect: Z = 0.97 (P = 0.33)

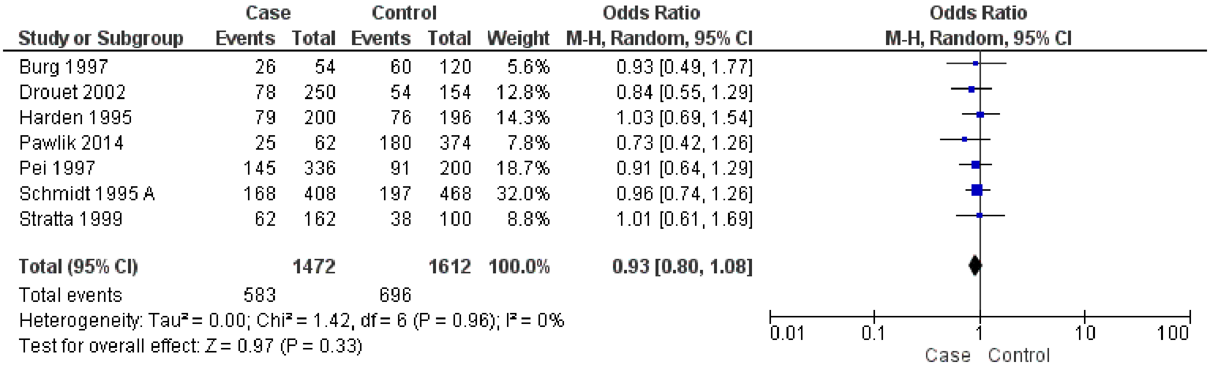

Supplementary Figure S2j: Forest plot - *ACE* I/D investigation, individuals with IgA nephropathy compared to healthy controls in a European population (*ACE* insertion compared to deletion).

Study or Subgroup	Case Events	Case Total	Control Events	Control Total	Weight	Odds Ratio M-H, Random, 95% CI
Burg 1997	44	92	60	120	12.2%	0.92 [0.53, 1.58]
Pawlik 2014	101	218	180	374	32.1%	0.93 [0.67, 1.30]
Stratta 2004	96	234	134	342	31.2%	1.08 [0.77, 1.52]
Zsom 2011	62	146	198	400	24.6%	0.75 [0.51, 1.10]
**Total (95% CI)**		**690**		**1236**	**100.0%**	**0.92 [0.76, 1.12]**
Total events	303		572			

Heterogeneity: Tau² = 0.00; Chi² = 1.91, df = 3 (P = 0.59); I² = 0%
Test for overall effect: Z = 0.82 (P = 0.41)

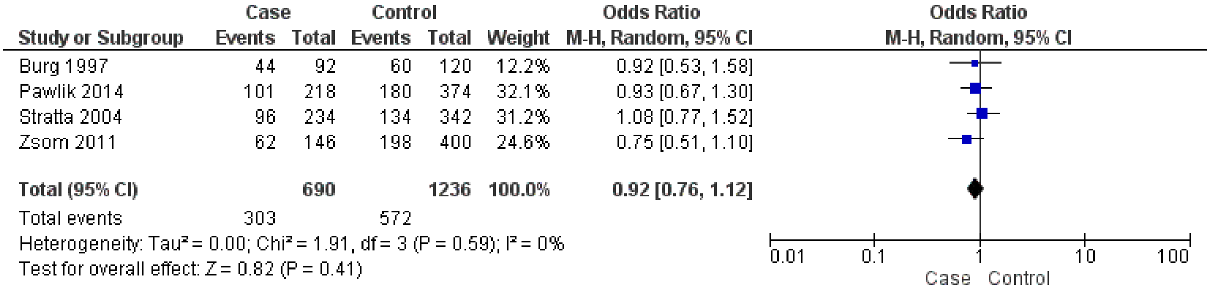

Supplementary Figure S2k: Forest plot - *ACE* I/D investigation, individuals with primary glomerulonephritis compared to healthy controls in a European population (*ACE* insertion compared to deletion).

Study or Subgroup	Case Events	Case Total	Control Events	Control Total	Weight	Odds Ratio M-H, Random, 95% CI
Beige 1997	251	538	244	538	27.2%	1.05 [0.83, 1.34]
El-Essawy 2002	246	588	170	362	24.7%	0.81 [0.62, 1.06]
Hueso 2004	122	360	96	226	18.2%	0.69 [0.49, 0.98]
Stratta 2008	122	338	112	338	20.0%	1.14 [0.83, 1.57]
Viklický 2001	28	60	599	1306	9.9%	1.03 [0.61, 1.73]
**Total (95% CI)**		**1884**		**2770**	**100.0%**	**0.93 [0.77, 1.11]**
Total events	769		1221			

Heterogeneity: Tau² = 0.02; Chi² = 6.59, df = 4 (P = 0.16); I² = 39%
Test for overall effect: Z = 0.80 (P = 0.42)

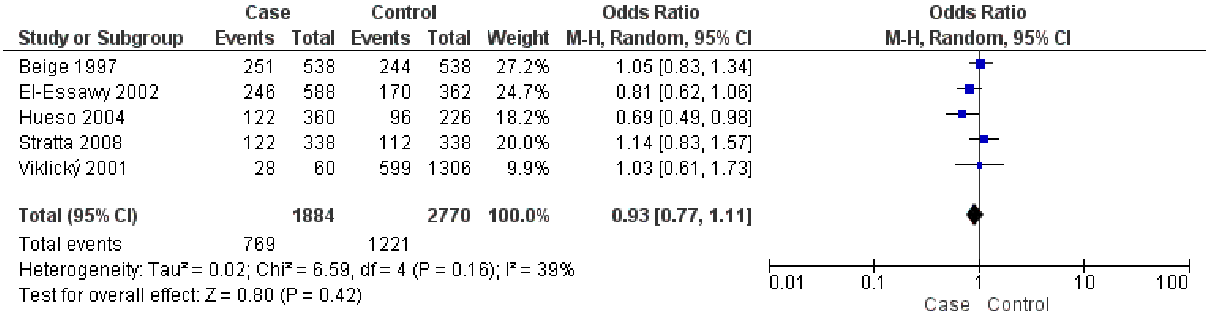

Supplementary Figure S2l: Forest plot - *ACE* I/D investigation, individuals who have had renal transplants compared to healthy controls in a European population (*ACE* insertion compared to deletion).

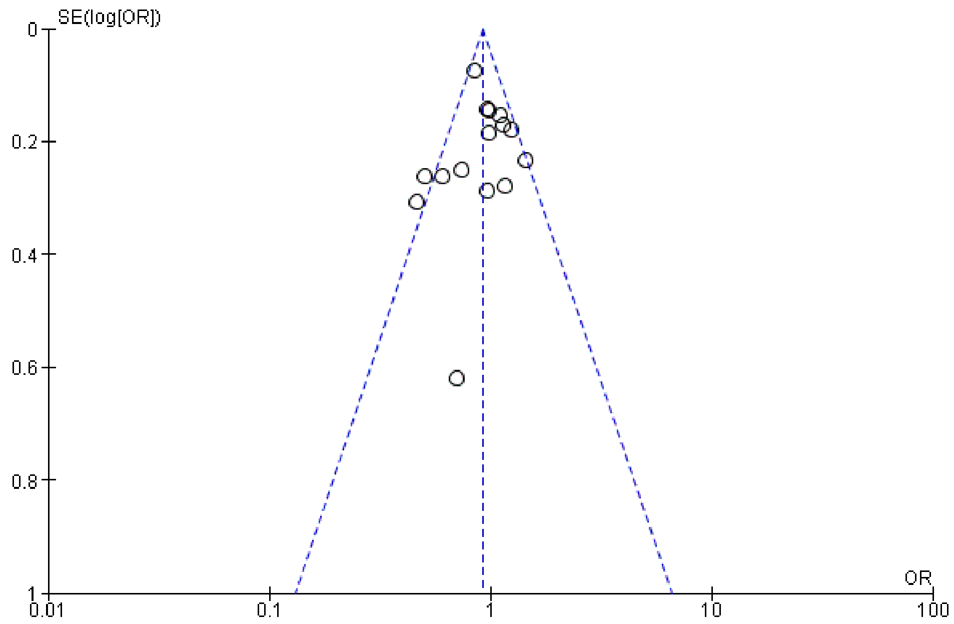

Supplementary Figure S3a: Funnel plot - *ACE* I/D investigation, individuals with type 1 diabetic nephropathy compared to individuals with type 1 diabetes mellitus in a European population (*ACE* insertion compared to deletion).

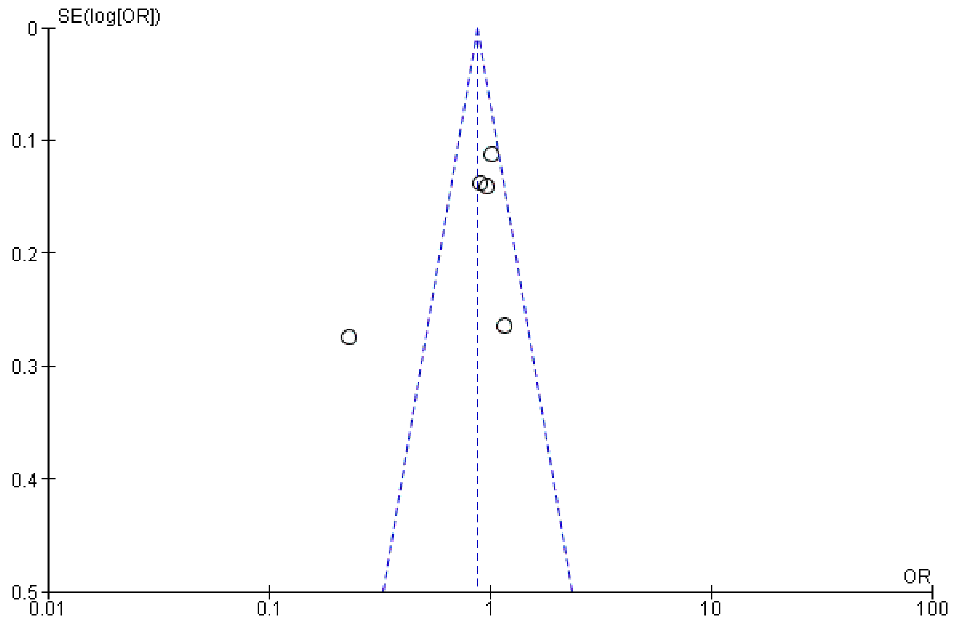

Supplementary Figure S3b: Funnel plot - *ACE* I/D investigation, individuals with type 2 diabetic nephropathy compared to individuals with type 2 diabetes mellitus in a European population (*ACE* insertion compared to deletion).

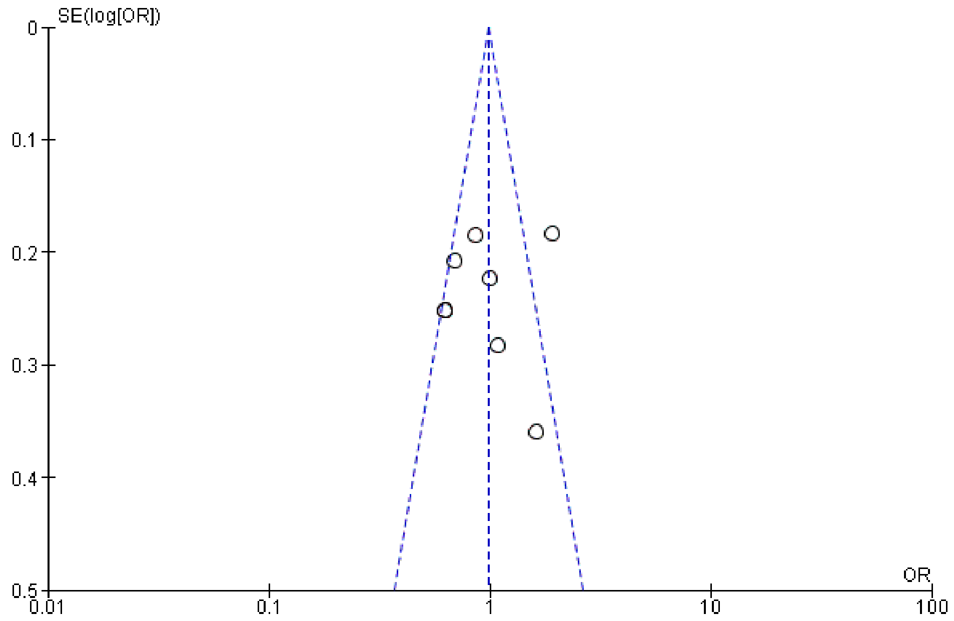

Supplementary Figure S3c: Funnel plot - *ACE* I/D investigation, individuals with type 2 diabetic nephropathy compared to individuals with type 2 diabetes mellitus in a Middle Eastern population (*ACE* insertion compared to deletion).

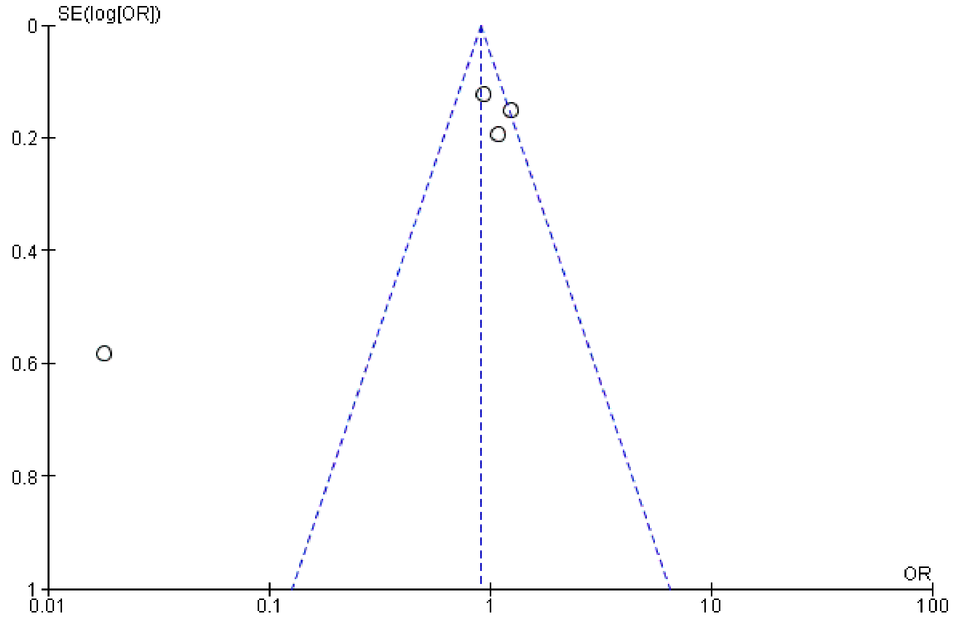

Supplementary Figure S3d: Funnel plot - *ACE* I/D investigation, individuals with chronic kidney disease compared to healthy controls in a South Asian population (*ACE* insertion compared to deletion).

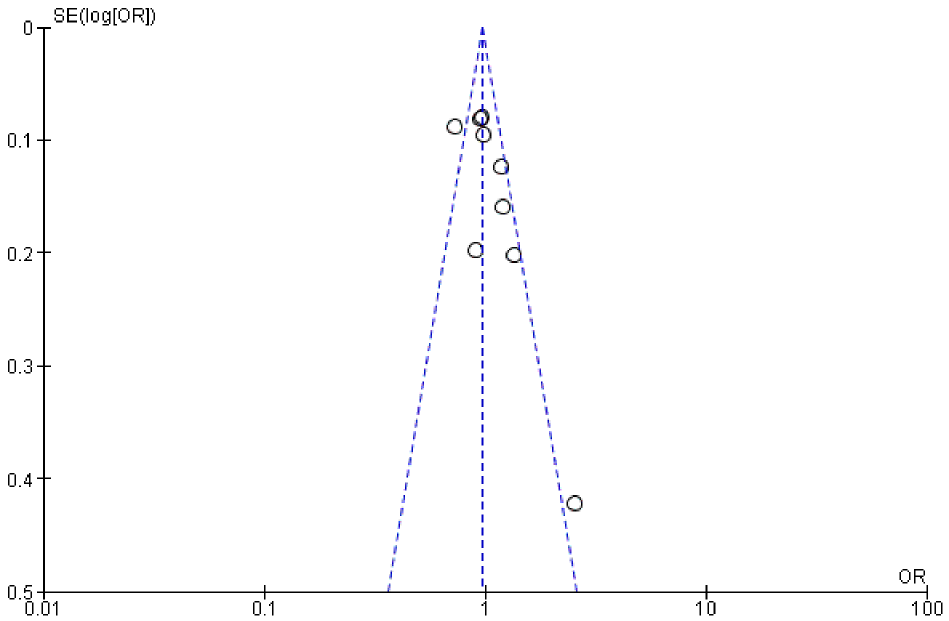

Supplementary Figure S3e: Funnel plot - *ACE* I/D investigation, individuals with end-stage renal disease compared to healthy controls in a European population (*ACE* insertion compared to deletion).

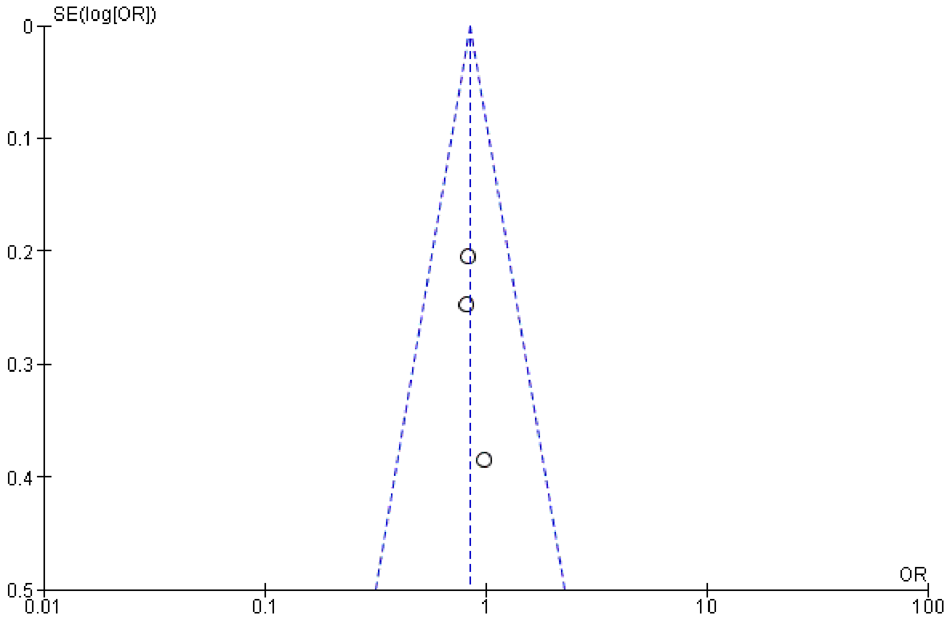

Supplementary Figure S3f: Funnel plot - *ACE* I/D investigation, individuals with end-stage renal disease compared to healthy controls in a Middle Eastern population (*ACE* insertion compared to deletion).

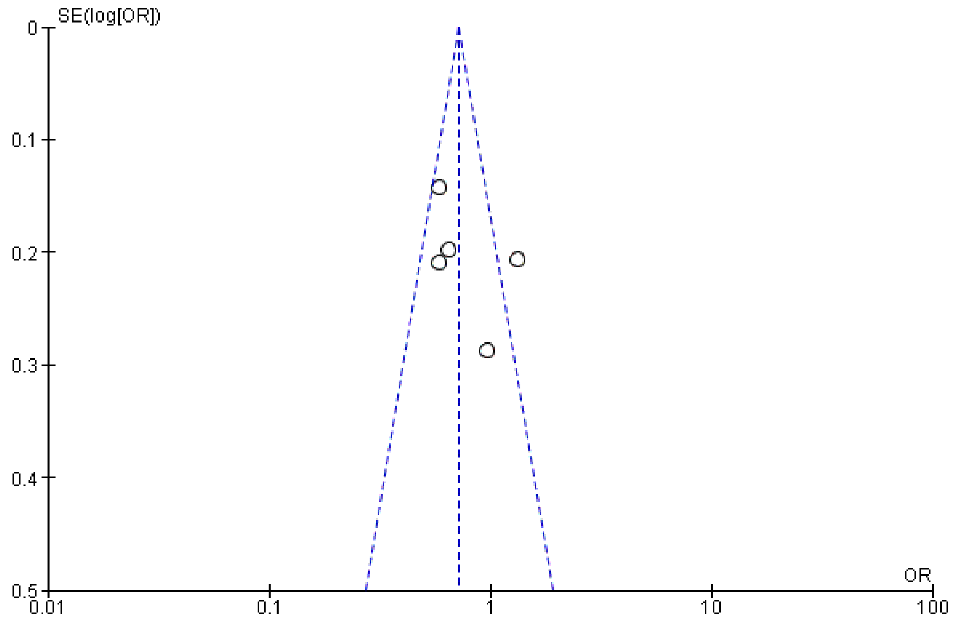

Supplementary Figure S3g: Funnel plot - *ACE* I/D investigation, individuals with end-stage renal disease linked to type 2 diabetic nephropathy compared to individuals with type 2 diabetes mellitus in an East Asian population (*ACE* insertion compared to deletion).

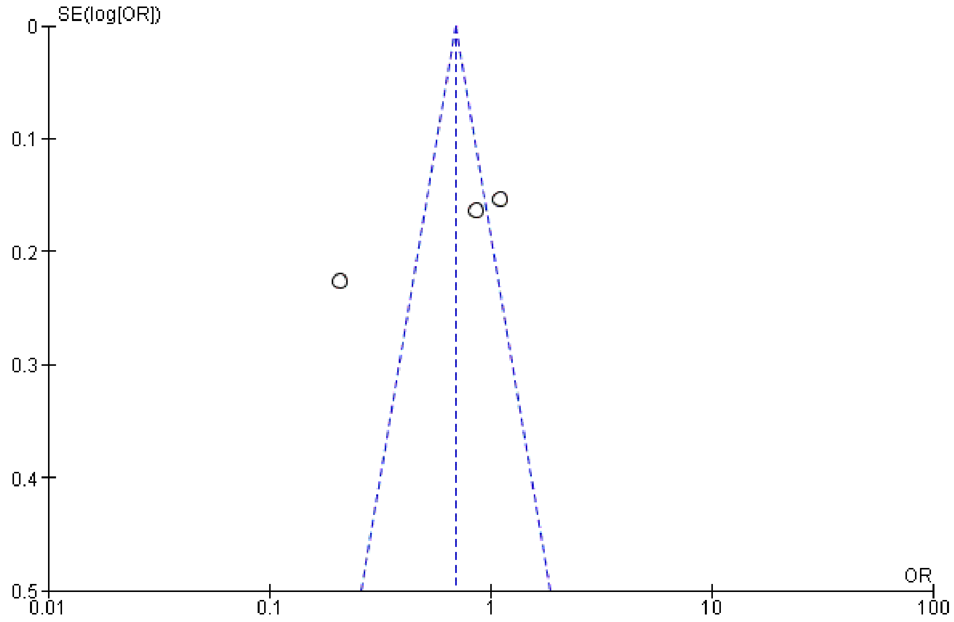

Supplementary Figure S3h: Funnel plot - *ACE* I/D investigation, individuals with end-stage renal disease linked to type 2 diabetic nephropathy compared to individuals with type 2 diabetes mellitus in a European population (*ACE* insertion compared to deletion).

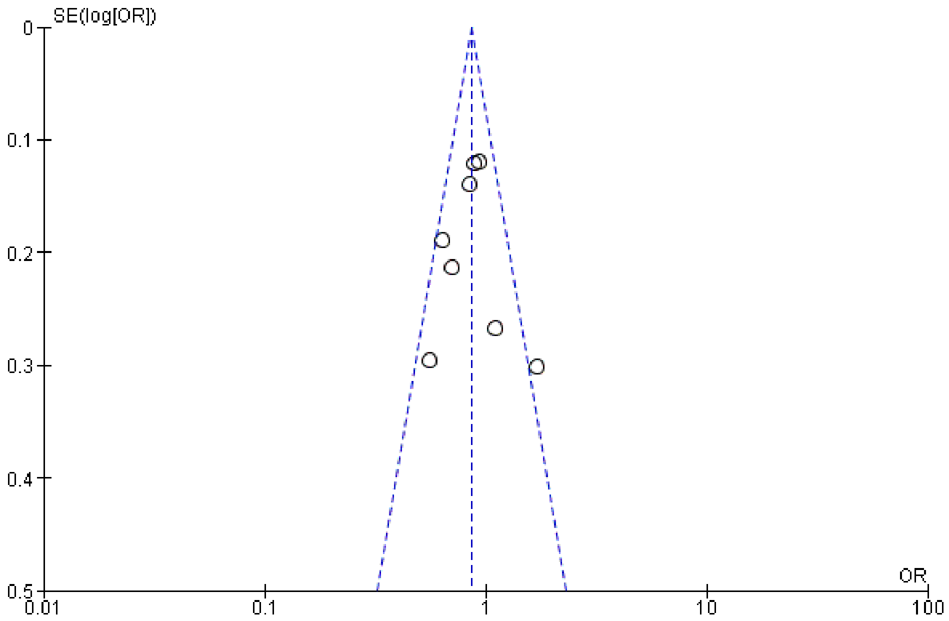

Supplementary Figure S3i: Funnel plot - *ACE* I/D investigation, individuals with IgA nephropathy compared to healthy controls in an East Asian population (*ACE* insertion compared to deletion).

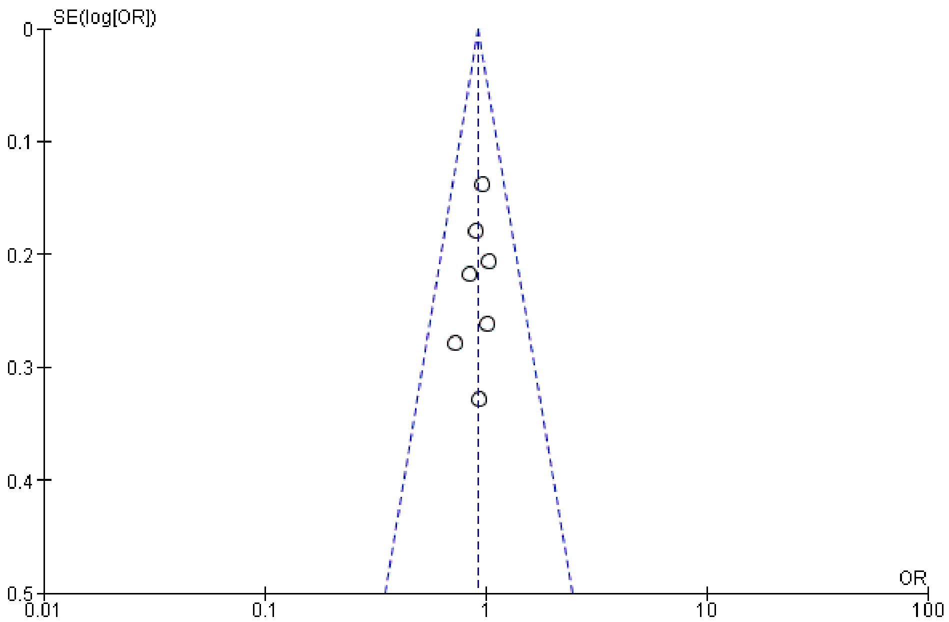

Supplementary Figure S3j: Funnel plot - *ACE* I/D investigation, individuals with IgA nephropathy compared to healthy controls in a European population (*ACE* insertion compared to deletion).

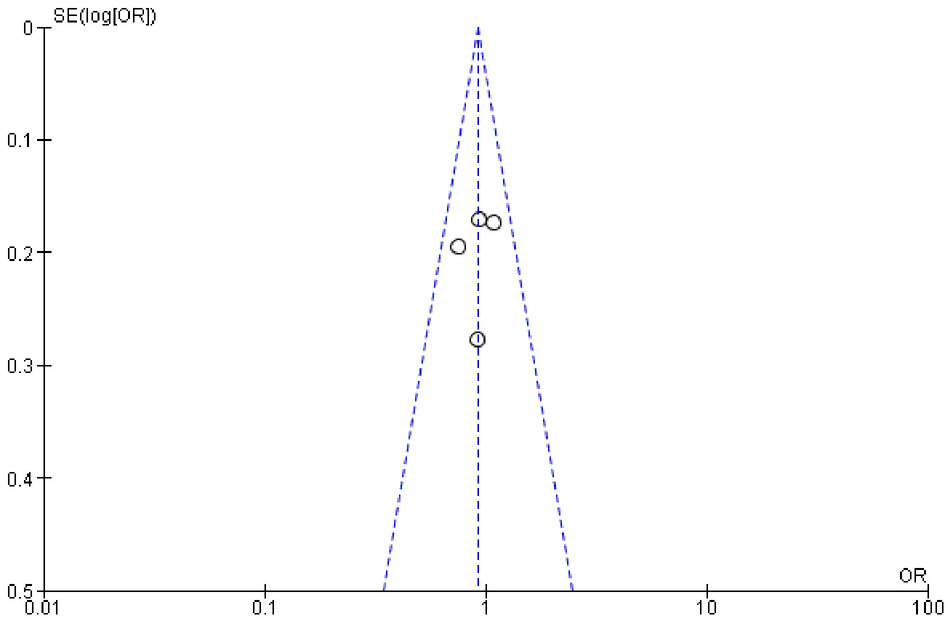

Supplementary Figure S3k: Funnel plot - *ACE* I/D investigation, individuals with primary glomerulonephritis compared to healthy controls in a European population (*ACE* insertion compared to deletion).

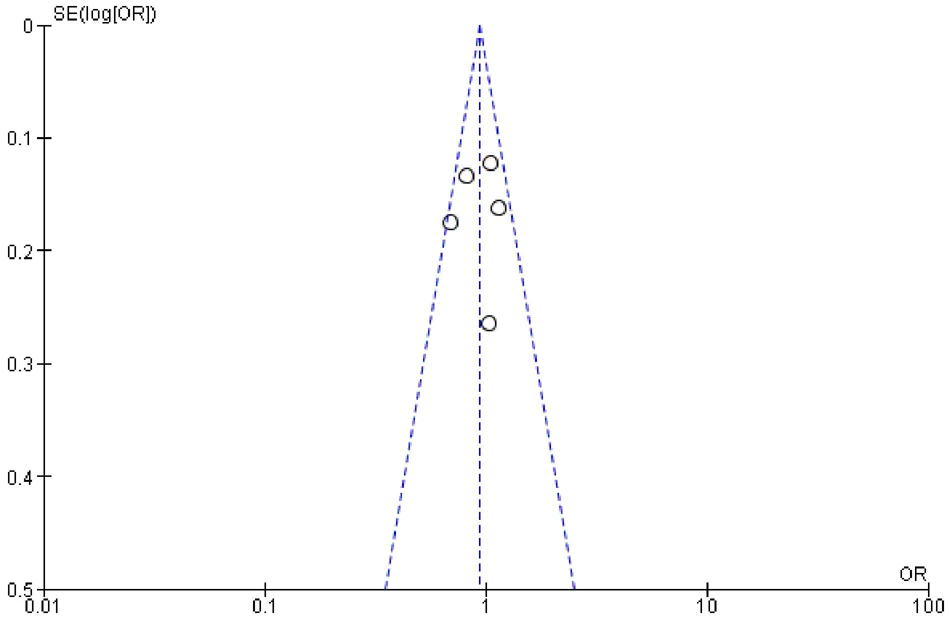

Supplementary Figure S3l: Funnel plot - *ACE* I/D investigation, individuals who have had renal transplants compared to healthy controls in a European population (*ACE* insertion compared to deletion).

Study or Subgroup	Case Events	Case Total	Control Events	Control Total	Weight	Odds Ratio M-H, Random, 95% CI	Odds Ratio M-H, Random, 95% CI
Doria 1996	158	278	96	150	15.1%	0.74 [0.49, 1.12]	
Fogarty 1996	114	190	129	200	15.1%	0.83 [0.55, 1.24]	
Möllsten 2008	62	96	220	394	13.2%	1.44 [0.91, 2.29]	
Ringel 1997	145	268	257	452	19.5%	0.89 [0.66, 1.21]	
Schmidt 1996	121	216	132	240	16.6%	1.04 [0.72, 1.51]	
van Ittersum 2000	31	60	238	376	10.8%	0.62 [0.36, 1.07]	
Walder 1998	60	110	63	88	9.6%	0.48 [0.26, 0.86]	
**Total (95% CI)**		**1218**		**1900**	**100.0%**	**0.85 [0.68, 1.06]**	
Total events	691		1135				
Heterogeneity: Tau² = 0.04; Chi² = 11.57, df = 6 (P = 0.07); I² = 48%							
Test for overall effect: Z = 1.43 (P = 0.15)							

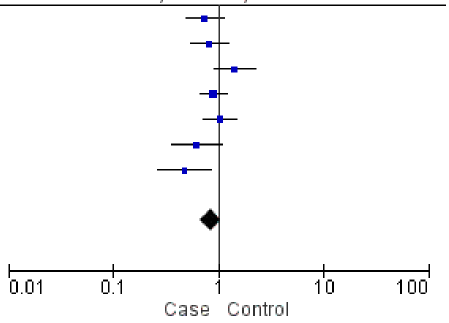

Supplementary Figure S4a: Forest plot - *AGT* rs699 investigation, individuals with type 1 diabetic nephropathy compared to individuals with type 1 diabetes mellitus in a European population (*AGT* rs699 T allele compared to C allele).

Study or Subgroup	Case Events	Case Total	Control Events	Control Total	Weight	Odds Ratio M-H, Random, 95% CI	Odds Ratio M-H, Random, 95% CI
Ohno 1996	16	50	25	106	16.8%	1.52 [0.72, 3.21]	
Oue 1999	6	42	13	60	11.8%	0.60 [0.21, 1.74]	
Thomas 2001	18	102	79	510	20.4%	1.17 [0.67, 2.05]	
Wang 2016	19	194	154	946	21.6%	0.56 [0.34, 0.92]	
Wu 2000	21	142	26	82	18.5%	0.37 [0.19, 0.72]	
Young 1998	4	40	25	108	11.0%	0.37 [0.12, 1.14]	
**Total (95% CI)**		**570**		**1812**	**100.0%**	**0.69 [0.43, 1.11]**	
Total events	84		322				
Heterogeneity: Tau² = 0.21; Chi² = 13.02, df = 5 (P = 0.02); I² = 62%							
Test for overall effect: Z = 1.54 (P = 0.12)							

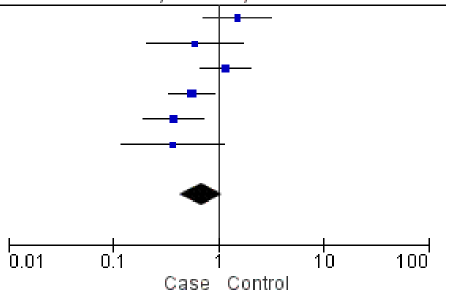

Supplementary Figure S4b: Forest plot - *AGT* rs699 investigation, individuals with type 2 diabetic nephropathy compared to individuals with type 2 diabetes mellitus in an East Asian population (*AGT* rs699 T allele compared to C allele).

Study or Subgroup	Case Events	Case Total	Control Events	Control Total	Weight	Odds Ratio M-H, Random, 95% CI	Odds Ratio M-H, Random, 95% CI
Fradin 2002	45	78	139	236	7.5%	0.95 [0.57, 1.60]	
Freire 1998	134	230	148	236	14.5%	0.83 [0.57, 1.20]	
Makuc 2017	294	552	394	750	41.4%	1.03 [0.83, 1.28]	
Schmidt 1996	149	254	117	214	14.9%	1.18 [0.81, 1.70]	
Zychma 2000	133	254	242	486	21.8%	1.11 [0.82, 1.50]	
**Total (95% CI)**		**1368**		**1922**	**100.0%**	**1.03 [0.89, 1.18]**	
Total events	755		1040				
Heterogeneity: Tau² = 0.00; Chi² = 2.11, df = 4 (P = 0.72); I² = 0%							
Test for overall effect: Z = 0.39 (P = 0.70)							

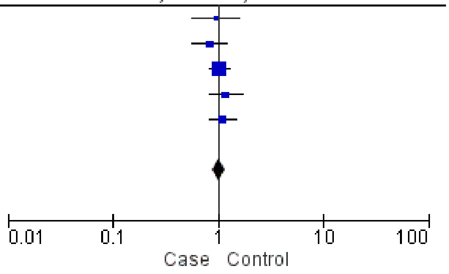

Supplementary Figure S4c: Forest plot - *AGT* rs699 investigation, individuals with type 2 diabetic nephropathy compared to individuals with type 2 diabetes mellitus in a European population (*AGT* rs699 T allele compared to C allele).

Study or Subgroup	Case Events	Case Total	Control Events	Control Total	Weight	Odds Ratio M-H, Random, 95% CI
Eroglu 2008	48	92	62	112	25.1%	0.88 [0.51, 1.53]
Reis 2011	114	216	89	222	36.0%	1.67 [1.14, 2.44]
Sancakdar 2015	189	388	95	200	38.8%	1.05 [0.75, 1.48]
**Total (95% CI)**		**696**		**534**	**100.0%**	**1.19 [0.82, 1.71]**
Total events	351		246			

Heterogeneity: Tau² = 0.06; Chi² = 4.72, df = 2 (P = 0.09); I² = 58%
Test for overall effect: Z = 0.91 (P = 0.36)

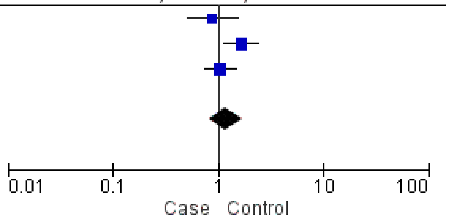

Supplementary Figure S4d: Forest plot - *AGT* rs699 investigation, individuals with type 2 diabetic nephropathy compared to individuals with type 2 diabetes mellitus in a Middle Eastern population (*AGT* rs699 T allele compared to C allele).

Study or Subgroup	Protective Events	Protective Total	Risk Events	Risk Total	Weight	Odds Ratio M-H, Random, 95% CI
Kawada 1997	91	424	64	404	31.1%	1.45 [1.02, 2.07]
Su 2017	194	1268	279	1478	38.3%	0.78 [0.63, 0.95]
Wang 2003	76	492	64	366	30.6%	0.86 [0.60, 1.24]
**Total (95% CI)**		**2184**		**2248**	**100.0%**	**0.97 [0.67, 1.41]**
Total events	361		407			

Heterogeneity: Tau² = 0.08; Chi² = 9.16, df = 2 (P = 0.01); I² = 78%
Test for overall effect: Z = 0.14 (P = 0.89)

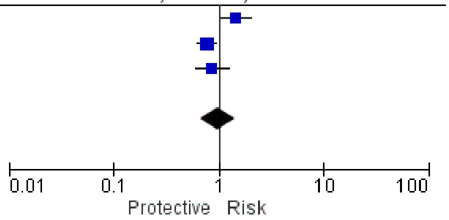

Supplementary Figure S4e: Forest plot - *AGT* rs699 investigation, individuals with end-stage renal disease compared to healthy controls in an East Asian population (*AGT* rs699 T allele compared to C allele).

Study or Subgroup	Case Events	Case Total	Control Events	Control Total	Weight	Odds Ratio M-H, Random, 95% CI
Gao 2015	144	700	125	620	41.2%	1.03 [0.78, 1.34]
Guo 2016	19	90	16	90	5.4%	1.24 [0.59, 2.60]
Huang 2010	35	260	40	240	12.3%	0.78 [0.48, 1.27]
Kim 2009	89	476	115	600	31.5%	0.97 [0.71, 1.32]
Lau 2004	27	236	31	188	9.6%	0.65 [0.38, 1.14]
**Total (95% CI)**		**1762**		**1738**	**100.0%**	**0.94 [0.79, 1.12]**
Total events	314		327			

Heterogeneity: Tau² = 0.00; Chi² = 3.18, df = 4 (P = 0.53); I² = 0%
Test for overall effect: Z = 0.67 (P = 0.50)

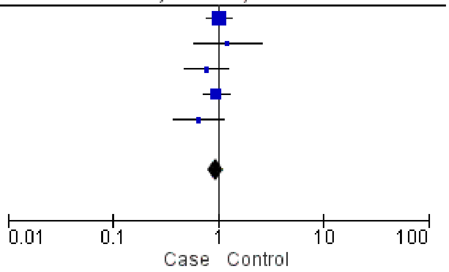

Supplementary Figure S4f: Forest plot - *AGT* rs699 investigation, individuals with IgA nephropathy compared to healthy controls in an East Asian population (*AGT* rs699 T allele compared to C allele).

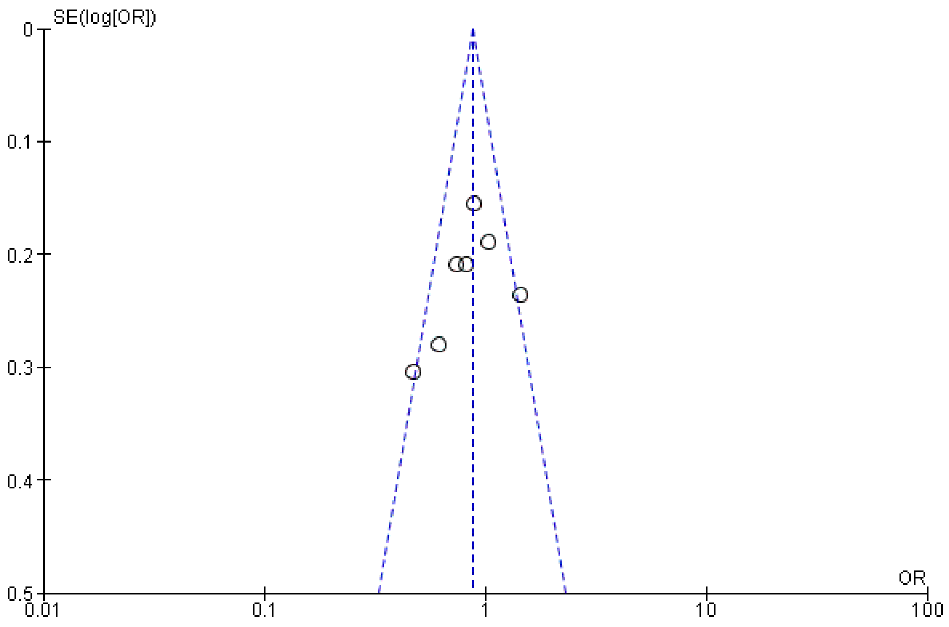

Supplementary Figure S5a: Funnel plot - *AGT* rs699 investigation, individuals with type 1 diabetic nephropathy compared to individuals with type 1 diabetes mellitus in a European population (*AGT* rs699 T allele compared to C allele).

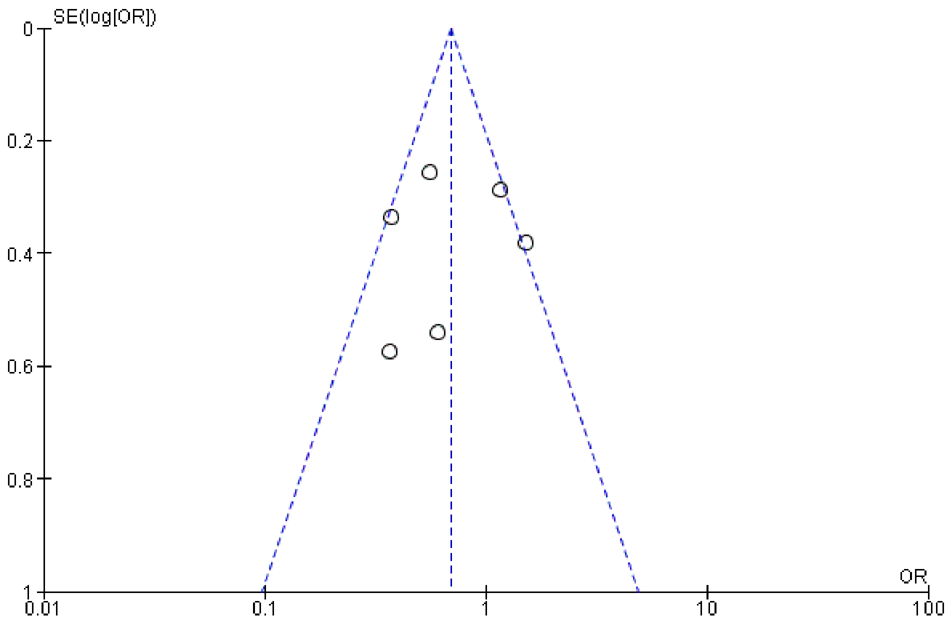

Supplementary Figure S5b: Funnel plot - *AGT* rs699 investigation, individuals with type 2 diabetic nephropathy compared to individuals with type 2 diabetes mellitus in an East Asian population (*AGT* rs699 T allele compared to C allele).

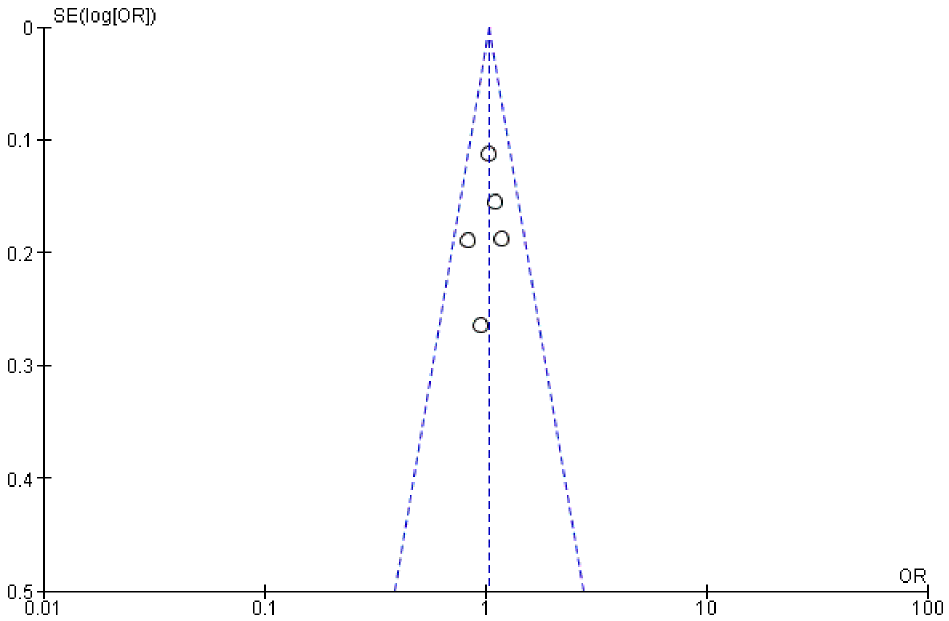

Supplementary Figure S5c: Funnel plot - *AGT* rs699 investigation, individuals with type 2 diabetic nephropathy compared to individuals with type 2 diabetes mellitus in a European population (*AGT* rs699 T allele compared to C allele).

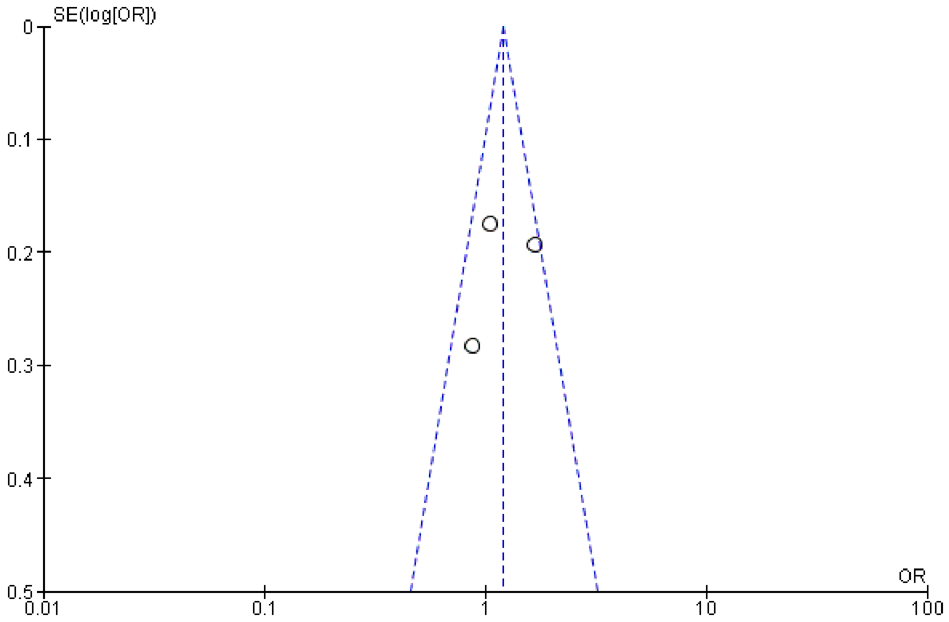

Supplementary Figure S5d: Funnel plot - *AGT* rs699 investigation, individuals with type 2 diabetic nephropathy compared to individuals with type 2 diabetes mellitus in a Middle Eastern population (*AGT* rs699 T allele compared to C allele).

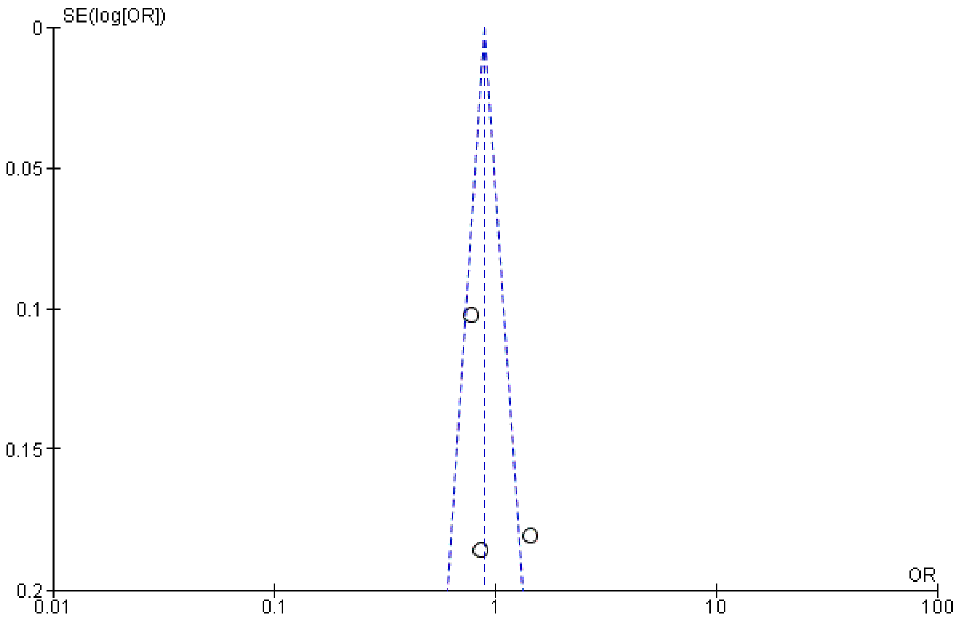

Supplementary Figure S5e: Funnel plot - *AGT* rs699 investigation, individuals with end-stage renal disease compared to healthy controls in an East Asian population (*AGT* rs699 T allele compared to C allele).

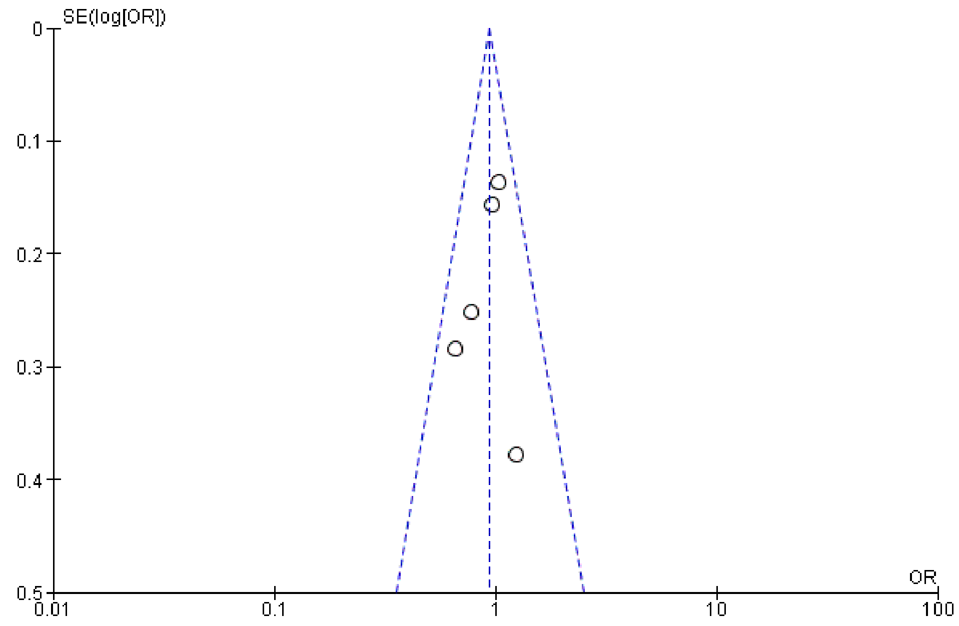

Supplementary Figure S5f: Funnel plot - *AGT* rs699 investigation, individuals with IgA nephropathy compared to healthy controls in an East Asian population (*AGT* rs699 T allele compared to C allele).

Study or Subgroup	Case Events	Case Total	Control Events	Control Total	Weight	Odds Ratio M-H, Random, 95% CI
Currie 2010	1029	1414	1052	1470	33.2%	1.06 [0.90, 1.25]
Doria 1997	99	146	119	158	12.2%	0.69 [0.42, 1.14]
Möllsten 2008	78	96	290	394	10.4%	1.55 [0.89, 2.72]
Savage 1999	136	190	137	194	14.5%	1.05 [0.67, 1.63]
Tarnow 1996	287	396	274	380	21.4%	1.02 [0.74, 1.40]
van Ittersum 2000	47	60	238	376	8.3%	2.10 [1.10, 4.01]
**Total (95% CI)**		**2302**		**2972**	**100.0%**	**1.10 [0.89, 1.35]**
Total events	1676		2110			

Heterogeneity: Tau² = 0.03; Chi² = 8.89, df = 5 (P = 0.11); I² = 44%
Test for overall effect: Z = 0.87 (P = 0.38)

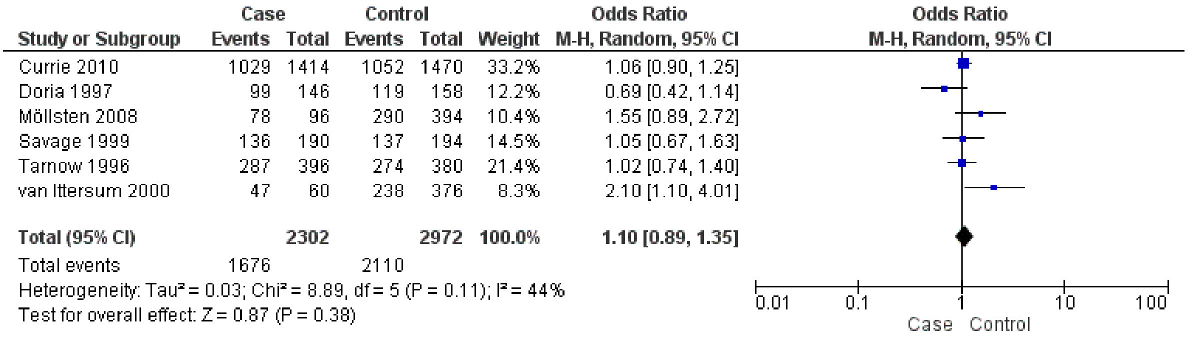

Supplementary Figure S6a: Forest plot - *AGTR1* rs5186 investigation, individuals with type 1 diabetic nephropathy compared to individuals with type 1 diabetes mellitus in a European population (*AGTR1* rs5186 A allele compared to C allele).

Study or Subgroup	Case Events	Case Total	Control Events	Control Total	Weight	Odds Ratio M-H, Random, 95% CI
Thomas 2001	99	102	483	510	30.2%	1.84 [0.55, 6.20]
Wu 2000	127	142	74	82	54.2%	0.92 [0.37, 2.26]
Young 1998	38	40	103	108	15.7%	0.92 [0.17, 4.96]
**Total (95% CI)**		**284**		**700**	**100.0%**	**1.13 [0.58, 2.20]**
Total events	264		660			

Heterogeneity: Tau² = 0.00; Chi² = 0.90, df = 2 (P = 0.64); I² = 0%
Test for overall effect: Z = 0.37 (P = 0.71)

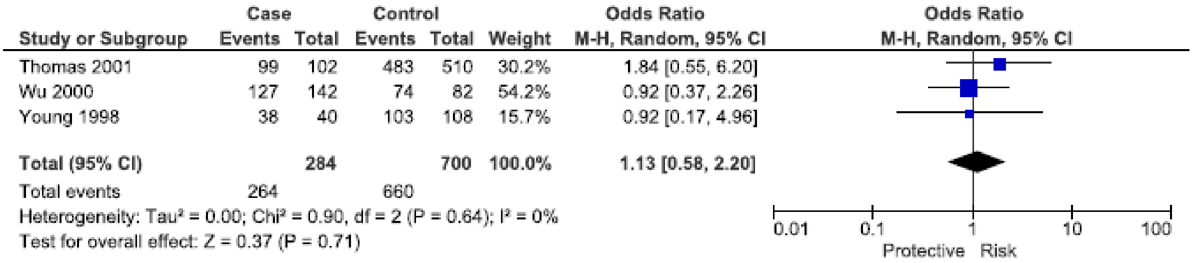

Supplementary Figure S6b: Forest plot - *AGTR1* rs5186 investigation, individuals with type 2 diabetic nephropathy compared to individuals with type 2 diabetes mellitus in an East Asian population (*AGTR1* rs5186 A allele compared to C allele).

Study or Subgroup	Case Events	Case Total	Control Events	Control Total	Weight	Odds Ratio M-H, Random, 95% CI
Gao 2015	659	702	577	620	45.1%	1.14 [0.74, 1.77]
Huang 2010	243	260	220	240	19.1%	1.30 [0.66, 2.54]
Kim 2009	452	476	576	600	25.7%	0.78 [0.44, 1.40]
Lau 2004	227	236	178	188	10.1%	1.42 [0.56, 3.56]
**Total (95% CI)**		**1674**		**1648**	**100.0%**	**1.09 [0.81, 1.46]**
Total events	1581		1551			

Heterogeneity: Tau² = 0.00; Chi² = 1.85, df = 3 (P = 0.60); I² = 0%
Test for overall effect: Z = 0.55 (P = 0.58)

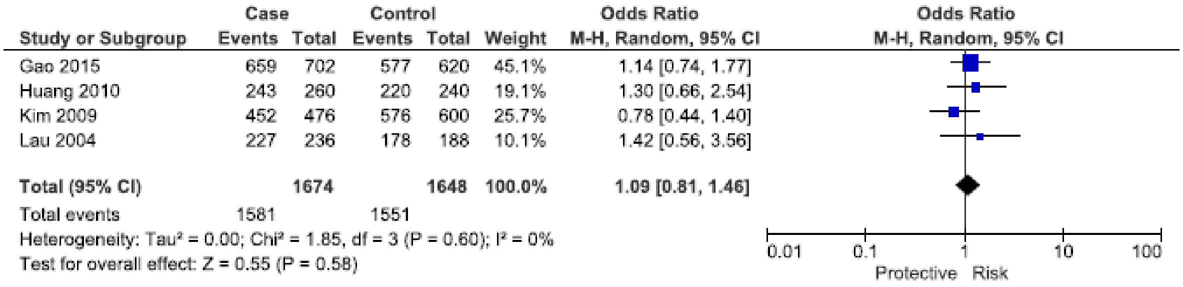

Supplementary Figure S6c: Forest plot - *AGTR1* rs5186 investigation, individuals with IgA nephropathy compared to healthy controls in an East Asian population (*AGTR1* rs5186 A allele compared to C allele).

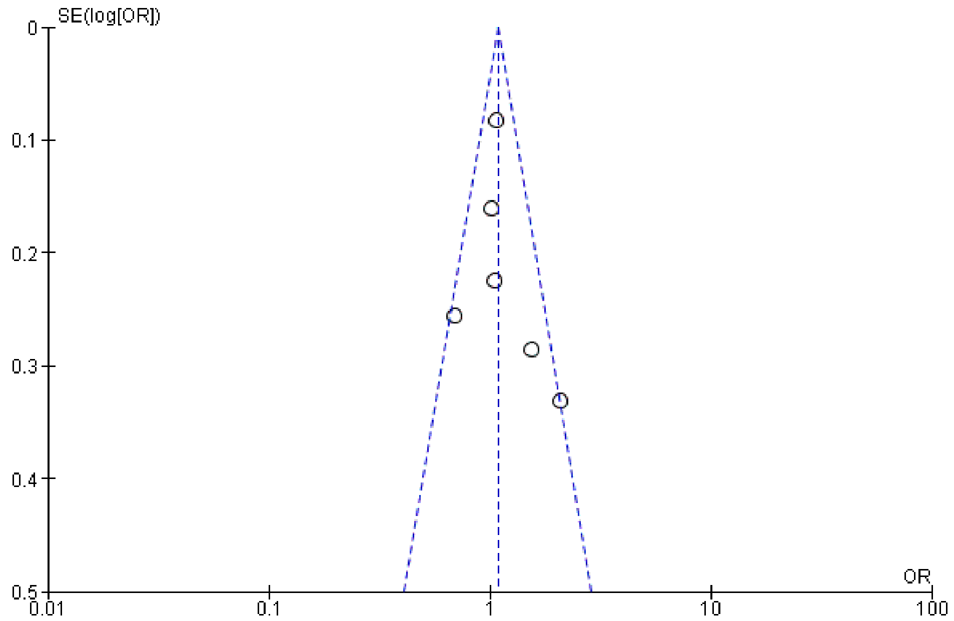

Supplementary Figure S7a: Funnel plot - *AGTR1* rs5186 investigation, individuals with type 1 diabetic nephropathy compared to individuals with type 1 diabetes mellitus in a European population (*AGTR1* rs5186 A allele compared to C allele).

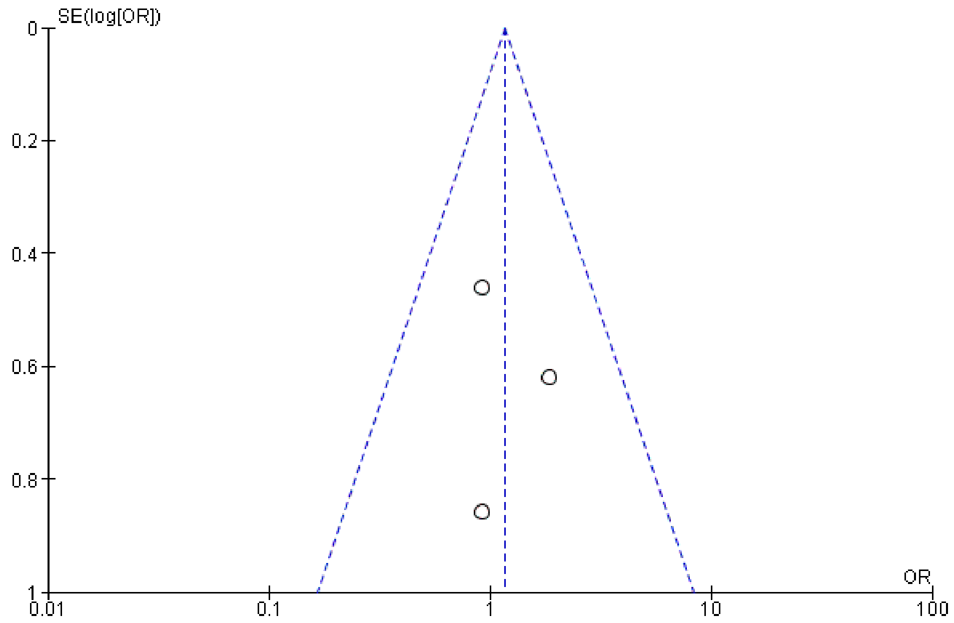

Supplementary Figure S7b: Funnel plot - *AGTR1* rs5186 investigation, individuals with type 2 diabetic nephropathy compared to individuals with type 2 diabetes mellitus in an East Asian population (*AGTR1* rs5186 A allele compared to C allele).

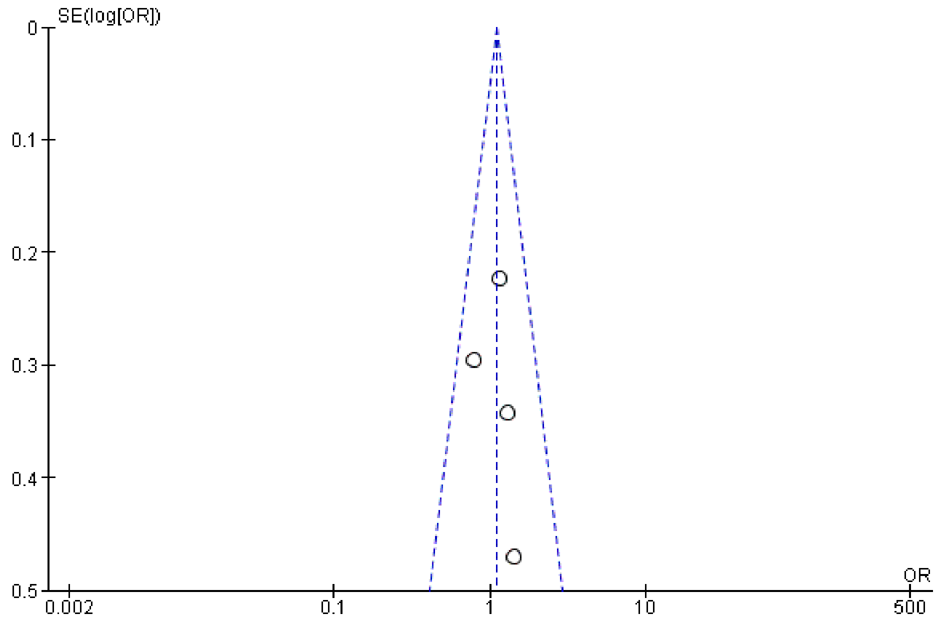

Supplementary Figure S7c: Funnel plot - *AGTR1* rs5186 investigation, individuals with IgA nephropathy compared to healthy controls in an East Asian population (*AGTR1* rs5186 A allele compared to C allele).
