# Supplementary material for: Genetic associations between genes in the renin-angiotensin-aldosterone system and renal disease: a systematic review and meta-analysis
Source: BMJ Open. 2019 May 1;9(4):e026777. doi: 10.1136/bmjopen-2018-026777 (PMC6501980; doi:10.1136/bmjopen-2018-026777)
Supplement: Supplementary data [file bmjopen-2018-026777supp003.pdf]

Supplementary Data File S1. Allele frequencies for all control populations which consisted of healthy individuals alongside the dbSNP reported frequencies

***AGT* rs699 – East Asian populations included in meta-analysis**

- IgAN vs healthy controls
  - Five populations included in analysis
  - Allele frequencies are as follows:

AGT	Study	Population	Sample Size	Reference allele frequency	Alternative allele frequency
**rs699**	Gao J et al.(49)	East Asian	538	A=0.202	G=0.798
**rs699**	Guo Y et al.(77)	East Asian	1265	A=0.178	G=0.822
**rs699**	Huang HD et al.(50)	East Asian	329	A=0.167	G=0.833
**rs699**	Kim SM et al.(51)	East Asian	587	A=0.192	G=0.808
**rs699**	Lau YK et al.(25)	East Asian	212	A=0.165	G=0.835

- ESRD vs healthy controls
  - Three populations included in analysis
  - Allele frequencies:

AGT	Study	Population	Sample Size	Reference allele frequency	Alternative allele frequency
**rs699**	Kawada N et al.(22)	East Asian	424	A=0.158	G=0.841
**rs699**	Su SL et al.(157)	East Asian	1373	A=0.189	G=0.811
**rs699**	Wang AY et al.(63)	East Asian	429	A=0.175	G=0.825

- dbSNP frequencies:

AGT	Study	Population	Sample Size	Reference allele frequency	Alternative allele frequency
**rs699**	gnomAD - Genomes	East Asian	1622	A=0.156	G=0.844
**rs699**	1000Genomes	East Asian	1008	A=0.147	G=0.853

- Chi-square calculations for reference allele frequency comparing meta-analysed data with dbSNP data:

Group	gnomAD - Genomes	1000Genomes
***AGT* rs699 – East Asian populations IgAN vs Healthy Controls**	0.999516	0.999582
***AGT* rs699 – East Asian populations ESRD vs Healthy Controls**	0.984359	0.985654

***AGT* rs699 – European populations included in meta-analysis**

- ESRD vs healthy controls
  - Four populations included in analysis
  - Allele frequencies are as follows:

AGT	**Study**	**Population**	**Sample Size**	**Reference allele frequency**	**Alternative allele frequency**
**rs699**	Beige J et al. (146)	European	538	A=0.573	G=0.427
**rs699**	Buraczynska M et al.(65)	European	1265	A=0.542	G=0.458
**rs699**	Losito A et al.(67)	European	329	A=0.562	G=0.438
**rs699**	Lovati E et al.(147)	European	587	A=0.601	G=0.399

- dbSNP frequencies:

AGT	**Study**	**Population**	**Sample Size**	**Reference allele frequency**	**Alternative allele frequency**
**rs699**	gnomAD - Genomes	European	18440	A=0.5616	G=0.4384
**rs699**	gnomAD - Exomes	European	133914	A=0.57665	G=0.42335
**rs699**	1000Genomes	European	1006	A=0.588	G=0.412
**rs699**	ExAC	European	73258	A=0.5747	G=0.4253

- Chi-square calculations for alternative allele frequency comparing meta-analysed data with dbSNP data:

**Group**	**gnomAD - Genomes**	**gnomAD - Exomes**	**1000Genomes**	**ExAC**
***AGT* rs699 – European populations ESRD vs Healthy Controls**	0.979214	0.980521	0.981483	0.980354

***AGTR1* rs5186 – East Asian populations included in meta-analysis**

- IgAN vs healthy controls
  - Four populations included in analysis
  - Allele frequencies are as follows:

AGTR1	**Study**	**Population**	**Sample Size**	**Reference allele frequency**	**Alternative allele frequency**
**rs5186**	Gao J et al.(49)	East Asian	661	A=0.931	G=0.069
**rs5186**	Huang HD et al.(50)	East Asian	250	A=0.917	G=0.083
**rs5186**	Kim SM et al.(51)	East Asian	538	A=0.960	G=0.04
**rs5186**	Lau YK et al.(25)	East Asian	212	A=0.947	G=0.053

- dbSNP frequencies:

AGTR1	**Study**	**Population**	**Sample Size**	**Reference allele frequency**	**Alternative allele frequency**
**rs5186**	gnomAD - Genomes	East Asian	1618	A=0.944	C=0.056
**rs5186**	1000Genomes	East Asian	1008	A=0.940	C=0.060

- Chi-square calculations for alternative allele frequency comparing meta-analysed data with dbSNP data:

**Group**	**gnomAD - Genomes**	**1000Genomes**
***AGTR1* rs5186 – East Asian populations IgAN vs Healthy Controls**	0.999615	0.999559

ACE insertion/deletion – European, East Asian, South Asian and Middle Eastern populations included in meta-analysis

- Four reported rs numbers for this SNP rs numbers (rs13447447, rs1799752, rs4340, rs4646994) were not found in dbSNP and therefore no reported allele frequencies were available for comparison.
