# Supplementary material for: Genetic associations between genes in the renin-angiotensin-aldosterone system and renal disease: a systematic review and meta-analysis
Source: BMJ Open. 2019 May 1;9(4):e026777. doi: 10.1136/bmjopen-2018-026777 (PMC6501980; doi:10.1136/bmjopen-2018-026777)
Supplement: Author's manuscript [file bmjopen-2018-026777.draft_revisions.pdf]

BMJ Open is committed to open peer review. As part of this commitment we make the peer review history of every article we publish publicly available.

When an article is published we post the peer reviewers' comments and the authors' responses online. We also post the versions of the paper that were used during peer review. These are the versions that the peer review comments apply to.

The versions of the paper that follow are the versions that were submitted during the peer review process. They are not the versions of record or the final published versions. They should not be cited or distributed as the published version of this manuscript.

BMJ Open is an open access journal and the full, final, typeset and author-corrected version of record of the manuscript is available on our site with no access controls, subscription charges or pay-per-view fees (http://bmjopen.bmj.com).

If you have any questions on BMJ Open's open peer review process please email info.bmjopen@bmj.com

BMJ Open

Genetic associations between genes in the renin-angiotensin-aldosterone system and renal disease: a systematic review and meta-analysis

Journal:	*BMJ Open*
Manuscript ID	bmjopen-2018-026777
Article Type:	Research
Date Submitted by the Author:	19-Sep-2018
Complete List of Authors:	Smyth, Laura; Queen's University Belfast Centre for Public Health, Epidemiology and Public Health Research Group<br>Cañadas-Garre, Marisa; Queen's University Belfast Centre for Public Health, Epidemiology and Public Health Research Group<br>Cappa, Ruaidhri; Queen's University Belfast Centre for Public Health, Epidemiology and Public Health Research Group<br>Maxwell, Alexander; Queen's University Belfast Centre for Public Health, Centre for Public Health; Belfast City Hospital, Regional Nephrology Unit<br>McKnight, Amy; Queen's University Belfast, Centre for Public Health
Keywords:	CKD, NEPHROLOGY, RAAS, Meta-analysis, Renin-angiotensin-aldosterone system, Kidney

SCHOLARONE™
Manuscripts

For peer review only

Genetic associations between genes in the renin-angiotensin-aldosterone system and renal disease: a systematic review and meta-analysis

Laura J. Smyth[1*], Marisa Cañadas-Garre[1], Ruaidhri C. Cappa[1], Alexander P. Maxwell[1,2], Amy Jayne McKnight[1]

[1.] Epidemiology and Public Health Research Group, Centre for Public Health, Queen's University Belfast, Belfast, United Kingdom

[2.] Regional Nephrology Unit, Belfast City Hospital, Belfast, United Kingdom

*Corresponding author email: lsmyth42@qub.ac.uk

SHORT TITLE

A meta-analysis of RAAS gene variants and CKD

WORD COUNT (excluding Abstract, Key words, References, Figures, Tables, Statements and Contributions)

3,016

ABSTRACT

Background

Chronic kidney disease (CKD) is defined by abnormalities in kidney structure and/or function present for more than three months. Worldwide, both the incidence and prevalence rates of CKD are increasing. The renin-angiotensin-aldosterone system (RAAS) regulates fluid and electrolyte balance through the kidney. RAAS activation is associated with hypertension, which is directly implicated in causation and progression of CKD. RAAS blockade, using drugs targeting individual RAAS mediators and receptors, has proven to be reno-protective.

Objectives

To assess genomic variants present within RAAS genes; *ACE*, *ACE2*, *AGT*, *AGTR1*, *AGTR2* and *REN*, for their association with CKD.

Design and Data Sources

A systematic review and meta-analysis of observational research was performed to evaluate the RAAS gene polymorphisms in CKD using both PubMed and Web of Science databases. Eligible articles included case-control studies of a defined kidney disease and included genotype counts.

Eligibility Criteria

Any paper was removed from the analysis if it was not written in English or Spanish, was a non-human study, was a paediatric study, was not a case-control study, did not have a renal disease phenotype, did not include data for the gene of interest, was a gene expression based study or had a pharmaceutical drug focus.

For peer review only - http://bmjopen.bmj.com/site/about/guidelines.xhtml

Results

A total of 3,286 studies were identified, 108 of which met the inclusion criteria. Genetic variants which had been reported in at least three independent publications for populations with the same ethnicity were then determined and quantitative analyses subsequently performed.

Three variants returned significant results in populations with different ethnicities at P <0.05; *ACE* insertion, *AGT* rs699-T allele, and *AGTR1* rs5186-A allele; all had small effect sizes and each variant was associated with a reduced risk of CKD development.

Conclusions

Further biological pathway and functional analyses of the RAAS gene polymorphisms will help define how variation in components of the RAAS pathway contribute to CKD.

Key words

CKD, kidney, meta-analysis, RAAS, renin-angiotensin-aldosterone system

ARTICLE SUMMARY

Strengths and limitations of the study

- Strength: Individuals with microalbuminuria were excluded from both the case and control definitions to improve the discrimination between individuals with and without renal disease as microalbuminuria may regress, remain stable or progress to macroalbuminuria.
- Strength: Due to previously reported heterogeneity between different ethnic groups, we included this as a risk factor and performed each analysis per ethnicity.
- Limitation: Some studies in our search could not be included in quantitative analysis as they lacked information relating to genotype counts and had an unclear measure and definition of albuminuria for both cases and controls.

Hypothesis statement

- We hypothesise that there will be an association between genetic variants within RAAS genes and CKD.

INTRODUCTION

Chronic kidney disease (CKD) is defined as a progressive loss of renal function measured as a reduction in glomerular filtration rate (GFR) to <60 mL/min/1.73m^2, or the presence of persistent urinary abnormalities including albuminuria and/or structural alterations which have been present for at least three months(1). CKD is an increasing public health issue given its associated morbidities, premature mortality and management of advanced CKD is a significant burden on health care budgets worldwide(2,3). There is substantial evidence that inherited genetic variants(4), the presence of diabetes(5) or hypertension(6) and an individual's ethnicity(7,8) directly influence the development of various CKD phenotypes.

The renin-angiotensin-aldosterone system (RAAS) is a homeostatic endocrine system which is of critical importance to the regulation of blood pressure and maintenance of fluid and electrolyte balance(9,10). Renin, secreted from the juxtaglomerular apparatus in response to reduced renal

For peer review only - http://bmjopen.bmj.com/site/about/guidelines.xhtml

perfusion pressure, catalyses the conversion of angiotensinogen to angiotensin I(11). Subsequently, angiotensin I converting enzyme (*ACE*) cleaves angiotensin I to generate angiotensin II, which regulates heart and kidney function by binding to and activating angiotensin II receptors (type I and type II)(11,12). The angiotensin II type I receptors are responsible for multiple biologic actions of RAAS including vasoconstriction and sodium reabsorption(11–14).

Increased RAAS activation is linked to progression of CKD of different aetiologies, especially diabetic nephropathy (DN)(11,15–18), and is mediated by hypertensive injury(15,17) and accelerated renal fibrosis(19). The physiological relevance of this pathway in the progression of CKD has focused attention on RAAS components including *ACE*, angiotensin I converting enzyme 2 (*ACE2*), angiotensinogen (*AGT*), angiotensin II receptor type 1 (*AGTR1*), angiotensin II receptor type 2 (*AGTR2*) and renin (*REN*), as candidate genes for various CKD-related phenotypes. Multiple studies have implicated RAAS gene variants in the progression of CKD(20–26).

This manuscript describes a systematic review and meta-analysis to examine published data reporting genetic variants present within six of the RAAS candidate genes: *ACE*, *ACE2*, *AGT*, *AGTR1*, *AGTR2* and *REN*, for a range of CKD phenotypes and ethnicities, to help define their putative roles as risk factors for CKD.

METHODS

Search Strategy: A systematic search was undertaken following recognised methods, the Meta-analysis of Observational Studies in Epidemiology (MOOSE) guidelines(27), by two investigators. PubMed and Web of Science online databases were searched for studies between the inception of each database and 31st December 2016. All search terms are detailed in Supplementary Table S1. Reference lists from included publications were also manually searched. Two authors (LJS and MCG) independently conducted the literature search, screened the articles and extracted the data. In the case of any disagreement, a third author (RCC) considered the articles. A range of CKD phenotypes were included in this analysis, the case and control definitions are included in Table 1.

Table 1: Phenotypic comparisons included in this analysis

Case Group	Control Group
Autosomal dominant polycystic kidney disease	Healthy controls
Atherosclerotic renal artery stenosis	Healthy controls
Balkan endemic nephropathy	Healthy controls
Chronic glomerulonephritis	Healthy controls
Chronic kidney disease	Healthy controls
Diabetic nephropathy*	Diabetes mellitus
Diabetic nephropathy*	Healthy controls
End-stage renal disease	Healthy controls
End-stage renal disease	Type 1 diabetes mellitus
Focal segmental glomerulosclerosis	Healthy controls
Glomerulonephritis	Healthy controls
Hypertension-related renal disease	Healthy controls with hypertension
Hypertension-related renal disease	Healthy controls
IgA Nephropathy	Healthy controls
Interstitial nephritis	Healthy controls
Lupus nephritis	Systemic lupus erythematosus

For peer review only - http://bmjopen.bmj.com/site/about/guidelines.xhtml

Lupus nephritis	Healthy controls
Minimal change nephrotic syndrome	Healthy controls
Non-Balkan endemic nephropathy	Healthy controls
Nephroangiosclerosis	Healthy controls
Polycystic kidney disease	Healthy controls
Primary membranous glomerulonephritis	Healthy controls
Primary membranous glomerulonephritis	Organ donors
Renal transplant recipients	Healthy controls
Renal transplant recipients	Kidney Donors
Type 1 diabetic nephropathy*	Type 1 diabetes mellitus
Type 1 diabetic nephropathy linked to end-stage renal disease	Healthy controls
Type 1 diabetic nephropathy linked to end-stage renal disease	Type 1 diabetes mellitus
Type 2 diabetic nephropathy*	Type 2 diabetes mellitus
Type 2 diabetic nephropathy linked to end-stage renal disease	Healthy controls
Type 2 diabetic nephropathy linked to end-stage renal disease	Type 2 diabetes mellitus

* In the studies including diabetic nephropathy as cases, only individuals with reported macroalbuminuria or proteinuria were included. Individuals with microalbuminuria were excluded.

Inclusion/Exclusion Criteria: Inclusion criteria were judged against a standardised list of agreed criteria (LJS, MCG and AJM), whereby the English or Spanish language publication described an adult, human case-control study, of a defined kidney disease. In the rare instances when suspected duplicate data was identified within two or more included articles, only the article either published first, or that with the larger number of participants was included.

Articles were excluded if they included paediatric subjects, were a pharmacological based study reporting clinical trials of medications, did not contain genotypic data for the correct gene or were non-human studies.

Data extraction: Where available, the size of each study, case group disease definition and number of individuals, control group definition and number of individuals, ethnicity, genetic variant, genotype in the format of allele one–heterozygote–allele two and allele counts were recorded and calculated in spreadsheets by two authors (LJS and MCG). Articles were re-assessed where any disagreement occurred and a third reviewer was employed. Ethnicities were recorded from the articles and re-coded following the International Genome Sample Resources' online guidance(28). Where any population did not align to a listed population code, a new one was created for the purposes of this study. All ethnicity codes are available in Supplementary Table S2. The data collected was divided into disease phenotype groups to ensure a high level of homogeneity.

Statistical Analysis: Each genetic variant which had been investigated and reported in at least three independent publications for the same ethnicity and phenotype was included in quantitative statistical analyses. Review Manager (RevMan) 5.3 (The Cochrane Collaboration, The Nordic Cochrane Centre, Copenhagen, Denmark) was employed to facilitate the analysis of allele frequencies. For each SNP, the total number of alleles were recorded per case and control group. Hardy Weinberg was calculated for all included studies. Statistical analyses were performed using the random effects model as heterogeneity was expected. For each SNP, this analysis provided the P value, odds ratio (OR) and 95% confidence intervals (CI). It also facilitated the assessment of the heterogeneity level using the I^2 statistic(29). Forest plots and funnel plots were generated automatically to assess publication bias and the significance value was set at P<0.05 (LJS and RCC).

The quality of the studies was assessed by all available quality control measures including reported genotype completion rate, phenotype description, microalbuminuria exclusion and Hardy Weinberg

For peer review only - http://bmjopen.bmj.com/site/about/guidelines.xhtml

equilibrium. No sensitivity analysis was performed. All study methodology conformed to the MOOSE criteria(27). No published protocol is available for this review. The workflow followed a consistent pattern for each gene. A summary of this is included in Figure 1.

Figure 1: Workflow pattern

RESULTS

The database searches returned 3,286 results, 138 of which remained following the application of inclusion and exclusion criteria and the removal of any SNP which was not reported on at least three occasions. Several articles included data for more than one gene, signifying that they have emerged multiple times throughout the database searches; the total number of individual articles was therefore 108. The search strategies are included as Supplementary Figures S1a-f. All excluded studies are listed in Supplementary Tables S3a-f

The total number of subjects analysed within these studies (n=108) was 17,782 individuals with renal disease and 21,199 individuals acting as controls. For SNPs in three of the RAAS genes, *ACE2*, *AGTR2* and *REN*, there were less than three independent populations studied. A summary table detailing the main results is included as Table 2.

Table 2: Summary of most significant result for each selected gene

Gene	Articles returned (n)	Articles analysed (n)	Max. individuals in analysis (n)	Most significant result	P Value	OR (95% CL)	I^2 (%)
ACE	366	91	32,666	I/D (EAS)	0.008	0.80 (0.67, 0.94)	68
ACE2	1413	0	NA		NA	NA	NA
AGT	656	31	11,210	rs699 (EUR)	0.002	0.84 (0.76, 0.94)	0
AGTR1	191	16	6917	rs5186 (SAS)	0.001	0.71 (0.58, 0.87)	37
AGTR2	28	0	NA		NA	NA	NA
REN	632	0	NA		NA	NA	NA

Abbreviations: EAS, East Asian; EUR, European; NA, Not Applicable; SAS, South Asian.

ACE

A total of 14 quantitative analyses were completed for the insertion / deletion (I/D) polymorphism located within *ACE* in eight phenotypes, details of which are included in Supplementary Table S4. Three quantitative analyses returned a significant result. The first analysis comprised 11 publications, each studying an East Asian population with T2DN (type 2 diabetes and nephropathy) and compared with T2DM (type 2 diabetes mellitus without nephropathy). Figure 2a displays these results, P= 0.009; OR= 0.74; 95% CI= 0.59, 0.93, I^2= 55%, showing that the presence of the insertion variant at this *ACE* locus was significantly associated with this phenotype. The insertion provides a lower risk of developing T2DN in an East Asian population.

Figure 2a: *ACE* I/D investigation, individuals with type 2 diabetic nephropathy compared to individuals with type 2 diabetes mellitus in an East Asian Population (ACE insertion variant compared to deletion variant).

For peer review only

For peer review only - http://bmjopen.bmj.com/site/about/guidelines.xhtml

The *ACE* insertion variant was similarly significantly associated with a lower risk of T2DN compared with T2DM in a South Asian population, despite the presence of a high level of heterogeneity (P= 0.01; OR= 0.57; 95% CI= 0.37, 0.87; I^2= 89%). The comparison of East Asian individuals with end-stage renal disease (ESRD) compared to a healthy population with no evidence of renal disease also showed a significant association with moderate levels of heterogeneity (P= 0.008; OR= 0.8; 95% CI= 0.67, 0.94; I^2= 68%). These results are included as Figures 2b and 2c respectively.

Figure 2b: *ACE* I/D investigation, individuals with type 2 diabetic nephropathy compared to individuals with type 2 diabetes mellitus in a South Asian Population (insertion compared to deletion).

Figure 2c: *ACE* I/D investigation, individuals with end-stage renal disease compared to healthy controls in an East Asian Population (insertion compared to deletion).

In each analysis (T2DN vs. T2DM; ESRD vs. normal), the presence of the *ACE* insertion was associated with a lower risk of developing the CKD phenotype in the respective populations. The non-significant results are included in Supplementary Figures S2a-S2k.

AGT

Seven quantitative analyses were completed for rs699, where the T allele was compared to the C allele. Details of each of these comparisons are included in Supplementary Table S5. One significant result was identified - the comparison of ESRD with healthy controls in a European population. The results were (P=0.002; OR= 0.84; 95% CI= 0.76, 0.94; I^2= 0%) and are shown in Figure 2d. These results indicate that the presence of the T allele is associated with a lower risk of developing ESRD in this population. The non-significant results are included in Supplementary Figures S2l-S2q.

Figure 2d: *AGT* rs699 investigation, individuals with end-stage renal disease compared to healthy controls in a European Population (AGT rs699 T allele compared to C allele).

AGTR1

Four quantitative analyses were performed for *AGTR1* rs5186, comparing the A allele with the C. Details of these comparisons are included within Supplementary Table S6. One of the four studies returned a significant result for association, the comparison of T2DN with T2DM in a South Asian population (P= 0.001; OR= 0.71; 95% CI= 0.58, 0.87; I^2= 37%). This result is shown in Figure 2e. These results indicate that the presence of the A allele is associated with a lower risk of developing T2DN in this population. The non-significant results are included in Supplementary Figures S2r-S2t.

Figure 2e: *AGTR1* rs5186 investigation, individuals with type 2 diabetic nephropathy compared to individuals with type 2 diabetes mellitus in a South Asian Population (AGTR1 rs5186 A allele compared to C allele).

All funnel plots for these analyses are included in Supplementary Figure S3. Only the plots for *ACE* I/D CKD in a South Asian population and *ACE* I/D T2DN in a European population showed a tendency for publication bias.

Studies not complying with Hardy Weinberg equilibrium are shown in Supplementary Table S7.

For peer review only - http://bmjopen.bmj.com/site/about/guidelines.xhtml

DISCUSSION

Investigations into RAAS genetic variants previously reported to have been associated with a range of CKD phenotypes were undertaken. A total of 3,286 studies were identified, 108 of which met the inclusion criteria. Subsequently, 25 quantitative analyses were completed for three RAAS genes where there were at least three independent population studies of the RAAS gene variants. Five significant results within three genes were obtained at the significance level $p<0.05$, each revealing an association with CKD.

ACE is encoded by *DCP1* and is a key component of the RAAS. It catalyses the modification of angiotensin I to II, which is more biologically active(30). *ACE* is the most frequently studied gene of the RAAS(31). First sequenced in 1992(32), this 287 bp ALU repetitive element at intron 16(32) is located on chromosome 17 and is represented by four individual SNPs, rs4646994, rs1799752, rs4340 and rs13447447. Further understanding of its genetic architecture and disease associations may enable patient groups to benefit from targeted therapies with ACE inhibitors(33).

Since 1994, the association of *ACE* and DN has been rigorously investigated(33,34), with studies returning conflicting results. A meta-analysis undertaken in 2005 by Ng and colleagues(35) reported a statistically significant result wherein the *ACE* insertion was associated with protection from development of diabetic nephropathy in Asians and Caucasians. A second meta-analysis undertaken in 2012 also identified an association between the *ACE* I/D polymorphism and the development of diabetic nephropathy to ESRD, in that the presence of the deletion was associated with ESRD susceptibility(36). Despite having differences in the inclusion criteria to our meta-analysis, in that these investigations did not include any article published since 2011(35,36) and included individuals with microalbuminuria as cases(35), we returned similar results to the 2005 previous meta-analysis. Including individuals with microalbuminuria in the control population may cause challenges with phenotype definition as microalbuminuria may regress, remain stable, or progress to macroalbuminuria(37–39) over time. Individuals with microalbuminuria were therefore excluded from both case and control definitions to clarify the phenotypes in our review.

In our meta-analysis of *ACE*, which comprised 14,509 individuals with CKD and 18,157 individuals as controls from 94 population groups, we identified three significant associations of the *ACE* I/D polymorphism with CKD. Due to previously reported heterogeneity between different ethnic groups(35), we included this as a risk factor and performed each analysis per ethnicity (Table S2).

Firstly, comparisons between T2DN and T2DM in East Asian and South Asian populations returned significant results highlighting a protective effect of the *ACE* insertion in the development of diabetic nephropathy ($P=0.009$ and $P=0.01$ respectively). This result was mimicked in the comparison of individuals with ESRD, which was not caused by diabetic nephropathy, and healthy control individuals in an East Asian population ($P=0.008$). The *ACE* I/D polymorphism remains a well characterised genetic locus associated with the progression of diabetic nephropathy.

AGT encodes the AGT glycoprotein, which is created in the liver and facilitates the creation of angiotensin I(11,40). It is located on chromosome 1. Several investigations have been conducted into the *AGT* gene variants and their association with risk of CKD(40–43). Among these, Zhou and colleagues undertook a meta-analysis investigation into *AGT* rs699 and its association with ESRD(40). The results of this study are agreement with ours in relation to the European ethnicity. Our meta-analysis encompassing 5,076 individuals with renal disease and 6,134 individuals without; the T allele provided a protective effect in ESRD development ($P=0.002$) within this European population.

For peer review only - http://bmjopen.bmj.com/site/about/guidelines.xhtml

The *AGTR1* gene has been shown to be involved in the regulation of blood pressure, fluid and electrolyte balance(11). It may also have a role in inflammation and vasoconstriction(44). SNP rs5186, an AC nucleotide substitution at position 1166 in the 3' untranslated region of chromosome 3, is reportedly able to be recognised by microRNA-155. When the A allele is present at this locus, microRNA-155 is able to undergo complementary base-pairing with AGTR1 messenger RNA to suppress translation. However this is not possible when the alternative C allele is present, resulting in increased AGTR1 protein levels(45). This interplay may affect blood pressure regulation and warrants further investigation(45).

Previous studies have identified associations between rs5186 in *AGTR1* and diseases including coronary artery disease(46), systemic lupus erythematosus(47) and cancer(48). Several smaller studies had also been undertaken to assess links between this gene and renal disease(25,49–54). We conducted this study to provide a clearer understanding of the effect of this SNP on CKD.

This meta-analysis of *AGTR1* variants included 3,197 individuals with renal disease and 3,720 controls investigating rs5186. One significant result was identified; that the presence of an A allele at this locus provided a lower risk of developing T2DN in a South Asian population (P=0.001).

One meta-analysis published in 2014(55) assessed AGTR1 in individuals with CKD, ESRD, IgA nephropathy or vesicoureteral reflux. This meta-analysis identified that rs5186 was not associated with any of these diseases, which corresponds to the results from our investigation.

The three remaining RAAS genes included in this meta-analysis, *ACE2*, *AGTR2* and *REN,* have not been researched as extensively as the *ACE, AGT* and *AGTR1* for associations with renal disease. Very few articles were identified describing genetic association studies for these genes at the data extraction stage of the analysis, and those that were, were removed prior to the quantitative analysis stage, mainly due to the inclusion of paediatric individuals or non-human approaches, as outlined in Figures S1b, S1e and S1f. Further research into the *ACE2*, *AGTR2* and *REN* genes and their polymorphisms should be undertaken to elucidate their role in CKD and ESRD.

Some studies in our search could not be included in quantitative analysis as they lacked information relating to genotype counts and had an unclear measure and definition of albuminuria for both cases and controls, which could constitute a limitation. Publication bias was reported in two of our quantitative analyses, but was not found in the analyses which provided our significant results. Lack of clarity in phenotype definitions, along with unclear descriptions of ethnicities, inherently challenge the use of meta-analyses of different populations as a valid instrument to uncover robust associations. CKD itself has a range of causes including glomerular damage and declining eGFR without albuminuria. Other confounding factors such as hypertension and cardiovascular disease, and a lack of prospective follow up of included individuals, which would ensure phenotypes are robust and stable, may also cause conflicting results.

CONCLUSION

This meta-analysis of the RAAS pathway genes and their association with renal disease has provided evidence for five significant associations with individually small effect sizes that may cumulatively contribute to dysfunction of the RAAS pathway leading to kidney disease. The insertion in *ACE* I/D polymorphism was a protection factor for the development of diabetic nephropathy in individuals with type 2 diabetes mellitus from both East and South Asian origin, and for ESRD in an East Asian population. In Europeans, the T allele of the *AGT* rs699 conferred a lower risk of ESRD development

For peer review only - http://bmjopen.bmj.com/site/about/guidelines.xhtml

in healthy population. The A allele in *AGTR1* rs5186 acted as a protection factor for renal disease development in South Asian population.

Further study into the specific ethnicities and investigations into a broader range of RAAS linked genes, or a deeper analysis of them including investigations of more variants, may pinpoint the molecular basis underlying the role of pathway in kidney disease. Network analysis and functional studies enlightening the mechanisms involved ultimately will be required to complete the picture of RAAS variation in renal traits.

STATEMENTS AND CONTRIBUTIONS

Acknowledgements and Funding

LJS is the recipient of a Northern Ireland Kidney Research Fund Fellowship.

MCG is funded by a Science Foundation Ireland-Department for the Economy (SFI-DfE) Investigator Program Partnership Award (15/IA/3152).

RCC is the recipient of a Department for the Economy (DfE) PhD studentship.

None of the funding bodies had a role in the study design or analysis.

Statement of competing interests

The authors declare that they have no competing interests.

Availability of data and material

The datasets generated and/or analysed during the current study are available from the corresponding author on reasonable request.

Author contributions

LJS formulated the research plan, conducted the analysis, interpreted the data, drafted the manuscript and revised the manuscript.

MCG conducted the analysis, interpreted the data and revised the manuscript.

RCC conducted the analysis, interpreted the data and revised the manuscript.

APM acquired the funding, managed the project, interpreted the data and revised the manuscript.

AJM acquired the funding, formulated the research plan, interpreted the data, managed the project and revised the manuscript.

All authors read and approved the final manuscript.

For peer review only - http://bmjopen.bmj.com/site/about/guidelines.xhtml

REFERENCES

1. Levey AS, Coresh J. Chronic kidney disease. Lancet. 2012 Jan 14;379(9811):165–80.
2. Glassock RJ, Warnock DG, Delanaye P. The global burden of chronic kidney disease: Estimates, variability and pitfalls. Nat Rev Nephrol. 2017;13(2):104–14.
3. Mills KT, Xu Y, Zhang W, Bundy JD, Chen C-S, Kelly TN, et al. A systematic analysis of world-wide population-based data on the global burden of chronic kidney disease in 2010 HHS Public Access. Kidney Int. 2015;88(5):950–7.
4. Smyth LJ, Duffy S, Maxwell AP, McKnight AJ. Genetic and epigenetic factors influencing chronic kidney disease. Am J Physiol Renal Physiol. 2014;307(7):F757-76.
5. Brancati FL, Whelton PK, Randall BL, Neaton JD, Stamler J, Klag MJ. Risk of end-stage renal disease in diabetes mellitus: a prospective cohort study of men screened for MRFIT. Multiple Risk Factor Intervention Trial. JAMA. 1997;278(23):2069–74.
6. Klag MJ, Whelton PK, Randall BL, Neaton JD, Brancati FL, Ford CE, et al. Blood pressure and end-stage renal disease in men. N Engl J Med. 1996;334(1):13–8.
7. Cowie CC, Port FK, Wolfe RA, Savage PJ, Moll PP, Hawthorne VM. Disparities in Incidence of Diabetic End-Stage Renal Disease According to Race and Type of Diabetes. N Engl J Med. 1989;321(16):1074–9.
8. Mathur R, Dreyer G, Yaqoob MM, Hull SA. Ethnic differences in the progression of chronic kidney disease and risk of death in a UK diabetic population: an observational cohort study. BMJ Open. 2018;8(3):e020145.
9. Velez JCQ. The importance of the intrarenal renin-angiotensin system. Nat Clin Pract Nephrol. 2009;5(2):89–100.
10. Nishiyama A, Kim-Mitsuyama S. New Approaches to Blockade of the Renin–Angiotensin–Aldosterone System: Overview of Regulation of the Renin–Angiotensin–Aldosterone System. J Pharmacol Sci. 2010;113(4):289–91.
11. Remuzzi G, Perico N, Macia M, Ruggenenti P. The role of renin-angiotensin-aldosterone system in the progression of chronic kidney disease. Kidney Int Suppl. 2005;68(99):S57–65.
12. Nishiyama A, Kobori H. Independent regulation of renin–angiotensin–aldosterone system in the kidney. Clin Exp Nephrol. 2018;doi: 10.1007/s10157-018-1567-1.
13. Carey RM. The intrarenal renin-angiotensin system in hypertension. Adv Chronic Kidney Dis. 2015;22(3):204–10.
14. Zhang F, Liu H, Liu D, Liu Y, Li H, Tan X, et al. Effects of RAAS Inhibitors in Patients with Kidney Disease. Curr Hypertens Rep. 2017;19(9):72.
15. Coll E, Campos B, González-Núñez D, Botey A, Poch E. Association between the A1166C polymorphism of the angiotensin II receptor type 1 and progression of chronic renal insufficiency. J Nephrol. 2003;16(3):357–64.
16. Bermejo S, García CO, Rodríguez E, Barrios C, Otero S, Mojal S, et al. The renin-angiotensin-aldosterone system blockade in patients with advanced diabetic kidney disease. Nefrologia. 2017;32(2):197–206.
17. Yamout H, Lazich I, Bakris GL. Blood pressure, hypertension, RAAS blockade, and drug therapy in diabetic kidney disease. Adv Chronic Kidney Dis. 2014;21(3):281–6.

For peer review only - http://bmjopen.bmj.com/site/about/guidelines.xhtml

18. Rahimi Z. The Role of Renin Angiotensin Aldosterone System Genes in Diabetic Nephropathy. Can J Diabetes. 2016;40(2):178–83.

19. Mezzano S a, Ruiz-Ortega M, Egido J. Angiotensin II and renal fibrosis. Hypertension. 2001;38(3 Pt 2):635–8.

20. Kelly TN, Raj D, Rahman M, Kretzler M, Kallem RR, Ricardo AC, et al. The role of renin-angiotensin-aldosterone system genes in the progression of chronic kidney disease: Findings from the Chronic Renal Insufficiency Cohort (CRIC) study. Nephrol Dial Transplant. 2015;30(10):1711–8.

21. Ramanathan G, Elumalai R, Periyasamy S, Lakkakula BVKS. Renin gene rs1464816 polymorphism contributes to chronic kidney disease progression in ADPKD. J Biomed Sci. 2016;23(1):1–7.

22. Kawada N, Moriyama T, Yokoyama K, Yamauchi A, Ando A, Mikami H, et al. Renin-angiotensin system component gene polymorphisms in Japanese maintenance haemodialysis patients. Nephrology. 1997;3(4):521–6.

23. Parsa A, Lovett DH, Peden EA, Zhu L, Seldin MF, Criswell LA. Renin-angiotensin system gene polymorphisms predict the progression to renal insufficiency among Asians with lupus nephritis. Genes Immun. 2005;6(3):217–24.

24. Özkaya O, Söylemezoğlu O, Gönen S, Misirlioğolu M, Tuncer S, Kalman S, et al. Renin-angiotensin system gene polymorphisms: Association with susceptibility to Henoch-Schonlein purpura and renal involvement. Clin Rheumatol. 2006;25(6):861–5.

25. Lau YK, Woo KT, Choong HL, Zhao Y, Tan HB, Chong SMCF, et al. Renin-angiotensin system gene polymorphisms: Its impact on IgAN and its progression to end-stage renal failure among Chinese in Singapore. Nephron - Physiol. 2004;97(1):1–9.

26. Mtiraoui N, Ezzidi I, Turki A, Chaieb M, Mahjoub T, Almawi WY. Renin-angiotensin-aldosterone system genotypes and haplotypes affect the susceptibility to nephropathy in type 2 diabetes patients. J Renin Angiotensin Aldosterone Syst. 2011;12(4):572–80.

27. Stroup DF, Berlin JA, Morton SC, Olkin I, Williamson GD, Rennie D, et al. Meta-analysis of observational studies in epidemiology: a proposal for reporting. Meta-analysis Of Observational Studies in Epidemiology (MOOSE) group. JAMA. 2000;283(15):2008–12.

28. International Genome Population [Internet]. Available from: www.internationalgenome.org/category/population

29. Higgins JPT, Thompson SG, Deeks JJ, Altman DG. Measuring inconsistency in meta-analyses. BMJ Br Med J. 2003;327(7414):557–60.

30. Rieder MJ, Taylor SL, Clark AG, Nickerson DA. Sequence variation in the human angiotensin converting enzyme. Nat Genet. 1999;22(1):59–62.

31. Moon JY. Recent update of renin-angiotensin-aldosterone system in the pathogenesis of hypertension. Electrolyte Blood Press. 2013;11(2):41–5.

32. Rigat B, Hubert C, Corvol P, Soubrier R. PCR detection of the insertion/deletion polymorphism of the human angiotensin converting enzyme gene (DCP1) (dipeptidyl carboxypeptidase 1). Nucleic Acids Res. 1992;20(6):1433.

33. Ha S-K. ACE Insertion/Deletion Polymorphism and Diabetic Nephropathy: Clinical Implications of Genetic Information. J Diabetes Res. 2014;2014:1–8.

For peer review only - http://bmjopen.bmj.com/site/about/guidelines.xhtml

For peer review only

34. Marre M, Bernadet P, Gallois Y, Savagner F, Guyene TT, Hallab M, et al. Relationships between angiotensin I converting enzyme gene polymorphism, plasma levels, and diabetic retinal and renal complications. Diabetes. 1994;43(3):384–8.

35. Ng DPK, Tai BC, Koh D, Tan KW, Chia KS. Angiotensin-I converting enzyme insertion/deletion polymorphism and its association with diabetic nephropathy: A meta-analysis of studies reported between 1994 and 2004 and comprising 14,727 subjects. Diabetologia. 2005;48(5):1008–16.

36. Yu Z-Y, Chen L-S, Zhang L-C, Zhou T-B. Meta-analysis of the relationship between ACE I/D gene polymorphism and end-stage renal disease in patients with diabetic nephropathy. Nephrology (Carlton). 2012;17(5):480–7.

37. Caramori ML, Fioretto P, Mauer M. The need for early predictors of diabetic nephropathy risk: Is albumin excretion rate sufficient? Diabetes. 2000;49(9):1399–408.

38. Perkins BA, Ficociello LH, Silva KH, Finkelstein DM, Warram JH, Krolewski AS. Regression of microalbuminuria in type 1 diabetes. N Engl J Med. 2003;348(23):2285–93.

39. Zelmanovitz T, Gerchman F, Balthazar AP, Thomazelli FC, Matos JD, Canani LH. Diabetic nephropathy. Diabetol Metab Syndr. 2009;1(1):10.

40. Zhou T-B, Yin S-S, Qin Y-H. Association of angiotensinogen M235T gene polymorphism with end-stage renal disease risk: a meta-analysis. Mol Biol Rep. 2013;40(2):765–72.

41. Turgut S, AkIn F, AkcIlar R, Ayada C, Turgut G. Angiotensin converting enzyme I/D, angiotensinogen M235T and AT1-R A/C1166 gene polymorphisms in patients with acromegaly. Mol Biol Rep. 2011;38(1):569–76.

42. Wang YJ, Pan Y. Angiotensinogen gene M235T polymorphism and risk of coronary artery disease: a meta-analysis. Mol Med Rep. 2012;6(4):884–8.

43. Lin R, Lei Y, Yuan Z, Ju H, Li D. Angiotensinogen Gene M235T and T174M Polymorphisms and Susceptibility of Pre-Eclampsia: A Meta-Analysis. Ann Hum Genet. 2012;76(5):377–86.

44. Chow BSM, Allen TJ. Angiotensin II type 2 receptor (AT2R) in renal and cardiovascular disease. Clin Sci. 2016;130(15):1307–26.

45. Ceolotto G, Papparella I, Bortoluzzi A, Strapazzon G, Ragazzo F, Bratti P, et al. Interplay between miR-155, AT1R A1166C polymorphism, and AT1R expression in young untreated hypertensives. Am J Hypertens. 2011;24(2):241–6.

46. Duan LJ, Wang XD. Analysis of correlations between coronary heart disease and haplotypes of the angiotensin II receptor type 1 (AGTR1) gene. Genet Mol Res. 2016;15(1):doi: 10.4238/gmr.15017457.

47. Baniamerian H, Bahrehmand F, Vaisi-Raygani A, Rahimi Z, Pourmotabbed T. Angiotensin type 1 receptor A1166C polymorphism and systemic lupus erythematosus: correlation with cellular immunity and oxidative stress markers. Lupus. 2017;26(14):1534–9.

48. Pringle KG, Delforce SJ, Wang Y, Ashton KA, Proietto A, Otton G, et al. Renin–angiotensin system gene polymorphisms and endometrial cancer. Endocr Connect. 2016;5(3):128–35.

49. Gao J, Yu Q-L, Fu R-G, Wei L-T, Wang M, Dong F-M, et al. Lack of Association Between Polymorphisms in AGT and ATR1 and IgA Nephropathy in a Chinese Population. Genet Test Mol Biomarkers. 2015;19(12):710–3.

50. Huang HD, Lin FJ, Li XJ, Wang LR, Jiang GR. Genetic polymorphisms of the renin-angiotensin-

For peer review only

For peer review only - http://bmjopen.bmj.com/site/about/guidelines.xhtml

aldosterone system in Chinese patients with end-stage renal disease secondary to IgA nephropathy. Chin Med J (Engl). 2010;123(22):3238–42.

51. Kim SM, Chin HJ, Oh YK, Kim YS, Kim S, Lim CS. Blood pressure-related genes and the progression of IgA nephropathy. Nephron Clin Pract. 2009;113(4):c301-8.

52. Savage DA, Feeney SA, Fogarty DG, Maxwell AP. Risk of developing diabetic nephropathy is not associated with synergism between the angiotensin II (type 1) receptor C1166 allele and poor glycaemic control. Nephrol Dial Transpl. 1999;14(4):891–4.

53. Van Ittersum FJ, De Man AME, Thijssen S, De Knijff P, Slagboom E, Smulders Y, et al. Genetic polymorphisms of the renin-angiotensin system and complications of insulin-dependent diabetes mellitus. Nephrol Dial Transplant. 2000;15(7):1000–7.

54. Prasad P, Tiwari AK, Kumar KP, Ammini A, Gupta A, Gupta R, et al. Chronic renal insufficiency among Asian Indians with type 2 diabetes: I. Role of RAAS gene polymorphisms. BMC Med Genet. 2006;7(1):42.

55. Braliou GG, Grigoriadou AMG, Kontou PI, Bagos PG. The role of genetic polymorphisms of the Renin-Angiotensin System in renal diseases: A meta-analysis. Comput Struct Biotechnol J. 2014;10(7):1–7.

For peer review only

For peer review only - http://bmjopen.bmj.com/site/about/guidelines.xhtml

Figure 1: Workflow pattern

Electronic Database Search

PubMed and Web of Science Databases were searched yielding 3,286 results in total for RAAS genes (*ACE*, *ACE2*, *AGT*, *AGTR1*, *AGTR2* and *REN*)

↓

Duplicate Papers Removed

403 duplicate papers were removed from each gene search. Duplicates remained between genes as several articles contained data for several genes.

↓

Abstract Screening

2,363 articles were removed as they did not meet the inclusion criteria.*

↓

Full Text Screening

52 articles were removed as they included overlaps in patient groups published elsewhere or contained no data.

↓

Inclusion of Articles Identified from Bibliographies

53 papers were identified from bibliographies during full-text screening.

↓

Final Study Selection for Data Extraction

521** papers met the inclusion criteria for all genes and were included for data extraction. 222 individual articles.

↓

Final Study Selection for Quantitative Analysis

84 articles were removed as the data provided did not generate at least three populations with the same ethnicity per SNP. 138 papers were included in the quantitative analysis.

*Inclusion criteria: Any paper was removed from the analysis if it was not written in English or Spanish, was a non-human study, was a paediatric study, was not a case-control study, did not have a renal disease phenotype, did not include data for the gene of interest, was a gene expression based study or had a pharmaceutical drug focus.

**Final selection: Several papers provided data for > 1 gene, so this figure includes duplicates.

Figure 2a: ***ACE*** **I/D investigation, individuals with type 2 diabetic nephropathy compared to individuals with type 2 diabetes mellitus in an East Asian Population (insertion compared to deletion)**

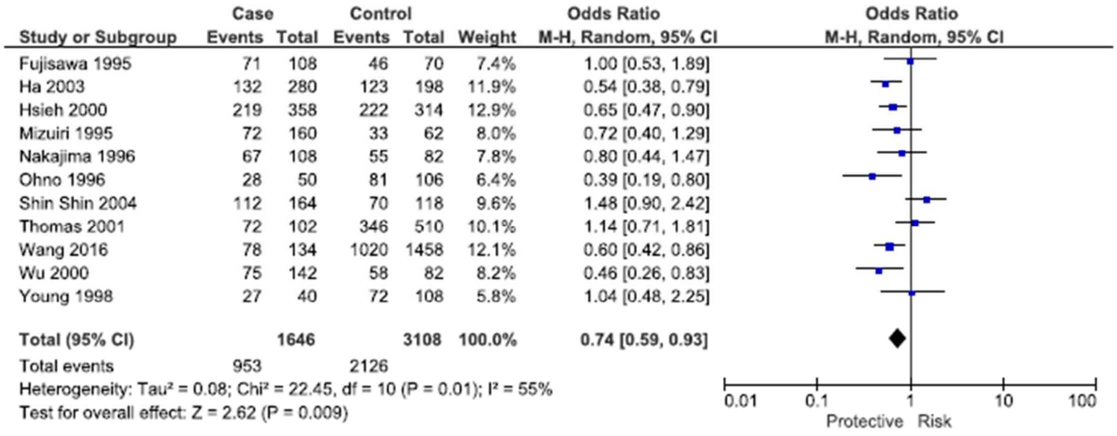

Study or Subgroup	Case Events	Case Total	Control Events	Control Total	Weight	Odds Ratio M-H, Random, 95% CI
Fujisawa 1995	71	108	46	70	7.4%	1.00 [0.53, 1.89]
Ha 2003	132	280	123	198	11.9%	0.54 [0.38, 0.79]
Hsieh 2000	219	358	222	314	12.9%	0.65 [0.47, 0.90]
Mizuiri 1995	72	160	33	62	8.0%	0.72 [0.40, 1.29]
Nakajima 1996	67	108	55	82	7.8%	0.80 [0.44, 1.47]
Ohno 1996	28	50	81	106	6.4%	0.39 [0.19, 0.80]
Shin Shin 2004	112	164	70	118	9.6%	1.48 [0.90, 2.42]
Thomas 2001	72	102	346	510	10.1%	1.14 [0.71, 1.81]
Wang 2016	78	134	1020	1458	12.1%	0.60 [0.42, 0.86]
Wu 2000	75	142	58	82	8.2%	0.46 [0.26, 0.83]
Young 1998	27	40	72	108	5.8%	1.04 [0.48, 2.25]
**Total (95% CI)**		**1646**		**3108**	**100.0%**	**0.74 [0.59, 0.93]**
Total events	953		2126			

Heterogeneity: $Tau^2 = 0.08$; $Chi^2 = 22.45$, df = 10 (P = 0.01); $I^2 = 55\%$
Test for overall effect: Z = 2.62 (P = 0.009)

Figure 2b: ***ACE*** **I/D investigation, individuals with type 2 diabetic nephropathy compared to individuals with type 2 diabetes mellitus in a South Asian Population (insertion compared to deletion)**

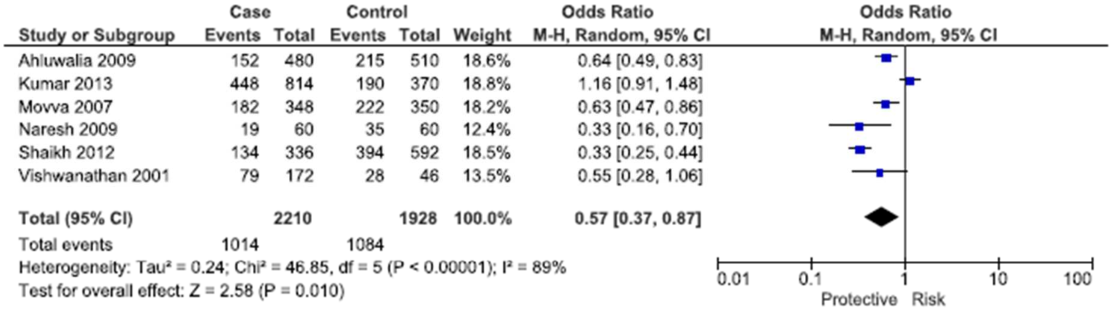

Study or Subgroup	Case Events	Case Total	Control Events	Control Total	Weight	Odds Ratio M-H, Random, 95% CI
Ahluwalia 2009	152	480	215	510	18.6%	0.64 [0.49, 0.83]
Kumar 2013	448	814	190	370	18.8%	1.16 [0.91, 1.48]
Movva 2007	182	348	222	350	18.2%	0.63 [0.47, 0.86]
Naresh 2009	19	60	35	60	12.4%	0.33 [0.16, 0.70]
Shaikh 2012	134	336	394	592	18.5%	0.33 [0.25, 0.44]
Vishwanathan 2001	79	172	28	46	13.5%	0.55 [0.28, 1.06]
**Total (95% CI)**		**2210**		**1928**	**100.0%**	**0.57 [0.37, 0.87]**
Total events	1014		1084			

Heterogeneity: $Tau^2 = 0.24$; $Chi^2 = 46.85$, df = 5 (P < 0.00001); $I^2 = 89\%$
Test for overall effect: Z = 2.58 (P = 0.010)

Figure 2c: ***ACE*** **I/D investigation, individuals with end-stage renal disease compared to healthy controls in an East Asian Population (insertion compared to deletion)**

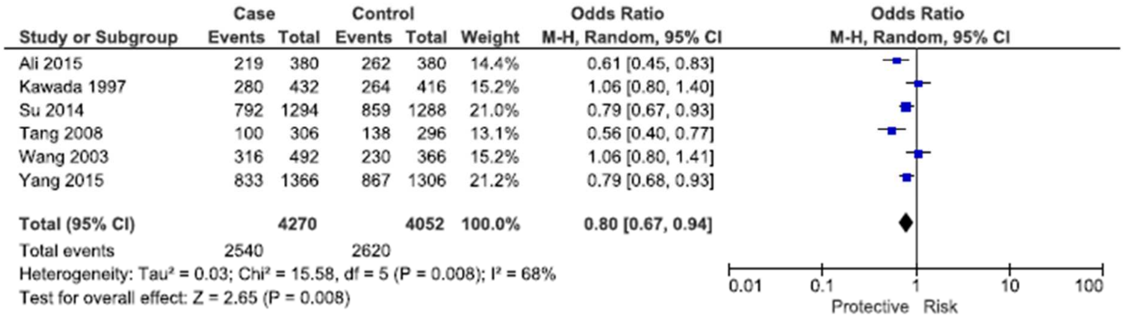

Study or Subgroup	Case Events	Case Total	Control Events	Control Total	Weight	Odds Ratio M-H, Random, 95% CI
Ali 2015	219	380	262	380	14.4%	0.61 [0.45, 0.83]
Kawada 1997	280	432	264	416	15.2%	1.06 [0.80, 1.40]
Su 2014	792	1294	859	1288	21.0%	0.79 [0.67, 0.93]
Tang 2008	100	306	138	296	13.1%	0.56 [0.40, 0.77]
Wang 2003	316	492	230	366	15.2%	1.06 [0.80, 1.41]
Yang 2015	833	1366	867	1306	21.2%	0.79 [0.68, 0.93]
**Total (95% CI)**		**4270**		**4052**	**100.0%**	**0.80 [0.67, 0.94]**
Total events	2540		2620			

Heterogeneity: $Tau^2 = 0.03$; $Chi^2 = 15.58$, df = 5 (P = 0.008); $I^2 = 68\%$
Test for overall effect: Z = 2.65 (P = 0.008)

Figure 2d: ***AGT*** **rs699 investigation, individuals with end-stage renal disease compared to healthy controls in a European Population (T allele compared to C)**

Study or Subgroup	Case Events	Case Total	Control Events	Control Total	Weight	Odds Ratio M-H, Random, 95% CI
Beige 1996	285	538	307	536	20.1%	0.84 [0.66, 1.07]
Buraczynska 2006	734	1490	564	1040	46.2%	0.82 [0.70, 0.96]
Losito 2002	165	320	190	338	12.4%	0.83 [0.61, 1.13]
Lovati 2001	299	520	393	654	21.3%	0.90 [0.71, 1.14]
**Total (95% CI)**		**2868**		**2568**	**100.0%**	**0.84 [0.76, 0.94]**
Total events	1483		1454			

Heterogeneity: $Tau^2 = 0.00$; $Chi^2 = 0.42$, df = 3 (P = 0.94); $I^2 = 0\%$
Test for overall effect: Z = 3.14 (P = 0.002)

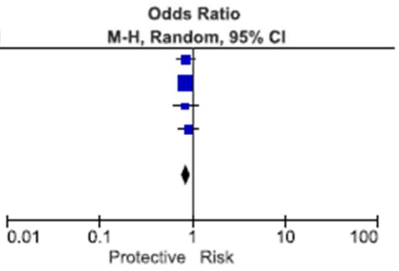

For peer review only

Figure 2e: ***AGTR1*** **rs5186 investigation, individuals with type 2 diabetic nephropathy compared to individuals with type 2 diabetes mellitus in a South Asian Population (A allele compared to C allele)**

Study or Subgroup	Case Events	Case Total	Control Events	Control Total	Weight	Odds Ratio M-H, Random, 95% CI
Ahluwalia 2009	320	480	381	510	34.5%	0.68 [0.51, 0.89]
Prasad 2006	360	386	417	450	13.0%	1.10 [0.64, 1.87]
Shah 2013	789	1192	840	1122	52.5%	0.66 [0.55, 0.79]
**Total (95% CI)**		**2058**		**2082**	**100.0%**	**0.71 [0.58, 0.87]**
Total events	1469		1638			

Heterogeneity: $Tau^2 = 0.01$; $Chi^2 = 3.19$, df = 2 (P = 0.20); $I^2 = 37\%$
Test for overall effect: Z = 3.22 (P = 0.001)

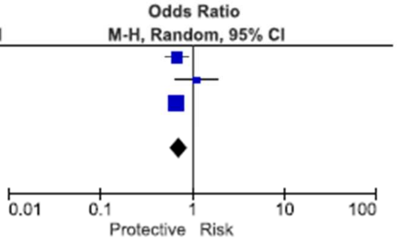

Supplementary Figure S1a: *ACE* Study Flow Diagram

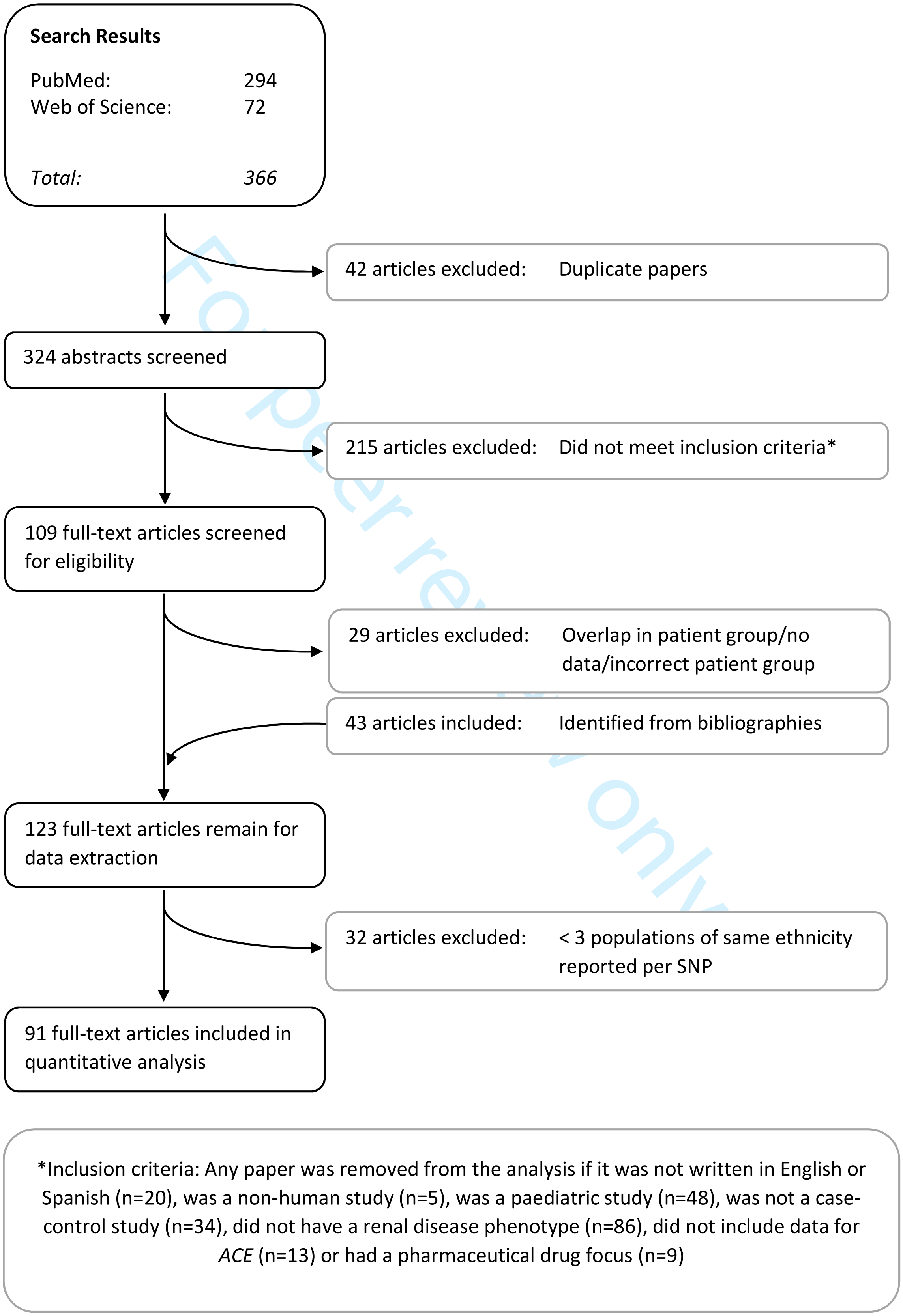

Supplementary Figure S1b: *ACE2* Study Flow Diagram

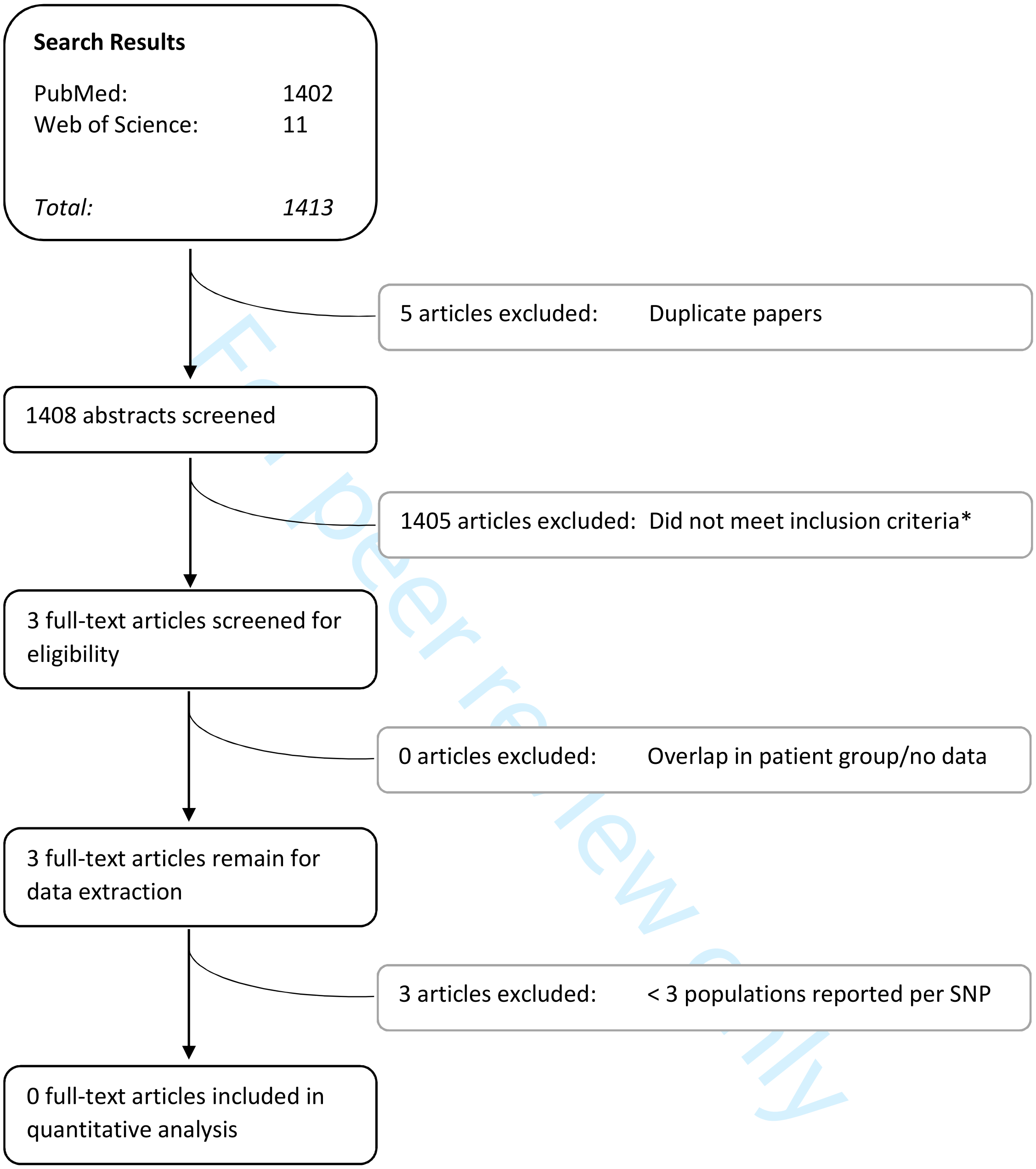

*Inclusion criteria: Any paper was removed from the analysis if it was not written in English or Spanish (n=52), was a non-human study (n=96), was a paediatric study (n=135), was not a case-control study (n=33), did not have a renal disease phenotype (n=630), did not include data for *ACE2* (n=415) or had a pharmaceutical drug focus (n=44)

For peer review only - http://bmjopen.bmj.com/site/about/guidelines.xhtml

Supplementary Figure S1c: *AGT* Study Flow Diagram

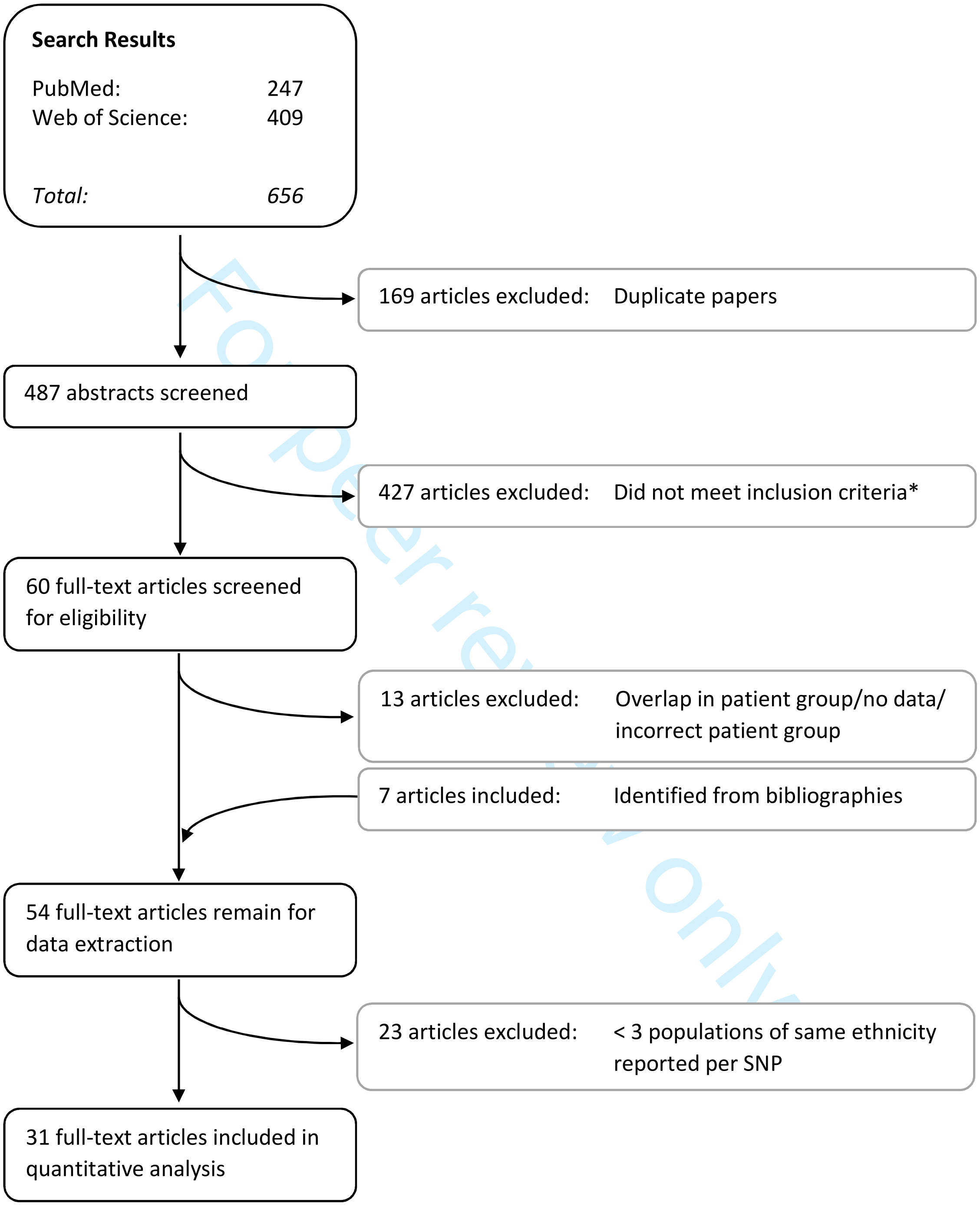

*Inclusion criteria: Any paper was removed from the analysis if it was not written in English or Spanish (n=16), was a non-human study (n=112), was a paediatric study (n=37), was not a case-control study (n=49), did not have a renal disease phenotype (n=127), did not include data for *AGT* (n=69), was a gene expression based study (n=1) or had a pharmaceutical drug focus (n=16)

Supplementary Figure S1d: *AGTR1* Study Flow Diagram

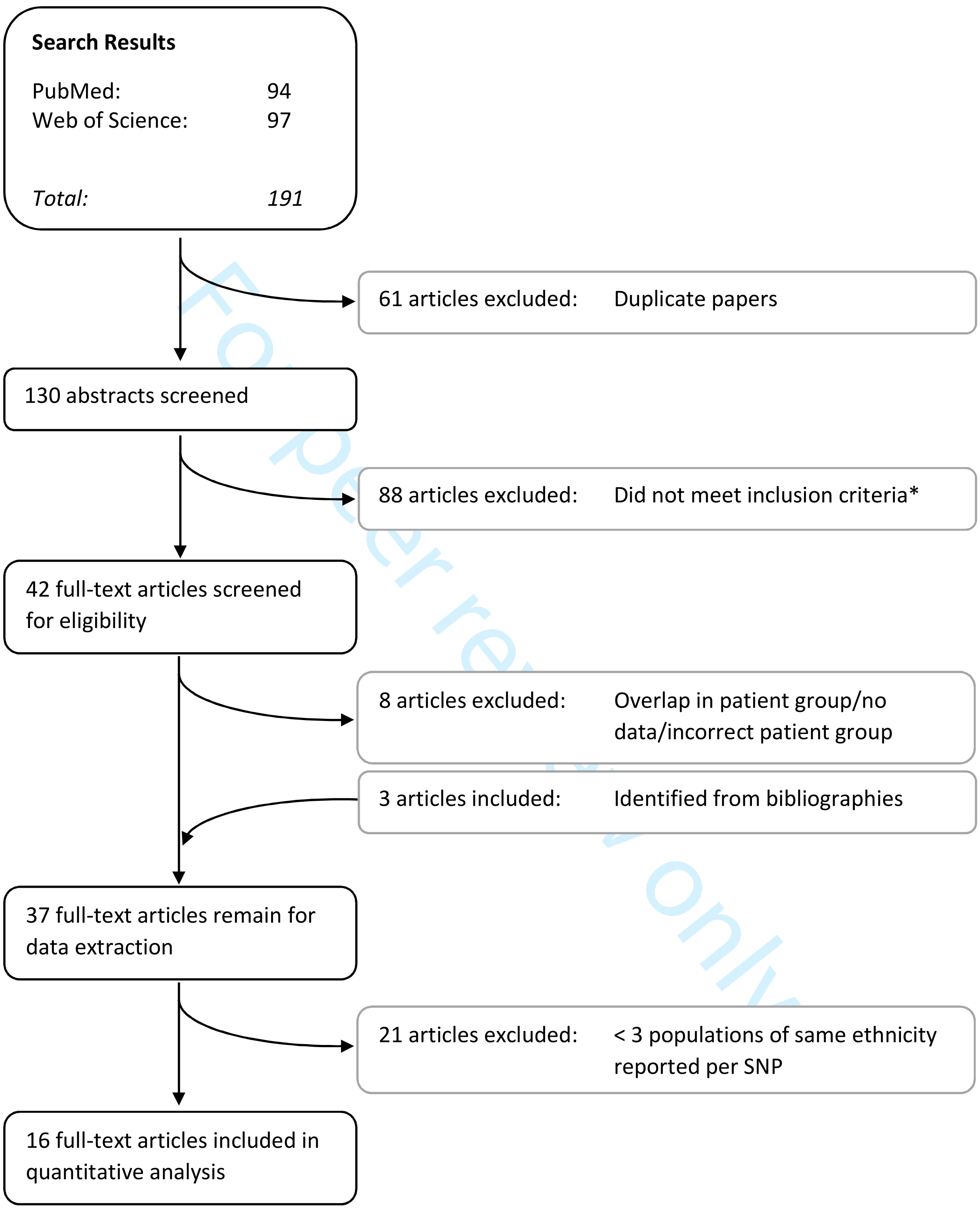

*Inclusion criteria: Any paper was removed from the analysis if it was not written in English or Spanish (n=8), was a non-human study (n=10), was a paediatric study (n=19), was not a case-control study (n=14), did not have a renal disease phenotype (n=25), did not include data for *AGTR1* (n=2), was a gene expression based study (n=1) or had a pharmaceutical drug focus (n=9)

For peer review only

Supplementary Figure S1e: *AGTR2* Study Flow Diagram

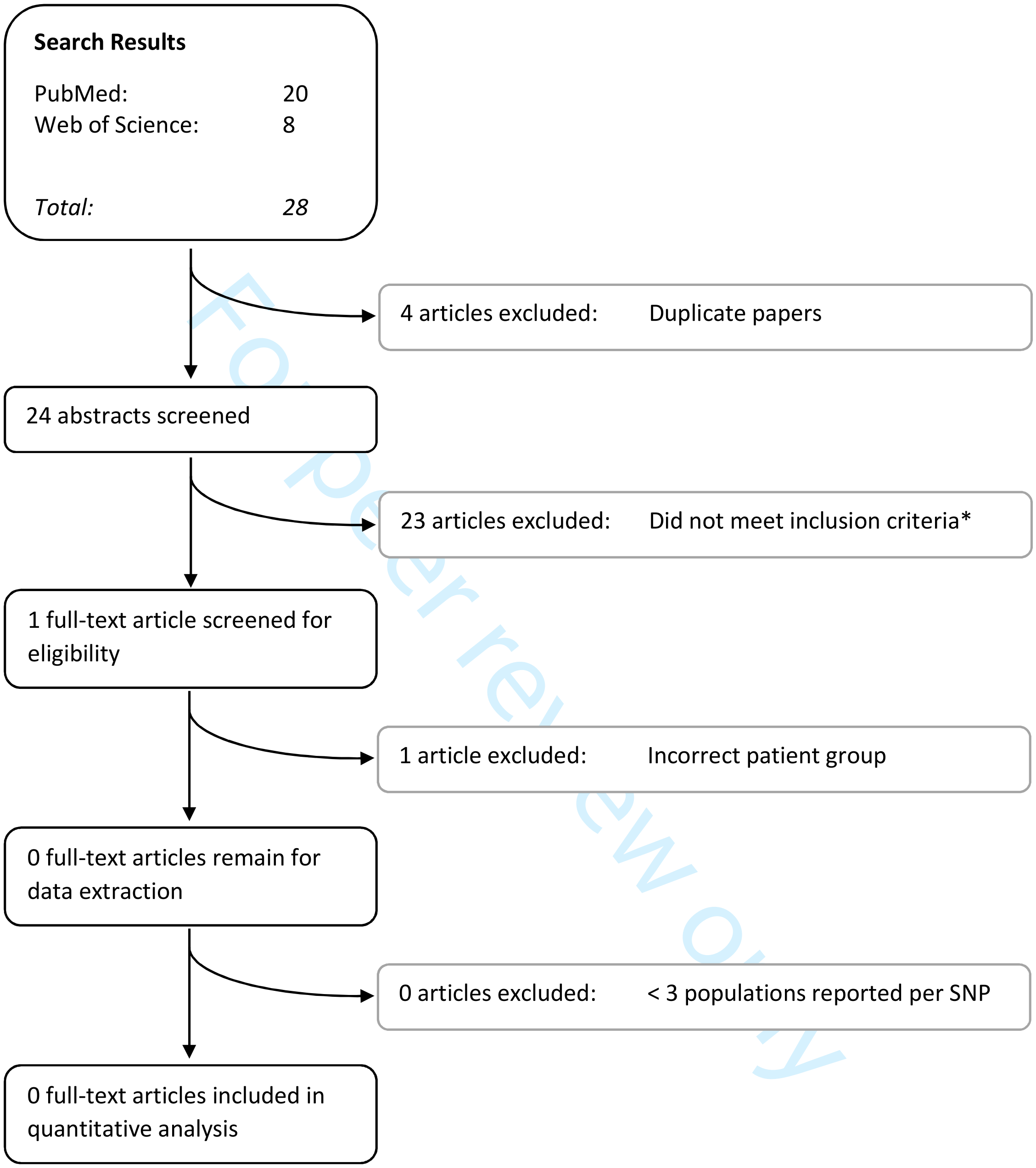

*Inclusion criteria: Any paper was removed from the analysis if it was not written in English or Spanish (n=1), was a non-human study (n=1), was a paediatric study (n=6), was not a case-control study (n=4), did not have a renal disease phenotype (n=5) or did not include data for *AGTR2* (n=6)

Supplementary Figure S1f: *REN* Study Flow Diagram

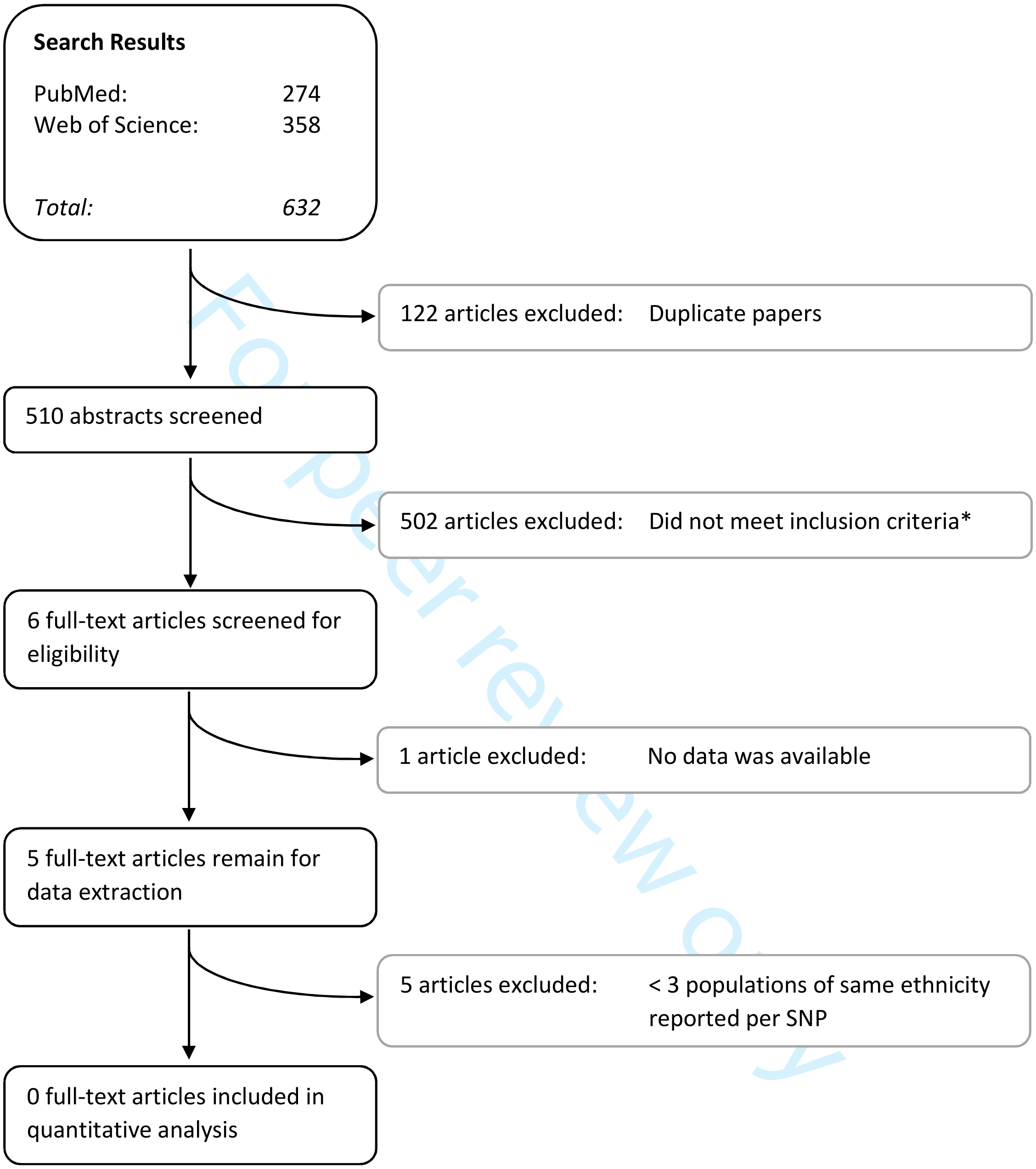

*Inclusion criteria: Any paper was removed from the analysis if it was not written in English or Spanish (n=17), was a non-human study (n=69), was a paediatric study (n=45), was not a case-control study (n=64), did not have a renal disease phenotype (n=166), did not include data for *REN* (n=124), was a gene expression based study (n=3) or had a pharmaceutical drug focus (n=14)

For peer review only

ACE

Supplementary Figure S2a: *ACE* I/D investigation, individuals with type 1 diabetic nephropathy compared to individuals with type 1 diabetes mellitus in a European Population (insertion compared to deletion)

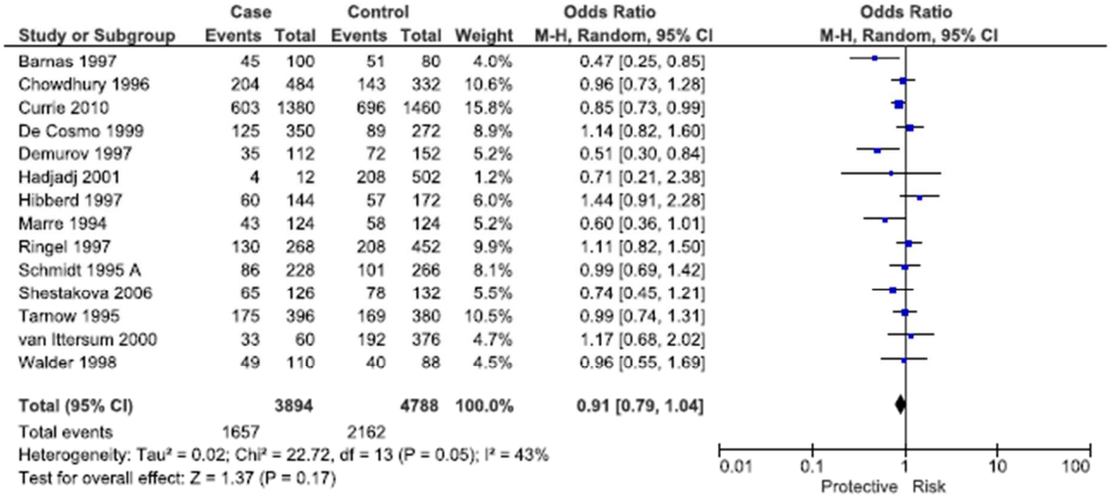

Supplementary Figure S2b: *ACE* I/D investigation, individuals with type 2 diabetic nephropathy compared to individuals with type 2 diabetes mellitus in a European Population (insertion compared to deletion)

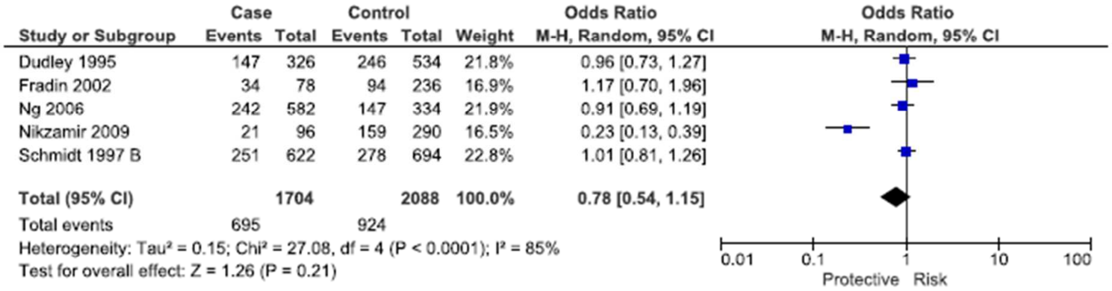

Supplementary Figure S2c: *ACE* I/D investigation, individuals with type 2 diabetic nephropathy compared to individuals with type 2 diabetes mellitus in a Middle Eastern Population (insertion compared to deletion)

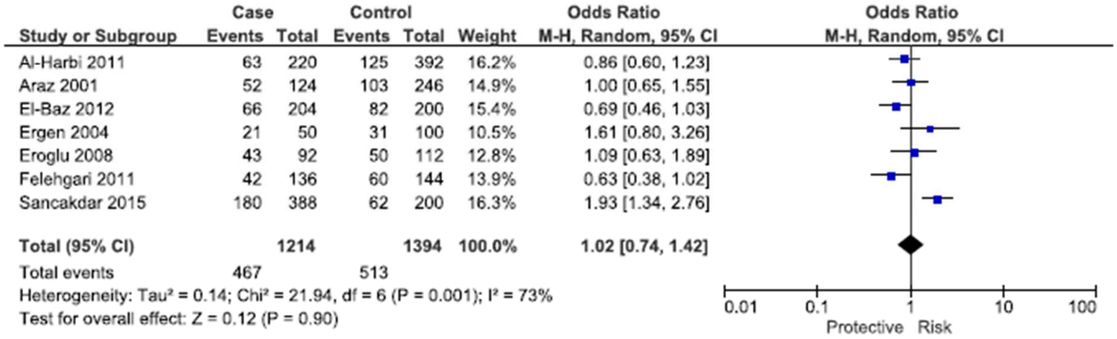

Supplementary Figure S2d: *ACE* I/D investigation, individuals with IgA nephropathy compared to healthy controls in an East Asian Population (insertion compared to deletion)

Study or Subgroup	Case Events	Case Total	Control Events	Control Total	Weight	Odds Ratio M-H, Random, 95% CI
Guo 2016	54	90	42	90	6.5%	1.71 [0.95, 3.10]
Huang 2010	155	260	168	240	12.5%	0.63 [0.44, 0.92]
Jung 2010	298	522	360	600	19.2%	0.89 [0.70, 1.13]
Lau 2004	154	236	137	188	10.7%	0.70 [0.46, 1.06]
Suzuki 2004	378	638	328	540	19.5%	0.94 [0.74, 1.19]
Yoon 2002	204	382	268	466	17.2%	0.85 [0.64, 1.11]
Yorioka 1995	67	96	139	206	7.8%	1.11 [0.66, 1.88]
Yoshida 1995	57	106	62	92	6.7%	0.56 [0.32, 1.00]
**Total (95% CI)**		**2330**		**2422**	**100.0%**	**0.86 [0.72, 1.02]**
Total events	1367		1504			

Heterogeneity: Tau² = 0.02; Chi² = 12.42, df = 7 (P = 0.09); I² = 44%
Test for overall effect: Z = 1.79 (P = 0.07)

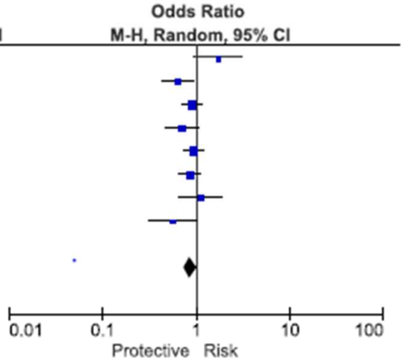

Supplementary Figure S2e: *ACE* I/D investigation, individuals with IgA nephropathy compared to healthy controls in a European Population (insertion compared to deletion)

Study or Subgroup	Case Events	Case Total	Control Events	Control Total	Weight	Odds Ratio M-H, Random, 95% CI
Burg 1997	26	54	60	120	5.6%	0.93 [0.49, 1.77]
Drouet 2002	78	250	54	154	12.8%	0.84 [0.55, 1.29]
Harden 1995	79	200	76	196	14.3%	1.03 [0.69, 1.54]
Pawlik 2014	25	62	180	374	7.8%	0.73 [0.42, 1.26]
Pei 1997	145	336	91	200	18.7%	0.91 [0.64, 1.29]
Schmidt 1995 B	168	408	197	468	32.0%	0.96 [0.74, 1.26]
Stratta 1999	62	162	38	100	8.8%	1.01 [0.61, 1.69]
**Total (95% CI)**		**1472**		**1612**	**100.0%**	**0.93 [0.80, 1.08]**
Total events	583		696			

Heterogeneity: Tau² = 0.00; Chi² = 1.42, df = 6 (P = 0.96); I² = 0%
Test for overall effect: Z = 0.97 (P = 0.33)

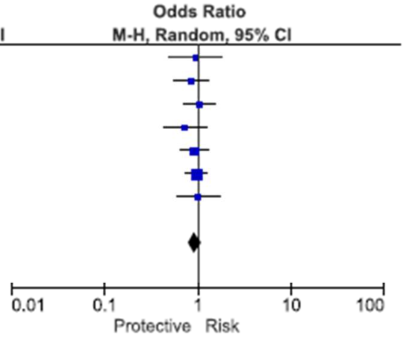

Supplementary Figure S2f: *ACE* I/D investigation, individuals with chronic kidney disease compared to healthy controls in a South Asian Population (insertion compared to deletion)

Study or Subgroup	Case Events	Case Total	Control Events	Control Total	Weight	Odds Ratio M-H, Random, 95% CI
Anbazhagan 2009	124	236	99	196	26.9%	1.08 [0.74, 1.58]
Chen 2014	314	466	617	898	28.0%	0.94 [0.74, 1.20]
Nagamani 2015	152	294	195	422	27.6%	1.25 [0.92, 1.68]
Shanmuganathan 2015	10	60	55	60	17.6%	0.02 [0.01, 0.06]
**Total (95% CI)**		**1056**		**1576**	**100.0%**	**0.53 [0.24, 1.14]**
Total events	600		966			

Heterogeneity: Tau² = 0.53; Chi² = 50.55, df = 3 (P < 0.00001); I² = 94%
Test for overall effect: Z = 1.63 (P = 0.10)

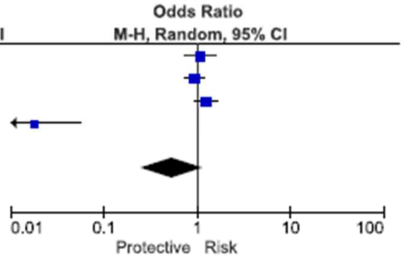

Supplementary Figure S2g: *ACE* I/D investigation, individuals with end-stage renal disease compared to healthy controls in a European Population (insertion compared to deletion)

Study or Subgroup	Case Events	Case Total	Control Events	Control Total	Weight	Odds Ratio M-H, Random, 95% CI
Aucella 2003	315	922	916	2614	15.6%	0.96 [0.82, 1.13]
Buraczynska 2006	688	1490	492	1040	15.6%	0.96 [0.82, 1.12]
Dixit 2002	32	52	17	44	2.4%	2.54 [1.11, 5.80]
Losito 2002	135	320	128	338	9.7%	1.20 [0.88, 1.64]
McLaughlin 1996	654	1644	353	742	14.9%	0.73 [0.61, 0.87]
Nicod 2002	282	520	261	520	12.1%	1.18 [0.92, 1.50]
Ortiz 2003	68	234	80	258	7.7%	0.91 [0.62, 1.34]
Schmidt 1996	105	212	80	190	7.5%	1.35 [0.91, 2.00]
van der Sman-de Beer 2005	400	830	459	944	14.4%	0.98 [0.82, 1.18]
**Total (95% CI)**		**6224**		**6690**	**100.0%**	**1.01 [0.88, 1.16]**
Total events	2679		2786			

Heterogeneity: $Tau^2 = 0.02$; $Chi^2 = 22.57$, df = 8 (P = 0.004); $I^2 = 65\%$
Test for overall effect: Z = 0.17 (P = 0.86)

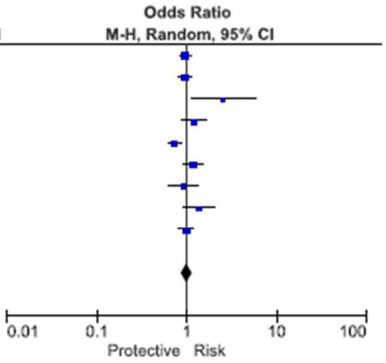

Supplementary Figure S2h: *ACE* I/D investigation, individuals with primary glomerulonephritis compared to healthy controls in a European Population (insertion compared to deletion)

Study or Subgroup	Case Events	Case Total	Control Events	Control Total	Weight	Odds Ratio M-H, Random, 95% CI
Burg 1997	44	92	60	120	12.2%	0.92 [0.53, 1.58]
Pawlik 2014	101	218	180	374	32.1%	0.93 [0.67, 1.30]
Stratta 2004	96	234	134	342	31.2%	1.08 [0.77, 1.52]
Zsom 2011	62	146	198	400	24.6%	0.75 [0.51, 1.10]
**Total (95% CI)**		**690**		**1236**	**100.0%**	**0.92 [0.76, 1.12]**
Total events	303		572			

Heterogeneity: $Tau^2 = 0.00$; $Chi^2 = 1.91$, df = 3 (P = 0.59); $I^2 = 0\%$
Test for overall effect: Z = 0.82 (P = 0.41)

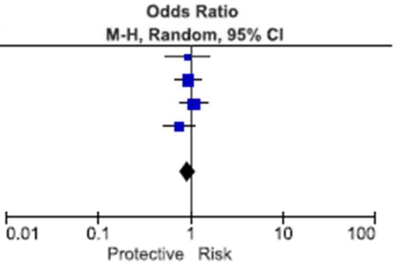

Supplementary Figure S2i: *ACE* I/D investigation, individuals who have had renal transplants compared to healthy controls in a European Population (insertion compared to deletion)

Study or Subgroup	Case Events	Case Total	Control Events	Control Total	Weight	Odds Ratio M-H, Random, 95% CI
Beige 1997	251	538	244	538	27.2%	1.05 [0.83, 1.34]
El-Essawy 2002	246	588	170	362	24.7%	0.81 [0.62, 1.06]
Hueso 2004	122	360	96	226	18.2%	0.69 [0.49, 0.98]
Stratta 2008	122	338	112	338	20.0%	1.14 [0.83, 1.57]
Viklický 2001	28	60	599	1306	9.9%	1.03 [0.61, 1.73]
**Total (95% CI)**		**1884**		**2770**	**100.0%**	**0.93 [0.77, 1.11]**
Total events	769		1221			

Heterogeneity: $Tau^2 = 0.02$; $Chi^2 = 6.59$, df = 4 (P = 0.16); $I^2 = 39\%$
Test for overall effect: Z = 0.80 (P = 0.42)

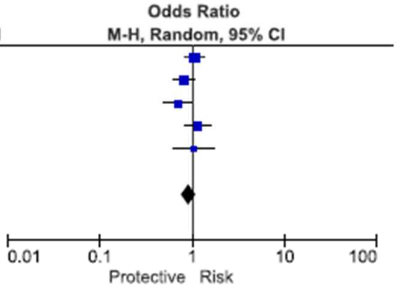

Supplementary Figure S2j: *ACE* I/D investigation, individuals with end-stage renal disease linked to type 2 diabetic nephropathy compared to individuals with type 2 diabetes mellitus in an East Asian Population (insertion compared to deletion)

	Case		Control			Odds Ratio
Study or Subgroup	Events	Total	Events	Total	Weight	M-H, Random, 95% CI
Doi 1996	115	200	168	248	20.6%	0.64 [0.44, 0.95]
Jayapalan 2010	163	254	93	162	20.1%	1.33 [0.89, 1.99]
Lu 2016	238	420	307	444	24.0%	0.58 [0.44, 0.77]
Park 2005	103	206	111	176	19.9%	0.59 [0.39, 0.88]
Taniwaki 2001	53	84	88	138	15.5%	0.97 [0.55, 1.71]
**Total (95% CI)**		**1164**		**1168**	**100.0%**	**0.76 [0.55, 1.05]**
Total events	672		767			

Heterogeneity: Tau² = 0.09; Chi² = 13.42, df = 4 (P = 0.009); I² = 70%
Test for overall effect: Z = 1.66 (P = 0.10)

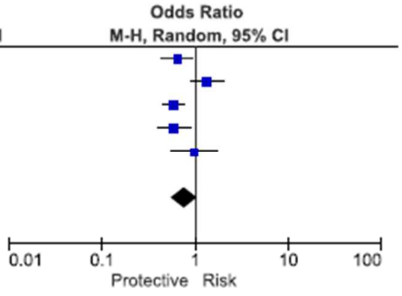

Supplementary Figure S2k: *ACE* I/D investigation, individuals with end-stage renal disease linked to type 2 diabetic nephropathy compared to individuals with type 2 diabetes mellitus in a European Population (insertion compared to deletion)

	Case		Control			Odds Ratio
Study or Subgroup	Events	Total	Events	Total	Weight	M-H, Random, 95% CI
Grzeszczak 1998	128	254	244	508	33.9%	1.10 [0.81, 1.49]
Ringel 1997	150	322	141	280	33.7%	0.86 [0.62, 1.18]
Schmidt 1997 A	29	122	416	694	32.4%	0.21 [0.13, 0.32]
**Total (95% CI)**		**698**		**1482**	**100.0%**	**0.59 [0.24, 1.44]**
Total events	307		801			

Heterogeneity: Tau² = 0.58; Chi² = 38.91, df = 2 (P < 0.00001); I² = 95%
Test for overall effect: Z = 1.16 (P = 0.25)

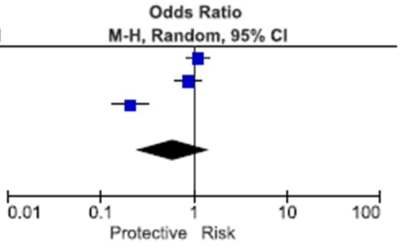

AGT

Supplementary Figure S2l: *AGT* rs699 investigation, individuals with type 1 diabetic nephropathy compared to individuals with type 1 diabetes mellitus in a European Population (T allele compared to C)

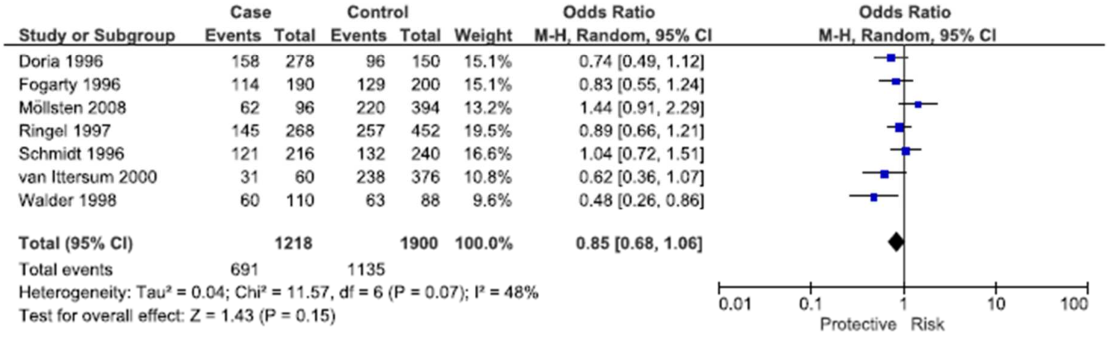

Supplementary Figure S2m: *AGT* rs699 investigation, individuals with type 2 diabetic nephropathy compared to individuals with type 2 diabetes mellitus in an East Asian Population (T allele compared to C)

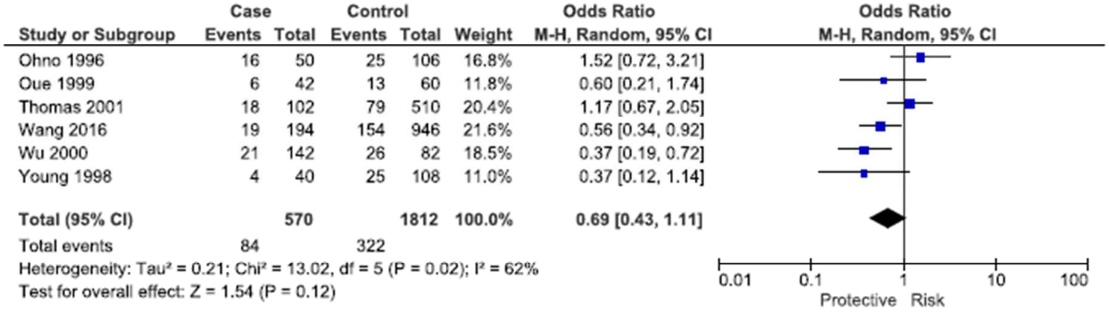

Supplementary Figure S2n: *AGT* rs699 investigation, individuals with type 2 diabetic nephropathy compared to individuals with type 2 diabetes mellitus in a European Population (T allele compared to C)

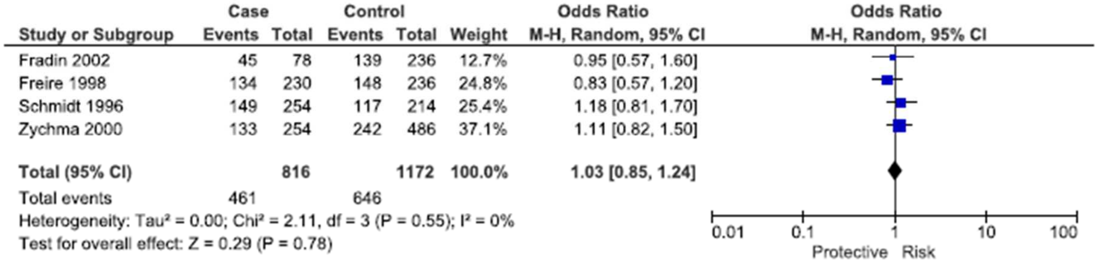

Supplementary Figure S2o: *AGT* rs699 investigation, individuals with type 2 diabetic nephropathy compared to individuals with type 2 diabetes mellitus in a Middle Eastern Population (T allele compared to C)

Study or Subgroup	Case Events	Case Total	Control Events	Control Total	Weight	Odds Ratio M-H, Random, 95% CI
Eroglu 2008	48	92	62	112	25.1%	0.88 [0.51, 1.53]
Reis 2011	114	216	89	222	36.0%	1.67 [1.14, 2.44]
Sancakdar 2015	189	388	95	200	38.8%	1.05 [0.75, 1.48]
**Total (95% CI)**		**696**		**534**	**100.0%**	**1.19 [0.82, 1.71]**
Total events	351		246			

Heterogeneity: Tau² = 0.06; Chi² = 4.72, df = 2 (P = 0.09); I² = 58%
Test for overall effect: Z = 0.91 (P = 0.36)

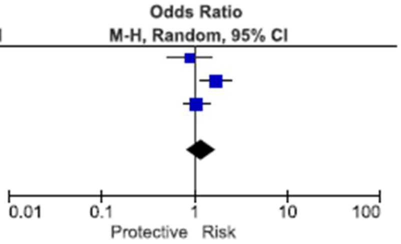

Supplementary Figure S2p: *AGT* rs699 investigation, individuals with IgA nephropathy compared to healthy controls in an East Asian Population (T allele compared to C)

Study or Subgroup	Case Events	Case Total	Control Events	Control Total	Weight	Odds Ratio M-H, Random, 95% CI
Gao 2015	144	700	125	620	41.2%	1.03 [0.78, 1.34]
Guo 2016	19	90	16	90	5.4%	1.24 [0.59, 2.60]
Huang 2010	35	260	40	240	12.3%	0.78 [0.48, 1.27]
Kim 2009	89	476	115	600	31.5%	0.97 [0.71, 1.32]
Lau 2004	27	236	31	188	9.6%	0.65 [0.38, 1.14]
**Total (95% CI)**		**1762**		**1738**	**100.0%**	**0.94 [0.79, 1.12]**
Total events	314		327			

Heterogeneity: Tau² = 0.00; Chi² = 3.18, df = 4 (P = 0.53); I² = 0%
Test for overall effect: Z = 0.67 (P = 0.50)

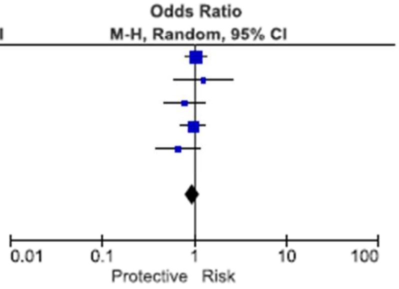

Supplementary Figure S2q: *AGT* rs699 investigation, individuals with end-stage renal disease compared to healthy controls in an East Asian Population (T allele compared to C)

Study or Subgroup	Case Events	Case Total	Control Events	Control Total	Weight	Odds Ratio M-H, Random, 95% CI
Kawada 1997	91	424	64	404	30.9%	1.45 [1.02, 2.07]
Su 2014	193	1294	230	1288	38.7%	0.81 [0.65, 0.99]
Wang 2003	76	492	64	366	30.4%	0.86 [0.60, 1.24]
**Total (95% CI)**		**2210**		**2058**	**100.0%**	**0.99 [0.69, 1.41]**
Total events	360		358			

Heterogeneity: Tau² = 0.07; Chi² = 8.04, df = 2 (P = 0.02); I² = 75%
Test for overall effect: Z = 0.07 (P = 0.94)

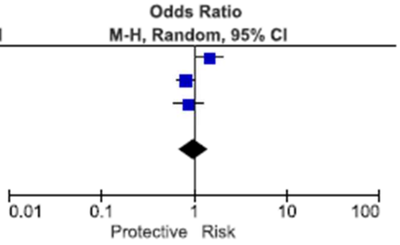

AGTR1

Supplementary Figure S2r: *AGTR1* rs5186 investigation, individuals with type 1 diabetic nephropathy compared to individuals with type 1 diabetes mellitus in a European Population (A allele compared to C allele)

Study or Subgroup	Case Events	Case Total	Control Events	Control Total	Weight	Odds Ratio M-H, Random, 95% CI
Currie 2010	1029	1414	1052	1470	33.2%	1.06 [0.90, 1.25]
Doria 1997	99	146	119	158	12.2%	0.69 [0.42, 1.14]
Möllsten 2008	78	96	290	394	10.4%	1.55 [0.89, 2.72]
Savage 1999	136	190	137	194	14.5%	1.05 [0.67, 1.63]
Tarnow 1996	287	396	274	380	21.4%	1.02 [0.74, 1.40]
van Ittersum 2000	47	60	238	376	8.3%	2.10 [1.10, 4.01]
**Total (95% CI)**		**2302**		**2972**	**100.0%**	**1.10 [0.89, 1.35]**
Total events	1676		2110			

Heterogeneity: Tau^2 = 0.03; Chi^2 = 8.89, df = 5 (P = 0.11); I^2 = 44%
Test for overall effect: Z = 0.87 (P = 0.38)

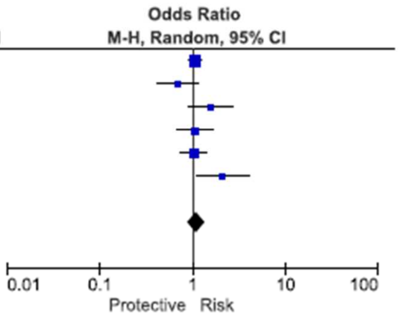

Supplementary Figure S2s: *AGTR1* rs5186 investigation, individuals with type 2 diabetic nephropathy compared to individuals with type 2 diabetes mellitus in an East Asian Population (A allele compared to C allele)

Study or Subgroup	Case Events	Case Total	Control Events	Control Total	Weight	Odds Ratio M-H, Random, 95% CI
Thomas 2001	99	102	483	510	30.2%	1.84 [0.55, 6.20]
Wu 2000	127	142	74	82	54.2%	0.92 [0.37, 2.26]
Young 1998	38	40	103	108	15.7%	0.92 [0.17, 4.96]
**Total (95% CI)**		**284**		**700**	**100.0%**	**1.13 [0.58, 2.20]**
Total events	264		660			

Heterogeneity: Tau^2 = 0.00; Chi^2 = 0.90, df = 2 (P = 0.64); I^2 = 0%
Test for overall effect: Z = 0.37 (P = 0.71)

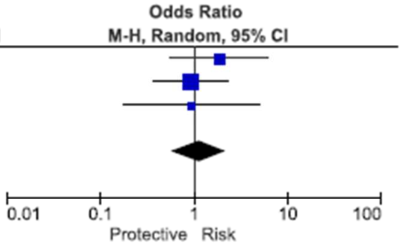

Supplementary Figure S2t: *AGTR1* rs5186 investigation, individuals with IgA nephropathy compared to healthy controls in an East Asian Population (A allele compared to C allele)

Study or Subgroup	Case Events	Case Total	Control Events	Control Total	Weight	Odds Ratio M-H, Random, 95% CI
Gao 2015	659	702	577	620	45.1%	1.14 [0.74, 1.77]
Huang 2010	243	260	220	240	19.1%	1.30 [0.66, 2.54]
Kim 2009	452	476	576	600	25.7%	0.78 [0.44, 1.40]
Lau 2004	227	236	178	188	10.1%	1.42 [0.56, 3.56]
**Total (95% CI)**		**1674**		**1648**	**100.0%**	**1.09 [0.81, 1.46]**
Total events	1581		1551			

Heterogeneity: Tau^2 = 0.00; Chi^2 = 1.85, df = 3 (P = 0.60); I^2 = 0%
Test for overall effect: Z = 0.55 (P = 0.58)

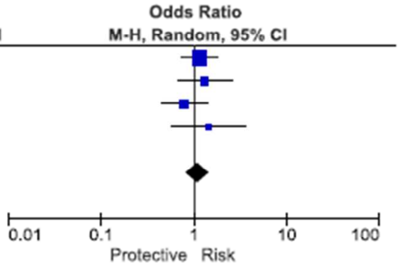

Supplementary Figure S3: Funnel Plots

***ACE* I/D investigation, individuals with type 2 diabetic nephropathy compared to individuals with type 2 diabetes mellitus in an East Asian Population (insertion compared to deletion)**

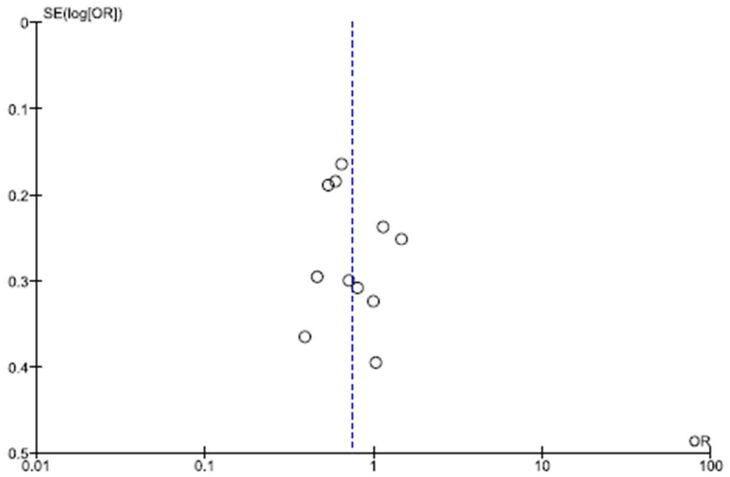

***ACE* I/D investigation, individuals with type 2 diabetic nephropathy compared to individuals with type 2 diabetes mellitus in a South Asian Population (insertion compared to deletion)**

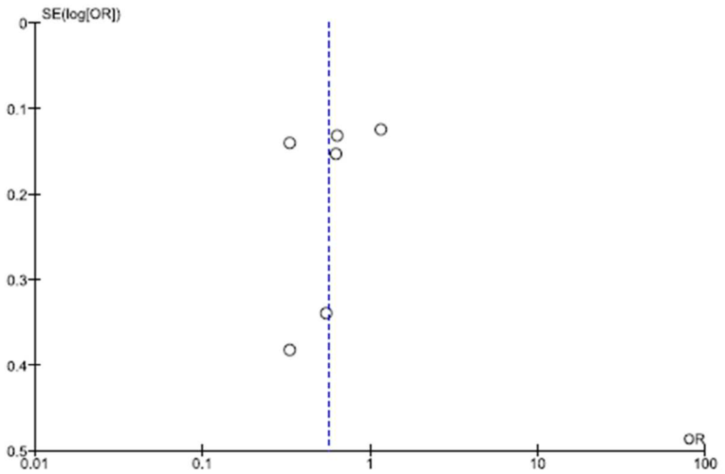

ACE **I/D investigation, individuals with end-stage renal disease compared to healthy controls in an East Asian Population (insertion compared to deletion)**

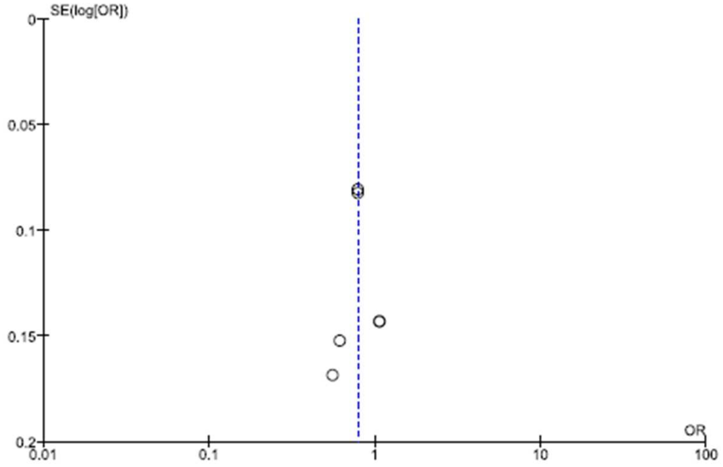

ACE **I/D investigation, individuals with type 1 diabetic nephropathy compared to individuals with type 1 diabetes mellitus in a European Population (insertion compared to deletion)**

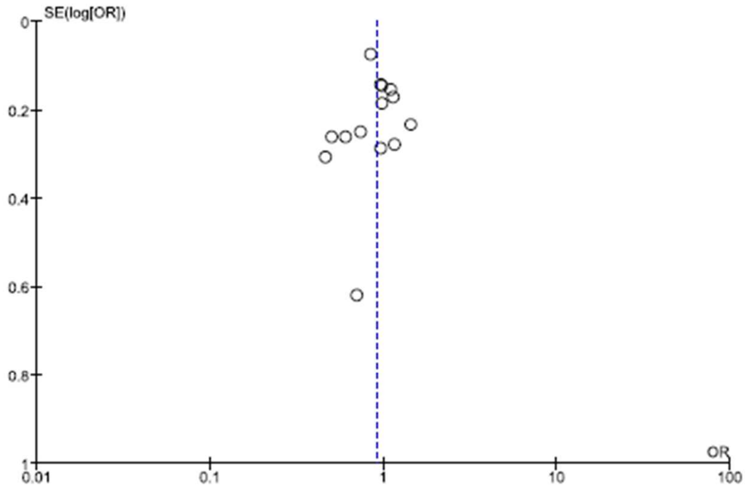

ACE **I/D investigation, individuals with type 2 diabetic nephropathy compared to individuals with type 2 diabetes mellitus in a European Population (insertion compared to deletion)**

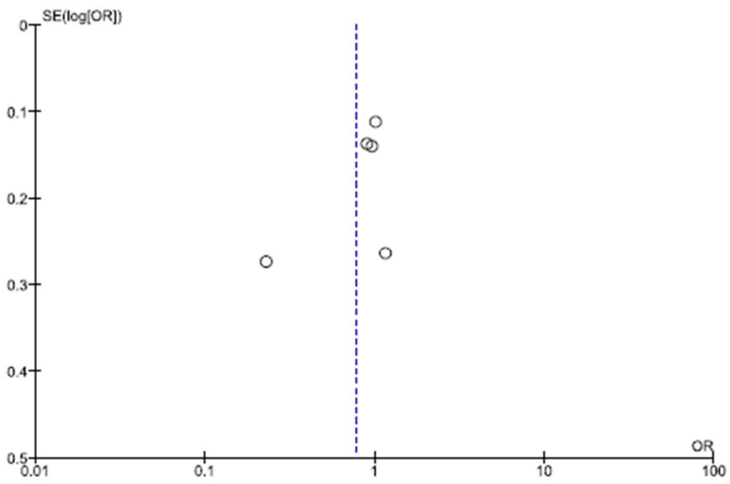

ACE **I/D investigation, individuals with type 2 diabetic nephropathy compared to individuals with type 2 diabetes mellitus in a Middle Eastern Population (insertion compared to deletion)**

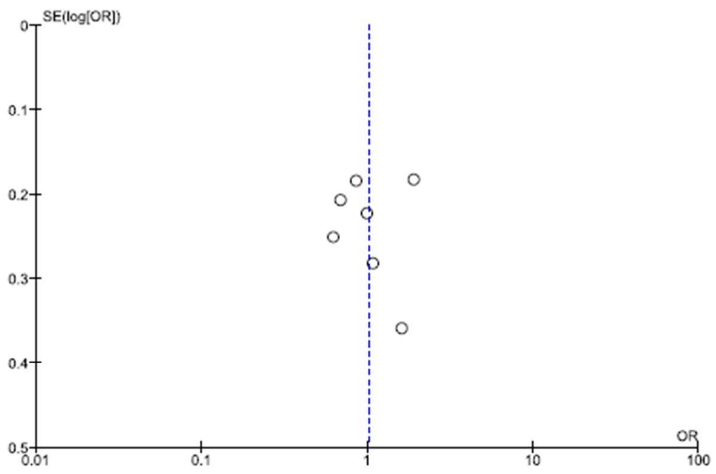

***ACE* I/D investigation, individuals with IgA nephropathy compared to healthy controls in an East Asian Population (insertion compared to deletion)**

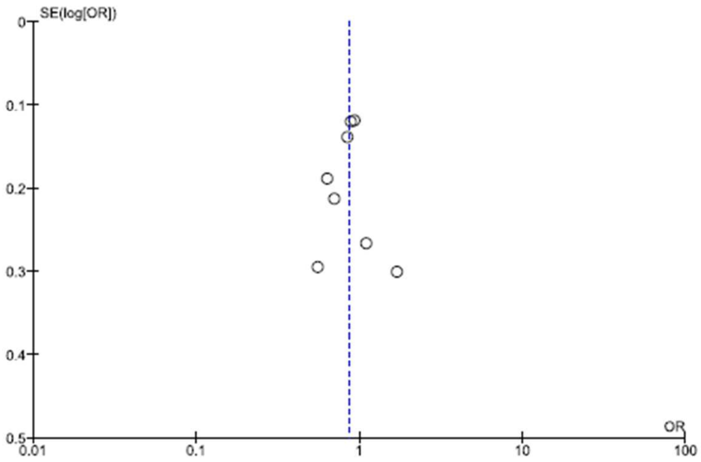

***ACE* I/D investigation, individuals with IgA nephropathy compared to healthy controls in a European Population (insertion compared to deletion)**

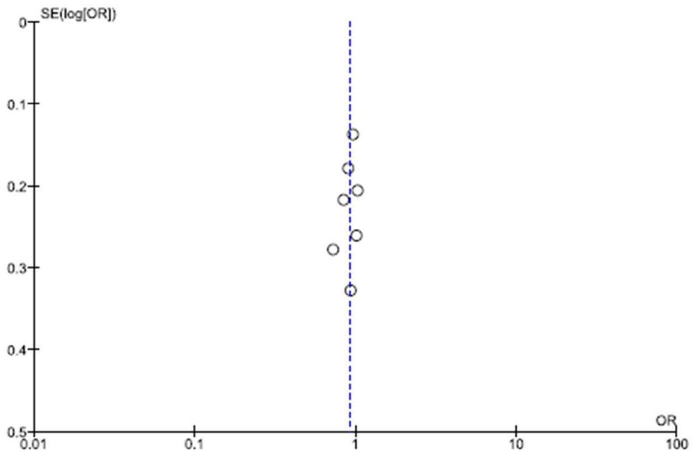

***ACE* I/D investigation, individuals with chronic kidney disease compared to healthy controls in a South Asian Population (insertion compared to deletion)**

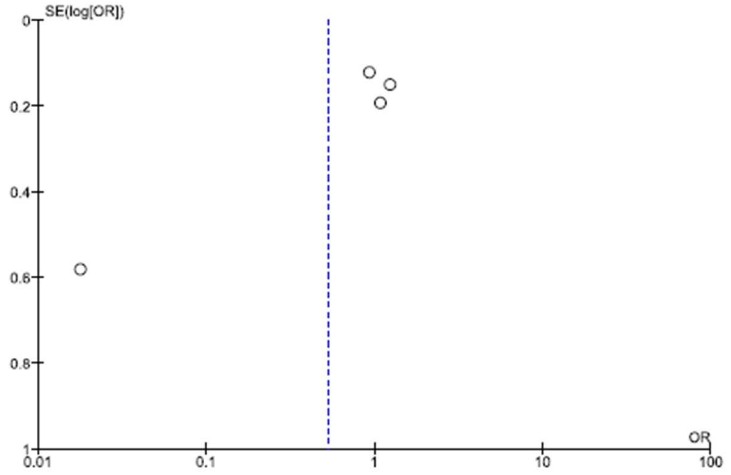

***ACE* I/D investigation, individuals with end-stage renal disease compared to healthy controls in a European Population (insertion compared to deletion)**

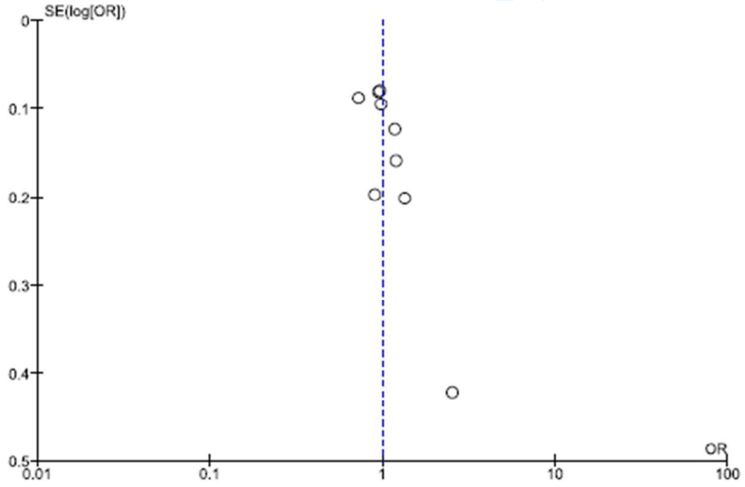

For peer review only

***ACE* I/D investigation, individuals with primary glomerulonephritis compared to healthy controls in a European Population (insertion compared to deletion)**

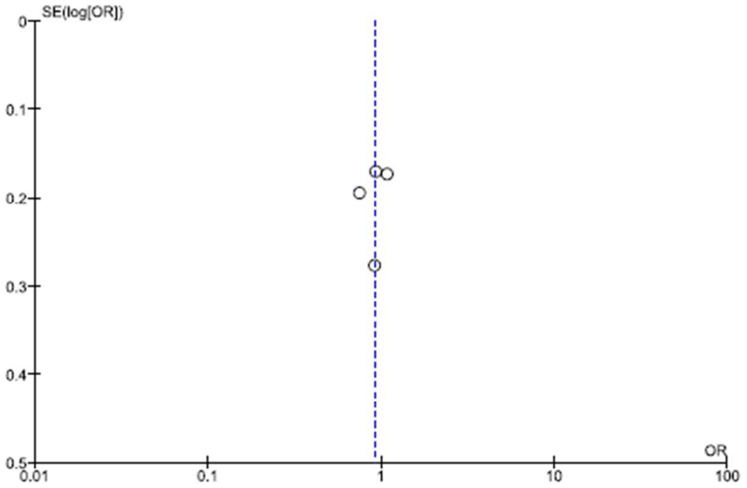

***ACE* I/D investigation, individuals who have had renal transplants compared to healthy controls in a European Population (insertion compared to deletion)**

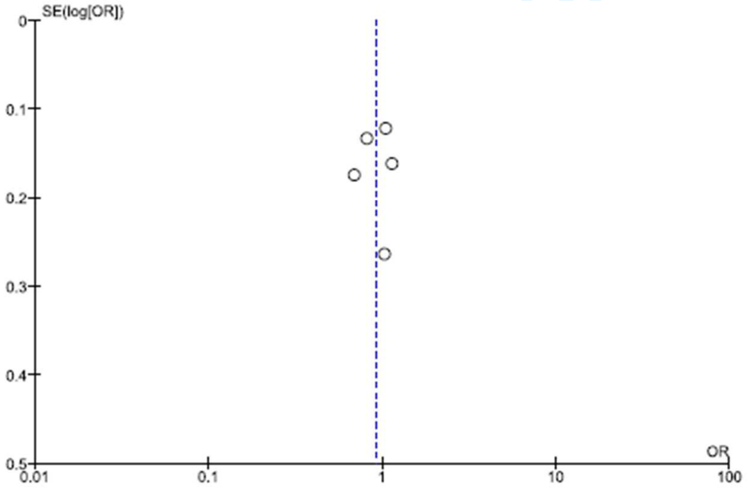

ACE **I/D investigation, individuals with end-stage renal disease linked to type 2 diabetic nephropathy compared to individuals with type 2 diabetes mellitus in an East Asian Population (insertion compared to deletion)**

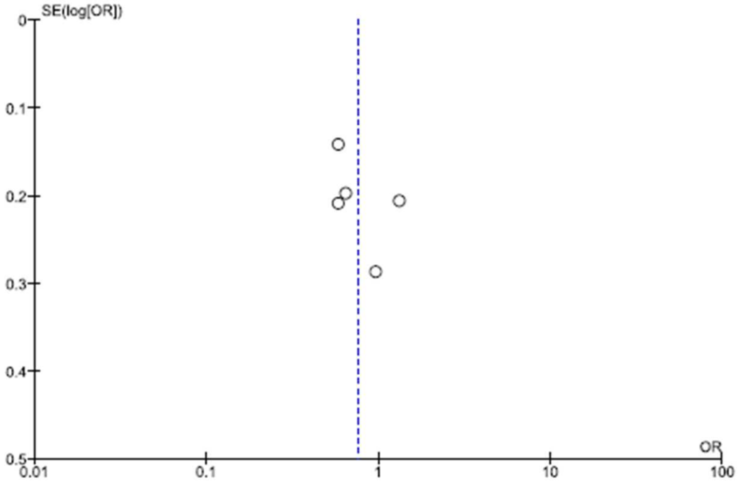

ACE **I/D investigation, individuals with end-stage renal disease linked to type 2 diabetic nephropathy compared to individuals with type 2 diabetes mellitus in a European Population (insertion compared to deletion)**

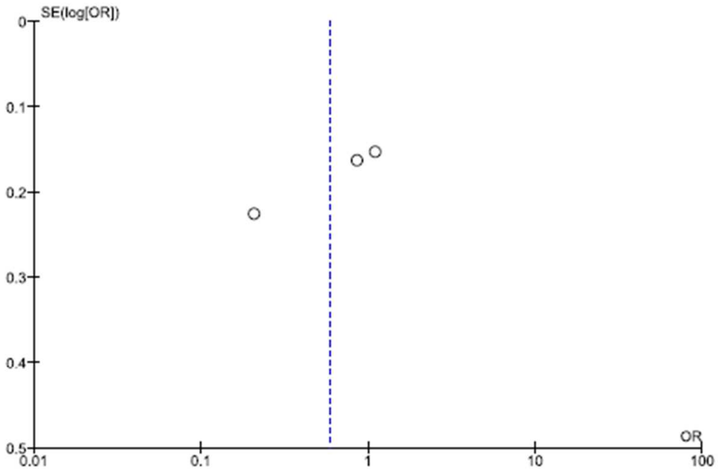

***AGT* rs699 investigation, individuals with end-stage renal disease compared to healthy controls in a European Population (T allele compared to C)**

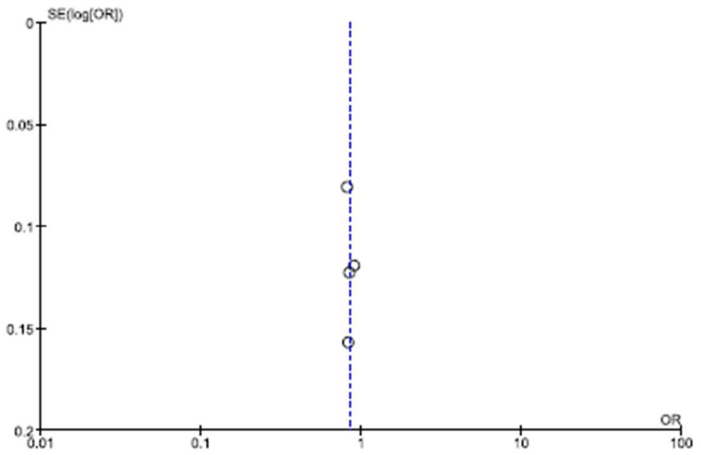

***AGT* rs699 investigation, individuals with type 1 diabetic nephropathy compared to individuals with type 1 diabetes mellitus in a European Population (T allele compared to C)**

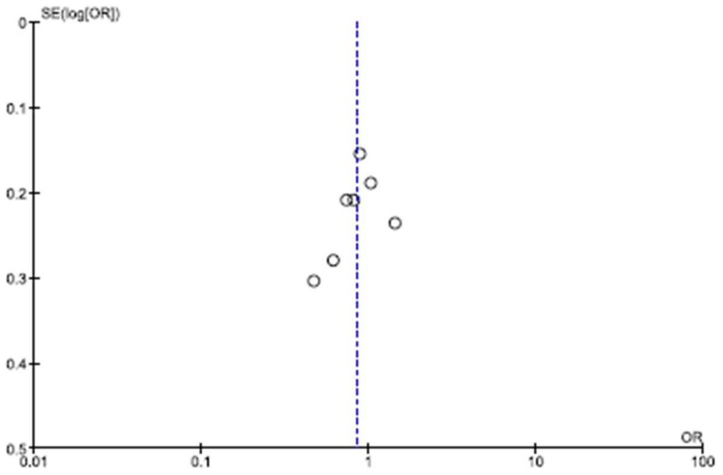

AGT **rs699 investigation, individuals with type 2 diabetic nephropathy compared to individuals with type 2 diabetes mellitus in an East Asian Population (T allele compared to C)**

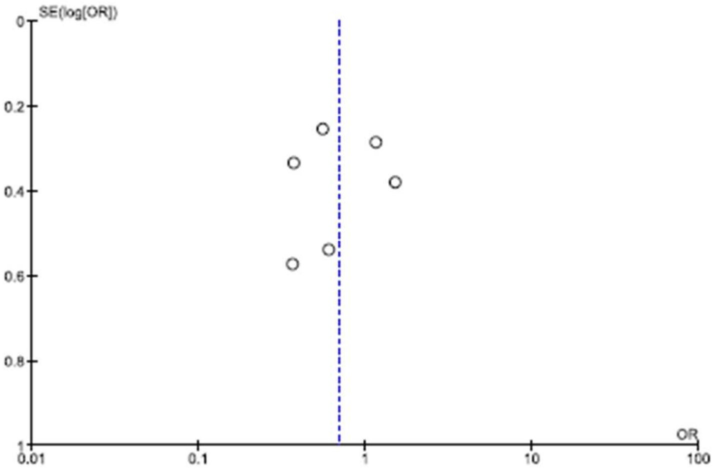

AGT **rs699 investigation, individuals with type 2 diabetic nephropathy compared to individuals with type 2 diabetes mellitus in a European Population (T allele compared to C)**

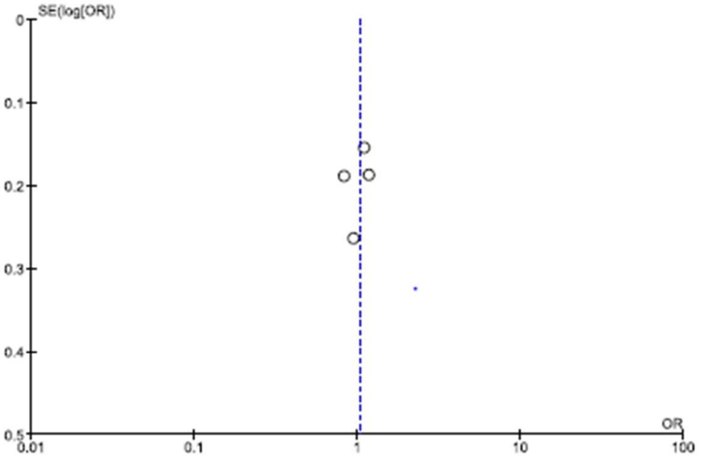

***AGT* rs699 investigation, individuals with type 2 diabetic nephropathy compared to individuals with type 2 diabetes mellitus in a Middle Eastern Population (T allele compared to C)**

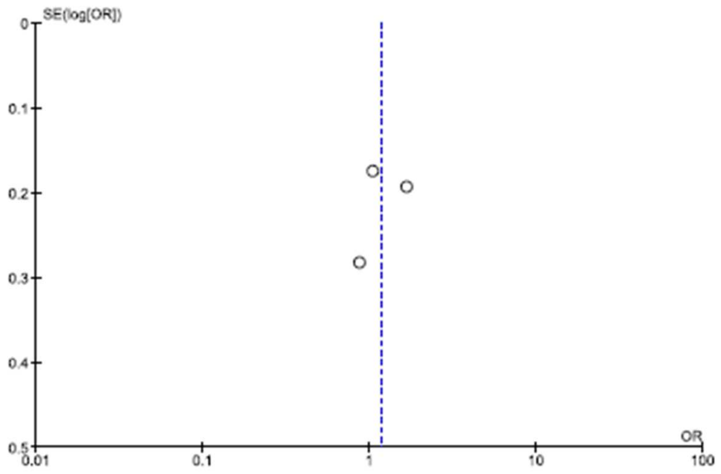

***AGT* rs699 investigation, individuals with IgA nephropathy compared to healthy controls in an East Asian Population (T allele compared to C)**

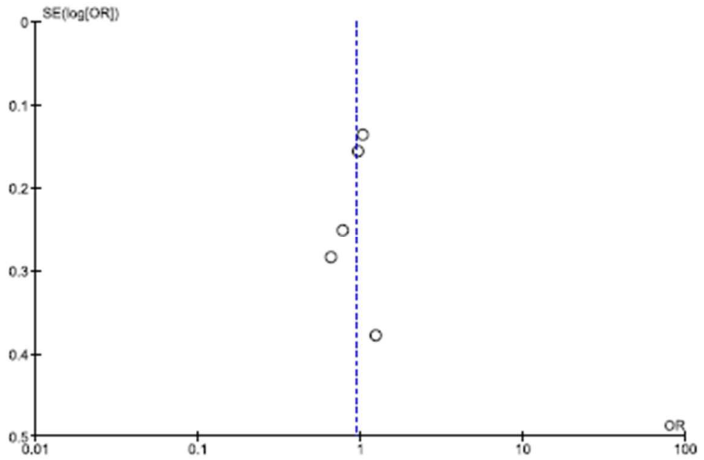

***AGT* rs699 investigation, individuals with end-stage renal disease compared to healthy controls in an East Asian Population (T allele compared to C)**

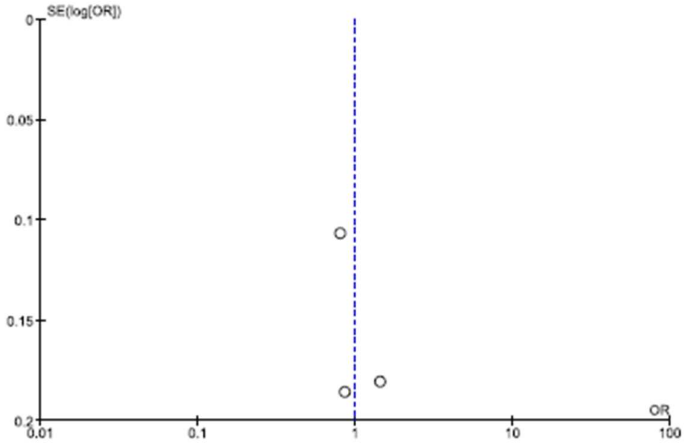

AGTR1 **rs5186 investigation, individuals with type 2 diabetic nephropathy compared to individuals with type 2 diabetes mellitus in a South Asian Population (A allele compared to C allele)**

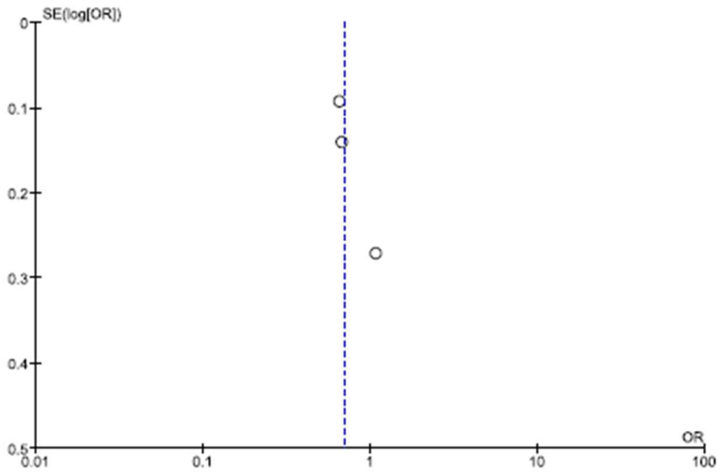

AGTR1 **rs5186 investigation, individuals with type 1 diabetic nephropathy compared to individuals with type 1 diabetes mellitus in a European Population (A allele compared to C allele)**

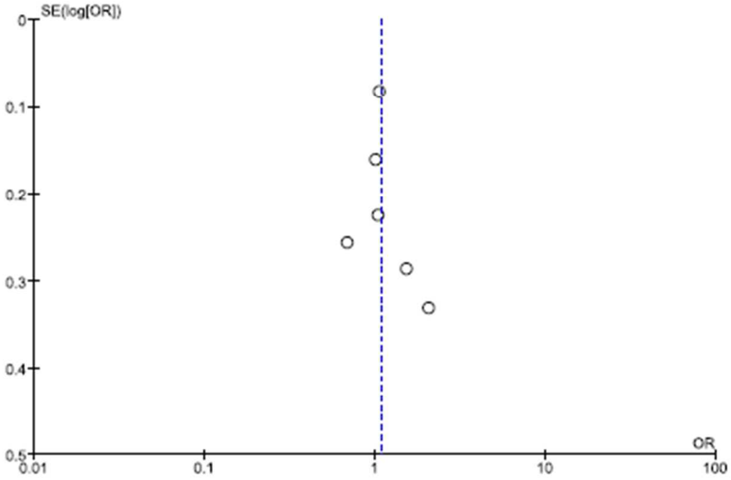

For peer review only

***AGTR1* rs5186 investigation, individuals with type 2 diabetic nephropathy compared to individuals with type 2 diabetes mellitus in an East Asian Population (A allele compared to C allele)**

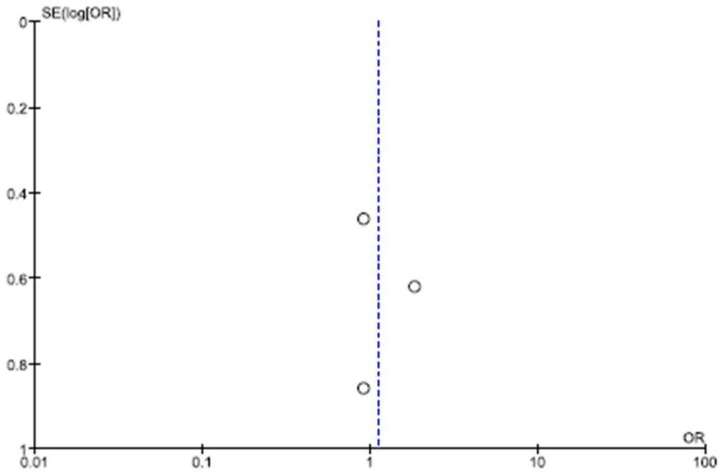

***AGTR1* rs5186 investigation, individuals with IgA nephropathy compared to healthy controls in an East Asian Population (A allele compared to C allele)**

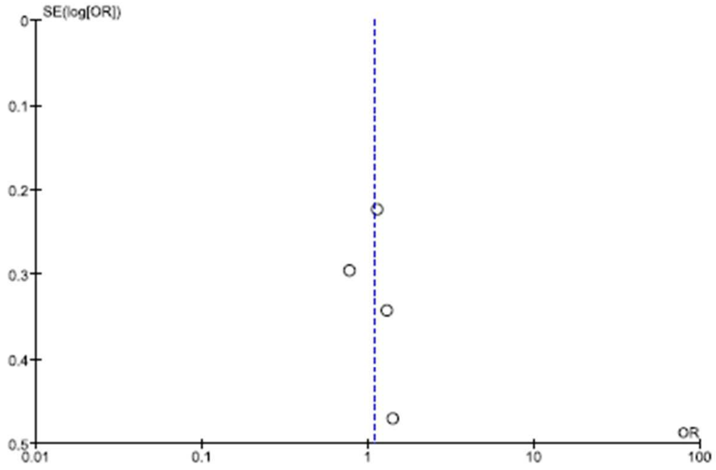

peer review only

Supplementary Table S1: Search Terms

Gene	**Search Terms**
ACE	Angiotensin I Converting Enzyme<br>Angiotensin I Converting Enzyme (Peptidyl-Dipeptidase A) 1<br>Dipeptidyl Carboxypeptidase I<br>CD143 Antigen<br>Kininase II<br>DCP1<br>DCP<br>Angiotensin I Converting Enzyme Peptidyl-Dipeptidase A 1 Transcript<br>Angiotensin Converting Enzyme, Somatic Isoform<br>Peptidyl-Dipeptidase A<br>Carboxycathepsin<br>Testicular ECA<br>Peptidase P<br>EC 3.4.15.1<br>EC 3.2.1.-<br>CD143<br>MVCD3<br>ACE1<br>ACE<br>ICH<br><br><u>AND</u><br><br>Kidney<br>Nephrology<br>Nephropathy<br>Renal<br><br><u>AND</u><br><br>SNP<br>Polymorphism<br>Variant<br>Allele<br>Genotype
ACE2	Angiotensin I Converting Enzyme 2<br>Angiotensin I Converting Enzyme (Peptidyl-Dipeptidase A) 2<br>Angiotensin-Converting Enzyme Homolog<br>ACE-Related Carboxypeptidase<br>Metalloprotease MPROT15<br>Peptidyl-Dipeptidase A<br>ACEH<br>EC 3.4.17.23<br>EC 3.4.17<br>ACE2<br><br><u>AND</u>

For peer review only

	Kidney<br>Nephrology<br>Nephropathy<br>Renal<br><br>AND<br><br>SNP<br>Polymorphism<br>Variant<br>Allele<br>Genotype
AGT	Angiotensinogen<br>Angiotensinogen (Serpin Peptidase Inhibitor, Clade A, Member 8)<br>Serpin Peptidase Inhibitor, Clade A, Member 8<br>Serpin A8<br>SERPINA8<br>Serine (Or Cysteine) Proteinase Inhibitor<br>Alpha-1 Antiproteinase, Antitrypsin<br>Alpha-1 Antiproteinase<br>Pre-Angiotensinogen<br>Angiotensin II<br>Angiotensin I<br>Antitrypsin<br>ANHU<br>AGT<br><br>AND<br><br>Kidney<br>Nephrology<br>Nephropathy<br>Renal<br><br>AND<br><br>SNP<br>Polymorphism<br>Variant<br>Allele<br>Genotype
AGTR1	Angiotensin II Receptor Type 1<br>Angiotensin II Receptor, Type 1<br>AGTR1B<br>AT1AR<br>AT1BR<br>AT2R1<br>AT1<br>Type-1B Angiotensin II Receptor<br>Angiotensin II Type-1 Receptor<br>Angiotensin Receptor 1B

For peer review only

	AGTR1A<br>AT2R1B<br>HAT1R<br>AG2S<br>AT1B<br>AT1R<br>AGTR1<br>AT2R1A<br><br>AND<br><br>Kidney<br>Nephrology<br>Nephropathy<br>Renal<br><br>AND<br><br>SNP<br>Polymorphism<br>Variant<br>Allele<br>Genotype
AGTR2	Angiotensin II Receptor Type 2<br>Angiotensin II Receptor, Type 2<br>Angiotensin II Type-2 Receptor<br>AT2<br>Angiotensin Receptor 2<br>ATGR2<br>MRX88<br><br>AND<br><br>Kidney<br>Nephrology<br>Nephropathy<br>Renal<br><br>AND<br><br>SNP<br>Polymorphism<br>Variant<br>Allele<br>Genotype
REN	Renin<br>Angiotensinogenase<br>EC 3.4.23.15<br>Angiotensin-Forming Enzyme<br>Renin Precursor, Renal<br>EC 3.4.23

	HNFJ2<br>REN<br><br><u>AND</u><br><br>Kidney<br>Nephrology<br>Nephropathy<br>Renal<br><br><u>AND</u><br><br>SNP<br>Polymorphism<br>Variant<br>Allele<br>Genotype
Additional filters added to database searches	English / Spanish language<br>Human studies<br>Case / Control studies<br>Not clinical trials<br>Not review articles<br>Not a case report<br>Not a meta-analysis

Supplementary Table S2: Ethnicity Codes

**Population**	**Population Code**
Ad Mixed American (Southern USA and Central America)	AMR
African American	AFR
East Asian	EAS
European	EUR
Middle Eastern	ME
Mix of ethnicities	MIX
North African	NA
South Asian	SAS

For peer review only

Supplementary Table S3a: Excluded studies from the ACE search

Included in accompanying MS Excel Workbook

*Exclusion stage relates to the stage at which the article was removed as shown in the flow diagram (Supplementary Figure S1a)

Supplementary Table S3b: Excluded studies from the ACE2 search

Included in accompanying MS Excel Workbook

*Exclusion stage relates to the stage at which the article was removed as shown in the flow diagram (Supplementary Figure S1b)

Supplementary Table S3c: Excluded studies from the AGT search

Included in accompanying MS Excel Workbook

*Exclusion stage relates to the stage at which the article was removed as shown in the flow diagram (Supplementary Figure S1c)

Supplementary Table S3d: Excluded studies from the AGTR1 search

Included in accompanying MS Excel Workbook

*Exclusion stage relates to the stage at which the article was removed as shown in the flow diagram (Supplementary Figure S1d)

Supplementary Table S3e: Excluded studies from the AGTR2 search

Included in accompanying MS Excel Workbook

*Exclusion stage relates to the stage at which the article was removed as shown in the flow diagram (Supplementary Figure S1e)

Supplementary Table S3f: Excluded studies from the REN search

Included in accompanying MS Excel Workbook

*Exclusion stage relates to the stage at which the article was removed as shown in the flow diagram (Supplementary Figure S1f)

For peer review only

Supplementary Table S4: Data included in quantitative analysis for *ACE*

				Cases				Controls			
Author	Year	Ethnicity	SNP	Definition	N	I Frequency	D Frequency	Definition	N	I Frequency	D Frequency
Anbazhagan K et al.(55)	2009	SAS	Ins/Del	CKD	118	124	112	Healthy	98	99	97
Chen WJ et al.(56)	2014	SAS	Ins/Del	CKD	233	314	152	Healthy	449	617	281
Nagamani S et al.(57)	2015	SAS	Ins/Del	CKD	147	152	142	Healthy	221	195	227
Shanmuganathan R et al.(58)	2015	SAS	Ins/Del	CKD	30	10	50	Healthy	30	55	5
Ali A et al.(59)	2015	EAS	Ins/Del	ESRD	190	219	161	Healthy	190	262	118
Kawada N et al.(22)	1997	EAS	Ins/Del	ESRD	216	280	152	Healthy	208	264	152
Su SL et al.(60)	2014	EAS	Ins/Del	ESRD	647	792	502	Healthy	644	859	429
Tang FY et al.(61)	2008	EAS	Ins/Del	ESRD	153	100	206	Healthy	148	138	158
Wang AY et al.(62)	2003	EAS	Ins/Del	ESRD	246	316	176	Healthy	183	230	136
Yang HY et al.(63)	2015	EAS	Ins/Del	ESRD	683	833	533	Healthy	653	867	439
Buraczynska M et al.(64)	2006	EUR	Ins/Del	ESRD	745	688	802	Healthy	520	492	548
Dixit M et al.(65)	2002	EUR	Ins/Del	ESRD	26	32	20	Healthy	22	17	27
Losito A et al.(66)	2002	EUR	Ins/Del	ESRD	160	135	185	Healthy	169	128	210
McLaughlin KJ et al.(67)	1996	EUR	Ins/Del	ESRD	460	654	990	Healthy	371	353	389
Nicod J et al.(68)	2002	EUR	Ins/Del	ESRD	260	282	238	Healthy	260	261	259
Ortiz MA et al.(69)	2003	EUR	Ins/Del	ESRD	117	68	166	Healthy	129	80	178
Schmidt A et al.(70)	1996	EUR	Ins/Del	ESRD	106	105	107	Healthy	95	80	110
van der Sman-de Beer F et al.(71)	2005	EUR	Ins/Del	ESRD	415	400	430	Healthy	472	459	485
Aucella F et al.(72)	2003	EUR	Ins/Del	ESRD	461	315	607	Healthy	1307	916	1698
Guo Y et al.(73)	2016	EAS	Ins/Del	IgAN	45	54	36	Healthy	45	42	48
Huang HD et al.(50)	2010	EAS	Ins/Del	IgAN	130	155	105	Healthy	120	168	72
Jung ES et al.(74)	2010	EAS	Ins/Del	IgAN	261	298	224	Healthy	300	360	240
Lau YK et al.(25)	2004	EAS	Ins/Del	IgAN	118	154	82	Healthy	94	137	51
Suzuki H et al.(75)	2004	EAS	Ins/Del	IgAN	319	378	260	Healthy	270	328	212
Yoon HJ et al.(76)	2002	EAS	Ins/Del	IgAN	191	204	178	Healthy	233	268	198

For peer review only

Yorioka T et al.(77)	1995	EAS	Ins/Del	IgAN	48	67	29	Healthy	104	139	67
Yoshida H et al.(78)	1995	EAS	Ins/Del	IgAN	53	57	49	Healthy	46	62	30
Burg M et al. *(79)	1997	EUR	Ins/Del	IgAN	70	26	28	Healthy	60	60	60
Drouet M et al.(80)	2002	EUR	Ins/Del	IgAN	125	78	172	Healthy	83	54	100
Harden PN et al.(81)	1995	EUR	Ins/Del	IgAN	100	79	121	Healthy	98	76	120
Pawlik M et al. *(82)	2014	EUR	Ins/Del	IgAN	31	25	37	Healthy	187	180	194
Pei Y et al.(83)	1997	EUR	Ins/Del	IgAN	168	145	191	Healthy	100	91	109
Schmidt S et al. (B)(84)	1995	EUR	Ins/Del	IgAN	204	168	240	Healthy	234	197	271
Stratta P et al.(85)	1999	EUR	Ins/Del	IgAN	81	62	100	Healthy	50	38	62
Burg M et al. *(79)	1997	EUR	Ins/Del	Primary GN	46	44	48	Healthy	60	60	60
Pawlik M et al. *(82)	2014	EUR	Ins/Del	Primary GN	109	101	117	Healthy	187	180	194
Stratta P et al.(86)	2004	EUR	Ins/Del	Primary GN	117	96	138	Organ donors	171	134	208
Zsom M et al.(87)	2011	EUR	Ins/Del	Primary GN	73	62	84	Healthy	200	198	202
Beige J et al.(88)	1997	EUR	Ins/Del	RTx recipients	269	251	287	Kidney Donor	269	244	294
El-Essawy AB et al.(89)	2002	EUR	Ins/Del	RTx recipients	294	246	342	Healthy	181	170	192
Hueso M et al.(90)	2004	EUR	Ins/Del	RTx recipients	180	122	238	Healthy & cadaveric renal allograft donors	113	96	130
Stratta P et al. (91)	2008	EUR	Ins/Del	RTx recipients	169	122	216	Healthy	169	112	226
Viklický O et al.(92)	2001	EUR	Ins/Del	RTx recipients	30	28	32	Healthy	653	599	707
Barnas U et al.(93)	1997	EUR	Ins/Del	T1DN	63	45	55	T1DM	59	51	29
Chowdhury TA et al.(94)	1996	EUR	Ins/Del	T1DN	242	204	280	T1DM	166	143	189
Currie D et al.(95)	2010	EUR	Ins/Del	T1DN	718	603	777	T1DM	749	696	764
De Cosmo S et al.(96)	1999	EUR	Ins/Del	T1DN	175	125	225	T1DM	136	89	183
Demurov LM et al.(97)	1997	EUR	Ins/Del	T1DN	56	35	77	T1DM	76	72	80

For peer review only

Hadjadj S et al.(98)	2001	EUR	Ins/Del	T1DN	6	4	8	T1DM	251	208	294
Hibberd ML et al.(99)	1997	EUR	Ins/Del	T1DN	72	60	84	T1DM	86	57	115
Marre M et al.(34)	1994	EUR	Ins/Del	T1DN	62	43	81	T1DM	62	58	66
Ringel J et al. *(100)	1997	EUR	Ins/Del	T1DN	134	130	138	T1DM	226	208	244
Schmidt S et al. (A)(101)	1995	EUR	Ins/Del	T1DN	114	86	142	T1DM	133	101	165
Shestakova MV et al.(102)	2006	EUR	Ins/Del	T1DN	63	65	61	T1DM	66	78	54
Tarnow L et al.(103)	1995	EUR	Ins/Del	T1DN	198	175	221	T1DM	190	169	211
van Ittersum FJ et al.(53)	2000	EUR	Ins/Del	T1DN	30	33	27	T1DM	188	192	184
Walder B et al.(104)	1998	EUR	Ins/Del	T1DN	55	49	61	T1DM	44	40	48
Fujisawa T et al.(105)	1995	EAS	Ins/Del	T2DN	54	71	37	T2DM	35	46	24
Ha SK et al.(106)	2003	EAS	Ins/Del	T2DN	140	132	148	T2DM	99	123	75
Hsieh MC et al.(107)	2000	EAS	Ins/Del	T2DN	179	219	139	T2DM	157	222	92
Mizuiri S et al.(108)	1995	EAS	Ins/Del	T2DN	80	72	88	T2DM	31	33	29
Nakajima S et al.(109)	1996	EAS	Ins/Del	T2DN	54	67	41	T2DM	41	55	27
Ohno T et al.(110)	1996	EAS	Ins/Del	T2DN	25	28	22	T2DM	53	81	25
Shin Y et al.(111)	2004	EAS	Ins/Del	T2DN	82	112	52	T2DM	59	70	48
Thomas GN et al.(112)	2001	EAS	Ins/Del	T2DN	51	72	30	T2DM	255	346	164
Wang M et al.(113)	2016	EAS	Ins/Del	T2DN	106	78	56	T2DM	741	1020	438
Wu SH et al.(114)	2000	EAS	Ins/Del	T2DN	71	75	67	T2DM	41	58	24
Young RP et al.(115)	1998	EAS	Ins/Del	T2DN	20	27	13	T2DM	54	72	36
Dudley CR et al.(116)	1995	EUR	Ins/Del	T2DN	163	246	288	T2DM	267	246	288
Fradin S et al.(117)	2002	EUR	Ins/Del	T2DN	39	94	142	T2DM	118	94	142
Ng DPK et al.(118)	2006	EUR	Ins/Del	T2DN	291	147	187	T2DM	167	147	187
Nikzamir A et al.(119)	2009	EUR	Ins/Del	T2DN	48	159	131	T2DM	145	159	131
Schmidt S et al. (B)(120)	1997	EUR	Ins/Del	T2DN	311	278	416	T2DM	347	278	416
Al-Harbi EM et al.(121)	2011	ME	Ins/Del	T2DN	110	63	157	T2DM	196	125	267
Araz M et al.(122)	2001	ME	Ins/Del	T2DN	62	52	72	T2DM	123	103	143
El-Baz R et al.(123)	2012	ME	Ins/Del	T2DN	102	66	138	T2DM	100	82	118
Ergen HA et al.(124)	2004	ME	Ins/Del	T2DN	25	21	29	T2DM	50	31	69
Eroglu Z et al.(125)	2008	ME	Ins/Del	T2DN	46	43	49	T2DM	56	50	62
Felehgari V et al.(126)	2011	ME	Ins/Del	T2DN	68	42	94	T2DM	72	60	84
Sancakdar E et al.(127)	2015	ME	Ins/Del	T2DN	194	180	208	T2DM	100	62	138

For peer review only

Ahluwalia TS et al.(128)	2009	SAS	Ins/Del	T2DN	240	152	328	T2DM	200	215	
Kumar R et al.(129)	2013	SAS	Ins/Del	T2DN	407	448	366	T2DM	185	190	
Movva S et al.(130)	2007	SAS	Ins/Del	T2DN	174	182	166	T2DM	175	222	
Naresh VV et al.(131)	2009	SAS	Ins/Del	T2DN	30	19	41	T2DM	30	35	
Shaikh R et al.(132)	2012	SAS	Ins/Del	T2DN	168	134	202	T2DM	296	394	
Vishwanathan V et al.(133)	2001	SAS	Ins/Del	T2DN	86	79	93	T2DM	23	28	
Doi Y et al.(134)	1996	EAS	Ins/Del	T2DN-ESRD	100	115	85	T2DM	124	168	
Jayapalan JJ et al.(135)	2010	EAS	Ins/Del	T2DN-ESRD	127	163	91	T2DM	81	93	
Lu M et al.(136)	2016	EAS	Ins/Del	T2DN-ESRD	210	238	182	T2DM	222	307	
Park HC et al.(137)	2005	EAS	Ins/Del	T2DN-ESRD	103	103	103	T2DM	88	111	
Taniwaki H et al.(138)	2001	EAS	Ins/Del	T2DN-ESRD	42	53	31	T2DM	69	88	
Grzeszczak W et al.(139)	1998	EUR	Ins/Del	T2DN-ESRD	127	128	126	T2DM	254	244	
Ringel J et al. *(100)	1997	EUR	Ins/Del	T2DN-ESRD	161	150	172	T2DM	140	141	
Schmidt S et al. (A)(140)	1997	EUR	Ins/Del	T2DN-ESRD	61	29	93	T2DM	347	416	

*Burg M et al., Pawlik M et al., and Ringel J et al., are included twice as these articles contained two phenotypic comparisons

Abbreviations: EAS, East Asian; EUR, European; ME, Middle Eastern; SAS, South Asian; CKD, Chronic Kidney Disease; ESRD, End-Stage Renal Disease; IgAN, IgA Nephropathy; GN, Glomerulonephritis; RTx, Renal Transplant; T1DM, Type 1 Diabetes Mellitus; T1DN, Type 1 Diabetic Nephropathy; T2DM, Type 2 Diabetes Mellitus; T2DN, Type 2 Diabetic Nephropathy.

For peer review only

Supplementary Table S5: Data included in quantitative analysis for *AGT*

				Cases				Controls			
**Author**	**Year**	**Ethnicity**	**SNP**	**Definition**	**N**	**T Allele Frequency**	**C Allele Frequency**	**Definition**	**N**	**T Allele Frequency**	**C Allele Frequency**
Beige J et al. (141)	1996	EUR	rs699	ESRD	269	285	253	Healthy	269	307	229
Buraczynska M et al.(64)	2006	EUR	rs699	ESRD	745	734	756	Healthy	520	564	476
Losito A et al.(66)	2002	EUR	rs699	ESRD	160	165	155	Healthy	169	190	148
Lovati E et al.(142)	2001	EUR	rs699	ESRD	260	299	221	Healthy	327	393	261
Doria A et al.(143)	1996	EUR	rs699	T1DN	139	158	120	T1DM	75	96	54
Fogarty DG et al.(144)	1996	EUR	rs699	T1DN	95	114	76	T1DM	100	129	71
Möllsten A et al.(145)	2008	EUR	rs699	T1DN	73	62	34	T1DM	197	220	174
Ringel J et al.(100)	1997	EUR	rs699	T1DN	134	145	123	T1DM	226	257	195
Schmidt S et al.*(146)	1996	EUR	rs699	T1DN	108	121	95	T1DM	120	132	108
van Ittersum FJ et al.(53)	2000	EUR	rs699	T1DN	30	31	29	T1DM	188	238	138
Walder B et al.(104)	1998	EUR	rs699	T1DN	55	60	50	T1DM	44	63	25
Ohno T et al.(110)	1996	EAS	rs699	T2DN	25	16	34	T2DM	53	25	81
Oue T et al.(147)	1999	EAS	rs699	T2DN	27	6	36	T2DM	40	13	47
Thomas GN et al.(112)	2001	EAS	rs699	T2DN	51	18	84	T2DM	255	79	431
Wang M et al.(113)	2016	EAS	rs699	T2DN	189	19	175	T2DM	473	154	792
Wu SH et al.(114)	2000	EAS	rs699	T2DN	71	21	121	T2DM	41	26	56
Young RP et al.(115)	1998	EAS	rs699	T2DN	20	4	36	T2DM	54	25	83
Fradin S et al.(117)	2002	EUR	rs699	T2DN	39	45	33	T2DM	118	139	97
Freire MB et al.(148)	1998	EUR	rs699	T2DN	117	134	96	T2DM	125	148	88
Schmidt S et al.*(146)	1996	EUR	rs699	T2DN	127	149	105	T2DM	107	117	97
Zychma MJ et al.(149)	2000	EUR	rs699	T2DN	127	133	121	T2DM	243	242	244
Eroglu Z et al.(125)	2008	ME	rs699	T2DN	46	48	44	T2DM	56	62	50
Reis KA et al.(150)	2011	ME	rs699	T2DN	108	114	102	T2DM	111	89	133
Sancakdar E et al.(127)	2015	ME	rs699	T2DN	194	189	199	T2DM	100	95	105
Gao J et al.(49)	2015	EAS	rs699	IgAN	351	144	556	Healthy	310	125	495
Guo Y et al.(73)	2016	EAS	rs699	IgAN	45	19	71	Healthy	45	16	74

For peer review only

Huang HD et al.(50)	2010	EAS	rs699	IgAN	130	35	225	Healthy	120	40	200
Kim SM et al.(51)	2009	EAS	rs699	IgAN	238	89	387	Healthy	300	115	485
Lau YK et al.(25)	2004	EAS	rs699	IgAN	118	27	209	Healthy	94	31	157
Kawada N et al.(22)	1997	EAS	rs699	ESRD	216	91	333	Healthy	208	64	340
Su SL et al.(60)	2014	EAS	rs699	ESRD	647	193	1101	Healthy	644	230	1058
Wang AY et al.(62)	2003	EAS	rs699	ESRD	246	76	416	Healthy	183	64	302

*Schmidt S et al., is included twice as the article contained two phenotypic comparisons.

Abbreviations: EAS, East Asian; EUR, European; ME, Middle Eastern; ESRD, End-Stage Renal Disease; IgAN, IgA Nephropathy; T1DM, Type 1 Diabetes Mellitus; T1DN, Type 1 Diabetic Nephropathy; T2DM, Type 2 Diabetes Mellitus; T2DN, Type 2 Diabetic Nephropathy.

For peer review only

Supplementary Table S6: Data included in quantitative analysis for *AGTR1*

				Cases				Controls			
Author	Year	Ethnicity	SNP	Definition	N	A Allele Frequency	C Allele Frequency	Definition	N	A Allele Frequency	C Allele Frequency
Gao J et al.(49)	2015	EAS	rs5186	IgAN	351	659	43	Healthy	310	577	43
Huang HD et al.(50)	2010	EAS	rs5186	IgAN	130	243	17	Healthy	120	220	20
Kim SM et al.(51)	2009	EAS	rs5186	IgAN	238	452	24	Healthy	300	576	24
Lau YK et al.(25)	2004	EAS	rs5186	IgAN	118	227	9	Healthy	94	178	10
Currie D et al.(95)	2010	EUR	rs5186	T1DN	718	1029	385	T1DM	749	1052	418
Doria A et al.(151)	1997	EUR	rs5186	T1DN	73	99	47	T1DM	79	119	39
Möllsten A et al.(145)	2008	EUR	rs5186	T1DN	72	78	18	T1DM	197	290	104
Savage DA et al.(52)	1999	EUR	rs5186	T1DN	95	136	54	T1DM	97	137	57
Tarnow L et al.(152)	1996	EUR	rs5186	T1DN	198	287	109	T1DM	190	274	106
van Ittersum FJ et al.(53)	2000	EUR	rs5186	T1DN	30	47	13	T1DM	188	238	138
Thomas GN et al.(112)	2001	EAS	rs5186	T2DN	51	99	3	T2DM	255	483	27
Wu SH et al.(114)	2000	EAS	rs5186	T2DN	71	127	15	T2DM	41	74	8
Young RP et al.(115)	1998	EAS	rs5186	T2DN	20	38	2	T2DM	54	103	5
Ahluwalia TS et al.(128)	2009	SAS	rs5186	T2DN	240	320	160	T2DM	255	381	129
Prasad P et al.(54)	2006	SAS	rs5186	T2DN	196	360	26	T2DM	225	417	33
Shah VN et al.(153)	2013	SAS	rs5186	T2DN	596	789	403	T2DM	566	840	282

Abbreviations: EAS, East Asian; EUR, European; SAS, South Asian; IgAN, IgA Nephropathy; T1DM, Type 1 Diabetes Mellitus; T1DN, Type 1 Diabetic Nephropathy; T2DM, Type 2 Diabetes Mellitus; T2DN, Type 2 Diabetic Nephropathy.

For peer review only

Supplementary Table S7. Included studies not complying with Hardy Weinberg equilibrium in cases or controls

					Cases		Controls	
**Gene**	**Author**	**Year**	**Ethnicity**	**SNP**	**Definition**	**P value (where significant)**	**Definition**	**P value (where significant)**
ACE	Jung ES	2010	EAS	Ins/Del	IgAN		Healthy	0.016
	Shanmuganathan R	2015	SAS	Ins/Del	CKD	$1.31x10^{-04}$	Healthy	$1.9x10^{-05}$
	Tang FY	2008	EAS	Ins/Del	ESRD	$1.8x10^{-05}$	Healthy	$1x10^{-06}$
	Nicod J	2002	EUR	Ins/Del	ESRD	0.0008	Healthy	0.004
	McLaughlin KJ	1996	EUR	Ins/Del	ESRD	0.04	Healthy	
	Yorioka T	1995	EAS	Ins/Del	IgAN	0.013	Healthy	
	Yoshida H	1995	EAS	Ins/Del	IgAN	0.01	Healthy	
	Yoon HJ	2002	EAS	Ins/Del	IgAN	0.002	Healthy	
	Schmidt S	1995	EUR	Ins/Del	IgAN	0.007	Healthy	
	Suzuki H	2004	EAS	Ins/Del	IgAN	0.036	Healthy	0.004
	Zsom M	2011	EUR	Ins/Del	Primary GN	0.005	Healthy	
	Currie D	2010	EUR	Ins/Del	T1DN		T1DM	0.005
	Ringel J	1997	EUR	Ins/Del	T1DN		T1DM	0.018
	Tarnow L	1995	EUR	Ins/Del	T1DN		T1DM	0.013
	Schmidt S	1995	EUR	Ins/Del	T1DN	0.002	T1DM	
	Mizuiri S	1995	EAS	Ins/Del	T2DN	0.019	T2DM	
	Hsieh MC	2000	EAS	Ins/Del	T2DN	$4.2x10^{-05}$	T2DM	0.004
	Ha SK	2003	EAS	Ins/Del	T2DN		T2DM	0.026
	Schmidt S	1997	EUR	Ins/Del	T2DN	0.0147	T2DM	
	El-Baz R	2012	ME	Ins/Del	T2DN	0.0025	T2DM	$1x10^{-06}$
	Sancakdar E	2015	ME	Ins/Del	T2DN	0.0076	T2DM	0.003
	Kumar R	2013	SAS	Ins/Del	T2DN	0.0016	T2DM	0.0003
	Park HC	2005	EAS	Ins/Del	T2DN-ESRD		T2DM	0.022
AGT	Fogarty DG	1996	EUR	rs699	T1DN		T1DM	0.044
	Wu SH	2000	EAS	rs699	T2DN	0.0008	T2DM	
	Freire MB	1998	EUR	rs699	T2DN	0.022	T2DM	
	Reis KA	2011	ME	rs699	T2DN	0.006	T2DM	

For peer review only

	Fradin S	2002	EUR	rs699	T2DN	0.008	T2DM	
AGTR1	Ahluwalia TS	2009	SAS	rs5186	T2DN		T2DM	0.0002
	Shah VN	2013	SAS	rs5186	T2DN		T2DM	$4.2x10^{-08}$
	Lau YK	2004	EAS	rs5186	IgAN	0.038	Healthy	
	Möllsten A	2008	EUR	rs5186	T1DN	0.029	T1DM	

Abbreviations: EAS, East Asian; EUR, European; ME, Middle Eastern; SAS, South Asian; CKD, Chronic Kidney Disease; ESRD, End-Stage Renal Disease; GN, Glomerulonephritis; IgAN, IgA Nephropathy; T1DM, Type 1 Diabetes Mellitus; T1DN, Type 1 Diabetic Nephropathy; T2DM, Type 2 Diabetes Mellitus; T2DN, Type 2 Diabetic Nephropathy.

For peer review only

Supplementary Table S3a

Exclusion S	Title	Authors	PMID	Reason
1	Angiotensin I - C	Cambien, F	9519756	No data for ACE
1	Association of Tl	Sener EF, T	24126814	No data for ACE
1	Clinical utility of	Elmonem N	25407738	No data for ACE
1	DDOST, PRKCSH	Hoverfelt A	20490454	No data for ACE
1	Effect of a polyn	Morita T, It	10543322	No data for ACE
1	Effects of erythr	Glicklich D,	10428268	No data for ACE
1	Impact of the pr	Lely AT, var	18077786	No data for ACE
1	Kinin-dependen	Molinaro G	17003818	No data for ACE
1	Manganese sup	Lee SJ, Cho	16324912	No data for ACE
1	Nephropathy in	Orchard TJ,	12164879	No data for ACE
1	Neuropeptide Y	Ito, H; Mor	10363627	No data for ACE
1	Polymorphisms	Frojdo, S; S	16211375	No data for ACE
1	Role of glycaemi	Powrie JK,	7819935	No data for ACE
1	Connexin 43 is n	Gerl, M; Ku	24062052	Non-human
1	Kallikrein and ar	Catanzaro	6186886	Non-human
1	Multi-species co	Hamilton N	23408978	Non-human
1	N-domain angio	Bueno, V; F	15194348	Non-human
1	Renal angiotens	Stoneking,	9719278	Non-human
1	Altered activitie	Sharma, JN	25964383	Not a case-control study
1	Angiotensin con	Yildiz A, Ya	12832741	Not a case-control study
1	Angiotensin-con	So WY, Ma	16395257	Not a case-control study
1	Angiotensin-con	AkÃ§ay A,	15013293	Not a case-control study
1	Angiotensin-con	Woo KT, La	18536822	Not a case-control study
1	Angiotensin-con	Beige J, Off	9550656	Not a case-control study
1	Angiotensin-I co	Ng, D; Tai,	15830182	Not a case-control study
1	Antihypertensiv	Kuznetsova	10918550	Not a case-control study
1	Antiproteinuric	Haneda M,	15364166	Not a case-control study
1	Association betv	Huang, ZK;	26000752	Not a case-control study
1	Association betv	van Essen (	8538349	Not a case-control study
1	Association of tl	Akcay A, Se	15385810	Not a case-control study
1	Captopril enhan	Di Paolo S,	12499886	Not a case-control study
1	Contribution of	Wong TY, S	11431175	Not a case-control study
1	Correlates of AC	Nikzamir A,	17986476	Not a case-control study
1	Deletion insertic	BjÃ¶rck S,	9269704	Not a case-control study
1	Disease progres	Woo KT, La	17601378	Not a case-control study
1	Effect of deletio	Parving HH	8806248	Not a case-control study
1	Gene polymorpl	Zhang G, W	17984617	Not a case-control study
1	Genetics and th	Marre, M	10097900	Not a case-control study
1	Genetics of angi	Costerouss	9247746	Not a case-control study
1	Hereditary facto	Marre, M;	10922971	Not a case-control study
1	Impact of geneti	Siekierka-H	19681973	Not a case-control study
1	Insertion/deletic	Osono E, K	9820440	Not a case-control study
1	Insertion/deletic	Fedor R, As	21620105	Not a case-control study
1	Is the presence	Ng, DPK; Ta	18523141	Not a case-control study
1	Is there a role o	Isbir CS, Ak	11525534	Not a case-control study
1	M235T angioter	Staessen, J.	10100088	Not a case-control study
1	Microfluidic chi	Sohni YR, B	12554058	Not a case-control study
1	Mistyping of the	Ueda, S; He	8863184	Not a case-control study

For peer review only

1	PAI-1 4G/5G anc	Aucella F, M	12748347	Not a case-control study
1	Risk factors for t	Oue, T; Nar	10580616	Not a case-control study
1	Survival in type :	PadrÃ³-Mic	19014923	Not a case-control study
1	The effect of an	Nakayama	16312263	Not a case-control study
1	A novel human I	Barash U, C	20007507	Not a renal disease focus
1	A study on the a	Chmaisse H	19861867	Not a renal disease focus
1	Abnormal hepat	Drenth JP,	15057895	Not a renal disease focus
1	ACE and PC-1 ge	de Azevedc	12126783	Not a renal disease focus
1	ACE gene inserti	Dell'omo, G	16889537	Not a renal disease focus
1	Albuminuria anc	Thomas GN	11200871	Not a renal disease focus
1	alpha-adducin a	Clark, CJ; D	11116113	Not a renal disease focus
1	Angiotensin con	Nagi DK, M	8582133	Not a renal disease focus
1	Angiotensin con	Miller JA, S	8995725	Not a renal disease focus
1	Angiotensin I co	Tassiulas, I	9710341	Not a renal disease focus
1	Angiotensin I-co	Costerouss	9830503	Not a renal disease focus
1	Angiotensin I-co	Vasku, A; S	9607178	Not a renal disease focus
1	Angiotensin-con	Mansoor Q	22607040	Not a renal disease focus
1	Angiotensin-con	Negi VS, De	25957879	Not a renal disease focus
1	Angiotensin-con	Catarsi P, R	16002416	Not a renal disease focus
1	Angiotensin-con	Douglas G,	15338496	Not a renal disease focus
1	Angiotensin-con	Pedersen-E	19820429	Not a renal disease focus
1	Angiotensin-con	Stefansson	10855732	Not a renal disease focus
1	Angiotensin-con	Singh M, Si	27030424	Not a renal disease focus
1	Angiotensin-con	Uhm WS, L	12043886	Not a renal disease focus
1	Angiotensin-Cor	Lachurie, M	7796503	Not a renal disease focus
1	Angiotensin-con	Al-Awadhi	17631741	Not a renal disease focus
1	Angiotensin-con	Prkacin I, N	11505631	Not a renal disease focus
1	Are the angioter	Dikmen M,	16791358	Not a renal disease focus
1	Association betv	Boomsma I	15830186	Not a renal disease focus
1	Association of A	Bahramali I	26861937	Not a renal disease focus
1	Association of A	Alsafar H, H	26491214	Not a renal disease focus
1	Association of ar	Rabbani M	18711292	Not a renal disease focus
1	Association of ar	Nikzamir A	18154415	Not a renal disease focus
1	Association of ar	RodrÃ-gue	11345362	Not a renal disease focus
1	Association of A	Singh, PP; N	16621107	Not a renal disease focus
1	Association of B	Moholisa R	23730990	Not a renal disease focus
1	Association of cl	Elmonem N	26589000	Not a renal disease focus
1	Association of cl	Kitamura, H	9951811	Not a renal disease focus
1	Association of p	Meng, N; Z	25359286	Not a renal disease focus
1	Association of re	Conen D, G	18698212	Not a renal disease focus
1	Association of th	Mayer, NJ;	11836444	Not a renal disease focus
1	Carotid intima-n	NergizoÄŸl	10493570	Not a renal disease focus
1	CCL18: a urinary	Boot RG, V	16736095	Not a renal disease focus
1	COL4A1 mutatic	Weng, YC;	22522439	Not a renal disease focus
1	Comprehensive	Kato, N; Su	10953993	Not a renal disease focus
1	Contribution of	Mondorf U	9524045	Not a renal disease focus
1	Deletion polymc	Gharavi AG	8677872	Not a renal disease focus
1	Detection of the	Matsumotc	11106834	Not a renal disease focus
1	Different impact	Olivieri O,	11791024	Not a renal disease focus
1	Distribution of d	Diamantop	10821349	Not a renal disease focus

1	Distribution of h	Hussain N,	23448612	Not a renal disease focus
1	Effects of angiot	Kulah E, Du	17625392	Not a renal disease focus
1	Efficacy of Korea	Kwon, DH;	23717118	Not a renal disease focus
1	Elevated urinary	Clausen, P;	10872702	Not a renal disease focus
1	Frequencies Of \	Zee, Ryl; Be	7882587	Not a renal disease focus
1	Genetic polymo	Deuster, PA	23543093	Not a renal disease focus
1	Genetic polymo	Olivieri O, T	10567188	Not a renal disease focus
1	Genetic risk for	Missouris C	8821841	Not a renal disease focus
1	Genetic risk of a	van Onna M	15326089	Not a renal disease focus
1	Genetic variants	Szperl M, D	19043368	Not a renal disease focus
1	Hepatocystin is	Overton JD	26671672	Not a renal disease focus
1	Impact of mater	Pfab T, Stir	17563539	Not a renal disease focus
1	Increased amou	Suehiro T, I	15164285	Not a renal disease focus
1	Increased D allel	Morrison C	11381371	Not a renal disease focus
1	Increased expre	Yamani MH	17097490	Not a renal disease focus
1	Increased frequ	Onizuka M,	16044138	Not a renal disease focus
1	Independent, M	Morris, Bj;	8104754	Not a renal disease focus
1	Inhibition of tiss	Montgome	9797175	Not a renal disease focus
1	Insertion/Deleti	de Martino	21477733	Not a renal disease focus
1	Late-onset acid	Mehler M,	9923	Not a renal disease focus
1	Leukocyte beta-	Raghavan S	6770675	Not a renal disease focus
1	Lys(173)Arg and	Komiya I, Y	10720581	Not a renal disease focus
1	Mutations in PR	Li A, Davila	12529853	Not a renal disease focus
1	No association b	Yildiz A, Ak	10657713	Not a renal disease focus
1	Peripheral vascu	Thomas GN	14632699	Not a renal disease focus
1	Pharmacogeneti	Okamura A	10535720	Not a renal disease focus
1	Polymorphisms	Deckers IA,	24978482	Not a renal disease focus
1	Relationship of b	Fu Y, Katsu	15894833	Not a renal disease focus
1	Relationship of e	Heltianu C,	15784171	Not a renal disease focus
1	Relationship of t	Huang XH,	9544854	Not a renal disease focus
1	Renal ACE immu	Mizuiri S, Y	9469501	Not a renal disease focus
1	Renal changes o	Marre, M; I	10082486	Not a renal disease focus
1	Renal outcome a	Molad Y, G	11071585	Not a renal disease focus
1	Renin-angiotens	BuraczyÅ„s	14502296	Not a renal disease focus
1	Renovascular di	Pizzolo F, M	14718831	Not a renal disease focus
1	Synergistic effec	Barlassina (	10720960	Not a renal disease focus
1	Testing of poten	Schoenfeld	24667567	Not a renal disease focus
1	The angiotensin	Ebert, MPA	16365022	Not a renal disease focus
1	The Captopril Pr	Hansson L,	9495662	Not a renal disease focus
1	The presence of	GÃ¼ngÃ¶r	21332339	Not a renal disease focus
1	[A study on angi	Nishina M.	9014479	Not English or Spanish
1	[Angiotensin-co	Krajina-Anc	23120809	Not English or Spanish
1	[Angiotensin-co	Guan T, Liu	10436947	Not English or Spanish
1	[Arterial hyperte	Oko A, Åoc	14974362	Not English or Spanish
1	[Association bet	Wang L, Pa	10923445	Not English or Spanish
1	[Association bet	Qiu MY, Xie	25815490	Not English or Spanish
1	[Association bet	Miloserdov	11234416	Not English or Spanish
1	[Association of a	Lu LH, Cher	18393230	Not English or Spanish
1	[Association of t	Kamysheva	15532370	Not English or Spanish
1	[Association of t	BuraczyÅ„s	12476891	Not English or Spanish

For peer review only

1	[Correlative stuc	Wu XX, Zhe	15698501	Not English or Spanish
1	[Genetic predisp	Bzoma B, D	19112833	Not English or Spanish
1	[I/D relationship	Kaliev RR, E	16078593	Not English or Spanish
1	[Is PstI polymorp	Grzeszczak	9499204	Not English or Spanish
1	[Polymorphism s	Kutyrina IM	10420452	Not English or Spanish
1	[Relationship be	Wu S, Xiang	9596955	Not English or Spanish
1	[Relationship be	Moleda P, I	17941464	Not English or Spanish
1	[Relationship be	Liao L, Lei M	12016801	Not English or Spanish
1	[Renin-angioten	BuraczyÅ„s	11865575	Not English or Spanish
1	[Study on candic	Lu J, Li LM,	12848919	Not English or Spanish
1	ACE and AT1 rec	Kostic, M; S	15179569	Paediatric individuals
1	ACE gene inserti	Sekerli E, K	19603195	Paediatric individuals
1	ACE gene inserti	Serdaroglu	16208534	Paediatric individuals
1	ACE gene polym	Haszon I, Fr	12478352	Paediatric individuals
1	ACE gene polym	Tanaka R, I	9590186	Paediatric individuals
1	ACE gene polym	Sasongko T	16421456	Paediatric individuals
1	ACE gene polym	Fahmy ME,	18792483	Paediatric individuals
1	ACE gene polym	Celik US, N	16825089	Paediatric individuals
1	ACE I/D gene po	Oktem F, Si	14986085	Paediatric individuals
1	ACE I/D gene po	Hohenfelln	10452281	Paediatric individuals
1	Angiotensin con	Al-Eisa A, H	11487079	Paediatric individuals
1	Angiotensin con	Bajpai M, P	14713838	Paediatric individuals
1	Angiotensin con	Patil SJ, Gu	16272677	Paediatric individuals
1	Angiotensin I co	Pavlovic, M	9950302	Paediatric individuals
1	Angiotensin I-co	Pavlovic, M	9354852	Paediatric individuals
1	Angiotensin-con	Rigoli, L; Ch	15470205	Paediatric individuals
1	Angiotensin-con	al-Eisa A, H	10986863	Paediatric individuals
1	Angiotensin-con	Zhou J, Tiar	15315169	Paediatric individuals
1	Angiotensin-con	Tsai IJ, Yang	16645262	Paediatric individuals
1	Angiotensin-con	BatiniÄ‡ D,	25997642	Paediatric individuals
1	Angiotensinoger	Gallego, PH	18413222	Paediatric individuals
1	Association of A	Dhandapar	26154535	Paediatric individuals
1	Association of ai	Laksmi NK,	20149750	Paediatric individuals
1	Association of tł	Shahid S, A	22033511	Paediatric individuals
1	DD genotype of	Alasehirli B	22017506	Paediatric individuals
1	Effect of angiote	Saber-Ayac	20418353	Paediatric individuals
1	Gene polymorpł	Kaplan Ä°, S	25262176	Paediatric individuals
1	Genetic polymoı	Frishberg Y	9853248	Paediatric individuals
1	Glycosphingolipi	Dawson G.	5016302	Paediatric individuals
1	HPSE2 mutation	Bulum B, Ã	25924634	Paediatric individuals
1	Impact of ACE I/	Hohenfelln	11354781	Paediatric individuals
1	Impact of comm	Hussein A,	25939993	Paediatric individuals
1	Implication of ge	Hahn H, Ku	16133060	Paediatric individuals
1	Implications of c	Ozen S, Alik	10401028	Paediatric individuals
1	Is ACE gene poly	ErdoÄŸan H	15138870	Paediatric individuals
1	Late effects on r	BÃ¡rdi E, O	15390293	Paediatric individuals
1	Low renin-angio	Peruzzi L, L	16006956	Paediatric individuals
1	Modification of	Rangel M, c	25170764	Paediatric individuals
1	Polymorphisms	Amoroso A	9870486	Paediatric individuals
1	Polymorphisms	Maruyama	11354780	Paediatric individuals

For peer review only

1	Polymorphisms	Ece A, Teke	16109085	Paediatric individuals
1	Polymorphisms	Savvidou A	20022049	Paediatric individuals
1	Renin-angiotens	Papp F, Frie	12579405	Paediatric individuals
1	Renin-angiotens	Ozkaya, O;	16521052	Paediatric individuals
1	Renin-angiotens	Pardo R, M	12579398	Paediatric individuals
1	Renin-angiotens	Liu KP, Lin (	15045574	Paediatric individuals
1	Role of platelet-	Tanaka R, I	10430976	Paediatric individuals
1	Significance of A	Hori C, Hira	11474225	Paediatric individuals
1	High serum enal	Elung-Jense	11881130	Pharmaceutical drug focus
1	ACE gene polym	Parving, HH	18199798	Pharmaceutical drug focus
1	Chronic proteinu	Ruggenenti	10616844	Pharmaceutical drug focus
1	Effect of angiote	Penno G, C	9726242	Pharmaceutical drug focus
1	Enalapril and los	Rashtchiza(	17222813	Pharmaceutical drug focus
1	Long-term renop	Andersen S	12716812	Pharmaceutical drug focus
1	Randomized pla	Kvetny J, G	11181984	Pharmaceutical drug focus
1	Renin-angiotens	Noroozian a	17524880	Pharmaceutical drug focus
1	The influence of	Luik PT, Ho	12856080	Pharmaceutical drug focus
2	Influence of gen	Parchwani	26214998	Incorrect patient group
2	Polymorphism o	Woo, KT; La	15504143	Overlap in patient group
2	Polymorphism o	Frimat, L; P	11053482	Incorrect patient group
2	Interaction of M	Rahimi Z, H	21942443	Incorrect patient group
2	The frequency o	Rahimi Z, F	20853144	Incorrect patient group
2	Polymorphism o	Hanyu, O; I	9509566	Incorrect patient group
2	Angiotensin I co	Gutierrez, (	9258285	No data
2	The angiotensin	Hibberd, M	9025006	Incorrect patient group
2	Genetic Predisp	Doria, A; W	7909524	Incorrect patient group
2	Prognostic value	Hadjadj S, F	18523145	No data
2	Association betv	Hadjadj S, T	17376814	No data
2	Combinational e	Osawa N, K	17143591	No data
2	Frequency of an	Degirmenc	16178979	No data
2	Genetic variatio	Boright AP,	15793268	Incorrect patient group
2	Effects of the ge	Luther Y, B	14610337	No data
2	ACE gene polym	Lau YK, Wo	12119485	Overlap in patient group
2	Association of tl	Gohda T, M	11770799	No data
2	The DD genotyp	Azar ST, Za	11428725	Incorrect patient group
2	Genetic polymoi	Lovati E, Ri	11422735	No data
2	Association of a	Szelestei T,	10977777	No data
2	Lack of synergis	Tarnow L, K	10907125	No data
2	The ACE insertio	van Dijk MA	10831637	Incorrect patient group
2	The renin--angic	Ong-Ajyoot	10511770	No data
2	ACE, PAI-1, deco	De Cosmo S	10495473	Incorrect patient group
2	Effect of ACE gei	Kuramoto I	10023638	Incorrect patient group
2	Angiotensin-con	Jeffers BW,	9264004	No data
2	Hypertensive ne	Kario K, Kai	9081678	Incorrect patient group
2	Relationships be	Oh TG, Shir	8854649	Incorrect patient group
2	Genetic polymoi	BuraczyÅ„s	12898858	No data
3	[Angiotensin-1 c	Ortega-Pier	17570179	< 3 populations reported per SNP
3	Angiotensin con	Fernández-	9607207	< 3 populations reported per SNP
3	Angiotensin-con	Abbas D et	21976404	< 3 populations reported per SNP
3	Angiotensin-con	Lee DY et a	9434071	< 3 populations reported per SNP

For peer review only

3	Angiotensin-I Cc	Kumar A et al.		< 3 populations reported per SNP
3	Association betv	Uemasu J ε	9180368	< 3 populations reported per SNP
3	Association betv	Huo P et al	25227524	< 3 populations reported per SNP
3	Association of ar	Akai Y et al	10099886	< 3 populations reported per SNP
3	Association of tl	Li X et al.	20540812	< 3 populations reported per SNP
3	Chronic renal ins	Prasad P et	WOS:00023	< 3 populations reported per SNP
3	Clinical impact o	Amorim et	23362199	< 3 populations reported per SNP
3	Contribution of ¡	Marre M et	9120002	< 3 populations reported per SNP
3	DNA polymorph	Freire MBS	9794558	< 3 populations reported per SNP
3	Gene polymorpl	Su SL et al.	22147663	< 3 populations reported per SNP
3	Genes involved i	Litovkina O	24727057	< 3 populations reported per SNP
3	Genetic Clues To	Krcunovic Z	WOS:00028	< 3 populations reported per SNP
3	Genetic polymor	Miura J et a	10499884	< 3 populations reported per SNP
3	Genetic polymor	Fabris B et	15662219	< 3 populations reported per SNP
3	Genetic risk fact	Tripathi G ε	18242170	< 3 populations reported per SNP
3	Genetic variants	Shaikh R et	24737640	< 3 populations reported per SNP
3	Identification of	Ezzidi I et a	19787680	< 3 populations reported per SNP
3	Influence of ang	Ramanatha	27748299	< 3 populations reported per SNP
3	Influence of gen	Parchwani	26214998	< 3 populations reported per SNP
3	Influence of the	Merta M et	12697976	< 3 populations reported per SNP
3	Lack of associati	Arfa I et al.	18404607	< 3 populations reported per SNP
3	Polymorphism o	Aucella F et	10773756	< 3 populations reported per SNP
3	Polymorphism o	Ayed K et a	16635753	< 3 populations reported per SNP
3	Polymorphisms	Sprovieri SI	15934435	< 3 populations reported per SNP
3	Renin-angiotens	Parsa A et a	15789057	< 3 populations reported per SNP
3	The DD genotyp	Vleming LJ	10099885	< 3 populations reported per SNP
3	The effect of pol	Möllsten A	18413189	< 3 populations reported per SNP
3	The presence of	Canani LH ε	16108844	< 3 populations reported per SNP

For peer review only

Supplementary Table S3b

Exclusion S	Title	Authors	PMID	Reason
1	ACACÎ² gen	Shah VN, C	23081748	No data for ACE2
1	ACE gene ir	Lee YJ, Tsai	12032106	No data for ACE2
1	ACE variant	Ahluwalia T	19108684	No data for ACE2
1	[Angiotens	Ortega-Pier	17570179	No data for ACE2
1	[Clinical an	Diez Ojea B	21113220	No data for ACE2
1	A disease h	Ng DP, Plac	16936219	No data for ACE2
1	A functiona	MÃ¶llsten	17192491	No data for ACE2
1	A HindIII pc	Onaran M,	19616291	No data for ACE2
1	A Lack of Si	Jannot AS,	26829596	No data for ACE2
1	A leucine r	Freedman	17205963	No data for ACE2
1	A new poly	Smolenicka	9589699	No data for ACE2
1	A novel me	Chen Y, Bu	19162184	No data for ACE2
1	A polymorp	Bhandary U	21624462	No data for ACE2
1	A rare hapl	Martin RJ,	19783860	No data for ACE2
1	A risk allele	Genovese G	20668430	No data for ACE2
1	A simultan	Papacostas	6688659	No data for ACE2
1	ACE gene p	Lau YK, Wo	12119485	No data for ACE2
1	ACE gene p	ViklickÃ½ C	11239522	No data for ACE2
1	ACE gene p	Felehgari V	20830509	No data for ACE2
1	ACE genoty	Stratta P, S	19034872	No data for ACE2
1	ACE, PAI-1,	De Cosmo S	10495473	No data for ACE2
1	ADAMTS13	Rurali E, Nc	23733198	No data for ACE2
1	African anc	Behar DM,	20144966	No data for ACE2
1	Albuminuri	Thomas GN	11200871	No data for ACE2
1	Aldosteron	Lajer M, Sc	16759311	No data for ACE2
1	Aldosteron	Yilmaz M, S	25957425	No data for ACE2
1	Allelic varia	Patente TA	25862415	No data for ACE2
1	An integrat	Nanayakka	24351856	No data for ACE2
1	Analysis of	Ali A, Vasu	21421653	No data for ACE2
1	Angiotensi	FernÃ¡ndez	9607207	No data for ACE2
1	Angiotensi	Yildiz A, Ya	12832741	No data for ACE2
1	Angiotensi	Hueso M, A	15284309	No data for ACE2
1	Angiotensi	Schmidt S,	9269698	No data for ACE2
1	Angiotensi	Tassiulas IC	9710341	No data for ACE2
1	Angiotensi	Grzeszczak	9727375	No data for ACE2
1	Angiotensi	Stratta P, C	10352195	No data for ACE2
1	Angiotensi	So WY, Ma	16395257	No data for ACE2
1	Angiotensi	Abbas D, Ez	21976404	No data for ACE2
1	Angiotensi	Douglas G,	15338496	No data for ACE2
1	Angiotensi	Araz M, Yil	11640993	No data for ACE2
1	Angiotensi	Uhm WS, L	12043886	No data for ACE2
1	Angiotensi	Jeffers BW,	9264004	No data for ACE2
1	Angiotensi	Lee DY, Kim	9434071	No data for ACE2
1	Angiotensi	Al-Harbi EM	21207118	No data for ACE2
1	Angiotensi	Beige J, Sch	9259361	No data for ACE2
1	Angiotensi	AkÃ§ay A, (	15013293	No data for ACE2
1	Angiotensi	Woo KT, La	18536822	No data for ACE2
1	Angiotensi	Mizuiri S, H	7477652	No data for ACE2

For peer review only

1	Angiotensi	Ortiz MA, D	15012717	No data for ACE2
1	Angiotensi	Schmidt A,	8785402	No data for ACE2
1	Angiotensi	Beige J, Off	9550656	No data for ACE2
1	Angiotensi	Arzu Ergen	15365253	No data for ACE2
1	Angiotensi	Zychma MJ	11096141	No data for ACE2
1	Anti-glutatl	Aguilera I, A	18308775	No data for ACE2
1	APOL1 vari	Papeta N, K	21997397	No data for ACE2
1	Apolipopro	Lipkowitz M	22832513	No data for ACE2
1	Assessmen	Karsli Cepp	21859400	No data for ACE2
1	Associatior	Ohno T, Ka	8596493	No data for ACE2
1	Associatior	Prasad P, T	20353610	No data for ACE2
1	Associatior	Kavanagh D	21103979	No data for ACE2
1	Associatior	Wu LS, Hsie	19506043	No data for ACE2
1	Associatior	Doi Y, Yosh	8720609	No data for ACE2
1	Associatior	Melander C	11114699	No data for ACE2
1	Associatior	Uemasu J, I	9180368	No data for ACE2
1	Associatior	Hadjadj S, T	17376814	No data for ACE2
1	Associatior	van Essen C	8538349	No data for ACE2
1	Associatior	Li WL, Lu C.	26125729	No data for ACE2
1	Associatior	Ferraresso	21851254	No data for ACE2
1	Associatior	Huo P, Zhai	25227524	No data for ACE2
1	Associatior	Xiong H, Xi	19682085	No data for ACE2
1	Associatior	Vigo Gago I	15887856	No data for ACE2
1	Associatior	de Souza C	17914260	No data for ACE2
1	Associatior	Morris BJ, I	11907646	No data for ACE2
1	Associatior	Szelestei T,	10977777	No data for ACE2
1	Associatior	Pawlik M, N	23681285	No data for ACE2
1	Associatior	Tang FY, Liu	18629615	No data for ACE2
1	Associatior	Pei Y, Schol	9259580	No data for ACE2
1	Associatior	Cai Z, Chen	22720119	No data for ACE2
1	Associatior	Park HJ, Kir	24621205	No data for ACE2
1	Associatior	Siddarth M	23619522	No data for ACE2
1	Associatior	Nagase S, S	12701818	No data for ACE2
1	Associatior	BÃ¶ger CA,	21980298	No data for ACE2
1	Associatior	Bellini MH,	17498125	No data for ACE2
1	Associatior	Thaha M, P	18793530	No data for ACE2
1	Associatior	Ezzidi I, Mt	18413207	No data for ACE2
1	Associatior	Santos KG,	21255858	No data for ACE2
1	Associatior	Issac MS, A	24151105	No data for ACE2
1	Associatior	Yoshida T, I	19424605	No data for ACE2
1	Associatior	Yoshida T, I	19724895	No data for ACE2
1	Associatior	Vieira SM, I	21962117	No data for ACE2
1	Associatior	Yoshida T, I	19578796	No data for ACE2
1	Associatior	Yoshida T, I	19288030	No data for ACE2
1	Associatior	Yoshida T, I	19406964	No data for ACE2
1	Associatior	Datta SK, K	20954980	No data for ACE2
1	Associatior	Turki A, Ma	23727064	No data for ACE2
1	Associatior	Bostrom M	20466664	No data for ACE2
1	Associatior	Liu Y, Zhou	26841219	No data for ACE2
1	Associatior	Eroglu Z, Ce	18413162	No data for ACE2

For peer review only

1	Associatior	Gohda T, M	11770799	No data for ACE2
1	Associatior	Keene KL, I	18184924	No data for ACE2
1	Associatior	Akcay A, Se	15385810	No data for ACE2
1	Associatior	Neugebaue	10868974	No data for ACE2
1	Associatior	Hodgkinsor	14578247	No data for ACE2
1	Associatior	Sener EF, T	24126814	No data for ACE2
1	Associatior	Genovese (	20647424	No data for ACE2
1	Associatior	Pazik J, OÅ,	22210424	No data for ACE2
1	Associatior	Zhao YG, Sł	23044313	No data for ACE2
1	Associatior	Lavery GG,	11916625	No data for ACE2
1	Associatior	Kim SK, Par	24654912	No data for ACE2
1	Associatior	Zhu AN, Ya	25867326	No data for ACE2
1	Balkan end	Andonova I	15365959	No data for ACE2
1	Base excisi	Cai Z, Guo \	22780951	No data for ACE2
1	BsmI polym	Zhang H, W	22245613	No data for ACE2
1	Calpain 10	Demirci H,	18554168	No data for ACE2
1	Cardiac hyp	Wang AY, C	12675870	No data for ACE2
1	Carnosine a	Janssen B, I	16046297	No data for ACE2
1	Catalase/su	SÃ¶zmen E	11440784	No data for ACE2
1	Characteriz	Munkert A,	18927121	No data for ACE2
1	Characteriz	Armstrong	23762448	No data for ACE2
1	Chronic prc	Ruggenenti	10616844	No data for ACE2
1	Chronic rer	Marra M, M	23129820	No data for ACE2
1	Circulating	Anguiano, I	25813276	No data for ACE2
1	Circulating	Caplin B, N	20010544	No data for ACE2
1	Clinical anc	Kim IW, Mc	22183771	No data for ACE2
1	Clinical imp	Amorim CE	23362199	No data for ACE2
1	Clinical util	Elmonem M	25407738	No data for ACE2
1	Combinatic	Osawa N, K	17143591	No data for ACE2
1	Common va	Ahluwalia T	21573905	No data for ACE2
1	Contributic	Wong TY, S	11431175	No data for ACE2
1	Copy Numb	Ruchi R, Ge	25933006	No data for ACE2
1	Correlates	Nikzamir A,	17986476	No data for ACE2
1	CYP2C9 anc	MiroÅ¡evic	24024895	No data for ACE2
1	CYP3A4 ger	Wang YY, Z	23217392	No data for ACE2
1	Cys327Cys	KalousovÃ¡	24667032	No data for ACE2
1	DDOST, PR	Hoverfelt A	20490454	No data for ACE2
1	Deletion in	BjÃ¶rck S, I	9269704	No data for ACE2
1	Dense map	Nelson GW	20124285	No data for ACE2
1	Deregulate	Denizot Y, A	10978389	No data for ACE2
1	Different ir	Olivieri O, (	11791024	No data for ACE2
1	Differential	Freedman I	21698141	No data for ACE2
1	Differential	Shankland .	10916090	No data for ACE2
1	Dinucleotic	Maeda S, H	11576356	No data for ACE2
1	Disease prc	Woo KT, La	17601378	No data for ACE2
1	Distributior	Thaha M, Y	23770786	No data for ACE2
1	DNA polym	Freire MB,	9794558	No data for ACE2
1	DNA repair	Radwan WI	25310768	No data for ACE2
1	Donor age	De Meyer I	22835948	No data for ACE2
1	Donor DNA	Rutkowska	18290564	No data for ACE2

For peer review only

1	Effect of a (	Morita T, It	10543322	No data for ACE2
1	Effect of AC	Kuramoto I	10023638	No data for ACE2
1	Effect of he	Baum L, Ng	15983323	No data for ACE2
1	Effect of hu	Tarng DC, T	11675410	No data for ACE2
1	Effects of tl	Luther Y, Ba	14610337	No data for ACE2
1	Efficient sc	Zhang LR, S	16952345	No data for ACE2
1	Elevated M	Kaunisto M	19366862	No data for ACE2
1	Elevated pl	Dieplinger	8432847	No data for ACE2
1	Elevation o	Korponay-S	14570724	No data for ACE2
1	Endothelial	Marson BP,	20849252	No data for ACE2
1	Endothelial	Yilmaz E, M	20005399	No data for ACE2
1	Endothelial	ReiterovÃ¡	12077489	No data for ACE2
1	Endothelial	Shimizu T, (	12413777	No data for ACE2
1	Endothelial	Ahluwalia T	18401556	No data for ACE2
1	Endothelial	Buraczynsk	15299097	No data for ACE2
1	Endothelial	de Prado A	12402580	No data for ACE2
1	Endothelial	Wang Y, Kil	10570094	No data for ACE2
1	Endothelial	Shoukry A,	22313046	No data for ACE2
1	Endothelial	Asakimori Y	11549906	No data for ACE2
1	Endothelial	Bhandary U	18331440	No data for ACE2
1	Endothelial	Kerkeni M,	19376104	No data for ACE2
1	eNOS 4a/b	Rahimi Z, R	23594559	No data for ACE2
1	Epidermal g	Magistroni	12653106	No data for ACE2
1	Estimated g	Otero Gonz	26152646	No data for ACE2
1	Estrogen re	Cho NH, Sh	15189505	No data for ACE2
1	Evaluation	Palmer ND,	24551085	No data for ACE2
1	Evaluation	Hirakawa S	12830465	No data for ACE2
1	Evaluation	Barnas U, S	9084972	No data for ACE2
1	Evidence fo	Lamnissou	15727257	No data for ACE2
1	Exclusion o	Wanic K, Pl	18753673	No data for ACE2
1	Exome seq	Sanna-Cher	21697813	No data for ACE2
1	Expression	Metalidis C	21544031	No data for ACE2
1	Factor B su	Nishimukai	3272818	No data for ACE2
1	Frequency	Degirmenc	16178979	No data for ACE2
1	Gene muta	Hoshida Y,	11896204	No data for ACE2
1	Gene polyr	Su SL, Lu KC	22147663	No data for ACE2
1	Gene polyr	Zhang G, W	17984617	No data for ACE2
1	Gene-gene	Su SL, Yang	24977181	No data for ACE2
1	Gene-polyr	Burg M, Me	9352153	No data for ACE2
1	Genes invo	Litovkina O	24727057	No data for ACE2
1	Genetic ana	Szarvas N, S	26826462	No data for ACE2
1	Genetic ana	Freedman I	11071967	No data for ACE2
1	Genetic an	HorovÃ¡ E,	22849862	No data for ACE2
1	Genetic an	Zhang D, G	22024547	No data for ACE2
1	Genetic de	St Peter SD	12364860	No data for ACE2
1	Genetic po	Rambausel	3118258	No data for ACE2
1	Genetic po	Zsom M, FÂ	22111818	No data for ACE2
1	Genetic po	Jimenez-So	22817530	No data for ACE2
1	Genetic po	Atanasova	15708542	No data for ACE2
1	Genetic po	Buraczynsk	16384824	No data for ACE2

For peer review only

1	Genetic po	Huang HD,	21163122	No data for ACE2
1	Genetic po	Lovati E, Ri	11422735	No data for ACE2
1	Genetic pre	Kumar R, Sl	23846111	No data for ACE2
1	Genetic pre	Nikolov IG,	1820343	No data for ACE2
1	Genetic pre	Doria A, Wa	7909524	No data for ACE2
1	Genetic reg	Kramer J, S	9334852	No data for ACE2
1	Genetic risl	Tripathi G,	18242170	No data for ACE2
1	Genetic var	Pirulli D, Cr	19229831	No data for ACE2
1	Genetic var	Shaikh R, Sl	24737640	No data for ACE2
1	Genetic var	Pai P, Hind	9474350	No data for ACE2
1	Genetic var	Boright AP,	15793268	No data for ACE2
1	Genetic var	Tayo BO, Kı	22956460	No data for ACE2
1	Genetic var	Kure M, Pe	21277817	No data for ACE2
1	Genetic var	Xu CW, Wu	22932273	No data for ACE2
1	Genotypic	Spare MK,	12919757	No data for ACE2
1	Genotyping	Wijnen PA,	18028890	No data for ACE2
1	Glu298Asp	MÃ¶llsten	17101543	No data for ACE2
1	Glutathion	Suvakov S,	23034843	No data for ACE2
1	Glutathion	Chen B, Bai	22116675	No data for ACE2
1	G-Protein b	Zychma MJ	10970984	No data for ACE2
1	G-protein b	ViklickÃ½ C	12424427	No data for ACE2
1	Haplotype	Doi K, Noiri	16215641	No data for ACE2
1	HDL choles	Radeau T, V	10754410	No data for ACE2
1	Hidden pop	Berger M, S	16333311	No data for ACE2
1	High preval	Tripathi G,	17042963	No data for ACE2
1	Homocyste	Tamura T, I	9740165	No data for ACE2
1	How to har	Saint-Marc	26316426	No data for ACE2
1	Hypertensi	Basset el-E	11926202	No data for ACE2
1	Hypertensi	Kario K, Kai	9081678	No data for ACE2
1	Identificati	Araki S, Ng	12874455	No data for ACE2
1	Identificati	Yamada Y,	23539754	No data for ACE2
1	Identificati	Torregrossa	20448065	No data for ACE2
1	Identificati	Yu H, Bowd	9535413	No data for ACE2
1	Identificati	Jacobasch (	7315101	No data for ACE2
1	Identificati	Li M, Foo JI	26028593	No data for ACE2
1	Identificati	Yang T, Me	24582653	No data for ACE2
1	Identificati	Toncheva D	15365958	No data for ACE2
1	Identificati	Ezzidi I, Mt	19787680	No data for ACE2
1	Immune re	Nobuoka Y	24454479	No data for ACE2
1	Immunoglo	Demaine A	2985495	No data for ACE2
1	Impact of a	Bantis C, H	21476902	No data for ACE2
1	Impact of C	Elens L, Cap	24052064	No data for ACE2
1	Impact of C	de Jonge H	24024898	No data for ACE2
1	Impact of C	Yaowakulp	26635230	No data for ACE2
1	Impact of c	Cho JH, Yoo	22310591	No data for ACE2
1	Impact of c	Krajewska I	19857655	No data for ACE2
1	Impact of E	Eller P, Hoc	17848394	No data for ACE2
1	Impact of g	Siekierka-H	19681973	No data for ACE2
1	Impact of r	El-Din Bess	21854353	No data for ACE2
1	Impact of p	SchÃ¶n S, F	16164625	No data for ACE2

For peer review only

1	Impact of p	Jung ES, Kir	21150220	No data for ACE2
1	In vivo acti	Lee JP, Yan	21429967	No data for ACE2
1	Increased f	Hsieh MC, I	10862639	No data for ACE2
1	Increased f	BlÃ¼thner	10200987	No data for ACE2
1	Increased l	Siddarth M	24216264	No data for ACE2
1	Influence o	Bantis C, H	21777344	No data for ACE2
1	Influence o	Marco MP,	11522087	No data for ACE2
1	Influence o	Provenzani	21922127	No data for ACE2
1	Influence o	Quteineh L	19067682	No data for ACE2
1	Influence o	Singh R, Ma	19486347	No data for ACE2
1	Influence o	Parchwani	26214998	No data for ACE2
1	Influence o	Nekooie-M	23649768	No data for ACE2
1	Influence o	Merta M, R	12697976	No data for ACE2
1	Influence o	Merta M, R	12212826	No data for ACE2
1	Insertion/d	Osono E, Ku	9820440	No data for ACE2
1	Insertion/d	Fedor R, As	21620105	No data for ACE2
1	Interaction	Stratta P, B	14767013	No data for ACE2
1	Interaction	Rahimi Z, H	21942443	No data for ACE2
1	Is increase	Reljic Z, Zla	25111321	No data for ACE2
1	Is Klotho F3	Ozdem S, Y	26022923	No data for ACE2
1	Is there a r	Isbir CS, Ak	11525534	No data for ACE2
1	Kinin-depe	Molinaro G	17003818	No data for ACE2
1	Lack of ass	Arfa I, Abid	18404607	No data for ACE2
1	Lack of ass	Wong TY, C	10352194	No data for ACE2
1	Lack of rela	Tarnow L, C	7729604	No data for ACE2
1	Lack of ser	Moeller S, C	24732864	No data for ACE2
1	Lack of syn	Tarnow L, K	10907125	No data for ACE2
1	Lipoprotein	Sechi LA, Zi	10469373	No data for ACE2
1	Low preval	Trochen N,	15304560	No data for ACE2
1	Major-histo	Welch TR, I	3458025	No data for ACE2
1	Manganese	Lee SJ, Cho	16324912	No data for ACE2
1	Manganese	Kidir V, Uz	26787049	No data for ACE2
1	Matrix met	Marson BP,	22302011	No data for ACE2
1	Matrix met	Cozzolino M	19221176	No data for ACE2
1	Membrano	Gradden C\	11181983	No data for ACE2
1	Microarray	Lim SC, Liu	19415232	No data for ACE2
1	Microfluidi	Sohni YR, B	12554058	No data for ACE2
1	MTHFR C67	El-Baz R, Se	22554825	No data for ACE2
1	Multiple su	Al-Kateb H,	17914031	No data for ACE2
1	Mutation a	Zhang S, M	15775720	No data for ACE2
1	MYH9 and	Colares VS,	24658608	No data for ACE2
1	MYH9 is a r	Kopp JB, Sr	18794856	No data for ACE2
1	MYH9 is as	Kao WH, Kl	18794854	No data for ACE2
1	MYH9-relat	Pecci A, Kle	24186861	No data for ACE2
1	Nephropat	Orchard TJ,	12164879	No data for ACE2
1	Nitric oxide	Passauer J,	15840038	No data for ACE2
1	Nitric oxide	Rippin JD, F	12687343	No data for ACE2
1	No associat	Schmidt S,	7485124	No data for ACE2
1	Nodular glo	Wang, M; Z	26973293	No data for ACE2
1	Non-muscl	Freedman I	19567477	No data for ACE2

For peer review only

1	NOS3 tagSI	Ramanatha	24824375	No data for ACE2
1	Nosocomia	Phipps LM,	22129760	No data for ACE2
1	Novel poly	Jacobson P	21206424	No data for ACE2
1	Novel sequ	Bahr C, Sch	16759312	No data for ACE2
1	Optimizatic	Thervet E, I	20393454	No data for ACE2
1	Oxidative s	Tiwari AK, I	18413200	No data for ACE2
1	PAI-1 4G/5	Aucella F, N	12748347	No data for ACE2
1	Paraoxonas	Eren Z, Kan	22555481	No data for ACE2
1	Paraoxonas	Agachan B,	15251338	No data for ACE2
1	Paraoxonas	Biasioli S, S	12790379	No data for ACE2
1	Paraoxonas	KovÃ¡cs TJ,	17173245	No data for ACE2
1	Paraoxonas	Pinizzotto I	11206400	No data for ACE2
1	Parathyroic	Gohda T, Sl	12046039	No data for ACE2
1	Patients wi	Sekine T, K	20200500	No data for ACE2
1	Pharmacog	Larriba J, In	20172323	No data for ACE2
1	Polymorph	Canani LH,	11916943	No data for ACE2
1	Polymorph	Frimat L, Pl	11053482	No data for ACE2
1	Polymorph	Ko GJ, Kang	18771471	No data for ACE2
1	Polymorph	Yorioka T, S	8529313	No data for ACE2
1	Polymorph	Hanyu O, H	9509566	No data for ACE2
1	Polymorph	Aucella F, V	10773756	No data for ACE2
1	Polymorph	Nair SS, Sar	25894154	No data for ACE2
1	Polymorph	Chen Y, Hu	18079690	No data for ACE2
1	Polymorph	Cooke JN, E	21968013	No data for ACE2
1	Polymorph	Zhou H, Ch	26627442	No data for ACE2
1	Polymorph	Narne P, Pc	25041504	No data for ACE2
1	Polymorph	Nannipieri	11408388	No data for ACE2
1	Polymorph	Cheng W, Z	21245129	No data for ACE2
1	Polymorph	Freedman I	19177153	No data for ACE2
1	Polymorph	Araki S, Ma	11151764	No data for ACE2
1	Polymorph	RajkoviÄ‡ I	21620813	No data for ACE2
1	Polymorph	Sprovieri SI	15934435	No data for ACE2
1	Posttranspl	BariÃ©ty J,	11158216	No data for ACE2
1	Prevalence	Shanmugar	26440392	No data for ACE2
1	Primary hy	van der Ho	22844106	No data for ACE2
1	Prognostic	Hadjadj S, I	18523145	No data for ACE2
1	Protein los	Wanner C,	7724034	No data for ACE2
1	Quantitativ	Tamano M,	15069174	No data for ACE2
1	RAGE and (	Franko B, B	26607824	No data for ACE2
1	Rare mutat	Sveinbjorns	25082825	No data for ACE2
1	Relation be	Tang FY, Zh	21045268	No data for ACE2
1	Relations b	Shin Shin Y	15331206	No data for ACE2
1	Relationshi	Crawford A	22790458	No data for ACE2
1	Relationshi	Lim SC, Gol	16922713	No data for ACE2
1	Relationshi	Agrawal S,	18067039	No data for ACE2
1	Relationshi	Xue JN, Gu	26782518	No data for ACE2
1	Relationshi	Movva S, A	17616353	No data for ACE2
1	Relationshi	El-Shehaby	23198772	No data for ACE2
1	Relationshi	Cusinato D	24528196	No data for ACE2
1	Relationshi	Santos KG,	16358232	No data for ACE2

For peer review only

1	Relationshi	Huang XH,	9544854	No data for ACE2
1	Relationshi	Oh TG, Shir	8854649	No data for ACE2
1	Relationshi	Marre M, E	8314010	No data for ACE2
1	Relationshi	Xin HW, Xic	19048245	No data for ACE2
1	Renin-angi	Lau YK, Wo	15153745	No data for ACE2
1	Renin-angi	Chen WJ, H	24907556	No data for ACE2
1	Replication	Maeda S, Ir	23543049	No data for ACE2
1	Resequenc	McKnight A	17319955	No data for ACE2
1	Re-Sequen	Hawkins G	26343748	No data for ACE2
1	Risk factors	Oue T, Nan	10580616	No data for ACE2
1	Risk of adv	Zanchi A, M	10652017	No data for ACE2
1	Role of alpl	Conway BR	15187197	No data for ACE2
1	Role of glyc	Powrie JK,	7819935	No data for ACE2
1	Role of the	Nicod J, Fre	11918733	No data for ACE2
1	Serum para	Suehiro T, I	12226554	No data for ACE2
1	Serum para	Itahara T, S	11480456	No data for ACE2
1	Sickle cell t	Hicks PJ, La	21849968	No data for ACE2
1	Single nucl	Alwohhaib	24821155	No data for ACE2
1	Sirolimus p	Djebli N, Ro	17048977	No data for ACE2
1	SIRTUIN 1 g	Shimoyama	22200427	No data for ACE2
1	Structural a	Zaehner T,	11012876	No data for ACE2
1	Suppressed	Matsui M,	24048373	No data for ACE2
1	Survival in	PadrÃ³-Mic	19014923	No data for ACE2
1	Synergism	Testa A, Ma	25435339	No data for ACE2
1	T(-786)-->C	Asakimori Y	12138283	No data for ACE2
1	Tacrolimus	Li L, Li CJ, Z	21331500	No data for ACE2
1	The A736V	Pelusi S, Gi	23433094	No data for ACE2
1	The ACE ins	van Dijk M	10831637	No data for ACE2
1	The acetyl-	Tang SC, Le	20519229	No data for ACE2
1	The angiot	Hibberd MI	9025006	No data for ACE2
1	The angiot	Lu M, Zhan	27633502	No data for ACE2
1	The associa	Geng F, Jia	22227166	No data for ACE2
1	The C825T	Shcherbak	11322658	No data for ACE2
1	The C825T	Fogarty DG	9833937	No data for ACE2
1	The DD ger	Azar ST, Za	11428725	No data for ACE2
1	The DD ger	Vleming LJ,	10099885	No data for ACE2
1	The effect	Nakayama	16312263	No data for ACE2
1	The effect	Akgul SU, C	22841242	No data for ACE2
1	The emerg	Nogueira E	17161359	No data for ACE2
1	The endoth	MÃ¶llsten	19246226	No data for ACE2
1	The freque	Rahimi Z, F	20853144	No data for ACE2
1	The functic	Zhang W, Y	25447894	No data for ACE2
1	The impact	Kurzawski I	16044099	No data for ACE2
1	The influen	McDonoug	19373489	No data for ACE2
1	The influen	ReiterovÃ¡	15287194	No data for ACE2
1	The influen	ReiterovÃ¡	16526315	No data for ACE2
1	The interac	Stratta P, C	22101623	No data for ACE2
1	The manga	Liu L, Zheng	18989629	No data for ACE2
1	The N-acet	Neugebaue	7851073	No data for ACE2
1	The numbe	Stern M, El	18444913	No data for ACE2

For peer review only

1	The prevale	SÃ¡nchez-F	19715905	No data for ACE2
1	The PTPN2	Sfar I, Gorg	19328948	No data for ACE2
1	The renin--	Ong-Ajyoot	10511770	No data for ACE2
1	The respon	Hodgkinsor	12606529	No data for ACE2
1	The role of	Dixit M, Ma	12571380	No data for ACE2
1	The role of	De Cosmo S	12147786	No data for ACE2
1	The serum	Paragh G, S	9736814	No data for ACE2
1	The V16A p	MÃ¶llsten	19834686	No data for ACE2
1	Thiopurine	Aghdaie MI	21819368	No data for ACE2
1	Time of dru	Musuamba	19855314	No data for ACE2
1	Toward per	Lampreabe	20970553	No data for ACE2
1	TPMT but r	Kurzawski I	19229528	No data for ACE2
1	Tripterygiu	Wen J, Li L,	19100464	No data for ACE2
1	Uromoduli	KÃ¶ttgen A	19959715	No data for ACE2
1	Variants of	Li GS, Zhan	17228361	No data for ACE2
1	Vitamin D r	Mostowska	23065277	No data for ACE2
1	XbaI GLUT1	Stefanidis I	19822956	No data for ACE2
1	A knock-in	Arsenault F	24121508	Non-human
1	An inducibl	Chowdhury	23336180	Non-human
1	Cardiac ang	Ferrario CM	26873967	Non-human
1	Carnosine t	Peters V, Sc	21833769	Non-human
1	Chronic rer	Stockelmar	9689017	Non-human
1	Control of I	Shaws TB, I	4856818	Non-human
1	Decreased	Uchio K, M	11014984	Non-human
1	Detection c	Singh KP, R	10087325	Non-human
1	Effect of ph	Tenenhous	7873297	Non-human
1	Enhanced a	de Morais S	2501210	Non-human
1	Fecal carria	Alonso S, M	17661289	Non-human
1	First WNK4	Gandolfi B,	23285264	Non-human
1	From man	Olabisi O, A	27509583	Non-human
1	Gain-of-fur	Li N, Nakan	14695357	Non-human
1	Generation	Fujimoto K,	17827337	Non-human
1	Increased i	Ijuin T, Yu Y	18573875	Non-human
1	Kallikrein a	Catanzaro (	6186886	Non-human
1	Kallikrein g	Liu K, Li QZ,	19307730	Non-human
1	Metabolic f	Fujino H, Ya	12442637	Non-human
1	Multi-speci	Hamilton N	23408978	Non-human
1	MyoD cont	Xiong Q, Ch	20336382	Non-human
1	N-Domain a	Bueno V, Pa	15194348	Non-human
1	Nitric oxide	Migliori M,	10394113	Non-human
1	Overexpres	Liu X, Zhao	19279555	Non-human
1	Purification	Sekine M, I	7983056	Non-human
1	Rat Ace alle	Kamilic, J; I	21788250	Non-human
1	Re-enginee	Davies B, H	26840484	Non-human
1	Role of ang	Yang, XH; W	22580272	Non-human
1	Susceptibili	Kleymenov	14604887	Non-human
1	Targeted d	FrÃ¶hlich L	20427744	Non-human
1	Telomerase	Sachsinger	11454711	Non-human
1	Testosteroı	Smolen TN,	8437129	Non-human
1	The activat	Wilson CH,	20531296	Non-human

For peer review only

1	The gene II	Marion E, K	12086927	Non-human
1	The mll-AF9	Dobson CL,	10393173	Non-human
1	The Pex1-G	Hiebler S, M	24503136	Non-human
1	The T309G	de Martino	25415135	Non-human
1	Tissue-spec	Hu JF, Orug	9482664	Non-human
1	Transgenic	Liu Z, Segav	21303955	Non-human
1	Tubule-spe	Zhou D, Li Y	22622501	Non-human
1	A common	Wang H, Li	21282199	Non-human
1	A functiona	Herrera VL,	25615575	Non-human
1	Allelic Varia	Jia Z, Johns	26172442	Non-human
1	Alternative	Ren S, Nguy	15788398	Non-human
1	An alternat	Wang Y, Ne	11786430	Non-human
1	ARHGDIA n	Gee HY, Sai	23867502	Non-human
1	Atrial natriu	Nakagawa	25027872	Non-human
1	Autosomal	Ryan S, Ver	20519437	Non-human
1	BAP1 misse	Morrison A	25830670	Non-human
1	Blocking th	StenstrÃ¶m	19657284	Non-human
1	Characteriz	Qiu H, Dai I	18508765	Non-human
1	Characteriz	Gui J, Song	17389928	Non-human
1	CpG island	Gillespie GA	1709739	Non-human
1	Cystic echir	Mitrea IL, Io	23075460	Non-human
1	Cytotoxicity	Ilinskaya OI	14733942	Non-human
1	DAPIT Over	Kontro H, C	26161955	Non-human
1	Dynamin-2	Hyndman K	26791826	Non-human
1	Evidence th	Bittner MA	8626670	Non-human
1	Expression	Zhang CJ, R	8294513	Non-human
1	Factor VIIa	Sundaram J	24977291	Non-human
1	Fluorescen	Liu J, Ernst	15489225	Non-human
1	Functional	Tonna S, Da	18206321	Non-human
1	Generation	Roy A, Goo	25477473	Non-human
1	Hepatitis C	Hiasa Y, Ku	18459156	Non-human
1	Holocarbox	Liu D, Zemp	24840043	Non-human
1	Human GRI	Wang Z, Ze	26667412	Non-human
1	Hypoxia ind	Ward ME, T	16276418	Non-human
1	Î±1,3-galac	Park JY, Par	22775484	Non-human
1	Identificati	Wang L, Ve	25906147	Non-human
1	Identificati	Jung KA, Ch	23305850	Non-human
1	Implication	Heilig C, Br	14578187	Non-human
1	Luteinizing	Apaja PM, T	16495341	Non-human
1	Maitotoxin	Sinkins WG	19794142	Non-human
1	Mammalian	Zhao Y, Zha	26061549	Non-human
1	Mediation	Yao H, Bricl	20966046	Non-human
1	Mitochond	Chandler R	19088183	Non-human
1	Modulation	Gray SG, Ig	12691522	Non-human
1	N-terminal	Bodenstein	17220356	Non-human
1	Organizatio	Cabral CM,	12181335	Non-human
1	p300 intera	Eid JE, Kung	11030627	Non-human
1	p38MAPK c	Wong ES, L	19619499	Non-human
1	Pitchfork re	Kinzel D, Bo	20643351	Non-human
1	Presence o	Teixeira AC	23387326	Non-human

For peer review only

1	Primary hy	Vidgren G,	22486513	Non-human
1	Production	Turner N, F	8006020	Non-human
1	PTPN2 con	Spalinger M	25492475	Non-human
1	Recombina	Velan B, Kr	1849451	Non-human
1	Renal angi	Stoneking,	9719278	Non-human
1	Renal colle	Hyndman K	23608660	Non-human
1	Role of the	Hedlund M	8642245	Non-human
1	Sprouty1 is	Basson MA	15691764	Non-human
1	Three nove	Martin V, B	11956212	Non-human
1	TNFÎ± Amp	Thiyagaraja	26065428	Non-human
1	Truncated	Rau H, Rea	10098508	Non-human
1	Use of the	Gallagher A	12874458	Non-human
1	YME1L con	Stiburek L,	22262461	Non-human
1	Analysis of	Wolford JK,	11350189	Not a case-control study
1	Bardet-Bie	HjortshÃ¸j	20120035	Not a case-control study
1	Brief Repor	Zhou XJ, Na	24943867	Not a case-control study
1	Chromosor	Leak TS, La	20144192	Not a case-control study
1	DNA-based	Olschwang	7552140	Not a case-control study
1	Whole-gen	Cordell HJ,	19959718	Not a case-control study
1	A family of	Nagase T, N	12050233	Not a case-control study
1	A Novel vo	Sexton A, R	26323595	Not a case-control study
1	Clinical ass	Sato N, Kat	15001591	Not a case-control study
1	Familial no	Opocher G,	17102081	Not a case-control study
1	Genetic co	Emery AE,	4976958	Not a case-control study
1	Genome-w	Teumer A,	26631737	Not a case-control study
1	Identificati	Fiskerstran	20007846	Not a case-control study
1	Mistyping	Ueda S, He	8863184	Not a case-control study
1	MYH9 gene	Kopp JB, W	20807613	Not a case-control study
1	Novel muta	Xie J, Hao X	26039629	Not a case-control study
1	Novel SMA	Simon AJ, L	24197801	Not a case-control study
1	Porphyria i	Schneider-	19350426	Not a case-control study
1	Presentatic	Lorenzo V,	16912707	Not a case-control study
1	RFLP-DR be	Mytilineos	1975140	Not a case-control study
1	Superoxide	Beckman G	4775457	Not a case-control study
1	Verificatior	Barlow-Ste	19287242	Not a case-control study
1	A prospecti	Kuypers DR	21118736	Not a case-control study
1	ABCB1 (MD	Tavira B, G	25781547	Not a case-control study
1	ABCB1 poly	GarcÃ-a M,	22886152	Not a case-control study
1	Acute kidn	Kirwan CJ,	22005822	Not a case-control study
1	An efficien	Eisenberge	25646624	Not a case-control study
1	An unusual	Gambaro G	15814540	Not a case-control study
1	Antiproteir	Haneda M,	15364166	Not a case-control study
1	Captopril e	Di Paolo S,	12499886	Not a case-control study
1	Role of the	Yoshida H,	7593601	Not a case-control study
1	Synergistic	Wakahara,	17303661	Not a case-control study
1	The effect	Haufroid V,	15167702	Not a case-control study
1	A classifica	Wachsmutl	1016198	Not a renal disease focus
1	A functiona	Wang W, P	22593040	Not a renal disease focus
1	A functiona	Cao Q, Ju X	23209702	Not a renal disease focus
1	A method f	Artemov A,	26320181	Not a renal disease focus

For peer review only

1	A novel hu	Barash U, C	20007507	Not a renal disease focus
1	A novel ME	Braga E, Lo	21423093	Not a renal disease focus
1	A novel rec	Larsson T, Y	15687325	Not a renal disease focus
1	A significan	Wang YH, Y	19686770	Not a renal disease focus
1	A single nu	Hirata H, N	12845675	Not a renal disease focus
1	Aberrant m	Yuan J, Luo	12874023	Not a renal disease focus
1	Abnormal F	Schwienbac	10749116	Not a renal disease focus
1	Absence of	Cooney KA,	10389923	Not a renal disease focus
1	Acetylator	Hein DW, F	3677303	Not a renal disease focus
1	Adult leydi	Carvajal-Ca	16757530	Not a renal disease focus
1	Adult renal	Chapman-F	21665507	Not a renal disease focus
1	Allelic loss	Mochida J,	18554638	Not a renal disease focus
1	Alterations	Audebert M	10987279	Not a renal disease focus
1	Altered ger	Stoyanova	15662135	Not a renal disease focus
1	Alternative	Tomlinson	16288035	Not a renal disease focus
1	Amylase Î±	Jain S, Roy	24225843	Not a renal disease focus
1	An analysis	Webster AI	9758595	Not a renal disease focus
1	An intronic	Chen T, Jac	15382067	Not a renal disease focus
1	Analysis of	BernuÃ©s	9844606	Not a renal disease focus
1	Analysis of	Ricketts C,	19551141	Not a renal disease focus
1	Analysis of	Farker K, Le	10667834	Not a renal disease focus
1	Angiotensi	Cambien F,	9519756	Not a renal disease focus
1	Angiotensi	Stefansson	10855732	Not a renal disease focus
1	Antiepider	Shankar S,	16459265	Not a renal disease focus
1	Apolipopro	Moore LE, I	19808960	Not a renal disease focus
1	Are cell cyc	Mandal RK,	20822933	Not a renal disease focus
1	Associatior	Xiao J, Zhu	22172840	Not a renal disease focus
1	Associatior	Hirata H, O	15319295	Not a renal disease focus
1	Associatior	Mittal RD, I	21668377	Not a renal disease focus
1	Associatior	Mittal RD, S	21700414	Not a renal disease focus
1	Associatior	Kesarwani	18668224	Not a renal disease focus
1	Associatior	Wang W, G	22455395	Not a renal disease focus
1	Associatior	Hashemi M	26475383	Not a renal disease focus
1	Associatior	Srivastava I	22374248	Not a renal disease focus
1	Associatior	Gangwar R,	20380574	Not a renal disease focus
1	Associatior	Srivastava I	21784671	Not a renal disease focus
1	Associatior	Mayer NJ, I	11836444	Not a renal disease focus
1	Base excisi	Mittal RD, I	22019847	Not a renal disease focus
1	Biallelic ina	Hamano K,	11792959	Not a renal disease focus
1	Bladder ca	Srivastava I	20826916	Not a renal disease focus
1	Blastemal e	Natrajan R,	17145858	Not a renal disease focus
1	Breast canc	Truong T, L	24919398	Not a renal disease focus
1	Cancer risk	Garre P, Ma	24061861	Not a renal disease focus
1	Cancer risk	Geoffroy-P	11410879	Not a renal disease focus
1	Cancer risk	Win AK, Cle	21171015	Not a renal disease focus
1	Candidate g	Longuemau	10383153	Not a renal disease focus
1	Caspase 9 a	Gangwar R,	19412632	Not a renal disease focus
1	Characteriz	Bushey RT,	21164388	Not a renal disease focus
1	CHEK2 is a	Cybulski C,	15492928	Not a renal disease focus
1	Chromoph	Abrahams I	15569050	Not a renal disease focus

For peer review only

1	Chromosor	Chung WY,	8842727	Not a renal disease focus
1	Circulating	Deprimo SE	17605814	Not a renal disease focus
1	Clear cell p	Shi SS, Sher	24294381	Not a renal disease focus
1	Clear-cell p	Rohan SM,	21602815	Not a renal disease focus
1	Coding mut	O'Keefe D,	9311733	Not a renal disease focus
1	Combined	Pei J, Feder	20461753	Not a renal disease focus
1	Common g	SchÃ¶del J,	22406644	Not a renal disease focus
1	Comprehen	Kondo K, Ya	11921283	Not a renal disease focus
1	Congenital	Gordeuk VI	14726398	Not a renal disease focus
1	Constant al	Pan CC, Chu	18085521	Not a renal disease focus
1	Constitutio	Roversi G, I	26482194	Not a renal disease focus
1	CYP24A1 ar	Jacobs ET, '	23423976	Not a renal disease focus
1	CYP2E1 ger	Farker K, Le	9760005	Not a renal disease focus
1	Cytochrom	Chang I, Fu	25027399	Not a renal disease focus
1	Cytochrom	Katoh T, Ina	7697828	Not a renal disease focus
1	Defects in t	Perkins DJ,	14966282	Not a renal disease focus
1	Developme	Pang Y, Li W	18778725	Not a renal disease focus
1	Distinct pat	Kito H, Suzu	12442000	Not a renal disease focus
1	DNA repair	Mandal RK,	20070155	Not a renal disease focus
1	Do DNA rep	Gangwar R,	19815090	Not a renal disease focus
1	Dominant-n	Maynard M	17998805	Not a renal disease focus
1	Downregul	Narimatsu	25315157	Not a renal disease focus
1	Duplicatior	Fischer J, P.	9715275	Not a renal disease focus
1	Effect of a t	Huang Z, Lu	26323945	Not a renal disease focus
1	EGF-r gene	Moch H, Sa	9664910	Not a renal disease focus
1	EGFR seque	Franco-Her	17284372	Not a renal disease focus
1	Elevated cc	Takashi M,	2515181	Not a renal disease focus
1	Endothelial	Ziaei SA, Sa	23015399	Not a renal disease focus
1	Epigenetic	Morrissey (	11585766	Not a renal disease focus
1	ERBB4 gen	Hashemi M	27609473	Not a renal disease focus
1	Expression	Vuononvirt	19318497	Not a renal disease focus
1	Expression	Eickelmanr	8313512	Not a renal disease focus
1	Expression	Chung BI, M	14565870	Not a renal disease focus
1	Familial cle	Woodward	10807693	Not a renal disease focus
1	Familial rer	Liu H, Sund	21621909	Not a renal disease focus
1	FHIT gene a	Bugert P, W	9290948	Not a renal disease focus
1	Fluid intake	Wang J, Wu	23632476	Not a renal disease focus
1	Fluorescen	Jiang H, Bai	25119636	Not a renal disease focus
1	Frequent a	Nagy A, Bal	12918080	Not a renal disease focus
1	Functional	Horvath A,	19549888	Not a renal disease focus
1	Functional	Hung MS, L	20625391	Not a renal disease focus
1	Functional	Zhu J, Qin (	20572163	Not a renal disease focus
1	Gain-of-fur	Hirota S, Ni	11276010	Not a renal disease focus
1	Gene-envir	Semenza JC	11323315	Not a renal disease focus
1	Genetic alt	Feng C, Xio	26662507	Not a renal disease focus
1	Genetic an	Smits KM, S	18245539	Not a renal disease focus
1	Genetic an	Williams LH	17145815	Not a renal disease focus
1	Genetic an	Cherubini E	26081799	Not a renal disease focus
1	Genetic Po	Adabi Z, Mc	26319608	Not a renal disease focus
1	Genetic po	Cao Q, Qin	21538578	Not a renal disease focus

For peer review only

1	Genetic po	Habib SL, D	17932460	Not a renal disease focus
1	Genetic Va	Zhao R, Liu	26066055	Not a renal disease focus
1	Genetic vai	Mandal RK,	23054000	Not a renal disease focus
1	Genetic vai	Nuver J, Lu	15900213	Not a renal disease focus
1	Genetic vai	Tan X, Wan	24157827	Not a renal disease focus
1	Genomic ai	Gerlinger M	24487277	Not a renal disease focus
1	Germline B	Ohar JA, Ch	26719535	Not a renal disease focus
1	Germline B	de la Fouch	25080371	Not a renal disease focus
1	Germline B	Popova T, H	23684012	Not a renal disease focus
1	Glutathion	Sweeney C,	10794492	Not a renal disease focus
1	Glutathion	Moore LE, I	17617661	Not a renal disease focus
1	Glutathion	Buzio L, De	14504370	Not a renal disease focus
1	Glutathion	Delbanco E	11218045	Not a renal disease focus
1	GSTM1, GS	ChirilÄƒ DN	26158735	Not a renal disease focus
1	GSTT1, GST	Salinas-SÃ¡	21458313	Not a renal disease focus
1	HABP2 G53	Tomsic J, Fu	26745718	Not a renal disease focus
1	HER2 prote	Sasaki Y, Sa	24551292	Not a renal disease focus
1	hOGG1 Ser	Zhao H, Qin	21166493	Not a renal disease focus
1	Hypoxia-re	Koshikawa	16357173	Not a renal disease focus
1	Identificati	Zatyka M, c	12097293	Not a renal disease focus
1	Immunoex	GiuffrÃ¨ G,	17390038	Not a renal disease focus
1	Impact of C	Farker K, Le	9784018	Not a renal disease focus
1	Impact of g	Ahmad ST,	22054067	Not a renal disease focus
1	Impact of n	Pfab T, Stir	17563539	Not a renal disease focus
1	Impact of N	Srivastava I	23872201	Not a renal disease focus
1	Impact of r	Gangawar I	19242824	Not a renal disease focus
1	Increased e	Wang WH,	8910385	Not a renal disease focus
1	Increased e	Brown LF, E	8238242	Not a renal disease focus
1	Influence o	Kesarwani	20804486	Not a renal disease focus
1	Influence o	BrÃ¼ning T	9285043	Not a renal disease focus
1	Influence o	Marques I,	23645041	Not a renal disease focus
1	Influence o	Gangwar R,	19041121	Not a renal disease focus
1	Inhibition k	Kotelevets	9603913	Not a renal disease focus
1	Inhibition c	Kondo K, Kl	12086860	Not a renal disease focus
1	Inhibition c	Montgome	9797175	Not a renal disease focus
1	Insertion/d	de Martino	21477733	Not a renal disease focus
1	Int7G24A v	Chen T, Jac	16428477	Not a renal disease focus
1	Investigatir	Uyar OA, Ka	21308654	Not a renal disease focus
1	Isocitrate d	Rakheja D,	21225914	Not a renal disease focus
1	JAK3 in clea	de Martino	21868263	Not a renal disease focus
1	KIT express	Petit A, Cas	15105658	Not a renal disease focus
1	Lack of ass	Piccoli MF,	17980059	Not a renal disease focus
1	LMP2, a no	Zheng G, Cl	22705098	Not a renal disease focus
1	Loss of BAF	Mochel MC	25972334	Not a renal disease focus
1	Loss of het	Drake KM,	19789318	Not a renal disease focus
1	Loss of het	Sithananda	2566144	Not a renal disease focus
1	Loss of het	Bosnar MH	9341897	Not a renal disease focus
1	Mapping o	Tang W, Fu	22228101	Not a renal disease focus
1	Matrix met	Awakura Y,	16466849	Not a renal disease focus
1	MDM2 SNF	Hirata H, H	17634539	Not a renal disease focus

For peer review only

1	Meningiom	Zang KD.	11528114	Not a renal disease focus
1	Metanephr	Mantoan P	23551615	Not a renal disease focus
1	MicroRNA	Wei H, Ke H	22517515	Not a renal disease focus
1	Microsatell	Machin P, (	12139636	Not a renal disease focus
1	Mixed epitl	Pierson CR,	11381370	Not a renal disease focus
1	Mucinous t	Shen SS, Rc	17240302	Not a renal disease focus
1	Mutations	Barnabas N	11967908	Not a renal disease focus
1	Mutations	Gallou C, Jc	10408776	Not a renal disease focus
1	Novel soma	Yang K, Lin	10454237	Not a renal disease focus
1	Occupatior	Moore LE, I	20663906	Not a renal disease focus
1	Oncocytic p	Mai KT, Kol	18251779	Not a renal disease focus
1	Oncocytic p	Xia QY, Rac	23826421	Not a renal disease focus
1	p53 mutati	Imai Y, Strc	7824511	Not a renal disease focus
1	Patients wi	Skeldon SC,	22883484	Not a renal disease focus
1	Phase 1 tria	Puzanov I, S	25294187	Not a renal disease focus
1	Phosphodie	Faucz FR, H	20881257	Not a renal disease focus
1	Point muta	Cairns P, Ev	9671402	Not a renal disease focus
1	Polymorph	Mandal RK,	20590474	Not a renal disease focus
1	Polymorph	Du Y, Su T,	23069849	Not a renal disease focus
1	Polymorph	George GP,	21396853	Not a renal disease focus
1	Polymorph	Mandal RK,	22382185	Not a renal disease focus
1	Polymorph	Deckers IA,	24978482	Not a renal disease focus
1	Polymorph	Smits KM, S	17982751	Not a renal disease focus
1	Polymorph	Sasaki M, T	15041720	Not a renal disease focus
1	Prevalence	van Houwe	15932632	Not a renal disease focus
1	Progestero	Mai KT, Tec	18081814	Not a renal disease focus
1	Re-assessm	WiesenhÃ¼	17479278	Not a renal disease focus
1	Recurrent s	Torrezan G	24909261	Not a renal disease focus
1	Reduced ex	Thompson	8971182	Not a renal disease focus
1	Relationshi	Chen J, Che	22296350	Not a renal disease focus
1	Relevance o	Schraml P,	12937142	Not a renal disease focus
1	Renal cell c	De Martinc	20083259	Not a renal disease focus
1	Renal cell c	Hughson M	8959640	Not a renal disease focus
1	Renal cell c	Lv C, Bai Z,	26191297	Not a renal disease focus
1	Renal cell c	Karami S, B	18566013	Not a renal disease focus
1	Renin in blo	Taylor GM,	2450464	Not a renal disease focus
1	Replicative	Singh V, Jai	25218484	Not a renal disease focus
1	Retrospect	Singh N, Sa	25913740	Not a renal disease focus
1	Rhabdoid t	Sredni ST, T	25494491	Not a renal disease focus
1	Risk factor	Wang G, Hc	22000673	Not a renal disease focus
1	Risk of rena	Ahmad ST,	22281432	Not a renal disease focus
1	Role of MN	Srivastava I	20574775	Not a renal disease focus
1	Role of the	Hammerscl	17143471	Not a renal disease focus
1	RTK/ERK pa	Chen Y, Xin	24223781	Not a renal disease focus
1	Somatic ina	Prowse AH,	9106522	Not a renal disease focus
1	Somatic mu	Alimov A, L	10628321	Not a renal disease focus
1	Somatic mu	Di Renzo M	10734314	Not a renal disease focus
1	Structural k	Forman JR,	19408298	Not a renal disease focus
1	Subtype-sp	Williams RI	20332316	Not a renal disease focus
1	Telomerase	Dahse R, Fi	10504477	Not a renal disease focus

For peer review only

1	TERT Prom	de Biase D,	26148423	Not a renal disease focus
1	The beta-su	Glaser T, Le	3014343	Not a renal disease focus
1	The E3 liga	Zhang L, Ar	17694067	Not a renal disease focus
1	The extrac	Blokhuis M	18049911	Not a renal disease focus
1	The Int7G2	Castillejo A	19930569	Not a renal disease focus
1	The polym	Qin C, Cao	21778301	Not a renal disease focus
1	The polym	Huang CY,	21982800	Not a renal disease focus
1	The pVHL-a	Clifford SC,	11526493	Not a renal disease focus
1	The relatio	Peng CT, Ch	12469224	Not a renal disease focus
1	The role of	Chang WS,	22993329	Not a renal disease focus
1	Trisomy 7-l	Zhuang Z, F	9731534	Not a renal disease focus
1	Tumor-spe	Zhao Q, Cal	23444225	Not a renal disease focus
1	Two single	Wang WC,	25217002	Not a renal disease focus
1	Uncommon	Brennan P,	17517688	Not a renal disease focus
1	Variants in	Qin C, Cher	25239121	Not a renal disease focus
1	VHL alterat	Brauch H, V	10766184	Not a renal disease focus
1	VHL and HI	Lessi F, Ma	24446253	Not a renal disease focus
1	Vitamin D r	Arjumand V	21931993	Not a renal disease focus
1	Von Hippel	Moore LE, I	22022277	Not a renal disease focus
1	Whole-exo	Wang L, Tsu	22156295	Not a renal disease focus
1	XRCC3 Thr2	Loghin A, B	26682510	Not a renal disease focus
1	A biallelic g	Nicod J, Bru	12788845	Not a renal disease focus
1	A complem	Funato M,	24906628	Not a renal disease focus
1	A functiona	Luo F, Wan	19635985	Not a renal disease focus
1	A functiona	Svensson-F	21052022	Not a renal disease focus
1	A genome-	Ferrari R, G	26154020	Not a renal disease focus
1	A homoalle	Greenberg	8406453	Not a renal disease focus
1	A human p	Garrone NF	19364400	Not a renal disease focus
1	A labor- an	Tavira B, G	24830959	Not a renal disease focus
1	A low-frequ	Waterwort	25164947	Not a renal disease focus
1	A naturally	Fouladkou	15140763	Not a renal disease focus
1	A novel mis	Kovacs P, H	15220220	Not a renal disease focus
1	A novel ph	Tham E, Ek	25966638	Not a renal disease focus
1	A novel STX	Linglart A,	15800843	Not a renal disease focus
1	A pilot stud	Brophy DF,	19210273	Not a renal disease focus
1	A polymorp	MohÃ¡s M,	19834870	Not a renal disease focus
1	A study on	Chmaisse H	19861867	Not a renal disease focus
1	A two-stag	Kuo TY, Kar	22534794	Not a renal disease focus
1	A WNK4 ge	Mendes AI,	21236712	Not a renal disease focus
1	Abnormal h	Drenth JP,	15057895	Not a renal disease focus
1	Absence of	Fukumoto	8675577	Not a renal disease focus
1	ACE and PC	de Azevedo	12126783	Not a renal disease focus
1	ACE gene in	Dell'omo G	16889537	Not a renal disease focus
1	ADAM33: a	Holgate ST,	16257631	Not a renal disease focus
1	Admixture	Bercovici S,	21385031	Not a renal disease focus
1	Adrenal hy	Horvath A,	17178847	Not a renal disease focus
1	Allelic varia	RodrÃguez	15681296	Not a renal disease focus
1	Allelic varia	Zschocke J,	16965804	Not a renal disease focus
1	Alleviating	Grabczyk E	11121484	Not a renal disease focus
1	Allotypes o	Davidson J	6399879	Not a renal disease focus

For peer review only

1	Alpha-1-pro	O'Donoghu	8296629	Not a renal disease focus
1	alpha-addu	Clark CJ, Da	11116113	Not a renal disease focus
1	Alu-Alu rec	Franke G, B	19280651	Not a renal disease focus
1	AMP-activa	Mishra R, C	18250161	Not a renal disease focus
1	An insertio	Rahmutula	10770265	Not a renal disease focus
1	An intron 4	Yokoyama	10867538	Not a renal disease focus
1	Analyses of	Berge KE, B	9298743	Not a renal disease focus
1	Analysis of	Zhu J, Frosc	7641206	Not a renal disease focus
1	Analysis of	Farook VS,	12359146	Not a renal disease focus
1	Analysis of	Takatani R,	26479409	Not a renal disease focus
1	Analysis of	Peros E, Ge	15754029	Not a renal disease focus
1	Analysis of	Hamon Y, L	26607765	Not a renal disease focus
1	Angiotensi	Nagi DK, M	8582133	Not a renal disease focus
1	Angiotensi	Miller JA, S	8995725	Not a renal disease focus
1	Angiotensi	VaskÅ¯ A, S	9607178	Not a renal disease focus
1	Angiotensi	GutiÃ©rrez	9258285	Not a renal disease focus
1	Angiotensi	Mansoor Q	22607040	Not a renal disease focus
1	Angiotensi	Negi VS, De	25957879	Not a renal disease focus
1	Angiotensi	Catarsi P, R	16002416	Not a renal disease focus
1	Angiotensi	Pedersen-B	19820429	Not a renal disease focus
1	Angiotensi	Singh M, Si	27030424	Not a renal disease focus
1	ANGIOTENS	LACHURIE,	7796503	Not a renal disease focus
1	Angiotensi	Al-Awadhi	17631741	Not a renal disease focus
1	Angiotensi	Prkacin I, N	11505631	Not a renal disease focus
1	Are the ang	Dikmen M,	16791358	Not a renal disease focus
1	Assessmen	He F, Liu M	27793583	Not a renal disease focus
1	Association	Lewis JP, Pa	18443202	Not a renal disease focus
1	Association	Holstein A,	15963101	Not a renal disease focus
1	Association	Matsubara	10688808	Not a renal disease focus
1	Association	Boomsma I	15830186	Not a renal disease focus
1	Association	Sprovieri SI	16047641	Not a renal disease focus
1	Association	Song SB, Jin	21342026	Not a renal disease focus
1	Association	Liu H, Zhao	18235092	Not a renal disease focus
1	Association	Orozco G, S	15641066	Not a renal disease focus
1	Association	Bahramali I	26861937	Not a renal disease focus
1	Association	Akai Y, Sato	10099886	Not a renal disease focus
1	Association	Alsafar H, H	26491214	Not a renal disease focus
1	Association	Rabbani M.	18711292	Not a renal disease focus
1	Association	Nikzamir A,	18154415	Not a renal disease focus
1	Association	RodrÃ-guez	11345362	Not a renal disease focus
1	Association	Glorioso N,	17446437	Not a renal disease focus
1	Association	Moholisa R	23730990	Not a renal disease focus
1	Association	Lin CH, Che	24433515	Not a renal disease focus
1	Association	Zhou X, Wa	20406624	Not a renal disease focus
1	Association	Omori S, Ta	18162508	Not a renal disease focus
1	Association	Elmonem M	26589000	Not a renal disease focus
1	Association	Bochud M,	16612255	Not a renal disease focus
1	Association	Vague P, Du	9165217	Not a renal disease focus
1	Association	Kamide K, I	17143180	Not a renal disease focus
1	Association	Ma Y, Ni W	21332417	Not a renal disease focus

For peer review only

1	Associatior	Sucker C, H	17327284	Not a renal disease focus
1	Associatior	Cho ML, Ch	12765304	Not a renal disease focus
1	Associatior	Russo CJ, M	16103266	Not a renal disease focus
1	Associatior	Meng, N; Z	25359286	Not a renal disease focus
1	Associatior	Moez P, So	21818561	Not a renal disease focus
1	Associatior	Conen D, G	18698212	Not a renal disease focus
1	Associatior	Schwab M,	18209482	Not a renal disease focus
1	Associatior	Cakir OO, Y	26945655	Not a renal disease focus
1	Associatior	Hayakawa	17161225	Not a renal disease focus
1	Associatior	Zhou H, Mc	23303384	Not a renal disease focus
1	Associatior	Li X, An J, G	20540812	Not a renal disease focus
1	Associatior	Vazgiourak	17971359	Not a renal disease focus
1	Associatior	Busjahn A,	15107590	Not a renal disease focus
1	Associatior	Kawasaki A	20849588	Not a renal disease focus
1	Associatior	Kawasaki A	20617138	Not a renal disease focus
1	Autosomal	Magen D, C	25560765	Not a renal disease focus
1	Autosomal	JÃ¼ppner H	16831926	Not a renal disease focus
1	Azathioprir	SchÃ¼tz E,	8721407	Not a renal disease focus
1	Bimodal di:	Mason CC,	21299892	Not a renal disease focus
1	Biological r	Haufroid V,	9342622	Not a renal disease focus
1	Bobby Sox	Choi YA, Se	24885382	Not a renal disease focus
1	BRAF muta	Choueiri Tk	22727996	Not a renal disease focus
1	Calpain-10	Buraczynsk	23021796	Not a renal disease focus
1	C-antineuti	Griffith ME	8671812	Not a renal disease focus
1	Carotid inti	NergizoÄŸl	10493570	Not a renal disease focus
1	CCL18: a ur	Boot RG, V	16736095	Not a renal disease focus
1	cGMP-depe	Citterio L, F	24060892	Not a renal disease focus
1	Characteriz	Mark MR, C	8621659	Not a renal disease focus
1	Cholesteryl	Meguro S,	11369008	Not a renal disease focus
1	Class II HLA	Gilchrist FC	11393660	Not a renal disease focus
1	Clinical anc	Lombardi G	24064758	Not a renal disease focus
1	Clinical anc	Gomy I, Mc	20567917	Not a renal disease focus
1	Clinical anc	Chacon-Cai	20447124	Not a renal disease focus
1	Clinical imp	van Woerd	15253729	Not a renal disease focus
1	Clinical phe	Han G, Wai	23946278	Not a renal disease focus
1	Clinical pre	Erlic Z, Ryb	19825962	Not a renal disease focus
1	Common v:	Sakiyama M	24882840	Not a renal disease focus
1	Compleme	da Rosa Uti	16164698	Not a renal disease focus
1	Comprehei	Monico CG	17460142	Not a renal disease focus
1	Concentrat	Castegnaro	1820331	Not a renal disease focus
1	Contributic	Mondorf U	9524045	Not a renal disease focus
1	Contributic	Lasram K, E	24636221	Not a renal disease focus
1	Control of :	Wyatt RJ, F	6445926	Not a renal disease focus
1	Crystal stru	Lietha D, Cl	11597998	Not a renal disease focus
1	CYP1B1 mu	Campos-M	19234632	Not a renal disease focus
1	CYP3A5 gei	Fromm MF	16141800	Not a renal disease focus
1	CYP4F2 ger	Deng S, Zhu	20227456	Not a renal disease focus
1	Cystinosin	Ivanova EA	27083281	Not a renal disease focus
1	Cytochrom	Yen JH, Che	14611903	Not a renal disease focus
1	Deletion pc	Gharavi AG	8677872	Not a renal disease focus

For peer review only

1	Delineatior	Horn D, Wi	24129430	Not a renal disease focus
1	Detection c	Pirulli D, Gi	11699734	Not a renal disease focus
1	Detection c	Matsumotc	11106834	Not a renal disease focus
1	Developme	Coutton C,	20043897	Not a renal disease focus
1	Differential	Mulivor RA	686677	Not a renal disease focus
1	Differential	Simon P, Sc	17982254	Not a renal disease focus
1	Distinct pat	Liu J, Nealo	15537666	Not a renal disease focus
1	Distributior	Diamantop	10821349	Not a renal disease focus
1	Distributior	Hussain N,	23448612	Not a renal disease focus
1	Distributior	Zhang L, Ja	7731160	Not a renal disease focus
1	DNase II pc	Shin HD, Pa	15723160	Not a renal disease focus
1	Does comp	Kavanagh D	16061287	Not a renal disease focus
1	Early (2008	Thomas CP	24207018	Not a renal disease focus
1	Effect of sy	Mangos GJ,	17132537	Not a renal disease focus
1	Effects of a	Kulah E, Du	17625392	Not a renal disease focus
1	Elevated ur	Clausen P, .	10872702	Not a renal disease focus
1	Endothelial	Tang FY, Xi	19965945	Not a renal disease focus
1	Endothelial	Ksiazek P, \	14580231	Not a renal disease focus
1	Endothelial	Tantawy A/	25699607	Not a renal disease focus
1	Enhanced a	Nakano T, (	11980680	Not a renal disease focus
1	eNOS gene	Bazzaz JT, /	20565248	Not a renal disease focus
1	Epistatic ro	Voskarides	23516419	Not a renal disease focus
1	Evaluation	Rumsby G,	15327387	Not a renal disease focus
1	Evidence o	Tsukada T,	9535806	Not a renal disease focus
1	Examinatio	Friend LR, I	8800585	Not a renal disease focus
1	Exome seq	Kiando SR,	26147384	Not a renal disease focus
1	Exome-wid	Cutcutache	25597018	Not a renal disease focus
1	Exploring g	Castro-SÃ¡r	26082521	Not a renal disease focus
1	Financial in	Bilgel F, Ga	26218985	Not a renal disease focus
1	Frequencie	Zee RY, Ber	7882587	Not a renal disease focus
1	Functional	Permana P.	10873397	Not a renal disease focus
1	Functional	Hoogendoo	15221787	Not a renal disease focus
1	Functional	Montes T, T	19255449	Not a renal disease focus
1	Functional	Stec DE, Ro	17341693	Not a renal disease focus
1	Functional	Coto E, Tav	20617557	Not a renal disease focus
1	Functional	Lumb MJ, D	10960483	Not a renal disease focus
1	Further stu	Danpure CJ	3416563	Not a renal disease focus
1	G protein b	Thibaudin I	15200440	Not a renal disease focus
1	GBPI, a nov	Liu QR, Zha	12974676	Not a renal disease focus
1	Gender diff	Hokimoto S	25264752	Not a renal disease focus
1	Gene struc	FernÃ¡ndez	10373324	Not a renal disease focus
1	Gene-gene	Zhou XJ, Lu	21905002	Not a renal disease focus
1	Genetic an	AgÃºndez J	8946479	Not a renal disease focus
1	Genetic cha	Ruiz-Lloren	14722919	Not a renal disease focus
1	Genetic dia	Morey M, (	21902834	Not a renal disease focus
1	Genetic dis	Westra D, \	20106822	Not a renal disease focus
1	Genetic po	Hovnik T, D	19752172	Not a renal disease focus
1	Genetic po	Ã–ktem F, /	24841661	Not a renal disease focus
1	Genetic po	Olivieri O, T	10567188	Not a renal disease focus
1	Genetic pre	Tousoulis D	23479071	Not a renal disease focus

For peer review only

1	Genetic risl	Missouris C	8821841	Not a renal disease focus
1	Genetic risl	van Onna M	15326089	Not a renal disease focus
1	Genetic vai	Jiang DK, M	25802187	Not a renal disease focus
1	Genetic vai	Szperl M, D	19043368	Not a renal disease focus
1	Genetic vai	Sanghera D	17900266	Not a renal disease focus
1	Genetic vai	Bespalova I	22322903	Not a renal disease focus
1	Genetic vai	Jin HS, Hon	20090362	Not a renal disease focus
1	Genetics, c	Ghiggeri GI	12500226	Not a renal disease focus
1	Genome-w	Matsuo H,	25646370	Not a renal disease focus
1	Genotype a	Tavilani H,	25264968	Not a renal disease focus
1	Genotype-p	Vikkath N,	25952756	Not a renal disease focus
1	Genotype-p	WittstrÃ¶n	24555745	Not a renal disease focus
1	Genotyping	Warpeha K	10506586	Not a renal disease focus
1	Germline n	Angrist M,	8896568	Not a renal disease focus
1	Germline n	Salomon R,	8896569	Not a renal disease focus
1	Germline V	Stanojevic I	17688370	Not a renal disease focus
1	Glutathione	Mohamme	26773925	Not a renal disease focus
1	Glutathione	Yang Y, Kac	15492856	Not a renal disease focus
1	Glutathione	Delles C, Pa	18551009	Not a renal disease focus
1	G-protein b	Beige J, Rin	10978395	Not a renal disease focus
1	G-Protein b	Beige J, Ho	10205246	Not a renal disease focus
1	G-protein b	Zagradisnik	15337465	Not a renal disease focus
1	G-protein r	Muskalla A	25301854	Not a renal disease focus
1	GSTM1, GS	Mittal RD, I	19407363	Not a renal disease focus
1	Haplotypes	Pullmann R	18240231	Not a renal disease focus
1	Hepatocyst	Overton JD	26671672	Not a renal disease focus
1	High aldost	Escher G, C	19151144	Not a renal disease focus
1	Higher freq	Barris-Olive	22796398	Not a renal disease focus
1	Higher thrc	Bavbek N, (	24580410	Not a renal disease focus
1	Homozygoi	Moulson Cl	16297189	Not a renal disease focus
1	Human G(s	Makita N, S	17962410	Not a renal disease focus
1	Human GFI	Angrist M, .	9545641	Not a renal disease focus
1	Hyperchole	Gragnoli C.	21554682	Not a renal disease focus
1	Identificati	Kokubo Y, I	15309683	Not a renal disease focus
1	Identificati	Stoetzel C,	17160889	Not a renal disease focus
1	Identificati	Mornet E,	9781036	Not a renal disease focus
1	Identificati	Tyynismaa	10737991	Not a renal disease focus
1	Identificati	Schmitz B,	25210049	Not a renal disease focus
1	Identificati	Purdue PE,	1703535	Not a renal disease focus
1	Identificati	Yik WY, Ste	19105186	Not a renal disease focus
1	Identificati	Stopkova P	15121481	Not a renal disease focus
1	Identificati	Kunishima	11776386	Not a renal disease focus
1	IFT27, enco	Aldahmesh	24488770	Not a renal disease focus
1	Immunolog	Wise PJ, Da	2443389	Not a renal disease focus
1	Impact of c	Miki I, Aoya	12544691	Not a renal disease focus
1	Impact of g	Kamei H, M	23153768	Not a renal disease focus
1	Impact of t	Ristau T, Pa	24675670	Not a renal disease focus
1	In vitro trai	Prazeres H,	21551259	Not a renal disease focus
1	Increased a	Suehiro T, I	15164285	Not a renal disease focus
1	Increased D	Morrison C	11381371	Not a renal disease focus

For peer review only

1	Increased e	Yamani MH	17097490	Not a renal disease focus
1	Increased f	Galbusera I	10397728	Not a renal disease focus
1	Increased f	Onizuka M,	16044138	Not a renal disease focus
1	Increased r	Abdgawad	16792675	Not a renal disease focus
1	Independe	Morris BJ, Z	8104754	Not a renal disease focus
1	Inducible n	Martin J, Pa	15940772	Not a renal disease focus
1	Influence o	Yanagimacl	20030680	Not a renal disease focus
1	INPPL1 is a	HyvÃ¶nen	22486725	Not a renal disease focus
1	Insight into	Habib SL.	19265534	Not a renal disease focus
1	Integrated	Sobek AK, E	23010210	Not a renal disease focus
1	Interaction	Wang W, S	23190752	Not a renal disease focus
1	Intron 4 po	Lee YH, Kim	15119548	Not a renal disease focus
1	IRAK1b, a r	Jensen LE, W	11397809	Not a renal disease focus
1	Is factor V I	Krieg S, Stu	16411392	Not a renal disease focus
1	Is polymor	Gluba A, Ba	19505886	Not a renal disease focus
1	Isolation ar	LÃ©vesque	9295060	Not a renal disease focus
1	Klotho gen	Xu C, Song	23756195	Not a renal disease focus
1	Lack of ass	Dell'Omo G	17563560	Not a renal disease focus
1	Lack of ass	Orozco G, M	18078626	Not a renal disease focus
1	Lack of ass	Tangri N, A	21565945	Not a renal disease focus
1	Lack of gen	Wang J, Liu	26307914	Not a renal disease focus
1	Late-onset	Mehler M,	9923	Not a renal disease focus
1	Leukocyte I	Raghavan S	6770675	Not a renal disease focus
1	Lipoprotein	Laschkolnig	24760552	Not a renal disease focus
1	Loss of fun	Stiburek L,	19295170	Not a renal disease focus
1	LRRK2 G23	Gopalai AA	25243190	Not a renal disease focus
1	Lys(173)Arg	Komiya I, Y	10720581	Not a renal disease focus
1	Manganese	Tugcu V, Oz	17628794	Not a renal disease focus
1	Manganese	Mohamme	24819633	Not a renal disease focus
1	Matrix met	Bahrehmar	25416694	Not a renal disease focus
1	Matrix met	Buraczynsk	26025700	Not a renal disease focus
1	Mechanism	Shimamotc	2676859	Not a renal disease focus
1	Missense n	Moon YW,	11007037	Not a renal disease focus
1	MKS1 regu	Slaats GG, I	26490104	Not a renal disease focus
1	Molecular a	Udager AM	25602792	Not a renal disease focus
1	Molecular I	Lovati E, Fe	10523024	Not a renal disease focus
1	Molecular I	Bertelli M,	15505382	Not a renal disease focus
1	Molecular c	Hou XH, So	26413058	Not a renal disease focus
1	Molecular c	Wolford JK,	10607908	Not a renal disease focus
1	Molecular c	GÃ¼rntke S	25224765	Not a renal disease focus
1	Multiorgan	Notari S, M	20098730	Not a renal disease focus
1	Mutation a	Assink K, Sc	12753286	Not a renal disease focus
1	Mutation s	Wang SH, W	22447252	Not a renal disease focus
1	Mutational	Kinoshita Y	22577109	Not a renal disease focus
1	Mutations	Li A, Davila	12529853	Not a renal disease focus
1	Mutations	Siemiatkow	24084495	Not a renal disease focus
1	Mutations	Calado RT,	17478638	Not a renal disease focus
1	Mutations	Tucker EJ, W	24385928	Not a renal disease focus
1	MYH9-relat	Seri M, Pec	12792306	Not a renal disease focus
1	N-acetyltra	Juhanson P	18402670	Not a renal disease focus

For peer review only

1	Natural his	Deegan PB,	16227523	Not a renal disease focus
1	New insigh	Hooker E, E	22982678	Not a renal disease focus
1	NFkappaB a	Romzova M	17002901	Not a renal disease focus
1	N-glycosyla	Riedl E, Koe	20460427	Not a renal disease focus
1	No associat	Wolf G, Par	12147803	Not a renal disease focus
1	No associat	Yildiz A, Ak	10657713	Not a renal disease focus
1	No associat	Klauke B, W	18582803	Not a renal disease focus
1	No associat	Lieb W, Bol	16822233	Not a renal disease focus
1	Nosocomia	Naseer U, N	17295678	Not a renal disease focus
1	Novel miss	Zhang C, Zh	22934535	Not a renal disease focus
1	Novel muta	Smith SJ, Ru	10320521	Not a renal disease focus
1	Novel muta	Yuan ZF, W	26253413	Not a renal disease focus
1	Novel NEK8	Grampa V,	26967905	Not a renal disease focus
1	Origin of ra	Baxter GD,	2558964	Not a renal disease focus
1	Overexpres	Coulter-Ma	15802217	Not a renal disease focus
1	Oxidative s	CalÃ² LA, Pa	12897089	Not a renal disease focus
1	Paraoxonas	Zhang B, Et	14579940	Not a renal disease focus
1	Paraoxonas	Bhatnagar	22854640	Not a renal disease focus
1	Paraoxonas	Murata M,	15270786	Not a renal disease focus
1	Paraverteb	Lee SH, Aga	22261628	Not a renal disease focus
1	Peripheral	Thomas GN	14632699	Not a renal disease focus
1	Persistence	Dudding BA	5682941	Not a renal disease focus
1	Pharmacolo	Miyata N, S	25237136	Not a renal disease focus
1	Phenotypic	Travaglini L	23386033	Not a renal disease focus
1	Phosphoryl	Dennis PB,	9614086	Not a renal disease focus
1	Polymorph	Houldswor	25858271	Not a renal disease focus
1	Polymorph	Wolford JK,	11513557	Not a renal disease focus
1	Polymorph	Ikeda Y, Su	15384959	Not a renal disease focus
1	Position of	Pecci A, Pa	18059020	Not a renal disease focus
1	PPP2R2B C	Chen CM, H	18484086	Not a renal disease focus
1	Predicting	Whitfield M	17084425	Not a renal disease focus
1	Pre-treatm	Ahmed WH	23538996	Not a renal disease focus
1	Prevalence	Nazareth N	25994082	Not a renal disease focus
1	Proinflamm	Borgmann	12722027	Not a renal disease focus
1	Proteasom	Gragnoli C.	22934761	Not a renal disease focus
1	Protein exp	Lim SD, Kim	17685929	Not a renal disease focus
1	Proteinase	Gencik M, I	11115080	Not a renal disease focus
1	PYGM expr	de Luna N,	25240406	Not a renal disease focus
1	Q222R poly	Panneer D,	23963431	Not a renal disease focus
1	Random m	Nickerson I	11241843	Not a renal disease focus
1	Reduced ex	Carter A, Ba	2890163	Not a renal disease focus
1	Regulatory	Oczos J, Gri	22956172	Not a renal disease focus
1	Relationshi	Fu Y, Katsu	15894833	Not a renal disease focus
1	Relationshi	Heltianu C,	15784171	Not a renal disease focus
1	Relationshi	Tripi LM, M	16729301	Not a renal disease focus
1	Renal ACE i	Mizuiri S, Y	9469501	Not a renal disease focus
1	Renal chan	Marre M, B	10082486	Not a renal disease focus
1	Renal onco	Hes O, Mich	18196270	Not a renal disease focus
1	Renal outco	Molad Y, G	11071585	Not a renal disease focus
1	Renin-angi	Parsa A, Lo	15789057	Not a renal disease focus

For peer review only

1	Renin-angi	BuraczyÅ„s	14502296	Not a renal disease focus
1	Renovascul	Pizzolo F, M	14718831	Not a renal disease focus
1	Resequenc	Coto E, Reg	22765922	Not a renal disease focus
1	Role of P45	BrÃ¸sen K, 2	2060250	Not a renal disease focus
1	Role of PTP	LÃ³pez-Me	26458874	Not a renal disease focus
1	S-adenosyl	Feld JJ, Mo	20854821	Not a renal disease focus
1	Selected AC	Benhaj Mb	21612638	Not a renal disease focus
1	Serum aryl	Inoue M, Su	11092501	Not a renal disease focus
1	Serum extr	Kimura F, H	12663605	Not a renal disease focus
1	Serum para	Ikeda Y, Su	9591753	Not a renal disease focus
1	Serum para	Atar A, Gec	26795139	Not a renal disease focus
1	Single nucl	Basson JJ, c	25063733	Not a renal disease focus
1	Single-nucl	Nishijima T	25151207	Not a renal disease focus
1	SIRT1 gene	Weyrich P,	19014491	Not a renal disease focus
1	Slight asso	Capoluong	16806146	Not a renal disease focus
1	SNP Varian	Coulter-Ma	25854853	Not a renal disease focus
1	Soluble FLT	Jin J, Sison	23063127	Not a renal disease focus
1	Somatic mu	Weber A, H	22573489	Not a renal disease focus
1	Spectrum c	Guggilla SR	25982861	Not a renal disease focus
1	Spondyloer	Briggs TA, F	26951490	Not a renal disease focus
1	Structural c	Hall AV, An	7528745	Not a renal disease focus
1	Structure o	Hocking HG	18252712	Not a renal disease focus
1	Studies of a	Rousseaux	6215190	Not a renal disease focus
1	Synergistic	Barlassina (	10720960	Not a renal disease focus
1	System-Wi	Zhang W, V	26949039	Not a renal disease focus
1	T-cell recep	Niven MJ, C	1969400	Not a renal disease focus
1	Testing of p	Schoenfeld	24667567	Not a renal disease focus
1	The action	Smith NJ, S	19398560	Not a renal disease focus
1	The associa	Cikomola JC	27461879	Not a renal disease focus
1	The associa	Atay AE, Ak	24403014	Not a renal disease focus
1	The associa	Safarinejad	20505681	Not a renal disease focus
1	The calcine	Di Sole F, B	19556366	Not a renal disease focus
1	The Captop	Hansson L,	9495662	Not a renal disease focus
1	The conser	Mesa-Torre	24957194	Not a renal disease focus
1	The effect c	Ward NC, C	24984178	Not a renal disease focus
1	The epiden	Price LB, Jo	24345742	Not a renal disease focus
1	The gene e	Cramer SD,	10484776	Not a renal disease focus
1	The inhibit	Wan KF, Sa	12624098	Not a renal disease focus
1	The Leu262	Hatunic M,	19185381	Not a renal disease focus
1	The major a	Coulter-Ma	15963748	Not a renal disease focus
1	The non-m	Freedman I	20523037	Not a renal disease focus
1	The presen	GÃ¼ngÃ¶r	21332339	Not a renal disease focus
1	The Preval	Dreisbach A	16202848	Not a renal disease focus
1	The PTPN2	Jagiello P, A	16320352	Not a renal disease focus
1	The R620W	Reddy MV,	16052172	Not a renal disease focus
1	The rapid e	Nguyen TM	14665637	Not a renal disease focus
1	The role of	Safarinejad	20586099	Not a renal disease focus
1	The role of	Åžener EF,	24936541	Not a renal disease focus
1	The role of	Lee HT, Lin	25671815	Not a renal disease focus
1	The role of	Gluba A, Pi	19939821	Not a renal disease focus

For peer review only

For peer review only - http://bmjopen.bmj.com/site/about/guidelines.xhtml

1	The role of	Xu F, Morir	15139850	Not a renal disease focus
1	The role of	Celi FS, Cop	18349068	Not a renal disease focus
1	The sirtuin	Kanda T, Sa	26388050	Not a renal disease focus
1	The VHL sh	Liu Y, Yang	27196060	Not a renal disease focus
1	Three nove	Kamide K, T	15110905	Not a renal disease focus
1	Three nove	Fukushima	24088670	Not a renal disease focus
1	Three nove	Jap TS, Chiu	21293852	Not a renal disease focus
1	Thrombin a	Donmez A,	15668188	Not a renal disease focus
1	Thromboti	Girolami A,	21842307	Not a renal disease focus
1	TMPRSS6 r	Pei SN, Ma	24782651	Not a renal disease focus
1	TNFAIP3 ge	Kim SK, Cho	24489017	Not a renal disease focus
1	Trisomy 4 l	Beghini A, I	10812167	Not a renal disease focus
1	Tropical cal	Derikx MH,	19404200	Not a renal disease focus
1	Ubiquitin li	Shen Y, Bal	16979136	Not a renal disease focus
1	Use of a Hi	Chang DC, I	26606528	Not a renal disease focus
1	Variable nu	Aoi N, Som	15785005	Not a renal disease focus
1	Variant scr	Prochazka I	12490143	Not a renal disease focus
1	Variants in	Wang Y, W	26448018	Not a renal disease focus
1	Vascular er	Chen WC, C	12719950	Not a renal disease focus
1	Vascular er	MaÅ‚kiewic	27367286	Not a renal disease focus
1	VEGF-A anc	Amosco MI	27668980	Not a renal disease focus
1	VHL c.505 T	Bender BU,	11483638	Not a renal disease focus
1	VHL P25L is	Pettman RI	16884327	Not a renal disease focus
1	von Hippel-	Hoffman M	11331612	Not a renal disease focus
1	When shou	Erlic Z, Neu	19067729	Not a renal disease focus
1	Whole Exo	Ma SL, Veg	26107949	Not a renal disease focus
1	[A search fo	Voron'ko O	16080611	Not English or Spanish
1	[A study or	Nishina M.	9014479	Not English or Spanish
1	[Acquired r	Liu Z, Gong	12126523	Not English or Spanish
1	[Activation	Braga EA, L	17086988	Not English or Spanish
1	[Angiotensi	Krajina-Anc	23120809	Not English or Spanish
1	[Angiotensi	Guan T, Liu	10436947	Not English or Spanish
1	[Arterial hy	Oko A, Åoc	14974362	Not English or Spanish
1	[Associatio	Fu LL, Lin Y	22487833	Not English or Spanish
1	[Associatio	Wang L, Pa	10923445	Not English or Spanish
1	[Associatio	Qiu MY, Xie	25815490	Not English or Spanish
1	[Associatio	Miloserdov	11234416	Not English or Spanish
1	[Associatio	Zhang NN,	23225056	Not English or Spanish
1	[Associatio	Sun XJ, Liu	17407071	Not English or Spanish
1	[Associatio	Lu LH, Cher	18393230	Not English or Spanish
1	[Associatio	Sun JY, Wa	24059111	Not English or Spanish
1	[Associatio	Lin S, Qu H,	12421496	Not English or Spanish
1	[Associatio	Kamysheva	15532370	Not English or Spanish
1	[Associatio	BuraczyÅ„s	12476891	Not English or Spanish
1	[Associative	Chistiakov I	12469627	Not English or Spanish
1	[Children w	Qin M, Gor	25619354	Not English or Spanish
1	[Correlatio	Sun ZJ, Wa	12905707	Not English or Spanish
1	[Correlatio	Ren YY, Du	16480581	Not English or Spanish
1	[Correlative	Wu XX, Zhe	15698501	Not English or Spanish
1	[Distributic	Dong JJ, Zh	18476541	Not English or Spanish

For peer review only

1	[DNA repai	Akhmadish	25715450	Not English or Spanish
1	[Effect of ti	Lin YF, Ma	19094710	Not English or Spanish
1	[Expressior	Wang X, W	27430690	Not English or Spanish
1	[Factors re:	Fu SJ, Wan	19114346	Not English or Spanish
1	[Gene mut:	Song Y, Ma	24229582	Not English or Spanish
1	[Gene poly	Shao M, Ye	23981990	Not English or Spanish
1	[Genetic pr	Bzoma B, D	19112833	Not English or Spanish
1	[GENETIC R	Apolihin OI	26665756	Not English or Spanish
1	[I/D relatio	Kaliev RR, E	16078593	Not English or Spanish
1	[Inactivatio	MikhaÄ-ler	18389622	Not English or Spanish
1	[Increased	Gozzoli V, F	11075242	Not English or Spanish
1	[Is PstI poly	Grzeszczak	9499204	Not English or Spanish
1	[Lipid pero:	Eiselt J, Rac	7762187	Not English or Spanish
1	[Mutationa	Zhao F, Yu	25224051	Not English or Spanish
1	[Polymorpl	ZieliÅ„ska E	12868187	Not English or Spanish
1	[Polymorpl	Xu JS, Bai Y	27346408	Not English or Spanish
1	[Polymorpl	Kutyrina IM	10420452	Not English or Spanish
1	[Relationsh	Wu S, Xian	9596955	Not English or Spanish
1	[Relationsh	Moleda P, I	17941464	Not English or Spanish
1	[Relationsh	Liao L, Lei N	12016801	Not English or Spanish
1	[Renal effe	Alinovi R, B	12197269	Not English or Spanish
1	[Renin-ang	BuraczyÅ„s	11865575	Not English or Spanish
1	[Screening	Yu H, Yao L	16027793	Not English or Spanish
1	[Search for	Zotova EV,	15125229	Not English or Spanish
1	[Study on c	Lu J, Li LM,	12848919	Not English or Spanish
1	[The associ	Li C, Dong Y	11930675	Not English or Spanish
1	[The functi	Rakhov DA,	1475876	Not English or Spanish
1	[Uric acid s	Simmonds	118367	Not English or Spanish
1	A common	Zhang Z, Qu	18820179	Paediatric individuals
1	A molecula	Al-Hamed I	23595123	Paediatric individuals
1	A polymorp	Imai Y, Oda	7586149	Paediatric individuals
1	ACE and AT	KostiÄ‡ M,	15179569	Paediatric individuals
1	ACE gene ir	Sekerli E, K	19603195	Paediatric individuals
1	ACE gene ir	Serdaroglu	16208534	Paediatric individuals
1	ACE gene p	Tanaka R, I	9590186	Paediatric individuals
1	ACE gene p	Sasongko T	16421456	Paediatric individuals
1	ACE gene p	Fahmy ME,	18792483	Paediatric individuals
1	ACE gene p	Celik US, N	16825089	Paediatric individuals
1	ADAMTS13	Choi HS, Ch	21488199	Paediatric individuals
1	Age and CY	Gijsen V, M	21930396	Paediatric individuals
1	Angiotensi	Al-Eisa A, H	11487079	Paediatric individuals
1	Angiotensi	Bajpai M, P	14713838	Paediatric individuals
1	Angiotensi	Patil SJ, Gu	16272677	Paediatric individuals
1	Angiotensi	Pavlovic M,	9354852	Paediatric individuals
1	Angiotensi	Rigoli L, Ch	15470205	Paediatric individuals
1	Angiotensi	al-Eisa A, H	10986863	Paediatric individuals
1	Angiotensi	Zhou J, Tiar	15315169	Paediatric individuals
1	Angiotensi	Tsai IJ, Yan	16645262	Paediatric individuals
1	Angiotensi	BatiniÄ‡ D,	25997642	Paediatric individuals
1	Associatior	Dhandapar	26154535	Paediatric individuals

For peer review only

1	Associatior	Dursun H, I	23570878	Paediatric individuals
1	Associatior	Shahid S, A	22033511	Paediatric individuals
1	Associatior	Xu ED, Xiac	27323137	Paediatric individuals
1	Associatior	Åšladowska	26227630	Paediatric individuals
1	Bardet-Bie	Benzinou M	17003356	Paediatric individuals
1	BRAF muta	Chami R, Yi	26014474	Paediatric individuals
1	Clinical util	SantÃ-n S, I	21415313	Paediatric individuals
1	Coagulatio	Fadel FI, Els	26829282	Paediatric individuals
1	Componen	Åšwierzko A	26850322	Paediatric individuals
1	Conversion	Lapeyraque	24435759	Paediatric individuals
1	Differentia	Vockley J, M	6437214	Paediatric individuals
1	Disruption	Ozaltin F, Ib	21722858	Paediatric individuals
1	Distributio	Kurachi Y, C	11547947	Paediatric individuals
1	Effect of ar	Saber-Ayac	20418353	Paediatric individuals
1	EIF2AK3 mu	Jahnavi S, F	24168455	Paediatric individuals
1	Endothelial	Dursun H, I	16941147	Paediatric individuals
1	Evaluation	Graham C,	16397046	Paediatric individuals
1	G protein b	Hocher B, F	12000471	Paediatric individuals
1	Gene polyr	Kaplan Ä°, S	25262176	Paediatric individuals
1	Genetic po	Frishberg Y	11096050	Paediatric individuals
1	Genetic po	Chiou YH, V	22610055	Paediatric individuals
1	Genetic po	Frishberg Y	9853248	Paediatric individuals
1	HbA1c leve	DeÃ¡k B, De	12682823	Paediatric individuals
1	HLA extenc	Lagueruela	1974661	Paediatric individuals
1	HLA-DQA1	Gbadegesir	25349203	Paediatric individuals
1	Impact of c	Hussein A,	25939993	Paediatric individuals
1	Inflammas	Pontillo A,	26182076	Paediatric individuals
1	Inherited m	Lynch ED, C	9399897	Paediatric individuals
1	Is paraoxor	Bilge I, Sirir	16175651	Paediatric individuals
1	Late effects	BÃ¡rdi E, O	15390293	Paediatric individuals
1	Linkage an	Hahn WH, S	20372823	Paediatric individuals
1	Modificatic	Rangel M, c	25170764	Paediatric individuals
1	Molecular a	Van Norstra	17967976	Paediatric individuals
1	Mutations	Tory K, Rou	19177160	Paediatric individuals
1	Nitric oxide	Alasehirli B	19371282	Paediatric individuals
1	Nitric oxide	Balat A, Ala	17365914	Paediatric individuals
1	Novel carb	Sapio MR, V	25875328	Paediatric individuals
1	Paraoxnase	Al-Eisa AA,	26780374	Paediatric individuals
1	Paraoxona	Biyikli NK, A	16565923	Paediatric individuals
1	Phosphodi	Hahn WH, S	20563733	Paediatric individuals
1	Platelet-act	Xu H, Iijima	10873870	Paediatric individuals
1	Platelet-act	Xu H, Iijima	9853251	Paediatric individuals
1	Polymorph	Hahn WH, S	21047277	Paediatric individuals
1	Polymorph	Maruyama	11354780	Paediatric individuals
1	Polymorph	Ece A, Teke	16109085	Paediatric individuals
1	Polymorph	Savvidou A	20022049	Paediatric individuals
1	PON1 Q192	Vasconcelo	24972570	Paediatric individuals
1	Prostanoid	NÃ¼sing RI	9262230	Paediatric individuals
1	Renin-angi	Papp F, Frie	12579405	Paediatric individuals
1	Report of a	Uhrhamme	18629513	Paediatric individuals

For peer review only

1	Endothelial	Tantawy A/	25263931	Paediatric individuals
1	Major histo	McEnery P	2702084	Paediatric individuals
1	ACE I/D ger	Hohenfelln	10452281	Paediatric individuals
1	Impact of A	Hohenfelln	11354781	Paediatric individuals
1	Role of plat	Tanaka R, I	10430976	Paediatric individuals
1	ACE gene p	Haszon I, Fr	12478352	Paediatric individuals
1	Renin-angi	Pardo R, M	12579398	Paediatric individuals
1	ACE I/D ger	Oktem F, Si	14986085	Paediatric individuals
1	Is ACE gene	ErdoÄŸan I	15138870	Paediatric individuals
1	Renin-angi	Liu KP, Lin (	15045574	Paediatric individuals
1	Roles of pa	BarÃ¡th A,	16912512	Paediatric individuals
1	Implication	Hahn H, Ku	16133060	Paediatric individuals
1	Changes in	Kaukinen A	20020158	Paediatric individuals
1	Renal mani	Han KH, Le	21210153	Paediatric individuals
1	C1GALT1 p	He X, Zhao	22544166	Paediatric individuals
1	Cost-effect	Krall P, Pine	24162162	Paediatric individuals
1	MMP-1 anc	Djuric T, Ziv	24414606	Paediatric individuals
1	Muscle inv	Park E, Cho	24912603	Paediatric individuals
1	Coinheritar	Lennon R, S	25739341	Paediatric individuals
1	Deficient fu	Kvittingen I	6622096	Paediatric individuals
1	Significance	Hori C, Hira	11474225	Paediatric individuals
1	Induction c	Ma J, Mats	21451433	Paediatric individuals
1	Sudden inf	RomÃ¡n R,	18075835	Paediatric individuals
1	The polymo	SimoÅ„ska-	18615395	Paediatric individuals
1	Thiopurine	Dervieux T,	10594482	Paediatric individuals
1	Activation	Karlsson J,	25481751	Paediatric individuals
1	Activation	Fischer DC,	19176689	Paediatric individuals
1	Associatior	Laksmi NK,	20149750	Paediatric individuals
1	Clinicopath	Huo Z, Lu T	27760550	Paediatric individuals
1	DD genotyp	Alasehirli B	22017506	Paediatric individuals
1	Effect of pa	Yilmaz A, E	19967651	Paediatric individuals
1	Endothelial	Elshamaa M	21519233	Paediatric individuals
1	Endothelial	Rusai K, Va	18334945	Paediatric individuals
1	ETV6-NTRK	Knezevich S	9823307	Paediatric individuals
1	Expression	Tsuruga K,	26058859	Paediatric individuals
1	Founder m	Elia A, Vosk	20805693	Paediatric individuals
1	Genes in th	van Eerde A	22558067	Paediatric individuals
1	Genetic an	Khan AO, A	24997176	Paediatric individuals
1	Genetic he	Shimozawa	12794690	Paediatric individuals
1	Genetic po	Sata F, Kura	20059664	Paediatric individuals
1	Genomic re	Vervoort V	12404110	Paediatric individuals
1	Glutathione	Elhasid R, K	20348973	Paediatric individuals
1	Glycosphin	Dawson G.	5016302	Paediatric individuals
1	Heterogen	Wildin RS,	7913579	Paediatric individuals
1	HLA class II	Djilali-Saiah	17050030	Paediatric individuals
1	HPSE2 mut	Bulum B, Ã	25924634	Paediatric individuals
1	Hypophosp	Rutsch F, B	20016754	Paediatric individuals
1	Implication	Ozen S, Alik	10401028	Paediatric individuals
1	Lethal cysti	Simpson M	19303681	Paediatric individuals
1	Loss of nep	Bergmann	18371931	Paediatric individuals

For peer review only

1	Low renin-a	Peruzzi L, L	16006956	Paediatric individuals
1	Molecular (	Elhayek D,	24252324	Paediatric individuals
1	Mutation s	Winberg J,	24416387	Paediatric individuals
1	Mutational	Boyer O, Be	20591883	Paediatric individuals
1	Mutations	Frank V, Ha	23418306	Paediatric individuals
1	Novel ATP6	Stover EH,	12414817	Paediatric individuals
1	Polymorph	Amoroso A	9870486	Paediatric individuals
1	Renin-angi	Ozkaya O, S	16521052	Paediatric individuals
1	RET and GD	Jeanpierre	21490379	Paediatric individuals
1	Role of tru	Debiec H, N	15464186	Paediatric individuals
1	Streptokina	Tewodros V	7783598	Paediatric individuals
1	The associa	Rotman Y,	20684021	Paediatric individuals
1	Variability i	Ferroni MA	8880047	Paediatric individuals
1	CYP2C9 ger	Joy MS, Do	19669737	Pharmaceutical drug focus
1	Enalapril ar	Rashtchizad	17222813	Pharmaceutical drug focus
1	Exaggerate	Simonsen J	21749320	Pharmaceutical drug focus
1	Long-term	Andersen S	12716812	Pharmaceutical drug focus
1	Renin-angi	Noroozian	17524880	Pharmaceutical drug focus
1	Venous res	Mitchell A,	14586390	Pharmaceutical drug focus
1	Associatior	Xin HW, Liu	24691060	Pharmaceutical drug focus
1	Associatior	Liu X, Li J, F	25587129	Pharmaceutical drug focus
1	Comparing	Cai J, Huang	22149319	Pharmaceutical drug focus
1	Compariso	Niioka T, Sa	23073468	Pharmaceutical drug focus
1	CYP2C9*2 a	Ragia G, Ta	24464600	Pharmaceutical drug focus
1	CYP3A5 ger	Picard N, R	21389905	Pharmaceutical drug focus
1	CYP3A5 ger	Hesselink D	18334918	Pharmaceutical drug focus
1	CYP3A5 po	Song J, Kim	22947591	Pharmaceutical drug focus
1	Cytochrom	Winoto J, S	21543025	Pharmaceutical drug focus
1	Drug-resist	Hantz S, Ga	20961907	Pharmaceutical drug focus
1	Effect of ar	Penno G, C	9726242	Pharmaceutical drug focus
1	Effect of de	Parving HH	8806248	Pharmaceutical drug focus
1	Effects of e	Glicklich D,	10428268	Pharmaceutical drug focus
1	Endothelial	Cheema BS	23260854	Pharmaceutical drug focus
1	Endothelial	Cherney DZ	19037250	Pharmaceutical drug focus
1	Genetic ana	Singer JB, H	17563401	Pharmaceutical drug focus
1	Genetic an	Jacobson P	22334041	Pharmaceutical drug focus
1	Genetic ass	Watson B J	8794836	Pharmaceutical drug focus
1	Genetic po	Vu D, Tellez	23721685	Pharmaceutical drug focus
1	Genetic var	Tapirdamaz	25014506	Pharmaceutical drug focus
1	Human thio	Weyer N, K	11417444	Pharmaceutical drug focus
1	Impact of t	Ro H, Min S	23149441	Pharmaceutical drug focus
1	Impact of t	Lely AT, van	18077786	Pharmaceutical drug focus
1	In vivo CYP	de Jonge H	21753749	Pharmaceutical drug focus
1	Individualiz	Chen SY, Li	23432535	Pharmaceutical drug focus
1	Influence o	Kagaya H, N	22106207	Pharmaceutical drug focus
1	Lack of asso	Hoffmann I	21871624	Pharmaceutical drug focus
1	Matrix met	Kurzawski I	16945025	Pharmaceutical drug focus
1	Matrix met	Drozdzik A,	19778329	Pharmaceutical drug focus
1	Modulating	Abdin AA, H	19553142	Pharmaceutical drug focus
1	Nifedipine	Verhaar MC	10533616	Pharmaceutical drug focus

For peer review only

1	Pharmacog	Okamura A	10535720	Pharmaceutical drug focus
1	Pharmacog	Lennard L,	2758725	Pharmaceutical drug focus
1	Presence o	Ragia G, Pe	19891554	Pharmaceutical drug focus
1	Randomize	Kvetny J, G	11181984	Pharmaceutical drug focus
1	Risk of tacr	Hooper DK,	22491658	Pharmaceutical drug focus
1	The influen	Luik PT, Ho	12856080	Pharmaceutical drug focus
1	The norbor	Kessler M,	10854736	Pharmaceutical drug focus
3	Investigatio	Currie D, M	20854388	< 3 populations reported per SNP
3	Polymorph	Frojdo, S; S	16211375	< 3 populations reported per SNP
3	Angiotensi	Yang, HY; L	23477970	< 3 populations reported per SNP

For peer review only

Supplementary Table S3c

Exclusion S	Title	Authors	PMID	Reason
1	Identificati	Chen, Z; W	27756246	Gene expression based study
1	A disease h	Ng, DPK; Pl	16936219	No data for AGT
1	A polymorp	Mollsten, A	21316998	No data for AGT
1	A rare hapl	Martin, RJL	19783860	No data for AGT
1	ACACÎ² gen	Shah VN, C	23081748	No data for AGT
1	ACE gene p	Felehgari, V	20830509	No data for AGT
1	alpha(1)-ar	Perdu, J; Gi	16531799	No data for AGT
1	Analysis of	Drouet, M;	12005241	No data for AGT
1	Angiotensii	Yildiz A, Ya	12832741	No data for AGT
1	Angiotensii	Haque, SF;	NA	No data for AGT
1	Angiotensii	Schmidt, S;	9269698	No data for AGT
1	Angiotensii	Grzeszczak	9727375	No data for AGT
1	Angiotensii	Stratta, P; (	10352195	No data for AGT
1	Angiotensii	El-Banawy,	NA	No data for AGT
1	Angiotensii	Buraczynsk	12187084	No data for AGT
1	Angiotensii	Yoneda, A;	12187255	No data for AGT
1	Angiotensii	Uhm, WS; I	12043886	No data for AGT
1	Angiotensii	Woo KT, La	18536822	No data for AGT
1	Angiotensii	Schmidt, A;	8785402	No data for AGT
1	Angiotensii	Ergen, HA;	NA	No data for AGT
1	Angiotensii	Tarnow L, (	8671962	No data for AGT
1	Associatior	Golmoham	NA	No data for AGT
1	Associatior	Gao, JX; Zh	26045844	No data for AGT
1	Associatior	Conen D, G	18698212	No data for AGT
1	Associatior	Bagci, B; Ba	27118566	No data for AGT
1	Carnosine a	Janssen, B;	16046297	No data for AGT
1	Clinical imp	Amorim, CI	23362199	No data for AGT
1	Defining Ur	Abushufa, A	25546407	No data for AGT
1	Different M	Nakayama,	19293592	No data for AGT
1	DNA polym	Freire, MBS	9794558	No data for AGT
1	Effect of AC	BÃ¶ger CA,	16961167	No data for AGT
1	Effects of e	Glicklich, D	10428268	No data for AGT
1	Endothelial	Cheema, BS	23260854	No data for AGT
1	GENETIC PI	DORIA, A; V	7909524	No data for AGT
1	Haplotype	Doi, K; Noir	16215641	No data for AGT
1	Hereditary	Marre, M; I	10922971	No data for AGT
1	High preval	Tripathi G,	17042963	No data for AGT
1	Impact of F	Jung, ES; Ki	21150220	No data for AGT
1	Increased e	Ulrich, C; H	16476718	No data for AGT
1	Insertion/d	Demurov, L	NA	No data for AGT
1	Involvemer	Yamada, K;	11349729	No data for AGT
1	Kinin-depe	Molinaro G	17003818	No data for AGT
1	Lack Of Rel	Tarnow, L;	7729604	No data for AGT
1	Neuropept	Ito, H; Mor	10363627	No data for AGT
1	NO ASSOCI	SCHMIDT, S	7485124	No data for AGT
1	Plasma ren	Deinum, J;	10462269	No data for AGT
1	Polymorph	Liu, ZH; Li, I	NA	No data for AGT
1	Polymorph	Chistyakov,	NA	No data for AGT

For peer review only

1	Polymorph	Hanyu, O; H	9509566	No data for AGT
1	Polymorph	Yorioka, T;	8529313	No data for AGT
1	Polymorph	Frojdo, S; S	16211375	No data for AGT
1	Proteomic	Kwak, NJ; W	21136694	No data for AGT
1	Relations b	Shin, YS; Ba	15331206	No data for AGT
1	Relationshi	Movva, S; A	17616353	No data for AGT
1	Relationshi	Oh TG, Shin	8854649	No data for AGT
1	Relationshi	Marre, M; I	8314010	No data for AGT
1	Renal perfu	Lansang, M	12086929	No data for AGT
1	Role of tran	Inigo, P; La	14558330	No data for AGT
1	Serological	Coppo, R; A	17988266	No data for AGT
1	Susceptibili	Hanna, MC	25316403	No data for AGT
1	Synergistic	Doria, A; O	9389421	No data for AGT
1	Synergistic	Wakahara,	17303661	No data for AGT
1	The angiote	Hibberd, M	9025006	No data for AGT
1	The angiote	Lu, M; Zhan	27633502	No data for AGT
1	The effect o	Nakayama,	16312263	No data for AGT
1	The freque	Rahimi, Z; F	20853144	No data for AGT
1	The influen	Maixnerova	17328840	No data for AGT
1	The influen	Maixnerova	18498720	No data for AGT
1	The ras res	Bonomo, JA	25027322	No data for AGT
1	Transformi	August, P; S	NA	No data for AGT
1	20-Hydroxy	Savas, S; W	NA	Non-human
1	Acute eleva	Frisbee, JC;	10329253	Non-human
1	Adenosine	Gao, X; Pele	25251152	Non-human
1	Adrenomed	Caron, K; H	17360661	Non-human
1	Angiotensin	Shahid, SM	24035938	Non-human
1	Angiotensin	Jonsson, JR	11438504	Non-human
1	Angiotensin	Noshiro, T;	NA	Non-human
1	Appropriat	Catanzaro I	9931123	Non-human
1	Association	Soria, LA; C	19665052	Non-human
1	Blood press	Venegas-Po	21900645	Non-human
1	Blood press	Bohlender,	22728903	Non-human
1	Cardiac ang	Ferrario CM	26873967	Non-human
1	CCN1 expre	Bonda, TA;	23690222	Non-human
1	Chronic hyp	Merrill, DC;	8613528	Non-human
1	Compensat	Wang, Q; H	11380824	Non-human
1	Co-operatio	Stepinski, J	11016869	Non-human
1	Cosegregat	Yu, H; Harr	9794718	Non-human
1	Effect of so	Maekawa,	8720419	Non-human
1	Effect of th	Handtrack,	17333097	Non-human
1	Effects of p	Omogbai, E	15834453	Non-human
1	Effects of S	Ott, IM; Alt	22900035	Non-human
1	Endotheliu	Pruneau, D	8453977	Non-human
1	Hemodyna	Abreu, Np;	18293204	Non-human
1	Hyperplast	Pauletto, P	8156626	Non-human
1	Hypertensi	Marley, WS	10567853	Non-human
1	Hypervolem	Morgan, TK	16796982	Non-human
1	Increased a	Turkstra, E;	10523346	Non-human
1	Inhibition o	Wang, XM;	26883268	Non-human

For peer review only

1	Inhibition c	Eitle, E; Hir	9575805	Non-human
1	Long-Term	Ameer, OZ;	27880955	Non-human
1	Losartan ar	Srisawat, U	25947921	Non-human
1	Low Blood	Kisanuki, Y	20516397	Non-human
1	Mechanica	Prager-Kho	25712904	Non-human
1	Mediation	Sun, DQ; Sa	11504952	Non-human
1	Metformin	Gomes, ME	16133490	Non-human
1	Mice lackin	Cole, JM; K	12574101	Non-human
1	N-domain a	Bueno, V; F	15194348	Non-human
1	Nebivolol t	Wang, Y; Zh	23263161	Non-human
1	Nitric oxide	Parekh, N;	8780230	Non-human
1	Overexpres	Liu, XL; Zha	19279555	Non-human
1	Rat Ace alle	Kamilic, J; H	21788250	Non-human
1	Regulation	Kim, SM; Cl	17515456	Non-human
1	Renal cyst g	Fonseca, JM	24429399	Non-human
1	Role of A(1	Jackson, EK	22874760	Non-human
1	Role of ang	Yang, XH; W	22580272	Non-human
1	Role of enc	Moreno, JM	12657567	Non-human
1	Role of neu	Wegner, M	8759244	Non-human
1	Sodium-Nit	Lovell, Hb;	7706899	Non-human
1	The angiote	Laesser, M;	14995942	Non-human
1	The angiote	Hunley, TE;	10652034	Non-human
1	The geneti	Heringer-W	22652430	Non-human
1	Transfer of	Jiang, J; Ste	9040448	Non-human
1	Advanced (	Bondeva, T	24030251	Non-human
1	AKAP150 is	Navedo, M	18174462	Non-human
1	Altered reg	O'Tierney,	18192845	Non-human
1	An essentia	Hisada, Y; S	11555672	Non-human
1	Angiotensi	Broome, M	11065204	Non-human
1	Angiotensi	Pupilli, C; L	10215323	Non-human
1	Angiotensi	Sparks, MA	21242463	Non-human
1	Angiotensi	Touyz, RM;	15753233	Non-human
1	Angiotensi	Maeda A, T	23902639	Non-human
1	Angiotensi	Bohlender .	10720595	Non-human
1	Axl mediate	Korshunov,	17923589	Non-human
1	Sodium-Nit	Lovell, Hb;	7562480	Non-human
1	Calcium/Ca	Prasad, AM	26077587	Non-human
1	Cardiac phe	van Esch, JH	20071356	Non-human
1	Chronic reg	Melo, LG; V	9815091	Non-human
1	Complex in	Fellner, SK;	19880669	Non-human
1	Connexin 4	Machura, K	25241776	Non-human
1	Connexin 4	Gerl, M; Ku	24062052	Non-human
1	Contributic	Frisbee, JC;	10775129	Non-human
1	Correction	Lumb, MJ;	12737622	Non-human
1	Cross-speci	Sun, RX; Ch	25467400	Non-human
1	Cytoprotec	Hegarty, N.	12081565	Non-human
1	Developme	Lusch, A; Ka	23668542	Non-human
1	DPP-4 Inhib	Alter, ML; (	23171828	Non-human
1	Effect of th	Knier, B; Co	21610512	Non-human
1	Functional	Tonna, S; D	18206321	Non-human

For peer review only

1	Gene Trapp	Park, S; Liu,	22547438	Non-human
1	Genetic-Co	Kim, Hs; Kr	7708716	Non-human
1	Human GRI	Wang, Z; Ze	26667412	Non-human
1	Identificati	Bauer, Y; H	21357272	Non-human
1	Knockdowr	Raison, D; (	23720345	Non-human
1	Lack of an	Kittikulsuth	24431204	Non-human
1	Lack of in v	Bonvini, JM	10966489	Non-human
1	Loss of Not	Ragot, H; M	27296994	Non-human
1	Lysine-Spec	Williams, JS	22534796	Non-human
1	Macula Del	Lu, Y; Wei,	26647426	Non-human
1	Mechanism	Todkar, A;	25071088	Non-human
1	mPGES-1 d	Jia, ZJ; Liu,	22237797	Non-human
1	Natriuretic	Demerath,	24717731	Non-human
1	Nephron-sp	Ramkumar,	25995108	Non-human
1	Nitric oxide	Liang, MY;	10432402	Non-human
1	NOS1-depe	Hyndman,	25391901	Non-human
1	N-terminal	Bodenstein	17220356	Non-human
1	Organizatic	Cabral, CM	12181335	Non-human
1	Pathophysi	Igarashi, K;	25252154	Non-human
1	Physiologic	Le, TH; Kim	12963678	Non-human
1	Possible ro	Ramkumar,	NA	Non-human
1	Pressor and	Hirooka, Y;	8934360	Non-human
1	Reciprocal	Kurt, B; Kur	21209011	Non-human
1	Regulation	Tipsmark, C	12654889	Non-human
1	Regulation	Liu, JL; Zucl	10066676	Non-human
1	Renal angic	Stoneking,	9719278	Non-human
1	Renal redo	Yogi, A; Me	18195161	Non-human
1	Role of NO	Carlstrom,	18987286	Non-human
1	Signal trans	Yousif, MH	16287213	Non-human
1	Specific pre	Bird, IM; M	9687288	Non-human
1	Stimulatior	Kurtz, A; Go	9575895	Non-human
1	Strain diffe	Smit-van O	11984748	Non-human
1	Thromboxa	Sparks, MA	23150508	Non-human
1	Vascular ar	Muller, DN	9039087	Non-human
1	A systemat	Feng, X; Zh	23178513	Not a case-control study
1	ACE gene p	Schena, FP;	11473656	Not a case-control study
1	Allergic rea	Alencar, RC	20180980	Not a case-control study
1	Angiotensir	Ng, D; Tai,	15830182	Not a case-control study
1	Antihypert	Kuznetsova	10918550	Not a case-control study
1	Associatior	Huang, ZK;	26000752	Not a case-control study
1	Associatior	Ding, W; W	21607620	Not a case-control study
1	Associatior	Ji, LD; Zhan	20087216	Not a case-control study
1	Gastric-Car	Straccapan	7726143	Not a case-control study
1	Weber-Chr	Sterling, KA	23216240	Not a case-control study
1	Whole-Ger	Cordell, HJ;	19959718	Not a case-control study
1	A Synergist	Rodriguez-I	19546528	Not a case-control study
1	Associatior	Zhou, TB; Y	23971628	Not a case-control study
1	CYP3A5 an	Bochud, M;	19290795	Not a case-control study
1	Diabetic ne	Rogus, JJ; N	9461232	Not a case-control study
1	Disorders c	Connell, JM	11469810	Not a case-control study

For peer review only

1	Genetic de	Schmidt, S;	NA	Not a case-control study
1	Genetics ar	Marre, M	10097900	Not a case-control study
1	Genetics of	Costerouss	9247746	Not a case-control study
1	HLA genes	Griffith, ME	9493788	Not a case-control study
1	Improveme	Matsunaga	19603250	Not a case-control study
1	Is the prese	Ng, DPK; Ta	18523141	Not a case-control study
1	M235T ang	Staessen, J.	10100088	Not a case-control study
1	microRNAs	Marques, F	26663185	Not a case-control study
1	Mistyping c	Ueda, S; He	8863184	Not a case-control study
1	Nonmodula	Hollenberg	16672145	Not a case-control study
1	Normative	Prasad, P; T	18027817	Not a case-control study
1	Partial Dele	Nogueira, F	10737993	Not a case-control study
1	Risk of dev	Savage, DA	10328465	Not a case-control study
1	The deletio	Staessen, J.	9488209	Not a case-control study
1	The Phenot	Korner, PI	21208016	Not a case-control study
1	The renopr	Locatelli, F;	10048500	Not a case-control study
1	Varicella-zc	Velez, AMA	NA	Not a case-control study
1	Add-on ang	Agarwal, R	11380832	Not a case-control study
1	Analysis of	Torres VE, (	22205355	Not a case-control study
1	Angiotensii	Akcay, A; O	15013293	Not a case-control study
1	Angiotensii	Narita, I; G	12911556	Not a case-control study
1	Angiotensii	Hopkins, PI	11997278	Not a case-control study
1	Antiproteir	Haneda, M	15364166	Not a case-control study
1	Associatior	Pei, Y; Scho	9259580	Not a case-control study
1	Associatior	Akcay, A; S	15385810	Not a case-control study
1	Contributic	Wong, TYH	11431175	Not a case-control study
1	Gene Polyr	Zhang, G; V	17984617	Not a case-control study
1	Impact of g	Siekierka-H	19681973	Not a case-control study
1	Polymorph	Frimat, L; P	11053482	Not a case-control study
1	Survival in	Padro-Miqu	19014923	Not a case-control study
1	The role of	Kelly TN, Ra	25906781	Not a case-control study
1	Major clini	Alderman,	NA	Not a case-control study
1	NHLBI Fam	Williams, R	10964005	Not a case-control study
1	Germline g	Audenet F,	23911636	Not a renal disease focus
1	Impact of r	Pfab T, Stir	17563539	Not a renal disease focus
1	Insertion/D	de Martino	21477733	Not a renal disease focus
1	Polymorph	Deckers, IA	24978482	Not a renal disease focus
1	The angiot	Ebert, MPA	16365022	Not a renal disease focus
1	Variants in	Andreotti, (	20047954	Not a renal disease focus
1	alpha(1)-Ar	Greene, DN	22215832	Not a renal disease focus
1	VASODILAT	DUBEY, RK	8169847	Not a renal disease focus
1	2 NOVEL P(	MORIYAMA	8656071	Not a renal disease focus
1	A Computa	Guillaud, F;	20683640	Not a renal disease focus
1	A new theo	Eggers, AE	26243176	Not a renal disease focus
1	A Polymorp	Sugimoto, I	18936345	Not a renal disease focus
1	A study on	Chmaisse H	19861867	Not a renal disease focus
1	ACE gene ii	Dell'omo, G	16889537	Not a renal disease focus
1	Alanine : gl	Danpure, C	12686111	Not a renal disease focus
1	alpha(1)-ar	Callea, F; G	9279535	Not a renal disease focus
1	Alpha1-ant	Taddei C, A	10563543	Not a renal disease focus

For peer review only

1	Alpha-1-pro	O'Donoghu	8296629	Not a renal disease focus
1	alpha-addu	Clark, CJ; D	11116113	Not a renal disease focus
1	Alternative	Matamala,	26141700	Not a renal disease focus
1	An Erythro	Schulz, EG;	21912181	Not a renal disease focus
1	Analyses of	Berge, KE; I	9298743	Not a renal disease focus
1	ANCA-asso	Morris, H; I	21821620	Not a renal disease focus
1	Angiotensi	Tassiulas, I	9710341	Not a renal disease focus
1	Angiotensi	Costerouss	9830503	Not a renal disease focus
1	Angiotensi	Vasku, A; S	9607178	Not a renal disease focus
1	Angiotensi	Sanderford	11009467	Not a renal disease focus
1	Angiotensi	Spaanderm	15350256	Not a renal disease focus
1	Angiotensi	Miller, JA; T	10969844	Not a renal disease focus
1	ANGIOTEN:	NAGI, DK; M	8582133	Not a renal disease focus
1	Angiotensi	Pedersen-E	19820429	Not a renal disease focus
1	Angiotensi	Stefansson,	10855732	Not a renal disease focus
1	Angiotensi	Badr, S; Ral	NA	Not a renal disease focus
1	Associatior	Boomsma I	15830186	Not a renal disease focus
1	Associatior	Nalbantogl	23151617	Not a renal disease focus
1	Associatior	Song SB, Jir	21342026	Not a renal disease focus
1	Associatior	Assali, A; G	21570644	Not a renal disease focus
1	Associatior	Rabbani M.	18711292	Not a renal disease focus
1	Associatior	RodrÃ-gue:	11345362	Not a renal disease focus
1	Associatior	Singh, PP; I	16621107	Not a renal disease focus
1	Associatior	Mokretar, I	26670794	Not a renal disease focus
1	Associatior	Meng, N; Z	25359286	Not a renal disease focus
1	Associatior	Mayer, NJ;	NA	Not a renal disease focus
1	Associatior	Mashimo, Y	18398332	Not a renal disease focus
1	Blunted cG	Supaporn, T	8914042	Not a renal disease focus
1	Blunted rer	Hopkins, PI	8728297	Not a renal disease focus
1	C-antineutr	Griffith ME	8671812	Not a renal disease focus
1	Clinical anc	Huang CC, (	26313793	Not a renal disease focus
1	Comprehen	Kato, N; Su	10953993	Not a renal disease focus
1	Comprehen	Monico CG	17460142	Not a renal disease focus
1	Contributic	Mondorf U	9524045	Not a renal disease focus
1	Crystal stru	Zhang, XX;	12899834	Not a renal disease focus
1	CYP3A5 ger	Fromm MF	16141800	Not a renal disease focus
1	Deep-targe	Pescatello,	NA	Not a renal disease focus
1	Detection c	Matsumoto	11106834	Not a renal disease focus
1	Effect of A(	Poduri, A; K	18200034	Not a renal disease focus
1	Effects of a	Kulah, E; Dı	17625392	Not a renal disease focus
1	Effects of o	Maeda, K; I	16678545	Not a renal disease focus
1	Efficacy of	Kwon, DH;	23717118	Not a renal disease focus
1	Elevated ur	Clausen, P;	10872702	Not a renal disease focus
1	Endothelial	Page, A; Re	16093452	Not a renal disease focus
1	ENHANCED	NIELSEN, C	8393795	Not a renal disease focus
1	Eprosartan	Vase, H; La	18341678	Not a renal disease focus
1	Evaluation	Rumsby G,	15327387	Not a renal disease focus
1	FREQUENC	ZEE, RYL; B	7882587	Not a renal disease focus
1	Functional	Coto E, Pala	20594303	Not a renal disease focus
1	Functional	Lumb MJ, D	10960483	Not a renal disease focus

For peer review only

1	Functional	Gainer, JV;	15611369	Not a renal disease focus
1	Further stu	Danpure CJ	3416563	Not a renal disease focus
1	Genetic po	Deuster, PA	23543093	Not a renal disease focus
1	Genetic po	Wu, CK; Tsa	19330904	Not a renal disease focus
1	Genetic po	Olivieri, O;	10567188	Not a renal disease focus
1	Genetic pre	Tousoulis D	23479071	Not a renal disease focus
1	Genetic risl	van Onna, I	15326089	Not a renal disease focus
1	Genetic var	Ichikawa, N	24549414	Not a renal disease focus
1	Genetic var	Hsu, CCC; E	16396964	Not a renal disease focus
1	Genomic as	Turner ST, I	22566498	Not a renal disease focus
1	Genotype-p	Watkins, W	19770777	Not a renal disease focus
1	Genotypic i	Mehri, S; K	20580725	Not a renal disease focus
1	G-protein b	Zagradisnik	15337465	Not a renal disease focus
1	Identificati	Purdue PE,	1703535	Not a renal disease focus
1	Increased e	Yamani, MI	17097490	Not a renal disease focus
1	Independe	Morris, Bj;	8104754	Not a renal disease focus
1	Inhibition c	Montgome	9797175	Not a renal disease focus
1	Interaction	Bruning TA	7866595	Not a renal disease focus
1	Is the influe	Muthumala	17624939	Not a renal disease focus
1	Lack of ass	Kato, N; Su	9495254	Not a renal disease focus
1	Low-salt di	Manunta, P	14684671	Not a renal disease focus
1	Lys(173)Arg	Komiya I, Y	10720581	Not a renal disease focus
1	Middle cer	Thomas, GI	12865608	Not a renal disease focus
1	Molecular g	Corvol, P; S	9296068	Not a renal disease focus
1	Molecular-	Krieger, Je; NA		Not a renal disease focus
1	Mutation-b	Coulter-Ma	18282470	Not a renal disease focus
1	No associat	Wolf, G; Pa	12147803	Not a renal disease focus
1	No associat	Ishikawa, K	9607391	Not a renal disease focus
1	No Evidenc	Liktor, B; C	23370555	Not a renal disease focus
1	Overexpres	Coulter-Ma	15802217	Not a renal disease focus
1	Oxidative s	CalÃ² LA, Pa	12897089	Not a renal disease focus
1	Peripheral	Thomas, GI	14632699	Not a renal disease focus
1	Pharmacolo	Miyata N, S	25237136	Not a renal disease focus
1	Phenotype	Gong L, Che	24474657	Not a renal disease focus
1	Polymorph	Baudin, B	15640279	Not a renal disease focus
1	Polymorph	Bofinger, A	11317203	Not a renal disease focus
1	Potential B	Grisk, O	23852615	Not a renal disease focus
1	Rab1 Small	Zhuang, XL	20861236	Not a renal disease focus
1	Renal chan	Marre, M; I	10082486	Not a renal disease focus
1	Renal haen	Skov, K; Ma	17083073	Not a renal disease focus
1	Renin-aldo	Hanukoglu,	18634878	Not a renal disease focus
1	Renin-angi	BuraczyÅ„s	14502296	Not a renal disease focus
1	Renin-Angi	Joyce-Tan, NA		Not a renal disease focus
1	Risk given b	Li, Q; Sun, L	26391364	Not a renal disease focus
1	Role of ACE	Cordonnier	11708761	Not a renal disease focus
1	Role of GRI	Chen, K; Fu	24218433	Not a renal disease focus
1	Serum liver	Larizza, D; I	10664223	Not a renal disease focus
1	Single nucl	Demir AK, I	27936341	Not a renal disease focus
1	Single Stran	Bettinaglio	12270765	Not a renal disease focus
1	SNP Varian	Coulter-Ma	25854853	Not a renal disease focus

For peer review only

1	Somatic Vo	Gilcrease, I	8522307	Not a renal disease focus
1	The conser	Mesa-Torre	24957194	Not a renal disease focus
1	The geneti	Stanczuk, G	17653872	Not a renal disease focus
1	The interac	Gatti, RR; S	22791701	Not a renal disease focus
1	The M235T	van den Bo	17921816	Not a renal disease focus
1	The major	Coulter-Ma	15963748	Not a renal disease focus
1	The presen	GÃ¼ngÃ¶r	21332339	Not a renal disease focus
1	The state a	Price, DA; I	10232494	Not a renal disease focus
1	Ubiquitin li	Shen Y, Bal	16979136	Not a renal disease focus
1	Vitamin B6	Hoyer-Kuhi	24385516	Not a renal disease focus
1	[A study or	Nishina M.	9014479	Not written in English or Spanish
1	[AGTR1 A1	Yin X, Li H,	23505107	Not written in English or Spanish
1	[Arterial hy	Ermolenko	7700	Not written in English or Spanish
1	[Associatio	Xue Y, Cher	11798574	Not written in English or Spanish
1	[Associatio	BuraczyÅ„s	12476891	Not written in English or Spanish
1	[Effect of e	Kaliuzhin V	24261234	Not written in English or Spanish
1	[Genetic pr	Bzoma B, D	19112833	Not written in English or Spanish
1	[Identificat	Savost'ianc	12500539	Not written in English or Spanish
1	[Is PstI poly	Grzeszczak	9499204	Not written in English or Spanish
1	[Pharmaco	Mormol' IA	25286611	Not written in English or Spanish
1	[Polymorpl	Chistiakov	10576062	Not written in English or Spanish
1	[Polymorpl	Kutyrina IM	10420452	Not written in English or Spanish
1	[Relationsh	Moleda P, I	17941464	Not written in English or Spanish
1	[Relationsh	Liao L, Lei I	12016801	Not written in English or Spanish
1	[Relationsh	Wang J, Zhu	10514536	Not written in English or Spanish
1	[Renin-ang	BuraczyÅ„s	11865575	Not written in English or Spanish
1	A-20C angi	Nakanishi,	14648325	Paediatric Individuals
1	ACE and AT	Kostic, M; S	15179569	Paediatric Individuals
1	ACE gene p	Tanaka, R;	9590186	Paediatric Individuals
1	ACE gene p	Fahmy, ME	18792483	Paediatric Individuals
1	Angiotensi	Pavlovic, M	9950302	Paediatric Individuals
1	Angiotensi	Pavlovic, M	9354852	Paediatric Individuals
1	Angiotensi	Siomou, E;	17515833	Paediatric Individuals
1	Angiotensi	Rigoli, L; Ch	15470205	Paediatric Individuals
1	Angiotensi	Gallego, PH	18413222	Paediatric Individuals
1	Donor and	Buscher, R;	21309964	Paediatric Individuals
1	Effects of g	Tabel, Y; Be	16525944	Paediatric Individuals
1	Gene Polyr	Kaplan, I; S	25262176	Paediatric Individuals
1	Genetic coı	Yim, HE; Ba	17597658	Paediatric Individuals
1	Genetic po	Elshamaa, I	21859496	Paediatric Individuals
1	Genetic po	Frishberg, \	9853248	Paediatric Individuals
1	Impact of c	Hussein, A;	25939993	Paediatric Individuals
1	Polymorph	Maruyama	11354780	Paediatric Individuals
1	Polymorph	Ece, A; Tek	16109085	Paediatric Individuals
1	Renin-angi	Papp, F; Fri	12579405	Paediatric Individuals
1	Renin-angi	Liu, DS; Lu, NA		Paediatric Individuals
1	ACE gene p	Haszon, I; F	12478352	Paediatric Individuals
1	Angiotensi	Hohenfelln	10353402	Paediatric Individuals
1	Implication	Hahn, H; Kı	16133060	Paediatric Individuals
1	Renin-angi	Pardo, R; M	12579398	Paediatric Individuals

For peer review only

1	Renin-angi	Liu, KP; Lin,	15045574	Paediatric Individuals
1	Autosomal	Geller, DS;	16611713	Paediatric Individuals
1	Genetic po	Yim, HE; Ju	14764974	Paediatric Individuals
1	Genetic risl	Taranta, A;	19110485	Paediatric Individuals
1	Hemorrhag	Levin M, Pi	2783733	Paediatric Individuals
1	Identificati	Forino, M;	14673707	Paediatric Individuals
1	Implication	Ozen S, Alik	10401028	Paediatric Individuals
1	Low renin-a	Peruzzi, L; I	16006956	Paediatric Individuals
1	Posterior u	Bajpai, M; I	15643266	Paediatric Individuals
1	Renal tubu	Lacoste M,	16790508	Paediatric Individuals
1	Renin-angi	Ozkaya, O;	16521052	Paediatric Individuals
1	The juxtagl	Taugner R,	3128915	Paediatric Individuals
1	Variants of	Halkas, AC;	9475086	Paediatric Individuals
1	ACE gene p	Parving, HH	18199798	Pharmaceutical drug focus
1	Angiotensi	Cambien, F	9519756	Pharmaceutical drug focus
1	Associatior	Kuriyama, S	11675943	Pharmaceutical drug focus
1	CYP2C9 ger	Joy MS, Do	19669737	Pharmaceutical drug focus
1	Effects of lo	Argani, H; G	18261537	Pharmaceutical drug focus
1	Efficacy an	Axelsson, A	25533774	Pharmaceutical drug focus
1	Enalapril ar	Rashtchizad	17222813	Pharmaceutical drug focus
1	Exaggerate	Simonsen, .	21749320	Pharmaceutical drug focus
1	High serum	Elung-Jense	11881130	Pharmaceutical drug focus
1	Long-term	Andersen S	12716812	Pharmaceutical drug focus
1	Losartan de	Campistol,	10432413	Pharmaceutical drug focus
1	Renin-angi	Noroozian	17524880	Pharmaceutical drug focus
1	Randomize	Amara, AB;	20061926	Pharmaceutical drug focus
1	Renal impli	Hollenberg	11459212	Pharmaceutical drug focus
1	The influen	Goyache-G	24241364	Pharmaceutical drug focus
1	The influen	Luik, PT; Ho	12856080	Pharmaceutical drug focus
2	U.K. Prospe	Dudley CR,	8587251	No data
2	The renin--	Ong-Ajyoot	10511770	No data
2	The relatio	Shestakova	WOS:0002	No data
2	Relationshi	Wang, JJ; Z	11717948	No data
2	Polymorph	Chistyakov,	WOS:0000	No data
2	Polymorph	Woo, KT; La	15504143	No data
2	Lack of ass	Wong, TYH	10352194	No data
2	Influence o	Bantis, C; Iv	15031629	No data
2	Genetic po	Buraczyńsk	12898858	No data
2	Effects of tl	Luther Y, Ba	14610337	No data
2	Combinatic	Osawa	17143591	No data
2	Renin-angi	Mtiraoui, N	21421655	Incorrect patient group
2	Angiotensi	Gutierrez	9258285	No data
3	ACE variant	Ahluwalia T	19108684	< 3 populations reported per SNP
3	Analysis of	Anbazhaga	19520069	< 3 populations reported per SNP
3	Angiotensi	Lee KB et a	12950120	< 3 populations reported per SNP
3	Angiotensi	Reis KA et a	16228848	< 3 populations reported per SNP
3	Angiotensi	Gnanasaml	26482465	< 3 populations reported per SNP
3	Associatior	Pawlik M et	23681285	< 3 populations reported per SNP
3	Associatior	Pei Y et al.	9259580	< 3 populations reported per SNP
3	Chronic rer	Prasad P et	16672053	< 3 populations reported per SNP

For peer review only

3	Contributic	Marre M et	9120002	< 3 populations reported per SNP
3	Gene polyr	Su SL et al.	22147663	< 3 populations reported per SNP
3	Genes invo	Litovkina O	24727057	< 3 populations reported per SNP
3	Genetic po	Miura J et a	10499884	< 3 populations reported per SNP
3	Genetic po	Zsom M et	22111818	< 3 populations reported per SNP
3	Genetic po	Fabris B et	15662219	< 3 populations reported per SNP
3	Genetic var	Shaikh R et	24737640	< 3 populations reported per SNP
3	Hypertensi	El-Essawy A	11926202	< 3 populations reported per SNP
3	Interaction	Stratta P et	14767013	< 3 populations reported per SNP
3	M235T Pol	Sarkar S et	25660845	< 3 populations reported per SNP
3	Polymorph	Ayed K et a	16635753	< 3 populations reported per SNP
3	Polymorph	Sprovieri SI	15934435	< 3 populations reported per SNP
3	Renin-angi	Parsa A et a	15789057	< 3 populations reported per SNP
3	Renin-angi	Mtiraoui N	21421655	< 3 populations reported per SNP
3	Renin-angi	Chen WJ et	24907556	< 3 populations reported per SNP

For peer review only

Supplementary Table S3d

**Exclusion S**	**Title**	**Authors**	**PMID**	**Reason**
1	Identificati	Chen, Z; W	27756246	Gene expression based study
1	Polymorph	Liu, ZH; Li, LS		No data for AGTR1
1	The effect	Nakayama	16312263	No data for AGTR1
1	The geneti	Heringer-W	22652430	Non-human
1	Angiotensi	Maeda A, T	23902639	Non-human
1	Cardiac ph	van Esch, JI	20071356	Non-human
1	CCN1 expr	Bonda, TA;	23690222	Non-human
1	Human GR	Wang, Z; Z	26667412	Non-human
1	Losartan ar	Srisawat, U	25947921	Non-human
1	The angiot	Hunley, TE;	10652034	Non-human
1	Mechanism	Todkar, A;	25071088	Non-human
1	Physiologic	Le, TH; Kim	12963678	Non-human
1	Specific pr	Bird, IM; M	9687288	Non-human
1	A systemat	Feng, X; Zh	23178513	Not a case-control study
1	Associatior	Ding, W; W	21607620	Not a case-control study
1	Associatior	Zhou, TB; Y	23971628	Not a case-control study
1	Genetic de	Cardinal-Fe	22436318	Not a case-control study
1	A Synergist	Rodriguez-I	19546528	Not a case-control study
1	Add-on ang	Agarwal, R	11380832	Not a case-control study
1	Analysis of	Torres VE,	22205355	Not a case-control study
1	Angiotensi	Woo KT, La	18536822	Not a case-control study
1	Associatior	Akcay A, Se	15385810	Not a case-control study
1	Gene Polyn	Zhang, G; V	17984617	Not a case-control study
1	Impact of g	Siekierka-H	19681973	Not a case-control study
1	Influence o	Bantis, C; Iv	15031629	Not a case-control study
1	Polymorph	Frimat, L; P	11053482	Not a case-control study
1	Survival in	Padro-Miqu	19014923	Not a case-control study
1	Angiotensi	Miller, JA; T	10969844	Not a renal disease focus
1	Associatior	Song SB, Jir	21342026	Not a renal disease focus
1	Associatior	Assali, A; G	21570644	Not a renal disease focus
1	Eprosartan	Vase, H; La	18341678	Not a renal disease focus
1	Functional	Coto E, Pala	20594303	Not a renal disease focus
1	Genetic po	Wu, CK; Tsa	19330904	Not a renal disease focus
1	Genetic risl	van Onna, I	15326089	Not a renal disease focus
1	Genetic vai	Ichikawa, M	24549414	Not a renal disease focus
1	Genetic vai	Hsu, CCC; E	16396964	Not a renal disease focus
1	Genomic as	Turner ST, I	22566498	Not a renal disease focus
1	Increased e	Yamani, MI	17097490	Not a renal disease focus
1	Peripheral	Thomas, GI	14632699	Not a renal disease focus
1	Polymorph	Deckers, IA	24978482	Not a renal disease focus
1	Polymorph	Bofinger, A	11317203	Not a renal disease focus
1	Renin-angi	BuraczyÅ„s	14502296	Not a renal disease focus
1	Renin-Angi	Joyce-Tan, SM; Zain, SI		Not a renal disease focus
1	Single nucl	Demir AK, I	27936341	Not a renal disease focus
1	The M235T	van den Bo	17921816	Not a renal disease focus

For peer review only

1	Angiotensi	Sparks, MA	21242463	Not a renal disease focus
1	Associatior	Conen D, G	18698212	Not a renal disease focus
1	Compreher	Kato, N; Su	10953993	Not a renal disease focus
1	Deep-targe	Pescatello, LS; Schifanc		Not a renal disease focus
1	Genetic Clu	Krcunovic, Z; Novakovi		Not a renal disease focus
1	Polymorph	Baudin, B	15640279	Not a renal disease focus
1	Renin-angi	Lau YK, Wo	15153745	Not a renal disease focus
1	[AGTR1 A1	Yin X, Li H,	23505107	Not written in English or Spanish
1	[Associatio	BuraczyÅ„s	12476891	Not written in English or Spanish
1	[Effect of e	Kaliuzhin V	24261234	Not written in English or Spanish
1	[Genetic pr	Bzoma B, D	19112833	Not written in English or Spanish
1	[Identificat	Savost'ianc	12500539	Not written in English or Spanish
1	[Pharmaco	Mormol' IA	25286611	Not written in English or Spanish
1	[Polymorpl	Chistiakov	10576062	Not written in English or Spanish
1	[Renin-ang	BuraczyÅ„s	11865575	Not written in English or Spanish
1	ACE gene p	Haszon, I; F	12478352	Paediatric Individuals
1	Renin-angi	Papp, F; Fri	12579405	Paediatric Individuals
1	ACE and AT	Kostic, M; S	15179569	Paediatric Individuals
1	Angiotensi	Hohenfelln	10353402	Paediatric Individuals
1	Angiotensi	Gallego, PH	18413222	Paediatric Individuals
1	Donor and	Buscher, R;	21309964	Paediatric Individuals
1	Effects of g	Tabel Y, Be	16525944	Paediatric Individuals
1	Genetic po	Yim, HE; Ju	14764974	Paediatric Individuals
1	Genetic po	Elshamaa, I	21859496	Paediatric Individuals
1	Genetic po	Frishberg, Y	9853248	Paediatric Individuals
1	Genetic risl	Taranta, A;	19110485	Paediatric Individuals
1	Impact of c	Hussein, A;	25939993	Paediatric Individuals
1	Polymorph	Maruyama,	11354780	Paediatric Individuals
1	Polymorph	Ece, A; Tek	16109085	Paediatric Individuals
1	Renin-angi	Pardo, R; M	12579398	Paediatric Individuals
1	Renin-angi	Liu, KP; Lin,	15045574	Paediatric Individuals
1	Implication	Ozen S, Alik	10401028	Paediatric Individuals
1	Mapping ca	Weber, S; L	20605837	Paediatric Individuals
1	Renin-angi	Ozkaya, O;	16521052	Paediatric Individuals
1	Effect of AC	BÃ¶ger CA,	16961167	Pharmaceutical drug focus
1	Chronic rer	Prasad P, T	16672053	Pharmaceutical drug focus
1	CYP2C9 ger	Joy MS, Do	19669737	Pharmaceutical drug focus
1	Enalapril ar	Rashtchizad	17222813	Pharmaceutical drug focus
1	Evaluation	Palmer, ND	24551085	Pharmaceutical drug focus
1	Long-term	Andersen S	12716812	Pharmaceutical drug focus
1	The influen	Goyache-G	24241364	Pharmaceutical drug focus
1	Genetic po	BuraczyÅ„s	12898858	Pharmaceutical drug focus
1	Genotypic i	Mehri, S; K	20580725	Pharmaceutical drug focus
2	A polymorp	Möllsten A,	21316998	Incorrect patient group
2	Associatior	Yoshida T, I	19056482	Overlap in patient group
2	Combinatic	Osawa N, K	17143591	No data
2	Effects of tl	Luther Y, Ba	14610337	No data

For peer review only

2	Gene polyr	Su, SL; Lu, I	22147663	Overlap in patient group
2	Lack of syn	Tarnow L, I	10907125	No data
2	Polymorph	Woo, KT; La	15504143	Overlap in patient group
2	The renin--	Ong-Ajyoot	10511770	No data
3	Polymorph	Ayed K et a	16635753	< 3 populations reported per SNP
3	Angiotensi	Buraczynsk	12187084	< 3 populations reported per SNP
3	Genetic po	Buraczynsk	16384824	< 3 populations reported per SNP
3	Renin-angi	Chen WJ et	24907556	< 3 populations reported per SNP
3	Hypertensi	El-Essawy A	WOS:0001	< 3 populations reported per SNP
3	Genetic po	Fabris B et	15662219	< 3 populations reported per SNP
3	Relationshi	Fradin S et	11938025	< 3 populations reported per SNP
3	Susceptibili	Hanna MO	25316403	< 3 populations reported per SNP
3	Renin-angi	Kawada N e	WOS:0000	< 3 populations reported per SNP
3	GENETIC CI	Krcunovic Z	WOS:0002	< 3 populations reported per SNP
3	Angiotensi	Lee KB et a	12950120	< 3 populations reported per SNP
3	Genes invo	Litovkina O	24727057	< 3 populations reported per SNP
3	Polymorph	Losito A et	WOS:0001	< 3 populations reported per SNP
3	Contributic	Marre M et	9120002	< 3 populations reported per SNP
3	Associatior	Pei Y et al.	9259580	< 3 populations reported per SNP
3	Polymorph	Sprovieri SI	15934435	< 3 populations reported per SNP
3	Interaction	Stratta P et	14767013	< 3 populations reported per SNP
3	Gene polyr	Su SL et al.	22147663	< 3 populations reported per SNP
3	Gene-Gene	Su SL et al.	24977181	< 3 populations reported per SNP
3	Associatior	Yoshida T e	19406964	< 3 populations reported per SNP
3	Genetic po	Zsom M et	22111818	< 3 populations reported per SNP

For peer review only

Supplementary Table S3e

**Exclusion S**	**Title**	**Authors**	**PMID**	**Reason**
1	Associatior	Pei Y, Scho	9259580	No data for AGTR2
1	Synergistic	Doria A, Or	9389421	No data for AGTR2
1	Lack of syn	Tarnow L, I	10907125	No data for AGTR2
1	Angiotensi	Miller JA, T	10969844	No data for AGTR2
1	Polymorph	Frimat L, P	11053482	No data for AGTR2
1	Altered reg	O'Tierney,	18192845	No data for AGTR2
1	The angiot	Hunley, TE	10652034	Non-human
1	Renin-angi	Pardo, R; M	12579398	Not a case-control study
1	Associatior	Akcay A, Se	15385810	Not a case-control study
1	Whole-gen	Cordell HJ,	19959718	Not a case-control study
1	Associatior	Laksmi NK,	20149750	Not a case-control study
1	Angiotensi	Hohenfellr	10353402	Not a renal disease focus
1	Angiotensi	Yoneda, A;	12187255	Not a renal disease focus
1	Genetic po	Yim, HE; Ju	14764974	Not a renal disease focus
1	Polymorph	Baudin, B	15640279	Not a renal disease focus
1	No evidenc	Struwe E, K	16944335	Not a renal disease focus
1	[Effect of e	Kaliuzhin V	24261234	Not written in English or Spanish
1	No evidenc	Hiraoka M,	11260384	Paediatric Individuals
1	Polymorph	Maruyama	11354780	Paediatric Individuals
1	Angiotensi	Rigoli L, Ch	15470205	Paediatric Individuals
1	Implicatior	Hahn H, Ku	16133060	Paediatric Individuals
1	Angiotensi	Siomou, E;	17515833	Paediatric Individuals
1	Associatior	Miranda D	24995698	Paediatric Individuals
2	Angiotensi	Pedersen-E	19820429	Incorrect patient group

For peer review only

Supplementary Table S3f

Exclusion S	Title	Authors	PMID	Reason
1	Increased amount of the angiotens	Suehiro T, Morita T, Inoue I	15164285	Gene expression based
1	Increased expression of monocytic	Ulrich, C; Heine, GH; Garcia	16476718	Gene expression based
1	Synergistic expression of angiotens	Wakahara, S; Konoshita, T;	17303661	Gene expression based
1	A biallelic gene polymorphism of C\	Nicod J, Bruhin D, Auer L, V	12788845	No data for REN
1	A candidate gene approach to gene	Yamamoto, R; Nagasawa, Y	21737517	No data for REN
1	A polymorphism in the angiotensin	Mollsten, A; Vionnet, N; For	21316998	No data for REN
1	ACACÎ² gene (rs2268388) and AGTR	Shah VN, Cheema BS, Sharr	23081748	No data for REN
1	ACE variants interact with the RAS	Ahluwalia TS, Ahuja M, Rai	19108684	No data for REN
1	ACE, PAI-1, decorin and Warner hel	De Cosmo, S; Margaglione,	10495473	No data for REN
1	Aldosterone synthase (CYP11B2)-34	Lajer, M; Schjoedt, KJ; Jacol	16759311	No data for REN
1	Aldosterone Synthase CYP11B2 Ger	Yilmaz, M; Sari, I; Bagci, B; (	25957425	No data for REN
1	Analysis of insertion/deletion polyn	Ali A, Vasudevan R, Ismail P	21421653	No data for REN
1	Analysis of polymorphism in Renin	Anbazhagan, K; Sampathku	19520069	No data for REN
1	Angiotensin converting enzyme ger	Yildiz A, Yazici H, Cine N, Ka	12832741	No data for REN
1	Angiotensin I converting enzyme ar	Gutierrez, C; Vendrell, J; Pa	9258285	No data for REN
1	Angiotensin I converting enzyme ge	Schmidt, S; Ritz, E	9269698	No data for REN
1	Angiotensin II type 1 receptor (A1	El-Banawy, H; Bedair, R; Mo	NA	No data for REN
1	Angiotensin II type 1 receptor gene	Buraczynska, M; Ksiazek, P;	12187084	No data for REN
1	Angiotensin-converting enzyme (AC	So, WY; Ma, RCW; Ozaki, R;	16395257	No data for REN
1	Angiotensin-converting enzyme pol	Ortiz, MA; De Prado, A; Dor	15012717	No data for REN
1	Angiotensin-I converting enzyme ge	Ergen, HA; Hatemi, H; Agac	NA	No data for REN
1	Angiotensin-II type 1 receptor gene	Tarnow L, Cambien F, Rossi	8671962	No data for REN
1	Angiotensinogen gene polymorphis	Gnanasambandan, R; Eluma	26482465	No data for REN
1	Angiotensinogen M235T and chyma	Zychma, MJ; Zukowska-Szcz	11096141	No data for REN
1	Apparent Mineralocorticoid Excess	Alzahrani, AS; Aljuhani, N; (	24936560	No data for REN
1	Assessment of matrix Gla protein, K	Karsli CeppioÄŸlu S, Yurdun	21859400	No data for REN
1	Association analysis of ADPRT1, AKI	Prasad, P; Tiwari, AK; Kuma	20353610	No data for REN
1	Association between angiotensin co	Uemasu, J; Nakaoka, A; Kav	9180368	No data for REN
1	Association between CCDC132, FD>	Niu D, Ren Y, Xie L, Sun J, Lu	26370181	No data for REN
1	Association of aldosterone synthase	Pawlik M, Mostowska A, Lia	23681285	No data for REN
1	Association of apolipoprotein E gen	Lahrach H, Essiarab F, Timir	25155022	No data for REN
1	Association of glutathione S-transfe	Datta SK, Kumar V, Pathak I	20954980	No data for REN
1	Association of plasminogen activato	Xu F, Liu H, Sun Y.	26616527	No data for REN
1	Association of sequence polymorph	Bai Y, Guo Z, Xu J, Zhang J, (	24576051	No data for REN
1	Association of the angiotensinogen	Reis KA, EbinÃ§ FA, KoÃ§ E,	21500980	No data for REN
1	Association of the genetic polymor	Li, X; An, J; Guo, R; Jin, Z; Li,	20540812	No data for REN
1	Association studies between the HS	Lavery, GG; McTernan, CL; I	11916625	No data for REN
1	Association study of ACE polymorpl	Gong, AM; Li, XY; Wang, YQ	22729880	No data for REN
1	Associations between apolipoprote	Arikan H, Koc M, Sari H, Tug	17763167	No data for REN
1	BcÄ±Ä±--RFLP profiles for serum an	Ozdemir O, Kayatas M, Ceti	25394530	No data for REN
1	Blood Pressure-Related Genes and	Kim, SM; Chin, HJ; Oh, YK; K	19729965	No data for REN
1	Circulating angiotensin-converting (	Anguiano, L; Riera, M; Pasc	25813276	No data for REN
1	Clinical impact of an angiotensin I-c	Amorim, CEN; Nogueira, E;	23362199	No data for REN
1	Combinational effect of genes for tl	Osawa, N; Koya, D; Araki, S;	17143591	No data for REN
1	Different Mechanisms for the Progr	Nakayama, Y; Nonoguchi, H	19293592	No data for REN

For peer review only

For peer review only - http://bmjopen.bmj.com/site/about/guidelines.xhtml

1	Distribution of ACE I/D Polymorphis	Shaikh, R; Shahid, SM; Naw	NA	No data for REN
1	DNA polymorphisms in the ACE ger	Freire, MBS; van Dijk, DJ; Er	9794558	No data for REN
1	DNA repair genes XPD and XRCC1 p	Radwan WM, Elbarbary HS,	25310768	No data for REN
1	Effect of ACE gene on diabetic nepł	Kuramoto, N; Iizuka, T; Ito,	10023638	No data for REN
1	Effect of IL-6 C-572G polymorphism	Chen SY, Chen CH, Huang Y	20954977	No data for REN
1	Effects of erythropoietin, angiotens	Glicklich, D; Kapoian, T; Mia	10428268	No data for REN
1	Effects of the genetic polymorphisr	Luther, Y; Bantis, C; Ivens, K	14610337	No data for REN
1	Endothelial nitric oxide synthase ge	Cherney, DZI; Scholey, JW; ;	19037250	No data for REN
1	Factors affecting progression of rer	Locatelli, F; Manzoni, C; Ma	9387138	No data for REN
1	Familial renal glucosuria: SLC5A2 m	Calado, J; Loeffler, J; Sakalli	16518345	No data for REN
1	GAS6 intron 8 c.834 + 7G > A gene		Erkoc R, Cikrikcioglu MA, Ai	25869052	No data for REN
1	Gene polymorphisms of angiotensi	Su, SL; Lu, KC; Lin, YF; Hsu, `	22147663	No data for REN
1	Gene-Gene Interactions in Renin-A	Su, SL; Yang, HY; Wu, CC; Le	NA	No data for REN
1	Genes involved in the regulation of	Litovkina, O; Nekipelova, E;	24727057	No data for REN
1	Genetic Clues To The Etiology Of Ba	Krcunovic, Z; Novakovic, I; I	NA	No data for REN
1	Genetic determination of TNF and	Buraczynska K, Koziol-Mont	15600254	No data for REN
1	Genetic polymorphisms of the renii	Buraczynska, M; Ksiazek, P;	16384824	No data for REN
1	Genetic polymorphisms of the renii	Huang, HD; Lin, FJ; Li, XJ; W	21163122	No data for REN
1	Genetic polymorphisms of the renii	Buraczynska, M; Ksiazek, P;	16384824	No data for REN
1	Genetic variants of ACE (Insertion/I	Shaikh, R; Shahid, SM; Man	24737640	No data for REN
1	High prevalence of ACE DD genotyp	Tripathi G, Dharmani P, Kha	17042963	No data for REN
1	Identification of Cathepsin L as a Pc	Bauer, Y; Hess, P; Qiu, CB; K	21357272	No data for REN
1	Impact of aldosterone synthase ger	Bantis C, Heering PJ, Siekier	21476902	No data for REN
1	Impact of interaction of cigarette sı	Yang HY, Lu KC, Fang WH, L	23477970	No data for REN
1	Impact of interferon-gamma and in	Masutani, K; Miyake, K; Nal	12552499	No data for REN
1	Impact of nitric oxide synthase Glu	El-Din Bessa SS, Hamdy SM,	21854353	No data for REN
1	Impact of Polymorphisms of the Ge	Jung, ES; Kim, SM; Cha, RH;	21150220	No data for REN
1	Increased frequency of angiotensin	Hsieh, MC; Lin, SR; Hsieh, T	10862639	No data for REN
1	Influence of aldosterone synthase g	Bantis, C; Heering, PJ; Stang	21777344	No data for REN
1	Influence of angiotensin converting	Ramanathan, G; Ghosh, S; E	27748299	No data for REN
1	Influence of cytokine gene polymor	Bantis, C; Heering, PJ; Luthe	15308875	No data for REN
1	Influence of the endothelial nitric o	Merta M, ReiterovÃ¡ J, Tesa	12212826	No data for REN
1	Interaction between gene polymor|	Stratta, P; Bermond, F; Gua	14767013	No data for REN
1	Interleukin 1 receptor antagonist ai	Shu KH, Cheng CH, Wu MJ,	15717635	No data for REN
1	Lack of association between the an	Arfa I, Abid A, Nouira S, Ellc	18404607	No data for REN
1	Lack of association between the he	Fujita H, Narita T, Meguro H	10586428	No data for REN
1	Lack of association between transie	Chen WC, Chen SY, Chen CH	20540633	No data for REN
1	Lack Of Relationship Between An In	Tarnow, L; Cambien, F; Ross	7729604	No data for REN
1	Lack of synergism between long-tei	Tarnow, L; Kjeld, T; Knudsei	10907125	No data for REN
1	Manganese Superoxide Dismutase	Mohammedi, K; Bellili-Mun	24819633	No data for REN
1	Manganese superoxide dismutase,	Kidir V, Uz E, Yigit A, Altunta	26787049	No data for REN
1	Mthfr C677T, A1298C And Ace I/D I	El-Baz R, Settin A, Ismaeel A	22554825	No data for REN
1	Nodular glomerulosclerosis and rer	Wang, M; Zhang, XX; Song,	26973293	No data for REN
1	Non-relation of parathyroid hormo	Chen JB, Chou FF, Hsu KT.	15083922	No data for REN
1	Platelet glycoprotein IIb HPA-3 a/b	Wu JH, Zhang DW, Cheng X	22880801	No data for REN
1	Polymorphism of angiotensin conve	Frimat, L; Philippe, C; Magh	11053482	No data for REN
1	Polymorphism of renin-angiotensin	Losito, A; Kalidas, K; Santon	12454231	No data for REN

1	Polymorphism of renin-angiotensin	Woo, KT; Lau, YK; Choong, I	15504143	No data for REN
1	Polymorphism of the angiotensin I-	Hanyu O, Hanawa H, Nakag	9509566	No data for REN
1	Polymorphisms in NADPH oxidase (	Zhou H, Chen M, Zhu Y, Wa	26627442	No data for REN
1	Polymorphisms in the gene encodir	Frojdo, S; Sjolind, L; Parkkoi	16211375	No data for REN
1	Polymorphisms of the renin-angiot	Sprovieri, SRS; Sens, YAS	15934435	No data for REN
1	Predicting the development of diab	William, J; Hogan, D; Batlle,	15822056	No data for REN
1	Pronatriodilatin gene polymorphisr	Nannipieri, M; Penno, G; Pu	10405209	No data for REN
1	Relationship between angiotensino	Wang J, Zhu X, Yang L, Liu Y	11717948	No data for REN
1	Relationship between GSTs gene pc	Agrawal S, Tripathi G, Khan	18067039	No data for REN
1	Renal clearance of endogenous ery	Nowicki M, Kokot F, Kokot I	7759206	No data for REN
1	Renal perfusion and the renal hem	Lansang, MC; Hollenberg, N	12086929	No data for REN
1	Renin-aldosterone response, urinar	Hanukoglu, A; Edelheit, O; S	18634878	No data for REN
1	Renin-angiotensin system compone	Kawada, N; Moriyama, T; Y	NA	No data for REN
1	Renin-angiotensin system gene pol	Lau YK, Woo KT, Choong HL	15153745	No data for REN
1	Renin-angiotensin-aldosterone syst	Chen, WJ; Huang, YL; Shiue,	24907556	No data for REN
1	Role of ADDUCIN Gly460Trp, ACE I/	Sancakdar, E; Ates, K; Kama	NA	No data for REN
1	Role of the alpha-adducin genotype	Nicod, J; Frey, BM; Frey, FJ;	11918733	No data for REN
1	Serological and genetic factors in ea	Coppo, R; Amore, A; Chiesa	17988266	No data for REN
1	SIRTUIN 1 gene polymorphisms are	Shimoyama Y, Mitsuda Y, Ts	22200427	No data for REN
1	Structural analysis of the 11 beta-h	Zaehner, T; Plueshke, V; Fre	11012876	No data for REN
1	Study of the association of -667 aq	Kang SW, Kim YW, Kim YH,	17763164	No data for REN
1	Study on 3'-UTR length polymorphi	Sui W, Zheng C, Yang M, Ou	26554293	No data for REN
1	Survival in type 2 diabetic patients i	Padro-Miquel, A; Alia-Ramo	19014923	No data for REN
1	The DD genotype of the ACE gene p	Azar, ST; Zalloua, P; Medlej,	11428725	No data for REN
1	The DD genotype of the ACE gene p	Vleming, LJ; van der Pijl, JW	10099885	No data for REN
1	The effect of polymorphisms in the	Mollsten, A; Kockum, I; Sve	18413189	No data for REN
1	The influence of G-protein beta3-su	ReiterovÃ¡ J, Miroslav M, St	15287194	No data for REN
1	The influence of the endothelin-cor	ReiterovÃ¡ J, Merta M, Stek	16526315	No data for REN
1	The relationship between genetic a	Shestakova, MV; Vikulova, (	NA	No data for REN
1	The relationship between the TGF-l	Li Y, Liu FY, Peng YM, Li J, Su	20446778	No data for REN
1	The renin--angiotensin system gene	Ong-Ajyooth S, Ong-Ajyootl	10511770	No data for REN
1	Toll-like receptor 9 SNPs are suscep	Chen YT, Wei CC, Ng KL, Ch	23964786	No data for REN
1	U.K. Prospective Diabetes Study. X\	Dudley CR, Keavney B, Strat	8587251	No data for REN
1	Vitamin D binding protein and the r	Speeckaert MM, Glorieux G	18721734	No data for REN
1	Vitamin D receptor genetic variants	Tripathi G, Sharma R, Sharn	20722565	No data for REN
1	20-Hydroxyeicosatetraenoic Acid (H	Savas, S; Wei, SZ; Hsu, MH;	NA	Non-human
1	Adenosine A(1) receptor-dependen	Gao, X; Peleli, M; Zollbrecht	25251152	Non-human
1	Adrenomedullin gene expression di	Caron, K; Hagaman, J; Nishi	17360661	Non-human
1	Altered regulation of renal interstit	O'Tierney, PF; Komolova, M	18192845	Non-human
1	Alternative splicing of vitamin D-24	Ren S, Nguyen L, Wu S, Enc	15788398	Non-human
1	An essential role of angiotensin II re	Hisada, Y; Sugaya, T; Tanaka	11555672	Non-human
1	Angiotensin converting enzyme (AC	Shahid, SM; Fatima, SN; Ma	24035938	Non-human
1	Angiotensin II mesenteric and renal	Broome, M; Aneman, A; Ha	11065204	Non-human
1	Angiotensin-converting enzyme inh	Jonsson, JR; Clouston, AD; A	11438504	Non-human
1	Angiotensin-Ii Enhances Norepinep	NOSHIRO, T; SHIMIZU, K; W	NA	Non-human
1	Antihypertensive Role of Tissue Kal	Waeckel, L; Potier, L; Cholle	22669897	Non-human
1	Appropriate regulation of renin anc	Catanzaro DF, Chen R, Yan Y	9931123	Non-human

For peer review only

1	Blood pressure and renal hemodyn	Kumari, S; Sharma, N; Thak	27350671	Non-human
1	Blood pressure and renin-angioten:	Bohlender, J; Bader, M; Me	22728903	Non-human
1	Cardiac angiotensin-(1-12) expressi	Ferrario CM, VonCannon J,	26873967	Non-human
1	Cardiovascular dysfunction in Zucke	Marsh, SA; Powell, PC; Agar	17351065	Non-human
1	Chronic hypertension and altered b	Merrill, DC; Thompson, MW	8613528	Non-human
1	Collecting duct-specific knockout of	Gao, Y; Stuart, D; Pollock, JS	27707708	Non-human
1	Compensatory up-regulation of ang	Wang, Q; Hummler, E; Mail	11380824	Non-human
1	Control of renin secretion from kidr	Dong Y, Wang M, Nong L, W	25824806	Non-human
1	Cosegregation of spontaneously hy	Gao, Y; Stuart, D; Pollock, JS	27707708	Non-human
1	Dendritic cell nuclear protein-1, a n	Zhou T, Wang S, Ren H, Qi X	20693543	Non-human
1	Effect Of Angiotensin-Converting Er	NOSHIRO, T; WAY, D; MCGI	1648463	Non-human
1	Effect Of Nitric-Oxide On Renin Sec	GREENBERG, SG; HE, XR; SC	7771523	Non-human
1	Effect of SWL on renal hemodynam	Yilmaz, E; Mert, C; Keskil, Z;	22945811	Non-human
1	Effect of the angiotensinogen geno	Handtrack, C; Cordasic, N; K	17333097	Non-human
1	Effects of plasma kallikrein deficien	Bird, JE; Smith, PL; Wang, X	22398951	Non-human
1	Elevated blood pressures in mice la	Shesely, EG; Maeda, N; Kim	8917564	Non-human
1	Endothelium-Dependent Relaxatior	STEPHAN, D; BILLING, A; KR	8606521	Non-human
1	Functional genetic variation in amir	Tonna, S; Dandapani, SV; U	18206321	Non-human
1	Gene Trapping Uncovers Sex-Specif	Park, S; Liu, XB; Davis, DR; S	22547438	Non-human
1	Genetic analysis of the S-A and Na+	Lodwick, D; Zagato, L; Kaise	9535139	Non-human
1	GPR48 Increases Mineralocorticoid	Wang, J; Li, XY; Ke, YY; Lu, Y	22135314	Non-human
1	Hypertension in unilaterally nephre	Marley, WS; Bowen, R; Got	10567853	Non-human
1	Hypervolemia of pregnancy is not n	Morgan, TK; Rohrwasser, A	16796982	Non-human
1	Knockdown of parathyroid hormon	Raison, D; Coquard, C; Hoch	23720345	Non-human
1	Lack of an effect of collecting duct-s	Kittikulsuth, W; Stuart, D; V	24431204	Non-human
1	Local Renal Circadian Clocks Contro	Tokonami, N; Mordasini, D;	24652800	Non-human
1	Low Blood Pressure in Endothelial C	Kisanuki, YY; Emoto, N; Ohu	20516397	Non-human
1	Metformin prevents the impairmer	Gomes, MB; Cailleaux, S; Ti	16133490	Non-human
1	Mice lacking endothelial ACE - Norr	Cole, JM; Khokhlova, N; Sut	12574101	Non-human
1	Mice with targeted disruption of th	Langaa, S; Bloksgaard, M; B	22237802	Non-human
1	N-domain angiotensin I-converting	Bueno, V; Palos, M; Ronchi,	15194348	Non-human
1	Nebivolol treatment improves resis	Wang, Y; Zhang, MS; Liu, Y	23263161	Non-human
1	Nephron-specific deletion of the pr	Ramkumar, N; Stuart, D; Ca	25995108	Non-human
1	Parallel regulation of renin and lysc	Schmid, J; Oelbe, M; Saftig,	23229015	Non-human
1	Physiological impact of increased e:	Le, TH; Kim, HS; Allen, AM;	12963678	Non-human
1	Pituitary adenylate cyclase-activatir	Hautmann, M; Friis, UG; De	17360952	Non-human
1	Preserved macula densa-dependen	Schweda, F; Wagner, C; Kra	12475747	Non-human
1	Rat Ace allele variation determines	Kamilic, J; Hamming, I; Lely,	21788250	Non-human
1	Reciprocal expression of connexin 4	Kurt, B; Kurtz, L; Sequeira-L	21209011	Non-human
1	Regulation of renin secretion and e	Kim, SM; Chen, LM; Faulhab	17515456	Non-human
1	Renal cyst growth is the main deter	Fonseca, JM; Bastos, AP; Ar	24429399	Non-human
1	Replacement of connexin 40 by cor	Kurtz, L; Gerl, M; Kriz, W; W	19474190	Non-human
1	Role of angiotensin-converting enzy	Yang, XH; Wang, YH; Wang,	22580272	Non-human
1	Role of blood pressure and the reni	Zhang, MZ; Wang, SW; Yang	22114203	Non-human
1	Role of cGMP-kinase II in the contrc	Wagner, C; Pfeifer, A; Ruth,	9788971	Non-human
1	Role of neutral endopeptidase 24.1	Wegner, M; HirthDietrich, C	8759244	Non-human
1	Stimulation of renin release by pros	Schweda, F; Klar, J; Narumiy	15113745	Non-human

For peer review only

1	Stimulation of renin secretion by ni	Kurtz, A; Gotz, KH; Hamann	9539809	Non-human
1	Stimulation of renin secretion by N(	Kurtz, A; Gotz, KH; Hamann	9575895	Non-human
1	The angiotensin II receptor blocker	Laesser, M; Oi, Y; Ewert, S;	14995942	Non-human
1	The angiotensin type II receptor tor	Hunley, TE; Tamura, M; Sto	10652034	Non-human
1	The elevated blood pressure of hur	Wang, Z; Armando, I; Asico,	17259440	Non-human
1	The genetic deletion of Mas abolish	Heringer-Walther, S; Gemb	22652430	Non-human
1	Thyroid hormones stimulate renal e	Pinto, ACOD; Barbosa, CML	NA	Non-human
1	Transfer of a salt-resistant renin all(	Jiang, J; Stec, DE; Drummor	9040448	Non-human
1	Vascular and renal effects of vasope	Cheng, ZJ; Gronholm, T; Lou	16093923	Non-human
1	Vascular angiotensin-converting en	Muller, DN; Bohlender, J; H	9039087	Non-human
1	A female with X-linked Alport syndr	Mohammad, M; Nanra, R; (	24337245	Not a case-control stuc
1	A Genetic Variant in the Distal Enha	Makino, Y; Konoshita, T; Or	26366736	Not a case-control stuc
1	A Synergistic Association of ACE I/D	Rodriguez-Perez, JC; Macias	19546528	Not a case-control stuc
1	ACE gene polymorphism in focal se	Kuzmanic, D; Jelakovic, B; B	NA	Not a case-control stuc
1	Alport Syndrome in Women and Gi	Savige, J; Colville, D; Rheaul	27287265	Not a case-control stuc
1	Angiotensin converting enzyme ger	Miller, JA; Scholey, JW; Tha	8995725	Not a case-control stuc
1	Angiotensin-converting enzyme ger	Beige, J; Scherer, S; Weber,	9259361	Not a case-control stuc
1	Angiotensin-converting enzyme ger	Akcay, A; Ozdemir, FN; Atac	15013293	Not a case-control stuc
1	Angiotensin-converting-enzyme ins	Beige, J; Offermann, G; Dist	9550656	Not a case-control stuc
1	Angiotensinogen concentrations an	Bohlender J, MÃ©nard J, G	10720595	Not a case-control stuc
1	Angiotensinogen gene variation and	Narita, I; Goto, S; Saito, N; S	12911556	Not a case-control stuc
1	Antihypertensive treatment modula	Kuznetsova, T; Staessen, JA	10918550	Not a case-control stuc
1	Assessing genetic susceptibility to c	Tanaka, N; Babazono, T	16174281	Not a case-control stuc
1	Association between Angiotensin I-	Huang, ZK; Wu, B; Tao, J; Ha	26000752	Not a case-control stuc
1	Association between two genetic p	Ding, W; Wang, FR; Fang, Q	21607620	Not a case-control stuc
1	Association of angiotensinogen gen	Pei, Y; Scholey, J; Thai, K; Su	9259580	Not a case-control stuc
1	Association of the genetic polymor	Akcay, A; Sezer, S; Ozdemir	15385810	Not a case-control stuc
1	Bilateral high origins of testicular ar	Geller, DS; Zhang, JH; Zenna	16611713	Not a case-control stuc
1	Catalase activity, allelic variations ir	Mohammedi, K; Patente, TA	24057136	Not a case-control stuc
1	Contribution of gene polymorphism	Anguiano, L; Riera, M; Pasc	25813276	Not a case-control stuc
1	Correlates of ACE activity in macroa	Amorim, CEN; Nogueira, E;	23362199	Not a case-control stuc
1	Deletion insertion polymorphism of	Bjorck, S; Blohme, G; Sylver	9269704	Not a case-control stuc
1	Diabetes, nephropathy, and the rer	Hollenberg, NK	16601578	Not a case-control stuc
1	Diagnosis of a case of Gitelman's sy	Kageyama, K; Terui, K; Shoj	16370563	Not a case-control stuc
1	Disorders of mineralocorticoid synt	Connell, JMC; Fraser, R; Dav	11469810	Not a case-control stuc
1	Epidemiology and genetics of calcif	O'Brien, KD	17963677	Not a case-control stuc
1	Expert Guidelines for the Managem	Savige, J; Gregory, M; Gross	23349312	Not a case-control stuc
1	Frequencies of apolipoprotein E all(	Su YY, Zhang YF, Yang S, Wa	25707516	Not a case-control stuc
1	Gene Polymorphisms of the Renin-/	Zhang, G; Wang, H; Wang, I	17984617	Not a case-control stuc
1	Genetic determinants of diabetic re	Schmidt, S; Ritz, E	NA	Not a case-control stuc
1	Genetic polymorphisms of renin-an	BuraczyÅ„ska M, Grzebalsk	12898858	Not a case-control stuc
1	Genetics and the prediction of com	Marre, M	10097900	Not a case-control stuc
1	Genetics of diabetic nephropathy	Rippin, JD; Patel, A; Bain, SC	11554775	Not a case-control stuc
1	Genome-Wide Association Analysis	Musani, SK; Fox, ER; Kraja, /	25561047	Not a case-control stuc
1	Identification of a novel mutation ir	Riepe, FG; Krone, N; Morlot	12679457	Not a case-control stuc
1	Impact of genetic polymorphisms o	Siekierka-Harreis, M; Kuhr,	19681973	Not a case-control stuc
1	Impact of polymorphisms in the rer	Orenes-Pinero, E; Hernande	21507890	Not a case-control stuc

1	Increased left ventricular mass in n	Sato, A; Tarnow, L; Parving,	9727905	Not a case-control stuc
1	Influence of cytokine gene polymor	Bantis C, Heering P, Aker S,	18300111	Not a case-control stuc
1	Influence of genetic polymorphism	Bantis, C; Ivens, K; Kreusser	15031629	Not a case-control stuc
1	Is there a role of angiotensin-conve	Isbir, CS; Akgun, S; Yilmaz, I	11525534	Not a case-control stuc
1	Juxtaglomerular cell tumor of the k	Endoh, Y; Motoyama, T; Ha	9211527	Not a case-control stuc
1	Normative genetic profiles of RAAS	Prasad, P; Thelma, BK	18027817	Not a case-control stuc
1	Novel mutations in the inverted for	Xie J, Hao X, Azeloglu EU, R	26039629	Not a case-control stuc
1	Prognostic role of serum ACE activi	Wong, TYH; Szeto, CC; Chov	11979350	Not a case-control stuc
1	Randomized Controlled Trial: Lisino	Amara, AB; Sharma, A; Alex	20061926	Not a case-control stuc
1	Rapid detection and quantification	Zhou H, Hoek M, Yi P, Rohn	24591025	Not a case-control stuc
1	Relationships between HLA-A, -B, -I	Ozdemir BH, Izdemir FN, Cc	15354972	Not a case-control stuc
1	Renal angiomyolipoma - Further im	Stone, CH; Lee, MW; Amin,	11371226	Not a case-control stuc
1	Renal Tubular Dysgenesis in Israel:	Moldavsky, M	19344005	Not a case-control stuc
1	Renin-angiotensin system gene pol	Parsa, A; Lovett, DH; Peden	15789057	Not a case-control stuc
1	Renoprotective efficacy of renin-an	Narita, I; Goto, S; Saito, N; S	14684698	Not a case-control stuc
1	Rho kinase polymorphism influence	Seasholtz, TM; Wessel, J; Ra	16585408	Not a case-control stuc
1	Risk of developing diabetic nephrop	Savage, DA; Feeney, SA; Fo	10328465	Not a case-control stuc
1	Role Of The Deletion Polymorphism	YOSHIDA, H; MITARAI, T; KA	7593601	Not a case-control stuc
1	Successful treatment of decompen	Csatary, LK; Schnabel, R; Ba	10216467	Not a case-control stuc
1	The deletion/insertion polymorphis	Staessen, JA; Wang, JG; Gin	9488209	Not a case-control stuc
1	The effects of angiotensin-converti	Draman, CR; Kong, NCT; Ga	19037561	Not a case-control stuc
1	The Epithelial Sodium Channel gam	Busst, CJ; Bloomer, LDS; Scu	22006290	Not a case-control stuc
1	The kidney in diabetes: How to con	Mogensen, CE	11158852	Not a case-control stuc
1	The role of renin-angiotensin-aldos	Kelly TN, Raj D, Rahman M,	25906781	Not a case-control stuc
1	Tip lesion variant of primary focal a	Mungan S, Turkmen E, Aydi	25857429	Not a case-control stuc
1	Tip variant of focal segmental glom	Trivedi M, Pasari A, Chowdh	25721429	Not a case-control stuc
1	Twenty-one additional cases of fan	Calado, J; Sznajer, Y; Metzg	18622023	Not a case-control stuc
1	[Possible pathogenetic role of 11 be	Morales MA, Carvajal CA, O	18769825	Not a renal disease foc
1	A clinical phenotype mimicking esse	Rossi E, Farnetti E, Nicoli D,	21525970	Not a renal disease foc
1	A Computational Model of the Circu	Guillaud, F; Hannaert, P	20683640	Not a renal disease foc
1	A functional variant of the NEDD4L	Svensson-FÃ¤rbom P, Wahl	21052022	Not a renal disease foc
1	A new mutation, R563Q, of the bet	Rayner, BL; Owen, EP; King,	12714866	Not a renal disease foc
1	A new theory of essential hyperten	Eggers, AE	26243176	Not a renal disease foc
1	A role for CETP TaqIB polymorphism	Asselbergs, FW; Moore, JH;	16623947	Not a renal disease foc
1	ACE and PC-1 gene polymorphisms	de Azevedo, MJ; Dalmaz, CA	12126783	Not a renal disease foc
1	ACE gene insertion/deletion polym	Lee, YJ; Tsai, JCR	12032106	Not a renal disease foc
1	Activation of the hypothalamic-pitu	Walker BR, Andrew R, Escol	24712576	Not a renal disease foc
1	ADAM33: a newly identified gene ir	Holgate ST, Davies DE, Pow	16257631	Not a renal disease foc
1	Adsorption of cytotoxic anti-HLA ar	DeVito LD, Sollinger HW, Bu	2186524	Not a renal disease foc
1	Albuminuria and the renin-angioter	Thomas, GN; Critchley, JAJH	11200871	Not a renal disease foc
1	Alpha Adducin G460T Variant is a R	Soualmia, H; Ben Romdhan	27349000	Not a renal disease foc
1	Alpha-adducin polymorphism in hy	Barlassina C, Norton GR, Sa	10912759	Not a renal disease foc
1	alpha-Adducin polymorphisms and	Manunta, P; Cusi, D; Barlass	9607177	Not a renal disease foc
1	Amelioration of genetic hypertensi	Sanada, H; Yatabe, J; Midor	16636192	Not a renal disease foc
1	Amiloride, a specific drug for hyper	Baker EH, Duggal A, Dong Y	12105131	Not a renal disease foc
1	Androgen-Sensitive Hypertension A	Wu, CC; Mei, SJ; Cheng, J; D	23641057	Not a renal disease foc
1	Angiotensin converting enzyme ger	Fernandez-Llama, P; Poch, I	9607207	Not a renal disease foc

1	Angiotensin II sensitivity in nonpreg	Spaanderman, MEA; Ekhart	15350256	Not a renal disease foc
1	Angiotensin II type 1 receptor gene	Miller, JA; Thai, K; Scholey,	10969844	Not a renal disease foc
1	Angiotensin II Type 1A Receptors in	Sparks, MA; Parsons, KK; St	21242463	Not a renal disease foc
1	Angiotensin II type 2 receptor gene	Yoneda, A; Cascio, S; Green	12187255	Not a renal disease foc
1	Angiotensin II-dependent chronic h	Touyz, RM; Mercure, C; He,	15753233	Not a renal disease foc
1	Angiotensin-converting enzyme (AC	Mansoor Q, Javaid A, Bilal N	22607040	Not a renal disease foc
1	Angiotensin-converting enzyme ger	Wipff J, Gallier G, Dieude P,	19132786	Not a renal disease foc
1	Angiotensin-converting enzyme ger	Stefansson, B; Ricksten, A; I	10855732	Not a renal disease foc
1	Angiotensin-converting enzyme ger	Singh M, Singh AK, Singh S,	27030424	Not a renal disease foc
1	Angiotensin-converting enzyme ger	Uhm, WS; Lee, HS; Chung, Y	12043886	Not a renal disease foc
1	ANGIOTENSIN-CONVERTING ENZYM	LACHURIE, ML; AZIZI, M; GU	7796503	Not a renal disease foc
1	Angiotensin-converting enzyme ger	Prkacin I, Novak B, SertiÄ‡ J	11505631	Not a renal disease foc
1	Angiotensinogen and angiotensin II	Lee, KB; Kim, UK	12950120	Not a renal disease foc
1	Angiotensinogen genotype affects i	Hopkins, PN; Hunt, SC; Jeur	11997278	Not a renal disease foc
1	Angiotensinogen T235 and ACE inse	Young, RP; Chan, JCN; Critcl	9540028	Not a renal disease foc
1	Apparent mineralocorticoid excess:	Mantero, F; Palermo, M; Pe	8732999	Not a renal disease foc
1	Association between plasma activit	Boomsma F, Pedersen-Bjerg	15830186	Not a renal disease foc
1	Association Between Polymorphism	Li, Y; Li, NF; Yao, XG; Heizat	27555048	Not a renal disease foc
1	Association between polymorphism	Sprovieri, SRS; Sens, YA; Ma	16047641	Not a renal disease foc
1	Association between RAS gene poly	Nalbantoglu, S; Tabel, Y; Mi	23151617	Not a renal disease foc
1	Association between renin-angioter	Song SB, Jin HS, Hong KW, L	21342026	Not a renal disease foc
1	Association of ACE gene D polymor	Bahramali, E; Rajabi, M; Jan	26861937	Not a renal disease foc
1	Association of angiotensin-converti	Zhou, J; Zheng, SC; Wang, Z	26823847	Not a renal disease foc
1	Association of angiotensinogen M2	RodrÃ-guez-PÃ©rez JC, Roc	11345362	Not a renal disease foc
1	Association of hypertension with T5	Baker, EH; Dong, YB; Sagne	9593408	Not a renal disease foc
1	Association of Polymorphisms in En	Mokretar, K; Velinov, H; Po	26670794	Not a renal disease foc
1	Association of renin-angiotensin an	Conen D, Glynn RJ, Buring J	18698212	Not a renal disease foc
1	Associations between the human ir	Frossard PM, Parvez SH, Les	14647005	Not a renal disease foc
1	Atrial Natriuretic Peptide Locally Cc	Nakagawa, H; Oberwinkler,	25027872	Not a renal disease foc
1	Beneficial role of D allele in control	Kumari, S; Sharma, N; Thak	27350671	Not a renal disease foc
1	Biochemical and genetic characteri:	Ozdemir O, Kayatas M, Ceti	25394530	Not a renal disease foc
1	Blunted renal vascular response to	Hopkins, PN; Lifton, RP; Hol	8728297	Not a renal disease foc
1	CARD8 rs2043211 polymorphism is	Chen Y, Ren X, Li C, Xing S, I	25790751	Not a renal disease foc
1	Cardiovascular effects of aldosteroı	Escoubet B, Couffignal C, La	23852419	Not a renal disease foc
1	Clinical and laboratory characteriza	Dong Y, Wang M, Nong L, W	25824806	Not a renal disease foc
1	Common genetic variants and haplo	Barlassina, C; Dal Fiume, C;	17510212	Not a renal disease foc
1	Common genetic variants in the chı	Yu L, Jiang L, Zhou XJ, Zhu L	20113265	Not a renal disease foc
1	Common variation in KLKB1 and es:	Lu, XF; Zhao, WY; Huang, JF	17318641	Not a renal disease foc
1	Comprehensive analysis of the reni	Kato, N; Sugiyama, T; Morit	10953993	Not a renal disease foc
1	Connexin 40 is dispensable for vasc	Machura, K; Neubauer, B; M	25241776	Not a renal disease foc
1	Connexin 43 is not essential for the	Gerl, M; Kurt, B; Kurtz, A; W	24062052	Not a renal disease foc
1	Contribution of angiotensin I conve	Mondorf UF, Russ A, Wiese	9524045	Not a renal disease foc
1	CYP2C9 genotype modifies activity	Osawa, N; Koya, D; Araki, S;	17143591	Not a renal disease foc
1	CYP3A5 and ABCB1 genes and hype	Bochud, M; Bovet, P; Burnie	19290795	Not a renal disease foc
1	CYP3A5 genotype is associated witl	Fromm MF, Schmidt BM, Pa	16141800	Not a renal disease foc
1	Deletion polymorphism of the angi	Gharavi AG, Lipkowitz MS, I	8677872	Not a renal disease foc
1	Detection of the association betwe	Matsumoto, A; Iwashima, Y	11106834	Not a renal disease foc

For peer review only

1	Effect of the plasminogen-plasmin s	Knier, B; Cordasic, N; Klanke	21610512	Not a renal disease foc
1	Efficacy of large doses of IL-2-activa	Cao S, Wang YL, Ren XB, Yu	21812652	Not a renal disease foc
1	Endothelial nitric oxide synthase ge	Page, A; Reich, H; Zhou, J; L	16093452	Not a renal disease foc
1	Evaluation of risk factors for the de	Barnas, U; Schmidt, A; Illiev	9084972	Not a renal disease foc
1	Evaluation Of The Sa Locus In Huma	Nabika, T; Bonnardeaux, A;	7843754	Not a renal disease foc
1	Exaggerated natriuresis during clan	Simonsen, JA; Rasmussen, I	21749320	Not a renal disease foc
1	Extensive personal experience - Exa	Dave-Sharma, S; Wilson, RC	9661590	Not a renal disease foc
1	Frequencies Of Variants Of Candida	Zee, Ryl; Bennett, Cl; Schrad	7882587	Not a renal disease foc
1	Gene polymorphism of vascular enc	Yang, JW; Hutchinson, IV; S	21080079	Not a renal disease foc
1	Genetic polymorphism of Na-K cotr	Cusi D, Stella P, Pozzoli E, B	2258861	Not a renal disease foc
1	Genetic polymorphisms of the renir	Olivieri, O; Trabetti, E; Graz	10567188	Not a renal disease foc
1	Genetic polymorphisms of the renir	Fabris, B; Bortoletto, M; Ca	15662219	Not a renal disease foc
1	Genetic risk of atherosclerotic rena	van Onna, M; Kroon, AA; Ho	15326089	Not a renal disease foc
1	Genetic variant of the renin-angiote	Ichikawa, M; Konoshita, T; I	24549414	Not a renal disease foc
1	Genetic variants in five novel loci in	Jiang DK, Ma XP, Yu H, Cao	25802187	Not a renal disease foc
1	Genetic variants in hypertensive pa	Szperl, M; Dzielinska, Z; Ros	19043368	Not a renal disease foc
1	Genetic variants in the inositol phos	Tan J, Yu CY, Wang ZH, Chen	25683757	Not a renal disease foc
1	Genetic variation in the KCNMA1 po	Tomas, M; Vazquez, E; Fern	18854754	Not a renal disease foc
1	Genetic variation of the renin-angic	Hsu, CCC; Bray, MS; Kao, W	16396964	Not a renal disease foc
1	Genetic-Control Of Blood-Pressure	Kim, Hs; Krege, Jh; Kluckma	7708716	Not a renal disease foc
1	Genomic association analysis identi	Turner ST, Bailey KR, Schwa	22566498	Not a renal disease foc
1	Genotype-phenotype analysis of an	Watkins, WS; Hunt, SC; Will	19770777	Not a renal disease foc
1	Genotypic interactions of renin-ang	Mehri, S; Koubaa, N; Hamm	20580725	Not a renal disease foc
1	Haplotype-based case-control study	Kobayashi, Y; Nakayama, T;	16097366	Not a renal disease foc
1	High aldosterone-to-renin variants	Escher G, Cristiano M, Caus	19151144	Not a renal disease foc
1	Higher thrombin activatable fibrino	Bavbek N, Ceri M, Akdeniz I	24580410	Not a renal disease foc
1	Identification of diuretic non-respo	Yang, YY; Lin, HC; Lin, MW;	21692745	Not a renal disease foc
1	Impact of maternal angiotensinoge	Pfab T, Stirnberg B, Sohn A,	17563539	Not a renal disease foc
1	Incidence of renal failure and nephr	Temme J, Peters F, Lange K,	22237748	Not a renal disease foc
1	Inefficient arterial hypertension cor	Morales-Suarez-Varela, MM	21471972	Not a renal disease foc
1	Inhibition of tissue angiotensin con	Montgomery HE, Kiernan LA	9797175	Not a renal disease foc
1	Insertion-deletion polymorphism in	Bayoumi, RA; Simsek, M; Ya	16900885	Not a renal disease foc
1	Is angiotensin-converting enzyme in	Mao, YQ; Xu, X; Wang, X; Zh	26760503	Not a renal disease foc
1	Is the influence of variation in the A	Muthumala, A; Gable, DR; P	17624939	Not a renal disease foc
1	Juxtaglomerular cell tumor: A morp	Kuroda, N; Maris, S; Monzo	22939575	Not a renal disease foc
1	Liver pyruvate kinase polymorphism	Wang H, Chu W, Das SK, Re	12196482	Not a renal disease foc
1	Long-term follow-up of patients wit	Puricelli E, Bettinelli A, Bors	20219833	Not a renal disease foc
1	Low-salt diet and diuretic effect on	Manunta, P; Bianchi, G	14684671	Not a renal disease foc
1	Lys(173)Arg and -344T/C variants o	Komiya I, Yamada T, Takara	10720581	Not a renal disease foc
1	Malignant Nephrosclerosis in a Pati	Yamanouchi, M; Ubara, Y; I	26466703	Not a renal disease foc
1	Mechanisms of suppression of rena	Shimamoto K, Masuda A, A	2676859	Not a renal disease foc
1	Melatonin prevents maternal fructo	Tain, YL; Leu, S; Wu, KLH; Le	24867192	Not a renal disease foc
1	Middle cerebral artery stenosis in t	Thomas, GN; Lin, JW; Lam,	12865608	Not a renal disease foc
1	miR149 rs71428439 polymorphism	Wang Z, Wei M, Ren Y, Liu I	25213695	Not a renal disease foc
1	Molecular genetics of the renin-ang	Corvol, P; Soubrier, F; Jeune	9296068	Not a renal disease foc
1	Molecular variants of the sodium/h	Zhu, HD; Sagnella, GA; Don	15201541	Not a renal disease foc
1	Molecular-Biology Of Hypertension	Krieger, Je; Dzau, Vj	NA	Not a renal disease foc

1	Natriuretic peptides buffer renin-de	Demerath, T; Staffel, J; Schr	24717731	Not a renal disease foc
1	No Evidence for the Expression of F	Liktor, B; Csomor, P; Szasz,	23370555	Not a renal disease foc
1	Nonmodulation and essential hyper	Hollenberg, NK; Williams, G	16672145	Not a renal disease foc
1	Novel SLC12A3 mutations in Chines	Shao L, Ren H, Wang W, Zha	18287808	Not a renal disease foc
1	Oxidative stress-related factors in E	CalÃ² LA, Pagnin E, Davis PA	12897089	Not a renal disease foc
1	Papillary renal cell carcinoma: a clir	Liu K, Ren Y, Pang L, Qi Y, Jia	26339402	Not a renal disease foc
1	Peripheral vascular disease in type	Thomas, GN; Critchley, JAJH	14632699	Not a renal disease foc
1	Pharmacogenetic association of the	Arnett, DK; Davis, BR; Ford,	15967849	Not a renal disease foc
1	Phenotype-genotype interactions o	Song, XY; Lee, SY; Ma, RCW	19479237	Not a renal disease foc
1	Polymorphism in angiotensin II rece	Baudin, B	15640279	Not a renal disease foc
1	Polymorphism of the aldosterone s	Ko, GJ; Kang, YS; Lee, MH; S	18771471	Not a renal disease foc
1	Polymorphisms in genes of the reni	Deckers, IA; van den Brandt	24978482	Not a renal disease foc
1	Polymorphisms of the renin-angiote	Bofinger, A; Hawley, C; Fish	11317203	Not a renal disease foc
1	Possible role for nephron-derived a	Ramkumar, N; Stuart, D; Ca	NA	Not a renal disease foc
1	Prevalence of angiotensin convertir	Shanmuganathan R, Kumar	26440392	Not a renal disease foc
1	Randomised controlled trial of dual	Mogensen, CE; Neldam, S; T	11110735	Not a renal disease foc
1	Recurrence of the R947X mutation	Fernandes-Rosa FL, de Cast	16757525	Not a renal disease foc
1	Renal ACE immunohistochemical lo	Mizuiri, S; Yoshikawa, H; Ta	9469501	Not a renal disease foc
1	Renal genetic mechanisms of essen	Manunta, P; Cerutti, R; Berr	9377722	Not a renal disease foc
1	Renal haemodynamics are not relat	Skov, K; Madsen, JK; Hanse	17083073	Not a renal disease foc
1	Renal Mechanisms of Association b	Tomaszewski, M; Eales, J; D	25918036	Not a renal disease foc
1	Renal redox-sensitive signaling, but	Yogi, A; Mercure, C; Touyz,	18195161	Not a renal disease foc
1	Renin in blood vessels in human pu	Taylor GM, Cook HT, Sheffie	2450464	Not a renal disease foc
1	Renin Production In Congenital Me	TSUCHIDA, Y; SHIMIZU, K; H	8385325	Not a renal disease foc
1	Renin-angiotensin system gene pol	BuraczyÅ„ska M, Pijanowsk	14502296	Not a renal disease foc
1	Renin-Angiotensin System Gene Va	Joyce-Tan, SM; Zain, SM; Sa	NA	Not a renal disease foc
1	Renin-Angiotensin System Polymor	Forman, JP; Fisher, NDL; Po	18550936	Not a renal disease foc
1	Risk given by AGT polymorphisms ir	Li, Q; Sun, LJ; Du, J; Ran, PZ,	26391364	Not a renal disease foc
1	Role of ACE inhibitors in patients w	Cordonnier, DJ; Zaoui, P; Ha	11708761	Not a renal disease foc
1	Role of GRK4 in the Regulation of A	Chen, K; Fu, CJ; Chen, CY; Li	24218433	Not a renal disease foc
1	Serum paraoxonase-1 gene polymo	Atar A, Gedikbasi A, Sonme	26795139	Not a renal disease foc
1	Single Strand Conformation Polymo	Bettinaglio, P; Galbusera, A	12270765	Not a renal disease foc
1	Synergistic effect of alpha-adducin	Barlassina, C; Schork, NJ; M	10720960	Not a renal disease foc
1	Systemic nitric oxide clamping in nc	Simonsen, JA; Rasmussen, I	19785629	Not a renal disease foc
1	TGF-beta1 gene polymorphisms anc	EbinÃ§ FA, Derici U, GÃ¶ne	18197538	Not a renal disease foc
1	The alpha-adducin Gly460Trp polyn	Pescatello LS, Blanchard BE	17472579	Not a renal disease foc
1	The association between vitamin D	Maia, J; da Silva, AS; do Car	27536155	Not a renal disease foc
1	The association of the R563Q genot	Jones ES, Owen EP, Rayner	22895453	Not a renal disease foc
1	The brain and salt-sensitive hyperte	Leenen, FHH; Ruzicka, M; H	11884268	Not a renal disease foc
1	The cardiovascular system in famili	Jakobsen, NFB; Laugesen, E	NA	Not a renal disease foc
1	The Connexin40 A96S Mutation Cau	Lubkemeier, I; Machura, K;	21597036	Not a renal disease foc
1	The interaction of AGT and NOS3 ge	Gatti, RR; Santos, PS; Sena,	22791701	Not a renal disease foc
1	The M235T polymorphism in thean	van den Born, BJH; van Mon	17921816	Not a renal disease foc
1	The presence of PAI-1 4G/5G and A	GÃ¼ngÃ¶r Y, KayataÅŸ M, Y	21332339	Not a renal disease foc
1	The state and responsiveness of the	Price, DA; De'Oliveira, JMF;	10232494	Not a renal disease foc
1	Transforming growth factor-beta(1)	Suthanthiran, M; Li, BG; Son	10725360	Not a renal disease foc
1	Trientine and renin-angiotensin sys	Moya-Olano L, Milne HM, F	22029676	Not a renal disease foc

For peer review only

1	Unclassified renal cell carcinoma: a	Hu ZY, Pang LJ, Qi Y, Kang X	25120763	Not a renal disease foc
1	Urinary UMOD excretion and chron	Wu CH, Lee CT, Lee CH, Che	21332338	Not a renal disease foc
1	Vitamin D Deficiency in the Pathoge	Rostand, SG	24929953	Not a renal disease foc
1	Vitamin D receptor gene polymorpl	Cottone S, Guarino L, Arsen	25500899	Not a renal disease foc
1	Wilms' tumor protein (-KTS) modul	Steege, A; Fahling, M; Palie	18496514	Not a renal disease foc
1	[A novel COL4A5 splicing mutation	Tang Z, Dai Y, Wan Z, Zhan	19065523	Not English or Spanish
1	[Angiotensin-converting enyme ins	Krajina-AndriceviÄ‡ M, Ziba	23120809	Not English or Spanish
1	[Angiotensin-converting enzyme ge	Guan T, Liu Z, Chen Z.	10436947	Not English or Spanish
1	[Arterial hypertension and chronic	Ermolenko VM, Lifshits NL,	7700	Not English or Spanish
1	[Association of single nucleotide pc	Wang ZH, Chen N, Pan XX,	16796905	Not English or Spanish
1	[Association of the renin-angiotens	BuraczyÅ„ska M, Ksiazek P,	12476891	Not English or Spanish
1	[Correlation between HLA-DQA1 all	Ren S, Tong L, Xilin G, Sui J,	11836690	Not English or Spanish
1	[Correlation of cyclin D1 overexpres	Ren YY, Du LJ, Feng ZQ, Len	16480581	Not English or Spanish
1	[Gene mutation analysis of X-linked	Song Y, Ma HW, Li F, Hu M,	24229582	Not English or Spanish
1	[Genetic predisposition to systemic	Bzoma B, Debska-SlizieÅ„ A	19112833	Not English or Spanish
1	[Is PstI polymorphism of the angiot	Grzeszczak W, Zychma M, Z	9499204	Not English or Spanish
1	[Morphofunctional characteristics	Sokolova RI, Bobusheva GS,	2678677	Not English or Spanish
1	[Mutations in NPHS2 in familial ste	Fu R, Chen XM, Wang QH,	19099831	Not English or Spanish
1	[Relationship between I/D polymor	Moleda P, Majkowska L, Sa	17941464	Not English or Spanish
1	[Renin-angiotensin system genes in	BuraczyÅ„ska M, JÃ³Åºwiak	11865575	Not English or Spanish
1	[Study on the association between	Yang HY, Tai WL, Yuan HY,	15192842	Not English or Spanish
1	Genomics of type 1 diabetes mellit	Nosikov, VV	NA	Not English or Spanish
1	A rare case of juvenile hypertensior	Paragliola, RM; Capoluongc	26084817	Paediatric individuals
1	ACE and AT1 receptor gene polymc	Kostic, M; Stankovic, A; Zivk	15179569	Paediatric individuals
1	ACE gene insertion/deletion polym	Serdaroglu, E; Mir, S; Berde	16208534	Paediatric individuals
1	ACE gene polymorphism and renal	Haszon, I; Friedman, AL; Pa	12478352	Paediatric individuals
1	ACE gene polymorphism in Egyptiar	Fahmy, ME; Fattouh, AM; H	18792483	Paediatric individuals
1	ACE gene polymorphism in Turkish	Celik US, Noyan A, Bayazit	16825089	Paediatric individuals
1	ACE I/D gene polymorphism in prim	Oktem, F; Sirin, A; Bilge, I; E	14986085	Paediatric individuals
1	An insertion/deletion ACE polymor	Kaczmarczyk, M; Loniewska	22674971	Paediatric individuals
1	Angiotensin converting enzyme ger	Bajpai, M; Pratap, A; Somit	14713838	Paediatric individuals
1	Angiotensin-converting enzyme ger	Al-Eisa, A; Haider, MZ; Sriva	10986863	Paediatric individuals
1	Angiotensinogen gene T235 variant	Gallego, PH; Shephard, N; E	18413222	Paediatric individuals
1	Association of angiotensin converti	Narasimhan, KL; Madhu, K;	NA	Paediatric individuals
1	Association of angiotensin type 2 re	Miranda, DM; Dos Santos,	24995698	Paediatric individuals
1	Autosomal dominant pseudohypoa	Geller, DS; Zhang, JH; Zenn	16611713	Paediatric individuals
1	DD Genotype of ACE Gene in Boys:	Alasehirli, B; Balat, A; Buyul	22017506	Paediatric individuals
1	Donor and recipient ACE I/D genot	Buscher, R; Nagel, D; Finkel	21309964	Paediatric individuals
1	Earlier Onset of Complications in Yc	Dart, AB; Martens, PJ; Rigat	24130346	Paediatric individuals
1	Effect of angiotensin-converting en	Saber-Ayad M, Sabry S, Abc	20418353	Paediatric individuals
1	Effects of genetic polymorphisms o	Tabel, Y; Berdeli, A; Mir, S;	16525944	Paediatric individuals
1	Genetic polymorphism of the renin	Yim, HE; Jung, MI; Choi, BM	14764974	Paediatric individuals
1	Genetic polymorphisms of the renii	Frishberg, Y; Becker-Cohen,	9853248	Paediatric individuals
1	Genetic risk factors in typical haem	Taranta, A; Gianviti, A; Palm	19110485	Paediatric individuals
1	Heterozygous Loss-of-Function SEC	Bolar, NA; Golzio, C; Zivna,	27392076	Paediatric individuals
1	Impact of ACE I/D gene polymorphi	Hohenfellner, K; Wingen, A	11354781	Paediatric individuals
1	Impact of common functional polyr	Hussein, A; Elderwy, AA; As	25939993	Paediatric individuals

1	Implication of genetic variations in	Hahn, H; Ku, SE; Kim, KS; Pa	16133060	Paediatric individuals
1	Implications of certain genetic poly	Ozen S, Alikasifoglu M, Saat	10401028	Paediatric individuals
1	Increased HLA- A*11 in Chinese chi	Cheung W, Ren EC, Chan SH	11956863	Paediatric individuals
1	Low renin-angiotensin system activ	Peruzzi, L; Lombardo, F; Am	16006956	Paediatric individuals
1	Mapping candidate regions and ger	Weber, S; Landwehr, C; Ren	20605837	Paediatric individuals
1	MCP1 2518 A/G polymorphism affe	Besbas N, Kalyoncu M, Cil C	26335292	Paediatric individuals
1	Nitric oxide synthase gene polymor	Balat A, Alasehirli B, Oguzka	17365914	Paediatric individuals
1	NPHS2 variation in Chinese souther	Dai Y, Yang H, Gao P, Liu WI	25112471	Paediatric individuals
1	Polymorphisms of renin-angiotensi	Maruyama, K; Yoshida, M; I	11354780	Paediatric individuals
1	Posterior urethral valves: Prelimina	Bajpai, M; Pratap, A; Tripatl	15643266	Paediatric individuals
1	Prioritization and burden analysis o	Nicolaou, N; Pulit, SL; Nijma	26489027	Paediatric individuals
1	Renal tubular dysgenesis, a not unc	Lacoste M, Cai Y, Guicharna	16790508	Paediatric individuals
1	Renin-angiotensin gene polymorph	Papp, F; Friedman, AL; Bere	12579405	Paediatric individuals
1	Renin-angiotensin system gene pol	Liu, DS; Lu, F; Zhai, SH; Wei,	NA	Paediatric individuals
1	Renin-angiotensin system gene pol	Ozkaya, O; Soylemezoglu, C	16521052	Paediatric individuals
1	Renin-angiotensin system polymor	Pardo, R; Malaga, S; Coto, E	12579398	Paediatric individuals
1	Renin-angiotensin system polymor	Liu, KP; Lin, CY; Chen, HJ; W	15045574	Paediatric individuals
1	Risk factors for loss of residual rena	Ha, IS; Yap, HK; Munarriz, R	25874598	Paediatric individuals
1	The juxtaglomerular apparatus in B	Taugner R, Waldherr R, Sey	3128915	Paediatric individuals
1	The role of vitamin D receptor gene	Goknar N, Ã–ktem F, Torun	26908058	Paediatric individuals
1	Add-on angiotensin receptor blocka	Agarwal, R	11380832	Pharmaceutical drug fc
1	Antiproteinuric effect of candesarta	Haneda, M; Kikkawa, R; Sak	15364166	Pharmaceutical drug fc
1	Application of Direct Renin Inhibitic	Mende, CW	20490905	Pharmaceutical drug fc
1	Captopril enhances transforming gr	Di Paolo S, Schena A, Stallo	12499886	Pharmaceutical drug fc
1	DPP-4 Inhibition on Top of Angioter	Alter, ML; Ott, IM; von Web	23171828	Pharmaceutical drug fc
1	Effects of losartan and enalapril on	Argani, H; Ghorbanihaghjo,	18261537	Pharmaceutical drug fc
1	Enalapril and losartan affect lipid pe	Rashtchizadeh, N; Aghaeish	17222813	Pharmaceutical drug fc
1	Losartan decreases plasma levels o	Campistol, JM; Inigo, P; Jim	10432413	Pharmaceutical drug fc
1	Pentoxifylline for Renoprotection ir	Navarro-Gonzalez, JF; Murc	21144773	Pharmaceutical drug fc
1	Renal implications of angiotensin re	Hollenberg, NK	11459212	Pharmaceutical drug fc
1	Renin-angiotensin system polymor	Noroozianavval, M; Argani,	17524880	Pharmaceutical drug fc
1	The influence of renin-angiotensin	Goyache-Goni, B; Aranda-La	24241364	Pharmaceutical drug fc
1	The influence of the ACE (I/D) polyr	Luik, PT; Hoogenberg, K; Ke	12856080	Pharmaceutical drug fc
1	Thiazolidinediones and the renal ar	Goenka, N; Kotonya, C; Pen	18230694	Pharmaceutical drug fc
2	Relationship between the renin-angi	Wu S, Xiang K, Zheng T, Su	11776100	No data
3	Chronic renal insufficiency among A	Prasad P et al.	16672053	< 3 populations reporte
3	M235T polymorphism in the AGT ge	Sarkar S, Gupta V, Kumar A	25660845	< 3 populations reporte
3	Plasma renin and prorenin and reni	Deinum J et al.	10462269	< 3 populations reporte
3	Renin gene rs1464816 polymorphis	Ramanathan G, Elumalai R	26753721	< 3 populations reporte
3	Renin–angiotensin–aldosterone sys	Mtiraoui N et al.	21421655	< 3 populations reporte

For peer review only

l study
l study
l study

For peer review only

For peer review only

For peer review only

For peer review only

dy
dy
dy
dy
dy
dy
dy
dy
dy
dy
dy
dy
dy
dy
dy
dy
dy
dy
dy
dy
dy
dy
dy
dy
dy
dy
dy
dy
dy
dy
dy
dy
dy
dy
dy
dy
dy

For peer review only

dy
dy
dy
dy
dy
dy
dy
dy
dy
dy
dy
dy
dy
dy
dy
dy
dy
dy
dy
dy
dy
dy
dy
dy
dy
dy
dy
us
us
us
us
us
us
us
us
us
us
us
us
us
us
us
us
us
us
us
us

For peer review only

us
us
us
us
us
us
us
us
us
us
us
us
us
us
us
us
us
us
us
us
us
us
us
us
us
us
us
us
us
us
us
us
us
us
us
us
us
us
us
us
us
us
us
us
us
us
us

For peer review only

us
us
us
us
us
us
us
us
us
us
us
us
us
us
us
us
us
us
us
us
us
us
us
us
us
us
us
us
us
us
us
us
us
us
us
us
us
us
us
us
us
us
us
us
us
us
us

For peer review only

us
us
us
us
us
us
us
us
us
us
us
us
us
us
us
us
us
us
us
us
us
us
us
us
us
us
us
us
us
us
us
us
us
us
us
us
us
us
us
us
us
us
us
us
us
us
us

For peer review only

us
us
us
us
us

For peer review only

ocus
ocus
ocus
ocus
ocus
ocus
ocus
ocus
ocus
ocus
ocus
ocus
ocus
ocus

ed per SNP
ed per SNP
ed per SNP
ed per SNP
ed per SNP

For peer review only

PRISMA 2009 Flow Diagram

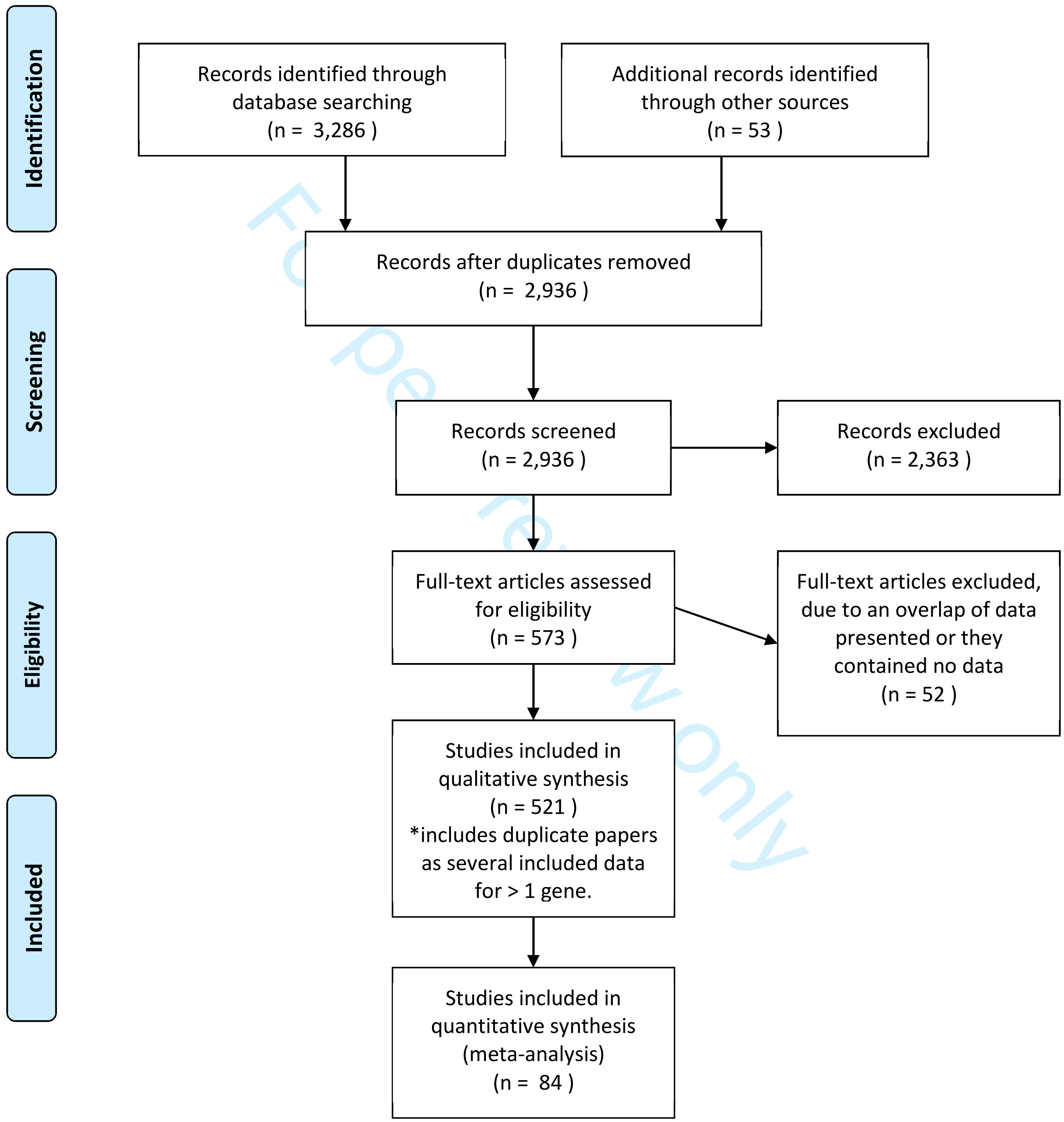

From: Moher D, Liberati A, Tetzlaff J, Altman DG, The PRISMA Group (2009). *P*referred *R*eporting *I*tems for *S*ystematic Reviews and *M*eta-*A*nalyses: The PRISMA Statement. PLoS Med 6(7): e1000097. doi:10.1371/journal.pmed1000097

For more information, visit www.prisma-statement.org.

For peer review only - http://bmjopen.bmj.com/site/about/guidelines.xhtml

PRISMA 2009 Checklist

Section/topic	#	Checklist item	Reported on page #
**TITLE**			
Title	1	Identify the report as a systematic review, meta-analysis, or both.	1
**ABSTRACT**			
Structured summary	2	Provide a structured summary including, as applicable: background; objectives; data sources; study eligibility criteria, participants, and interventions; study appraisal and synthesis methods; results; limitations; conclusions and implications of key findings; systematic review registration number.	1&2
**INTRODUCTION**			
Rationale	3	Describe the rationale for the review in the context of what is already known.	2&3
Objectives	4	Provide an explicit statement of questions being addressed with reference to participants, interventions, comparisons, outcomes, and study design (PICOS).	1&3
**METHODS**			
Protocol and registration	5	Indicate if a review protocol exists, if and where it can be accessed (e.g., Web address), and, if available, provide registration information including registration number.	5
Eligibility criteria	6	Specify study characteristics (e.g., PICOS, length of follow-up) and report characteristics (e.g., years considered, language, publication status) used as criteria for eligibility, giving rationale.	4
Information sources	7	Describe all information sources (e.g., databases with dates of coverage, contact with study authors to identify additional studies) in the search and date last searched.	3-4
Search	8	Present full electronic search strategy for at least one database, including any limits used, such that it could be repeated.	3
Study selection	9	State the process for selecting studies (i.e., screening, eligibility, included in systematic review, and, if applicable, included in the meta-analysis).	3
Data collection process	10	Describe method of data extraction from reports (e.g., piloted forms, independently, in duplicate) and any processes for obtaining and confirming data from investigators.	4
Data items	11	List and define all variables for which data were sought (e.g., PICOS, funding sources) and any assumptions and simplifications made.	4
Risk of bias in individual studies	12	Describe methods used for assessing risk of bias of individual studies (including specification of whether this was done at the study or outcome level), and how this information is to be used in any data synthesis.	4
Summary measures	13	State the principal summary measures (e.g., risk ratio, difference in means).	4
Synthesis of results	14	Describe the methods of handling data and combining results of studies, if done, including measures of consistency (e.g., I^2) for each meta-analysis.	4

For peer review only

For peer review only - http://bmjopen.bmj.com/site/about/guidelines.xhtml

PRISMA 2009 Checklist

Section/topic	#	Checklist item	Reported on page #
Risk of bias across studies	15	Specify any assessment of risk of bias that may affect the cumulative evidence (e.g., publication bias, selective reporting within studies).	4
Additional analyses	16	Describe methods of additional analyses (e.g., sensitivity or subgroup analyses, meta-regression), if done, indicating which were pre-specified.	5
**RESULTS**			
Study selection	17	Give numbers of studies screened, assessed for eligibility, and included in the review, with reasons for exclusions at each stage, ideally with a flow diagram.	5
Study characteristics	18	For each study, present characteristics for which data were extracted (e.g., study size, PICOS, follow-up period) and provide the citations.	4&5
Risk of bias within studies	19	Present data on risk of bias of each study and, if available, any outcome level assessment (see item 12).	4-6
Results of individual studies	20	For all outcomes considered (benefits or harms), present, for each study: (a) simple summary data for each intervention group (b) effect estimates and confidence intervals, ideally with a forest plot.	5&6
Synthesis of results	21	Present results of each meta-analysis done, including confidence intervals and measures of consistency.	5&6
Risk of bias across studies	22	Present results of any assessment of risk of bias across studies (see Item 15).	5&6
Additional analysis	23	Give results of additional analyses, if done (e.g., sensitivity or subgroup analyses, meta-regression [see Item 16]).	NA
**DISCUSSION**			
Summary of evidence	24	Summarize the main findings including the strength of evidence for each main outcome; consider their relevance to key groups (e.g., healthcare providers, users, and policy makers).	7-9
Limitations	25	Discuss limitations at study and outcome level (e.g., risk of bias), and at review-level (e.g., incomplete retrieval of identified research, reporting bias).	8-9
Conclusions	26	Provide a general interpretation of the results in the context of other evidence, and implications for future research.	7-9
**FUNDING**			
Funding	27	Describe sources of funding for the systematic review and other support (e.g., supply of data); role of funders for the systematic review.	9

From: Moher D, Liberati A, Tetzlaff J, Altman DG, The PRISMA Group (2009). Preferred Reporting Items for Systematic Reviews and Meta-Analyses: The PRISMA Statement. PLoS Med 6(7): e1000097. doi:10.1371/journal.pmed1000097

For more information, visit: **www.prisma-statement.org**.

For peer review only - http://bmjopen.bmj.com/site/about/guidelines.xhtml

Reporting checklist for meta-analysis of observational studies.

Based on the MOOSE guidelines.

Instructions to authors

Complete this checklist by entering the page numbers from your manuscript where readers will find each of the items listed below.

Your article may not currently address all the items on the checklist. Please modify your text to include the missing information. If you are certain that an item does not apply, please write "n/a" and provide a short explanation.

Upload your completed checklist as an extra file when you submit to a journal.

In your methods section, say that you used the MOOSE reporting guidelines, and cite them as:

Stroup DF, Berlin JA, Morton SC, Olkin I, Williamson GD, Rennie D, Moher D, Becker BJ, Sipe TA, Thacker SB. Meta-analysis of observational studies in epidemiology: a proposal for reporting. Meta-analysis Of Observational Studies in Epidemiology (MOOSE) group. JAMA. 2000; 283(15):2008-2012.

	Reporting Item	Page Number
#1	Identify the study as a meta-analysis of observational research	1
#2	Provide a structured summary including, as applicable: background; objectives; data sources; study eligibility criteria, participants, and interventions; study appraisal and synthesis methods; results; limitations; conclusions and implications of key findings; systematic review registration number (From PRISMA checklist)	1&2
#3a	Problem definition	3
#3b	Hypothesis statement	2
#3c	Description of study outcomes	3
#3d	Type of exposure or intervention used	3
#3e	Type of study designs used	3

For peer review only

	#3f	Study population	3
Search strategy	#4a	Qualifications of searchers (eg, librarians and investigators)	3-4
	#4b	Search strategy, including time period included in the synthesis and keywords	4
	#4c	Effort to include all available studies, including contact with authors	4
	#4d	Databases and registries searched	3
	#4e	Search software used, name and version, including special features used (eg, explosion)	4
	#4f	Use of hand searching (eg, reference lists of obtained articles)	3
	#4g	List of citations located and those excluded, including justification	3
	#4h	Method of addressing articles published in languages other than English	4
	#4i	Method of handling abstracts and unpublished studies	4
	#4j	Description of any contact with authors	NA
	#5a	Description of relevance or appropriateness of studies gathered for assessing the hypothesis to be tested	3&4
	#5b	Rationale for the selection and coding of data (eg, sound clinical principles or convenience)	3&4
	#5c	Documentation of how data were classified and coded (eg, multiple raters, blinding, and interrater reliability)	3&4
	#5d	Assessment of confounding (eg, comparability of cases and controls in studies where appropriate)	4
	#5e	Assessment of study quality, including blinding of quality assessors; stratification or regression on possible predictors of study results	4
	#5f	Assessment of heterogeneity	4
	#5g	Description of statistical methods (eg, complete description of	4-5

For peer review only

	fixed or random effects models, justification of whether the chosen models account for predictors of study results, dose-response models, or cumulative meta-analysis) in sufficient detail to be replicated	
#5h	Provision of appropriate tables and graphics	4-6 & additional materials
#6a	Graphic summarizing individual study estimates and overall estimate	Figures & Supplementary Figures
#6b	Table giving descriptive information for each study included	ST4-ST6
#6c	Results of sensitivity testing (eg, subgroup analysis)	5
#6d	Indication of statistical uncertainty of findings	4-6
#7a	Quantitative assessment of bias (eg. publication bias)	4-6
#7b	Justification for exclusion (eg, exclusion of non–English-language citations)	3
#7c	Assessment of quality of included studies	3-6
#8a	Consideration of alternative explanations for observed results	7-8
#8b	Generalization of the conclusions (ie, appropriate for the data presented and within the domain of the literature review)	8-9
#8c	Guidelines for future research	8-9
#8d	Disclosure of funding source	9

Reproduced with permission from JAMA. 2000. 283(15):2008-2012. Copyright © 2000 American Medical Association. All rights reserved.This checklist can be completed online using https://www.goodreports.org/, a tool made by the EQUATOR Network in collaboration with Penelope.ai

For peer review only

BMJ Open

Genetic associations between genes in the renin-angiotensin-aldosterone system and renal disease: a systematic review and meta-analysis

Journal:	*BMJ Open*
Manuscript ID	bmjopen-2018-026777.R1
Article Type:	Research
Date Submitted by the Author:	28-Jan-2019
Complete List of Authors:	Smyth, Laura; Queen's University Belfast Centre for Public Health, Epidemiology and Public Health Research Group<br>Cañadas-Garre, Marisa; Queen's University Belfast Centre for Public Health, Epidemiology and Public Health Research Group<br>Cappa, Ruaidhri; Queen's University Belfast Centre for Public Health, Epidemiology and Public Health Research Group<br>Maxwell, Alexander; Queen's University Belfast Centre for Public Health, Centre for Public Health; Belfast City Hospital, Regional Nephrology Unit<br>McKnight, Amy; Queen's University Belfast, Centre for Public Health
**Primary Subject Heading**:	Genetics and genomics
Secondary Subject Heading:	Renal medicine, Public health
Keywords:	CKD, NEPHROLOGY, RAAS, Meta-analysis, Renin-angiotensin-aldosterone system, Kidney

SCHOLARONE™
Manuscripts

Genetic associations between genes in the renin-angiotensin-aldosterone system and renal disease: a systematic review and meta-analysis

Laura J. Smyth*, Marisa Cañadas-Garre, Ruaidhri C. Cappa, Alexander P. Maxwell, Amy Jayne McKnight

Epidemiology and Public Health Research Group, Centre for Public Health, Queen's University Belfast, Belfast, United Kingdom

*Corresponding author email: laura.smyth@qub.ac.uk

SHORT TITLE

A meta-analysis of RAAS gene variants and CKD

WORD COUNT (excluding Abstract, Keywords, References, Figures, Tables, Statements and Contributions)

3,599

ABSTRACT

Background

Chronic kidney disease (CKD) is defined by abnormalities in kidney structure and/or function present for more than three months. Worldwide, both the incidence and prevalence rates of CKD are increasing. The renin-angiotensin-aldosterone system (RAAS) regulates fluid and electrolyte balance through the kidney. RAAS activation is associated with hypertension, which is directly implicated in causation and progression of CKD. RAAS blockade, using drugs targeting individual RAAS mediators and receptors, has proven to be reno-protective.

Objectives

To assess genomic variants present within RAAS genes; *ACE*, *ACE2*, *AGT*, *AGTR1*, *AGTR2* and *REN*, for association with CKD.

Design and Data Sources

A systematic review and meta-analysis of observational research was performed to evaluate the RAAS gene polymorphisms in CKD using both PubMed and Web of Science databases with publication date between the inception of each database and 31/12/2018. Eligible articles included case-control studies of a defined kidney disease and included genotype counts.

Eligibility Criteria

Any paper was removed from the analysis if it was not written in English or Spanish, was a non-human study, was a paediatric study, was not a case-control study, did not have a renal disease phenotype, did not include data for the genes, was a gene expression based study or had a pharmaceutical drug focus.

For peer review only

Results

A total of 3,531 studies were identified, 114 of which met the inclusion criteria. Genetic variants reported in at least three independent publications for populations with the same ethnicity were determined and quantitative analyses performed.

Three variants returned significant results in populations with different ethnicities at $p<0.05$; *ACE* insertion, *AGT* rs699-T allele, and *AGTR1* rs5186-A allele; each variant was associated with a reduced risk of CKD development.

Conclusions

Further biological pathway and functional analyses of the RAAS gene polymorphisms will help define how variation in components of the RAAS pathway contribute to CKD.

Keywords

CKD, kidney, meta-analysis, RAAS, renin-angiotensin-aldosterone system

ARTICLE SUMMARY

Strengths and limitations of the study

- Strength: Individuals with microalbuminuria were excluded from both the case and control definitions to improve the discrimination between individuals with and without renal disease as microalbuminuria may regress, remain stable or progress to macroalbuminuria.
- Strength: Due to previously reported heterogeneity between different ethnic groups, we included this as a risk factor and performed each analysis per ethnicity.
- Limitation: Some studies in our search could not be included in quantitative analysis as they lacked information relating to genotype counts and had an unclear measure and definition of albuminuria for both cases and controls.

Hypothesis statement

- We hypothesise that there will be an association between genetic variants within RAAS genes and CKD.

INTRODUCTION

Chronic kidney disease (CKD) is defined as a progressive loss of renal function measured as a reduction in glomerular filtration rate (GFR) to <60 mL/min/1.73m^2, or the presence of persistent urinary abnormalities including albuminuria and/or structural alterations which have been present for at least three months(1). CKD is an increasing public health issue given its associated morbidities, premature mortality and management of advanced CKD is a significant burden on health care budgets worldwide(2,3). There is substantial evidence that inherited genetic variants(4), the presence of diabetes(5) or hypertension(6) and an individual's ethnicity(7,8) directly influence the development of various CKD phenotypes.

The renin-angiotensin-aldosterone system (RAAS) is a homeostatic endocrine system which is of critical importance to the regulation of blood pressure and maintenance of fluid and electrolyte balance(9,10). Renin, secreted from the juxtaglomerular apparatus in response to reduced renal

For peer review only - http://bmjopen.bmj.com/site/about/guidelines.xhtml

perfusion pressure, catalyses the conversion of angiotensinogen to angiotensin I(11). Subsequently, angiotensin I converting enzyme (*ACE*) cleaves angiotensin I to generate angiotensin II, which regulates heart and kidney function by binding to and activating angiotensin II receptors (type I and type II)(11,12). The angiotensin II type I receptors are responsible for multiple biologic actions of RAAS including vasoconstriction and sodium reabsorption(11–14).

Increased RAAS activation is linked to progression of CKD of different aetiologies, especially diabetic nephropathy (DN)(11,15–18), and is mediated by hypertensive injury(17) and accelerated renal fibrosis(19). The physiological relevance of this pathway in the progression of CKD has focused attention on RAAS components including *ACE*, angiotensin I converting enzyme 2 (*ACE2*), angiotensinogen (*AGT*), angiotensin II receptor type 1 (*AGTR1*), angiotensin II receptor type 2 (*AGTR2*) and renin (*REN*), as candidate genes for various CKD-related phenotypes. Multiple studies have implicated RAAS gene variants in the progression of CKD(20–26).

This manuscript describes a systematic review and meta-analysis to examine published data reporting genetic variants present within six of the RAAS candidate genes: *ACE*, *ACE2*, *AGT*, *AGTR1*, *AGTR2* and *REN*, for a range of CKD phenotypes and ethnicities, to help define their putative roles as risk factors for CKD.

METHODS

Search Strategy: A systematic search was undertaken following recognised methods, the Meta-analysis of Observational Studies in Epidemiology (MOOSE) guidelines(27), by two investigators. PubMed and Web of Science online databases were searched for studies published between the inception of each database and 31st December 2018. All search terms are detailed in Supplementary Table S1.

The general strategy of the searches was to follow the structure; (all alternative versions of each gene name, separated by the boolean operator OR) AND (kidney OR nephrology OR nephropathy OR renal) AND (SNP OR polymorphism OR variant OR allele OR genotype). For each search term, where an appropriate MeSH term was available, the query included the quoted search term OR the MeSH term. Additional filters including English OR Spanish languages, human studies, case control studies, not clinical trials, not review articles, not a case report, not a meta-analysis were applied.

Reference lists from included publications were also manually searched. Two authors (LJS and MCG) independently conducted the literature search, screened the articles and extracted the data. In the case of any disagreement, a third author (RCC) considered the articles. A range of CKD phenotypes were included in this analysis, the case and control definitions are included in Table 1.

Table 1: Phenotypic comparisons included in this analysis

Case Group	Control Group
Autosomal dominant polycystic kidney disease	Healthy controls
Atherosclerotic renal artery stenosis	Healthy controls
Balkan endemic nephropathy	Healthy controls
Chronic glomerulonephritis	Healthy controls
Chronic kidney disease	Healthy controls
Diabetic nephropathy*	Diabetes mellitus
Diabetic nephropathy*	Healthy controls
End-stage renal disease	Healthy controls

For peer review only - http://bmjopen.bmj.com/site/about/guidelines.xhtml

End-stage renal disease	Type 1 diabetes mellitus
Focal segmental glomerulosclerosis	Healthy controls
Glomerulonephritis	Healthy controls
Hypertension-related renal disease	Healthy controls with hypertension
Hypertension-related renal disease	Healthy controls
IgA Nephropathy	Healthy controls
Interstitial nephritis	Healthy controls
Lupus nephritis	Systemic lupus erythematosus
Lupus nephritis	Healthy controls
Minimal change nephrotic syndrome	Healthy controls
Non-Balkan endemic nephropathy	Healthy controls
Nephroangiosclerosis	Healthy controls
Polycystic kidney disease	Healthy controls
Primary membranous glomerulonephritis	Healthy controls
Primary membranous glomerulonephritis	Organ donors
Renal transplant recipients	Healthy controls
Renal transplant recipients	Kidney Donors
Type 1 diabetic nephropathy*	Type 1 diabetes mellitus
Type 1 diabetic nephropathy linked to end-stage renal disease	Healthy controls
Type 1 diabetic nephropathy linked to end-stage renal disease	Type 1 diabetes mellitus
Type 2 diabetic nephropathy*	Type 2 diabetes mellitus
Type 2 diabetic nephropathy linked to end-stage renal disease	Healthy controls
Type 2 diabetic nephropathy linked to end-stage renal disease	Type 2 diabetes mellitus

* In the studies including diabetic nephropathy as cases, only individuals with reported macroalbuminuria or proteinuria were included. Individuals with microalbuminuria were excluded.

Inclusion/Exclusion Criteria: Inclusion criteria were judged against a standardised list of agreed criteria (LJS, MCG and AJM), whereby the English or Spanish language publication described an adult, human case-control study, of a defined kidney disease. In the rare instances when suspected duplicate data was identified within two or more included articles, only the article either published first, or that with the larger number of participants was included.

Articles were excluded if they included paediatric subjects, were a pharmacological based study reporting clinical trials of medications, did not contain genotypic data for the correct gene or were non-human studies.

Data extraction: Where available, the size of each study, case group disease definition and number of individuals, control group definition and number of individuals, ethnicity, genetic variant, genotype in the format of allele one–heterozygote–allele two and allele counts were recorded and calculated in spreadsheets by two authors (LJS and MCG). Articles were re-assessed where any disagreement occurred and a third reviewer was employed. Ethnicities were recorded from the articles and re-coded following the International Genome Sample Resources' online guidance(28). Where any population did not align to a listed population code, a new one was created for the purposes of this study. All ethnicity codes are available in Supplementary Table S2. The data collected was divided into disease phenotype groups to ensure a high level of homogeneity.

Statistical Analysis: Each genetic variant which had been investigated and reported in at least three independent publications for the same ethnicity and phenotype was included in quantitative statistical analyses. Review Manager (RevMan) 5.3 (The Cochrane Collaboration, The Nordic Cochrane Centre, Copenhagen, Denmark) was employed to facilitate the analysis of allele frequencies. For each SNP,

For peer review only - http://bmjopen.bmj.com/site/about/guidelines.xhtml

the total number of alleles were recorded per case and control group. Hardy Weinberg equilibrium was calculated for all included studies, for cases and controls separately. Statistical analyses were performed using the random effects model as heterogeneity was expected. For each SNP, this analysis provided the P value, odds ratio (OR) and 95% confidence intervals (CI). It also facilitated the assessment of the heterogeneity level using the I^2 statistic(29). Forest plots and funnel plots were generated automatically to assess publication bias and the significance value was set at P<0.05 (LJS and RCC).

The genotyping quality of the studies was assessed by reported genotype completion rate and Hardy Weinberg equilibrium. Phenotypes included in this analysis are shown in Table 1; individuals with microalbuminuria were excluded alongside the studies that focused on disease progression. No sensitivity analysis was performed. All study methodology conformed to the MOOSE criteria(27). No published protocol is available for this review. The workflow followed a consistent pattern for each gene. A summary of this is included in Figure 1.

Figure 1: Workflow pattern

Patient and public involvement: Neither patients nor the public were directly involved in the design of this study, which analysed previously published data available in the public domain.

RESULTS

The database searches returned 3,531 results, 144 of which remained following the application of inclusion and exclusion criteria and the removal of any SNP which was not reported on at least three occasions. Several articles included data for more than one gene, signifying that they have emerged multiple times throughout the database searches; the total number of individual articles was therefore 114. The search strategies are included as Supplementary Figures S1a-f. All excluded studies are listed in Supplementary Tables S3a-f

The total number of subjects analysed within these studies (n=114) was 18,231 individuals with renal disease and 21,887 individuals acting as controls. For SNPs in three of the RAAS genes, *ACE2*, *AGTR2* and *REN*, there were less than three independent populations studied. A summary table detailing the main results is included as Table 2.

For peer review only

For peer review only - http://bmjopen.bmj.com/site/about/guidelines.xhtml

171 **Table 2:** Summary of the most significant result for each included gene

172

Gene	Articles returned (n)	Articles analysed (n)	Max. individuals in analysis (n)	Most significant result	P Value	OR (95% CL)	I^2 (%)	Allele	Average Allele Frequency - Controls
ACE	380	95	33,247	I/D (EAS)	0.008	0.80 (0.67, 0.94)	68	Insertion	0.62
ACE2	1556	0	NA		NA	NA	NA	NA	NA
AGT	693	33	13,234	rs699 (EUR)	0.002	0.84 (0.76, 0.94)	0	T	0.57
AGTR1	200	16	6917	rs5186 (SAS)	0.001	0.71 (0.58, 0.87)	37	A	0.81
AGTR2	29	0	NA		NA	NA	NA	NA	NA
REN	673	0	NA		NA	NA	NA	NA	NA

173

174 Abbreviations: EAS, East Asian; EUR, European; NA, Not Applicable; SAS, South Asian.

175

For peer review only

For peer review only - http://bmjopen.bmj.com/site/about/guidelines.xhtml

ACE

A total of 15 quantitative analyses were completed for the insertion / deletion (I/D) polymorphism located within *ACE* in eight phenotypes, details of which are included in Supplementary Table S4. Three quantitative analyses returned a significant result. The first analysis comprised 11 publications, each studying an East Asian population with T2DN (type 2 diabetes and nephropathy) and compared with T2DM (type 2 diabetes mellitus without nephropathy). Figure 2a displays these results, P= 0.009; OR= 0.74; 95% CI= 0.59, 0.93, I^2= 55%, showing that the presence of the insertion variant at this *ACE* locus was significantly associated with this phenotype. The insertion provides a lower risk of developing T2DN in an East Asian population as demonstrated in five studies, which contributed to 51.5% of the weight in this analysis. Figure 2b shows the associated funnel plot for this analysis.

Figure 2a: Forest plot - *ACE* I/D investigation, individuals with type 2 diabetic nephropathy compared to individuals with type 2 diabetes mellitus in an East Asian population (*ACE* insertion compared to deletion).

Figure 2b: Funnel plot - *ACE* I/D investigation, individuals with type 2 diabetes and nephropathy compared to individuals with type 2 diabetes mellitus (without nephropathy) in an East Asian population (*ACE* insertion compared to deletion).

The *ACE* insertion variant was similarly significantly associated with a lower risk of T2DN compared with T2DM in a South Asian population, despite the presence of a high level of heterogeneity (P= 0.01; OR= 0.57; 95% CI= 0.37, 0.87; I^2= 89%). This was consistent with the direction of effect in four studies, which contributed to 67.7% of the weight in this analysis (Figure 2c). The funnel plot for this analysis is displayed within Figure 2d.

Figure 2c: Forest plot - *ACE* I/D investigation, individuals with type 2 diabetes and nephropathy compared to individuals with type 2 diabetes mellitus (without nephropathy) in a South Asian population (*ACE* insertion compared to deletion).

Figure 2d: Funnel plot - *ACE* I/D investigation, individuals with type 2 diabetic nephropathy compared to individuals with type 2 diabetes mellitus in a South Asian population (*ACE* insertion compared to deletion).

The comparison of East Asian individuals with end-stage renal disease (ESRD) compared to a healthy population with no evidence of renal disease also showed a significant association with moderate levels of heterogeneity (P= 0.008; OR= 0.8; 95% CI= 0.67, 0.94; I^2= 68%). Four studies, supporting 69.7% of the weight in the analysis had shown this effect (Figure 2e). Figure 2f shows the associated funnel plot for this analysis.

Figure 2e: Forest plot - *ACE* I/D investigation, individuals with end-stage renal disease compared to healthy controls in an East Asian population (*ACE* insertion compared to deletion).

Figure 2f: Funnel plot - *ACE* I/D investigation, individuals with end-stage renal disease compared to healthy controls in an East Asian population (*ACE* insertion compared to deletion).

In each analysis (T2DN vs. T2DM; ESRD vs. normal), the presence of the *ACE* insertion was associated with a lower risk of developing the CKD phenotype in the respective populations. The non-significant

For peer review only - http://bmjopen.bmj.com/site/about/guidelines.xhtml

forest plots are included in Supplementary Figures S2a-S2l and the associated funnel plots within Supplementary Figures S3a-S3l.

AGT

Seven quantitative analyses were completed for rs699, where the T allele was compared to the C allele. Details of each of these comparisons are included in Supplementary Table S5. One significant result was identified - the comparison of ESRD with healthy controls in a European population. The results were (P=0.002; OR= 0.84; 95% CI= 0.76, 0.94; I^2= 0%) and are shown in Figure 2g. Only the study with population size over 2000 patients, supporting 46.2% of the weight in the meta-analysis, had achieved significance with the original figures. These results indicate that the presence of the T allele is associated with a lower risk of developing ESRD in this population. The funnel plot for this analysis is displayed within Figure 2h. The forest plots containing the non-significant results are included in Supplementary Figures S4a-S4f, and the associated funnel plots within Supplementary Figures S5a-S5f.

Figure 2g: Forest plot - *AGT* rs699 investigation, individuals with end-stage renal disease compared to healthy controls in a European population (*AGT* rs699 T allele compared to C allele).

Figure 2h: Funnel plot - *AGT* rs699 investigation, individuals with end-stage renal disease compared to healthy controls in a European population (*AGT* rs699 T allele compared to C allele).

AGTR1

Four quantitative analyses were performed for *AGTR1* rs5186, comparing the A allele with the C. Details of these comparisons are included within Supplementary Table S6. One of the four studies returned a significant result for association, the comparison of T2DN with T2DM in a South Asian population (P= 0.001; OR= 0.71; 95% CI= 0.58, 0.87; I^2= 37%). This result resembled the findings of two of the three studies which contributed 87% of the weight in the meta-analysis, as shown in Figure 2i. Figure 2j contains the associated funnel plot. These results indicate that the presence of the A allele is associated with a lower risk of developing T2DN in this population. The non-significant results in forest plot format are included in Supplementary Figures S6a-S6c and in funnel plot format within Supplementary Figures S7a-S7c.

Figure 2i: Forest plot - *AGTR1* rs5186 investigation, individuals with type 2 diabetic nephropathy compared to individuals with type 2 diabetes mellitus in a South Asian population (*AGTR1* rs5186 A allele compared to C allele).

Figure 2j: Funnel plot - *AGTR1* rs5186 investigation, individuals with type 2 diabetes and nephropathy compared to individuals with type 2 diabetes mellitus (without nephropathy) in a South Asian population (*AGTR1* rs5186 A allele compared to C allele).

Studies not complying with Hardy Weinberg equilibrium are shown in Supplementary Table S7. Allele frequencies for healthy control populations included in this analysis were assessed. None showed signs of bias in any included study, in comparison to all available dbSNP reported frequencies as shown in Supplementary Data File S1.

For peer review only

DISCUSSION

Investigations into RAAS genetic variants previously reported to have been associated with a range of CKD phenotypes were undertaken. A total of 3,531 studies were identified, 114 of which met the inclusion criteria. Subsequently, 26 quantitative analyses were completed for three RAAS genes where there were at least three independent population studies of the RAAS gene variants. Five significant results within three genes were obtained at the significance level $p<0.05$, each revealing an association with CKD.

ACE is encoded by *DCP1* and is a key component of the RAAS. It catalyses the modification of angiotensin I to II, which is more biologically active(30). *ACE* is the most frequently studied gene of the RAAS(31). First sequenced in 1992(32), this 287 bp ALU repetitive element at intron 16(32) is located on chromosome 17 and is represented by four individual SNPs, rs4646994, rs1799752, rs4340 and rs13447447. Further understanding of its genetic architecture and disease associations may enable patient groups to benefit from targeted therapies with ACE inhibitors(33).

Since 1994, the association of *ACE* and DN has been rigorously investigated(33,34), with studies returning conflicting results. A meta-analysis undertaken in 2005 by Ng and colleagues(35) reported a statistically significant result wherein the *ACE* insertion was associated with protection from development of diabetic nephropathy in Asians and Caucasians. A second meta-analysis undertaken in 2012 also identified an association between the *ACE* I/D polymorphism and the development of diabetic nephropathy to ESRD, in that the presence of the deletion was associated with ESRD susceptibility(36). Despite having differences in the inclusion criteria to our meta-analysis, in that these investigations did not include any article published since 2011(35,36) and included individuals with microalbuminuria as cases(35), we returned similar results to the 2005 previous meta-analysis. Including individuals with microalbuminuria in the control population may cause challenges with phenotype definition as microalbuminuria may regress, remain stable, or progress to macroalbuminuria(37–39) over time. Individuals with microalbuminuria were therefore excluded from both case and control definitions to clarify the phenotypes in our review.

In our meta-analysis of *ACE*, which comprised 15,265 individuals with CKD and 18,474 individuals as controls from 98 population groups, we identified three significant associations of the *ACE* I/D polymorphism with CKD. Due to previously reported heterogeneity between different ethnic groups(35), we included this as a risk factor and performed each analysis per ethnicity (Table S2). Despite some of the allele frequency distributions varying across different ethnicities, the direction of the effect was consistent among the different ethnicities: where population had different MAFs, they often reported similar odds ratios in the meta-analysis.

Firstly, comparisons between T2DN and T2DM in East Asian and South Asian populations returned significant results highlighting a protective effect of the *ACE* insertion in the development of diabetic nephropathy (P=0.009 and P=0.01 respectively). This result was mimicked in the comparison of individuals with ESRD, which was not caused by diabetic nephropathy, and healthy control individuals in an East Asian population (P=0.008). The *ACE* I/D polymorphism remains a well characterised genetic locus associated with the progression of diabetic nephropathy.

AGT encodes the AGT glycoprotein, which is created in the liver and facilitates the creation of angiotensin I(11,40). It is located on chromosome 1. Several investigations have been conducted into the *AGT* gene variants and their association with risk of CKD(40–43). Among these, Zhou and colleagues undertook a meta-analysis investigation into *AGT* rs699 and its association with ESRD(40). The results of this study are agreement with ours in relation to the European ethnicity. Our meta-

For peer review only - http://bmjopen.bmj.com/site/about/guidelines.xhtml

analysis encompassing 5, 463 individuals with renal disease and 6,385 individuals without; the T allele provided a protective effect in ESRD development (P=0.002) within this European population.

In European and Middle Eastern populations, the allele distribution of rs699 were very similar for the comparison of T2DN vs T2DM, yet different from the East Asian population. This may have had an impact on the results, potentially limiting the robustness of association analyses across ethnicities.

The *AGTR1* gene has been shown to be involved in the regulation of blood pressure, fluid and electrolyte balance(11). It may also have a role in inflammation and vasoconstriction(44). SNP rs5186, an AC nucleotide substitution at position 1166 in the 3' untranslated region of chromosome 3, is reportedly able to be recognised by microRNA-155. When the A allele is present at this locus, microRNA-155 is able to undergo complementary base-pairing with AGTR1 messenger RNA to suppress translation. However this is not possible when the alternative C allele is present, resulting in increased AGTR1 protein levels(45). This interplay may affect blood pressure regulation and warrants further investigation(45).

Previous studies have identified associations between rs5186 in *AGTR1* and diseases including coronary artery disease(46), systemic lupus erythematosus(47) and cancer(48). Several smaller studies had also been undertaken to assess links between this gene and renal disease(25,49–54). We conducted this study to provide a clearer understanding of the effect of this SNP on CKD.

This meta-analysis of *AGTR1* variants included 3,197 individuals with renal disease and 3,720 controls investigating rs5186. One significant result was identified; that the presence of an A allele at this locus provided a lower risk of developing T2DN in a South Asian population (P=0.001).

One meta-analysis published in 2014(55) assessed AGTR1 in individuals with CKD, ESRD, IgA nephropathy or vesicoureteral reflux. This meta-analysis identified that rs5186 was not associated with any of these diseases, which corresponds to the results from our investigation.

The three remaining RAAS genes included in this meta-analysis, *ACE2*, *AGTR2* and *REN,* have not been researched as extensively as the *ACE, AGT* and *AGTR1* for associations with renal disease. Very few articles were identified describing genetic association studies for these genes at the data extraction stage of the analysis, and those that were, were removed prior to the quantitative analysis stage, mainly due to the inclusion of paediatric individuals or non-human approaches, as outlined in Figures S1b, S1e and S1f. Further research into the *ACE2*, *AGTR2* and *REN* genes and their polymorphisms should be undertaken to elucidate their role in CKD and ESRD.

Some studies in our search could not be included in quantitative analysis as they lacked information relating to genotype counts and had an unclear measure and definition of albuminuria for both cases and controls, which could constitute a limitation. Results from GWAS studies would have strengthened the analysis, but unfortunately they usually only report significant SNPs. Absolute frequencies are not usually reported at individual SNP level for such large-scale studies. Publication bias was reported in two of our quantitative analyses, but was not found in the analyses which provided our significant results. Lack of clarity in phenotype definitions, along with unclear descriptions of ethnicities, inherently challenge the use of meta-analyses of different populations as a valid instrument to uncover robust associations. CKD itself has a range of causes including glomerular damage and declining eGFR without albuminuria. Other confounding factors such as hypertension and cardiovascular disease, and a lack of prospective follow up of included individuals, which would ensure phenotypes are robust and stable, may also cause conflicting results.

For peer review only - http://bmjopen.bmj.com/site/about/guidelines.xhtml

CONCLUSION

This meta-analysis of the RAAS pathway genes and their association with renal disease has provided evidence for five significant associations with individually small effect sizes that may cumulatively contribute to dysfunction of the RAAS pathway leading to kidney disease. The insertion in *ACE* I/D polymorphism was a protection factor for the development of diabetic nephropathy in individuals with type 2 diabetes mellitus from both East and South Asian origin, and for ESRD in an East Asian population. In Europeans, the T allele of the *AGT* rs699 conferred a lower risk of ESRD development in healthy population. The A allele in *AGTR1* rs5186 acted as a protection factor for renal disease development in South Asian population.

Further study into the specific ethnicities and investigations into a broader range of RAAS linked genes, or a deeper analysis of them including investigations of more variants, may pinpoint the molecular basis underlying the role of pathway in kidney disease. Network analysis and functional studies enlightening the mechanisms involved ultimately will be required to complete the picture of RAAS variation in renal traits.

For peer review only

STATEMENTS AND CONTRIBUTIONS

Acknowledgements and Funding

This work has been partly funded by the Medical Research Council (Award reference MC_PC_15025) and the Public Health Agency R&D Division (Award Reference STL/4760/13). LJS is the recipient of a Northern Ireland Kidney Research Fund Fellowship. MCG is funded by a Science Foundation Ireland-Department for the Economy (SFI-DfE) Investigator Program Partnership Award (15/IA/3152). RCC is the recipient of a Department for the Economy (DfE) PhD studentship. None of the funding bodies had a role in the study design or analysis.

Statement of competing interests

The authors declare that they have no competing interests.

Availability of data and material

The datasets generated and/or analysed during the current study are available from the corresponding author on reasonable request.

Author contributions

LJS formulated the research plan, conducted the analysis, interpreted the data, drafted the manuscript and revised the manuscript.

MCG conducted the analysis, interpreted the data and revised the manuscript.

RCC conducted the analysis, interpreted the data and revised the manuscript.

APM acquired the funding, interpreted the data, managed the project and revised the manuscript.

AJM acquired the funding, formulated the research plan, interpreted the data, managed the project and revised the manuscript.

All authors read and approved the final manuscript.

For peer review only

REFERENCES

1. Levey AS, Coresh J. Chronic kidney disease. Lancet. 2012 Jan 14;379(9811):165–80.
2. Glassock RJ, Warnock DG, Delanaye P. The global burden of chronic kidney disease: Estimates, variability and pitfalls. Nat Rev Nephrol. 2017;13(2):104–14.
3. Mills KT, Xu Y, Zhang W, Bundy JD, Chen C-S, Kelly TN, et al. A systematic analysis of world-wide population-based data on the global burden of chronic kidney disease in 2010 HHS Public Access. Kidney Int. 2015;88(5):950–7.
4. Smyth LJ, Duffy S, Maxwell AP, McKnight AJ. Genetic and epigenetic factors influencing chronic kidney disease. Am J Physiol Renal Physiol. 2014;307(7):F757-76.
5. Brancati FL, Whelton PK, Randall BL, Neaton JD, Stamler J, Klag MJ. Risk of end-stage renal disease in diabetes mellitus: a prospective cohort study of men screened for MRFIT. Multiple Risk Factor Intervention Trial. JAMA. 1997;278(23):2069–74.
6. Klag MJ, Whelton PK, Randall BL, Neaton JD, Brancati FL, Ford CE, et al. Blood pressure and end-stage renal disease in men. N Engl J Med. 1996;334(1):13–8.
7. Cowie CC, Port FK, Wolfe RA, Savage PJ, Moll PP, Hawthorne VM. Disparities in Incidence of Diabetic End-Stage Renal Disease According to Race and Type of Diabetes. N Engl J Med. 1989;321(16):1074–9.
8. Mathur R, Dreyer G, Yaqoob MM, Hull SA. Ethnic differences in the progression of chronic kidney disease and risk of death in a UK diabetic population: an observational cohort study. BMJ Open. 2018;8(3):e020145.
9. Velez JCQ. The importance of the intrarenal renin-angiotensin system. Nat Clin Pr Nephrol. 2009;5(2):89–100.
10. Nishiyama A, Kim-Mitsuyama S. New Approaches to Blockade of the Renin–Angiotensin–Aldosterone System: Overview of Regulation of the Renin–Angiotensin–Aldosterone System. J Pharmacol Sci. 2010;113(4):289–91.
11. Remuzzi G, Perico N, Macia M, Ruggenenti P. The role of renin-angiotensin-aldosterone system in the progression of chronic kidney disease. Kidney Int Suppl. 2005;68(99):S57–65.
12. Nishiyama A, Kobori H. Independent regulation of renin–angiotensin–aldosterone system in the kidney. Clin Exp Nephrol. 2018;doi: 10.1007/s10157-018-1567-1.
13. Carey RM. The intrarenal renin-angiotensin system in hypertension. Adv Chronic Kidney Dis. 2015;22(3):204–10.
14. Zhang F, Liu H, Liu D, Liu Y, Li H, Tan X, et al. Effects of RAAS Inhibitors in Patients with Kidney Disease. Curr Hypertens Rep. 2017;19(9):72.
15. Coll E, Campos B, González-Núñez D, Botey A, Poch E. Association between the A1166C polymorphism of the angiotensin II receptor type 1 and progression of chronic renal insufficiency. J Nephrol. 2003;16(3):357–64.
16. Bermejo S, García CO, Rodríguez E, Barrios C, Otero S, Mojal S, et al. The renin-angiotensin-aldosterone system blockade in patients with advanced diabetic kidney disease. Nefrologia. 2017;32(2):197–206.
17. Yamout H, Lazich I, Bakris GL. Blood pressure, hypertension, RAAS blockade, and drug therapy in diabetic kidney disease. Adv Chronic Kidney Dis. 2014;21(3):281–6.

For peer review only - http://bmjopen.bmj.com/site/about/guidelines.xhtml

18. Rahimi Z. The Role of Renin Angiotensin Aldosterone System Genes in Diabetic Nephropathy. Can J Diabetes. 2016;40(2):178–83.

19. Mezzano S a, Ruiz-Ortega M, Egido J. Angiotensin II and renal fibrosis. Hypertension. 2001;38(3 Pt 2):635–8.

20. Kelly TN, Raj D, Rahman M, Kretzler M, Kallem RR, Ricardo AC, et al. The role of renin-angiotensin-aldosterone system genes in the progression of chronic kidney disease: Findings from the Chronic Renal Insufficiency Cohort (CRIC) study. Nephrol Dial Transplant. 2015;30(10):1711–8.

21. Ramanathan G, Elumalai R, Periyasamy S, Lakkakula BVKS. Renin gene rs1464816 polymorphism contributes to chronic kidney disease progression in ADPKD. J Biomed Sci. 2016;23(1):1–7.

22. Kawada N, Moriyama T, Yokoyama K, Yamauchi A, Ando A, Mikami H, et al. Renin-angiotensin system component gene polymorphisms in Japanese maintenance haemodialysis patients. Nephrology. 1997;3(4):521–6.

23. Parsa A, Lovett DH, Peden EA, Zhu L, Seldin MF, Criswell LA. Renin-angiotensin system gene polymorphisms predict the progression to renal insufficiency among Asians with lupus nephritis. Genes Immun. 2005;6(3):217–24.

24. Özkaya O, Söylemezoğlu O, Gönen S, Misirlioğolu M, Tuncer S, Kalman S, et al. Renin-angiotensin system gene polymorphisms: Association with susceptibility to Henoch-Schonlein purpura and renal involvement. Clin Rheumatol. 2006;25(6):861–5.

25. Lau YK, Woo KT, Choong HL, Zhao Y, Tan HB, Chong SMCF, et al. Renin-angiotensin system gene polymorphisms: Its impact on IgAN and its progression to end-stage renal failure among Chinese in Singapore. Nephron - Physiol. 2004;97(1):1–9.

26. Mtiraoui N, Ezzidi I, Turki A, Chaieb M, Mahjoub T, Almawi WY. Renin-angiotensin-aldosterone system genotypes and haplotypes affect the susceptibility to nephropathy in type 2 diabetes patients. J Renin Angiotensin Aldosterone Syst. 2011;12(4):572–80.

27. Stroup DF, Berlin JA, Morton SC, Olkin I, Williamson GD, Rennie D, et al. Meta-analysis of observational studies in epidemiology: a proposal for reporting. Meta-analysis Of Observational Studies in Epidemiology (MOOSE) group. JAMA. 2000;283(15):2008–12.

28. International Genome Population [Internet]. Available from: www.internationalgenome.org/category/population

29. Higgins JPT, Thompson SG, Deeks JJ, Altman DG. Measuring inconsistency in meta-analyses. BMJ Br Med J. 2003;327(7414):557–60.

30. Rieder MJ, Taylor SL, Clark AG, Nickerson DA. Sequence variation in the human angiotensin converting enzyme. Nat Genet. 1999;22(1):59–62.

31. Moon JY. Recent update of renin-angiotensin-aldosterone system in the pathogenesis of hypertension. Electrolyte Blood Press. 2013;11(2):41–5.

32. Rigat B, Hubert C, Corvol P, Soubrier R. PCR detection of the insertion/deletion polymorphism of the human angiotensin converting enzyme gene (DCP1) (dipeptidyl carboxypeptidase 1). Nucleic Acids Res. 1992;20(6):1433.

33. Ha S-K. ACE Insertion/Deletion Polymorphism and Diabetic Nephropathy: Clinical Implications of Genetic Information. J Diabetes Res. 2014;2014:1–8.

For peer review only

For peer review only - http://bmjopen.bmj.com/site/about/guidelines.xhtml

34. Marre M, Bernadet P, Gallois Y, Savagner F, Guyene TT, Hallab M, et al. Relationships between angiotensin I converting enzyme gene polymorphism, plasma levels, and diabetic retinal and renal complications. Diabetes. 1994;43(3):384–8.

35. Ng DPK, Tai BC, Koh D, Tan KW, Chia KS. Angiotensin-I converting enzyme insertion/deletion polymorphism and its association with diabetic nephropathy: A meta-analysis of studies reported between 1994 and 2004 and comprising 14,727 subjects. Diabetologia. 2005;48(5):1008–16.

36. Yu Z-Y, Chen L-S, Zhang L-C, Zhou T-B. Meta-analysis of the relationship between ACE I/D gene polymorphism and end-stage renal disease in patients with diabetic nephropathy. Nephrology (Carlton). 2012;17(5):480–7.

37. Caramori ML, Fioretto P, Mauer M. The need for early predictors of diabetic nephropathy risk: Is albumin excretion rate sufficient? Diabetes. 2000;49(9):1399–408.

38. Perkins BA, Ficociello LH, Silva KH, Finkelstein DM, Warram JH, Krolewski AS. Regression of microalbuminuria in type 1 diabetes. N Engl J Med. 2003;348(23):2285–93.

39. Zelmanovitz T, Gerchman F, Balthazar AP, Thomazelli FC, Matos JD, Canani LH. Diabetic nephropathy. Diabetol Metab Syndr. 2009;1(1):10.

40. Zhou T-B, Yin S-S, Qin Y-H. Association of angiotensinogen M235T gene polymorphism with end-stage renal disease risk: a meta-analysis. Mol Biol Rep. 2013;40(2):765–72.

41. Turgut S, AkIn F, AkcIlar R, Ayada C, Turgut G. Angiotensin converting enzyme I/D, angiotensinogen M235T and AT1-R A/C1166 gene polymorphisms in patients with acromegaly. Mol Biol Rep. 2011;38(1):569–76.

42. Wang YJ, Pan Y. Angiotensinogen gene M235T polymorphism and risk of coronary artery disease: a meta-analysis. Mol Med Rep. 2012;6(4):884–8.

43. Lin R, Lei Y, Yuan Z, Ju H, Li D. Angiotensinogen Gene M235T and T174M Polymorphisms and Susceptibility of Pre-Eclampsia: A Meta-Analysis. Ann Hum Genet. 2012;76(5):377–86.

44. Chow BSM, Allen TJ. Angiotensin II type 2 receptor (AT2R) in renal and cardiovascular disease. Clin Sci. 2016;130(15):1307–26.

45. Ceolotto G, Papparella I, Bortoluzzi A, Strapazzon G, Ragazzo F, Bratti P, et al. Interplay between miR-155, AT1R A1166C polymorphism, and AT1R expression in young untreated hypertensives. Am J Hypertens. 2011;24(2):241–6.

46. Duan LJ, Wang XD. Analysis of correlations between coronary heart disease and haplotypes of the angiotensin II receptor type 1 (AGTR1) gene. Genet Mol Res. 2016;15(1):doi: 10.4238/gmr.15017457.

47. Baniamerian H, Bahrehmand F, Vaisi-Raygani A, Rahimi Z, Pourmotabbed T. Angiotensin type 1 receptor A1166C polymorphism and systemic lupus erythematosus: correlation with cellular immunity and oxidative stress markers. Lupus. 2017;26(14):1534–9.

48. Pringle KG, Delforce SJ, Wang Y, Ashton KA, Proietto A, Otton G, et al. Renin–angiotensin system gene polymorphisms and endometrial cancer. Endocr Connect. 2016;5(3):128–35.

49. Gao J, Yu Q-L, Fu R-G, Wei L-T, Wang M, Dong F-M, et al. Lack of Association Between Polymorphisms in AGT and ATR1 and IgA Nephropathy in a Chinese Population. Genet Test Mol Biomarkers. 2015;19(12):710–3.

50. Huang HD, Lin FJ, Li XJ, Wang LR, Jiang GR. Genetic polymorphisms of the renin-angiotensin-

For peer review only

For peer review only - http://bmjopen.bmj.com/site/about/guidelines.xhtml

aldosterone system in Chinese patients with end-stage renal disease secondary to IgA nephropathy. Chin Med J (Engl). 2010;123(22):3238–42.

51. Kim SM, Chin HJ, Oh YK, Kim YS, Kim S, Lim CS. Blood pressure-related genes and the progression of IgA nephropathy. Nephron Clin Pr. 2009;113(4):c301-8.

52. Savage DA, Feeney SA, Fogarty DG, Maxwell AP. Risk of developing diabetic nephropathy is not associated with synergism between the angiotensin II (type 1) receptor C1166 allele and poor glycaemic control. Nephrol Dial Transpl. 1999;14(4):891–4.

53. Van Ittersum FJ, De Man AME, Thijssen S, De Knijff P, Slagboom E, Smulders Y, et al. Genetic polymorphisms of the renin-angiotensin system and complications of insulin-dependent diabetes mellitus. Nephrol Dial Transplant. 2000;15(7):1000–7.

54. Prasad P, Tiwari AK, Kumar KP, Ammini A, Gupta A, Gupta R, et al. Chronic renal insufficiency among Asian Indians with type 2 diabetes: I. Role of RAAS gene polymorphisms. BMC Med Genet. 2006;7(1):42.

55. Braliou GG, Grigoriadou AMG, Kontou PI, Bagos PG. The role of genetic polymorphisms of the Renin-Angiotensin System in renal diseases: A meta-analysis. Comput Struct Biotechnol J. 2014;10(7):1–7.

For peer review only

For peer review only - http://bmjopen.bmj.com/site/about/guidelines.xhtml

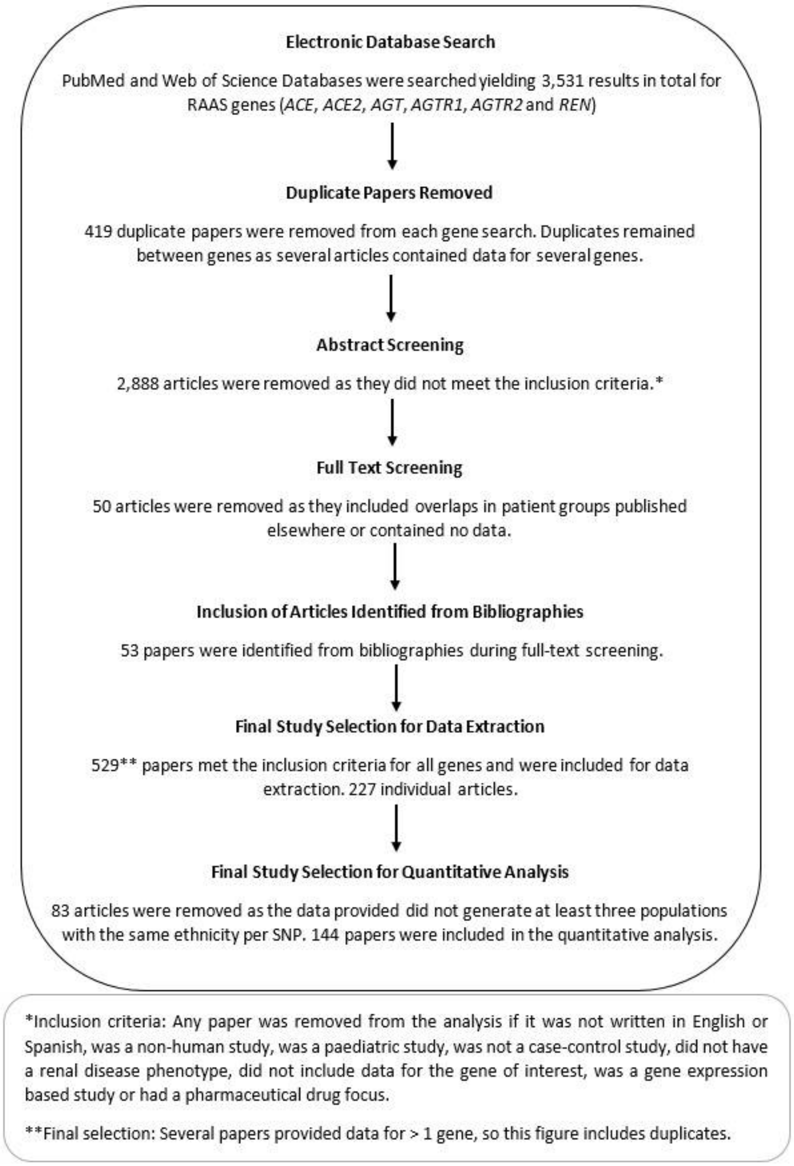

Figure 1: Workflow pattern

55x76mm (300 x 300 DPI)

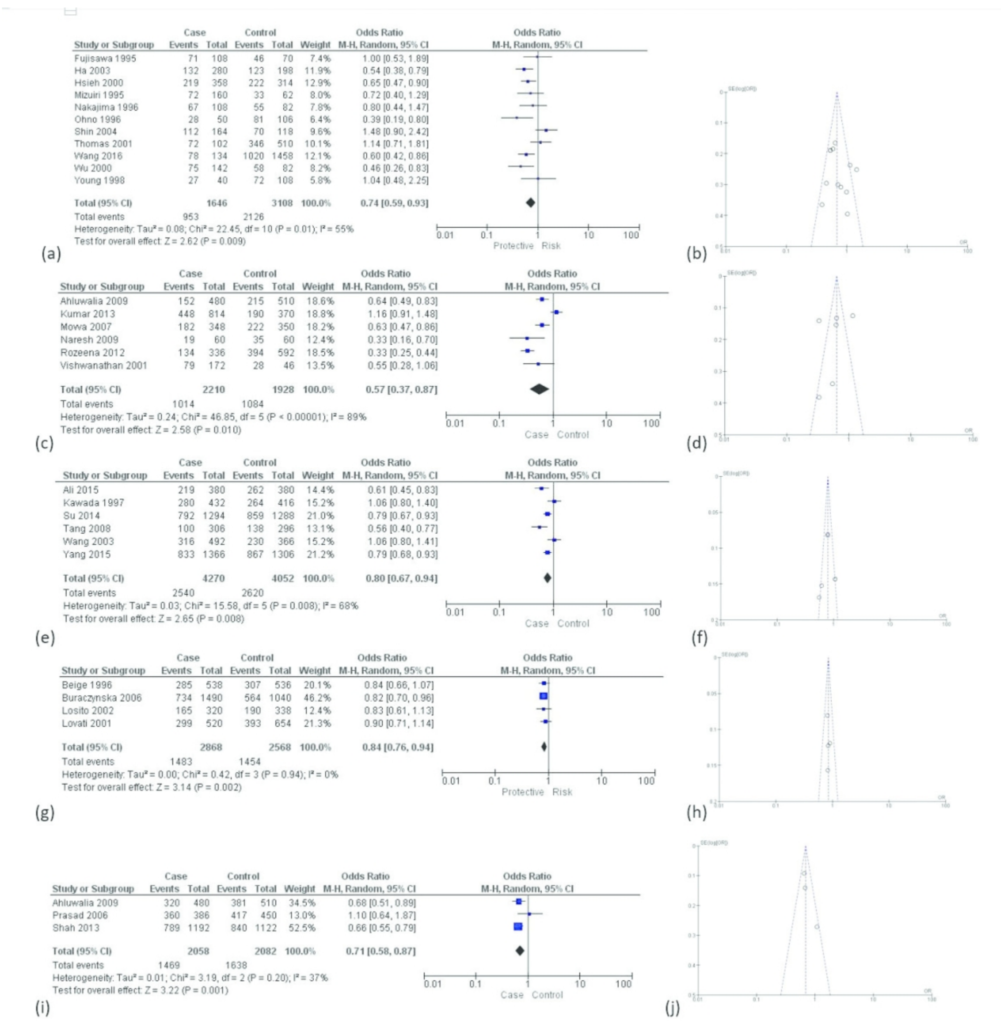

Figure 2: Forest and funnel plots for statistically significant results

119x124mm (300 x 300 DPI)

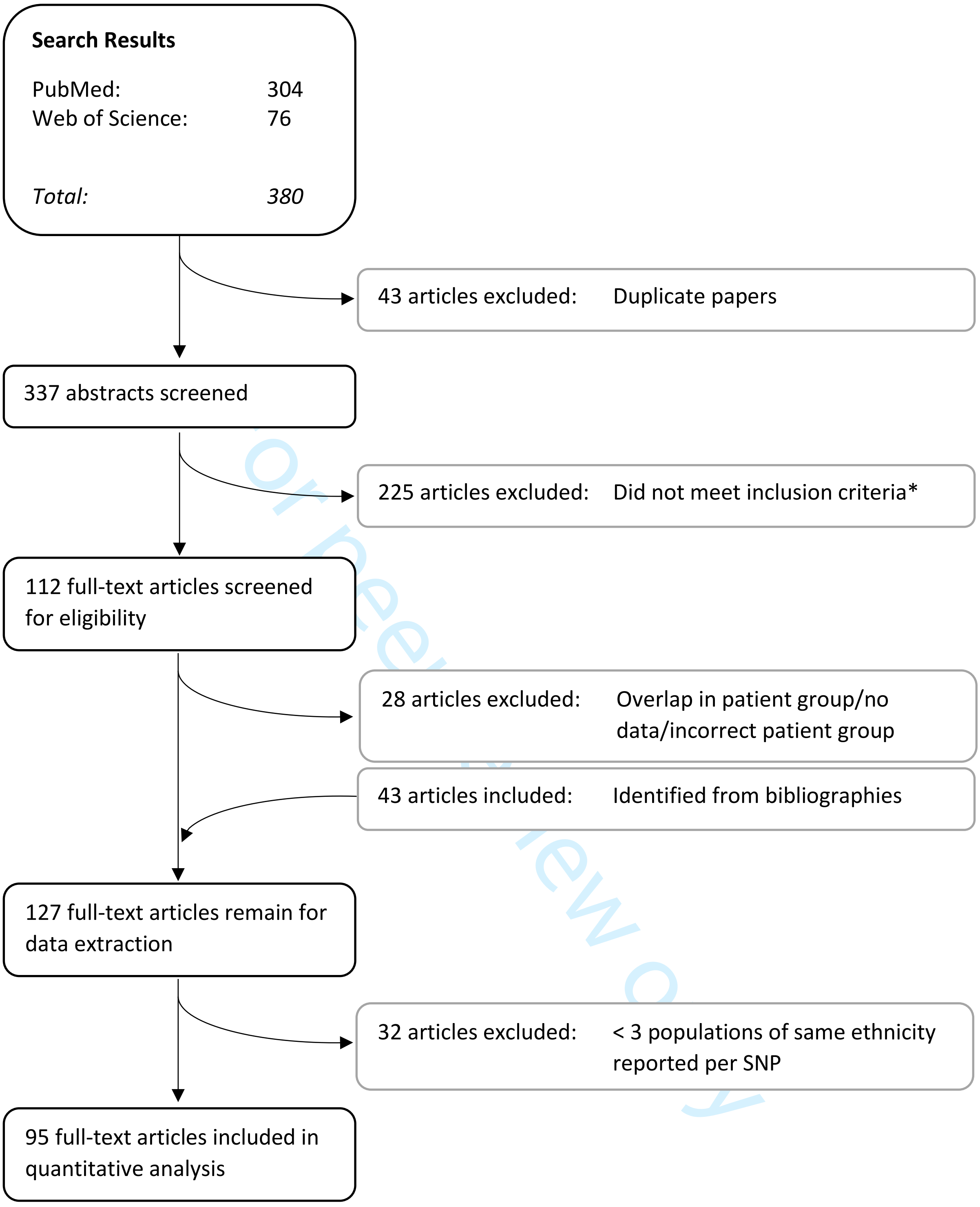

*Inclusion criteria: Any paper was removed from the analysis if it was not written in English or Spanish (n=20), was a non-human study (n=5), was a paediatric study (n=52), was not a case-control study (n=36), did not have a renal disease phenotype (n=90), did not include data for *ACE* (n=13) or had a pharmaceutical drug focus (n=9)

Supplementary Figure S1a: *ACE* Study Flow Diagram

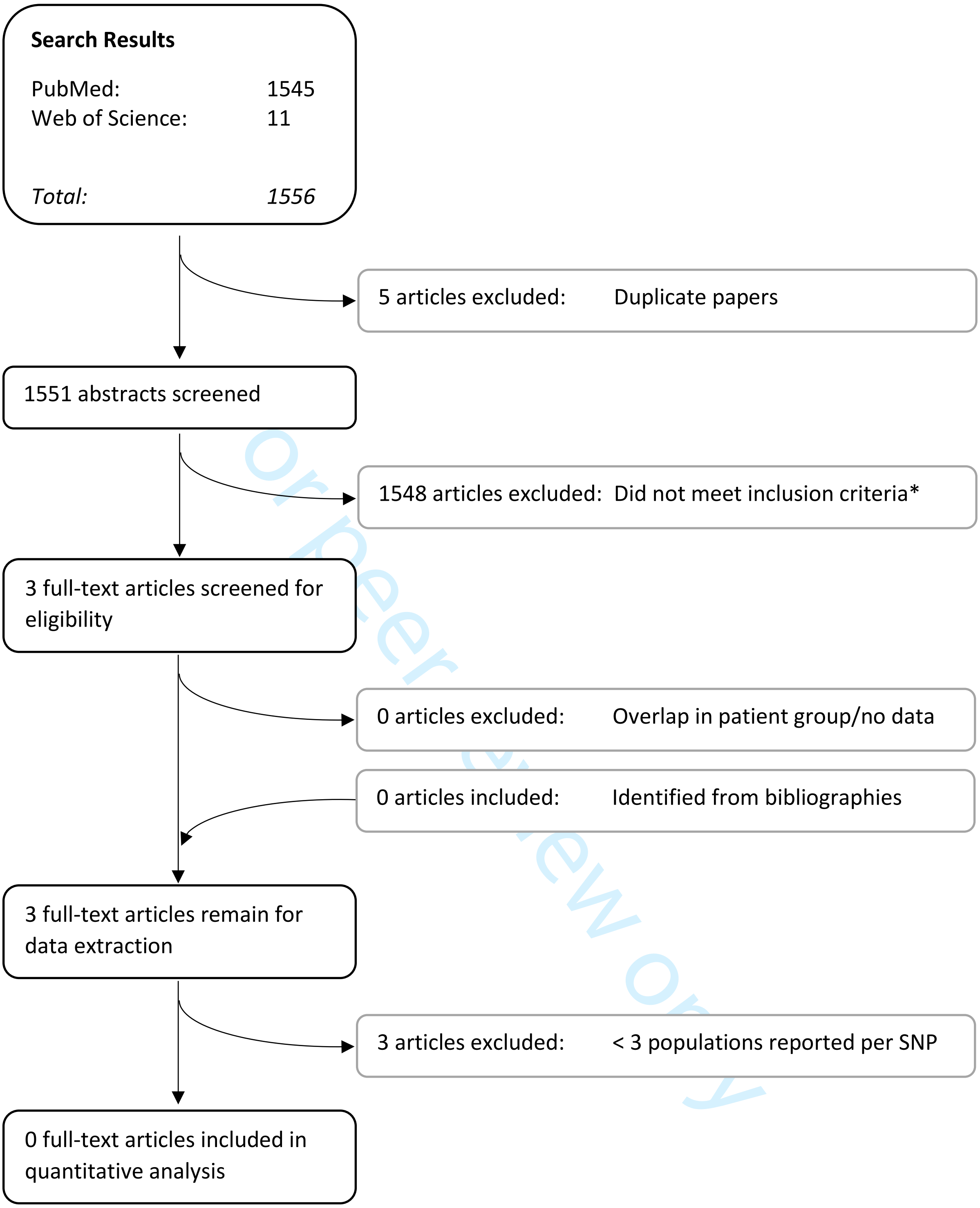

*Inclusion criteria: Any paper was removed from the analysis if it was not written in English or Spanish (n=55), was a non-human study (n=119), was a paediatric study (n=149), was not a case-control study (n=53), did not have a renal disease phenotype (n=693), did not include data for *ACE2* (n=435) or had a pharmaceutical drug focus (n=44)

Supplementary Figure S1b: *ACE2* Study Flow Diagram

For peer review only - http://bmjopen.bmj.com/site/about/guidelines.xhtml

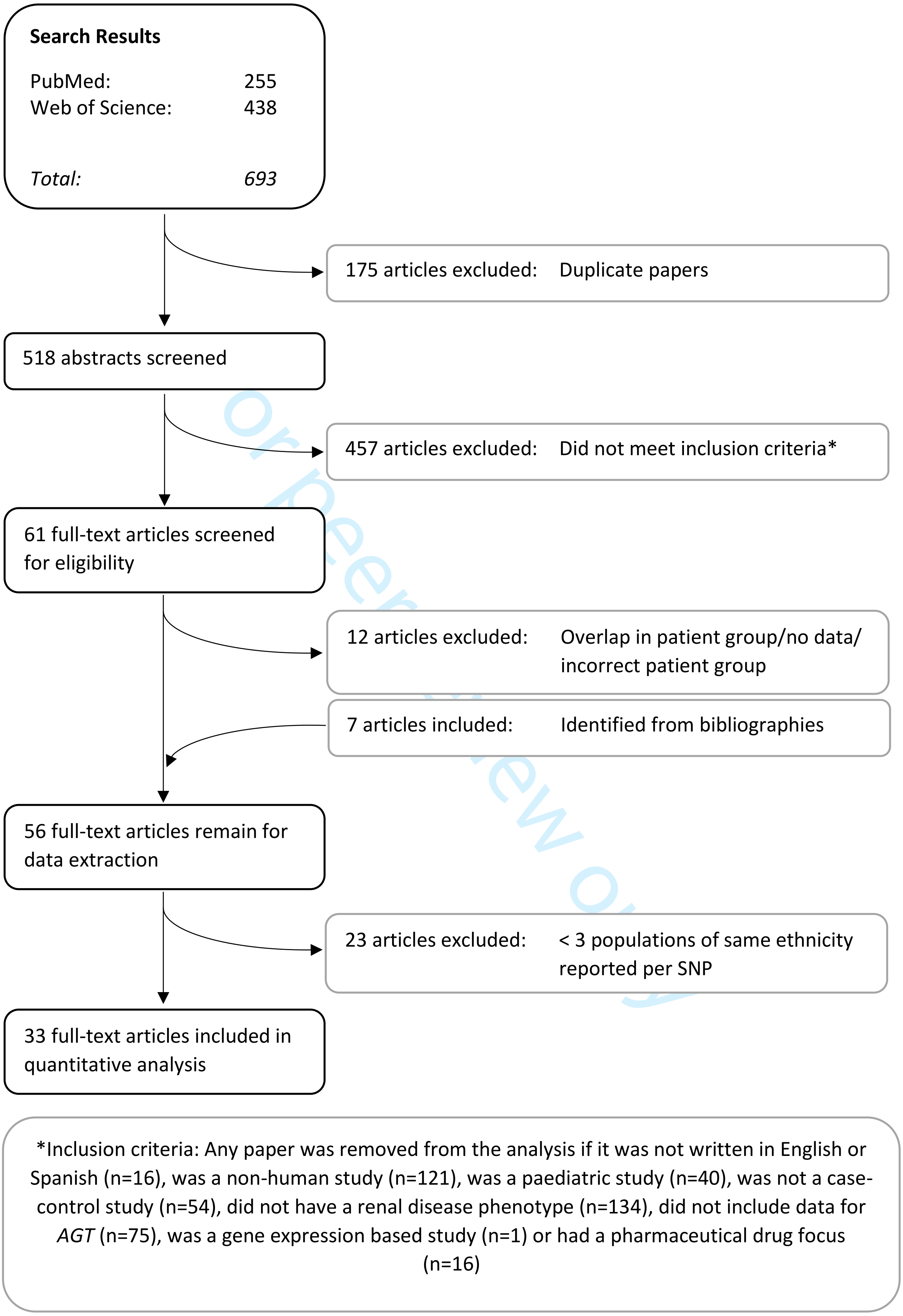

Supplementary Figure S1c: *AGT* Study Flow Diagram

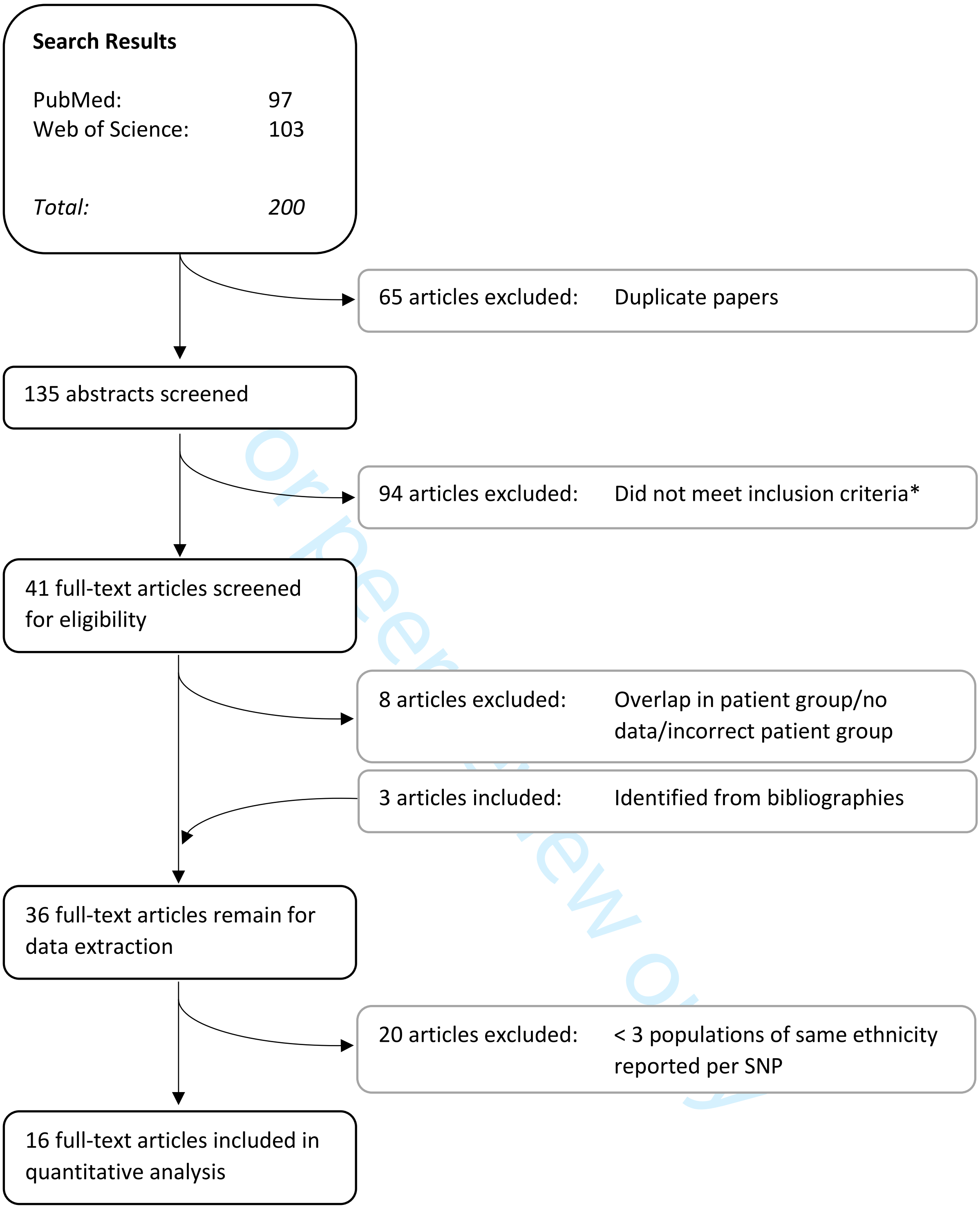

*Inclusion criteria: Any paper was removed from the analysis if it was not written in English or Spanish (n=8), was a non-human study (n=13), was a paediatric study (n=21), was not a case-control study (n=14), did not have a renal disease phenotype (n=25), did not include data for *AGTR1* (n=3), was a gene expression based study (n=1) or had a pharmaceutical drug focus (n=9)

Supplementary Figure S1d: *AGTR1* Study Flow Diagram

For peer review only - http://bmjopen.bmj.com/site/about/guidelines.xhtml

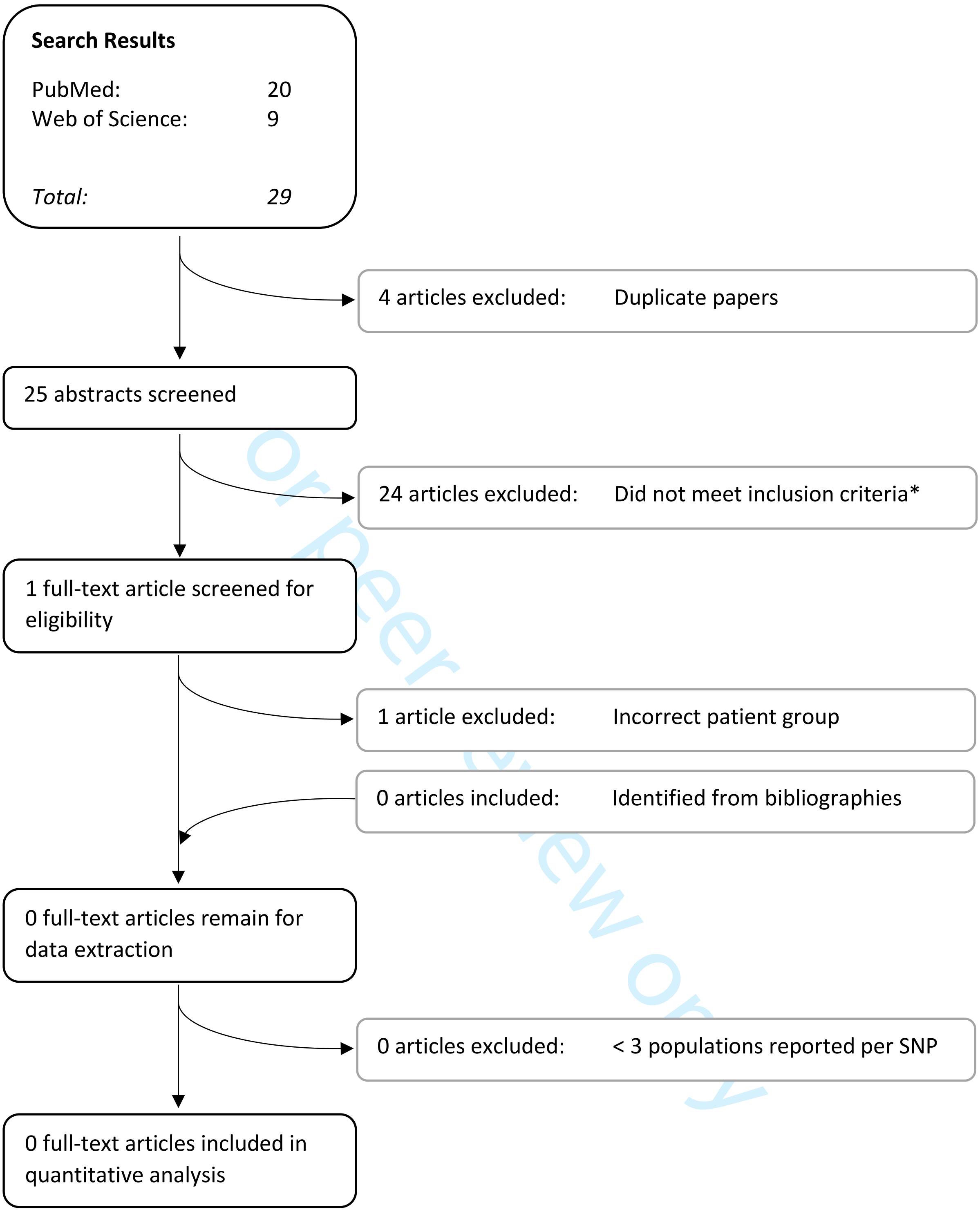

*Inclusion criteria: Any paper was removed from the analysis if it was not written in English or Spanish (n=1), was a non-human study (n=2), was a paediatric study (n=6), was not a case-control study (n=4), did not have a renal disease phenotype (n=5) or did not include data for *AGTR2* (n=6)

Supplementary Figure S1e: *AGTR2* Study Flow Diagram

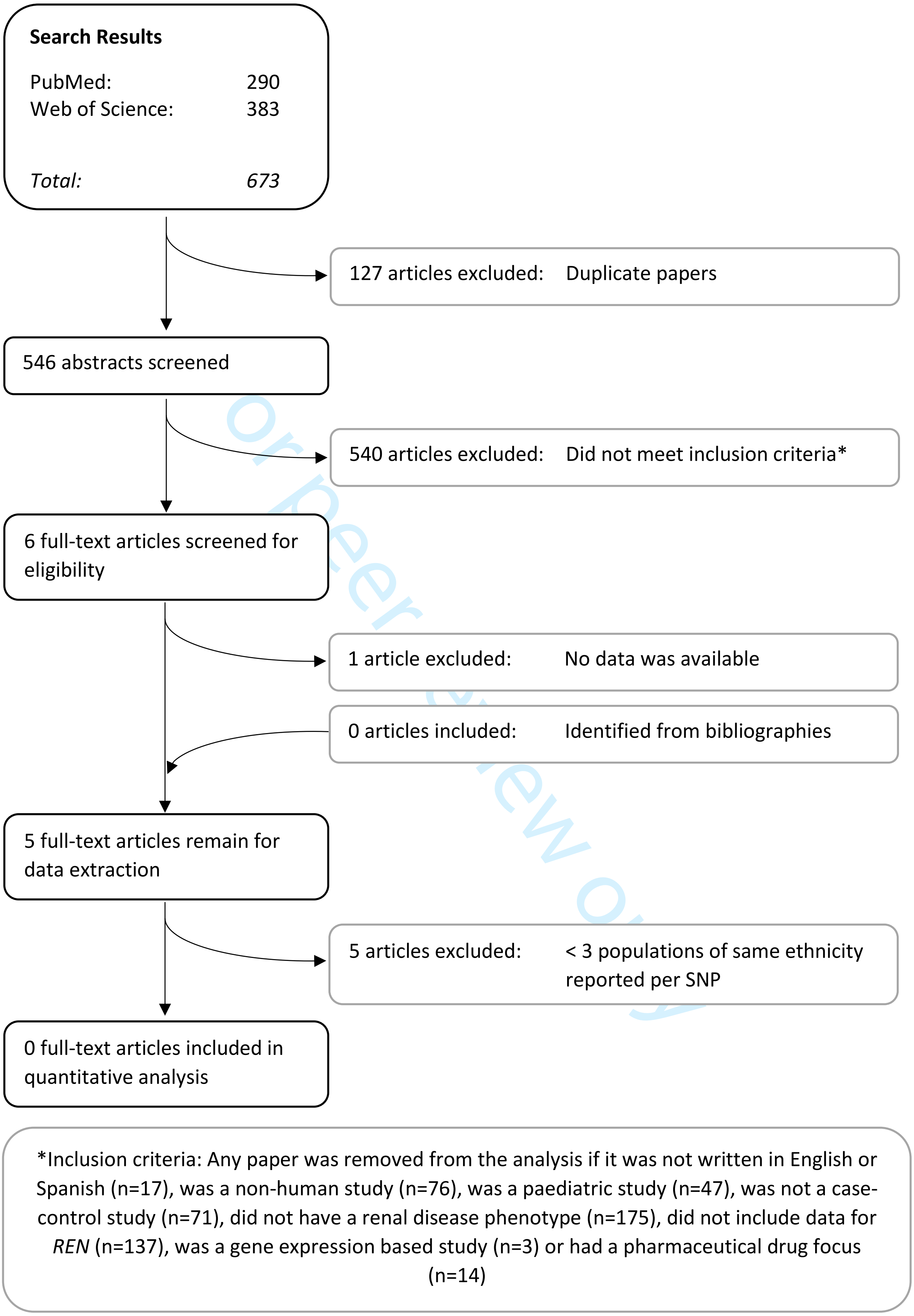

Supplementary Figure S1f: *REN* Study Flow Diagram

Study or Subgroup	Case Events	Case Total	Control Events	Control Total	Weight	Odds Ratio M-H, Random, 95% CI
Barnas 1997	45	100	51	80	4.0%	0.47 [0.25, 0.85]
Chowdhury 1996	204	484	143	332	10.6%	0.96 [0.73, 1.28]
Currie 2010	603	1380	696	1460	15.8%	0.85 [0.73, 0.99]
De Cosmo 1999	125	350	89	272	8.9%	1.14 [0.82, 1.60]
Demurov 1997	35	112	72	152	5.2%	0.51 [0.30, 0.84]
Hadjadj 2001	4	12	208	502	1.2%	0.71 [0.21, 2.38]
Hibberd 1997	60	144	57	172	6.0%	1.44 [0.91, 2.28]
Marre 1994	43	124	58	124	5.2%	0.60 [0.36, 1.01]
Ringel 1997	130	268	208	452	9.9%	1.11 [0.82, 1.50]
Schmidt 1995 A	86	228	101	266	8.1%	0.99 [0.69, 1.42]
Shestakova 2006	65	126	78	132	5.5%	0.74 [0.45, 1.21]
Tarnow 1995	175	396	169	380	10.5%	0.99 [0.74, 1.31]
van Ittersum 2000	33	60	192	376	4.7%	1.17 [0.68, 2.02]
Walder 1998	49	110	40	88	4.5%	0.96 [0.55, 1.69]
**Total (95% CI)**		**3894**		**4788**	**100.0%**	**0.91 [0.79, 1.04]**
Total events	1657		2162			

Heterogeneity: Tau² = 0.02; Chi² = 22.72, df = 13 (P = 0.05); I² = 43%
Test for overall effect: Z = 1.37 (P = 0.17)

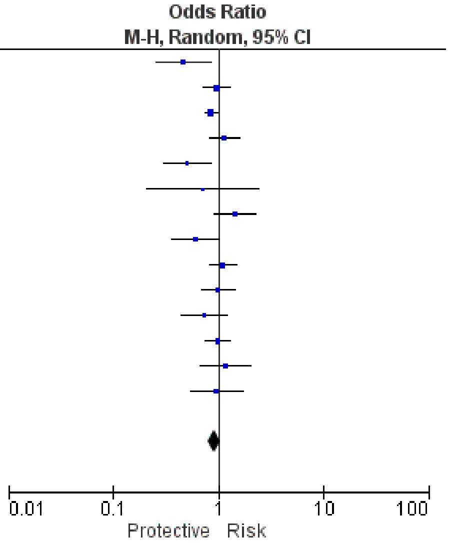

Supplementary Figure S2a: Forest plot - *ACE* I/D investigation, individuals with type 1 diabetic nephropathy compared to individuals with type 1 diabetes mellitus in a European population (*ACE* insertion compared to deletion).

Study or Subgroup	Case Events	Case Total	Control Events	Control Total	Weight	Odds Ratio M-H, Random, 95% CI
Dudley 1995	147	326	246	534	21.8%	0.96 [0.73, 1.27]
Fradin 2002	34	78	94	236	16.9%	1.17 [0.70, 1.96]
Ng 2006	242	582	147	334	21.9%	0.91 [0.69, 1.19]
Nikzamir 2009	21	96	159	290	16.5%	0.23 [0.13, 0.39]
Schmidt 1997 B	251	622	278	694	22.8%	1.01 [0.81, 1.26]
**Total (95% CI)**		**1704**		**2088**	**100.0%**	**0.78 [0.54, 1.15]**
Total events	695		924			

Heterogeneity: Tau² = 0.15; Chi² = 27.08, df = 4 (P < 0.0001); I² = 85%
Test for overall effect: Z = 1.26 (P = 0.21)

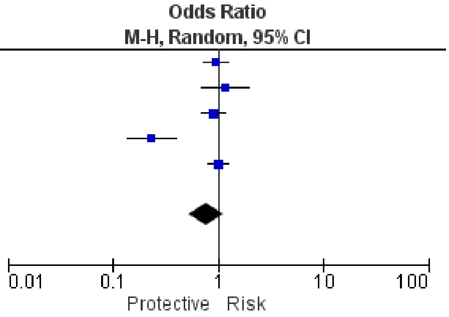

Supplementary Figure S2b: Forest plot - *ACE* I/D investigation, individuals with type 2 diabetic nephropathy compared to individuals with type 2 diabetes mellitus in a European population (*ACE* insertion compared to deletion).

For peer review only - http://bmjopen.bmj.com/site/about/guidelines.xhtml

	Case		Control			Odds Ratio
Study or Subgroup	Events	Total	Events	Total	Weight	M-H, Random, 95% CI
Al-Harbi 2011	63	220	125	392	12.7%	0.86 [0.60, 1.23]
Alharbi 2017	31	122	47	122	10.2%	0.54 [0.31, 0.94]
Araz 2001	52	124	103	246	11.7%	1.00 [0.65, 1.55]
El-Baz 2012	66	204	82	200	12.1%	0.69 [0.46, 1.03]
Ergen 2004	21	50	31	100	8.4%	1.61 [0.80, 3.26]
Eroglu 2008	43	92	50	112	10.2%	1.09 [0.63, 1.89]
Felehgari 2011	42	136	60	144	11.0%	0.63 [0.38, 1.02]
Rahimi 2012	42	136	60	144	11.0%	0.63 [0.38, 1.02]
Sancakdar 2015	180	388	62	200	12.7%	1.93 [1.34, 2.76]
**Total (95% CI)**		**1472**		**1660**	**100.0%**	**0.91 [0.67, 1.22]**
Total events	540		620			

Heterogeneity: Tau² = 0.15; Chi² = 29.48, df = 8 (P = 0.0003); I² = 73%
Test for overall effect: Z = 0.65 (P = 0.52)

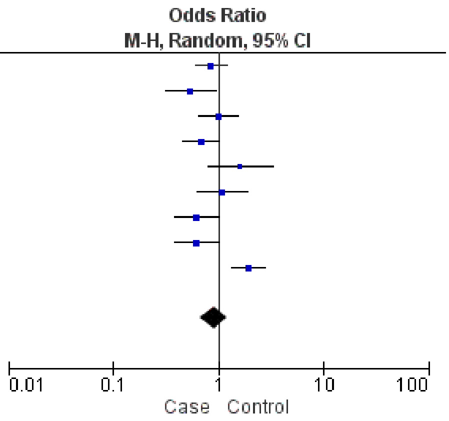

Supplementary Figure S2c: Forest plot - *ACE* I/D investigation, individuals with type 2 diabetic nephropathy compared to individuals with type 2 diabetes mellitus in a Middle Eastern population (*ACE* insertion compared to deletion).

	Case		Control			Odds Ratio
Study or Subgroup	Events	Total	Events	Total	Weight	M-H, Random, 95% CI
Ahluwalia 2009	124	236	99	196	26.9%	1.08 [0.74, 1.58]
Chen 2014	314	466	617	898	28.0%	0.94 [0.74, 1.20]
Nagamani 2015	152	294	195	422	27.6%	1.25 [0.92, 1.68]
Shanmuganathan 2015	10	60	55	60	17.6%	0.02 [0.01, 0.06]
**Total (95% CI)**		**1056**		**1576**	**100.0%**	**0.53 [0.24, 1.14]**
Total events	600		966			

Heterogeneity: Tau² = 0.53; Chi² = 50.55, df = 3 (P < 0.00001); I² = 94%
Test for overall effect: Z = 1.63 (P = 0.10)

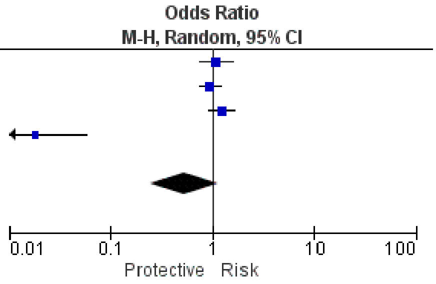

Supplementary Figure S2d: Forest plot - *ACE* I/D investigation, individuals with chronic kidney disease compared to healthy controls in a South Asian population (*ACE* insertion compared to deletion).

For peer review only

Study or Subgroup	Case Events	Case Total	Control Events	Control Total	Weight	Odds Ratio M-H, Random, 95% CI
Aucella 2003	315	922	916	2614	15.6%	0.96 [0.82, 1.13]
Buraczynska 2006	688	1490	492	1040	15.6%	0.96 [0.82, 1.12]
Dixit 2002	32	52	17	44	2.4%	2.54 [1.11, 5.80]
Losito 2002	135	320	128	338	9.7%	1.20 [0.88, 1.64]
McLaughlin 1996	654	1644	353	742	14.9%	0.73 [0.61, 0.87]
Nicod 2002	282	520	261	520	12.1%	1.18 [0.92, 1.50]
Ortiz 2003	68	234	80	258	7.7%	0.91 [0.62, 1.34]
Schmidt 1996	105	212	80	190	7.5%	1.35 [0.91, 2.00]
van der Sman-de Beer 2005	400	830	459	944	14.4%	0.98 [0.82, 1.18]
**Total (95% CI)**		**6224**		**6690**	**100.0%**	**1.01 [0.88, 1.16]**
Total events	2679		2786			

Heterogeneity: Tau² = 0.02; Chi² = 22.57, df = 8 (P = 0.004); I² = 65%
Test for overall effect: Z = 0.17 (P = 0.86)

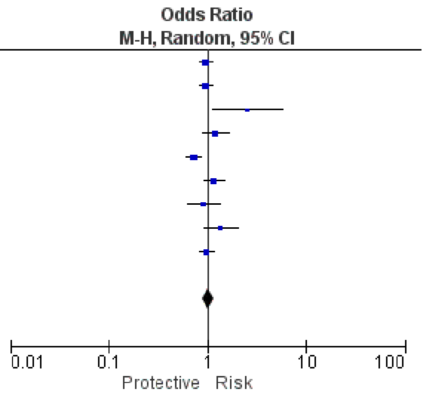

Supplementary Figure S2e: Forest plot - *ACE* I/D investigation, individuals with end-stage renal disease compared to healthy controls in a European population (*ACE* insertion compared to deletion).

Study or Subgroup	Protective Events	Protective Total	Risk Events	Risk Total	Weight	Odds Ratio M-H, Random, 95% CI
Abuaisha 2018	37	172	50	200	34.8%	0.82 [0.51, 1.33]
Rahimi 2017	64	190	89	234	50.9%	0.83 [0.55, 1.23]
Zaare Nahandi 2017	23	60	21	54	14.3%	0.98 [0.46, 2.08]
**Total (95% CI)**		**422**		**488**	**100.0%**	**0.85 [0.64, 1.13]**
Total events	124		160			

Heterogeneity: Tau² = 0.00; Chi² = 0.16, df = 2 (P = 0.92); I² = 0%
Test for overall effect: Z = 1.15 (P = 0.25)

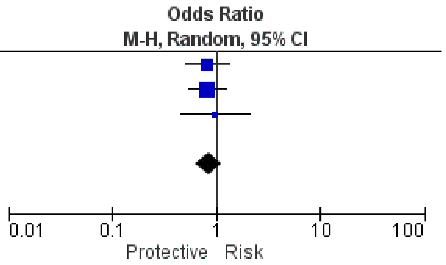

Supplementary Figure S2f: Forest plot - *ACE* I/D investigation, individuals with end-stage renal disease compared to healthy controls in a Middle Eastern population (*ACE* insertion compared to deletion).

For peer review only

Study or Subgroup	Case Events	Case Total	Control Events	Control Total	Weight	Odds Ratio M-H, Random, 95% CI
Doi 1996	115	200	168	248	20.6%	0.64 [0.44, 0.95]
Jayapalan 2010	163	254	93	162	20.1%	1.33 [0.89, 1.99]
Lu 2016	238	420	307	444	24.0%	0.58 [0.44, 0.77]
Park 2005	103	206	111	176	19.9%	0.59 [0.39, 0.88]
Taniwaki 2001	53	84	88	138	15.5%	0.97 [0.55, 1.71]
**Total (95% CI)**		**1164**		**1168**	**100.0%**	**0.76 [0.55, 1.05]**
Total events	672		767			

Heterogeneity: Tau² = 0.09; Chi² = 13.42, df = 4 (P = 0.009); I² = 70%

Test for overall effect: Z = 1.66 (P = 0.10)

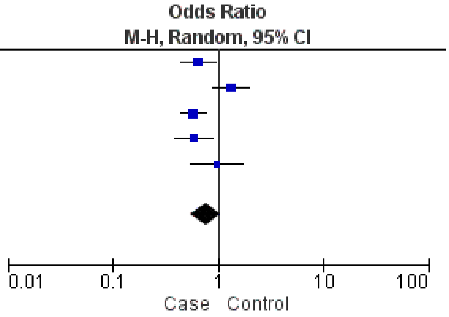

Supplementary Figure S2g: Forest plot - *ACE* I/D investigation, individuals with end-stage renal disease linked to type 2 diabetic nephropathy compared to individuals with type 2 diabetes mellitus in an East Asian population (*ACE* insertion compared to deletion).

Study or Subgroup	Case Events	Case Total	Control Events	Control Total	Weight	Odds Ratio M-H, Random, 95% CI
Grzeszczak 1998	128	254	244	508	33.9%	1.10 [0.81, 1.49]
Ringel 1997	150	322	141	280	33.7%	0.86 [0.62, 1.18]
Schmidt 1997 A	29	122	416	694	32.4%	0.21 [0.13, 0.32]
**Total (95% CI)**		**698**		**1482**	**100.0%**	**0.59 [0.24, 1.44]**
Total events	307		801			

Heterogeneity: Tau² = 0.58; Chi² = 38.91, df = 2 (P < 0.00001); I² = 95%

Test for overall effect: Z = 1.16 (P = 0.25)

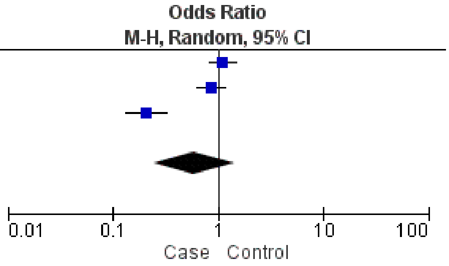

Supplementary Figure S2h: Forest plot - *ACE* I/D investigation, individuals with end-stage renal disease linked to type 2 diabetic nephropathy compared to individuals with type 2 diabetes mellitus in a European population (*ACE* insertion compared to deletion).

For peer review only

Study or Subgroup	Case Events	Case Total	Control Events	Control Total	Weight	Odds Ratio M-H, Random, 95% CI
Guo 2016	54	90	42	90	6.5%	1.71 [0.95, 3.10]
Huang 2010	155	260	168	240	12.5%	0.63 [0.44, 0.92]
Jung 2010	298	522	360	600	19.2%	0.89 [0.70, 1.13]
Lau 2004	154	236	137	188	10.7%	0.70 [0.46, 1.06]
Suzuki 2004	378	638	328	540	19.5%	0.94 [0.74, 1.19]
Yoon 2002	204	382	268	466	17.2%	0.85 [0.64, 1.11]
Yorioka 1995	67	96	139	206	7.8%	1.11 [0.66, 1.88]
Yoshida 1995	57	106	62	92	6.7%	0.56 [0.32, 1.00]
**Total (95% CI)**		**2330**		**2422**	**100.0%**	**0.86 [0.72, 1.02]**
Total events	1367		1504			

Heterogeneity: Tau² = 0.02; Chi² = 12.42, df = 7 (P = 0.09); I² = 44%

Test for overall effect: Z = 1.79 (P = 0.07)

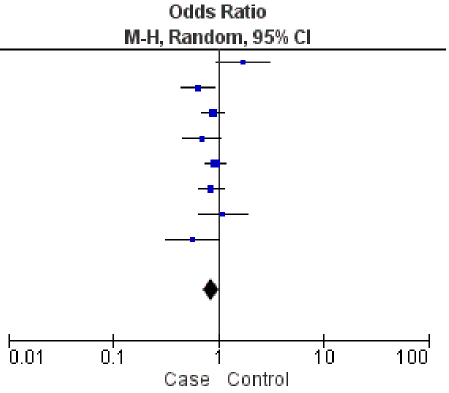

Supplementary Figure S2i: Forest plot - *ACE* I/D investigation, individuals with IgA nephropathy compared to healthy controls in an East Asian population (*ACE* insertion compared to deletion).

Study or Subgroup	Case Events	Case Total	Control Events	Control Total	Weight	Odds Ratio M-H, Random, 95% CI
Burg 1997	26	54	60	120	5.6%	0.93 [0.49, 1.77]
Drouet 2002	78	250	54	154	12.8%	0.84 [0.55, 1.29]
Harden 1995	79	200	76	196	14.3%	1.03 [0.69, 1.54]
Pawlik 2014	25	62	180	374	7.8%	0.73 [0.42, 1.26]
Pei 1997	145	336	91	200	18.7%	0.91 [0.64, 1.29]
Schmidt 1995 A	168	408	197	468	32.0%	0.96 [0.74, 1.26]
Stratta 1999	62	162	38	100	8.8%	1.01 [0.61, 1.69]
**Total (95% CI)**		**1472**		**1612**	**100.0%**	**0.93 [0.80, 1.08]**
Total events	583		696			

Heterogeneity: Tau² = 0.00; Chi² = 1.42, df = 6 (P = 0.96); I² = 0%

Test for overall effect: Z = 0.97 (P = 0.33)

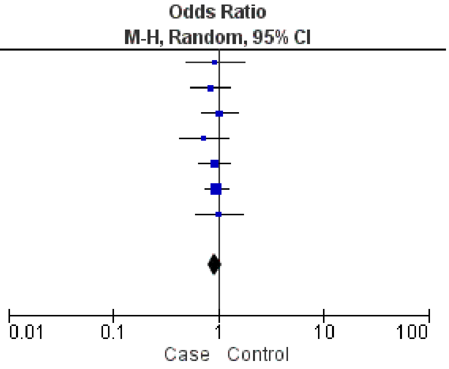

Supplementary Figure S2j: Forest plot - *ACE* I/D investigation, individuals with IgA nephropathy compared to healthy controls in a European population (*ACE* insertion compared to deletion).

For peer review only

Study or Subgroup	Case Events	Case Total	Control Events	Control Total	Weight	Odds Ratio M-H, Random, 95% CI
Burg 1997	44	92	60	120	12.2%	0.92 [0.53, 1.58]
Pawlik 2014	101	218	180	374	32.1%	0.93 [0.67, 1.30]
Stratta 2004	96	234	134	342	31.2%	1.08 [0.77, 1.52]
Zsom 2011	62	146	198	400	24.6%	0.75 [0.51, 1.10]
**Total (95% CI)**		**690**		**1236**	**100.0%**	**0.92 [0.76, 1.12]**
Total events	303		572			

Heterogeneity: Tau² = 0.00; Chi² = 1.91, df = 3 (P = 0.59); I² = 0%
Test for overall effect: Z = 0.82 (P = 0.41)

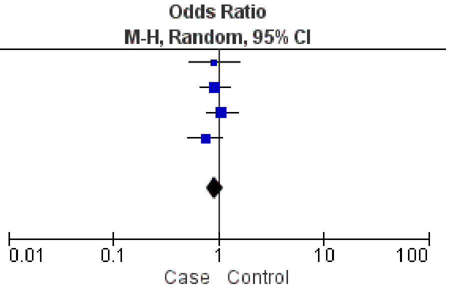

Supplementary Figure S2k: Forest plot - *ACE* I/D investigation, individuals with primary glomerulonephritis compared to healthy controls in a European population (*ACE* insertion compared to deletion).

Study or Subgroup	Case Events	Case Total	Control Events	Control Total	Weight	Odds Ratio M-H, Random, 95% CI
Beige 1997	251	538	244	538	27.2%	1.05 [0.83, 1.34]
El-Essawy 2002	246	588	170	362	24.7%	0.81 [0.62, 1.06]
Hueso 2004	122	360	96	226	18.2%	0.69 [0.49, 0.98]
Stratta 2008	122	338	112	338	20.0%	1.14 [0.83, 1.57]
Viklický 2001	28	60	599	1306	9.9%	1.03 [0.61, 1.73]
**Total (95% CI)**		**1884**		**2770**	**100.0%**	**0.93 [0.77, 1.11]**
Total events	769		1221			

Heterogeneity: Tau² = 0.02; Chi² = 6.59, df = 4 (P = 0.16); I² = 39%
Test for overall effect: Z = 0.80 (P = 0.42)

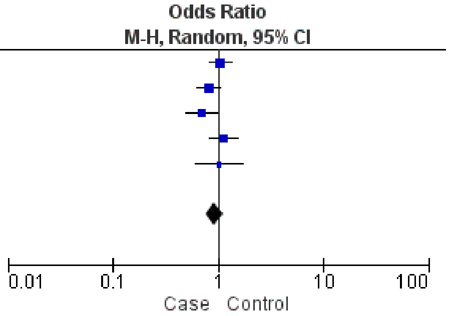

Supplementary Figure S2l: Forest plot - *ACE* I/D investigation, individuals who have had renal transplants compared to healthy controls in a European population (*ACE* insertion compared to deletion).

For peer review only

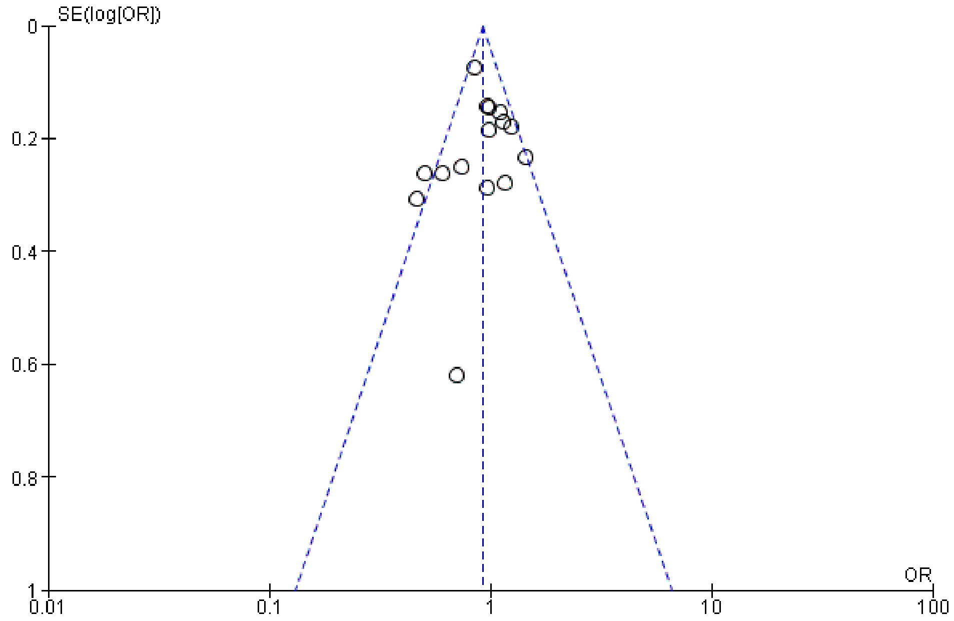

Supplementary Figure S3a: Funnel plot - *ACE* I/D investigation, individuals with type 1 diabetic nephropathy compared to individuals with type 1 diabetes mellitus in a European population (*ACE* insertion compared to deletion).

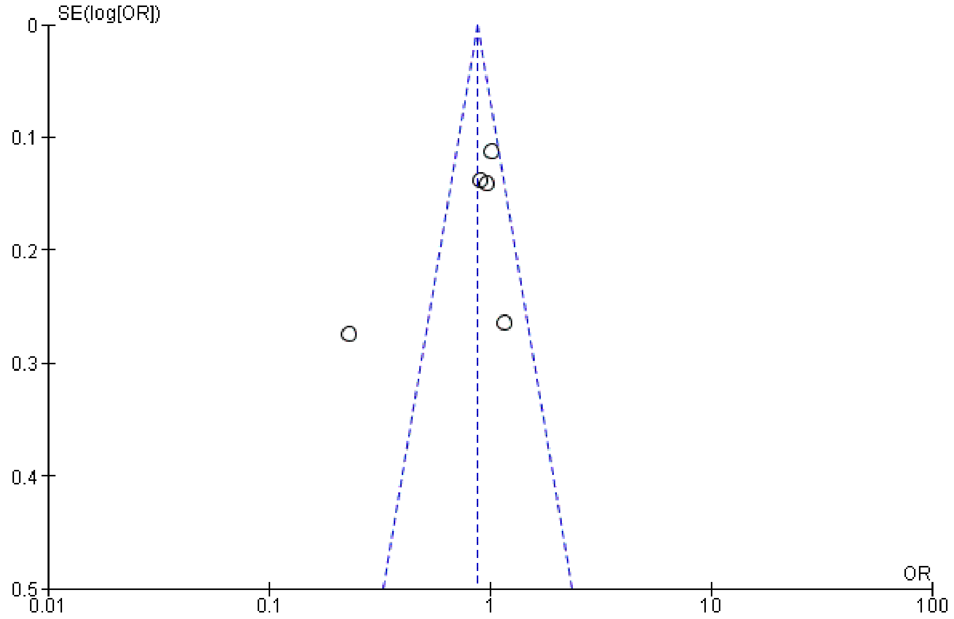

Supplementary Figure S3b: Funnel plot - *ACE* I/D investigation, individuals with type 2 diabetic nephropathy compared to individuals with type 2 diabetes mellitus in a European population (*ACE* insertion compared to deletion).

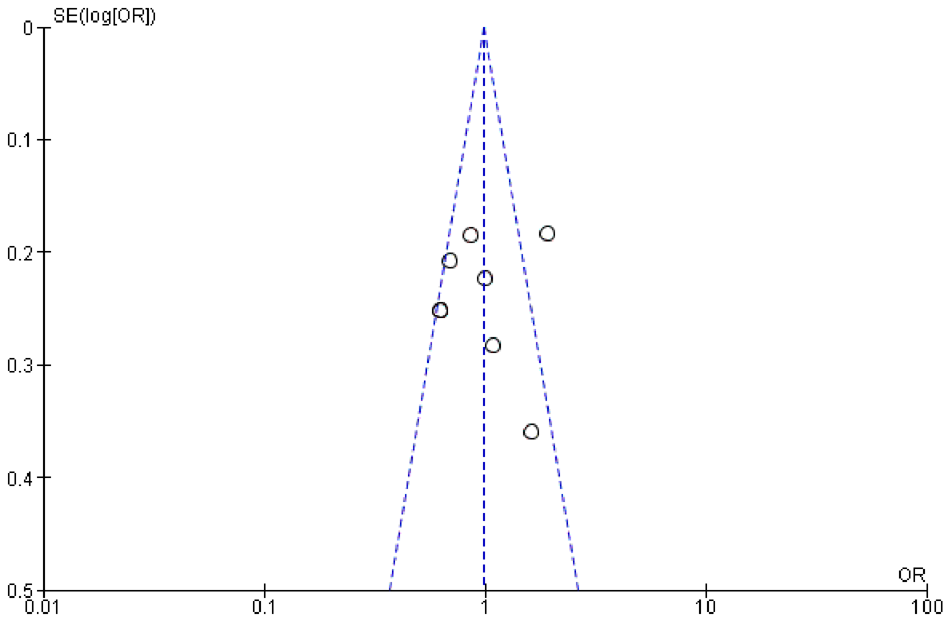

Supplementary Figure S3c: Funnel plot - *ACE* I/D investigation, individuals with type 2 diabetic nephropathy compared to individuals with type 2 diabetes mellitus in a Middle Eastern population (*ACE* insertion compared to deletion).

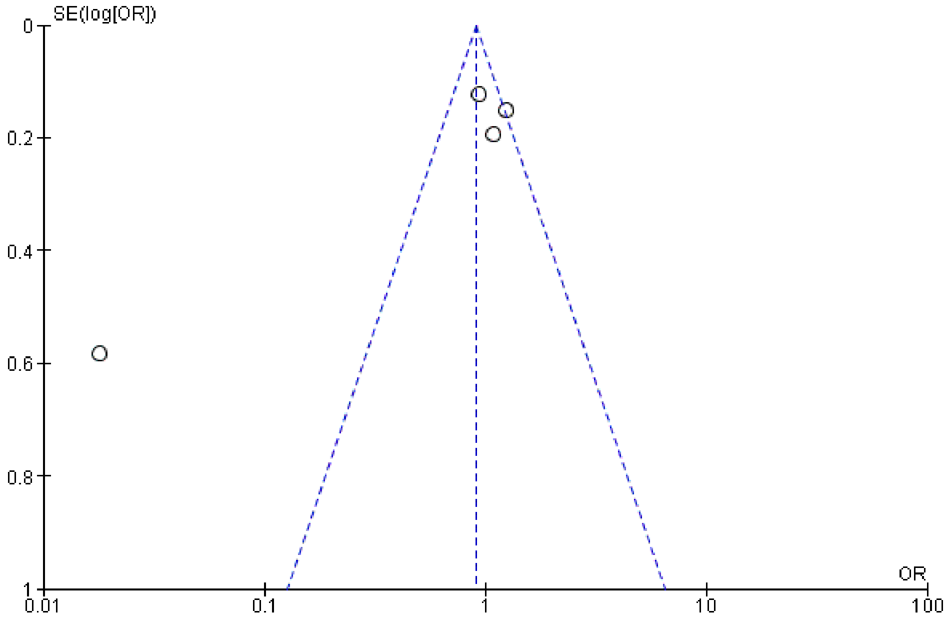

Supplementary Figure S3d: Funnel plot - *ACE* I/D investigation, individuals with chronic kidney disease compared to healthy controls in a South Asian population (*ACE* insertion compared to deletion).

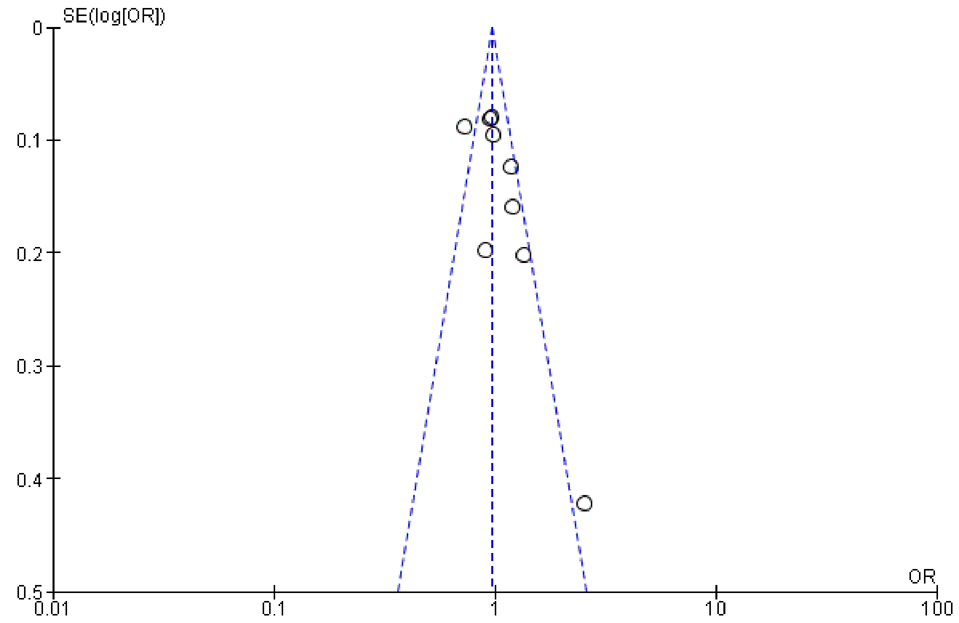

Supplementary Figure S3e: Funnel plot - *ACE* I/D investigation, individuals with end-stage renal disease compared to healthy controls in a European population (*ACE* insertion compared to deletion).

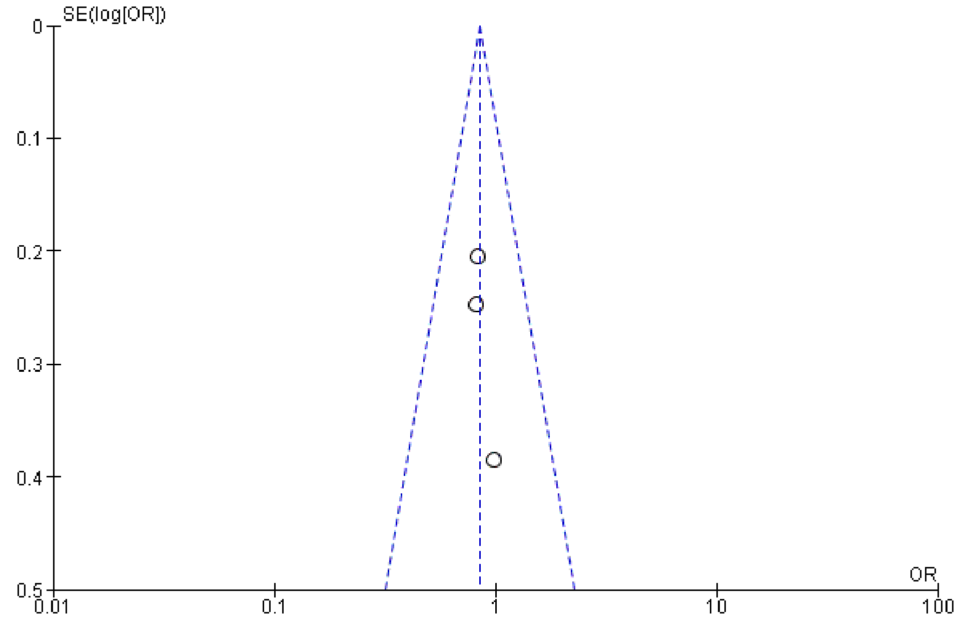

Supplementary Figure S3f: Funnel plot - *ACE* I/D investigation, individuals with end-stage renal disease compared to healthy controls in a Middle Eastern population (*ACE* insertion compared to deletion).

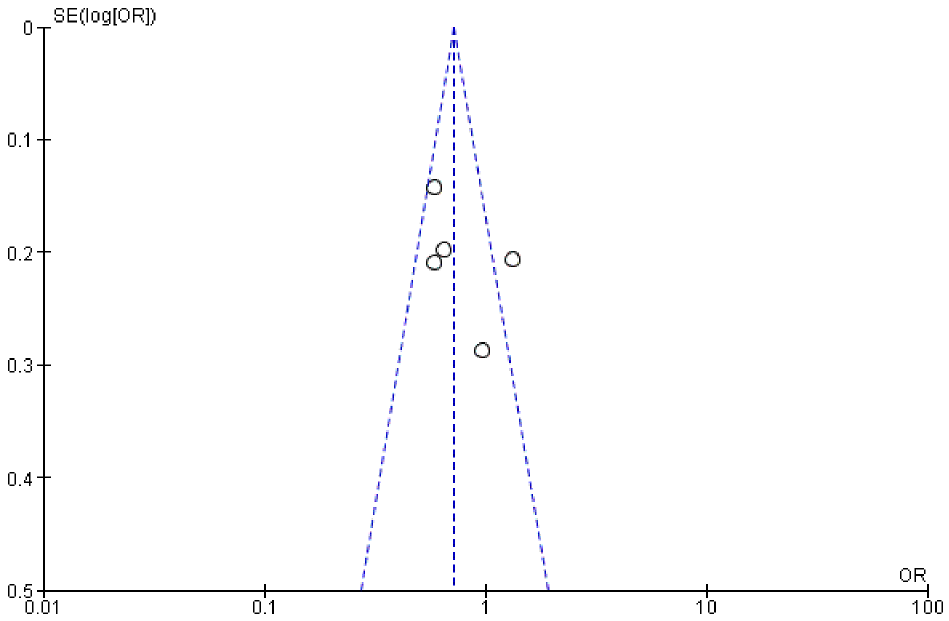

Supplementary Figure S3g: Funnel plot - *ACE* I/D investigation, individuals with end-stage renal disease linked to type 2 diabetic nephropathy compared to individuals with type 2 diabetes mellitus in an East Asian population (*ACE* insertion compared to deletion).

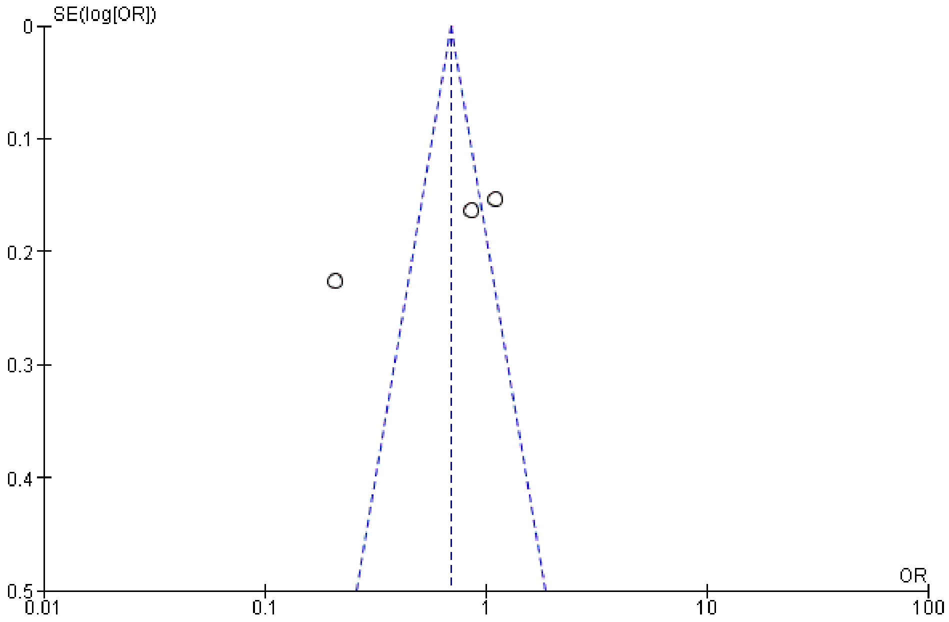

Supplementary Figure S3h: Funnel plot - *ACE* I/D investigation, individuals with end-stage renal disease linked to type 2 diabetic nephropathy compared to individuals with type 2 diabetes mellitus in a European population (*ACE* insertion compared to deletion).

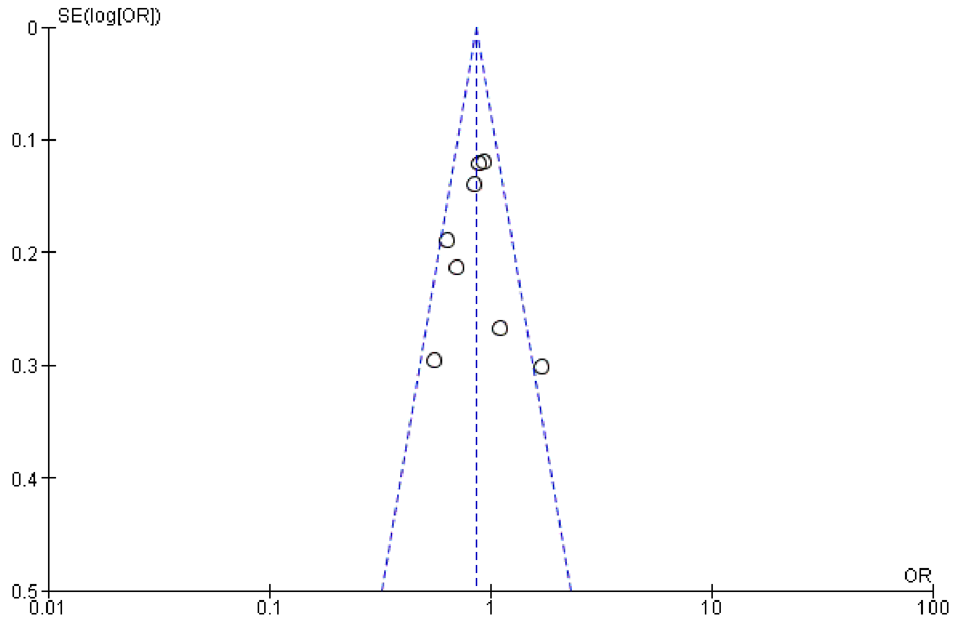

Supplementary Figure S3i: Funnel plot - *ACE* I/D investigation, individuals with IgA nephropathy compared to healthy controls in an East Asian population (*ACE* insertion compared to deletion).

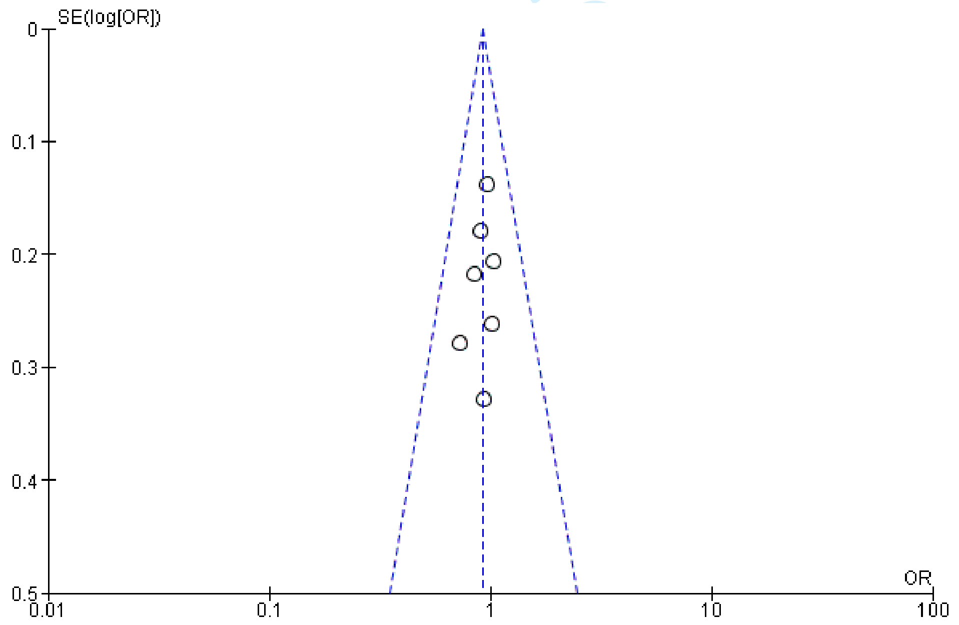

Supplementary Figure S3j: Funnel plot - *ACE* I/D investigation, individuals with IgA nephropathy compared to healthy controls in a European population (*ACE* insertion compared to deletion).

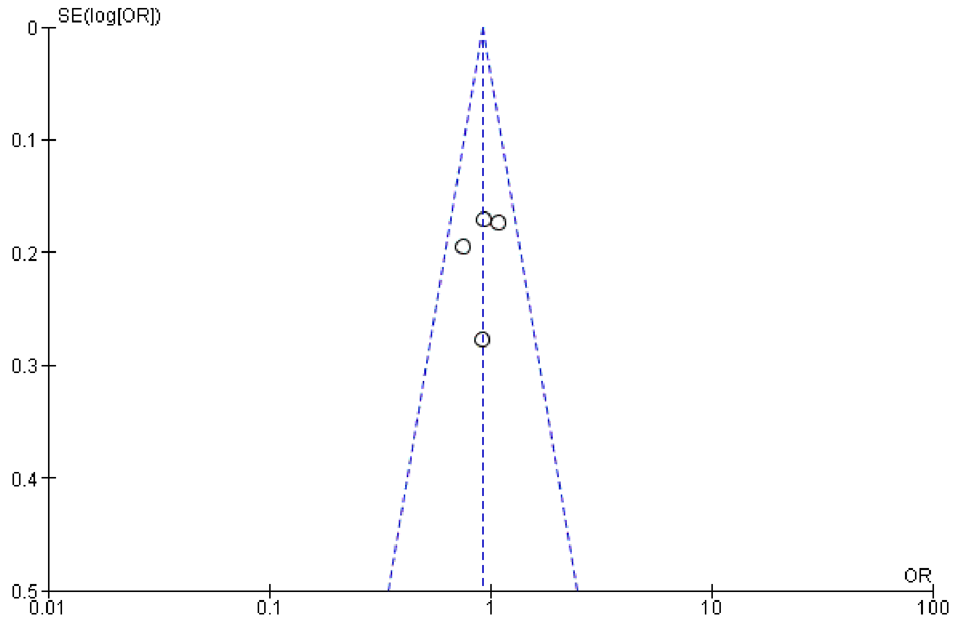

Supplementary Figure S3k: Funnel plot - *ACE* I/D investigation, individuals with primary glomerulonephritis compared to healthy controls in a European population (*ACE* insertion compared to deletion).

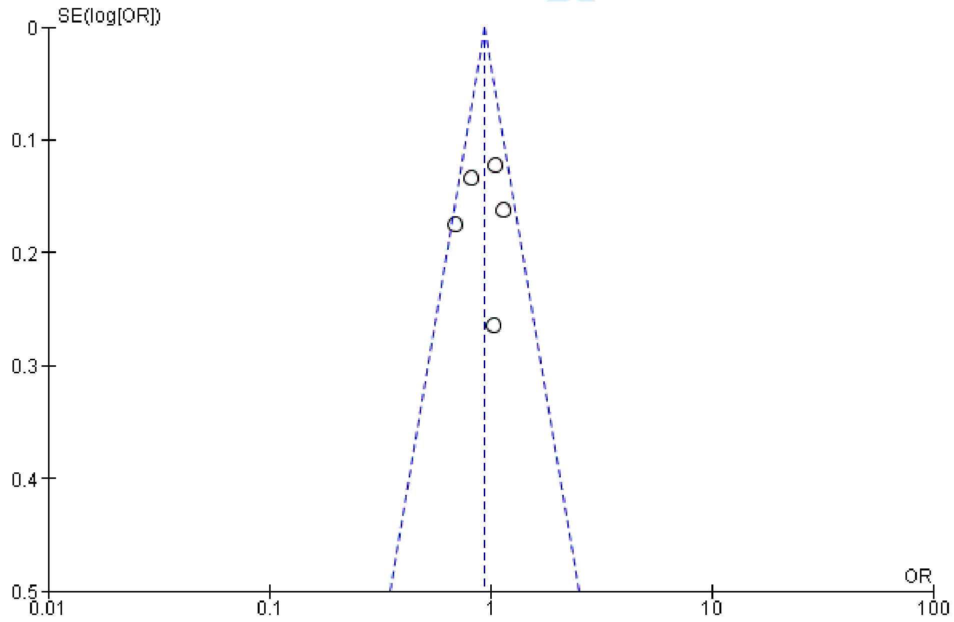

Supplementary Figure S3l: Funnel plot - *ACE* I/D investigation, individuals who have had renal transplants compared to healthy controls in a European population (*ACE* insertion compared to deletion).

Study or Subgroup	Case Events	Case Total	Control Events	Control Total	Weight	Odds Ratio M-H, Random, 95% CI
Doria 1996	158	278	96	150	15.1%	0.74 [0.49, 1.12]
Fogarty 1996	114	190	129	200	15.1%	0.83 [0.55, 1.24]
Möllsten 2008	62	96	220	394	13.2%	1.44 [0.91, 2.29]
Ringel 1997	145	268	257	452	19.5%	0.89 [0.66, 1.21]
Schmidt 1996	121	216	132	240	16.6%	1.04 [0.72, 1.51]
van Ittersum 2000	31	60	238	376	10.8%	0.62 [0.36, 1.07]
Walder 1998	60	110	63	88	9.6%	0.48 [0.26, 0.86]
**Total (95% CI)**		**1218**		**1900**	**100.0%**	**0.85 [0.68, 1.06]**
Total events	691		1135			

Heterogeneity: Tau² = 0.04; Chi² = 11.57, df = 6 (P = 0.07); I² = 48%
Test for overall effect: Z = 1.43 (P = 0.15)

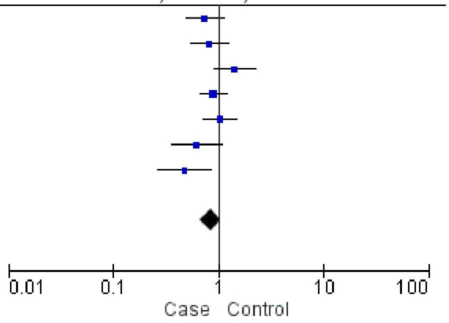

Supplementary Figure S4a: Forest plot - *AGT* rs699 investigation, individuals with type 1 diabetic nephropathy compared to individuals with type 1 diabetes mellitus in a European population (*AGT* rs699 T allele compared to C allele).

Study or Subgroup	Case Events	Case Total	Control Events	Control Total	Weight	Odds Ratio M-H, Random, 95% CI
Ohno 1996	16	50	25	106	16.8%	1.52 [0.72, 3.21]
Oue 1999	6	42	13	60	11.8%	0.60 [0.21, 1.74]
Thomas 2001	18	102	79	510	20.4%	1.17 [0.67, 2.05]
Wang 2016	19	194	154	946	21.6%	0.56 [0.34, 0.92]
Wu 2000	21	142	26	82	18.5%	0.37 [0.19, 0.72]
Young 1998	4	40	25	108	11.0%	0.37 [0.12, 1.14]
**Total (95% CI)**		**570**		**1812**	**100.0%**	**0.69 [0.43, 1.11]**
Total events	84		322			

Heterogeneity: Tau² = 0.21; Chi² = 13.02, df = 5 (P = 0.02); I² = 62%
Test for overall effect: Z = 1.54 (P = 0.12)

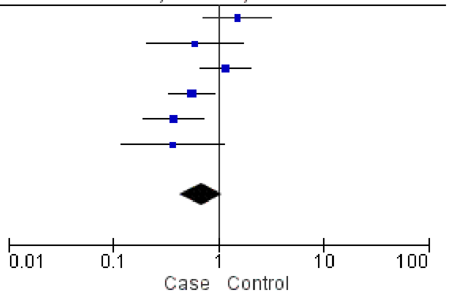

Supplementary Figure S4b: Forest plot - *AGT* rs699 investigation, individuals with type 2 diabetic nephropathy compared to individuals with type 2 diabetes mellitus in an East Asian population (*AGT* rs699 T allele compared to C allele).

Study or Subgroup	Case Events	Case Total	Control Events	Control Total	Weight	Odds Ratio M-H, Random, 95% CI
Fradin 2002	45	78	139	236	7.5%	0.95 [0.57, 1.60]
Freire 1998	134	230	148	236	14.5%	0.83 [0.57, 1.20]
Makuc 2017	294	552	394	750	41.4%	1.03 [0.83, 1.28]
Schmidt 1996	149	254	117	214	14.9%	1.18 [0.81, 1.70]
Zychma 2000	133	254	242	486	21.8%	1.11 [0.82, 1.50]
**Total (95% CI)**		**1368**		**1922**	**100.0%**	**1.03 [0.89, 1.18]**
Total events	755		1040			

Heterogeneity: Tau² = 0.00; Chi² = 2.11, df = 4 (P = 0.72); I² = 0%
Test for overall effect: Z = 0.39 (P = 0.70)

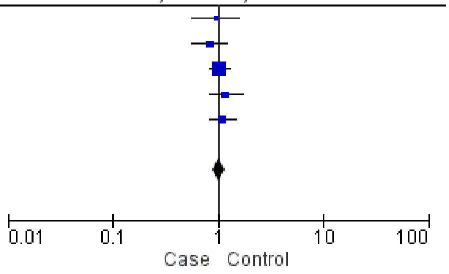

Supplementary Figure S4c: Forest plot - *AGT* rs699 investigation, individuals with type 2 diabetic nephropathy compared to individuals with type 2 diabetes mellitus in a European population (*AGT* rs699 T allele compared to C allele).

For peer review only

Study or Subgroup	Case Events	Case Total	Control Events	Control Total	Weight	Odds Ratio M-H, Random, 95% CI
Eroglu 2008	48	92	62	112	25.1%	0.88 [0.51, 1.53]
Reis 2011	114	216	89	222	36.0%	1.67 [1.14, 2.44]
Sancakdar 2015	189	388	95	200	38.8%	1.05 [0.75, 1.48]
**Total (95% CI)**		**696**		**534**	**100.0%**	**1.19 [0.82, 1.71]**
Total events	351		246			

Heterogeneity: Tau² = 0.06; Chi² = 4.72, df = 2 (P = 0.09); I² = 58%
Test for overall effect: Z = 0.91 (P = 0.36)

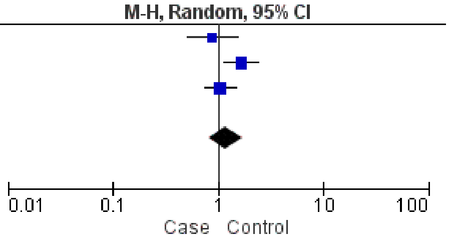

Supplementary Figure S4d: Forest plot - *AGT* rs699 investigation, individuals with type 2 diabetic nephropathy compared to individuals with type 2 diabetes mellitus in a Middle Eastern population (*AGT* rs699 T allele compared to C allele).

Study or Subgroup	Protective Events	Protective Total	Risk Events	Risk Total	Weight	Odds Ratio M-H, Random, 95% CI
Kawada 1997	91	424	64	404	31.1%	1.45 [1.02, 2.07]
Su 2017	194	1268	279	1478	38.3%	0.78 [0.63, 0.95]
Wang 2003	76	492	64	366	30.6%	0.86 [0.60, 1.24]
**Total (95% CI)**		**2184**		**2248**	**100.0%**	**0.97 [0.67, 1.41]**
Total events	361		407			

Heterogeneity: Tau² = 0.08; Chi² = 9.16, df = 2 (P = 0.01); I² = 78%
Test for overall effect: Z = 0.14 (P = 0.89)

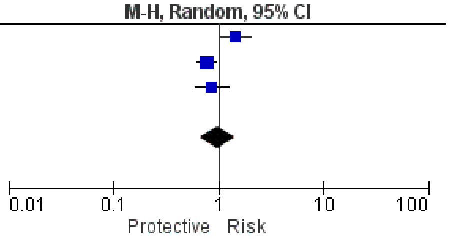

Supplementary Figure S4e: Forest plot - *AGT* rs699 investigation, individuals with end-stage renal disease compared to healthy controls in an East Asian population (*AGT* rs699 T allele compared to C allele).

Study or Subgroup	Case Events	Case Total	Control Events	Control Total	Weight	Odds Ratio M-H, Random, 95% CI
Gao 2015	144	700	125	620	41.2%	1.03 [0.78, 1.34]
Guo 2016	19	90	16	90	5.4%	1.24 [0.59, 2.60]
Huang 2010	35	260	40	240	12.3%	0.78 [0.48, 1.27]
Kim 2009	89	476	115	600	31.5%	0.97 [0.71, 1.32]
Lau 2004	27	236	31	188	9.6%	0.65 [0.38, 1.14]
**Total (95% CI)**		**1762**		**1738**	**100.0%**	**0.94 [0.79, 1.12]**
Total events	314		327			

Heterogeneity: Tau² = 0.00; Chi² = 3.18, df = 4 (P = 0.53); I² = 0%
Test for overall effect: Z = 0.67 (P = 0.50)

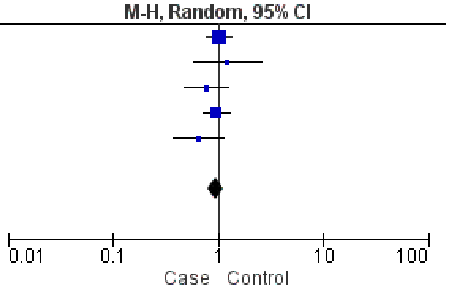

Supplementary Figure S4f: Forest plot - *AGT* rs699 investigation, individuals with IgA nephropathy compared to healthy controls in an East Asian population (*AGT* rs699 T allele compared to C allele).

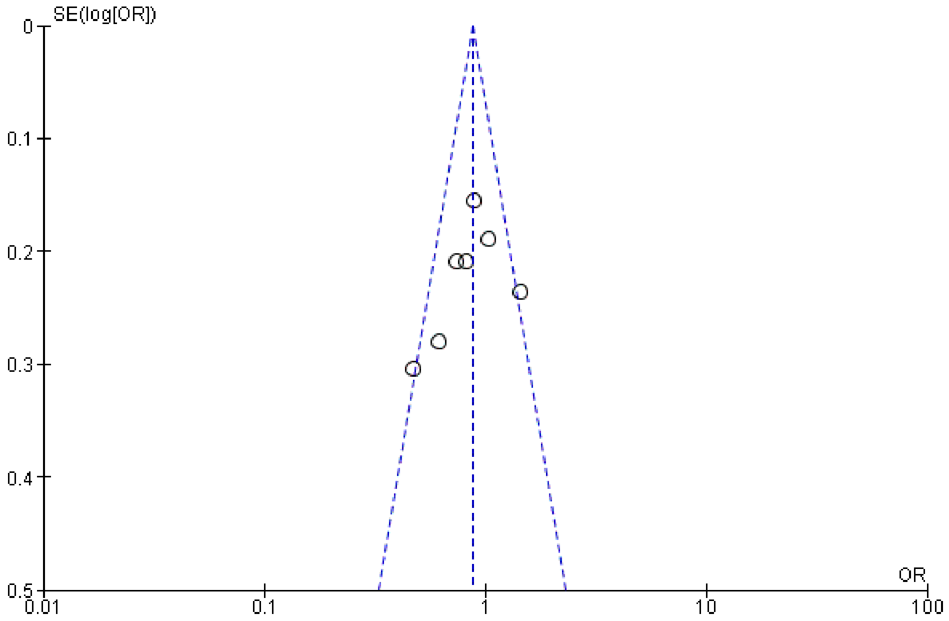

Supplementary Figure S5a: Funnel plot - *AGT* rs699 investigation, individuals with type 1 diabetic nephropathy compared to individuals with type 1 diabetes mellitus in a European population (*AGT* rs699 T allele compared to C allele).

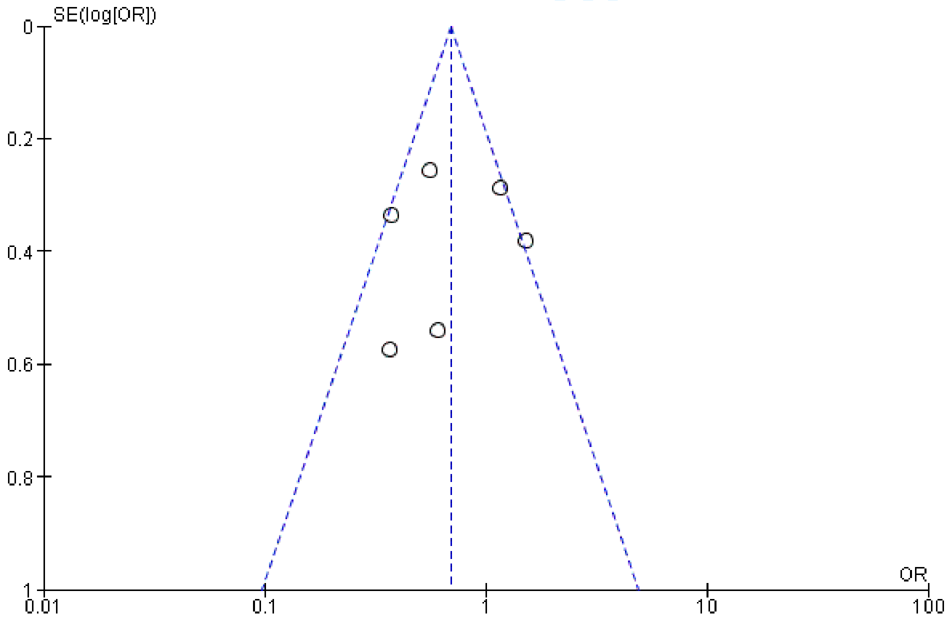

Supplementary Figure S5b: Funnel plot - *AGT* rs699 investigation, individuals with type 2 diabetic nephropathy compared to individuals with type 2 diabetes mellitus in an East Asian population (*AGT* rs699 T allele compared to C allele).

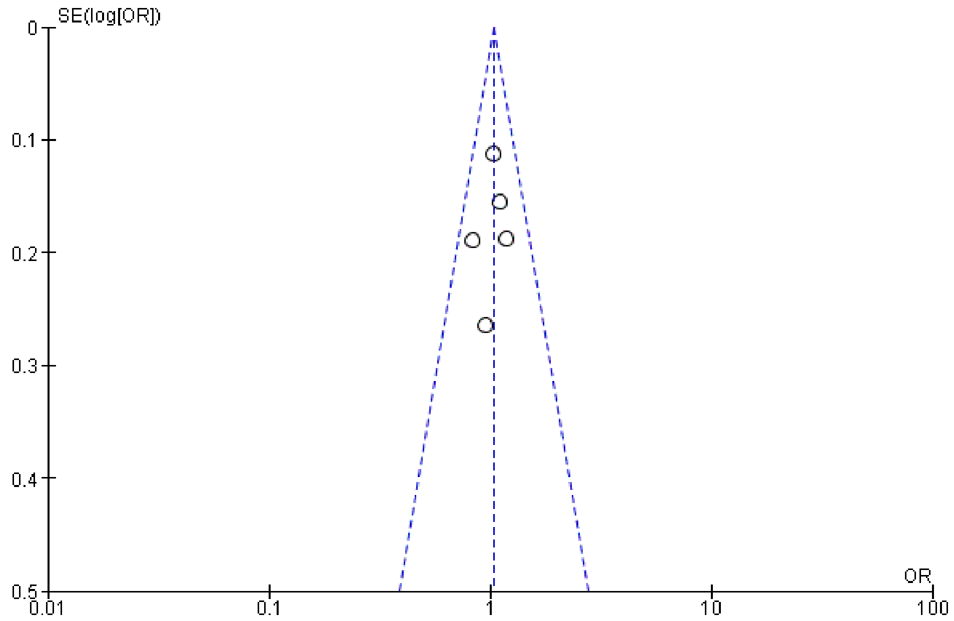

Supplementary Figure S5c: Funnel plot - *AGT* rs699 investigation, individuals with type 2 diabetic nephropathy compared to individuals with type 2 diabetes mellitus in a European population (*AGT* rs699 T allele compared to C allele).

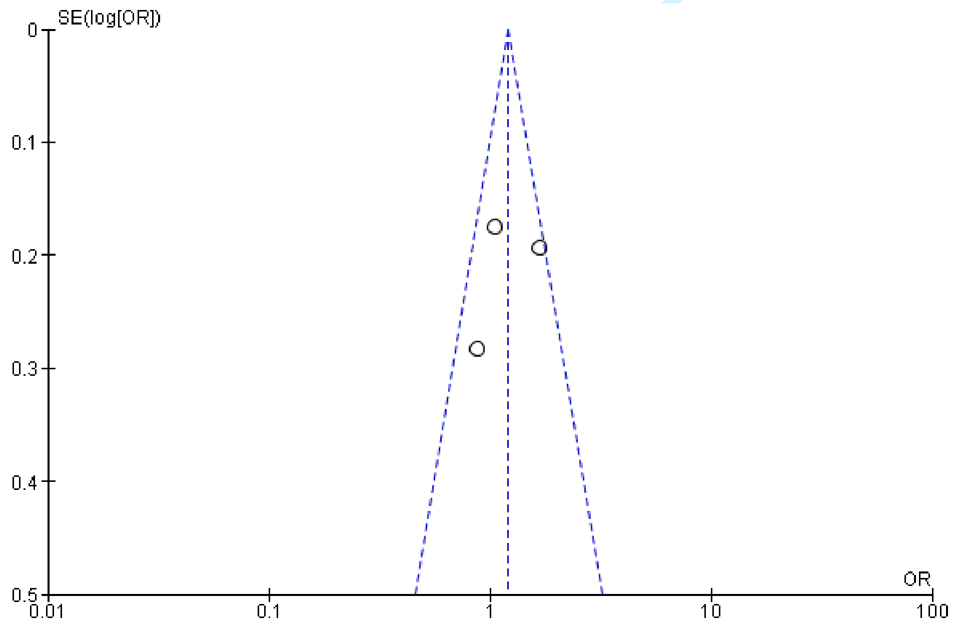

Supplementary Figure S5d: Funnel plot - *AGT* rs699 investigation, individuals with type 2 diabetic nephropathy compared to individuals with type 2 diabetes mellitus in a Middle Eastern population (*AGT* rs699 T allele compared to C allele).

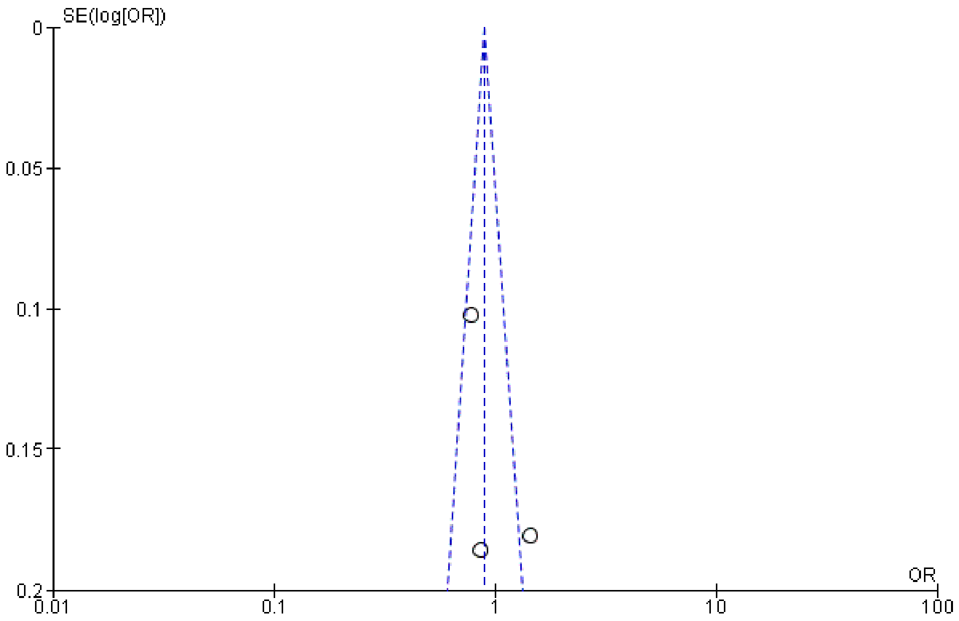

Supplementary Figure S5e: Funnel plot - *AGT* rs699 investigation, individuals with end-stage renal disease compared to healthy controls in an East Asian population (*AGT* rs699 T allele compared to C allele).

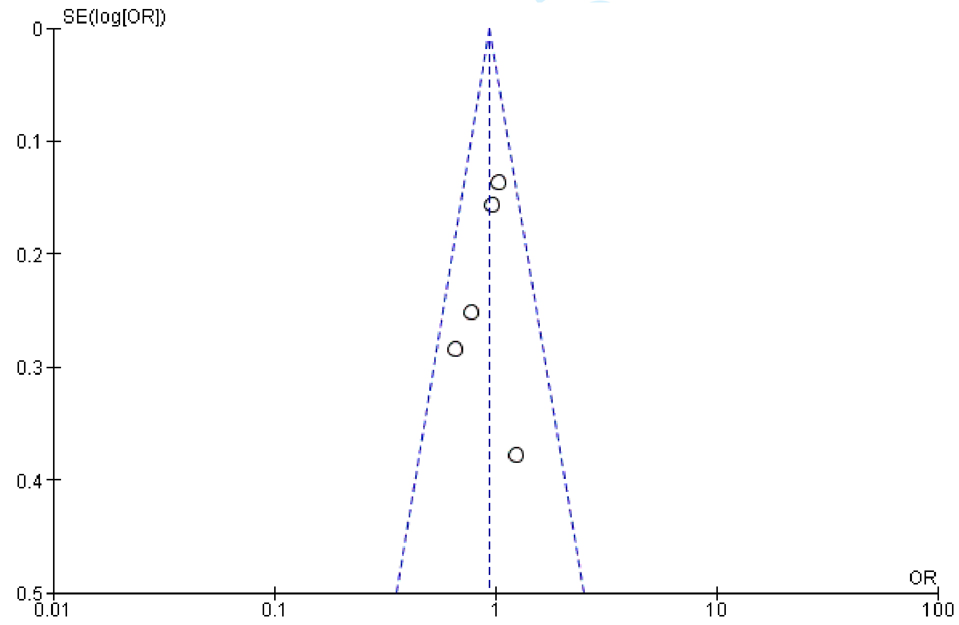

Supplementary Figure S5f: Funnel plot - *AGT* rs699 investigation, individuals with IgA nephropathy compared to healthy controls in an East Asian population (*AGT* rs699 T allele compared to C allele).

Study or Subgroup	Case Events	Case Total	Control Events	Control Total	Weight	Odds Ratio M-H, Random, 95% CI
Currie 2010	1029	1414	1052	1470	33.2%	1.06 [0.90, 1.25]
Doria 1997	99	146	119	158	12.2%	0.69 [0.42, 1.14]
Möllsten 2008	78	96	290	394	10.4%	1.55 [0.89, 2.72]
Savage 1999	136	190	137	194	14.5%	1.05 [0.67, 1.63]
Tarnow 1996	287	396	274	380	21.4%	1.02 [0.74, 1.40]
van Ittersum 2000	47	60	238	376	8.3%	2.10 [1.10, 4.01]
**Total (95% CI)**		**2302**		**2972**	**100.0%**	**1.10 [0.89, 1.35]**
Total events	1676		2110			

Heterogeneity: Tau² = 0.03; Chi² = 8.89, df = 5 (P = 0.11); I² = 44%
Test for overall effect: Z = 0.87 (P = 0.38)

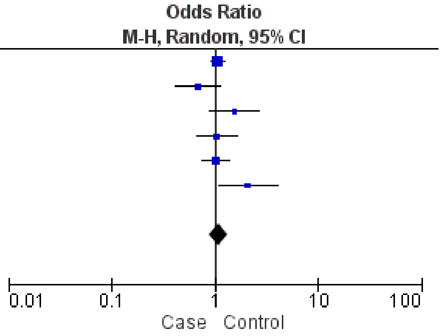

Supplementary Figure S6a: Forest plot - *AGTR1* rs5186 investigation, individuals with type 1 diabetic nephropathy compared to individuals with type 1 diabetes mellitus in a European population (*AGTR1* rs5186 A allele compared to C allele).

Study or Subgroup	Case Events	Case Total	Control Events	Control Total	Weight	Odds Ratio M-H, Random, 95% CI
Thomas 2001	99	102	483	510	30.2%	1.84 [0.55, 6.20]
Wu 2000	127	142	74	82	54.2%	0.92 [0.37, 2.26]
Young 1998	38	40	103	108	15.7%	0.92 [0.17, 4.96]
**Total (95% CI)**		**284**		**700**	**100.0%**	**1.13 [0.58, 2.20]**
Total events	264		660			

Heterogeneity: Tau² = 0.00; Chi² = 0.90, df = 2 (P = 0.64); I² = 0%
Test for overall effect: Z = 0.37 (P = 0.71)

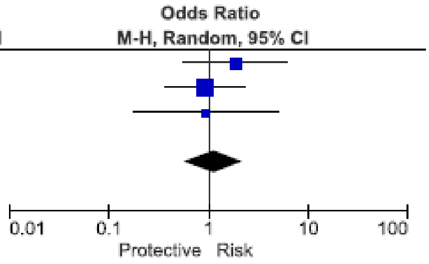

Supplementary Figure S6b: Forest plot - *AGTR1* rs5186 investigation, individuals with type 2 diabetic nephropathy compared to individuals with type 2 diabetes mellitus in an East Asian population (*AGTR1* rs5186 A allele compared to C allele).

Study or Subgroup	Case Events	Case Total	Control Events	Control Total	Weight	Odds Ratio M-H, Random, 95% CI
Gao 2015	659	702	577	620	45.1%	1.14 [0.74, 1.77]
Huang 2010	243	260	220	240	19.1%	1.30 [0.66, 2.54]
Kim 2009	452	476	576	600	25.7%	0.78 [0.44, 1.40]
Lau 2004	227	236	178	188	10.1%	1.42 [0.56, 3.56]
**Total (95% CI)**		**1674**		**1648**	**100.0%**	**1.09 [0.81, 1.46]**
Total events	1581		1551			

Heterogeneity: Tau² = 0.00; Chi² = 1.85, df = 3 (P = 0.60); I² = 0%
Test for overall effect: Z = 0.55 (P = 0.58)

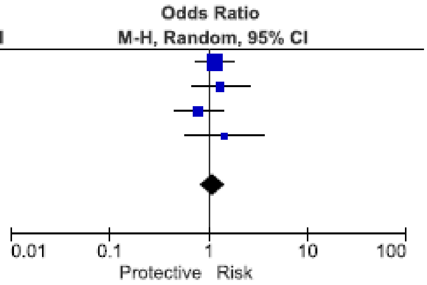

Supplementary Figure S6c: Forest plot - *AGTR1* rs5186 investigation, individuals with IgA nephropathy compared to healthy controls in an East Asian population (*AGTR1* rs5186 A allele compared to C allele).

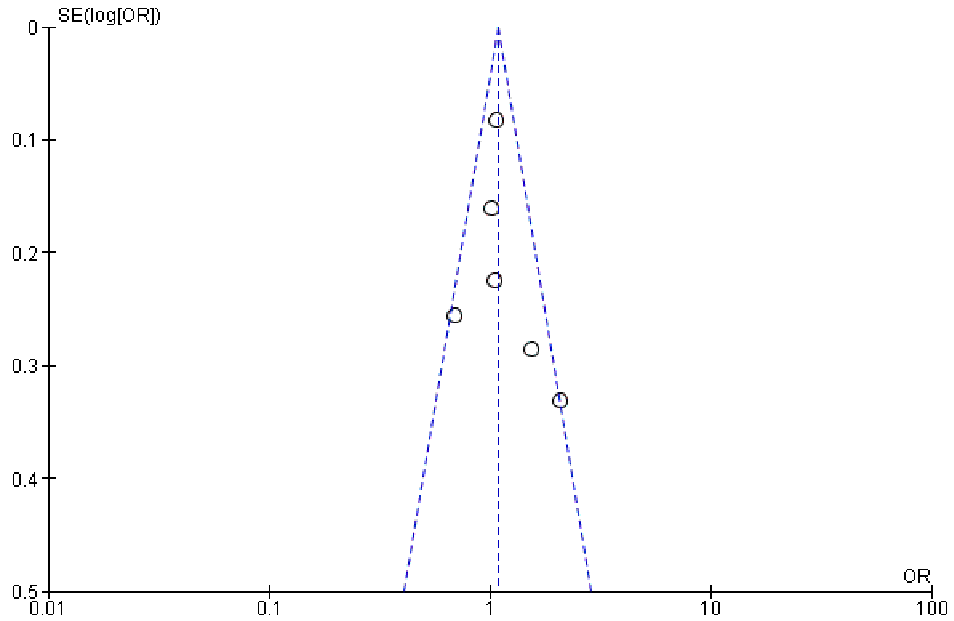

Supplementary Figure S7a: Funnel plot - *AGTR1* rs5186 investigation, individuals with type 1 diabetic nephropathy compared to individuals with type 1 diabetes mellitus in a European population (*AGTR1* rs5186 A allele compared to C allele).

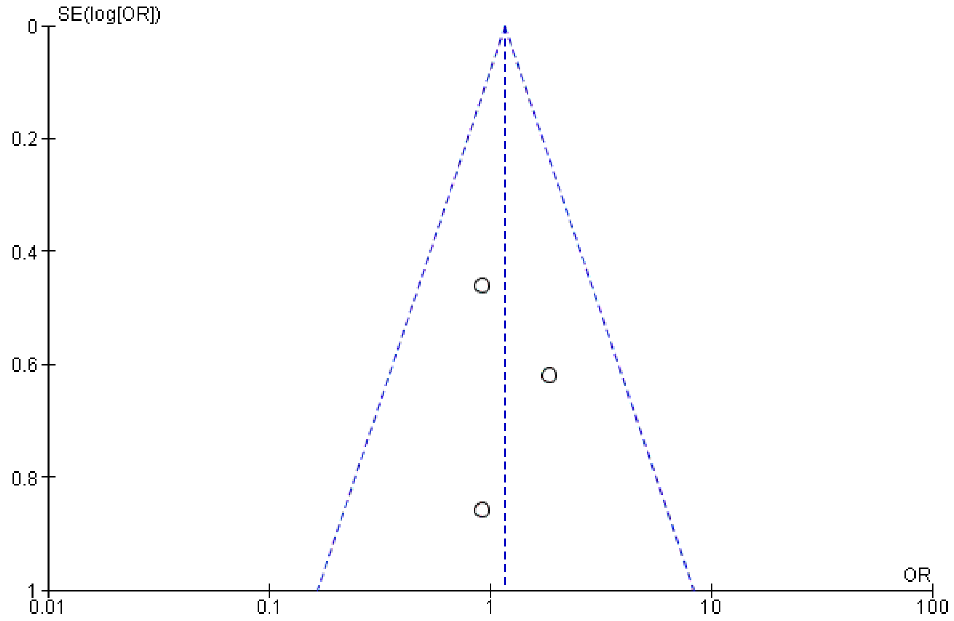

Supplementary Figure S7b: Funnel plot - *AGTR1* rs5186 investigation, individuals with type 2 diabetic nephropathy compared to individuals with type 2 diabetes mellitus in an East Asian population (*AGTR1* rs5186 A allele compared to C allele).

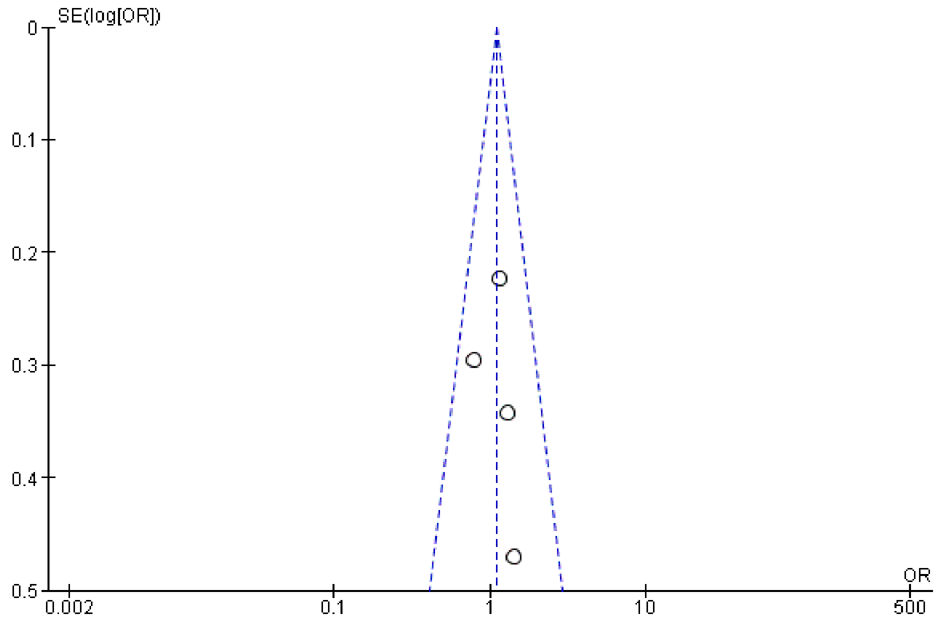

Supplementary Figure S7c: Funnel plot - *AGTR1* rs5186 investigation, individuals with IgA nephropathy compared to healthy controls in an East Asian population (*AGTR1* rs5186 A allele compared to C allele).

peer review only

Supplementary Table S1: Search Terms

Gene	Search Terms
ACE	Angiotensin I Converting Enzyme<br>Angiotensin I Converting Enzyme (Peptidyl-Dipeptidase A) 1<br>Dipeptidyl Carboxypeptidase I<br>CD143 Antigen<br>Kininase II<br>DCP1<br>DCP<br>Angiotensin I Converting Enzyme Peptidyl-Dipeptidase A 1<br>Transcript<br>Angiotensin Converting Enzyme, Somatic Isoform<br>Peptidyl-Dipeptidase A<br>Carboxycathepsin<br>Testicular ECA<br>Peptidase P<br>EC 3.4.15.1<br>EC 3.2.1.-<br>CD143<br>MVCD3<br>ACE1<br>ACE<br>ICH<br><br>AND<br><br>Kidney<br>Nephrology<br>Nephropathy<br>Renal<br><br>AND<br><br>SNP<br>Polymorphism<br>Variant<br>Allele<br>Genotype
ACE2	Angiotensin I Converting Enzyme 2<br>Angiotensin I Converting Enzyme (Peptidyl-Dipeptidase A) 2<br>Angiotensin-Converting Enzyme Homolog<br>ACE-Related Carboxypeptidase<br>Metalloprotease MPROT15<br>Peptidyl-Dipeptidase A<br>ACEH<br>EC 3.4.17.23<br>EC 3.4.17<br>ACE2<br><br>AND

For peer review only

	Kidney<br>Nephrology<br>Nephropathy<br>Renal<br><br><u>AND</u><br><br>SNP<br>Polymorphism<br>Variant<br>Allele<br>Genotype
AGT	Angiotensinogen<br>Angiotensinogen (Serpin Peptidase Inhibitor, Clade A, Member 8)<br>Serpin Peptidase Inhibitor, Clade A, Member 8<br>Serpin A8<br>SERPINA8<br>Serine (Or Cysteine) Proteinase Inhibitor<br>Alpha-1 Antiproteinase, Antitrypsin<br>Alpha-1 Antiproteinase<br>Pre-Angiotensinogen<br>Angiotensin II<br>Angiotensin I<br>Antitrypsin<br>ANHU<br>AGT<br><br><u>AND</u><br><br>Kidney<br>Nephrology<br>Nephropathy<br>Renal<br><br><u>AND</u><br><br>SNP<br>Polymorphism<br>Variant<br>Allele<br>Genotype
AGTR1	Angiotensin II Receptor Type 1<br>Angiotensin II Receptor, Type 1<br>AGTR1B<br>AT1AR<br>AT1BR<br>AT2R1<br>AT1<br>Type-1B Angiotensin II Receptor<br>Angiotensin II Type-1 Receptor<br>Angiotensin Receptor 1B

For peer review only

	AGTR1A<br>AT2R1B<br>HAT1R<br>AG2S<br>AT1B<br>AT1R<br>AGTR1<br>AT2R1A<br><br><u>AND</u><br><br>Kidney<br>Nephrology<br>Nephropathy<br>Renal<br><br><u>AND</u><br><br>SNP<br>Polymorphism<br>Variant<br>Allele<br>Genotype
AGTR2	Angiotensin II Receptor Type 2<br>Angiotensin II Receptor, Type 2<br>Angiotensin II Type-2 Receptor<br>AT2<br>Angiotensin Receptor 2<br>ATGR2<br>MRX88<br><br><u>AND</u><br><br>Kidney<br>Nephrology<br>Nephropathy<br>Renal<br><br><u>AND</u><br><br>SNP<br>Polymorphism<br>Variant<br>Allele<br>Genotype
REN	Renin<br>Angiotensinogenase<br>EC 3.4.23.15<br>Angiotensin-Forming Enzyme<br>Renin Precursor, Renal<br>EC 3.4.23

For peer review only

	HNFJ2<br>REN<br><br><u>AND</u><br><br>Kidney<br>Nephrology<br>Nephropathy<br>Renal<br><br><u>AND</u><br><br>SNP<br>Polymorphism<br>Variant<br>Allele<br>Genotype
Additional filters added to database searches	English / Spanish language<br>Human studies<br>Case / Control studies<br>Not clinical trials<br>Not review articles<br>Not a case report<br>Not a meta-analysis

For peer review only

Supplementary Table S2: Ethnicity Codes

**Population**	**Population Code**
Ad Mixed American (Southern USA and Central America)	AMR
African American	AFR
East Asian	EAS
European	EUR
Middle Eastern	ME
Mix of ethnicities	MIX
North African	NA
South Asian	SAS

For peer review only

Supplementary Table S3a: Excluded studies from the *ACE* search

*Exclusion stage relates to the stage at which the article was removed as shown in the flow diagram (Supplementary Figure S1a)

Exclusion Stage	Title	Authors	Pubmed ID or WoS ID if Pubmed ID not available	Reason
1	Angiotensin I - Converting enzyme gene polymorphism modulates the consequences of in utero growth retardation on plasma insulin in young adults	Cambien F et al.	9519756	No data for ACE
1	Clinical utility of chitotriosidase enzyme activity in nephropathic cystinosis.	Elmonem MA et al.	25407738	No data for ACE
1	Polymorphisms in the gene encoding angiotensin I converting enzyme 2 and diabetic nephropathy	Frojdo S et al.	16211375	No data for ACE
1	Effects of erythropoietin, angiotensin II, and angiotensin-converting enzyme inhibitor on erythroid precursors in patients with posttransplantation erythrocytosis.	Glicklich D et al.	10428268	No data for ACE
1	DDOST, PRKCSH and LGALS3, which encode AGE-receptors 1, 2 and 3, respectively, are not associated with diabetic nephropathy in type 1 diabetes.	Hoverfelt A et al.	20490454	No data for ACE
1	Neuropeptide YY1 receptor polymorphism as a prognostic predictor in Japanese patients with IgA nephropathy	Ito H et al.	10363627	No data for ACE
1	Manganese superoxide dismutase gene polymorphism (V16A) is associated with stages of albuminuria in Korean type 2 diabetic patients.	Lee SJ et al.	16324912	No data for ACE
1	Impact of the preintervention rate of renal function decline on outcome of renoprotective intervention.	Lely AT et al.	18077786	No data for ACE

For peer review only

1	Kinin-dependent hypersensitivity reactions in hemodialysis: metabolic and genetic factors.	Molinaro G et al.	17003818	No data for ACE
1	Effect of a polymorphism of endothelial nitric oxide synthase gene in Japanese patients with IgA nephropathy.	Morita T et al.	10543322	No data for ACE
1	Nephropathy in type 1 diabetes: a manifestation of insulin resistance and multiple genetic susceptibilities? Further evidence from the Pittsburgh Epidemiology of Diabetes Complication Study.	Orchard TJ et al.	12164879	No data for ACE
1	Role of glycaemic control in development of microalbuminuria in patients with insulin dependent diabetes.	Powrie JK et al.	7819935	No data for ACE
1	Association of TNF-Î± -308 G > A and ACE I/D gene polymorphisms in hemodialysis patients with arteriovenous fistula thrombosis.	Sener EF et al.	24126814	No data for ACE
1	N-domain angiotensin I-converting enzyme expression in renal artery of Wistar, Wistar Kyoto, and spontaneously hypertensive rats	Bueno V et al.	15194348	Non-human study
1	Kallikrein and amylase contents in tissues from a mutant mouse model for human cystic fibrosis.	Catanzaro OL et al.	6186886	Non-human study
1	Connexin 43 is not essential for the control of renin synthesis and secretion	Gerl M et al.	24062052	Non-human study
1	Multi-species comparative analysis of the equine ACE gene identifies a highly conserved potential transcription factor binding site in intron 16.	Hamilton NA et al.	23408978	Non-human study
1	Renal angiotensin converting enzyme promotes renal damage during ureteral obstruction	Stoneking BJ et al.	9719278	Non-human study
1	Angiotensin-converting enzyme genotype is a predictive factor in the peak panel-reactive antibody response.	Akcay A et al.	15013293	Not a case-control study
1	Association of the genetic polymorphisms of the renin-angiotensin system and endothelial nitric oxide synthase with chronic renal transplant dysfunction.	Akcay A et al.	15385810	Not a case-control study

For peer review only

1	PAI-1 4G/5G and ACE I/D gene polymorphisms and the occurrence of myocardial infarction in patients on intermittent dialysis.	Aucella F et al.	12748347	Not a case-control study
1	Angiotensin-converting-enzyme insertion/deletion genotype and long-term renal allograft survival.	Beige J et al.	9550656	Not a case-control study
1	Deletion insertion polymorphism of the angiotensin converting enzyme gene and progression of diabetic nephropathy.	Bjorck S et al.	9269704	Not a case-control study
1	Genetics of angiotensin I-converting enzyme	Costerousse O et al.	9247746	Not a case-control study
1	Captopril enhances transforming growth factor (TGF)-beta1 expression in peripheral blood mononuclear cells: a mechanism independent from angiotensin converting enzyme inhibition? A study in cyclosporine-treated kidney-transplanted patients.	Di Paolo S et al.	12499886	Not a case-control study
1	Insertion/deletion polymorphism of the angiotensin-converting enzyme predicts left ventricular hypertrophy after renal transplantation.	Fedor R et al.	21620105	Not a case-control study
1	Antiproteinuric effect of candesartan cilexetil in Japanese subjects with type 2 diabetes and nephropathy.	Haneda M et al.	15364166	Not a case-control study
1	Association between Angiotensin I-Converting Enzyme Insertion/Deletion Polymorphism and Prognosis of Kidney Transplantation: A Meta-Analysis	Huang ZK et al.	26000752	Not a case-control study
1	Is there a role of angiotensin-converting enzyme gene polymorphism in the failure of arteriovenous femoral shunts for hemodialysis?	Isbir CS et al.	11525534	Not a case-control study
1	Smoking has no impact on survival and it is not associated with ACE gene I/D polymorphism in hemodialysis patients.	Kiss I et al.	28058974	Not a case-control study
1	Antihypertensive treatment modulates the association between the D/I ACE gene polymorphism and left ventricular hypertrophy: a meta-analysis	Kuznetsova T et al.	10918550	Not a case-control study

For peer review only

1	Genetics and the prediction of complications in type 1 diabetes	Marre M	10097900	Not a case-control study
1	Hereditary factors in the development of diabetic renal disease	Marre M et al.	10922971	Not a case-control study
1	The effect of angiotensin receptor blockade ARB on the regression of left ventricular hypertrophy in hemodialysis patients: comparison between patients with D allele and non-D allele ACE gene polymorphism.	Nakayama M et al.	16312263	Not a case-control study
1	Role of ACE and IL-1Î² Gene Polymorphisms in Erythropoeitin Hyporesponsive Patients with Chronic Kidney Disease with Anemia.	Nand N et al.	28457029	Not a case-control study
1	Angiotensin-I converting enzyme insertion/deletion polymorphism and its association with diabetic nephropathy: a meta-analysis of studies reported between 1994 and 2004 and comprising 14,727 subjects	Ng D et al.	15830182	Not a case-control study
1	Is the presence of retinopathy of practical value in defining cases of diabetic nephropathy in genetic association studies? The experience with the ACE insertion/deletion polymorphism in 53 studies comprising 17,791 subjects	Ng DPK et al.	18523141	Not a case-control study
1	Correlates of ACE activity in macroalbuminuric type 2 diabetic patients treated with chronic ACE inhibition.	Nikzamir A et al.	17986476	Not a case-control study
1	Insertion/deletion polymorphism in intron 16 of the ACE gene and left ventricular hypertrophy in patients with end-stage renal disease.	Osono E et al.	9820440	Not a case-control study
1	Risk factors for the progression of microalbuminuria in Japanese type 2 diabetic patients - a 10-year follow-up study	Oue T et al.	10580616	Not a case-control study
1	Survival in type 2 diabetic patients in dialysis and the number of risk alleles in polymorphisms of the renin-angiotensin system genes.	Padro-Miquel A et al.	19014923	Not a case-control study

For peer review only

1	Effect of deletion polymorphism of angiotensin converting enzyme gene on progression of diabetic nephropathy during inhibition of angiotensin converting enzyme: observational follow up study.	Parving HH et al.	8806248	Not a case-control study
1	Altered activities of kininase II, an angiotensin converting enzyme, prekallikrein, and nitric oxide in Kuwaiti patients with type 2 diabetes	Sharma JN et al.	25964383	Not a case-control study
1	Impact of genetic polymorphisms of the renin-angiotensin system and of non-genetic factors on kidney transplant function--a single-center experience.	Siekierka-Harreis M et al.	19681973	Not a case-control study
1	Angiotensin-converting enzyme (ACE) inhibition in type 2, diabetic patients-- interaction with ACE insertion/deletion polymorphism.	So WY et al.	16395257	Not a case-control study
1	Microfluidic chip-based method for genotyping microsatellites, VNTRs and insertion/deletion polymorphisms.	Sohni YR et al.	12554058	Not a case-control study
1	M235T angiotensinogen gene polymorphism and cardiovascular renal risk	Staessen JA et al.	10100088	Not a case-control study
1	Mistyping of the human angiotensin-converting enzyme gene polymorphism: Frequency, causes and possible methods to avoid errors in typing	Ueda S et al.	8863184	Not a case-control study
1	Association between angiotensin-converting-enzyme gene polymorphism and failure of renoprotective therapy.	van Essen GG et al.	8538349	Not a case-control study
1	Contribution of gene polymorphisms in the renin-angiotensin system to macroangiopathy in patients with diabetic nephropathy.	Wong TY et al.	11431175	Not a case-control study
1	Disease progression, response to ACEI/ATRA therapy and influence of ACE gene in IgA nephritis.	Woo KT et al.	17601378	Not a case-control study
1	Angiotensin-converting enzyme inhibitor versus angiotensin 2 receptor antagonist therapy and the influence of angiotensin-converting enzyme gene polymorphism in IgA nephritis.	Woo KT et al.	18536822	Not a case-control study

For peer review only

1	Angiotensin converting enzyme gene polymorphism and development of post-transplant erythrocytosis.	Yildiz A et al.	12832741	Not a case-control study
1	Gene polymorphisms of the renin-angiotensin-aldosterone system and angiotensin II type 1-receptor activating antibodies in renal rejection.	Zhang G et al.	17984617	Not a case-control study
1	Angiotensin-converting enzyme gene polymorphism in Kuwaiti patients with systemic lupus erythematosus.	Al-Awadhi AM et al.	17631741	Not a renal disease focus
1	Association of Angiotensin Converting Enzyme Insertion-Deletion Polymorphism with Hypertension in Emiratis with Type 2 Diabetes Mellitus and Its Interaction with Obesity Status.	Alsafar H et al.	26491214	Not a renal disease focus
1	Association of ACE gene D polymorphism with left ventricular hypertrophy in patients with diastolic heart failure: a case-control study.	Bahramali E et al.	26861937	Not a renal disease focus
1	A novel human heparanase splice variant, T5, endowed with protumorigenic characteristics.	Barash U et al.	20007507	Not a renal disease focus
1	Synergistic effect of alpha-adducin and ACE genes causes blood pressure changes with body sodium and volume expansion.	Barlassina C et al.	10720960	Not a renal disease focus
1	Isolated polycystic liver disease genes define effectors of polycystin-1 function.	Besse W et al.	28375157	Not a renal disease focus
1	Association between plasma activities of semicarbazide-sensitive amine oxidase and angiotensin-converting enzyme in patients with type 1 diabetes mellitus.	Boomsma F et al.	15830186	Not a renal disease focus
1	CCL18: a urinary marker of Gaucher cell burden in Gaucher patients.	Boot RG et al.	16736095	Not a renal disease focus
1	Renin-angiotensin system gene polymorphisms: assessment of the risk of coronary heart disease.	Buraczynska M et al.	14502296	Not a renal disease focus
1	Angiotensin-converting enzyme (ACE) haplotypes and cyclosporine A (CsA) response: a model of the complex relationship between ACE quantitative trait locus and pathological phenotypes.	Catarsi P et al.	16002416	Not a renal disease focus

For peer review only

1	A study on the association between angiotensin-I converting enzyme I/D dimorphism and type-2 diabetes mellitus.	Chmaisse HN et al.	19861867	Not a renal disease focus
1	alpha-adducin and angiotensin I-converting enzyme polymorphisms in essential hypertension	Clark CJ et al.	11116113	Not a renal disease focus
1	Elevated urinary albumin excretion is not linked to the angiotensin I-converting enzyme gene polymorphism in clinically healthy subjects	Clausen P et al.	10872702	Not a renal disease focus
1	Association of renin-angiotensin and endothelial nitric oxide synthase gene polymorphisms with blood pressure progression and incident hypertension: prospective cohort study.	Conen D et al.	18698212	Not a renal disease focus
1	Angiotensin I-converting enzyme (kininase II) in cardiovascular and renal regulations and diseases	Costerousse O et al.	9830503	Not a renal disease focus
1	ACE and PC-1 gene polymorphisms in normoalbuminuric Type 1 diabetic patients: a 10-year prospective study.	de Azevedo MJ et al.	12126783	Not a renal disease focus
1	Insertion/Deletion Polymorphism of Angiotensin I-converting Enzyme Gene Is Linked With Chromophobe Renal Cell Carcinoma	de Martino M et al.	21477733	Not a renal disease focus
1	Polymorphisms in genes of the renin-angiotensin-aldosterone system and renal cell cancer risk: interplay with hypertension and intakes of sodium, potassium and fluid.	Deckers IA et al.	24978482	Not a renal disease focus
1	ACE gene insertion/deletion polymorphism modulates capillary permeability in hypertension	Dell'omo G et al.	16889537	Not a renal disease focus
1	Genetic polymorphisms associated with exertional rhabdomyolysis	Deuster PA et al.	23543093	Not a renal disease focus
1	Distribution of different HLA antigens in Greek hypertensives according to the angiotensin-converting enzyme genotype.	Diamantopoulos EJ et al.	10821349	Not a renal disease focus
1	Are the angiotensin-converting enzyme gene and activity risk factors for stroke?	Dikmen M et al.	16791358	Not a renal disease focus
1	Angiotensin-converting enzyme (insertion/deletion) and endothelial nitric oxide synthase polymorphisms in patients with systemic lupus erythematosus.	Douglas G et al.	15338496	Not a renal disease focus

For peer review only

1	Abnormal hepatocystin caused by truncating PRKCSH mutations leads to autosomal dominant polycystic liver disease.	Drenth JP et al.	15057895	Not a renal disease focus
1	The angiotensin I-converting enzyme gene insertion/deletion polymorphism is linked to early gastric cancer	Ebert MPA et al.	16365022	Not a renal disease focus
1	Association of chitotriosidase enzyme activity and genotype with the risk of nephropathy in type 2 diabetes.	Elmonem MA et al.	26589000	Not a renal disease focus
1	Relationship of bradykinin B2 receptor gene polymorphism with essential hypertension and left ventricular hypertrophy.	Fu Y et al.	15894833	Not a renal disease focus
1	Deletion polymorphism of the angiotensin-converting enzyme gene is independently associated with left ventricular mass and geometric remodeling in systemic hypertension.	Gharavi AG et al.	8677872	Not a renal disease focus
1	The presence of PAI-1 4G/5G and ACE DD genotypes increases the risk of early-stage AVF thrombosis in hemodialysis patients.	Gungor Y et al.	21332339	Not a renal disease focus
1	The Captopril Prevention Project (CAPPP) in hypertension--baseline data and current status.	Hansson L et al.	9495662	Not a renal disease focus
1	Relationship of eNOS gene variants to diseases that have in common an endothelial cell dysfunction.	Heltianu C et al.	15784171	Not a renal disease focus
1	Relationship of the angiotensin-converting enzyme gene polymorphism to glucose intolerance, insulin resistance, and hypertension in NIDDM.	Huang XH et al.	9544854	Not a renal disease focus
1	Distribution of human leukocyte antigen alleles in systemic lupus erythematosus patients with angiotensin converting enzyme insertion/deletion polymorphism.	Hussain N et al.	23448612	Not a renal disease focus
1	Comprehensive analysis of the renin-angiotensin gene polymorphisms with relation to hypertension in the Japanese	Kato N et al.	10953993	Not a renal disease focus
1	Association of clinical manifestations with HLA-B alleles in Takayasu arteritis	Kitamura H et al.	9951811	Not a renal disease focus
1	Lys(173)Arg and -344T/C variants of CYP11B2 in Japanese patients with low-renin hypertension.	Komiya I et al.	10720581	Not a renal disease focus

For peer review only

1	Effects of angiotensin-converting enzyme gene polymorphism and serum vitamin D levels on ambulatory blood pressure measurement and left ventricular mass in Turkish hypertensive population.	Kulah E et al.	17625392	Not a renal disease focus
1	Efficacy of Korean Red Ginseng by Single Nucleotide Polymorphism in Obese Women: Randomized, Double-blind, Placebo-controlled Trial	Kwon DH et al.	23717118	Not a renal disease focus
1	Angiotensin-Converting Enzyme Gene Polymorphism Has No Influence On The Circulating Renin-Angiotensin-Aldosterone System Or Blood-Pressure In Normotensive Subjects	Lachurie Ml et al.	7796503	Not a renal disease focus
1	Mutations in PRKCSH cause isolated autosomal dominant polycystic liver disease.	Li A et al.	12529853	Not a renal disease focus
1	Angiotensin-converting enzyme (ACE) gene II genotype protects against the development of diabetic peripheral neuropathy in type 2 diabetes mellitus.	Mansoor Q et al.	22607040	Not a renal disease focus
1	Renal changes on hyperglycemia and angiotensin-converting enzyme in type 1 diabetes	Marre M et al.	10082486	Not a renal disease focus
1	Detection of the association between a deletion polymorphism in the gene encoding angiotensin I-converting enzyme and advanced diabetic retinopathy	Matsumoto A et al.	11106834	Not a renal disease focus
1	Association of the D allele of the angiotensin I converting enzyme polymorphism with malignant vascular injury	Mayer NJ et al.	11836444	Not a renal disease focus
1	Late-onset acid maltase deficiency. Detection of patients and heterozygotes by urinary enzyme assay.	Mehler M et al.	9923	Not a renal disease focus
1	Association of polymorphisms of angiotensin I converting enzyme 2 with retinopathy in type 2 diabetes mellitus among Chinese individuals	Meng N et al.	25359286	Not a renal disease focus
1	Angiotensin converting enzyme gene polymorphism and renal hemodynamic function in early diabetes.	Miller JA et al.	8995725	Not a renal disease focus

For peer review only

1	Genetic risk for renal artery stenosis: association with deletion polymorphism in angiotensin 1-converting enzyme gene.	Missouris CG et al.	8821841	Not a renal disease focus
1	Renal ACE immunohistochemical localization in NIDDM patients with nephropathy.	Mizuiri S et al.	9469501	Not a renal disease focus
1	Association of B2 receptor polymorphisms and ACE activity with ACE inhibitor-induced angioedema in black and mixed-race South Africans.	Moholisa RR et al.	23730990	Not a renal disease focus
1	Renal outcome and vascular morbidity in systemic lupus erythematosus (SLE): lack of association with the angiotensin-converting enzyme gene polymorphism.	Molad Y et al.	11071585	Not a renal disease focus
1	Contribution of angiotensin I converting enzyme gene polymorphism and angiotensinogen gene polymorphism to blood pressure regulation in essential hypertension.	Mondorf UF et al.	9524045	Not a renal disease focus
1	Inhibition of tissue angiotensin converting enzyme activity prevents malignant hypertension in TGR(mREN2)27.	Montgomery HE et al.	9797175	Not a renal disease focus
1	Independent, Marked Associations Of Alleles Of The Insulin-Receptor And Dipeptidyl Carboxypeptidase-I Genes With Essential-Hypertension	Morris Bj et al.	8104754	Not a renal disease focus
1	Increased D allele frequency of the angiotensin-converting enzyme gene in pulmonary fibrosis.	Morrison CD et al.	11381371	Not a renal disease focus
1	Angiotensin converting enzyme (ACE) insertion/deletion (I/D) polymorphism, and diabetic retinopathy in subjects with IDDM and NIDDM.	Nagi DK et al.	8582133	Not a renal disease focus
1	Angiotensin-converting enzyme (ACE) gene insertion/deletion polymorphism is not a risk factor for hypertension in SLE nephritis.	Negi VS et al.	25957879	Not a renal disease focus
1	Carotid intima-media thickness and ACE-gene polymorphism in hemodialysis patients.	Nergizoglu G et al.	10493570	Not a renal disease focus
1	Association of angiotensin-converting enzyme gene insertion/deletion polymorphism with metabolic syndrome in Iranians with type 2 diabetes mellitus.	Nikzamir A et al.	18154415	Not a renal disease focus

For peer review only

1	Angiotensin Converting Enzyme Gene Insertion/Deletion Variant and Familial Mediterranean Fever-related Amyloidosis.	Nursal AF et al.	29891744	Not a renal disease focus
1	Pharmacogenetic analysis of the effect of angiotensin-converting enzyme inhibitor on restenosis after percutaneous transluminal coronary angioplasty.	Okamura A et al.	10535720	Not a renal disease focus
1	Genetic polymorphisms of the renin-angiotensin system and atheromatous renal artery stenosis.	Olivieri O et al.	10567188	Not a renal disease focus
1	Different impact of deletion polymorphism of gene on the risk of renal and coronary artery disease.	Olivieri O et al.	11791024	Not a renal disease focus
1	Increased frequency of the angiotensin-converting enzyme gene D-allele is associated with noninfectious pulmonary dysfunction following allogeneic stem cell transplant.	Onizuka M et al.	16044138	Not a renal disease focus
1	Hepatocystin is Essential for TRPM7 Function During Early Embryogenesis.	Overton JD et al.	26671672	Not a renal disease focus
1	Angiotensin-converting enzyme and angiotensin II receptor subtype 2 genotypes in type 1 diabetes and severe hypoglycaemia requiring emergency treatment: a case cohort study.	Pedersen-Bjergaard U et al.	19820429	Not a renal disease focus
1	Impact of maternal angiotensinogen M235T polymorphism and angiotensin-converting enzyme insertion/deletion polymorphism on blood pressure, protein excretion and fetal outcome in pregnancy.	Pfab T et al.	17563539	Not a renal disease focus
1	Renovascular disease: effect of ACE gene deletion polymorphism and endovascular revascularization.	Pizzolo F et al.	14718831	Not a renal disease focus
1	Angiotensin-converting enzyme gene polymorphism in patients with systemic lupus.	Prkacin I et al.	11505631	Not a renal disease focus
1	The relationship between ACE/AGT gene polymorphisms and the risk of diabetic retinopathy in Chinese patients with type 2 diabetes.	Qiao YC et al.	29378484	Not a renal disease focus
1	Association of angiotensin-converting enzyme gene dimorphisms with severity of lupus disease.	Rabbani MA et al.	18711292	Not a renal disease focus

For peer review only

1	Leukocyte beta-glucosidase in homozygotes and heterozygotes for Gaucher disease.	Raghavan SS et al.	6770675	Not a renal disease focus
1	Association of angiotensinogen M235T and A(-6)G gene polymorphisms with coronary heart disease with independence of essential hypertension: the PROCAGENE study. Prospective Cardiac Gene.	Rodriquez-Perez JC et al.	11345362	Not a renal disease focus
1	Testing of potential glycan-based heparanase inhibitors in a fluorescence activity assay using either bacterial heparinase II or human heparanase.	Schoenfeld AK et al.	24667567	Not a renal disease focus
1	Angiotensin-converting enzyme gene I/D polymorphism increases the susceptibility to hypertension and additive diseases: A study on North Indian patients.	Singh M et al.	27030424	Not a renal disease focus
1	Association of APOE (Hha1) and ACE (I/D) gene polymorphisms with type 2 diabetes mellitus in North West India	Singh PP et al.	16621107	Not a renal disease focus
1	Angiotensin-converting enzyme gene I/D polymorphism in malignant hypertension.	Stefansson B et al.	10855732	Not a renal disease focus
1	Increased amount of the angiotensin-converting enzyme (ACE) mRNA originating from the ACE allele with deletion.	Suehiro T et al.	15164285	Not a renal disease focus
1	Genetic variants in hypertensive patients with coronary artery disease and coexisting atheromatous renal artery stenosis.	Szperl M et al.	19043368	Not a renal disease focus
1	Angiotensin I converting enzyme gene polymorphisms in systemic lupus erythematosus: decreased prevalence of DD genotype in African American patients	Tassiulas IO et al.	9710341	Not a renal disease focus
1	Albuminuria and the renin-angiotensin system gene polymorphisms in type-2-diabetic and in normoglycemic hypertensive Chinese.	Thomas GN et al.	11200871	Not a renal disease focus
1	Peripheral vascular disease in Type 2 diabetic Chinese patients: associations with metabolic indices, concomitant vascular disease and genetic factors.	Thomas GN et al.	14632699	Not a renal disease focus
1	Angiotensin-converting enzyme gene polymorphism and vascular manifestations in Korean patients with SLE.	Uhm WS et al.	12043886	Not a renal disease focus

For peer review only

1	Genetic risk of atherosclerotic renal artery disease: the candidate gene approach in a renal angiography cohort.	van Onna M et al.	15326089	Not a renal disease focus
1	Angiotensin I-converting enzyme and angiotensinogen gene interaction and prediction of essential hypertension	Vasku A et al.	9607178	Not a renal disease focus
1	Association between ACE gene polymorphisms and Alzheimer's disease in Han population in Hebei Peninsula	Wang XL et al.	WOS:000412148800122	Not a renal disease focus
1	COL4A1 mutations in patients with sporadic late-onset intracerebral hemorrhage	Weng YC et al.	22522439	Not a renal disease focus
1	Increased expression of angiotensin II type 1 receptor (AGTR1) in heart transplant recipients with recurrent rejection.	Yamani MH et al.	17097490	Not a renal disease focus
1	No association between deletion-type angiotensin-converting enzyme gene polymorphism and left-ventricular hypertrophy in hemodialysis patients.	Yildiz A et al.	10657713	Not a renal disease focus
1	Frequencies Of Variants Of Candidate Genes In Different Age-Groups Of Hypertensives	Zee Ryl et al.	7882587	Not a renal disease focus
1	[Renin-angiotensin system genes in chronic glomerulonephritis].	Buraczynska M et al.	11865575	Not English or Spanish
1	[Association of the renin-angiotensin system gene polymorphism with nephropathy in type II diabetes].	Buraczynska M et al.	12476891	Not English or Spanish
1	[Genetic predisposition to systemic complications of arterial hypertension in maintenance haemodialysis patients].	Bzoma B et al.	19112833	Not English or Spanish
1	[Is PstI polymorphism of the angiotensin I converting enzyme gene associated with nephropathy development in non-insulin-dependent diabetes mellitus (preliminary study)].	Grzeszczak W et al.	9499204	Not English or Spanish
1	[Angiotensin-converting enzyme gene polymorphism and the clinical pathological features and progression in lupus nephritis].	Guan T et al.	10436947	Not English or Spanish
1	[I/D relationship between polymorphism of ACE gene and progression of chronic glomerulonephritis].	Kaliev RR et al.	16078593	Not English or Spanish
1	[Association of the complex of polymorphic markers of ACE genes, aldosteron synthetase and endothelial synthetase of nitric oxide with progression of chronic glomerulonephritis].	Kamysheva ES et al.	15532370	Not English or Spanish

For peer review only

1	[Angiotensin-converting enyme insertion/deletion polymorphism and blood pressure regulation in type 2 diabetic patients].	Krajina-Andricevic M et al.	23120809	Not English or Spanish
1	[Polymorphism studies of angiotensin converting enzyme gene in chronic glomerulonephritis].	Kutyrina IM et al.	10420452	Not English or Spanish
1	[Relationship between serum angiotensin I-converting enzyme activity and diabetic nephropathy in patients with type II diabetes].	Liao L et al.	12016801	Not English or Spanish
1	[Study on candidate genes of benazepril related cough in Chinese hypertensives].	Lu J et al.	12848919	Not English or Spanish
1	[Association of alpha-adducin and angiotensin converting enzyme gene polymorphisms with salt-sensitive hypertension and early renal injury].	Lu LH et al.	18393230	Not English or Spanish
1	[Association between insertion-deletion polymorphism of the angiotensin-converting enzyme gene and development of angiopathies in patients with non-insulin dependent diabetes mellitus from the Chuvash Republic].	Miloserdova OV et al.	11234416	Not English or Spanish
1	[Relationship between I/D polymorphism of angiotensin I converting enzyme gene and microvascular complications in type 2 diabetic patients].	Moleda P et al.	17941464	Not English or Spanish
1	[A study on angiotensin-I converting enzyme polymorphism in CAPD patients].	Nishina M	9014479	Not English or Spanish
1	[Arterial hypertension in glomerulonephritis].	Oko A et al.	14974362	Not English or Spanish
1	[Association between angiotensin-converting enzyme 2 gene polymorphisms and childhood primary nephrotic syndrome].	Qiu MY et al.	25815490	Not English or Spanish
1	[Association between ACE gene polymorphism and therapeutic responsiveness of ACEI in diabetic nephropathy].	Wang L et al.	10923445	Not English or Spanish
1	[Relationship between angiotensin 1 converting enzyme gene polymorphism and diabetic nephropathy].	Wu S et al.	9596955	Not English or Spanish

For peer review only

1	[Correlative study between angiotensin-converting enzyme gene polymorphism and hepatorenal syndrome].	Wu XX et al.	15698501	Not English or Spanish
1	DD genotype of ACE gene in boys: may it be a risk factor for minimal change nephrotic syndrome?	Alasehirli B et al.	22017506	Paediatric individuals
1	Angiotensin-converting enzyme gene insertion/deletion polymorphism and renal damage in childhood uropathies.	al-Eisa A et al.	10986863	Paediatric individuals
1	Angiotensin converting enzyme gene insertion/deletion polymorphism in idiopathic nephrotic syndrome in Kuwaiti Arab children.	Al-Eisa A et al.	11487079	Paediatric individuals
1	Polymorphisms in angiotensin-converting enzyme gene and severity of renal disease in Henoch-Schoenlein patients. Italian Group of Renal Immunopathology.	Amoroso A et al.	9870486	Paediatric individuals
1	Angiotensin converting enzyme gene polymorphism in Asian Indian children with congenital uropathies.	Bajpai M et al.	14713838	Paediatric individuals
1	Late effects on renal glomerular and tubular function in childhood cancer survivors.	Bardi E et al.	15390293	Paediatric individuals
1	Angiotensin-converting enzyme genotype is not a significant genetic risk factor for idiopathic nephrotic syndrome in Croatian children.	Batinc D et al.	25997642	Paediatric individuals
1	HPSE2 mutations in urofacial syndrome, non-neurogenic neurogenic bladder and lower urinary tract dysfunction.	Bulum B et al.	25924634	Paediatric individuals
1	ACE gene polymorphism in Turkish children with nephrotic syndrome.	Celik US et al.	16825089	Paediatric individuals
1	Glycosphingolipid levels in an unusual neurovisceral storage disease characterized by lactosylceramide galactosyl hydrolase deficiency: lactosylceramidosis.	Dawson G	5016302	Paediatric individuals
1	Association of ACE and MDR1 Gene Polymorphisms with Steroid Resistance in Children with Idiopathic Nephrotic Syndrome.	Dhandapani MC et al.	26154535	Paediatric individuals
1	Polymorphisms of the angiotensin converting enzyme and angiotensin II type 1 receptor genes and renal scarring in non-uropathic children with recurrent urinary tract infection.	Ece A et al.	16109085	Paediatric individuals

For peer review only

1	Is ACE gene polymorphism a risk factor for renal scarring with low-grade reflux?	Erdogan H et al.	15138870	Paediatric individuals
1	ACE gene polymorphism in Egyptian children with idiopathic nephrotic syndrome.	Fahmy ME et al.	18792483	Paediatric individuals
1	Genetic polymorphisms of the renin-angiotensin system and the outcome of focal segmental glomerulosclerosis in children.	Frishberg Y et al.	9853248	Paediatric individuals
1	Angiotensinogen gene T235 variant: a marker for the development of persistent microalbuminuria in children and adolescents with type 1 diabetes mellitus	Gallego PH et al.	18413222	Paediatric individuals
1	Implication of genetic variations in congenital obstructive nephropathy.	Hahn H et al.	16133060	Paediatric individuals
1	Angiotensin-converting enzyme insertion/deletion gene polymorphism in Egyptian children with systemic lupus erythematosus: a possible relation to proliferative nephritis.	Hammad A et al.	27956582	Paediatric individuals
1	ACE gene polymorphism and renal scarring in primary vesicoureteric reflux.	Haszon I et al.	12478352	Paediatric individuals
1	ACE I/D gene polymorphism predicts renal damage in congenital uropathies.	Hohenfellner K et al.	10452281	Paediatric individuals
1	Impact of ACE I/D gene polymorphism on congenital renal malformations.	Hohenfellner K et al.	11354781	Paediatric individuals
1	Significance of ACE genotypes and medical treatments in childhood focal glomerulosclerosis.	Hori C et al.	11474225	Paediatric individuals
1	Impact of common functional polymorphisms in renin angiotensin system genes on the risk of renal parenchymal scarring following childhood urinary tract infection.	Hussein A et al.	25939993	Paediatric individuals
1	Gene polymorphisms of adducin GLY460TRP, ACE I/D, AND AGT M235T in pediatric hypertension patients.	Kaplan I et al.	25262176	Paediatric individuals
1	ACE serum level and I/D gene polymorphism in children with obstructive uropathies and other congenital anomalies of the kidney and urinary tract.	Kostadinova ES et al.	27206329	Paediatric individuals

For peer review only

1	ACE and AT1 receptor gene polymorphisms and renal scarring in urinary bladder dysfunction	Kostic M et al.	15179569	Paediatric individuals
1	Association of angiotensin converting enzyme and angiotensin type 2 receptor gene polymorphisms with renal damage in posterior urethral valves.	Laksmi NK et al.	20149750	Paediatric individuals
1	Renin-angiotensin system polymorphisms in Taiwanese primary vesicoureteral reflux.	Liu KP et al.	15045574	Paediatric individuals
1	Polymorphisms of renin-angiotensin system genes in childhood IgA nephropathy.	Maruyama K et al.	11354780	Paediatric individuals
1	Angiotensin-Converting Enzyme Gene Polymorphism in Children with Idiopathic Nephrotic Syndrome, Effect on Biopsy Findings.	Monajemzadeh M et al.	28481137	Paediatric individuals
1	ACE I/D gene polymorphism in primary FSGS and steroid-sensitive nephrotic syndrome.	Oktem F et al.	14986085	Paediatric individuals
1	Implications of certain genetic polymorphisms in scarring in vesicoureteric reflux: importance of ACE polymorphism.	Ozen S et al.	10401028	Paediatric individuals
1	Renin-angiotensin system gene polymorphisms: association with susceptibility to Henoch-Schonlein purpura and renal involvement	Ozkaya O et al.	16521052	Paediatric individuals
1	Renin-angiotensin gene polymorphism in children with uremia and essential hypertension.	Papp F et al.	12579405	Paediatric individuals
1	Renin-angiotensin system polymorphisms and renal scarring.	Pardo R et al.	12579398	Paediatric individuals
1	Angiotensin converting enzyme gene polymorphism in Indian children with steroid sensitive nephrotic syndrome.	Patil SJ et al.	16272677	Paediatric individuals
1	Angiotensin I-converting enzyme-gene-polymorphism: Relationship to albumin excretion and blood pressure in pediatric patients with type-I-diabetes mellitus	Pavlovic M et al.	9354852	Paediatric individuals
1	Angiotensin I converting enzyme and angiotensinogen gene polymorphisms related to 24-h blood pressure in paediatric type I diabetes mellitus	Pavlovic M et al.	9950302	Paediatric individuals

For peer review only

1	Low renin-angiotensin system activity gene polymorphism and dysplasia associated with posterior urethral valves.	Peruzzi L et al.	16006956	Paediatric individuals
1	Modification of epigenetic patterns in low birth weight children: importance of hypomethylation of the ACE gene promoter.	Rangel M et al.	25170764	Paediatric individuals
1	Angiotensin-converting enzyme and angiotensin type 2 receptor gene genotype distributions in Italian children with congenital uropathies	Rigoli L et al.	15470205	Paediatric individuals
1	Effect of angiotensin-converting enzyme gene insertion/deletion polymorphism on steroid resistance in Egyptian children with idiopathic nephrotic syndrome.	Saber-Ayad M et al.	20418353	Paediatric individuals
1	ACE gene polymorphism in children with nephrotic syndrome in the Indonesian population.	Sasongko TH et al.	16421456	Paediatric individuals
1	Polymorphisms of the TNF-alpha and ACE genes, and renal scarring in infants with urinary tract infection.	Savvidou A et al.	20022049	Paediatric individuals
1	ACE gene insertion/deletion polymorphism and renal scarring in children with urinary tract infections.	Sekerli E et al.	19603195	Paediatric individuals
1	ACE gene insertion/deletion polymorphism in childhood idiopathic nephrotic syndrome.	Serdaroglu E et al.	16208534	Paediatric individuals
1	Association of the ACE-II genotype with the risk of nephrotic syndrome in Pakistani children.	Shahid S et al.	22033511	Paediatric individuals
1	ACE gene polymorphism in childhood IgA nephropathy: association with clinicopathologic findings.	Tanaka R et al.	9590186	Paediatric individuals
1	Role of platelet-activating factor acetylhydrolase gene mutation in Japanese childhood IgA nephropathy.	Tanaka R et al.	10430976	Paediatric individuals
1	Angiotensin-converting enzyme gene polymorphism in children with idiopathic nephrotic syndrome.	Tsai IJ et al.	16645262	Paediatric individuals
1	Angiotensin-converting enzyme gene insertion/deletion polymorphism in children with Henoch-Schonlein purpua nephritis.	Zhou J et al.	15315169	Paediatric individuals
1	Estimation of the relationship between the polymorphisms of selected genes: ACE, AGTR1, TGFÎ²1 and GNB3 with the occurrence of primary vesicoureteral reflux.	Zyczkowski M et al.	27988909	Paediatric individuals

For peer review only

1	Long-term renoprotective effects of losartan in diabetic nephropathy: interaction with ACE insertion/deletion genotype?	Andersen S et al.	12716812	Pharmaceutical drug focus
1	High serum enalaprilat in chronic renal failure	Elung-Jensen T et al.	11881130	Pharmaceutical drug focus
1	Randomized placebo-controlled trial of perindopril in normotensive, normoalbuminuric patients with type 1 diabetes mellitus.	Kvetny J et al.	11181984	Pharmaceutical drug focus
1	The influence of the ACE (I/D) polymorphism on systemic and renal vascular responses to angiotensins in normotensive, normoalbuminuric Type 1 diabetes mellitus.	Luik PT et al.	12856080	Pharmaceutical drug focus
1	Renin-angiotensin system polymorphisms and hemoglobin level in renal allografts: a comparative study between losartan and enalapril.	Noroozianavval M et al.	17524880	Pharmaceutical drug focus
1	ACE gene polymorphism and losartan treatment in type 2 diabetic patients with nephropathy	Parving HH et al.	18199798	Pharmaceutical drug focus
1	Effect of angiotensin-converting enzyme (ACE) gene polymorphism on progression of renal disease and the influence of ACE inhibition in IDDM patients: findings from the EUCLID Randomized Controlled Trial. EURODIAB Controlled Trial of Lisinopril in IDDM.	Penno G et al.	9726242	Pharmaceutical drug focus
1	Enalapril and losartan affect lipid peroxidation in renal transplant recipients with renin-angiotensin system polymorphisms.	Rashtchizadeh N et al.	17222813	Pharmaceutical drug focus
1	Chronic proteinuric nephropathies. II. Outcomes and response to treatment in a prospective cohort of 352 patients: differences between women and men in relation to the ACE gene polymorphism. Gruppo Italiano di Studi Epidemologici in Nefrologia (Gisen)	Ruggenenti P et al.	10616844	Pharmaceutical drug focus
2	The DD genotype of the ACE gene polymorphism is associated with diabetic nephropathy in the type-1 diabetics.	Azar ST et al.	11428725	Incorrect patient group

For peer review only

2	Genetic variation at the ACE gene is associated with persistent microalbuminuria and severe nephropathy in type 1 diabetes: the DCCT/EDIC Genetics Study.	Boright AP et al.	15793268	Incorrect patient group
2	ACE, PAI-1, decorin and Werner helicase genes are not associated with the development of renal disease in European patients with type 1 diabetes.	De Cosmo S et al.	10495473	Incorrect patient group
2	Genetic Predisposition To Diabetic Nephropathy - Evidence For A Role Of The Angiotensin I-Converting Enzyme Gene	Doria A et al.	7909524	Incorrect patient group
2	Polymorphism of angiotensin converting enzyme, angiotensinogen, and angiotensin II type 1 receptor genes and end-stage renal failure in IgA nephropathy: IGARAS - A study of 274 men	Frimat L et al.	11053482	Incorrect patient group
2	Polymorphism of the angiotensin I-converting enzyme gene in diabetic nephropathy in type II diabetic patients with proliferative retinopathy	Hanyu O et al.	9509566	Incorrect patient group
2	The angiotensin I-converting enzyme (ACE) locus is strongly associated with age and duration of diabetes in patients with type I diabetes	Hibberd ML et al.	9025006	Incorrect patient group
2	Hypertensive nephropathy and the gene for angiotensin-converting enzyme.	Kario K et al.	9081678	Incorrect patient group
2	Effect of ACE gene on diabetic nephropathy in NIDDM patients with insulin resistance.	Kuramoto N et al.	10023638	Incorrect patient group
2	Relationships between angiotensin I converting enzyme gene polymorphism and renal complications in Korean IDDM patients.	Oh TG et al.	8854649	Incorrect patient group
2	The frequency of factor V Leiden mutation, ACE gene polymorphism, serum ACE activity and response to ACE inhibitor and angiotensin II receptor antagonist drugs in Iranians type II diabetic patients with microalbuminuria.	Rahimi Z et al.	20853144	Incorrect patient group
2	Interaction of MTHFR 1298C with ACE D allele augments the risk of diabetic nephropathy in Western Iran.	Rahimi Z et al.	21942443	Incorrect patient group

For peer review only

2	The ACE insertion/deletion polymorphism has no influence on progression of renal function loss in autosomal dominant polycystic kidney disease.	van Dijk MA et al.	10831637	Incorrect patient group
2	Genetic polymorphisms of renin-angiotensin system and progression of interstitial nephritis.	Buraczynska M et al.	12898858	No data
2	Frequency of angiotensin-converting enzyme gene polymorphism in Turkish type 2 diabetic patients.	Degirmenci I et al.	16178979	No data
2	Association of the DD genotype and development of Japanese type 2 diabetic nephropathy.	Gohda T et al.	11770799	No data
2	Angiotensin I converting enzyme and angiotensinogen gene polymorphisms in non-insulin-dependent diabetes mellitus. Lack of relationship with diabetic nephropathy and retinopathy in a Caucasian Mediterranean population	Gutierrez C et al.	9258285	No data
2	Association between angiotensin-converting enzyme gene polymorphisms and diabetic nephropathy: case-control, haplotype, and family-based study in three European populations.	Hadjadj S et al.	17376814	No data
2	Prognostic value of the insertion/deletion polymorphism of the ACE gene in type 2 diabetic subjects: results from the Non-insulin-dependent Diabetes, Hypertension, Microalbuminuria or Proteinuria, Cardiovascular Events, and Ramipril (DIABHYCAR), Diabete de type 2, Nephropathie et Genetique (DIAB2NEP...	Hadjadj S et al.	18523145	No data
2	Angiotensin-converting enzyme gene polymorphism in non-insulin dependent diabetes mellitus and its relationship with diabetic nephropathy.	Jeffers BW et al.	9264004	No data
2	Genetic polymorphisms of the renin-angiotensin-aldosterone system in end-stage renal disease.	Lovati E et al.	11422735	No data
2	Effects of the genetic polymorphisms of the renin-angiotensin system on focal segmental glomerulosclerosis.	Luther Y et al.	14610337	No data
2	The renin--angiotensin system gene polymorphisms and clinicopathological correlations in IgA nephropathy.	Ong-Ajyooth S et al.	10511770	No data

For peer review only

2	Combinational effect of genes for the renin-angiotensin system in conferring susceptibility to diabetic nephropathy.	Osawa N et al.	17143591	No data
2	Association of a uteroglobin polymorphism with rate of progression in patients with IgA nephropathy.	Szelestei T et al.	10977777	No data
2	Lack of synergism between long-term poor glycaemic control and three gene polymorphisms of the renin angiotensin system on risk of developing diabetic nephropathy in type I diabetic patients.	Tarnow L et al.	10907125	No data
2	ACE gene polymorphism and disease progression of IgA nephropathy in Asians in Singapore.	Lau YK et al.	12119485	Overlap in patient group
2	Polymorphism of renin-angiotensin system genes in IgA nephropathy	Woo KT et al.	15504143	Overlap in patient group
3	Angiotensin-converting enzyme (ACE) serum levels and gene polymorphism in Egyptian patients with systemic lupus erythematosus.	Abbas D et al.	21976404	< 3 populations reported per SNP
3	Association of an insertion polymorphism of angiotensin-converting enzyme gene with the activity of lupus nephritis.	Akai Y et al.	10099886	< 3 populations reported per SNP
3	Clinical impact of an angiotensin I-converting enzyme insertion/deletion and kinin B2 receptor +9/-9 polymorphisms in the prognosis of renal transplantation	Amorim et al.	23362199	< 3 populations reported per SNP
3	Lack of association between the angiotensin-converting enzyme gene (I/D) polymorphism and diabetic nephropathy in Tunisian type 2 diabetic patients.	Arfa I et al.	18404607	< 3 populations reported per SNP
3	Polymorphism of the angiotensin-converting enzyme gene in end-stage renal failure patients.	Aucella F et al.	10773756	< 3 populations reported per SNP
3	Polymorphism of the renin–angiotensin–aldosterone system in patients with chronic allograft dysfunction	Ayed K et al.	16635753	< 3 populations reported per SNP
3	The presence of allele D of angiotensin-converting enzyme polymorphism is associated with diabetic nephropathy in patients with less than 10 years duration of Type 2 diabetes.	Canani LH et al.	16108844	< 3 populations reported per SNP

For peer review only

3	Identification of specific angiotensin-converting enzyme variants and haplotypes that confer risk and protection against type 2 diabetic nephropathy.	Ezzidi I et al.	19787680	< 3 populations reported per SNP
3	Genetic polymorphisms of the renin-angiotensin-aldosterone system and renal insufficiency in essential hypertension	Fabris B et al.	15662219	< 3 populations reported per SNP
3	Angiotensin converting enzyme gene I/D polymorphism in essential hypertension and nephroangiosclerosis.	Fernández-Llama P et al.	9607207	< 3 populations reported per SNP
3	DNA polymorphisms in the ACE gene, serum ACE activity and the risk of nephropathy in insulin-dependent diabetes mellitus	Freire MBS et al.	9794558	< 3 populations reported per SNP
3	Association between genetic polymorphisms of ACE & eNOS and diabetic nephropathy.	Huo P et al.	25227524	< 3 populations reported per SNP
3	Genetic Clues To The Etiology Of Balkan Endemic Nephropathy: Investigating The Role Of Ace And At1R Polymorphisms	Krcunovic Z et al.	WOS:000287217500011	< 3 populations reported per SNP
3	Angiotensin-I Converting Enzyme Polymorphism and Diabetic Nephropathy in North India	Kumar A et al.	NA	< 3 populations reported per SNP
3	Angiotensin-converting enzyme gene polymorphism in patients with minimal-change nephrotic syndrome and focal segmental glomerulosclerosis.	Lee DY et al.	9434071	< 3 populations reported per SNP
3	Association of the genetic polymorphisms of the ACE gene and the eNOS gene with lupus nephropathy in northern Chinese population.	Li X et al.	20540812	< 3 populations reported per SNP
3	Genes involved in the regulation of vascular homeostasis determine renal survival rate in patients with chronic glomerulonephritis	Litovkina O et al.	24727057	< 3 populations reported per SNP
3	Contribution of genetic polymorphism in the renin-angiotensin system to the development of renal complications in insulin-dependent diabetes	Marre M et al.	9120002	< 3 populations reported per SNP
3	Influence of the alpha-adducin and ACE gene polymorphism on the progression of autosomal-dominant polycystic kidney disease.	Merta M et al.	12697976	< 3 populations reported per SNP

For peer review only

3	Genetic polymorphism of renin-angiotensin system is not associated with diabetic vascular complications in Japanese subjects with long-term insulin dependent diabetes mellitus.	Miura J et al.	10499884	< 3 populations reported per SNP
3	The effect of polymorphisms in the renin-angiotensin-aldosterone system on diabetic nephropathy risk.	Möllsten A et al.	18413189	< 3 populations reported per SNP
3	[Angiotensin-1 converting enzyme insertion/deletion gene polymorphism in a Mexican population with diabetic nephropathy].	Ortega-Pierres LE et al.	17570179	< 3 populations reported per SNP
3	Influence of genetic variability at the ACE locus in intron 16 on Diabetic Nephropathy in T1DM patients.	Parchwani DN et al.	26214998	< 3 populations reported per SNP
3	Renin-angiotensin system gene polymorphisms predict the progression to renal insufficiency among Asians with lupus nephritis.	Parsa A et al.	15789057	< 3 populations reported per SNP
3	Chronic renal insufficiency among Asian Indians with type 2 diabetes: I. Role of RAAS gene polymorphisms	Prasad P et al.	WOS:000238369400001	< 3 populations reported per SNP
3	Influence of angiotensin converting enzyme (ACE) gene rs4362 polymorphism on the progression of kidney failure in patients with autosomal dominant polycystic kidney disease (ADPKD)	Ramanathan G et al.	27748299	< 3 populations reported per SNP
3	Genetic variants of ACE (Insertion/Deletion) and AGT (M268T) genes in patients with diabetes and nephropathy.	Shaikh R et al.	24737640	< 3 populations reported per SNP
3	Polymorphisms of the renin-angiotensin system genes in Brazilian patients with lupus nephropathy.	Sprovieri SR et al.	15934435	< 3 populations reported per SNP
3	Gene polymorphisms of angiotensin-converting enzyme and angiotensin II Type 1 receptor among chronic kidney disease patients in a Chinese population	Su SL et al.	22147663	< 3 populations reported per SNP
3	Genetic risk factors for renal failure among north Indian ESRD patients.	Tripathi G et al.	18242170	< 3 populations reported per SNP
3	Association between angiotensin converting enzyme gene polymorphism and clinical features in autosomal dominant polycystic kidney disease.	Uemasu J et al.	9180368	< 3 populations reported per SNP
3	The DD genotype of the ACE gene polymorphism is associated with progression of diabetic nephropathy to end stage renal failure in IDDM.	Vleming LJ et al.	10099885	< 3 populations reported per SNP

For peer review only

Supplementary Table S3b: Excluded studies from the *ACE2* search

*Exclusion stage relates to the stage at which the article was removed as shown in the flow diagram (Supplementary Figure S1b)

Exclusion Stage	Title	Authors	Pubmed ID or WoS ID if Pubmed ID not available	Reason
1	Angiotensin converting enzyme gene I/D polymorphism in essential hypertension and nephroangiosclerosis	Fernandez-Llama P et al.	9607207	No data for ACE2
1	Genetic predisposition to Balkan endemic nephropathy: ability to hydroxylate debrisoquine as a host risk factor.	Nikolov IG et al.	1820343	No data for ACE2
1	Immunoglobulin C mu gene restriction fragment length polymorphisms associated with chronic renal failure.	Demaine AG et al.	2985495	No data for ACE2
1	Genetic polymorphism of C3 and Bf in IgA nephropathy.	Rambausek M et al.	3118258	No data for ACE2
1	Factor B subtypes in Japanese patients with IgA nephropathy and with idiopathic membranous nephropathy.	Nishimukai H et al.	3272818	No data for ACE2
1	Major-histocompatibility-complex extended haplotypes in membranoproliferative glomerulonephritis.	Welch TR et al.	3458025	No data for ACE2
1	A simultaneous study of the polymorphism of five proteins in the serum and the urine of nephrotic patients.	Papacostas S et al.	6688659	No data for ACE2
1	Identification of mutants of pyruvate kinase from red blood cells by means of trypsinization, electrophoresis, kinetic properties and immunological methods.	Jacobasch G et al.	7315101	No data for ACE2
1	Angiotensin-converting enzyme polymorphism and development of diabetic nephropathy in non-insulin-dependent diabetes mellitus.	Mizuiri S et al.	7477652	No data for ACE2
1	No association of converting enzyme insertion/deletion polymorphism with immunoglobulin A glomerulonephritis.	Schmidt S et al.	7485124	No data for ACE2
1	Protein loss and genetic polymorphism of apolipoprotein(a) modulate serum lipoprotein(a) in CAPD patients.	Wanner C et al.	7724034	No data for ACE2

For peer review only

1	Lack of relationship between an insertion/deletion polymorphism in the angiotensin I-converting enzyme gene and diabetic nephropathy and proliferative retinopathy in IDDM patients.	Tarnow L et al.	7729604	No data for ACE2
1	Role of glycaemic control in development of microalbuminuria in patients with insulin dependent diabetes.	Powrie JK et al.	7819935	No data for ACE2
1	The N-acetyltransferase (NAT) gene: an early risk marker for diabetic nephropathy in Japanese type 2 diabetic patients?	Neugebauer S et al.	7851073	No data for ACE2
1	Genetic predisposition to diabetic nephropathy. Evidence for a role of the angiotensin I--converting enzyme gene.	Doria A et al.	7909524	No data for ACE2
1	Relationships between angiotensin I converting enzyme gene polymorphism, plasma levels, and diabetic retinal and renal complications.	Marre M et al.	8314010	No data for ACE2
1	Elevated plasma concentrations of lipoprotein(a) in patients with end-stage renal disease are not related to the size polymorphism of apolipoprotein(a).	Dieplinger H et al.	8432847	No data for ACE2
1	Polymorphism of the angiotensin converting enzyme gene and clinical aspects of IgA nephropathy.	Yorioka T et al.	8529313	No data for ACE2
1	Association between angiotensin-converting-enzyme gene polymorphism and failure of renoprotective therapy.	van Essen GG et al.	8538349	No data for ACE2
1	Association analyses of the polymorphisms of angiotensin-converting enzyme and angiotensinogen genes with diabetic nephropathy in Japanese non-insulin-dependent diabetics.	Ohno T et al.	8596493	No data for ACE2
1	Association between a polymorphism in the angiotensin-converting enzyme gene and microvascular complications in Japanese patients with NIDDM.	Doi Y et al.	8720609	No data for ACE2
1	Angiotensin-converting enzyme polymorphism in patients with terminal renal failure.	Schmidt A et al.	8785402	No data for ACE2
1	Relationships between angiotensin I converting enzyme gene polymorphism and renal complications in Korean IDDM patients.	Oh TG et al.	8854649	No data for ACE2

For peer review only

1	The angiotensin I-converting enzyme (ACE) locus is strongly associated with age and duration of diabetes in patients with type I diabetes.	Hibberd ML et al.	9025006	No data for ACE2
1	Hypertensive nephropathy and the gene for angiotensin-converting enzyme.	Kario K et al.	9081678	No data for ACE2
1	Evaluation of risk factors for the development of nephropathy in patients with IDDM: insertion/deletion angiotensin converting enzyme gene polymorphism, hypertension and metabolic control.	Barnas U et al.	9084972	No data for ACE2
1	Association between angiotensin converting enzyme gene polymorphism and clinical features in autosomal dominant polycystic kidney disease.	Uemasu J et al.	9180368	No data for ACE2
1	Angiotensin-converting enzyme genotype and renal allograft survival.	Beige J et al.	9259361	No data for ACE2
1	Association of angiotensinogen gene T235 variant with progression of immunoglobin A nephropathy in Caucasian patients.	Pei Y et al.	9259580	No data for ACE2
1	Angiotensin-converting enzyme gene polymorphism in non-insulin dependent diabetes mellitus and its relationship with diabetic nephropathy.	Jeffers BW et al.	9264004	No data for ACE2
1	Angiotensin I converting enzyme gene polymorphism and diabetic nephropathy in type II diabetes.	Schmidt S et al.	9269698	No data for ACE2
1	Deletion insertion polymorphism of the angiotensin converting enzyme gene and progression of diabetic nephropathy.	Bjorck S et al.	9269704	No data for ACE2
1	Genetic regulation of the impaired immune response to hepatitis-B vaccine associated with low TCR density in end stage renal disease patients: contribution of complement C4 and factor B alleles.	Kramer J et al.	9334852	No data for ACE2
1	Gene-polymorphisms of angiotensin converting enzyme and endothelial nitric oxide synthase in patients with primary glomerulonephritis.	Burg M et al.	9352153	No data for ACE2
1	Angiotensin-converting enzyme gene polymorphism in patients with minimal-change nephrotic syndrome and focal segmental glomerulosclerosis.	Lee DY et al.	9434071	No data for ACE2
1	Genetic variants of microsomal metabolism and susceptibility to hydrocarbon-associated glomerulonephritis.	Pai P et al.	9474350	No data for ACE2

For peer review only

1	Polymorphism of the angiotensin I-converting enzyme gene in diabetic nephropathy in type II diabetic patients with proliferative retinopathy.	Hanyu O et al.	9509566	No data for ACE2
1	Identification of human plasma kallikrein gene polymorphisms and evaluation of their role in end-stage renal disease.	Yu H et al.	9535413	No data for ACE2
1	Relationship of the angiotensin-converting enzyme gene polymorphism to glucose intolerance, insulin resistance, and hypertension in NIDDM.	Huang XH et al.	9544854	No data for ACE2
1	Angiotensin-converting-enzyme insertion/deletion genotype and long-term renal allograft survival.	Beige J et al.	9550656	No data for ACE2
1	A new polymorphic restriction site in the human 11 beta-hydroxysteroid dehydrogenase type 2 gene.	Smolenicka Z et al.	9589699	No data for ACE2
1	Angiotensin I converting enzyme gene polymorphisms in systemic lupus erythematosus: decreased prevalence of DD genotype in African American patients.	Tassiulas IO et al.	9710341	No data for ACE2
1	Angiotensin I-converting enzyme gene polymorphisms: relationship to nephropathy in patients with non-insulin dependent diabetes mellitus.	Grzeszczak W et al.	9727375	No data for ACE2
1	The serum paraoxonase activity in patients with chronic renal failure and hyperlipidemia.	Paragh G et al.	9736814	No data for ACE2
1	Homocysteine, B vitamins, and vascular-access thrombosis in patients treated with hemodialysis.	Tamura T et al.	9740165	No data for ACE2
1	DNA polymorphisms in the ACE gene, serum ACE activity and the risk of nephropathy in insulin-dependent diabetes mellitus.	Freire MB et al.	9794558	No data for ACE2
1	Insertion/deletion polymorphism in intron 16 of the ACE gene and left ventricular hypertrophy in patients with end-stage renal disease.	Osono E et al.	9820440	No data for ACE2
1	The C825T polymorphism in the human G-protein beta3 subunit gene is not associated with diabetic nephropathy in Type I diabetes mellitus.	Fogarty DG et al.	9833937	No data for ACE2
1	Effect of ACE gene on diabetic nephropathy in NIDDM patients with insulin resistance.	Kuramoto N et al.	10023638	No data for ACE2
1	The DD genotype of the ACE gene polymorphism is associated with progression of diabetic nephropathy to end stage renal failure in IDDM.	Vleming LJ et al.	10099885	No data for ACE2

For peer review only

1	Increased frequency of G-protein beta 3-subunit 825 T allele in dialyzed patients with type 2 diabetes.	Bluthner M et al.	10200987	No data for ACE2
1	Lack of association of angiotensin-converting enzyme (DD/II) and angiotensinogen M235T gene polymorphism with renal function among Chinese patients with type II diabetes.	Wong TY et al.	10352194	No data for ACE2
1	Angiotensin I-converting enzyme genotype significantly affects progression of IgA glomerulonephritis in an italian population.	Stratta P et al.	10352195	No data for ACE2
1	Lipoprotein(a) and apolipoprotein(a) isoforms and proteinuria in patients with moderate renal failure.	Sechi LA et al.	10469373	No data for ACE2
1	ACE, PAI-1, decorin and Werner helicase genes are not associated with the development of renal disease in European patients with type 1 diabetes.	De Cosmo S et al.	10495473	No data for ACE2
1	The renin--angiotensin system gene polymorphisms and clinicopathological correlations in IgA nephropathy.	Ong-Ajyooth S et al.	10511770	No data for ACE2
1	Effect of a polymorphism of endothelial nitric oxide synthase gene in Japanese patients with IgA nephropathy.	Morita T et al.	10543322	No data for ACE2
1	Endothelial nitric oxide synthase gene polymorphism in intron 4 affects the progression of renal failure in non-diabetic renal diseases.	Wang Y et al.	10570094	No data for ACE2
1	Risk factors for the progression of microalbuminuria in Japanese type 2 diabetic patients--a 10 year follow-up study.	Oue T et al.	10580616	No data for ACE2
1	Chronic proteinuric nephropathies. II. Outcomes and response to treatment in a prospective cohort of 352 patients: differences between women and men in relation to the ACE gene polymorphism. Gruppo Italiano di Studi Epidemologici in Nefrologia (Gisen)	Ruggenenti P et al.	10616844	No data for ACE2
1	Risk of advanced diabetic nephropathy in type 1 diabetes is associated with endothelial nitric oxide synthase gene polymorphism.	Zanchi A et al.	10652017	No data for ACE2
1	HDL cholesterol and TaqIB cholesteryl ester transfer protein gene polymorphism in renal transplant recipients.	Radeau T et al.	10754410	No data for ACE2

For peer review only

1	Polymorphism of the angiotensin-converting enzyme gene in end-stage renal failure patients.	Aucella F et al.	10773756	No data for ACE2
1	The ACE insertion/deletion polymorphism has no influence on progression of renal function loss in autosomal dominant polycystic kidney disease.	van Dijk MA et al.	10831637	No data for ACE2
1	Increased frequency of angiotensin-converting enzyme DD genotype in patients with type 2 diabetes in Taiwan.	Hsieh MC et al.	10862639	No data for ACE2
1	Association of the nitric oxide synthase gene polymorphism with an increased risk for progression to diabetic nephropathy in type 2 diabetes.	Neugebauer S et al.	10868974	No data for ACE2
1	Lack of synergism between long-term poor glycaemic control and three gene polymorphisms of the renin angiotensin system on risk of developing diabetic nephropathy in type I diabetic patients.	Tarnow L et al.	10907125	No data for ACE2
1	Differential expression of cyclin-dependent kinase inhibitors in human glomerular disease: role in podocyte proliferation and maturation.	Shankland SJ et al.	10916090	No data for ACE2
1	G-Protein beta(3) subunit C825T variant, nephropathy and hypertension in patients with type 2 (Non-insulin-dependent) diabetes mellitus.	Zychma MJ et al.	10970984	No data for ACE2
1	Association of a uteroglobin polymorphism with rate of progression in patients with IgA nephropathy.	Szelestei T et al.	10977777	No data for ACE2
1	Deregulated platelet-activating factor levels and acetylhydrolase activity in patients with idiopathic IgA nephropathy.	Denizot Y et al.	10978389	No data for ACE2
1	Structural analysis of the 11beta-hydroxysteroid dehydrogenase type 2 gene in end-stage renal disease.	Zaehner T et al.	11012876	No data for ACE2
1	Polymorphism of angiotensin converting enzyme, angiotensinogen, and angiotensin II type 1 receptor genes and end-stage renal failure in IgA nephropathy: IGARAS--a study of 274 Men.	Frimat L et al.	11053482	No data for ACE2
1	Genetic analysis of nitric oxide and endothelin in end-stage renal disease.	Freedman BI et al.	11071967	No data for ACE2
1	Angiotensinogen M235T and chymase gene CMA/B polymorphisms are not associated with nephropathy in type II diabetes.	Zychma MJ et al.	11096141	No data for ACE2

For peer review only

1	Association between a variant in the 11 beta-hydroxysteroid dehydrogenase type 2 gene and primary hypertension.	Melander O et al.	11114699	No data for ACE2
1	Polymorphisms of human paraoxonase 1 gene (PON1) and susceptibility to diabetic nephropathy in type I diabetes mellitus.	Araki S et al.	11151764	No data for ACE2
1	Posttransplantation relapse of FSGS is characterized by glomerular epithelial cell transdifferentiation.	Bariety J et al.	11158216	No data for ACE2
1	Membranous nephropathy, hydrocarbon exposure and genetic variants of hydrocarbon detoxification.	Gradden CW et al.	11181983	No data for ACE2
1	Albuminuria and the renin-angiotensin system gene polymorphisms in type-2-diabetic and in normoglycemic hypertensive Chinese.	Thomas GN et al.	11200871	No data for ACE2
1	Paraoxonase2 polymorphisms are associated with nephropathy in Type II diabetes.	Pinizzotto M et al.	11206400	No data for ACE2
1	ACE gene polymorphism and long-term renal graft function.	Viklicky O et al.	11239522	No data for ACE2
1	The C825T polymorphism in the G-protein beta3 subunit gene and diabetic complications in IDDM patients.	Shcherbak NS et al.	11322658	No data for ACE2
1	Polymorphisms in the hANP (human atrial natriuretic peptide) gene, albuminuria, and hypertension.	Nannipieri M et al.	11408388	No data for ACE2
1	Genetic polymorphisms of the renin-angiotensin-aldosterone system in end-stage renal disease.	Lovati E et al.	11422735	No data for ACE2
1	The DD genotype of the ACE gene polymorphism is associated with diabetic nephropathy in the type-1 diabetics.	Azar ST et al.	11428725	No data for ACE2
1	Contribution of gene polymorphisms in the renin-angiotensin system to macroangiopathy in patients with diabetic nephropathy.	Wong TY et al.	11431175	No data for ACE2
1	Catalase/superoxide dismutase (SOD) and catalase/paraoxonase (PON) ratios may implicate poor glycemic control.	Sozmen EY et al.	11440784	No data for ACE2
1	Serum paraoxonase and arylesterase activities in hemodialysis patients.	Itahara T et al.	11480456	No data for ACE2
1	Influence of BsmI vitamin D receptor gene polymorphism on the response to a single bolus of calcitrol in hemodialysis patients.	Marco MP et al.	11522087	No data for ACE2

For peer review only

1	Is there a role of angiotensin-converting enzyme gene polymorphism in the failure of arteriovenous femoral shunts for hemodialysis?	Isbir CS et al.	11525534	No data for ACE2
1	Endothelial nitric oxide synthase intron 4 polymorphism influences the progression of renal disease.	Asakimori Y et al.	11549906	No data for ACE2
1	Dinucleotide repeat polymorphism of matrix metalloproteinase-9 gene is associated with diabetic nephropathy.	Maeda S et al.	11576356	No data for ACE2
1	Angiotensin-converting enzyme gene polymorphism and microvascular complications in Turkish type 2 diabetic patients.	Araz M et al.	11640993	No data for ACE2
1	Effect of human OGG1 1245C-->G gene polymorphism on 8-hydroxy-2'-deoxyguanosine levels of leukocyte DNA among patients undergoing chronic hemodialysis.	Tarng DC et al.	11675410	No data for ACE2
1	Association of the DD genotype and development of Japanese type 2 diabetic nephropathy.	Gohda T et al.	11770799	No data for ACE2
1	Different impact of deletion polymorphism of gene on the risk of renal and coronary artery disease.	Olivieri O et al.	11791024	No data for ACE2
1	Gene mutations in lymphoproliferative disorders of T and NK/T cell phenotypes developing in renal transplant patients.	Hoshida Y et al.	11896204	No data for ACE2
1	Association of a functional inducible nitric oxide synthase promoter variant with complications in type 2 diabetes.	Morris BJ et al.	11907646	No data for ACE2
1	Association studies between the HSD11B2 gene (encoding human 11beta-hydroxysteroid dehydrogenase type 2), type 1 diabetes mellitus and diabetic nephropathy.	Lavery GG et al.	11916625	No data for ACE2
1	Polymorphism in ecto-nucleotide pyrophosphatase/phosphodiesterase 1 gene (ENPP1/PC-1) and early development of advanced diabetic nephropathy in type 1 diabetes.	Canani LH et al.	11916943	No data for ACE2
1	Role of the alpha-adducin genotype on renal disease progression.	Nicod J et al.	11918733	No data for ACE2
1	Hypertension after renal transplantation and polymorphism of genes involved in essential hypertension: ACE, AGT, AT1 R and ecNOS.	Basset el-EA et al.	11926202	No data for ACE2

For peer review only

1	ACE gene insertion/deletion polymorphism associated with 1998 World Health Organization definition of metabolic syndrome in Chinese type 2 diabetic patients.	Lee YJ et al.	12032106	No data for ACE2
1	Angiotensin-converting enzyme gene polymorphism and vascular manifestations in Korean patients with SLE.	Uhm WS et al.	12043886	No data for ACE2
1	Parathyroid hormone gene polymorphism and secondary hyperparathyroidism in hemodialysis patients.	Gohda T et al.	12046039	No data for ACE2
1	Endothelial nitric oxide synthase affects the progression of autosomal dominant polycystic kidney disease.	Reiterova J et al.	12077489	No data for ACE2
1	ACE gene polymorphism and disease progression of IgA nephropathy in Asians in Singapore.	Lau YK et al.	12119485	No data for ACE2
1	T(-786)-->C polymorphism of the endothelial nitric oxide synthase gene influences the progression of renal disease.	Asakimori Y et al.	12138283	No data for ACE2
1	The role of PC-1 and ACE genes in diabetic nephropathy in type 1 diabetic patients: evidence for a polygenic control of kidney disease progression.	De Cosmo S et al.	12147786	No data for ACE2
1	Nephropathy in type 1 diabetes: a manifestation of insulin resistance and multiple genetic susceptibilities? Further evidence from the Pittsburgh Epidemiology of Diabetes Complication Study.	Orchard TJ et al.	12164879	No data for ACE2
1	Influence of the endothelial nitric oxide synthase polymorphism on the progression of autosomal dominant polycystic kidney disease and IgA nephropathy.	Merta M et al.	12212826	No data for ACE2
1	Serum paraoxonase (PON1) concentration in patients undergoing hemodialysis.	Suehiro T et al.	12226554	No data for ACE2
1	Genetic determinants of delayed graft function after kidney transplantation.	St Peter SD et al.	12364860	No data for ACE2
1	Endothelial nitric oxide synthase gene polymorphism in dialysis patients.	de Prado A et al.	12402580	No data for ACE2
1	Endothelial nitric oxide synthase gene and the development of diabetic nephropathy.	Shimizu T et al.	12413777	No data for ACE2

For peer review only

1	G-protein beta-3-subunit and eNOS gene polymorphism in transplant recipients with long-term renal graft function.	Viklicky O et al.	12424427	No data for ACE2
1	Microfluidic chip-based method for genotyping microsatellites, VNTRs and insertion/deletion polymorphisms.	Sohni YR et al.	12554058	No data for ACE2
1	The role of ACE gene polymorphism in rapidity of progression of focal segmental glomerulosclerosis.	Dixit M et al.	12571380	No data for ACE2
1	The response of antioxidant genes to hyperglycemia is abnormal in patients with type 1 diabetes and diabetic nephropathy.	Hodgkinson AD et al.	12606529	No data for ACE2
1	Epidermal growth factor receptor polymorphism and autosomal dominant polycystic kidney disease.	Magistroni R et al.	12653106	No data for ACE2
1	Cardiac hypertrophy and remodeling in relation to ACE and angiotensinogen genes genotypes in Chinese dialysis patients.	Wang AY et al.	12675870	No data for ACE2
1	Nitric oxide synthase gene polymorphisms and diabetic nephropathy.	Rippin JD et al.	12687343	No data for ACE2
1	Influence of the alpha-adducin and ACE gene polymorphism on the progression of autosomal-dominant polycystic kidney disease.	Merta M et al.	12697976	No data for ACE2
1	Association of ecNOS gene polymorphisms with end stage renal diseases.	Nagase S et al.	12701818	No data for ACE2
1	PAI-1 4G/5G and ACE I/D gene polymorphisms and the occurrence of myocardial infarction in patients on intermittent dialysis.	Aucella F et al.	12748347	No data for ACE2
1	Paraoxonase activity and paraoxonase 1 gene polymorphism in patients with uremia.	Biasioli S et al.	12790379	No data for ACE2
1	Evaluation of genetic variation and association in the matrix metalloproteinase 9 (MMP9) gene in ESRD patients.	Hirakawa S et al.	12830465	No data for ACE2
1	Angiotensin converting enzyme gene polymorphism and development of post-transplant erythrocytosis.	Yildiz A et al.	12832741	No data for ACE2
1	Identification of a common risk haplotype for diabetic nephropathy at the protein kinase C-beta1 (PRKCB1) gene locus.	Araki S et al.	12874455	No data for ACE2
1	Genotypic and phenotypic properties of coagulase-negative staphylococci causing dialysis catheter-related sepsis.	Spare MK et al.	12919757	No data for ACE2

For peer review only

1	Elevation of IgG antibodies against tissue transglutaminase as a diagnostic tool for coeliac disease in selective IgA deficiency.	Korponay-Szabo IR et al.	14570724	No data for ACE2
1	Association of the p22phox component of NAD(P)H oxidase with susceptibility to diabetic nephropathy in patients with type 1 diabetes.	Hodgkinson AD et al.	14578247	No data for ACE2
1	Effects of the genetic polymorphisms of the renin-angiotensin system on focal segmental glomerulosclerosis.	Luther Y et al.	14610337	No data for ACE2
1	Interaction between gene polymorphisms of nitric oxide synthase and renin-angiotensin system in the progression of membranous glomerulonephritis.	Stratta P et al.	14767013	No data for ACE2
1	Angiotensin-converting enzyme polymorphism gene and evolution of nephropathy to end-stage renal disease.	Ortiz MA et al.	15012717	No data for ACE2
1	Angiotensin-converting enzyme genotype is a predictive factor in the peak panel-reactive antibody response.	Akcay A et al.	15013293	No data for ACE2
1	Quantitative polymorphism of complement receptor type 1 (CR1) in patients undergoing haemodialysis.	Tamano M et al.	15069174	No data for ACE2
1	Renin-angiotensin system gene polymorphisms: its impact on IgAN and its progression to end-stage renal failure among Chinese in Singapore.	Lau YK et al.	15153745	No data for ACE2
1	Role of alpha-adducin DNA polymorphisms in the genetic predisposition to diabetic nephropathy.	Conway BR et al.	15187197	No data for ACE2
1	Estrogen receptor is significantly associated with the epithelioid variants of renal angiomyolipoma: a clinicopathological and immunohistochemical study of 67 cases.	Cho NH et al.	15189505	No data for ACE2
1	Paraoxonase 192 polymorphism and its relationship to serum lipids in Turkish renal transplant recipients.	Agachan B et al.	15251338	No data for ACE2
1	Angiotensin converting enzyme genotype and chronic allograft nephropathy in protocol biopsies.	Hueso M et al.	15284309	No data for ACE2
1	The influence of G-protein beta3-subunit gene and endothelial nitric oxide synthase gene in exon 7 polymorphisms on progression of autosomal dominant polycystic kidney disease.	Reiterova J et al.	15287194	No data for ACE2

For peer review only

1	Endothelial nitric oxide synthase gene intron 4 polymorphism in patients with end-stage renal disease.	Buraczynska M et al.	15299097	No data for ACE2
1	Low prevalence of nonconservative mutations of serum and glucocorticoid-regulated kinase (SGK1) gene in hypertensive and renal patients.	Trochen N et al.	15304560	No data for ACE2
1	Relations between eNOS Glu298Asp polymorphism and progression of diabetic nephropathy.	Shin Shin Y et al.	15331206	No data for ACE2
1	Angiotensin-converting enzyme (insertion/deletion) and endothelial nitric oxide synthase polymorphisms in patients with systemic lupus erythematosus.	Douglas G et al.	15338496	No data for ACE2
1	Angiotensin-I converting enzyme gene polymorphism in Turkish type 2 diabetic patients.	Arzu Ergen H et al.	15365253	No data for ACE2
1	Identification of NQO1 and GSTs genotype frequencies in Bulgarian patients with Balkan endemic nephropathy.	Toncheva DI et al.	15365958	No data for ACE2
1	Balkan endemic nephropathy and genetic variants of glutathione S-transferases.	Andonova IE et al.	15365959	No data for ACE2
1	Association of the genetic polymorphisms of the renin-angiotensin system and endothelial nitric oxide synthase with chronic renal transplant dysfunction.	Akcay A et al.	15385810	No data for ACE2
1	Genetic polymorphisms of cytochrome P450 among patients with Balkan endemic nephropathy (BEN).	Atanasova SY et al.	15708542	No data for ACE2
1	Evidence for association of endothelial cell nitric oxide synthase gene polymorphism with earlier progression to end-stage renal disease in a cohort of Hellens from Greece and Cyprus.	Lamnissou K et al.	15727257	No data for ACE2
1	Mutation analysis of autosomal dominant polycystic kidney disease genes in Han Chinese.	Zhang S et al.	15775720	No data for ACE2
1	Genetic variation at the ACE gene is associated with persistent microalbuminuria and severe nephropathy in type 1 diabetes: the DCCT/EDIC Genetics Study.	Boright AP et al.	15793268	No data for ACE2

For peer review only

1	Nitric oxide- and EDHF-mediated arteriolar tone in uremia is unaffected by selective inhibition of vascular cytochrome P450 2C9.	Passauer J et al.	15840038	No data for ACE2
1	Association between vitamin D receptor FokI. Polymorphism and serum parathyroid hormone level in patients with chronic renal failure.	Vigo Gago E et al.	15887856	No data for ACE2
1	Polymorphisms of the renin-angiotensin system genes in Brazilian patients with lupus nephropathy.	Sprovieri SR et al.	15934435	No data for ACE2
1	Effect of hepatic lipase -514C->T polymorphism and its interactions with apolipoprotein C3 -482C->T and apolipoprotein E exon 4 polymorphisms on the risk of nephropathy in chinese type 2 diabetic patients.	Baum L et al.	15983323	No data for ACE2
1	The impact of thiopurine s-methyltransferase polymorphism on azathioprine-induced myelotoxicity in renal transplant recipients.	Kurzawski M et al.	16044099	No data for ACE2
1	Carnosine as a protective factor in diabetic nephropathy: association with a leucine repeat of the carnosinase gene CNDP1.	Janssen B et al.	16046297	No data for ACE2
1	Impact of polymorphisms in the genes encoding xylosyltransferase I and a homologue in type 1 diabetic patients with and without nephropathy.	Schon S et al.	16164625	No data for ACE2
1	Frequency of angiotensin-converting enzyme gene polymorphism in Turkish type 2 diabetic patients.	Degirmenci I et al.	16178979	No data for ACE2
1	Haplotype analysis of NAD(P)H oxidase p22 phox polymorphisms in end-stage renal disease.	Doi K et al.	16215641	No data for ACE2
1	The effect of angiotensin receptor blockade ARB on the regression of left ventricular hypertrophy in hemodialysis patients: comparison between patients with D allele and non-D allele ACE gene polymorphism.	Nakayama M et al.	16312263	No data for ACE2
1	Manganese superoxide dismutase gene polymorphism (V16A) is associated with stages of albuminuria in Korean type 2 diabetic patients.	Lee SJ et al.	16324912	No data for ACE2
1	Hidden population substructures in an apparently homogeneous population bias association studies.	Berger M et al.	16333311	No data for ACE2
1	Relationship of p22phox C242T polymorphism with nephropathy in type 2 diabetic patients.	Santos KG et al.	16358232	No data for ACE2

For peer review only

1	Genetic polymorphisms of the renin-angiotensin system in end-stage renal disease.	Buraczynska M et al.	16384824	No data for ACE2
1	Angiotensin-converting enzyme (ACE) inhibition in type 2, diabetic patients-- interaction with ACE insertion/deletion polymorphism.	So WY et al.	16395257	No data for ACE2
1	The influence of the endothelin-converting enzyme-1 gene polymorphism on the progression of autosomal dominant polycystic kidney disease.	Reiterova J et al.	16526315	No data for ACE2
1	Aldosterone synthase (CYP11B2) -344T/C polymorphism is not associated with the initiation and progression of diabetic nephropathy in Caucasian Type 1 diabetic patients.	Lajer M et al.	16759311	No data for ACE2
1	Novel sequence variants in the human xylosyltransferase I gene and their role in diabetic nephropathy.	Bahr C et al.	16759312	No data for ACE2
1	Relationship between common functional polymorphisms of the p22phox gene (-930A > G and +242C > T) and nephropathy as a result of Type 2 diabetes in a Chinese population.	Lim SC et al.	16922713	No data for ACE2
1	A disease haplotype for advanced nephropathy in type 2 diabetes at the ACE locus.	Ng DP et al.	16936219	No data for ACE2
1	Efficient screening method of the thiopurine methyltransferase polymorphisms for patients considering taking thiopurine drugs in a Chinese Han population in Henan Province (central China).	Zhang LR et al.	16952345	No data for ACE2
1	Kinin-dependent hypersensitivity reactions in hemodialysis: metabolic and genetic factors.	Molinaro G et al.	17003818	No data for ACE2
1	High prevalence of ACE DD genotype among north Indian end stage renal disease patients.	Tripathi G et al.	17042963	No data for ACE2
1	Sirolimus population pharmacokinetic/pharmacogenetic analysis and bayesian modelling in kidney transplant recipients.	Djebli N et al.	17048977	No data for ACE2
1	Glu298Asp and NOS4ab polymorphisms in diabetic nephropathy.	Mollsten A et al.	17101543	No data for ACE2

For peer review only

1	Combinational effect of genes for the renin-angiotensin system in conferring susceptibility to diabetic nephropathy.	Osawa N et al.	17143591	No data for ACE2
1	The emergence of cytomegalovirus resistance to ganciclovir therapy in kidney transplant recipients.	Nogueira E et al.	17161359	No data for ACE2
1	Paraoxonase gene polymorphism and serum activity in progressive IgA nephropathy.	Kovacs TJ et al.	17173245	No data for ACE2
1	A functional polymorphism in the manganese superoxide dismutase gene and diabetic nephropathy.	Mollsten A et al.	17192491	No data for ACE2
1	A leucine repeat in the carnosinase gene CNDP1 is associated with diabetic end-stage renal disease in European Americans.	Freedman BI et al.	17205963	No data for ACE2
1	Variants of C1GALT1 gene are associated with the genetic susceptibility to IgA nephropathy.	Li GS et al.	17228361	No data for ACE2
1	Resequencing of genes for transforming growth factor beta1 (TGFB1) type 1 and 2 receptors (TGFBR1, TGFBR2), and association analysis of variants with diabetic nephropathy.	McKnight AJ et al.	17319955	No data for ACE2
1	Association between angiotensin-converting enzyme gene polymorphisms and diabetic nephropathy: case-control, haplotype, and family-based study in three European populations.	Hadjadj S et al.	17376814	No data for ACE2
1	Association of endothelial nitric oxide synthase gene intron 4 polymorphism with end-stage renal disease.	Bellini MH et al.	17498125	No data for ACE2
1	[Angiotensin-1 converting enzyme insertion/deletion gene polymorphism in a Mexican population with diabetic nephropathy].	Ortega-Pierres LE et al.	17570179	No data for ACE2
1	Disease progression, response to ACEI/ATRA therapy and influence of ACE gene in IgA nephritis.	Woo KT et al.	17601378	No data for ACE2
1	Relationship of angiotensin-converting enzyme gene polymorphism with nephropathy associated with Type 2 diabetes mellitus in Asian Indians.	Movva S et al.	17616353	No data for ACE2
1	Impact of ENPP1 genotype on arterial calcification in patients with end-stage renal failure.	Eller P et al.	17848394	No data for ACE2

For peer review only

1	Multiple superoxide dismutase 1/splicing factor serine alanine 15 variants are associated with the development and progression of diabetic nephropathy: the Diabetes Control and Complications Trial/Epidemiology of Diabetes Interventions and Complications Genetics study.	Al-Kateb H et al.	17914031	No data for ACE2
1	Association between vitamin D receptor gene polymorphisms and susceptibility to chronic kidney disease and periodontitis.	de Souza CM et al.	17914260	No data for ACE2
1	Gene polymorphisms of the renin-angiotensin-aldosterone system and angiotensin II type 1-receptor activating antibodies in renal rejection.	Zhang G et al.	17984617	No data for ACE2
1	Correlates of ACE activity in macroalbuminuric type 2 diabetic patients treated with chronic ACE inhibition.	Nikzamir A et al.	17986476	No data for ACE2
1	Genotyping with a dried blood spot method: a useful technique for application in pharmacogenetics.	Wijnen PA et al.	18028890	No data for ACE2
1	Relationship between GSTs gene polymorphism and susceptibility to end stage renal disease among North Indians.	Agrawal S et al.	18067039	No data for ACE2
1	Polymorphism of the endothelial nitric oxide synthase gene is associated with diabetic retinopathy in a cohort of West Africans.	Chen Y et al.	18079690	No data for ACE2
1	Association of the distal region of the ectonucleotide pyrophosphatase/phosphodiesterase 1 gene with type 2 diabetes in an African-American population enriched for nephropathy.	Keene KL et al.	18184924	No data for ACE2
1	Genetic risk factors for renal failure among north Indian ESRD patients.	Tripathi G et al.	18242170	No data for ACE2
1	Donor DNA is detected in recipient blood for years after kidney transplantation using sensitive forensic medicine methods.	Rutkowska J et al.	18290564	No data for ACE2
1	Anti-glutathione S-transferase T1 antibody-mediated rejection in C4d-positive renal allograft recipients.	Aguilera I et al.	18308775	No data for ACE2
1	Endothelial nitric oxide synthase polymorphisms are associated with hypertension and cardiovascular disease in renal transplantation.	Bhandary UV et al.	18331440	No data for ACE2
1	Endothelial nitric oxide synthase gene haplotypes and diabetic nephropathy among Asian Indians.	Ahluwalia TS et al.	18401556	No data for ACE2

For peer review only

1	Lack of association between the angiotensin-converting enzyme gene (I/D) polymorphism and diabetic nephropathy in Tunisian type 2 diabetic patients.	Arfa I et al.	18404607	No data for ACE2
1	Association of the angiotensinogen M235T and angiotensin-converting enzyme insertion/deletion gene polymorphisms in Turkish type 2 diabetic patients with and without nephropathy.	Eroglu Z et al.	18413162	No data for ACE2
1	Oxidative stress pathway genes and chronic renal insufficiency in Asian Indians with Type 2 diabetes.	Tiwari AK et al.	18413200	No data for ACE2
1	Association of endothelial nitric oxide synthase Glu298Asp, 4b/a, and -786T>C gene variants with diabetic nephropathy.	Ezzidi I et al.	18413207	No data for ACE2
1	The number of activating KIR genes inversely correlates with the rate of CMV infection/reactivation in kidney transplant recipients.	Stern M et al.	18444913	No data for ACE2
1	Prognostic value of the insertion/deletion polymorphism of the ACE gene in type 2 diabetic subjects: results from the Non-insulin-dependent Diabetes, Hypertension, Microalbuminuria or Proteinuria, Cardiovascular Events, and Ramipril (DIABHYCAR), Diabete de type 2, Nephropathie et Genetique (DIAB2NEP...	Hadjadj S et al.	18523145	No data for ACE2
1	Angiotensin-converting enzyme inhibitor versus angiotensin 2 receptor antagonist therapy and the influence of angiotensin-converting enzyme gene polymorphism in IgA nephritis.	Woo KT et al.	18536822	No data for ACE2
1	Calpain 10 SNP-44 gene polymorphism affects susceptibility to type 2 diabetes mellitus and diabetic-related conditions.	Demirci H et al.	18554168	No data for ACE2
1	Association of angiotensin-converting enzyme and endothelial Nitric Oxide synthase gene polymorphisms with vascular disease in ESRD patients in a Chinese population.	Tang FY et al.	18629615	No data for ACE2
1	Exclusion of polymorphisms in carnosinase genes (CNDP1 and CNDP2) as a cause of diabetic nephropathy in type 1 diabetes: results of large case-control and follow-up studies.	Wanic K et al.	18753673	No data for ACE2

For peer review only

1	Polymorphism of the aldosterone synthase gene is not associated with progression of diabetic nephropathy, but associated with hypertension in type 2 diabetic patients.	Ko GJ et al.	18771471	No data for ACE2
1	Association of endothelial nitric oxide synthase Glu298Asp polymorphism with end-stage renal disease.	Thaha M et al.	18793530	No data for ACE2
1	MYH9 is associated with nondiabetic end-stage renal disease in African Americans.	Kao WH et al.	18794854	No data for ACE2
1	MYH9 is a major-effect risk gene for focal segmental glomerulosclerosis.	Kopp JB et al.	18794856	No data for ACE2
1	Characterization of the transcriptional regulation of the human MT1-MMP gene and association of risk reduction for focal-segmental glomerulosclerosis with two functional promoter SNPs.	Munkert A et al.	18927121	No data for ACE2
1	The manganese superoxide dismutase Val16Ala polymorphism is associated with decreased risk of diabetic nephropathy in Chinese patients with type 2 diabetes.	Liu L et al.	18989629	No data for ACE2
1	Survival in type 2 diabetic patients in dialysis and the number of risk alleles in polymorphisms of the renin-angiotensin system genes.	Padro-Miquel A et al.	19014923	No data for ACE2
1	ACE genotype, body weight changes and target organ damage in renal transplant recipients.	Stratta P et al.	19034872	No data for ACE2
1	Relationships between thiopurine S-methyltransferase polymorphism and azathioprine-related adverse drug reactions in Chinese renal transplant recipients.	Xin HW et al.	19048245	No data for ACE2
1	Influence of CYP3A5 genetic polymorphism on tacrolimus daily dose requirements and acute rejection in renal graft recipients.	Quteineh L et al.	19067682	No data for ACE2
1	Tripterygium wilfordii hook f increase the blood concentration of tacrolimus.	Wen J et al.	19100464	No data for ACE2
1	ACE variants interact with the RAS pathway to confer risk and protection against type 2 diabetic nephropathy.	Ahluwalia TS et al.	19108684	No data for ACE2
1	A novel method for monitoring glucocorticoid-induced changes of the glucocorticoid receptor in kidney transplant recipients.	Chen Y et al.	19162184	No data for ACE2

For peer review only

1	Polymorphisms in the non-muscle myosin heavy chain 9 gene (MYH9) are strongly associated with end-stage renal disease historically attributed to hypertension in African Americans.	Freedman BI et al.	19177153	No data for ACE2
1	Matrix metalloproteinase-1 and matrix metalloproteinase-3 gene promoter polymorphisms are associated with mortality in haemodialysis patients.	Cozzolino M et al.	19221176	No data for ACE2
1	TPMT but not ITPA gene polymorphism influences the risk of azathioprine intolerance in renal transplant recipients.	Kurzawski M et al.	19229528	No data for ACE2
1	Genetic variant of C1GalT1 contributes to the susceptibility to IgA nephropathy.	Pirulli D et al.	19229831	No data for ACE2
1	The endothelial nitric oxide synthase gene and risk of diabetic nephropathy and development of cardiovascular disease in type 1 diabetes.	Mollsten A et al.	19246226	No data for ACE2
1	Association of genetic variants with chronic kidney disease in Japanese individuals with type 2 diabetes mellitus.	Yoshida T et al.	19288030	No data for ACE2
1	The PTPN22 C1858T (R620W) functional polymorphism in kidney transplantation.	Sfar I et al.	19328948	No data for ACE2
1	Elevated MBL concentrations are not an indication of association between the MBL2 gene and type 1 diabetes or diabetic nephropathy.	Kaunisto MA et al.	19366862	No data for ACE2
1	The influence of carnosinase gene polymorphisms on diabetic nephropathy risk in African-Americans.	McDonough CW et al.	19373489	No data for ACE2
1	Endothelial nitric oxide synthetase, methylenetetrahydrofolate reductase polymorphisms, and cardiovascular complications in Tunisian patients with nondiabetic renal disease.	Kerkeni M et al.	19376104	No data for ACE2
1	Association of genetic variants with chronic kidney disease in Japanese individuals.	Yoshida T et al.	19406964	No data for ACE2
1	Microarray analysis of multiple candidate genes and associated plasma proteins for nephropathy secondary to type 2 diabetes among Chinese individuals.	Lim SC et al.	19415232	No data for ACE2

For peer review only

1	Association of gene polymorphisms with chronic kidney disease in high- or low-risk subjects defined by conventional risk factors.	Yoshida T et al.	19424605	No data for ACE2
1	Influence of genetic polymorphisms in GSTM1, GSTM3, GSTT1 and GSTP1 on allograft outcome in renal transplant recipients.	Singh R et al.	19486347	No data for ACE2
1	Association and interaction analyses of genetic variants in ADIPOQ, ENPP1, GHSR, PPARgamma and TCF7L2 genes for diabetic nephropathy in a Taiwanese population with type 2 diabetes.	Wu LS et al.	19506043	No data for ACE2
1	Non-muscle myosin heavy chain 9 gene MYH9 associations in African Americans with clinically diagnosed type 2 diabetes mellitus-associated ESRD.	Freedman BI et al.	19567477	No data for ACE2
1	Association of genetic variants with chronic kidney disease in individuals with different lipid profiles.	Yoshida T et al.	19578796	No data for ACE2
1	A HindIII polymorphism of fibronectin gene is associated with nephrolithiasis.	Onaran M et al.	19616291	No data for ACE2
1	Impact of genetic polymorphisms of the renin-angiotensin system and of non-genetic factors on kidney transplant function--a single-center experience.	Siekierka-Harreis M et al.	19681973	No data for ACE2
1	Association between inosine triphosphate pyrophosphohydrolase deficiency and azathioprine-related adverse drug reactions in the Chinese kidney transplant recipients.	Xiong H et al.	19682085	No data for ACE2
1	The prevalence of uridine diphosphate-glucuronosyltransferase 1A9 (UGT1A9) gene promoter region single-nucleotide polymorphisms T-275A and C-2152T and its influence on mycophenolic acid pharmacokinetics in stable renal transplant patients.	Sanchez-Fructuoso AI et al.	19715905	No data for ACE2
1	Association of gene polymorphisms with chronic kidney disease in Japanese individuals.	Yoshida T et al.	19724895	No data for ACE2
1	A rare haplotype of the vitamin D receptor gene is protective against diabetic nephropathy.	Martin RJ et al.	19783860	No data for ACE2

For peer review only

1	Identification of specific angiotensin-converting enzyme variants and haplotypes that confer risk and protection against type 2 diabetic nephropathy.	Ezzidi I et al.	19787680	No data for ACE2
1	XbaI GLUT1 gene polymorphism and the risk of type 2 diabetes with nephropathy.	Stefanidis I et al.	19822956	No data for ACE2
1	The V16A polymorphism in SOD2 is associated with increased risk of diabetic nephropathy and cardiovascular disease in type 1 diabetes.	Mollsten A et al.	19834686	No data for ACE2
1	Time of drug administration, CYP3A5 and ABCB1 genotypes, and analytical method influence tacrolimus pharmacokinetics: a population pharmacokinetic study.	Musuamba FT et al.	19855314	No data for ACE2
1	Impact of donor-dependent genetic factors on long-term renal graft function.	Krajewska M et al.	19857655	No data for ACE2
1	Uromodulin levels associate with a common UMOD variant and risk for incident CKD.	Kottgen A et al.	19959715	No data for ACE2
1	Endothelial nitric oxide synthase (eNOS) gene polymorphism in early term chronic allograft nephropathy.	Yilmaz E et al.	20005399	No data for ACE2
1	Circulating methylarginine levels and the decline in renal function in patients with chronic kidney disease are modulated by DDAH1 polymorphisms.	Caplin B et al.	20010544	No data for ACE2
1	Dense mapping of MYH9 localizes the strongest kidney disease associations to the region of introns 13 to 15.	Nelson GW et al.	20124285	No data for ACE2
1	African ancestry allelic variation at the MYH9 gene contributes to increased susceptibility to non-diabetic end-stage kidney disease in Hispanic Americans.	Behar DM et al.	20144966	No data for ACE2
1	Pharmacogenetics of immunosuppressant polymorphism of CYP3A5 in renal transplant recipients.	Larriba J et al.	20172323	No data for ACE2
1	Patients with Epstein-Fechtner syndromes owing to MYH9 R702 mutations develop progressive proteinuric renal disease.	Sekine T et al.	20200500	No data for ACE2

For peer review only

1	Association analysis of ADPRT1, AKR1B1, RAGE, GFPT2 and PAI-1 gene polymorphisms with chronic renal insufficiency among Asian Indians with type-2 diabetes.	Prasad P et al.	20353610	No data for ACE2
1	Optimization of initial tacrolimus dose using pharmacogenetic testing.	Thervet E et al.	20393454	No data for ACE2
1	Identification of GDNF gene sequence variations in patients with medullary sponge kidney disease.	Torregrossa R et al.	20448065	No data for ACE2
1	Association of polymorphisms in the klotho gene with severity of non-diabetic ESRD in African Americans.	Bostrom MA et al.	20466664	No data for ACE2
1	DDOST, PRKCSH and LGALS3, which encode AGE-receptors 1, 2 and 3, respectively, are not associated with diabetic nephropathy in type 1 diabetes.	Hoverfelt A et al.	20490454	No data for ACE2
1	The acetyl-coenzyme A carboxylase beta (ACACB) gene is associated with nephropathy in Chinese patients with type 2 diabetes.	Tang SC et al.	20519229	No data for ACE2
1	Association of trypanolytic ApoL1 variants with kidney disease in African Americans.	Genovese G et al.	20647424	No data for ACE2
1	A risk allele for focal segmental glomerulosclerosis in African Americans is located within a region containing APOL1 and MYH9.	Genovese G et al.	20668430	No data for ACE2
1	ACE gene polymorphism and serum ACE activity in Iranians type II diabetic patients with macroalbuminuria.	Felehgari V et al.	20830509	No data for ACE2
1	Endothelial nitric oxide genotypes and haplotypes are not associated with end-stage renal disease.	Marson BP et al.	20849252	No data for ACE2
1	The frequency of factor V Leiden mutation, ACE gene polymorphism, serum ACE activity and response to ACE inhibitor and angiotensin II receptor antagonist drugs in Iranians type II diabetic patients with microalbuminuria.	Rahimi Z et al.	20853144	No data for ACE2
1	Association of glutathione S-transferase M1 and T1 gene polymorphism with oxidative stress in diabetic and nondiabetic chronic kidney disease.	Datta SK et al.	20954980	No data for ACE2
1	Toward personalized medicine in renal transplantation.	Lampreabe I et al.	20970553	No data for ACE2

For peer review only

1	Relation between development of cardiovascular disease and the C242T CYBA polymorphism of the NADPH oxidase in ESRD patients.	Tang FY et al.	21045268	No data for ACE2
1	Association analysis of Notch pathway signalling genes in diabetic nephropathy.	Kavanagh D et al.	21103979	No data for ACE2
1	[Clinical and genetic basis of hypertensive nephrosclerosis. NEFROSEN Study].	Diez Ojea B et al.	21113220	No data for ACE2
1	Impact of polymorphisms of the genes encoding angiotensin II-forming enzymes on the progression of IgA nephropathy.	Jung ES et al.	21150220	No data for ACE2
1	Genetic polymorphisms of the renin-angiotensin-aldosterone system in Chinese patients with end-stage renal disease secondary to IgA nephropathy.	Huang HD et al.	21163122	No data for ACE2
1	Novel polymorphisms associated with tacrolimus trough concentrations: results from a multicenter kidney transplant consortium.	Jacobson PA et al.	21206424	No data for ACE2
1	Angiotensin-converting enzyme gene polymorphisms and T2DM in a case-control association study of the Bahraini population.	Al-Harbi EM et al.	21207118	No data for ACE2
1	Polymorphisms in the nonmuscle myosin heavy chain 9 gene (MYH9) are associated with the progression of IgA nephropathy in Chinese.	Cheng W et al.	21245129	No data for ACE2
1	Association of eNOS gene polymorphisms with renal disease in Caucasians with type 2 diabetes.	Santos KG et al.	21255858	No data for ACE2
1	Genetic variation in the matrix metalloproteinase genes and diabetic nephropathy in type 1 diabetes.	Kure M et al.	21277817	No data for ACE2
1	Tacrolimus dosing in Chinese renal transplant recipients: a population-based pharmacogenetics study.	Li L et al.	21331500	No data for ACE2
1	Analysis of insertion/deletion polymorphisms of the angiotensin converting enzyme gene in Malaysian end-stage renal disease patients.	Ali A et al.	21421653	No data for ACE2
1	In vivo activity of epoxide hydrolase according to sequence variation affects the progression of human IgA nephropathy.	Lee JP et al.	21429967	No data for ACE2
1	Impact of aldosterone synthase gene C-344T polymorphism on IgA nephropathy.	Bantis C et al.	21476902	No data for ACE2

For peer review only

1	Expression of CYP3A5 and P-glycoprotein in renal allografts with histological signs of calcineurin inhibitor nephrotoxicity.	Metalidis C et al.	21544031	No data for ACE2
1	Common variants in CNDP1 and CNDP2, and risk of nephropathy in type 2 diabetes.	Ahluwalia TS et al.	21573905	No data for ACE2
1	Insertion/deletion polymorphism of the angiotensin-converting enzyme predicts left ventricular hypertrophy after renal transplantation.	Fedor R et al.	21620105	No data for ACE2
1	Polymorphisms of pon1 and pon2 genes in hemodialyzed patients.	Rajkovic MG et al.	21620813	No data for ACE2
1	A polymorphism of NADPH oxidase p22 phox is associated with reduced susceptibility to acute rejection in renal allograft recipients.	Bhandary UV et al.	21624462	No data for ACE2
1	Exome sequencing identified MYO1E and NEIL1 as candidate genes for human autosomal recessive steroid-resistant nephrotic syndrome.	Sanna-Cherchi S et al.	21697813	No data for ACE2
1	Differential effects of MYH9 and APOL1 risk variants on FRMD3 Association with Diabetic ESRD in African Americans.	Freedman BI et al.	21698141	No data for ACE2
1	Influence of aldosterone synthase gene C-344T polymorphism on focal segmental glomerulosclerosis.	Bantis C et al.	21777344	No data for ACE2
1	Thiopurine S-methyltransferase polymorphism in Iranian kidney transplant recipients.	Aghdaie MH et al.	21819368	No data for ACE2
1	Sickle cell trait is not independently associated with susceptibility to end-stage renal disease in African Americans.	Hicks PJ et al.	21849968	No data for ACE2
1	Association between CYP3A5 polymorphisms and blood pressure in kidney transplant recipients receiving calcineurin inhibitors.	Ferraresso M et al.	21851254	No data for ACE2
1	Impact of nitric oxide synthase Glu298Asp polymorphism on the development of end-stage renal disease in type 2 diabetic Egyptian patients.	El-Din Bessa SS et al.	21854353	No data for ACE2
1	Assessment of matrix Gla protein, Klotho gene polymorphisms, and oxidative stress in chronic kidney disease.	Karsli Ceppioglu S et al.	21859400	No data for ACE2
1	Influence of CYP3A5 and ABCB1 gene polymorphisms and other factors on tacrolimus dosing in Caucasian liver and kidney transplant patients.	Provenzani A et al.	21922127	No data for ACE2

For peer review only

1	Interaction of MTHFR 1298C with ACE D allele augments the risk of diabetic nephropathy in Western Iran.	Rahimi Z et al.	21942443	No data for ACE2
1	Association of genetic variants in the promoter region of genes encoding p22phox (CYBA) and glutamate cysteine ligase catalytic subunit (GCLC) and renal disease in patients with type 1 diabetes mellitus.	Vieira SM et al.	21962117	No data for ACE2
1	Polymorphisms in MYH9 are associated with diabetic nephropathy in European Americans.	Cooke JN et al.	21968013	No data for ACE2
1	Angiotensin-converting enzyme (ACE) serum levels and gene polymorphism in Egyptian patients with systemic lupus erythematosus.	Abbas D et al.	21976404	No data for ACE2
1	Association of eGFR-Related Loci Identified by GWAS with Incident CKD and ESRD.	Boger CA et al.	21980298	No data for ACE2
1	APOL1 variants increase risk for FSGS and HIVAN but not IgA nephropathy.	Papeta N et al.	21997397	No data for ACE2
1	Genetic and functional effects of membrane metalloendopeptidase on diabetic nephropathy development.	Zhang D et al.	22024547	No data for ACE2
1	The interactions of age, sex, body mass index, genetics, and steroid weight-based doses on tacrolimus dosing requirement after adult kidney transplantation.	Stratta P et al.	22101623	No data for ACE2
1	Genetic polymorphisms and the risk of progressive renal failure in elderly Hungarian patients.	Zsom M et al.	22111818	No data for ACE2
1	Glutathione S-transferases T1 null genotype is associated with susceptibility to aristolochic acid nephropathy.	Chen B et al.	22116675	No data for ACE2
1	Nosocomial Pneumocystis jirovecii pneumonia: lessons from a cluster in kidney transplant recipients.	Phipps LM et al.	22129760	No data for ACE2
1	Gene polymorphisms of angiotensin-converting enzyme and angiotensin II type 1 receptor among chronic kidney disease patients in a Chinese population.	Su SL et al.	22147663	No data for ACE2
1	Clinical and genetic factors affecting tacrolimus trough levels and drug-related outcomes in Korean kidney transplant recipients.	Kim IW et al.	22183771	No data for ACE2

For peer review only

1	SIRTUIN 1 gene polymorphisms are associated with cholesterol metabolism and coronary artery calcification in Japanese hemodialysis patients.	Shimoyama Y et al.	22200427	No data for ACE2
1	Association of UDP-glucuronosyltransferase 1A9 (UGT1A9) gene polymorphism with kidney allograft function.	Pazik J et al.	22210424	No data for ACE2
1	The association of the UGT1A8, SLCO1B3 and ABCC2/ABCG2 genetic polymorphisms with the pharmacokinetics of mycophenolic acid and its phenolic glucuronide metabolite in Chinese individuals.	Geng F et al.	22227166	No data for ACE2
1	BsmI polymorphisms in vitamin D receptor gene are associated with diabetic nephropathy in type 2 diabetes in the Han Chinese population.	Zhang H et al.	22245613	No data for ACE2
1	Matrix metalloproteinase (MMP)-2 genetic variants modify the circulating MMP-2 levels in end-stage kidney disease.	Marson BP et al.	22302011	No data for ACE2
1	Impact of cytochrome P450 3A and ATP-binding cassette subfamily B member 1 polymorphisms on tacrolimus dose-adjusted trough concentrations among Korean renal transplant recipients.	Cho JH et al.	22310591	No data for ACE2
1	Endothelial nitric oxide synthase gene polymorphisms and the risk of diabetic nephropathy in type 2 diabetes mellitus.	Shoukry A et al.	22313046	No data for ACE2
1	MTHFR C677T, A1298C and ACE I/D polymorphisms as risk factors for diabetic nephropathy among type 2 diabetic patients.	El-Baz R et al.	22554825	No data for ACE2
1	Paraoxonase 1 polymorphisms in patients with primary glomerulonephritis: a single-center study in Turkey.	Eren Z et al.	22555481	No data for ACE2
1	Association of base excision repair gene polymorphisms with ESRD risk in a Chinese population.	Cai Z et al.	22720119	No data for ACE2
1	Base excision repair gene polymorphisms are associated with inflammation in patients undergoing chronic hemodialysis.	Cai Z et al.	22780951	No data for ACE2
1	Relationship between antioxidant enzyme genotype and activity and kidney function: a case-control study.	Crawford A et al.	22790458	No data for ACE2

For peer review only

1	Genetic polymorphisms located in genes related to immune and inflammatory processes are associated with end-stage renal disease: a preliminary study.	Jimenez-Sousa MA et al.	22817530	No data for ACE2
1	Apolipoprotein L1 gene variants associate with hypertension-attributed nephropathy and the rate of kidney function decline in African Americans.	Lipkowitz MS et al.	22832513	No data for ACE2
1	Donor age and ABCB1 1199G>A genetic polymorphism are independent factors affecting long-term renal function after kidney transplantation.	De Meyer M et al.	22835948	No data for ACE2
1	The effect of glutathion S-transferase polymoprhisms and anti-GSTT1 antibodies on allograft functions in recipients of renal transplant.	Akgul SU et al.	22841242	No data for ACE2
1	Primary hyperoxaluria type 1, a too often missed diagnosis and potentially treatable cause of end-stage renal disease in adults: results of the Dutch cohort.	van der Hoeven SM et al.	22844106	No data for ACE2
1	Genetic and functional analyses of MRAS and HNF1A genes in diabetes and diabetic nephropathy.	Horova E et al.	22849862	No data for ACE2
1	Genetic variation in thrombin-activatable fibrinolysis inhibitor is associated with the risk of diabetic nephropathy.	Xu CW et al.	22932273	No data for ACE2
1	Genetic variation in APOL1 and MYH9 genes is associated with chronic kidney disease among Nigerians.	Tayo BO et al.	22956460	No data for ACE2
1	Glutathione S-transferase A1, M1, P1 and T1 null or low-activity genotypes are associated with enhanced oxidative damage among haemodialysis patients.	Suvakov S et al.	23034843	No data for ACE2
1	Association of vitamin D receptor FokI and ApaI polymorphisms with human cytomegalovirus disease in the first three months following kidney transplantation.	Zhao YG et al.	23044313	No data for ACE2
1	Vitamin D receptor gene BsmI, FokI, ApaI and TaqI polymorphisms and the risk of systemic lupus erythematosus.	Mostowska A et al.	23065277	No data for ACE2
1	ACACÎ² gene (rs2268388) and AGTR1 gene (rs5186) polymorphism and the risk of nephropathy in Asian Indian patients with type 2 diabetes.	Shah VN et al.	23081748	No data for ACE2

For peer review only

1	Chronic renal impairment and DDAH2-1151 A/C polymorphism determine ADMA levels in type 2 diabetic subjects.	Marra M et al.	23129820	No data for ACE2
1	Relationship of BsmI polymorphism of vitamin D receptor gene with left ventricular hypertrophy and atherosclerosis in hemodialysis patients.	El-Shehaby AM et al.	23198772	No data for ACE2
1	CYP3A4 genetic polymorphisms predict cyclosporine-related clinical events in Chinese renal transplant recipients.	Wang YY et al.	23217392	No data for ACE2
1	Clinical impact of an angiotensin I-converting enzyme insertion/deletion and kinin B2 receptor +9/-9 polymorphisms in the prognosis of renal transplantation.	Amorim CE et al.	23362199	No data for ACE2
1	The A736V TMPRSS6 polymorphism influences hepcidin and iron metabolism in chronic hemodialysis patients: TMPRSS6 and hepcidin in hemodialysis.	Pelusi S et al.	23433094	No data for ACE2
1	Identification of chromosome 3q28 and ALPK1 as susceptibility loci for chronic kidney disease in Japanese individuals by a genome-wide association study.	Yamada Y et al.	23539754	No data for ACE2
1	Replication study for the association of 3 SNP loci identified in a genome-wide association study for diabetic nephropathy in European type 1 diabetes with diabetic nephropathy in Japanese patients with type 2 diabetes.	Maeda S et al.	23543049	No data for ACE2
1	eNOS 4a/b polymorphism and its interaction with eNOS G894T variants in type 2 diabetes mellitus: modifying the risk of diabetic nephropathy.	Rahimi Z et al.	23594559	No data for ACE2
1	Association of CYP1A1 gene polymorphism with chronic kidney disease: a case control study.	Siddarth M et al.	23619522	No data for ACE2
1	Influence of GSTO2 (N142D) genetic polymorphism on acute renal rejection.	Nekooie-Marnany N et al.	23649768	No data for ACE2
1	Association of aldosterone synthase (CYP11B2) gene -344T/C polymorphism with the risk of primary chronic glomerulonephritis in the Polish population.	Pawlik M et al.	23681285	No data for ACE2
1	Association of POL1, MALT1, MC4R, PHLPP and DSEL single nucleotide polymorphisms in chromosome 18q region with type 2 diabetes in Tunisians.	Turki A et al.	23727064	No data for ACE2

For peer review only

1	ADAMTS13 predicts renal and cardiovascular events in type 2 diabetic patients and response to therapy.	Rurali E et al.	23733198	No data for ACE2
1	Characterization of three vasopressin receptor 2 variants: an apparent polymorphism (V266A) and two loss-of-function mutations (R181C and M311V).	Armstrong SP et al.	23762448	No data for ACE2
1	Distribution of dimethylarginine-dimethylaminohydrolase-II (DDAH2) gene polymorphism in hemodialysis patients.	Thaha M et al.	23770786	No data for ACE2
1	Genetic predisposition for development of nephropathy in type 2 diabetes mellitus.	Kumar R et al.	23846111	No data for ACE2
1	CYP2C9 and ABCG2 polymorphisms as risk factors for developing adverse drug reactions in renal transplant patients taking fluvastatin: a case-control study.	Mirosevic Skvrce N et al.	24024895	No data for ACE2
1	Impact of CYP3A5 genotype on tacrolimus versus midazolam clearance in renal transplant recipients: new insights in CYP3A5-mediated drug metabolism.	de Jonge H et al.	24024898	No data for ACE2
1	Suppressed soluble Fms-like tyrosine kinase-1 production aggravates atherosclerosis in chronic kidney disease.	Matsui M et al.	24048373	No data for ACE2
1	Impact of CYP3A4*22 allele on tacrolimus pharmacokinetics in early period after renal transplantation: toward updated genotype-based dosage guidelines.	Elens L et al.	24052064	No data for ACE2
1	Association of TNF-Î± -308 G > A and ACE I/D gene polymorphisms in hemodialysis patients with arteriovenous fistula thrombosis.	Sener EF et al.	24126814	No data for ACE2
1	Association of E-selectin gene polymorphism and serum PAPP-A with carotid atherosclerosis in end-stage renal disease.	Issac MS et al.	24151105	No data for ACE2
1	MYH9-related disease: a novel prognostic model to predict the clinical evolution of the disease based on genotype-phenotype correlations.	Pecci A et al.	24186861	No data for ACE2
1	Increased level of organochlorine pesticides in chronic kidney disease patients of unknown etiology: role of GSTM1/GSTT1 polymorphism.	Siddarth M et al.	24216264	No data for ACE2

For peer review only

1	An integrative study of the genetic, social and environmental determinants of chronic kidney disease characterized by tubulointerstitial damages in the North Central Region of Sri Lanka.	Nanayakkara S et al.	24351856	No data for ACE2
1	Immune response following liver transplantation compared to kidney transplantation: usefulness of monitoring peripheral blood CD4+ adenosine triphosphate activity and cytochrome P450 3A5 genotype assay.	Nobuoka Y et al.	24454479	No data for ACE2
1	Relationship of CYP3A5 genotype and ABCB1 diplotype to tacrolimus disposition in Brazilian kidney transplant patients.	Cusinato DA et al.	24528196	No data for ACE2
1	Evaluation of candidate nephropathy susceptibility genes in a genome-wide association study of African American diabetic kidney disease.	Palmer ND et al.	24551085	No data for ACE2
1	Identification of novel mutations of PKD1 gene in Chinese patients with autosomal dominant polycystic kidney disease by targeted next-generation sequencing.	Yang T et al.	24582653	No data for ACE2
1	Association of BH3 interacting domain death agonist (BID) gene polymorphisms with proteinuria of immunoglobulin A nephropathy.	Park HJ et al.	24621205	No data for ACE2
1	Association studies of cytochrome P450, family 2, subfamily E, polypeptide 1 (CYP2E1) gene polymorphisms with acute rejection in kidney transplantation recipients.	Kim SK et al.	24654912	No data for ACE2
1	MYH9 and APOL1 gene polymorphisms and the risk of CKD in patients with lupus nephritis from an admixture population.	Colares VS et al.	24658608	No data for ACE2
1	Cys327Cys polymorphism of the PAPP-A gene (pregnancy associated plasma protein A) is related to mortality of long term hemodialysis patients.	Kalousova M et al.	24667032	No data for ACE2
1	Genes involved in the regulation of vascular homeostasis determine renal survival rate in patients with chronic glomerulonephritis.	Litovkina O et al.	24727057	No data for ACE2
1	Lack of serologic evidence to link IgA nephropathy with celiac disease or immune reactivity to gluten.	Moeller S et al.	24732864	No data for ACE2
1	Genetic variants of ACE (Insertion/Deletion) and AGT (M268T) genes in patients with diabetes and nephropathy.	Shaikh R et al.	24737640	No data for ACE2

For peer review only

1	Single nucleotide polymorphisms at erythropoietin, superoxide dismutase 1, splicing factor, arginine/serin-rich 15 and plasmacytoma variant translocation genes association with diabetic nephropathy.	Alwohhaib M et al.	24821155	No data for ACE2
1	NOS3 tagSNPs does not modify the chronic kidney disease progression in autosomal dominant polycystic kidney disease.	Ramanathan G et al.	24824375	No data for ACE2
1	Renin-angiotensin-aldosterone system related gene polymorphisms and urinary total arsenic is related to chronic kidney disease.	Chen WJ et al.	24907556	No data for ACE2
1	Gene-gene interactions in renin-angiotensin-aldosterone system contributes to end-stage renal disease susceptibility in a Han Chinese population.	Su SL et al.	24977181	No data for ACE2
1	Polymorphisms in oxidative stress pathway genes and risk of diabetic nephropathy in South Indian type 2 diabetic patients.	Narne P et al.	25041504	No data for ACE2
1	Rare mutations associating with serum creatinine and chronic kidney disease.	Sveinbjornsson G et al.	25082825	No data for ACE2
1	Is increased susceptibility to Balkan endemic nephropathy in carriers of common GSTA1 (*A/*B) polymorphism linked with the catalytic role of GSTA1 in ochratoxin a biotransformation? Serbian case control study and in silico analysis.	Reljic Z et al.	25111321	No data for ACE2
1	Association between genetic polymorphisms of ACE & eNOS and diabetic nephropathy.	Huo P et al.	25227524	No data for ACE2
1	DNA repair genes XPD and XRCC1 polymorphisms and risk of end-stage renal disease in Egyptian population.	Radwan WM et al.	25310768	No data for ACE2
1	Clinical utility of chitotriosidase enzyme activity in nephropathic cystinosis.	Elmonem MA et al.	25407738	No data for ACE2
1	Synergism between asymmetric dimethylarginine (ADMA) and a genetic marker of uric acid in CKD progression.	Testa A et al.	25435339	No data for ACE2
1	The functional Q84R polymorphism of TRIB3 gene is associated with diabetic nephropathy in Chinese type 2 diabetic patients.	Zhang W et al.	25447894	No data for ACE2

For peer review only

1	Circulating angiotensin-converting enzyme 2 activity in patients with chronic kidney disease without previous history of cardiovascular disease	Anguiano L et al.	25813276	No data for ACE2
1	Allelic variations in the CYBA gene of NADPH oxidase and risk of kidney complications in patients with type 1 diabetes.	Patente TA et al.	25862415	No data for ACE2
1	Associations between INSR and MTOR polymorphisms in type 2 diabetes mellitus and diabetic nephropathy in a Northeast Chinese Han population.	Zhu AN et al.	25867326	No data for ACE2
1	Polymorphism of the CYP3A5 gene and its effect on tacrolimus blood level.	Nair SS et al.	25894154	No data for ACE2
1	Copy Number Variation at the APOL1 Locus.	Ruchi R et al.	25933006	No data for ACE2
1	Aldosterone Synthase CYP11B2 Gene Promoter Polymorphism in a Turkish Population With Chronic Kidney Disease.	Yilmaz M et al.	25957425	No data for ACE2
1	Is Klotho F352V Polymorphism the Missing Piece of the Bone Loss Puzzle in Renal Transplant Recipients?	Ozdem S et al.	26022923	No data for ACE2
1	Identification of new susceptibility loci for IgA nephropathy in Han Chinese.	Li M et al.	26028593	No data for ACE2
1	Association between C1GALT1 variants and genetic susceptibility to IgA nephropathy in Uygur.	Li WL et al.	26125729	No data for ACE2
1	Estimated glomerular filtration rate (eGFR), 25(OH) D3, chronic kidney disease (CKD), the MYH9 (myosin heavy chain 9) gene in old and very elderly people.	Otero Gonzalez A et al.	26152646	No data for ACE2
1	Influence of genetic variability at the ACE locus in intron 16 on Diabetic Nephropathy in T1DM patients.	Parchwani DN et al.	26214998	No data for ACE2
1	How to handle missed or delayed doses of tacrolimus in renal transplant recipients? A pharmacokinetic investigation.	Saint-Marcoux F et al.	26316426	No data for ACE2
1	Re-Sequencing of the APOL1-APOL4 and MYH9 Gene Regions in African Americans Does Not Identify Additional Risks for CKD Progression.	Hawkins GA et al.	26343748	No data for ACE2

For Peer review only

1	Prevalence of angiotensin converting enzyme (ACE) gene insertion/deletion polymorphism in South Indian population with hypertension and chronic kidney disease.	Shanmuganathan R et al.	26440392	No data for ACE2
1	RAGE and CYBA polymorphisms are associated with microalbuminuria and end-stage renal disease onset in a cohort of type 1 diabetes mellitus patients over a 20-year follow-up.	Franko B et al.	26607824	No data for ACE2
1	Polymorphisms in NADPH oxidase CYBA gene modify the risk of ESRD in patients with chronic glomerulonephritis.	Zhou H et al.	26627442	No data for ACE2
1	Impact of CYP3A5 polymorphism on trough concentrations and outcomes of tacrolimus minimization during the early period after kidney transplantation.	Yaowakulpatana K et al.	26635230	No data for ACE2
1	Relationship between rs1047763 polymorphism of the C1GALT1 gene and susceptibility to immunoglobulin A nephropathy in Xinjiang Uyghur people.	Xue JN et al.	26782518	No data for ACE2
1	Manganese superoxide dismutase, glutathione peroxidase and catalase gene polymorphisms and clinical outcomes in acute kidney injury.	Kidir V et al.	26787049	No data for ACE2
1	Genetic analysis and functional characterization of novel mutations in a series of patients with atypical hemolytic uremic syndrome.	Szarvas N et al.	26826462	No data for ACE2
1	A Lack of Significant Effect of POR*28 Allelic Variant on Tacrolimus Exposure in Kidney Transplant Recipients.	Jannot AS et al.	26829596	No data for ACE2
1	Association of RAC1 Gene Polymorphisms with Primary End-Stage Renal Disease in Chinese Renal Recipients.	Liu Y et al.	26841219	No data for ACE2
1	Nodular glomerulosclerosis and renin angiotensin system in Chinese patients with type 2 diabetes	Wang M et al.	26973293	No data for ACE2
1	Genome-Wide Association Study of Acute Renal Graft Rejection.	Ghisdal L et al.	27272414	No data for ACE2
1	The angiotensin-I converting enzyme gene I/D variation contributes to end-stage renal disease risk in Chinese patients with type 2 diabetes receiving hemodialysis.	Lu M et al.	27633502	No data for ACE2

For peer review only

1	Pin1 and secondary hyperparathyroidism of chronic kidney disease: gene polymorphisms and protein levels.	Zhao Y et al.	27876426	No data for ACE2
1	Lysine 63 ubiquitination is involved in the progression of tubular damage in diabetic nephropathy.	Pontrelli P et al.	27881486	No data for ACE2
1	Mutational Analysis of Agxt in Tunisian Population with Primary Hyperoxaluria Type 1.	M'dimegh S et al.	27935012	No data for ACE2
1	Mutation screening of ACKR3 and COPS8 in kidney cancer cases from the CONFIRM study.	Mahmoodi M et al.	28063109	No data for ACE2
1	Glutathione Peroxidase 1 Gene Polymorphism in Nephrolithiasis Patients From South of Iran.	Aghakhani R et al.	28174350	No data for ACE2
1	Association of MMP-9 gene polymorphisms with nephrolithiasis patients.	Mehde AA et al.	28205286	No data for ACE2
1	Galactosylation of IgA1 Is Associated with Common Variation in C1GALT1.	Gale DP et al.	28209808	No data for ACE2
1	Relationship of Serum Klotho Level With ACE Gene Polymorphism in Stable Kidney Allograft Recipients.	Zaare Nahandi M et al.	28270648	No data for ACE2
1	GSTM1 genotype is an independent prognostic factor in clear cell renal cell carcinoma.	Coric VM et al.	28284893	No data for ACE2
1	Effect of AMP-activated protein kinase subunit alpha 2 (PRKAA2) genetic polymorphisms on susceptibility to type 2 diabetes mellitus and diabetic nephropathy in a Chinese population.	Li Q et al.	28322508	No data for ACE2
1	Transcriptional Coactivator p300 and Silent Information Regulator 1 (SIRT1) Gene Polymorphism Associated with Diabetic Kidney Disease in a Chinese Cohort.	Tang K et al.	28444663	No data for ACE2
1	Association of polymorphic variants of PTPN22, TNF and VDR genes in children with lupus nephritis: A study in Colombian family triads.	Garavito G et al.	28527290	No data for ACE2
1	The Contribution of MMP-7 Promoter Polymorphisms in Renal Cell Carcinoma.	Liao CH et al.	28652430	No data for ACE2
1	MDR-1 and CYP3A5 Polymorphisms in Pediatric Idiopathic Nephrotic Syndrome: Impact on Susceptibility and Response to Steroids (Preliminary Results).	Moussa A et al.	28792718	No data for ACE2

For peer review only

1	CYP3A genotypes of donors but not those of the patients increase the risk of acute rejection in renal transplant recipients on calcineurin inhibitors: a pilot study.	Gervasini G et al.	29043387	No data for ACE2
1	Association of EPHX2 R287Q Polymorphism with Diabetic Nephropathy in Chinese Type 2 Diabetic Patients.	Ma L et al.	29629376	No data for ACE2
1	Systemic redox biomarkers and their relationship to prognostic risk markers in autosomal dominant polycystic kidney disease and IgA nephropathy.	Tariq A et al.	29655960	No data for ACE2
1	Collagenase-1 (-1607 1G/2G), Gelatinase-A (-1306 C/T), Stromelysin-1 (-1171 5A/6A) functional promoter polymorphisms in risk prediction of type 2 diabetic nephropathy.	Gantala SR et al.	29883760	No data for ACE2
1	Influence of uridine diphosphate-glucuronosyltransferases (1A9) polymorphisms on mycophenolic acid pharmacokinetics in patients with renal transplant.	Ciftci HS et al.	30012031	No data for ACE2
1	CpG island in the region of an autosomal dominant polycystic kidney disease locus defines the 5' end of a gene encoding a putative proton channel.	Gillespie GA et al.	1709739	Non-human study
1	Recombinant human acetylcholinesterase is secreted from transiently transfected 293 cells as a soluble globular enzyme.	Velan B et al.	1849451	Non-human study
1	Enhanced acetaminophen toxicity in rats with bilirubin glucuronyl transferase deficiency.	de Morais SM et al.	2501210	Non-human study
1	Control of lysosomal acid phosphatase expression in man-mouse cell hybrids.	Shaws TB et al.	4856818	Non-human study
1	Kallikrein and amylase contents in tissues from a mutant mouse model for human cystic fibrosis.	Catanzaro OL et al.	6186886	Non-human study
1	Effect of phosphate supplementation on the expression of the mutant phenotype in murine X-linked hypophosphatemic rickets.	Tenenhouse HS et al.	7873297	Non-human study
1	Purification and characterization of UDP-GlcNAc:IV3 beta Gal-Gb4Cer beta-1,6-GlcNAc transferase from mouse kidney.	Sekine M et al.	7983056	Non-human study
1	Production and characterization of recombinant Goodpasture antigen in insect cells.	Turner N et al.	8006020	Non-human study

For peer review only

1	Expression of a dominant allele of human ARF1 inhibits membrane traffic in vivo.	Zhang CJ et al.	8294513	Non-human study
1	Testosterone modulation of N-acetylation in mouse kidney.	Smolen TN et al.	8437129	Non-human study
1	Evidence that syntaxin 1A is involved in storage in the secretory pathway.	Bittner MA et al.	8626670	Non-human study
1	Role of the ceramide-signaling pathway in cytokine responses to P-fimbriated Escherichia coli.	Hedlund M et al.	8642245	Non-human study
1	Tissue-specific imprinting of the mouse insulin-like growth factor II receptor gene correlates with differential allele-specific DNA methylation.	Hu JF et al.	9482664	Non-human study
1	Chronic renal failure in a mouse model of human adenine phosphoribosyltransferase deficiency.	Stockelman MG et al.	9689017	Non-human study
1	Renal angiotensin converting enzyme promotes renal damage during ureteral obstruction	Stoneking BJ et al.	9719278	Non-human study
1	Detection of mutation(s) or polymorphic loci in the genome of experimental animal and human cancer tissues by RAPD/AP-PCR depend on DNA polymerase.	Singh KP et al.	10087325	Non-human study
1	Truncated human leptin (delta133) associated with extreme obesity undergoes proteasomal degradation after defective intracellular transport.	Rau H et al.	10098508	Non-human study
1	The mll-AF9 gene fusion in mice controls myeloproliferation and specifies acute myeloid leukaemogenesis.	Dobson CL et al.	10393173	Non-human study
1	Nitric oxide-dependent renal vasodilatation is not altered in rat with rHuEpo-induced hypertension.	Migliori M et al.	10394113	Non-human study
1	Decreased matrix metalloproteinase activity in the kidneys of hereditary nephrotic mice (ICGN strain).	Uchio K et al.	11014984	Non-human study
1	p300 interacts with the nuclear proto-oncoprotein SYT as part of the active control of cell adhesion.	Eid JE et al.	11030627	Non-human study

For peer review only

1	Telomerase inhibition in RenCa, a murine tumor cell line with short telomeres, by overexpression of a dominant negative mTERT mutant, reveals fundamental differences in telomerase regulation between human and murine cells.	Sachsinger J et al.	11454711	Non-human study
1	An alternative promoter of the human neuronal nitric oxide synthase gene is expressed specifically in Leydig cells.	Wang Y et al.	11786430	Non-human study
1	Three novel sarco/endoplasmic reticulum Ca2+-ATPase (SERCA) 3 isoforms. Expression, regulation, and function of the membranes of the SERCA3 family.	Martin V et al.	11956212	Non-human study
1	The gene INPPL1, encoding the lipid phosphatase SHIP2, is a candidate for type 2 diabetes in rat and man.	Marion E et al.	12086927	Non-human study
1	Organizational diversity among distinct glycoprotein endoplasmic reticulum-associated degradation programs.	Cabral CM et al.	12181335	Non-human study
1	Metabolic fate of pitavastatin (NK-104), a new inhibitor of 3-hydroxy-3-methyl-glutaryl coenzyme A reductase. Effects on drug-metabolizing systems in rats and humans.	Fujino H et al.	12442637	Non-human study
1	Modulation of splicing events in histone deacetylase 3 by various extracellular and signal transduction pathways.	Gray SG et al.	12691522	Non-human study
1	Use of the tetracycline system for inducible protein synthesis in the kidney.	Gallagher AR et al.	12874458	Non-human study
1	Implications of glucose transporter protein type 1 (GLUT1)-haplodeficiency in embryonic stem cells for their survival in response to hypoxic stress.	Heilig C et al.	14578187	Non-human study
1	Susceptibility to vascular neoplasms but no increased susceptibility to renal carcinogenesis in Vhl knockout mice.	Kleymenova E et al.	14604887	Non-human study
1	Gain-of-function polymorphism in mouse and human Ltk: implications for the pathogenesis of systemic lupus erythematosus.	Li N et al.	14695357	Non-human study
1	Cytotoxicity of RNases is increased by cationization and counteracted by K(Ca) channels.	Ilinskaya ON et al.	14733942	Non-human study
1	N-Domain angiotensin I-converting enzyme expression in renal artery of Wistar, Wistar Kyoto, and spontaneously hypertensive rats.	Bueno V et al.	15194348	Non-human study

For peer review only

1	Fluorescence resonance energy transfer reports properties of syntaxin1a interaction with Munc18-1 in vivo.	Liu J et al.	15489225	Non-human study
1	Sprouty1 is a critical regulator of GDNF/RET-mediated kidney induction.	Basson MA et al.	15691764	Non-human study
1	Alternative splicing of vitamin D-24-hydroxylase: a novel mechanism for the regulation of extrarenal 1,25-dihydroxyvitamin D synthesis.	Ren S et al.	15788398	Non-human study
1	Hypoxia induces a functionally significant and translationally efficient neuronal NO synthase mRNA variant.	Ward ME et al.	16276418	Non-human study
1	Luteinizing hormone receptor ectodomain splice variant misroutes the full-length receptor into a subcompartment of the endoplasmic reticulum.	Apaja PM et al.	16495341	Non-human study
1	N-terminal residues control proteasomal degradation of RGS2, RGS4, and RGS5 in human embryonic kidney 293 cells.	Bodenstein J et al.	17220356	Non-human study
1	Characterization of transcriptional regulation of neurogranin by nitric oxide and the role of neurogranin in SNP-induced cell death: implication of neurogranin in an increased neuronal susceptibility to oxidative stress.	Gui J et al.	17389928	Non-human study
1	Fecal carriage of Escherichia coli O157:H7 and carcass contamination in cattle at slaughter in northern Italy.	Alonso S et al.	17661289	Non-human study
1	Generation and functional characterization of mice with a disrupted glutathione S-transferase, theta 1 gene.	Fujimoto K et al.	17827337	Non-human study
1	Functional genetic variation in aminopeptidase A (ENPEP): lack of clear association with focal and segmental glomerulosclerosis (FSGS).	Tonna S et al.	18206321	Non-human study
1	Hepatitis C virus replication is inhibited by 22beta-methoxyolean-12-ene-3beta, 24(4beta)-diol (ME3738) through enhancing interferon-beta.	Hiasa Y et al.	18459156	Non-human study
1	Characterization of a novel cardiac isoform of the cell cycle-related kinase that is regulated during heart failure.	Qiu H et al.	18508765	Non-human study
1	Increased insulin action in SKIP heterozygous knockout mice.	Ijuin T et al.	18573875	Non-human study
1	Mitochondrial dysfunction in mut methylmalonic acidemia.	Chandler RJ et al.	19088183	Non-human study
1	Overexpression of cytochrome P450 4F2 in mice increases 20-hydroxyeicosatetraenoic acid production and arterial blood pressure.	Liu X et al.	19279555	Non-human study

For peer review only

1	Kallikrein genes are associated with lupus and glomerular basement membrane-specific antibody-induced nephritis in mice and humans.	Liu K et al.	19307730	Non-human study
1	p38MAPK controls expression of multiple cell cycle inhibitors and islet proliferation with advancing age.	Wong ES et al.	19619499	Non-human study
1	Blocking the salt-inducible kinase 1 network prevents the increases in cell sodium transport caused by a hypertension-linked mutation in human alpha-adducin.	Stenstrom K et al.	19657284	Non-human study
1	Maitotoxin converts the plasmalemmal Ca(2+) pump into a Ca(2+)-permeable nonselective cation channel.	Sinkins WG et al.	19794142	Non-human study
1	MyoD control of SKIP expression during pig skeletal muscle development.	Xiong Q et al.	20336382	Non-human study
1	Targeted deletion of the Nesp55 DMR defines another Gnas imprinting control region and provides a mouse model of autosomal dominant PHP-Ib.	Frohlich LF et al.	20427744	Non-human study
1	Autosomal recessive polycystic kidney disease epithelial cell model reveals multiple basolateral epidermal growth factor receptor sorting pathways.	Ryan S et al.	20519437	Non-human study
1	The activating mutation R201C in GNAS promotes intestinal tumourigenesis in Apc(Min/+) mice through activation of Wnt and ERK1/2 MAPK pathways.	Wilson CH et al.	20531296	Non-human study
1	Pitchfork regulates primary cilia disassembly and left-right asymmetry.	Kinzel D et al.	20643351	Non-human study
1	Mediation of CTCF transcriptional insulation by DEAD-box RNA-binding protein p68 and steroid receptor RNA activator SRA.	Yao H et al.	20966046	Non-human study
1	A common polymorphism in the human aromatase gene alters the risk for polycystic ovary syndrome and modifies aromatase activity in vitro.	Wang H et al.	21282199	Non-human study
1	Transgenic overexpression of the extra-large GsÎ± variant XLÎ±s enhances GsÎ±-mediated responses in the mouse renal proximal tubule in vivo.	Liu Z et al.	21303955	Non-human study
1	Rat Ace allele variation determines susceptibility to AngII-induced renal damage	Kamilic J et al.	21788250	Non-human study
1	Carnosine treatment largely prevents alterations of renal carnosine metabolism in diabetic mice.	Peters V et al.	21833769	Non-human study

For peer review only

1	YME1L controls the accumulation of respiratory chain subunits and is required for apoptotic resistance, cristae morphogenesis, and cell proliferation.	Stiburek L et al.	22262461	Non-human study
1	Primary hyperoxaluria in Coton de Tulear.	Vidgren G et al.	22486513	Non-human study
1	Role of angiotensin-converting enzyme (ACE and ACE2) imbalance on tourniquet-induced remote kidney injury in a mouse hindlimb ischemia-reperfusion model	Yang XH et al.	22580272	Non-human study
1	Tubule-specific ablation of endogenous Î^{2}-catenin aggravates acute kidney injury in mice.	Zhou D et al.	22622501	Non-human study
1	Î±1,3-galactosyltransferase deficiency in germ-free miniature pigs increases N-glycolylneuraminic acids as the xenoantigenic determinant in pig-human xenotransplantation.	Park JY et al.	22775484	Non-human study
1	Cystic echinococcosis in Romania: an epidemiological survey of livestock demonstrates the persistence of hyperendemicity.	Mitrea IL et al.	23075460	Non-human study
1	First WNK4-hypokalemia animal model identified by genome-wide association in Burmese cats.	Gandolfi B et al.	23285264	Non-human study
1	Identification of aldo-keto reductases as NRF2-target marker genes in human cells.	Jung KA et al.	23305850	Non-human study
1	An inducible transgenic mouse model for familial hypertension with hyperkalaemia (Gordon's syndrome or pseudohypoaldosteronism type II).	Chowdhury JA et al.	23336180	Non-human study
1	Presence of N-glycosylated transthyretin in plasma of V30M carriers in familial amyloidotic polyneuropathy: an escape from ERAD.	Teixeira AC et al.	23387326	Non-human study
1	Multi-species comparative analysis of the equine ACE gene identifies a highly conserved potential transcription factor binding site in intron 16.	Hamilton NA et al.	23408978	Non-human study
1	Renal collecting duct NOS1 maintains fluid-electrolyte homeostasis and blood pressure.	Hyndman KA et al.	23608660	Non-human study
1	ARHGDIA mutations cause nephrotic syndrome via defective RHO GTPase signaling.	Gee HY et al.	23867502	Non-human study

For peer review only

1	A knock-in mouse model of human PHD2 gene-associated erythrocytosis establishes a haploinsufficiency mechanism.	Arsenault PR et al.	24121508	Non-human study
1	The Pex1-G844D mouse: a model for mild human Zellweger spectrum disorder.	Hiebler S et al.	24503136	Non-human study
1	Holocarboxylase synthetase interacts physically with nuclear receptor co-repressor, histone deacetylase 1 and a novel splicing variant of histone deacetylase 1 to repress repeats.	Liu D et al.	24840043	Non-human study
1	Factor VIIa binding to endothelial cell protein C receptor protects vascular barrier integrity in vivo.	Sundaram J et al.	24977291	Non-human study
1	Atrial natriuretic peptide locally counteracts the deleterious effects of cardiomyocyte mineralocorticoid receptor activation.	Nakagawa H et al.	25027872	Non-human study
1	The T309G murine double minute 2 gene polymorphism is an independent prognostic factor for patients with renal cell carcinoma.	de Martino M et al.	25415135	Non-human study
1	Generation of WNK1 knockout cell lines by CRISPR/Cas-mediated genome editing.	Roy A et al.	25477473	Non-human study
1	PTPN2 controls differentiation of CD4â�º T cells and limits intestinal inflammation and intestinal dysbiosis.	Spalinger MR et al.	25492475	Non-human study
1	A functional 12T-insertion polymorphism in the ATP1A1 promoter confers decreased susceptibility to hypertension in a male Sardinian population.	Herrera VL et al.	25615575	Non-human study
1	BAP1 missense mutation c.2054 A>T (p.E685V) completely disrupts normal splicing through creation of a novel 5' splice site in a human mesothelioma cell line.	Morrison A et al.	25830670	Non-human study
1	Identification of a receptor for extracellular renalase.	Wang L et al.	25906147	Non-human study
1	Mammalian target of rapamycin signaling inhibition ameliorates vascular calcification via Klotho upregulation.	Zhao Y et al.	26061549	Non-human study
1	TNFÎ± Amplifies DNaseI Expression in Renal Tubular Cells while IL-1Î² Promotes Nuclear DNaseI Translocation in an Endonuclease-Inactive Form.	Thiyagarajan D et al.	26065428	Non-human study
1	DAPIT Over-Expression Modulates Glucose Metabolism and Cell Behaviour in HEK293T Cells.	Kontro H et al.	26161955	Non-human study

For peer review only

1	Allelic Variants in Arhgef11 via the Rho-Rock Pathway Are Linked to Epithelial-Mesenchymal Transition and Contributes to Kidney Injury in the Dahl Salt-Sensitive Rat.	Jia Z et al.	26172442	Non-human study
1	Human GRK4Î³142V Variant Promotes Angiotensin II Type I Receptor-Mediated Hypertension via Renal Histone Deacetylase Type 1 Inhibition.	Wang Z et al.	26667412	Non-human study
1	Dynamin-2 is a novel NOS1Î² interacting protein and negative regulator in the collecting duct.	Hyndman KA et al.	26791826	Non-human study
1	Re-engineering the zinc fingers of PRDM9 reverses hybrid sterility in mice.	Davies B et al.	26840484	Non-human study
1	Cardiac angiotensin-(1-12) expression and systemic hypertension in rats expressing the human angiotensinogen gene.	Ferrario CM et al.	26873967	Non-human study
1	From man to fish: What can Zebrafish tell us about ApoL1 nephropathy?	Olabisi O et al.	27509583	Non-human study
1	Cabozantinib inhibits tumor growth and metastasis of a patient-derived xenograft model of papillary renal cell carcinoma with MET mutation.	Zhao H et al.	27715452	Non-human study
1	Renin-angiotensin system transgenic mouse model recapitulates pathophysiology similar to human preeclampsia with renal injury that may be mediated through VEGF.	Denney JM et al.	27927648	Non-human study
1	Inhibition of TRPC6 channels ameliorates renalÂ fibrosis and contributes to renal protectionÂ byÂ soluble klotho.	Wu YL et al.	27979597	Non-human study
1	Activated ERK1/2 increases CD44 in glomerular parietal epithelial cells leading to matrix expansion.	Roeder SS et al.	27998643	Non-human study
1	Parallel microarray profiling identifies ErbB4 as a determinant of cyst growth in ADPKD and a prognostic biomarker for disease progression.	Streets AJ et al.	28077374	Non-human study
1	Targeting mTOR Signaling Can Prevent the Progression of FSGS.	Zschiedrich S et al.	28270414	Non-human study
1	The leukotriene B(4)-leukotriene B(4) receptor axisÂ promotes cisplatin-induced acute kidney injuryÂ byÂ modulating neutrophil recruitment.	Deng B et al.	28318626	Non-human study
1	DNase-active TREX1 frame-shift mutants induce serologic autoimmunity in mice.	Sakai T et al.	28325644	Non-human study
1	Human SR-BII mediates SAA uptake and contributes to SAA pro-inflammatory signaling in vitro and in vivo.	Baranova IN et al.	28423002	Non-human study

For peer review only

1	Bim gene dosage is critical in modulating nephron progenitor survival in the absence of microRNAs during kidney development.	Cerqueira DM et al.	28446592	Non-human study
1	DBA/2J Haplotype on Distal Chromosome 2 Reduces Mertk Expression, Restricts Efferocytosis, and Increases Susceptibility to Atherosclerosis.	Kayashima Y et al.	28473436	Non-human study
1	Inhibition of HDAC enhances STAT acetylation, blocks NF-Î°B, and suppresses the renal inflammation and fibrosis in Npr1 haplotype male mice.	Kumar P et al.	28566502	Non-human study
1	The regulatory 1Î± subunit of protein kinase A modulates renal cystogenesis.	Ye H et al.	28615245	Non-human study
1	Functional analysis of human aromatic amino acid transporter MCT10/TAT1 using the yeast Saccharomyces cerevisiae.	Uemura S et al.	28754537	Non-human study
1	Kindlin-2 Association with Rho GDP-Dissociation Inhibitor Î± Suppresses Rac1 Activation and Podocyte Injury.	Sun Y et al.	28775002	Non-human study
1	Interference with GsÎ±-Coupled Receptor Signaling in Renin-Producing Cells Leads to Renal Endothelial Damage.	Lachmann P et al.	28775003	Non-human study
1	Blood Pressure Control by a Secreted FGFBP1 (Fibroblast Growth Factor-Binding Protein).	Tassi E et al.	29158353	Non-human study
1	Caspase-independent programmed cell death triggers Ca(2)PO(4) deposition in an in vitro model of nephrocalcinosis.	Priante G et al.	29208768	Non-human study
1	Knockout of the interleukin-36 receptor protects against renal ischemia-reperfusion injury by reduction of proinflammatory cytokines.	Nishikawa H et al.	29241623	Non-human study
1	Xyloketal B exerts antihypertensive effect in renovascular hypertensive rats via the NO-sGC-cGMP pathway and calcium signaling.	Zhao LY et al.	29595193	Non-human study
1	Mice Carrying a Dominant-Negative Human PI3K Mutation Are Protected From Obesity and Hepatic Steatosis but Not Diabetes.	Solheim MH et al.	29724723	Non-human study
1	A homozygous KAT2B variant modulates the clinical phenotype of ADD3 deficiency in humans and flies.	Goncalves S et al.	29768408	Non-human study
1	Haploinsufficiency of the Mouse Atp6v1b1 Gene Leads to a Mild Acid-Base Disturbance with Implications for Kidney Stone Disease.	Bourgeois S et al.	29843146	Non-human study
1	RFLP-DR beta and serological HLA-DR typing of 200 kidney recipients and 1000 controls.	Mytilineos J et al.	1975140	Not a case-control study

For peer review only

1	Superoxide dismutase isozymes in different human tissues, their genetic control and intracellular localization.	Beckman G et al.	4775457	Not a case-control study
1	Genetic counselling in lethal X-linked disorders.	Emery AE et al.	4976958	Not a case-control study
1	DNA-based presymptomatic diagnosis for the von Hippel-Lindau disease by linkage analysis.	Olschwang S et al.	7552140	Not a case-control study
1	Role of the deletion of polymorphism of the angiotensin converting enzyme gene in the progression and therapeutic responsiveness of IgA nephropathy.	Yoshida H et al.	7593601	Not a case-control study
1	Mistyping of the human angiotensin-converting enzyme gene polymorphism: frequency, causes and possible methods to avoid errors in typing.	Ueda S et al.	8863184	Not a case-control study
1	Analysis of linkage disequilibrium between polymorphisms in the KCNJ9 gene with type 2 diabetes mellitus in Pima Indians.	Wolford JK et al.	11350189	Not a case-control study
1	A family of autosomal dominant hypocalcemia with a positive correlation between serum calcium and magnesium: identification of a novel gain of function mutation (Ser(820)Phe) in the calcium-sensing receptor.	Nagase T et al.	12050233	Not a case-control study
1	Captopril enhances transforming growth factor (TGF)-beta1 expression in peripheral blood mononuclear cells: a mechanism independent from angiotensin converting enzyme inhibition? A study in cyclosporine-treated kidney-transplanted patients.	Di Paolo S et al.	12499886	Not a case-control study
1	Clinical assessment and mutation analysis of Kallmann syndrome 1 (KAL1) and fibroblast growth factor receptor 1 (FGFR1, or KAL2) in five families and 18 sporadic patients.	Sato N et al.	15001591	Not a case-control study
1	The effect of CYP3A5 and MDR1 (ABCB1) polymorphisms on cyclosporine and tacrolimus dose requirements and trough blood levels in stable renal transplant patients.	Haufroid V et al.	15167702	Not a case-control study
1	Antiproteinuric effect of candesartan cilexetil in Japanese subjects with type 2 diabetes and nephropathy.	Haneda M et al.	15364166	Not a case-control study
1	An unusual association of contralateral congenital small kidney, reduced renal function and hyperparathyroidism in sponge kidney patients: on the track of the molecular basis.	Gambaro G et al.	15814540	Not a case-control study

For peer review only

1	Presentation and role of transplantation in adult patients with type 1 primary hyperoxaluria and the I244T AGXT mutation: Single-center experience.	Lorenzo V et al.	16912707	Not a case-control study
1	Familial nonsyndromic pheochromocytoma.	Opocher G et al.	17102081	Not a case-control study
1	Synergistic expression of angiotensin-converting enzyme (ACE) and ACE2 in human renal tissue and confounding effects of hypertension on the ACE to ACE2 ratio	Wakahara et al.	17303661	Not a case-control study
1	Verification of consumers' experiences and perceptions of genetic discrimination and its impact on utilization of genetic testing.	Barlow-Stewart K et al.	19287242	Not a case-control study
1	Porphyria in Switzerland, 15 years experience.	Schneider-Yin X et al.	19350426	Not a case-control study
1	Whole-genome linkage and association scan in primary, nonsyndromic vesicoureteric reflux.	Cordell HJ et al.	19959718	Not a case-control study
1	Identification of a gene for renal-hepatic-pancreatic dysplasia by microarray-based homozygosity mapping.	Fiskerstrand T et al.	20007846	Not a case-control study
1	Bardet-Biedl syndrome in Denmark--report of 13 novel sequence variations in six genes.	Hjortshoj TD et al.	20120035	Not a case-control study
1	Chromosome 7p linkage and association study for diabetes related traits and type 2 diabetes in an African-American population enriched for nephropathy.	Leak TS et al.	20144192	Not a case-control study
1	MYH9 genetic variants associated with glomerular disease: what is the role for genetic testing?	Kopp JB et al.	20807613	Not a case-control study
1	A prospective, open-label, observational clinical cohort study of the association between delayed renal allograft function, tacrolimus exposure, and CYP3A5 genotype in adult recipients.	Kuypers DR et al.	21118736	Not a case-control study
1	Acute kidney injury reduces the hepatic metabolism of midazolam in critically ill patients.	Kirwan CJ et al.	22005822	Not a case-control study
1	ABCB1 polymorphisms are associated with cyclosporine-induced nephrotoxicity and gingival hyperplasia in renal transplant recipients.	Garcia M et al.	22886152	Not a case-control study

For peer review only

1	Novel SMARCAL1 bi-allelic mutations associated with a chromosomal breakage phenotype in a severe SIOD patient.	Simon AJ et al.	24197801	Not a case-control study
1	Brief Report: identification of MTMR3 as a novel susceptibility gene for lupus nephritis in northern Han Chinese by shared-gene analysis with IgA nephropathy.	Zhou XJ et al.	24943867	Not a case-control study
1	An efficient and comprehensive strategy for genetic diagnostics of polycystic kidney disease.	Eisenberger T et al.	25646624	Not a case-control study
1	ABCB1 (MDR-1) pharmacogenetics of tacrolimus in renal transplanted patients: a Next Generation Sequencing approach.	Tavira B et al.	25781547	Not a case-control study
1	Novel mutations in the inverted formin 2 gene of Chinese families contribute to focal segmental glomerulosclerosis.	Xie J et al.	26039629	Not a case-control study
1	A Novel von Hippel Lindau Gene Intronic Variant and Its Reclassification from VUS to Pathogenic: the Impact on a Large Family.	Sexton A et al.	26323595	Not a case-control study
1	Genome-wide Association Studies Identify Genetic Loci Associated With Albuminuria in Diabetes.	Teumer A et al.	26631737	Not a case-control study
1	Updated genetic testing of Italian patients referred with a clinical diagnosis of primary hyperoxaluria.	Pelle A et al.	26946417	Not a case-control study
1	Polypoid urothelial tumor with inverted growth pattern in the renal pelvis: morphologic and molecular characteristics of a unique diagnostic entity.	Wobker SE et al.	27574810	Not a case-control study
1	Haploinsufficiency in tumor predisposition syndromes: altered genomic transcription in morphologically normal cells heterozygous for VHL or TSC mutation.	Peri S et al.	27682873	Not a case-control study
1	CYP3A4*1G and CYP3A5*3 genetic polymorphisms alter the antihypertensive efficacy of amlodipine in patients with hypertension following renal transplantationâ€©.	Huang Y et al.	27841150	Not a case-control study
1	Clinical, Genetic and Innate Immunity Characteristics of Melanoma in Organ Transplant Recipients.	Brocard A et al.	27868139	Not a case-control study
1	Clinical impact of the CYP3A5 6986A>G allelic variant on kidney transplantation outcomes.	Flahault A et al.	27977332	Not a case-control study
1	Smoking has no impact on survival and it is not associated with ACE gene I/D polymorphism in hemodialysis patients.	Kiss I et al.	28058974	Not a case-control study

For peer review only

1	Evaluation of Glutathione Peroxidase and KCNJ11 Gene Polymorphisms in Patients with New Onset Diabetes Mellitus After Renal Transplantation.	Yalin GY et al.	28073131	Not a case-control study
1	The CYP3A biomarker 4Î²-hydroxycholesterol does not improve tacrolimus dose predictions early after kidney transplantation.	Storset E et al.	28146606	Not a case-control study
1	Prograf produces more benefits for CYP3A5 low expression patients in early stage after kidney transplantation.	Fan B et al.	28157649	Not a case-control study
1	The genetic and clinical spectrum of a large cohort of patients with distal renal tubular acidosis.	Palazzo V et al.	28233610	Not a case-control study
1	Impact of the CYP3A5 genotype on the distributions of dose-adjusted trough concentrations and incidence of rejection in Japanese renal transplant recipients receiving different tacrolimus formulations.	Niioka T et al.	28271256	Not a case-control study
1	Influence of ABCC2, CYP2C8, and CYP2J2 Polymorphisms on Tacrolimus and Mycophenolate Sodium-Based Treatment in Brazilian Kidney Transplant Recipients.	Genvigir FDV et al.	28316087	Not a case-control study
1	Role of ACE and IL-1Î² Gene Polymorphisms in Erythropoeitin Hyporesponsive Patients with Chronic Kidney Disease with Anemia.	Nand N et al.	28457029	Not a case-control study
1	Pretransplant 4Î²-hydroxycholesterol does not predict tacrolimus exposure or dose requirements during the first days after kidney transplantation.	Vanhove T et al.	28603840	Not a case-control study
1	Sequencing of FIC1, BSEP and MDR3 in a large cohort of patients with cholestasis revealed a high number of different genetic variants.	Droge C et al.	28733223	Not a case-control study
1	Low renal but high extrarenal phenotype variability in Schimke immuno-osseous dysplasia.	Lipska-Zietkiewicz BS et al.	28796785	Not a case-control study
1	Wuzhi Tablet (Schisandra sphenanthera Extract) is a Promising Tacrolimus-Sparing Agent for Renal Transplant Recipients Who are CYP3A5 Expressers: a Two-Phase Prospective Study.	Li J et al.	28864749	Not a case-control study
1	Crizotinib achieves long-lasting disease control in advanced papillary renal-cell carcinoma type 1 patients with MET mutations or amplification. EORTC 90101 CREATE trial.	Schoffski P et al.	29149761	Not a case-control study

For peer review only

1	Cost-effectiveness analysis of elbasvir-grazoprevir regimen for treating hepatitis C virus genotype 1 infection in stage 4-5 chronic kidney disease patients in France.	Maunoury F et al.	29543897	Not a case-control study
1	Late-onset acid maltase deficiency. Detection of patients and heterozygotes by urinary enzyme assay.	Mehler M et al.	9923	Not a renal disease focus
1	Differential inhibition of the products of the human alkaline phosphatase loci.	Mulivor RA et al.	686677	Not a renal disease focus
1	A classification of tumor development based on an analysis of enzymes in tissue sections of hypernephroid carcinoma in man.	Wachsmuth ED et al.	1016198	Not a renal disease focus
1	Identification of mutations associated with peroxisome-to-mitochondrion mistargeting of alanine/glyoxylate aminotransferase in primary hyperoxaluria type 1.	Purdue PE et al.	1703535	Not a renal disease focus
1	Concentrations of ochratoxin A in the urine of endemic nephropathy patients and controls in Bulgaria: lack of detection of 4-hydroxyochratoxin A.	Castegnaro M et al.	1820331	Not a renal disease focus
1	T-cell receptor beta-subunit gene polymorphism and autoimmune disease.	Niven MJ et al.	1969400	Not a renal disease focus
1	Role of P450IID6, the target of the sparteine-debrisoquin oxidation polymorphism, in the metabolism of imipramine.	Brosen K et al.	2060250	Not a renal disease focus
1	Immunological heterogeneity of hepatic alanine:glyoxylate aminotransferase in primary hyperoxaluria type 1.	Wise PJ et al.	2443389	Not a renal disease focus
1	Renin in blood vessels in human pulmonary tumors. An immunohistochemical and biochemical study.	Taylor GM et al.	2450464	Not a renal disease focus
1	Elevated concentrations of brain-type glycogen phosphorylase in renal cell carcinoma.	Takashi M et al.	2515181	Not a renal disease focus
1	Origin of rare Ha-ras alleles: relationship of VTR length to a 5' polymorphic Xho I site.	Baxter GD et al.	2558964	Not a renal disease focus
1	Loss of heterozygosity at the c-raf locus in small cell lung carcinoma.	Sithanandam G et al.	2566144	Not a renal disease focus
1	Mechanisms of suppression of renal kallikrein activity in low renin essential hypertension and renoparenchymal hypertension.	Shimamoto K et al.	2676859	Not a renal disease focus

For peer review only

1	Reduced expression of multiple forms of the alpha subunit of the stimulatory GTP-binding protein in pseudohypoparathyroidism type Ia.	Carter A et al.	2890163	Not a renal disease focus
1	The beta-subunit of follicle-stimulating hormone is deleted in patients with aniridia and Wilms' tumour, allowing a further definition of the WAGR locus.	Glaser T et al.	3014343	Not a renal disease focus
1	Further studies on the activity and subcellular distribution of alanine:glyoxylate aminotransferase in the livers of patients with primary hyperoxaluria type 1.	Danpure CJ et al.	3416563	Not a renal disease focus
1	Acetylator genotype-dependent metabolic activation of carcinogenic N-hydroxyarylamines by S-acetyl coenzyme A-dependent enzymes of inbred hamster tissue cytosols: relationship to arylamine N-acetyltransferase.	Hein DW et al.	3677303	Not a renal disease focus
1	Persistence of streptococcal group A antibody in patients with rheumatic valvular disease.	Dudding BA et al.	5682941	Not a renal disease focus
1	Studies of an abnormal serum albumin unstable upon storage.	Rousseaux J et al.	6215190	Not a renal disease focus
1	Allotypes of properdin factor B(Bf) and lymphocytotoxic antibody production.	Davidson JA et al.	6399879	Not a renal disease focus
1	Control of serum C3 levels by beta 1H and C3b inactivator.	Wyatt RJ et al.	6445926	Not a renal disease focus
1	Leukocyte beta-glucosidase in homozygotes and heterozygotes for Gaucher disease.	Raghavan SS et al.	6770675	Not a renal disease focus
1	Structural organization of the human neuronal nitric oxide synthase gene (NOS1).	Hall AV et al.	7528745	Not a renal disease focus
1	Analysis of meningiomas by methylation- and transcription-based clonality assays.	Zhu J et al.	7641206	Not a renal disease focus
1	Cytochrome P4501A1 gene polymorphism and homozygous deletion of the glutathione S-transferase M1 gene in urothelial cancer patients.	Katoh T et al.	7697828	Not a renal disease focus
1	Distribution of MHC class II alleles in primary systemic vasculitis.	Zhang L et al.	7731160	Not a renal disease focus

For peer review only

1	Angiotensin-converting enzyme gene polymorphism has no influence on the circulating renin-angiotensin-aldosterone system or blood pressure in normotensive subjects	Lachurie ML et al.	7796503	Not a renal disease focus
1	p53 mutations and MDM-2 amplification in renal cell cancers.	Imai Y et al.	7824511	Not a renal disease focus
1	Frequencies of variants of candidate genes in different age groups of hypertensives.	Zee RY et al.	7882587	Not a renal disease focus
1	Independent, marked associations of alleles of the insulin receptor and dipeptidyl carboxypeptidase-I genes with essential hypertension.	Morris BJ et al.	8104754	Not a renal disease focus
1	Increased expression of vascular permeability factor (vascular endothelial growth factor) and its receptors in kidney and bladder carcinomas.	Brown LF et al.	8238242	Not a renal disease focus
1	Alpha-1-proteinase inhibitor and pulmonary haemorrhage in systemic vasculitis.	O'Donoghue DJ et al.	8296629	Not a renal disease focus
1	Expression of NAD(P)H:quinone oxidoreductase and glutathione S-transferases alpha and pi in human renal cell carcinoma and in kidney cancer-derived cell lines.	Eickelmann P et al.	8313512	Not a renal disease focus
1	A homoallelic Gly317-->Asp mutation in ALPL causes the perinatal (lethal) form of hypophosphatasia in Canadian mennonites.	Greenberg CR et al.	8406453	Not a renal disease focus
1	Angiotensin converting enzyme (ACE) insertion/deletion (I/D) polymorphism, and diabetic retinopathy in subjects with IDDM and NIDDM.	Nagi DK et al.	8582133	Not a renal disease focus
1	Characterization of Gas6, a member of the superfamily of G domain-containing proteins, as a ligand for Rse and Axl.	Mark MR et al.	8621659	Not a renal disease focus
1	C-antineutrophil cytoplasmic antibody positivity in vasculitis patients is associated with the Z allele of alpha-1-antitrypsin, and P-antineutrophil cytoplasmic antibody positivity with the S allele.	Griffith ME et al.	8671812	Not a renal disease focus
1	Absence of mutations in parathyroid hormone (PTH)/PTH-related protein receptor complementary deoxyribonucleic acid in patients with pseudohypoparathyroidism type Ib.	Fukumoto S et al.	8675577	Not a renal disease focus
1	Deletion polymorphism of the angiotensin-converting enzyme gene is independently associated with left ventricular mass and geometric remodeling in systemic hypertension.	Gharavi AG et al.	8677872	Not a renal disease focus

For peer review only

1	Azathioprine pharmacogenetics: the relationship between 6-thioguanine nucleotides and thiopurine methyltransferase in patients after heart and kidney transplantation.	Schutz E et al.	8721407	Not a renal disease focus
1	Examination of the role of nitric oxide synthase and renal kallikrein as candidate genes for essential hypertension.	Friend LR et al.	8800585	Not a renal disease focus
1	Genetic risk for renal artery stenosis: association with deletion polymorphism in angiotensin 1-converting enzyme gene.	Missouris CG et al.	8821841	Not a renal disease focus
1	Chromosome 11p15.5 regional imprinting: comparative analysis of KIP2 and H19 in human tissues and Wilms' tumors.	Chung WY et al.	8842727	Not a renal disease focus
1	Germline mutations in glial cell line-derived neurotrophic factor (GDNF) and RET in a Hirschsprung disease patient.	Angrist M et al.	8896568	Not a renal disease focus
1	Germline mutations of the RET ligand GDNF are not sufficient to cause Hirschsprung disease.	Salomon R et al.	8896569	Not a renal disease focus
1	Increased expression of the insulin-like growth factor-II gene in Wilms' tumor is not dependent on loss of genomic imprinting or loss of heterozygosity.	Wang WH et al.	8910385	Not a renal disease focus
1	Genetic analysis of the NAT2 and CYP2D6 polymorphisms in white patients with non-insulin-dependent diabetes mellitus.	Agundez JA et al.	8946479	Not a renal disease focus
1	Renal cell carcinoma of end-stage renal disease: a histopathologic and molecular genetic study.	Hughson MD et al.	8959640	Not a renal disease focus
1	Reduced expression of the cyclin-dependent kinase inhibitor gene p57KIP2 in Wilms' tumor.	Thompson JS et al.	8971182	Not a renal disease focus
1	Angiotensin converting enzyme gene polymorphism and renal hemodynamic function in early diabetes.	Miller JA et al.	8995725	Not a renal disease focus
1	Somatic inactivation of the VHL gene in Von Hippel-Lindau disease tumors.	Prowse AH et al.	9106522	Not a renal disease focus
1	Association of diabetic neuropathy with Na/K ATPase gene polymorphism.	Vague P et al.	9165217	Not a renal disease focus
1	Angiotensin I-converting enzyme and angiotensinogen gene polymorphisms in non-insulin-dependent diabetes mellitus. Lack of relationship with diabetic nephropathy and retinopathy in a Caucasian Mediterranean population.	Gutierrez C et al.	9258285	Not a renal disease focus

For peer review only

1	Influence of polymorphisms of GSTM1 and GSTT1 for risk of renal cell cancer in workers with long-term high occupational exposure to trichloroethene.	Bruning T et al.	9285043	Not a renal disease focus
1	FHIT gene and the FRA3B region are not involved in the genetics of renal cell carcinomas.	Bugert P et al.	9290948	Not a renal disease focus
1	Isolation and characterization of UGT2B15(Y85): a UDP-glucuronosyltransferase encoded by a polymorphic gene.	Levesque E et al.	9295060	Not a renal disease focus
1	Analyses of mutations in the human renal kallikrein (hKLK1) gene and their possible relevance to blood pressure regulation and risk of myocardial infarction.	Berge KE et al.	9298743	Not a renal disease focus
1	Coding mutations in p57KIP2 are present in some cases of Beckwith-Wiedemann syndrome but are rare or absent in Wilms tumors.	O'Keefe D et al.	9311733	Not a renal disease focus
1	Loss of heterozygosity of the nm23-H1 gene in human renal cell carcinomas.	Bosnar MH et al.	9341897	Not a renal disease focus
1	Biological monitoring of workers exposed to low levels of 2-butoxyethanol.	Haufroid V et al.	9342622	Not a renal disease focus
1	Renal ACE immunohistochemical localization in NIDDM patients with nephropathy.	Mizuiri S et al.	9469501	Not a renal disease focus
1	The Captopril Prevention Project (CAPPP) in hypertension--baseline data and current status.	Hansson L et al.	9495662	Not a renal disease focus
1	Angiotensin I-converting enzyme gene polymorphism modulates the consequences of in utero growth retardation on plasma insulin in young adults.	Cambien F et al.	9519756	Not a renal disease focus
1	Contribution of angiotensin I converting enzyme gene polymorphism and angiotensinogen gene polymorphism to blood pressure regulation in essential hypertension.	Mondorf UF et al.	9524045	Not a renal disease focus
1	Evidence of association of the ecNOS gene polymorphism with plasma NO metabolite levels in humans.	Tsukada T et al.	9535806	Not a renal disease focus
1	Human GFRA1: cloning, mapping, genomic structure, and evaluation as a candidate gene for Hirschsprung disease susceptibility.	Angrist M et al.	9545641	Not a renal disease focus
1	Serum paraoxonase activity and its relationship to diabetic complications in patients with non-insulin-dependent diabetes mellitus.	Ikeda Y et al.	9591753	Not a renal disease focus

For peer review only

1	Inhibition by platelet-activating factor of Src- and hepatocyte growth factor-dependent invasiveness of intestinal and kidney epithelial cells. Phosphatidylinositol 3'-kinase is a critical mediator of tumor invasion.	Kotelevets L et al.	9603913	Not a renal disease focus
1	Angiotensin I-converting enzyme and angiotensinogen gene interaction and prediction of essential hypertension.	Vasku A et al.	9607178	Not a renal disease focus
1	Phosphorylation sites in the autoinhibitory domain participate in p70(s6k) activation loop phosphorylation.	Dennis PB et al.	9614086	Not a renal disease focus
1	EGF-r gene copy number changes in renal cell carcinoma detected by fluorescence in situ hybridization.	Moch H et al.	9664910	Not a renal disease focus
1	Point mutation and homozygous deletion of PTEN/MMAC1 in primary bladder cancers.	Cairns P et al.	9671402	Not a renal disease focus
1	Duplication and overexpression of the mutant allele of the MET proto-oncogene in multiple hereditary papillary renal cell tumours.	Fischer J et al.	9715275	Not a renal disease focus
1	Trisomy 7-harbouring non-random duplication of the mutant MET allele in hereditary papillary renal carcinomas.	Zhuang Z et al.	9731534	Not a renal disease focus
1	An analysis of phenotypic variation in the familial cancer syndrome von Hippel-Lindau disease: evidence for modifier effects.	Webster AR et al.	9758595	Not a renal disease focus
1	CYP2E1 genotyping in renal cell/urothelial cancer patients in comparison with control populations.	Farker K et al.	9760005	Not a renal disease focus
1	Identification of fifteen novel mutations in the tissue-nonspecific alkaline phosphatase (TNSALP) gene in European patients with severe hypophosphatasia.	Mornet E et al.	9781036	Not a renal disease focus
1	Impact of CYP2E1 genotype in renal cell and urothelial cancer patients.	Farker K et al.	9784018	Not a renal disease focus
1	Inhibition of tissue angiotensin converting enzyme activity prevents malignant hypertension in TGR(mREN2)27.	Montgomery HE et al.	9797175	Not a renal disease focus
1	Analysis of 3p allelic losses in renal cell carcinomas: comparison with cytogenetic results.	Bernues M et al.	9844606	Not a renal disease focus
1	Renal changes on hyperglycemia and angiotensin-converting enzyme in type 1 diabetes.	Marre M et al.	10082486	Not a renal disease focus
1	Association of an insertion polymorphism of angiotensin-converting enzyme gene with the activity of lupus nephritis.	Akai Y et al.	10099886	Not a renal disease focus

For peer review only

1	G-Protein beta3 subunit C825T variant and ambulatory blood pressure in essential hypertension.	Beige J et al.	10205246	Not a renal disease focus
1	Novel mutations in the 1alpha-hydroxylase (P450c1) gene in three families with pseudovitamin D-deficiency rickets resulting in loss of functional enzyme activity in blood-derived macrophages.	Smith SJ et al.	10320521	Not a renal disease focus
1	Gene structure, chromosomal location, and expression pattern of maleylacetoacetate isomerase.	Fernandez-Canon JM et al.	10373324	Not a renal disease focus
1	Candidate genetic modifiers of individual susceptibility to renal cell carcinoma: a study of polymorphic human xenobiotic-metabolizing enzymes.	Longuemaux S et al.	10383153	Not a renal disease focus
1	Absence of PTEN germ-line mutations in men with a potential inherited predisposition to prostate cancer.	Cooney KA et al.	10389923	Not a renal disease focus
1	Increased fragmentation of von Willebrand factor, due to abnormal cleavage of the subunit, parallels disease activity in recurrent hemolytic uremic syndrome and thrombotic thrombocytopenic purpura and discloses predisposition in families. The Italian Registry of Familial and Recurrent HUS/TTP.	Galbusera M et al.	10397728	Not a renal disease focus
1	Mutations of the VHL gene in sporadic renal cell carcinoma: definition of a risk factor for VHL patients to develop an RCC.	Gallou C et al.	10408776	Not a renal disease focus
1	Novel somatic mutations in the VHL gene in Swedish archived sporadic renal cell carcinomas.	Yang K et al.	10454237	Not a renal disease focus
1	The gene encoding hydroxypyruvate reductase (GRHPR) is mutated in patients with primary hyperoxaluria type II.	Cramer SD et al.	10484776	Not a renal disease focus
1	Carotid intima-media thickness and ACE-gene polymorphism in hemodialysis patients.	Nergizoglu G et al.	10493570	Not a renal disease focus
1	Telomerase activity and telomere lengths: alterations in renal cell carcinomas.	Dahse R et al.	10504477	Not a renal disease focus
1	Genotyping and functional analysis of a polymorphic (CCTTT)(n) repeat of NOS2A in diabetic retinopathy.	Warpeha KM et al.	10506586	Not a renal disease focus
1	Molecular basis of human salt sensitivity: the role of the 11beta-hydroxysteroid dehydrogenase type 2.	Lovati E et al.	10523024	Not a renal disease focus

For peer review only

1	Genetic polymorphisms of the renin-angiotensin system and atheromatous renal artery stenosis.	Olivieri O et al.	10567188	Not a renal disease focus
1	Molecular characterization of the human PEA15 gene on 1q21-q22 and association with type 2 diabetes mellitus in Pima Indians.	Wolford JK et al.	10607908	Not a renal disease focus
1	Somatic mutation and homozygous deletion of PTEN/MMAC1 gene of 10q23 in renal cell carcinoma.	Alimov A et al.	10628321	Not a renal disease focus
1	No association between deletion-type angiotensin-converting enzyme gene polymorphism and left-ventricular hypertrophy in hemodialysis patients.	Yildiz A et al.	10657713	Not a renal disease focus
1	Analysis of point mutation in exon 2 of CYP2E1 gene in renal cell/urothelial cancer patients in comparison with control population.	Farker K et al.	10667834	Not a renal disease focus
1	Association between diabetic retinopathy and genetic variations in alpha2beta1 integrin, a platelet receptor for collagen.	Matsubara Y et al.	10688808	Not a renal disease focus
1	Lys(173)Arg and -344T/C variants of CYP11B2 in Japanese patients with low-renin hypertension.	Komiya I et al.	10720581	Not a renal disease focus
1	Synergistic effect of alpha-adducin and ACE genes causes blood pressure changes with body sodium and volume expansion.	Barlassina C et al.	10720960	Not a renal disease focus
1	Somatic mutations of the MET oncogene are selected during metastatic spread of human HNSC carcinomas.	Di Renzo MF et al.	10734314	Not a renal disease focus
1	Identification of fifteen novel PHEX gene mutations in Finnish patients with hypophosphatemic rickets.	Tyynismaa H et al.	10737991	Not a renal disease focus
1	Abnormal RNA expression of 11p15 imprinted genes and kidney developmental genes in Wilms' tumor.	Schwienbacher C et al.	10749116	Not a renal disease focus
1	VHL alterations in human clear cell renal cell carcinoma: association with advanced tumor stage and a novel hot spot mutation.	Brauch H et al.	10766184	Not a renal disease focus
1	An insertion/deletion polymorphism in intron 18 of the type B human natriuretic peptide receptor gene is not associated with cerebral infarction.	Rahmutula D et al.	10770265	Not a renal disease focus
1	Glutathione S-transferase M1, T1, and P1 polymorphisms as risk factors for renal cell carcinoma: a case-control study.	Sweeney C et al.	10794492	Not a renal disease focus
1	Familial clear cell renal cell carcinoma (FCRC): clinical features and mutation analysis of the VHL, MET, and CUL2 candidate genes.	Woodward ER et al.	10807693	Not a renal disease focus

For peer review only

1	Trisomy 4 leading to duplication of a mutated KIT allele in acute myeloid leukemia with mast cell involvement.	Beghini A et al.	10812167	Not a renal disease focus
1	Distribution of different HLA antigens in Greek hypertensives according to the angiotensin-converting enzyme genotype.	Diamantopoulos EJ et al.	10821349	Not a renal disease focus
1	Angiotensin-converting enzyme gene I/D polymorphism in malignant hypertension.	Stefansson B et al.	10855732	Not a renal disease focus
1	An intron 4 gene polymorphism in endothelial cell nitric oxide synthase might modulate volume-dependent hypertension in patients on hemodialysis.	Yokoyama K et al.	10867538	Not a renal disease focus
1	Elevated urinary albumin excretion is not linked to the angiotensin I-converting enzyme gene polymorphism in clinically healthy subjects.	Clausen P et al.	10872702	Not a renal disease focus
1	Functional analyses of amino acid substitutions Arg883Ser and Asp905Tyr of protein phosphatase-1 G-subunit.	Permana PA et al.	10873397	Not a renal disease focus
1	Functional synergism between the most common polymorphism in human alanine:glyoxylate aminotransferase and four of the most common disease-causing mutations.	Lumb MJ et al.	10960483	Not a renal disease focus
1	G-protein beta(3)-subunit C825T genotype and nephropathy in diabetes mellitus.	Beige J et al.	10978395	Not a renal disease focus
1	Alterations of the DNA repair gene OGG1 in human clear cell carcinomas of the kidney.	Audebert M et al.	10987279	Not a renal disease focus
1	Missense mutation of the MET gene detected in human glioma.	Moon YW et al.	11007037	Not a renal disease focus
1	Renal outcome and vascular morbidity in systemic lupus erythematosus (SLE): lack of association with the angiotensin-converting enzyme gene polymorphism.	Molad Y et al.	11071585	Not a renal disease focus
1	Serum arylesterase/diazoxonase activity and genetic polymorphisms in patients with type 2 diabetes.	Inoue M et al.	11092501	Not a renal disease focus
1	Detection of the association between a deletion polymorphism in the gene encoding angiotensin I-converting enzyme and advanced diabetic retinopathy.	Matsumoto A et al.	11106834	Not a renal disease focus
1	Proteinase 3 gene polymorphisms and Wegener's granulomatosis.	Gencik M et al.	11115080	Not a renal disease focus

For peer review only

1	alpha-adducin and angiotensin I-converting enzyme polymorphisms in essential hypertension.	Clark CJ et al.	11116113	Not a renal disease focus
1	Alleviating transcript insufficiency caused by Friedreich's ataxia triplet repeats.	Grabczyk E et al.	11121484	Not a renal disease focus
1	Glutathione transferase activities in renal carcinomas and adjacent normal renal tissues: factors influencing renal carcinogenesis induced by xenobiotics.	Delbanco EH et al.	11218045	Not a renal disease focus
1	Random mutagenesis-PCR to introduce alterations into defined DNA sequences for validation of SNP and mutation detection methods.	Nickerson ML et al.	11241843	Not a renal disease focus
1	Gain-of-function mutation at the extracellular domain of KIT in gastrointestinal stromal tumours.	Hirota S et al.	11276010	Not a renal disease focus
1	Gene-environment interactions in renal cell carcinoma.	Semenza JC et al.	11323315	Not a renal disease focus
1	von Hippel-Lindau protein mutants linked to type 2C VHL disease preserve the ability to downregulate HIF.	Hoffman MA et al.	11331612	Not a renal disease focus
1	Association of angiotensinogen M235T and A(-6)G gene polymorphisms with coronary heart disease with independence of essential hypertension: the PROCAGENE study. Prospective Cardiac Gene.	Rodriquez-Perez JC et al.	11345362	Not a renal disease focus
1	Cholesteryl ester transfer protein polymorphism associated with macroangiopathy in Japanese patients with type 2 diabetes.	Meguro S et al.	11369008	Not a renal disease focus
1	Mixed epithelial and stromal tumor of the kidney lacks the genetic alterations of cellular congenital mesoblastic nephroma.	Pierson CR et al.	11381370	Not a renal disease focus
1	Increased D allele frequency of the angiotensin-converting enzyme gene in pulmonary fibrosis.	Morrison CD et al.	11381371	Not a renal disease focus
1	Class II HLA associations with autoantibodies in scleroderma: a highly significant role for HLA-DP.	Gilchrist FC et al.	11393660	Not a renal disease focus
1	IRAK1b, a novel alternative splice variant of interleukin-1 receptor-associated kinase (IRAK), mediates interleukin-1 signaling and has prolonged stability.	Jensen LE et al.	11397809	Not a renal disease focus
1	Cancer risk in heterozygotes for ataxia-telangiectasia.	Geoffroy-Perez B et al.	11410879	Not a renal disease focus
1	VHL c.505 T>C mutation confers a high age related penetrance but no increased overall mortality.	Bender BU et al.	11483638	Not a renal disease focus

For peer review only

1	Angiotensin-converting enzyme gene polymorphism in patients with systemic lupus.	Prkacin I et al.	11505631	Not a renal disease focus
1	Polymorphism screening of the insulin receptor-related receptor gene (INSRR) on 1q in Pima Indians.	Wolford JK et al.	11513557	Not a renal disease focus
1	The pVHL-associated SCF ubiquitin ligase complex: molecular genetic analysis of elongin B and C, Rbx1 and HIF-1alpha in renal cell carcinoma.	Clifford SC et al.	11526493	Not a renal disease focus
1	Meningioma: a cytogenetic model of a complex benign human tumor, including data on 394 karyotyped cases.	Zang KD	11528114	Not a renal disease focus
1	Epigenetic inactivation of the RASSF1A 3p21.3 tumor suppressor gene in both clear cell and papillary renal cell carcinoma.	Morrissey C et al.	11585766	Not a renal disease focus
1	Crystal structures of NK1-heparin complexes reveal the basis for NK1 activity and enable engineering of potent agonists of the MET receptor.	Lietha D et al.	11597998	Not a renal disease focus
1	Detection of AGXT bgene mutations by denaturing high-performance liquid chromatography for diagnosis of hyperoxaluria type 1.	Pirulli D et al.	11699734	Not a renal disease focus
1	Identification of six novel MYH9 mutations and genotype-phenotype relationships in autosomal dominant macrothrombocytopenia with leukocyte inclusions.	Kunishima S et al.	11776386	Not a renal disease focus
1	Biallelic inactivation of the von Hippel-Lindau tumor suppressor gene in sporadic renal cell carcinoma.	Hamano K et al.	11792959	Not a renal disease focus
1	Association of the D allele of the angiotensin I converting enzyme polymorphism with malignant vascular injury.	Mayer NJ et al.	11836444	Not a renal disease focus
1	Comprehensive mutational analysis of the VHL gene in sporadic renal cell carcinoma: relationship to clinicopathological parameters.	Kondo K et al.	11921283	Not a renal disease focus
1	Mutations in the von Hippel-Lindau (VHL) gene refine differential diagnostic criteria in renal cell carcinoma.	Barnabas N et al.	11967908	Not a renal disease focus
1	Enhanced activity of variant phospholipase C-delta1 protein (R257H) detected in patients with coronary artery spasm.	Nakano T et al.	11980680	Not a renal disease focus
1	Inhibition of HIF is necessary for tumor suppression by the von Hippel-Lindau protein.	Kondo K et al.	12086860	Not a renal disease focus

For peer review only

1	Identification of cyclin D1 and other novel targets for the von Hippel-Lindau tumor suppressor gene by expression array analysis and investigation of cyclin D1 genotype as a modifier in von Hippel-Lindau disease.	Zatyka M et al.	12097293	Not a renal disease focus
1	ACE and PC-1 gene polymorphisms in normoalbuminuric Type 1 diabetic patients: a 10-year prospective study.	de Azevedo MJ et al.	12126783	Not a renal disease focus
1	Microsatellite instability and immunostaining for MSH-2 and MLH-1 in cutaneous and internal tumors from patients with the Muir-Torre syndrome.	Machin P et al.	12139636	Not a renal disease focus
1	No association between a genetic variant of the p22(phox) component of NAD(P)H oxidase and the incidence and progression of IgA nephropathy.	Wolf G et al.	12147803	Not a renal disease focus
1	Analysis of MGEA5 on 10q24.1-q24.3 encoding the beta-O-linked N-acetylglucosaminidase as a candidate gene for type 2 diabetes mellitus in Pima Indians.	Farook VS et al.	12359146	Not a renal disease focus
1	Distinct patterns of chromosomal losses in clinically synchronous and asynchronous bilateral renal cell carcinoma.	Kito H et al.	12442000	Not a renal disease focus
1	The relationship among the polymorphisms of SULT1A1, 1A2 and different types of cancers in Taiwanese.	Peng CT et al.	12469224	Not a renal disease focus
1	Variant screening of PRKAB2, a type 2 diabetes mellitus susceptibility candidate gene on 1q in Pima Indians.	Prochazka M et al.	12490143	Not a renal disease focus
1	Genetics, clinical and pathological features of glomerulonephritis associated with mutations of nonmuscle myosin IIA (Fechtner syndrome).	Ghiggeri GM et al.	12500226	Not a renal disease focus
1	Mutations in PRKCSH cause isolated autosomal dominant polycystic liver disease.	Li A et al.	12529853	Not a renal disease focus
1	Impact of clarithromycin resistance and CYP2C19 genetic polymorphism on treatment efficacy of Helicobacter pylori infection with lansoprazole- or rabeprazole-based triple therapy in Japan.	Miki I et al.	12544691	Not a renal disease focus
1	The inhibitory gamma subunit of the type 6 retinal cGMP phosphodiesterase functions to link c-Src and G-protein-coupled receptor kinase 2 in a signaling unit that regulates p42/p44 mitogen-activated protein kinase by epidermal growth factor.	Wan KF et al.	12624098	Not a renal disease focus

For peer review only

1	Serum extracellular superoxide dismutase in patients with type 2 diabetes: relationship to the development of micro- and macrovascular complications.	Kimura F et al.	12663605	Not a renal disease focus
1	Vascular endothelial growth factor gene polymorphism is associated with calcium oxalate stone disease.	Chen WC et al.	12719950	Not a renal disease focus
1	Proinflammatory genotype of interleukin-1 and interleukin-1 receptor antagonist is associated with ESRD in proteinase 3-ANCA vasculitis patients.	Borgmann S et al.	12722027	Not a renal disease focus
1	Mutation analysis and clinical implications of von Willebrand factor-cleaving protease deficiency.	Assink K et al.	12753286	Not a renal disease focus
1	Association of homozygous deletion of the Humhv3005 and the VH3-30.3 genes with renal involvement in systemic lupus erythematosus.	Cho ML et al.	12765304	Not a renal disease focus
1	A biallelic gene polymorphism of CYP11B2 predicts increased aldosterone to renin ratio in selected hypertensive patients.	Nicod J et al.	12788845	Not a renal disease focus
1	MYH9-related disease: May-Hegglin anomaly, Sebastian syndrome, Fechtner syndrome, and Epstein syndrome are not distinct entities but represent a variable expression of a single illness.	Seri M et al.	12792306	Not a renal disease focus
1	A single nucleotide polymorphism in the matrix metalloproteinase-1 promoter is associated with conventional renal cell carcinoma.	Hirata H et al.	12845675	Not a renal disease focus
1	Aberrant methylation and silencing of ARHI, an imprinted tumor suppressor gene in which the function is lost in breast cancers.	Yuan J et al.	12874023	Not a renal disease focus
1	Oxidative stress-related factors in Bartter's and Gitelman's syndromes: relevance for angiotensin II signalling.	Calo LA et al.	12897089	Not a renal disease focus
1	Frequent allelic changes at chromosome 7q34 but lack of mutation of the BRAF in papillary renal cell tumors.	Nagy A et al.	12918080	Not a renal disease focus
1	Relevance of nuclear and cytoplasmic von hippel lindau protein expression for renal carcinoma progression.	Schraml P et al.	12937142	Not a renal disease focus
1	GBPI, a novel gastrointestinal- and brain-specific PP1-inhibitory protein, is activated by PKC and inactivated by PKA.	Liu QR et al.	12974676	Not a renal disease focus
1	Renin-angiotensin system gene polymorphisms: assessment of the risk of coronary heart disease.	Buraczynska M et al.	14502296	Not a renal disease focus
1	Glutathione S-transferases M1-1 and T1-1 as risk modifiers for renal cell cancer associated with occupational exposure to chemicals.	Buzio L et al.	14504370	Not a renal disease focus

For peer review only

1	Expression of the proto-oncogene Axl in renal cell carcinoma.	Chung BI et al.	14565870	Not a renal disease focus
1	Paraoxonase (Pon1) Q192R polymorphism and serum Pon1 activity in diabetic patients on maintenance hemodialysis.	Zhang B et al.	14579940	Not a renal disease focus
1	Endothelial nitric oxide synthase gene intron 4 polymorphism in type 2 diabetes mellitus.	Ksiazek P et al.	14580231	Not a renal disease focus
1	Cytochrome P450 and manganese superoxide dismutase genes polymorphisms in systemic lupus erythematosus.	Yen JH et al.	14611903	Not a renal disease focus
1	Peripheral vascular disease in Type 2 diabetic Chinese patients: associations with metabolic indices, concomitant vascular disease and genetic factors.	Thomas GN et al.	14632699	Not a renal disease focus
1	The rapid effects of 1,25-dihydroxyvitamin D3 require the vitamin D receptor and influence 24-hydroxylase activity: studies in human skin fibroblasts bearing vitamin D receptor mutations.	Nguyen TM et al.	14665637	Not a renal disease focus
1	Renovascular disease: effect of ACE gene deletion polymorphism and endovascular revascularization.	Pizzolo F et al.	14718831	Not a renal disease focus
1	Genetic characterization and structural analysis of VHL Spanish families to define genotype-phenotype correlations.	Ruiz-Llorente S et al.	14722919	Not a renal disease focus
1	Congenital disorder of oxygen sensing: association of the homozygous Chuvash polycythemia VHL mutation with thrombosis and vascular abnormalities but not tumors.	Gordeuk VR et al.	14726398	Not a renal disease focus
1	Defects in translational regulation mediated by the alpha subunit of eukaryotic initiation factor 2 inhibit antiviral activity and facilitate the malignant transformation of human fibroblasts.	Perkins DJ et al.	14966282	Not a renal disease focus
1	Polymorphisms of the CYP1B1 gene as risk factors for human renal cell cancer.	Sasaki M et al.	15041720	Not a renal disease focus
1	Abnormal hepatocystin caused by truncating PRKCSH mutations leads to autosomal dominant polycystic liver disease.	Drenth JP et al.	15057895	Not a renal disease focus
1	KIT expression in chromophobe renal cell carcinoma: comparative immunohistochemical analysis of KIT expression in different renal cell neoplasms.	Petit A et al.	15105658	Not a renal disease focus

For peer review only

1	Association of the serum and glucocorticoid regulated kinase (sgk1) gene with QT interval.	Busjahn A et al.	15107590	Not a renal disease focus
1	Three novel missense mutations of WNK4, a kinase mutated in inherited hypertension, in Japanese hypertensives: implication of clinical phenotypes.	Kamide K et al.	15110905	Not a renal disease focus
1	Intron 4 polymorphism of the endothelial nitric oxide synthase gene is associated with the development of lupus nephritis.	Lee YH et al.	15119548	Not a renal disease focus
1	Identification of PIK3C3 promoter variant associated with bipolar disorder and schizophrenia.	Stopkova P et al.	15121481	Not a renal disease focus
1	The role of the LRPPRC (leucine-rich pentatricopeptide repeat cassette) gene in cytochrome oxidase assembly: mutation causes lowered levels of COX (cytochrome c oxidase) I and COX III mRNA.	Xu F et al.	15139850	Not a renal disease focus
1	A naturally occurring human Nedd4-2 variant displays impaired ENaC regulation in Xenopus laevis oocytes.	Fouladkou F et al.	15140763	Not a renal disease focus
1	Increased amount of the angiotensin-converting enzyme (ACE) mRNA originating from the ACE allele with deletion.	Suehiro T et al.	15164285	Not a renal disease focus
1	G protein beta3 subunit C825T polymorphism in primary IgA nephropathy.	Thibaudin L et al.	15200440	Not a renal disease focus
1	A novel missense substitution (Val1483Ile) in the fatty acid synthase gene (FAS) is associated with percentage of body fat and substrate oxidation rates in nondiabetic Pima Indians.	Kovacs P et al.	15220220	Not a renal disease focus
1	Functional analysis of polymorphisms in the promoter regions of genes on 22q11.	Hoogendoorn B et al.	15221787	Not a renal disease focus
1	Clinical implications of mutation analysis in primary hyperoxaluria type 1.	van Woerden CS et al.	15253729	Not a renal disease focus
1	Paraoxonase 1 Gln/Arg polymorphism is associated with the risk of microangiopathy in Type 2 diabetes mellitus.	Murata M et al.	15270786	Not a renal disease focus
1	Identification of 108 SNPs in TSC, WNK1, and WNK4 and their association with hypertension in a Japanese general population.	Kokubo Y et al.	15309683	Not a renal disease focus
1	Association of a haplotype of matrix metalloproteinase (MMP)-1 and MMP-3 polymorphisms with renal cell carcinoma.	Hirata H et al.	15319295	Not a renal disease focus
1	Genetic risk of atherosclerotic renal artery disease: the candidate gene approach in a renal angiography cohort.	van Onna M et al.	15326089	Not a renal disease focus

For peer review only

1	Evaluation of mutation screening as a first line test for the diagnosis of the primary hyperoxalurias.	Rumsby G et al.	15327387	Not a renal disease focus
1	G-protein beta3 subunit gene C825T polymorphism in patients with vesico-ureteric reflux.	Zagradisnik B et al.	15337465	Not a renal disease focus
1	An intronic variant of the TGFBR1 gene is associated with carcinomas of the kidney and bladder.	Chen T et al.	15382067	Not a renal disease focus
1	Polymorphisms in the 5'-upstream region of the PKCbeta gene in Japanese patients with Type 2 diabetes.	Ikeda Y et al.	15384959	Not a renal disease focus
1	Glutathione S-transferase T1 deletion is a risk factor for developing end-stage renal disease in diabetic patients.	Yang Y et al.	15492856	Not a renal disease focus
1	CHEK2 is a multiorgan cancer susceptibility gene.	Cybulski C et al.	15492928	Not a renal disease focus
1	Molecular basis of hypoxanthine-guanine phosphoribosyltransferase deficiency in Italian Lesch-Nyhan patients: identification of nine novel mutations.	Bertelli M et al.	15505382	Not a renal disease focus
1	Distinct patterns of abnormal GNAS imprinting in familial and sporadic pseudohypoparathyroidism type IB.	Liu J et al.	15537666	Not a renal disease focus
1	Chromophobe renal cell carcinoma: a comparative study of histological, immunohistochemical and ultrastructural features using high throughput tissue microarray.	Abrahams NA et al.	15569050	Not a renal disease focus
1	Association of a functional single-nucleotide polymorphism of PTPN22, encoding lymphoid protein phosphatase, with rheumatoid arthritis and systemic lupus erythematosus.	Orozco G et al.	15641066	Not a renal disease focus
1	Altered gene expression in phenotypically normal renal cells from carriers of tumor suppressor gene mutations.	Stoyanova R et al.	15662135	Not a renal disease focus
1	Thrombin activatable fibrinolysis inhibitor in BehÃ§et's disease.	Donmez A et al.	15668188	Not a renal disease focus
1	Allelic variants of the human scavenger receptor class B type 1 and paraoxonase 1 on coronary heart disease: genotype-phenotype correlations.	Rodriquez-Esparragon F et al.	15681296	Not a renal disease focus
1	A novel recessive mutation in fibroblast growth factor-23 causes familial tumoral calcinosis.	Larsson T et al.	15687325	Not a renal disease focus

For peer review only

1	DNase II polymorphisms associated with risk of renal disorder among systemic lupus erythematosus patients.	Shin HD et al.	15723160	Not a renal disease focus
1	Analysis of the apolipoprotein(a) size polymorphism in patients with systemic lupus erythematosus.	Peros E et al.	15754029	Not a renal disease focus
1	Relationship of eNOS gene variants to diseases that have in common an endothelial cell dysfunction.	Heltianu C et al.	15784171	Not a renal disease focus
1	Variable number of tandem repeat of the 5'-flanking region of type-C human natriuretic peptide receptor gene influences blood pressure levels in obesity-associated hypertension.	Aoi N et al.	15785005	Not a renal disease focus
1	Renin-angiotensin system gene polymorphisms predict the progression to renal insufficiency among Asians with lupus nephritis.	Parsa A et al.	15789057	Not a renal disease focus
1	A novel STX16 deletion in autosomal dominant pseudohypoparathyroidism type Ib redefines the boundaries of a cis-acting imprinting control element of GNAS.	Linglart A et al.	15800843	Not a renal disease focus
1	Overexpression of human alanine:glyoxylate aminotransferase in Escherichia coli: renaturation from guanidine-HCl and affinity for pyridoxal phosphate co-factor.	Coulter-Mackie MB et al.	15802217	Not a renal disease focus
1	Association between plasma activities of semicarbazide-sensitive amine oxidase and angiotensin-converting enzyme in patients with type 1 diabetes mellitus.	Boomsma F et al.	15830186	Not a renal disease focus
1	Relationship of bradykinin B2 receptor gene polymorphism with essential hypertension and left ventricular hypertrophy.	Fu Y et al.	15894833	Not a renal disease focus
1	Genetic variation in the bleomycin hydrolase gene and bleomycin-induced pulmonary toxicity in germ cell cancer patients.	Nuver J et al.	15900213	Not a renal disease focus
1	Prevalence of von Hippel-Lindau gene mutations in sporadic renal cell carcinoma: results from The Netherlands cohort study.	van Houwelingen KP et al.	15932632	Not a renal disease focus
1	Inducible nitric oxide synthase polymorphism is associated with susceptibility to Henoch-SchÃ¶nlein purpura in northwestern Spain.	Martin J et al.	15940772	Not a renal disease focus
1	Association between CYP2C9 slow metabolizer genotypes and severe hypoglycaemia on medication with sulphonylurea hypoglycaemic agents.	Holstein A et al.	15963101	Not a renal disease focus

For peer review only

1	The major allele of the alanine:glyoxylate aminotransferase gene: nine novel mutations and polymorphisms associated with primary hyperoxaluria type 1.	Coulter-Mackie MB et al.	15963748	Not a renal disease focus
1	Angiotensin-converting enzyme (ACE) haplotypes and cyclosporine A (CsA) response: a model of the complex relationship between ACE quantitative trait locus and pathological phenotypes.	Catarsi P et al.	16002416	Not a renal disease focus
1	Increased frequency of the angiotensin-converting enzyme gene D-allele is associated with noninfectious pulmonary dysfunction following allogeneic stem cell transplant.	Onizuka M et al.	16044138	Not a renal disease focus
1	Association between polymorphisms of the renin-angiotensin system and more severe histological forms of lupus nephritis.	Sprovieri SR et al.	16047641	Not a renal disease focus
1	The R620W C/T polymorphism of the gene PTPN22 is associated with SLE independently of the association of PDCD1.	Reddy MV et al.	16052172	Not a renal disease focus
1	Does complement factor B have a role in the pathogenesis of atypical HUS?	Kavanagh D et al.	16061287	Not a renal disease focus
1	Association of NEDD4L ubiquitin ligase with essential hypertension.	Russo CJ et al.	16103266	Not a renal disease focus
1	CYP3A5 genotype is associated with elevated blood pressure.	Fromm MF et al.	16141800	Not a renal disease focus
1	Complement factor B allotypes in the susceptibility and severity of coeliac disease in patients and relatives.	da Rosa Utiyama SR et al.	16164698	Not a renal disease focus
1	The Prevalence of CYP2C8, 2C9, 2J2, and soluble epoxide hydrolase polymorphisms in African Americans with hypertension.	Dreisbach AW et al.	16202848	Not a renal disease focus
1	Natural history of Fabry disease in females in the Fabry Outcome Survey.	Deegan PB et al.	16227523	Not a renal disease focus
1	ADAM33: a newly identified gene in the pathogenesis of asthma.	Holgate ST et al.	16257631	Not a renal disease focus
1	Alternative splicing of fibroblast growth factor receptor 3 produces a secreted isoform that inhibits fibroblast growth factor-induced proliferation and is repressed in urothelial carcinoma cell lines.	Tomlinson DC et al.	16288035	Not a renal disease focus
1	Homozygous and compound heterozygous mutations in ZMPSTE24 cause the laminopathy restrictive dermopathy.	Moulson CL et al.	16297189	Not a renal disease focus

For peer review only

1	The PTPN22 620W allele is a risk factor for Wegener's granulomatosis.	Jagiello P et al.	16320352	Not a renal disease focus
1	Hypoxia-regulated expression of attenuated diphtheria toxin A fused with hypoxia-inducible factor-1alpha oxygen-dependent degradation domain preferentially induces apoptosis of hypoxic cells in solid tumor.	Koshikawa N et al.	16357173	Not a renal disease focus
1	Is factor V Leiden a risk factor for thrombotic microangiopathies without severe ADAMTS 13 deficiency?	Krieg S et al.	16411392	Not a renal disease focus
1	Int7G24A variant of transforming growth factor-beta receptor type I is associated with invasive breast cancer.	Chen T et al.	16428477	Not a renal disease focus
1	Antiepidermal growth factor variant III scFv fragment: effect of radioiodination method on tumor targeting and normal tissue clearance.	Shankar S et al.	16459265	Not a renal disease focus
1	Matrix metalloproteinase-9 polymorphisms and renal cell carcinoma in a Japanese population.	Awakura Y et al.	16466849	Not a renal disease focus
1	Association of CYP3A5 genotypes with blood pressure and renal function in African families.	Bochud M et al.	16612255	Not a renal disease focus
1	Relationship of serum paraoxonase 1 activity and paraoxonase 1 genotype to risk of systemic lupus erythematosus.	Tripi LM et al.	16729301	Not a renal disease focus
1	CCL18: a urinary marker of Gaucher cell burden in Gaucher patients.	Boot RG et al.	16736095	Not a renal disease focus
1	Adult leydig cell tumors of the testis caused by germline fumarate hydratase mutations.	Carvajal-Carmona LG et al.	16757530	Not a renal disease focus
1	Are the angiotensin-converting enzyme gene and activity risk factors for stroke?	Dikmen M et al.	16791358	Not a renal disease focus
1	Increased neutrophil membrane expression and plasma level of proteinase 3 in systemic vasculitis are not a consequence of the - 564 A/G promotor polymorphism.	Abdgawad M et al.	16792675	Not a renal disease focus
1	Slight association between type 1 diabetes and "ff" VDR FokI genotype in patients from the Italian Lazio Region. Lack of association with diabetes complications.	Capoluongo E et al.	16806146	Not a renal disease focus

1	No association of the CYP3A5*1 allele with blood pressure and left ventricular mass and geometry: the KORA/MONICA Augsburg echocardiographic substudy.	Lieb W et al.	16822233	Not a renal disease focus
1	Autosomal-dominant pseudohypoparathyroidism type Ib is caused by different microdeletions within or upstream of the GNAS locus.	Juppner H et al.	16831926	Not a renal disease focus
1	VHL P25L is not a pathogenic von Hippel-Lindau mutation: a family study.	Pettman RK et al.	16884327	Not a renal disease focus
1	ACE gene insertion/deletion polymorphism modulates capillary permeability in hypertension.	Dell'omo G et al.	16889537	Not a renal disease focus
1	Allelic variation in the CNDP1 gene and its lack of association with longevity and coronary heart disease.	Zschocke J et al.	16965804	Not a renal disease focus
1	Ubiquitin ligase gp78 increases solubility and facilitates degradation of the Z variant of alpha-1-antitrypsin.	Shen Y et al.	16979136	Not a renal disease focus
1	NFkappaB and its inhibitor IkappaB in relation to type 2 diabetes and its microvascular and atherosclerotic complications.	Romzova M et al.	17002901	Not a renal disease focus
1	Predicting the impact of population level risk reduction in cardio-vascular disease and stroke on acute hospital admission rates over a 5 year period--a pilot study.	Whitfield MD et al.	17084425	Not a renal disease focus
1	Increased expression of angiotensin II type 1 receptor (AGTR1) in heart transplant recipients with recurrent rejection.	Yamani MH et al.	17097490	Not a renal disease focus
1	Effect of synthetic corticosteroids on vascular reactivity in the human forearm.	Mangos GJ et al.	17132537	Not a renal disease focus
1	Association of genetic polymorphisms of ACADSB and COMT with human hypertension.	Kamide K et al.	17143180	Not a renal disease focus
1	Role of the STK15 Phe31Ile polymorphism in renal cell carcinoma.	Hammerschmied CG et al.	17143471	Not a renal disease focus
1	Genetic and epigenetic analysis of CHEK2 in sporadic breast, colon, and ovarian cancers.	Williams LH et al.	17145815	Not a renal disease focus
1	Blastemal expression of type I insulin-like growth factor receptor in Wilms' tumors is driven by increased copy number and correlates with relapse.	Natrajan R et al.	17145858	Not a renal disease focus

For peer review only

1	Identification of a novel BBS gene (BBS12) highlights the major role of a vertebrate-specific branch of chaperonin-related proteins in Bardet-Biedl syndrome.	Stoetzel C et al.	17160889	Not a renal disease focus
1	Association of the C825T polymorphism of the G-protein beta3 subunit gene with hypertension, obesity, hyperlipidemia, insulin resistance, diabetes, diabetic complications, and diabetic therapies among Japanese.	Hayakawa T et al.	17161225	Not a renal disease focus
1	Adrenal hyperplasia and adenomas are associated with inhibition of phosphodiesterase 11A in carriers of PDE11A sequence variants that are frequent in the population.	Horvath A et al.	17178847	Not a renal disease focus
1	Mucinous tubular and spindle cell carcinoma of kidney is probably a variant of papillary renal cell carcinoma with spindle cell features.	Shen SS et al.	17240302	Not a renal disease focus
1	EGFR sequence variations and real-time quantitative polymerase chain reaction analysis of gene dosage in brain metastases of solid tumors.	Franco-Hernandez C et al.	17284372	Not a renal disease focus
1	Nosocomial outbreak of CTX-M-15-producing E. coli in Norway.	Naseer U et al.	17295678	Not a renal disease focus
1	Association of genotypes of thrombin-activatable fibrinolysis inhibitors with thrombotic microangiopathies--a pilot study.	Sucker C et al.	17327284	Not a renal disease focus
1	Functional polymorphism in human CYP4F2 decreases 20-HETE production.	Stec DE et al.	17341693	Not a renal disease focus
1	Immunoexpression of lactoferrin in human sporadic renal cell carcinomas.	Giuffre G et al.	17390038	Not a renal disease focus
1	Association of ATP1A1 and dear single-nucleotide polymorphism haplotypes with essential hypertension: sex-specific and haplotype-specific effects.	Glorioso N et al.	17446437	Not a renal disease focus
1	Comprehensive mutation screening in 55 probands with type 1 primary hyperoxaluria shows feasibility of a gene-based diagnosis.	Monico CG et al.	17460142	Not a renal disease focus
1	Mutations in the SBDS gene in acquired aplastic anemia.	Calado RT et al.	17478638	Not a renal disease focus
1	Re-assessment of the influence of polymorphisms of phase-II metabolic enzymes on renal cell cancer risk of trichloroethylene-exposed workers.	Wiesenhutter B et al.	17479278	Not a renal disease focus

For peer review only

1	Uncommon CHEK2 mis-sense variant and reduced risk of tobacco-related cancers: case control study.	Brennan P et al.	17517688	Not a renal disease focus
1	Impact of maternal angiotensinogen M235T polymorphism and angiotensin-converting enzyme insertion/deletion polymorphism on blood pressure, protein excretion and fetal outcome in pregnancy.	Pfab T et al.	17563539	Not a renal disease focus
1	Lack of association between endothelial nitric oxide synthase gene polymorphisms, microalbuminuria and endothelial dysfunction in hypertensive men.	Dell'Omo G et al.	17563560	Not a renal disease focus
1	Circulating protein biomarkers of pharmacodynamic activity of sunitinib in patients with metastatic renal cell carcinoma: modulation of VEGF and VEGF-related proteins.	Deprimo SE et al.	17605814	Not a renal disease focus
1	Glutathione S-transferase polymorphisms, cruciferous vegetable intake and cancer risk in the Central and Eastern European Kidney Cancer Study.	Moore LE et al.	17617661	Not a renal disease focus
1	Effects of angiotensin-converting enzyme gene polymorphism and serum vitamin D levels on ambulatory blood pressure measurement and left ventricular mass in Turkish hypertensive population.	Kulah E et al.	17625392	Not a renal disease focus
1	Manganese superoxide dismutase (Mn-SOD) gene polymorphisms in urolithiasis.	Tugcu V et al.	17628794	Not a renal disease focus
1	Angiotensin-converting enzyme gene polymorphism in Kuwaiti patients with systemic lupus erythematosus.	Al-Awadhi AM et al.	17631741	Not a renal disease focus
1	MDM2 SNP309 polymorphism as risk factor for susceptibility and poor prognosis in renal cell carcinoma.	Hirata H et al.	17634539	Not a renal disease focus
1	Protein expression and mutational analysis of epidermal growth factor receptor in renal angiomyolipomas.	Lim SD et al.	17685929	Not a renal disease focus
1	Germline VHL gene mutations in three Serbian families with von Hippel-Lindau disease.	Stanojevic BR et al.	17688370	Not a renal disease focus
1	The E3 ligase HACE1 is a critical chromosome 6q21 tumor suppressor involved in multiple cancers.	Zhang L et al.	17694067	Not a renal disease focus
1	Genetic variation in the paraoxonase-3 (PON3) gene is associated with serum PON1 activity.	Sanghera DK et al.	17900266	Not a renal disease focus
1	Genetic polymorphisms in OGG1 and their association with angiomyolipoma, a benign kidney tumor in patients with tuberous sclerosis.	Habib SL et al.	17932460	Not a renal disease focus

For peer review only

1	Human G(salpha) mutant causes pseudohypoparathyroidism type Ia/neonatal diarrhea, a potential cell-specific role of the palmitoylation cycle.	Makita N et al.	17962410	Not a renal disease focus
1	Association of the nitric oxide synthase (eNOS) gene polymorphism with increased risk for both lupus glomerulonephritis and rheumatoid arthritis in a single genetically homogeneous population.	Vazgiourakis V et al.	17971359	Not a renal disease focus
1	Lack of association between matrix metalloproteinase-1 (MMP-1) promoter polymorphism and risk of renal cell carcinoma.	Piccoli MF et al.	17980059	Not a renal disease focus
1	Differential regulation of serum- and glucocorticoid-inducible kinase 1 (SGK1) splice variants based on alternative initiation of transcription.	Simon P et al.	17982254	Not a renal disease focus
1	Polymorphisms in genes related to activation or detoxification of carcinogens might interact with smoking to increase renal cancer risk: results from The Netherlands Cohort Study on diet and cancer.	Smits KM et al.	17982751	Not a renal disease focus
1	Dominant-negative HIF-3 alpha 4 suppresses VHL-null renal cell carcinoma progression.	Maynard MA et al.	17998805	Not a renal disease focus
1	The extracolonic cancer spectrum in females with the common 'South African' hMLH1 c.C1528T mutation.	Blokhuis MM et al.	18049911	Not a renal disease focus
1	Position of nonmuscle myosin heavy chain IIA (NMMHC-IIA) mutations predicts the natural history of MYH9-related disease.	Pecci A et al.	18059020	Not a renal disease focus
1	Lack of association of a functional single nucleotide polymorphism of PTPN22, encoding lymphoid protein phosphatase, with susceptibility to Henoch-SchÃ¶nlein purpura.	Orozco G et al.	18078626	Not a renal disease focus
1	Progesterone receptor reactivity in renal oncocytoma and chromophobe renal cell carcinoma.	Mai KT et al.	18081814	Not a renal disease focus
1	Constant allelic alteration on chromosome 16p (TSC2 gene) in perivascular epithelioid cell tumour (PEComa): genetic evidence for the relationship of PEComa with angiomyolipoma.	Pan CC et al.	18085521	Not a renal disease focus
1	Association of angiotensin-converting enzyme gene insertion/deletion polymorphism with metabolic syndrome in Iranians with type 2 diabetes mellitus.	Nikzamir A et al.	18154415	Not a renal disease focus
1	Association of CDKAL1, IGF2BP2, CDKN2A/B, HHEX, SLC30A8, and KCNJ11 with susceptibility to type 2 diabetes in a Japanese population.	Omori S et al.	18162508	Not a renal disease focus

For peer review only

1	Renal oncocytoma with and without intravascular extension into the branches of renal vein have the same morphological, immunohistochemical and genetic features.	Hes O et al.	18196270	Not a renal disease focus
1	Association of SGK1 gene polymorphisms with type 2 diabetes.	Schwab M et al.	18209482	Not a renal disease focus
1	Association of a functional cytochrome P450 4F2 haplotype with urinary 20-HETE and hypertension.	Liu H et al.	18235092	Not a renal disease focus
1	Haplotypes of the HRES-1 endogenous retrovirus are associated with development and disease manifestations of systemic lupus erythematosus.	Pullmann R Jr et al.	18240231	Not a renal disease focus
1	Genetic and epigenetic alterations in the von hippel-lindau gene: the influence on renal cancer prognosis.	Smits KM et al.	18245539	Not a renal disease focus
1	AMP-activated protein kinase inhibits transforming growth factor-beta-induced Smad3-dependent transcription and myofibroblast transdifferentiation.	Mishra R et al.	18250161	Not a renal disease focus
1	Oncocytic papillary renal cell carcinoma with solid architecture: mimic of renal oncocytoma.	Mai KT et al.	18251779	Not a renal disease focus
1	Structure of the N-terminal region of complement factor H and conformational implications of disease-linked sequence variations.	Hocking HG et al.	18252712	Not a renal disease focus
1	The role of type 1 and type 2 5'-deiodinase in the pathophysiology of the 3,5,3'-triiodothyronine toxicosis of McCune-Albright syndrome.	Celi FS et al.	18349068	Not a renal disease focus
1	N-acetyltransferase 8, a positional candidate for blood pressure and renal regulation: resequencing, association and in silico study.	Juhanson P et al.	18402670	Not a renal disease focus
1	Association analysis in african americans of European-derived type 2 diabetes single nucleotide polymorphisms from whole-genome association studies.	Lewis JP et al.	18443202	Not a renal disease focus
1	PPP2R2B CAG repeat length in the Han Chinese in Taiwan: Association analyses in neurological and psychiatric disorders and potential functional implications.	Chen CM et al.	18484086	Not a renal disease focus
1	Glutathione S-transferase variants and hypertension.	Delles C et al.	18551009	Not a renal disease focus
1	Allelic loss analysis of tumor suppressor genes regardless of heterozygosity: von Hippel-Lindau gene loss in renal cell carcinoma.	Mochida J et al.	18554638	Not a renal disease focus

For peer review only

1	Renal cell carcinoma, occupational pesticide exposure and modification by glutathione S-transferase polymorphisms.	Karami S et al.	18566013	Not a renal disease focus
1	No association between single nucleotide polymorphisms and the development of nephrotoxicity after orthotopic heart transplantation.	Klauke B et al.	18582803	Not a renal disease focus
1	Association of GSTM3 intron 6 variant with cigarette smoking, tobacco chewing and alcohol as modifier factors for prostate cancer risk.	Kesarwani P et al.	18668224	Not a renal disease focus
1	Association of renin-angiotensin and endothelial nitric oxide synthase gene polymorphisms with blood pressure progression and incident hypertension: prospective cohort study.	Conen D et al.	18698212	Not a renal disease focus
1	Association of angiotensin-converting enzyme gene dimorphisms with severity of lupus disease.	Rabbani MA et al.	18711292	Not a renal disease focus
1	Development of human cell models for assessing the carcinogenic potential of chemicals.	Pang Y et al.	18778725	Not a renal disease focus
1	SIRT1 genetic variants associate with the metabolic response of Caucasians to a controlled lifestyle intervention--the TULIP Study.	Weyrich P et al.	19014491	Not a renal disease focus
1	Influence of XPD and APE1 DNA repair gene polymorphism on bladder cancer susceptibility in north India.	Gangwar R et al.	19041121	Not a renal disease focus
1	Genetic variants in hypertensive patients with coronary artery disease and coexisting atheromatous renal artery stenosis.	Szperl M et al.	19043368	Not a renal disease focus
1	When should genetic testing be obtained in a patient with phaeochromocytoma or paraganglioma?	Erlic Z et al.	19067729	Not a renal disease focus
1	Identification of novel mutations and sequence variation in the Zellweger syndrome spectrum of peroxisome biogenesis disorders.	Yik WY et al.	19105186	Not a renal disease focus
1	High aldosterone-to-renin variants of CYP11B2 and pregnancy outcome.	Escher G et al.	19151144	Not a renal disease focus
1	The Leu262Val polymorphism of presenilin associated rhomboid like protein (PARL) is associated with earlier onset of type 2 diabetes and increased urinary microalbumin creatinine ratio in an Irish case-control population.	Hatunic M et al.	19185381	Not a renal disease focus
1	A pilot study of genetic polymorphisms and hemodialysis vascular access thrombosis.	Brophy DF et al.	19210273	Not a renal disease focus

For peer review only

1	CYP1B1 mutations in Spanish patients with primary congenital glaucoma: phenotypic and functional variability.	Campos-Mollo E et al.	19234632	Not a renal disease focus
1	Impact of nucleotide excision repair ERCC2 and base excision repair APEX1 genes polymorphism and its association with recurrence after adjuvant BCG immunotherapy in bladder cancer patients of North India.	Gangawar R et al.	19242824	Not a renal disease focus
1	Functional basis of protection against age-related macular degeneration conferred by a common polymorphism in complement factor B.	Montes T et al.	19255449	Not a renal disease focus
1	Insight into mechanism of oxidative DNA damage in angiomyolipomas from TSC patients.	Habib SL	19265534	Not a renal disease focus
1	Alu-Alu recombination underlies the vast majority of large VHL germline deletions: Molecular characterization and genotype-phenotype correlations in VHL patients.	Franke G et al.	19280651	Not a renal disease focus
1	Loss of function of Sco1 and its interaction with cytochrome c oxidase.	Stiburek L et al.	19295170	Not a renal disease focus
1	Expression of hepatocyte growth factor and its receptor met in Wilms' tumors and nephrogenic rests reflects their roles in kidney development.	Vuononvirta R et al.	19318497	Not a renal disease focus
1	A human polymorphism affects NEDD4L subcellular targeting by leading to two isoforms that contain or lack a C2 domain.	Garrone NF et al.	19364400	Not a renal disease focus
1	The action and mode of binding of thiazolidinedione ligands at free fatty acid receptor 1.	Smith NJ et al.	19398560	Not a renal disease focus
1	Tropical calcific pancreatitis and its association with CTRC and SPINK1 (p.N34S) variants.	Derikx MH et al.	19404200	Not a renal disease focus
1	GSTM1, GSTM3 and GSTT1 gene variants and risk of benign prostate hyperplasia in North India.	Mittal RD et al.	19407363	Not a renal disease focus
1	Structural bioinformatics mutation analysis reveals genotype-phenotype correlations in von Hippel-Lindau disease and suggests molecular mechanisms of tumorigenesis.	Forman JR et al.	19408298	Not a renal disease focus
1	Caspase 9 and caspase 8 gene polymorphisms and susceptibility to bladder cancer in north Indian population.	Gangwar R et al.	19412632	Not a renal disease focus
1	Is polymorphism within eNOS gene associated with the late onset of myocardial infarction? A pilot study.	Gluba A et al.	19505886	Not a renal disease focus

For peer review only

1	Functional phosphodiesterase 11A mutations may modify the risk of familial and bilateral testicular germ cell tumors.	Horvath A et al.	19549888	Not a renal disease focus
1	Analysis of germline variants in CDH1, IGFBP3, MMP1, MMP3, STK15 and VEGF in familial and sporadic renal cell carcinoma.	Ricketts C et al.	19551141	Not a renal disease focus
1	The calcineurin homologous protein-1 increases Na(+)/H(+) -exchanger 3 trafficking via ezrin phosphorylation.	Di Sole F et al.	19556366	Not a renal disease focus
1	A functional variant of NEDD4L is associated with hypertension, antihypertensive response, and orthostatic hypotension.	Luo F et al.	19635985	Not a renal disease focus
1	A significantly joint effect between arsenic and occupational exposures and risk genotypes/diplotypes of CYP2E1, GSTO1 and GSTO2 on risk of urothelial carcinoma.	Wang YH et al.	19686770	Not a renal disease focus
1	Genetic polymorphisms in genes encoding antioxidant enzymes are associated with diabetic retinopathy in type 1 diabetes.	Hovnik T et al.	19752172	Not a renal disease focus
1	Loss of heterozygosity at 2q37 in sporadic Wilms' tumor: putative role for miR-562.	Drake KM et al.	19789318	Not a renal disease focus
1	Apolipoprotein E/C1 locus variants modify renal cell carcinoma risk.	Moore LE et al.	19808960	Not a renal disease focus
1	Do DNA repair genes OGG1, XRCC3 and XRCC7 have an impact on susceptibility to bladder cancer in the North Indian population?	Gangwar R et al.	19815090	Not a renal disease focus
1	Angiotensin-converting enzyme and angiotensin II receptor subtype 2 genotypes in type 1 diabetes and severe hypoglycaemia requiring emergency treatment: a case cohort study.	Pedersen-Bjergaard U et al.	19820429	Not a renal disease focus
1	Clinical predictors and algorithm for the genetic diagnosis of pheochromocytoma patients.	Erlic Z et al.	19825962	Not a renal disease focus
1	A polymorphism within the fructosamine-3-kinase gene is associated with HbA1c Levels and the onset of type 2 diabetes mellitus.	Mohas M et al.	19834870	Not a renal disease focus
1	A study on the association between angiotensin-I converting enzyme I/D dimorphism and type-2 diabetes mellitus.	Chmaisse HN et al.	19861867	Not a renal disease focus
1	The Int7G24A variant of transforming growth factor-beta receptor type I is a risk factor for colorectal cancer in the male Spanish population: a case-control study.	Castillejo A et al.	19930569	Not a renal disease focus

For peer review only

1	The role of polymorphisms within paraoxonases (192 Gln/Arg in PON1 and 311Ser/Cys in PON2) in the modulation of cardiovascular risk: a pilot study.	Gluba A et al.	19939821	Not a renal disease focus
1	Endothelial nitric oxide synthase and nicotinamide adenosine dinucleotide phosphate oxidase p22phox gene (C242T) polymorphisms and systemic lupus erythematosus in a Chinese Population.	Tang FY et al.	19965945	Not a renal disease focus
1	A novel human heparanase splice variant, T5, endowed with protumorigenic characteristics.	Barash U et al.	20007507	Not a renal disease focus
1	Influence of CYP3A5 and ABCB1 gene polymorphisms on calcineurin inhibitor-related neurotoxicity after hematopoietic stem cell transplantation.	Yanagimachi M et al.	20030680	Not a renal disease focus
1	Development of a multiplex ligation-dependent probe amplification (MLPA) assay for quantification of the OCRL1 gene.	Coutton C et al.	20043897	Not a renal disease focus
1	DNA repair gene X-ray repair cross-complementing group 1 and xeroderma pigmentosum group D polymorphisms and risk of prostate cancer: a study from North India.	Mandal RK et al.	20070155	Not a renal disease focus
1	Renal cell carcinoma Fuhrman grade and histological subtype correlate with complete polymorphic deletion of glutathione S-transferase M1 gene.	De Martino M et al.	20083259	Not a renal disease focus
1	Genetic variations in the sodium balance-regulating genes ENaC, NEDD4L, NDFIP2 and USP2 influence blood pressure and hypertension.	Jin HS et al.	20090362	Not a renal disease focus
1	Multiorgan detection and characterization of protease-resistant prion protein in a case of variant CJD examined in the United States.	Notari S et al.	20098730	Not a renal disease focus
1	Genetic disorders in complement (regulating) genes in patients with atypical haemolytic uraemic syndrome (aHUS).	Westra D et al.	20106822	Not a renal disease focus
1	CYP4F2 gene V433M polymorphism is associated with ischemic stroke in the male Northern Chinese Han population.	Deng S et al.	20227456	Not a renal disease focus
1	Subtype-specific FBXW7 mutation and MYCN copy number gain in Wilms' tumor.	Williams RD et al.	20332316	Not a renal disease focus
1	Association of selected variants in genes involved in cell cycle and apoptosis with bladder cancer risk in North Indian population.	Gangwar R et al.	20380574	Not a renal disease focus
1	Association of CAPN10 gene with insulin sensitivity, glucose tolerance and renal function in essential hypertensive patients.	Zhou X et al.	20406624	Not a renal disease focus

For peer review only

1	Clinical and molecular features of familial and sporadic cases of von Hippel-Lindau disease from Mexico.	Chacon-Camacho OF et al.	20447124	Not a renal disease focus
1	N-glycosylation of carnosinase influences protein secretion and enzyme activity: implications for hyperglycemia.	Riedl E et al.	20460427	Not a renal disease focus
1	Combined classical cytogenetics and microarray-based genomic copy number analysis reveal frequent 3;5 rearrangements in clear cell renal cell carcinoma.	Pei J et al.	20461753	Not a renal disease focus
1	The association of glutathione-S-transferase gene polymorphisms (GSTM1, GSTT1, GSTP1) with idiopathic male infertility.	Safarinejad MR et al.	20505681	Not a renal disease focus
1	The non-muscle Myosin heavy chain 9 gene (MYH9) is not associated with lupus nephritis in African Americans.	Freedman BI et al.	20523037	Not a renal disease focus
1	Association of the genetic polymorphisms of the ACE gene and the eNOS gene with lupus nephropathy in northern Chinese population.	Li X et al.	20540812	Not a renal disease focus
1	eNOS gene polymorphism association with retinopathy in type 1 diabetes.	Bazzaz JT et al.	20565248	Not a renal disease focus
1	Clinical and molecular characterization of Brazilian families with von Hippel-Lindau disease: a need for delineating genotype-phenotype correlation.	Gomy I et al.	20567917	Not a renal disease focus
1	Functional polymorphisms in cell death pathway genes and risk of renal cell carcinoma.	Zhu J et al.	20572163	Not a renal disease focus
1	Role of MMP-3 and MMP-9 and their haplotypes in risk of bladder cancer in North Indian cohort.	Srivastava P et al.	20574775	Not a renal disease focus
1	The role of endothelial nitric oxide synthase (eNOS) T-786C, G894T, and 4a/b gene polymorphisms in the risk of idiopathic male infertility.	Safarinejad MR et al.	20586099	Not a renal disease focus
1	Polymorphic variants of DNA repair gene XRCC3 and XRCC7 and risk of prostate cancer: a study from North Indian population.	Mandal RK et al.	20590474	Not a renal disease focus
1	Association of TNFAIP3 polymorphism with susceptibility to systemic lupus erythematosus in a Japanese population.	Kawasaki A et al.	20617138	Not a renal disease focus
1	Functional polymorphisms in the CYP3A4, CYP3A5, and CYP21A2 genes in the risk for hypertension in pregnancy.	Coto E et al.	20617557	Not a renal disease focus
1	Functional polymorphism of the CK2alpha intronless gene plays oncogenic roles in lung cancer.	Hung MS et al.	20625391	Not a renal disease focus

For peer review only

1	Occupational trichloroethylene exposure and renal carcinoma risk: evidence of genetic susceptibility by reductive metabolism gene variants.	Moore LE et al.	20663906	Not a renal disease focus
1	Influence of caspases 8 and 9 gene promoter polymorphism on prostate cancer susceptibility and early development of hormone refractory prostate cancer.	Kesarwani P et al.	20804486	Not a renal disease focus
1	Are cell cycle and apoptosis genes associated with prostate cancer risk in North Indian population?	Mandal RK et al.	20822933	Not a renal disease focus
1	Bladder cancer risk associated with genotypic polymorphism of the matrix metalloproteinase-1 and 7 in North Indian population.	Srivastava P et al.	20826916	Not a renal disease focus
1	Association of TNFAIP3 interacting protein 1, TNIP1 with systemic lupus erythematosus in a Japanese population: a case-control association study.	Kawasaki A et al.	20849588	Not a renal disease focus
1	S-adenosyl methionine improves early viral responses and interferon-stimulated gene induction in hepatitis C nonresponders.	Feld JJ et al.	20854821	Not a renal disease focus
1	Phosphodiesterase 11A (PDE11A) genetic variants may increase susceptibility to prostatic cancer.	Faucz FR et al.	20881257	Not a renal disease focus
1	A functional variant of the NEDD4L gene is associated with beneficial treatment response with Î²-blockers and diuretics in hypertensive patients.	Svensson-Farbom P et al.	21052022	Not a renal disease focus
1	Characterization of UDP-glucuronosyltransferase 2A1 (UGT2A1) variants and their potential role in tobacco carcinogenesis.	Bushey RT et al.	21164388	Not a renal disease focus
1	hOGG1 Ser326Cys polymorphism and renal cell carcinoma risk in a Chinese population.	Zhao H et al.	21166493	Not a renal disease focus
1	Cancer risks for monoallelic MUTYH mutation carriers with a family history of colorectal cancer.	Win AK et al.	21171015	Not a renal disease focus
1	Isocitrate dehydrogenase 1/2 mutational analyses and 2-hydroxyglutarate measurements in Wilms tumors.	Rakheja D et al.	21225914	Not a renal disease focus
1	A WNK4 gene variant relates to osteoporosis and not to hypertension in the Portuguese population.	Mendes AI et al.	21236712	Not a renal disease focus
1	Three novel mutations in the PHEX gene in Chinese subjects with hypophosphatemic rickets extends genotypic variability.	Jap TS et al.	21293852	Not a renal disease focus

For peer review only

1	Bimodal distribution of RNA expression levels in human skeletal muscle tissue.	Mason CC et al.	21299892	Not a renal disease focus
1	Investigating paraoxonase-1 gene Q192R and L55M polymorphism in patients with renal cell cancer.	Uyar OA et al.	21308654	Not a renal disease focus
1	The presence of PAI-1 4G/5G and ACE DD genotypes increases the risk of early-stage AVF thrombosis in hemodialysis patients.	Gungor Y et al.	21332339	Not a renal disease focus
1	Association of genetic polymorphisms of CYP 2C19 with hypertension in a Chinese Han population.	Ma Y et al.	21332417	Not a renal disease focus
1	Association between renin-angiotensin-aldosterone system-related genes and blood pressure in a Korean population.	Song SB et al.	21342026	Not a renal disease focus
1	Admixture aberration analysis: application to mapping in admixed population using pooled DNA.	Bercovici S et al.	21385031	Not a renal disease focus
1	Polymorphisms and haplotypes in caspases 8 and 9 genes and risk for prostate cancer: a case-control study in cohort of North India.	George GP et al.	21396853	Not a renal disease focus
1	A novel MECA3 region in human 3p21.3 harboring putative tumor suppressor genes and oncogenes.	Braga E et al.	21423093	Not a renal disease focus
1	GSTT1, GSTM1, and CYP1B1 gene polymorphisms and susceptibility to sporadic renal cell cancer.	Salinas-Sanchez AS et al.	21458313	Not a renal disease focus
1	Insertion/deletion polymorphism of angiotensin I-converting enzyme gene is linked with chromophobe renal cell carcinoma.	de Martino M et al.	21477733	Not a renal disease focus
1	Genetic polymorphisms in APE1 are associated with renal cell carcinoma risk in a Chinese population.	Cao Q et al.	21538578	Not a renal disease focus
1	In vitro transforming potential, intracellular signaling properties, and sensitivity to a kinase inhibitor (sorafenib) of RET proto-oncogene variants Glu511Lys, Ser649Leu, and Arg886Trp.	Prazeres H et al.	21551259	Not a renal disease focus
1	Hypercholesterolemia and a candidate gene within the 12q24 locus.	Gragnoli C.	21554682	Not a renal disease focus
1	Lack of association of Klotho gene variants with valvular and vascular calcification in Caucasians: a candidate gene study of the Framingham Offspring Cohort.	Tangri N et al.	21565945	Not a renal disease focus

For peer review only

1	Clear-cell papillary renal cell carcinoma: molecular and immunohistochemical analysis with emphasis on the von Hippel-Lindau gene and hypoxia-inducible factor pathway-related proteins.	Rohan SM et al.	21602815	Not a renal disease focus
1	Selected AGXT gene mutations analysis provides a genetic diagnosis in 28% of Tunisian patients with primary hyperoxaluria.	Benhaj Mbarek I et al.	21612638	Not a renal disease focus
1	Familial renal cell carcinoma from the Swedish Family-Cancer Database.	Liu H et al.	21621909	Not a renal disease focus
1	Adult renal cell carcinoma with rhabdoid morphology represents a neoplastic dedifferentiation analogous to sarcomatoid carcinoma.	Chapman-Fredricks JR et al.	21665507	Not a renal disease focus
1	Association of caspases with an increased prostate cancer risk in north Indian population.	Mittal RD et al.	21668377	Not a renal disease focus
1	Association of death receptor 4, Caspase 3 and 5 gene polymorphism with increased risk to bladder cancer in North Indians.	Mittal RD et al.	21700414	Not a renal disease focus
1	The polymorphisms in the VHL and HIF1A genes are associated with the prognosis but not the development of renal cell carcinoma.	Qin C et al.	21778301	Not a renal disease focus
1	Association of single nucleotide polymorphisms in promoter of matrix metalloproteinase-2, 8 genes with bladder cancer risk in Northern India.	Srivastava P et al.	21784671	Not a renal disease focus
1	Association of PTPN22 gene polymorphism and systemic lupus erythematosus in a cohort of Egyptian patients: impact on clinical and laboratory results.	Moez P et al.	21818561	Not a renal disease focus
1	Thrombotic events in MYH9 gene-related autosomal macrothrombocytopenias (old May-Hegglin, Sebastian, Fechtner and Epstein syndromes).	Girolami A et al.	21842307	Not a renal disease focus
1	JAK3 in clear cell renal cell carcinoma: mutational screening and clinical implications.	de Martino M et al.	21868263	Not a renal disease focus
1	Genetic diagnosis of X-linked dominant Hypophosphatemic Rickets in a cohort study: tubular reabsorption of phosphate and 1,25(OH)2D serum levels are associated with PHEX mutation type.	Morey M et al.	21902834	Not a renal disease focus
1	Gene-gene interaction of BLK, TNFSF4, TRAF1, TNFAIP3, and REL in systemic lupus erythematosus.	Zhou XJ et al.	21905002	Not a renal disease focus
1	Vitamin D receptor FokI and BsmI gene polymorphism and its association with grade and stage of renal cell carcinoma in North Indian population.	Arjumand W et al.	21931993	Not a renal disease focus

For peer review only

1	The polymorphisms of P53 codon 72 and MDM2 SNP309 and renal cell carcinoma risk in a low arsenic exposure area.	Huang CY et al.	21982800	Not a renal disease focus
1	Risk factor for clear cell renal cell carcinoma in Chinese population: a case-control study.	Wang G et al.	22000673	Not a renal disease focus
1	Base excision repair pathway genes polymorphism in prostate and bladder cancer risk in North Indian population.	Mittal RD et al.	22019847	Not a renal disease focus
1	Von Hippel-Lindau (VHL) inactivation in sporadic clear cell renal cancer: associations with germline VHL polymorphisms and etiologic risk factors.	Moore LE et al.	22022277	Not a renal disease focus
1	Impact of glutathione transferase M1, T1, and P1 gene polymorphisms in the genetic susceptibility of North Indian population to renal cell carcinoma.	Ahmad ST et al.	22054067	Not a renal disease focus
1	Whole-exome sequencing of human pancreatic cancers and characterization of genomic instability caused by MLH1 haploinsufficiency and complete deficiency.	Wang L et al.	22156295	Not a renal disease focus
1	Association between urothelial carcinoma after kidney transplantation and aristolochic acid exposure: the potential role of aristolochic acid in HRas and TP53 gene mutations.	Xiao J et al.	22172840	Not a renal disease focus
1	Mapping of the UGT1A locus identifies an uncommon coding variant that affects mRNA expression and protects from bladder cancer.	Tang W et al.	22228101	Not a renal disease focus
1	Paravertebral ligament ossification in vitamin D-resistant rickets: incidence, clinical significance, and genetic evaluation.	Lee SH et al.	22261628	Not a renal disease focus
1	Risk of renal cell carcinoma and polymorphism in phase I xenobiotic metabolizing CYP1A1 and CYP2D6 enzymes.	Ahmad ST et al.	22281432	Not a renal disease focus
1	Relationship between CYP1A1 genetic polymorphisms and renal cancer in China.	Chen J et al.	22296350	Not a renal disease focus
1	Genetic variations in the ADAMTS12 gene are associated with schizophrenia in Puerto Rican patients of Spanish descent.	Bespalova IN et al.	22322903	Not a renal disease focus
1	Association of promoter polymorphisms in MMP2 and TIMP2 with prostate cancer susceptibility in North India.	Srivastava P et al.	22374248	Not a renal disease focus
1	Polymorphisms in base-excision & nucleotide-excision repair genes & prostate cancer risk in north Indian population.	Mandal RK et al.	22382185	Not a renal disease focus
1	Common genetic variants at the 11q13.3 renal cancer susceptibility locus influence binding of HIF to an enhancer of cyclin D1 expression.	Schodel J et al.	22406644	Not a renal disease focus

For peer review only

1	Mutation screening of the EYA1, SIX1, and SIX5 genes in an East Asian cohort with branchio-oto-renal syndrome.	Wang SH et al.	22447252	Not a renal disease focus
1	Association of Ku70 A-31G polymorphism and risk of renal cell carcinoma in a Chinese population.	Wang W et al.	22455395	Not a renal disease focus
1	INPPL1 is associated with the metabolic syndrome in men with Type 1 diabetes, but not with diabetic nephropathy.	Hyvonen ME et al.	22486725	Not a renal disease focus
1	MicroRNA target site polymorphisms in the VHL-HIF1Î± pathway predict renal cell carcinoma risk.	Wei H et al.	22517515	Not a renal disease focus
1	A two-stage matched case-control study on multiple hypertensive candidate genes in Han Chinese.	Kuo TY et al.	22534794	Not a renal disease focus
1	Somatic mutation analysis of the SDHB, SDHC, SDHD, and RET genes in the clinical assessment of sporadic and hereditary pheochromocytoma.	Weber A et al.	22573489	Not a renal disease focus
1	Mutational analysis of patients with FGF23-related hypophosphatemic rickets.	Kinoshita Y et al.	22577109	Not a renal disease focus
1	A functional polymorphism C-1310G in the promoter region of Ku70/XRCC6 is associated with risk of renal cell carcinoma.	Wang W et al.	22593040	Not a renal disease focus
1	Angiotensin-converting enzyme (ACE) gene II genotype protects against the development of diabetic peripheral neuropathy in type 2 diabetes mellitus.	Mansoor Q et al.	22607040	Not a renal disease focus
1	LMP2, a novel immunohistochemical marker to distinguish renal oncocytoma from the eosinophilic variant of chromophobe renal cell carcinoma.	Zheng G et al.	22705098	Not a renal disease focus
1	BRAF mutations in metanephric adenoma of the kidney.	Choueiri T et al.	22727996	Not a renal disease focus
1	Resequencing the whole MYH7 gene (including the intronic, promoter, and 3' UTR sequences) in hypertrophic cardiomyopathy.	Coto E et al.	22765922	Not a renal disease focus
1	Higher frequency of paraoxonase gene polymorphism and cardiovascular impairment among Brazilian Fabry Disease patients.	Barris-Oliveira AC et al.	22796398	Not a renal disease focus
1	Paraoxonase 1 (PON1) C/T-108 association with longitudinal mean arterial blood pressure.	Bhatnagar V et al.	22854640	Not a renal disease focus

For peer review only

1	Patients with Lynch syndrome mismatch repair gene mutations are at higher risk for not only upper tract urothelial cancer but also bladder cancer.	Skeldon SC et al.	22883484	Not a renal disease focus
1	Novel missense mutations of WNK1 in patients with hypokalemic salt-losing tubulopathies.	Zhang C et al.	22934535	Not a renal disease focus
1	Proteasome modulator 9 and depression in type 2 diabetes.	Gragnoli C	22934761	Not a renal disease focus
1	Regulatory regions of the paraoxonase 1 (PON1) gene are associated with neovascular age-related macular degeneration (AMD).	Oczos J et al.	22956172	Not a renal disease focus
1	New insights into Dok-4 PTB domain structure and function.	Hooker E et al.	22982678	Not a renal disease focus
1	The role of XRCC6 T-991C functional polymorphism in renal cell carcinoma.	Chang WS et al.	22993329	Not a renal disease focus
1	Integrated multiplex ligation dependent probe amplification (MLPA) assays for the detection of alterations in the HEXB, GM2A and SMARCAL1 genes to support the diagnosis of Morbus Sandhoff, M. Tay-Sachs variant AB and Schimke immuno-osseous dysplasia in humans.	Sobek AK et al.	23010210	Not a renal disease focus
1	Endothelial nitric oxide synthase Glu298Asp polymorphism as a risk factor for prostate cancer.	Ziaei SA et al.	23015399	Not a renal disease focus
1	Calpain-10 gene polymorphisms in type 2 diabetes and its micro- and macrovascular complications.	Buraczynska M et al.	23021796	Not a renal disease focus
1	Genetic variants in metabolizing genes NQO1, NQO2, MTHFR and risk of prostate cancer: a study from North India.	Mandal RK et al.	23054000	Not a renal disease focus
1	Soluble FLT1 binds lipid microdomains in podocytes to control cell morphology and glomerular barrier function.	Jin J et al.	23063127	Not a renal disease focus
1	Polymorphism in protein tyrosine phosphatase receptor delta is associated with the risk of clear cell renal cell carcinoma.	Du Y et al.	23069849	Not a renal disease focus
1	Impact of glutathione S-transferase T1 gene polymorphisms on acute cellular rejection in living donor liver transplantation.	Kamei H et al.	23153768	Not a renal disease focus
1	Interaction of C1GALT1-IL5RA on the susceptibility to IgA nephropathy in Southern Han Chinese.	Wang W et al.	23190752	Not a renal disease focus
1	A functional variant in the MTOR promoter modulates its expression and is associated with renal cell cancer risk.	Cao Q et al.	23209702	Not a renal disease focus

For peer review only

1	Association of the formiminotransferase N-terminal sub-domain containing gene and thrombospondin, type 1, domain-containing 7A gene with the prevalence of vertebral fracture in 2427 consecutive autopsy cases.	Zhou H et al.	23303384	Not a renal disease focus
1	Phenotypic spectrum and prevalence of INPP5E mutations in Joubert syndrome and related disorders.	Travaglini L et al.	23386033	Not a renal disease focus
1	CYP24A1 and CYP27B1 polymorphisms modulate vitamin D metabolism in colon cancer cells.	Jacobs ET et al.	23423976	Not a renal disease focus
1	Tumor-specific isoform switch of the fibroblast growth factor receptor 2 underlies the mesenchymal and malignant phenotypes of clear cell renal cell carcinomas.	Zhao Q et al.	23444225	Not a renal disease focus
1	Distribution of human leukocyte antigen alleles in systemic lupus erythematosus patients with angiotensin converting enzyme insertion/deletion polymorphism.	Hussain N et al.	23448612	Not a renal disease focus
1	Genetic predisposition to left ventricular hypertrophy and the potential involvement of cystatin-C in untreated hypertension.	Tousoulis D et al.	23479071	Not a renal disease focus
1	Epistatic role of the MYH9/APOL1 region on familial hematuria genes.	Voskarides K et al.	23516419	Not a renal disease focus
1	Pre-treatment role of inosine triphosphate pyrophosphatase polymorphism for predicting anemia in Egyptian hepatitis C virus patients.	Ahmed WH et al.	23538996	Not a renal disease focus
1	Metanephric adenoma and solid variant of papillary renal cell carcinoma: common and distinctive features.	Mantoan Padilha M et al.	23551615	Not a renal disease focus
1	Fluid intake, genetic variants of UDP-glucuronosyltransferases, and bladder cancer risk.	Wang J et al.	23632476	Not a renal disease focus
1	Influence of survivin (BIRC5) and caspase-9 (CASP9) functional polymorphisms in renal cell carcinoma development: a study in a southern European population.	Marques I et al.	23645041	Not a renal disease focus
1	Germline BAP1 mutations predispose to renal cell carcinomas.	Popova T et al.	23684012	Not a renal disease focus
1	Association of B2 receptor polymorphisms and ACE activity with ACE inhibitor-induced angioedema in black and mixed-race South Africans.	Moholisa RR et al.	23730990	Not a renal disease focus

For peer review only

1	Klotho gene polymorphism of rs3752472 is associated with the risk of urinary calculi in the population of Han nationality in Eastern China.	Xu C et al.	23756195	Not a renal disease focus
1	Oncocytic papillary renal cell carcinoma: a clinicopathological study emphasizing distinct morphology, extended immunohistochemical profile and cytogenetic features.	Xia QY et al.	23826421	Not a renal disease focus
1	Impact of MMP-3 and TIMP-3 gene polymorphisms on prostate cancer susceptibility in North Indian cohort.	Srivastava P et al.	23872201	Not a renal disease focus
1	Clinical phenotypes of Chinese primary hyperparathyroidism patients are associated with the calcium-sensing receptor gene R990G polymorphism.	Han G et al.	23946278	Not a renal disease focus
1	Q222R polymorphism in DNAse I gene is a risk factor for nephritis in South Indian SLE patients.	Panneer D et al.	23963431	Not a renal disease focus
1	cGMP-dependent protein kinase 1 polymorphisms underlie renal sodium handling impairment.	Citterio L et al.	24060892	Not a renal disease focus
1	Cancer risk and overall survival in mismatch repair proficient hereditary non-polyposis colorectal cancer, Lynch syndrome and sporadic colorectal cancer.	Garre P et al.	24061861	Not a renal disease focus
1	Clinical and Genetic Factors Associated With Severe Hematological Toxicity in Glioblastoma Patients During Radiation Plus Temozolomide Treatment: A Prospective Study.	Lombardi G et al.	24064758	Not a renal disease focus
1	Mutations in the mevalonate kinase (MVK) gene cause nonsyndromic retinitis pigmentosa.	Siemiatkowska AM et al.	24084495	Not a renal disease focus
1	Three novel mutations in the carnitine-acylcarnitine translocase (CACT) gene in patients with CACT deficiency and in healthy individuals.	Fukushima T et al.	24088670	Not a renal disease focus
1	Delineation of PIGV mutation spectrum and associated phenotypes in hyperphosphatasia with mental retardation syndrome.	Horn D et al.	24129430	Not a renal disease focus
1	Genetic variation in the GSTM3 promoter confer risk and prognosis of renal cell carcinoma by reducing gene expression.	Tan X et al.	24157827	Not a renal disease focus
1	Early (2008-2010) hospital outbreak of Klebsiella pneumoniae producing OXA-48 carbapenemase in the UK.	Thomas CP et al.	24207018	Not a renal disease focus
1	RTK/ERK pathway under natural selection associated with prostate cancer.	Chen Y et al.	24223781	Not a renal disease focus

For peer review only

1	Amylase Î±-1A (AMY1A): a novel immunohistochemical marker to differentiate chromophobe renal cell carcinoma from benign oncocytoma.	Jain S et al.	24225843	Not a renal disease focus
1	Clear cell papillary renal cell carcinoma: a clinicopathological study emphasizing ultrastructural features and cytogenetic heterogeneity.	Shi SS et al.	24294381	Not a renal disease focus
1	The epidemic of extended-spectrum-Î²-lactamase-producing Escherichia coli ST131 is driven by a single highly pathogenic subclone, H30-Rx.	Price LB et al.	24345742	Not a renal disease focus
1	Mutations in the UQCC1-interacting protein, UQCC2, cause human complex III deficiency associated with perturbed cytochrome b protein expression.	Tucker EJ et al.	24385928	Not a renal disease focus
1	The association of endothelial nitric oxide synthase gene G894T polymorphism and serum nitric oxide concentration with microalbuminuria in patients with gestational diabetes.	Atay AE et al.	24403014	Not a renal disease focus
1	Association of candidate genetic variants with restless legs syndrome in end stage renal disease: a multicenter case-control study in Taiwan.	Lin CH et al.	24433515	Not a renal disease focus
1	VHL and HIF-1Î±: gene variations and prognosis in early-stage clear cell renal cell carcinoma.	Lessi F et al.	24446253	Not a renal disease focus
1	Genomic architecture and evolution of clear cell renal cell carcinomas defined by multiregion sequencing.	Gerlinger M et al.	24487277	Not a renal disease focus
1	IFT27, encoding a small GTPase component of IFT particles, is mutated in a consanguineous family with Bardet-Biedl syndrome.	Aldahmesh MA et al.	24488770	Not a renal disease focus
1	TNFAIP3 gene polymorphisms associated with differential susceptibility to rheumatoid arthritis and systemic lupus erythematosus in the Korean population.	Kim SK et al.	24489017	Not a renal disease focus
1	HER2 protein overexpression and gene amplification in upper urinary tract urothelial carcinoma-an analysis of 171 patients.	Sasaki Y et al.	24551292	Not a renal disease focus
1	Genotype-phenotype correlations, and retinal function and structure in von Hippel-Lindau disease.	Wittstrom E et al.	24555745	Not a renal disease focus
1	Higher thrombin activatable fibrinolysis inhibitor levels are associated with inflammation in attack-free familial Mediterranean fever patients.	Bavbek N et al.	24580410	Not a renal disease focus
1	Contribution of CDKAL1 rs7756992 and IGF2BP2 rs4402960 polymorphisms in type 2 diabetes, diabetic complications, obesity risk and hypertension in the Tunisian population.	Lasram K et al.	24636221	Not a renal disease focus

For peer review only

1	Testing of potential glycan-based heparanase inhibitors in a fluorescence activity assay using either bacterial heparinase II or human heparanase.	Schoenfeld AK et al.	24667567	Not a renal disease focus
1	Impact of the common genetic associations of age-related macular degeneration upon systemic complement component C3d levels.	Ristau T et al.	24675670	Not a renal disease focus
1	Lipoprotein (a) concentrations, apolipoprotein (a) phenotypes, and peripheral arterial disease in three independent cohorts.	Laschkolnig A et al.	24760552	Not a renal disease focus
1	TMPRSS6 rs855791 polymorphism influences the susceptibility to iron deficiency anemia in women at reproductive age.	Pei SN et al.	24782651	Not a renal disease focus
1	Manganese superoxide dismutase (SOD2) polymorphisms, plasma advanced oxidation protein products (AOPP) concentration and risk of kidney complications in subjects with type 1 diabetes.	Mohammedi K et al.	24819633	Not a renal disease focus
1	A labor- and cost-effective non-optical semiconductor (Ion Torrent) next-generation sequencing of the SLC12A3 and CLCNKA/B genes in Gitelman's syndrome patients.	Tavira B et al.	24830959	Not a renal disease focus
1	Genetic polymorphisms of paraoxonase1 192 and glutathione peroxidase1 197 enzymes in familial Mediterranean fever.	Oktem F et al.	24841661	Not a renal disease focus
1	Common variants of cGKII/PRKG2 are not associated with gout susceptibility.	Sakiyama M et al.	24882840	Not a renal disease focus
1	Bobby Sox homology regulates odontoblast differentiation of human dental pulp stem cells/progenitors.	Choi YA et al.	24885382	Not a renal disease focus
1	A complement factor B mutation in a large kindred with atypical hemolytic uremic syndrome.	Funato M et al.	24906628	Not a renal disease focus
1	Recurrent somatic mutation in DROSHA induces microRNA profile changes in Wilms tumour.	Torrezan GT et al.	24909261	Not a renal disease focus
1	Breast cancer risk, nightwork, and circadian clock gene polymorphisms.	Truong T et al.	24919398	Not a renal disease focus
1	The role of endothelial nitric oxide synthase gene G894T and intron 4 VNTR polymorphisms in hemodialysis patients with vascular access thrombosis.	Sener EF et al.	24936541	Not a renal disease focus
1	The consensus-based approach for gene/enzyme replacement therapies and crystallization strategies: the case of human alanine-glyoxylate aminotransferase.	Mesa-Torres N et al.	24957194	Not a renal disease focus

For peer review only

1	Polymorphisms in genes of the renin-angiotensin-aldosterone system and renal cell cancer risk: interplay with hypertension and intakes of sodium, potassium and fluid.	Deckers IA et al.	24978482	Not a renal disease focus
1	The effect of a single nucleotide polymorphism of the CYP4F2 gene on blood pressure and 20-hydroxyeicosatetraenoic acid excretion after weight loss.	Ward NC et al.	24984178	Not a renal disease focus
1	Cytochrome P450 1B1 polymorphisms and risk of renal cell carcinoma in men.	Chang I et al.	25027399	Not a renal disease focus
1	Single nucleotide polymorphism-single nucleotide polymorphism interactions among inflammation genes in the genetic architecture of blood pressure in the Framingham Heart Study.	Basson JJ et al.	25063733	Not a renal disease focus
1	Germline BAP1 mutations predispose also to multiple basal cell carcinomas.	de la Fouchardiere A et al.	25080371	Not a renal disease focus
1	Fluorescence in situ hybridization of chromosomeÂ 17 polysomy in breast cancer using thin tissue sections causes the loss of CEP17 and HER2 signals.	Jiang H et al.	25119636	Not a renal disease focus
1	Single-nucleotide polymorphisms in the UDP-glucuronosyltransferase 1A-3' untranslated region are associated with atazanavir-induced nephrolithiasis in patients with HIV-1 infection: a pharmacogenetic study.	Nishijima T et al.	25151207	Not a renal disease focus
1	A low-frequency variant in MAPK14 provides mechanistic evidence of a link with myeloperoxidase: a prognostic cardiovascular risk marker.	Waterworth DM et al.	25164947	Not a renal disease focus
1	Identification of genetic markers for treatment success in heart failure patients: insight from cardiac resynchronization therapy.	Schmitz B et al.	25210049	Not a renal disease focus
1	Two single nucleotide polymorphisms in the von Hippel-Lindau tumor suppressor gene in Taiwanese with renal cell carcinoma.	Wang WC et al.	25217002	Not a renal disease focus
1	Replicative study of GWAS TP63C/T, TERTC/T, and SLC14A1C/T with susceptibility to bladder cancer in North Indians.	Singh V et al.	25218484	Not a renal disease focus
1	Molecular epidemiology of extended-spectrum beta-lactamase (ESBL)-positive Klebsiella pneumoniae from bloodstream infections and risk factors for mortality.	Gurntke S et al.	25224765	Not a renal disease focus
1	Pharmacologic rescue of an enzyme-trafficking defect in primary hyperoxaluria 1.	Miyata N et al.	25237136	Not a renal disease focus

For peer review only

1	Variants in angiogenesis-related genes and the risk of clear cell renal cell carcinoma.	Qin C et al.	25239121	Not a renal disease focus
1	PYGM expression analysis in white blood cells: a complementary tool for diagnosing McArdle disease?	de Luna N et al.	25240406	Not a renal disease focus
1	LRRK2 G2385R and R1628P mutations are associated with an increased risk of Parkinson's disease in the Malaysian population.	Gopalai AA et al.	25243190	Not a renal disease focus
1	Gender differences in impact of CYP2C19 polymorphism on development of coronary artery disease.	Hokimoto S et al.	25264752	Not a renal disease focus
1	Genotype and phenotype frequencies of paraoxonase 1 in fertile and infertile men.	Tavilani H et al.	25264968	Not a renal disease focus
1	Phase 1 trial of tivantinib in combination with sorafenib in adult patients with advanced solid tumors.	Puzanov I et al.	25294187	Not a renal disease focus
1	G-protein receptor kinase 4 polymorphism and response to antihypertensive therapy.	Muskalla AM et al.	25301854	Not a renal disease focus
1	Downregulation of NDUFB6 due to 9p24.1-p13.3 loss is implicated in metastatic clear cell renal cell carcinoma.	Narimatsu T et al.	25315157	Not a renal disease focus
1	Association of polymorphisms of angiotensin I converting enzyme 2 with retinopathy in type 2 diabetes mellitus among Chinese individuals	Meng N et al.	25359286	Not a renal disease focus
1	Matrix metalloproteinase 9 polymorphisms and systemic lupus erythematosus: correlation with systemic inflammatory markers and oxidative stress.	Bahrehmand F et al.	25416694	Not a renal disease focus
1	Rhabdoid tumor predisposition syndrome.	Sredni ST et al.	25494491	Not a renal disease focus
1	Autosomal recessive lissencephaly with cerebellar hypoplasia is associated with a loss-of-function mutation in CDK5.	Magen D et al.	25560765	Not a renal disease focus
1	Exome-wide Sequencing Shows Low Mutation Rates and Identifies Novel Mutated Genes in Seminomas.	Cutcutache I et al.	25597018	Not a renal disease focus
1	Molecular and immunohistochemical characterization reveals novel BRAF mutations in metanephric adenoma.	Udager AM et al.	25602792	Not a renal disease focus
1	Genome-wide association study of clinically defined gout identifies multiple risk loci and its association with clinical subtypes.	Matsuo H et al.	25646370	Not a renal disease focus

For peer review only

1	The role of hOGG1 C1245G polymorphism in the susceptibility to lupus nephritis and modulation of the plasma 8-OHdG in patients with systemic lupus erythematosus.	Lee HT et al.	25671815	Not a renal disease focus
1	Endothelial nitric oxide synthase gene intron 4 variable number tandem repeat polymorphism in Î²-thalassemia major: relation to cardiovascular complications.	Tantawy AA et al.	25699607	Not a renal disease focus
1	Genetic variants in five novel loci including CFB and CD40 predispose to chronic hepatitis B.	Jiang DK et al.	25802187	Not a renal disease focus
1	SNP Variants in RET and PAX2 and Their Possible Contribution to the Primary Hyperoxaluria Type 1 Phenotype.	Coulter-Mackie MB	25854853	Not a renal disease focus
1	Polymorphic differences in the SOD-2 gene may affect the pathogenesis of nephropathy in patients with diabetes and diabetic complications.	Houldsworth A et al.	25858271	Not a renal disease focus
1	Retrospective analysis of FFPE based Wilms' Tumor samples through copy number and somatic mutation related Molecular Inversion Probe Based Array.	Singh N et al.	25913740	Not a renal disease focus
1	Genotype-phenotype analysis of von Hippel-Lindau syndrome in fifteen Indian families.	Vikkath N et al.	25952756	Not a renal disease focus
1	Angiotensin-converting enzyme (ACE) gene insertion/deletion polymorphism is not a risk factor for hypertension in SLE nephritis.	Negi VS et al.	25957879	Not a renal disease focus
1	A novel phenotype in N-glycosylation disorders: Gillessen-Kaesbach-Nishimura skeletal dysplasia due to pathogenic variants in ALG9.	Tham E et al.	25966638	Not a renal disease focus
1	Loss of BAP1 Expression in Basal Cell Carcinomas in Patients With Germline BAP1 Mutations.	Mochel MC et al.	25972334	Not a renal disease focus
1	Spectrum of mutations in the ATP binding domain of ATP7B gene of Wilson Disease in a regional Indian cohort.	Guggilla SR et al.	25982861	Not a renal disease focus
1	Prevalence of Mycobacterium avium subsp. paratuberculosis and Escherichia coli in blood samples from patients with inflammatory bowel disease.	Nazareth N et al.	25994082	Not a renal disease focus
1	Matrix metalloproteinase-2 (MMP-2) gene polymorphism and cardiovascular comorbidity in type 2 diabetes patients.	Buraczynska M et al.	26025700	Not a renal disease focus
1	Genetic Variants in Caveolin-1 and RhoA/ROCK1 Are Associated with Clear Cell Renal Cell Carcinoma Risk in a Chinese Population.	Zhao R et al.	26066055	Not a renal disease focus

For peer review only

1	Genetic and Functional Analysis of Polymorphisms in the Human Dopamine Receptor and Transporter Genes in Small Cell Lung Cancer.	Cherubini E et al.	26081799	Not a renal disease focus
1	Exploring genotype-phenotype relationships in Bardet-Biedl syndrome families.	Castro-Sanchez S et al.	26082521	Not a renal disease focus
1	Whole Exome Sequencing Reveals Novel PHEX Splice Site Mutations in Patients with Hypophosphatemic Rickets.	Ma SL et al.	26107949	Not a renal disease focus
1	Exome sequencing in seven families and gene-based association studies indicate genetic heterogeneity and suggest possible candidates for fibromuscular dysplasia.	Kiando SR et al.	26147384	Not a renal disease focus
1	TERT Promoter Mutations in Papillary Thyroid Microcarcinomas.	de Biase D et al.	26148423	Not a renal disease focus
1	A genome-wide screening and SNPs-to-genes approach to identify novel genetic risk factors associated with frontotemporal dementia.	Ferrari R et al.	26154020	Not a renal disease focus
1	GSTM1, GSTT1 and GSTP1 Genetic Variants in Multiple Urologic Cancers.	Chirila DN et al.	26158735	Not a renal disease focus
1	Renal cell carcinoma risk is associated with the interactions of APOE, VHL and MTHFR gene polymorphisms.	Lv C et al.	26191297	Not a renal disease focus
1	Financial incentives for kidney donation: A comparative case study using synthetic controls.	Bilgel F et al.	26218985	Not a renal disease focus
1	Novel mutations of the ATP7B gene in Han Chinese families with pre-symptomatic Wilson's disease.	Yuan ZF et al.	26253413	Not a renal disease focus
1	Lack of genetic association of the TGM2 gene with schizophrenia in a Chinese population.	Wang J et al.	26307914	Not a renal disease focus
1	Genetic Polymorphism of MMP2 Gene and Susceptibility to Prostate Cancer.	Adabi Z et al.	26319608	Not a renal disease focus
1	A method for predicting target drug efficiency in cancer based on the analysis of signaling pathway activation.	Artemov A et al.	26320181	Not a renal disease focus
1	Effect of a functional polymorphism in the pre-miR-146a gene on the risk and prognosis of renal cell carcinoma.	Huang Z et al.	26323945	Not a renal disease focus
1	The sirtuin inhibitor sirtinol inhibits hepatitis A virus (HAV) replication by inhibiting HAV internal ribosomal entry site activity.	Kanda T et al.	26388050	Not a renal disease focus

For peer review only

1	Molecular characterization of multidrug-resistant Klebsiella pneumoniae isolates.	Hou XH et al.	26413058	Not a renal disease focus
1	Variants in Vitamin D Binding Protein Gene Are Associated With Gestational Diabetes Mellitus.	Wang Y et al.	26448018	Not a renal disease focus
1	Role of PTPN22 and CSK gene polymorphisms as predictors of susceptibility and clinical heterogeneity in patients with Henoch-SchÃ¶nlein purpura (IgA vasculitis).	Lopez-Mejias R et al.	26458874	Not a renal disease focus
1	Association of polymorphisms in PRKCI gene and risk of prostate cancer in a sample of Iranian Population.	Hashemi M et al.	26475383	Not a renal disease focus
1	Analysis of Multiple Families With Single Individuals Affected by Pseudohypoparathyroidism Type Ib (PHP1B) Reveals Only One Novel Maternally Inherited GNAS Deletion.	Takatani R et al.	26479409	Not a renal disease focus
1	Constitutional de novo deletion of the FBXW7 gene in a patient with focal segmental glomerulosclerosis and multiple primitive tumors.	Roversi G et al.	26482194	Not a renal disease focus
1	MKS1 regulates ciliary INPP5E levels in Joubert syndrome.	Slaats GG et al.	26490104	Not a renal disease focus
1	Association of Angiotensin Converting Enzyme Insertion-Deletion Polymorphism with Hypertension in Emiratis with Type 2 Diabetes Mellitus and Its Interaction with Obesity Status.	Alsafar H et al.	26491214	Not a renal disease focus
1	Association of chitotriosidase enzyme activity and genotype with the risk of nephropathy in type 2 diabetes.	Elmonem MA et al.	26589000	Not a renal disease focus
1	Use of a High-Density Protein Microarray to Identify Autoantibodies in Subjects with Type 2 Diabetes Mellitus and an HLA Background Associated with Reduced Insulin Secretion.	Chang DC et al.	26606528	Not a renal disease focus
1	Analysis of urinary cathepsin C for diagnosing Papillon-LefÃ¨vre syndrome.	Hamon Y et al.	26607765	Not a renal disease focus
1	Genetic alteration in notch pathway is associated with better prognosis in renal cell carcinoma.	Feng C et al.	26662507	Not a renal disease focus
1	Genotype-guided tacrolimus dosing in African-American kidney transplant recipients.	Sanghavi K et al.	26667830	Not a renal disease focus
1	Hepatocystin is Essential for TRPM7 Function During Early Embryogenesis.	Overton JD et al.	26671672	Not a renal disease focus

For peer review only

1	XRCC3 Thr241Met and XPD Lys751Gln gene polymorphisms and risk of clear cell renal cell carcinoma.	Loghin A et al.	26682510	Not a renal disease focus
1	Germline BAP1 Mutational Landscape of Asbestos-Exposed Malignant Mesothelioma Patients with Family History of Cancer.	Ohar JA et al.	26719535	Not a renal disease focus
1	HABP2 G534E Variant in Papillary Thyroid Carcinoma.	Tomsic J et al.	26745718	Not a renal disease focus
1	Glutathione peroxidase-1 gene (GPX1) variants, oxidative stress and risk of kidney complications in people with type 1 diabetes.	Mohammedi K et al.	26773925	Not a renal disease focus
1	Serum paraoxonase-1 gene polymorphism and enzyme activity in patients with urolithiasis.	Atar A et al.	26795139	Not a renal disease focus
1	Association of ACE gene D polymorphism with left ventricular hypertrophy in patients with diastolic heart failure: a case-control study.	Bahramali E et al.	26861937	Not a renal disease focus
1	Association of the BsmI, ApaI, TaqI, Tru9I and FokI Polymorphisms of the Vitamin D Receptor Gene with Nephrolithiasis in the Turkish Population.	Cakir OO et al.	26945655	Not a renal disease focus
1	System-Wide Modulation of HECT E3 Ligases with Selective Ubiquitin Variant Probes.	Zhang W et al.	26949039	Not a renal disease focus
1	Spondyloenchondrodysplasia Due to Mutations in ACP5: A Comprehensive Survey.	Briggs TA et al.	26951490	Not a renal disease focus
1	Novel NEK8 Mutations Cause Severe Syndromic Renal Cystic Dysplasia through YAP Dysregulation.	Grampa V et al.	26967905	Not a renal disease focus
1	Angiotensin-converting enzyme gene I/D polymorphism increases the susceptibility to hypertension and additive diseases: A study on North Indian patients.	Singh M et al.	27030424	Not a renal disease focus
1	Cystinosin deficiency causes podocyte damage and loss associated with increased cell motility.	Ivanova EA et al.	27083281	Not a renal disease focus
1	The VHL short variant involves in protein quality control.	Liu Y et al.	27196060	Not a renal disease focus
1	Vascular endothelial growth factor polymorphism (-460 T/C) is related to hypertension-associated chronic kidney disease.	Malkiewicz A et al.	27367286	Not a renal disease focus
1	The association between fructosamine-3 kinase 900C/G polymorphism, transferrin polymorphism and human herpesvirus-8 infection in diabetics living in South Kivu.	Cikomola JC et al.	27461879	Not a renal disease focus

For peer review only

1	Pro198Leu Polymorphism in the Glutathione Peroxidase 1 Gene Contributes to Diabetic Peripheral Neuropathy in Type 2 Diabetes Patients.	Buraczynska M et al.	27592002	Not a renal disease focus
1	ERBB4 gene polymorphisms and the risk of prostate cancer in a sample of Iranian Population.	Hashemi M et al.	27609473	Not a renal disease focus
1	VEGF-A and VEGFR1 SNPs associate with preeclampsia in a Philippine population.	Amosco MD et al.	27668980	Not a renal disease focus
1	Assessment of Human Tribbles Homolog 3 Genetic Variation (rs2295490) Effects on Type 2 Diabetes Patients with Glucose Control and Blood Pressure Lowering Treatment.	He F et al.	27793583	Not a renal disease focus
1	TET2 binds the androgen receptor and loss is associated with prostate cancer.	Nickerson ML et al.	27819678	Not a renal disease focus
1	PCSK9 genetic variants and risk of type 2 diabetes: a mendelian randomisation study.	Schmidt AF et al.	27908689	Not a renal disease focus
1	Hypertension is a characteristic complication of X-linked hypophosphatemia.	Nakamura Y et al.	28025445	Not a renal disease focus
1	URAT1 inhibition by ALPK1 is associated with uric acid homeostasis.	Kuo TM et al.	28039413	Not a renal disease focus
1	Autophagy-related gene LRRK2 is likely a susceptibility gene for systemic lupus erythematosus in northern Han Chinese.	Zhang YM et al.	28099919	Not a renal disease focus
1	Association Study of Klotho Gene Polymorphism With Calcium Oxalate Stones in The Uyghur Population of Xinjiang, China.	Ali A et al.	28116736	Not a renal disease focus
1	Rare and low-frequency coding variants alter human adult height.	Marouli E et al.	28146470	Not a renal disease focus
1	An association between overexpression of DNA methyltransferase 3B4 and clear cell renal cell carcinoma.	Liu Y et al.	28160561	Not a renal disease focus
1	BARD1 Gene Polymorphisms Confer Nephroblastoma Susceptibility.	Fu W et al.	28161399	Not a renal disease focus
1	Genetic susceptibility variants for lung cancer: replication study and assessment as expression quantitative trait loci.	Pintarelli G et al.	28181565	Not a renal disease focus
1	Relationship between salivary/pancreatic amylase and body mass index: a systems biology approach.	Bonnefond A et al.	28228143	Not a renal disease focus

For peer review only

1	Development of hypomelanotic macules is associated with constitutive activated mTORC1 in tuberous sclerosis complex.	Moller LB et al.	28336152	Not a renal disease focus
1	A Low-Frequency Inactivating AKT2 Variant Enriched in the Finnish Population Is Associated With Fasting Insulin Levels and Type 2 Diabetes Risk.	Manning A et al.	28341696	Not a renal disease focus
1	Cost-effectiveness Analysis for Genotyping before Allopurinol Treatment to Prevent Severe Cutaneous Adverse Drug Reactions.	Ke CH et al.	28365572	Not a renal disease focus
1	Hepatitis C viral load, genotype, and increased risk of developing end-stage renal disease: REVEAL-HCV study.	Lai TS et al.	28370058	Not a renal disease focus
1	Isolated polycystic liver disease genes define effectors of polycystin-1 function.	Besse W et al.	28375157	Not a renal disease focus
1	Role of MMP-7 in the pathogenesis of systemic lupus erythematosus (SLE).	Vira H et al.	28420044	Not a renal disease focus
1	Frequent somatic mutations in epigenetic regulators in newly diagnosed chronic myeloid leukemia.	Togasaki E et al.	28452984	Not a renal disease focus
1	Ocular Pathology of Oculocerebrorenal Syndrome of Lowe: Novel Mutations and Genotype-Phenotype Analysis.	Song E et al.	28473699	Not a renal disease focus
1	A novel, de novo mutation in the PRKAG2 gene: infantile-onset phenotype and the signaling pathway involved.	Xu Y et al.	28550180	Not a renal disease focus
1	Genome-wide association study of erythrocyte density in sickle cell disease patients.	Ilboudo Y et al.	28552477	Not a renal disease focus
1	Functional polymorphism at the miR-502-binding site in the 3' untranslated region of the SETD8 gene increased the risk of prostate cancer in a sample of Iranian population.	Narouie B et al.	28578017	Not a renal disease focus
1	Acute lymphoblastic leukemia and genetic variations in BHMT gene: Case-control study and computational characterization.	Bellampalli R et al.	28582843	Not a renal disease focus
1	Unusual clinical outcome of primary Hyperoxaluria type 1 in Tunisian patients carrying 33_34InsC mutation.	Mbarek IB et al.	28619084	Not a renal disease focus
1	Disease-linked mutations in factor H reveal pivotal role of cofactor activity in self-surface-selective regulation of complement activation.	Kerr H et al.	28637873	Not a renal disease focus
1	Glomerular Hyperfiltration in Obese African American Hypertensive Patients Is Associated With Elevated Urinary Mitochondrial-DNA Copy Number.	Eirin A et al.	28641368	Not a renal disease focus

For peer review only

1	Expression and secretion of the Giardia duodenalis variant surface protein 9B10A by transfected trophozoites causes damage to epithelial cell monolayers mediated by protease activity.	Cabrera-Licona A et al.	28668253	Not a renal disease focus
1	Identification of sequence polymorphisms in the mitochondrial cytochrome c oxidase genes as risk factors for hepatocellular carcinoma.	Wang H et al.	28703354	Not a renal disease focus
1	Genome-wide association study identifies inversion in the CTRB1-CTRB2 locus to modify risk for alcoholic and non-alcoholic chronic pancreatitis.	Rosendahl J et al.	28754779	Not a renal disease focus
1	A role of the endothelial nitric oxide system in acute renal colic caused by ureteral stone.	Bulbul E et al.	28802544	Not a renal disease focus
1	An apical MRCK-driven morphogenetic pathway controls epithelial polarity.	Zihni C et al.	28825699	Not a renal disease focus
1	Frequent Genetic Aberrations in the CDK4 Pathway in Acral Melanoma Indicate the Potential for CDK4/6 Inhibitors in Targeted Therapy.	Kong Y et al.	28830923	Not a renal disease focus
1	Association between interferon-induced helicase (IFIH1) rs1990760 polymorphism and seasonal variation in the onset of type 1 diabetes mellitus.	Jermendy A et al.	28929635	Not a renal disease focus
1	AGXT2 rs37369 polymorphism predicts the renal function in patients with chronic heart failure.	Hu XL et al.	28942034	Not a renal disease focus
1	The Endothelial Nitric Oxide Synthase Gene Polymorphism is Associated with the Susceptibility to Immunoglobulin a Nephropathy in Chinese Population.	Gao J et al.	28946141	Not a renal disease focus
1	APOL1, CDKN2A/CDKN2B, and HDAC9 polymorphisms and small vessel ischemic stroke.	Akinyemi R et al.	28975602	Not a renal disease focus
1	Polymorphisms of DNA repair genes XRCC1 and LIG4 and idiopathic male infertility.	Ghasemi H et al.	28991497	Not a renal disease focus
1	Alcohol consumption, variability in alcohol dehydrogenase genes and risk of renal cell carcinoma.	Antwi SO et al.	29023769	Not a renal disease focus
1	Rare ADAR and RNASEH2B variants and a type I interferon signature in glioma and prostate carcinoma risk and tumorigenesis.	Beyer U et al.	29030706	Not a renal disease focus
1	Familial Mediterranean fever mutations are hypermorphic mutations that specifically decrease the activation threshold of the Pyrin inflammasome.	Jamilloux Y et al.	29040788	Not a renal disease focus

For peer review only

1	Circulating Endothelial Markers in Retinal Vasculopathy With Cerebral Leukoencephalopathy and Systemic Manifestations.	Pelzer N et al.	29114091	Not a renal disease focus
1	Relationship between rs854560 PON1 Gene Polymorphism and Tobacco Smoking with Coronary Artery Disease.	Iwanicka J et al.	29118461	Not a renal disease focus
1	Polymorphism of ERCC1 rs3212986 in Chinese Han women with preeclampsia.	Liu MC et al.	29153678	Not a renal disease focus
1	Analysis of the influence of the T393C polymorphism of the GNAS gene on the clinical expression of primary hyperparathyroidism.	Piedra M et al.	29179855	Not a renal disease focus
1	Association Between Klotho Gene Polymorphism and Markers of Bone Metabolism in Patients Receiving Maintenance Hemodialysis in Iran.	Nazarian A et al.	29190606	Not a renal disease focus
1	Association Between HACE1 Gene Polymorphisms and Wilms' Tumor Risk in a Chinese Population.	Jia W et al.	29243987	Not a renal disease focus
1	PDE1A polymorphism contributes to the susceptibility of nephrolithiasis.	Yang Z et al.	29262781	Not a renal disease focus
1	Genetic screening and functional analysis of CASP9 mutations in a Chinese cohort with neural tube defects.	Liu XZ et al.	29365368	Not a renal disease focus
1	The relationship between ACE/AGT gene polymorphisms and the risk of diabetic retinopathy in Chinese patients with type 2 diabetes.	Qiao YC et al.	29378484	Not a renal disease focus
1	HIPK2 polymorphisms rs2058265, rs6464214, and rs7456421 were associated with kidney stone disease in Chinese males not females.	Lin H et al.	29428801	Not a renal disease focus
1	The association of endothelial nitric oxide synthase gene single nucleotide polymorphisms with paediatric systemic lupus erythematosus.	Zhu J et al.	29465350	Not a renal disease focus
1	Common Variants in ALPL Gene Contribute to the Risk of Kidney Stones in the Han Chinese Population.	Li X et al.	29489416	Not a renal disease focus
1	Impact of eNOS 27-bp VNTR (4b/a) gene polymorphism with the risk of Systemic Lupus Erythematosus in south Indian subjects.	Katkam SK et al.	29524578	Not a renal disease focus
1	Rationale, design, and preliminary results of the Quebec Warfarin Cohort Study.	Perreault S et al.	29542828	Not a renal disease focus
1	The evaluation of two genetic polymorphisms of paraoxonase 1 in patients with pulmonary embolism.	Basol N et al.	29682786	Not a renal disease focus

For peer review only

1	HCMV UL97 phosphotransferase gene mutations may be associated with antiviral resistance in immunocompromised patients in BelÃ©m, PA, Northern Brazil.	Silva DFLD et al.	29768545	Not a renal disease focus
1	Screening for mutation hotspots in Bardet-Biedl syndrome patients from India.	Chandrasekar SP et al.	29806606	Not a renal disease focus
1	Angiotensin Converting Enzyme Gene Insertion/Deletion Variant and Familial Mediterranean Fever-related Amyloidosis.	Nursal AF et al.	29891744	Not a renal disease focus
1	Base Excision Repair Gene Polymorphisms and Wilms Tumor Susceptibility.	Zhu J et al.	29937070	Not a renal disease focus
1	Concomitance of Polymorphisms in Glutathione Transferase Omega Genes Is Associated with Risk of Clear Cell Renal Cell Carcinoma.	Radic TM et al.	30224590	Not a renal disease focus
1	Dissociation of Fatty Liver and Insulin Resistance in I148M PNPLA3 Carriers: Differences in Diacylglycerol (DAG) FA18:1 Lipid Species as a Possible Explanation.	Franko A et al.	30227635	Not a renal disease focus
1	[Uric acid stones in children. Identification and therapy of a newly detected defect of adenine-phosphoribosyltransferase (author's transl)].	Simmonds HA	118367	Not English or Spanish
1	[The functional properties of the erythrocyte membranes in patients with chronic glomerulonephritis].	Rakhov DA et al.	1475876	Not English or Spanish
1	[Lipid peroxidation and the antioxidant system in bicarbonate hemodialysis and acetate-free biofiltration].	Eiselt J et al.	7762187	Not English or Spanish
1	[A study on angiotensin-I converting enzyme polymorphism in CAPD patients].	Nishina M	9014479	Not English or Spanish
1	[Is PstI polymorphism of the angiotensin I converting enzyme gene associated with nephropathy development in non-insulin-dependent diabetes mellitus (preliminary study)].	Grzeszczak W et al.	9499204	Not English or Spanish
1	[Relationship between angiotensin 1 converting enzyme gene polymorphism and diabetic nephropathy].	Wu S et al.	9596955	Not English or Spanish
1	[Polymorphism studies of angiotensin converting enzyme gene in chronic glomerulonephritis].	Kutyrina IM et al.	10420452	Not English or Spanish
1	[Angiotensin-converting enzyme gene polymorphism and the clinical pathological features and progression in lupus nephritis].	Guan T et al.	10436947	Not English or Spanish

For peer review only

1	[Association between ACE gene polymorphism and therapeutic responsiveness of ACEI in diabetic nephropathy].	Wang L et al.	10923445	Not English or Spanish
1	[Increased clinical and economic advantages using PROSIT (proteinuria screening and intervention) in type 2 diabetic patients].	Gozzoli V et al.	11075242	Not English or Spanish
1	[Association between insertion-deletion polymorphism of the angiotensin-converting enzyme gene and development of angiopathies in patients with non-insulin dependent diabetes mellitus from the Chuvash Republic].	Miloserdova OV et al.	11234416	Not English or Spanish
1	[Renin-angiotensin system genes in chronic glomerulonephritis].	Buraczynska M et al.	11865575	Not English or Spanish
1	[The association between polymorphism of endothelial nitric oxide synthase gene and diabetic nephropathy].	Li C et al.	11930675	Not English or Spanish
1	[Relationship between serum angiotensin I-converting enzyme activity and diabetic nephropathy in patients with type II diabetes].	Liao L et al.	12016801	Not English or Spanish
1	[Acquired mutation of type II transforming growth factor-beta receptor gene in glomerulosclerosis].	Liu Z et al.	12126523	Not English or Spanish
1	[Renal effects of low doses of mercury].	Alinovi R et al.	12197269	Not English or Spanish
1	[Association of ecNOS 4 b/a polymorphism and end-stage chronic renal failure].	Lin S et al.	12421496	Not English or Spanish
1	[Associative analysis of the connection between mutation C1167T in the catalase gene and polymorphic marker D6S392 nearby the Mn-dependent superoxide dismutase gene with diabetic microangiopathies].	Chistiakov D et al.	12469627	Not English or Spanish
1	[Association of the renin-angiotensin system gene polymorphism with nephropathy in type II diabetes].	Buraczynska M et al.	12476891	Not English or Spanish
1	[Study on candidate genes of benazepril related cough in Chinese hypertensives].	Lu J et al.	12848919	Not English or Spanish
1	[Polymorphism at the glutathione S-transferase pi locus as a risk factor for ifosfamide nephrotoxicity in children].	Zielinska E et al.	12868187	Not English or Spanish
1	[Correlation analysis between WNK4 gene and essential hypertension].	Sun ZJ et al.	12905707	Not English or Spanish

For peer review only

1	[Arterial hypertension in glomerulonephritis].	Oko A et al.	14974362	Not English or Spanish
1	[Search for the association of polymorphic markers for genes coding for antioxidant defense enzymes, with development of diabetic polyneuropathies in patients with type 1 diabetes mellitus].	Zotova EV et al.	15125229	Not English or Spanish
1	[Association of the complex of polymorphic markers of ACE genes, aldosteron synthetase and endothelial synthetase of nitric oxide with progression of chronic glomerulonephritis].	Kamysheva ES et al.	15532370	Not English or Spanish
1	[Correlative study between angiotensin-converting enzyme gene polymorphism and hepatorenal syndrome].	Wu XX et al.	15698501	Not English or Spanish
1	[Screening for new binding proteins which interact with BM2 of influenza B virus with yeast two-hybrid system].	Yu H et al.	16027793	Not English or Spanish
1	[I/D relationship between polymorphism of ACE gene and progression of chronic glomerulonephritis].	Kaliev RR et al.	16078593	Not English or Spanish
1	[A search for association between the polymorphic markers of PON1 and PON2 genes and diabetic nephropathy in patients with type I diabetes mellitus].	Voron'ko OE et al.	16080611	Not English or Spanish
1	[Correlation of cyclin D1 overexpression to mutations of von hippel-lindau gene in renal clear cell carcinoma].	Ren YY et al.	16480581	Not English or Spanish
1	[Activation of RHOA gene transcription in epithelial tumors may be caused by gene amplification and/or demethylation of its promotor region].	Braga EA et al.	17086988	Not English or Spanish
1	[Association of aldosterone synthase gene -344 T/C polymorphism with early renal damage in Han nationality with essential hypertension].	Sun XJ et al.	17407071	Not English or Spanish
1	[Relationship between I/D polymorphism of angiotensin I converting enzyme gene and microvascular complications in type 2 diabetic patients].	Moleda P et al.	17941464	Not English or Spanish
1	[Inactivation of the VHL gene in sporadic clear cell renal cancer].	Mikhailenko DS et al.	18389622	Not English or Spanish
1	[Association of alpha-adducin and angiotensin converting enzyme gene polymorphisms with salt-sensitive hypertension and early renal injury].	Lu LH et al.	18393230	Not English or Spanish
1	[Distribution of polymorphism in endothelial nitric oxide synthase gene in Singapore Chinese and its association with diabetic nephropathy].	Dong JJ et al.	18476541	Not English or Spanish

For peer review only

1	[Effect of tissue factor pathway inhibitor on apoptosis of rat mesangial cells and Fas and bcl-2 expression].	Lin YF et al.	19094710	Not English or Spanish
1	[Genetic predisposition to systemic complications of arterial hypertension in maintenance haemodialysis patients].	Bzoma B et al.	19112833	Not English or Spanish
1	[Factors responsible for inter-individual variations in dosage/concentration of tacrolimus in renal transplant recipients].	Fu SJ et al.	19114346	Not English or Spanish
1	[Association analysis of genetic polymorphisms of TCF7L2, CDKAL1, SLC30A8, HHEX genes and microvascular complications of type 2 diabetes mellitus].	Fu LL et al.	22487833	Not English or Spanish
1	[Angiotensin-converting enyme insertion/deletion polymorphism and blood pressure regulation in type 2 diabetic patients].	Krajina-Andricevic M et al.	23120809	Not English or Spanish
1	[Association between mannose-binding lectin 2 gene and protein kinase C-beta 1 gene polymorphisms and type 2 diabetic macrovascular complications in northern Chinese Han population].	Zhang NN et al.	23225056	Not English or Spanish
1	[Gene polymorphisms of CYP3A5 and MDR-1 in Hans renal transplant recipients in Hunan Province].	Shao M et al.	23981990	Not English or Spanish
1	[Association of CYP3A5 and MDR1 genetic polymorphisms with the blood concentration of tacrolimus in Chinese liver and renal transplant recipients].	Sun JY et al.	24059111	Not English or Spanish
1	[Gene mutation analysis of X-linked hypophosphatemic rickets].	Song Y et al.	24229582	Not English or Spanish
1	[Mutational analysis of MYO1E in children with sporadic steroid-resistant nephrotic syndrome in Chinese Han ethnic group].	Zhao F et al.	25224051	Not English or Spanish
1	[Children with idiopathic hypogonadotropic hypogonadism: clinical data analysis and mutations analysis of KAL1 and FGFR1 gene].	Qin M et al.	25619354	Not English or Spanish
1	[DNA repair XRCC1, XPD genes polymorphism as associated with the development of bladder cancer and renal cell carcinoma].	Akhmadishina LZ et al.	25715450	Not English or Spanish
1	[Association between angiotensin-converting enzyme 2 gene polymorphisms and childhood primary nephrotic syndrome].	Qiu MY et al.	25815490	Not English or Spanish
1	[GENETIC RISK FACTORS FOR MULTIPLE KIDNEY STONE FORMATION IN THE RUSSIAN POPULATION].	Apolihin OI et al.	26665756	Not English or Spanish

For peer review only

1	[Polymorphism at the miR-502 binding site in the 3' untranslated region of SET8 gene is associated with the risk of clear cell renal cell carcinoma].	Xu JS et al.	27346408	Not English or Spanish
1	[Expression of cadherin17 in metanephric adenoma and its value in differential diagnosis].	Wang X et al.	27430690	Not English or Spanish
1	[Clinical and immunological analysis of patients with activated phosphoinositide 3-kinase Î´ syndrome resulting from PIK3CD mutation].	Tang WJ et al.	28072954	Not English or Spanish
1	[Molecular features of metanephric adenoma and their values in differential diagnosis].	Wang X et al.	28072975	Not English or Spanish
1	[Early diagnosis of risk for developing calcium oxalate urolithiasis].	Apolikhin OI et al.	28845932	Not English or Spanish
1	HLA extended haplotypes in steroid-sensitive nephrotic syndrome of childhood.	Lagueruela CC et al.	1974661	Paediatric individuals
1	Major histocompatibility complex antigens in steroid-responsive nephrotic syndrome.	McEnery PT et al.	2702084	Paediatric individuals
1	Glycosphingolipid levels in an unusual neurovisceral storage disease characterized by lactosylceramide galactosyl hydrolase deficiency: lactosylceramidosis.	Dawson G.	5016302	Paediatric individuals
1	Differentiation of human adult and fetal intestinal alkaline phosphatases with monoclonal antibodies.	Vockley J et al.	6437214	Paediatric individuals
1	Deficient fumarylacetoacetate fumarylhydrolase activity in lymphocytes and fibroblasts from patients with hereditary tyrosinemia.	Kvittingen EA et al.	6622096	Paediatric individuals
1	A polymorphism at codon 160 of human O6-methylguanine-DNA methyltransferase gene in young patients with adult type cancers and functional assay.	Imai Y et al.	7586149	Paediatric individuals
1	Streptokinase activity among group A streptococci in relation to streptokinase genotype, plasminogen binding, and disease manifestations.	Tewodros W et al.	7783598	Paediatric individuals
1	Heterogeneous AVPR2 gene mutations in congenital nephrogenic diabetes insipidus.	Wildin RS et al.	7913579	Paediatric individuals
1	Variability in the rate of 6-mercaptopurine methylation in the erythrocytes, liver and kidney in an Italian population.	Ferroni MA et al.	8880047	Paediatric individuals

For peer review only

1	Prostanoid biosynthesis by blood monocytes of children with hyperprostaglandin E syndrome.	Nusing RM et al.	9262230	Paediatric individuals
1	Angiotensin I-converting enzyme-gene-polymorphism: relationship to albumin excretion and blood pressure in pediatric patients with type-I-diabetes mellitus.	Pavlovic M et al.	9354852	Paediatric individuals
1	Inherited mutations in PTEN that are associated with breast cancer, cowden disease, and juvenile polyposis.	Lynch ED et al.	9399897	Paediatric individuals
1	ACE gene polymorphism in childhood IgA nephropathy: association with clinicopathologic findings.	Tanaka R et al.	9590186	Paediatric individuals
1	ETV6-NTRK3 gene fusions and trisomy 11 establish a histogenetic link between mesoblastic nephroma and congenital fibrosarcoma.	Knezevich SR et al.	9823307	Paediatric individuals
1	Genetic polymorphisms of the renin-angiotensin system and the outcome of focal segmental glomerulosclerosis in children.	Frishberg Y et al.	9853248	Paediatric individuals
1	Platelet-activating factor acetylhydrolase gene mutation in Japanese nephrotic children.	Xu H et al.	9853251	Paediatric individuals
1	Polymorphisms in angiotensin-converting enzyme gene and severity of renal disease in Henoch-Schoenlein patients. Italian Group of Renal Immunopathology.	Amoroso A et al.	9870486	Paediatric individuals
1	Implications of certain genetic polymorphisms in scarring in vesicoureteric reflux: importance of ACE polymorphism.	Ozen S et al.	10401028	Paediatric individuals
1	Role of platelet-activating factor acetylhydrolase gene mutation in Japanese childhood IgA nephropathy.	Tanaka R et al.	10430976	Paediatric individuals
1	ACE I/D gene polymorphism predicts renal damage in congenital uropathies.	Hohenfellner K et al.	10452281	Paediatric individuals
1	Thiopurine methyltransferase activity and its relationship to the occurrence of rejection episodes in paediatric renal transplant recipients treated with azathioprine.	Dervieux T et al.	10594482	Paediatric individuals
1	Platelet-activating factor acetylhydrolase gene mutation in Japanese children with Escherichia coli O157-associated hemolytic uremic syndrome.	Xu H et al.	10873870	Paediatric individuals
1	Angiotensin-converting enzyme gene insertion/deletion polymorphism and renal damage in childhood uropathies.	al-Eisa A et al.	10986863	Paediatric individuals

For peer review only

1	Genetic polymorphism in paraoxonase is a risk factor for childhood focal segmental glomerulosclerosis.	Frishberg Y et al.	11096050	Paediatric individuals
1	Polymorphisms of renin-angiotensin system genes in childhood IgA nephropathy.	Maruyama K et al.	11354780	Paediatric individuals
1	Impact of ACE I/D gene polymorphism on congenital renal malformations.	Hohenfellner K et al.	11354781	Paediatric individuals
1	Significance of ACE genotypes and medical treatments in childhood focal glomerulosclerosis.	Hori C et al.	11474225	Paediatric individuals
1	Angiotensin converting enzyme gene insertion/deletion polymorphism in idiopathic nephrotic syndrome in Kuwaiti Arab children.	Al-Eisa A et al.	11487079	Paediatric individuals
1	Distribution and development of CLN2 protein, the late-infantile neuronal ceroid lipofuscinosis gene product.	Kurachi Y et al.	11547947	Paediatric individuals
1	G protein beta3 subunit 825T genotype is not associated with differing outcome in pediatric renal transplant recipients.	Hocher B et al.	12000471	Paediatric individuals
1	Genomic rearrangements of EYA1 account for a large fraction of families with BOR syndrome.	Vervoort VS et al.	12404110	Paediatric individuals
1	Novel ATP6V1B1 and ATP6V0A4 mutations in autosomal recessive distal renal tubular acidosis with new evidence for hearing loss.	Stover EH et al.	12414817	Paediatric individuals
1	ACE gene polymorphism and renal scarring in primary vesicoureteric reflux.	Haszon I et al.	12478352	Paediatric individuals
1	Renin-angiotensin system polymorphisms and renal scarring.	Pardo R et al.	12579398	Paediatric individuals
1	Renin-angiotensin gene polymorphism in children with uremia and essential hypertension.	Papp F et al.	12579405	Paediatric individuals
1	HbA1c levels and erythrocyte transport functions in complication-free type 1 diabetic children and adolescents.	Deak B et al.	12682823	Paediatric individuals
1	Genetic heterogeneity of peroxisome biogenesis disorders among Japanese patients: evidence for a founder haplotype for the most common PEX10 gene mutation.	Shimozawa N et al.	12794690	Paediatric individuals
1	Angiotensin converting enzyme gene polymorphism in Asian Indian children with congenital uropathies.	Bajpai M et al.	14713838	Paediatric individuals

For peer review only

1	ACE I/D gene polymorphism in primary FSGS and steroid-sensitive nephrotic syndrome.	Oktem F et al.	14986085	Paediatric individuals
1	Renin-angiotensin system polymorphisms in Taiwanese primary vesicoureteral reflux.	Liu KP et al.	15045574	Paediatric individuals
1	Is ACE gene polymorphism a risk factor for renal scarring with low-grade reflux?	Erdogan H et al.	15138870	Paediatric individuals
1	ACE and AT1 receptor gene polymorphisms and renal scarring in urinary bladder dysfunction.	Kostic M et al.	15179569	Paediatric individuals
1	Angiotensin-converting enzyme gene insertion/deletion polymorphism in children with Henoch-Schonlein purpua nephritis.	Zhou J et al.	15315169	Paediatric individuals
1	Late effects on renal glomerular and tubular function in childhood cancer survivors.	Bardi E et al.	15390293	Paediatric individuals
1	Role of truncating mutations in MME gene in fetomaternal alloimmunisation and antenatal glomerulopathies.	Debiec H et al.	15464186	Paediatric individuals
1	Angiotensin-converting enzyme and angiotensin type 2 receptor gene genotype distributions in Italian children with congenital uropathies.	Rigoli L et al.	15470205	Paediatric individuals
1	Low renin-angiotensin system activity gene polymorphism and dysplasia associated with posterior urethral valves.	Peruzzi L et al.	16006956	Paediatric individuals
1	Polymorphisms of the angiotensin converting enzyme and angiotensin II type 1 receptor genes and renal scarring in non-uropathic children with recurrent urinary tract infection.	Ece A et al.	16109085	Paediatric individuals
1	Implication of genetic variations in congenital obstructive nephropathy.	Hahn H et al.	16133060	Paediatric individuals
1	Is paraoxonase 192 gene polymorphism a risk factor for membranoproliferative glomerulonephritis in children?	Bilge I et al.	16175651	Paediatric individuals
1	ACE gene insertion/deletion polymorphism in childhood idiopathic nephrotic syndrome.	Serdaroglu E et al.	16208534	Paediatric individuals
1	Angiotensin converting enzyme gene polymorphism in Indian children with steroid sensitive nephrotic syndrome.	Patil SJ et al.	16272677	Paediatric individuals
1	Evaluation of the antitumor efficacy, pharmacokinetics, and pharmacodynamics of the histone deacetylase inhibitor depsipeptide in childhood cancer models in vivo.	Graham C et al.	16397046	Paediatric individuals

For peer review only

1	ACE gene polymorphism in children with nephrotic syndrome in the Indonesian population.	Sasongko TH et al.	16421456	Paediatric individuals
1	Renin-angiotensin system gene polymorphisms: association with susceptibility to Henoch-Schonlein purpura and renal involvement.	Ozkaya O et al.	16521052	Paediatric individuals
1	Paraoxonase 1 192 and 55 polymorphisms in nephrotic children.	Biyikli NK et al.	16565923	Paediatric individuals
1	Angiotensin-converting enzyme gene polymorphism in children with idiopathic nephrotic syndrome.	Tsai IJ et al.	16645262	Paediatric individuals
1	ACE gene polymorphism in Turkish children with nephrotic syndrome.	Celik US et al.	16825089	Paediatric individuals
1	Roles of paraoxonase and oxidative stress in adolescents with uraemic, essential or obesity-induced hypertension.	Barath A et al.	16912512	Paediatric individuals
1	Endothelial nitric oxide synthase gene intron 4 a/b VNTR polymorphism in children with APSGN.	Dursun H et al.	16941147	Paediatric individuals
1	Bardet-Biedl syndrome gene variants are associated with both childhood and adult common obesity in French Caucasians.	Benzinou M et al.	17003356	Paediatric individuals
1	HLA class II influences humoral autoimmunity in patients with type 2 autoimmune hepatitis.	Djilali-Saiah et al.	17050030	Paediatric individuals
1	Nitric oxide synthase gene polymorphisms in children with primary nocturnal enuresis: a preliminary study.	Balat A et al.	17365914	Paediatric individuals
1	Molecular and functional characterization of novel glycerol-3-phosphate dehydrogenase 1 like gene (GPD1-L) mutations in sudden infant death syndrome.	Van Norstrand DW et al.	17967976	Paediatric individuals
1	Sudden infant death syndrome and activating GNAS1 gene mutations.	Roman R et al.	18075835	Paediatric individuals
1	Endothelial nitric oxide synthase gene T-786C and 27-bp repeat gene polymorphisms in retinopathy of prematurity.	Rusai K et al.	18334945	Paediatric individuals
1	Loss of nephrocystin-3 function can cause embryonic lethality, Meckel-Gruber-like syndrome, situs inversus, and renal-hepatic-pancreatic dysplasia.	Bergmann C et al.	18371931	Paediatric individuals
1	The polymorphism in insulin receptor substrate-1 gene and birth weight in neonates at term.	Simonska-Cichocka E et al.	18615395	Paediatric individuals

For peer review only

1	Report of a family segregating mutations in both the APC and MSH2 genes: juvenile onset of colorectal cancer in a double heterozygote.	Uhrhammer N et al.	18629513	Paediatric individuals
1	ACE gene polymorphism in Egyptian children with idiopathic nephrotic syndrome.	Fahmy ME et al.	18792483	Paediatric individuals
1	A common RET variant is associated with reduced newborn kidney size and function.	Zhang Z et al.	18820179	Paediatric individuals
1	Activation of the AKT/mTOR pathway in autosomal recessive polycystic kidney disease (ARPKD).	Fischer DC et al.	19176689	Paediatric individuals
1	Mutations of NPHP2 and NPHP3 in infantile nephronophthisis.	Tory K et al.	19177160	Paediatric individuals
1	Lethal cystic kidney disease in Amish neonates associated with homozygous nonsense mutation of NPHP3.	Simpson MA et al.	19303681	Paediatric individuals
1	Nitric oxide synthase gene polymorphisms in children with minimal change nephrotic syndrome.	Alasehirli B et al.	19371282	Paediatric individuals
1	ACE gene insertion/deletion polymorphism and renal scarring in children with urinary tract infections.	Sekerli E et al.	19603195	Paediatric individuals
1	Effect of paraoxonase 1 gene polymorphisms on clinical course of Henoch-SchÃ¶nlein purpura.	Yilmaz A et al.	19967651	Paediatric individuals
1	Hypophosphatemia, hyperphosphaturia, and bisphosphonate treatment are associated with survival beyond infancy in generalized arterial calcification of infancy.	Rutsch F et al.	20016754	Paediatric individuals
1	Changes in glomerular mesangium in kidneys with congenital nephrotic syndrome of the Finnish type.	Kaukinen A et al.	20020158	Paediatric individuals
1	Polymorphisms of the TNF-alpha and ACE genes, and renal scarring in infants with urinary tract infection.	Savvidou A et al.	20022049	Paediatric individuals
1	Genetic polymorphisms of 17 Î²-hydroxysteroid dehydrogenase 3 and the risk of hypospadias.	Sata F et al.	20059664	Paediatric individuals
1	Association of angiotensin converting enzyme and angiotensin type 2 receptor gene polymorphisms with renal damage in posterior urethral valves.	Laksmi NK et al.	20149750	Paediatric individuals
1	Glutathione S-transferase T1-null seems to be associated with graft failure in hematopoietic SCT.	Elhasid R et al.	20348973	Paediatric individuals

For peer review only

1	Linkage and association study of discoidin domain receptor 1 as a novel susceptibility gene for childhood IgA nephropathy.	Hahn WH et al.	20372823	Paediatric individuals
1	Effect of angiotensin-converting enzyme gene insertion/deletion polymorphism on steroid resistance in Egyptian children with idiopathic nephrotic syndrome.	Saber-Ayad M et al.	20418353	Paediatric individuals
1	Phosphodiesterase-5 gene (PDE5A) polymorphisms are associated with progression of childhood IgA nephropathy.	Hahn WH et al.	20563733	Paediatric individuals
1	Mutational analysis of the PLCE1 gene in steroid resistant nephrotic syndrome.	Boyer O et al.	20591883	Paediatric individuals
1	The association of genetic variability in patatin-like phospholipase domain-containing protein 3 (PNPLA3) with histological severity of nonalcoholic fatty liver disease.	Rotman Y et al.	20684021	Paediatric individuals
1	Founder mutations in the ATP6V1B1 gene explain most Cypriot cases of distal renal tubular acidosis: first prenatal diagnosis.	Elia A et al.	20805693	Paediatric individuals
1	Polymorphisms of insulin-like growth factor-1 (IGF-1) and IGF-1 receptor (IGF-1R) contribute to pathologic progression in childhood IgA nephropathy.	Hahn WH et al.	21047277	Paediatric individuals
1	Renal manifestations of patients with MYH9-related disorders.	Han KH et al.	21210153	Paediatric individuals
1	Clinical utility of genetic testing in children and adults with steroid-resistant nephrotic syndrome.	Santin S et al.	21415313	Paediatric individuals
1	Induction of podocyte-derived VEGF ameliorates podocyte injury and subsequent abnormal glomerular development caused by puromycin aminonucleoside.	Ma J et al.	21451433	Paediatric individuals
1	ADAMTS13 gene mutations in children with hemolytic uremic syndrome.	Choi HS et al.	21488199	Paediatric individuals
1	RET and GDNF mutations are rare in fetuses with renal agenesis or other severe kidney development defects.	Jeanpierre C et al.	21490379	Paediatric individuals
1	Endothelial nitric oxide synthase gene intron4 VNTR polymorphism in patients with chronic kidney disease.	Elshamaa MF et al.	21519233	Paediatric individuals
1	Disruption of PTPRO causes childhood-onset nephrotic syndrome.	Ozaltin F et al.	21722858	Paediatric individuals

For peer review only

1	Age and CYP3A5 genotype affect tacrolimus dosing requirements after transplant in pediatric heart recipients.	Gijsen V et al.	21930396	Paediatric individuals
1	DD genotype of ACE gene in boys: may it be a risk factor for minimal change nephrotic syndrome?	Alasehirli B et al.	22017506	Paediatric individuals
1	Association of the ACE-II genotype with the risk of nephrotic syndrome in Pakistani children.	Shahid S et al.	22033511	Paediatric individuals
1	C1GALT1 polymorphisms are associated with Henoch-SchÃ¶nlein purpura nephritis.	He X et al.	22544166	Paediatric individuals
1	Genes in the ureteric budding pathway: association study on vesico-ureteral reflux patients.	van Eerde AM et al.	22558067	Paediatric individuals
1	Genetic polymorphisms influence the steroid treatment of children with idiopathic nephrotic syndrome.	Chiou YH et al.	22610055	Paediatric individuals
1	Mutations in NEK8 link multiple organ dysplasia with altered Hippo signalling and increased c-MYC expression.	Frank V et al.	23418306	Paediatric individuals
1	Association of eNOS gene intron 4 a/b VNTR polymorphisms in children with nephrotic syndrome.	Dursun H et al.	23570878	Paediatric individuals
1	A molecular genetic analysis of childhood nephrotic syndrome in a cohort of Saudi Arabian families.	Al-Hamed MH et al.	23595123	Paediatric individuals
1	Cost-effective PKHD1 genetic testing for autosomal recessive polycystic kidney disease.	Krall P et al.	24162162	Paediatric individuals
1	EIF2AK3 mutations in South Indian children with permanent neonatal diabetes mellitus associated with Wolcott-Rallison syndrome.	Jahnavi S et al.	24168455	Paediatric individuals
1	Molecular diagnosis of distal renal tubular acidosis in Tunisian patients: proposed algorithm for Northern Africa populations for the ATP6V1B1, ATP6V0A4 and SCL4A1 genes.	Elhayek D et al.	24252324	Paediatric individuals
1	MMP-1 and -3 haplotype is associated with congenital anomalies of the kidney and urinary tract.	Djuric T et al.	24414606	Paediatric individuals
1	Mutation screening and array comparative genomic hybridization using a 180K oligonucleotide array in VACTERL association.	Winberg J et al.	24416387	Paediatric individuals
1	Conversion from twice- to once-daily tacrolimus in pediatric kidney recipients: a pharmacokinetic and bioequivalence study.	Lapeyraque AL et al.	24435759	Paediatric individuals

For peer review only

1	Muscle involvement in Dent disease 2.	Park E et al.	24912603	Paediatric individuals
1	PON1 Q192R polymorphism (rs662) is associated with childhood embryonal tumors.	Vasconcelos GM et al.	24972570	Paediatric individuals
1	Genetic analysis of strictly defined Leber congenital amaurosis with (and without) neurodevelopmental delay.	Khan AO et al.	24997176	Paediatric individuals
1	Modification of epigenetic patterns in low birth weight children: importance of hypomethylation of the ACE gene promoter.	Rangel M et al.	25170764	Paediatric individuals
1	Gene polymorphisms of adducin GLY460TRP, ACE I/D, AND AGT M235T in pediatric hypertension patients.	Kaplan I et al.	25262176	Paediatric individuals
1	Endothelial nitric oxide synthase gene intron 4 VNTR polymorphism in sickle cell disease: relation to vasculopathy and disease severity.	Tantawy AA et al.	25263931	Paediatric individuals
1	HLA-DQA1 and PLCG2 Are Candidate Risk Loci for Childhood-Onset Steroid-Sensitive Nephrotic Syndrome.	Gbadegesin RA et al.	25349203	Paediatric individuals
1	Activation of human telomerase reverse transcriptase through gene fusion in clear cell sarcoma of the kidney.	Karlsson J et al.	25481751	Paediatric individuals
1	Coinheritance of COL4A5 and MYO1E mutations accentuate the severity of kidney disease.	Lennon R et al.	25739341	Paediatric individuals
1	Novel carboxypeptidase A6 (CPA6) mutations identified in patients with juvenile myoclonic and generalized epilepsy.	Sapio MR et al.	25875328	Paediatric individuals
1	HPSE2 mutations in urofacial syndrome, non-neurogenic neurogenic bladder and lower urinary tract dysfunction.	Bulum B et al.	25924634	Paediatric individuals
1	Impact of common functional polymorphisms in renin angiotensin system genes on the risk of renal parenchymal scarring following childhood urinary tract infection.	Hussein A et al.	25939993	Paediatric individuals
1	Angiotensin-converting enzyme genotype is not a significant genetic risk factor for idiopathic nephrotic syndrome in Croatian children.	Batinic D et al.	25997642	Paediatric individuals
1	BRAF mutations in pediatric metanephric tumors.	Chami R et al.	26014474	Paediatric individuals
1	Expressions of mRNA for innate immunity-associated functional molecules in urinary sediment in immunoglobulin A nephropathy.	Tsuruga K et al.	26058859	Paediatric individuals

For peer review only

1	Association of ACE and MDR1 Gene Polymorphisms with Steroid Resistance in Children with Idiopathic Nephrotic Syndrome.	Dhandapani MC et al.	26154535	Paediatric individuals
1	Inflammasome polymorphisms in juvenile systemic lupus erythematosus.	Pontillo A et al.	26182076	Paediatric individuals
1	Associations of the eNOS G894T gene polymorphism with target organ damage in children with newly diagnosed primary hypertension.	Sladowska-Kozlowska J et al.	26227630	Paediatric individuals
1	Paraoxnase1 Gene Polymorphism in Childhood Idiopathic Nephrotic Syndrome.	Al-Eisa AA et al.	26780374	Paediatric individuals
1	Coagulation, thrombophilia and patency of arteriovenous fistula in children undergoing haemodialysis compared with healthy volunteers: a prospective analysis.	Fadel FI et al.	26829282	Paediatric individuals
1	Components of the lectin pathway of complement activation in paediatric patients of intensive care units.	Swierzko AS et al.	26850322	Paediatric individuals
1	ACE serum level and I/D gene polymorphism in children with obstructive uropathies and other congenital anomalies of the kidney and urinary tract.	Kostadinova ES et al.	27206329	Paediatric individuals
1	Association study between matrix metalloproteinase-9 gene (MMP9) polymorphisms and the risk of Henoch-SchÃ¶nlein purpura in children.	Xu ED et al.	27323137	Paediatric individuals
1	Clinicopathological features and BRAF(V600E) mutations in patients with isolated hypothalamic-pituitary Langerhans cell histiocytosis.	Huo Z et al.	27760550	Paediatric individuals
1	BRAF exon 15 mutations in pediatric renal stromal tumors: prevalence in metanephric stromal tumors.	Marsden L et al.	27769870	Paediatric individuals
1	Angiotensin-converting enzyme insertion/deletion gene polymorphism in Egyptian children with systemic lupus erythematosus: a possible relation to proliferative nephritis.	Hammad A et al.	27956582	Paediatric individuals
1	Estimation of the relationship between the polymorphisms of selected genes: ACE, AGTR1, TGFÎ²1 and GNB3 with the occurrence of primary vesicoureteral reflux.	Zyczkowski M et al.	27988909	Paediatric individuals
1	Spectrum of mutations in Chinese children with steroid-resistant nephrotic syndrome.	Wang F et al.	28204945	Paediatric individuals
1	Lectin pathway factors in patients suffering from juvenile idiopathic arthritis.	Kasperkiewicz K et al.	28405017	Paediatric individuals

For peer review only

1	Association of Endothelial Nitric Oxide Synthase Gene Polymorphism with Susceptibility and Nephritis Development of Henoch-SchÃ¶nlein Purpura in Chinese Han Children.	Wang A et al.	28409662	Paediatric individuals
1	Angiotensin-Converting Enzyme Gene Polymorphism in Children with Idiopathic Nephrotic Syndrome, Effect on Biopsy Findings.	Monajemzadeh M et al.	28481137	Paediatric individuals
1	Inducible nitric oxide synthase gene polymorphisms are associated with a risk of nephritis in Henoch-SchÃ¶nlein purpura children.	Jiang J et al.	28593405	Paediatric individuals
1	Inborn errors in RNA polymerase III underlie severe varicella zoster virus infections.	Ogunjimi B et al.	28783042	Paediatric individuals
1	Association of single-nucleotide polymorphism in the FKBP5 gene with response to steroids in pediatric patients with primary nephrotic syndromeâ€©.	Du N et al.	28992850	Paediatric individuals
1	Monogenic diabetes in overweight and obese youth diagnosed with type 2 diabetes: the TODAY clinical trial.	Kleinberger JW et al.	29758564	Paediatric individuals
1	Evaluation of Genetic Polymorphisms for Determining Steroid Response in Nephrotic Children.	Kara A et al.	30143489	Paediatric individuals
1	A randomized clinical trial of age and genotype-guided tacrolimus dosing after pediatric solid organ transplantation.	Min S et al.	30178515	Paediatric individuals
1	Pharmacogenetics of acute azathioprine toxicity: relationship to thiopurine methyltransferase genetic polymorphism.	Lennard L et al.	2758725	Pharmaceutical drug focus
1	Genetic association of 11 beta-hydroxysteroid dehydrogenase type 2 (HSD11B2) flanking microsatellites with essential hypertension in blacks.	Watson B Jr et al.	8794836	Pharmaceutical drug focus
1	Effect of deletion polymorphism of angiotensin converting enzyme gene on progression of diabetic nephropathy during inhibition of angiotensin converting enzyme: observational follow up study.	Parving HH et al.	8806248	Pharmaceutical drug focus
1	Effect of angiotensin-converting enzyme (ACE) gene polymorphism on progression of renal disease and the influence of ACE inhibition in IDDM patients: findings from the EUCLID Randomized Controlled Trial. EURODIAB Controlled Trial of Lisinopril in IDDM.	Penno G et al.	9726242	Pharmaceutical drug focus
1	Effects of erythropoietin, angiotensin II, and angiotensin-converting enzyme inhibitor on erythroid precursors in patients with posttransplantation erythrocytosis.	Glicklich D et al.	10428268	Pharmaceutical drug focus

For peer review only

1	Nifedipine improves endothelial function in hypercholesterolemia, independently of an effect on blood pressure or plasma lipids.	Verhaar MC et al.	10533616	Pharmaceutical drug focus
1	Pharmacogenetic analysis of the effect of angiotensin-converting enzyme inhibitor on restenosis after percutaneous transluminal coronary angioplasty.	Okamura A et al.	10535720	Pharmaceutical drug focus
1	The norbornenyl moiety of cyclothiazide determines the preference for flip-flop variants of AMPA receptor subunits.	Kessler M et al.	10854736	Pharmaceutical drug focus
1	Randomized placebo-controlled trial of perindopril in normotensive, normoalbuminuric patients with type 1 diabetes mellitus.	Kvetny J et al.	11181984	Pharmaceutical drug focus
1	Human thiopurine S-methyltransferase activity in uremia and after renal transplantation.	Weyer N et al.	11417444	Pharmaceutical drug focus
1	Long-term renoprotective effects of losartan in diabetic nephropathy: interaction with ACE insertion/deletion genotype?	Andersen S et al.	12716812	Pharmaceutical drug focus
1	The influence of the ACE (I/D) polymorphism on systemic and renal vascular responses to angiotensins in normotensive, normoalbuminuric Type 1 diabetes mellitus.	Luik PT et al.	12856080	Pharmaceutical drug focus
1	Venous response to nitroglycerin is enhanced in young, healthy carriers of the 825T allele of the G protein beta3 subunit gene (GNB3).	Mitchell A et al.	14586390	Pharmaceutical drug focus
1	Matrix metalloproteinase-1 gene polymorphism in renal transplant patients with and without gingival enlargement.	Kurzawski M et al.	16945025	Pharmaceutical drug focus
1	Enalapril and losartan affect lipid peroxidation in renal transplant recipients with renin-angiotensin system polymorphisms.	Rashtchizadeh N et al.	17222813	Pharmaceutical drug focus
1	Renin-angiotensin system polymorphisms and hemoglobin level in renal allografts: a comparative study between losartan and enalapril.	Noroozianavval M et al.	17524880	Pharmaceutical drug focus
1	Genetic analysis of fluvastatin response and dyslipidemia in renal transplant recipients.	Singer JB et al.	17563401	Pharmaceutical drug focus
1	Impact of the preintervention rate of renal function decline on outcome of renoprotective intervention.	Lely AT et al.	18077786	Pharmaceutical drug focus
1	CYP3A5 genotype is not associated with a higher risk of acute rejection in tacrolimus-treated renal transplant recipients.	Hesselink DA et al.	18334918	Pharmaceutical drug focus
1	Endothelial nitric oxide synthase gene polymorphisms and the renal hemodynamic response to L-arginine.	Cherney DZ et al.	19037250	Pharmaceutical drug focus

For peer review only

1	Modulating effect of atorvastatin on paraoxonase 1 activity in type 2 diabetic Egyptian patients with or without nephropathy.	Abdin AA et al.	19553142	Pharmaceutical drug focus
1	CYP2C9 genotype and pharmacodynamic responses to losartan in patients with primary and secondary kidney diseases.	Joy MS et al.	19669737	Pharmaceutical drug focus
1	Matrix metalloproteinase-3 gene polymorphism in renal transplant patients with gingival overgrowth.	Drozdzik A et al.	19778329	Pharmaceutical drug focus
1	Presence of CYP2C9*3 allele increases risk for hypoglycemia in Type 2 diabetic patients treated with sulfonylureas.	Ragia G et al.	19891554	Pharmaceutical drug focus
1	Drug-resistant cytomegalovirus in transplant recipients: a French cohort study.	Hantz S et al.	20961907	Pharmaceutical drug focus
1	CYP3A5 genotype does not influence everolimus in vitro metabolism and clinical pharmacokinetics in renal transplant recipients.	Picard N et al.	21389905	Pharmaceutical drug focus
1	Cytochrome P450 polymorphisms and the response of lupus nephritis to cyclophosphamide therapy.	Winoto J et al.	21543025	Pharmaceutical drug focus
1	Exaggerated natriuresis during clamping of systemic NO supply in healthy young men.	Simonsen JA et al.	21749320	Pharmaceutical drug focus
1	In vivo CYP3A activity is significantly lower in cyclosporine-treated as compared with tacrolimus-treated renal allograft recipients.	de Jonge H et al.	21753749	Pharmaceutical drug focus
1	Lack of association between the Trp719Arg polymorphism in kinesin-like protein-6 and cardiovascular risk and efficacy of atorvastatin among subjects with diabetes on dialysis: the 4D study.	Hoffmann MM et al.	21871624	Pharmaceutical drug focus
1	Influence of NAT2 polymorphisms on sulfamethoxazole pharmacokinetics in renal transplant recipients.	Kagaya H et al.	22106207	Pharmaceutical drug focus
1	Comparing antihypertensive effect and plasma ciclosporin concentration between amlodipine and valsartan regimens in hypertensive renal transplant patients receiving ciclosporin therapy.	Cai J et al.	22149319	Pharmaceutical drug focus
1	Genetic and clinical determinants of early, acute calcineurin inhibitor-related nephrotoxicity: results from a kidney transplant consortium.	Jacobson PA et al.	22334041	Pharmaceutical drug focus
1	Risk of tacrolimus toxicity in CYP3A5 nonexpressors treated with intravenous nicardipine after kidney transplantation.	Hooper DK et al.	22491658	Pharmaceutical drug focus
1	CYP3A5 polymorphism effect on cyclosporine pharmacokinetics in living donor renal transplant recipients: analysis by population pharmacokinetics.	Song J et al.	22947591	Pharmaceutical drug focus

For peer review only

1	Comparison of pharmacokinetics and pharmacogenetics of once- and twice-daily tacrolimus in the early stage after renal transplantation.	Niioka T et al.	23073468	Pharmaceutical drug focus
1	Impact of tacrolimus intraindividual variability and CYP3A5 genetic polymorphism on acute rejection in kidney transplantation.	Ro H et al.	23149441	Pharmaceutical drug focus
1	Endothelial nitric oxide synthase gene polymorphisms and renal responsiveness to RAS inhibition therapy in type 2 diabetic Asian Indians.	Cheema BS et al.	23260854	Pharmaceutical drug focus
1	Individualization of tacrolimus dosage basing on cytochrome P450 3A5 polymorphism--a prospective, randomized, controlled study.	Chen SY et al.	23432535	Pharmaceutical drug focus
1	Genetic polymorphisms of UGT1A8, UGT1A9 and HNF-1Î± and gastrointestinal symptoms in renal transplant recipients taking mycophenolic acid.	Vu D et al.	23721685	Pharmaceutical drug focus
1	CYP2C9*2 allele increases risk for hypoglycemia in POR*1/*1 type 2 diabetic patients treated with sulfonylureas.	Ragia G et al.	24464600	Pharmaceutical drug focus
1	Association of CYP3A4*18B and CYP3A5*3 polymorphism with cyclosporine-related liver injury in Chinese renal transplant recipients.	Xin HW et al.	24691060	Pharmaceutical drug focus
1	Genetic variance in ABCB1 and CYP3A5 does not contribute toward the development of chronic kidney disease after liver transplantation.	Tapirdamaz O et al.	25014506	Pharmaceutical drug focus
1	Associations of HSD11B1 polymorphisms with tacrolimus concentrations in Chinese renal transplant recipients with prednisone combined therapy.	Liu X et al.	25587129	Pharmaceutical drug focus
3	Impact of interaction of cigarette smoking with angiotensin-converting enzyme polymorphisms on end-stage renal disease risk in a Han Chinese population	Yang HY et al.	23477970	< 3 populations reported per SNP
3	Polymorphisms in the gene encoding angiotensin I converting enzyme 2 and diabetic nephropathy	Frojdo S et al.	16211375	< 3 populations reported per SNP
3	Investigation of ACE, ACE2 and AGTR1 genes for association with nephropathy in Type 1 diabetes mellitus.	Currie D et al.	20854388	< 3 populations reported per SNP

For peer review only

Supplementary Table S3c: Excluded studies from the *AGT* search

*Exclusion stage relates to the stage at which the article was removed as shown in the flow diagram (Supplementary Figure S1c)

Exclusion Stage	Title	Authors	Pubmed ID or WoS ID if Pubmed ID not available	Reason
1	Identification of potential candidate genes for hypertensive nephropathy based on gene expression profile	Chen Z et al.	27756246	Gene expression based study
1	Defining Uremic Arterial Functional Abnormalities in Patients Recently Started on Haemodialysis: Combined In Vivo and Ex Vivo Assessment	Abushufa AM et al.	25546407	No data for AGT
1	Angiotensin I Converting Enzyme Gene Polymorphism in Type 2 Diabetes Mellitus with Nephropathy in Saudi Population	Alharbi SA et al.	WOS:000415063100003	No data for AGT
1	Clinical impact of an angiotensin I-converting enzyme insertion/deletion and kinin B2 receptor +9/-9 polymorphisms in the prognosis of renal transplantation	Amorim CEN et al.	23362199	No data for AGT
1	Transforming growth factor beta and progression of renal disease	August P et al.	WOS:000185812000016	No data for AGT
1	Associations of fractalkine receptor (CX3CR1) and CCR5 gene variants with hypertension, diabetes and atherosclerosis in chronic renal failure patients undergoing hemodialysis	Bagci B et al.	27118566	No data for AGT
1	Effect of ACE and AT-2 inhibitors on mortality and progression to microalbuminuria in a nested case-control study of diabetic nephropathy in diabetes mellitus type 2: results from the GENDIAN study.	Boger CA et al.	16961167	No data for AGT
1	The ras responsive transcription factor RREB1 is a novel candidate gene for type 2 diabetes associated end-stage kidney disease	Bonomo JA et al.	25027322	No data for AGT
1	Angiotensin II type 1 receptor gene polymorphism in end-stage renal disease	Buraczyńska M et al.	12187084	No data for AGT

For peer review only

1	Endothelial nitric oxide synthase gene polymorphisms and renal responsiveness to RAS inhibition therapy in type 2 diabetic Asian Indians	Cheema BS et al.	23260854	No data for AGT
1	Polymorphism of angiotensin II receptor gene and microangiopathies in patients with insulin-dependent diabetes mellitus	Chistyakov DA et al.	WOS:000084973600016	No data for AGT
1	Association of renin-angiotensin and endothelial nitric oxide synthase gene polymorphisms with blood pressure progression and incident hypertension: prospective cohort study.	Conen D et al.	18698212	No data for AGT
1	Serological and genetic factors in early recurrence of IgA nephropathy after renal transplantation	Coppo R et al.	17988266	No data for AGT
1	Plasma renin and prorenin and renin gene variation in patients with insulin-dependent diabetes mellitus and nephropathy	Deinum J et al.	10462269	No data for AGT
1	Insertion/deletion polymorphism of the angiotensin-converting enzyme gene in normalcy and among diabetics with vascular complications	Demurov LM et al.	WOS:A1997XE98500008	No data for AGT
1	Haplotype analysis of NAD(P)H oxidase p22 phox polymorphisms in end-stage renal disease	Doi K et al.	16215641	No data for AGT
1	Genetic predisposition to diabetic nephropathy. Evidence for a role of the angiotensin I--converting enzyme gene	Doria A et al.	7909524	No data for AGT
1	Synergistic effect of angiotensin II type 1 receptor genotype and poor glycaemic control on risk of nephropathy in IDDM	Doria A et al.	9389421	No data for AGT
1	Analysis of three genetic markers in IgA nephropathy patients from a single region	Drouet M et al.	12005241	No data for AGT
1	Angiotensin II type 1 receptor (A1166C) gene polymorphism in Egyptian adult hemodialysis patients	El-Banawy H et al.	WOS:000365886400009	No data for AGT
1	Angiotensin-I converting enzyme gene polymorphism in Turkish type 2 diabetic patients	Ergen HA et al.	WOS:000223974500007	No data for AGT

For peer review only

1	ACE gene polymorphism and serum ACE activity in Iranians type II diabetic patients with macroalbuminuria	Felehgari V et al.	20830509	No data for AGT
1	DNA polymorphisms in the ACE gene, serum ACE activity and the risk of nephropathy in insulin-dependent diabetes mellitus	Freire MBS et al.	9794558	No data for AGT
1	Polymorphisms in the gene encoding angiotensin I converting enzyme 2 and diabetic nephropathy	Frojdo S et al.	16211375	No data for AGT
1	Association of fibronectin Msp iv polymorphism and diabetic nephropathy susceptibility in Chinese Han population	Gao JX et al.	26045844	No data for AGT
1	Effects of erythropoietin, angiotensin II, and angiotensin-converting enzyme inhibitor on erythroid precursors in patients with posttransplantation erythrocytosis	Glicklich D et al.	10428268	No data for AGT
1	Association of Angiotensin converting Enzyme (ACE) gene polymorphism and diabetic nephropathy	Golmohamadi T et al.	WOS:000242619000003	No data for AGT
1	Angiotensin I-converting enzyme gene polymorphisms: Relationship to nephropathy in patients with non-insulin dependent diabetes mellitus	Grzeszczak W et al.	9727375	No data for AGT
1	Susceptibility and progression of end stage renal disease are not associated with angiotensin II type 1 receptor gene polymorphism	Hanna MOF et al.	25316403	No data for AGT
1	Polymorphism of the angiotensin I-converting enzyme gene in diabetic nephropathy in type II diabetic patients with proliferative retinopathy	Hanyu O et al.	9509566	No data for AGT
1	Angiotensin Converting Enzyme Insertion/Deletion gene polymorphism and genomic sequence in Diabetic Nephropathy	Haque SF et al.	WOS:000300381000004	No data for AGT
1	The angiotensin I-converting enzyme (ACE) locus is strongly associated with age and duration of diabetes in patients with type I diabetes	Hibberd ML et al.	9025006	No data for AGT
1	Role of transforming growth factor beta-1 gene polymorphisms in the development of chronic allograft nephropathy in renal transplant recipients	Inigo P et al.	14558330	No data for AGT

1	Neuropeptide YY1 receptor polymorphism as a prognostic predictor in Japanese patients with IgA nephropathy	Ito H et al.	10363627	No data for AGT
1	Carnosine as a protective factor in diabetic nephropathy - Association with a leucine repeat of the carnosinase gene CNDP1	Janssen B et al.	16046297	No data for AGT
1	Impact of Polymorphisms of the Genes Encoding Angiotensin II-Forming Enzymes on the Progression of IgA Nephropathy	Jung ES et al.	21150220	No data for AGT
1	Proteomic analysis of alpha-1-antitrypsin in immunoglobulin A nephropathy	Kwak NJ et al.	21136694	No data for AGT
1	Renal perfusion and the renal hemodynamic response to blocking the renin system in diabetes - Are the forces leading to vasodilation and vasoconstriction linked?	Lansang MC et al.	12086929	No data for AGT
1	Polymorphism in IgA nephropathy	Liu ZH et al.	WOS:A1997WW80600013	No data for AGT
1	The angiotensin-I converting enzyme gene I/D variation contributes to end-stage renal disease risk in Chinese patients with type 2 diabetes receiving hemodialysis	Lu M et al.	27633502	No data for AGT
1	The influence of three endothelin-1 polymorphisms on the progression of IgA nephropathy	Maixnerova D et al.	17328840	No data for AGT
1	The influence of two megsin polymorphisms on the progression of IgA nephropathy	Maixnerova D et al.	18498720	No data for AGT
1	Relationships Between Angiotensin-I Converting-Enzyme Gene Polymorphism, Plasma-Levels, And Diabetic Retinal And Renal Complications	Marre M et al.	8314010	No data for AGT
1	Hereditary factors in the development of diabetic renal disease	Marre M et al.	10922971	No data for AGT
1	A rare haplotype of the vitamin D receptor gene is protective against diabetic nephropathy	Martin RJL et al.	19783860	No data for AGT
1	Mutational Analysis of Agxt in Tunisian Population with Primary Hyperoxaluria Type 1.	M'dimegh S et al.	27935012	No data for AGT
1	Kinin-dependent hypersensitivity reactions in hemodialysis: metabolic and genetic factors.	Molinaro G et al.	17003818	No data for AGT

For peer review only - http://bmjopen.bmj.com/site/about/guidelines.xhtml

1	A polymorphism in the angiotensin II type 1 receptor gene has different effects on the risk of diabetic nephropathy in men and women	Mollsten A et al.	21316998	No data for AGT
1	Relationship of angiotensin-converting enzyme gene polymorphism with nephropathy associated with Type 2 diabetes mellitus in Asian Indians	Movva S et al.	17616353	No data for AGT
1	Relationship of Serum Klotho Level With ACE Gene Polymorphism in Stable Kidney Allograft Recipients	Nahandi MZ et al.	28270648	No data for AGT
1	The effect of angiotensin receptor blockade (ARB) on the regression of left ventricular hypertrophy in hemodialysis patients: comparison between patients with D allele and non-D allele (ACE gene polymorphism)	Nakayama M et al.	16312263	No data for AGT
1	Different Mechanisms for the Progression of CKD with ACE Gene Polymorphisms	Nakayama Y et al.	19293592	No data for AGT
1	A disease haplotype for advanced nephropathy in type 2 diabetes at the ACE locus	Ng DPK et al.	16936219	No data for AGT
1	Relationships between angiotensin I converting enzyme gene polymorphism and renal complications in Korean IDDM patients.	Oh TG et al.	8854649	No data for AGT
1	alpha(1)-antitrypsin gene polymorphisms are not associated with renal arterial fibromuscular dysplasia	Perdu J et al.	16531799	No data for AGT
1	The frequency of factor V Leiden mutation, ACE gene polymorphism, serum ACE activity and response to ACE inhibitor and angiotensin II receptor antagonist drugs in Iranians type II diabetic patients with microalbuminuria	Rahimi Z et al.	20853144	No data for AGT
1	AGTR1 rs5186 variants in patients with type 2 diabetes mellitus and nephropathy	Razi F et al.	WOS:000419720600009	No data for AGT
1	Angiotensin-converting enzyme polymorphism in patients with terminal renal failure	Schmidt A et al.	8785402	No data for AGT

For peer review only

1	No association of converting enzyme insertion/deletion polymorphism with immunoglobulin A glomerulonephritis	Schmidt S et al.	7485124	No data for AGT
1	Angiotensin I converting enzyme gene polymorphism and diabetic nephropathy in type II diabetes	Schmidt S et al.	9269698	No data for AGT
1	ACACÎ^2 gene (rs2268388) and AGTR1 gene (rs5186) polymorphism and the risk of nephropathy in Asian Indian patients with type 2 diabetes.	Shah VN et al.	23081748	No data for AGT
1	Relations between eNOS Glu298Asp polymorphism and progression of diabetic nephropathy	Shin Shin Y et al.	15331206	No data for AGT
1	Angiotensin I-converting enzyme genotype significantly affects progression of IgA glomerulonephritis in an Italian population	Stratta P et al.	10352195	No data for AGT
1	The association of LOX-1 rs1050283 polymorphism with renal hypertension susceptibility in a Chinese population	Sun YC et al.	WOS:000395739000087	No data for AGT
1	Lack Of Relationship Between An Insertion Deletion Polymorphism In The Angiotensin I-Converting Enzyme Gene And Diabetic Nephropathy And Proliferative Retinopathy In Iddm Patients	Tarnow L et al.	7729604	No data for AGT
1	Angiotensin-II type 1 receptor gene polymorphism and diabetic microangiopathy.	Tarnow L et al.	8671962	No data for AGT
1	High prevalence of ACE DD genotype among north Indian end stage renal disease patients.	Tripathi G et al.	17042963	No data for AGT
1	Angiotensin-converting enzyme gene polymorphism and vascular manifestations in Korean patients with SLE	Uhm WS et al.	12043886	No data for AGT
1	Increased expression of monocytic angiotensin-converting enzyme in dialysis patients with cardiovascular disease	Ulrich C et al.	16476718	No data for AGT
1	Synergistic expression of angiotensin-converting enzyme (ACE) and ACE2 in human renal tissue and confounding effects of hypertension on the ACE to ACE2 ratio	Wakahara S et al.	17303661	No data for AGT
1	Angiotensin-converting enzyme inhibitor versus angiotensin 2 receptor antagonist therapy and the influence of angiotensin-converting enzyme gene polymorphism in IgA nephritis.	Woo KT et al.	18536822	No data for AGT

For peer review only

1	Involvement of platelet-derived growth factor and histocompatibility of DRB 1 in chronic renal allograft nephropathy	Yamada K et al.	11349729	No data for AGT
1	Angiotensin converting enzyme gene polymorphism and development of post-transplant erythrocytosis.	Yildiz A et al.	12832741	No data for AGT
1	Angiotensin II type 2 receptor gene is not responsible for familial vesicoureteral reflux	Yoneda A et al.	12187255	No data for AGT
1	Polymorphism Of The Angiotensin-Converting Enzyme Gene And Clinical Aspects Of Iga Nephropathy	Yorioka T et al.	8529313	No data for AGT
1	Interleukin-4 (IL4)-590C/T (rs2243250) gene polymorphism is not associated with diabetic nephropathy (DN) in Caucasians with type 2 diabetes mellitus (T2DM)	Zavrsnik M et al.	29514038	No data for AGT
1	Hemodynamic Parameters During Normal And Hypertensive Pregnancy In Rats: Evaluation Of Renal Salt And Water Transporters	Abreu N et al.	18293204	Non-human study
1	DPP-4 Inhibition on Top of Angiotensin Receptor Blockade Offers a New Therapeutic Approach for Diabetic Nephropathy	Alter ML et al.	23171828	Non-human study
1	Long-Term Angiotensin II Receptor Blockade Limits Hypertension, Aortic Dysfunction, and Structural Remodeling in a Rat Model of Chronic Kidney Disease	Ameer OZ et al.	27880955	Non-human study
1	Effects of Diets with Different Proportions of Protein/Carbohydrate on Retinal Manifestations in db Mice	Arimura E et al.	29475908	Non-human study
1	Identification of Cathepsin L as a Potential Sex-Specific Biomarker for Renal Damage	Bauer Y et al.	21357272	Non-human study
1	Blood pressure (BP) and renal vasoconstrictor responses to acute blockade of nitric oxide: persistence of renal vasoconstriction despite normalization of BP with either verapamil or sodium nitroprusside.	Baylis C et al.	7562480	Non-human study
1	Specific pregnancy-induced angiotensin II type-1 receptor expression in ovine uterine artery does not involve formation of alternate splice variants or alternate promoter usage	Bird IM et al.	9687288	Non-human study

For peer review only

1	N-terminal residues control proteasomal degradation of RGS2, RGS4, and RGS5 in human embryonic kidney 293 cells	Bodenstein J et al.	17220356	Non-human study
1	Angiotensinogen concentrations and renin clearance : implications for blood pressure regulation.	Bohlender J et al.	10720595	Non-human study
1	Blood pressure and renin-angiotensin system resetting in transgenic rats with elevated plasma Val(5)-angiotensinogen	Bohlender J et al.	22728903	Non-human study
1	CCN1 expression in interleukin-6 deficient mouse kidney in experimental model of heart failure	Bonda TA et al.	23690222	Non-human study
1	Advanced Glycated End-Products Affect HIF-Transcriptional Activity in Renal Cells	Bondeva T et al.	24030251	Non-human study
1	Lack of in vivo function of osteopontin in experimental anti-GBM nephritis	Bonvini JM et al.	10966489	Non-human study
1	Hypoxia/Reoxygenation of Rat Renal Arteries Impairs Vasorelaxation via Modulation of Endothelium-Independent sGC/cGMP/PKG Signaling	Braun D et al.	29773995	Non-human study
1	Angiotensin II mesenteric and renal vasoregulation: Dissimilar modulatory effects with nitroprusside	Broome M et al.	11065204	Non-human study
1	N-domain angiotensin I-converting enzyme expression in renal artery of Wistar, Wistar Kyoto, and spontaneously hypertensive rats	Bueno V et al.	15194348	Non-human study
1	Organizational diversity among distinct glycoprotein endoplasmic reticulum-associated degradation programs	Cabral CM et al.	12181335	Non-human study
1	Role of NOX2 in the regulation of afferent arteriole responsiveness	Carlstrom M et al.	18987286	Non-human study
1	Adrenomedullin gene expression differences in mice do not affect blood pressure but modulate hypertension-induced pathology in males	Caron K et al.	17360661	Non-human study
1	Appropriate regulation of renin and blood pressure in 45-kb human renin/human angiotensinogen transgenic mice.	Catanzaro DF et al.	9931123	Non-human study
1	Mice lacking endothelial ACE - Normal blood pressure with elevated angiotensin	Cole JM et al.	12574101	Non-human study

For peer review only

1	Natriuretic peptides buffer renin-dependent hypertension	Demerath T et al.	24717731	Non-human study
1	Renin-angiotensin system transgenic mouse model recapitulates pathophysiology similar to human preeclampsia with renal injury that may be mediated through VEGF.	Denney JM et al.	27927648	Non-human study
1	Mycophenolate mofetil prevents cerebrovascular injury in stroke-prone spontaneously hypertensive rats	Dhande IS et al.	28011882	Non-human study
1	Inhibition of proximal tubular fluid absorption by nitric oxide and atrial natriuretic peptide in rat kidney	Eitle E et al.	9575805	Non-human study
1	Complex interactions of NO/cGMP/PKG systems on Ca2+ signaling in afferent arteriolar vascular smooth muscle	Fellner SK et al.	19880669	Non-human study
1	Cardiac angiotensin-(1-12) expression and systemic hypertension in rats expressing the human angiotensinogen gene.	Ferrario CM et al.	26873967	Non-human study
1	Attenuation of accelerated renal cystogenesis in Pkd1 mice by renin-angiotensin system blockade	Fitzgibbon WR et al.	29021226	Non-human study
1	Renal cyst growth is the main determinant for hypertension and concentrating deficit in Pkd1-deficient mice	Fonseca JM et al.	24429399	Non-human study
1	Acute elevations in salt intake and reduced renal mass hypertension compromise arteriolar dilation in rat cremaster muscle	Frisbee JC et al.	10329253	Non-human study
1	Contribution of cytochrome P-450 omega-hydroxylase to altered arteriolar reactivity with high-salt diet and hypertension	Frisbee JC et al.	10775129	Non-human study
1	Adenosine A(1) receptor-dependent and independent pathways in modulating renal vascular responses to angiotensin II	Gao X et al.	25251152	Non-human study
1	Connexin 43 is not essential for the control of renin synthesis and secretion	Gerl M et al.	24062052	Non-human study
1	Metformin prevents the impairment of endothelium-dependent vascular relaxation induced by high glucose challenge in rabbit isolated perfused kidneys	Gomes MB et al.	16133490	Non-human study
1	Effect of the angiotensinogen genotype on experimental hypertension in mice	Handtrack C et al.	17333097	Non-human study

For peer review only

1	Cytoprotective effects of nitrates in a cellular model of hydronephrosis	Hegarty NJ et al.	12081565	Non-human study
1	The genetic deletion of Mas abolishes salt induced hypertension in mice	Heringer-Walther S et al.	22652430	Non-human study
1	Pressor and sympathoexcitatory effects of nitric oxide in the rostral ventrolateral medulla	Hirooka Y et al.	8934360	Non-human study
1	An essential role of angiotensin II receptor type 1a in recipient kidney, not in transplanted peripheral blood leukocytes, in progressive immune-mediated renal injury	Hisada Y et al.	11555672	Non-human study
1	The angiotensin type II receptor tonically inhibits angiotensin-converting enzyme in AT2 null mutant mice	Hunley TE et al.	10652034	Non-human study
1	NOS1-dependent negative feedback regulation of the epithelial sodium channel in the collecting duct	Hyndman KA et al.	25391901	Non-human study
1	Collecting Duct Nitric Oxide Synthase 1 beta Activation Maintains Sodium Homeostasis During High Sodium Intake Through Suppression of Aldosterone and Renal Angiotensin II Pathways	Hyndman KA et al.	29066445	Non-human study
1	Pathophysiological roles of adrenomedullin-RAMP2 system in acute and chronic cerebral ischemia	Igarashi K et al.	25252154	Non-human study
1	Role of A(1) receptors in renal sympathetic neurotransmission in the mouse kidney	Jackson EK et al.	22874760	Non-human study
1	mPGES-1 deletion potentiates urine concentrating capability after water deprivation	Jia ZJ et al.	22237797	Non-human study
1	Transfer of a salt-resistant renin allele raises blood pressure in Dahl salt-sensitive rats	Jiang J et al.	9040448	Non-human study
1	Angiotensin-converting enzyme inhibition attenuates the progression of rat hepatic fibrosis	Jonsson JR et al.	11438504	Non-human study
1	Rat Ace allele variation determines susceptibility to AngII-induced renal damage	Kamilic J et al.	21788250	Non-human study
1	Genetic-Control Of Blood-Pressure And The Angiotensinogen Locus	Kim HS et al.	7708716	Non-human study

For peer review only

1	Regulation of renin secretion and expression in mice deficient in ss 1-and ss 2-adrenergic receptors	Kim SM et al.	17515456	Non-human study
1	Low Blood Pressure in Endothelial Cell-Specific Endothelin 1 Knockout Mice	Kisanuki YY et al.	20516397	Non-human study
1	Lack of an effect of collecting duct-specific deletion of adenylyl cyclase 3 on renal Na+ and water excretion or arterial pressure	Kittikulsuth W et al.	24431204	Non-human study
1	Effect of the plasminogen-plasmin system on hypertensive renal and cardiac damage	Knier B et al.	21610512	Non-human study
1	Axl mediates vascular remodeling induced by deoxycorticosterone acetate-salt hypertension	Korshunov VA et al.	17923589	Non-human study
1	Reciprocal expression of connexin 40 and 45 during phenotypical changes in renin-secreting cells	Kurt B et al.	21209011	Non-human study
1	Stimulation of renin secretion by NO donors is related to the cAMP pathway	Kurtz A et al.	9575895	Non-human study
1	The angiotensin II receptor blocker candesartan improves survival and mesenteric perfusion in an acute porcine endotoxin model	Laesser M et al.	14995942	Non-human study
1	Physiological impact of increased expression of the AT(1) angiotensin receptor	Le TH et al.	12963678	Non-human study
1	Nitric oxide reduces the molecular activity of Na+,K+-ATPase in opossum kidney cells	Liang MY et al.	10432402	Non-human study
1	Regulation of sympathetic nerve activity in heart failure - A role for nitric oxide and angiotensin II	Liu JL et al.	10066676	Non-human study
1	Overexpression of cytochrome P450 4F2 in mice increases 20-hydroxyeicosatetraenoic acid production and arterial blood pressure	Liu XL et al.	19279555	Non-human study
1	Sodium-Nitroprusside Increases Glomerular Capillary Hydraulic Conductivity In Isolated Rat Glomeruli	Lovell HB et al.	7706899	Non-human study
1	Macula Densa Nitric Oxide Synthase 1 beta Protects against Salt-Sensitive Hypertension	Lu Y et al.	26647426	Non-human study
1	Correction of an enzyme trafficking defect in hereditary kidney stone disease in vitro	Lumb MJ et al.	12737622	Non-human study

For peer review only

1	Development and Analysis of Alpha 1-Antitrypsin Neoglycoproteins: The Impact of Additional N-Glycosylation Sites on Serum Half-Life	Lusch A et al.	23668542	Non-human study
1	Connexin 40 is dispensable for vascular renin cell recruitment but is indispensable for vascular baroreceptor control of renin secretion	Machura K et al.	25241776	Non-human study
1	Angiotensin receptor-binding protein ATRAP/Agtrap inhibits metabolic dysfunction with visceral obesity.	Maeda A et al.	23902639	Non-human study
1	Effect of sodium nitroprusside on norepinephrine overflow and antidiuresis induced by stimulation of renal nerves in anesthetized dogs	Maekawa H et al.	8720419	Non-human study
1	Hypertension in unilaterally nephrectomized rats induced by single-kidney transfection with angiotensinogen cDNA	Marley WS et al.	10567853	Non-human study
1	A novel rodent model of pregnancy complications associated with genetically determined angiotensin-converting enzyme (ACE) activity	Mata-Greenwood E et al.	29360395	Non-human study
1	Chronic regulation of arterial blood pressure by ANP: role of endogenous vasoactive endothelial factors	Melo LG et al.	9815091	Non-human study
1	Chronic hypertension and altered baroreflex responses in transgenic mice containing the human renin and human angiotensinogen genes	Merrill DC et al.	8613528	Non-human study
1	Role of endothelium-derived relaxing factors in the renal response to vasoactive agents in hypothyroid rats	Moreno JM et al.	12657567	Non-human study
1	Hypervolemia of pregnancy is not maintained in mice chronically overexpressing angiotensinogen	Morgan TK et al.	16796982	Non-human study
1	Vascular angiotensin-converting enzyme expression regulates local angiotensin II	Muller DN et al.	9039087	Non-human study
1	AKAP150 is required for stuttering persistent Ca2+ sparklets and angiotensin II-induced hypertension	Navedo MF et al.	18174462	Non-human study
1	Angiotensin-Ii Enhances Norepinephrine Spillover During Sympathetic Activation In Conscious Rabbits	Noshiro T et al.	WOS:A1994NP99600022	Non-human study

For peer review only

1	Effects of potassium adaptation on blood pressure and pressor responses in normotensive and renal hypertensive Wistar rats	Omogbai EL et al.	15834453	Non-human study
1	Altered regulation of renal interstitial hydrostatic pressure and the renal renin-angiotensin system in the absence of atrial natriuretic peptide	O'Tierney PF et al.	18192845	Non-human study
1	Effects of Stimulation of Soluble Guanylate Cyclase on Diabetic Nephropathy in Diabetic eNOS Knockout Mice on Top of Angiotensin II Receptor Blockade	Ott IM et al.	22900035	Non-human study
1	Nitric oxide modulates angiotensin II- and norepinephrine-dependent vasoconstriction in rat kidney	Parekh N et al.	8780230	Non-human study
1	Gene Trapping Uncovers Sex-Specific Mechanisms for Upstream Stimulatory Factors 1 and 2 in Angiotensinogen Expression	Park S et al.	22547438	Non-human study
1	Hyperplastic Growth Of Aortic Smooth-Muscle Cells In Renovascular Hypertensive Rabbits Is Characterized By The Expansion Of An Immature Cell Phenotype	Pauletto P et al.	8156626	Non-human study
1	Mechanical Basis of Osmosensory Transduction in Magnocellular Neurosecretory Neurones of the Rat Supraoptic Nucleus	Prager-Khoutorsky M et al.	25712904	Non-human study
1	Calcium/Calmodulin-Dependent Kinase II Inhibition in Smooth Muscle Reduces Angiotensin II-Induced Hypertension by Controlling Aortic Remodeling and Baroreceptor Function	Prasad AM et al.	26077587	Non-human study
1	Endothelium-Dependent Control Of Vascular Tone In The Rabbit Kidney After Ischemia And Reperfusion	Pruneau D et al.	8453977	Non-human study
1	Angiotensin II stimulates the synthesis and secretion of vascular permeability factor vascular endothelial growth factor in human mesangial cells	Pupilli C et al.	10215323	Non-human study
1	Loss of Notch3 Signaling in Vascular Smooth Muscle Cells Promotes Severe Heart Failure Upon Hypertension	Ragot H et al.	27296994	Non-human study
1	Knockdown of parathyroid hormone related protein in smooth muscle cells alters renal hemodynamics but not blood pressure	Raison D et al.	23720345	Non-human study

For peer review only

1	Nephron-specific deletion of the prorenin receptor causes a urine concentration defect	Ramkumar N et al.	25995108	Non-human study
1	Possible role for nephron-derived angiotensinogen in angiotensin-II dependent hypertension	Ramkumar N et al.	WOS:000380233800013	Non-human study
1	20-Hydroxyeicosatetraenoic Acid (HETE)-dependent Hypertension in Human Cytochrome P450 (CYP) 4A11 Transgenic Mice NORMALIZATION OF BLOOD PRESSURE BY SODIUM RESTRICTION, HYDROCHLOROTHIAZIDE, OR BLOCKADE OF THE TYPE 1 ANGIOTENSIN II RECEPTOR	Savas S et al.	WOS:000380826700046	Non-human study
1	Angiotensin converting enzyme (ACE) gene expression in experimentally induced liver cirrhosis in rats	Shahid SM et al.	24035938	Non-human study
1	Strain differences in angiotensin-converting enzyme and angiotensin II type I receptor expression. Possible implications for experimental chronic renal transplant failure	Smit-van Oosten A et al.	11984748	Non-human study
1	Association of a novel polymorphism in the bovine PPARGC1A gene with growth, slaughter and meat quality traits in Brangus steers	Soria LA et al.	19665052	Non-human study
1	Angiotensin II Type 1A Receptors in Vascular Smooth Muscle Cells Do Not Influence Aortic Remodeling in Hypertension	Sparks MA et al.	21242463	Non-human study
1	Thromboxane Receptors in Smooth Muscle Promote Hypertension, Vascular Remodeling, and Sudden Death	Sparks MA et al.	23150508	Non-human study
1	Losartan and Sodium Nitroprusside Effectively Protect against Renal Impairments after Ischemia and Reperfusion in Rats	Srisawat U et al.	25947921	Non-human study
1	Natriuretic Peptide Receptor Guanylyl Cyclase-A in Podocytes is Renoprotective but Dispensable for Physiologic Renal Function	Staffel J et al.	27153922	Non-human study
1	Co-operation between particulate and soluble guanylyl cyclase systems in the rat renal glomeruli	Stepinski J et al.	11016869	Non-human study
1	Renal angiotensin converting enzyme promotes renal damage during ureteral obstruction	Stoneking BJ et al.	9719278	Non-human study
1	Mediation of tubuloglomerular feedback by adenosine: Evidence from mice lacking adenosine 1 receptors	Sun DQ et al.	11504952	Non-human study

For peer review only

1	Cross-species transcriptomic analysis elucidates constitutive aryl hydrocarbon receptor activity	Sun RX et al.	25467400	Non-human study
1	Blood Pressure Control by a Secreted FGFBP1 (Fibroblast Growth Factor-Binding Protein).	Tassi E et al.	29158353	Non-human study
1	Regulation of Na+/K+-ATPase activity by nitric oxide in the kidney and gill of the brown trout (Salmo trutta)	Tipsmark CK et al.	12654889	Non-human study
1	Mechanisms of Renal. Control of Potassium Homeostasis in Complete Aldosterone Deficiency	Todkar A et al.	25071088	Non-human study
1	Functional genetic variation in aminopeptidase A (ENPEP): Lack of clear association with focal and segmental glomerulosclerosis (FSGS)	Tonna S et al.	18206321	Non-human study
1	Angiotensin II-dependent chronic hypertension and cardiac hypertrophy are unaffected by gp91phox-containing NADPH oxidase	Touyz RM et al.	15753233	Non-human study
1	Increased availability of nitric oxide leads to enhanced nitric oxide dependency of tubuloglomerular feedback in the contralateral kidney of rats with2-kidney, 1-clip Goldblatt hypertension	Turkstra E et al.	10523346	Non-human study
1	Cardiac phenotype and angiotensin II levels in AT(1a), AT(1b), and AT(2) receptor single, double, and triple knockouts	van Esch JHM et al.	20071356	Non-human study
1	Blood pressure and renal hemodynamic responses to acute angiotensin II infusion are enhanced in a female mouse model of systemic lupus erythematosus	Venegas-Pont M et al.	21900645	Non-human study
1	Compensatory up-regulation of angiotensin II subtype 1 receptors in alpha ENaC knockout heterozygous mice	Wang Q et al.	11380824	Non-human study
1	Inhibition of Nitric Oxide Synthase 1 Induces Salt-Sensitive Hypertension in Nitric Oxide Synthase 1 alpha Knockout and Wild-Type Mice	Wang XM et al.	26883268	Non-human study
1	Nebivolol treatment improves resistant arterial function and reduces ventricular hypertrophy and angiotensin II in spontaneously hypertension rats	Wang Y et al.	23263161	Non-human study

For peer review only

1	Human GRK4 gamma(142V) Variant Promotes Angiotensin II Type I Receptor-Mediated Hypertension via Renal Histone Deacetylase Type 1 Inhibition	Wang Z et al.	26667412	Non-human study
1	Role of neutral endopeptidase 24.11 in AV fistular rat model of heart failure	Wegner M et al.	8759244	Non-human study
1	Lysine-Specific Demethylase 1: An Epigenetic Regulator of Salt-Sensitive Hypertension	Williams JS et al.	22534796	Non-human study
1	Role of angiotensin-converting enzyme (ACE and ACE2) imbalance on tourniquet-induced remote kidney injury in a mouse hindlimb ischemia-reperfusion model	Yang XH et al.	22580272	Non-human study
1	Renal redox-sensitive signaling, but not blood pressure, is attenuated by Nox1 knockout in angiotensin II-dependent chronic hypertension	Yogi A et al.	18195161	Non-human study
1	Signal transduction through Ca2+/calmodulin-dependent Ras-GTPase and protein kinase II contributes to development of diabetes-induced renal vascular dysfunction	Yousif MH	16287213	Non-human study
1	Cosegregation of spontaneously hypertensive rat renin gene with elevated blood pressure in an F-2 generation	Yu H et al.	9794718	Non-human study
1	Add-on angiotensin receptor blockade with maximized ACE inhibition	Agarwal R	11380832	Not a case-control study
1	Angiotensin-converting enzyme genotype is a predictive factor in the peak panel-reactive antibody response	Akcay A et al.	15013293	Not a case-control study
1	Association of the genetic polymorphisms of the renin-angiotensin system and endothelial nitric oxide synthase with chronic renal transplant dysfunction	Akcay A et al.	15385810	Not a case-control study
1	Major clinical trials of hypertension - What should be done next?	Alderman MH et al.	WOS:000230012700001	Not a case-control study
1	Allergic reaction related to ramipril use: a case report	Alencar RC et al.	20180980	Not a case-control study

For peer review only

1	CYP3A5 and ABCB1 genes and hypertension	Bochud M et al.	19290795	Not a case-control study
1	Disorders of mineralocorticoid synthesis	Connell JMC et al.	11469810	Not a case-control study
1	Whole-Genome Linkage and Association Scan in Primary, Nonsyndromic Vesicoureteric Reflux	Cordell HJ et al.	19959718	Not a case-control study
1	Genetics of angiotensin I-converting enzyme	Costerousse O et al.	9247746	Not a case-control study
1	Renin Angiotensin System and Cytokines in Chronic Kidney Disease: Clinical and Experimental Evidence	da Silva AAS et al.	28820061	Not a case-control study
1	Association between two genetic polymorphisms of the renin-angiotensin-aldosterone system and diabetic nephropathy: a meta-analysis	Ding W et al.	21607620	Not a case-control study
1	A systematic review and meta-analysis of the association between angiotensin II type 1 receptor A1166C gene polymorphism and myocardial infarction susceptibility	Feng X et al.	23178513	Not a case-control study
1	Polymorphism of angiotensin converting enzyme, angiotensinogen, and angiotensin II type 1 receptor genes and end-stage renal failure in IgA nephropathy: IGARAS - A study of 274 men	Frimat L et al.	11053482	Not a case-control study
1	HLA genes in ANCA-associated vasculitides	Griffith ME et al.	9493788	Not a case-control study
1	Antiproteinuric effect of candesartan cilexetil in Japanese subjects with type 2 diabetes and nephropathy	Haneda M et al.	15364166	Not a case-control study
1	Nonmodulation and essential hypertension	Hollenberg NK et al.	16672145	Not a case-control study
1	Angiotensinogen genotype affects renal and adrenal responses to angiotensin II in essential hypertension	Hopkins PN et al.	11997278	Not a case-control study
1	Association between Angiotensin I-Converting Enzyme Insertion/Deletion Polymorphism and Prognosis of Kidney Transplantation: A Meta-Analysis	Huang ZK et al.	26000752	Not a case-control study

For peer review only

1	Association of angiotensinogen gene M235T and angiotensin-converting enzyme gene I/D polymorphisms with essential hypertension in Han Chinese population: a meta-analysis	Ji LD et al.	20087216	Not a case-control study
1	The role of renin-angiotensin-aldosterone system genes in the progression of chronic kidney disease: findings from the Chronic Renal Insufficiency Cohort (CRIC) study.	Kelly TN et al.	25906781	Not a case-control study
1	The Phenotypic Patterns of Essential Hypertension Are the Key to Identifying "High Blood Pressure" Genes	Korner PI	21208016	Not a case-control study
1	Glucocorticoids Reduce Aberrant O-Glycosylation of IgA1 in IgA Nephropathy Patients	Kosztyu P et al.	29529610	Not a case-control study
1	Antihypertensive treatment modulates the association between the D/I ACE gene polymorphism and left ventricular hypertrophy: a meta-analysis	Kuznetsova T et al.	10918550	Not a case-control study
1	The renoprotective effect of antihypertensive drugs	Locatelli F et al.	10048500	Not a case-control study
1	microRNAs in Essential Hypertension and Blood Pressure Regulation	Marques FZ et al.	26663185	Not a case-control study
1	Genetics and the prediction of complications in type 1 diabetes	Marre M	10097900	Not a case-control study
1	Improvement of nephrotic syndrome by intensive lipid-lowering therapy in a patient with lipoprotein glomerulopathy	Matsunaga A et al.	19603250	Not a case-control study
1	Mechanisms of plasminogen activator inhibitor 1 action in stromal remodeling and related diseases	Milenkovic J et al.	29097819	Not a case-control study
1	Angiotensinogen gene variation and renoprotective efficacy of renin-angiotensin system blockade in IgA nephropathy	Narita I et al.	12911556	Not a case-control study
1	Angiotensin-I converting enzyme insertion/deletion polymorphism and its association with diabetic nephropathy: a meta-analysis of studies reported between 1994 and 2004 and comprising 14,727 subjects	Ng D et al.	15830182	Not a case-control study

For peer review only

1	Is the presence of retinopathy of practical value in defining cases of diabetic nephropathy in genetic association studies? The experience with the ACE insertion/deletion polymorphism in 53 studies comprising 17,791 subjects	Ng DPK et al.	18523141	Not a case-control study
1	Partial Deletion of the AGXT Gene (EX1_EX7del): A New Genotype in Hyperoxaluria Type 1	Nogueira PK et al.	10737993	Not a case-control study
1	A case of rapid amelioration of hepatitis C virus-associated cryoglobulinemic membranoproliferative glomerulonephritis treated by interferon-free directly acting antivirals for HCV in the absence of immunosuppressant	Obata F et al.	28509128	Not a case-control study
1	Survival in type 2 diabetic patients in dialysis and the number of risk alleles in polymorphisms of the renin-angiotensin system genes	Padro-Miquel A et al.	19014923	Not a case-control study
1	Normative genetic profiles of RAAS pathway gene Polymorphisms in north Indian and south Indian Populations	Prasad P et al.	18027817	Not a case-control study
1	Stress, Genes, and Hypertension. Contribution of the ISIAH Rat Strain Study	Redina et al.	29909475	Not a case-control study
1	A Synergistic Association of ACE I/D and eNOS G894T Gene Variants with the Progression of Immunoglobulin A Nephropathy - A Pilot Study	Rodriguez-Perez JC et al.	19546528	Not a case-control study
1	Diabetic nephropathy is associated with AGT polymorphism T235 - Results of a family-based study	Rogus JJ et al.	9461232	Not a case-control study
1	Risk of developing diabetic nephropathy is not associated with synergism between the angiotensin II (type 1) receptor C-1166 allele and poor glycaemic control	Savage DA et al.	10328465	Not a case-control study
1	ACE gene polymorphism and IgA nephropathy: An ethnically homogeneous study and a meta-analysis	Schena FP et al.	11473656	Not a case-control study
1	Genetic determinants of diabetic renal disease and their impact on therapeutic interventions	Schmidt S et al.	WOS:A1997YJ60500008	Not a case-control study

For peer review only

1	Impact of genetic polymorphisms of the renin-angiotensin system and of non-genetic factors on kidney transplant function - a single-center experience	Siekierka-Harreis M et al.	19681973	Not a case-control study
1	The deletion/insertion polymorphism of the angiotensin converting enzyme gene and cardiovascular-renal risk	Staessen JA et al.	9488209	Not a case-control study
1	M235T angiotensinogen gene polymorphism and cardiovascular renal risk	Staessen JA et al.	10100088	Not a case-control study
1	Weber-Christian Disease Associated with the Tip Variant of Focal Segmental Glomerulosclerosis: A Case Report	Sterling KA et al.	23216240	Not a case-control study
1	Gastric-Carcinoma With Osteoclast-Like Giant-Cells - Report Of 4 Cases	Straccapansa V et al.	7726143	Not a case-control study
1	Analysis of baseline parameters in the HALT polycystic kidney disease trials.	Torres VE et al.	22205355	Not a case-control study
1	Mistyping of the human angiotensin-converting enzyme gene polymorphism: Frequency, causes and possible methods to avoid errors in typing	Ueda S et al.	8863184	Not a case-control study
1	Varicella-zoster virus (VZV) and alpha 1 antitrypsin: a fatal outcome in a patient affected by endemic pemphigus foliaceus	Velez AMA et al.	WOS:000305514500009	Not a case-control study
1	NHLBI Family Blood Pressure Program: Methodology and recruitment in the HyperGEN network	Williams RR et al.	10964005	Not a case-control study
1	Contribution of gene polymorphisms in the renin-angiotensin system to macroangiopathy in patients with diabetic nephropathy	Wong TYH et al.	11431175	Not a case-control study
1	Gene Polymorphisms of the Renin-AngiotensinAldosterone system and angiotensin 11 type I-Receptor activating antibodies in renal rejection	Zhang G et al.	17984617	Not a case-control study
1	Associations between angiotensinogen M235T polymorphisms and the risk of diabetic nephropathy: A meta-analysis	Zhou B et al.	29775675	Not a case-control study
1	Association of angiotensin II type-1 receptor A1166C gene polymorphism with the susceptibility of end-stage renal disease	Zhou TB et al.	23971628	Not a case-control study

For peer review only

1	Variants in blood pressure genes and the risk of renal cell carcinoma	Andreotti G et al.	20047954	Not a renal disease focus
1	Association of angiotensin II type 1 receptor gene A1166C polymorphism with the presence of diabetes mellitus and metabolic syndrome in patients with documented coronary artery disease	Assali A et al.	21570644	Not a renal disease focus
1	Germline genetic variations at 11q13 and 12p11 locus modulate age at onset for renal cell carcinoma.	Audenet F et al.	23911636	Not a renal disease focus
1	Angiotensinogen M235T and angiotensin-converting enzyme I/D gene polymorphism and their association with type 2 diabetes in Egypt	Badr S et al.	WOS:000309728600182	Not a renal disease focus
1	Polymorphism in angiotensin II receptor genes and hypertension	Baudin B	15640279	Not a renal disease focus
1	Analyses of mutations in the human renal kallikrein (hKLK1) gene and their possible relevance to blood pressure regulation and risk of myocardial infarction	Berge KE et al.	9298743	Not a renal disease focus
1	Single Strand Conformation Polymorphism (SSCP) as a quick and reliable method to genotype M235T polymorphism of angiotensinogen gene	Bettinaglio P et al.	12270765	Not a renal disease focus
1	Polymorphisms of the renin-angiotensin system in patients with multifocal renal arterial fibromuscular dysplasia	Bofinger A et al.	11317203	Not a renal disease focus
1	Association between plasma activities of semicarbazide-sensitive amine oxidase and angiotensin-converting enzyme in patients with type 1 diabetes mellitus.	Boomsma F et al.	15830186	Not a renal disease focus
1	Can the choice of diet undermine the potential genetic risk of AT1R 1166A>C gene polymorphism?	Bozina T et al.	30205174	Not a renal disease focus
1	Structure-Based Analysis of Single Nucleotide Variants in the Renin-Angiotensinogen Complex	Brown DK et al.	28302554	Not a renal disease focus
1	Interactions between serotonin and endogenous and exogenous noradrenaline in the human forearm.	Bruning TA et al.	7866595	Not a renal disease focus

For peer review only

1	Renin-angiotensin system gene polymorphisms: assessment of the risk of coronary heart disease.	Buraczyńska M et al.	14502296	Not a renal disease focus
1	Oxidative stress-related factors in Bartter's and Gitelman's syndromes: relevance for angiotensin II signalling.	Calo LA et al.	12897089	Not a renal disease focus
1	alpha(1)-antitrypsin (AAT) deficiency and ANCA-positive systemic vasculitis: genetic and clinical implications	Callea F et al.	9279535	Not a renal disease focus
1	Evaluation of Alpha-1 Antitrypsin Levels and SERPINA1 Gene Polymorphisms in Sickle Cell Disease	Carvalho MOS et al.	29163550	Not a renal disease focus
1	Role of GRK4 in the Regulation of Arterial AT(1) Receptor in Hypertension	Chen K et al.	24218433	Not a renal disease focus
1	A study on the association between angiotensin-I converting enzyme I/D dimorphism and type-2 diabetes mellitus.	Chmaisse HN et al.	19861867	Not a renal disease focus
1	alpha-adducin and angiotensin I-converting enzyme polymorphisms in essential hypertension	Clark CJ et al.	11116113	Not a renal disease focus
1	Elevated urinary albumin excretion is not linked to the angiotensin I-converting enzyme gene polymorphism in clinically healthy subjects	Clausen P et al.	10872702	Not a renal disease focus
1	Role of ACE inhibitors in patients with diabetes mellitus	Cordonnier DJ et al.	11708761	Not a renal disease focus
1	Molecular genetics of the renin-angiotensin-aldosterone system in human hypertension	Corvol P et al.	9296068	Not a renal disease focus
1	Angiotensin I-converting enzyme (kininase II) in cardiovascular and renal regulations and diseases	Costerousse O et al.	9830503	Not a renal disease focus
1	Functional polymorphisms in genes of the Angiotensin and Serotonin systems and risk of hypertrophic cardiomyopathy: AT1R as a potential modifier.	Coto E et al.	20594303	Not a renal disease focus
1	SNP Variants in RET and PAX2 and Their Possible Contribution to the Primary Hyperoxaluria Type 1 Phenotype	Coulter-Mackie MB	25854853	Not a renal disease focus
1	Overexpression of human alanine:glyoxylate aminotransferase in Escherichia coli: renaturation from guanidine-HCl and affinity for pyridoxal phosphate co-factor.	Coulter-Mackie MB et al.	15802217	Not a renal disease focus

For peer review only

1	The major allele of the alanine:glyoxylate aminotransferase gene: nine novel mutations and polymorphisms associated with primary hyperoxaluria type 1.	Coulter-Mackie MB et al.	15963748	Not a renal disease focus
1	Mutation-based diagnostic testing for primary hyperoxaluria type 1: survey of results.	Coulter-Mackie MB et al.	18282470	Not a renal disease focus
1	Further studies on the activity and subcellular distribution of alanine:glyoxylate aminotransferase in the livers of patients with primary hyperoxaluria type 1.	Danpure CJ et al.	3416563	Not a renal disease focus
1	Alanine : glyoxylate aminotransferase peroxisome-to-mitochondrion mistargeting in human hereditary kidney stone disease	Danpure CJ et al.	12686111	Not a renal disease focus
1	Insertion/Deletion Polymorphism of Angiotensin I-converting Enzyme Gene Is Linked With Chromophobe Renal Cell Carcinoma	de Martino M et al.	21477733	Not a renal disease focus
1	Polymorphisms in genes of the renin-angiotensin-aldosterone system and renal cell cancer risk: Interplay with hypertension and intakes of sodium, potassium and fluid	Deckers IA et al.	24978482	Not a renal disease focus
1	ACE gene insertion/deletion polymorphism modulates capillary permeability in hypertension	Dell'omo G et al.	16889537	Not a renal disease focus
1	Single nucleotide polymorphism of adiponectin +276 G/T is associated with the susceptibility to essential hypertension in a Turkish population.	Demir AK et al.	27936341	Not a renal disease focus
1	Genetic polymorphisms associated with exertional rhabdomyolysis	Deuster PA et al.	23543093	Not a renal disease focus
1	Vasodilator-derived nitric oxide inhibits fetal calf serum- and angiotensin-II-induced growth of renal arteriolar smooth muscle cells	Dubey RK et al.	8169847	Not a renal disease focus
1	The angiotensin I-converting enzyme gene insertion/deletion polymorphism is linked to early gastric cancer	Ebert MPA et al.	16365022	Not a renal disease focus
1	A new theory of essential hypertension based on analysis of the association between a polymorphism of the alpha(2)-adrenoceptor at the 10q24-q26 locus and hypertension in African-Americans	Eggers AE	26243176	Not a renal disease focus

For peer review only

1	CYP3A5 genotype is associated with elevated blood pressure.	Fromm MF et al.	16141800	Not a renal disease focus
1	Functional variant of CYP4A11 20-hydroxyeicosatetraenoic acid synthase is associated with essential hypertension	Gainer JV et al.	15611369	Not a renal disease focus
1	The interaction of AGT and NOS3 gene polymorphisms with conventional risk factors increases predisposition to hypertension	Gatti RR et al.	22791701	Not a renal disease focus
1	Somatic Von Hippel-Lindau Mutation In Clear-Cell Papillary Cystadenoma Of The Epididymis	Gilcrease MZ et al.	8522307	Not a renal disease focus
1	Phenotype-genotype analysis in two Chinese families with Liddle syndrome.	Gong L et al.	24474657	Not a renal disease focus
1	alpha(1)-Antitrypsin Deficiency in Fraternal Twins Born With Familial Spontaneous Pneumothorax	Greene DN et al.	22215832	Not a renal disease focus
1	C-antineutrophil cytoplasmic antibody positivity in vasculitis patients is associated with the Z allele of alpha-1-antitrypsin, and P-antineutrophil cytoplasmic antibody positivity with the S allele.	Griffith ME et al.	8671812	Not a renal disease focus
1	Potential Benefits of Rho-kinase Inhibition in Arterial Hypertension	Grisk O	23852615	Not a renal disease focus
1	A Computational Model of the Circulating Renin-Angiotensin System and Blood Pressure Regulation	Guillaud F et al.	20683640	Not a renal disease focus
1	The presence of PAI-1 4G/5G and ACE DD genotypes increases the risk of early-stage AVF thrombosis in hemodialysis patients.	Gungor Y et al.	21332339	Not a renal disease focus
1	Renin-aldosterone response, urinary Na/K ratio and growth in pseudohypoaldosteronism patients with mutations in epithelial sodium channel (ENaC) subunit genes	Hanukoglu A et al.	18634878	Not a renal disease focus
1	Blunted renal vascular response to angiotensin II is associated with a common Variant of the angiotensinogen gene and obesity	Hopkins PN et al.	8728297	Not a renal disease focus
1	Vitamin B6 in Primary Hyperoxaluria I: First Prospective Trial after 40 Years of Practice	Hoyer-Kuhn H et al.	24385516	Not a renal disease focus

For peer review only

1	Genetic variation of the renin-angiotensin system and chronic kidney disease progression in black individuals in the atherosclerosis risk in communities study	Hsu CCC et al.	16396964	Not a renal disease focus
1	Clinical and Genetic Factors Associated With Thiazide-Induced Hyponatremia.	Huang CC et al.	26313793	Not a renal disease focus
1	A case control association study of ACE gene polymorphism (I/D) with hypertension in Punjabi population from Faisalabad, Pakistan	Hussain M et al.	29058472	Not a renal disease focus
1	Genetic variant of the renin-angiotensin system and prevalence of type 2 diabetes mellitus: a modest but significant effect of aldosterone synthase	Ichikawa M et al.	24549414	Not a renal disease focus
1	No association between alpha-adducin 460 polymorphism and essential hypertension in a Japanese population	Ishikawa K et al.	9607391	Not a renal disease focus
1	Renin-Angiotensin System Gene Variants and Type 2 Diabetes Mellitus: Influence of Angiotensinogen	Joyce-Tan SM et al.	WOS:000370250500001	Not a renal disease focus
1	Lack of association between the alpha-adducin locus and essential hypertension in the Japanese population	Kato N et al.	9495254	Not a renal disease focus
1	Comprehensive analysis of the renin-angiotensin gene polymorphisms with relation to hypertension in the Japanese	Kato N et al.	10953993	Not a renal disease focus
1	Lys(173)Arg and -344T/C variants of CYP11B2 in Japanese patients with low-renin hypertension.	Komiya I et al.	10720581	Not a renal disease focus
1	Molecular-Biology Of Hypertension	Krieger Je et al.	WOS:A1991GF39900002	Not a renal disease focus
1	Effects of angiotensin-converting enzyme gene polymorphism and serum vitamin D levels on ambulatory blood pressure measurement and left ventricular mass in Turkish hypertensive population	Kulah E et al.	17625392	Not a renal disease focus
1	Efficacy of Korean Red Ginseng by Single Nucleotide Polymorphism in Obese Women: Randomized, Double-blind, Placebo-controlled Trial	Kwon DH et al.	23717118	Not a renal disease focus
1	Serum liver enzymes in Turner syndrome	Larizza D et al.	10664223	Not a renal disease focus

For peer review only

1	Risk given by AGT polymorphisms in inducing susceptibility to essential hypertension among isolated populations from a remote region of China: A case-control study among the isolated populations	Li Q et al.	26391364	Not a renal disease focus
1	Single-trait and multi-trait genome-wide association analyses identify novel loci for blood pressure in African-ancestry populations.	Liang J et al.	28498854	Not a renal disease focus
1	No Evidence for the Expression of Renin-Angiotensin-Aldosterone System in Otosclerotic Stapes Footplates	Liktor B et al.	23370555	Not a renal disease focus
1	Functional synergism between the most common polymorphism in human alanine:glyoxylate aminotransferase and four of the most common disease-causing mutations.	Lumb MJ et al.	10960483	Not a renal disease focus
1	Effects of organic anion transporting polypeptide IBI haplotype on pharmacokinetics of pravastatin, valsartan, and temocapril	Maeda K et al.	16678545	Not a renal disease focus
1	Low-salt diet and diuretic effect on blood pressure and organ damage	Manunta P et al.	14684671	Not a renal disease focus
1	Renal changes on hyperglycemia and angiotensin-converting enzyme in type 1 diabetes	Marre M et al.	10082486	Not a renal disease focus
1	Association of TNFRSF4 gene polymorphisms with essential hypertension	Mashimo Y et al.	18398332	Not a renal disease focus
1	Alternative transcripts of the SERPINA1 gene in alpha-1 antitrypsin deficiency	Matamala N et al.	26141700	Not a renal disease focus
1	Detection of the association between a deletion polymorphism in the gene encoding angiotensin I-converting enzyme and advanced diabetic retinopathy	Matsumoto A et al.	11106834	Not a renal disease focus
1	Association of the D allele of the angiotensin I converting enzyme polymorphism with malignant vascular injury	Mayer NJ et al.	WOS:000173608200004	Not a renal disease focus
1	Genotypic interactions of renin-angiotensin system genes with diabetes type 2 in a Tunisian population	Mehri S et al.	20580725	Not a renal disease focus

1	Association of polymorphisms of angiotensin I converting enzyme 2 with retinopathy in type 2 diabetes mellitus among Chinese individuals	Meng N et al.	25359286	Not a renal disease focus
1	The consensus-based approach for gene/enzyme replacement therapies and crystallization strategies: the case of human alanine-glyoxylate aminotransferase.	Mesa-Torres N et al.	24957194	Not a renal disease focus
1	Angiotensin II type 1 receptor gene polymorphism and the response to hyperglycemia in early type 1 diabetes	Miller JA et al.	10969844	Not a renal disease focus
1	Pharmacologic rescue of an enzyme-trafficking defect in primary hyperoxaluria 1.	Miyata N et al.	25237136	Not a renal disease focus
1	Association of Polymorphisms in Endothelial Nitric Oxide Synthesis and Renin-Angiotensin-Aldosterone System with Developing of Coronary Artery Disease in Bulgarian Patients	Mokretar K et al.	26670794	Not a renal disease focus
1	Contribution of angiotensin I converting enzyme gene polymorphism and angiotensinogen gene polymorphism to blood pressure regulation in essential hypertension.	Mondorf UF et al.	9524045	Not a renal disease focus
1	Comprehensive mutation screening in 55 probands with type 1 primary hyperoxaluria shows feasibility of a gene-based diagnosis.	Monico CG et al.	17460142	Not a renal disease focus
1	Inhibition of tissue angiotensin converting enzyme activity prevents malignant hypertension in TGR(mREN2)27.	Montgomery HE et al.	9797175	Not a renal disease focus
1	Two novel point mutations in the lecithin:cholesterol acyltransferase (LCAT) gene resulting in LCAT deficiency: LCAT (G873 deletion) and LCAT (Gly344-->Ser)	Moriyama K et al.	8656071	Not a renal disease focus
1	Independent, Marked Associations Of Alleles Of The Insulin-Receptor And Dipeptidyl Carboxypeptidase-I Genes With Essential-Hypertension	Morris BJ et al.	8104754	Not a renal disease focus
1	ANCA-associated vasculitis is linked to carriage of the Z allele of alpha(1) antitrypsin and its polymers	Morris H et al.	21821620	Not a renal disease focus
1	Is the influence of variation in the ACE gene on the prospective risk of Type 2 diabetes in middle-aged men modified by obesity?	Muthumala A et al.	17624939	Not a renal disease focus

For peer review only

1	Angiotensin converting enzyme (ACE) insertion/deletion (I/D) polymorphism, and diabetic retinopathy in subjects with IDDM and NIDDM	Nagi Dk et al.	8582133	Not a renal disease focus
1	Association between RAS gene polymorphisms (ACE I/D, AGT M235T) and Henoch-Schonlein purpura in a Turkish population	Nalbantoglu S et al.	23151617	Not a renal disease focus
1	Enhanced renal production of cyclic GMP and reduced free water clearance during sodium nitroprusside infusion in healthy man	Nielsen CB et al.	8393795	Not a renal disease focus
1	Alpha-1-proteinase inhibitor and pulmonary haemorrhage in systemic vasculitis.	O'Donoghue DJ et al.	8296629	Not a renal disease focus
1	Genetic polymorphisms of the renin-angiotensin system and atheromatous renal artery stenosis	Olivieri O et al.	10567188	Not a renal disease focus
1	Endothelial nitric oxide synthase gene/gender interactions and the renal hemodynamic response to angiotensin II	Page A et al.	16093452	Not a renal disease focus
1	Angiotensin-converting enzyme and angiotensin II receptor subtype 2 genotypes in type 1 diabetes and severe hypoglycaemia requiring emergency treatment: a case cohort study	Pedersen-Bjergaard U et al.	19820429	Not a renal disease focus
1	Deep-targeted exon sequencing reveals renal polymorphisms associate with postexercise hypotension among African Americans	Pescatello LS et al.	WOS:000387445200013	Not a renal disease focus
1	Impact of maternal angiotensinogen M235T polymorphism and angiotensin-converting enzyme insertion/deletion polymorphism on blood pressure, protein excretion and fetal outcome in pregnancy.	Pfab T et al.	17563539	Not a renal disease focus
1	Effect of ACE inhibitors and beta-blockers on homocysteine levels in essential hypertension	Poduri A et al.	18200034	Not a renal disease focus
1	The state and responsiveness of the renin-angiotensin-aldosterone system in patients with type II diabetes mellitus	Price DA et al.	10232494	Not a renal disease focus
1	Identification of mutations associated with peroxisome-to-mitochondrion mistargeting of alanine/glyoxylate aminotransferase in primary hyperoxaluria type 1.	Purdue PE et al.	1703535	Not a renal disease focus
1	The relationship between ACE/AGT gene polymorphisms and the risk of diabetic retinopathy in Chinese patients with type 2 diabetes.	Qiao YC et al.	29378484	Not a renal disease focus

For peer review only

1	Association of angiotensin-converting enzyme gene dimorphisms with severity of lupus disease.	Rabbani MA et al.	18711292	Not a renal disease focus
1	Association of angiotensinogen M235T and A(-6)G gene polymorphisms with coronary heart disease with independence of essential hypertension: the PROCAGENE study. Prospective Cardiac Gene.	Rodriquez-Perez JC et al.	11345362	Not a renal disease focus
1	Evaluation of mutation screening as a first line test for the diagnosis of the primary hyperoxalurias.	Rumsby G et al.	15327387	Not a renal disease focus
1	Angiotensin II acutely attenuates range of arterial baroreflex control of renal sympathetic nerve activity	Sanderford MG et al.	11009467	Not a renal disease focus
1	An Erythropoietin Gene Polymorphism in the Hypoxia-Responsive Element at Position 3434 Is Possibly Associated with Hypertension	Schulz EG et al.	21912181	Not a renal disease focus
1	Ubiquitin ligase gp78 increases solubility and facilitates degradation of the Z variant of alpha-1-antitrypsin.	Shen Y et al.	16979136	Not a renal disease focus
1	Association of APOE (Hha1) and ACE (I/D) gene polymorphisms with type 2 diabetes mellitus in North West India	Singh PP et al.	16621107	Not a renal disease focus
1	Renal haemodynamics are not related to genotypes in offspring of parents with essential hypertension	Skov K et al.	17083073	Not a renal disease focus
1	Association between renin-angiotensin-aldosterone system-related genes and blood pressure in a Korean population.	Song SB et al.	21342026	Not a renal disease focus
1	Angiotensin II sensitivity in nonpregnant formerly preeclamptic women and healthy parous controls	Spaanderman MEA et al.	15350256	Not a renal disease focus
1	The genetic predisposition to produce high levels of TGF-beta 1 impacts on the severity of eclampsia/pre-eclampsia	Stanczuk GA et al.	17653872	Not a renal disease focus
1	Angiotensin-converting enzyme gene I/D polymorphism in malignant hypertension	Stefansson B et al.	10855732	Not a renal disease focus
1	A Polymorphism Regulates CYP4A11 Transcriptional Activity and Is Associated With Hypertension in a Japanese Population	Sugimoto K et al.	18936345	Not a renal disease focus

For peer review only

1	Blunted cGMP response to agonists and enhanced glomerular cyclic 3',5'-nucleotide phosphodiesterase activities in experimental congestive heart failure	Supaporn T et al.	8914042	Not a renal disease focus
1	Alpha1-antitrypsin phenotypes and anti-neutrophil cytoplasmic auto-antibodies in inflammatory bowel disease.	Taddei C et al.	10563543	Not a renal disease focus
1	Angiotensin I converting enzyme gene polymorphisms in systemic lupus erythematosus: decreased prevalence of DD genotype in African American patients	Tassiulas IO et al.	9710341	Not a renal disease focus
1	Middle cerebral artery stenosis in type II diabetic Chinese patients is associated with conventional risk factors but not with polymorphisms of the renin-angiotensin system genes	Thomas GN et al.	12865608	Not a renal disease focus
1	Peripheral vascular disease in type 2 diabetic Chinese patients: associations with metabolic indices, concomitant vascular disease and genetic factors	Thomas GN et al.	14632699	Not a renal disease focus
1	Genetic predisposition to left ventricular hypertrophy and the potential involvement of cystatin-C in untreated hypertension.	Tousoulis D et al.	23479071	Not a renal disease focus
1	Genomic association analysis identifies multiple loci influencing antihypertensive response to an angiotensin II receptor blocker.	Turner ST et al.	22566498	Not a renal disease focus
1	The M235T polymorphism in theangiotensinogen gene is associated with the risk of malignant hypertension in white patients	van den Born BJH et al.	17921816	Not a renal disease focus
1	Genetic risk of atherosclerotic renal artery disease - The candidate gene approach in a renal angiography cohort	van Onna M et al.	15326089	Not a renal disease focus
1	Eprosartan modulates the reflex activation of the sympathetic nervous system in sodium restricted healthy humans	Vase H et al.	18341678	Not a renal disease focus
1	Angiotensin I-converting enzyme and angiotensinogen gene interaction and prediction of essential hypertension	Vasku A et al.	9607178	Not a renal disease focus
1	Association between ACE gene polymorphisms and Alzheimer's disease in Han population in Hebei Peninsula	Wang XL et al.	WOS:000412148800122	Not a renal disease focus
1	Genotype-phenotype analysis of angiotensinogen polymorphisms and essential hypertension: the importance of haplotypes	Watkins WS et al.	19770777	Not a renal disease focus

For peer review only

1	No association between a genetic variant of the p22(phox) component of NAD(P)H oxidase and the incidence and progression of IgA nephropathy	Wolf G et al.	12147803	Not a renal disease focus
1	Genetic polymorphisms of the angiotensin II type 1 receptor gene and diastolic heart failure	Wu CK et al.	19330904	Not a renal disease focus
1	Increased expression of angiotensin II type 1 receptor (AGTR1) in heart transplant recipients with recurrent rejection	Yamani MH et al.	17097490	Not a renal disease focus
1	G-protein beta 3 subunit gene C825T polymorphism in patients with vesico-ureteric reflux	Zagradisnik B et al.	15337465	Not a renal disease focus
1	Frequencies of variants of candidate genes in different age groups of hypertensives	Zee RY et al.	7882587	Not a renal disease focus
1	Crystal structure of alanine: Glyoxylate aminotransferase and the relationship between genotype and enzymatic phenotype in primary hyperoxaluria type 1	Zhang XX et al.	12899834	Not a renal disease focus
1	Rab1 Small GTP-Binding Protein Regulates Cell Surface Trafficking of the Human Calcium-Sensing Receptor	Zhuang XL et al.	20861236	Not a renal disease focus
1	[Renin-angiotensin system genes in chronic glomerulonephritis].	Buraczyńska M et al.	11865575	Not written in English or Spanish
1	[Association of the renin-angiotensin system gene polymorphism with nephropathy in type II diabetes].	Buraczyńska M et al.	12476891	Not written in English or Spanish
1	[Genetic predisposition to systemic complications of arterial hypertension in maintenance haemodialysis patients].	Bzoma B et al.	19112833	Not written in English or Spanish
1	[Polymorphism of gene encoding vascular angiotensin II receptor and microangiopathies in patients with insulin-dependent diabetes mellitus].	Chistiakov DA et al.	10576062	Not written in English or Spanish
1	[Arterial hypertension and chronic hemodialysis].	Ermolenko VM et al.	7700	Not written in English or Spanish
1	[Is PstI polymorphism of the angiotensin I converting enzyme gene associated with nephropathy development in non-insulin-dependent diabetes mellitus (preliminary study)].	Grzeszczak W et al.	9499204	Not written in English or Spanish

1	[Effect of eprosartan on the hemostatic system in patients with chronic kidney disease associated with hereditary thrombophilia].	Kaliuzhin VV et al.	24261234	Not written in English or Spanish
1	[Polymorphism studies of angiotensin converting enzyme gene in chronic glomerulonephritis].	Kutyrina IM et al.	10420452	Not written in English or Spanish
1	[Relationship between serum angiotensin I-converting enzyme activity and diabetic nephropathy in patients with type II diabetes].	Liao L et al.	12016801	Not written in English or Spanish
1	[Relationship between I/D polymorphism of angiotensin I converting enzyme gene and microvascular complications in type 2 diabetic patients].	Moleda P et al.	17941464	Not written in English or Spanish
1	[Pharmacogenetic aspects of candesartan application for the treatment of arterial hypertension in patients with chronic pyelonephritis].	Mormol' IA	25286611	Not written in English or Spanish
1	[A study on angiotensin-I converting enzyme polymorphism in CAPD patients].	Nishina M	9014479	Not written in English or Spanish
1	[Identification of the locus associated with diabetic nephropathy in type 1 diabetes mellitus].	Savost'ianov KV et al.	12500539	Not written in English or Spanish
1	[Relationships of angiotensinogen gene M235T variant with diabetic nephropathy in Chinese type 2 diabetes mellitus].	Wang J et al.	10514536	Not written in English or Spanish
1	[Association between angiotensin-II receptor gene type I polymorphism and diabetic nephropathy in type 2 diabetes mellitus].	Xue Y et al.	11798574	Not written in English or Spanish
1	[AGTR1 A1166C polymorphism is associated with risk of diabetic nephropathy].	Yin X et al.	23505107	Not written in English or Spanish
1	Posterior urethral valves: Preliminary observations on the significance of plasma renin activity as a prognostic marker	Bajpai M et al.	15643266	Paediatric Individuals
1	Donor and recipient ACE I/D genotype are associated with loss of renal function in children following renal transplantation	Buscher R et al.	21309964	Paediatric Individuals
1	Polymorphisms of the angiotensin converting enzyme and angiotensin II type 1 receptor genes and renal scarring in non-uropathic children with recurrent urinary tract infection	Ece A et al.	16109085	Paediatric Individuals

For peer review only

1	Genetic polymorphism of ACE and the angiotensin II type1 receptor genes in children with chronic kidney disease	Elshamaa MF et al.	21859496	Paediatric Individuals
1	ACE gene polymorphism in Egyptian children with idiopathic nephrotic syndrome	Fahmy ME et al.	18792483	Paediatric Individuals
1	Identification of a novel splice site mutation of CLCN5 gene and characterization of a new alternative 5 ' UTR end of ClC-5 mRNA in human renal tissue and leukocytes	Forino M et al.	14673707	Paediatric Individuals
1	Genetic polymorphisms of the renin-angiotensin system and the outcome of focal segmental glomerulosclerosis in children	Frishberg Y et al.	9853248	Paediatric Individuals
1	Angiotensinogen gene T235 variant: a marker for the development of persistent microalbuminuria in children and adolescents with type 1 diabetes mellitus	Gallego PH et al.	18413222	Paediatric Individuals
1	Autosomal dominant pseudohypoaldosteronism type 1: Mechanisms, evidence for neonatal lethality, and phenotypic expression in adults	Geller DS et al.	16611713	Paediatric Individuals
1	Implication of genetic variations in congenital obstructive nephropathy	Hahn H et al.	16133060	Paediatric Individuals
1	Variants of alpha(1)-proteinase inhibitor in black and white South African patients with focal glomerulosclerosis and minimal change nephrotic syndrome	Halkas AC et al.	9475086	Paediatric Individuals
1	Angiotensin-converting enzyme insertion/deletion gene polymorphism in Egyptian children with systemic lupus erythematosus: a possible relation to proliferative nephritis.	Hammad A et al.	27956582	Paediatric Individuals
1	ACE gene polymorphism and renal scarring in primary vesicoureteric reflux	Haszon I et al.	12478352	Paediatric Individuals
1	Angiotensin type 2 receptor is important in the normal development of the ureter	Hohenfellner K et al.	10353402	Paediatric Individuals
1	Impact of common functional polymorphisms in renin angiotensin system genes on the risk of renal parenchymal scarring following childhood urinary tract infection	Hussein A et al.	25939993	Paediatric Individuals

For peer review only

1	Gene Polymorphisms of Adducin GLY460TRP, ACE I/D, and AGT M235T in Pediatric Hypertension Patients	Kaplan I et al.	25262176	Paediatric Individuals
1	ACE and AT1 receptor gene polymorphisms and renal scarring in urinary bladder dysfunction	Kostic M et al.	15179569	Paediatric Individuals
1	Renal tubular dysgenesis, a not uncommon autosomal recessive disorder leading to oligohydramnios: Role of the Renin-Angiotensin system.	Lacoste M et al.	16790508	Paediatric Individuals
1	Hemorrhagic shock and encephalopathy: clinical, pathologic, and biochemical features.	Levin M et al.	2783733	Paediatric Individuals
1	Renin-angiotensin system gene polymorphisms in children with Henoch-Schonlein purpura in West China	Liu DS et al.	WOS:000284763300006	Paediatric Individuals
1	Renin-angiotensin system polymorphisms in Taiwanese primary vesicoureteral reflux	Liu KP et al.	15045574	Paediatric Individuals
1	Polymorphisms of renin-angiotensin system genes in childhood IgA nephropathy	Maruyama K et al.	11354780	Paediatric Individuals
1	Angiotensin-Converting Enzyme Gene Polymorphism in Children with Idiopathic Nephrotic Syndrome, Effect on Biopsy Findings.	Monajemzadeh M et al.	28481137	Paediatric Individuals
1	A-20C angiotensinogen gene polymorphism and proteinuria in childhood IgA nephropathy	Nakanishi K et al.	14648325	Paediatric Individuals
1	Implications of certain genetic polymorphisms in scarring in vesicoureteric reflux: importance of ACE polymorphism.	Ozen S et al.	10401028	Paediatric Individuals
1	Renin-angiotensin system gene polymorphisms: association with susceptibility to Henoch-Schonlein purpura and renal involvement	Ozkaya O et al.	16521052	Paediatric Individuals
1	Renin-angiotensin gene polymorphism in children with uremia and essential hypertension	Papp F et al.	12579405	Paediatric Individuals
1	Renin-angiotensin system polymorphisms and renal scarring	Pardo R et al.	12579398	Paediatric Individuals
1	Angiotensin I-converting enzyme-gene-polymorphism: Relationship to albumin excretion and blood pressure in pediatric patients with type-I-diabetes mellitus	Pavlovic M et al.	9354852	Paediatric Individuals

For peer review only

1	Angiotensin I converting enzyme and angiotensinogen gene polymorphisms related to 24-h blood pressure in paediatric type I diabetes mellitus	Pavlovic M et al.	9950302	Paediatric Individuals
1	Low renin-angiotensin system activity gene polymorphism and dysplasia associated with posterior urethral valves	Peruzzi L et al.	16006956	Paediatric Individuals
1	Angiotensin-converting enzyme and angiotensin type 2 receptor gene genotype distributions in Italian children with congenital uropathies	Rigoli L et al.	15470205	Paediatric Individuals
1	Angiotensin II type 2 receptor gene polymorphism in Caucasian children with a wide spectrum of congenital anomalies of the kidney and urinary tract	Siomou E et al.	17515833	Paediatric Individuals
1	Effects of genetic polymorphisms of the renin-angiotensin system in children with nephrotic syndrome	Tabel Y et al.	16525944	Paediatric Individuals
1	ACE gene polymorphism in childhood IgA nephropathy: Association with clinicopathologic findings	Tanaka R et al.	9590186	Paediatric Individuals
1	Genetic risk factors in typical haemolytic uraemic syndrome	Taranta A et al.	19110485	Paediatric Individuals
1	The juxtaglomerular apparatus in Bartter's syndrome and related tubulopathies. An immunocytochemical and electron microscopic study.	Taugner R et al.	3128915	Paediatric Individuals
1	Genetic polymorphism of the renin-angiotensin system on the development of primary vesicoureteral reflux	Yim HE et al.	14764974	Paediatric Individuals
1	Genetic control of VEGF and TGF-beta 1 gene polymorphisms in childhood urinary tract infection and vesicoureteral reflux	Yim HE et al.	17597658	Paediatric Individuals
1	Estimation of the relationship between the polymorphisms of selected genes: ACE, AGTR1, TGF beta 1 and GNB3 with the occurrence of primary vesicoureteral reflux	Zyczkowski et al.	27988909	Paediatric Individuals
1	Randomized Controlled Trial: Lisinopril Reduces Proteinuria, Ammonia, and Renal Polypeptide Tubular Catabolism in Patients With Chronic Allograft Nephropathy	Amara AB et al.	20061926	Pharmaceutical drug focus

For peer review only

1	Long-term renoprotective effects of losartan in diabetic nephropathy: interaction with ACE insertion/deletion genotype?	Andersen S et al.	12716812	Pharmaceutical drug focus
1	Effects of losartan and enalapril on high-sensitivity C-reactive protein and total antioxidant in renal transplant recipients with renin-angiotensin system polymorphisms	Argani H et al.	18261537	Pharmaceutical drug focus
1	Efficacy and safety of the angiotensin II receptor blocker losartan for hypertrophic cardiomyopathy: the INHERIT randomised, double-blind, placebo-controlled trial	Axelsson A et al.	25533774	Pharmaceutical drug focus
1	Angiotensin I - Converting enzyme gene polymorphism modulates the consequences of in utero growth retardation on plasma insulin in young adults	Cambien F et al.	9519756	Pharmaceutical drug focus
1	Losartan decreases plasma levels of TGF-beta 1 in transplant patients with chronic allograft nephropathy	Campistol JM et al.	10432413	Pharmaceutical drug focus
1	High serum enalaprilat in chronic renal failure	Elung-Jensen et al.	11881130	Pharmaceutical drug focus
1	The influence of renin-angiotensin system genotypes on the antiproteinuric response to high doses of olmesartan in non-diabetic protein uric nephropathies	Goyache-Goni B et al.	24241364	Pharmaceutical drug focus
1	Renal implications of angiotensin receptor blockers	Hollenberg NK	11459212	Pharmaceutical drug focus
1	CYP2C9 genotype and pharmacodynamic responses to losartan in patients with primary and secondary kidney diseases.	Joy MS et al.	19669737	Pharmaceutical drug focus
1	Association of angiotensinogen gene polymorphism with erythropoietin-induced hypertension: a preliminary report	Kuriyama S et al.	11675943	Pharmaceutical drug focus
1	The influence of the ACE (I/D) polymorphism on systemic and renal vascular responses to angiotensins in normotensive, normoalbuminuric Type 1 diabetes mellitus	Luik PT et al.	12856080	Pharmaceutical drug focus
1	Renin-angiotensin system polymorphisms and hemoglobin level in renal allografts: A comparative study between losartan and enalapril	Noroozianavval M et al.	17524880	Pharmaceutical drug focus
1	ACE gene polymorphism and losartan treatment in type 2 diabetic patients with nephropathy	Parving HH et al.	18199798	Pharmaceutical drug focus

For peer review only

1	Enalapril and losartan affect lipid peroxidation in renal transplant recipients with renin-angiotensin system polymorphisms	Rashtchizadeh N et al.	17222813	Pharmaceutical drug focus
1	Exaggerated natriuresis during clamping of systemic NO supply in healthy young men	Simonsen JA et al.	21749320	Pharmaceutical drug focus
2	Influence of genetic polymorphisms of the renin-angiotensin system on IgA nephropathy	Bantis C et al.	15031629	No data
2	Genetic polymorphisms of renin-angiotensin system and progression of interstitial nephritis.	Buraczyńska M et al.	12898858	No data
2	Polymorphism of the angiotensinogen gene and genetic predisposition to diabetic nephropathy in diabetes mellitus type 1	Chistyakov DA et al.	WOS:000079798700011	No data
2	U.K. Prospective Diabetes Study. XV: Relationship of renin-angiotensin system gene polymorphisms with microalbuminuria in NIDDM.	Dudley CR et al.	8587251	No data
2	Angiotensin I-converting enzyme and angiotensinogen gene polymorphisms in non-insulin-dependent diabetes mellitus. Lack of relationship with diabetic nephropathy and retinopathy in a Caucasian Mediterranean population.	Gutierrez C et al.	9258285	No data
2	Effects of the genetic polymorphisms of the renin-angiotensin system on focal segmental glomerulosclerosis.	Luther Y et al.	14610337	No data
2	The renin--angiotensin system gene polymorphisms and clinicopathological correlations in IgA nephropathy.	Ong-Ajyooth S et al.	10511770	No data
2	Combinational effect of genes for the renin-angiotensin system in conferring susceptibility to diabetic nephropathy.	Osawa N et al.	17143591	No data
2	The relationship between genetic and haemodynamic factors in diabetic nephropathy (DN): Case-control study in type 1 diabetes mellitus (T1DM)	Shestakova MV et al.	WOS:000242410600010	No data
2	Relationship between angiotensinogen gene M235T variant with diabetic nephropathy in Chinese NIDDM	Wang JJ et al.	11717948	No data
2	Lack of association of angiotensin-converting enzyme (DID/II) and angiotensinogen M235T gene polymorphism with renal function among Chinese patients with type II diabetes	Wong TYH et al.	10352194	No data

For peer review only

2	Polymorphism of renin-angiotensin system genes in IgA nephropathy	Woo KT et al.	15504143	No data
3	ACE variants interact with the RAS pathway to confer risk and protection against type 2 diabetic nephropathy.	Ahluwalia TS et al.	19108684	< 3 populations reported per SNP
3	Analysis of polymorphism in renin angiotensin system and other related genes in South Indian chronic kidney disease patients.	Anbazhagan K et al.	19520069	< 3 populations reported per SNP
3	Polymorphism of the renin–angiotensin–aldosterone system in patients with chronic allograft dysfunction	Ayed K et al.	16635753	< 3 populations reported per SNP
3	Renin-angiotensin-aldosterone system related gene polymorphisms and urinary total arsenic is related to chronic kidney disease	Chen WJ et al.	24907556	< 3 populations reported per SNP
3	Hypertension after renal transplantation and polymorphism of genes involved in essential hypertension: ACE, AGT, AT1R and ecNOS	El-Essawy AB et al.	11926202	< 3 populations reported per SNP
3	Genetic polymorphisms of the renin-angiotensin-aldosterone system and renal insufficiency in essential hypertension.	Fabris B et al.	15662219	< 3 populations reported per SNP
3	Angiotensinogen gene polymorphisms and progression of chronic kidney disease in ADPKD patients	Gnanasambandan R et al.	26482465	< 3 populations reported per SNP
3	Angiotensinogen and angiotensin II type 1 receptor gene polymorphism in patients with autosomal dominant polycystic kidney disease: effect on hypertension and ESRD.	Lee KB et al.	12950120	< 3 populations reported per SNP
3	Genes involved in the regulation of vascular homeostasis determine renal survival rate in patients with chronic glomerulonephritis	Litovkina O et al.	24727057	< 3 populations reported per SNP
3	Contribution of genetic polymorphism in the renin-angiotensin system to the development of renal complications in insulin-dependent diabetes	Marre M et al.	9120002	< 3 populations reported per SNP
3	Genetic polymorphism of renin-angiotensin system is not associated with diabetic vascular complications in Japanese subjects with long-term insulin dependent diabetes mellitus.	Miura J et al.	10499884	< 3 populations reported per SNP
3	Renin-angiotensin-aldosterone system genotypes and haplotypes affect the susceptibility to nephropathy in type 2 diabetes patients	Mtiraoui N et al.	21421655	< 3 populations reported per SNP

For peer review only

3	Renin-angiotensin system gene polymorphisms predict the progression to renal insufficiency among Asians with lupus nephritis	Parsa A et al.	15789057	< 3 populations reported per SNP
3	Association of aldosterone synthase (CYP11B2) gene -344T/C polymorphism with the risk of primary chronic glomerulonephritis in the Polish population.	Pawlik M et al.	23681285	< 3 populations reported per SNP
3	Association of angiotensinogen gene T235 variant with progression of immunoglobin A nephropathy in Caucasian patients	Pei Y et al.	9259580	< 3 populations reported per SNP
3	Chronic renal insufficiency among Asian Indians with type 2 diabetes: I. Role of RAAS gene polymorphisms.	Prasad P et al.	16672053	< 3 populations reported per SNP
3	Angiotensinogen and plasminogen activator inhibitor-1 gene polymorphism in relation to renovascular disease.	Reis KA et al.	16228848	< 3 populations reported per SNP
3	M235T Polymorphism in the AGT Gene and A/GI8-83 Substitution in the REN Gene Correlate with End-Stage Renal Disease	Sarkar S et al.	25660845	< 3 populations reported per SNP
3	Genetic variants of ACE (Insertion/Deletion) and AGT (M268T) genes in patients with diabetes and nephropathy	Shaikh R et al.	24737640	< 3 populations reported per SNP
3	Polymorphisms of the renin-angiotensin system genes in Brazilian patients with lupus nephropathy.	Sprovieri SR et al.	15934435	< 3 populations reported per SNP
3	Interaction between gene polymorphisms of nitric oxide synthase and renin-angiotensin system in the progression of membranous glomerulonephritis	Stratta P et al.	14767013	< 3 populations reported per SNP
3	Gene polymorphisms of angiotensin-converting enzyme and angiotensin II type 1 receptor among chronic kidney disease patients in a Chinese population.	Su SL et al.	22147663	< 3 populations reported per SNP
3	Genetic polymorphisms and the risk of progressive renal failure in elderly Hungarian patients.	Zsom M et al.	22111818	< 3 populations reported per SNP

For peer review only

Supplementary Table S3d: Excluded studies from the *AGTR1* search

*Exclusion stage relates to the stage at which the article was removed as shown in the flow diagram (Supplementary Figure S1d)

Exclusion Stage	Title	Authors	Pubmed ID or WoS ID if Pubmed ID not available	Reason
1	Identification of potential candidate genes for hypertensive nephropathy based on gene expression profile	Chen Z et al.	27756246	Gene expression based study
1	Polymorphism in IgA nephropathy	Liu ZH et al.	WOS:A1997WW80600013	No data for AGTR1
1	The effect of angiotensin receptor blockade ARB on the regression of left ventricular hypertrophy in hemodialysis patients: comparison between patients with D allele and non-D allele ACE gene polymorphism.	Nakayama M et al.	16312263	No data for AGTR1
1	Angiotensin II receptor type 1 A1166C modifies the association between angiotensinogen M235T and chronic kidney disease	Su SL et al.	29296205	No data for AGTR1
1	Specific pregnancy-induced angiotensin II type-1 receptor expression in ovine uterine artery does not involve formation of alternate splice variants or alternate promoter usage	Bird IM et al.	9687288	Non-human study
1	CCN1 expression in interleukin-6 deficient mouse kidney in experimental model of heart failure	Bonda TA et al.	23690222	Non-human study
1	The genetic deletion of Mas abolishes salt induced hypertension in mice	Heringer-Walther S et al.	22652430	Non-human study
1	The angiotensin type II receptor tonically inhibits angiotensin-converting enzyme in AT2 null mutant mice	Hunley TE et al.	10652034	Non-human study
1	Collecting Duct Nitric Oxide Synthase 1 beta Activation Maintains Sodium Homeostasis During High Sodium Intake Through Suppression of Aldosterone and Renal Angiotensin II Pathways	Hyndman KA et al.	29066445	Non-human study
1	Physiological impact of increased expression of the AT(1) angiotensin receptor	Le TH et al.	12963678	Non-human study

For peer review only

1	Angiotensin receptor-binding protein ATRAP/Agtrap inhibits metabolic dysfunction with visceral obesity.	Maeda A et al.	23902639	Non-human study
1	A novel rodent model of pregnancy complications associated with genetically determined angiotensin-converting enzyme (ACE) activity	Mata-Greenwood E et al.	29360395	Non-human study
1	Losartan and Sodium Nitroprusside Effectively Protect against Renal Impairments after Ischemia and Reperfusion in Rats	Srisawat U et al.	25947921	Non-human study
1	Blood Pressure Control by a Secreted FGFBP1 (Fibroblast Growth Factor-Binding Protein).	Tassi E et al.	29158353	Non-human study
1	Mechanisms of Renal. Control of Potassium Homeostasis in Complete Aldosterone Deficiency	Todkar A et al.	25071088	Non-human study
1	Cardiac phenotype and angiotensin II levels in AT(1a), AT(1b), and AT(2) receptor single, double, and triple knockouts	van Esch JHM et al.	20071356	Non-human study
1	Human GRK4 gamma(142V) Variant Promotes Angiotensin II Type I Receptor-Mediated Hypertension via Renal Histone Deacetylase Type 1 Inhibition	Wang Z et al.	26667412	Non-human study
1	Add-on angiotensin receptor blockade with maximized ACE inhibition	Agarwal R	11380832	Not a case-control study
1	Association of the genetic polymorphisms of the renin-angiotensin system and endothelial nitric oxide synthase with chronic renal transplant dysfunction.	Akcay A et al.	15385810	Not a case-control study
1	Influence of genetic polymorphisms of the renin-angiotensin system on IgA nephropathy	Bantis C et al.	15031629	Not a case-control study
1	Genetic determinants of acute renal damage risk and prognosis: a systematic review	Cardinal-Fernandez P et al.	22436318	Not a case-control study
1	Association between two genetic polymorphisms of the renin-angiotensin-aldosterone system and diabetic nephropathy: a meta-analysis	Ding W et al.	21607620	Not a case-control study
1	A systematic review and meta-analysis of the association between angiotensin II type 1 receptor A1166C gene polymorphism and myocardial infarction susceptibility	Feng X et al.	23178513	Not a case-control study

For peer review only

1	Polymorphism of angiotensin converting enzyme, angiotensinogen, and angiotensin II type 1 receptor genes and end-stage renal failure in IgA nephropathy: IGARAS - A study of 274 men	Frimat L et al.	11053482	Not a case-control study
1	Survival in type 2 diabetic patients in dialysis and the number of risk alleles in polymorphisms of the renin-angiotensin system genes	Padro-Miquel A et al.	19014923	Not a case-control study
1	A Synergistic Association of ACE I/D and eNOS G894T Gene Variants with the Progression of Immunoglobulin A Nephropathy - A Pilot Study	Rodriguez-Perez JC et al.	19546528	Not a case-control study
1	Impact of genetic polymorphisms of the renin-angiotensin system and of non-genetic factors on kidney transplant function - a single-center experience	Siekierka-Harreis M et al.	19681973	Not a case-control study
1	Analysis of baseline parameters in the HALT polycystic kidney disease trials.	Torres VE et al.	22205355	Not a case-control study
1	Angiotensin-converting enzyme inhibitor versus angiotensin 2 receptor antagonist therapy and the influence of angiotensin-converting enzyme gene polymorphism in IgA nephritis.	Woo KT et al.	18536822	Not a case-control study
1	Gene Polymorphisms of the Renin-AngiotensinAldosterone system and angiotensin 11 type I-Receptor activating antibodies in renal rejection	Zhang G et al.	17984617	Not a case-control study
1	Association of angiotensin II type-1 receptor A1166C gene polymorphism with the susceptibility of end-stage renal disease	Zhou TB et al.	23971628	Not a case-control study
1	Association of angiotensin II type 1 receptor gene A1166C polymorphism with the presence of diabetes mellitus and metabolic syndrome in patients with documented coronary artery disease	Assali A et al.	21570644	Not a renal disease focus
1	Polymorphism in angiotensin II receptor genes and hypertension	Baudin B	15640279	Not a renal disease focus
1	Polymorphisms of the renin-angiotensin system in patients with multifocal renal arterial fibromuscular dysplasia	Bofinger A et al.	11317203	Not a renal disease focus
1	Can the choice of diet undermine the potential genetic risk of AT1R 1166A>C gene polymorphism?	Bozina T et al.	30205174	Not a renal disease focus
1	Renin-angiotensin system gene polymorphisms: assessment of the risk of coronary heart disease.	Buraczynska M et al.	14502296	Not a renal disease focus

1	Association of renin-angiotensin and endothelial nitric oxide synthase gene polymorphisms with blood pressure progression and incident hypertension: prospective cohort study.	Conen D et al.	18698212	Not a renal disease focus
1	Functional polymorphisms in genes of the Angiotensin and Serotonin systems and risk of hypertrophic cardiomyopathy: AT1R as a potential modifier.	Coto E et al.	20594303	Not a renal disease focus
1	Polymorphisms in genes of the renin-angiotensin-aldosterone system and renal cell cancer risk: Interplay with hypertension and intakes of sodium, potassium and fluid	Deckers IA et al.	24978482	Not a renal disease focus
1	Single nucleotide polymorphism of adiponectin +276 G/T is associated with the susceptibility to essential hypertension in a Turkish population.	Demir AK et al.	27936341	Not a renal disease focus
1	Genetic variation of the renin-angiotensin system and chronic kidney disease progression in black individuals in the atherosclerosis risk in communities study	Hsu CCC et al.	16396964	Not a renal disease focus
1	Genetic variant of the renin-angiotensin system and prevalence of type 2 diabetes mellitus: a modest but significant effect of aldosterone synthase	Ichikawa M et al.	24549414	Not a renal disease focus
1	Renin-Angiotensin System Gene Variants and Type 2 Diabetes Mellitus: Influence of Angiotensinogen	Joyce-Tan SM et al.	26682227	Not a renal disease focus
1	Comprehensive analysis of the renin-angiotensin gene polymorphisms with relation to hypertension in the Japanese	Kato N et al.	10953993	Not a renal disease focus
1	Renin-angiotensin system gene polymorphisms: its impact on IgAN and its progression to end-stage renal failure among Chinese in Singapore.	Lau YK et al.	15153745	Not a renal disease focus
1	Angiotensin II type 1 receptor gene polymorphism and the response to hyperglycemia in early type 1 diabetes	Miller JA et al.	10969844	Not a renal disease focus
1	Deep-targeted exon sequencing reveals renal polymorphisms associate with postexercise hypotension among African Americans	Pescatello LS et al.	27940662	Not a renal disease focus
1	Association between renin-angiotensin-aldosterone system-related genes and blood pressure in a Korean population.	Song SB et al.	21342026	Not a renal disease focus
1	Angiotensin II Type 1A Receptors in Vascular Smooth Muscle Cells Do Not Influence Aortic Remodeling in Hypertension	Sparks MA et al.	21242463	Not a renal disease focus

For peer review only

1	Peripheral vascular disease in type 2 diabetic Chinese patients: associations with metabolic indices, concomitant vascular disease and genetic factors	Thomas GN et al.	14632699	Not a renal disease focus
1	Genomic association analysis identifies multiple loci influencing antihypertensive response to an angiotensin II receptor blocker.	Turner ST et al.	22566498	Not a renal disease focus
1	The M235T polymorphism in theangiotensinogen gene is associated with the risk of malignant hypertension in white patients	van den Born BJH et al.	17921816	Not a renal disease focus
1	Genetic risk of atherosclerotic renal artery disease - The candidate gene approach in a renal angiography cohort	van Onna M et al.	15326089	Not a renal disease focus
1	Eprosartan modulates the reflex activation of the sympathetic nervous system in sodium restricted healthy humans	Vase H et al.	18341678	Not a renal disease focus
1	Genetic polymorphisms of the angiotensin II type 1 receptor gene and diastolic heart failure	Wu CK et al.	19330904	Not a renal disease focus
1	Increased expression of angiotensin II type 1 receptor (AGTR1) in heart transplant recipients with recurrent rejection	Yamani MH et al.	17097490	Not a renal disease focus
1	[Renin-angiotensin system genes in chronic glomerulonephritis].	Buraczynska M et al.	11865575	Not written in English or Spanish
1	[Association of the renin-angiotensin system gene polymorphism with nephropathy in type II diabetes].	Buraczynska M et al.	12476891	Not written in English or Spanish
1	[Genetic predisposition to systemic complications of arterial hypertension in maintenance haemodialysis patients].	Bzoma B et al.	19112833	Not written in English or Spanish
1	[Polymorphism of gene encoding vascular angiotensin II receptor and microangiopathies in patients with insulin-dependent diabetes mellitus].	Chistiakov DA et al.	10576062	Not written in English or Spanish
1	[Effect of eprosartan on the hemostatic system in patients with chronic kidney disease associated with hereditary thrombophilia].	Kaliuzhin VV et al.	24261234	Not written in English or Spanish

For peer review only

1	[Pharmacogenetic aspects of candesartan application for the treatment of arterial hypertension in patients with chronic pyelonephritis].	Mormol' IA	25286611	Not written in English or Spanish
1	[Identification of the locus associated with diabetic nephropathy in type 1 diabetes mellitus].	Savost'ianov KV et al.	12500539	Not written in English or Spanish
1	[AGTR1 A1166C polymorphism is associated with risk of diabetic nephropathy].	Yin X et al.	23505107	Not written in English or Spanish
1	Donor and recipient ACE I/D genotype are associated with loss of renal function in children following renal transplantation	Buscher R et al.	21309964	Paediatric Individuals
1	Polymorphisms of the angiotensin converting enzyme and angiotensin II type 1 receptor genes and renal scarring in non-uropathic children with recurrent urinary tract infection	Ece A et al.	16109085	Paediatric Individuals
1	Cyclosporine A responsive congenital nephrotic syndrome with single heterozygous variants in NPHS1, NPHS2, and PLCE1	Eichinger A et al.	29663071	Paediatric Individuals
1	Genetic polymorphism of ACE and the angiotensin II type1 receptor genes in children with chronic kidney disease	Elshamaa MF et al.	21859496	Paediatric Individuals
1	Genetic polymorphisms of the renin-angiotensin system and the outcome of focal segmental glomerulosclerosis in children	Frishberg Y et al.	9853248	Paediatric Individuals
1	Angiotensinogen gene T235 variant: a marker for the development of persistent microalbuminuria in children and adolescents with type 1 diabetes mellitus	Gallego PH et al.	18413222	Paediatric Individuals
1	ACE gene polymorphism and renal scarring in primary vesicoureteric reflux	Haszon I et al.	12478352	Paediatric Individuals
1	Angiotensin type 2 receptor is important in the normal development of the ureter	Hohenfellner K et al.	10353402	Paediatric Individuals
1	Impact of common functional polymorphisms in renin angiotensin system genes on the risk of renal parenchymal scarring following childhood urinary tract infection	Hussein A et al.	25939993	Paediatric Individuals
1	ACE and AT1 receptor gene polymorphisms and renal scarring in urinary bladder dysfunction	Kostic M et al.	15179569	Paediatric Individuals

For peer review only

1	Renin-angiotensin system polymorphisms in Taiwanese primary vesicoureteral reflux	Liu KP et al.	15045574	Paediatric Individuals
1	Polymorphisms of renin-angiotensin system genes in childhood IgA nephropathy	Maruyama K et al.	11354780	Paediatric Individuals
1	Implications of certain genetic polymorphisms in scarring in vesicoureteric reflux: importance of ACE polymorphism.	Ozen S et al.	10401028	Paediatric Individuals
1	Renin-angiotensin system gene polymorphisms: association with susceptibility to Henoch-Schonlein purpura and renal involvement	Ozkaya O et al.	16521052	Paediatric Individuals
1	Renin-angiotensin gene polymorphism in children with uremia and essential hypertension	Papp F et al.	12579405	Paediatric Individuals
1	Renin-angiotensin system polymorphisms and renal scarring	Pardo R et al.	12579398	Paediatric Individuals
1	Effects of genetic polymorphisms of the renin-angiotensin system in children with nephrotic syndrome.	Tabel Y et al.	16525944	Paediatric Individuals
1	Genetic risk factors in typical haemolytic uraemic syndrome	Taranta A et al.	19110485	Paediatric Individuals
1	Mapping candidate regions and genes for congenital anomalies of the kidneys and urinary tract (CAKUT) by array-based comparative genomic hybridization	Weber S et al.	20605837	Paediatric Individuals
1	Genetic polymorphism of the renin-angiotensin system on the development of primary vesicoureteral reflux	Yim HE et al.	14764974	Paediatric Individuals
1	Estimation of the relationship between the polymorphisms of selected genes: ACE, AGTR1, TGFÎ²1 and GNB3 with the occurrence of primary vesicoureteral reflux.	Zyczkowski M et al.	27988909	Paediatric Individuals
1	Long-term renoprotective effects of losartan in diabetic nephropathy: interaction with ACE insertion/deletion genotype?	Andersen S et al.	12716812	Pharmaceutical drug focus
1	Effect of ACE and AT-2 inhibitors on mortality and progression to microalbuminuria in a nested case-control study of diabetic nephropathy in diabetes mellitus type 2: results from the GENDIAN study.	Boger CA et al.	16961167	Pharmaceutical drug focus
1	Genetic polymorphisms of renin-angiotensin system and progression of interstitial nephritis.	Buraczynska M et al.	12898858	Pharmaceutical drug focus

For peer review only

1	The influence of renin-angiotensin system genotypes on the antiproteinuric response to high doses of olmesartan in non-diabetic protein uric nephropathies	Goyache-Goni B et al.	24241364	Pharmaceutical drug focus
1	CYP2C9 genotype and pharmacodynamic responses to losartan in patients with primary and secondary kidney diseases.	Joy MS et al.	19669737	Pharmaceutical drug focus
1	Genotypic interactions of renin-angiotensin system genes with diabetes type 2 in a Tunisian population	Mehri S et al.	20580725	Pharmaceutical drug focus
1	Evaluation of Candidate Nephropathy Susceptibility Genes in a Genome-Wide Association Study of African American Diabetic Kidney Disease	Palmer ND et al.	24551085	Pharmaceutical drug focus
1	Chronic renal insufficiency among Asian Indians with type 2 diabetes: I. Role of RAAS gene polymorphisms.	Prasad P et al.	16672053	Pharmaceutical drug focus
1	Enalapril and losartan affect lipid peroxidation in renal transplant recipients with renin-angiotensin system polymorphisms	Rashtchizadeh N et al.	17222813	Pharmaceutical drug focus
2	A polymorphism in the angiotensin II type 1 receptor gene has different effects on the risk of diabetic nephropathy in men and women	Möllsten A et al.	21316998	Incorrect patient group
2	Effects of the genetic polymorphisms of the renin-angiotensin system on focal segmental glomerulosclerosis	Luther Y et al.	14610337	No data
2	The renin--angiotensin system gene polymorphisms and clinicopathological correlations in IgA nephropathy.	Ong-Ajyooth S et al.	10511770	No data
2	Combinational effect of genes for the renin-angiotensin system in conferring susceptibility to diabetic nephropathy.	Osawa N et al.	17143591	No data
2	Lack of synergism between long-term poor glycaemic control and three gene polymorphisms of the renin angiotensin system on risk of developing diabetic nephropathy in type I diabetic patients.	Tarnow L et al.	10907125	No data
2	Gene polymorphisms of angiotensin-converting enzyme and angiotensin II Type 1 receptor among chronic kidney disease patients in a Chinese population	Su SL et al.	22147663	Overlap in patient group
2	Polymorphism of renin-angiotensin system genes in IgA nephropathy	Woo KT et al.	15504143	Overlap in patient group
2	Association of a polymorphism of the apolipoprotein E gene with chronic kidney disease in Japanese individuals with metabolic syndrome	Yoshida T et al.	19056482	Overlap in patient group

For peer review only

3	Polymorphism of the renin–angiotensin–aldosterone system in patients with chronic allograft dysfunction	Ayed K et al.	16635753	< 3 populations reported per SNP
3	Angiotensin II type 1 receptor gene polymorphism in end-stage renal disease	Buraczynska M et al.	12187084	< 3 populations reported per SNP
3	Genetic polymorphisms of the renin-angiotensin system in end-stage renal disease.	Buraczynska M et al.	16384824	< 3 populations reported per SNP
3	Renin-angiotensin-aldosterone system related gene polymorphisms and urinary total arsenic is related to chronic kidney disease.	Chen WJ et al.	24907556	< 3 populations reported per SNP
3	Hypertension after renal transplantation and polymorphism of genes involved in essential hypertension: ACE, AGT, AT1R and ecNOS	El-Essawy AB et al.	WOS:000174306600003 (11926202)	< 3 populations reported per SNP
3	Genetic polymorphisms of the renin-angiotensin-aldosterone system and renal insufficiency in essential hypertension	Fabris B et al.	15662219	< 3 populations reported per SNP
3	Relationship between polymorphisms in the renin-angiotensin system and nephropathy in type 2 diabetic patients.	Fradin S et al.	11938025	< 3 populations reported per SNP
3	Susceptibility and progression of end stage renal disease are not associated with angiotensin II type 1 receptor gene polymorphism	Hanna MO et al.	25316403	< 3 populations reported per SNP
3	Renin-angiotensin system component gene polymorphisms in Japanese maintenance haemodialysis patients	Kawada N et al.	WOS:000071880400005	< 3 populations reported per SNP
3	GENETIC CLUES TO THE ETIOLOGY OF BALKAN ENDEMIC NEPHROPATHY: INVESTIGATING THE ROLE OF ACE AND AT1R POLYMORPHISMS	Krcunovic Z et al.	WOS:000287217500011	< 3 populations reported per SNP
3	Angiotensinogen and angiotensin II type 1 receptor gene polymorphism in patients with autosomal dominant polycystic kidney disease: Effect on hypertension and ESRD	Lee KB et al.	12950120	< 3 populations reported per SNP

For peer review only

3	Genes involved in the regulation of vascular homeostasis determine renal survival rate in patients with chronic glomerulonephritis	Litovkina O et al.	24727057	< 3 populations reported per SNP
3	Polymorphism of renin-angiotensin system genes in dialysis patients - association with cerebrovascular disease	Losito A et al.	WOS:000179668600025 (12454231)	< 3 populations reported per SNP
3	Contribution of genetic polymorphism in the renin-angiotensin system to the development of renal complications in insulin-dependent diabetes	Marre M et al.	9120002	< 3 populations reported per SNP
3	Association of angiotensinogen gene T235 variant with progression of immunoglobin A nephropathy in Caucasian patients.	Pei Y et al.	9259580	< 3 populations reported per SNP
3	Polymorphisms of the renin-angiotensin system genes in Brazilian patients with lupus nephropathy.	Sprovieri SR et al.	15934435	< 3 populations reported per SNP
3	Interaction between gene polymorphisms of nitric oxide synthase and renin-angiotensin system in the progression of membranous glomerulonephritis.	Stratta P et al.	14767013	< 3 populations reported per SNP
3	Gene-Gene Interactions in Renin-Angiotensin-Aldosterone System Contributes to End-Stage Renal Disease Susceptibility in a Han Chinese Population	Su SL et al.	24977181	< 3 populations reported per SNP
3	Association of Genetic Variants with Chronic Kidney Disease in Japanese Individuals	Yoshida T et al.	19406964	< 3 populations reported per SNP
3	Genetic polymorphisms and the risk of progressive renal failure in elderly Hungarian patients.	Zsom M et al.	22111818	< 3 populations reported per SNP

For peer review only

Supplementary Table S3e: Excluded studies from the *AGTR2* search

*Exclusion stage relates to the stage at which the article was removed as shown in the flow diagram (Supplementary Figure S1e)

Exclusion Stage	Title	Authors	Pubmed ID	Reason
1	Association of angiotensinogen gene T235 variant with progression of immunoglobin A nephropathy in Caucasian patients.	Pei Y et al.	9259580	No data for AGTR2
1	Synergistic effect of angiotensin II type 1 receptor genotype and poor glycaemic control on risk of nephropathy in IDDM.	Doria A et al.	9389421	No data for AGTR2
1	Lack of synergism between long-term poor glycaemic control and three gene polymorphisms of the renin angiotensin system on risk of developing diabetic nephropathy in type I diabetic patients.	Tarnow L et al.	10907125	No data for AGTR2
1	Angiotensin II type 1 receptor gene polymorphism and the response to hyperglycemia in early type 1 diabetes.	Miller JA et al.	10969844	No data for AGTR2
1	Polymorphism of angiotensin converting enzyme, angiotensinogen, and angiotensin II type 1 receptor genes and end-stage renal failure in IgA nephropathy: IGARAS--a study of 274 Men.	Frimat L et al.	11053482	No data for AGTR2
1	Altered regulation of renal interstitial hydrostatic pressure and the renal renin-angiotensin system in the absence of atrial natriuretic peptide	O'Tierney PF et al.	18192845	No data for AGTR2
1	The angiotensin type II receptor tonically inhibits angiotensin-converting enzyme in AT2 null mutant mice	Hunley TE et al.	10652034	Non-human study
1	A novel rodent model of pregnancy complications associated with genetically determined angiotensin-converting enzyme (ACE) activity	Mata-Greenwood E et al.	29360395	Non-human study
1	Renin-angiotensin system polymorphisms and renal scarring	Pardo R et al.	12579398	Not a case-control study

For peer review only

1	Association of the genetic polymorphisms of the renin-angiotensin system and endothelial nitric oxide synthase with chronic renal transplant dysfunction.	Akcay A et al.	15385810	Not a case-control study
1	Whole-genome linkage and association scan in primary, nonsyndromic vesicoureteric reflux.	Cordell HJ et al.	19959718	Not a case-control study
1	Association of angiotensin converting enzyme and angiotensin type 2 receptor gene polymorphisms with renal damage in posterior urethral valves.	Laksmi NK et al.	20149750	Not a case-control study
1	Angiotensin type 2 receptor is important in the normal development of the ureter	Hohenfellner K et al.	10353402	Not a renal disease focus
1	Angiotensin II type 2 receptor gene is not responsible for familial vesicoureteral reflux	Yoneda A et al.	12187255	Not a renal disease focus
1	Genetic polymorphism of the renin-angiotensin system on the development of primary vesicoureteral reflux	Yim HE et al.	14764974	Not a renal disease focus
1	Polymorphism in angiotensin II receptor genes and hypertension	Baudin B et al.	15640279	Not a renal disease focus
1	No evidence for angiotensin type 2 receptor gene polymorphism in intron 1 in patients with coarctation of the aorta and Ullrich-Turner syndrome.	Struwe E et al.	16944335	Not a renal disease focus
1	[Effect of eprosartan on the hemostatic system in patients with chronic kidney disease associated with hereditary thrombophilia].	Kaliuzhin VV et al.	24261234	Not written in English or Spanish
1	No evidence for AT2R gene derangement in human urinary tract anomalies.	Hiraoka M et al.	11260384	Paediatric Individuals
1	Polymorphisms of renin-angiotensin system genes in childhood IgA nephropathy.	Maruyama K et al.	11354780	Paediatric Individuals
1	Angiotensin-converting enzyme and angiotensin type 2 receptor gene genotype distributions in Italian children with congenital uropathies.	Rigoli L et al.	15470205	Paediatric Individuals
1	Implication of genetic variations in congenital obstructive nephropathy.	Hahn H et al.	16133060	Paediatric Individuals

For peer review only

1	Angiotensin II type 2 receptor gene polymorphism in Caucasian children with a wide spectrum of congenital anomalies of the kidney and urinary tract	Siomou E et al.	17515833	Paediatric Individuals
1	Association of angiotensin type 2 receptor gene polymorphisms with ureteropelvic junction obstruction in Brazilian patients.	Miranda DM et al.	24995698	Paediatric Individuals
2	Angiotensin-converting enzyme and angiotensin II receptor subtype 2 genotypes in type 1 diabetes and severe hypoglycaemia requiring emergency treatment: a case cohort study.	Pedersen-Bjergaard U et al.	19820429	Incorrect patient group

For peer review only

Supplementary Table S3f: Excluded studies from the *REN* search

*Exclusion stage relates to the stage at which the article was removed as shown in the flow diagram (Supplementary Figure S1f)

Exclusion Stage	Title	Authors	Pubmed ID or WoS ID if Pubmed ID not available	Reason
1	Increased amount of the angiotensin-converting enzyme (ACE) mRNA originating from the ACE allele with deletion.	Suehiro T et al.	15164285	Gene expression based study
1	Increased expression of monocytic angiotensin-converting enzyme in dialysis patients with cardiovascular disease	Ulrich C et al.	16476718	Gene expression based study
1	Synergistic expression of angiotensin-converting enzyme (ACE) and ACE2 in human renal tissue and confounding effects of hypertension on the ACE to ACE2 ratio	Wakahara S et al.	17303661	Gene expression based study
1	Insertion / Deletion Polymorphism of Angiotensin Converting Enzyme Gene Does Not Contribute to Chronic Kidney Disease in Palestine	Abuaisha AM et al.	WOS:000433049000003	No data for REN
1	Relationship between GSTs gene polymorphism and susceptibility to end stage renal disease among North Indians.	Agrawal S et al.	18067039	No data for REN
1	ACE variants interact with the RAS pathway to confer risk and protection against type 2 diabetic nephropathy.	Ahluwalia TS et al.	19108684	No data for REN
1	Analysis of insertion/deletion polymorphisms of the angiotensin converting enzyme gene in Malaysian end-stage renal disease patients.	Ali A et al.	21421653	No data for REN
1	Apparent Mineralocorticoid Excess Caused By A Novel Mutation In 11-Beta Hydroxysteroid Dehydrogenase Type 2 Enzyme: Its Genetics And Response To Therapy	Alzahrani AS et al.	24936560	No data for REN
1	Clinical impact of an angiotensin I-converting enzyme insertion/deletion and kinin B2 receptor +9/-9 polymorphisms in the prognosis of renal transplantation	Amorim CEN et al.	23362199	No data for REN

For peer review only

1	Analysis of polymorphism in Renin Angiotensin System and other related genes in South Indian chronic kidney disease patients	Anbazhagan K et al.	19520069	No data for REN
1	Circulating angiotensin-converting enzyme 2 activity in patients with chronic kidney disease without previous history of cardiovascular disease	Anguiano L et al.	25813276	No data for REN
1	Lack of association between the angiotensin-converting enzyme gene (I/D) polymorphism and diabetic nephropathy in Tunisian type 2 diabetic patients.	Arfa I et al.	18404607	No data for REN
1	Associations between apolipoprotein E gene polymorphism and plasminogen activator inhibitor-1 and atherogenic lipid profile in dialysis patients.	Arikan H et al.	17763167	No data for REN
1	The DD genotype of the ACE gene polymorphism is associated with diabetic nephropathy in type-1 diabetics	Azar ST et al.	11428725	No data for REN
1	Association of sequence polymorphism in the mitochondrial D-loop with chronic kidney disease.	Bai Y et al.	24576051	No data for REN
1	Impact of aldosterone synthase gene C-344T polymorphism on IgA nephropathy.	Bantis C et al.	21476902	No data for REN
1	Influence of cytokine gene polymorphisms on focal segmental glomerulosclerosis	Bantis C et al.	15308875	No data for REN
1	Influence of aldosterone synthase gene C-344T polymorphism on focal segmental glomerulosclerosis	Bantis C et al.	21777344	No data for REN
1	Identification of Cathepsin L as a Potential Sex-Specific Biomarker for Renal Damage	Bauer Y et al.	21357272	No data for REN
1	Determinants of Progression in Early Autosomal Dominant Polycystic Kidney Disease: Is it Blood Pressure or Renin-Angiotensin-Aldosterone-System Blockade?	Brosnahan GM et al.	29564978	No data for REN
1	Genetic determination of TNF and myeloperoxidase production in dialyzed patients with diabetic nephropathy.	Buraczynska M et al.	15600254	No data for REN

For peer review only

1	Genetic polymorphisms of the renin-angiotensin system in end-stage renal disease	Buraczynska M et al.	16384824	No data for REN
1	Angiotensin II type 1 receptor gene polymorphism in end-stage renal disease	Buraczynska M et al.	12187084	No data for REN
1	Familial renal glucosuria: SLC5A2 mutation analysis and evidence of salt-wasting	Calado J et al.	16518345	No data for REN
1	Non-relation of parathyroid hormone gene polymorphisms to secondary hyperparathyroidism in Chinese hemodialysis patients.	Chen JB et al.	15083922	No data for REN
1	Effect of IL-6 C-572G polymorphism on idiopathic membranous nephropathy risk in a Han Chinese population.	Chen SY et al.	20954977	No data for REN
1	Lack of association between transient receptor potential cation channel 6 polymorphisms and primary membranous glomerulonephritis.	Chen WC et al.	20540633	No data for REN
1	Renin-angiotensin-aldosterone system related gene polymorphisms and urinary total arsenic is related to chronic kidney disease	Chen WJ et al.	24907556	No data for REN
1	Toll-like receptor 9 SNPs are susceptible to the development and progression of membranous glomerulonephritis: 27 years follow-up in Taiwan.	Chen YT et al.	23964786	No data for REN
1	Endothelial nitric oxide synthase gene polymorphisms and the renal hemodynamic response to L-arginine	Cherney DZI et al.	19037250	No data for REN
1	Influence of uridine diphosphate-glucuronosyltransferases (1A9) polymorphisms on mycophenolic acid pharmacokinetics in patients with renal transplant.	Ciftci HS et al.	30012031	No data for REN
1	Serological and genetic factors in early recurrence of IgA nephropathy after renal transplantation	Coppo R et al.	17988266	No data for REN
1	Association of glutathione S-transferase M1 and T1 gene polymorphism with oxidative stress in diabetic and nondiabetic chronic kidney disease.	Datta SK et al.	20954980	No data for REN
1	ACE, PAI-1, decorin and Warner helicase genes are not associated with the development of renal disease in European patients with Type 1 diabetes	De Cosmo S et al.	10495473	No data for REN
1	U.K. Prospective Diabetes Study. XV: Relationship of renin-angiotensin system gene polymorphisms with microalbuminuria in NIDDM.	Dudley CR et al.	8587251	No data for REN

For peer review only

1	Angiotensin II type 1 receptor (A1166C) gene polymorphism in Egyptian adult hemodialysis patients	El-Banawy H et al.	WOS:000365886400009	No data for REN
1	Mthfr C677T, A1298C And Ace I/D Polymorphisms As Risk Factors For Diabetic Nephropathy Among Type 2 Diabetic Patients.	El-Baz R et al.	22554825	No data for REN
1	Impact of nitric oxide synthase Glu298Asp polymorphism on the development of end-stage renal disease in type 2 diabetic Egyptian patients.	El-Din Bessa SS et al.	21854353	No data for REN
1	Angiotensin-I converting enzyme gene polymorphism in Turkish type 2 diabetic patients	Ergen HA et al.	15365253	No data for REN
1	GAS6 intron 8 c.834 + 7G > A gene polymorphism in diabetic nephropathy.	Erkoc R et al.	25869052	No data for REN
1	DNA polymorphisms in the ACE gene, serum ACE activity and the risk of nephropathy in insulin-dependent diabetes mellitus	Freire MBS et al.	9794558	No data for REN
1	Polymorphism of angiotensin converting enzyme, angiotensinogen, and angiotensin II type 1 receptor genes and end-stage renal failure in IgA nephropathy: IGARAS - A study of 274 men	Frimat L et al.	11053482	No data for REN
1	Polymorphisms in the gene encoding angiotensin I converting enzyme 2 and diabetic nephropathy	Frojdo S et al.	16211375	No data for REN
1	Lack of association between the heparan sulfate proteoglycan gene polymorphism and diabetic nephropathy in Japanese NIDDM with proliferative diabetic retinopathy.	Fujita H et al.	10586428	No data for REN
1	Effects of erythropoietin, angiotensin II, and angiotensin-converting enzyme inhibitor on erythroid precursors in patients with posttransplantation erythrocytosis	Glicklich D et al.	10428268	No data for REN
1	Angiotensinogen gene polymorphisms and progression of chronic kidney disease in ADPKD patients	Gnanasambandan R et al.	26482465	No data for REN
1	Association study of ACE polymorphisms and systemic lupus erythematosus in Northern Chinese Han population	Gong AM et al.	22729880	No data for REN

For peer review only

1	Angiotensin I converting enzyme and angiotensinogen gene polymorphisms in non-insulin-dependent diabetes mellitus. Lack of relationship with diabetic nephropathy and retinopathy in a Caucasian Mediterranean population	Gutierrez C et al.	9258285	No data for REN
1	Renin-aldosterone response, urinary Na/K ratio and growth in pseudohypoaldosteronism patients with mutations in epithelial sodium channel (ENaC) subunit genes	Hanukoglu A et al.	18634878	No data for REN
1	Polymorphism of the angiotensin I-converting enzyme gene in diabetic nephropathy in type II diabetic patients with proliferative retinopathy.	Hanyu O et al.	9509566	No data for REN
1	Increased frequency of angiotensin-converting enzyme DD genotype in patients with type 2 diabetes in Taiwan	Hsieh MC et al.	10862639	No data for REN
1	Genetic polymorphisms of the renin-angiotensin-aldosterone system in Chinese patients with end-stage renal disease secondary to IgA nephropathy	Huang HD et al.	21163122	No data for REN
1	Impact of Polymorphisms of the Genes Encoding Angiotensin II-Forming Enzymes on the Progression of IgA Nephropathy	Jung ES et al.	21150220	No data for REN
1	Study of the association of -667 aquaporin-2 (AQP-2) A/G promoter polymorphism with the incidence and clinical course of chronic kidney disease in Korea.	Kang SW et al.	17763164	No data for REN
1	Assessment of matrix Gla protein, Klotho gene polymorphisms, and oxidative stress in chronic kidney disease.	Karsli Ceppioglu S et al.	21859400	No data for REN
1	Renin-angiotensin system component gene polymorphisms in Japanese maintenance haemodialysis patients	Kawada N et al.	WOS:000071880400005	No data for REN
1	Manganese superoxide dismutase, glutathione peroxidase and catalase gene polymorphisms and clinical outcomes in acute kidney injury.	Kidir V et al.	26787049	No data for REN
1	Blood Pressure-Related Genes and the Progression of IgA Nephropathy	Kim SM et al.	19729965	No data for REN
1	Genetic Clues To The Etiology Of Balkan Endemic Nephropathy: Investigating The Role Of Ace And At1R Polymorphisms	Krcunovic Z et al.	WOS:000287217500011	No data for REN

For peer review only

1	Effect of ACE gene on diabetic nephropathy in NIDDM patients with insulin resistance	Kuramoto N et al.	10023638	No data for REN
1	Association of apolipoprotein E gene polymorphism with end-stage renal disease and hyperlipidemia in patients on long-term hemodialysis.	Lahrach H et al.	25155022	No data for REN
1	Aldosterone synthase (CYP11B2)-344T/C polymorph ism is not associated with the initiation and progression of diabetic nephropathy in Caucasian Type 1 diabetic patients	Lajer M et al.	16759311	No data for REN
1	Renal perfusion and the renal hemodynamic response to blocking the renin system in diabetes - Are the forces leading to vasodilation and vasoconstriction linked?	Lansang MC et al.	12086929	No data for REN
1	Renin-angiotensin system gene polymorphisms: its impact on IgAN and its progression to end-stage renal failure among Chinese in Singapore.	Lau YK et al.	15153745	No data for REN
1	Association studies between the HSD11B2 gene (encoding human 11 beta-hydroxysteroid dehydrogenase type 2), type 1 diabetes mellitus and diabetic nephropathy	Lavery GG et al.	11916625	No data for REN
1	Association of the genetic polymorphisms of the ACE gene and the eNOS gene with lupus nephropathy in northern Chinese population	Li X et al.	20540812	No data for REN
1	The relationship between the TGF-beta1 gene -509C/T polymorphism and tubulointerstitial damage resulting from primary nephrotic syndrome.	Li Y et al.	20446778	No data for REN
1	Genes involved in the regulation of vascular homeostasis determine renal survival rate in patients with chronic glomerulonephritis	Litovkina O et al.	24727057	No data for REN
1	Factors affecting progression of renal insufficiency	Locatelli F et al.	9387138	No data for REN
1	Polymorphism of renin-angiotensin system genes in dialysis patients - association with cerebrovascular disease	Losito A et al.	12454231	No data for REN
1	Genetic polymorphisms of the renin-angiotensin-aldosterone system in end-stage renal disease	Lovati E et al.	11422735	No data for REN

For peer review only

1	Effects of the genetic polymorphisms of the renin-angiotensin system on focal segmental glomerulosclerosis	Luther Y et al.	14610337	No data for REN
1	Angiotensinogen (AGT) gene missense polymorphisms (rs699 and rs4762) and diabetic nephropathy in Caucasians with type 2 diabetes mellitus.	Makuc J et al.	28488548	No data for REN
1	Impact of interferon-gamma and interleukin-4 gene polymorphisms on development and progression of IgA nephropathy in Japanese patients	Masutani K et al.	12552499	No data for REN
1	Influence of the endothelial nitric oxide synthase polymorphism on the progression of autosomal dominant polycystic kidney disease and IgA nephropathy.	Merta M et al.	12212826	No data for REN
1	Manganese Superoxide Dismutase (SOD2) Polymorphisms, Plasma Advanced Oxidation Protein Products (AOPP) Concentration and Risk of Kidney Complications in Subjects with Type 1 Diabetes	Mohammedi K et al.	24819633	No data for REN
1	The effect of polymorphisms in the renin-angiotensin-aldosterone system on diabetic nephropathy risk	Mollsten A et al.	18413189	No data for REN
1	A polymorphism in the angiotensin II type 1 receptor gene has different effects on the risk of diabetic nephropathy in men and women	Mollsten A et al.	21316998	No data for REN
1	Different Mechanisms for the Progression of CKD with ACE Gene Polymorphisms	Nakayama Y et al.	19293592	No data for REN
1	Pronatriodilatin gene polymorphisms, microvascular permeability, and diabetic nephropathy in type 1 diabetes mellitus	Nannipieri M et al.	10405209	No data for REN
1	A biallelic gene polymorphism of CYP11B2 predicts increased aldosterone to renin ratio in selected hypertensive patients.	Nicod J et al.	12788845	No data for REN
1	Role of the alpha-adducin genotype on renal disease progression	Nicod J et al.	11918733	No data for REN
1	Association between CCDC132, FDX1 and TNFSF13 gene polymorphisms and the risk of IgA nephropathy.	Niu D et al.	26370181	No data for REN
1	Chemerin rs17173608 and vaspin rs2236242 gene variants on the risk of end stage renal disease (ESRD) and correlation with plasma malondialdehyde (MDA) level.	Nomani H et al.	29644922	No data for REN

For peer review only

1	Renal clearance of endogenous erythropoietin in patients with proteinuria.	Nowicki M et al.	7759206	No data for REN
1	The renin--angiotensin system gene polymorphisms and clinicopathological correlations in IgA nephropathy.	Ong-Ajyooth S et al.	10511770	No data for REN
1	Angiotensin-converting enzyme polymorphism gene and evolution of nephropathy to end-stage renal disease	Ortiz MA et al.	15012717	No data for REN
1	Combinational effect of genes for the renin-angiotensin system in conferring susceptibility to diabetic nephropathy	Osawa N et al.	17143591	No data for REN
1	Bcii--RFLP profiles for serum amiloid A1 and mutated MEFV gene prevalence in chronic renal failure patients requiring long-term hemodialysis.	Ozdemir O et al.	25394530	No data for REN
1	Survival in type 2 diabetic patients in dialysis and the number of risk alleles in polymorphisms of the renin-angiotensin system genes	Padro-Miquel A et al.	19014923	No data for REN
1	Association of aldosterone synthase (CYP11B2) gene -344T/C polymorphism with the risk of primary chronic glomerulonephritis in the Polish population.	Pawlik M et al.	23681285	No data for REN
1	Does copy number variation of APOL1 gene affect the susceptibility to focal segmental glomerulosclerosis?	Peng T et al.	28494221	No data for REN
1	Association analysis of ADPRT1, AKR1B1, RAGE, GFPT2 and PAI-1 gene polymorphisms with chronic renal insufficiency among Asian Indians with type-2 diabetes	Prasad P et al.	20353610	No data for REN
1	DNA repair genes XPD and XRCC1 polymorphisms and risk of end-stage renal disease in Egyptian population.	Radwan WM et al.	25310768	No data for REN
1	ACE I/D and MMP-7 A-181G variants and the risk of end stage renal disease	Rahimi Z et al.	28447048	No data for REN
1	Influence of angiotensin converting enzyme (ACE) gene rs4362 polymorphism on the progression of kidney failure in patients with autosomal dominant polycystic kidney disease (ADPKD)	Ramanathan G et al.	27748299	No data for REN

For peer review only

1	Aldosterone synthase gene is not a major susceptibility gene for progression of chronic kidney disease in patients with autosomal dominant polycystic kidney disease	Ramanathan G et al.	28540892	No data for REN
1	AGTR1 rs5186 variants in patients with type 2 diabetes mellitus and nephropathy	Razi F et al.	WOS:000419720600009	No data for REN
1	Association of the angiotensinogen M235T and APO E gene polymorphisms in Turkish type 2 diabetic patients with and without nephropathy.	Reis KA et al.	21500980	No data for REN
1	The influence of the endothelin-converting enzyme-1 gene polymorphism on the progression of autosomal dominant polycystic kidney disease.	Reiterova J et al.	16526315	No data for REN
1	The influence of G-protein beta3-subunit gene and endothelial nitric oxide synthase gene in exon 7 polymorphisms on progression of autosomal dominant polycystic kidney disease.	Reiterova J et al.	15287194	No data for REN
1	Role of ADDUCIN Gly460Trp, ACE I/D and AGT M235T Gene Polymorphisms in Genetic Susceptibility to Diabetic Nephropathy	Sancakdar E et al.	WOS:000367541400005	No data for REN
1	Angiotensin I converting enzyme gene polymorphism and diabetic nephropathy in type II diabetes	Schmidt S et al.	9269698	No data for REN
1	ACACÎ² gene (rs2268388) and AGTR1 gene (rs5186) polymorphism and the risk of nephropathy in Asian Indian patients with type 2 diabetes.	Shah VN et al.	23081748	No data for REN
1	Genetic variants of ACE (Insertion/Deletion) and AGT (M268T) genes in patients with diabetes and nephropathy	Shaikh R et al.	24737640	No data for REN
1	Distribution of ACE I/D Polymorphism in the Patients of Diabetes and Nephropathy in Pakistan	Shaikh R et al.	WOS:000312053000001	No data for REN
1	The relationship between genetic and haemodynamic factors in diabetic nephropathy (DN): Case-control study in type 1 diabetes mellitus (T1DM)	Shestakova MV et al.	WOS:000242410600010	No data for REN

For peer review only

1	SIRTUIN 1 gene polymorphisms are associated with cholesterol metabolism and coronary artery calcification in Japanese hemodialysis patients.	Shimoyama Y et al.	22200427	No data for REN
1	Interleukin 1 receptor antagonist and tumor necrosis factor-alpha gene polymorphism in patients with end-stage renal failure.	Shu KH et al.	15717635	No data for REN
1	Angiotensin-converting enzyme (ACE) inhibition in type 2, diabetic patients - interaction with ACE insertion/deletion polymorphism	So WY et al.	16395257	No data for REN
1	Vitamin D binding protein and the need for vitamin D in hemodialysis patients.	Speeckaert MM et al.	18721734	No data for REN
1	Polymorphisms of the renin-angiotensin system genes in Brazilian patients with lupus nephropathy	Sprovieri SRS et al.	15934435	No data for REN
1	Prospective study on the potential of RAAS blockade to halt renal disease in Alport syndrome patients with heterozygous mutations.	Stock J et al.	27402170	No data for REN
1	Interaction between gene polymorphisms of nitric oxide synthase and renin-angiotensin system in the progression of membranous glomerulonephritis	Stratta P et al.	14767013	No data for REN
1	Angiotensin II receptor type 1 A1166C modifies the association between angiotensinogen M235T and chronic kidney disease	Su SL et al.	29296205	No data for REN
1	Gene polymorphisms of angiotensin-converting enzyme and angiotensin II Type 1 receptor among chronic kidney disease patients in a Chinese population	Su SL et al.	22147663	No data for REN
1	Gene-Gene Interactions in Renin-Angiotensin-Aldosterone System Contributes to End-Stage Renal Disease Susceptibility in a Han Chinese Population	Su SL et al.	24977181	No data for REN
1	Study on 3'-UTR length polymorphism in peripheral blood mononuclear cells of uremia patient.	Sui W et al.	26554293	No data for REN
1	Angiotensin-II type 1 receptor gene polymorphism and diabetic microangiopathy.	Tarnow L et al.	8671962	No data for REN

For peer review only

1	Lack Of Relationship Between An Insertion Deletion Polymorphism In The Angiotensin I-Converting Enzyme Gene And Diabetic Nephropathy And Proliferative Retinopathy In Iddm Patients	Tarnow L et al.	7729604	No data for REN
1	Lack of synergism between long-term poor glycaemic control and three gene polymorphisms of the renin angiotensin system on risk of developing diabetic nephropathy in Type I diabetic patients	Tarnow L et al.	10907125	No data for REN
1	High prevalence of ACE DD genotype among north Indian end stage renal disease patients.	Tripathi G et al.	17042963	No data for REN
1	Vitamin D receptor genetic variants among patients with end-stage renal disease.	Tripathi G et al.	20722565	No data for REN
1	Association between angiotensin converting enzyme gene polymorphism and clinical features in autosomal dominant polycystic kidney disease	Uemasu J et al.	9180368	No data for REN
1	The DD genotype of the ACE gene polymorphism is associated with progression of diabetic nephropathy to end stage renal failure in IDDM	Vleming LJ et al.	10099885	No data for REN
1	Relationship between angiotensinogen gene M235T variant with diabetic nephropathy in Chinese NIDDM.	Wang J et al.	11717948	No data for REN
1	Nodular glomerulosclerosis and renin angiotensin system in Chinese patients with type 2 diabetes	Wang M et al.	26973293	No data for REN
1	Predicting the development of diabetic nephropathy and its progression	William J et al.	15822056	No data for REN
1	Polymorphism of renin-angiotensin system genes in IgA nephropathy	Woo KT et al.	15504143	No data for REN
1	Platelet glycoprotein IIb HPA-3 a/b polymorphism is associated with native arteriovenous fistula thrombosis in chronic hemodialysis patients.	Wu JH et al.	22880801	No data for REN
1	Association of plasminogen activator inhibitor-1 gene polymorphism and type 2 diabetic nephropathy.	Xu F et al.	26616527	No data for REN
1	A candidate gene approach to genetic contributors to the development of IgA nephropathy	Yamamoto R et al.	21737517	No data for REN

For peer review only

1	Impact of interaction of cigarette smoking with angiotensin-converting enzyme polymorphisms on end-stage renal disease risk in a Han Chinese population.	Yang HY et al.	23477970	No data for REN
1	Angiotensin converting enzyme gene polymorphism and development of post-transplant erythrocytosis.	Yildiz A et al.	12832741	No data for REN
1	Aldosterone Synthase CYP11B2 Gene Promoter Polymorphism in a Turkish Population With Chronic Kidney Disease	Yilmaz M et al.	25957425	No data for REN
1	Structural analysis of the 11 beta-hydroxysteroid dehydrogenase type 2 gene in end-stage renal disease	Zaehner T et al.	11012876	No data for REN
1	Arg913Gln of SLC12A3 gene promotes development and progression of end-stage renal disease in Chinese type 2 diabetes mellitus	Zhang R et al.	28744814	No data for REN
1	Pin1 and secondary hyperparathyroidism of chronic kidney disease: gene polymorphisms and protein levels.	Zhao Y et al.	27876426	No data for REN
1	Polymorphisms in NADPH oxidase CYBA gene modify the risk of ESRD in patients with chronic glomerulonephritis.	Zhou H et al.	26627442	No data for REN
1	Angiotensinogen M235T and chymase gene CMA/B polymorphisms are not associated with nephropathy in type II diabetes	Zychma MJ et al.	11096141	No data for REN
1	Effects of Diets with Different Proportions of Protein/Carbohydrate on Retinal Manifestations in db Mice	Arimura E et al.	29475908	Non-human study
1	Effects of plasma kallikrein deficiency on haemostasis and thrombosis in mice: Murine Ortholog of the Fletcher Trait	Bird JE et al.	22398951	Non-human study
1	Blood pressure and renin-angiotensin system resetting in transgenic rats with elevated plasma Val(5)-angiotensinogen	Bohlender J et al.	22728903	Non-human study
1	Angiotensin II mesenteric and renal vasoregulation: Dissimilar modulatory effects with nitroprusside	Broome M et al.	11065204	Non-human study
1	N-domain angiotensin I-converting enzyme expression in renal artery of Wistar, Wistar Kyoto, and spontaneously hypertensive rats	Bueno V et al.	15194348	Non-human study
1	Adrenomedullin gene expression differences in mice do not affect blood pressure but modulate hypertension-induced pathology in males	Caron K et al.	17360661	Non-human study

For peer review only

1	Appropriate regulation of renin and blood pressure in 45-kb human renin/human angiotensinogen transgenic mice.	Catanzaro DF et al.	9931123	Non-human study
1	Vascular and renal effects of vasopeptidase inhibition and angiotensin-converting enzyme blockade in spontaneously diabetic Goto-Kakizaki rats	Cheng ZJ et al.	16093923	Non-human study
1	Mice lacking endothelial ACE - Normal blood pressure with elevated angiotensin	Cole JM et al.	12574101	Non-human study
1	Thyroid hormones stimulate renal expression of CFTR	de Andrade Pinto AC et al.	17595518	Non-human study
1	Renin-angiotensin system transgenic mouse model recapitulates pathophysiology similar to human preeclampsia with renal injury that may be mediated through VEGF.	Denney JM et al.	27927648	Non-human study
1	Cardiac angiotensin-(1-12) expression and systemic hypertension in rats expressing the human angiotensinogen gene.	Ferrario CM et al.	26873967	Non-human study
1	Attenuation of accelerated renal cystogenesis in Pkd1 mice by renin-angiotensin system blockade	Fitzgibbon WR et al.	29021226	Non-human study
1	Renal cyst growth is the main determinant for hypertension and concentrating deficit in Pkd1-deficient mice	Fonseca JM et al.	24429399	Non-human study
1	Adenosine A(1) receptor-dependent and independent pathways in modulating renal vascular responses to angiotensin II	Gao X et al.	25251152	Non-human study
1	Collecting duct-specific knockout of nitric oxide synthase 3 impairs water excretion in a sex-dependent manner	Gao Y et al.	27707708	Non-human study
1	Metformin prevents the impairment of endothelium-dependent vascular relaxation induced by high glucose challenge in rabbit isolated perfused kidneys	Gomes MB et al.	16133490	Non-human study
1	Effect Of Nitric-Oxide On Renin Secretion .1. Studies In Isolated Juxtaglomerular Granular Cells	Greenberg SG et al.	7771523	Non-human study

For peer review only

1	Effect of the angiotensinogen genotype on experimental hypertension in mice	Handtrack C et al.	17333097	Non-human study
1	Pituitary adenylate cyclase-activating polypeptide stimulates renin secretion via activation of PAC1 receptors	Hautmann M et al.	17360952	Non-human study
1	The genetic deletion of Mas abolishes salt induced hypertension in mice	Heringer-Walther S et al.	22652430	Non-human study
1	An essential role of angiotensin II receptor type 1a in recipient kidney, not in transplanted peripheral blood leukocytes, in progressive immune-mediated renal injury	Hisada Y et al.	11555672	Non-human study
1	The angiotensin type II receptor tonically inhibits angiotensin-converting enzyme in AT2 null mutant mice	Hunley TE et al.	10652034	Non-human study
1	Collecting Duct Nitric Oxide Synthase 1 beta Activation Maintains Sodium Homeostasis During High Sodium Intake Through Suppression of Aldosterone and Renal Angiotensin II Pathways	Hyndman KA et al.	29066445	Non-human study
1	Transfer of a salt-resistant renin allele raises blood pressure in Dahl salt-sensitive rats	Jiang J et al.	9040448	Non-human study
1	Angiotensin-converting enzyme inhibition attenuates the progression of rat hepatic fibrosis	Jonsson JR et al.	11438504	Non-human study
1	Rat Ace allele variation determines susceptibility to AngII-induced renal damage	Kamilic J et al.	21788250	Non-human study
1	Regulation of renin secretion and expression in mice deficient in ss 1- and ss 2-adrenergic receptors	Kim SM et al.	17515456	Non-human study
1	Low Blood Pressure in Endothelial Cell-Specific Endothelin 1 Knockout Mice	Kisanuki YY et al.	20516397	Non-human study
1	Lack of an effect of collecting duct-specific deletion of adenylyl cyclase 3 on renal Na+ and water excretion or arterial pressure	Kittikulsuth W et al.	24431204	Non-human study

For peer review only

1	A High Fat Diet During Pregnancy and Lactation Induces Cardiac and Renal Abnormalities in GLUT4+/- Male Mice	Kruse M et al.	28750406	Non-human study
1	Blood pressure and renal hemodynamic responses to acute angiotensin II infusion are enhanced in a female mouse model of systemic lupus erythematosus	Kumari S et al.	27350671	Non-human study
1	Control of renin secretion from kidneys with renin cell hyperplasia	Kurt B et al.	24285498	Non-human study
1	Reciprocal expression of connexin 40 and 45 during phenotypical changes in renin-secreting cells	Kurt B et al.	21209011	Non-human study
1	Stimulation of renin secretion by NO donors is related to the cAMP pathway	Kurtz A et al.	9575895	Non-human study
1	Stimulation of renin secretion by nitric oxide is mediated by phosphodiesterase 3	Kurtz A et al.	9539809	Non-human study
1	Replacement of connexin 40 by connexin 45 causes ectopic localization of renin-producing cells in the kidney but maintains in vivo control of renin gene expression	Kurtz L et al.	19474190	Non-human study
1	Interference with GsÎ±-Coupled Receptor Signaling in Renin-Producing Cells Leads to Renal Endothelial Damage.	Lachmann P et al.	28775003	Non-human study
1	The angiotensin II receptor blocker candesartan improves survival and mesenteric perfusion in an acute porcine endotoxin model	Laesser M et al.	14995942	Non-human study
1	Mice with targeted disruption of the acyl-CoA binding protein display attenuated urine concentrating ability and diminished renal aquaporin-3 abundance	Langaa S et al.	22237802	Non-human study
1	Physiological impact of increased expression of the AT(1) angiotensin receptor	Le TH et al.	12963678	Non-human study
1	Genetic analysis of the S-A and Na+/K+-ATPase alpha(1) genes in the Milan hypertensive rat	Lodwick D et al.	9535139	Non-human study

For peer review only

1	Hypertension in unilaterally nephrectomized rats induced by single-kidney transfection with angiotensinogen cDNA	Marley WS et al.	10567853	Non-human study
1	Cardiovascular dysfunction in Zucker obese and Zucker diabetic fatty rats: role of hydronephrosis	Marsh SA et al.	17351065	Non-human study
1	A novel rodent model of pregnancy complications associated with genetically determined angiotensin-converting enzyme (ACE) activity	Mata-Greenwood E et al.	29360395	Non-human study
1	Chronic hypertension and altered baroreflex responses in transgenic mice containing the human renin and human angiotensinogen genes	Merrill DC et al.	8613528	Non-human study
1	Hypervolemia of pregnancy is not maintained in mice chronically overexpressing angiotensinogen	Morgan TK et al.	16796982	Non-human study
1	Vascular angiotensin-converting enzyme expression regulates local angiotensin II	Muller DN et al.	9039087	Non-human study
1	Angiotensin-Ii Enhances Norepinephrine Spillover During Sympathetic Activation In Conscious Rabbits	Noshiro T et al.	8203585	Non-human study
1	Effect Of Angiotensin-Converting Enzyme-Inhibition On Renal Norepinephrine Spillover Rate And Baroreflex Responses In Conscious Rabbits	Noshiro T et al.	1648463	Non-human study
1	Altered regulation of renal interstitial hydrostatic pressure and the renal renin-angiotensin system in the absence of atrial natriuretic peptide	O'Tierney PF et al.	18192845	Non-human study
1	Gene Trapping Uncovers Sex-Specific Mechanisms for Upstream Stimulatory Factors 1 and 2 in Angiotensinogen Expression	Park S et al.	22547438	Non-human study
1	Knockdown of parathyroid hormone related protein in smooth muscle cells alters renal hemodynamics but not blood pressure	Raison D et al.	23720345	Non-human study
1	Nephron-specific deletion of the prorenin receptor causes a urine concentration defect	Ramkumar N et al.	25995108	Non-human study
1	Alternative splicing of vitamin D-24-hydroxylase: a novel mechanism for the regulation of extrarenal 1,25-dihydroxyvitamin D synthesis.	Ren S et al.	15788398	Non-human study

For peer review only

1	20-Hydroxyeicosatetraenoic Acid (HETE)-dependent Hypertension in Human Cytochrome P450 (CYP) 4A11 Transgenic Mice NORMALIZATION OF BLOOD PRESSURE BY SODIUM RESTRICTION, HYDROCHLOROTHIAZIDE, OR BLOCKADE OF THE TYPE 1 ANGIOTENSIN II RECEPTOR	Savas S et al.	27298316	Non-human study
1	Parallel regulation of renin and lysosomal integral membrane protein 2 in renin-producing cells: further evidence for a lysosomal nature of renin secretory vesicles	Schmid J et al.	23229015	Non-human study
1	Stimulation of renin release by prostaglandin E(2) is mediated by EP(2) and EP(4) receptors in mouse kidneys	Schweda F et al.	15113745	Non-human study
1	Preserved macula densa-dependent renin secretion in A(1) adenosine receptor knockout mice	Schweda F et al.	12475747	Non-human study
1	Angiotensin converting enzyme (ACE) gene expression in experimentally induced liver cirrhosis in rats	Shahid SM et al.	24035938	Non-human study
1	Elevated blood pressures in mice lacking endothelial nitric oxide synthase	Shesely EG et al.	8917564	Non-human study
1	Endothelium-Dependent Relaxation In The Isolated Rat-Kidney - Impairment By Cyclosporine-A	Stephan D et al.	8606521	Non-human study
1	Local Renal Circadian Clocks Control Fluid-Electrolyte Homeostasis and BP	Tokonami N et al.	24652800	Non-human study
1	Functional genetic variation in aminopeptidase A (ENPEP): Lack of clear association with focal and segmental glomerulosclerosis (FSGS)	Tonna S et al.	18206321	Non-human study
1	Antihypertensive Role of Tissue Kallikrein in Hyperaldosteronism in the Mouse	Waeckel L et al.	22669897	Non-human study
1	Role of cGMP-kinase II in the control of renin secretion and renin expression	Wagner C et al.	9788971	Non-human study
1	GPR48 Increases Mineralocorticoid Receptor Gene Expression	Wang J et al.	22135314	Non-human study
1	Compensatory up-regulation of angiotensin II subtype 1 receptors in alpha ENaC knockout heterozygous mice	Wang Q et al.	11380824	Non-human study

For peer review only

1	Nebivolol treatment improves resistant arterial function and reduces ventricular hypertrophy and angiotensin II in spontaneously hypertension rats	Wang Y et al.	23263161	Non-human study
1	The elevated blood pressure of human GRK4 gamma A142V transgenic mice is not associated with increased ROS production	Wang Z et al.	17259440	Non-human study
1	Role of neutral endopeptidase 24.11 in AV fistular rat model of heart failure	Wegner M et al.	8759244	Non-human study
1	Role of angiotensin-converting enzyme (ACE and ACE2) imbalance on tourniquet-induced remote kidney injury in a mouse hindlimb ischemia-reperfusion model	Yang XH et al.	22580272	Non-human study
1	Effect of SWL on renal hemodynamics: could a change in renal artery contraction-relaxation responses be the cause?	Yilmaz E et al.	22945811	Non-human study
1	Cosegregation of spontaneously hypertensive rat renin gene with elevated blood pressure in an F-2 generation	Yu H et al.	9794718	Non-human study
1	Role of blood pressure and the renin-angiotensin system in development of diabetic nephropathy (DN) in eNOS(-/-) db/db mice	Zhang MZ et al.	22114203	Non-human study
1	Dendritic cell nuclear protein-1, a novel depression-related protein, upregulates corticotropin-releasing hormone expression.	Zhou T et al.	20693543	Non-human study
1	Angiotensin-converting enzyme genotype is a predictive factor in the peak panel-reactive antibody response	Akcay A et al.	15013293	Not a case-control study
1	Association of the genetic polymorphisms of the renin-angiotensin system and endothelial nitric oxide synthase with chronic renal transplant dysfunction	Akcay A et al.	15385810	Not a case-control study
1	Randomized Controlled Trial: Lisinopril Reduces Proteinuria, Ammonia, and Renal Polypeptide Tubular Catabolism in Patients With Chronic Allograft Nephropathy	Amara AB et al.	20061926	Not a case-control study
1	Influence of cytokine gene polymorphisms on IgA nephropathy.	Bantis C et al.	18300111	Not a case-control study
1	Influence of genetic polymorphisms of the renin-angiotensin system on IgA nephropathy	Bantis C et al.	15031629	Not a case-control study

For peer review only

1	Angiotensin-converting-enzyme insertion/deletion genotype and long-term renal allograft survival	Beige J et al.	9550656	Not a case-control study
1	Angiotensin-converting enzyme genotype and renal allograft survival	Beige J et al.	9259361	Not a case-control study
1	Deletion insertion polymorphism of the angiotensin converting enzyme gene and progression of diabetic nephropathy	Bjorck S et al.	9269704	Not a case-control study
1	Angiotensinogen concentrations and renin clearance : implications for blood pressure regulation.	Bohlender J et al.	10720595	Not a case-control study
1	Genetic polymorphisms of renin-angiotensin system and progression of interstitial nephritis.	Buraczynska M et al.	12898858	Not a case-control study
1	The Epithelial Sodium Channel gamma-Subunit Gene and Blood Pressure Family Based Association, Renal Gene Expression, and Physiological Analyses	Busst CJ et al.	22006290	Not a case-control study
1	Twenty-one additional cases of familial renal glucosuria: absence of genetic heterogeneity, high prevalence of private mutations and further evidence of volume depletion	Calado J et al.	18622023	Not a case-control study
1	Discovery of a novel dominant mutation in the REN gene after forty years of renal disease: a case report	Clissold RL et al.	28701203	Not a case-control study
1	Disorders of mineralocorticoid synthesis	Connell JMC et al.	11469810	Not a case-control study
1	Successful treatment of decompensated chronic viral hepatitis by bursal disease virus vaccine	Csatary LK et al.	10216467	Not a case-control study
1	Natriuretic peptides buffer renin-dependent hypertension	Demerath T et al.	24717731	Not a case-control study
1	Association between two genetic polymorphisms of the renin-angiotensin-aldosterone system and diabetic nephropathy: a meta-analysis	Ding W et al.	21607620	Not a case-control study
1	The effects of angiotensin-converting enzyme gene polymorphism on the progression of immunoglobulin A nephropathy in Malaysian patients	Draman CR et al.	19037561	Not a case-control study

1	Juxtaglomerular cell tumor of the kidney: Report of a non-functioning variant	Endoh Y et al.	9211527	Not a case-control study
1	Prograf produces more benefits for CYP3A5 low expression patients in early stage after kidney transplantation.	Fan B et al.	28157649	Not a case-control study
1	Diabetes, nephropathy, and the renin system	Hollenberg NK	16601578	Not a case-control study
1	Association between Angiotensin I-Converting Enzyme Insertion/Deletion Polymorphism and Prognosis of Kidney Transplantation: A Meta-Analysis	Huang ZK et al.	26000752	Not a case-control study
1	Is there a role of angiotensin-converting enzyme gene polymorphism in the failure of arteriovenous femoral shunts for hemodialysis?	Isbir CS et al.	11525534	Not a case-control study
1	Hyperuricemia, Acute and Chronic Kidney Disease, Hypertension, and Cardiovascular Disease: Report of a Scientific Workshop Organized by the National Kidney Foundation	Johnson RJ et al.	29496260	Not a case-control study
1	Diagnosis of a case of Gitelman's syndrome based on renal clearance studies and gene analysis of a novel mutation of the thiazide-sensitive Na-Cl cotransporter	Kageyama K et al.	16370563	Not a case-control study
1	Paricalcitol as an Antiproteinuric Agent Can Result in the Deterioration of Renal and Heart Function in a Patient with Fabry Disease	Keber T et al.	28596512	Not a case-control study
1	The role of renin-angiotensin-aldosterone system genes in the progression of chronic kidney disease: findings from the Chronic Renal Insufficiency Cohort (CRIC) study.	Kelly TN et al.	25906781	Not a case-control study
1	Smoking has no impact on survival and it is not associated with ACE gene I/D polymorphism in hemodialysis patients.	Kiss I et al.	28058974	Not a case-control study
1	ACE gene polymorphism in focal segmental glomerulosclerosis and membranous glomerulonephritis - Is observed difference of clinical significance?	Kuzmanic D et al.	WOS:000087339600012	Not a case-control study
1	Antihypertensive treatment modulates the association between the D/I ACE gene polymorphism and left ventricular hypertrophy: a meta-analysis	Kuznetsova T et al.	10918550	Not a case-control study

For peer review only

1	Bilateral high origins of testicular arteries: a rare variant.	Li J et al.	22732819	Not a case-control study
1	A Genetic Variant in the Distal Enhancer Region of the Human Renin Gene Affects Renin Expression	Makino Y et al.	26366736	Not a case-control study
1	Genetics and the prediction of complications in type 1 diabetes	Marre M	10097900	Not a case-control study
1	Angiotensin converting enzyme gene polymorphism and renal hemodynamic function in early diabetes	Miller JA et al.	8995725	Not a case-control study
1	The kidney in diabetes: How to control renal and related cardiovascular complications	Mogensen CE	11158852	Not a case-control study
1	A female with X-linked Alport syndrome and compound heterozygous COL4A5 mutations	Mohammad M et al.	24337245	Not a case-control study
1	Catalase activity, allelic variations in the catalase gene and risk of kidney complications in patients with type 1 diabetes	Mohammedi K et al.	24057136	Not a case-control study
1	Renal Tubular Dysgenesis in Israel: Pathologist's Experience and Literature Review	Moldavsky M	19344005	Not a case-control study
1	Tip lesion variant of primary focal and segmental glomerulosclerosis: clinicopathological analysis of 20 cases.	Mungan S et al.	25857429	Not a case-control study
1	Genome-Wide Association Analysis of Plasma B-Type Natriuretic Peptide in Blacks The Jackson Heart Study	Musani SK et al.	25561047	Not a case-control study
1	Angiotensinogen gene variation and renoprotective efficacy of renin-angiotensin system blockade in IgA nephropathy	Narita I et al.	12911556	Not a case-control study
1	Renoprotective efficacy of renin-angiotensin inhibitors in IgA nephropathy is influenced by ACE A2350G polymorphism	Narita I et al.	14684698	Not a case-control study
1	Correlates of ACE activity in macroalbuminuric type 2 diabetic patients treated with chronic ACE inhibition.	Nikzamir et al.	17986476	Not a case-control study
1	Epidemiology and genetics of calcific aortic valve disease	O'Brien KD	17963677	Not a case-control study

For peer review only - http://bmjopen.bmj.com/site/about/guidelines.xhtml

1	Impact of polymorphisms in the renin-angiotensin-aldosterone system on hypertrophic cardiomyopathy	Orenes-Pinero E et al.	21507890	Not a case-control study
1	Relationships between HLA-A, -B, -DQ and -DR antigens and interstitial fibrosis in renal allografts.	Ozdemir BH et al.	15354972	Not a case-control study
1	Renin-angiotensin system gene polymorphisms predict the progression to renal insufficiency among Asians with lupus nephritis	Parsa A et al.	15789057	Not a case-control study
1	Association of angiotensinogen gene T235 variant with progression of immunoglobin A nephropathy in Caucasian patients	Pei Y et al.	9259580	Not a case-control study
1	Normative genetic profiles of RAAS pathway gene Polymorphisms in north Indian and south Indian Populations	Prasad P et al.	18027817	Not a case-control study
1	Identification of a novel mutation in the human mineralocorticoid receptor gene in a German family with autosomal-dominant pseudohypoaldosteronism type 1: Further evidence for marked interindividual clinical heterogeneity	Riepe FG et al.	12679457	Not a case-control study
1	Genetics of diabetic nephropathy	Rippin JD et al.	11554775	Not a case-control study
1	A Synergistic Association of ACE I/D and eNOS G894T Gene Variants with the Progression of Immunoglobulin A Nephropathy - A Pilot Study	Rodriquez-Perez JC et al.	19546528	Not a case-control study
1	Increased left ventricular mass in normotensive type 1 diabetic patients with diabetic nephropathy	Sato A et al.	9727905	Not a case-control study
1	Risk of developing diabetic nephropathy is not associated with synergism between the angiotensin II (type 1) receptor C-1166 allele and poor glycaemic control	Savage DA et al.	10328465	Not a case-control study
1	Alport Syndrome in Women and Girls	Savige J et al.	27287265	Not a case-control study
1	Expert Guidelines for the Management of Alport Syndrome and Thin Basement Membrane Nephropathy	Savige J et al.	23349312	Not a case-control study
1	Genetic determinants of diabetic renal disease and their impact on therapeutic interventions	Schmidt S et al.	9407416	Not a case-control study

For peer review only

1	Rho kinase polymorphism influences blood pressure and systemic vascular resistance in human twins - Role of heredity	Seasholtz TM et al.	16585408	Not a case-control study
1	Impact of genetic polymorphisms of the renin-angiotensin system and of non-genetic factors on kidney transplant function - a single-center experience	Siekierka-Harreis M et al.	19681973	Not a case-control study
1	The deletion/insertion polymorphism of the angiotensin converting enzyme gene and cardiovascular-renal risk	Staessen JA et al.	9488209	Not a case-control study
1	Renal angiomyolipoma - Further immunophenotypic characterization of an expanding morphologic spectrum	Stone CH et al.	11371226	Not a case-control study
1	Frequencies of apolipoprotein E alleles in depressed patients undergoing hemodialysis--a case-control study.	Su YY et al.	25707516	Not a case-control study
1	Assessing genetic susceptibility to diabetic nephropathy	Tanaka N et al.	16174281	Not a case-control study
1	Tip variant of focal segmental glomerulosclerosis: is it truly a benign variant?	Trivedi M et al.	25721429	Not a case-control study
1	Roles of Loss of Chromosome 14q Allele in the Prognosis of Renal Cell Carcinoma with C-reactive Protein Abnormity.	Wang G et al.	28875953	Not a case-control study
1	Contribution of gene polymorphisms in the renin-angiotensin system to macroangiopathy in patients with diabetic nephropathy	Wong et al.	11431175	Not a case-control study
1	Prognostic role of serum ACE activity on outcome of type 2 diabetic patients on chronic ambulatory peritoneal dialysis	Wong TYH et al.	11979350	Not a case-control study
1	Novel mutations in the inverted formin 2 gene of Chinese families contribute to focal segmental glomerulosclerosis.	Xie J et al.	26039629	Not a case-control study
1	Role Of The Deletion Polymorphism Of The Angiotensin-Converting Enzyme Gene In The Progression And Therapeutic Responsiveness Of Iga Nephropathy	Yoshida H et al.	7593601	Not a case-control study
1	Gene Polymorphisms of the Renin-AngiotensinAldosterone system and angiotensin 11 type I-Receptor activating antibodies in renal rejection	Zhang G et al.	17984617	Not a case-control study

For peer review only

1	Rapid detection and quantification of apolipoprotein L1 genetic variants and total levels in plasma by ultra-performance liquid chromatography/tandem mass spectrometry.	Zhou H et al.	24591025	Not a case-control study
1	Pharmacogenetic association of the angiotensin-converting enzyme insertion/deletion polymorphism on blood pressure and cardiovascular risk in relation to antihypertensive treatment - The genetics of hypertension-associated treatment (GenHAT) study	Arnett DK et al.	15967849	Not a renal disease focus
1	A role for CETP TaqIB polymorphism in determining susceptibility to atrial fibrillation: a nested case control study	Asselbergs FW et al.	16623947	Not a renal disease focus
1	Serum paraoxonase-1 gene polymorphism and enzyme activity in patients with urolithiasis.	Atar A et al.	26795139	Not a renal disease focus
1	Association of ACE gene D polymorphism with left ventricular hypertrophy in patients with diastolic heart failure: a case-control study	Bahramali E et al.	26861937	Not a renal disease focus
1	Amiloride, a specific drug for hypertension in black people with T594M variant?	Baker EH et al.	12105131	Not a renal disease focus
1	Association of hypertension with T594M mutation in beta subunit of epithelial sodium channels in black people resident in London	Baker EH et al.	9593408	Not a renal disease focus
1	Alpha-adducin polymorphism in hypertensives of South African ancestry.	Barlassina C et al.	10912759	Not a renal disease focus
1	Common genetic variants and haplotypes in renal CLCNKA gene are associated to salt-sensitive hypertension	Barlassina C et al.	17510212	Not a renal disease focus
1	Synergistic effect of alpha-adducin and ACE genes causes blood pressure changes with body sodium and volume expansion	Barlassina C et al.	10720960	Not a renal disease focus
1	Evaluation of risk factors for the development of nephropathy in patients with IDDM: Insertion/deletion angiotensin converting enzyme gene polymorphism, hypertension and metabolic control	Barnas U et al.	9084972	Not a renal disease focus
1	Polymorphism in angiotensin II receptor genes and hypertension	Baudin B et al.	15640279	Not a renal disease focus
1	Higher thrombin activatable fibrinolysis inhibitor levels are associated with inflammation in attack-free familial Mediterranean fever patients.	Bavbek N et al.	24580410	Not a renal disease focus

For peer review only

1	Insertion-deletion polymorphism in the angiotensin-converting enzyme (ACE) gene among Sudanese, Somalis, Emiratis, and Omanis	Bayoumi RA et al.	16900885	Not a renal disease focus
1	Single Strand Conformation Polymorphism (SSCP) as a quick and reliable method to genotype M235T polymorphism of angiotensinogen gene	Bettinaglio P et al.	12270765	Not a renal disease focus
1	CYP3A5 and ABCB1 genes and hypertension	Bochud M et al.	19290795	Not a renal disease focus
1	Polymorphisms of the renin-angiotensin system in patients with multifocal renal arterial fibromuscular dysplasia	Bofinger A et al.	11317203	Not a renal disease focus
1	Association between plasma activities of semicarbazide-sensitive amine oxidase and angiotensin-converting enzyme in patients with type 1 diabetes mellitus.	Boomsma F et al.	15830186	Not a renal disease focus
1	Structure-Based Analysis of Single Nucleotide Variants in the Renin-Angiotensinogen Complex	Brown DK et al.	28302554	Not a renal disease focus
1	Renin-angiotensin system gene polymorphisms: assessment of the risk of coronary heart disease.	Buraczynska M et al.	14502296	Not a renal disease focus
1	Oxidative stress-related factors in Bartter's and Gitelman's syndromes: relevance for angiotensin II signalling.	Calo LA et al.	12897089	Not a renal disease focus
1	Efficacy of large doses of IL-2-activated human leukocyte antigen haploidentical peripheral blood stem cells on refractory metastatic renal cell carcinoma.	Cao S et al.	21812652	Not a renal disease focus
1	Biochemical and genetic characterization of 11 beta-hydroxysteroid dehydrogenase type 2 in low-renin essential hypertensives.	Carvajal CA et al.	15643127	Not a renal disease focus
1	Role of GRK4 in the Regulation of Arterial AT(1) Receptor in Hypertension	Chen K et al.	24218433	Not a renal disease focus
1	CARD8 rs2043211 polymorphism is associated with gout in a Chinese male population.	Chen Y et al.	25790751	Not a renal disease focus
1	Association of renin-angiotensin and endothelial nitric oxide synthase gene polymorphisms with blood pressure progression and incident hypertension: prospective cohort study.	Conen D et al.	18698212	Not a renal disease focus
1	Role of ACE inhibitors in patients with diabetes mellitus	Cordonnier DJ et al.	11708761	Not a renal disease focus

For peer review only

1	Molecular genetics of the renin-angiotensin-aldosterone system in human hypertension	Corvol P et al.	9296068	Not a renal disease focus
1	Vitamin D receptor gene polymorphisms and plasma renin activity in essential hypertensive individuals.	Cottone S et al.	25500899	Not a renal disease focus
1	Genetic polymorphism of Na-K cotransport in essential hypertension.	Cusi D et al.	2258861	Not a renal disease focus
1	Extensive personal experience - Examination of genotype and phenotype relationships in 14 patients with apparent mineralocorticoid excess	Dave-Sharma S et al.	9661590	Not a renal disease focus
1	ACE and PC-1 gene polymorphisms in normoalbuminuric Type 1 diabetic patients - A 10-year prospective study	de Azevedo MJ et al.	12126783	Not a renal disease focus
1	Polymorphisms in genes of the renin-angiotensin-aldosterone system and renal cell cancer risk: Interplay with hypertension and intakes of sodium, potassium and fluid	Deckers IA et al.	24978482	Not a renal disease focus
1	Adsorption of cytotoxic anti-HLA antibodies with HLA class I immunosorbant beads.	DeVito LD et al.	2186524	Not a renal disease focus
1	Clinical and laboratory characterization of 114 cases of Castleman disease patients from a single centre: paraneoplastic pemphigus is an unfavourable prognostic factor.	Dong Y et al.	25824806	Not a renal disease focus
1	CYP2C9 genotype modifies activity of the renin-angiotensin-aldosterone system in hypertensive men	Donner KM et al.	19593208	Not a renal disease focus
1	TGF-beta1 gene polymorphisms and peritoneal equilibration test results in CAPD patients.	Ebinc FA et al.	18197538	Not a renal disease focus
1	A new theory of essential hypertension based on analysis of the association between a polymorphism of the alpha(2)-adrenoceptor at the 10q24-q26 locus and hypertension in African-Americans	Eggers AE	26243176	Not a renal disease focus
1	High aldosterone-to-renin variants of CYP11B2 and pregnancy outcome.	Escher G et al.	19151144	Not a renal disease focus
1	Cardiovascular effects of aldosterone: insight from adult carriers of mineralocorticoid receptor mutations.	Escoubet B et al.	23852419	Not a renal disease focus

For peer review only

1	Genetic polymorphisms of the renin-angiotensin-aldosterone system and renal insufficiency in essential hypertension	Fabris B et al.	15662219	Not a renal disease focus
1	Recurrence of the R947X mutation in unrelated families with autosomal dominant pseudohypoaldosteronism type 1: evidence for a mutational hot spot in the mineralocorticoid receptor gene.	Fernandes-Rosa FL et al.	16757525	Not a renal disease focus
1	Angiotensin converting enzyme gene I/D polymorphism in essential hypertension and nephroangiosclerosis	Fernandez-Llama P et al.	9607207	Not a renal disease focus
1	Renin-Angiotensin System Polymorphisms and Risk of Hypertension: Influence of Environmental Factors	Forman JP et al.	18550936	Not a renal disease focus
1	CYP3A5 genotype is associated with elevated blood pressure.	Fromm MF et al.	16141800	Not a renal disease focus
1	Associations between the human insulin gene 5' VNTR and clinical variables of the renin-angiotensin system.	Frossard PM et al.	14647005	Not a renal disease focus
1	The interaction of AGT and NOS3 gene polymorphisms with conventional risk factors increases predisposition to hypertension	Gatti RR et al.	22791701	Not a renal disease focus
1	Connexin 43 is not essential for the control of renin synthesis and secretion	Gerl M et al.	24062052	Not a renal disease focus
1	Deletion polymorphism of the angiotensin-converting enzyme gene is independently associated with left ventricular mass and geometric remodeling in systemic hypertension.	Gharavi AG et al.	8677872	Not a renal disease focus
1	A Computational Model of the Circulating Renin-Angiotensin System and Blood Pressure Regulation	Guillaud F et al.	20683640	Not a renal disease focus
1	The presence of PAI-1 4G/5G and ACE DD genotypes increases the risk of early-stage AVF thrombosis in hemodialysis patients.	Gungor Y et al.	21332339	Not a renal disease focus
1	ADAM33: a newly identified gene in the pathogenesis of asthma.	Holgate ST et al.	16257631	Not a renal disease focus
1	Nonmodulation and essential hypertension	Hollenberg NK et al.	16672145	Not a renal disease focus

For peer review only

1	Angiotensinogen genotype affects renal and adrenal responses to angiotensin II in essential hypertension	Hopkins PN et al.	11997278	Not a renal disease focus
1	Blunted renal vascular response to angiotensin II is associated with a common Variant of the angiotensinogen gene and obesity	Hopkins PN et al.	8728297	Not a renal disease focus
1	Genetic variation of the renin-angiotensin system and chronic kidney disease progression in black individuals in the atherosclerosis risk in communities study	Hsu CCC et al.	16396964	Not a renal disease focus
1	Unclassified renal cell carcinoma: a clinicopathological, comparative genomic hybridization, and whole-genome exon sequencing study.	Hu ZY et al.	25120763	Not a renal disease focus
1	A case control association study of ACE gene polymorphism (I/D) with hypertension in Punjabi population from Faisalabad, Pakistan	Hussain M et al.	29058472	Not a renal disease focus
1	Genetic variant of the renin-angiotensin system and prevalence of type 2 diabetes mellitus: a modest but significant effect of aldosterone synthase	Ichikawa M et al.	24549414	Not a renal disease focus
1	The cardiovascular system in familial hypocalciuric hypercalcemia: a cross-sectional study on physiological effects of inactivating variants in the calcium-sensing receptor gene	Jakobsen NFB et al.	27418061	Not a renal disease focus
1	Genetic variants in five novel loci including CFB and CD40 predispose to chronic hepatitis B.	Jiang DK et al.	25802187	Not a renal disease focus
1	The association of the R563Q genotype of the ENaC with phenotypic variation in Southern Africa.	Jones ES et al.	22895453	Not a renal disease focus
1	Renin-Angiotensin System Gene Variants and Type 2 Diabetes Mellitus: Influence of Angiotensinogen	Joyce-Tan SM et al.	26682227	Not a renal disease focus
1	Comprehensive analysis of the renin-angiotensin gene polymorphisms with relation to hypertension in the Japanese	Kato N et al.	10953993	Not a renal disease focus
1	Genetic-Control Of Blood-Pressure And The Angiotensinogen Locus	Kim HS et al.	7708716	Not a renal disease focus
1	Effect of the plasminogen-plasmin system on hypertensive renal and cardiac damage	Knier B et al.	21610512	Not a renal disease focus

For peer review only

1	Polymorphism of the aldosterone synthase gene is not associated with progression of diabetic nephropathy, but associated with hypertension in type 2 diabetic patients	Ko GJ et al.	18771471	Not a renal disease focus
1	Haplotype-based case-control study revealing an association between the adrenomedullin gene and proteinuria in subjects with essential hypertension	Kobayashi Y et al.	16097366	Not a renal disease focus
1	Lys(173)Arg and -344T/C variants of CYP11B2 in Japanese patients with low-renin hypertension.	Komiya I et al.	10720581	Not a renal disease focus
1	Molecular-Biology Of Hypertension	Krieger JE et al.	1832415	Not a renal disease focus
1	Beneficial role of D allele in controlling ACE levels: a study among Brahmins of north India	Kumari S et al.	27350671	Not a renal disease focus
1	Juxtaglomerular cell tumor: A morphological, immunohistochemical and genetic study of six cases	Kuroda N et al.	22939575	Not a renal disease focus
1	Angiotensin-converting enzyme gene polymorphism has no influence on the circulating renin-angiotensin-aldosterone system or blood pressure in normotensive subjects	Lachurie ML et al.	7796503	Not a renal disease focus
1	Angiotensinogen and angiotensin II type 1 receptor gene polymorphism in patients with autosomal dominant polycystic kidney disease: Effect on hypertension and ESRD	Lee KB et al.	12950120	Not a renal disease focus
1	ACE gene insertion/deletion polymorphism associated with 1998 World Health Organization definition of metabolic syndrome in Chinese type 2 diabetic patients	Lee YJ et al.	12032106	Not a renal disease focus
1	The brain and salt-sensitive hypertension	Leenen FHH et al.	11884268	Not a renal disease focus
1	Risk given by AGT polymorphisms in inducing susceptibility to essential hypertension among isolated populations from a remote region of China: A case-control study among the isolated populations	Li Q et al.	26391364	Not a renal disease focus
1	Association Between Polymorphisms of ADRBK1 Gene and Plasma Renin Activity in Hypertensive Patients: A Case-Control Study	Li Y et al.	27555048	Not a renal disease focus
1	No Evidence for the Expression of Renin-Angiotensin-Aldosterone System in Otosclerotic Stapes Footplates	Liktor B et al.	23370555	Not a renal disease focus

For peer review only

1	Papillary renal cell carcinoma: a clinicopathological and whole-genome exon sequencing study.	Liu K et al.	26339402	Not a renal disease focus
1	Common variation in KLKB1 and essential hypertension risk: tagging-SNP haplotype analysis in a case-control study	Lu XF et al.	17318641	Not a renal disease focus
1	The Connexin40 A96S Mutation Causes Renin-Dependent Hypertension	Lubkemeier I et al.	21597036	Not a renal disease focus
1	Connexin 40 is dispensable for vascular renin cell recruitment but is indispensable for vascular baroreceptor control of renin secretion	Machura K et al.	25241776	Not a renal disease focus
1	The association between vitamin D receptor gene polymorphisms (TaqI and FokI), Type 2 diabetes, and micro-/macrovascular complications in postmenopausal women	Maia J et al.	27536155	Not a renal disease focus
1	Angiotensin-converting enzyme (ACE) gene II genotype protects against the development of diabetic peripheral neuropathy in type 2 diabetes mellitus.	Mansoor Q et al.	22607040	Not a renal disease focus
1	Apparent mineralocorticoid excess: Type I and type II	Mantero F et al.	8732999	Not a renal disease focus
1	Low-salt diet and diuretic effect on blood pressure and organ damage	Manunta P et al.	14684671	Not a renal disease focus
1	Renal genetic mechanisms of essential hypertension	Manunta P et al.	9377722	Not a renal disease focus
1	alpha-Adducin polymorphisms and renal sodium handling in essential hypertensive patients	Manunta P et al.	9607177	Not a renal disease focus
1	Is angiotensin-converting enzyme inhibitors/angiotensin receptor blockers therapy protective against prostate cancer?	Mao YQ et al.	26760503	Not a renal disease focus
1	Detection of the association between a deletion polymorphism in the gene encoding angiotensin I-converting enzyme and advanced diabetic retinopathy	Matsumoto A et al.	11106834	Not a renal disease focus
1	Genotypic interactions of renin-angiotensin system genes with diabetes type 2 in a Tunisian population	Mehri S et al.	20580725	Not a renal disease focus

For peer review only

1	Angiotensin II type 1 receptor gene polymorphism and the response to hyperglycemia in early type 1 diabetes	Miller JA et al.	10969844	Not a renal disease focus
1	Renal ACE immunohistochemical localization in NIDDM patients with nephropathy	Mizuiri S et al.	9469501	Not a renal disease focus
1	Randomised controlled trial of dual blockade of renin-angiotensin system in patients with hypertension, microalbuminuria, and non-insulin dependent diabetes: the candesartan and Lisinopril microalbuminuria (CALM) study	Mogensen CE et al.	11110735	Not a renal disease focus
1	Association of Polymorphisms in Endothelial Nitric Oxide Synthesis and Renin-Angiotensin-Aldosterone System with Developing of Coronary Artery Disease in Bulgarian Patients	Mokretar K et al.	26670794	Not a renal disease focus
1	Contribution of angiotensin I converting enzyme gene polymorphism and angiotensinogen gene polymorphism to blood pressure regulation in essential hypertension.	Mondorf UF et al.	9524045	Not a renal disease focus
1	Inhibition of tissue angiotensin converting enzyme activity prevents malignant hypertension in TGR(mREN2)27.	Montgomery HE et al.	9797175	Not a renal disease focus
1	[Possible pathogenetic role of 11 beta-hydroxysteroid dehydrogenase type 1 (11betaHSD1) gene polymorphisms in arterial hypertension].	Morales MA et al.	18769825	Not a renal disease focus
1	Inefficient arterial hypertension control in patients with metabolic syndrome and its link to renin-angiotensin-aldosterone system polymorphisms	Morales-Suarez-Varela MM et al.	21471972	Not a renal disease focus
1	Trientine and renin-angiotensin system blockade ameliorate progression of glomerular morphology in hypertensive experimental diabetic nephropathy.	Moya-Olano L et al.	22029676	Not a renal disease focus
1	Is the influence of variation in the ACE gene on the prospective risk of Type 2 diabetes in middle-aged men modified by obesity?	Muthumala A et al.	17624939	Not a renal disease focus
1	Evaluation Of The Sa Locus In Human Hypertension	Nabika T et al.	7843754	Not a renal disease focus
1	Atrial Natriuretic Peptide Locally Counteracts the Deleterious Effects of Cardiomyocyte Mineralocorticoid Receptor Activation	Nakagawa H et al.	25027872	Not a renal disease focus

For peer review only

1	Association between RAS gene polymorphisms (ACE I/D, AGT M235T) and Henoch-Schonlein purpura in a Turkish population	Nalbantoglu S et al.	23151617	Not a renal disease focus
1	Urine exosomes from healthy and hypertensive pregnancies display elevated level of alpha-subunit and cleaved alpha- and gamma-subunits of the epithelial sodium channel-ENaC	Nielsen MR et al.	28405801	Not a renal disease focus
1	Angiotensin Converting Enzyme Gene Insertion/Deletion Variant and Familial Mediterranean Fever-related Amyloidosis.	Nursal AF et al.	29891744	Not a renal disease focus
1	Genetic polymorphisms of the renin-angiotensin system and atheromatous renal artery stenosis	Olivieri O et al.	10567188	Not a renal disease focus
1	Three Reportedly Unrelated Families With Liddle Syndrome Inherited From a Common Ancestor.	Pagani L et al.	29229744	Not a renal disease focus
1	Endothelial nitric oxide synthase gene/gender interactions and the renal hemodynamic response to angiotensin II	Page A et al.	16093452	Not a renal disease focus
1	The alpha-adducin Gly460Trp polymorphism and the antihypertensive effects of exercise among men with high blood pressure.	Pescatello LS et al.	17472579	Not a renal disease focus
1	Impact of maternal angiotensinogen M235T polymorphism and angiotensin-converting enzyme insertion/deletion polymorphism on blood pressure, protein excretion and fetal outcome in pregnancy.	Pfab T et al.	17563539	Not a renal disease focus
1	The state and responsiveness of the renin-angiotensin-aldosterone system in patients with type II diabetes mellitus	Price DA et al.	10232494	Not a renal disease focus
1	Angiotensin-converting enzyme gene polymorphism in patients with systemic lupus.	Prkacin I et al.	11505631	Not a renal disease focus
1	Long-term follow-up of patients with Bartter syndrome type I and II.	Puricelli E et al.	20219833	Not a renal disease focus
1	The relationship between ACE/AGT gene polymorphisms and the risk of diabetic retinopathy in Chinese patients with type 2 diabetes.	Qiao YC et al.	29378484	Not a renal disease focus
1	Possible role for nephron-derived angiotensinogen in angiotensin-II dependent hypertension	Ramkumar N et al.	26755736	Not a renal disease focus

For peer review only

1	A new mutation, R563Q, of the beta subunit of the epithelial sodium channel associated with low-renin, low-aldosterone hypertension	Rayner BL et al.	12714866	Not a renal disease focus
1	Network-based regularization for high dimensional SNP data in the case-control study of Type 2 diabetes.	Ren J et al.	28511641	Not a renal disease focus
1	Pigsties near dwellings as a potential risk factor for the prevalence of Japanese encephalitis virus in adult in Shanxi, China.	Ren X et al.	28592296	Not a renal disease focus
1	Association of angiotensinogen M235T and A(-6)G gene polymorphisms with coronary heart disease with independence of essential hypertension: the PROCAGENE study. Prospective Cardiac Gene.	Rodriquez-Perez JC et al.	11345362	Not a renal disease focus
1	A clinical phenotype mimicking essential hypertension in a newly discovered family with Liddle's syndrome.	Rossi E et al.	21525970	Not a renal disease focus
1	Vitamin D Deficiency in the Pathogenesis of Hypertension: Still an Unsettled Question	Rostand SG et al.	24929953	Not a renal disease focus
1	Amelioration of genetic hypertension by suppression of renal G protein-coupled receptor kinase type 4 expression	Sanada H et al.	16636192	Not a renal disease focus
1	Prevalence of angiotensin converting enzyme (ACE) gene insertion/deletion polymorphism in South Indian population with hypertension and chronic kidney disease.	Shanmuganathan R et al.	26440392	Not a renal disease focus
1	Novel SLC12A3 mutations in Chinese patients with Gitelman's syndrome.	Shao L et al.	18287808	Not a renal disease focus
1	Mechanisms of suppression of renal kallikrein activity in low renin essential hypertension and renoparenchymal hypertension.	Shimamoto K et al.	2676859	Not a renal disease focus
1	Systemic nitric oxide clamping in normal humans guided by total peripheral resistance	Simonsen JA et al.	19785629	Not a renal disease focus
1	Exaggerated natriuresis during clamping of systemic NO supply in healthy young men	Simonsen JA et al.	21749320	Not a renal disease focus

For peer review only

1	Angiotensin-converting enzyme gene I/D polymorphism increases the susceptibility to hypertension and additive diseases: A study on North Indian patients.	Singh M et al.	27030424	Not a renal disease focus
1	Renal haemodynamics are not related to genotypes in offspring of parents with essential hypertension	Skov K et al.	17083073	Not a renal disease focus
1	Association between renin-angiotensin-aldosterone system-related genes and blood pressure in a Korean population.	Song SB et al.	21342026	Not a renal disease focus
1	Phenotype-genotype interactions on renal function in type 2 diabetes: an analysis using structural equation modelling	Song XY et al.	19479237	Not a renal disease focus
1	Alpha Adducin G460T Variant is a Risk Factor for Hypertension in Tunisian Population	Soualmia H et al.	27349000	Not a renal disease focus
1	Angiotensin II sensitivity in nonpregnant formerly preeclamptic women and healthy parous controls	Spaanderman MEA et al.	15350256	Not a renal disease focus
1	Angiotensin II Type 1A Receptors in Vascular Smooth Muscle Cells Do Not Influence Aortic Remodeling in Hypertension	Sparks MA et al.	21242463	Not a renal disease focus
1	Association between polymorphisms of the renin-angiotensin system and more severe histological forms of lupus nephritis	Sprovieri SRS et al.	16047641	Not a renal disease focus
1	Wilms' tumor protein (-KTS) modulates renin gene transcription	Steege A et al.	18496514	Not a renal disease focus
1	Angiotensin-converting enzyme gene I/D polymorphism in malignant hypertension	Stefansson B et al.	10855732	Not a renal disease focus
1	Transforming growth factor-beta(1) hyperexpression in African-American hypertensives: A novel mediator of hypertension and/or target organ damage	Suthanthiran M et al.	10725360	Not a renal disease focus
1	A functional variant of the NEDD4L gene is associated with beneficial treatment response with Î²-blockers and diuretics in hypertensive patients.	Svensson-Farbom P et al.	21052022	Not a renal disease focus
1	Genetic variants in hypertensive patients with coronary artery disease and coexisting atheromatous renal artery stenosis	Szperl M et al.	19043368	Not a renal disease focus

For peer review only

1	Melatonin prevents maternal fructose intake-induced programmed hypertension in the offspring: roles of nitric oxide and arachidonic acid metabolites	Tain YL et al.	24867192	Not a renal disease focus
1	Genetic variants in the inositol phosphate metabolism pathway and risk of different types of cancer.	Tan J et al.	25683757	Not a renal disease focus
1	Renin in blood vessels in human pulmonary tumors. An immunohistochemical and biochemical study.	Taylor GM et al.	2450464	Not a renal disease focus
1	Incidence of renal failure and nephroprotection by RAAS inhibition in heterozygous carriers of X-chromosomal and autosomal recessive Alport mutations.	Temme J et al.	22237748	Not a renal disease focus
1	Peripheral vascular disease in type 2 diabetic Chinese patients: associations with metabolic indices, concomitant vascular disease and genetic factors	Thomas GN et al.	14632699	Not a renal disease focus
1	Albuminuria and the renin-angiotensin system gene polymorphisms in type-2-diabetic and in normoglycemic hypertensive Chinese	Thomas GN et al.	11200871	Not a renal disease focus
1	Middle cerebral artery stenosis in type II diabetic Chinese patients is associated with conventional risk factors but not with polymorphisms of the renin-angiotensin system genes	Thomas GN et al.	12865608	Not a renal disease focus
1	Genetic variation in the KCNMA1 potassium channel alpha subunit as risk factor for severe essential hypertension and myocardial infarction	Tomas M et al.	18854754	Not a renal disease focus
1	Renal Mechanisms of Association between Fibroblast Growth Factor 1 and Blood Pressure	Tomaszewski M et al.	25918036	Not a renal disease focus
1	Angiotensin II-dependent chronic hypertension and cardiac hypertrophy are unaffected by gp91phox-containing NADPH oxidase	Touyz RM et al.	15753233	Not a renal disease focus
1	Renin Production In Congenital Mesoblastic Nephroma In Comparison With That In Wilms-Tumor	Tsuchida Y et al.	8385325	Not a renal disease focus
1	Genomic association analysis identifies multiple loci influencing antihypertensive response to an angiotensin II receptor blocker.	Turner ST et al.	22566498	Not a renal disease focus

For peer review only

1	Angiotensin-converting enzyme gene polymorphism and vascular manifestations in Korean patients with SLE	Uhm WS et al.	12043886	Not a renal disease focus
1	The M235T polymorphism in theangiotensinogen gene is associated with the risk of malignant hypertension in white patients	van den Born BJH et al.	17921816	Not a renal disease focus
1	Genetic risk of atherosclerotic renal artery disease - The candidate gene approach in a renal angiography cohort	van Onna M et al.	15326089	Not a renal disease focus
1	Activation of the hypothalamic-pituitary-adrenal axis in adults with mineralocorticoid receptor haploinsufficiency.	Walker BR et al.	24712576	Not a renal disease focus
1	Liver pyruvate kinase polymorphisms are associated with type 2 diabetes in northern European Caucasians.	Wang H et al.	12196482	Not a renal disease focus
1	miR149 rs71428439 polymorphism and risk of clear cell renal cell carcinoma: a case-control study.	Wang Z et al.	25213695	Not a renal disease focus
1	Genotype-phenotype analysis of angiotensinogen polymorphisms and essential hypertension: the importance of haplotypes	Watkins WS et al.	19770777	Not a renal disease focus
1	Angiotensin-converting enzyme gene does not contribute to genetic susceptibility to systemic sclerosis in European Caucasians.	Wipff J et al.	19132786	Not a renal disease focus
1	Androgen-Sensitive Hypertension Associates with Upregulated Vascular CYP4A12-20-HETE Synthase	Wu CC et al.	23641057	Not a renal disease focus
1	Urinary UMOD excretion and chronic kidney disease in gout patients: cross-sectional case-control study.	Wu CH et al.	21332338	Not a renal disease focus
1	Unknown face of known drugs - what else can we expect from angiotensin converting enzyme inhibitors?	Wzgarda A et al.	28087255	Not a renal disease focus
1	Malignant Nephrosclerosis in a Patient with Familial Mediterranean Fever	Yamanouchi M et al.	26466703	Not a renal disease focus
1	Gene polymorphism of vascular endothelial growth factor-1154 G > A is associated with hypertensive nephropathy in a Hispanic population	Yang JW et al.	21080079	Not a renal disease focus
1	TBX6 compound inheritance leads to congenital vertebral malformations in humans and mice.	Yang N et al.	30307510	Not a renal disease focus

For peer review only

1	Identification of diuretic non-responders with poor long-term clinical outcomes: a 1-year follow-up of 176 non-azotaemic cirrhotic patients with moderate ascites	Yang YY et al.	21692745	Not a renal disease focus
1	Renal redox-sensitive signaling, but not blood pressure, is attenuated by Nox1 knockout in angiotensin II-dependent chronic hypertension	Yogi A et al.	18195161	Not a renal disease focus
1	Angiotensin II type 2 receptor gene is not responsible for familial vesicoureteral reflux	Yoneda A et al.	12187255	Not a renal disease focus
1	Angiotensinogen T235 and ACE insertion/deletion polymorphisms associated with albuminuria in Chinese type 2 diabetic patients	Young RP et al.	9540028	Not a renal disease focus
1	Common genetic variants in the chromogranin a promoter are associated with renal injury in IgA nephropathy patients with malignant hypertension.	Yu L et al.	20113265	Not a renal disease focus
1	Frequencies Of Variants Of Candidate Genes In Different Age-Groups Of Hypertensives	Zee RY et al.	7882587	Not a renal disease focus
1	Association of angiotensin-converting enzyme gene polymorphisms with Crohn's disease in a Chinese Han population	Zhou J et al.	26823847	Not a renal disease focus
1	Molecular variants of the sodium/hydrogen exchanger type 3 gene and essential hypertension	Zhu HD et al.	15201541	Not a renal disease focus
1	[Renin-angiotensin system genes in chronic glomerulonephritis].	Buraczynska M et al.	11865575	Not English or Spanish
1	[Association of the renin-angiotensin system gene polymorphism with nephropathy in type II diabetes].	Buraczynska M et al.	12476891	Not English or Spanish
1	[Genetic predisposition to systemic complications of arterial hypertension in maintenance haemodialysis patients].	Bzoma B et al.	19112833	Not English or Spanish
1	[Arterial hypertension and chronic hemodialysis].	Ermolenko VM et al.	7700	Not English or Spanish
1	[Mutations in NPHS2 in familial steroid-resistant nephrotic syndrome in Southern Chinese Han ethnic group].	Fu R et al.	19099831	Not English or Spanish

1	[Is PstI polymorphism of the angiotensin I converting enzyme gene associated with nephropathy development in non-insulin-dependent diabetes mellitus (preliminary study)].	Grzeszczak W et al.	9499204	Not English or Spanish
1	[Angiotensin-converting enzyme gene polymorphism and the clinical pathological features and progression in lupus nephritis].	Guan T et al.	10436947	Not English or Spanish
1	[Angiotensin-converting enyme insertion/deletion polymorphism and blood pressure regulation in type 2 diabetic patients].	Krajina-Andricevic M et al.	23120809	Not English or Spanish
1	[Relationship between I/D polymorphism of angiotensin I converting enzyme gene and microvascular complications in type 2 diabetic patients].	Moleda P et al.	17941464	Not English or Spanish
1	[Genomics of type 1 diabetes mellitus and its late complications]	Nosikov VV	15042845	Not English or Spanish
1	[Correlation between HLA-DQA1 allele and anaphylactoid purpura in juvenile Hans residing in Inner Mongolia].	Ren S et al.	11836690	Not English or Spanish
1	[Correlation of cyclin D1 overexpression to mutations of von hippel-lindau gene in renal clear cell carcinoma].	Ren YY et al.	16480581	Not English or Spanish
1	[Morphofunctional characteristics of endocrine nephropathy in primary aldosteronism].	Sokolova RI et al.	2678677	Not English or Spanish
1	[Gene mutation analysis of X-linked hypophosphatemic rickets].	Song Y et al.	24229582	Not English or Spanish
1	[A novel COL4A5 splicing mutation causing Alport syndrome in a Chinese family].	Tang Z et al.	19065523	Not English or Spanish
1	[Association of single nucleotide polymorphism of megsin gene with IgA nephropathy].	Wang ZH et al.	16796905	Not English or Spanish
1	[Study on the association between the polymorphism of HLA-DQA1 alleles and type 2 diabetes in Yunnan Han nationality].	Yang HY et al.	15192842	Not English or Spanish
1	DD Genotype of ACE Gene in Boys: May it be a Risk Factor for Minimal Change Nephrotic Syndrome?	Alasehirli B et al.	22017506	Paediatric individuals
1	Angiotensin-converting enzyme gene insertion/deletion polymorphism and renal damage in childhood uropathies	Al-Eisa A et al.	10986863	Paediatric individuals

1	Angiotensin converting enzyme gene polymorphism in Asian Indian children with congenital uropathies	Bajpai M et al.	14713838	Paediatric individuals
1	Posterior urethral valves: Preliminary observations on the significance of plasma renin activity as a prognostic marker	Bajpai M et al.	15643266	Paediatric individuals
1	Nitric oxide synthase gene polymorphisms in children with primary nocturnal enuresis: a preliminary study.	Balat A et al.	17365914	Paediatric individuals
1	MCP1 2518 A/G polymorphism affects progression of childhood focal segmental glomerulosclerosis.	Besbas N et al.	26335292	Paediatric individuals
1	Heterozygous Loss-of-Function SEC61A1 Mutations Cause Autosomal-Dominant Tubulo-Interstitial and Glomerulocystic Kidney Disease with Anemia	Bolar NA et al.	27392076	Paediatric individuals
1	Donor and recipient ACE I/D genotype are associated with loss of renal function in children following renal transplantation	Buscher R et al.	21309964	Paediatric individuals
1	ACE gene polymorphism in Turkish children with nephrotic syndrome.	Celik US et al.	16825089	Paediatric individuals
1	Increased HLA- A*11 in Chinese children with steroid-responsive nephrotic syndrome.	Cheung W et al.	11956863	Paediatric individuals
1	Liddle syndrome: clinical and genetic profiles.	Cui Y et al.	27896928	Paediatric individuals
1	NPHS2 variation in Chinese southern infants with late steroid-resistant nephrotic syndrome.	Dai Y et al.	25112471	Paediatric individuals
1	Earlier Onset of Complications in Youth With Type 2 Diabetes	Dart AB et al.	24130346	Paediatric individuals
1	ACE gene polymorphism in Egyptian children with idiopathic nephrotic syndrome	Fahmy ME et al.	18792483	Paediatric individuals
1	Genetic polymorphisms of the renin-angiotensin system and the outcome of focal segmental glomerulosclerosis in children	Frishberg Y et al.	9853248	Paediatric individuals
1	Angiotensinogen gene T235 variant: a marker for the development of persistent microalbuminuria in children and adolescents with type 1 diabetes mellitus	Gallego PH et al.	18413222	Paediatric individuals

For peer review only

1	Autosomal dominant pseudohypoaldosteronism type 1: Mechanisms, evidence for neonatal lethality, and phenotypic expression in adults	Geller DS et al.	16611713	Paediatric individuals
1	The role of vitamin D receptor gene polymorphisms in Turkish infants with urolithiasis.	Goknar N et al.	26908058	Paediatric individuals
1	Risk factors for loss of residual renal function in children treated with chronic peritoneal dialysis	Ha IS et al.	25874598	Paediatric individuals
1	Implication of genetic variations in congenital obstructive nephropathy	Hahn H et al.	16133060	Paediatric individuals
1	ACE gene polymorphism and renal scarring in primary vesicoureteric reflux	Haszon I et al.	12478352	Paediatric individuals
1	Impact of ACE I/D gene polymorphism on congenital renal malformations	Hohenfellner K et al.	11354781	Paediatric individuals
1	Impact of common functional polymorphisms in renin angiotensin system genes on the risk of renal parenchymal scarring following childhood urinary tract infection	Hussein A et al.	25939993	Paediatric individuals
1	An insertion/deletion ACE polymorphism and kidney size in Polish full-term newborns	Kaczmarczyk M et al.	22674971	Paediatric individuals
1	ACE and AT1 receptor gene polymorphisms and renal scarring in urinary bladder dysfunction	Kostic M et al.	15179569	Paediatric individuals
1	Renal tubular dysgenesis, a not uncommon autosomal recessive disorder leading to oligohydramnios: Role of the Renin-Angiotensin system.	Lacoste M et al.	16790508	Paediatric individuals
1	Renin-angiotensin system gene polymorphisms in children with Henoch-Schonlein purpura in West China	Liu DS et al.	20702504	Paediatric individuals
1	Renin-angiotensin system polymorphisms in Taiwanese primary vesicoureteral reflux	Liu KP et al.	15045574	Paediatric individuals
1	Polymorphisms of renin-angiotensin system genes in childhood IgA nephropathy	Maruyama K et al.	11354780	Paediatric individuals
1	Association of angiotensin type 2 receptor gene polymorphisms with ureteropelvic junction obstruction in Brazilian patients	Miranda DM et al.	24995698	Paediatric individuals

For peer review only

1	Association of angiotensin converting enzyme and angiotensin type 2 receptor gene polymorphisms with renal damage in posterior urethral valves	Narasimhan KL et al.	WOS:000290840100006	Paediatric individuals
1	Prioritization and burden analysis of rare variants in 208 candidate genes suggest they do not play a major role in CAKUT	Nicolaou N et al.	26489027	Paediatric individuals
1	ACE I/D gene polymorphism in primary FSGS and steroid-sensitive nephrotic syndrome	Oktem F et al.	14986085	Paediatric individuals
1	Implications of certain genetic polymorphisms in scarring in vesicoureteric reflux: importance of ACE polymorphism.	Ozen S et al.	10401028	Paediatric individuals
1	Renin-angiotensin system gene polymorphisms: association with susceptibility to Henoch-Schonlein purpura and renal involvement	Ozkaya O et al.	16521052	Paediatric individuals
1	Renin-angiotensin gene polymorphism in children with uremia and essential hypertension	Papp F et al.	12579405	Paediatric individuals
1	A rare case of juvenile hypertension: coexistence of type 2 multiple endocrine neoplasia -related bilateral pheochromocytoma and reninoma in a young patient with ACE gene polymorphism	Paragliola RM et al.	26084817	Paediatric individuals
1	Renin-angiotensin system polymorphisms and renal scarring	Pardo R et al.	12579398	Paediatric individuals
1	Low renin-angiotensin system activity gene polymorphism and dysplasia associated with posterior urethral valves	Peruzzi L et al.	16006956	Paediatric individuals
1	Effect of angiotensin-converting enzyme gene insertion/deletion polymorphism on steroid resistance in Egyptian children with idiopathic nephrotic syndrome.	Saber-Ayad M et al.	20418353	Paediatric individuals
1	ACE gene insertion/deletion polymorphism in childhood idiopathic nephrotic syndrome	Serdaroglu E et al.	16208534	Paediatric individuals
1	Effects of genetic polymorphisms of the renin-angiotensin system in children with nephrotic syndrome	Tabel Y et al.	16525944	Paediatric individuals
1	Genetic risk factors in typical haemolytic uraemic syndrome	Taranta A et al.	19110485	Paediatric individuals

For peer review only

1	The juxtaglomerular apparatus in Bartter's syndrome and related tubulopathies. An immunocytochemical and electron microscopic study.	Taugner R et al.	3128915	Paediatric individuals
1	Mapping candidate regions and genes for congenital anomalies of the kidneys and urinary tract (CAKUT) by array-based comparative genomic hybridization	Weber S et al.	20605837	Paediatric individuals
1	Genetic polymorphism of the renin-angiotensin system on the development of primary vesicoureteral reflux	Yim HE et al.	14764974	Paediatric individuals
1	The association of endothelial nitric oxide synthase gene single nucleotide polymorphisms with paediatric systemic lupus erythematosus	Zhu J et al.	29465350	Paediatric individuals
1	Add-on angiotensin receptor blockade with maximized ACE inhibition	Agarwal R	11380832	Pharmaceutical drug focus
1	DPP-4 Inhibition on Top of Angiotensin Receptor Blockade Offers a New Therapeutic Approach for Diabetic Nephropathy	Alter ML et al.	23171828	Pharmaceutical drug focus
1	Effects of losartan and enalapril on high-sensitivity C-reactive protein and total antioxidant in renal transplant recipients with renin-angiotensin system polymorphisms	Argani H et al.	18261537	Pharmaceutical drug focus
1	Losartan decreases plasma levels of TGF-beta 1 in transplant patients with chronic allograft nephropathy	Campistol JM et al.	10432413	Pharmaceutical drug focus
1	Captopril enhances transforming growth factor (TGF)-beta1 expression in peripheral blood mononuclear cells: a mechanism independent from angiotensin converting enzyme inhibition? A study in cyclosporine-treated kidney-transplanted patients.	Di Paolo S et al.	12499886	Pharmaceutical drug focus
1	Thiazolidinediones and the renal and hormonal response to water immersion-induced volume expansion in type 2 diabetes mellitus	Goenka N et al.	18230694	Pharmaceutical drug focus
1	The influence of renin-angiotensin system genotypes on the antiproteinuric response to high doses of olmesartan in non-diabetic protein uric nephropathies	Goyache-Goni B et al.	24241364	Pharmaceutical drug focus
1	Antiproteinuric effect of candesartan cilexetil in Japanese subjects with type 2 diabetes and nephropathy	Haneda M et al.	15364166	Pharmaceutical drug focus

For peer review only

1	Renal implications of angiotensin receptor blockers	Hollenberg NK	11459212	Pharmaceutical drug focus
1	The influence of the ACE (I/D) polymorphism on systemic and renal vascular responses to angiotensins in normotensive, normoalbuminuric Type 1 diabetes mellitus	Luik PT et al.	12856080	Pharmaceutical drug focus
1	Application of Direct Renin Inhibition to Chronic Kidney Disease	Mende CW	20490905	Pharmaceutical drug focus
1	Pentoxifylline for Renoprotection in Diabetic Nephropathy: the PREDIAN study. Rationale and basal results	Navarro-Gonzalez JF et al.	21144773	Pharmaceutical drug focus
1	Renin-angiotensin system polymorphisms and hemoglobin level in renal allografts: A comparative study between losartan and enalapril	Noroozianavval M et al.	17524880	Pharmaceutical drug focus
1	Enalapril and losartan affect lipid peroxidation in renal transplant recipients with renin-angiotensin system polymorphisms	Rashtchizadeh N et al.	17222813	Pharmaceutical drug focus
2	Relationship between the renin-angiotensin system genes and diabetic nephropathy in the Chinese.	Wu S et al.	11776100	No data
3	Plasma renin and prorenin and renin gene variation in patients with insulin-dependent diabetes mellitus and nephropathy.	Deinum J et al.	10462269	< 3 populations reported per SNP
3	Renin–angiotensin–aldosterone system genotypes and haplotypes affect the susceptibility to nephropathy in type 2 diabetes patients	Mtiraoui N et al.	21421655	< 3 populations reported per SNP
3	Chronic renal insufficiency among Asian Indians with type 2 diabetes: I. Role of RAAS gene polymorphisms	Prasad P et al.	16672053	< 3 populations reported per SNP
3	Renin gene rs1464816 polymorphism contributes to chronic kidney disease progression in ADPKD.	Ramanathan G et al.	26753721	< 3 populations reported per SNP
3	M235T polymorphism in the AGT gene and A/G(I8-83) substitution in the REN gene correlate with end-stage renal disease	Sarkar S et al.	25660845	< 3 populations reported per SNP

For peer review only

Supplementary Table S4: Data included in quantitative analysis for *ACE*

				Cases				Controls			
Author	PMID/ WOS ID	Ethnicity	SNP	Definition	N	I Frequency	D Frequency	Definition	N	I Frequency	D Frequency
Anbazhagan K et al.	19520069	SAS	Ins/Del	CKD	118	124	112	Healthy	98	99	97
Chen WJ et al.	24907556	SAS	Ins/Del	CKD	233	314	152	Healthy	449	617	281
Nagamani S et al.	None	SAS	Ins/Del	CKD	147	152	142	Healthy	221	195	227
Shanmuganathan R et al.	26440392	SAS	Ins/Del	CKD	30	10	50	Healthy	30	55	5
Ali A et al.	21421653	EAS	Ins/Del	ESRD	190	219	161	Healthy	190	262	118
Kawada N et al.	000071880400005	EAS	Ins/Del	ESRD	216	280	152	Healthy	208	264	152
Su SL et al.	24977181	EAS	Ins/Del	ESRD	647	792	502	Healthy	644	859	429
Tang FY et al.	18629615	EAS	Ins/Del	ESRD	153	100	206	Healthy	148	138	158
Wang AY et al.	12675870	EAS	Ins/Del	ESRD	246	316	176	Healthy	183	230	136
Yang HY et al.	23477970	EAS	Ins/Del	ESRD	683	833	533	Healthy	653	867	439
Buraczynska M et al.	16384824	EUR	Ins/Del	ESRD	745	688	802	Healthy	520	492	548
Dixit M et al.	12571380	EUR	Ins/Del	ESRD	26	32	20	Healthy	22	17	27
Losito A et al.	12454231	EUR	Ins/Del	ESRD	160	135	185	Healthy	169	128	210
McLaughlin KJ et al.	8901844	EUR	Ins/Del	ESRD	460	654	990	Healthy	371	353	389
Nicod J et al.	11918733	EUR	Ins/Del	ESRD	260	282	238	Healthy	260	261	259
Ortiz MA et al.	15012717	EUR	Ins/Del	ESRD	117	68	166	Healthy	129	80	178
Schmidt A et al.	8785402	EUR	Ins/Del	ESRD	106	105	107	Healthy	95	80	110
van der Sman-de Beer F et al.	16221224	EUR	Ins/Del	ESRD	415	400	430	Healthy	472	459	485
Aucella F et al.	12748347	EUR	Ins/Del	ESRD	461	315	607	Healthy	1307	916	1698
Abuaisha AM et al.	000433049000003	ME	Ins/Del	ESRD	86	37	135	Healthy	100	50	150
Rahimi Z et al.	28447048	ME	Ins/Del	ESRD	99	64	126	Healthy	117	89	145
Zaare Nahandi et al.	28270648	ME	Ins/Del	ESRD	30	23	37	Healthy	27	21	33

For peer review only

Guo Y et al.	00038665 3400063	EAS	Ins/Del	IgAN	45	54	36	Healthy	45	42	48
Huang HD et al.	21163122	EAS	Ins/Del	IgAN	130	155	105	Healthy	120	168	72
Jung ES et al.	21150220	EAS	Ins/Del	IgAN	261	298	224	Healthy	300	360	240
Lau YK et al.	15153745	EAS	Ins/Del	IgAN	118	154	82	Healthy	94	137	51
Suzuki H et al.	15481848	EAS	Ins/Del	IgAN	319	378	260	Healthy	270	328	212
Yoon HJ et al.	12220450	EAS	Ins/Del	IgAN	191	204	178	Healthy	233	268	198
Yorioka T et al.	8529313	EAS	Ins/Del	IgAN	48	67	29	Healthy	104	139	67
Yoshida H et al.	7593601	EAS	Ins/Del	IgAN	53	57	49	Healthy	46	62	30
Burg M et al.*	9352153	EUR	Ins/Del	IgAN	70	26	28	Healthy	60	60	60
Drouet M et al.	12005241	EUR	Ins/Del	IgAN	125	78	172	Healthy	83	54	100
Harden PN et al.	7791440	EUR	Ins/Del	IgAN	100	79	121	Healthy	98	76	120
Pawlik M et al.*	23681285	EUR	Ins/Del	IgAN	31	25	37	Healthy	187	180	194
Pei Y et al.	9259580	EUR	Ins/Del	IgAN	168	145	191	Healthy	100	91	109
Schmidt S et al. (B)	7485124	EUR	Ins/Del	IgAN	204	168	240	Healthy	234	197	271
Stratta P et al.	10352195	EUR	Ins/Del	IgAN	81	62	100	Healthy	50	38	62
Burg M et al.*	9352153	EUR	Ins/Del	Primary GN	46	44	48	Healthy	60	60	60
Pawlik M et al.*	23681285	EUR	Ins/Del	Primary GN	109	101	117	Healthy	187	180	194
Stratta P et al.	14767013	EUR	Ins/Del	Primary GN	117	96	138	Organ donors	171	134	208
Zsom M et al.	22111818	EUR	Ins/Del	Primary GN	73	62	84	Healthy	200	198	202
Beige J et al.	9259361	EUR	Ins/Del	RTx recipients	269	251	287	Kidney Donor	269	244	294
El-Essawy AB et al.	11926202	EUR	Ins/Del	RTx recipients	294	246	342	Healthy	181	170	192
Hueso M et al.	15284309	EUR	Ins/Del	RTx recipients	180	122	238	Healthy & cadaveric renal	113	96	130

For peer review only

								allograft donors			
Stratta P et al.	19034872	EUR	Ins/Del	RTx recipients	169	122	216	Healthy	169	112	226
Viklický O et al.	11239522	EUR	Ins/Del	RTx recipients	30	28	32	Healthy	653	599	707
Barnas U et al.	9084972	EUR	Ins/Del	T1DN	63	45	55	T1DM	59	51	29
Chowdhury TA et al.	8877296	EUR	Ins/Del	T1DN	242	204	280	T1DM	166	143	189
Currie D et al.	20854388	EUR	Ins/Del	T1DN	718	603	777	T1DM	749	696	764
De Cosmo S et al.	10495473	EUR	Ins/Del	T1DN	175	125	225	T1DM	136	89	183
Demurov LM et al.	A1997XE98500008	EUR	Ins/Del	T1DN	56	35	77	T1DM	76	72	80
Hadjadj S et al.	11181802	EUR	Ins/Del	T1DN	6	4	8	T1DM	251	208	294
Hibberd ML et al.	9025006	EUR	Ins/Del	T1DN	72	60	84	T1DM	86	57	115
Marre M et al.	8314010	EUR	Ins/Del	T1DN	62	43	81	T1DM	62	58	66
Ringel J et al.*	9049480	EUR	Ins/Del	T1DN	134	130	138	T1DM	226	208	244
Schmidt S et al. (A)	7783416	EUR	Ins/Del	T1DN	114	86	142	T1DM	133	101	165
Shestakova MV et al.	000242410600010	EUR	Ins/Del	T1DN	63	65	61	T1DM	66	78	54
Tarnow L et al.	7729604	EUR	Ins/Del	T1DN	198	175	221	T1DM	190	169	211
van Ittersum FJ et al.	10862638	EUR	Ins/Del	T1DN	30	33	27	T1DM	188	192	184
Walder B et al.	None	EUR	Ins/Del	T1DN	55	49	61	T1DM	44	40	48
Fujisawa T et al.	7555560	EAS	Ins/Del	T2DN	54	71	37	T2DM	35	46	24
Ha SK et al.	12722028	EAS	Ins/Del	T2DN	140	132	148	T2DM	99	123	75
Hsieh MC et al.	10862639	EAS	Ins/Del	T2DN	179	219	139	T2DM	157	222	92
Mizuiri S et al.	7477652	EAS	Ins/Del	T2DN	80	72	88	T2DM	31	33	29
Nakajima S et al.	8941475	EAS	Ins/Del	T2DN	54	67	41	T2DM	41	55	27
Ohno T et al.	8596493	EAS	Ins/Del	T2DN	25	28	22	T2DM	53	81	25
Shin Shin Y et al.	15331206	EAS	Ins/Del	T2DN	82	112	52	T2DM	59	70	48
Thomas GN et al.	11200871	EAS	Ins/Del	T2DN	51	72	30	T2DM	255	346	164
Wang M et al.	26973293	EAS	Ins/Del	T2DN	106	78	56	T2DM	741	1020	438
Wu SH et al.	11776100	EAS	Ins/Del	T2DN	71	75	67	T2DM	41	58	24

For peer review only

Young RP et al.	9540028	EAS	Ins/Del	T2DN	20	27	13	T2DM	54	72	36
Dudley CR et al.	8587251	EUR	Ins/Del	T2DN	163	246	288	T2DM	267	246	288
Fradin S et al.	11938025	EUR	Ins/Del	T2DN	39	94	142	T2DM	118	94	142
Ng DP et al.	16936219	EUR	Ins/Del	T2DN	291	147	187	T2DM	167	147	187
Nikzamir A et al.	19502259	EUR	Ins/Del	T2DN	48	159	131	T2DM	145	159	131
Schmidt S et al. (B)	9269698	EUR	Ins/Del	T2DN	311	278	416	T2DM	347	278	416
Al-Harbi EM et al.	21207118	ME	Ins/Del	T2DN	110	63	157	T2DM	196	125	267
Alharbi SA et al.	00041506 3100003	ME	Ins/Del	T2DN	61	31	91	T2DM	61	47	75
Araz M et al.	11640993	ME	Ins/Del	T2DN	62	52	72	T2DM	123	103	143
El-Baz R et al.	22554825	ME	Ins/Del	T2DN	102	66	138	T2DM	100	82	118
Ergen HA et al.	15365253	ME	Ins/Del	T2DN	25	21	29	T2DM	50	31	69
Eroglu Z et al.	18413162	ME	Ins/Del	T2DN	46	43	49	T2DM	56	50	62
Felehgari V et al.	20830509	ME	Ins/Del	T2DN	68	42	94	T2DM	72	60	84
Sancakdar E et al.	00036754 1400005	ME	Ins/Del	T2DN	194	180	208	T2DM	100	62	138
Ahluwalia TS et al.	19108684	SAS	Ins/Del	T2DN	240	152	328	T2DM	200	215	
Kumar R et al.	23846111	SAS	Ins/Del	T2DN	407	448	366	T2DM	185	190	
Movva S et al.	17616353	SAS	Ins/Del	T2DN	174	182	166	T2DM	175	222	
Naresh VV et al.	20535249	SAS	Ins/Del	T2DN	30	19	41	T2DM	30	35	
Shaikh R et al.	00031205 3000001	SAS	Ins/Del	T2DN	168	134	202	T2DM	296	394	
Vishwanathan V et al.	11867868	SAS	Ins/Del	T2DN	86	79	93	T2DM	23	28	
Doi Y et al.	8720609	EAS	Ins/Del	T2DN-ESRD	100	115	85	T2DM	124	168	
Jayapalan JJ et al.	21031056	EAS	Ins/Del	T2DN-ESRD	127	163	91	T2DM	81	93	
Lu M et al.	27633502	EAS	Ins/Del	T2DN-ESRD	210	238	182	T2DM	222	307	
Park HC et al.	16385653	EAS	Ins/Del	T2DN-ESRD	103	103	103	T2DM	88	111	

For peer review only

Taniwaki H et al.	11522715	EAS	Ins/Del	T2DN-ESRD	42	53	31	T2DM	69	88	
Grzeszczak W et al.	9727375	EUR	Ins/Del	T2DN-ESRD	127	128	126	T2DM	254	244	
Ringel J et al.*	9049480	EUR	Ins/Del	T2DN-ESRD	161	150	172	T2DM	140	141	
Schmidt S et al. (A)	9075119	EUR	Ins/Del	T2DN-ESRD	61	29	93	T2DM	347	416	

*Burg M et al., Pawlik M et al., and Ringel J et al., are included twice as these articles contained two phenotypic comparisons

Abbreviations: EAS, East Asian; EUR, European; ME, Middle Eastern; SAS, South Asian; CKD, Chronic Kidney Disease; ESRD, End-Stage Renal Disease; IgAN, IgA Nephropathy; GN, Glomerulonephritis; RTx, Renal Transplant; T1DM, Type 1 Diabetes Mellitus; T1DN, Type 1 Diabetic Nephropathy; T2DM, Type 2 Diabetes Mellitus; T2DN, Type 2 Diabetic Nephropathy.

For peer review only

Supplementary Table S5: Data included in quantitative analysis for *AGT*

				Cases				Controls			
Author	PMID/ WOS ID	Ethnicity	SNP	Definition	N	T Allele Frequency	C Allele Frequency	Definition	N	T Allele Frequency	C Allele Frequency
Beige J et al.	8856207	EUR	rs699	ESRD	269	285	253	Healthy	269	307	229
Buraczynska M et al.	16384824	EUR	rs699	ESRD	745	734	756	Healthy	520	564	476
Losito A et al.	12454231	EUR	rs699	ESRD	160	165	155	Healthy	169	190	148
Lovati E et al.	11422735	EUR	rs699	ESRD	260	299	221	Healthy	327	393	261
Doria A et al.	8621207	EUR	rs699	T1DN	139	158	120	T1DM	75	96	54
Fogarty DG et al.	8772723	EUR	rs699	T1DN	95	114	76	T1DM	100	129	71
Möllsten A et al.	18413189	EUR	rs699	T1DN	73	62	34	T1DM	197	220	174
Ringel J et al.	9049480	EUR	rs699	T1DN	134	145	123	T1DM	226	257	195
Schmidt S et al.*	8918618	EUR	rs699	T1DN	108	121	95	T1DM	120	132	108
van Ittersum FJ et al.	10862638	EUR	rs699	T1DN	30	31	29	T1DM	188	238	138
Walder B et al.	None	EUR	rs699	T1DN	55	60	50	T1DM	44	63	25
Ohno T et al.	8596493	EAS	rs699	T2DN	25	16	34	T2DM	53	25	81
Oue T et al.	10580616	EAS	rs699	T2DN	27	6	36	T2DM	40	13	47
Thomas GN et al.	11200871	EAS	rs699	T2DN	51	18	84	T2DM	255	79	431
Wang M et al.	26973293	EAS	rs699	T2DN	189	19	175	T2DM	473	154	792
Wu SH et al.	11776100	EAS	rs699	T2DN	71	21	121	T2DM	41	26	56
Young RP et al.	9540028	EAS	rs699	T2DN	20	4	36	T2DM	54	25	83
Fradin S et al.	11938025	EUR	rs699	T2DN	39	45	33	T2DM	118	139	97
Freire MB et al.	9535411	EUR	rs699	T2DN	117	134	96	T2DM	125	148	88
Makuc J et al.	28488548	EUR	rs699	T2DN	276	294	258	T2DM	375	394	356
Schmidt S et al.*	8918618	EUR	rs699	T2DN	127	149	105	T2DM	107	117	97
Zychma MJ et al.	11096141	EUR	rs699	T2DN	127	133	121	T2DM	243	242	244
Eroglu Z et al.	18413162	ME	rs699	T2DN	46	48	44	T2DM	56	62	50
Reis KA et al.	21500980	ME	rs699	T2DN	108	114	102	T2DM	111	89	133
Sancakdar E et al.	000367541 400005	ME	rs699	T2DN	194	189	199	T2DM	100	95	105

For peer review only

Gao J et al.	26588355	EAS	rs699	IgAN	351	144	556	Healthy	310	125	495
Guo Y et al.	000386653 400063	EAS	rs699	IgAN	45	19	71	Healthy	45	16	74
Huang HD et al.	21163122	EAS	rs699	IgAN	130	35	225	Healthy	120	40	200
Kim SM et al.	19729965	EAS	rs699	IgAN	238	89	387	Healthy	300	115	485
Lau YK et al.	15153745	EAS	rs699	IgAN	118	27	209	Healthy	94	31	157
Kawada N et al.	000071880 400005	EAS	rs699	ESRD	216	91	333	Healthy	208	64	340
Su SL et al.	29296205	EAS	rs699	ESRD	634	194	1074	Healthy	739	279	1478
Wang AY et al.	12675870	EAS	rs699	ESRD	246	76	416	Healthy	183	64	302

*Schmidt S et al., is included twice as the article contained two phenotypic comparisons.

Abbreviations: EAS, East Asian; EUR, European; ME, Middle Eastern; ESRD, End-Stage Renal Disease; IgAN, IgA Nephropathy; T1DM, Type 1 Diabetes Mellitus; T1DN, Type 1 Diabetic Nephropathy; T2DM, Type 2 Diabetes Mellitus; T2DN, Type 2 Diabetic Nephropathy.

For peer review only

Supplementary Table S6: Data included in quantitative analysis for *AGTR1*

				Cases				Controls			
**Author**	**PMID**	**Ethnicity**	**SNP**	**Definition**	**N**	**A Allele Frequency**	**C Allele Frequency**	**Definition**	**N**	**A Allele Frequency**	**C Allele Frequency**
Gao J et al.	26588355	EAS	rs5186	IgAN	351	659	43	Healthy	310	577	43
Huang HD et al.	21163122	EAS	rs5186	IgAN	130	243	17	Healthy	120	220	20
Kim SM et al.	19729965	EAS	rs5186	IgAN	238	452	24	Healthy	300	576	24
Lau YK et al.	15153745	EAS	rs5186	IgAN	118	227	9	Healthy	94	178	10
Currie D et al.	20854388	EUR	rs5186	T1DN	718	1029	385	T1DM	749	1052	418
Doria A et al.	9389421	EUR	rs5186	T1DN	73	99	47	T1DM	79	119	39
Möllsten A et al.	18413189	EUR	rs5186	T1DN	72	78	18	T1DM	197	290	104
Savage DA et al.	10328465	EUR	rs5186	T1DN	95	136	54	T1DM	97	137	57
Tarnow L et al.	8671962	EUR	rs5186	T1DN	198	287	109	T1DM	190	274	106
van Ittersum FJ et al.	10862638	EUR	rs5186	T1DN	30	47	13	T1DM	188	238	138
Thomas GN et al.	11200871	EAS	rs5186	T2DN	51	99	3	T2DM	255	483	27
Wu SH et al.	11776100	EAS	rs5186	T2DN	71	127	15	T2DM	41	74	8
Young RP et al.	9540028	EAS	rs5186	T2DN	20	38	2	T2DM	54	103	5
Ahluwalia TS et al.	19108684	SAS	rs5186	T2DN	240	320	160	T2DM	255	381	129
Prasad P et al.	16672053	SAS	rs5186	T2DN	196	360	26	T2DM	225	417	33
Shah VN et al.	23081748	SAS	rs5186	T2DN	596	789	403	T2DM	566	840	282

Abbreviations: EAS, East Asian; EUR, European; SAS, South Asian; IgAN, IgA Nephropathy; T1DM, Type 1 Diabetes Mellitus; T1DN, Type 1 Diabetic Nephropathy; T2DM, Type 2 Diabetes Mellitus; T2DN, Type 2 Diabetic Nephropathy.

For peer review only

Supplementary Table S7. Included studies not complying with Hardy Weinberg equilibrium in cases or controls

					Cases		Controls	
**Gene**	**Author**	**PMID/ WODS ID**	**Ethnicity**	**SNP**	**Definition**	**P value (where significant)**	**Definition**	**P value (where significant)**
ACE	Jung ES et al.	21150220	EAS	Ins/Del	IgAN		Healthy	0.016
	Shanmuganathan R et al.	26440392	SAS	Ins/Del	CKD	$1.31x10^{-04}$	Healthy	$1.9x10^{-05}$
	Tang FY et al.	18629615	EAS	Ins/Del	ESRD	$1.8x10^{-05}$	Healthy	$1x10^{-06}$
	Nicod J et al.	11918733	EUR	Ins/Del	ESRD	0.0008	Healthy	0.004
	McLaughlin KJ et al.	8901844	EUR	Ins/Del	ESRD	0.04	Healthy	
	Yorioka T et al.	8529313	EAS	Ins/Del	IgAN	0.013	Healthy	
	Yoshida H et al.	7593601	EAS	Ins/Del	IgAN	0.01	Healthy	
	Yoon HJ et al.	12220450	EAS	Ins/Del	IgAN	0.002	Healthy	
	Schmidt S et al.	7485124	EUR	Ins/Del	IgAN	0.007	Healthy	
	Suzuki H et al.	15481848	EAS	Ins/Del	IgAN	0.036	Healthy	0.004
	Zsom M et al.	22111818	EUR	Ins/Del	Primary GN	0.005	Healthy	
	Currie D et al.	20854388	EUR	Ins/Del	T1DN		T1DM	0.005
	Ringel J et al.	9049480	EUR	Ins/Del	T1DN		T1DM	0.018
	Tarnow L et al.	7729604	EUR	Ins/Del	T1DN		T1DM	0.013
	Schmidt S et al.	7783416	EUR	Ins/Del	T1DN	0.002	T1DM	
	Mizuiri S et al.	7477652	EAS	Ins/Del	T2DN	0.019	T2DM	
	Hsieh MC et al.	10862639	EAS	Ins/Del	T2DN	$4.2x10^{-05}$	T2DM	0.004
	Ha SK et al.	12722028	EAS	Ins/Del	T2DN		T2DM	0.026
	Schmidt S et al.	9269698	EUR	Ins/Del	T2DN	0.0147	T2DM	
	El-Baz R et al.	22554825	ME	Ins/Del	T2DN	0.0025	T2DM	$1x10^{-06}$
	Sancakdar E et al.	000367541400005	ME	Ins/Del	T2DN	0.0076	T2DM	0.003
	Kumar R et al.	23846111	SAS	Ins/Del	T2DN	0.0016	T2DM	0.0003
	Park HC et al.	16385653	EAS	Ins/Del	T2DN-ESRD		T2DM	0.022
AGT	Fogarty DG et al.	8772723	EUR	rs699	T1DN		T1DM	0.044
	Wu SH et al.	11776100	EAS	rs699	T2DN	0.0008	T2DM	
	Freire MB et al.	9535411	EUR	rs699	T2DN	0.022	T2DM	
	Reis KA et al.	21500980	ME	rs699	T2DN	0.006	T2DM	

For peer review only

	Fradin S et al.	11938025	EUR	rs699	T2DN	0.008	T2DM	
	Su SL et al.	29296205	EAS	rs699	ESRD		Healthy	0.010
AGTR1	Ahluwalia TS et al.	19108684	SAS	rs5186	T2DN		T2DM	0.0002
	Shah VN et al.	23081748	SAS	rs5186	T2DN		T2DM	$4.2x10^{-08}$
	Lau YK et al.	15153745	EAS	rs5186	IgAN	0.038	Healthy	
	Möllsten A et al.	8856207	EUR	rs5186	T1DN	0.029	T1DM	

Abbreviations: EAS, East Asian; EUR, European; ME, Middle Eastern; SAS, South Asian; CKD, Chronic Kidney Disease; ESRD, End-Stage Renal Disease; GN, Glomerulonephritis; IgAN, IgA Nephropathy; T1DM, Type 1 Diabetes Mellitus; T1DN, Type 1 Diabetic Nephropathy; T2DM, Type 2 Diabetes Mellitus; T2DN, Type 2 Diabetic Nephropathy.

For peer review only

Supplementary Data File S1. Allele frequencies for all control populations which consisted of healthy individuals alongside the dbSNP reported frequencies

***AGT* rs699 – East Asian populations included in meta-analysis**

- IgAN vs healthy controls
  - Five populations included in analysis
  - Allele frequencies are as follows:

AGT	Study	Population	Sample Size	Reference allele frequency	Alternative allele frequency
**rs699**	Gao J et al.(49)	East Asian	538	A=0.202	G=0.798
**rs699**	Guo Y et al.(77)	East Asian	1265	A=0.178	G=0.822
**rs699**	Huang HD et al.(50)	East Asian	329	A=0.167	G=0.833
**rs699**	Kim SM et al.(51)	East Asian	587	A=0.192	G=0.808
**rs699**	Lau YK et al.(25)	East Asian	212	A=0.165	G=0.835

- ESRD vs healthy controls
  - Three populations included in analysis
  - Allele frequencies:

AGT	Study	Population	Sample Size	Reference allele frequency	Alternative allele frequency
**rs699**	Kawada N et al.(22)	East Asian	424	A=0.158	G=0.841
**rs699**	Su SL et al.(157)	East Asian	1373	A=0.189	G=0.811
**rs699**	Wang AY et al.(63)	East Asian	429	A=0.175	G=0.825

- dbSNP frequencies:

AGT	Study	Population	Sample Size	Reference allele frequency	Alternative allele frequency
**rs699**	gnomAD - Genomes	East Asian	1622	A=0.156	G=0.844
**rs699**	1000Genomes	East Asian	1008	A=0.147	G=0.853

- Chi-square calculations for reference allele frequency comparing meta-analysed data with dbSNP data:

Group	gnomAD - Genomes	1000Genomes
***AGT* rs699 – East Asian populations IgAN vs Healthy Controls**	0.999516	0.999582
***AGT* rs699 – East Asian populations ESRD vs Healthy Controls**	0.984359	0.985654

For peer review only

***AGT* rs699 – European populations included in meta-analysis**

- ESRD vs healthy controls
  - Four populations included in analysis
  - Allele frequencies are as follows:

AGT	Study	Population	Sample Size	Reference allele frequency	Alternative allele frequency
**rs699**	Beige J et al. (146)	European	538	A=0.573	G=0.427
**rs699**	Buraczynska M et al.(65)	European	1265	A=0.542	G=0.458
**rs699**	Losito A et al.(67)	European	329	A=0.562	G=0.438
**rs699**	Lovati E et al.(147)	European	587	A=0.601	G=0.399

- dbSNP frequencies:

AGT	Study	Population	Sample Size	Reference allele frequency	Alternative allele frequency
**rs699**	gnomAD - Genomes	European	18440	A=0.5616	G=0.4384
**rs699**	gnomAD - Exomes	European	133914	A=0.57665	G=0.42335
**rs699**	1000Genomes	European	1006	A=0.588	G=0.412
**rs699**	ExAC	European	73258	A=0.5747	G=0.4253

- Chi-square calculations for alternative allele frequency comparing meta-analysed data with dbSNP data:

Group	gnomAD - Genomes	gnomAD - Exomes	1000Genomes	ExAC
***AGT* rs699 – European populations ESRD vs Healthy Controls**	0.979214	0.980521	0.981483	0.980354

For peer review only

***AGTR1* rs5186 – East Asian populations included in meta-analysis**

- IgAN vs healthy controls
  - Four populations included in analysis
  - Allele frequencies are as follows:

AGTR1	Study	Population	Sample Size	Reference allele frequency	Alternative allele frequency
**rs5186**	Gao J et al.(49)	East Asian	661	A=0.931	G=0.069
**rs5186**	Huang HD et al.(50)	East Asian	250	A=0.917	G=0.083
**rs5186**	Kim SM et al.(51)	East Asian	538	A=0.960	G=0.04
**rs5186**	Lau YK et al.(25)	East Asian	212	A=0.947	G=0.053

- dbSNP frequencies:

AGTR1	Study	Population	Sample Size	Reference allele frequency	Alternative allele frequency
**rs5186**	gnomAD - Genomes	East Asian	1618	A=0.944	C=0.056
**rs5186**	1000Genomes	East Asian	1008	A=0.940	C=0.060

- Chi-square calculations for alternative allele frequency comparing meta-analysed data with dbSNP data:

Group	gnomAD - Genomes	1000Genomes
***AGTR1* rs5186 – East Asian populations IgAN vs Healthy Controls**	0.999615	0.999559

For peer review only

ACE insertion/deletion – European, East Asian, South Asian and Middle Eastern populations included in meta-analysis

- Four reported rs numbers for this SNP rs numbers (rs13447447, rs1799752, rs4340, rs4646994) were not found in dbSNP and therefore no reported allele frequencies were available for comparison.

For peer review only

PRISMA 2009 Flow Diagram

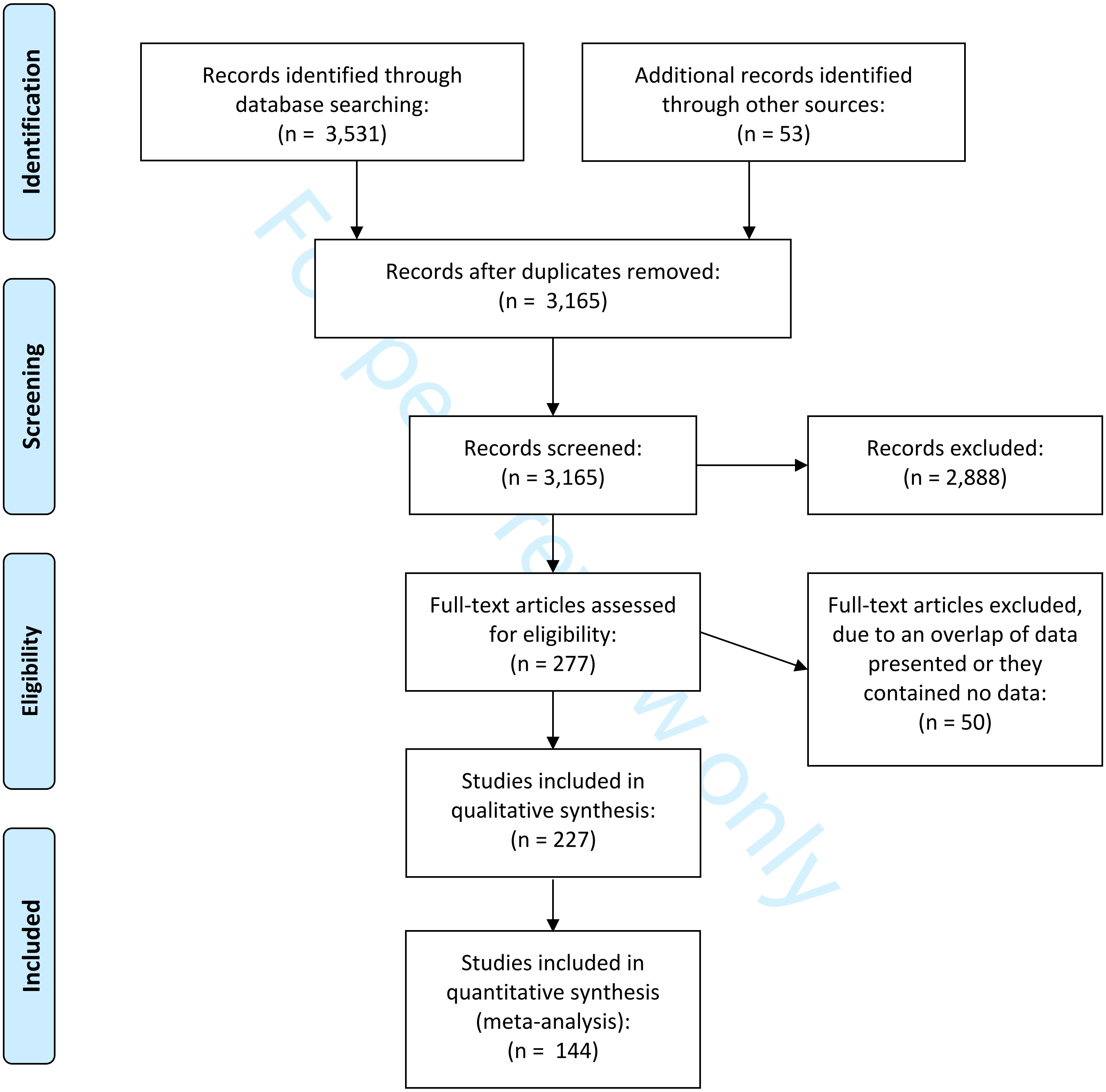

From: Moher D, Liberati A, Tetzlaff J, Altman DG, The PRISMA Group (2009). *P*referred *R*eporting *I*tems for *S*ystematic Reviews and *M*eta-*A*nalyses: The PRISMA Statement. PLoS Med 6(7): e1000097. doi:10.1371/journal.pmed1000097

For more information, visit www.prisma-statement.org.

For peer review only - http://bmjopen.bmj.com/site/about/guidelines.xhtml

PRISMA 2009 Checklist

Section/topic	#	Checklist item	Reported on page #
**TITLE**			
Title	1	Identify the report as a systematic review, meta-analysis, or both.	1
**ABSTRACT**			
Structured summary	2	Provide a structured summary including, as applicable: background; objectives; data sources; study eligibility criteria, participants, and interventions; study appraisal and synthesis methods; results; limitations; conclusions and implications of key findings; systematic review registration number.	1&2
**INTRODUCTION**			
Rationale	3	Describe the rationale for the review in the context of what is already known.	2&3
Objectives	4	Provide an explicit statement of questions being addressed with reference to participants, interventions, comparisons, outcomes, and study design (PICOS).	1-3
**METHODS**			
Protocol and registration	5	Indicate if a review protocol exists, if and where it can be accessed (e.g., Web address), and, if available, provide registration information including registration number.	5
Eligibility criteria	6	Specify study characteristics (e.g., PICOS, length of follow-up) and report characteristics (e.g., years considered, language, publication status) used as criteria for eligibility, giving rationale.	4
Information sources	7	Describe all information sources (e.g., databases with dates of coverage, contact with study authors to identify additional studies) in the search and date last searched.	3-5
Search	8	Present full electronic search strategy for at least one database, including any limits used, such that it could be repeated.	3 and STS1
Study selection	9	State the process for selecting studies (i.e., screening, eligibility, included in systematic review, and, if applicable, included in the meta-analysis).	3-4
Data collection process	10	Describe method of data extraction from reports (e.g., piloted forms, independently, in duplicate) and any processes for obtaining and confirming data from investigators.	4-5
Data items	11	List and define all variables for which data were sought (e.g., PICOS, funding sources) and any assumptions and simplifications made.	4-5
Risk of bias in individual studies	12	Describe methods used for assessing risk of bias of individual studies (including specification of whether this was done at the study or outcome level), and how this information is to be used in any data synthesis.	4-5
Summary measures	13	State the principal summary measures (e.g., risk ratio, difference in means).	4-5
Synthesis of results	14	Describe the methods of handling data and combining results of studies, if done, including measures of consistency (e.g., I^2) for each meta-analysis.	4-5

For peer review only

For peer review only - http://bmjopen.bmj.com/site/about/guidelines.xhtml

PRISMA 2009 Checklist

Section/topic	#	Checklist item	Reported on page #
Risk of bias across studies	15	Specify any assessment of risk of bias that may affect the cumulative evidence (e.g., publication bias, selective reporting within studies).	4-5
Additional analyses	16	Describe methods of additional analyses (e.g., sensitivity or subgroup analyses, meta-regression), if done, indicating which were pre-specified.	5
**RESULTS**			
Study selection	17	Give numbers of studies screened, assessed for eligibility, and included in the review, with reasons for exclusions at each stage, ideally with a flow diagram.	5 and SFS1
Study characteristics	18	For each study, present characteristics for which data were extracted (e.g., study size, PICOS, follow-up period) and provide the citations.	4&5
Risk of bias within studies	19	Present data on risk of bias of each study and, if available, any outcome level assessment (see item 12).	4-6
Results of individual studies	20	For all outcomes considered (benefits or harms), present, for each study: (a) simple summary data for each intervention group (b) effect estimates and confidence intervals, ideally with a forest plot.	7&8
Synthesis of results	21	Present results of each meta-analysis done, including confidence intervals and measures of consistency.	7&8
Risk of bias across studies	22	Present results of any assessment of risk of bias across studies (see Item 15).	7&8
Additional analysis	23	Give results of additional analyses, if done (e.g., sensitivity or subgroup analyses, meta-regression [see Item 16]).	NA
**DISCUSSION**			
Summary of evidence	24	Summarize the main findings including the strength of evidence for each main outcome; consider their relevance to key groups (e.g., healthcare providers, users, and policy makers).	7-10
Limitations	25	Discuss limitations at study and outcome level (e.g., risk of bias), and at review-level (e.g., incomplete retrieval of identified research, reporting bias).	2, 9&10
Conclusions	26	Provide a general interpretation of the results in the context of other evidence, and implications for future research.	7-10
**FUNDING**			
Funding	27	Describe sources of funding for the systematic review and other support (e.g., supply of data); role of funders for the systematic review.	12

From: Moher D, Liberati A, Tetzlaff J, Altman DG, The PRISMA Group (2009). Preferred Reporting Items for Systematic Reviews and Meta-Analyses: The PRISMA Statement. PLoS Med 6(7): e1000097. doi:10.1371/journal.pmed1000097

For more information, visit: **www.prisma-statement.org**.

For peer review only - http://bmjopen.bmj.com/site/about/guidelines.xhtml

Reporting checklist for meta-analysis of observational studies.

Based on the MOOSE guidelines.

Instructions to authors

Complete this checklist by entering the page numbers from your manuscript where readers will find each of the items listed below.

Your article may not currently address all the items on the checklist. Please modify your text to include the missing information. If you are certain that an item does not apply, please write "n/a" and provide a short explanation.

Upload your completed checklist as an extra file when you submit to a journal.

In your methods section, say that you used the MOOSE reporting guidelines, and cite them as:

Stroup DF, Berlin JA, Morton SC, Olkin I, Williamson GD, Rennie D, Moher D, Becker BJ, Sipe TA, Thacker SB. Meta-analysis of observational studies in epidemiology: a proposal for reporting. Meta-analysis Of Observational Studies in Epidemiology (MOOSE) group. JAMA. 2000; 283(15):2008-2012.

For peer review only

		Reporting Item	Page Number
	#1	Identify the study as a meta-analysis of observational research	1
	#2	Provide a structured summary including, as applicable: background; objectives; data sources; study eligibility	1&2

		criteria, participants, and interventions; study appraisal and synthesis methods; results; limitations; conclusions and implications of key findings; systematic review registration number (From PRISMA checklist)	
	#3a	Problem definition	3
	#3b	Hypothesis statement	2
	#3c	Description of study outcomes	3-5
	#3d	Type of exposure or intervention used	3-4
	#3e	Type of study designs used	3-4
	#3f	Study population	3-4
Search strategy	#4a	Qualifications of searchers (eg, librarians and investigators)	3-4
	#4b	Search strategy, including time period included in the synthesis and keywords	3-4
	#4c	Effort to include all available studies, including contact with authors	4
	#4d	Databases and registries searched	3-4
	#4e	Search software used, name and version, including special features used (eg, explosion)	4
	#4f	Use of hand searching (eg, reference lists of obtained articles)	3

For peer review only

#4g	List of citations located and those excluded, including justification	5&Supplementary Tables
#4h	Method of addressing articles published in languages other than English	4
#4i	Method of handling abstracts and unpublished studies	3-4
#4j	Description of any contact with authors	NA
#5a	Description of relevance or appropriateness of studies gathered for assessing the hypothesis to be tested	3&4
#5b	Rationale for the selection and coding of data (eg, sound clinical principles or convenience)	3&4
#5c	Documentation of how data were classified and coded (eg, multiple raters, blinding, and interrater reliability)	3&4
#5d	Assessment of confounding (eg, comparability of cases and controls in studies where appropriate)	4&5
#5e	Assessment of study quality, including blinding of quality assessors; stratification or regression on possible predictors of study results	4&5
#5f	Assessment of heterogeneity	4&5
#5g	Description of statistical methods (eg, complete description of fixed or random effects models, justification of whether the chosen models account for predictors of study results,	4&5

For peer review only

	dose-response models, or cumulative meta-analysis) in sufficient detail to be replicated	
#5h	Provision of appropriate tables and graphics	3,4&6 & additional materials
#6a	Graphic summarizing individual study estimates and overall estimate	Figures & Supplementary Figures
#6b	Table giving descriptive information for each study included	STS4-STS6
#6c	Results of sensitivity testing (eg, subgroup analysis)	5
#6d	Indication of statistical uncertainty of findings	4&5
#7a	Quantitative assessment of bias (eg. publication bias)	5
#7b	Justification for exclusion (eg, exclusion of non–English-language citations)	4
#7c	Assessment of quality of included studies	3-5
#8a	Consideration of alternative explanations for observed results	9-11
#8b	Generalization of the conclusions (ie, appropriate for the data presented and within the domain of the literature review)	9-11
#8c	Guidelines for future research	9-11
#8d	Disclosure of funding source	12

For peer review only

Reproduced with permission from JAMA. 2000. 283(15):2008-2012. Copyright © 2000 American Medical Association. All rights reserved.This checklist can be completed online using https://www.goodreports.org/, a tool made by the EQUATOR Network in collaboration with Penelope.ai

For peer review only
